# Worldwide trends in body-mass index, underweight, overweight, and obesity from 1975 to 2016: a pooled analysis of 2416 population-based measurement studies in 128·9 million children, adolescents, and adults

**DOI:** 10.1016/S0140-6736(17)32129-3

**Published:** 2017-12-16

**Authors:** NCD Risk Factor Collaboration (NCD-RisC), Leandra Abarca-Gómez, Ziad A Abdeen, Zargar Abdul Hamid, Niveen M Abu-Rmeileh, Benjamin Acosta-Cazares, Cecilia Acuin, Robert J Adams, Wichai Aekplakorn, Kaosar Afsana, Carlos A Aguilar-Salinas, Charles Agyemang, Alireza Ahmadvand, Wolfgang Ahrens, Kamel Ajlouni, Nazgul Akhtaeva, Hazzaa M Al-Hazzaa, Amani Rashed Al-Othman, Rajaa Al-Raddadi, Fadia Al Buhairan, Shahla Al Dhukair, Mohamed M Ali, Osman Ali, Ala'a Alkerwi, Mar Alvarez-Pedrerol, Eman Aly, Deepak N Amarapurkar, Philippe Amouyel, Antoinette Amuzu, Lars Bo Andersen, Sigmund A Anderssen, Dolores S Andrade, Lars H Ängquist, Ranjit Mohan Anjana, Hajer Aounallah-Skhiri, Joana Araújo, Inger Ariansen, Tahir Aris, Nimmathota Arlappa, Dominique Arveiler, Krishna K Aryal, Thor Aspelund, Felix K Assah, Maria Cecília F Assunção, May Soe Aung, Mária Avdicová, Ana Azevedo, Fereidoun Azizi, Bontha V Babu, Suhad Bahijri, Jennifer L Baker, Nagalla Balakrishna, Mohamed Bamoshmoosh, Maciej Banach, Piotr Bandosz, José R Banegas, Carlo M Barbagallo, Alberto Barceló, Amina Barkat, Aluisio JD Barros, Mauro VG Barros, Iqbal Bata, Anwar M Batieha, Rosangela L Batista, Assembekov Batyrbek, Louise A Baur, Robert Beaglehole, Habiba Ben Romdhane, Judith Benedics, Mikhail Benet, James E Bennett, Antonio Bernabe-Ortiz, Gailute Bernotiene, Heloisa Bettiol, Aroor Bhagyalaxmi, Sumit Bharadwaj, Santosh K Bhargava, Zaid Bhatti, Zulfiqar A Bhutta, Hongsheng Bi, Yufang Bi, Anna Biehl, Mukharram Bikbov, Bihungum Bista, Dusko J Bjelica, Peter Bjerregaard, Espen Bjertness, Marius B Bjertness, Cecilia Björkelund, Anneke Blokstra, Simona Bo, Martin Bobak, Lynne M Boddy, Bernhard O Boehm, Heiner Boeing, Jose G Boggia, Carlos P Boissonnet, Marialaura Bonaccio, Vanina Bongard, Pascal Bovet, Lien Braeckevelt, Lutgart Braeckman, Marjolijn CE Bragt, Imperia Brajkovich, Francesco Branca, Juergen Breckenkamp, João Breda, Hermann Brenner, Lizzy M Brewster, Garry R Brian, Lacramioara Brinduse, Graziella Bruno, H B(as) Bueno-de-Mesquita, Anna Bugge, Marta Buoncristiano, Genc Burazeri, Con Burns, Antonio Cabrera de León, Joseph Cacciottolo, Hui Cai, Tilema Cama, Christine Cameron, José Camolas, Günay Can, Ana Paula C Cândido, Mario Capanzana, Vincenzo Capuano, Viviane C Cardoso, Axel C Carlsson, Maria J Carvalho, Felipe F Casanueva, Juan-Pablo Casas, Carmelo A Caserta, Snehalatha Chamukuttan, Angelique W Chan, Queenie Chan, Himanshu K Chaturvedi, Nishi Chaturvedi, Chien-Jen Chen, Fangfang Chen, Huashuai Chen, Shuohua Chen, Zhengming Chen, Ching-Yu Cheng, Angela Chetrit, Ekaterina Chikova-Iscener, Arnaud Chiolero, Shu-Ti Chiou, Adela Chirita-Emandi, María-Dolores Chirlaque, Belong Cho, Yumi Cho, Kaare Christensen, Diego G Christofaro, Jerzy Chudek, Renata Cifkova, Eliza Cinteza, Frank Claessens, Els Clays, Hans Concin, Susana C Confortin, Cyrus Cooper, Rachel Cooper, Tara C Coppinger, Simona Costanzo, Dominique Cottel, Chris Cowell, Cora L Craig, Ana B Crujeiras, Alexandra Cucu, Graziella D'Arrigo, Eleonora d'Orsi, Jean Dallongeville, Albertino Damasceno, Camilla T Damsgaard, Goodarz Danaei, Rachel Dankner, Thomas M Dantoft, Saeed Dastgiri, Luc Dauchet, Kairat Davletov, Guy De Backer, Dirk De Bacquer, Amalia De Curtis, Giovanni de Gaetano, Stefaan De Henauw, Paula Duarte de Oliveira, Karin De Ridder, Delphine De Smedt, Mohan Deepa, Alexander D Deev, Abbas Dehghan, Hélène Delisle, Francis Delpeuch, Valérie Deschamps, Klodian Dhana, Augusto F Di Castelnuovo, Juvenal Soares Dias-da-Costa, Alejandro Diaz, Zivka Dika, Shirin Djalalinia, Ha TP Do, Annette J Dobson, Maria Benedetta Donati, Chiara Donfrancesco, Silvana P Donoso, Angela Döring, Maria Dorobantu, Ahmad Reza Dorosty, Kouamelan Doua, Wojciech Drygas, Jia Li Duan, Charmaine Duante, Vesselka Duleva, Virginija Dulskiene, Vilnis Dzerve, Elzbieta Dziankowska-Zaborszczyk, Eruke E Egbagbe, Robert Eggertsen, Gabriele Eiben, Ulf Ekelund, Jalila El Ati, Paul Elliott, Reina Engle-Stone, Rajiv T Erasmus, Cihangir Erem, Louise Eriksen, Johan G Eriksson, Jorge Escobedo-de la Peña, Alun Evans, David Faeh, Caroline H Fall, Victoria Farrugia Sant'Angelo, Farshad Farzadfar, Francisco J Felix-Redondo, Trevor S Ferguson, Romulo A Fernandes, Daniel Fernández-Bergés, Daniel Ferrante, Marika Ferrari, Catterina Ferreccio, Jean Ferrieres, Joseph D Finn, Krista Fischer, Eric Monterubio Flores, Bernhard Föger, Leng Huat Foo, Ann-Sofie Forslund, Maria Forsner, Heba M Fouad, Damian K Francis, Maria do Carmo Franco, Oscar H Franco, Guillermo Frontera, Flavio D Fuchs, Sandra C Fuchs, Yuki Fujita, Takuro Furusawa, Zbigniew Gaciong, Mihai Gafencu, Daniela Galeone, Fabio Galvano, Manoli Garcia-de-la-Hera, Dickman Gareta, Sarah P Garnett, Jean-Michel Gaspoz, Magda Gasull, Louise Gates, Harald Geiger, Johanna M Geleijnse, Anoosheh Ghasemian, Simona Giampaoli, Francesco Gianfagna, Tiffany K Gill, Jonathan Giovannelli, Aleksander Giwercman, Justyna Godos, Sibel Gogen, Rebecca A Goldsmith, David Goltzman, Helen Gonçalves, Margot González-Leon, Juan P González-Rivas, Marcela Gonzalez-Gross, Frederic Gottrand, Antonio Pedro Graça, Sidsel Graff-Iversen, Dušan Grafnetter, Aneta Grajda, Maria G Grammatikopoulou, Ronald D Gregor, Tomasz Grodzicki, Anders Grøntved, Giuseppe Grosso, Gabriella Gruden, Vera Grujic, Dongfeng Gu, Emanuela Gualdi-Russo, Pilar Guallar-Castillón, Ong Peng Guan, Elias F Gudmundsson, Vilmundur Gudnason, Ramiro Guerrero, Idris Guessous, Andre L Guimaraes, Martin C Gulliford, Johanna Gunnlaugsdottir, Marc Gunter, Xiuhua Guo, Yin Guo, Prakash C Gupta, Rajeev Gupta, Oye Gureje, Beata Gurzkowska, Laura Gutierrez, Felix Gutzwiller, Farzad Hadaegh, Charalambos A Hadjigeorgiou, Khairil Si-Ramlee, Jytte Halkjær, Ian R Hambleton, Rebecca Hardy, Rachakulla Hari Kumar, Maria Hassapidou, Jun Hata, Alison J Hayes, Jiang He, Regina Heidinger-Felso, Mirjam Heinen, Marleen Elisabeth Hendriks, Ana Henriques, Leticia Hernandez Cadena, Sauli Herrala, Victor M Herrera, Isabelle Herter-Aeberli, Ramin Heshmat, Ilpo Tapani Hihtaniemi, Sai Yin Ho, Suzanne C Ho, Michael Hobbs, Albert Hofman, Wilma M Hopman, Andrea RVR Horimoto, Claudia M Hormiga, Bernardo L Horta, Leila Houti, Christina Howitt, Thein Thein Htay, Aung Soe Htet, Maung Maung Than Htike, Yonghua Hu, José María Huerta, Constanta Huidumac Petrescu, Martijn Huisman, Abdullatif Husseini, Chinh Nguyen Huu, Inge Huybrechts, Nahla Hwalla, Jolanda Hyska, Licia Iacoviello, Anna G Iannone, Jesús M Ibarluzea, Mohsen M Ibrahim, Nayu Ikeda, M Arfan Ikram, Vilma E Irazola, Muhammad Islam, Aziz al-Safi Ismail, Vanja Ivkovic, Masanori Iwasaki, Rod T Jackson, Jeremy M Jacobs, Hashem Jaddou, Tazeen Jafar, Kazi M Jamil, Konrad Jamrozik, Imre Janszky, Juel Jarani, Grazyna Jasienska, Ana Jelakovic, Bojan Jelakovic, Garry Jennings, Seung-Lyeal Jeong, Chao Qiang Jiang, Santa Magaly Jiménez-Acosta, Michel Joffres, Mattias Johansson, Jost B Jonas, Torben Jørgensen, Pradeep Joshi, Dragana P Jovic, Jacek Józwiak, Anne Juolevi, Gregor Jurak, Vesna Jureša, Rudolf Kaaks, Anthony Kafatos, Eero O Kajantie, Ofra Kalter-Leibovici, Nor Azmi Kamaruddin, Efthymios Kapantais, Khem B Karki, Amir Kasaeian, Joanne Katz, Jussi Kauhanen, Prabhdeep Kaur, Maryam Kavousi, Gyulli Kazakbaeva, Ulrich Keil, Lital Keinan Boker, Sirkka Keinänen-Kiukaanniemi, Roya Kelishadi, Cecily Kelleher, Han CG Kemper, Andre P Kengne, Alina Kerimkulova, Mathilde Kersting, Timothy Key, Yousef Saleh Khader, Davood Khalili, Young-Ho Khang, Mohammad Khateeb, Kay-Tee Khaw, Ilse MSL Khouw, Ursula Kiechl-Kohlendorfer, Stefan Kiechl, Japhet Killewo, Jeongseon Kim, Yeon-Yong Kim, Jeannette Klimont, Jurate Klumbiene, Michael Knoflach, Bhawesh Koirala, Elin Kolle, Patrick Kolsteren, Paul Korrovits, Jelena Kos, Seppo Koskinen, Katsuyasu Kouda, Viktoria A Kovacs, Sudhir Kowlessur, Slawomir Koziel, Wolfgang Kratzer, Susi Kriemler, Peter Lund Kristensen, Steinar Krokstad, Daan Kromhout, Herculina S Kruger, Ruzena Kubinova, Renata Kuciene, Diana Kuh, Urho M Kujala, Zbigniew Kulaga, R Krishna Kumar, Marie Kunešová, Pawel Kurjata, Yadlapalli S Kusuma, Kari Kuulasmaa, Catherine Kyobutungi, Quang Ngoc La, Fatima Zahra Laamiri, Tiina Laatikainen, Carl Lachat, Youcef Laid, Tai Hing Lam, Orlando Landrove, Vera Lanska, Georg Lappas, Bagher Larijani, Lars E Laugsand, Laura Lauria, Avula Laxmaiah, Khanh Le Nguyen Bao, Tuyen D Le, May Antonnette O Lebanan, Catherine Leclercq, Jeannette Lee, Jeonghee Lee, Terho Lehtimäki, Luz M León-Muñoz, Naomi S Levitt, Yanping Li, Christa L Lilly, Wei-Yen Lim, M Fernanda Lima-Costa, Hsien-Ho Lin, Xu Lin, Lars Lind, Allan Linneberg, Lauren Lissner, Mieczyslaw Litwin, Jing Liu, Helle-Mai Loit, Luis Lopes, Roberto Lorbeer, Paulo A Lotufo, José Eugenio Lozano, Dalia Luksiene, Annamari Lundqvist, Nuno Lunet, Per Lytsy, Guansheng Ma, Jun Ma, George LL Machado-Coelho, Aristides M Machado-Rodrigues, Suka Machi, Stefania Maggi, Dianna J Magliano, Emmanuella Magriplis, Alagappan Mahaletchumy, Bernard Maire, Marjeta Majer, Marcia Makdisse, Reza Malekzadeh, Rahul Malhotra, Kodavanti Mallikharjuna Rao, Sofia Malyutina, Yannis Manios, Jim I Mann, Enzo Manzato, Paula Margozzini, Anastasia Markaki, Oonagh Markey, Larissa P Marques, Pedro Marques-Vidal, Jaume Marrugat, Yves Martin-Prevel, Rosemarie Martin, Reynaldo Martorell, Eva Martos, Stefano Marventano, Shariq R Masoodi, Ellisiv B Mathiesen, Alicia Matijasevich, Tandi E Matsha, Artur Mazur, Jean Claude N Mbanya, Shelly R McFarlane, Stephen T McGarvey, Martin McKee, Stela McLachlan, Rachael M McLean, Scott B McLean, Breige A McNulty, Safiah Md Yusof, Sounnia Mediene-Benchekor, Jurate Medzioniene, Aline Meirhaeghe, Jørgen Meisfjord, Christa Meisinger, Ana Maria B Menezes, Geetha R Menon, Gert BM Mensink, Indrapal I Meshram, Andres Metspalu, Haakon E Meyer, Jie Mi, Kim F Michaelsen, Nathalie Michels, Kairit Mikkel, Jody C Miller, Cláudia S Minderico, Juan Francisco Miquel, J Jaime Miranda, Daphne Mirkopoulou, Erkin Mirrakhimov, Marjeta Mišigoj-Durakovic, Antonio Mistretta, Veronica Mocanu, Pietro A Modesti, Mostafa K Mohamed, Kazem Mohammad, Noushin Mohammadifard, Viswanathan Mohan, Salim Mohanna, Muhammad Fadhli Mohd Yusoff, Drude Molbo, Line T Møllehave, Niels C Møller, Dénes Molnár, Amirabbas Momenan, Charles K Mondo, Eric A Monterrubio, Kotsedi Daniel K Monyeki, Jin Soo Moon, Leila B Moreira, Alain Morejon, Luis A Moreno, Karen Morgan, Erik Lykke Mortensen, George Moschonis, Malgorzata Mossakowska, Aya Mostafa, Jorge Mota, Anabela Mota-Pinto, Mohammad Esmaeel Motlagh, Jorge Motta, Thet Thet Mu, Magdalena Muc, Maria Lorenza Muiesan, Martina Müller-Nurasyid, Neil Murphy, Jaakko Mursu, Elaine M Murtagh, Vera Musil, Iraj Nabipour, Gabriele Nagel, Balkish M Naidu, Harunobu Nakamura, Jana Námešná, Ei Ei K Nang, Vinay B Nangia, Martin Nankap, Sameer Narake, Paola Nardone, Eva Maria Navarrete-Muñoz, William A Neal, Ilona Nenko, Martin Neovius, Flavio Nervi, Chung T Nguyen, Nguyen D Nguyen, Quang Ngoc Nguyen, Ramfis E Nieto-Martínez, Guang Ning, Toshiharu Ninomiya, Sania Nishtar, Marianna Noale, Oscar A Noboa, Teresa Norat, Sawada Norie, Davide Noto, Mohannad Al Nsour, Dermot O'Reilly, Galina Obreja, Eiji Oda, Glenn Oehlers, Kyungwon Oh, Kumiko Ohara, Örn Olafsson, Maria Teresa Anselmo Olinto, Isabel O Oliveira, Maciej Oltarzewski, Mohd Azahadi Omar, Altan Onat, Sok King Ong, Lariane M Ono, Pedro Ordunez, Rui Ornelas, Ana P Ortiz, Merete Osler, Clive Osmond, Sergej M Ostojic, Afshin Ostovar, Johanna A Otero, Kim Overvad, Ellis Owusu-Dabo, Fred Michel Paccaud, Cristina Padez, Elena Pahomova, Andrzej Pajak, Domenico Palli, Alberto Palloni, Luigi Palmieri, Wen-Harn Pan, Songhomitra Panda-Jonas, Arvind Pandey, Francesco Panza, Dimitrios Papandreou, Soon-Woo Park, Winsome R Parnell, Mahboubeh Parsaeian, Ionela M Pascanu, Nikhil D Patel, Ivan Pecin, Mangesh S Pednekar, Nasheeta Peer, Petra H Peeters, Sergio Viana Peixoto, Markku Peltonen, Alexandre C Pereira, Napoleon Perez-Farinos, Cynthia M Pérez, Annette Peters, Janina Petkeviciene, Ausra Petrauskiene, Niloofar Peykari, Son Thai Pham, Daniela Pierannunzio, Iris Pigeot, Hynek Pikhart, Aida Pilav, Lorenza Pilotto, Francesco Pistelli, Freda Pitakaka, Aleksandra Piwonska, Pedro Plans-Rubió, Bee Koon Poh, Hermann Pohlabeln, Raluca M Pop, Stevo R Popovic, Miquel Porta, Marileen LP Portegies, Georg Posch, Dimitrios Poulimeneas, Hamed Pouraram, Akram Pourshams, Hossein Poustchi, Rajendra Pradeepa, Mathur Prashant, Jacqueline F Price, Jardena J Puder, Iveta Pudule, Maria Puiu, Margus Punab, Radwan F Qasrawi, Mostafa Qorbani, Tran Quoc Bao, Ivana Radic, Ricardas Radisauskas, Mahfuzar Rahman, Mahmudur Rahman, Olli Raitakari, Manu Raj, Sudha Ramachandra Rao, Ambady Ramachandran, Jacqueline Ramke, Elisabete Ramos, Rafel Ramos, Lekhraj Rampal, Sanjay Rampal, Ramon A Rascon-Pacheco, Josep Redon, Paul Ferdinand M Reganit, Lourdes Ribas-Barba, Robespierre Ribeiro, Elio Riboli, Fernando Rigo, Tobias F Rinke de Wit, Ana Rito, Raphael M Ritti-Dias, Juan A Rivera, Sian M Robinson, Cynthia Robitaille, Daniela Rodrigues, Fernando Rodríguez-Artalejo, María del Cristo Rodriguez-Perez, Laura A Rodríguez-Villamizar, Rosalba Rojas-Martinez, Nipa Rojroongwasinkul, Dora Romaguera, Kimmo Ronkainen, Annika Rosengren, Ian Rouse, Joel GR Roy, Adolfo Rubinstein, Frank J Rühli, Blanca Sandra Ruiz-Betancourt, Paola Russo, Marcin Rutkowski, Charumathi Sabanayagam, Harshpal S Sachdev, Olfa Saidi, Benoit Salanave, Eduardo Salazar Martinez, Diego Salmerón, Veikko Salomaa, Jukka T Salonen, Massimo Salvetti, Jose Sánchez-Abanto, Sandjaja, Susana Sans, Loreto Santa Marina, Diana A Santos, Ina S Santos, Osvaldo Santos, Renata Nunes dos Santos, Rute Santos, Jouko L Saramies, Luis B Sardinha, Nizal Sarrafzadegan, Kai-Uwe Saum, Savvas Savva, Mathilde Savy, Marcia Scazufca, Angelika Schaffrath Rosario, Herman Schargrodsky, Anja Schienkiewitz, Sabine Schipf, Carsten O Schmidt, Ida Maria Schmidt, Constance Schultsz, Aletta E Schutte, Aye Aye Sein, Abhijit Sen, Idowu O Senbanjo, Sadaf G Sepanlou, Luis Serra-Majem, Svetlana A Shalnova, Sanjib K Sharma, Jonathan E Shaw, Kenji Shibuya, Dong Wook Shin, Youchan Shin, Rahman Shiri, Alfonso Siani, Rosalynn Siantar, Abla M Sibai, Antonio M Silva, Diego Augusto Santos Silva, Mary Simon, Judith Simons, Leon A Simons, Agneta Sjöberg, Michael Sjöström, Sine Skovbjerg, Jolanta Slowikowska-Hilczer, Przemyslaw Slusarczyk, Liam Smeeth, Margaret C Smith, Marieke B Snijder, Hung-Kwan So, Eugène Sobngwi, Stefan Söderberg, Moesijanti YE Soekatri, Vincenzo Solfrizzi, Emily Sonestedt, Yi Song, Thorkild IA Sørensen, Maroje Soric, Charles Sossa Jérome, Aicha Soumare, Angela Spinelli, Igor Spiroski, Jan A Staessen, Hanspeter Stamm, Gregor Starc, Maria G Stathopoulou, Kaspar Staub, Bill Stavreski, Jostein Steene-Johannessen, Peter Stehle, Aryeh D Stein, George S Stergiou, Jochanan Stessman, Jutta Stieber, Doris Stöckl, Tanja Stocks, Jakub Stokwiszewski, Gareth Stratton, Karien Stronks, Maria Wany Strufaldi, Ramón Suárez-Medina, Chien-An Sun, Johan Sundström, Yn-Tz Sung, Jordi Sunyer, Paibul Suriyawongpaisal, Boyd A Swinburn, Rody G Sy, Lucjan Szponar, E Shyong Tai, Mari-Liis Tammesoo, Abdonas Tamosiunas, Eng Joo Tan, Xun Tang, Frank Tanser, Yong Tao, Mohammed Rasoul Tarawneh, Jakob Tarp, Carolina B Tarqui-Mamani, Oana-Florentina Tautu, Radka Taxová Braunerová, Anne Taylor, Félicité Tchibindat, Holger Theobald, Xenophon Theodoridis, Lutgarde Thijs, Betina H Thuesen, Anne Tjonneland, Hanna K Tolonen, Janne S Tolstrup, Murat Topbas, Roman Topór-Madry, María José Tormo, Michael J Tornaritis, Maties Torrent, Stefania Toselli, Pierre Traissac, Dimitrios Trichopoulos, Antonia Trichopoulou, Oanh TH Trinh, Atul Trivedi, Lechaba Tshepo, Maria Tsigga, Shoichiro Tsugane, Marshall K Tulloch-Reid, Fikru Tullu, Tomi-Pekka Tuomainen, Jaakko Tuomilehto, Maria L Turley, Per Tynelius, Themistoklis Tzotzas, Christophe Tzourio, Peter Ueda, Eunice E Ugel, Flora AM Ukoli, Hanno Ulmer, Belgin Unal, Hannu MT Uusitalo, Gonzalo Valdivia, Susana Vale, Damaskini Valvi, Yvonne T van der Schouw, Koen Van Herck, Hoang Van Minh, Lenie van Rossem, Natasja M Van Schoor, Irene GM van Valkengoed, Dirk Vanderschueren, Diego Vanuzzo, Lars Vatten, Tomas Vega, Toomas Veidebaum, Gustavo Velasquez-Melendez, Biruta Velika, Giovanni Veronesi, WM Monique Verschuren, Cesar G Victora, Giovanni Viegi, Lucie Viet, Eira Viikari-Juntura, Paolo Vineis, Jesus Vioque, Jyrki K Virtanen, Sophie Visvikis-Siest, Bharathi Viswanathan, Tiina Vlasoff, Peter Vollenweider, Henry Völzke, Sari Voutilainen, Martine Vrijheid, Alisha N Wade, Aline Wagner, Thomas Waldhör, Janette Walton, Wan Mohamad Wan Bebakar, Wan Nazaimoon Wan Mohamud, Rildo S Wanderley, Ming-Dong Wang, Qian Wang, Ya Xing Wang, Ying-Wei Wang, S Goya Wannamethee, Nicholas Wareham, Adelheid Weber, Niels Wedderkopp, Deepa Weerasekera, Peter H Whincup, Kurt Widhalm, Indah S Widyahening, Andrzej Wiecek, Alet H Wijga, Rainford J Wilks, Johann Willeit, Peter Willeit, Tom Wilsgaard, Bogdan Wojtyniak, Roy A Wong-McClure, Justin YY Wong, Jyh Eiin Wong, Tien Yin Wong, Jean Woo, Mark Woodward, Frederick C Wu, Jianfeng Wu, Shouling Wu, Haiquan Xu, Liang Xu, Uruwan Yamborisut, Weili Yan, Xiaoguang Yang, Nazan Yardim, Xingwang Ye, Panayiotis K Yiallouros, Agneta Yngve, Akihiro Yoshihara, Qi Sheng You, Novie O Younger-Coleman, Faudzi Yusoff, Muhammad Fadhli M Yusoff, Luciana Zaccagni, Vassilis Zafiropulos, Ahmad A Zainuddin, Sabina Zambon, Antonis Zampelas, Hana Zamrazilová, Tomasz Zdrojewski, Yi Zeng, Dong Zhao, Wenhua Zhao, Wei Zheng, Yingfeng Zheng, Bekbolat Zholdin, Maigeng Zhou, Dan Zhu, Baurzhan Zhussupov, Esther Zimmermann, Julio Zuñiga Cisneros, James Bentham, Mariachiara Di Cesare, Ver Bilano, Honor Bixby, Bin Zhou, Gretchen A Stevens, Leanne M Riley, Cristina Taddei, Kaveh Hajifathalian, Yuan Lu, Stefan Savin, Melanie J Cowan, Christopher J Paciorek, Adela Chirita-Emandi, Alison J Hayes, Joanne Katz, Roya Kelishadi, Andre Pascal Kengne, Young-Ho Khang, Avula Laxmaiah, Yanping Li, Jun Ma, J Jaime Miranda, Aya Mostafa, Martin Neovius, Cristina Padez, Lekhraj Rampal, Aubrianna Zhu, James E Bennett, Goodarz Danaei, Zulfiqar A Bhutta, Majid Ezzati

## Abstract

**Background:**

Underweight, overweight, and obesity in childhood and adolescence are associated with adverse health consequences throughout the life-course. Our aim was to estimate worldwide trends in mean body-mass index (BMI) and a comprehensive set of BMI categories that cover underweight to obesity in children and adolescents, and to compare trends with those of adults.

**Methods:**

We pooled 2416 population-based studies with measurements of height and weight on 128·9 million participants aged 5 years and older, including 31·5 million aged 5–19 years. We used a Bayesian hierarchical model to estimate trends from 1975 to 2016 in 200 countries for mean BMI and for prevalence of BMI in the following categories for children and adolescents aged 5–19 years: more than 2 SD below the median of the WHO growth reference for children and adolescents (referred to as moderate and severe underweight hereafter), 2 SD to more than 1 SD below the median (mild underweight), 1 SD below the median to 1 SD above the median (healthy weight), more than 1 SD to 2 SD above the median (overweight but not obese), and more than 2 SD above the median (obesity).

**Findings:**

Regional change in age-standardised mean BMI in girls from 1975 to 2016 ranged from virtually no change (−0·01 kg/m^2^ per decade; 95% credible interval −0·42 to 0·39, posterior probability [PP] of the observed decrease being a true decrease=0·5098) in eastern Europe to an increase of 1·00 kg/m^2^ per decade (0·69–1·35, PP>0·9999) in central Latin America and an increase of 0·95 kg/m^2^ per decade (0·64–1·25, PP>0·9999) in Polynesia and Micronesia. The range for boys was from a non-significant increase of 0·09 kg/m^2^ per decade (−0·33 to 0·49, PP=0·6926) in eastern Europe to an increase of 0·77 kg/m^2^ per decade (0·50–1·06, PP>0·9999) in Polynesia and Micronesia. Trends in mean BMI have recently flattened in northwestern Europe and the high-income English-speaking and Asia-Pacific regions for both sexes, southwestern Europe for boys, and central and Andean Latin America for girls. By contrast, the rise in BMI has accelerated in east and south Asia for both sexes, and southeast Asia for boys. Global age-standardised prevalence of obesity increased from 0·7% (0·4–1·2) in 1975 to 5·6% (4·8–6·5) in 2016 in girls, and from 0·9% (0·5–1·3) in 1975 to 7·8% (6·7–9·1) in 2016 in boys; the prevalence of moderate and severe underweight decreased from 9·2% (6·0–12·9) in 1975 to 8·4% (6·8–10·1) in 2016 in girls and from 14·8% (10·4–19·5) in 1975 to 12·4% (10·3–14·5) in 2016 in boys. Prevalence of moderate and severe underweight was highest in India, at 22·7% (16·7–29·6) among girls and 30·7% (23·5–38·0) among boys. Prevalence of obesity was more than 30% in girls in Nauru, the Cook Islands, and Palau; and boys in the Cook Islands, Nauru, Palau, Niue, and American Samoa in 2016. Prevalence of obesity was about 20% or more in several countries in Polynesia and Micronesia, the Middle East and north Africa, the Caribbean, and the USA. In 2016, 75 (44–117) million girls and 117 (70–178) million boys worldwide were moderately or severely underweight. In the same year, 50 (24–89) million girls and 74 (39–125) million boys worldwide were obese.

**Interpretation:**

The rising trends in children's and adolescents' BMI have plateaued in many high-income countries, albeit at high levels, but have accelerated in parts of Asia, with trends no longer correlated with those of adults.

**Funding:**

Wellcome Trust, AstraZeneca Young Health Programme.

## Introduction

Being underweight, overweight, or obese during childhood and adolescence is associated with adverse health consequences throughout the life-course. Underweight among children and adolescents is associated with higher risk of infectious diseases, and for girls of childbearing age, is associated with adverse pregnancy outcomes including maternal mortality, delivery complications, preterm birth, and intrauterine growth retardation.^1^, ^2^ Preventing and reversing excess weight in children and adolescents is also important for many reasons;^3^, ^4^ first, weight loss and maintenance after weight loss are hard to achieve,^5^ therefore gaining excess weight in childhood and adolescence is likely to lead to lifelong overweight and obesity.^6^ Second, being overweight in childhood and adolescence is associated with greater risk and earlier onset of chronic disorders such as type 2 diabetes.^3^, ^4^, ^7^, ^8^, ^9^ Third, childhood and adolescent obesity has adverse psychosocial consequences and lowers educational attainment.^3^, ^4^, ^10^, ^11^ Finally, children and adolescents are more susceptible to food marketing than adults, which makes reducing children's exposure to obesogenic foods necessary to protect them from harm.^3^, ^12^

**Research in context**

**Evidence before this study**

We searched MEDLINE (via PubMed) for articles published in any language between Jan 1, 1950, and July 12, 2017, using the search terms (“body size”[mh:noexp] OR “body height”[mh:noexp] OR “body weight”[mh:noexp] OR “birth weight”[mh:noexp] OR “overweight”[mh:noexp] OR “obesity”[mh] OR “thinness”[mh:noexp] OR “Waist-Hip Ratio”[mh:noexp] OR “Waist Circumference”[mh:noexp] OR “body mass index”[mh:noexp]) AND (“Humans”[mh]) AND (“Health Surveys”[mh] OR “Epidemiological Monitoring”[mh] OR “Prevalence”[mh]) NOT Comment[ptyp] NOT Case Reports[ptyp]. Articles were screened according to the inclusion and exclusion criteria described in the Appendix.

We identified three prior global analyses of mean body-mass index (BMI) or prevalence of overweight and obesity among adults. One of these studies also estimated the prevalence of underweight in adults. Another study also included data on overweight and obesity in children and adolescents, using a combination of measured and self-reported height and weight, and analysed in the same model as adults. A few multicountry studies and systematic reviews have reported, quantitatively or qualitatively, trends in overweight and obesity in children and adolescents, some also reporting underweight. To our knowledge, there is no global analysis of mean BMI, which is a summary measure of population distribution, or prevalence of underweight among children and adolescents aged 5–19 years.

**Added value of this study**

This study provides a complete picture of trends in mean BMI and prevalence of BMI categories that cover the underweight to obese range among children and adolescents aged 5–19 years, for all countries in the world with the longest observation period, and compares trends with those of adults. It includes the first global estimates of mean BMI and underweight prevalence for children and adolescents. We also present trends in the number of children, adolescents, and adults who are moderately or severely underweight and obese, and thus at risk of adverse health outcomes.

**Implications of all the available evidence**

Over the past four decades, mean BMI and obesity in children and adolescents aged 5–19 years have increased in most regions and countries. Despite this rise, more children and adolescents are moderately or severely underweight than obese, with the burden of underweight increasingly concentrated in south Asia and central, east and west Africa. The rise in children's and adolescents' BMI has plateaued, albeit at high levels, in many high-income countries but has accelerated in parts of Asia. There is a need for bridging the disconnect between policies that address underweight and overweight in children and adolescents to coherently address the large remaining underweight burden while curbing and reversing the rise in overweight and obesity.

Although trends in children's and adolescents' weight status have been documented in individual countries, little comparable information exists on worldwide trends, and none for mean body-mass index (BMI) and underweight. We pooled population-based data to estimate trends from 1975 to 2016 in mean BMI and in the prevalence of a comprehensive set of BMI categories that cover the underweight to obese range among children and adolescents for all countries in the world. To compare the trajectory of obesity and underweight in children and adolescents with that of adults, we also generated updated estimates for adults.^13^

## Methods

### Study design

We pooled and analysed population-based studies that had measured height and weight in people aged 5 years and older to estimate trends from 1975 to 2016 in mean BMI and BMI categories in 200 countries and territories (appendix). We started our analysis from 5 years of age because the definitions of underweight, overweight, and obesity change at 5 years of age.^14^ Further, children enter school at or around this age, which is associated with a change in their nutrition and physical activity.^15^

We present data on school-aged children and adolescents aged 5–19 years and on adults aged 20 years and older. We did separate analyses for children and adolescents and for adults for two reasons: first, cutoffs used to define underweight, overweight, and obesity for children and adolescents are different from those for adults and vary by age and sex because of the natural growth in childhood and adolescence.^16^ Second, the trajectory of the obesity epidemic in children and adolescents might be different from that of adults,^17^ motivating separate analyses of trends. Similarly, underweight in children and adolescents is typically targeted through school and community-based nutrition programmes, decoupling its trajectory from that of adults.

For children and adolescents, we analysed trends in mean BMI and prevalence of BMI in the following categories: more than 2 SD below the median of the WHO growth reference for children and adolescents^16^ (hereafter referred to as moderate and severe underweight), 2 SD to more than 1 SD below the median (mild underweight), 1 SD below the median to 1 SD above the median (healthy weight), more than 1 SD to 2 SD above the median (overweight but not obese), and more than 2 SD above the median (obesity). The cutoffs for calculating prevalence in these BMI categories were all age-specific and sex-specific and were applied to data in 1-year age bands. We used the WHO definitions because they include a comprehensive set of BMI categories ranging from moderate and severe underweight to obesity, defined based on symmetric thresholds of SDs from the reference population median. For adults, we analysed trends in mean BMI and prevalence of a comprehensive set of BMI categories as described in detail elsewhere and in the appendix.^13^ Results for children and adolescents are presented here; updated results for adults are presented in the appendix except when compared with children and adolescents.

### Data sources

Our methods for identifying and accessing data sources, and our inclusion and exclusion criteria, are described in the appendix. In summary, we used a database of population-based data on cardiometabolic risk factors collated by the Non-communicable Disease Risk Factor Collaboration (NCD-RisC), a worldwide network of health researchers and practitioners whose aim is to document systematically the worldwide trends and variations in non-communicable disease risk factors. The database was collated through multiple routes for identifying and accessing data. We accessed publicly available population-based multi-country and national measurement surveys, as well as the WHO STEPwise approach to Surveillance (STEPS) surveys. We requested, via WHO and its regional and country offices, help with identification and access to population-based surveys from ministries of health and other national health and statistical agencies. We also sent requests via the World Heart Federation to its national partners. We made a similar request to the authors of an earlier pooled analysis of cardiometabolic risk factors,^18^, ^19^, ^20^, ^21^ and invited them to reanalyse data from their studies and join NCD-RisC. To identify major sources not accessed through the above routes, we searched and reviewed published studies as detailed previously,^13^ and invited all eligible studies to join NCD-RisC. Finally, NCD-RisC members were periodically asked to review the list of sources from their country, to suggest additional sources not in the database, and to verify that the included data from their country met the inclusion criteria as listed in the appendix and that there were no duplicates.

The list of data sources and their characteristics is provided in the appendix. In summary, we included data collected on samples of a national, subnational (ie, covering one or more subnational regions, or more than three communities), or community (one or a few communities) population, which had measured height and weight. We did not use self-reported height and weight because they are subject to biases that vary by geography, time, age, sex, and socioeconomic characteristics.^22^, ^23^, ^24^ Because of these variations, approaches to correcting self-reported data leave residual bias.

### Statistical analysis

The statistical models used to estimate mean and prevalence by country, year, sex, and age are described in detail in a statistical paper and related substantive papers;^13^, ^25^, ^26^, ^27^, ^28^ the computer code is available from the NCD-RisC website. In summary, we organised countries into 21 regions, mostly based on geography and national income (appendix). The exception was high-income English-speaking countries (Australia, Canada, Ireland, New Zealand, the UK, and the USA), grouped together in one region because BMI and other cardiometabolic risk factors have similar trends in these countries, which can be distinct from other countries in their geographical regions.^13^, ^26^, ^27^, ^28^

The model had a hierarchical structure in which estimates for each country and year were informed by its own data, if available, and by data from other years in the same country and from other countries, especially those in the same region with data for similar time periods. The extent to which estimates for each country-year were influenced by data from other years and other countries depended on whether the country had data, the sample size of data, whether or not they were national, and the within-country and within-region variability of the available data. The model incorporated non-linear time trends comprising linear terms and a second-order random walk, all modelled hierarchically. The age association of BMI was modelled using a cubic spline to allow non-linear age patterns, which might vary across countries. The model accounted for the possibility that BMI in subnational and community samples might systematically differ from nationally representative ones, and have larger variation than in national studies. These features were implemented by including data-driven fixed-effect and random-effect terms for subnational and community data. The fixed effects adjusted for systematic differences between subnational or community studies and national studies. The random effects allowed national data to have larger influence on the estimates than subnational or community data with similar sample sizes. The model also accounted for rural–urban differences in BMI, through the use of data-driven fixed effects for rural-only and urban-only studies. These rural and urban effects were weighted by the difference between study-level and country-level urbanisation in the year when the study was done. The statistical model included a covariate (proportion of national population living in urban areas; data from the World Urbanization Prospects, 2014 revision) that is associated with, and helps predict, BMI.^29^ Results of model validation are reported elsewhere.^13^ We performed all analyses by sex, because there are differences in BMI levels and trends in relation to sex.^13^

We analysed the data on mean BMI and on each of the above prevalence categories separately. We re-scaled the estimated prevalence categories so that the sum of different categories was 1·0 in each age, sex, country, and year. The average scaling factors across draws ranged from 1·03 to 1·07, ie, the sum of the separately estimated prevalence categories was close to 1·0.

We fitted the statistical model with the Markov chain Monte Carlo (MCMC) algorithm, and obtained 5000 post-burn-in samples from the posterior distribution of model parameters, which were in turn used to obtain the posterior distributions of the above primary outcomes, ie, mean BMI and each of the prevalence categories. For model fitting, data on participants aged 5–19 years were included in the analysis of trends in children and adolescents, and on participants aged 18 years and older in the analysis of trends in adults. Data on participants aged 18 and 19 years were included in both sets of models because these groups form a transitional age from adolescence to adulthood, and hence help inform the estimates in both groups. Posterior estimates were made in 1-year age groups for ages 5–19 years and in 5-year age groups for those aged 20 years and older. For presentation, we used the posterior estimates for ages 5–19 years for children and adolescents, and for ages 20 years and older for adults. Age-standardised estimates were generated by taking weighted averages of age-sex-specific estimates, separately for children and adolescents and for adults, with use of age weights from the WHO standard population.^30^ Estimates for regions and the world were calculated as population-weighted averages of the constituent country estimates by age group and sex. The number of children, adolescents, and adults who were underweight, overweight, or obese was calculated by multiplying the corresponding age-specific prevalence by the population by country, year, and sex.

The reported credible intervals (CrI) represent the 2·5th to 97·5th percentiles of the posterior distributions. The uncertainties of our estimates, represented by the widths of the credible intervals, arise from uncertainty due to sampling in each data source; uncertainty associated with the variability of national data beyond what is accounted for by sampling; additional uncertainty associated with subnational and community data, and data that are from rural-only or urban-only samples; and uncertainty due to making estimates by country, year, and age when data were missing or scarce, in the country-year-age group unit for which estimates are made, in proximate time periods and ages in that country and in other countries in the same region. We also report the posterior probability (PP) that an estimated increase or decrease represents a truly increasing or decreasing trend.

### Role of the funding source

The funder of the study had no role in study design, data collection, analysis, interpretation, or writing of the report. Country and Regional Data Group members, JB, MDC, VB, HB, and BZ had full access to the data in the study. The corresponding author had final responsibility for the decision to submit for publication.

## Results

The results can be explored using dynamic visualisations and downloaded from the NCD-RisC website. We pooled 2416 population-based data sources with measurement of height and weight on 128·9 million people aged 5 years and older from 1975 to 2016 (appendix). 1099 sources included data on 24·1 million participants aged 5–17 years, 848 sources included data on 7·4 million participants aged 18–19 years, and 1820 sources included data on 97·4 million participants aged 20 years and older (appendix). Additional information on the age distribution of data sources and participants is shown in the appendix. 1187 (49%) of 2416 data sources were from national samples, 390 (16%) covered one or more subnational regions, and the remaining 839 (35%) were from one or a few communities (appendix). 583 (24%) of 2416 data sources were from before 1995 and another 1833 (76%) were from 1995 and later (appendix). The number of data sources per country in different regions ranged from 3·4 in the Caribbean to 54·0 in the high-income Asia-Pacific region (appendix).

**Figure 1:**
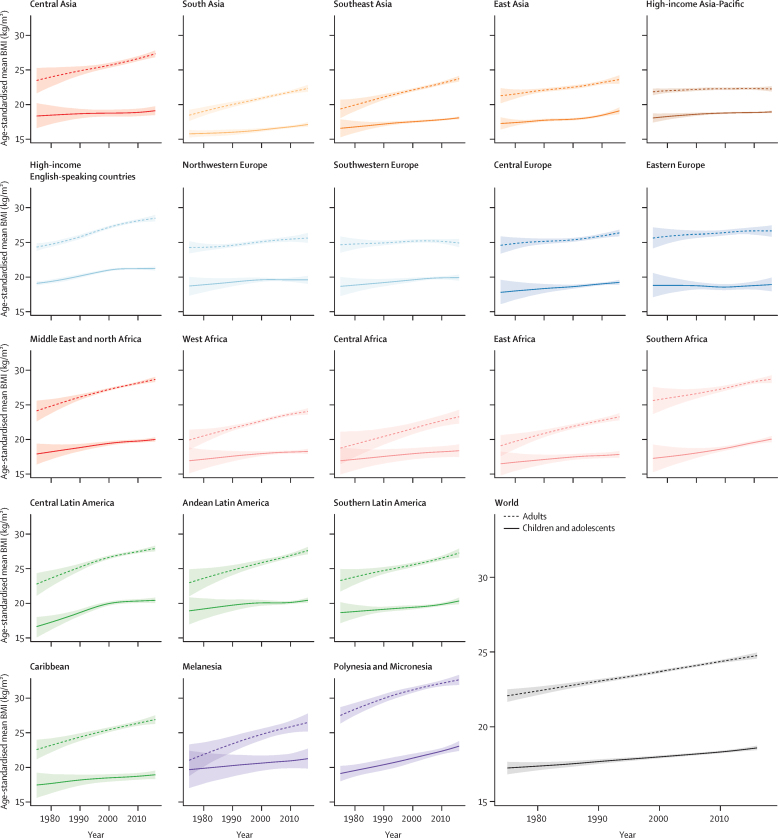
Trends in age-standardised mean BMI by sex and region in females Children and adolescents were aged 5–19 years and adults were aged 20 years and older. The lines show the posterior mean estimates and the shaded areas show the 95% credible intervals. See appendix for trends by country. BMI=body-mass index.

**Figure 2:**
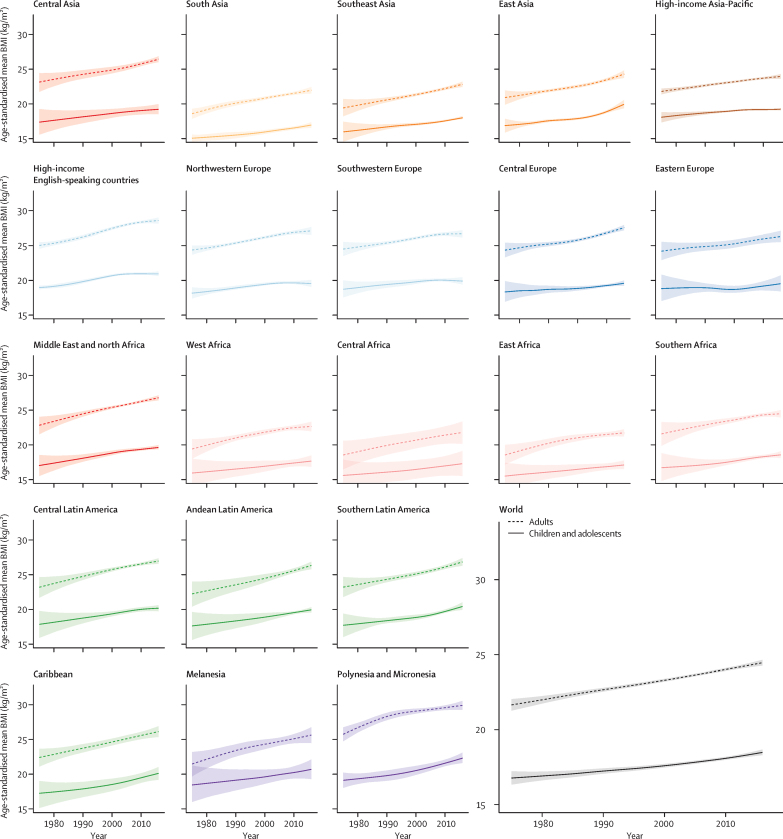
Trends in age-standardised mean BMI by sex and region in males Children and adolescents were aged 5–19 years and adults were aged 20 years and older. The lines show the posterior mean estimates and the shaded areas show the 95% credible intervals. See appendix for trends by country. BMI=body-mass index.

In 1975, the global age-standardised mean BMI of children and adolescents aged 5–19 years was 17·2 kg/m^2^ (95% CrI 16·8–17·6) for girls (figure 1) and 16·8 kg/m^2^ (16·3–17·2) for boys (figure 2). Mean BMI was lowest in south Asia, with an age-standardised mean of 15·8 kg/m^2^ (15·2–16·3) for girls and 15·0 kg/m^2^ (14·5–15·6) for boys, followed by east Africa (16·5 kg/m^2^ [14·8–18·2] for girls and 15·5 kg/m^2^ [13·6–17·4] for boys). Girls in Melanesia, Polynesia and Micronesia, and the high-income English-speaking region had the highest age-standardised mean BMI in 1975, all above 19·0 kg/m^2^. The highest mean BMIs for boys were those in Polynesia and Micronesia (19·1 kg/m^2^, 18·0–20·2), followed by the high-income English-speaking region.

From 1975 to 2016, children's and adolescents' age-standardised mean BMI increased globally and in most regions (Figure 1, Figure 2). The global increase was 0·32 kg/m^2^ per decade (95% CrI 0·23–0·41, PP of the observed increase being a true increase>0·9999) for girls and 0·40 kg/m^2^ per decade (0·30–0·50, PP>0·9999) for boys, leading to virtually identical age-standardised mean BMIs of 18·6 kg/m^2^ (18·4–18·7) for girls and 18·5 kg/m^2^ (18·3–18·7) for boys in 2016. The corresponding figures for adults were 24·8 kg/m^2^ (24·6–25·0) in women and 24·5 kg/m^2^ (24·3–24·6) in men.

Regional change in girls ranged from virtually no change (−0·01 kg/m^2^ per decade [95% CrI −0·42 to 0·39; PP of the observed decrease being a true decrease=0·5098]) in eastern Europe to 1·00 kg/m^2^ increase per decade (0·69–1·35, PP>0·9999) in central Latin America and 0·95 kg/m^2^ per decade (0·64–1·25, PP>0·9999) in Polynesia and Micronesia. The range for boys was from 0·09 kg/m^2^ per decade (−0·33 to 0·49; PP=0·6926) in eastern Europe to 0·77 kg/m^2^ per decade (0·50–1·06, PP>0·9999) in Polynesia and Micronesia. In some regions, children's and adolescents' BMI increased gradually over the four decades of analysis (Figure 1, Figure 2). However, there has been a recent flattening of trends in northwestern Europe and the high-income English-speaking and Asia-Pacific regions for both sexes, southwestern Europe for boys, and central and Andean Latin America for girls. With the exception of women in the high-income Asia-Pacific region, adult mean BMI continues to increase in all of these regions and sexes (Figure 1, Figure 2). By contrast with this plateauing, the rise in mean BMI has accelerated since around 2000 in east and south Asia for both sexes, and in southeast Asia for boys.

The lowest mean child and adolescent BMIs in 2016 were still those in south Asia and east Africa, with age-standardised mean BMIs between 16·9 and 17·9 kg/m^2^ for girls and boys; the highest were those in Polynesia and Micronesia for both sexes, followed by Melanesia and the high-income English-speaking region. Age-standardised mean BMIs of girls and boys in Polynesia and Micronesia, which were 23·1 kg/m^2^ (95% CrI 22·4–23·8) and 22·4 kg/m^2^ (21·6–23·1), respectively, were higher than those of adults in some regions. Children's and adolescents' age-standardised mean BMI was also more than 20 kg/m^2^ in Melanesia and many parts of Latin America and the Caribbean.

The regional rankings in 2016 differed slightly between children aged 5–9 years and adolescents aged 10–19 years (appendix). For example, the lowest mean BMI in children aged 5–9 years was seen in east Africa in both sexes, whereas in those aged 10–19 years, south Asian girls and boys had lower mean BMI than their African peers. Polynesians and Micronesians had the highest mean BMI in those aged 5–9 and 10–19 years, with the subsequent spots held by the high-income English-speaking region, regions in Latin America and the Caribbean, and Melanesia. Among these regions, central Latin America had a poorer ranking (ie, higher BMI relative to other regions) at age 10–19 years than at age 5–9 years, as did boys in the high-income English-speaking region. By contrast, east Asia performed worse in ranking in 5–9 years of age than it did in 10–19 years.

**Figure 3:**
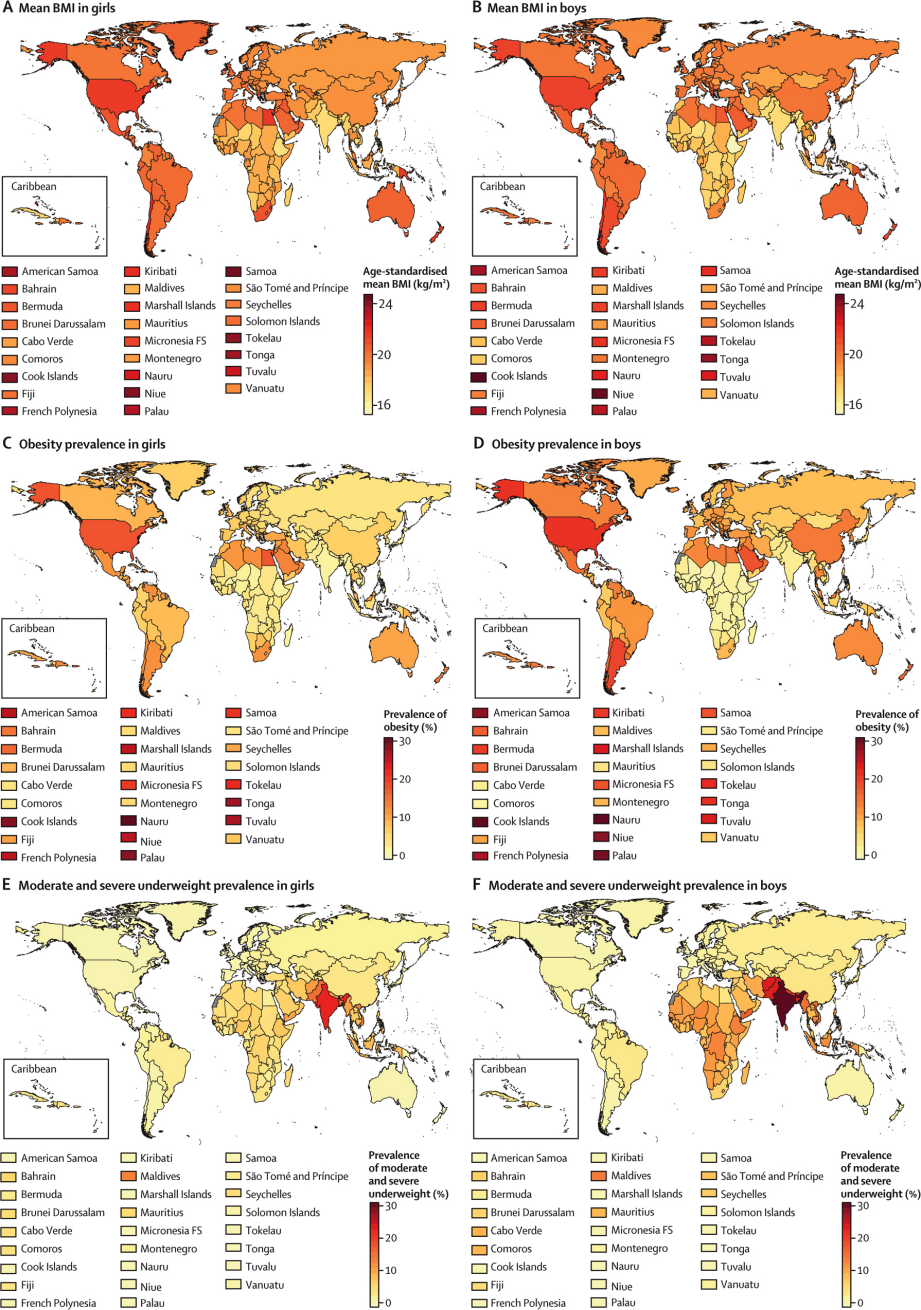
Age-standardised mean BMI, prevalence of obesity, and prevalence of moderate and severe underweight by sex and country in 2016 in children and adolescents Children and adolescents were aged 5–19 years. Obesity was defined as more than 2 SD above the median of the WHO growth reference. Moderate and severe underweight was defined as more than 2 SD below the median. See appendix for results for adults. BMI=body-mass index.

**Figure 4:**
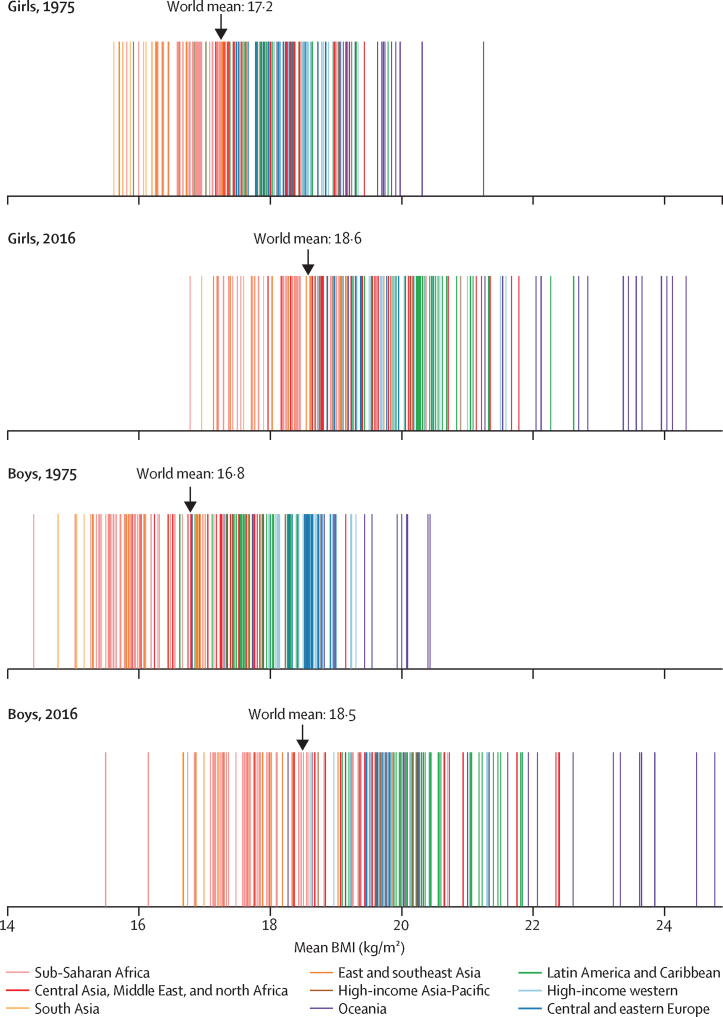
Age-standardised mean BMI in children and adolescents in 1975 and 2016 Each line shows one country. BMI=body-mass index.

The lowest age-standardised mean BMI over the 42 years of analysis among girls was in Bangladesh in 1975 (15·6 kg/m^2^, 95% CrI 13·2–17·9), and among boys was in Ethiopia in 1975 (14·4 kg/m^2^, 11·9–17·0; Figure 3, Figure 4). Age-standardised mean BMI in 1975 was less than 21 kg/m^2^ in every country, except for girls in American Samoa, who had an age-standardised mean BMI of 21·2 kg/m^2^ (20·6–21·9). From 1975 to 2016, age-standardised mean BMI increased by more than 0·25 kg/m^2^ per decade in 155 countries in girls, with the rise more than 1·0 kg/m^2^ per decade in some countries in Polynesia and in Mexico (PP of being a true rise>0·9999); in boys, the rise was more than 0·25 kg/m^2^ per decade in 189 countries and more than 1·0 kg/m^2^ per decade in the Cook Islands. When subsets of the analysis period are considered, before the year 2000, age-standardised mean BMI increased in almost every country. After 2000, there were non-significant declines in mean BMI in 29 countries for girls and 12 (mostly high-income) countries for boys.

In 2016, Ethiopia had the lowest age-standardised mean BMI for both sexes, 16·8 kg/m^2^ (95% CrI 15·6–17·9) for girls and 15·5 kg/m^2^ (14·4–16·6) for boys (Figure 3, Figure 4). Other countries with low BMI in both sexes in 2016 were Niger, Senegal, India, Bangladesh, Myanmar, and Cambodia. At the other extreme, age-standardised mean BMI was more than 24 kg/m^2^ in girls and boys in the Cook Islands and Niue and girls in Samoa, which was greater than that for adults of the same sex in 36 countries for girls and 59 countries for boys. Age-standardised mean BMI was between 22 and 24 kg/m^2^ in another 11 countries for girls and 10 countries for boys including in Polynesian and Micronesian islands, girls in the Bahamas and Chile, and boys in Qatar and Kuwait.

**Figure 5:**
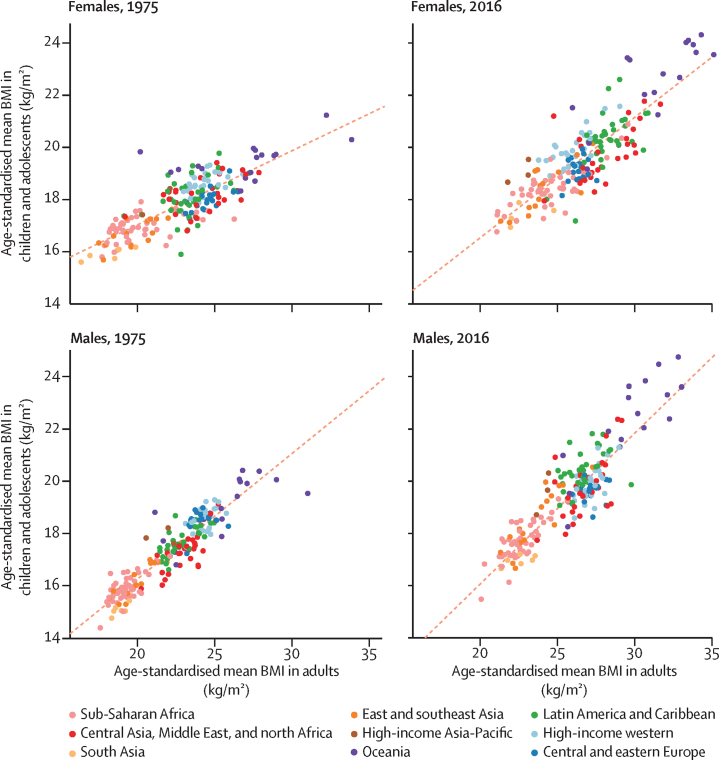
Comparison of age-standardised mean BMI in children and adolescents and in adults Children and adolescents were aged 5–19 years and adults were aged 20 years and older. Each point shows one country. The dotted line shows the linear association between the two outcomes. BMI=body-mass index.

**Figure 6:**
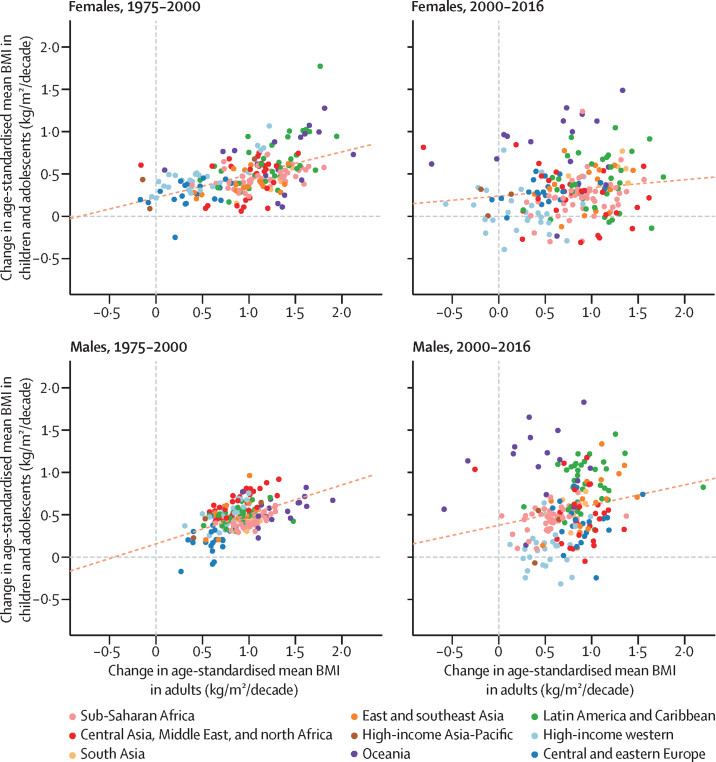
Comparison of change in age-standardised mean BMI in children and adolescents and in adults Children and adolescents were aged 5–19 years and adults were aged 20 years and older. Each point shows one country. The dotted line shows the linear association between the two outcomes. BMI=body-mass index.

The age-standardised mean BMI for children and adolescents and for adults were correlated in 1975 and 2016 (correlation coefficients 0·80 and 0·85 for females and 0·92 and 0·89 for males; figure 5). Changes in age-standardised mean BMI were moderately correlated between the two age groups before 2000 (correlation coefficient 0·52 for females and 0·51 for males) but only weakly after 2000 (correlation coefficient 0·14 for females and 0·21 for males; figure 6). The decoupling of BMI trends in children and adolescents and those of adults is due to a set of distinct regional phenomena: adults continued to gain weight in most western countries, where children's and adolescents' mean BMI stopped rising. By contrast, the rise in adult BMI seems to have plateaued in Oceania, albeit at high levels, whereas children's and adolescents' BMI continues to rise. In Latin America and the Caribbean, there is more variation in the rate of BMI increase in children and adolescents than in adults.

**Figure 7:**
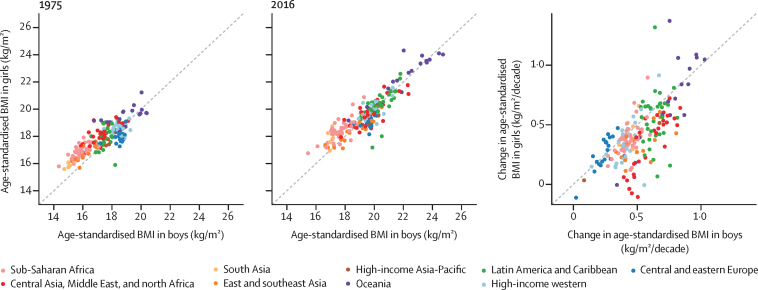
Comparison of age-standardised mean BMI in 1975 and 2016, and change per decade in age-standardised mean BMI from 1975 to 2016 by sex Each point shows one country. BMI=body-mass index.

In 1975, girls had higher age-standardised mean BMI than boys in most countries in sub-Saharan Africa, south Asia, and the Middle East and north Africa, and lower age-standardised mean BMI than boys in many countries in Europe and Latin America and the Caribbean (figure 7). Higher BMI in girls than boys was still seen in 2016 in many sub-Saharan African and south Asian countries. By contrast, the gap between sexes in BMI in the Middle East and north Africa shrank or reversed as boys gained more weight than girls. In Europe and Latin America, girls gained more weight than boys, closing the gap between sexes in BMI.

**Figure 8:**
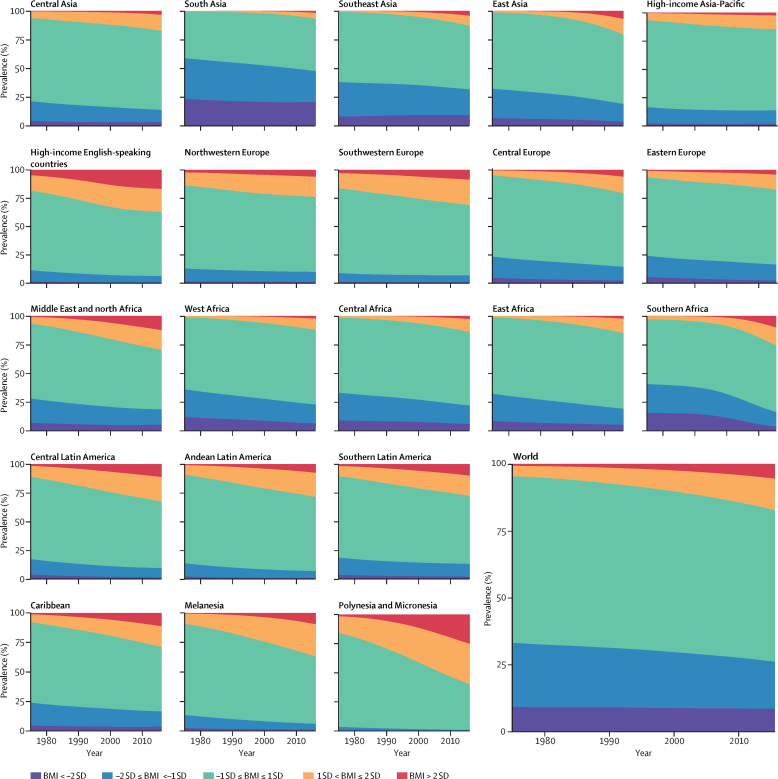
Trends in age-standardised prevalence of BMI categories in female children and adolescents by region Children and adolescents were aged 5–19 years. See appendix for results for adults. BMI=body-mass index.

**Figure 9:**
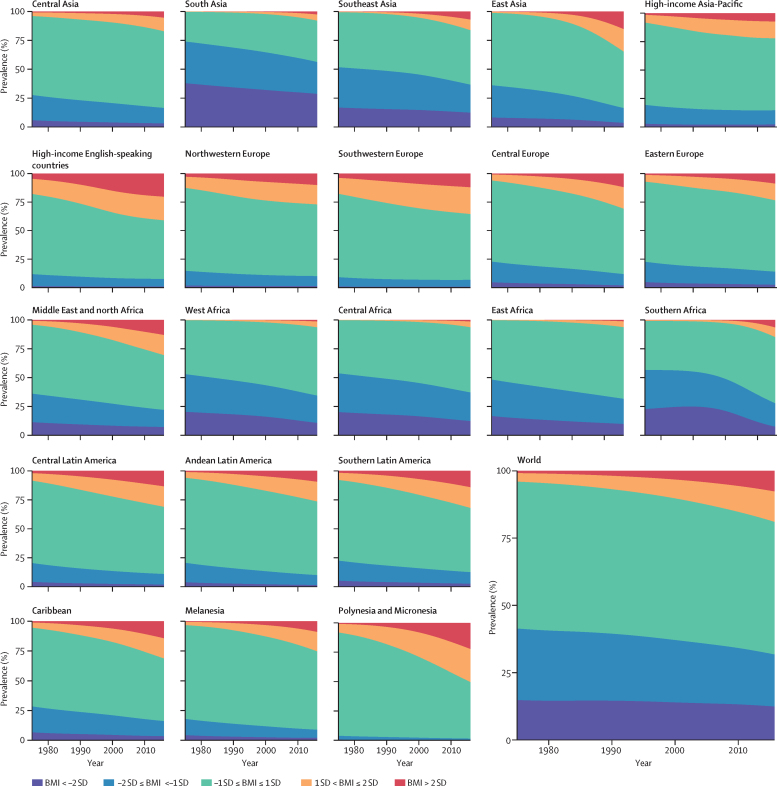
Trends in age-standardised prevalence of BMI categories in male children and adolescents by region Children and adolescents were aged 5–19 years. See appendix for results for adults. BMI=body-mass index.

Over the 42 years of analysis, the global age-standardised prevalence of obesity in children and adolescents increased from 0·7% (95% CrI 0·4–1·2) in 1975 to 5·6% (4·8–6·5) in 2016 in girls (figure 8), and from 0·9% (0·5–1·3) in 1975 to 7·8% (6·7–9·1) in 2016 in boys (figure 9). Obesity increased in every region, with proportional rise being smallest in high-income regions (on average 30–50% per decade) and largest in southern Africa (about 400% per decade, albeit from very low levels).

Globally, the prevalence of moderate and severe underweight changed less than the rise in obesity, from 9·2% (95% CrI 6·0–12·9) in 1975 to 8·4% (6·8–10·1) in 2016 in girls and from 14·8% (10·4–19·5) in 1975 to 12·4% (10·3–14·5) in 2016 in boys. The relatively small change in moderate and severe underweight prevalence at the global level, however, was partly due to faster population growth in regions where underweight prevalence is higher (eg, the share of children and adolescents living in south Asia, where prevalence is highest, increased from 20·5% in 1975 to 26·4% in 2016 in girls, and 21·1% in 1975 to 27·1% in 2016 in boys) while prevalence declined in most regions. The largest proportional decline in the prevalence of moderate and severe underweight occurred in Polynesia and Micronesia and in southern Africa in both sexes, where prevalence declined by an average of up to one third per decade for girls and by about one fifth per decade for boys from 1975 to 2016 (Figure 8, Figure 9). There was a non-significant rise of about 6% per decade (PP=0·6630) in underweight in girls in southeast Asia. Nonetheless, in most regions, the increase in the prevalence of overweight and obesity was larger than the decline in the prevalence of underweight (Figure 8, Figure 9), ie, the width of the BMI distribution increased.

Regionally, moderate and severe underweight prevalence was highest in south Asia over the entire analysis period, at 20·3% (95% CrI 15·3–25·8) in girls and 28·6% (22·3–35·0) in boys in 2016, having decreased from 23·0% (13·9–33·6) in girls and 37·8% (26·6–49·2) in boys in 1975. Prevalence of obesity was highest in Polynesia and Micronesia in both sexes, 25·4% (16·8–35·2) in girls and 22·4% (13·4–32·9) in boys, followed by the high-income English-speaking region.

Nationally, the prevalence of moderate and severe underweight was less than 1% among girls in 45 countries and among boys in 29 countries in 2016 (figure 3). Prevalence of moderate and severe underweight was high throughout south Asia, reaching 22·7% (95% CrI 16·7–29·6) among girls and 30·7% (23·5–38·0) among boys in India. Obesity prevalence was between 1% and 2% among girls in Cambodia, Burkina Faso, Vietnam, Ethiopia, India, Madagascar, Republic of the Congo, Japan, Nepal, Niger, and Chad. Obesity prevalence was less than 1% among boys in Uganda, Rwanda, Niger, Burkina Faso, Ethiopia, Guinea, Chad, and Senegal and between 1% and 2% in another 24 countries.

Conversely, obesity prevalence was more than 30% in girls in Nauru, the Cook Islands, and Palau and boys in the Cook Islands, Nauru, Palau, Niue, and American Samoa in 2016, and was also high, around or above 20%, in some countries in Polynesia and Micronesia, the Middle East and north Africa (eg, Egypt, Kuwait, Qatar, and Saudi Arabia), the Caribbean (Bermuda and Puerto Rico), and in the USA. In 1975, obesity prevalence was less than 10% in every country except Nauru and Bermuda, where it was still less than 20%. From 1975 to 2016, obesity prevalence increased in every country, although the increase was not statistically significant in some high-income countries.

**Figure 10:**
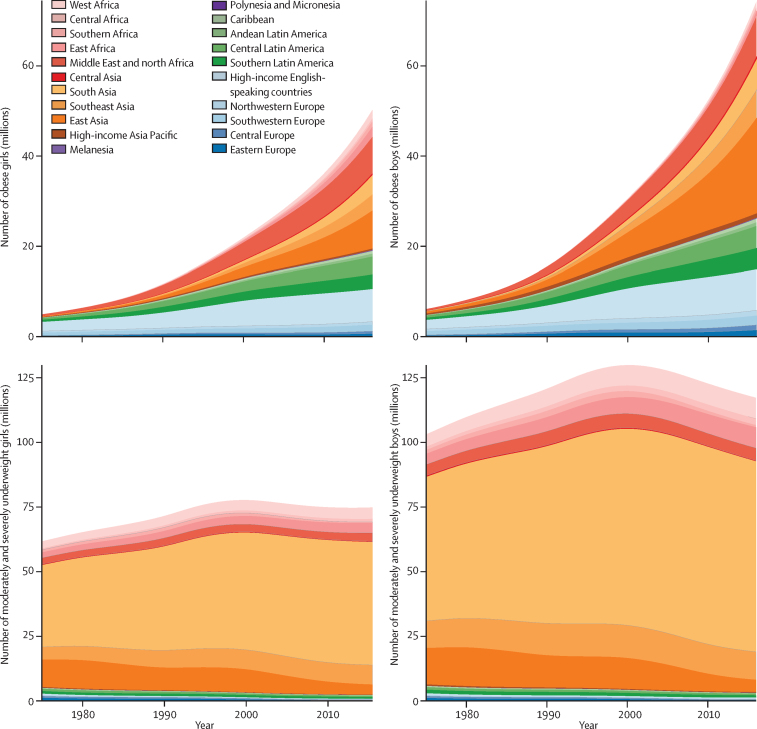
Trends in the number of children and adolescents with obesity and with moderate and severe underweight by region Children and adolescents were aged 5–19 years. See appendix for results for adults. BMI=body-mass index.

The number of moderately and severely underweight girls and boys worldwide peaked around the year 2000, and subsequently decreased to 75 (95% CrI 44–117) million girls and 117 (70–178) million boys in 2016, slightly higher than in 1975 (figure 10). In most regions, the number of moderately and severely underweight children and adolescents decreased despite population growth. The exceptions were south Asia; southeast Asia; and central, east, and west Africa, where population growth led to an increase in the absolute underweight burden, despite declining prevalence. 47·5 million (63%) of 75 million moderately and severely underweight girls and 73·6 million (63%) of 117 million underweight boys in the world lived in south Asia in 2016, substantially higher than its 27% share of the child and adolescent population.

The number of girls with obesity increased from 5 (95% CrI 1–14) million in 1975 to 50 (24–89) million in 2016. The number of boys with obesity increased from 6 (1–19) million in 1975 to 74 (39–125) million in 2016. 73% of the increase in the number of children and adolescents with obesity was due to increase in prevalence of obesity, 3% due to population growth and changes in age structure of the child and adolescent population, and another 24% due to the interaction of the two (appendix). The regions with the largest absolute increase in the number of children and adolescents with obesity were east Asia, the Middle East and north Africa, south Asia, and the high-income English-speaking region. The worldwide number of adult women with obesity increased from 69 (57–83) million in 1975 to 390 (363–418) million in 2016; the number of men with obesity increased from 31 (24–39) million in 1975 to 281 (257–307) million in 2016. An additional 213 million children and adolescents and 1·30 billion adults were in the overweight range, but below the threshold for obesity.

## Discussion

Mean BMI and prevalence of obesity increased worldwide in children and adolescents from 1975 to 2016, with the rate of change in mean BMI moderately correlated with that of adults until around 2000, but only weakly correlated afterwards. The trend in children's and adolescents' mean BMI has plateaued, albeit at high levels, in many high-income countries since around 2000, but has accelerated in east, south, and southeast Asia. Despite this rise, more children and adolescents worldwide are moderately or severely underweight than obese. However, if post-2000 trends continue, child and adolescent obesity is expected to surpass moderate and severe underweight by 2022.

No prior global analyses of mean BMI and underweight have been done in children aged over 5 years and adolescents. For overweight and obesity, our results are not directly comparable with those of Ng and colleagues^31^ because the two studies covered different age ranges (2–19 years in Ng and colleagues'^31^ study compared with 5–19 years in our study), used different classification systems for defining overweight and obesity (WHO in our study versus International Obesity Task Force [IOTF], by Ng and colleagues^31^), and differed in criteria for including data (only measured height and weight in our study versus measured and self-reported by Ng and colleagues^31^). Nonetheless, both studies concluded that the rise in excess weight in children and adolescents has plateaued in high-income countries but continues in low-income and middle-income countries. The plateau in children's and adolescents' overweight and obesity in high-income countries^32^, ^33^, ^34^ and the relatively rapid transition from underweight to overweight and obesity in low-income and middle-income countries^35^, ^36^ have also been noted in specific countries.

Our study is the first to make comparable estimates of mean BMI and the prevalence of a complete set of BMI categories with clinical and public health relevance—from underweight to obesity. We used an unprecedented amount of population-based data from almost all of the world's countries, while maintaining a high standard of data quality and using only measured height and weight data to avoid the bias in self-reported data. Characteristics of data sources were verified through repeated checks by NCD-RisC members, and data that could be systematically different from the general population were excluded, eg, those from samples of students or of ever-married women in ages and countries with low school enrolment or marriage rates. Data were analysed according to a common protocol to obtain the required mean and prevalence by age and sex. Finally, we used a statistical model that used all available data while giving more weight to national data than subnational and community studies, and took into account the epidemiological features of outcomes such as BMI by using non-linear time trends and age associations, and differences between rural and urban populations.

Despite using the most comprehensive global database of human anthropometry to date, some countries and regions had fewer data sources, especially those in the Caribbean, Polynesia and Micronesia, Melanesia, and central Asia. The scarcity of data is reflected in wider uncertainty intervals of our estimates for these countries and regions. Of sources with data on children and adolescents, 39·9% had data for 5–9 years of age, 50·3% for 10–14 years, and 78·9% for 15–19 years. Many sources with data on children aged 5–9 years were school-based measurement studies in high-income countries where school enrolment is almost universal. The relative paucity of data on children aged 5–9 years restricted our capacity to compare trends in this age group with those of adolescents, despite some evidence from high-income countries that trends are somewhat different before and after 10 years of age.^33^ Finally, although the age-dependent cutoffs for defining overweight and obesity in children and adolescents reflect natural growth in these ages, they are based on BMI distributions in a reference population, and not explicitly selected to represent optimal BMI levels for health in prospective studies, as done for adults, or optimal nutritional status, as done for children younger than 5 years. The reference population used by WHO,^16^ and the cutoffs for defining overweight and obesity, differ from those used by IOTF^37^, ^38^ and the US Centers for Disease Control and Prevention (CDC). Specifically, in the WHO classification, a BMI of 30 kg/m^2^ at ages 18–19 years corresponds to 2 SD (ie, about the 97·5th percentile) from the median of the reference population;^16^ in the IOTF classification, a BMI of 30 kg/m^2^ at age 18 years corresponds to the 98·6th percentile for girls and the 98·9th percentile for boys.^38^ While at 18 years the two systems classify the same children as obese, at younger ages a smaller proportion are classified as obese according to the IOTF definition compared with the WHO definition.^38^ For this reason, comparisons of overweight and obesity prevalence based on the three definitions^36^, ^39^, ^40^, ^41^, ^42^ found that prevalence using the WHO classification was higher than those of IOTF and CDC, but that trends are similar.

The effectiveness of interventions for overweight and obesity in children and adolescents has been reviewed in several systematic reviews and modelling studies,^3^, ^43^, ^44^, ^45^, ^46^, ^47^ but how they are selected for implementation and their post-implementation effects at the population level are rarely investigated.^48^ For this reason, there is no systematic information on the determinants of the divergent trends in BMI in children and adolescents and in adults, be it on food environments and behaviours or on policies that affect them. The plateauing of children's and adolescents' BMI in high-income countries as adult BMI continues to increase might be due to specific initiatives by governments, community groups, schools, and notable individuals that have increased public awareness about overweight and obesity in children, leading to changes in nutrition and activity that are sufficient to curb the rise in mean BMI.

A general feature of policies that target overweight and obesity in children and adolescents in high-income countries is a reluctance to use taxes and industry regulations to change eating and drinking behaviours.^12^, ^49^ Some middle-income countries are also adopting policies to combat overweight and obesity in children and adolescents, in some cases with a stronger emphasis on regulation and taxes than in high-income countries.^48^ While momentum might be gathering to use taxes and regulations to reduce the consumption of energy-dense foods, few policies and programmes attempt to make healthy foods such as whole grains and fresh fruits and vegetables more affordable through targeted price subsidies, (conditional) cash transfers and food vouchers, or healthy school meals.^50^ Unaffordability of healthy food options not only leads to social inequalities in overweight and obesity,^51^, ^52^ but might also limit the effect of policies that target unhealthy foods. Finally, efforts in population-based prevention of overweight and obesity in children and adolescents should be matched with enhancing access to health-care interventions for weight management and for reducing the adverse effects of obesity, including intensive behavioural therapy to change diet and exercise; screening for and management of hypertension, glucose intolerance, dyslipidaemia, and abnormal liver function in children and adolescents with obesity; and in extreme cases bariatric surgery.^4^, ^53^, ^54^

Our finding that the number of children and adolescents aged 5–19 years in the world who are moderately or severely underweight remains larger than those who are obese shows the continued need for policies that enhance food security in low-income countries and households, especially in south Asia. Yet the experiences of east Asia and Latin America and the Caribbean show that the transition from underweight to overweight and obesity can be rapid, and overwhelm the national capacity needed to engender a healthy transition. More broadly, in an unhealthy nutritional transition, an increase in nutrient-poor, energy-dense foods can lead to stunted growth along with weight gain in children, adolescents, and adults, resulting in higher BMI and worse health outcomes throughout the life-course. Therefore, the findings from our comprehensive analysis of trends in underweight, as well as overweight and obesity highlight the disconnect between the global dialogue on overweight and obesity, which has largely overlooked the remaining undernutrition burden, and the initiatives and donors focusing on undernutrition that have paid little attention to the looming burden of overweight and obesity, itself a risk factor for adverse pregnancy outcomes.^2^ The Sustainable Development Goals, which address poverty, education, nutrition, and universal health coverage, provide an opportunity for integrating policies that coherently address underweight and overweight in children and adolescents, and their health consequences, effectively and equitably. Doing so would require commitment from national and international agencies and donors for replacing the fragmented focus with an integrated approach.

Correspondence to: Prof Majid Ezzati, School of Public Health, Imperial College London, London W2 1PG, UK **majid.ezzati@imperial.ac.uk**
