# Supplementary material for: Worldwide trends in body-mass index, underweight, overweight, and obesity from 1975 to 2016: a pooled analysis of 2416 population-based measurement studies in 128·9 million children, adolescents, and adults
Source: Lancet. 2017 Dec 16;390(10113):2627–42. doi: 10.1016/S0140-6736(17)32129-3 (PMC5735219; doi:10.1016/S0140-6736(17)32129-3)
Supplement: Supplementary appendix [file mmc1.pdf]

THE LANCET

Supplementary appendix

This appendix formed part of the original submission and has been peer reviewed. We post it as supplied by the authors.

Supplement to: NCD Risk Factor Collaboration (NCD-RisC). Worldwide trends in body-mass index, underweight, overweight, and obesity from 1975 to 2016: a pooled analysis of 2416 population-based measurement studies in 128·9 million children, adolescents, and adults. *Lancet* 2017; published online Oct 10. http://dx.doi.org/10.1016/S0140-6736(17)32129-3.

Appendix

Worldwide trends in body-mass index, underweight, overweight, and obesity from 1975 to 2016: a pooled analysis of 2416 population-based measurement studies in 128.9 million children, adolescents, and adults

NCD Risk Factor Collaboration (NCD-RisC)

NCD Risk Factor Collaboration (NCD-RisC)

Pooled Analysis and Writing (* joint first authors)

James Bentham (University of Kent, UK; Imperial College London, UK)*; Mariachiara Di Cesare (Middlesex University, UK)*; Ver Bilano (Imperial College London, UK)*; Honor Bixby (Imperial College London, UK)*; Bin Zhou (Imperial College London, UK)*; Gretchen A Stevens (World Health Organization, Switzerland); Leanne M Riley (World Health Organization, Switzerland); Cristina Taddei (Imperial College London, UK); Kaveh Hajifathalian (Cleveland Clinic, USA); Yuan Lu (Yale University, USA); Stefan Savin (World Health Organization, Switzerland); Melanie J Cowan (World Health Organization, Switzerland); Christopher J Paciorek (University of California, Berkeley, USA); Adela Chirita-Emandi (Victor Babeș University of Medicine and Pharmacy Timișoara, Romania); Alison J Hayes (University of Sydney, Australia); Joanne Katz (Johns Hopkins University, USA); Roya Kelishadi (Isfahan University of Medical Sciences, Iran); Andre Pascal Kengne (South African Medical Research Council, South Africa); Young-Ho Khang (Seoul National University, South Korea); Avula Laxmaiah (National Institute of Nutrition, India); Yanping Li (Harvard TH Chan School of Public Health, USA); Jun Ma (Peking University, China); J Jaime Miranda (Universidad Peruana Cayetano Heredia, Peru); Aya Mostafa (Ain Shams University, Egypt); Martin Neovius (Karolinska Institutet, Sweden); Cristina Padez (University of Coimbra, Portugal); Lekhraj Rampal (Universiti Putra Malaysia, Malaysia); Aubrianna Zhu (Imperial College London, UK); James E Bennett (Imperial College London, UK); Goodarz Danaei (Harvard TH Chan School of Public Health, USA); Zulfiqar A Bhutta (The Hospital for Sick Children, Canada; Aga Khan University, Pakistan); Majid Ezzati (Imperial College London, UK)

Country and Regional Data (* equal contribution; listed alphabetically)

Leandra Abarca-Gómez (Caja Costarricense de Seguro Social, Costa Rica)*; Ziad A Abdeen (Al-Quds University, Palestine)*; Zargar Abdul Hamid (Center for Diabetes and Endocrine Care, India)*; Niveen M Abu-Rmeileh (Birzeit University, Palestine)*; Benjamin Acosta-Cazares (Instituto Mexicano del Seguro Social, Mexico)*; Cecilia Acuin (International Rice Research Institute, Philippines)*; Robert J Adams (The University of Adelaide, Australia)*; Wichai Aekplakorn (Mahidol University, Thailand)*; Kaosar Afsana (BRAC, Bangladesh)*; Carlos A Aguilar-Salinas (Instituto Nacional de Ciencias Médicas y Nutricion, Mexico)*; Charles Agyemang (University of Amsterdam, The Netherlands)*; Alireza Ahmadvand (Non-Communicable Diseases Research Center, Iran)*; Wolfgang Ahrens (Leibniz Institute for Prevention Research and Epidemiology - BIPS, Germany)*; Kamel Ajlouni (National Center for Diabetes and Endocrinology, Jordan)*; Nazgul Akhtaeva (Kazakh National Medical University, Kazakhstan)*; Hazzaa M Al-Hazzaa (King Saud University, Saudi Arabia)*; Amani Rashed Al-Othman (Kuwait Institute for Scientific Research, Kuwait)*; Rajaa Al-Raddadi (Ministry of Health, Saudi Arabia)*; Fadia AlBuhairan (King Abdullah International Medical Research Center, Saudi Arabia)*; Shahla AlDhukair (King Abdullah International Medical Research Center, Saudi Arabia)*; Mohamed M Ali (World Health Organization, Switzerland)*; Osman Ali (Universiti Malaysia Sabah, Malaysia)*; Ala'a Alkerwi (Luxembourg Institute of Health, Luxembourg)*; Mar Alvarez-Pedrerol (ISGlobal Centre for Research in Environmental Epidemiology, Spain)*; Eman Aly (World Health Organization Regional Office for the Eastern Mediterranean, Egypt)*; Deepak N Amarapurkar (Bombay Hospital and Medical Rsearch Centre, India)*; Philippe Amouyel (Lille University and Hospital, France)*; Antoinette Amuzu (London School of Hygiene & Tropical Medicine, UK)*; Lars Bo Andersen (Western Norway University of Applied Sciences, Norway)*; Sigmund A Anderssen (Norwegian School of Sport Sciences, Norway)*; Dolores S Andrade

(Universidad de Cuenca, Ecuador)*; Lars H Ängquist (Bispebjerg and Frederiksberg Hospitals, Denmark)*; Ranjit Mohan Anjana (Madras Diabetes Research Foundation, India)*; Hajer Aounallah-Skhiri (National Institute of Public Health, Tunisia)*; Joana Araújo (Universidade do Porto, Portugal)*; Inger Ariansen (Norwegian Institute of Public Health, Norway)*; Tahir Aris (Ministry of Health Malaysia, Malaysia)*; Nimmathota Arlappa (National Institute of Nutrition, India)*; Dominique Arveiler (University of Strasbourg and Strasbourg University Hospital, France)*; Krishna K Aryal (Nepal Health Research Council, Nepal)*; Thor Aspelund (University of Iceland, Iceland)*; Felix K Assah (University of Yaoundé 1, Cameroon)*; Maria Cecília F Assunção (Federal University of Pelotas, Brazil)*; May Soe Aung (University of Medicine 1, Myanmar)*; Mária Avdicová (Banska Bystrica Regional Authority of Public Health, Slovakia)*; Ana Azevedo (University of Porto Medical School, Portugal)*; Fereidoun Azizi (Shahid Beheshti University of Medical Sciences, Iran)*; Bontha V Babu (Indian Council of Medical Research, India)*; Suhad Bahijri (King Abdulaziz University, Saudi Arabia)*; Jennifer L Baker (Bispebjerg and Frederiksberg Hospital, Denmark)*; Nagalla Balakrishna (National Institute of Nutrition, India)*; Mohamed Bamoshmoosh (University of Science and Technology, Yemen)*; Maciej Banach (Medical University of Lodz, Poland)*; Piotr Bandosz (Medical University of Gdansk, Poland)*; José R Banegas (Universidad Autónoma de Madrid, Spain)*; Carlo M Barbagallo (University of Palermo, Italy)*; Alberto Barceló (Pan American Health Organization, USA)*; Amina Barkat (Mohammed V University de Rabat, Morocco)*; Aluisio JD Barros (Federal University of Pelotas, Brazil)*; Mauro VG Barros (University of Pernambuco, Brazil)*; Iqbal Bata (Dalhousie University, Canada)*; Anwar M Batieha (Jordan University of Science and Technology, Jordan)*; Rosangela L Batista (Federal University of Maranhao, Brazil)*; Assembekov Batyrbek (Kazakh National Medical University, Kazakhstan)*; Louise A Baur (University of Sydney, Australia)*; Robert Beaglehole (University of Auckland, New Zealand)*; Habiba Ben Romdhane (University Tunis El Manar,

Tunisia)*; Judith Benedics (Federal Ministry of Health and Women's Affairs, Austria)*; Mikhail Benet (CAFAM University Foundation, Colombia)*; James E Bennett (Imperial College London, UK)*; Antonio Bernabe-Ortiz (Universidad Peruana Cayetano Heredia, Peru)*; Gailute Bernotiene (Lithuanian University of Health Sciences, Lithuania)*; Heloisa Bettiol (University of São Paulo, Brazil)*; Aroor Bhagyalaxmi (B J Medical College, India)*; Sumit Bharadwaj (Chirayu Medical College, India)*; Santosh K Bhargava (Sunder Lal Jain Hospital, India)*; Zaid Bhatti (Aga Khan University, Pakistan)*; Zulfiqar A Bhutta (The Hospital for Sick Children, Canada; Aga Khan University, Pakistan)*; Hongsheng Bi (Shandong University of Traditional Chinese Medicine, China)*; Yufang Bi (Shanghai Jiao-Tong University School of Medicine, China)*; Anna Biehl (Norwegian Institute of Public Health, Norway)*; Mukharram Bikbov (Ufa Eye Research Institute, Russia)*; Bihungum Bista (Nepal Health Research Council, Nepal)*; Dusko J Bjelica (University of Montenegro, Montenegro)*; Peter Bjerregaard (University of Southern Denmark, Denmark; University of Greenland, Greenland)*; Espen Bjertness (University of Oslo, Norway)*; Marius B Bjertness (University of Oslo, Norway)*; Cecilia Björkelund (University of Gothenburg, Sweden)*; Anneke Blokstra (National Institute for Public Health and the Environment, The Netherlands)*; Simona Bo (University of Turin, Italy)*; Martin Bobak (University College London, UK)*; Lynne M Boddy (Liverpool John Moores University, UK)*; Bernhard O Boehm (Nanyang Technological University, Singapore)*; Heiner Boeing (German Institute of Human Nutrition, Germany)*; Jose G Boggia (Universidad de la República, Uruguay)*; Carlos P Boissonnet (CEMIC, Argentina)*; Marialaura Bonaccio (IRCCS Istituto Neurologico Mediterraneo Neuromed, Italy)*; Vanina Bongard (Toulouse University School of Medicine, France)*; Pascal Bovet (Ministry of Health, Seychelles; University of Lausanne, Switzerland)*; Lien Braeckevelt (Flemish Agency for Care and Health, Belgium)*; Lutgart Braeckman (Ghent University, Belgium)*; Marjolijn CE Bragt (FrieslandCampina, The

Netherlands)*; Imperia Brajkovich (Universidad Central de Venezuela, Venezuela)*; Francesco Branca (World Health Organization, Switzerland)*; Juergen Breckenkamp (Bielefeld University, Germany)*; João Breda (World Health Organization Regional Office for Europe, Denmark)*; Hermann Brenner (German Cancer Research Center, Germany)*; Lizzy M Brewster (University of Amsterdam, The Netherlands)*; Garry R Brian (The Fred Hollows Foundation New Zealand, New Zealand)*; Lacramioara Brinduse (Carol Davila University of Medicine and Pharmacy, Romania)*; Graziella Bruno (University of Turin, Italy)*; H B(as) Bueno-de-Mesquita (National Institute for Public Health and the Environment, The Netherlands)*; Anna Bugge (University of Southern Denmark, Denmark)*; Marta Buoncristiano (World Health Organization Regional Office for Europe, Denmark)*; Genc Burazeri (Institute of Public Health, Albania)*; Con Burns (Cork Institute of Technology, Ireland)*; Antonio Cabrera de León (Universidad de La Laguna, Spain)*; Joseph Cacciottolo (University of Malta, Malta)*; Hui Cai (Vanderbilt University, USA)*; Tilema Cama (Ministry of Health, Tonga)*; Christine Cameron (Canadian Fitness and Lifestyle Research Institute, Canada)*; José Camolas (Hospital Santa Maria, Portugal)*; Günay Can (Istanbul University, Turkey)*; Ana Paula C Cândido (Universidade Federal de Juiz de Fora, Brazil)*; Mario Capanzana (Food and Nutrition Research Institute, Philippines)*; Vincenzo Capuano (Cardiologia di Mercato S. Severino, Italy)*; Viviane C Cardoso (University of São Paulo, Brazil)*; Axel C Carlsson (Karolinska Institutet, Sweden)*; Maria J Carvalho (University of Porto, Portugal)*; Felipe F Casanueva (Santiago de Compostela University, Spain)*; Juan-Pablo Casas (University College London, UK)*; Carmelo A Caserta (Associazione Calabrese di Epatologia, Italy)*; Snehalatha Chamukuttan (India Diabetes Research Foundation, India)*; Angelique W Chan (Duke-NUS Medical School, Singapore)*; Queenie Chan (Imperial College London, UK)*; Himanshu K Chaturvedi (National Institute of Medical Statistics, India)*; Nishi Chaturvedi (University College London, UK)*; Chien-Jen Chen (Academia

Sinica, Taiwan)*; Fangfang Chen (Capital Institute of Pediatrics, China)*; Huashuai Chen (Duke University, USA)*; Shuohua Chen (Kailuan General Hospital, China)*; Zhengming Chen (University of Oxford, UK)*; Ching-Yu Cheng (Duke-NUS Medical School, Singapore)*; Angela Chetrit (The Gertner Institute for Epidemiology and Health Policy Research, Israel)*; Ekaterina Chikova-Iscener (National Centre of Public Health and Analyses, Bulgaria)*; Arnaud Chiolero (University of Bern, Switzerland)*; Shu-Ti Chiou (Ministry of Health and Welfare, Taiwan)*; Adela Chirita-Emandi (Victor Babeș University of Medicine and Pharmacy Timisoara, Romania)*; María-Dolores Chirlaque (Murcia Regional Health Council, Spain)*; Belong Cho (Seoul National University College of Medicine, South Korea)*; Yumi Cho (Korea Centers for Disease Control and Prevention, South Korea)*; Kaare Christensen (University of Southern Denmark, Denmark)*; Diego G Christofaro (Universidade Estadual Paulista, Brazil)*; Jerzy Chudek (Medical University of Silesia, Poland)*; Renata Cifkova (Charles University in Prague, Czech Republic)*; Eliza Cinteza (Carol Davila University of Medicine and Pharmacy, Romania)*; Frank Claessens (Katholieke Universiteit Leuven, Belgium)*; Els Clays (Ghent University, Belgium)*; Hans Concin (Agency for Preventive and Social Medicine, Austria)*; Susana C Confortin (Universidade Federal de Santa Catarina, Brazil)*; Cyrus Cooper (University of Southampton, UK)*; Rachel Cooper (University College London, UK)*; Tara C Coppinger (Cork Institute of Technology, Ireland)*; Simona Costanzo (IRCCS Istituto Neurologico Mediterraneo Neuromed, Italy)*; Dominique Cottel (Institut Pasteur de Lille, France)*; Chris Cowell (University of Sydney, Australia)*; Cora L Craig (Canadian Fitness and Lifestyle Research Institute, Canada)*; Ana B Crujeiras (CIBEROBN, Spain)*; Alexandra Cucu (National Institute of Public Health, Romania)*; Graziella D'Arrigo (National Council of Research, Italy)*; Eleonora d'Orsi (Universidade Federal de Santa Catarina, Brazil)*; Jean Dallongeville (Institut Pasteur de Lille, France)*; Albertino Damasceno (Eduardo Mondlane University, Mozambique)*; Camilla T

Damsgaard (University of Copenhagen, Denmark)*; Goodarz Danaei (Harvard TH Chan School of Public Health, USA)*; Rachel Dankner (The Gertner Institute for Epidemiology and Health Policy Research, Israel)*; Thomas M Dantoft (Research Centre for Prevention and Health, Denmark)*; Saeed Dastgiri (Tabriz Health Services Managemnet Centre, Iran)*; Luc Dauchet (Lille University Hospital, France)*; Kairat Davletov (Kazakh National Medical University, Kazakhstan)*; Guy De Backer (Ghent University, Belgium)*; Dirk De Bacquer (Ghent University, Belgium)*; Amalia De Curtis (IRCCS Istituto Neurologico Mediterraneo Neuromed, Italy)*; Giovanni de Gaetano (IRCCS Istituto Neurologico Mediterraneo Neuromed, Italy)*; Stefaan De Henauw (Ghent University, Belgium)*; Paula Duarte de Oliveira (Federal University of Pelotas, Brazil)*; Karin De Ridder (Scientific Institute of Public Health, Belgium)*; Delphine De Smedt (Ghent University, Belgium)*; Mohan Deepa (Madras Diabetes Research Foundation, India)*; Alexander D Deev (National Research Centre for Preventive Medicine, Russia)*; Abbas Dehghan (Erasmus Medical Center Rotterdam, The Netherlands)*; Hélène Delisle (University of Montreal, Canada)*; Francis Delpeuch (Institut de Recherche pour le Développement, France)*; Valérie Deschamps (French Public Health Agency, France)*; Klodian Dhana (Erasmus Medical Center Rotterdam, The Netherlands)*; Augusto F Di Castelnuovo (IRCCS Istituto Neurologico Mediterraneo Neuromed, Italy)*; Juvenal Soares Dias-da-Costa (Universidade do Vale do Rio dos Sinos, Brazil)*; Alejandro Diaz (National Council of Scientific and Technical Research, Argentina)*; Zivka Dika (University of Zagreb, Croatia)*; Shirin Djalalinia (Tehran University of Medical Sciences, Iran)*; Ha TP Do (National Institute of Nutrition, Vietnam)*; Annette J Dobson (University of Queensland, Australia)*; Maria Benedetta Donati (IRCCS Istituto Neurologico Mediterraneo Neuromed, Italy)*; Chiara Donfrancesco (Istituto Superiore di Sanità, Italy)*; Silvana P Donoso (Universidad de Cuenca, Ecuador)*; Angela Döring (Helmholtz Zentrum München, Germany)*; Maria Dorobantu (Carol Davila University of Medicine and Pharmacy,

Romania)*; Ahmad Reza Dorosty (Tehran University of Medical Sciences, Iran)*; Kouamelan Doua (Ministère de la Santé et de la Lutte Contre le Sida, Côte d'Ivoire)*; Wojciech Drygas (The Cardinal Wyszynski Institute of Cardiology, Poland)*; Jia Li Duan (Beijing Center for Disease Prevention and Control, China)*; Charmaine Duante (Food and Nutrition Research Institute, Philippines)*; Vesselka Duleva (National Centre of Public Health and Analyses, Bulgaria)*; Virginija Dulskiene (Lithuanian University of Health Sciences, Lithuania)*; Vilnis Dzerve (University of Latvia, Latvia)*; Elzbieta Dziankowska-Zaborszczyk (Medical University of Lodz, Poland)*; Eruke E Egbagbe (University of Benin, Nigeria)*; Robert Eggertsen (University of Gothenburg, Sweden)*; Gabriele Eiben (University of Skövde, Sweden)*; Ulf Ekelund (Norwegian School of Sport Sciences, Norway)*; Jalila El Ati (National Institute of Nutrition and Food Technology, Tunisia)*; Paul Elliott (Imperial College London, UK)*; Reina Engle-Stone (University of California Davis, USA)*; Rajiv T Erasmus (University of Stellenbosch, South Africa)*; Cihangir Erem (Karadeniz Technical University, Turkey)*; Louise Eriksen (University of Southern Denmark, Denmark)*; Johan G Eriksson (National Institute for Health and Welfare, Finland)*; Jorge Escobedo-de la Peña (Instituto Mexicano del Seguro Social, Mexico)*; Alun Evans (The Queen's University of Belfast, UK)*; David Faeh (University of Zurich, Switzerland)*; Caroline H Fall (University of Southampton, UK)*; Victoria Farrugia Sant'Angelo (Primary Health Care, Malta)*; Farshad Farzadfar (Tehran University of Medical Sciences, Iran)*; Francisco J Felix-Redondo (Centro de Salud Villanueva Norte, Spain)*; Trevor S Ferguson (The University of the West Indies, Jamaica)*; Romulo A Fernandes (Universidade Estadual Paulista, Brazil)*; Daniel Fernández-Bergés (Hospital Don Benito-Villanueva de la Serena, Spain)*; Daniel Ferrante (Ministry of Health, Argentina)*; Marika Ferrari (Council for Agricultural Research and Economics, Italy)*; Catterina Ferreccio (Pontificia Universidad Católica de Chile, Chile)*; Jean Ferrieres (Toulouse University School of Medicine, France)*; Joseph D Finn (University of Manchester,

UK)*; Krista Fischer (University of Tartu, Estonia)*; Eric Monterubio Flores (Instituto Nacional de Salud Pública, Mexico)*; Bernhard Föger (Agency for Preventive and Social Medicine, Austria)*; Leng Huat Foo (Universiti Sains Malaysia, Malaysia)*; Ann-Sofie Forslund (Umeå University, Sweden)*; Maria Forsner (Dalarna University, Sweden)*; Heba M Fouad (World Health Organization Regional Office for the Eastern Mediterranean, Egypt)*; Damian K Francis (The University of the West Indies, Jamaica)*; Maria do Carmo Franco (Federal University of São Paulo, Brazil)*; Oscar H Franco (Erasmus Medical Center Rotterdam, The Netherlands)*; Guillermo Frontera (Hospital Universitario Son Espases, Spain)*; Flavio D Fuchs (Hospital de Clinicas de Porto Alegre, Brazil)*; Sandra C Fuchs (Universidade Federal do Rio Grande do Sul, Brazil)*; Yuki Fujita (Kindai University, Japan)*; Takuro Furusawa (Kyoto University, Japan)*; Zbigniew Gaciong (Medical University of Warsaw, Poland)*; Mihai Gafencu (Victor Babeș University of Medicine and Pharmacy Timisoara, Romania)*; Daniela Galeone (Ministry of Health, Italy)*; Fabio Galvano (University of Catania, Italy)*; Manoli Garcia-de-la-Hera (CIBER en Epidemiología y Salud Pública, Spain)*; Dickman Gareta (University of KwaZulu-Natal, South Africa)*; Sarah P Garnett (University of Sydney, Australia)*; Jean-Michel Gaspoz (Geneva University Hospitals, Switzerland)*; Magda Gasull (CIBER en Epidemiología y Salud Pública, Spain)*; Louise Gates (Australian Bureau of Statistics, Australia)*; Harald Geiger (Agency for Preventive and Social Medicine, Austria)*; Johanna M Geleijnse (Wageningen University, The Netherlands)*; Anoosheh Ghasemian (Non-Communicable Diseases Research Center, Iran)*; Simona Giampaoli (Istituto Superiore di Sanità, Italy)*; Francesco Gianfagna (University of Insubria, Italy; IRCCS Istituto Neurologico Mediterraneo Neuromed, Italy)*; Tiffany K Gill (The University of Adelaide, Australia)*; Jonathan Giovannelli (Lille University Hospital, France)*; Aleksander Giwercman (Lund University, Sweden)*; Justyna Godos (University of Catania, Italy)*; Sibel Gogen (Ministry of Health, Turkey)*; Rebecca A Goldsmith (Ministry

of Health, Israel)*; David Goltzman (McGill University, Canada)*; Helen Gonçalves (Federal University of Pelotas, Brazil)*; Margot González-Leon (Instituto Mexicano del Seguro Social, Mexico)*; Juan P González-Rivas (The Andes Clinic of Cardio-Metabolic Studies, Venezuela)*; Marcela Gonzalez-Gross (Universidad Politécnica de Madrid, Spain)*; Frederic Gottrand (Université de Lille 2, France)*; Antonio Pedro Graça (Ministry of Health, Portugal)*; Sidsel Graff-Iversen (Norwegian Institute of Public Health, Norway)*; Dušan Grafnetter (Institute for Clinical and Experimental Medicine, Czech Republic)*; Aneta Grajda (The Children's Memorial Health Institute, Poland)*; Maria G Grammatikopoulou (Alexander Technological Educational Institute, Greece)*; Ronald D Gregor (Dalhousie University, Canada)*; Tomasz Grodzicki (Jagiellonian University Medical College, Poland)*; Anders Grøntved (University of Southern Denmark, Denmark)*; Giuseppe Grosso (Azienda Ospedaliera Universitaria Policlinico Vittorio Emanuele, Italy)*; Gabriella Gruden (University of Turin, Italy)*; Vera Grujic (University of Novi Sad, Serbia)*; Dongfeng Gu (National Center of Cardiovascular Diseases, China)*; Emanuela Gualdi-Russo (University of Ferrara, Italy)*; Pilar Guallar-Castillón (Universidad Autónoma de Madrid, Spain)*; Ong Peng Guan (Singapore Eye Research Institute, Singapore)*; Elias F Gudmundsson (Icelandic Heart Association, Iceland)*; Vilmundur Gudnason (University of Iceland, Iceland)*; Ramiro Guerrero (Universidad Icesi, Colombia)*; Idris Guessous (Geneva University Hospitals, Switzerland)*; Andre L Guimaraes (State University of Montes Claros, Brazil)*; Martin C Gulliford (King's College London, UK)*; Johanna Gunnlaugsdottir (Icelandic Heart Association, Iceland)*; Marc Gunter (International Agency for Research on Cancer, France)*; Xiuhua Guo (Capital Medical University, China)*; Yin Guo (Capital Medical University, China)*; Prakash C Gupta (Healis-Sekhsaria Institute for Public Health, India)*; Rajeev Gupta (Eternal Heart Care Centre & Research Institute, India)*; Oye Gureje (University of Ibadan, Nigeria)*; Beata Gurzkowska (The Children's Memorial Health Institute, Poland)*; Laura

Gutierrez (Institute for Clinical Effectiveness and Health Policy, Argentina)*; Felix Gutzwiller (University of Zurich, Switzerland)*; Farzad Hadaegh (Shahid Beheshti University of Medical Sciences, Iran)*; Charalambos A Hadjigeorgiou (Research and Education Institute of Child Health, Cyprus)*; Khairil Si-Ramlee (Ministry of Health, Brunei)*; Jytte Halkjær (Danish Cancer Society Research Centre, Denmark)*; Ian R Hambleton (The University of the West Indies, Barbados)*; Rebecca Hardy (University College London, UK)*; Rachakulla Hari Kumar (National Institute of Nutrition, India)*; Maria Hassapidou (Alexander Technological Educational Institute of Thessaloniki, Greece)*; Jun Hata (Kyushu University, Japan)*; Alison J Hayes (University of Sydney, Australia)*; Jiang He (Tulane University, USA)*; Regina Heidinger-Felso (University of Pécs, Hungary)*; Mirjam Heinen (University College Dublin, Ireland)*; Marleen Elisabeth Hendriks (Academic Medical Center of University of Amsterdam, The Netherlands)*; Ana Henriques (Universidade do Porto, Portugal)*; Leticia Hernandez Cadena (National Institute of Public Health, Mexico)*; Sauli Herrala (Oulu University Hospital, Finland)*; Victor M Herrera (Universidad Autónoma de Bucaramanga, Colombia)*; Isabelle Herter-Aeberli (ETH Zurich, Switzerland)*; Ramin Heshmat (Chronic Diseases Research Center, Iran)*; Ilpo Tapani Hihtaniemi (Imperial College London, UK)*; Sai Yin Ho (University of Hong Kong, China)*; Suzanne C Ho (The Chinese University of Hong Kong, China)*; Michael Hobbs (University of Western Australia, Australia)*; Albert Hofman (Erasmus Medical Center Rotterdam, The Netherlands)*; Wilma M Hopman (Kingston General Hospital, Canada)*; Andrea RVR Horimoto (Heart Institute, Brazil)*; Claudia M Hormiga (Fundación Oftalmológica de Santander, Colombia)*; Bernardo L Horta (Federal University of Pelotas, Brazil)*; Leila Houti (University of Oran 1, Algeria)*; Christina Howitt (The University of the West Indies, Barbados)*; Thein Thein Htay (Independent Public Health Specialist, Myanmar)*; Aung Soe Htet (Ministry of Health, Myanmar)*; Maung Maung Than Htike (Ministry of Health, Myanmar)*; Yonghua Hu (Peking University, China)*; José

María Huerta (CIBER en Epidemiología y Salud Pública, Spain)*; Constanta Huidumac Petrescu (National Institute of Public Health, Romania)*; Martijn Huisman (VU University Medical Center and VU University, The Netherlands)*; Abdullatif Husseini (Birzeit University, Palestine)*; Chinh Nguyen Huu (National Institute of Nutrition, Vietnam)*; Inge Huybrechts (International Agency for Research on Cancer, France)*; Nahla Hwalla (American University of Beirut, Lebanon)*; Jolanda Hyska (Institute of Public Health, Albania)*; Licia Iacoviello (IRCCS Istituto Neurologico Mediterraneo Neuromed, Italy; University of Insubria, Italy)*; Anna G Iannone (Cardiologia di Mercato S. Severino, Italy)*; Jesús M Ibarluzea (CIBER en Epidemiología y Salud Pública, Spain)*; Mohsen M Ibrahim (Cairo University, Egypt)*; Nayu Ikeda (National Institute of Health and Nutrition, Japan)*; M Arfan Ikram (Erasmus Medical Center Rotterdam, The Netherlands)*; Vilma E Irazola (Institute for Clinical Effectiveness and Health Policy, Argentina)*; Muhammad Islam (Aga Khan University, Pakistan)*; Aziz al-Safi Ismail (Universiti Sains Malaysia, Malaysia)*; Vanja Ivkovic (UHC Zagreb, Croatia)*; Masanori Iwasaki (Niigata University, Japan)*; Rod T Jackson (University of Auckland, New Zealand)*; Jeremy M Jacobs (Hadassah University Medical Center, Israel)*; Hashem Jaddou (Jordan University of Science and Technology, Jordan)*; Tazeen Jafar (Duke-NUS Medical School, Singapore)*; Kazi M Jamil (Kuwait Institute for Scientific Research, Kuwait)*; Konrad Jamrozik (University of Adelaide, Australia; deceased)*; Imre Janszky (Norwegian University of Science and Technology, Norway)*; Juel Jarani (Sports University of Tirana, Albania)*; Grazyna Jasienska (Jagiellonian University Medical College, Poland)*; Ana Jelaković (UHC Zagreb, Croatia)*; Bojan Jelaković (University of Zagreb School of Medicine, Croatia)*; Garry Jennings (Heart Foundation, Australia)*; Seung-Lyeal Jeong (National Health Insurance Service, South Korea)*; Chao Qiang Jiang (Guangzhou 12th Hospital, China)*; Santa Magaly Jiménez-Acosta (National Institute of Hygiene, Epidemiology and Microbiology, Cuba)*; Michel Joffres (Simon Fraser University, Canada)*;

Mattias Johansson (International Agency for Research on Cancer, France)*; Jost B Jonas (Ruprecht-Karls-University of Heidelberg, Germany)*; Torben Jørgensen (Research Centre for Prevention and Health, Denmark)*; Pradeep Joshi (World Health Organization Country Office, India)*; Dragana P Jovic (Institute of Public Health of Serbia, Serbia)*; Jacek Jóźwiak (Czestochowa University of Technology, Poland)*; Anne Juolevi (National Institute for Health and Welfare, Finland)*; Gregor Jurak (University of Ljubljana, Slovenia)*; Vesna Jureša (University of Zagreb, Croatia)*; Rudolf Kaaks (German Cancer Research Center, Germany)*; Anthony Kafatos (University of Crete, Greece)*; Eero O Kajantie (National Institute for Health and Welfare, Finland)*; Ofra Kalter-Leibovici (The Gertner Institute for Epidemiology and Health Policy Research, Israel)*; Nor Azmi Kamaruddin (Universiti Kebangsaan Malaysia, Malaysia)*; Efthymios Kapantais (Hellenic Medical Association for Obesity, Greece)*; Khem B Karki (Nepal Health Research Council, Nepal)*; Amir Kasaeian (Tehran University of Medical Sciences, Iran)*; Joanne Katz (Johns Hopkins Bloomberg School of Public Health, USA)*; Jussi Kauhanen (University of Eastern Finland, Finland)*; Prabhdeep Kaur (National Institute of Epidemiology, India)*; Maryam Kavousi (Erasmus Medical Center Rotterdam, The Netherlands)*; Gyulli Kazakbaeva (Ufa Eye Research Institute, Russia)*; Ulrich Keil (University of Münster, Germany)*; Lital Keinan Boker (Israel Center for Disease Control, Israel)*; Sirkka Keinänen-Kiukaanniemi (Oulu University Hospital, Finland)*; Roya Kelishadi (Isfahan University of Medical Sciences, Iran)*; Cecily Kelleher (University College Dublin, Ireland)*; Han CG Kemper (VU University Medical Center, The Netherlands)*; Andre P Kengne (South African Medical Research Council, South Africa)*; Alina Kerimkulova (Kyrgyz State Medical Academy, Kyrgyzstan)*; Mathilde Kersting (Research Institute of Child Nutrition, Germany)*; Timothy Key (University of Oxford, UK)*; Yousef Saleh Khader (Jordan University of Science and Technology, Jordan)*; Davood Khalili (Shahid Beheshti University of Medical Sciences, Iran)*; Young-Ho Khang (Seoul National University, South

Korea)*; Mohammad Khateeb (National Center for Diabetes and Endocrinology, Jordan)*; Kay-Tee Khaw (University of Cambridge, UK)*; Ilse MSL Khouw (FrieslandCampina, The Netherlands)*; Ursula Kiechl-Kohlendorfer (Medical University of Innsbruck, Austria)*; Stefan Kiechl (Medical University of Innsbruck, Austria)*; Japhet Killewo (Muhimbili University of Health and Allied Sciences, Tanzania)*; Jeongseon Kim (National Cancer Center, South Korea)*; Yeon-Yong Kim (National Health Insurance Service, South Korea)*; Jeannette Klimont (Statistics Austria, Austria)*; Jurate Klumbiene (Lithuanian University of Health Sciences, Lithuania)*; Michael Knoflach (Medical University of Innsbruck, Austria)*; Bhawesh Koirala (B P Koirala Institute of Health Sciences, Nepal)*; Elin Kolle (Norwegian School of Sport Sciences, Norway)*; Patrick Kolsteren (Institute of Tropical Medicine, Belgium)*; Paul Korrovits (Tartu University Clinics, Estonia)*; Jelena Kos (UHC Zagreb, Croatia)*; Seppo Koskinen (National Institute for Health and Welfare, Finland)*; Katsuyasu Kouda (Kindai University, Japan)*; Viktoria A Kovacs (National Institute of Pharmacy and Nutrition, Hungary)*; Sudhir Kowlessur (Ministry of Health and Quality of Life, Mauritius)*; Slawomir Koziel (Polish Academy of Sciences Anthropology Unit in Wroclaw, Poland)*; Wolfgang Kratzer (University Hospital Ulm, Germany)*; Susi Kriemler (University of Zürich, Switzerland)*; Peter Lund Kristensen (University of Southern Denmark, Denmark)*; Steinar Krokstad (Norwegian University of Science and Technology, Norway)*; Daan Kromhout (University of Groningen, The Netherlands)*; Herculina S Kruger (North-West University, South Africa)*; Ruzena Kubinova (National Institute of Public Health, Czech Republic)*; Renata Kuciene (Lithuanian University of Health Sciences, Lithuania)*; Diana Kuh (University College London, UK)*; Urho M Kujala (University of Jyväskylä, Finland)*; Zbigniew Kulaga (The Children's Memorial Health Institute, Poland)*; R Krishna Kumar (Amrita Institute of Medical Sciences, India)*; Marie Kunešová (Institute of Endocrinology, Czech Republic)*; Pawel Kurjata (The Cardinal Wyszynski Institute of Cardiology, Poland)*;

Yadlapalli S Kusuma (All India Institute of Medical Sciences, India)*; Kari Kuulasmaa (National Institute for Health and Welfare, Finland)*; Catherine Kyobutungi (African Population and Health Research Center, Kenya)*; Quang Ngoc La (Hanoi University of Public Health, Vietnam)*; Fatima Zahra Laamiri (Higher Institute of Nursing Professions and Technical Health Morocco, Morocco)*; Tiina Laatikainen (National Institute for Health and Welfare, Finland)*; Carl Lachat (Ghent University, Belgium)*; Youcef Laid (National Institute of Public Health of Algeria, Algeria)*; Tai Hing Lam (University of Hong Kong, China)*; Orlando Landrove (Ministerio de Salud Pública, Cuba)*; Vera Lanska (Institute for Clinical and Experimental Medicine, Czech Republic)*; Georg Lappas (Sahlgrenska Academy, Sweden)*; Bagher Larijani (Endocrinology and Metabolism Research Center, Iran)*; Lars E Laugsand (Norwegian University of Science and Technology, Norway)*; Laura Lauria (Istituto Superiore di Sanità, Italy)*; Avula Laxmaiah (Indian Council of Medical Research, India)*; Khanh Le Nguyen Bao (National Institute of Nutrition, Vietnam)*; Tuyen D Le (National Institute of Nutrition, Vietnam)*; May Antonnette O Lebanan (Indicium Research, Philippines)*; Catherine Leclercq (Food and Agriculture Organization, Italy)*; Jeannette Lee (National University of Singapore, Singapore)*; Jeonghee Lee (National Cancer Center, South Korea)*; Terho Lehtimäki (Tampere University Hospital, Finland)*; Luz M León-Muñoz (Universidad Autónoma de Madrid, Spain)*; Naomi S Levitt (University of Cape Town, South Africa)*; Yanping Li (Harvard TH Chan School of Public Health, USA)*; Christa L Lilly (West Virginia University, USA)*; Wei-Yen Lim (National University of Singapore, Singapore)*; M Fernanda Lima-Costa (Oswaldo Cruz Foundation Rene Rachou Research Institute, Brazil)*; Hsien-Ho Lin (National Taiwan University, Taiwan)*; Xu Lin (University of Chinese Academy of Sciences, China)*; Lars Lind (Uppsala University, Sweden)*; Allan Linneberg (Research Centre for Prevention and Health, Denmark)*; Lauren Lissner (University of Gothenburg, Sweden)*; Mieczyslaw Litwin (The Children's Memorial Health

Institute, Poland)*; Jing Liu (Capital Medical University Beijing An Zhen Hospital, China)*; Helle-Mai Loit (National Institute for Health Development, Estonia)*; Luis Lopes (University of Porto, Portugal)*; Roberto Lorbeer (University Medicine Greifswald, Germany)*; Paulo A Lotufo (University of São Paulo, Brazil)*; José Eugenio Lozano (Consejería de Sanidad Junta de Castilla y León, Spain)*; Dalia Luksiene (Lithuanian University of Health Sciences, Lithuania)*; Annamari Lundqvist (National Institute for Health and Welfare, Finland)*; Nuno Lunet (Universidade do Porto, Portugal)*; Per Lytsy (University of Uppsala, Sweden)*; Guansheng Ma (Peking University, China)*; Jun Ma (Peking University, China)*; George LL Machado-Coelho (Universidade Federal de Ouro Preto, Brazil)*; Aristides M Machado-Rodrigues (University of Coimbra, Portugal)*; Suka Machi (The Jikei University School of Medicine, Japan)*; Stefania Maggi (National Research Council, Italy)*; Dianna J Magliano (Baker Heart and Diabetes Institute, Australia)*; Emmanuella Magriplis (Agricultural University of Athens, Greece)*; Alagappan Mahaletchumy (Universiti Putra Malaysia, Malaysia)*; Bernard Maire (Institut de Recherche pour le Développement, France)*; Marjeta Majer (University of Zagreb, Croatia)*; Marcia Makdisse (Hospital Israelita Albert Einstein, Brazil)*; Reza Malekzadeh (Shiraz University of Medical Sciences, Iran)*; Rahul Malhotra (Duke-NUS Medical School, Singapore)*; Kodavanti Mallikharjuna Rao (National Institute of Nutrition, India)*; Sofia Malyutina (Institute of Internal and Preventive Medicine, Russia)*; Yannis Manios (Harokopio University, Greece)*; Jim I Mann (University of Otago, New Zealand)*; Enzo Manzato (University of Padova, Italy)*; Paula Margozzini (Pontificia Universidad Católica de Chile, Chile)*; Anastasia Markaki (Technological Educational Institute of Crete, Greece)*; Oonagh Markey (Loughborough University, UK)*; Larissa P Marques (Universidade Federal de Santa Catarina, Brazil)*; Pedro Marques-Vidal (Lausanne University Hospital, Switzerland)*; Jaume Marrugat (Institut Hospital del Mar d'Investigacions Mèdiques, Spain)*; Yves Martin-Prevel (Institut de Recherche pour le

Développement, France)*; Rosemarie Martin (Mary Immaculate College, Ireland)*; Reynaldo Martorell (Emory University, USA)*; Eva Martos (Hungarian Society of Sports Medicine, Hungary)*; Stefano Marventano (University of Catania, Italy)*; Shariq R Masoodi (Sher-i-Kashmir Institute of Medical Sciences, India)*; Ellisiv B Mathiesen (UiT The Arctic University of Norway, Norway)*; Alicia Matijasevich (University of São Paulo, Brazil)*; Tandi E Matsha (Cape Peninsula University of Technology, South Africa)*; Artur Mazur (University of Rzeszow, Poland)*; Jean Claude N Mbanya (University of Yaoundé 1, Cameroon)*; Shelly R McFarlane (The University of the West Indies, Jamaica)*; Stephen T McGarvey (Brown University, USA)*; Martin McKee (London School of Hygiene & Tropical Medicine, UK)*; Stela McLachlan (University of Edinburgh, UK)*; Rachael M McLean (University of Otago, New Zealand)*; Scott B McLean (Statistics Canada, Canada)*; Breige A McNulty (University College Dublin, Ireland)*; Safiah Md Yusof (International Medical University, Malaysia)*; Sounnia Mediene-Benchekor (University of Oran 1, Algeria)*; Jurate Medzioniene (Lithuanian University of Health Sciences, Lithuania)*; Aline Meirhaeghe (Institut National de la Santé et de la Recherche Médicale, France)*; Jørgen Meisfjord (Norwegian Institute of Public Health, Norway)*; Christa Meisinger (Helmholtz Zentrum München, Germany)*; Ana Maria B Menezes (Federal University of Pelotas, Brazil)*; Geetha R Menon (Indian Council of Medical Research, India)*; Gert BM Mensink (Robert Koch Institute, Germany)*; Indrapal I Meshram (National Institute of Nutrition, India)*; Andres Metspalu (University of Tartu, Estonia)*; Haakon E Meyer (University of Oslo, Norway)*; Jie Mi (Capital Institute of Pediatrics, China)*; Kim F Michaelsen (University of Copenhagen, Denmark)*; Nathalie Michels (Ghent University, Belgium)*; Kairit Mikkel (University of Tartu, Estonia)*; Jody C Miller (University of Otago, New Zealand)*; Cláudia S Minderico (Lusófona University, Portugal)*; Juan Francisco Miquel (Pontificia Universidad Católica de Chile, Chile)*; J Jaime Miranda (Universidad Peruana Cayetano Heredia, Peru)*; Daphne

Mirkopoulou (Democritus University, Greece)*; Erkin Mirrakhimov (Kyrgyz State Medical Academy, Kyrgyzstan)*; Marjeta Mišigoj-Durakovic (University of Zagreb, Croatia)*; Antonio Mistretta (University of Catania, Italy)*; Veronica Mocanu (Grigore T Popa University of Medicine and Pharmacy, Romania)*; Pietro A Modesti (Universita' degli Studi di Firenze, Italy)*; Mostafa K Mohamed (Ain Shams University, Egypt)*; Kazem Mohammad (Tehran University of Medical Sciences, Iran)*; Noushin Mohammadifard (Hypertension Research Center, Iran)*; Viswanathan Mohan (Madras Diabetes Research Foundation, India)*; Salim Mohanna (Universidad Peruana Cayetano Heredia, Peru)*; Muhammad Fadhli Mohd Yusoff (Ministry of Health Malaysia, Malaysia)*; Drude Molbo (University of Copenhagen, Denmark)*; Line T Møllehave (Research Centre for Prevention and Health, Denmark)*; Niels C Møller (University of Southern Denmark, Denmark)*; Dénes Molnár (University of Pécs, Hungary)*; Amirabbas Momenan (Shahid Beheshti University of Medical Sciences, Iran)*; Charles K Mondo (Mulago Hospital, Uganda)*; Eric A Monterrubio (Instituto Nacional de Salud Pública, Mexico)*; Kotsedi Daniel K Monyeki (University of Limpopo, South Africa)*; Jin Soo Moon (Seoul National University Children's Hospital, South Korea)*; Leila B Moreira (Universidade Federal do Rio Grande do Sul, Brazil)*; Alain Morejon (University Medical Science, Cuba)*; Luis A Moreno (Universidad de Zaragoza, Spain)*; Karen Morgan (RCSI Dublin, Ireland)*; Erik Lykke Mortensen (University of Copenhagen, Denmark)*; George Moschonis (Harokopio University, Greece)*; Malgorzata Mossakowska (International Institute of Molecular and Cell Biology, Poland)*; Aya Mostafa (Ain Shams University, Egypt)*; Jorge Mota (University of Porto, Portugal)*; Anabela Mota-Pinto (University of Coimbra, Portugal)*; Mohammad Esmaeel Motlagh (Ahvaz Jundishapur University of Medical Sciences, Iran)*; Jorge Motta (Gorgas Memorial Institute of Public Health, Panama)*; Thet Thet Mu (Department of Public Health, Myanmar)*; Magdalena Muc (University of Coimbra, Portugal)*; Maria Lorenza Muiesan (University of Brescia, Italy)*; Martina Müller-

Nurasyid (Helmholtz Zentrum München, Germany)*; Neil Murphy (International Agency for Research on Cancer, France)*; Jaakko Mursu (University of Eastern Finland, Finland)*; Elaine M Murtagh (Mary Immaculate College, Ireland)*; Vera Musil (University of Zagreb, Croatia)*; Iraj Nabipour (Bushehr University of Medical Sciences, Iran)*; Gabriele Nagel (Ulm University, Germany)*; Balkish M Naidu (Institute of Public Health, Malaysia)*; Harunobu Nakamura (Kobe University, Japan)*; Jana Námešná (Banska Bystrica Regional Authority of Public Health, Slovakia)*; Ei Ei K Nang (National University of Singapore, Singapore)*; Vinay B Nangia (Suraj Eye Institute, India)*; Martin Nankap (Helen Keller International, Cameroon)*; Sameer Narake (Healis-Sekhsaria Institute for Public Health, India)*; Paola Nardone (Istituto Superiore di Sanità, Italy)*; Eva Maria Navarrete-Muñoz (CIBER en Epidemiología y Salud Pública, Spain)*; William A Neal (West Virginia University, USA)*; Ilona Nenko (Jagiellonian University Medical College, Poland)*; Martin Neovius (Karolinska Institutet, Sweden)*; Flavio Nervi (Pontificia Universidad Católica de Chile, Chile)*; Chung T Nguyen (National Institute of Hygiene and Epidemiology, Vietnam)*; Nguyen D Nguyen (University of Pharmacy and Medicine of Ho Chi Minh City, Vietnam)*; Quang Ngoc Nguyen (Hanoi Medical University, Vietnam)*; Ramfis E Nieto-Martínez (Miami Veterans Affairs Healthcare System, USA)*; Guang Ning (Shanghai Jiao-Tong University School of Medicine, China)*; Toshiharu Ninomiya (Kyushu University, Japan)*; Sania Nishtar (Heartfile, Pakistan)*; Marianna Noale (National Research Council, Italy)*; Oscar A Noboa (Universidad de la República, Uruguay)*; Teresa Norat (Imperial College London, UK)*; Sawada Norie (National Cancer Center, Japan)*; Davide Noto (University of Palermo, Italy)*; Mohannad Al Nsour (Eastern Mediterranean Public Health Network, Jordan)*; Dermot O'Reilly (The Queen's University of Belfast, UK)*; Galina Obreja (State University of Medicine and Pharmacy, Moldova)*; Eiji Oda (Tachikawa General Hospital, Japan)*; Glenn Oehlers (Academic Hospital of Paramaribo, Suriname)*; Kyungwon Oh

(Korea Centers for Disease Control and Prevention, South Korea)*; Kumiko Ohara (Kobe University, Japan)*; Örn Olafsson (Icelandic Heart Association, Iceland)*; Maria Teresa Anselmo Olinto (University of Vale do Rio dos Sinos, Brazil)*; Isabel O Oliveira (Federal University of Pelotas, Brazil)*; Maciej Oltarzewski (National Food and Nutrition Institute, Poland)*; Mohd Azahadi Omar (Ministry of Health Malaysia, Malaysia)*; Altan Onat (Istanbul University, Turkey)*; Sok King Ong (Ministry of Health, Brunei)*; Lariane M Ono (Universidade Federal de Santa Catarina, Brazil)*; Pedro Ordunez (Pan American Health Organization, USA)*; Rui Ornelas (University of Madeira, Portugal)*; Ana P Ortiz (University of Puerto Rico, Puerto Rico)*; Merete Osler (Research Center for Prevention and Health, Denmark)*; Clive Osmond (MRC Lifecourse Epidemiology Unit, UK)*; Sergej M Ostojic (University of Novi Sad, Serbia)*; Afshin Ostovar (Bushehr University of Medical Sciences, Iran)*; Johanna A Otero (Fundación Oftalmológica de Santander, Colombia)*; Kim Overvad (Aarhus University, Denmark)*; Ellis Owusu-Dabo (Kwame Nkrumah University of Science and Technology, Ghana)*; Fred Michel Paccaud (Institute for Social and Preventive Medicine, Switzerland)*; Cristina Padez (University of Coimbra, Portugal)*; Elena Pahomova (University of Latvia, Latvia)*; Andrzej Pajak (Jagiellonian University Medical College, Poland)*; Domenico Palli (Cancer Prevention and Research Institute, Italy)*; Alberto Palloni (University of Wisconsin-Madison, USA)*; Luigi Palmieri (Istituto Superiore di Sanità, Italy)*; Wen-Harn Pan (Academia Sinica, Taiwan)*; Songhomitra Panda-Jonas (Ruprecht-Karls-University of Heidelberg, Germany)*; Arvind Pandey (National Institute of Medical Statistics, India)*; Francesco Panza (IRCCS Casa Sollievo della Sofferenza, Italy)*; Dimitrios Papandreou (Zayed University, United Arab Emirates)*; Soon-Woo Park (Catholic University of Daegu, South Korea)*; Winsome R Parnell (University of Otago, New Zealand)*; Mahboubeh Parsaeian (Tehran University of Medical Sciences, Iran)*; Ionela M Pascanu (Tg Mures University of Medicine and Pharmacy, Romania)*; Nikhil D Patel (Jivandeep Hospital,

India)*; Ivan Pećin (University of Zagreb School of Medicine, Croatia; UHC Zagreb, Croatia)*; Mangesh S Pednekar (Healis-Sekhsaria Institute for Public Health, India)*; Nasheeta Peer (South African Medical Research Council, South Africa)*; Petra H Peeters (University Medical Center Utrecht, The Netherlands)*; Sergio Viana Peixoto (Oswaldo Cruz Foundation Rene Rachou Research Institute, Brazil)*; Markku Peltonen (National Institute for Health and Welfare, Finland)*; Alexandre C Pereira (Heart Institute, Brazil)*; Napoleon Perez-Farinos (Spanish Agency of Consumer Affairs, Social Services and Nutrition, Spain)*; Cynthia M Pérez (University of Puerto Rico Medical Sciences Campus, Puerto Rico)*; Annette Peters (Helmholtz Zentrum München, Germany)*; Janina Petkeviciene (Lithuanian University of Health Sciences, Lithuania)*; Ausra Petrauskiene (Lithuanian University of Health Sciences, Lithuania)*; Niloofar Peykari (Ministry of Health and Medical Education, Iran)*; Son Thai Pham (Vietnam National Heart Institute, Vietnam)*; Daniela Pierannunzio (Istituto Superiore di Sanità, Italy)*; Iris Pigeot (Leibniz Institute for Prevention Research and Epidemiology - BIPS, Germany)*; Hynek Pikhart (University College London, UK)*; Aida Pilav (University of Sarajevo, Bosnia and Herzegovina)*; Lorenza Pilotto (Cardiovascular Prevention Centre Udine, Italy)*; Francesco Pistelli (University Hospital of Pisa, Italy)*; Freda Pitakaka (Ministry of Health and Medical Services, Solomon Islands)*; Aleksandra Piwonska (The Cardinal Wyszynski Institute of Cardiology, Poland)*; Pedro Plans-Rubió (Public Health Agency of Catalonia, Spain)*; Bee Koon Poh (Universiti Kebangsaan Malaysia, Malaysia)*; Hermann Pohlabeln (Leibniz Institute for Prevention Research and Epidemiology - BIPS, Germany)*; Raluca M Pop (Tg Mures University of Medicine and Pharmacy, Romania)*; Stevo R Popovic (University of Montenegro, Montenegro)*; Miquel Porta (Institut Hospital del Mar d'Investigacions Mèdiques, Spain)*; Marileen LP Portegies (Erasmus Medical Center Rotterdam, The Netherlands)*; Georg Posch (Agency for Preventive and Social Medicine, Austria)*; Dimitrios Poulimeneas (Alexander Technological Educational Institute, Greece)*;

Hamed Pouraram (Tehran University of Medical Sciences, Iran)*; Akram Pourshams (Digestive Oncology Research Center, Iran)*; Hossein Poustchi (Digestive Disease Research Institute, Iran)*; Rajendra Pradeepa (Madras Diabetes Research Foundation, India)*; Mathur Prashant (Indian Council of Medical Research, India)*; Jacqueline F Price (University of Edinburgh, UK)*; Jardena J Puder (Centre Hospitalier Universitaire Vaudois, Switzerland)*; Iveta Pudule (Centre for Disease and Prevention Control, Latvia)*; Maria Puiu (Victor Babeș University of Medicine and Pharmacy Timisoara, Romania)*; Margus Punab (Tartu University Clinics, Estonia)*; Radwan F Qasrawi (Al-Quds University, Palestine)*; Mostafa Qorbani (Alborz University of Medical Sciences, Iran)*; Tran Quoc Bao (Ministry of Health, Vietnam)*; Ivana Radic (University of Novi Sad, Serbia)*; Ricardas Radisauskas (Lithuanian University of Health Sciences, Lithuania)*; Mahfuzar Rahman (BRAC, Bangladesh)*; Mahmudur Rahman (Institute of Epidemiology Disease Control and Research, Bangladesh)*; Olli Raitakari (University of Turku, Finland)*; Manu Raj (Amrita Institute of Medical Sciences, India)*; Sudha Ramachandra Rao (National Institute of Epidemiology, India)*; Ambady Ramachandran (India Diabetes Research Foundation, India)*; Jacqueline Ramke (University of New South Wales, Australia)*; Elisabete Ramos (University of Porto Medical School, Portugal)*; Rafel Ramos (Institut Universitari d'Investigació en Atenció Primària Jordi Gol, Spain)*; Lekhraj Rampal (Universiti Putra Malaysia, Malaysia)*; Sanjay Rampal (University of Malaya, Malaysia)*; Ramon A Rascon-Pacheco (Instituto Mexicano del Seguro Social, Mexico)*; Josep Redon (University of Valencia, Spain)*; Paul Ferdinand M Reganit (University of the Philippines, Philippines)*; Lourdes Ribas-Barba (Nutrition Research Foundation, Spain)*; Robespierre Ribeiro (Minas Gerais State Secretariat for Health, Brazil; deceased)*; Elio Riboli (Imperial College London, UK)*; Fernando Rigo (Health Center San Agustín, Spain)*; Tobias F Rinke de Wit (PharmAccess Foundation, The Netherlands)*; Ana Rito (National Institute of Health Doutor Ricardo Jorge, Portugal)*; Raphael M Ritti-Dias

(Hospital Israelita Albert Einstein, Brazil)*; Juan A Rivera (Instituto Nacional de Salud Pública, Mexico)*; Sian M Robinson (University of Southampton, UK)*; Cynthia Robitaille (Public Health Agency of Canada, Canada)*; Daniela Rodrigues (University of Coimbra, Portugal)*; Fernando Rodríguez-Artalejo (Universidad Autónoma de Madrid, Spain)*; María del Cristo Rodriguez-Perez (Canarian Health Service, Spain)*; Laura A Rodríguez-Villamizar (Universidad Industrial de Santander, Colombia)*; Rosalba Rojas-Martinez (Instituto Nacional de Salud Pública, Mexico)*; Nipa Rojroongwasinkul (Mahidol University, Thailand)*; Dora Romaguera (CIBEROBN, Spain)*; Kimmo Ronkainen (University of Eastern Finland, Finland)*; Annika Rosengren (University of Gothenburg, Sweden)*; Ian Rouse (Fiji National University, Fiji)*; Joel GR Roy (Statistics Canada, Canada)*; Adolfo Rubinstein (Institute for Clinical Effectiveness and Health Policy, Argentina)*; Frank J Rühli (University of Zurich, Switzerland)*; Blanca Sandra Ruiz-Betancourt (Instituto Mexicano del Seguro Social, Mexico)*; Paola Russo (Institute of Food Sciences of the National Research Council, Italy)*; Marcin Rutkowski (Medical University of Gdansk, Poland)*; Charumathi Sabanayagam (Singapore Eye Research Institute, Singapore)*; Harshpal S Sachdev (Sitaram Bhartia Institute of Science and Research, India)*; Olfa Saidi (University Tunis El Manar, Tunisia)*; Benoit Salanave (French Public Health Agency, France)*; Eduardo Salazar Martinez (National Institute of Public Health, Mexico)*; Diego Salmerón (CIBER de Epidemiología y Salud Pública, Spain)*; Veikko Salomaa (National Institute for Health and Welfare, Finland)*; Jukka T Salonen (University of Helsinki, Finland)*; Massimo Salvetti (University of Brescia, Italy)*; Jose Sánchez-Abanto (National Institute of Health, Peru)*; Sandjaja (Ministry of Health, Indonesia)*; Susana Sans (Catalan Department of Health, Spain)*; Loreto Santa Marina (Public Health Subdelegate Office, Spain)*; Diana A Santos (Universidade de Lisboa, Portugal)*; Ina S Santos (Federal University of Pelotas, Brazil)*; Osvaldo Santos (Institute of Preventive Medicine and Public Health, Portugal)*; Renata Nunes dos Santos (University of

Sao Paulo Clinics Hospital, Brazil)*; Rute Santos (University of Porto, Portugal)*; Jouko L Saramies (South Karelia Social and Health Care District, Finland)*; Luis B Sardinha (Universidade de Lisboa, Portugal)*; Nizal Sarrafzadegan (Isfahan Cardiovascular Research Center, Iran)*; Kai-Uwe Saum (German Cancer Research Center, Germany)*; Savvas Savva (Research and Education Institute of Child Health, Cyprus)*; Mathilde Savy (Institut de Recherche pour le Développement, France)*; Marcia Scazufca (University of Sao Paulo Clinics Hospital, Brazil)*; Angelika Schaffrath Rosario (Robert Koch Institute, Germany)*; Herman Schargrodsky (Hospital Italiano de Buenos Aires, Argentina)*; Anja Schienkiewitz (Robert Koch Institute, Germany)*; Sabine Schipf (University Medicine of Greifswald, Germany)*; Carsten O Schmidt (University Medicine of Greifswald, Germany)*; Ida Maria Schmidt (Rigshospitalet, Denmark)*; Constance Schultsz (Academic Medical Center of University of Amsterdam, The Netherlands)*; Aletta E Schutte (South African Medical Research Council, South Africa; North-West University, South Africa)*; Aye Aye Sein (Ministry of Health, Myanmar)*; Abhijit Sen (Norwegian University of Science and Technology, Norway)*; Idowu O Senbanjo (Lagos State University College of Medicine, Nigeria)*; Sadaf G Sepanlou (Tehran University of Medical Sciences, Iran)*; Luis Serra-Majem (University of Las Palmas de Gran Canaria, Spain)*; Svetlana A Shalnova (National Research Centre for Preventive Medicine, Russia)*; Sanjib K Sharma (B P Koirala Institute of Health Sciences, Nepal)*; Jonathan E Shaw (Baker Heart and Diabetes Institute, Australia)*; Kenji Shibuya (The University of Tokyo, Japan)*; Dong Wook Shin (Samsung Medical Center, South Korea)*; Youchan Shin (Singapore Eye Research Institute, Singapore)*; Rahman Shiri (Finnish Institute of Occupational Health, Finland)*; Alfonso Siani (Institute of Food Sciences of the National Research Council, Italy)*; Rosalynn Siantar (Singapore Eye Research Institute, Singapore)*; Abla M Sibai (American University of Beirut, Lebanon)*; Antonio M Silva (Federal University of Maranhao, Brazil)*; Diego Augusto Santos Silva

(Federal University of Santa Catarina, Brazil)*; Mary Simon (India Diabetes Research Foundation, India)*; Judith Simons (St Vincent's Hospital, Australia)*; Leon A Simons (University of New South Wales, Australia)*; Agneta Sjöberg (University of Gothenburg, Sweden)*; Michael Sjöström (Karolinska Institutet, Sweden)*; Sine Skovbjerg (Research Centre for Prevention and Health, Denmark)*; Jolanta Slowikowska-Hilczer (Medical University of Lodz, Poland)*; Przemyslaw Slusarczyk (International Institute of Molecular and Cell Biology, Poland)*; Liam Smeeth (London School of Hygiene & Tropical Medicine, UK)*; Margaret C Smith (University of Oxford, UK)*; Marieke B Snijder (Academic Medical Center Amsterdam, The Netherlands)*; Hung-Kwan So (The Chinese University of Hong Kong, China)*; Eugène Sobngwi (University of Yaoundé 1, Cameroon)*; Stefan Söderberg (Umeå University, Sweden)*; Moesijanti YE Soekatri (Health Polytechnic Jakarta II Institute, Indonesia)*; Vincenzo Solfrizzi (University of Bari, Italy)*; Emily Sonestedt (Lund University, Sweden)*; Yi Song (Peking University, China)*; Thorkild IA Sørensen (University of Copenhagen, Denmark)*; Maroje Soric (University of Zagreb, Croatia)*; Charles Sossa Jérome (Institut Régional de Santé Publique, Benin)*; Aicha Soumare (University of Bordeaux, France)*; Angela Spinelli (Istituto Superiore di Sanità, Italy)*; Igor Spiroski (Institute of Public Health of Republic of Macedonia, Macedonia (TFYR))*; Jan A Staessen (University of Leuven, Belgium)*; Hanspeter Stamm (Lamprecht und Stamm Sozialforschung und Beratung AG, Switzerland)*; Gregor Starc (University of Ljubljana, Slovenia)*; Maria G Stathopoulou (INSERM, France)*; Kaspar Staub (University of Zurich, Switzerland)*; Bill Stavreski (Heart Foundation, Australia)*; Jostein Steene-Johannessen (Norwegian School of Sport Sciences, Norway)*; Peter Stehle (Bonn University, Germany)*; Aryeh D Stein (Emory University, USA)*; George S Stergiou (Sotiria Hospital, Greece)*; Jochanan Stessman (Hadassah University Medical Center, Israel)*; Jutta Stieber (Helmholtz Zentrum München, Germany; deceased)*; Doris Stöckl (Helmholtz Zentrum München, Germany)*; Tanja Stocks

(Lund University, Sweden)*; Jakub Stokwiszewski (National Institute of Public Health-National Institute of Hygiene, Poland)*; Gareth Stratton (Swansea University, UK)*; Karien Stronks (University of Amsterdam, The Netherlands)*; Maria Wany Strufaldi (Federal University of São Paulo, Brazil)*; Ramón Suárez-Medina (National Institute of Hygiene, Epidemiology and Microbiology, Cuba)*; Chien-An Sun (Fu Jen Catholic University, Taiwan)*; Johan Sundström (Uppsala University, Sweden)*; Yn-Tz Sung (The Chinese University of Hong Kong, China)*; Jordi Sunyer (ISGlobal Centre for Research in Environmental Epidemiology, Spain)*; Paibul Suriyawongpaisal (Mahidol University, Thailand)*; Boyd A Swinburn (The University of Auckland, New Zealand)*; Rody G Sy (University of the Philippines, Philippines)*; Lucjan Szponar (National Food and Nutrition Institute, Poland)*; E Shyong Tai (National University of Singapore, Singapore)*; Mari-Liis Tammesoo (University of Tartu, Estonia)*; Abdonas Tamosiunas (Lithuanian University of Health Sciences, Lithuania)*; Eng Joo Tan (University of Sydney, Australia)*; Xun Tang (Peking University, China)*; Frank Tanser (University of KwaZulu-Natal, South Africa)*; Yong Tao (Peking University, China)*; Mohammed Rasoul Tarawneh (Ministry of Health, Jordan)*; Jakob Tarp (University of Southern Denmark, Denmark)*; Carolina B Tarqui-Mamani (National Institute of Health, Peru)*; Oana-Florentina Tautu (Carol Davila University of Medicine and Pharmacy, Romania)*; Radka Taxová Braunerová (Institute of Endocrinology, Czech Republic)*; Anne Taylor (The University of Adelaide, Australia)*; Félicité Tchibindat (UNICEF, Cameroon)*; Holger Theobald (Karolinska Institutet, Sweden)*; Xenophon Theodoridis (Alexander Technological Educational Institute, Greece)*; Lutgarde Thijs (University of Leuven, Belgium)*; Betina H Thuesen (Research Centre for Prevention and Health, Denmark)*; Anne Tjonneland (Danish Cancer Society Research Centre, Denmark)*; Hanna K Tolonen (National Institute for Health and Welfare, Finland)*; Janne S Tolstrup (University of Southern Denmark, Denmark)*; Murat Topbas (Karadeniz

Technical University, Turkey)*; Roman Topór-Madry (Jagiellonian University Medical College, Poland)*; María José Tormo (Health Service of Murcia, Spain)*; Michael J Tornaritis (Research and Education Institute of Child Health, Cyprus)*; Maties Torrent (IB-SALUT Area de Salut de Menorca, Spain)*; Stefania Toselli (University of Bologna, Italy)*; Pierre Traissac (Institut de Recherche pour le Développement, France)*; Dimitrios Trichopoulos (Harvard TH Chan School of Public Health, USA; deceased)*; Antonia Trichopoulou (Hellenic Health Foundation, Greece)*; Oanh TH Trinh (University of Pharmacy and Medicine of Ho Chi Minh City, Vietnam)*; Atul Trivedi (Government Medical College, India)*; Lechaba Tshepo (Sefako Makgatho Health Science University, South Africa)*; Maria Tsigga (Alexander Technological Educational Institute, Greece)*; Shoichiro Tsugane (National Cancer Center, Japan)*; Marshall K Tulloch-Reid (The University of the West Indies, Jamaica)*; Fikru Tullu (Addis Ababa University, Ethiopia)*; Tomi-Pekka Tuomainen (University of Eastern Finland, Finland)*; Jaakko Tuomilehto (Dasman Diabetes Institute, Kuwait)*; Maria L Turley (Ministry of Health, New Zealand)*; Per Tynelius (Karolinska Institutet, Sweden)*; Themistoklis Tzotzas (Hellenic Medical Association for Obesity, Greece)*; Christophe Tzourio (University of Bordeaux, France)*; Peter Ueda (Harvard TH Chan School of Public Health, USA)*; Eunice E Ugel (Universidad Centro-Occidental Lisandro Alvarado, Venezuela)*; Flora AM Ukoli (Meharry Medical College, USA)*; Hanno Ulmer (Medical University of Innsbruck, Austria)*; Belgin Unal (Dokuz Eylul University, Turkey)*; Hannu MT Uusitalo (University of Tampere Tays Eye Center, Finland)*; Gonzalo Valdivia (Pontificia Universidad Católica de Chile, Chile)*; Susana Vale (Polytechnic Institute of Porto, Portugal)*; Damaskini Valvi (Harvard TH Chan School of Public Health, USA)*; Yvonne T van der Schouw (Utrecht University, The Netherlands)*; Koen Van Herck (Ghent University, Belgium)*; Hoang Van Minh (Hanoi University of Public Health, Vietnam)*; Lenie van Rossem (University Medical Center Utrecht, The Netherlands)*; Natasja M Van Schoor

(Amsterdam Public Health Research Institute, The Netherlands)*; Irene GM van Valkengoed (Academic Medical Center Amsterdam, The Netherlands)*; Dirk Vanderschueren (Katholieke Universiteit Leuven, Belgium)*; Diego Vanuzzo (Cardiovascular Prevention Centre Udine, Italy)*; Lars Vatten (Norwegian University of Science and Technology, Norway)*; Tomas Vega (Consejería de Sanidad Junta de Castilla y León, Spain)*; Toomas Veidebaum (National Institute for Health Development, Estonia)*; Gustavo Velasquez-Melendez (Universidade Federal de Minas Gerais, Brazil)*; Biruta Velika (Centre for Disease and Prevention Control, Latvia)*; Giovanni Veronesi (University of Insubria, Italy)*; WM Monique Verschuren (National Institute for Public Health and the Environment, The Netherlands)*; Cesar G Victora (Federal University of Pelotas, Brazil)*; Giovanni Viegi (Italian National Research Council, Italy)*; Lucie Viet (National Institute for Public Health and the Environment, The Netherlands)*; Eira Viikari-Juntura (Finnish Institute of Occupational Health, Finland)*; Paolo Vineis (Imperial College London, UK)*; Jesus Vioque (Universidad Miguel Hernandez, Spain)*; Jyrki K Virtanen (University of Eastern Finland, Finland)*; Sophie Visvikis-Siest (INSERM, France)*; Bharathi Viswanathan (Ministry of Health, Seychelles)*; Tiina Vlasoff (North Karelian Center for Public Health, Finland)*; Peter Vollenweider (Lausanne University Hospital, Switzerland)*; Henry Völzke (University Medicine Greifswald, Germany)*; Sari Voutilainen (University of Eastern Finland, Finland)*; Martine Vrijheid (ISGlobal Centre for Research in Environmental Epidemiology, Spain)*; Alisha N Wade (University of the Witwatersrand, South Africa)*; Aline Wagner (University of Strasbourg, France)*; Thomas Waldhör (Medical University of Vienna, Austria)*; Janette Walton (University College Cork, Ireland)*; Wan Mohamad Wan Bebakar (Universiti Sains Malaysia, Malaysia)*; Wan Nazaimoon Wan Mohamud (Institute for Medical Research, Malaysia)*; Rildo S Wanderley Jr. (University of Pernambuco, Brazil)*; Ming-Dong Wang (Public Health Agency of Canada, Canada)*; Qian Wang (Xinjiang Medical University, China)*; Ya Xing Wang (Capital

Medical University, China)*; Ying-Wei Wang (Ministry of Health and Welfare, Taiwan)*; S Goya Wannamethee (University College London, UK)*; Nicholas Wareham (University of Cambridge, UK)*; Adelheid Weber (Federal Ministry of Health and Women's Affairs, Austria)*; Niels Wedderkopp (University of Southern Denmark, Denmark)*; Deepa Weerasekera (Ministry of Health, New Zealand)*; Peter H Whincup (St George's, University of London, UK)*; Kurt Widhalm (Medical University of Vienna, Austria)*; Indah S Widyahening (Universitas Indonesia, Indonesia)*; Andrzej Wiecek (Medical University of Silesia, Poland)*; Alet H Wijga (National Institute for Public Health and the Environment, The Netherlands)*; Rainford J Wilks (The University of the West Indies, Jamaica)*; Johann Willeit (Medical University of Innsbruck, Austria)*; Peter Willeit (Medical University of Innsbruck, Austria)*; Tom Wilsgaard (UiT The Arctic University of Norway, Norway)*; Bogdan Wojtyniak (National Institute of Public Health-National Institute of Hygiene, Poland)*; Roy A Wong-McClure (Caja Costarricense de Seguro Social, Costa Rica)*; Justin YY Wong (Ministry of Health, Brunnei)*; Jyh Eiin Wong (Universiti Kebangsaan Malaysia, Malaysia)*; Tien Yin Wong (Duke-NUS Medical School, Singapore)*; Jean Woo (The Chinese University of Hong Kong, China)*; Mark Woodward (University of New South Wales, Australia; University of Oxford, UK)*; Frederick C Wu (University of Manchester, UK)*; Jianfeng Wu (Shandong University of Traditional Chinese Medicine, China)*; Shouling Wu (Kailuan General Hospital, China)*; Haiquan Xu (Institute of Food and Nutrition Development of Ministry of Agriculture, China)*; Liang Xu (Capital Medical University, China)*; Uruwan Yamborisut (Mahidol University, Thailand)*; Weili Yan (Children's Hospital of Fudan University, China)*; Xiaoguang Yang (Chinese Center for Disease Control and Prevention, China)*; Nazan Yardim (Ministry of Health, Turkey)*; Xingwang Ye (University of Chinese Academy of Sciences, China)*; Panayiotis K Yiallouros (University of Cyprus, Cyprus)*; Agneta Yngve (Uppsala University, Sweden)*; Akihiro Yoshihara (Niigata University,

Japan)*; Qi Sheng You (Capital Medical University, China)*; Novie O Younger-Coleman (The University of the West Indies, Jamaica)*; Faudzi Yusoff (Ministry of Health Malaysia, Malaysia)*; Muhammad Fadhli M Yusoff (Institute of Public Health, Malaysia)*; Luciana Zaccagni (University of Ferrara, Italy)*; Vassilis Zafiropulos (Technological Educational Institute of Crete, Greece)*; Ahmad A Zainuddin (Universiti Teknologi MARA, Malaysia)*; Sabina Zambon (University of Padova, Italy)*; Antonis Zampelas (Agricultural University of Athens, Greece)*; Hana Zamrazilová (Institute of Endocrinology, Czech Republic)*; Tomasz Zdrojewski (Medical University of Gdansk, Poland)*; Yi Zeng (Duke University, USA; Peking University, China, USA)*; Dong Zhao (Capital Medical University Beijing An Zhen Hospital, China)*; Wenhua Zhao (Chinese Center for Disease Control and Prevention, China)*; Wei Zheng (Vanderbilt University, USA)*; Yingfeng Zheng (Singapore Eye Research Institute, Singapore)*; Bekbolat Zholdin (West Kazakhstan State Medical University, Kazakhstan)*; Maigeng Zhou (Chinese Center for Disease Control and Prevention, China)*; Dan Zhu (Inner Mongolia Medical University, China)*; Baurzhan Zhussupov (Kazakh National Medical University, Kazakhstan)*; Esther Zimmermann (Bispebjerg and Frederiksberg Hospitals, Denmark)*; Julio Zuñiga Cisneros (Gorgas Memorial Institute of Public Health, Panama)*

Appendix 1: Data sources

We used a database of population-based data on cardiometabolic risk factors collated by the NCD Risk Factor Collaboration (NCD-RisC), a worldwide network of health researchers and practitioners whose aim is to document systematically the worldwide trends and variations in non-communicable disease (NCD) risk factors. The database was collated through multiple routes for identifying and accessing data. We accessed publicly available population-based multi-country and national measurement surveys (e.g., Demographic and Health Surveys (DHS), Global School-based student Health Surveys (GSHS), and surveys identified via the Inter-University Consortium for Political and Social Research and the European Health Interview & Health Examination Surveys Database) as well as the World Health Organization (WHO) STEPwise approach to Surveillance (STEPS) surveys. We requested, via WHO and its regional and country offices, from ministries of health and other national health and statistical agencies to identify and access population-based surveys. Requests were also sent via the World Heart Federation to its national partners. We made a similar request to the co-authors of an earlier pooled analysis of cardiometabolic risk factors,[1-4] and invited them to reanalyse data from their studies and join NCD-RisC. Finally, to identify major sources not accessed through the above routes, we searched and reviewed published studies as detailed previously,[5] and invited all eligible studies to join NCD-RisC.

Anonymised individual record data from sources included in NCD-RisC were reanalysed by the Pooled Analysis and Writing Group or by data holders according to a common protocol. Within each survey, we included participants aged five years and older who were not pregnant. We dropped participants with implausible body mass index (BMI) levels (defined as BMI <7 kg/m^2 or BMI >80 kg/m^2 for children and adolescents and BMI <10 kg/m^2 or BMI >80 kg/m^2

for adults) or with implausible height or weight values (defined as height <80 cm, height >250 cm, weight <8 kg or weight >300 kg) (<0.2% of all subjects).

We calculated mean BMI, prevalence of a comprehensive set of BMI categories, and associated standard errors by sex and age group (one-year age groups from 5 to 19 years, 20-29 years, followed by 10-year age groups and 80+ years). For children and adolescents aged 5-19 years, we analysed prevalence in the following BMI ranges: <-2SD from the median of the WHO growth reference for children and adolescents[6] (referred to as severe underweight hereafter), -2SD to <-1SD (moderate underweight), -1SD to 1SD, >1 SD to 2SD (overweight but not obese), and >2SD (obesity). The cut-offs for calculating prevalence in these BMI categories were all age-specific and were applied to data in single years of age. For adults, we analysed prevalence in the following BMI ranges: <18·5 kg/m^2, 18·5 kg/m^2 to <20 kg/m^2, 20 kg/m^2 to <25 kg/m^2, 25 kg/m^2 to <30 kg/m^2, 30 kg/m^2 to <35 kg/m^2, 35 kg/m^2 to <40 kg/m^2, and ≥40 kg/m^2. All analyses incorporated appropriate sample weights and complex survey design in calculating age-sex-specific means and prevalence, when applicable. To ensure summaries were prepared according to the study protocol, the Pooled Analysis and Writing Group provided computer code to NCD-RisC members who requested assistance. All submitted data were checked by at least two independent members of the Pooled Analysis and Writing Group. Questions and clarifications were discussed with NCD-RisC members and resolved before data were incorporated in the database.

Finally, we obtained data not accessed through the above routes by extracting from published reports of all additional national health surveys identified through the above-described strategies, four sub-national STEPS surveys, one CINDI surveys, and six MONICA sites which were not deposited in MONICA Data Centre. Data were extracted from published reports only

when reported by sex and in age groups no wider than 20 years. We also used data from a previous pooling study[1] when such data had not been accessed through the above routes.

NCD-RisC database is continuously updated through the above routes and through periodic requests to NCD-RisC members to help identify new data sources. In this paper, we used data from the NCD-RisC database for years 1975 to 2016.

Data inclusion and exclusion

Data sources were included in NCD-RisC database if:

- measured data on height, weight, waist circumference, or hip circumference were available;
- study participants were five years of age and older;
- data were collected using a probabilistic sampling method with a defined sampling frame;
- data were from population samples at the national, sub-national, or community level;
- data were collected in or after 1950; and
- data were from the countries and territories listed in Appendix Table 1.

We excluded all data sources that were solely based on self-reported weight and height without a measurement component. We also excluded data sources on population subgroups whose anthropometric status may differ systematically from the general population, including:

- studies that had included or excluded people based on their health status or cardiovascular risk;
- studies whose participants were only ethnic minorities;

- specific educational, occupational, or socioeconomic subgroups, with the exception noted below;
- those recruited through health facilities, with the exception noted below; and
- women aged 15-19 years in surveys which sampled only ever-married women or measured height and weight only among mothers.

We used school-based data in countries, and in age-sex groups, with school enrolment of 70% or higher. We used data whose sampling frame was health insurance schemes in countries where at least 80% of the population were insured. Finally, we used data collected through general practice and primary care systems in high-income countries and emerging economies in central Europe with universal insurance, because contact with the primary care systems tends to be at least as good as response rates for population-based surveys.

Data management

For each data source accessed through the primary and secondary data access process, we recorded the available information about the study population, period of measurement, sampling approach, and measurement methods. The information about study population was used to establish that each data source was population-based, and to assess whether it covered the whole country, multiple sub-national regions, or one or a small number of communities, and whether it was rural, urban, or combined.

We carefully checked all data sources in terms of how they met our inclusion and exclusion criteria. We identified duplicate data sources by comparing studies from the same country and year. Additionally, NCD-RisC members received the list of all data sources in the database and were asked to ensure that the included data from their country met the inclusion criteria and

that there were no duplicates. Data sources used in the analysis are listed in Appendix Table 2. The number of data sources and number of participants used in the analysis by age are provided in Appendix Table 3.

Appendix 2: Converting among mean BMI and prevalence of different BMI categories

As described in Methods, our primary outcomes were mean BMI, and the prevalences of a mutually exclusive and collectively exhaustive set of BMI categories. The categories for children and adolescents were: <-2SD from the median of the WHO growth reference for children and adolescents 5-19 years old (moderate and severe underweight) (BMI <-2SD is referred to as thinness by the WHO), -2SD to <-1SD (mild underweight), -1SD to 1SD (normal weight), >1SD to 2SD (overweight), and >2SD (obesity). For adults, they were <18.5 kg/m^2, 18.5 to <20 kg/m^2, 20 to <25 kg/m^2, 25 to <30 kg/m^2, 30 to <35 kg/m^2, 35 to <40 kg/m^2 and ≥ 40 kg/m^2.

In 6% of our data points for children and adolescents and 15% of our data points for adults, mostly extracted from published reports or from a previous pooling analysis,[5] data were available for only a subset of these outcomes, and/or were available for prevalences of other BMI ranges. In order to use these data, we developed conversion (or cross-walking) regressions to estimate all of our outcome variables from the available data.

The dependent variable in each regression was one of the primary outcomes on which data were only partially available. The independent variable was one or more of the above-mentioned metrics present in at least one study that did not report all primary outcomes. All regressions included terms for age, sex, the year of study, and country's income (natural logarithm of per-capita gross domestic product in 2011 international dollars), as well as regional random effects and interactions between predictors and age and sex, based on the Bayesian Information Criterion (BIC).[7] Mean BMI was inversed when used as a dependent or independent variable because the relationship between inverse mean BMI and the probit of prevalence was found to be closer to linear than relationships between other functions of mean

BMI. The coefficients of these regressions were estimated from data sources with individual-level data which could be used to calculate all included terms, excluding data points with fewer than 25 participants. The regression coefficients and number of data points used to estimate the coefficients are shown in Appendix Table 4 for children and adolescents and in Appendix Table 5 for adults.

All sources of uncertainty in the conversion, including the sampling uncertainty of the data on the available metrics, the uncertainty of the regression coefficients and random effects, and the regression residuals, were carried forward by using repeated draws from their respective distributions. We accounted for the correlation among the uncertainties of regression coefficients and random effects, by drawing from their joint posterior distribution.

Appendix Table 1: List of analysis regions and "super-regions", and countries in each region. The hierarchical structure of the statistical model consisted of country, region, super-region, and world.

Super-region	Region
**Sub-Saharan Africa (48)**	**Central Africa (6)**: Angola, Central African Republic, Congo, DR Congo, Equatorial Guinea, Gabon
	**East Africa (17)**: Burundi, Comoros, Djibouti, Eritrea, Ethiopia, Kenya, Madagascar, Malawi, Mauritius, Mozambique, Rwanda, Seychelles, Somalia, Sudan (former), Tanzania, Uganda, Zambia
	**Southern Africa (6)**: Botswana, Lesotho, Namibia, South Africa, Swaziland, Zimbabwe
	**West Africa (19)**: Benin, Burkina Faso, Cabo Verde, Cameroon, Chad, Cote d'Ivoire, Gambia, Ghana, Guinea, Guinea Bissau, Liberia, Mali, Mauritania, Niger, Nigeria, Sao Tome and Principe, Senegal, Sierra Leone, Togo
**Central Asia, Middle East and North Africa (28)**	**Central Asia (9)**: Armenia, Azerbaijan, Georgia, Kazakhstan, Kyrgyzstan, Mongolia, Tajikistan, Turkmenistan, Uzbekistan
	**Middle East and North Africa (19)**: Algeria, Bahrain, Egypt, Iran, Iraq, Jordan, Kuwait, Lebanon, Libya, Morocco, Occupied Palestinian Territory, Oman, Qatar, Saudi Arabia, Syrian Arab Republic, Tunisia, Turkey, United Arab Emirates, Yemen
**South Asia (6)**	**South Asia (6)**: Afghanistan, Bangladesh, Bhutan, India, Nepal, Pakistan
**East and Southeast Asia (16)**	**East Asia (4)**: China, China (Hong Kong SAR), North Korea, Taiwan
	**Southeast Asia (12)**: Brunei Darussalam, Cambodia, Indonesia, Lao PDR, Malaysia, Maldives, Myanmar, Philippines, Sri Lanka, Thailand, Timor-Leste, Viet Nam
**Oceania (17)**	**Polynesia and Micronesia (13)**: American Samoa, Cook Islands, French Polynesia, Kiribati, Marshall Islands, Micronesia (Federated States of), Nauru, Niue, Palau, Samoa, Tokelau, Tonga, Tuvalu
	**Melanesia (4):** Fiji, Papua New Guinea, Solomon Islands, Vanuatu
**High-income Asia Pacific (3)**	**High-income Asia Pacific (3)**: Japan, Singapore, South Korea
**Latin America and Caribbean (35)**	**Andean Latin America (3)**: Bolivia, Ecuador, Peru
	**Caribbean (18)**: Antigua and Barbuda, Bahamas, Barbados, Belize, Bermuda, Cuba, Dominica, Dominican Republic, Grenada, Guyana, Haiti, Jamaica, Puerto Rico, Saint Kitts and Nevis, Saint Lucia, Saint Vincent and the Grenadines, Suriname, Trinidad and Tobago
	**Central Latin America (9)**: Colombia, Costa Rica, El Salvador, Guatemala, Honduras, Mexico, Nicaragua, Panama, Venezuela
	**Southern Latin America (5)**: Argentina, Brazil, Chile, Paraguay, Uruguay

**High-income Western countries (27)**	**High-income English-speaking countries* (6)**: Australia, Canada, Ireland, New Zealand, United Kingdom, United States of America
	**North Western Europe (12)**: Austria, Belgium, Denmark, Finland, Germany, Greenland, Iceland, Luxembourg, Netherlands, Norway, Sweden, Switzerland
	**South Western Europe (9)**: Andorra, Cyprus, France, Greece, Israel, Italy, Malta, Portugal, Spain
**Central and Eastern Europe (20)**	**Central Europe (13)**: Albania, Bosnia and Herzegovina, Bulgaria, Croatia, Czech Republic, Hungary, Macedonia (TFYR), Montenegro, Poland, Romania, Serbia, Slovakia, Slovenia
	**Eastern Europe (7)**: Belarus, Estonia, Latvia, Lithuania, Moldova, Russian Federation, Ukraine

* Although high-income English-speaking countries are geographically separated, they exhibit remarkably similar trends in cardiometabolic risk factors and outcomes.[5, 8-10] They were therefore grouped together so that the statistical model shares information amongst them more than it does with other countries that are geographically closer but epidemiologically more distinct.

We did not have data on population by age group for American Samoa, Bermuda, Greenland, and Tokelau. Country-specific estimates were made but were not used in calculation of regional and global prevalences because the latter requires weighting by age-specific population.

Appendix Table 2: Data sources used in the analysis.

Country	Data years	Survey/study name/citation	Level of representative-ness	Rural, urban, or both	Age range as used for global analysis		Sample size		Note
					Female	Male	Female	Male	
Afghanistan	2013	National Nutrition Survey	National	both	10-49		18433		
Albania	2001	Shapo et al., Public Health Nutr 6:471-77, 2003	Community	urban	24+	24+	585	535	
Albania	2008-2009	DHS	National	both	15-49	15-49	7386	2978	
Albania	2013	Balkan Survey of Inactivity in Children (BASIC)	Subnational	both	5-16	5-16	4985	4986	
Albania	2013	Childhood Obesity Surveillance Initiative 3	National	both	6-9	6-9	1606	1714	10
Algeria	2003	STEPS	Community	both	25-64	25-64	2435	1612	
Algeria	2005	Transition and Health Impact in North Africa	National	both	35-70	35-70	2737	2004	
Algeria	2007-2009	The ISOR (InSulino-resistance in ORan) study	Community	urban	30-64	30-64	409	378	
American Samoa	1976-1978	McGarvey, Am J Clin Nutr 53(6 Suppl):1586S-1594S, 1991	National	both	5+	5+	1329	1017	
American Samoa	1990	McGarvey, Pac Health Dialog 8(1):157-62, 2001	National	both	25+	25+	484	359	
American Samoa	1994	McGarvey, Pac Health Dialog 8(1):157-62, 2001	National	both	25+	25+	241	165	
American Samoa	2004	STEPS	National	both	25-64	25-64	1060	949	
Antigua and Barbuda	2009	Global School-based Student Health Survey	National	both	13-17	13-17	122	70	
Argentina	1981-1985	Hernandez et al., Diabetes Res Clin Pract 3:277-83, 1987	Community	urban	20-74	20-74	414	395	
Argentina	1985-1986	INTERSALT	Community	urban	20-59	20-59	100	100	
Argentina	1995-1998	de Sereday et al., Diabetes Metab 30:335-9, 2004	Subnational	urban	25-74	25-74	1246	924	
Argentina	2003	CEDES-Programa VIGI+A-Banco Mundial, 2004	Community	urban	25-74	25-74	176	151	
Argentina	2004-2005	CArdiovascular Risk factors Multiple Evaluation in Latin America	Community	urban	25-64	25-64	742	733	
Argentina	2005	Encuensta Nacional de Nutricion y Salud 2005	National	both	10-49		6582		
Argentina	2006	Virasoro Survey	Community	urban	25-84	25-84	306	261	
Argentina	2008-2011	The VELA Project	Community	rural	5+	5+	543	380	
Argentina	2011-2012	CESCAS Study	Community	urban	35-74	35-74	2395	1584	
Argentina	2012	Global School-based Student Health Survey	National	both	13-17	13-17	8906	8337	
Armenia	1998	The health and nutritional status of children and women in Armenia	National	both	18-45		2420		
Armenia	2000	DHS	National	both	15-49		5982		
Armenia	2005	DHS	National	both	15-49	15-49	6232	1220	
Armenia	2016	STEPS	National	both	18-69	18-69	1447	604	
Australia	1975	Busselton Health Study	Community	urban	18+	18+	418	373	
Australia	1978	Busselton Health Study	Community	urban	18+	18+	550	462	
Australia	1980	Risk Factor Prevalence Study	National	urban	25-64	25-64	2781	2756	
Australia	1981	Busselton Health Study	Community	urban	18+	18+	290	225	
Australia	1983	MONICA, Newcastle	Subnational	urban	35-64	35-64	1244	1215	
Australia	1983	Risk Factor Prevalence Study	National	urban	25-64	25-64	3813	3731	
Australia	1985	Austrailian Council for Health, Physical Education and Recreation survey	National	both	7-12	7-12	702	707	
Australia	1988-1989	Dubbo Study of Australian Elderly	Community	urban	59+	59+	1219	877	
Australia	1988-1989	MONICA, Newcastle	Subnational	urban	35-64	35-64	671	672	
Australia	1988-1989	MONICA, Newcastle	Community	urban	25-34	25-34	84	70	
Australia	1989	Risk Factor Prevalence Study	National	urban	20-69	20-69	4678	4497	
Australia	1992-1993	Australia Longitudinal Study of Ageing	Community	urban	65+	65+	746	814	
Australia	1994	MONICA, Newcastle	Subnational	urban	35-64	35-64	688	637	
Australia	1994	MONICA, Perth inner	Community	urban	25-64	25-64	349	363	
Australia	1994	MONICA, Perth outer	Community	urban	25-64	25-64	387	373	
Australia	1995	National Nutrition Survey	National	both	5+	5+	6390	5983	
Australia	1996	The Nepean Longitudinal Cohort Study	Community	urban	7-8	7-8	215	221	
Australia	1996-1998	Western Australian AAA Screening Program	Community	urban		65-84		12194	
Australia	1999-2003	North West Adelaide Health Study	Community	urban	18+	18+	2122	1932	
Australia	1999-2000	The Australian Diabetes, Obesity and Lifestyle Study 1999-2000	National	both	25+	25+	6070	4991	
Australia	2000	Perth children	Community	both	25	25	334	266	
Australia	2004	The Longitudinal Study of Australian Children, K cohort (child)	National	both	5	5	431	425	
Australia	2004	The Nepean Longitudinal Cohort Study	Community	urban	14-15	14-15	149	143	
Australia	2004-2005	Janus et al., Med J Aust 187:147-52, 2007	Community	rural	25-74	25-74	423	383	
Australia	2004-2005	The Australian Diabetes, Obesity and Lifestyle Study 2004-2005	National	both	30+	30+	3472	2874	
Australia	2004-2006	North West Adelaide Health Study	Community	urban	20+	20+	1679	1523	
Australia	2006	The Longitudinal Study of Australian Children, K cohort (child)	National	both	6-7	6-7	2156	2245	
Australia	2007-2008	National Health Survey	National	both	18+	18+	5655	5279	
Australia	2008	The Longitudinal Study of Australian Children, B cohort (infant)	National	both	5	5	513	508	
Australia	2008	The Longitudinal Study of Australian Children, K cohort (child)	National	both	8-9	8-9	2023	2121	
Australia	2008-2010	North West Adelaide Health Study	Community	urban	24+	24+	1318	1168	
Australia	2010	The Longitudinal Study of Australian Children, B cohort (infant)	National	both	6-7	6-7	2014	2148	
Australia	2010	The Longitudinal Study of Australian Children, K cohort (child)	National	both	10-11	10-11	1945	2052	
Australia	2011-2012	Australian Health Survey	National	both	5+	5+	13011	12190	

Country	Data years	Survey/study name/citation	Level of representative-ness	Rural, urban, or both	Age range as used for global analysis		Sample size		Note
					Female	Male	Female	Male	
Australia	2012	The Longitudinal Study of Australian Children, B cohort (infant)	National	both	8-9	8-9	1947	2041	
Australia	2012	The Longitudinal Study of Australian Children, K cohort (child)	National	both	12-13	12-13	1851	1948	
Australia	2012	The Australian Diabetes, Obesity and Lifestyle Study 2012	National	both	37+	37+	2526	2048	
Australia	2014-2015	National Health Survey	National	both	5-18	5-18	1152	1152	
Austria	1977	Rhomberg, Wien Klin Wochenschr Suppl 127:1-30, 1981	Community	rural	20-64	20-64	316	295	
Austria	1986	CINDI	Community	both	25-64	25-64	715	657	
Austria	1990	The Austrian Conscription Database	National	both		17		47004	
Austria	1991	The Austrian Conscription Database	National	both		17		43864	
Austria	1992	The Austrian Conscription Database	National	both		17		42934	
Austria	1992	Vorarlberg Health Monitoring and Promotion Programme	Subnational	rural	18+	18+	18835	14161	
Austria	1993	The Austrian Conscription Database	National	both		17		41533	
Austria	1994	The Austrian Conscription Database	National	both		17		39354	
Austria	1995	The Austrian Conscription Database	National	both		17		38776	
Austria	1996	The Austrian Conscription Database	National	both		17		38618	
Austria	1997	The Austrian Conscription Database	National	both		17		39318	
Austria	1998	The Austrian Conscription Database	National	both		17		41909	
Austria	1998	Vorarlberg Health Monitoring and Promotion Programme	Subnational	rural	18+	18+	20915	16153	
Austria	1999	The Austrian Conscription Database	National	both		17		43076	
Austria	2000	The Austrian Conscription Database	National	both		17		43025	
Austria	2001	The Austrian Conscription Database	National	both		17		41847	
Austria	2002	The Austrian Conscription Database	National	both		17		41633	
Austria	2003	The Austrian Conscription Database	National	both		17		41627	
Austria	2004	The Austrian Conscription Database	National	both		17		41847	
Austria	2004	Vorarlberg Health Monitoring and Promotion Programme	Subnational	rural	18+	18+	23893	20160	
Austria	2004-2005	Vorarlberg Health Monitoring and Promotion Programme (VHM&PP)	Subnational	both	6-17	6-17	15823	17504	
Austria	2005	The Austrian Conscription Database	National	both		17		41829	
Austria	2006	The Austrian Conscription Database	National	both		17		43128	
Austria	2006-2007	HELENA	Community	urban	12-17	12-17	211	191	
Austria	2007	The Austrian Conscription Database	National	both		17		43299	
Austria	2008	The Austrian Conscription Database	National	both		17		43294	
Austria	2008-2009	Vorarlberg Health Monitoring and Promotion Programme (VHM&PP)	Subnational	both	6-17	6-17	15295	16847	
Austria	2009	The Austrian Conscription Database	National	both		17		44095	
Austria	2010	The Austrian Conscription Database	National	both		17		43390	
Austria	2010-2012	Austrian Study on Nutritional Status 2012	National	both	6-14	6-15	190	193	
Austria	2011	The Austrian Conscription Database	National	both		17		42755	
Austria	2012	The Austrian Conscription Database	National	both		17		41513	
Austria	2012-2013	Vorarlberg Health Monitoring and Promotion Programme (VHM&PP)	Subnational	both	6-17	6-17	13650	14759	
Austria	2013	The Austrian Conscription Database	National	both		17		40026	
Austria	2015-2016	Early vascular ageing (EVA)	Subnational	both	14-17	14-17	649	557	
Austria	2015-2016	Vorarlberg Health Monitoring and Promotion Programme (VHM&PP)	Subnational	both	6-17	6-17	12056	13041	
Azerbaijan	1996	Health and Nutrition Survey	National	both	19-59	19-59	295	121	
Azerbaijan	2001	Reproductive Health Survey (RHS)	National	both	20-44		1726		
Azerbaijan	2006	DHS	National	both	15-49	15-59	7868	2493	
Bahamas	2013	Global School-based Student Health Survey	National	both	13-17	13-17	533	460	
Bahrain	1991-1992	al-Mannai et al., J R Soc Health 116:30-2, 7-40, 1996	Community	both	20+	20+	153	137	
Bahrain		Musaiger et al., Ann Hum Biol 28:346-50, 2001	Community	both	30+	30+	216	298	
Bahrain	1998-1999	National Nutrition Survey	National	both	19+	19+	1181	1120	
Bahrain	2001-2004	Global Database on growth and malnutrition of school children and adolescents, WHO	National	both	6-20	6-19	1326	1268	
Bahrain	2007	STEPS	National	both	20-64	20-64	858	854	
Bahrain	2016	Global School-based Student Health Survey	National	both	13-17	13-17	2727	2979	
Bangladesh		Rahman et al., Hypertension 33:74-8, 1999	Community	rural	30+	30+	643	965	
Bangladesh	1996-1997	DHS	National	both	20-49		3384		
Bangladesh	1998	Zaman et al., J Health Popul Nutr 21:162-63, 2003	Community	rural	20+	20+	379	290	
Bangladesh	1999-2000	Hussain et al., Eur J Public Health, 17:291-96, 2007	Community	rural	20-59	20-59	2720	2037	
Bangladesh	1999-2000	DHS	National	both	20-49		3889		
Bangladesh	2000-2004	Nutritional Surveillance Project	National	rural	20-45		224251		
Bangladesh	2002	STEPS	National	rural	25-64	25-64	2038	2086	
Bangladesh	2002	STEPS	National	urban	25-64	25-64	3737	3533	
Bangladesh	2004	DHS	National	both	20-49		9165		
Bangladesh	2007	DHS	National	both	20-49		9037		
Bangladesh	2011	DHS	National	both	20+	15+	16679	5254	

Country	Data years	Survey/study name/citation	Level of representative-ness	Rural, urban, or both	Age range as used for global analysis		Sample size		Note
					Female	Male	Female	Male	
Bangladesh	2014	DHS	National	both	20-49		14963		
Bangladesh	2015	An assessment of BRAC Health Nutrition and Population Programme and benchmark survey of Sustainable Development Goal – 2015	National	rural	11+	35+	18227	5432	
Barbados	1987-1992	Barbados Eye Study	National	both	40-84	40-84	2627	1980	
Barbados	1991-1994	Cooper et al., Am J Public Health 87(2):160-68, 1997	Community	urban	25-100	25-100	482	329	
Barbados	1997-2002	The Barbados Incidence Studies of Eye Diseases II	National	both	40-84	40-84	1441	1004	
Barbados	1999-2000	The Survey on Health, Well-Being, and Aging in Latin America and the	Community	urban	60+	60+	866	559	2
Barbados	2011	Global School-based Student Health Survey	National	both	13-17	13-17	708	628	
Barbados	2011-2013	Health of the Nation (HotN)	National	both	25+	25+	703	455	
Belarus	2016	STEPS	National	both	18-69	18-69	2894	2085	
Belgium	1983-1985	MONICA, Luxembourg	Community	urban	35-64	35-64	936	944	
Belgium	1984-1985	Belgian Interuniversity Research on Nutrition and Health	National	both	25-74	25-74	5289	5897	
Belgium	1985-1987	INTERSALT, Charleroi	Community	urban	20-59	20-59	75	82	
Belgium	1985-1986	INTERSALT, Ghent	Community	urban	20-59	20-59	100	100	
Belgium	1985-1987	MONICA, Charleroi	Community	urban	25-64	25-64	327	347	
Belgium	1985-1987	MONICA, Ghent	Community	urban	25-64	25-64	459	549	
Belgium	1985-1990	Flemish Study on Environment, Genes and Health Outcomes	Community	rural	20-90	20-90	692	656	
Belgium	1987-1990	MONICA, Charleroi	Community	urban	25-64	25-64	301	325	
Belgium	1988-1990	MONICA, Ghent	Community	urban	25-64	25-64	449	456	
Belgium	1990-1993	MONICA, Charleroi	Community	urban	25-64	25-64	332	337	
Belgium	1990-1992	MONICA, Ghent	Community	urban	25-64	25-64	475	507	
Belgium	1991-1994	Flemish Study on Environment, Genes and Health Outcomes	Community	rural	26-88	26-88	416	393	
Belgium	1992-1995	Flemish Study on Environment, Genes and Health Outcomes	Community	rural	27-89	27-89	312	298	
Belgium	1994-1996	BIRNH Elderly: Belgian Interuniversity Research on Nutrition and Health in the Elderly	National	both	65-89	65-89	953	1142	
Belgium	1996-1998	Flemish Study on Environment, Genes and Health Outcomes	Community	rural	10-84	10-84	402	404	
Belgium	1998-2000	Flemish Study on Environment, Genes and Health Outcomes	Community	rural	10-80	10-80	217	220	
Belgium	1998	Flemish Study on Environment, Genes and Health Outcomes	Community	rural	32-86	32-86	349	320	
Belgium	1999-2001	Flemish Study on Environment, Genes and Health Outcomes	Community	rural	10-81	10-81	254	232	
Belgium	2001	Flemish Study on Environment, Genes and Health Outcomes	Community	rural	10-78	10-78	214	242	
Belgium	2002-2003	Flemish Study on Environment, Genes and Health Outcomes	Community	rural	10-81	10-81	197	174	
Belgium	2002-2005	Flemish Study on Environment, Genes and Health Outcomes	Community	rural	10-88	10-88	462	447	
Belgium	2002-2004	SPAH	Subnational	both	18-75	18-75	2308	2595	
Belgium	2003	The European Male Ageing Study	Community	both		40+		433	
Belgium	2005-2008	Flemish Study on Environment, Genes and Health Outcomes	Community	rural	10-89	10-89	470	462	
Belgium	2006-2007	HELENA	Community	urban	12-17	12-17	180	156	
Belgium	2007-2008	Childhood Obesity Surveillance Initiative 1	Subnational	both	6-9	6-9	61755	64322	10
Belgium	2007-2010	Identification and prevention of Dietary- and lifestyle-induced health EFfects In Children and infantS (IDEFICS)	Community	urban	5-9	5-9	834	822	
Belgium	2008	The European Male Ageing Study	Community	both		40+		383	
Belgium	2009-2010	Childhood Obesity Surveillance Initiative 2	Subnational	both	6-9	6-9	65370	67781	10
Belgium	2009-2013	Flemish Study on Environment, Genes and Health Outcomes	Community	rural	20-88	20-88	335	330	
Belgium	2010-2015	Flemish Study on Environment, Genes and Health Outcomes	Community	rural	15-87	15-87	409	388	
Belgium	2012-2013	Childhood Obesity Surveillance Initiative 3	Subnational	both	6-9	6-9	67876	70442	10
Belgium	2014-2015	Food Consumption Survey	National	both	5-64	5-64	1491	1481	
Belize	2004-2005	CAMDI	National	both	20+	20+	1019	599	
Belize	2011	Global School-based Student Health Survey	National	both	13	13	188	163	
Benin	1996	DHS	National	both	20-49		2138		
Benin	2001	DHS	National	both	15-49		5449		
Benin	2006	DHS	National	both	15-49		14898		
Benin	2007	STEPS	Community	urban	25-64	25-64	1508	955	
Benin	2008	STEPS	National	both	25-64	25-64	3365	3430	
Benin	2009	Global School-based Student Health Survey	National	both	13	13	45	101	
Benin	2011-2012	DHS	National	both	15-49		14592		
Benin	2016	Global School-based Student Health Survey	National	both	13	13	67	54	
Bhutan	2007	STEPS	Community	urban	25-74	25-74	1318	1125	
Bhutan	2014	STEPS	National	both	18-69	18-69	1674	1069	
Bolivia	1994	DHS	National	both	20-49		2128		
Bolivia	1998	DHS	National	both	20-49		3941		
Bolivia	2003	DHS	National	both	15-49		16349		
Bolivia	2005-2007	Baya Botti et al., Nutr Hosp 24(3):304-11, 2009	National	both	12-18	12-18	1841	1499	
Bolivia	2005	Reyes-Garcia et al., Soc Sci Med 69:571-78, 2009	Community	rural		25-84		206	
Bolivia	2008	DHS	National	both	15-49		15543		
Bolivia	2012	Global School-based Student Health Survey	National	both	13-15	13-15	1299	1264	

Country	Data years	Survey/study name/citation	Level of representative-ness	Rural, urban, or both	Age range as used for global analysis		Sample size		Note
					Female	Male	Female	Male	
Bosnia and Herzegovina	2002	Non-communicable disease risk factor survey, Federation of B&H	Subnational	both	25-64	25-64	1613	1118	
Bosnia and Herzegovina	2012	Non-communicable disease risk factor survey, Federation of B&H	Subnational	rural	18+	18+	1274	1191	
Bosnia and Herzegovina	2012	Non-communicable disease risk factor survey, Federation of B&H	Subnational	urban	18+	18+	697	591	
Botswana	2007	STEPS	National	both	25-64	25-64	2577	1243	
Botswana	2014	STEPS	National	both	15-69	15-69	2602	1298	
Brazil	1989	Pesquisa Nacional sobre Saude e Nutricao	National	both	5+	5+	27504	26642	
Brazil	1990-1991	Fornes et al., Rev Saude Publica 36:12-8, 2002	Community	urban	20+	20+	613	432	
Brazil	1991-1993	EPIDOSO	Community	urban	65+	65+	473	269	
Brazil	1992-1998	Moraes et al., Int J Cardiol 90:205-11, 2003	Community	urban	18+	18+	543	438	
Brazil	1995	The 1982 Pelotas (Brazil) Birth Cohort: 13 years follow-up	Community	urban	13	13	363	352	
Brazil	1995-1996	Cohort study from Porto Alegre	Community	urban	18+	18+	596	489	
Brazil	1995	Health and Nutrition Survey of Rio de Janeiro	Community	urban	60+	60+	385	248	
Brazil	1996-1997	The Bambui Cohort Study of Ageing	Community	urban	18+	18+	1335	931	
Brazil	1996	DHS	National	both	20-49		2884		
Brazil	1997	The 1982 Pelotas (Brazil) Birth Cohort: 15 years follow-up	Community	urban	15	15	513	559	
Brazil	1997	PPV	Subnational	both	20+	20+	9121	8063	
Brazil	1998	Belo Horizonte Heart Study	Community	urban	6-19	6-19	740	660	
Brazil	1999-2000	Pelotas cross-sectional survey	Community	urban	20-69	20-69	1096	839	
Brazil	1999-2000	The Survey on Health, Well-Being, and Aging in Latin America and the	Community	urban	60+	60+	1064	732	2
Brazil	2000	The 1982 Pelotas (Brazil) Birth Cohort: 18 years follow-up	Community	urban		18		2228	
Brazil	2001	The 1982 Pelotas (Brazil) Birth Cohort: 19 years follow-up	Community	urban	19		919		
Brazil	2001	de Freitas et al., Arq Bras Cardiol 88:191-99	Community	urban	15+	15+	331	310	
Brazil	2001-2003	Bustos et al., Nutr Metab Cardiovasc Dis 17:581-89, 2007	Community	both	22-28	22-28	1064	992	
Brazil	2002-2003	Pesquisa de Orcamentos Familiares	National	both	5+	5+	80162	81154	
Brazil	2003	PNAFS	Community	urban	20+	20+	1941	1155	
Brazil	2003	Women health in Southern Brazil	Community	urban	20-60		986		
Brazil	2003-2005	São Paulo Health and Ageing Study	Community	urban	65+	65+	1198	783	
Brazil	2004-2006	Hearts of Brazil	National	urban	18+	18+	626	550	
Brazil	2004-2005	The 1993 Pelotas (Brazil) Birth Cohort: 11 years follow-up	Community	urban	10-12	10-12	2257	2184	
Brazil	2004-2005	Ribeira Preto Birth Cohort	Community	urban	10-11	10-11	388	400	
Brazil	2004-2005	Pimenta et al., Arq Bras Cardiol 90:386-92, 2008	Community	rural	18-84	18+	286	291	
Brazil	2004-2005	The 1982 Pelotas (Brazil) Birth Cohort: 23years follow-up	Community	urban	23	23	1935	2173	
Brazil	2005-2006	Sao Luis Birth Cohort	Community	urban	7-8	7-8	325	347	
Brazil	2006	ATITUDE	Subnational	both	14-21	14-21	3484	2406	
Brazil	2006	The Ouro Preto Study	Community	urban	7-14	7-14	399	364	
Brazil	2006	Pesquisa Nacional de Demografia e Saude 2006	National	both	15-49		14783		
Brazil	2006-2007	SOFT study	Community	urban	18+	18+	1099	739	
Brazil	2006	Krause et al., J Aging Phys Act 17:387-97, 2009	Community	urban	60+	60+	1069	93	
Brazil	2007-2008	Scan J Med Sci Sports, 23(3):317-22	Community	urban	10-16	10-16	528	493	
Brazil	2008	The 1993 Pelotas (Brazil) Birth Cohort: 15 years follow-up	Community	urban	14-15	14-15	2095	2001	
Brazil	2008-2009	Pesquisa de Orcamentos Familiares	National	both	5+	5+	88152	85729	
Brazil	2008	The Bambui Cohort Study of Ageing	Community	urban	71+	71+	456	248	
Brazil	2009-2010	EpiFloripa Cohort Study of Ageing - Wave 1	Community	urban	60+	60+	1047	592	
Brazil	2010	Longitudinal Study of Health and Wellbeing in Preschool Age (Project ELOS-Pré)	Community	urban	5	5	247	255	
Brazil	2010-2011	The 2004 Pelotas (Brazil) Birth Cohort: 6 years follow-up	Community	urban	6-7	6-7	1632	1721	
Brazil	2010-2015	Baependi Heart Study	Community	urban	18+	18+	1125	780	
Brazil	2011	ATITUDE	Subnational	both	14-19	14-19	3658	2420	
Brazil	2011-2012	The 1993 Pelotas (Brazil) Birth Cohort: 18 years follow-up	Community	urban	17-19	17-19	2004	1970	
Brazil	2012	Longitudinal Study of Health and Wellbeing in Preschool Age (Project ELOS-Pré)	Community	urban	5-7	5-7	348	388	
Brazil	2012-2013	Prevalence of Leptin Polymorphism Gln223Arg	Community	urban	18+	18+	523	282	
Brazil	2012-2013	The 1982 Pelotas (Brazil) Birth Cohort: 30 years follow-up	Community	urban	30	30	1798	1753	
Brazil	2013	Pesquisas Nacional de Saude	National	both	18+	18+	32351	24918	
Brazil	2013-2014	EpiFloripa Cohort Study of Ageing - Wave 2	Community	urban	63+	63+	744	404	
Brazil	2014	Brazilian Guide to the Physical Fitnees related to Health Assessment and Lifestyle Habits	Community	urban	14-19	14-19	535	473	
Brazil	2014	Longitudinal Study of Health and Wellbeing in Preschool Age (Project ELOS-Pré)	Community	urban	7-9	7-9	204	232	
Brazil	2015	The 2004 Pelotas (Brazil) Birth Cohort: 11 years follow-up	Community	urban	10-11	10-11	1632	1736	
Brazil	2015-2016	The 1993 Pelotas (Brazil) Birth Cohort: 22-23 years follow-up	Community	urban	21-23	21-23	1872	1687	
Brunei Darussalam	2010-2011	National Health and Nutritional Status Survey (NHANSS)	National	both	5-75	5-75	1157	1027	
Brunei Darussalam	2015-2016	National Non-Communicable Diseases Survey (NNCDS)	National	both	18-69	18-69	1075	814	
Bulgaria	2004	National Nutrition Survey	National	both	20+	20+	515	515	
Bulgaria	2008	Childhood Obesity Surveillance Initiative 1	National	both	6-9	6-9	1255	1256	10

Country	Data years	Survey/study name/citation	Level of representative-ness	Rural, urban, or both	Age range as used for global analysis		Sample size		Note
					Female	Male	Female	Male	
Bulgaria	2013	Childhood Obesity Surveillance Initiative 3	National	both	6-9	6-9	1677	1671	10
Burkina Faso	1992-1993	DHS	National	both	20-49		3190		
Burkina Faso	1998-1999	DHS	National	both	20-49		3114		
Burkina Faso	2002	Vulnérabilité Alimentaire et Sécurité Nutritionnelle dans la Gnagna (VASN-Gnagna)	Subnational	rural	5+	5+	3522	1471	
Burkina Faso	2003	DHS	National	both	15-49		11001		
Burkina Faso	2004	Ouedraogo et al., Public Health Nutr 11:1280-87, 2008	Community	urban	35+	35+	1066	956	
Burkina Faso	2010	DHS	National	both	15-49		7755		
Burkina Faso	2013	STEPS	National	both	25-64	25-64	2250	2223	
Burundi	2010	DHS	National	both	15-49		4188		
Cabo Verde	2007	STEPS	National	both	25-64	25-64	1066	658	
Cambodia	2000	DHS	National	both	15-49		6915		
Cambodia	2005	DHS	National	both	15-49		8131		
Cambodia	2008	Anthropometrics Survey	National	both	20-49		5955		
Cambodia	2010	DHS	National	both	15-49		8856		
Cambodia	2010	STEPS	National	both	25-64	25-64	3343	1881	
Cambodia	2013	Global School-based Student Health Survey	National	both	13	13	228	175	
Cambodia	2014	DHS	National	both	15-49		10821		
Cameroon	1998-1999	ENHIP	Community	rural	15+	15+	735	523	
Cameroon	1998-1999	ENHIP	Community	urban	15+	15+	640	523	
Cameroon	1998	DHS	National	both	20-49		1429		
Cameroon	2003	STEPS	Subnational	urban	15+	15+	5490	3672	
Cameroon	2004	DHS	National	both	15-49		4646		
Cameroon	2007	Cameroon Burden of Diabetes - Second Survey	Subnational	urban	18+	18+	4123	3122	
Cameroon	2009	National Survey of Micronutrient Status and Consumption of Fortifiable Foods	National	both	15-49		816		
Cameroon	2011	DHS	National	both	15-49		7343		
Canada	1981	Canada Fitness Survey	National	both	7-64	7-64	7940	7432	
Canada	1985-1986	INTERSAL, StJohns	Community	urban	20-59	20-59	100	100	
Canada	1985-1988	MONICA, Halifax	Community	both	25-64	25-64	420	438	
Canada	1986-1992	Canada Heart Health Survey	National	both	18-74	18-74	9777	9644	
Canada	1991-1992	Canadian Study of Health and Aging	Community	both	70+	70+	348	236	
Canada	1993	Chen et al., Int J Obes Relat Metab Disord 22:771-77, 1998	Community	rural	18-74	18-74	988	803	
Canada	1995	MONICA, Halifax	Community	both	25-64	25-64	287	274	
Canada	1995-1997	CaMos	Subnational	both	25+	25+	6302	2792	
Canada	1996	Canadian Study of Health and Aging	Community	both	70+	70+	348	236	
Canada		PEI Nutrition Survey	Subnational	both	18-74	18-74	995	1000	
Canada	2005	CCHS	National	both	15+	15+	2031	1684	
Canada	2005-2008	Candian Multicentre Osteoporosis Study (CaMos)	Subnational	both	35+	35+	3661	1486	
Canada	2007-2009	Canadian Health Measures Survey, Cycle 1	National	both	6-79	6-79	2864	2703	
Canada	2008	CCHS	National	both	15+	15+	1988	1689	
Canada	2009-2011	Canadian Health Measures Survey, Cycle 2	National	both	5-79	5-79	3086	2870	
Canada	2012-2013	Canadian Health Measures Survey, Cycle 3	National	both	6-79	5-79	2676	2670	
Canada	2014-2015	Canadian Health Measures Survey, Cycle 4	National	both	5-79	5-79	2674	2697	
Central African Republic	1994-1995	DHS	National	both	20-49		1760		
Central African Republic	2010	STEPS	Community	both	25-64	25-64	1967	1846	
Chad	1996-1997	DHS	National	both	20-49		3262		
Chad	2004	DHS	National	both	20-49		2618		
Chad	2008	STEPS	Community	urban	25-64	25-64	845	995	
Chad	2014-2015	DHS	National	both	15-49		9733		
Chile	1989	INCLEN	Community	urban		35-65		199	
Chile	1992-1993	Miquel et al., Gastroenterology 115: 937-46, 1998	Community	urban	18+	18+	1031	657	
Chile	1999-2000	The Survey on Health, Well-Being, and Aging in Latin America and the	Community	urban	60+	60+	806	410	2
Chile	2000	Nervi et al., J Hepatol 45: 299 -305, 2006	Community	urban	18+	18+	624	335	
Chile	2001-2003	Bustos et al., Nutr Metab Cardiovasc Dis 17:581-89, 2007	Community	both	22-28	22-28	562	436	
Chile	2003	Encuesta Nacional de Salud	National	both	17+	17+	1867	1557	
Chile	2004-2005	CArdiovascular Risk factors Multiple Evaluation in Latin America	Community	urban	25-64	25-64	865	783	
Chile	2005	Palomo et al., Rev Med Chil 135:904-12, 2007	Community	urban	18-74	18-74	668	339	
Chile	2009-2010	Encuesta Nacional de Salud	National	both	15+	15+	2930	1977	
Chile	2011-2012	CESCAS Study	Community	urban	35-74	35-74	1027	922	
Chile	2013	Global School-based Student Health Survey	National	both	13-17	13-17	793	799	
China	1979-1982	East Beijing Study 1	Community	urban	20-84	20-84	380	361	
China	1982	China National Nutrition Survey	National	both	15-94	15-94	13683	14418	

Country	Data years	Survey/study name/citation	Level of representative-ness	Rural, urban, or both	Age range as used for global analysis		Sample size		Note
					Female	Male	Female	Male	
China	1983	Sino-MONICA Shanghai	Subnational	both	30-64	30-64	630	624	
China	1983-1984	Wang et al., Zhonghua Liu Xing Bing Xue Za Zhi 24:272-75, 2003	Community	both	25-64	25-64	832	877	
China	1984-1985	MONICA, Beijing	Community	both	25-64	25-64	856	813	
China	1984-1985	Sino-MONICA Beijing	Subnational	both	25-64	25-64	857	813	
China	1985	Chinese National Surveys on Students Constitution and Health	National	both	7-18	7-18	204796	205041	
China	1986	INTERSALT, Beijing	Community	urban	20-59	20-59	100	100	
China	1986	INTERSALT, Nanning	Community	both	20-59	20-59	100	100	
China	1986	INTERSALT, Tianjin	Community	urban	20-59	20-59	100	100	
China	1986-1989	Ewang et al., Zhonghua Liu Xing Bing Xue Za Zhi 26:394-9, 2005	Community	both		45-64		18244	
China	1986-1989	Sino-MONICA Shanghai	Subnational	both	25-64	25-64	753	675	
China	1987	INCLEN	Community	urban		35-65		989	
China	1988	Sino-MONICA Hebei	Subnational	both		25-64		800	
China	1988	Sino-MONICA Heilongjiang	Subnational	both	25-64	25-64	800	800	
China	1988	Sino-MONICA Henan	Subnational	both	25-64	25-64	427	345	
China	1988	Sino-MONICA Neimenggu	Subnational	both	25-64	25-64	400	396	
China	1988	Sino-MONICA Sichuan	Subnational	both	25-64	25-64	334	312	
China	1988	Sino-MONICA Shandong	Subnational	both	25-64	25-64	225	211	
China	1988-1989	Wang et al., Zhonghua Liu Xing Bing Xue Za Zhi 24:272-75, 2003	Community	both	25-64	25-64	731	873	
China	1988-1990	East Beijing Study 2	Community	urban	20-84	20-84	148	135	
China	1988-1989	MONICA, Beijing	Community	both	25-64	25-64	862	701	
China	1988-1989	Sino-MONICA Beijing	Subnational	both	25-64	25-64	862	701	
China	1988-1989	Sino-MONICA Jilin	Subnational	both	25-64	25-64	400	380	
China	1988-1989	Sino-MONICA Jiangxi	Subnational	both	25-64	25-64	386	379	
China	1988-1989	Sino-MONICA Liaoning	Subnational	both	25-64	25-64	734	728	
China	1989	China Health and Nutrition Study	National	both	5-45	5-45	2716	2556	3
China	1989	The Tianjin Project	Community	urban	25-64	25-64	3971	3894	
China	1989	Sino-MONICA Fujian	Subnational	both	25-64	25-64	191	179	
China	1989	Sino-MONICA Jiangsu	Subnational	both	25-64	25-64	399	398	
China	1990-1991	China Prospective Study	National	both		40-79		221194	
China	1991	China Health and Nutrition Study	National	both	5+	5+	5921	5580	3
China		Hua et al., Zhonghua Nei Ke Za Zhi 36:18-20, 1997	Community	rural	60+	60+	335	288	
China	1991	China National Hypertension Survey Epidemiology Follow-up Study	National	both	40+	40+	79040	75696	
China	1991	Sino-MONICA Shanghai	Subnational	both	30-64	30-64	624	564	
China	1991-1992	Fangshan Cohort Study	Community	urban	34-86	34-86	1736	871	
China	1992	China National Nutrition Survey	National	both	15-92	15-92	36271	33714	
China	1992-1993	Anzhen 02 Cohort Study	Community	urban	34-65	34-65	2120	2032	
China	1992	Huashan Study	Community	urban	35-75	35-75	965	892	
China	1992	Sino-MONICA Sichuan	Subnational	both	25-64	25-64	526	608	
China	1993	China Health and Nutrition Study	National	both	5+	5+	5563	5367	3
China	1993	Wang et al., Zhonghua Liu Xing Bing Xue Za Zhi 24:272-75, 2003	Community	both	25-64	25-64	617	822	
China	1993	MONICA, Beijing	Community	both	25-64	25-64	816	613	
China	1993	Sino-MONICA Anhui	Subnational	both	25-64	25-64	195	193	
China	1993	Sino-MONICA Beijing	Subnational	both	25-64	25-64	816	613	
China	1993	Sino-MONICA Jiangsu	Subnational	both	25-64	25-64	365	462	
China	1993	Sino-MONICA Liaoning	Subnational	both	25-64	25-64	500	493	
China	1995	Chinese National Surveys on Students Constitution and Health	National	both	7-18	7-18	101772	103009	
China	1996-2003	Wu et al., Osteoporos Int 15:751-59, 2004	Community	urban	18+		3418		
China	1996	Wang et al., Zhonghua Liu Xing Bing Xue Za Zhi 24:272-75, 2003	Community	both	25-64	25-64	721	735	
China	1996	The Tianjin Project	Community	urban	25-64	25-64	717	722	
China	1996-2000	Shanghai Women's Health Study	Community	urban	40-70		74915		
China	1997	China Health and Nutrition Study	National	both	5+	5+	5644	5546	3
China	1997	INTERMAP, Beijing	Community	rural	40-59	40-59	139	133	
China	1997	INTERMAP, Guangxi	Community	rural	40-59	40-59	138	140	
China	1997	INTERMAP, Shanxi	Community	rural	40-59	40-59	146	143	
China	1998	Shanghai Diabetes Study	Community	urban	25+	25+	1768	1264	
China	1998-2000	Jia et al., Obes Rev 3:157-65, 2002	Community	urban	20+	20+	1670	1106	
China		Chen et al., Zhonghua Yi Xue Za Zhi 85(40):2830-4, 2005	Subnational	both	35-85	35-85	10315	13549	
China	1999	Wang et al., Zhonghua Liu Xing Bing Xue Za Zhi 24:272-75, 2003	Community	both	25-64	25-64	685	818	
China	1999-2000	Xu et al., Public Health Nutr 8:47-51, 2005	Community	both	35+	35+	18902	18194	
China	2000	China Health and Nutrition Study	National	both	5+	5+	6097	5825	3
China	2000	Chinese National Surveys on Students Constitution and Health	National	both	7-18	7-18	108096	107997	

Country	Data years	Survey/study name/citation	Level of representative-ness	Rural, urban, or both	Age range as used for global analysis		Sample size		Note
					Female	Male	Female	Male	
China	2000-2001	The International Collaborative Study of Cardiovascular Disease in ASIA	National	both	35-74	35-74	8006	7512	
China	2001	Shanghai Diabetes Study	Community	urban	25+	25+	1768	1264	
China	2002	China National Nutrition and Health Survey	National	both	5-101	5-101	92687	84194	
China	2002	Ma et al., Zhonghua Liu Xing Bing Xue Za Zhi 25:1035-8, 2004	Subnational	both	18+	18+	7352	7352	
China	2002-2003	Fan et al., J Gastroenterol Hepatol 20:1825-32, 2005	Community	urban	25-74	25+	7767	5502	
China	2002-2006	Shanghai Men's Health Study	Community	urban		40-74		61445	
China	2004	Beijing Child and Adolescent Metabolic Syndrome study	Community	both	5-18	5-18	10412	10562	
China	2004	China Health and Nutrition Study	National	both	5+	5+	5507	5224	3
China	2004-2008	Xinjiang Children and Adolescent Survey	Subnational	both	7-18	7-18	2228	2025	
China	2004	Tian et al., Prev Med 48:59-63, 2009	Community	rural	15+	15+	1163313	1022669	
China	2004-2006	Pang et al., Intern Med 47:893-97, 2008	Community	rural	35+	35+	22962	22963	
China	2004-2006	Shanghai Women's Health Study	Community	urban	45-80		64545		
China	2004-2008	China Kadoorie Biobank baseline survey	Subnational	rural	35-74	35-74	162848	115792	
China	2004-2008	China Kadoorie Biobank baseline survey	Subnational	urban	35-74	35-74	132860	89219	
China	2004-2008	Shanghai Men's Health Study	Community	urban		41-80		54800	
China	2005	Chinese National Surveys on Students Constitution and Health	National	both	7-18	7-18	116704	117598	
China	2005	Ye et al., J Am Coll Cardiol 49:1798-805, 2007	Community	urban	50-70	50-70	906	743	
China	2005-2006	Zhou et al., World J Gastroenterol 13:6419-24, 2007	Community	urban	18-79	18-79	2063	1101	
China	2006	China Health and Nutrition Study	National	both	5+	5+	5397	4943	3
China	2006	Beijing Eye Study	Community	both	45+	45+	1820	1394	
China	2007	Beijing Child and Adolescent Metabolic Syndrome study	Community	urban	7-18	7-18	664	863	
China	2007-2008	China National Diabetes & Metabolic Disorders Study	National	both	20+	20+	27820	18419	
China	2007-2010	SAGE	National	both	50+	50+	6617	5759	
China	2007-2011	Shanghai Women's Health Study	Community	urban	47-83		52116		
China	2008	China Health and Retirement Longitudinal Study (CHARLS), pilot survey	Subnational	both	45+	45+	950	923	
China	2008-2010	Fangshan Family-based Ischemic Stroke Study in China (FISSIC) program	Community	rural	40+	40+	36449	19478	
China	2008-2011	Shanghai Men's Health Study	Community	urban		43-84		51948	
China	2009	China Health and Nutrition Study	National	both	5+	5+	5486	5174	3
China	2009	The nutrition-based comprehensive intervention study on childhood obesity in China	Subnational	urban	6-11	6-11	4269	4495	
China	2010	China Noncommunicable Disease Surveillance	National	both	18+	18+	53452	45066	
China	2010	Chinese National Surveys on Students Constitution and Health	National	both	7-18	7-18	107611	107611	
China	2011	Beijing Children Eye Study	Community	both	5-13	5-13	250	282	
China	2011	Beijing Pediatric Eye Study	Community	both	7-18	7-18	6968	6692	
China	2011	China Health and Nutrition Study	National	both	5+	5+	7478	6773	3
China	2011	Beijing Eye Study	Community	both	50+	50+	1895	1467	
China	2011-2012	China Health and Retirement Longitudinal Study (CHARLS), baseline survey	National	both	45+	45+	7005	6337	
China	2012	Shandong Children Study	Community	rural	5-18	5-18	1385	1663	
China	2012	Shandong Children Study	Community	urban	5-18	5-18	1378	1423	
China	2012-2013	The Kailuan Study	Community	urban	18+	18+	21385	80921	
China	2012	China Health and Retirement Longitudinal Study (CHARLS), wave 2 pilot survey	Subnational	both	45+	45+	934	856	
China	2012-2015	Shanghai Men's Health Study	Community	urban		47-87		40921	
China	2012-2015	Shanghai Women's Health Study	Community	urban	52-88		49592		
China	2013	Gobi Desert Children Eye Study	Community	both	6-21	6-21	761	800	
China	2013	China Health and Retirement Longitudinal Study (CHARLS), wave 2 survey	National	both	45+	45+	6582	5898	
China	2016	Greater Beijing School Children Myopia Study	Subnational	rural	6-18	6-18	12866	12873	
China	2016	Greater Beijing School Children Myopia Study	Subnational	urban	6-18	6-18	3650	4384	
China (Hong Kong SAR)	1985-1986	Shatin New Town Study	Community	urban	70+	70+	669	276	
China (Hong Kong SAR)	1991	The Hong Kong study on Health, health risk and quality of life in the Chinese elderly cohort	Community	both	70+	70+	944	943	
China (Hong Kong SAR)	1995-1996	Hong Kong Cardiovascular Risk Factor Prevalence Study 1995-1996	National	both	25-74	25-74	1478	1412	
China (Hong Kong SAR)	2005-2006	Hong Kong Growth Survey	National	both	7-19	7-19	7370	7472	
Colombia	1986	INTERSALT	Community	rural	20-59	20-59	95	96	
Colombia	1995	DHS	National	both	20-49		3068		
Colombia	2000	DHS	National	both	20-49		2929		
Colombia	2001	CINDI/CARMEN - Bucaramaga	Community	urban	25-74	25-74	1218	627	
Colombia	2002	CINDI/CARMEN - Bogota	Community	urban	25-74	25-74	570	322	
Colombia	2004-2005	CArdiovascular Risk factors Multiple Evaluation in Latin America	Community	urban	25-64	25-64	812	738	
Colombia	2005	DHS	National	both	5-64	5-64	57782	43440	
Colombia	2005	Encuesta Nacional de Situacion Nutricional	National	both	5-49	5-12	6088	2644	
Colombia	2007	Encuesta Nacional de Salud	National	both	18-69	18-69	7686	5462	
Colombia	2010	DHS	National	both	5-64	5-64	76793	65091	
Colombia	2010	STEPS	Subnational	both	15-64	15-64	1356	1034	

Country	Data years	Survey/study name/citation	Level of representative-ness	Rural, urban, or both	Age range as used for global analysis		Sample size		Note
					Female	Male	Female	Male	
Colombia	2014-2015	STEPS	Subnational	both	15-64	15-64	1177	978	
Comoros	1996	DHS	National	both	20-49		745		
Comoros	2011	STEPS	National	both	25-64	25-64	3500	1541	
Comoros	2012	DHS	National	both	15-49		4845		
Congo	1986	Enquête Brazzaville 1986	Community	urban	5-50	5-50	1079	129	
Congo	1986	Maire et al., Rev Epidemiol Sante Publique 40:252-58, 1992	Community	urban	25-45		558		
Congo	1987	Enquête Nationale Congo 1987	National	rural	13-49		1356		
Congo	1987	Maire et al., Rev Epidemiol Sante Publique 40:252-58, 1992	Community	rural	25-45		750		
Congo	1991	Enquête Brazzaville 1991	Community	urban	5-90	5-90	3149	2395	
Congo	1996	Enquête Brazzaville 1996	Community	urban	5-90	5-90	3073	2496	
Congo	2004	STEPS	Community	urban	25-64	25-64	956	1013	
Congo	2005	DHS	National	both	15-49		6266		
Congo	2011-2012	DHS	National	both	15-49		5060		
Cook Islands	2003	STEPS	National	both	25-64	25-64	958	925	
Cook Islands	2011	Global School-based Student Health Survey	National	both	13-17	13-17	543	530	
Cook Islands	2014	STEPS	National	both	18-64	18-64	469	456	
Cook Islands	2015	Global School-based Student Health Survey	National	both	13-17	13-17	313	305	
Costa Rica	2004	CAMDI	Community	urban	20+	20+	624	304	
Costa Rica	2004-2006	Costa Rican Longevity and Healthy Aging Study Pre-1945 Cohort Wave 1	National	both	60+	60+	1350	1163	
Costa Rica	2006-2008	Costa Rican Longevity and Healthy Aging Study Pre-1945 Cohort Wave 2	National	both	62+	62+	1102	944	
Costa Rica	2008-2009	Encuesta Nacional de Nutricion 2008-2009	National	both	45+		661		
Costa Rica	2009	Global School-based Student Health Survey	National	both	13-17	13	1308	356	
Costa Rica	2009-2010	Costa Rican Longevity and Healthy Aging Study Pre-1945 Cohort Wave 3	National	both	64+	64+	890	737	
Costa Rica	2010	Costa Rican National Cardiovascular Risk Factors Survey, 2010	National	both	20+	20+	1959	778	
Costa Rica	2010-2011	Costa Rican Longevity and Healthy Aging Study 1945-1955 Cohort Wave 1	National	both	54-66	54-66	1676	1058	
Costa Rica	2012-2014	Costa Rican Longevity and Healthy Aging Study 1945-1955 Cohort Wave 2	National	both	56-68	56-68	1470	867	
Cote d'Ivoire	1994	DHS	National	both	20-49		2682		
Cote d'Ivoire	1998-1999	DHS	National	both	15-49		2740		
Cote d'Ivoire	2005	STEPS	National	rural	15-64	15-64	1021	895	
Cote d'Ivoire	2005	STEPS	National	urban	15-64	15-64	1435	1071	
Cote d'Ivoire	2011-2012	DHS	National	both	15-49		4601		
Croatia	1997-1999	Budak A et al., Lijec Vjesn 125(1-2):32-5, 2003	National	both	25-100	25-100	2684	1763	
Croatia	2003-2004	School Health Survey	National	both	6-19	6-19	1299	1504	
Croatia	2005	Endemic Nephropathy and Arterial hypertension (ENAH)	Subnational	rural	18+	18+	367	264	
Croatia	2006-2008	The Cardiovascular risk factors in school age – intervention model development	National	both	6-20	6-20	5625	6012	
Croatia	2008	Endemic Nephropathy and Arterial hypertension (ENAH)	Subnational	rural	18+	18+	527	331	
Croatia	2010	Endemic Nephropathy and Arterial hypertension (ENAH)	Subnational	rural	18+	18+	393	252	
Croatia	2014	Croatian Physical Activity in Adolesence Longitudinal Study (CRO-PALS)	Community	urban	15-17	15-17	388	426	
Croatia	2015	Endemic Nephropathy and Arterial hypertension (ENAH) Follow-up Study	Subnational	rural	18+	18+	460	224	
Cuba	1981-1982	Berdasco, Eur J Clin Nutr 1994; 48 Suppl 3:S155-63; discussion S64, 1994	Subnational	both	20-59	20-59	18708	11355	
Cuba	1999-2000	The Survey on Health, Well-Being, and Aging in Latin America and the	Community	urban	60+	60+	1044	630	2
Cuba	2001-2002	National Survey of Risk Factors	National	both	20-60	20-60	11426	11426	
Cuba	2010	National Risk Factor Survey	National	both	15+	15+	3868	3344	
Cuba	2011	Non communicable disease risk factor in Cienfuegos	Community	urban	15-80	15-80	880	617	
Cyprus	1999-2000	Countrywide Integrated Noncommunicable Diseases Intervention Programme Cyprus	National	both	25-65	25-65	546	457	
Cyprus	2007-2008	Asthma Study Cyprus	National	both	15-18	15-18	490	368	
Cyprus	2007-2008	Childhood asthma and atopy in Cyprus	Subnational	both	7-9, 13-15	7-9, 13-15	590	566	
Cyprus	2007-2010	Identification and prevention of Dietary- and lifestyle-induced health EFfects In Children and infantS (IDEFICS)	Community	urban	5-9	5-9	1106	1129	
Czech Republic	1985	MONICA, Czech Republic	National	both	25-64	25-64	1303	1243	
Czech Republic	1988	MONICA, Czech Republic	National	both	25-64	25-64	1408	1357	
Czech Republic	1992	MONICA, Czech Republic	National	both	25-64	25-64	1207	1131	
Czech Republic	1997-1998	Czech post-MONICA	National	both	25-64	25-64	1665	1527	
Czech Republic	2000-2001	Czech post-MONICA	National	both	25-64	25-64	1690	1628	
Czech Republic	2001	6th nationwide anthropological survey of children and adolescents 2001	National	both	5-20	5-19	22523	18960	
Czech Republic	2002-2005	Health, Alcohol and Psychosocial factors In Eastern Europe	Subnational	urban	45-69	45-69	3888	3247	
Czech Republic	2007-2008	Czech post-MONICA	National	both	25-64	25-64	1861	1717	
Czech Republic	2008	Childhood Obesity Surveillance Initiative 1	National	both	6-9	6-9	470	445	10
Czech Republic	2010	Childhood Obesity Surveillance Initiative 2	National	both	6-9	6-9	633	638	10
Czech Republic	2013	Childhood Obesity Surveillance Initiative 3	National	both	6-9	6-9	695	762	10
Denmark	1972	The Danish Conscription Database	National	both		17-26		22101	1
Denmark	1973	The Danish Conscription Database	National	both		17-26		14065	1

Country	Data years	Survey/study name/citation	Level of representative-ness	Rural, urban, or both	Age range as used for global analysis		Sample size		Note
					Female	Male	Female	Male	
Denmark	1974	The Danish Conscription Database	National	both		17-26		14913	1
Denmark	1974-1975	Drivsholm et al., Diabet Med 18:126-32, 2001	Subnational	urban	60	60	306	360	
Denmark	1975	The Danish Conscription Database	National	both		17-26		21422	
Denmark	1975	Copenhagen School Health Records Register	Community	urban	6-13	6-13	13234	13387	
Denmark	1976	The Danish Conscription Database	National	both		17-26		27438	
Denmark	1976	Copenhagen School Health Records Register	Community	urban	6-13	6-13	13425	13506	
Denmark	1977	The Danish Conscription Database	National	both		17-26		25709	
Denmark	1977	Copenhagen School Health Records Register	Community	urban	6-13	6-13	12131	12426	
Denmark	1978	The Danish Conscription Database	National	both		17-26		13578	
Denmark	1978	Copenhagen School Health Records Register	Community	urban	6-13	6-13	12383	12555	
Denmark	1979	The Danish Conscription Database	National	both		17-26		6660	
Denmark	1979	Copenhagen School Health Records Register	Community	urban	6-13	6-13	11624	11659	
Denmark	1980	The Danish Conscription Database	National	both		17-26		2895	
Denmark	1980	Copenhagen School Health Records Register	Community	urban	6-13	6-13	11194	11234	
Denmark	1981	Copenhagen School Health Records Register	Community	urban	6-13	6-13	11349	11433	
Denmark	1981	The Danish Conscription Database	National	both		17-26		1749	
Denmark	1981-1983	Obesity Research Group-Copenhagen City Heart Study 2	Subnational	both		22-62		1140	
Denmark	1982	Copenhagen School Health Records Register	Community	urban	6-13	6-13	10746	10971	
Denmark	1982	The Danish Conscription Database	National	both		17-26		1184	
Denmark	1982-1984	MONICA, Glostrup	Community	urban	30-61	30-61	1844	1940	
Denmark	1983	Copenhagen School Health Records Register	Community	urban	6-13	6-13	7945	7956	
Denmark	1983	The Danish Conscription Database	National	both		17-26		761	
Denmark	1984	Copenhagen School Health Records Register	Community	urban	6-13	6-13	4947	4887	
Denmark	1984	The Danish Conscription Database	National	both		17-26		378	
Denmark	1984-1985	The Epidemiology of Gallstones in a 70 Year-Old Danish Population	Community	both	70	70	172	202	
Denmark	1985	Copenhagen School Health Records Register	Community	urban	6-13	6-13	4275	4266	
Denmark	1985	INTERSALT	Community	urban	20-59	20-59	100	99	
Denmark	1986	Copenhagen School Health Records Register	Community	urban	6-13	6-13	4462	4533	
Denmark	1986-1987	MONICA, Glostrup	Community	urban	29-61	29-61	753	746	
Denmark	1987	Copenhagen School Health Records Register	Community	urban	6-13	6-13	4544	4686	
Denmark	1987	Nilsson et al., J Intern Med 237:479-86, 1995	Community	urban		51		439	
Denmark	1988	Copenhagen School Health Records Register	Community	urban	6-13	6-13	4501	4738	
Denmark	1989	Copenhagen School Health Records Register	Community	urban	6-13	6-13	4598	4846	
Denmark	1990	Copenhagen School Health Records Register	Community	urban	6-13	6-13	4449	4670	
Denmark	1991	Copenhagen School Health Records Register	Community	urban	6-13	6-13	4811	4844	
Denmark	1991-1992	MONICA, Glostrup	Community	urban	29-61	29-61	816	808	
Denmark	1992	Copenhagen School Health Records Register	Community	urban	6-13	6-13	5104	5243	
Denmark	1992-1994	Obesity Research Group-Copenhagen City Heart Study 3	Subnational	both		33-73		922	
Denmark	1993	Copenhagen School Health Records Register	Community	urban	6-13	6-13	4890	4968	
Denmark	1993-1997	EPIC Aarhus	Community	urban	50-65	50-65	8717	8430	
Denmark	1993-1997	EPIC Copenhagen	Community	urban	50-65	50-65	21133	18729	
Denmark	1994	Copenhagen School Health Records Register	Community	urban	6-13	6-13	4005	4065	
Denmark	1995	Copenhagen School Health Records Register	Community	urban	6-13	6-13	5379	5437	
Denmark	1996	Copenhagen School Health Records Register	Community	urban	6-13	6-13	4670	4674	
Denmark	1996-1997	Drivsholm et al., Diabet Med 18:126-32, 2001	Subnational	urban	60	60	370	325	
Denmark	1997	Copenhagen School Health Records Register	Community	urban	7-13	7-13	4025	4105	
Denmark	1997-1998	The European Youth Heart Study	Community	both	8-18	8-18	532	485	
Denmark	1998	Copenhagen School Health Records Register	Community	urban	8-13	8-13	3253	3203	
Denmark	1999	Copenhagen School Health Records Register	Community	urban	9-13	9-13	2777	2860	
Denmark	2000	Copenhagen School Health Records Register	Community	urban	10-13	10-13	1923	1911	
Denmark	2001	Copenhagen School Health Records Register	Community	urban	11-13	11-13	1595	1594	
Denmark	2001-2002	The Copenhagen School Child Intervention Study	Community	urban	5-8	5-8	329	362	
Denmark	2002	Copenhagen School Health Records Register	Community	urban	12-13	12-13	895	860	
Denmark	2002-2003	Odense Androgen Study	Community	urban		20-29		783	
Denmark	2003	Copenhagen School Health Records Register	Community	urban	13	13	369	322	
Denmark	2003-2004	The European Youth Heart Study	Community	both	8-17	8-17	509	392	
Denmark	2004-2005	The Copenhagen School Child Intervention Study	Community	urban	8-11	8-11	130	121	
Denmark	2006	Danish Conscript Register	National	both		17-26		25064	
Denmark	2006-2008	The Health2006 Cohort	Community	urban	18-71	18-71	1916	1553	
Denmark	2007	Danish Conscript Register	National	both		17-26		27194	
Denmark	2007-2008	The Danish Health Examination Survey 2007-2008	National	both	18+	18+	10648	7349	

Country	Data years	Survey/study name/citation	Level of representative-ness	Rural, urban, or both	Age range as used for global analysis		Sample size		Note
					Female	Male	Female	Male	
Denmark	2008	Danish Conscript Register	National	both		17-26		24538	
Denmark	2008	The Copenhagen School Child Intervention Study	Community	urban	12-14	12-14	111	99	
Denmark	2009	Danish Conscript Register	National	both		17-26		27093	
Denmark	2009-2010	The European Youth Heart Study	Community	both	14-28	14-28	553	481	
Denmark	2010	Danish Conscript Register	National	both		17-26		30814	
Denmark	2011	Danish Conscript Register	National	both		17-26		30719	
Denmark	2011-2012	The OPUS School Meal Study	Subnational	both	8-11	8-11	388	427	
Denmark	2012	Danish Conscript Register	National	both		17-26		29651	
Denmark	2012-2015	The Danish study of Functional Disorders (DanFunD)	Subnational	urban	18-72	18-72	4034	3451	
Denmark	2013	Danish Conscript Register	National	both		17-26		30565	
Denmark	2013	LCoMotion	Subnational	both	11-14	11-14	365	353	
Denmark	2014	Danish Conscript Register	National	both		17-26		32397	
Denmark	2015	Danish Conscript Register	National	both		17-26		28907	
Dominica	2007	STEPS	National	both	15-64	15-64	568	460	
Dominica	2009	Global School-based Student Health Survey	National	both	13-17	13-17	542	509	
Dominican Republic	1991	DHS	National	both	20-49		1965		
Dominican Republic	1993	Aono et al., J Epidemiol 7(4):238-43, 1997	National	both	20-70	20-70	1149	767	
Dominican Republic	1996	DHS	National	both	15-49		7441		
Dominican Republic	1996-1998	Estudio De Los Factores De Riesgo Cardiovascular Y Sindrome Metabolico En La Republica Dominicana	National	both	25-85	25-85	2613	1349	
Dominican Republic	2013	DHS	National	both	15-49	15-59	8960	10433	
DR Congo	2001	Multiple Indicator Cluster Survey Round 2	National	both	15-49		5521		
DR Congo	2005	STEPS	Community	urban	15+	15+	1152	761	
DR Congo	2007	DHS	National	both	15-49		4140		
DR Congo	2013-2014	DHS	National	both	15-49		8163		
Ecuador	2003-2004	Garcia et al., Int J Cardiol 110:263-4, 2006	Community	urban		25-64		166	
Ecuador	2004	Ecuador Encuesta Demografica y de Salud Materna e Infantil	National	both	20-50		3533		
Ecuador	2004-2005	CArdiovascular Risk factors Multiple Evaluation in Latin America	Community	urban	25-64	25-64	814	813	
Ecuador	2008-2009	Food Nutrition and Health	Community	both	10-16	10-16	375	379	
Ecuador	2011-2013	Encuesta Nacional de Salud y Nutricion	National	both	5-59	5-59	25771	22922	
Egypt	1992	DHS	National	both	20-49		4654		
Egypt	1995	DHS	National	both	20-49		6499		
Egypt	2000	DHS	National	both	20-49		13602		
Egypt	2002	National Survey of Smoking, Obesity, Blood Pressure and Blood Glucose	National	both	5+	5+	5161	4397	
Egypt	2003	DHS	National	both	20-49		7930		
Egypt	2003-2004	Marzouk et al., Gut 56(8):1105-10, 2007	Community	rural	25+	25+	456	322	
Egypt	2005	STEPS	National	both	15-65	15-65	4430	4760	
Egypt	2005	DHS	National	both	20-49		16864		
Egypt	2007-2009	Mostafa et al., Gut 59(8):1135-40, 2010	Community	rural	35+	35+	843	642	
Egypt	2008	DHS	National	both	20-49	10-59	15243	14266	
Egypt	2011	Global School-based Student Health Survey	National	both	13-17	13-17	454	325	
Egypt	2011	STEPS	National	both	15-65	15-65	2975	1761	
Egypt	2014	DHS	National	both	20-49		18891		
Egypt	2015	DHS	National	both	15-59	15-59	8471	7235	
El Salvador	2002-2003	Ecuesta Nacional de Salud Familiar	National	both	15-49		3885		
El Salvador	2004	CAMDI	Community	urban	20+	20+	811	396	
El Salvador	2008	Ecuesta Nacional de Salud Familiar	National	both	15-49		6809		
Equatorial Guinea	2011	DHS	National	both	20- 49		1074		
Eritrea	1995	DHS	National	both	20-49		1621		
Eritrea	2002	DHS	National	both	20-49		3223		
Eritrea	2004	STEPS	National	both	15-64	15-64	1088	1113	
Eritrea	2010	STEPS	National	both	25-74	25-74	4283	1712	
Estonia	1984-1986	Abina et al., Blood Press 12:111-21, 2003	Community	urban	30-54	20-54	851	2477	
Estonia	1992-1994	Abina et al., Blood Press 12:111-21, 2003	Community	urban	20-54	20-54	678	921	
Estonia	1997	Pomerleau et al., Public Health Nutrition 3: 3-10, 2000	National	both	19-64	19-64	629	525	
Estonia	1999-2001	Abina et al., Blood Press 12:111-21, 2003	Community	urban	20-54	20-54	692	635	
Estonia	2002	Estonian Biobank	National	both	18+	18+	217	89	
Estonia	2003	Estonian Biobank	National	both	18+	18+	5688	2695	
Estonia	2003	The European Male Ageing Study	Community	both		40+		416	
Estonia	2004	Estonian Biobank	National	both	18+	18+	947	527	
Estonia	2007	Estonian Biobank	National	both	18+	18+	2187	1000	
Estonia	2007-2010	Identification and prevention of Dietary- and lifestyle-induced health EFfects In Children and infantS (IDEFICS)	Community	urban	5-9	5-9	634	558	

Country	Data years	Survey/study name/citation	Level of representative-ness	Rural, urban, or both	Age range as used for global analysis		Sample size		Note
					Female	Male	Female	Male	
Estonia	2008	Estonian Biobank	National	both	18+	18+	10990	5147	
Estonia	2008	The European Male Ageing Study	Community	both		40+		305	
Estonia	2009	Estonian Biobank	National	both	18+	18+	6493	3963	
Estonia	2010	Estonian Biobank	National	both	18+	18+	7045	4052	
Estonia	2011	Estonian Biobank	National	both	18+	18+	176	111	
Estonia	2012	Estonian Biobank	National	both	18+	18+	130	85	
Estonia	2013	Estonian Biobank	National	both	18+	18+	143	106	
Ethiopia	2000	DHS	National	both	15-49		13912		
Ethiopia	2005	DHS	National	both	15-49		6134		
Ethiopia	2006	STEPS	Subnational	urban	25-64	25-64	2294	1642	
Ethiopia	2011	DHS	National	both	15-49	15-59	15115	14330	
Fiji	2002	STEPS	National	both	15-64	15-64	3820	2684	
Fiji	2005-2007	Pacific Obesity Prevention in Communities - Ma'alahi Youth Project	Subnational	both	11-19	11-19	4109	3730	
Fiji	2007-2008	Pacific Obesity Prevention in Communities - Ma'alahi Youth Project	Subnational	both	13-22	13-22	1832	1492	
Fiji	2009	Fiji Eye Health Survey 2009	National	both	40+	40+	776	582	
Fiji	2010	Global School-based Student Health Survey	National	both	13-17	13-17	892	630	
Fiji	2011	STEPS	National	both	25-64	25-64	1416	1124	
Fiji	2016	Global School-based Student Health Survey	National	both	13-17	13-17	1469	1394	
Finland	1977	North Karelia project	Subnational	both	25-64	25-64	6223	5857	
Finland	1980	Young Finns Study 1980	National	rural	5-18	5-18	814	752	
Finland	1980	Young Finns Study 1980	National	urban	5-18	5-18	727	704	
Finland	1982	MONICA, North Karelia/Kuopio/Turku/Loimaa	Subnational	both	25-64	25-64	4659	4550	
Finland	1983	Young Finns Study 1983	National	rural	6-21	6-21	773	727	
Finland	1983	Young Finns Study 1983	National	urban	6-21	6-21	685	656	
Finland	1984	Finnish cohort of the FINE study	Community	rural		65-84		673	
Finland	1984-1989	Kuopio Ischaemic Heart Disease Risk factor Study	Subnational	both		42-61		2670	
Finland	1985-1986	INTERSALT, Joensuu	Community	urban	20-59	20-59	100	100	
Finland	1985	INTERSALT, Turku	Community	urban	20-59	20-59	100	100	
Finland	1986	Young Finns Study 1986	National	rural	9-24	9-24	631	594	
Finland	1986	Young Finns Study 1986	National	urban	9-24	9-24	666	587	
Finland	1987	MONICA, North Karelia/Kuopio/Turku/Loimaa	Subnational	both	25-64	25-64	3151	2896	
Finland	1989	Finnish cohort of the FINE study	Community	rural		70-89		446	
Finland	1990-1992	Oulu 35 Study	Community	urban	56-57	56-57	326	231	
Finland	1991-1993	Kuopio Ischaemic Heart Disease Risk factor Study	Subnational	both		46-64		1037	
Finland	1992	The National FINRISK Study	Subnational	both	25-64	25-64	3201	2849	
Finland	1994	Finnish cohort of the FINE study	Community	rural		75-94		266	
Finland	1996-1998	Oulu 35 Study	Community	urban	60-63	60-63	345	242	
Finland	1996-1998	Savitaipale Study, Baseline	Community	rural	40-66	40-66	574	574	
Finland	1997	The National FINRISK Study	National	both	25-74	25-75	4131	4128	
Finland	1998-2001	Kuopio Ischaemic Heart Disease Risk factor Study	Subnational	both	53-73	53-73	920	854	
Finland	2000	Finnish cohort of the FINE study	Community	rural		81-96		92	
Finland	2000-2001	Health 2000 Survey	National	both	30+	30+	3213	2656	
Finland	2001	Young Finns Study 2001	National	rural	24-39	24-39	393	346	
Finland	2001	Young Finns Study 2001	National	urban	24-39	24-39	769	658	
Finland	2001-2004	Helsinki Birth Cohort Study	Community	urban	56-69	56-69	1074	927	
Finland	2002	The National FINRISK Study	National	both	25-74	25-74	3826	3299	
Finland	2004-2005	FIN-D2D	Subnational	both	45-74	45-74	1461	1364	
Finland	2005	Mantyselka et al., Rheumatology (Oxford) 47(8):1235-38, 2008	Community	rural	30-65	30-65	241	230	
Finland	2005-2008	Kuopio Ischaemic Heart Disease Risk factor Study	Subnational	both	60-82	62-82	634	1241	
Finland	2007	The National FINRISK Study	National	both	25-74	25-74	3323	2934	
Finland	2007	Oulu 35 Study	Community	urban	71-73	71-73	271	182	
Finland	2007	Young Finns Study 2007	National	rural	30-45	30-45	431	374	
Finland	2007	Young Finns Study 2007	National	urban	30-45	30-45	714	602	
Finland	2007-2008	Savitaipale Study, Follow-up	Community	rural	51-75	51-75	483	430	
Finland	2008	Control group for Finnish male former elite athletes	National	both		61+		206	
Finland	2011	Young Finns Study 2011	National	rural	34-49	34-49	424	364	
Finland	2011	Young Finns Study 2011	National	urban	34-49	34-49	636	506	
Finland	2011-2012	Health 2011 Survey	National	both	30+	30+	2532	2041	
Finland	2012	The National FINRISK Study	National	both	25-74	25-74	3052	2774	
France	1985-1987	MONICA, Strasbourg	Subnational	both	35-64	35-64	713	664	
France	1985-1987	MONICA, Strasbourg	Community	both	25-34	25-34	78	65	

Country	Data years	Survey/study name/citation	Level of representative-ness	Rural, urban, or both	Age range as used for global analysis		Sample size		Note
					Female	Male	Female	Male	
France	1985-1987	MONICA, Toulouse	Subnational	both	35-64	35-64	644	675	
France	1986-1989	MONICA, Lille	Community	urban	25-64	25-64	732	878	
France	1988-1991	MONICA, Toulouse	Subnational	both		35-64		586	
France	1994-1996	MONICA, Toulouse	Subnational	both	35-64	35-64	566	608	
France	1995-1997	MONICA, Lille	Community	urban	36-67	36-67	590	598	
France	1995-1997	MONICA, Strasbourg	Subnational	both	35-64	35-64	523	526	
France	1996-2003	Jaquet et al., Diabetologia 48(5):849-55, 2005	Community	urban	25-34	25-34	164	173	
France	1999-2001	The Three city Study	Community	urban	65+	65+	3778	2423	
France	2000	Corpulence 7-9 ans	Subnational	both	7-9	7-9	796	786	
France	2004-2006	National Monitoring of Arterial Risk in Lille (MONA LISA Lille)	Subnational	urban	35-75	35-75	795	783	
France	2005-2007	National Monitoring of Arterial Risk in Bas-Rhin (MONA LISA Bas-Rhin)	Subnational	both	35-74	35-74	787	780	
France	2006-2007	Etude Nationale Nutrition Santé	National	both	5-74	5-74	2223	1582	
France	2006-2007	HELENA	Community	urban	12-17	12-17	165	122	
France	2006-2008	The Three city Study	Community	urban	72+	72+	1217	768	
France	2007	Corpulence 7-9 ans	National	both	7-9	7-9	1244	1281	
France	2011-2013	Enquête LIttorale Souffle Air Biologie EnvironnemenT (ELISABET) Dunkerque	Community	urban	40-64	40-64	812	761	
France	2011-2013	Enquête LIttorale Souffle Air Biologie EnvironnemenT (ELISABET) Lille	Community	urban	40-64	40-64.	857	758	
French Polynesia	2010	STEPS	National	both	18-64	18-64	1916	1458	
Gabon	2000	DHS	National	both	20-49		2082		
Gabon	2009	STEPS	Community	urban	15-64	15-64	1515	1051	
Gabon	2012	DHS	National	both	15-49		5066		
Gambia	1996-1997	National Survey of Blindness and Low Vision	National	both	26+	26+	2071	1733	
Gambia	2003	Siervo et al., Eur J Clin Nutr 60(4):455-63, 2006	Community	urban	35-50	35-50	50	50	
Gambia	2010	STEPS	National	both	25-64	25-64	1917	1610	
Gambia	2013	DHS	National	both	15-49		4180		
Georgia	2010	STEPS	National	both	18-64	18-64	4460	1842	
Georgia	2016	STEPS	National	both	18-69	18-69	2784	1188	
Germany	1982	MONICA, Erfurt	Community	urban	25-64	25-64	103	106	
Germany	1982-1984	MONICA, Chemnitz	Community	urban	25-64	25-64	295	267	
Germany	1982-1984	MONICA, Zwickau	Community	urban	25-64	25-64	276	246	
Germany	1982-1985	MONICA, Rest of Karl-Marx-Stadt County	Subnational	urban	25-64	25-64	657	592	
Germany	1982-1985	MONICA, Rest of DDR-MONICA	Subnational	urban	25-64	25-64	232	235	
Germany	1983-1984	MONICA, Halle County	Subnational	urban	25-64	25-64	1172	1110	
Germany	1983-1987	MONICA, Rhein-Neckar Region	Community	urban	35-64	35-64	1609	1489	
Germany	1984	The German Conscription Database	National	both		19		419719	
Germany	1984	German Cardiovascular Prevention Study (GCP) - National Health Survey 1984	Subnational	both	25-69	25-69	2366	2415	
Germany	1984-1985	MONICA, Berlin-Lichtenberg	Community	urban	25-64	25-64	635	593	
Germany	1984	MONICA, Bremen North/West	Community	urban	25-64	25-64	852	813	
Germany	1984-1985	MONICA, Augsburg	Community	both	25-64	25-64	1961	2005	
Germany	1984-1986	MONICA, Cottbus County	Community	urban	25-64	25-64	739	657	
Germany	1985	The German Conscription Database	National	both		19		402487	
Germany	1985-1986	INTERSALT, Cottbus	Community	urban	20-59	20-59	99	99	
Germany	1985-1986	INTERSALT, Heidelberg	Community	urban	20-59	20-59	99	97	
Germany	1985-1986	CINDI	Subnational	both	25-64	25-64	1990	1875	
Germany	1985-1986	INTERSALT, Bernried	Community	urban	20-59	20-59	98	99	
Germany	1986	The German Conscription Database	National	both		19		382632	
Germany	1987	The German Conscription Database	National	both		19		349083	
Germany	1987-1988	MONICA, Erfurt	Community	urban	25-64	25-64	909	871	
Germany	1988	The German Conscription Database	National	both		19		303265	
Germany	1988	German Cardiovascular Prevention Study (GCP) - National Health Survey 1988	Subnational	both	25-69	25-69	2678	2642	
Germany	1988	MONICA, Berlin-Lichtenberg	Community	urban	25-64	25-64	728	690	
Germany	1988	MONICA, Bremen North/West	Community	urban	25-69	25-69	632	619	
Germany	1988	MONICA, Bremen Center/South/East	Community	urban	25-69	25-69	582	499	
Germany	1988	MONICA, Chemnitz	Community	urban	25-64	25-64	382	288	
Germany	1988	MONICA, Zwickau	Community	urban	25-64	25-64	250	193	
Germany	1988-1989	CINDI	Subnational	both	25-64	25-64	1435	1361	
Germany	1988-1989	MONICA, Halle County	Subnational	urban	25-64	25-64	1201	959	
Germany	1988-1989	MONICA, Rest of Karl-Marx-Stadt County	Subnational	urban	25-64	25-64	626	541	
Germany	1989	The German Conscription Database	National	both		19		245740	
Germany	1989-1990	MONICA, Cottbus County	Community	urban	25-64	25-64	529	539	
Germany	1989-1990	MONICA, Augsburg	Community	both	25-64	25-64	1944	1933	

Country	Data years	Survey/study name/citation	Level of representative-ness	Rural, urban, or both	Age range as used for global analysis		Sample size		Note
					Female	Male	Female	Male	
Germany	1990	The German Conscription Database	National	both		19		206599	
Germany	1990-1992	European Community Respiratory Health Survey, Hamburg	Community	urban	20-47	20-47	138	146	
Germany	1990-1992	European Community Respiratory Health Survey, Erfurt	Community	urban	20-47	20-47	124	146	
Germany	1991	The German Conscription Database	National	both		19		138195	
Germany	1991-1992	MONICA, Bremen North/West	Community	urban	25-69	25-69	671	599	
Germany	1991-1992	MONICA, Bremen Center/South/East	Community	urban	25-69	25-69	546	524	
Germany	1991-1992	CINDI	Subnational	both	25-64	25-64	1400	1326	
Germany	1991-1992	German Cardiovascular Prevention Study (GCP) - National Health Survey 1991	Subnational	both	25-69	25-69	2670	2599	
Germany	1991-1992	First National Examination of life conditions, Environment and Health in East Germany 1991/92	Subnational	both	25-69	25-69	1155	1042	
Germany	1991-1992	MONICA, Erfurt	Community	urban	25-64	25-64	572	587	
Germany	1992	The German Conscription Database	National	both		19		220956	
Germany	1993	The German Conscription Database	National	both		19		188655	
Germany	1993-1994	MONICA, Chemnitz	Community	urban	25-64	25-64	424	408	
Germany	1993-1994	MONICA, Zwickau	Community	urban	25-64	25-64	186	139	
Germany	1994	The German Conscription Database	National	both		19		155426	
Germany	1994-1995	MONICA, Augsburg	Community	both	25-64	25-64	1968	1898	
Germany	1994-1998	EPIC Heidelberg	Community	urban	35-64	40-64	13458	11680	
Germany	1994-1998	EPIC Potsdam	Community	urban	35-64	40-64	15995	10224	
Germany	1995	The German Conscription Database	National	both		19		185762	
Germany	1996	The German Conscription Database	National	both		19		191260	
Germany	1997	The German Conscription Database	National	both		19		148738	
Germany	1997-1999	German National Health Interview and Examination Survey	National	both	18-79	18-79	3608	3435	
Germany	1997-2001	Study of Health in Pomerania (SHIP-0) baseline study	Subnational	both	20-80	20-80	2187	2111	
Germany	1998	The German Conscription Database	National	both		19		146528	
Germany	1999	The German Conscription Database	National	both		19		292732	
Germany	1999-2001	KORA S4 Study: Kooperative Research in the Region of Augsburg Survey 4	Community	both	24-75	24-75	2148	2076	
Germany	2000-2001	European Community Respiratory Health Survey, Hamburg	Community	urban	30-57	30-57	138	146	
Germany	2000-2001	European Community Respiratory Health Survey, Erfurt	Community	urban	30-57	30-57	124	146	
Germany	2000-2002	Epidemiological study of the chances of prevention, early recognition and optimal treatment of chronic diseases in an elderly population (ESTHER)	Subnational	both	50-75	50-75	5334	4344	
Germany	2000-2003	Heinz Nixdorf RECALL Study	Community	urban	45-74	45-74	2393	2375	
Germany	2002	Echinoccoccus Multilocularis and Internal Diseases in Leutkirch	Community	urban	12-65	12-65	1261	1171	
Germany	2002-2006	Study of Health in Pomerania (SHIP-1) 5-year follow-up	Subnational	both	25-85	25-85	1707	1583	
Germany	2003-2006	German Health Interview and Examination Survey for Children and Adolescents (KiGGS)	National	both	5-17	5-17	6260	6602	
Germany	2006-2007	HELENA	Community	urban	12-17	12-17	194	282	
Germany	2006-2008	KORA F4 Study: Kooperative Research in the Region of Augsburg Follow-Up of Survey 4	Community	both	31-81	31-81	1583	1480	
Germany	2007-2010	Identification and prevention of Dietary- and lifestyle-induced health EFfects In Children and infantS (IDEFICS)	Community	urban	5-9	5-9	762	772	
Germany	2008	The German Conscription Database	National	both		19		98926	
Germany	2008-2011	German Health Interview and Examination Survey for adults 2008-11 (DEGS1)	National	both	18-79	18-79	3650	3389	
Germany	2008-2011	Epidemiological study of the chances of prevention, early recognition and optimal treatment of chronic diseases in an elderly population (ESTHER)	Subnational	both	58-84	58-84	1622	1468	
Germany	2008-2012	Study of Health in Pomerania, second cohort (SHIP-TREND)	Subnational	both	20-79	20-79	2232	2099	
Germany	2009	The German Conscription Database	National	both		19		111455	
Germany	2010	The German Conscription Database	National	both		19		101911	
Ghana	1993	DHS	National	both	20-49		1650		
Ghana		Amoah et al., Ethn Dis 13(2 Suppl 2):S97-101, 2003	Community	both	25+	25+	2875	1857	
Ghana	1998	DHS	National	both	20-49		1981		
Ghana	2001	Addo et al., Ethn Dis 16(4):894-99, 2006	Community	rural	15+	15+	206	89	
Ghana	2001-2002	Cappuccio et al., Hypertension 43(5):1017-22, 2004	Community	both	35-84	35-84	338	194	
Ghana	2002	Amoah et al., Ethn Dis 13(2 Suppl 2):S97-101, 2003	Community	both	25+	25+	2947	1859	
Ghana	2003	DHS	National	both	15-49		4936		
Ghana	2003	Women's Health Study of Accra	Community	urban	18+		1328		
Ghana	2006	STEPS	Community	urban	25+	25+	1634	841	
Ghana	2007-2008	SAGE	National	both	50+	50+	1987	2192	
Ghana	2008	DHS	National	both	15-49		4458		
Ghana	2012-2014	Research on Obesity and Diabetes among African Migrants (RODAM), control group	Subnational	rural	25+	25+	676	431	
Ghana	2012-2014	Research on Obesity and Diabetes among African Migrants (RODAM), control group	Subnational	urban	25+	25+	1034	418	
Ghana	2014	DHS	National	both	15-49	15-59	4486	4416	
Greece	1991	Seven Countries Study	Subnational	both		70-89		177	
Greece	1991-1999	EPIC	National	both	19-86	19-86	16477	11578	
Greece	1997	The Didima Study	Community	both	18+	18+	373	265	
Greece	2000-2001	Karalis et al., BMC Public Health7:351, 2007	Community	rural	5+	5+	87	73	
Greece	2003	National Epidemiological Survey	National	both	13-19	13-19	7778	6676	

Country	Data years	Survey/study name/citation	Level of representative-ness	Rural, urban, or both	Age range as used for global analysis		Sample size		Note
					Female	Male	Female	Male	
Greece	2005	Daphne	Community	rural	17-18	17-18	57	41	
Greece	2006	Samos	Community	both	5-13	5-13	65	55	
Greece	2006-2007	HELENA, Athens	Community	urban	12-17	12-17	162	158	
Greece	2006-2007	HELENA, Heraklion	Community	urban	12-17	12-17	149	135	
Greece	2007-2009	Healthy Growth Study	Subnational	both	9-13	9-13	1284	1305	
Greece	2008-2009	Greek Childhood Obesity Study (GRECO)	National	both	10-12	10-12	2160	2033	
Greece	2010-2012	ADONUT	National	both	12-19	12-19	18675	18668	
Greece	2010-2011	Childhood Obesity Surveillance Initiative 2	National	both	6-9	6-9	2688	2581	10
Greece	2014	Evaluation of a Web-based Dietary Intervention among primary school children (NUTRI-WEB Children project)	Community	both	8-11	8-11	310	282	
Greece	2016	SKG-Elderly	Community	urban	60+	60+	63	51	
Greenland	2005-2010	Population Health Survey in Greenland	National	both	18+	18+	1714	1336	
Grenada	2011	STEPS	National	both	25-64	25-64	636	438	
Guatemala	1995	DHS	National	both	20-49		4547		
Guatemala	1998-1999	DHS	National	both	20-49		2173		
Guatemala	2001-2002	CAMDI	Community	urban	20+	20+	638	293	
Guatemala	2002	Reproducive Health Survey	National	both	15-49	15-59	7374	2164	
Guatemala	2003-2005	The Institute of Nutrition of Central America and Panama Nutrition Supplementation Trial Cohort	Community	both	25-41	25-41	288	268	
Guatemala	2008-2009	Encuesta Nacional de Salud Materno Infantil	National	both	15-49		15272		
Guatemala	2009	Global School-based Student Health Survey	National	both	13	13	649	489	
Guatemala	2014-2015	DHS	National	both	15-49		24195		
Guatemala	2015	Global School-based Student Health Survey	National	both	13	13	431	413	
Guinea	1999	DHS	National	both	20-49		2986		
Guinea	2005	DHS	National	both	15-49		3574		
Guinea	2009	STEPS	Subnational	both	15-64	15-64	1232	1124	
Guinea	2012	DHS	National	both	15-49		4229		
Guinea Bissau	2010	Multiple Indicator Cluster Survey	National	both	15-49		7678		
Guyana	2009	DHS	National	both	15-49	15-49	4575	3412	
Guyana	2010	Global School-based Student Health Survey	National	both	13-17	13-17	1261	988	
Haiti	1994-1995	DHS	National	both	20-49		1788		
Haiti	2000	DHS	National	both	15-49		9163		
Haiti	2005-2006	DHS	National	both	15-49		5011		
Haiti	2012	DHS	National	both	15-49		8993		
Honduras	1996	Honduras National Micronutrient Survey	National	both	20-40		722		
Honduras	2003-2004	CAMDI	Community	urban	20+	20+	764	428	
Honduras	2005-2006	DHS	National	both	15-49		18125		
Honduras	2011-2012	DHS	National	both	15-49		21097		
Honduras	2012	Global School-based Student Health Survey	National	both	13-17	13-17	728	702	
Hungary	1982-1984	MONICA, Budapest	Community	urban	35-64	35-64	737	774	
Hungary	1982-1983	MONICA, Pecs	Community	urban	35-64	35-64	861	823	
Hungary	1985	INTERSALT	Community	rural	20-59	20-59	100	100	
Hungary	1985-1988	First Hungarian Representative Nutrition Survey	National	both	19+	19+	8916	3079	
Hungary	1987-1988	MONICA, Budapest	Community	urban	35-64	35-64	1594	1413	
Hungary	1987-1988	MONICA, Pecs	Community	urban	35-64	35-64	1510	1573	
Hungary	1992-1994	Nutrition survey of the Hungarian population in a Randomized Trial between 1992-1994	National	both	18-35	18-35	1281	388	
Hungary	2003	The European Male Ageing Study	Community	both		40+		428	
Hungary	2006-2007	HELENA	Community	urban	12-17	12-17	197	197	
Hungary	2007-2010	Identification and prevention of Dietary- and lifestyle-induced health EFfects In Children and infantS (IDEFICS)	Community	urban	5-9	5-9	1169	1128	
Hungary	2008	The European Male Ageing Study	Community	both		40+		349	
Hungary	2010	Childhood Obesity Surveillance Initiative 2	National	both	6-9	6-9	682	553	10
Iceland	1974-1976	The Reykjavik Study (Men)	Subnational	urban		39-70		5533	
Iceland	1977-1979	The Reykjavik Study (Women)	Subnational	urban	42-71		3877		
Iceland	1979-1981	The Reykjavik Study (Men)	Subnational	urban		45-74		3235	
Iceland	1981-1984	The Reykjavik Study (Women)	Subnational	urban	46-75		3567		
Iceland	1983	MONICA, Arnes County	Community	rural	25-64	25-64	450	388	
Iceland	1983	MONICA, Reykjavik	Subnational	urban	25-64	25-64	461	434	
Iceland	1983-1985	The Reykjavik Study for the young	Subnational	urban	29-45	29-45	895	823	
Iceland	1985-1986	INTERSALT	Community	urban	20-59	20-59	100	100	
Iceland	1985-1987	The Reykjavik Study (Men)	Subnational	urban		51-79		2584	
Iceland	1987-1991	The Reykjavik Study (Women)	Subnational	urban	52-82		2993		
Iceland	1988-1989	MONICA, Arnes County	Community	rural	25-64	25-64	435	385	
Iceland	1988-1989	MONICA, Reykjavik	Subnational	urban	25-64	25-64	443	414	

Country	Data years	Survey/study name/citation	Level of representative-ness	Rural, urban, or both	Age range as used for global analysis		Sample size		Note
					Female	Male	Female	Male	
Iceland	1991-1994	The Reykjavik Study (Men)	Subnational	urban		70-86		797	
Iceland	1993-1994	MONICA, Arnes County	Community	rural	25-64	25-64	484	422	
Iceland	1993-1994	MONICA, Reykjavik	Subnational	urban	25-64	25-64	448	441	
Iceland	1994-1996	The Reykjavik Study (Women)	Subnational	urban	69-88		1101		
Iceland	2001-2003	The Reykjavik Study for the young	Subnational	urban	47-62	47-62	705	626	
Iceland	2002-2006	AGES-Reykjavik Study	Subnational	urban	66-96	66-96	3272	2413	
Iceland	2005-2011	Risk Evaluation For INfarct Estimates (REFINE)	Subnational	urban	20-73	20-73	3525	3402	
Iceland	2007-2011	AGES-Reykjavik Study - follow up visit	Subnational	urban	71-98	71-98	1928	1389	
Iceland	2010-2012	Risk Evaluation For INfarct Estimates (REFINE) follow-up visit (REFINELO)	Subnational	urban	26-74	26-74	667	653	
Iceland	2012-2013	Risk Evaluation For INfarct Estimates (REFINE) - follow-up visit (REFLOCT)	Subnational	urban	55-73	55-73	561	516	
India	1975-1979	Diet and Nutritional status of Rural population and Prevalnce of Hypertension	National	rural	5+	5+	30586	43230	
India	1986	INTERSALT	Community	urban	20-59	20-59	99	100	
India	1988-1990	NNMB survey	Subnational	rural	20+	20+	11914	9447	
India	1988-1989	Rmachandran et al., Diabetes Res Clin Pract 58(1):55-60, 2002	Community	urban	20-74	20-74	437	455	
India	1991-1995	Reddy et al., Obes Rev 3(3):197-202, 2002	Community	rural	35-64	35-64	1332	1070	
India	1991-1995	Reddy et al., Obes Rev 3(3):197-202, 2002	Community	urban	35-64	35-64	1594	1456	
India	1991-1994	Prabhakaran et al., Chronic Illn 3(1):8-19, 2007	Community	rural	35-64	35-64	630	542	
India	1991-1994	Prabhakaran et al., Chronic Illn 3(1):8-19, 2007	Community	urban	35-64	35-64	1455	1388	
India	1991-1997	Mumbai Cohort Study	Community	urban	35+	35+	59515	88658	
India	1992-1994	Jaipur Heart Watch 1	Community	rural	20-80	20-80	1147	1946	
India	1992-1994	Jaipur Heart Watch 1	Community	urban	20-80	20-80	782	1385	
India	1993-1994	Khongsdier, Eur J Clin Nutr 56(6):484-89, 2002	Community	both		18-59		575	
India	1995-1996	Epidemiology of blood pressure across cross-cultural populations of Visakhapatnam district, Andhra Pradesh, India	Community	rural	19-76	19-76	228	209	
India	1995	Shobana et al., Diabetes Res Clin Pract 42(3):181–86, 1998	Community	urban	20-74	20-74	1093	1061	
India	1995-1996	Kusuma et al., Ann Hum Biol 29(5):502-12, 2002	Community	both	25-84	25-84	737	747	
India	1995-1997	Aravind Comprehensive Eye Survey	Community	rural	40-90	40-90	2830	2308	
India	1995-1997	Kashmiri Adults	Subnational	both	40+	40+	2587	2496	
India	1996	Diet and Nutritional status of Rural population and Prevalnce of Hypertension	National	rural	5+	5+	27647	22094	
India	1996-1999	Chennai Urban Population Study	Community	urban	20+	20+	705	557	
India	1997	Ramachandran et al., Diabetes Res Clin Pract 44(3):207–13, 1999	Community	rural	20-74	20-74	879	738	
India	1998-1999	DHS	National	both	20-49		72538		
India	1998-2001	Chennai Prospective Study	Community	urban	35+	35+	235968	264848	
India	1999-2001	Jaipur Heart Watch 2	Community	urban	20-75	20-75	569	534	
India	1999-2002	New Delhi Birth Cohort	Community	urban	26-33	26-33	638	886	
India	2000	Ramachandran et al., Diabet Med,20(3):220-24, 2003	Subnational	urban	20-75	20-75	5257	4640	
India	2001	Diet and Nutritional status of Rural population and Prevalnce of Hypertension	National	rural	5+	5+	24845	18048	
India	2001-2004	Chennai Urban Rural Epidemiology Study	Community	urban	20+	20+	1254	1094	
India	2002-2003	Blood Pressure epidemiology in tribal, rural and urban communities of Orissa with special reference to physical and social parameters	Community	rural	18-80	18-80	186	200	
India	2002-2003	JHW-3	Community	urban	20-59	20-59	195	179	
India	2003-2004	ICMR RF/RHD Registry Jai Vigyan Mission Mode Project, Kochi	Subnational	both	5-16	5-16	13515	11327	
India	2003-2005	India STEPS	Subnational	rural	15-69	15-69	1468	1360	
India	2003-2005	India STEPS	Subnational	urban	15-69	15-69	1294	1263	
India	2003-2005	India STEPS	Subnational	rural	15-69	15-69	1338	1372	
India	2003-2005	India STEPS	Subnational	urban	15-69	15-69	1282	1282	
India	2003-2005	India STEPS	Subnational	urban	15-69	15-69	1265	1250	
India	2003-2005	India STEPS	Subnational	rural	15-69	15-69	1410	1460	
India	2003-2005	India STEPS	Subnational	urban	15-69	15-69	1254	1243	
India	2003-2005	India STEPS	Subnational	rural	15-69	15-69	1256	1252	
India	2003-2005	India STEPS	Subnational	urban	15-69	15-69	1261	1252	
India	2003-2005	India STEPS	Subnational	rural	15-69	15-69	1324	1199	
India	2003-2005	India STEPS	Subnational	urban	15-69	15-69	1252	1250	
India	2004-2005	JHW-4	Community	urban	20-59	20-59	473	413	
India	2005-2006	DHS	National	both	15-49	15-54	115828	71464	
India	2005-2006	ICMR RF/RHD Registry Jai Vigyan Mission Mode Project, Kochi	Subnational	both	5-16	5-16	10509	9754	
India	2005-2006	Diet and Nutritional status of Rural population and Prevalnce of Hypertension	National	rural	5+	5+	25327	20495	
India	2005-2007	Prevalence of cardiovascular risk factors in rural Tamil Nadu	Community	rural	25-65	25-65	5573	4927	
India	2006	Ramachandran et al., Diabetes Care 31(5):893-98, 2008	Community	both	20+	20+	3745	3321	
India	2006-2007	Kusuma et al., Asia Pac J Public Health 21(4):497-507, 2009	Community	urban	25-74	25-74	192	182	
India	2006-2008	Central India Eye and Medical Study	Community	rural	30+	30+	2520	2190	
India	2006-2008	Kashmiri Young Adults	Subnational	both	20-40	20-40	905	2119	
India	2006-2009	New Delhi Birth Cohort	Community	urban	33-38	33-38	445	650	

Country	Data years	Survey/study name/citation	Level of representative-ness	Rural, urban, or both	Age range as used for global analysis		Sample size		Note
					Female	Male	Female	Male	
India	2007-2008	Integrated Disease Surveillance Project Non-communicable Disease Risk Factors Survey	Subnational	both	15-64	15-64	3390	2674	
India	2007-2008	Integrated Disease Surveillance Project Non-communicable Disease Risk Factors Survey	Subnational	both	15-64	15-64	2403	1672	
India	2007-2008	Integrated Disease Surveillance Project Non-communicable Disease Risk Factors Survey	Subnational	both	15-64	15-64	2862	2797	
India	2007-2008	Integrated Disease Surveillance Project Non-communicable Disease Risk Factors Survey	Subnational	both	15-64	15-64	2921	3025	
India	2007-2008	Integrated Disease Surveillance Project Non-communicable Disease Risk Factors Survey	Subnational	both	15-64	15-64	2108	2232	
India	2007-2008	Integrated Disease Surveillance Project Non-communicable Disease Risk Factors Survey	Subnational	both	15-64	15-64	2928	2039	
India	2007-2008	Integrated Disease Surveillance Project Non-communicable Disease Risk Factors Survey	Subnational	both	15-64	15-64	3110	2094	
India	2007-2009	Prevalence of NCD risk factor in people above 15 year in Rural area Nagpur using WHO STEP approach	Community	rural	15+	15+	1828	1984	
India	2007-2008	SAGE	National	both	50+	50+	3149	3214	
India	2008-2010	ICMR India Diabetes Study	National	both	20+	20+	6889	6953	
India	2009-2010	Baseline Survey for the assessment of prevalence of risk factors of NCDs in Gandhinagar District	Community	rural	15-64	15-64	711	779	
India	2009-2010	Baseline Survey for the assessment of prevalence of risk factors of NCDs in Gandhinagar District	Community	urban	15-64	15-64	791	785	
India	2011-2012	Diet and Nutritional status of Rural population and Prevalnce of Hypertension	National	rural	5+	5+	43020	34410	
India	2012-2013	Health Survey in Anand School Children	Community	both	5-13	5-13	960	1629	
India	2012-2014	District Level Household and Facility Survey (DLHS) 4	National	both	5+	5+	603032	538812	
India	2014	Annual Health Survey-Chemical, Anthropometric	Subnational	both	5+	5+	681425	659676	
Indonesia	1983-1987	Strickland et al., Eur J Clin Nutr 48 Suppl 3: S98-108; discussion S-9, 1994	Community	both	18+	18+	564	447	
Indonesia	1993-1994	Indonesian Family Life Surveys	National	both	5+	5+	10209	8891	
Indonesia	1997-1998	Indonesian Family Life Surveys	National	both	5+	5+	13896	12157	
Indonesia	2000-2001	Indonesian Family Life Surveys	National	both	5+	5+	16217	15422	
Indonesia	2001	Ng et al., Bull World Health Organ 84(4):305-13, 2006	Community	both	25-74	25-74	1234	1261	
Indonesia	2001	STEPS/SURKESNAS	National	both	25-64	25-64	4775	4100	
Indonesia	2006	Jakarta Non Communicable Disease Risk Factor Surveillance	Community	urban	25-64	25-64	950	641	
Indonesia	2007-2008	Indonesian Family Life Surveys	National	both	5+	5+	19014	17751	
Indonesia	2011	SEANUTS	National	both	5-12	5-12	1380	1363	
Indonesia	2013	Population Health Basic Health Research 2013 (Riskesdas 2013)	National	both	5+	5+	155407	145977	
Indonesia	2014-2015	Indonesian Family Life Surveys	National	both	5+	5+	21543	20330	
Indonesia	2015	Global School-based Student Health Survey	National	both	13-17	13-17	4688	3845	
Iran	1990-1991	National Health Survey I	National	both	5-18	5-18	9038	8883	
Iran	1997-1998	Khadivzadeh, East Mediterr Health J 8(4-5):612-18, 2002	Community	urban	20-49		1513		
Iran	1999-2000	National Health Survey II	National	both	5-99	5-99	26636	23727	
Iran	1999-2001	Tehran Lipid and Glucose Study	Community	urban	5+	5+	7988	6339	
Iran	2000	ASADABADI Study	Community	urban	18+	18+	168	132	
Iran	2001	Isfahan Healthy Heart Program in Students, Arak rural	Community	rural	11-18	11-18	193	145	
Iran	2001	Isfahan Healthy Heart Program in Students, Arak urban	Community	urban	11-18	11-18	306	326	
Iran	2001	Isfahan Healthy Heart Program in Students, Isfahan rural	Community	rural	11-18	11-18	120	89	
Iran	2001	Isfahan Healthy Heart Program in Students, Isfahan urban	Community	urban	11-18	11-18	225	243	
Iran	2001	Isfahan Healthy Heart Program in Students, Najaf Abad rural	Community	rural	11-18	11-18	74	61	
Iran	2001	Isfahan Healthy Heart Program in Students, Najaf Abad urban	Community	urban	11-18	11-18	72	62	
Iran	2001	Isfahan Healthy Heart Program, Arak rural	Community	rural	19+	19+	1080	1023	
Iran	2001	Isfahan Healthy Heart Program, Arak urban	Community	urban	19+	19+	2124	2084	
Iran	2001	Isfahan Healthy Heart Program, Isfahan rural	Community	rural	19+	19+	233	232	
Iran	2001	Isfahan Healthy Heart Program, Isfahan urban	Community	urban	19+	19+	1912	1760	
Iran	2001	Isfahan Healthy Heart Program, Najaf Abad rural	Community	rural	19+	19+	416	405	
Iran	2001	Isfahan Healthy Heart Program, Najaf Abad urban	Community	urban	19+	19+	571	573	
Iran	2002-2005	Tehran Lipid and Glucose Study	Community	urban	5+	5+	3388	2736	
Iran	2003-2004	Childhood and Adolescence Surveillance and Prevention of Adult Noncommunicable Disease (CASPIAN)	National	both	6-18	6-18	10175	10793	
Iran	2003-2004	The Persian Gulf Healthy Heart Study	Subnational	urban	25-75	25-75	1973	1736	
Iran	2004	Hajian-Tilaki et al., Obes Rev 8(1):3-10, 2007	Community	urban	20-70	20-70	1800	1800	
Iran	2004-2008	Golestan Cohort Study Main Phase	Subnational	rural	40-75	40-75	22708	17298	
Iran	2004-2008	Golestan Cohort Study Main Phase	Community	urban	40-75	40-75	6100	3931	
Iran	2005	Dastgiri et al., J Public Health Nutr 9: 996-1000, 2006	Subnational	both	15-70	15-70	167	130	
Iran	2005	STEPS	National	both	15-64	15-64	39748	40722	
Iran	2005-2008	Tehran Lipid and Glucose Study	Community	urban	5+	5+	3808	3104	
Iran	2005-2006	Rashidy-Pour, Obes Rev (1):2-6, 2009	Subnational	both	30-70	30-70	2104	1695	
Iran	2006	STEPS	National	both	16-65	16-65	14617	14885	
Iran	2007	Isfahan Healthy Heart Program in Students, Arak rural	Community	rural	11-18	11-18	164	177	
Iran	2007	Isfahan Healthy Heart Program in Students, Arak urban	Community	urban	11-18	11-18	342	329	
Iran	2007	Isfahan Healthy Heart Program in Students, Isfahan rural	Community	rural	11-18	11-18	19	16	
Iran	2007	Isfahan Healthy Heart Program in Students, Isfahan urban	Community	urban	11-18	11-18	338	398	
Iran	2007	Isfahan Healthy Heart Program in Students, Najaf Abad rural	Community	rural	11-18	11-18	38	38	

Country	Data years	Survey/study name/citation	Level of representative-ness	Rural, urban, or both	Age range as used for global analysis		Sample size		Note
					Female	Male	Female	Male	
Iran	2007	Isfahan Healthy Heart Program in Students, Najaf Abad urban	Community	urban	11-18	11-18	67	43	
Iran	2007	STEPS	National	both	15-64	15-64	2312	2372	
Iran	2007	STEPS	National	both	15-64	15-64	14551	14867	
Iran	2007	Isfahan Healthy Heart Program, Arak rural	Community	rural	19+	19+	1024	1028	
Iran	2007	Isfahan Healthy Heart Program, Arak urban	Community	urban	19+	19+	1359	1424	
Iran	2007	Isfahan Healthy Heart Program, Isfahan rural	Community	rural	19+	19+	151	155	
Iran	2007	Isfahan Healthy Heart Program, Isfahan urban	Community	urban	19+	19+	1302	1309	
Iran	2007	Isfahan Healthy Heart Program, Najaf Abad rural	Community	rural	19+	19+	253	254	
Iran	2007	Isfahan Healthy Heart Program, Najaf Abad urban	Community	urban	19+	19+	542	494	
Iran	2008	STEPS	National	both	15-64	15-64	14353	14757	
Iran	2008-2010	Amol county study	Community	both	10+	10+	2651	3488	
Iran	2008-2010	Tehran city study	Community	urban	10+	10+	540	418	
Iran	2008-2009	Zahedan city study	Community	urban	10+	10+	1205	1377	
Iran	2008-2011	Tehran Lipid and Glucose Study	Community	urban	20+	20+	5884	4622	
Iran	2009	STEPS	National	both	15-64	15-64	14495	14834	
Iran	2009-2010	Childhood and Adolescence Surveillance and Prevention of Adult Noncommunicable Disease (CASPIAN)	National	both	10-18	10-18	2814	2799	
Iran	2009-2010	The Persian Gulf Healthy Heart Study	Subnational	urban	31-79	31-79	1016	834	
Iran	2010-2012	Golestan Cohort Study Second Phase	Subnational	rural	43-82	43-82	4919	4325	
Iran	2010-2012	Golestan Cohort Study Second Phase	Community	urban	43-82	43-82	1061	1091	
Iran	2011	STEPS	National	both	6-69	6-69	6552	4904	
Iran	2011-2012	Childhood and Adolescence Surveillance and Prevention of Adult Noncommunicable Disease (CASPIAN)	National	both	6-18	6-18	6443	6650	
Iran	2012	National Integrated Micronutrient Survey (NIMS) 2012	National	both	6-60	6-60	11088	10530	
Iran	2013-2014	Bushehr Elderly Health Program (BEH)	Community	urban	60+	60+	1514	1437	
Iran	2014-2015	Childhood and Adolescence Surveillance and Prevention of Adult Noncommunicable Disease (CASPIAN)	National	both	7-18	7-18	6970	7169	
Iran	2015	Iranian School Measurement Database	National	both	6-18	6-18	912818	911705	
Iraq	2006	STEPS	National	both	25-64	25-64	2252	2251	
Iraq	2012	Global School-based Student Health Survey	National	both	13	13	199	213	
Iraq	2015	STEPS	National	both	18+	18+	2307	1589	
Ireland	1997-1999	North/South Ireland Food Consumption Survey	National	both	18-64	18-64	698	613	
Ireland	1998	Survey of Lifestye, Attitudes and Nutritional in Ireland 1998	National	both	18+	18+	296	123	
Ireland	2002	Survey of Lifestye, Attitudes and Nutritional in Ireland 2002	National	both	18+	18+	215	164	
Ireland	2003-2004	National Children Food Survey	National	both	5-12	5-12	301	293	
Ireland	2005-2006	National Teens Food Survey	National	both	13-17	13-17	217	223	
Ireland	2006-2007	Survey of Lifestye, Attitudes and Nutritional in Ireland 2006-2007	National	both	18+	18+	1225	945	
Ireland	2008	Childhood Obesity Surveillance Initiative 1	National	both	6-9	6-9	1285	1098	10
Ireland	2008-2010	National Adult Nutrition Survey	National	both	18+	18+	696	658	
Ireland	2009-2011	The Irish Longitudinal Study on Ageing	National	both	50+	50+	3170	2693	
Ireland	2010	Murtagh et al., Pediatr Exerc Sci 25(2):300-7, 2013	Community	both	7-12	7-12	11	20	
Ireland	2010	Murtagh et al., Pediatr Exerc Sci 25(2):300-7, 2013	Community	both	7-12	7-12	19	19	
Ireland	2010	Murtagh et al., Pediatr Exerc Sci 25(2):300-7, 2013	Community	both	7-12	7-12	12	14	
Ireland	2010	Murtagh et al., Pediatr Exerc Sci 25(2):300-7, 2013	Community	both	7-12	7-12	21	16	
Ireland	2010	Childhood Obesity Surveillance Initiative 2	National	both	6-9	6-9	964	1022	10
Ireland	2012-2013	Childhood Obesity Surveillance Initiative 3	National	both	6-9	6-9	1054	1087	10
Ireland	2013-2016	Project Spraoi	Community	both	5-11	5-11	429	474	
Ireland	2015	Active Classrooms Study	Community	both	8-11	8-11	120	124	
Israel	1985-1986	MONICA, Tel Aviv	Community	urban	35-64	35-64	685	653	
Israel	1990-1991	The Jerusalem Longitudinal Cohort Study	Community	urban	69-70	69-70	199	244	
Israel	1997-1998	The Jerusalem Longitudinal Cohort Study	Community	urban	76-77	76-77	425	418	
Israel	1999-2001	Mabat First Israeli National Health and Nutrition Survey	National	both	25-64	25-64	1410	1371	
Israel	1999-2005	The Israel Glucose Intolerance, Obesity and Hypertention Study	National	urban	58-94	58-94	527	514	
Israel	2002-2007	Hadera District Study	Subnational	urban	25-78	25-78	538	548	
Israel	2003-2004	Mabat Youth First Israeli National Health and Nutrition Survey in 7th-12th grade students 2003-4	National	both	12-18	12-18	3073	2553	
Israel	2005-2006	The Jerusalem Longitudinal Cohort Study	Community	urban	83-85	83-85	583	490	
Israel	2005-2006	Mabat Zahav National Health and Nutrition Survey ages 65 and over 2005-6	National	urban	65+	65+	819	743	
Israel	2015-2016	Mabat Youth Second Israeli National Health and Nutrition Survey in 7th-12th grade students 2015-6	National	both	12-18	12-18	2412	2215	
Italy	1980-1982	Po river delta Epidemiological Study - first survey	Community	rural	8-64	8-64	1710	1573	
Italy	1982-1987	MONICA, Latina	Community	both	24-66	24-66	868	852	
Italy	1983-1996	Malattie cardiovascolari ATerosclerotiche Istituto Superiore di Sanità	Community	rural	18-77	18-77	4488	3948	
Italy	1985-1988	Pisa Epidemiological Study - first survey	Community	urban	5-90	5-90	2019	1834	
Italy	1985	Finland, Italy, Netherlands, Elderly (Fine-Italy)	Community	rural		65-84		650	
Italy	1985	INTERSALT, Naples	Community	urban	20-59	20-59	100	100	

Country	Data years	Survey/study name/citation	Level of representative-ness	Rural, urban, or both	Age range as used for global analysis		Sample size		Note
					Female	Male	Female	Male	
Italy	1986	INTERSALT, Bassiano	Community	urban	20-59	20-59	100	99	
Italy	1986	INTERSALT, Gubbio	Community	urban	20-59	20-59	100	99	
Italy	1986	INTERSALT, Mirano	Community	urban	20-59	20-59	100	100	
Italy	1986	MONICA, Friuli	Subnational	urban	25-64	25-64	918	921	
Italy	1986-1987	MONICA, Brianza	Subnational	urban	25-64	25-64	832	814	
Italy	1988-1991	Po river delta Epidemiological Study - second survey	Community	rural	8-73	8-73	1497	1341	
Italy	1989	Ventimiglia Heart Study	Community	rural	6+	6+	700	599	
Italy	1989	MONICA, Friuli	Subnational	urban	25-64	25-64	900	902	
Italy	1989-1990	MONICA, Brianza	Subnational	urban	25-64	25-64	786	787	
Italy	1990	Bruneck Study	Community	rural	40-79	40-79	450	469	
Italy	1991-1993	Pisa Epidemiological Study - second survey	Community	urban	8-97	8-97	1553	1288	
Italy	1992-1993	Italian Longitudinal Study on Aging	National	both	65-84	65-84	1455	1666	
Italy	1992-1998	Vobarno Study	Community	both	35-64	25-64	309	265	
Italy	1993-1994	MONICA, Brianza	Subnational	urban	25-64	25-64	856	801	
Italy	1993-1998	EPIC Florence	Community	urban	24-72	24-72	9968	3498	
Italy	1994	MONICA, Friuli	Subnational	urban	25-64	25-64	888	882	
Italy	1995	Bruneck Study	Community	rural	45-84	45-84	408	411	
Italy	1995-1996	Italian Longitudinal Study on Aging	National	both	69-90	69-90	779	970	
Italy	1995-1999	PROgetto Veneto Anziani	Subnational	both	65+	65+	1722	1187	
Italy	1997-1999	Lucca CUORE Study	Community	urban	25-84	25-84	1123	897	
Italy	1998-2000	InCHIANTI study	Community	both	15+	15+	681	560	
Italy	1998-1999	progetto VIP	Community	both	25-74	25-74	600	599	
Italy	1998-2002	Osservatorio Epidemiologico Cardiovascolare	National	both	35-74	35-74	4752	4870	
Italy	2000-2001	Sorveglianza Nutrizionale Infanzia e Adolescenza (SoNIA)	Subnational	both	8-9	8-9	413	444	
Italy	2000	Bruneck Study	Community	rural	50-89	50-89	361	331	
Italy	2000-2001	Italian Longitudinal Study on Aging	National	both	73-93	73-93	473	557	
Italy	2000-2003	PROgetto Veneto Anziani	Subnational	both	67+	67+	1331	795	
Italy	2001-2003	The Study of Asti	Community	both	45-64	45-64	878	780	
Italy	2002-2005	PROgetto Veneto Anziani	Subnational	both	68+	68+	1138	621	
Italy	2003	Sorveglianza Nutrizionale Infanzia e Adolescenza (SoNIA)	Subnational	both	5-6	5-6	1326	1355	
Italy	2003	The European Male Ageing Study	Community	both		40+		433	
Italy	2004-2005	Italian Project on the Epidemiolofy of Alzheimer's disease	National	both	65-84	65-84	1421	1569	
Italy	2004-2005	Vobarno study	Community	both	55-74	55-74	113	99	
Italy	2004-2008	Cardiolab project	National	urban	40+	40+	14782	19152	
Italy	2005-2007	Moli-family Study	Subnational	both	14-35	14-35	158	128	
Italy	2005	Bruneck Study	Community	rural	55-93	55-93	307	264	
Italy	2005-2010	Moli-sani Study	Subnational	both	35+	35+	12614	11694	
Italy	2006-2007	HELENA	Community	urban	12-17	12-17	185	119	
Italy	2007-2010	Identification and prevention of Dietary- and lifestyle-induced health EFfects In Children and infantS (IDEFICS)	Community	urban	5-9	5-9	847	893	
Italy	2008	Childhood Obesity Surveillance Initiative 1	National	both	6-9	6-9	3896	4101	10
Italy	2008	The European Male Ageing Study	Community	both		40+		346	
Italy	2008-2009	progetto VIP	Community	both	25-74	25-74	596	596	
Italy	2008-2012	Osservatorio Epidemiologico Cardiovascolare/Health Examination Survey	National	both	35-80	35-80	4332	4368	
Italy	2010	Childhood Obesity Surveillance Initiative 2	National	both	6-9	6-9	20195	21476	10
Italy	2010-2012	CArdiovascular risk MEtabolic syndrome LIver and Autoimmunity diseases (CA.ME.LI.A)	Community	both	18-75	18-75	514	477	
Italy	2010	Bruneck Study	Community	rural	60-98	60-98	259	225	
Italy	2011	Grosso et al., Nutrients 5: 4908-23, 2013	Community	rural	13-16	13-16	89	115	
Italy	2011	Grosso et al., Nutrients 5: 4908-23, 2013	Community	urban	13-16	13-16	419	512	
Italy	2011	CONVERGI Study	Community	urban	13-19	13-19	269	159	
Italy	2011-2012	Vobarno study	Community	both	49-62	49-62	143	107	
Italy	2012	Childhood Obesity Surveillance Initiative 3	National	both	6-9	6-9	22407	23140	10
Italy	2012-2014	Mistretta et al., Obesity Research & Clinical Practice 2016, online 3 June 2016	Community	urban	11-16	11-16	751	876	
Italy	2014	OKkio alla SALUTE	National	both	8-9	8-9	22855	24462	
Italy	2014-2016	Mediterranean healthy Eating, Aging and Lifestyles (MEAL) study	Subnational	urban	20+	20+	762	762	
Italy	2015	Bruneck Study	Community	rural	65-98	65-98	169	171	
Italy	2016	OKkio alla SALUTE	National	both	8-9	8-9	21457	22736	
Jamaica	1993	Zohoori et al., West Indian Med J 52(2):111-17, 2003	Community	urban	25-74	25-74	1245	845	
Jamaica	1994-1995	Cooper et al., Am J Public Health 87(2):160-68, 1997	Community	urban	25-100	25-100	833	597	
Jamaica	1998	Ragoobirsingh et al., Diabetes Obes Metab 6(1):23–27, 2004	National	both	25+	25+	945	552	
Jamaica	2000-2001	Jamaica Health and Lifestyle Survey	National	both	15-74	15-74	1281	653	
Jamaica	2005	Jamaican Youth Risk and Resiliency Behaviour Survey 2005	National	both	10-15	10-15	1386	1328	

Country	Data years	Survey/study name/citation	Level of representative-ness	Rural, urban, or both	Age range as used for global analysis		Sample size		Note
					Female	Male	Female	Male	
Jamaica	2006-2007	Jamaica Youth Risk and Resiliency Behaviour Survey 2006	National	both	15-19	15-19	701	585	
Jamaica	2007-2008	Jamaica Health and Lifestyle Survey	National	both	15-74	15-74	1904	862	
Jamaica	2010	Global School-based Student Health Survey	National	both	13-17	13-17	788	748	
Japan	1975	National Nutrition Survey	National	both	5-99	5-99	8542	6562	
Japan	1976	National Nutrition Survey	National	both	5-99	5-99	10022	7890	
Japan	1977	National Nutrition Survey	National	both	5-99	5-99	8094	6455	
Japan	1978	National Nutrition Survey	National	both	5-99	5-99	8695	6979	
Japan	1979	National Nutrition Survey	National	both	5-99	5-99	9031	7249	
Japan	1980	National Nutrition Survey	National	both	5-99	5-99	9437	7955	
Japan	1980-1983	Aito Town Study	Community	rural	20-77	20-77	970	741	
Japan	1981	National Nutrition Survey	National	both	5-99	5-99	7828	5922	
Japan	1982	National Nutrition Survey	National	both	5-99	5-99	8831	6845	
Japan	1983	National Nutrition Survey	National	both	5-99	5-99	8478	6609	
Japan	1984	National Nutrition Survey	National	both	5-99	5-99	8072	6389	
Japan	1985	National Nutrition Survey	National	both	5-99	5-99	8865	7461	
Japan	1985	INTERSALT, Osaka	Community	urban	20-59	20-59	97	100	
Japan	1985	INTERSALT, Tochigi	Community	urban	20-59	20-59	99	95	
Japan	1985	INTERSALT, Toyama	Community	urban	20-59	20-59	100	100	
Japan	1985-1986	Akabane Study	Community	urban	40-69	40-69	1022	812	
Japan	1986	National Nutrition Survey	National	both	5-99	5-99	8635	7280	
Japan	1987	National Nutrition Survey	National	both	5-99	5-99	8156	6424	
Japan	1987	Konan Town Study	Community	rural	20-79	20-79	88	70	
Japan	1988	National Nutrition Survey	National	both	5-99	5-99	8045	6885	
Japan	1988	Konan Town Study	Community	rural	20-79	20-79	85	76	
Japan	1989	National Nutrition Survey	National	both	5-99	5-99	6882	5767	
Japan	1989	Aito Town Study	Community	rural	25-84	25-74	525	529	
Japan	1989	Konan Town Study	Community	rural	20-79	20-79	63	58	
Japan	1990	National Nutrition Survey	National	both	5-99	5-99	7291	6080	
Japan	1990	Konan Town Study	Community	rural	20-79	20-79	51	27	
Japan	1990-1994	Japan Public Health Center-based prospective Study (JPHC Study), Cohort I	Subnational	both	40-59	40-59	14481	8749	
Japan	1991	National Nutrition Survey	National	both	5-99	5-99	7098	6036	
Japan	1991	Konan Town Study	Community	rural	20-79	20-79	116	93	
Japan	1991	Shigaraki Town Study	Community	rural	30-89	30-89	319	230	
Japan	1992	National Nutrition Survey	National	both	5-99	5-99	6656	5635	
Japan	1992	Konan Town Study	Community	rural	20-79	20-79	47	45	
Japan	1992	Shigaraki Town Study	Community	rural	30-89	30-89	385	288	
Japan	1993	Iwata kids health study	Community	both	10	10	485	513	
Japan	1993	National Nutrition Survey	National	both	5-99	5-99	6740	5708	
Japan	1993	Konan Town Study	Community	rural	20-79	20-79	65	54	
Japan	1993	Shigaraki Town Study	Community	rural	30-89	30-89	452	301	
Japan	1993-1994	Japan Public Health Center-based prospective Study (JPHC Study), Cohort II	Subnational	both	40-69	40-69	16190	8534	
Japan	1994	Iwata kids health study	Community	both	10	10	567	569	
Japan	1994	National Nutrition Survey	National	both	5-99	5-99	6386	5439	
Japan		Iki et al., Osteoporos Int 12(7):529:37, 2001	Subnational	both	20-79		3222		
Japan	1994	Konan Town Study	Community	rural	20-79	20-79	59	43	
Japan	1994	Shigaraki Town Study	Community	rural	30-89	30-89	336	251	
Japan	1995	Iwata kids health study	Community	both	10	10	567	524	
Japan	1995	National Nutrition Survey	National	both	5-99	5-99	6365	5480	
Japan	1995	Konan Town Study	Community	rural	20-79	20-79	61	45	
Japan	1995	Shigaraki Town Study	Community	rural	30-89	30-89	470	300	
Japan	1996	Iwata kids health study	Community	both	10	10	480	552	
Japan	1996	National Nutrition Survey	National	both	5-99	5-99	6185	5277	
Japan	1996	Shigaraki Town Study	Community	rural	30-89	30-89	152	86	
Japan	1996-1997	INTERMAP, AitoTown	Community	both	40-59	40-59	129	130	
Japan	1997	Iwata kids health study	Community	both	10	10	537	506	
Japan	1997	National Nutrition Survey	National	both	5-99	5-99	6068	5104	
Japan	1997	Shigaraki Town Study	Community	rural	30-89	30-89	100	61	
Japan	1997-1998	INTERMAP, Sapporo	Community	urban	40-59	40-59	148	149	
Japan	1997-1998	INTERMAP, Toyama	Community	urban	40-59	40-59	150	149	
Japan	1997-1998	INTERMAP, Wakayama	Community	urban	40-59	40-59	144	146	
Japan	1997-2000	Sudo et al., J Orthop Sci 13(5):413-18, 2008	Community	rural	45+	55+	785	261	

Country	Data years	Survey/study name/citation	Level of representative-ness	Rural, urban, or both	Age range as used for global analysis		Sample size		Note
					Female	Male	Female	Male	
Japan	1998	Iwata kids health study	Community	both	10	10	464	527	
Japan	1998	National Nutrition Survey	National	both	5-99	5-99	6249	5381	
Japan	1998	Niigata Study	Community	urban	70	70	284	287	
Japan	1999	Iwata kids health study	Community	both	10	10	463	468	
Japan	1999	National Nutrition Survey	National	both	5-99	5-99	5333	4367	
Japan	1999	Niigata Study	Community	urban	71	71	216	245	
Japan	2000	Iwata kids health study	Community	both	10	10	401	440	
Japan	2000	National Nutrition Survey	National	both	5-99	5-99	5430	4665	
Japan	2000	Niigata Study	Community	urban	72	72	202	233	
Japan	2001	Iwata kids health study	Community	both	10	10	414	452	
Japan	2001	National Nutrition Survey	National	both	5-99	5-99	5448	4527	
Japan	2001	The Japan Association of Health Service Database	Subnational	both	20+	20+	1231378	1471868	
Japan	2001	Niigata Study	Community	urban	73	73	201	235	
Japan	2002	Iwata kids health study	Community	both	10	10	398	496	
Japan	2002	National Nutrition Survey	National	both	5-99	5-99	4941	4104	
Japan	2002	Niigata Study	Community	urban	74	74	202	228	
Japan	2002-2003	The Hisayama Study	Community	rural	40+	40+	1884	1414	
Japan	2003	Iwata kids health study	Community	both	10	10	399	415	
Japan	2003	National Health and Nutrition Survey	National	both	5-99	5-99	4920	4035	
Japan	2003	Niigata Study	Community	urban	75	75	189	215	
Japan	2004	Iwata kids health study	Community	both	10	10	412	463	
Japan	2004	National Health and Nutrition Survey	National	both	5-99	5-99	3952	3384	
Japan	2004	Niigata Study	Community	urban	76	76	185	215	
Japan	2005	Iwata kids health study	Community	both	10	10	420	476	
Japan	2005	National Health and Nutrition Survey	National	both	5-99	5-99	3802	3154	
Japan	2005	Niigata Study	Community	urban	77	77	184	203	
Japan	2006	Iwata kids health study	Community	both	10	10	391	417	
Japan	2006	National Health and Nutrition Survey	National	both	5-99	5-99	4165	3522	
Japan	2006	Niigata Study	Community	urban	78	78	194	199	
Japan	2007	Fukuroi kids health study	Community	both	13-14	13-14	372	395	
Japan	2007	Iwata kids health study	Community	both	10	10	394	439	
Japan	2007	National Health and Nutrition Survey	National	both	5-99	5-99	4154	3520	
Japan	2007	Niigata Study	Community	urban	79	79	192	183	
Japan	2008	Fukuroi kids health study	Community	both	13-14	13-14	346	381	
Japan	2008	Iwata kids health study	Community	both	10	10	417	406	
Japan	2008	MEXT School Health Statistics	National	both	5-17	5-17	326957	326405	
Japan	2008	National Health and Nutrition Survey	National	both	5-99	5-99	4190	3518	
Japan	2008	Resident in Kanazawa City(age 40+)	Community	urban	40+	40+	11944	6562	
Japan	2008	Niigata Study	Community	urban	80	80	180	174	
Japan	2009	Fukuroi kids health study	Community	both	13-14	13-14	357	388	
Japan	2009	MEXT School Health Statistics	National	both	5-17	5-17	327098	326525	
Japan	2009	National Health and Nutrition Survey	National	both	5-99	5-99	4197	3486	
Japan	2010	Fukuroi kids health study	Community	both	13-14	13-14	387	360	
Japan	2010	MEXT School Health Statistics	National	both	5-17	5-17	326401	326509	
Japan	2010	National Health and Nutrition Survey	National	both	6-99	6-99	3822	3218	
Japan	2011	Fukuroi kids health study	Community	both	13-14	13-14	369	402	
Japan	2011	MEXT School Health Statistics	National	both	5-17	5-17	306383	305270	
Japan	2011	National Health and Nutrition Survey	National	both	5-99	5-99	3586	3020	
Japan	2011	The Tokyo Health Service Association Database	Community	urban	20+	20+	54028	82453	
Japan	2012	Fukuroi kids health study	Community	both	13-14	13-14	353	432	
Japan	2012	MEXT School Health Statistics	National	both	5-17	5-17	326572	326524	
Japan	2012	National Health and Nutrition Survey	National	both	5-99	5-99	13674	11298	
Japan	2013	Awaji Child Health Study	Community	both	10-14	10-14	203	198	
Japan	2013	Fukuroi kids health study	Community	both	13-14	13-14	404	387	
Japan	2013	MEXT School Health Statistics	National	both	5-17	5-17	327578	327923	
Japan	2013	National Health and Nutrition Survey	National	both	5-99	5-99	3637	3198	
Japan	2014	Awaji Child Health Study	Community	both	10-14	10-14	218	229	
Japan	2014	MEXT School Health Statistics	National	both	5-17	5-17	326884	327062	
Japan	2014	National Health and Nutrition Survey	National	both	5-99	5-99	3657	3208	
Japan	2014-2015	Nagaoka Health Screening	Community	both	20-89	20-89	4298	4938	
Japan	2015	Awaji Child Health Study	Community	both	10-14	10-14	228	230	

Country	Data years	Survey/study name/citation	Level of representative-ness	Rural, urban, or both	Age range as used for global analysis		Sample size		Note
					Female	Male	Female	Male	
Japan	2015	MEXT School Health Statistics	National	both	5-17	5-17	327210	326382	
Japan	2016	MEXT School Health Statistics	National	both	5-17	5-17	334444	334734	
Jordan	1994-1996	Ajlouni, Int J Obes Relat Metab Disord 22(7), 1998	Subnational	both	25+	25+	1787	1047	
Jordan	1997	DHS	National	both	20-49		3002		
Jordan	2002	DHS	National	both	20-49		4839		
Jordan	2004	Behavioural Risk Factor Surveillence Survey	National	both	18+	18+	473	236	
Jordan	2004	Khader et al., Metab Syndr Relat Disord 6(2):113-20, 2008	Community	both	25-59	25+	548	394	
Jordan	2007	Behavioural Risk Factor Surveillence Survey	National	both	18+	18+	433	332	
Jordan	2007	DHS	National	both	20-49		4451		
Jordan	2009	Metablic abnomalities and vitamin D study	National	both	7+	7+	3863	1601	
Jordan	2009	DHS	National	both	20-49		4054		
Jordan	2012	DHS	National	both	20-49		6357		
Jordan	2016-2017	National Cardiovascular Diseases and Diabetes Study (NCDDS)	National	both	18+	18+	2743	1187	
Kazakhstan		Balakhmetova et l., Ter Arkh 63(1):17-20, 1991	Community	urban		20-54		2886	
Kazakhstan	1995	DHS	National	both	15-49		3542		
Kazakhstan	1999	DHS	National	both	15-49		2227		
Kazakhstan	2015	Almaty STEPS	Subnational	both	18-69	18-69	1145	385	
Kazakhstan	2015	Shymkent STEPS	Subnational	both	18-69	18-69	808	400	
Kazakhstan	2015-2016	Aktobe STEPS	Subnational	both	18-69	18-69	1153	348	
Kenya	1985	INTERSALT	Community	rural	20-59	20-59	86	90	
Kenya	1993	DHS	National	both	20-49		3114		
Kenya	1998	DHS	National	both	20-49		3009		
Kenya	2003	DHS	National	both	15-49		7189		
Kenya	2008-2009	DHS	National	both	15-49		7828		
Kenya	2014	DHS	National	both	15-49		13469		
Kenya	2015	STEPS	National	both	18-69	18-69	2514	1751	
Kiribati	1981	Epidemiological survey of Kiribati	Subnational	rural	20+	20+	531	473	
Kiribati	1981	Epidemiological survey of Kiribati	Subnational	urban	20+	20+	906	939	
Kiribati	2004	STEPS	National	both	15-64	15-64	939	779	
Kiribati	2011	Global School-based Student Health Survey	National	both	13	13	156	122	
Kuwait	1980-1981	al-Isa, Ann Nutr Metab 41(5):307-14, 1997	Community	both		18+		959	
Kuwait	1993-1994	al-Isa, Ann Nutr Metab 41(5):307-14, 1997	Community	both		18+		1730	
Kuwait	1995-1996	Abdella et al., Diabetes Res Clin Pract 42(3):187-96, 1998	Subnational	both	20-84	20-84	1892	1099	
Kuwait	1995-1996	Abdella et al., Diabetes Res and Clin Pract 42(3):187–196, 1998	Subnational	both	20-84	20-84	1892	1099	
Kuwait	1998	Abiaka et al., , Biol Trace Elem Res 91(1):33-43, 2003	National	both	25-80	25-80	233	178	
Kuwait	2001	Kuwait nutrition surveillance system	National	both	5-18	5-18	5255	4378	
Kuwait	2002	Kuwait nutrition surveillance system	National	both	5-18	5-18	5146	5444	
Kuwait	2003	Kuwait nutrition surveillance system	National	both	5-18	5-18	4965	5028	
Kuwait	2004	Kuwait nutrition surveillance system	National	both	5-18	5-18	4792	4900	
Kuwait	2005	Kuwait nutrition surveillance system	National	both	5-18	5-18	5006	4915	
Kuwait	2006	Kuwait nutrition surveillance system	National	both	5-18	5-18	4931	5127	
Kuwait	2006	STEPS	National	both	20-65	20-65	1298	918	
Kuwait	2007	Kuwait nutrition surveillance system	National	both	5-18	5-18	5317	5330	
Kuwait	2008	Kuwait nutrition surveillance system	National	both	5-18	5-18	5425	5620	
Kuwait	2008-2009	National Nutrition Program for the State of Kuwait	National	urban	5+	5+	830	772	
Kuwait	2008-2010	World Health Survey	National	both	18+	18+	1619	1842	
Kuwait	2009	Kuwait nutrition surveillance system	National	both	5-18	5-18	5464	5249	
Kuwait	2011	Global School-based Student Health Survey	National	both	13-17	13-17	1274	1265	
Kuwait	2014	STEPS	National	both	18-69	18-69	2212	1382	
Kuwait	2015	Global School-based Student Health Survey	National	both	13-17	13-17	1553	1364	
Kyrgyzstan	1993	KyrgyzstanMultipurpose Poverty Surveys	National	both	18-60	18-60	2457	2457	
Kyrgyzstan	1997	DHS	National	both	15-49		3570		
Kyrgyzstan	2012	DHS	National	both	15-49		7516		
Kyrgyzstan	2013	STEPS	National	both	25-64	25-64	1600	942	
Lao PDR	2006	Multiple Indicator Cluster Survey 3	National	both	15-49		807		
Lao PDR	2008	STEPS	Community	urban	25-64	25-64	2352	1568	
Lao PDR	2013	STEPS	National	both	18-64	18-64	1461	984	
Latvia	1997	NUTRITION AND LIFESTYLE IN THE BALTIC REPUBLICS, WHO, 1997	National	both	19-50	19-50	732	703	
Latvia	2008	Childhood Obesity Surveillance Initiative 1	National	both	6-9	6-9	1600	1651	10
Latvia	2008-2009	Cardiovascular risk factor study	National	both	25-74	25-74	2398	1362	
Latvia	2010	Childhood Obesity Surveillance Initiative 2	National	both	6-9	6-9	1457	1381	10

Country	Data years	Survey/study name/citation	Level of representative-ness	Rural, urban, or both	Age range as used for global analysis		Sample size		Note
					Female	Male	Female	Male	
Latvia	2012	Childhood Obesity Surveillance Initiative 3	National	both	6-9	6-9	1677	1804	10
Lebanon	1997	Population and Housing Survey	National	both	20+	20+	715	501	
Lebanon	2008-2009	STEPS	National	both	18+	18+	1475	1318	
Lebanon	2011	Global School-based Student Health Survey	National	both	13-17	13-17	665	676	
Lesotho	1993	National survey on iodine, vitamin A and iron status of women and children in Lesotho	National	both	20-65		792		
Lesotho	2004-2005	DHS	National	both	15-49		3207		
Lesotho	2009-2010	DHS	National	both	15-49	15-59	3781	3216	
Lesotho	2012	STEPS	National	both	25-64	25-64	1442	726	
Lesotho	2014	DHS	National	both	15-49	15-59	3247	2863	
Liberia	2006-2007	DHS	National	both	15-49		6420		
Liberia	2011	STEPS	National	both	25-64	25-64	1253	998	
Liberia	2013	DHS	National	both	15-49	15-49	4719	4235	
Libya	1998-1999	Kadiki et al., Diabetes Metab 27(6):647-54, 2001	Community	both	15+	15+	398	228	
Libya	2009	STEPS	National	both	25-64	25-64	1563	1678	
Lithuania	1977-1980	Multifactorial Prevention of Ishaemic Heart Disease, Kaunas	Community	urban		45-59		5691	
Lithuania	1983-1985	MONICA, Kaunas	Community	urban	35-64	35-64	735	728	
Lithuania	1986-1987	MONICA, Kaunas	Community	urban	35-64	35-64	868	894	
Lithuania	1987	Countrywide Integrated Noncommunicable Diseases Intervention Programme survey	Subnational	rural	25-64	25-64	1434	1220	
Lithuania	1992-1993	MONICA, Kaunas	Community	urban	35-64	35-64	621	610	
Lithuania	1992-1993	Countrywide Integrated Noncommunicable Diseases Intervention Programme survey	Subnational	rural	25-64	25-64	798	617	
Lithuania	1997	Pomerleau, 2000	National	both	19+	19+	1130	966	
Lithuania	1998-1999	Countrywide Integrated Noncommunicable Diseases Intervention Programme survey	Subnational	rural	25-64	25-64	1021	816	
Lithuania	2001-2002	MONICA4	Community	urban	35-64	35-64	776	625	
Lithuania	2002	Pomerleau et al., Public Health Nutrition 3: 3-10, 2000	National	both	24-70	24-70	927	977	
Lithuania	2006-2007	Countrywide Integrated Noncommunicable Diseases Intervention Programme survey	Subnational	rural	25-64	25-64	972	718	
Lithuania	2006-2008	Health, Alcohol and Psychosocial factors In Eastern Europe	Community	urban	45-75	45-75	3874	3231	
Lithuania	2008	Childhood Obesity Surveillance Initiative 1	National	both	6-9	6-9	1649	1660	10
Lithuania	2010	Childhood Obesity Surveillance Initiative 2	National	both	6-9	6-9	3414	3307	10
Lithuania	2010-2012	Prevalence and risk factors of high blood pressure in 12-15-year-old Lithuanian children and adolescents (Study 1, 2010-2012)	Community	both	12-15	12-15	3963	3494	
Lithuania	2012-2013	Prevalence and risk factors of high blood pressure in 12-15-year-old Lithuanian children and adolescents (Study 2, 2012-2013)	Community	both	12-15	12-15	985	962	
Lithuania	2013	Childhood Obesity Surveillance Initiative 3	National	both	6-9	6-9	1313	1281	10
Luxembourg	2007-2009	Observation of cardiovascular risk factors in Luxembourg (ORISCAV-LUX)	National	both	18-69	18-69	735	696	
Macedonia (TFYR)	1999	Multiple Indicator Cluster Survey	National	both	15-45		1038		
Macedonia (TFYR)	2009	Annual assessment of nutritional status of school children aged 7-7	National	both	7	7	982	1087	
Macedonia (TFYR)	2010	Childhood Obesity Surveillance Initiative 2	National	both	6-9	6-9	1311	1427	10
Macedonia (TFYR)	2013	Childhood Obesity Surveillance Initiative 3	National	both	6-9	6-9	1071	1139	10
Madagascar	1997	Mauny et al., Ann Trop Med Parasitol 97(6):645-54, 2003	Community	both	15+	15+	283	248	
Madagascar	1997	DHS	National	both	20-49		2253		
Madagascar	2003-2004	DHS	National	both	15-49		7156		
Madagascar	2005	STEPS	Community	urban	25-64	25-64	2490	2596	
Madagascar	2008-2009	DHS	National	both	15-49		7869		
Malawi	1992	DHS	National	both	20-49		2102		
Malawi	1996	Chilima et al., Eur J Clin Nutr 52(9):643-9	Community	rural	55-94	55-94	185	86	
Malawi	2000	DHS	National	both	15-49		11491		
Malawi	2004	DHS	National	both	15-49		9752		
Malawi	2009	Global School-based Student Health Survey	National	both	13	13	382	260	
Malawi	2009	STEPS	National	both	25-64	25-64	3185	1666	
Malawi	2010	DHS	National	both	15-49		7119		
Malawi	2015-2016	DHS	National	both	15-49		7416		
Malaysia	1996	National Health and Morbidity Survey	National	both	30+	30+	10943	9925	
Malaysia	2002-2003	Malaysian Adult Nutrition Survey	National	both	18-59	18-59	3395	3302	
Malaysia	2004	Variation in the prevalence, awareness, and control of diabetes in a multiethnic	National	both	15+	15+	9169	6736	
Malaysia	2005	STEPS	National	both	25-64	25-64	1286	1286	
Malaysia	2006	The third national health and morbidity survey (NHMS III)	National	both	5+	5+	25487	22948	
Malaysia	2008	Metabolic Syndrome Study in Malaysia	National	rural	18+	18+	1368	753	
Malaysia	2008	Metabolic Syndrome Study in Malaysia	National	urban	18+	18+	1446	769	
Malaysia	2010-2011	SEANUTS	National	both	5-12	5-12	1352	1306	
Malaysia	2011	The National Health and Morbidity Survey	National	both	5+	5+	12267	11615	
Malaysia	2012	Global School-based Student Health Survey	National	both	13-17	13	12295	2536	
Malaysia	2012	Malaysian School-Based Health Survey	National	both	9-18	9-18	19660	20251	
Malaysia	2012-2013	Prevalence and factors associated with obesity among secondary schools in Petaling district	Subnational	urban	12-16	12-16	1366	882	

Country	Data years	Survey/study name/citation	Level of representative-ness	Rural, urban, or both	Age range as used for global analysis		Sample size		Note
					Female	Male	Female	Male	
Malaysia	2013-2014	Prevalence of obesity and factors associated among secondary school students in Batang Padang District	Subnational	both	12-17	12-17	3320	2928	
Malaysia	2014	Malaysian Adult Nutrition Survey	National	both	18-59	18-59	1533	1356	
Malaysia	2015	National Health Morbidity Survey 2015	National	both	5+	5+	12875	12146	
Maldives	2001	Multiple Indicator Cluster Survey	National	both	20-49		1145		
Maldives	2004	STEPS	Subnational	urban	25-64	25-64	1086	933	
Maldives	2009	Global School-based Student Health Survey	National	both	13-17	13-17	951	807	
Maldives	2009	DHS	National	both	20-49		5139		
Maldives	2011	STEPS	National	both	15-64	15-64	1059	660	
Maldives	2014	Global School-based Student Health Survey	National	both	13-17	13-17	1324	931	
Mali	1995-1996	DHS	National	both	20-49		3789		
Mali	1997	Torheim et al., Eur J Clin Nutr 58(4):594-604, 2004	Subnational	rural	25-44	25-44	337	237	
Mali	1999	Torheim et al., Public Health Nutr 8(4):387-94, 2005	Subnational	rural	25-44		191		
Mali	2001	DHS	National	both	15-49		10526		
Mali	2006	DHS	National	both	15-49		12512		
Mali	2007	STEPS	Community	both	15-64	15-64	1492	1036	
Mali	2012-2013	DHS	National	both	15-49		4647		
Malta	1984	MONICA, Malta	Community	urban	25-64	25-64	929	948	
Malta	1986	INTERSALT	Community	rural	20-59	20-59	100	100	
Malta	2008	Childhood Obesity Surveillance Initiative 1	National	both	6-9	6-9	1031	1084	10
Malta	2010	Childhood Obesity Surveillance Initiative 2	National	both	6-9	6-9	1170	1151	10
Malta	2013	Childhood Obesity Surveillance Initiative 3	National	both	6-9	6-9	1041	1022	10
Marshall Islands	2002	STEPS	National	both	15-64	15-64	1194	772	
Mauritania	2000-2001	DHS	National	both	20-49		2635		
Mauritania	2006	STEPS	Community	urban	15-64	15-64	1300	1132	
Mauritius	1987	Mauritius non communicable disease survey	National	both	25-74	25-74	2653	2347	
Mauritius	1992	Mauritius non communicable disease survey	National	both	25-74	25-74	3477	2985	
Mauritius	1998	Mauritius non communicable disease survey	National	both	25-74	25-74	3248	2566	
Mauritius	2009	Mauritius non communicable diseases survey	National	both	19+	19+	3389	2860	
Mauritius	2011	Global School-based Student Health Survey	National	both	13-17	13-17	1043	859	
Mauritius	2011	Global School-based Student Health Survey-Rodrigues	Subnational	both	13-17	13-17	546	425	
Mexico	1988-1989	Encuesta Nacional de Nutricion	National	both	12-49		16618		
Mexico	1992-1993	Encuesta Nacional de Enfermedades Cronicas	National	urban	20-69	20-69	8287	6040	
Mexico	1996	Sanchez-Castillo et al., Eur J Clin Nutr 55(10):833-40, 2001	Community	rural	18+	18+	149	104	
Mexico	1998-1999	Encuesta Nacional de Nutricion	National	both	12-49		17894		
Mexico	1998-2004	Mexico City Prospective Study	Community	urban	35-84	35-84	105313	51768	
Mexico	1999	National Survey on School Children	National	both	5-10	5-10	5034	4900	
Mexico	1999-2000	The Survey on Health, Well-Being, and Aging in Latin America and the	Community	urban	60+	60+	548	359	2
Mexico	2000	Encuesta Nacional de Salud	National	both	5+	5+	39233	22570	
Mexico	2001	The Mexican Health and Aging Study	National	both	50+	50+	1224	1031	
Mexico	2002	Encuesta Nacional Sobre Niveles de vida de los Hogares	National	both	5+	5+	13618	11603	
Mexico	2003	The Mexican Health and Aging Study	National	both	50+	50+	1162	893	
Mexico	2004-2005	CArdiovascular Risk factors Multiple Evaluation in Latin America	Community	urban	25-64	25-64	894	833	
Mexico	2005	Encuesta Nacional Sobre Niveles de vida de los Hogares	National	both	5+	5+	13162	11667	
Mexico	2006	Encuesta Nacional de Salud y Nutrición	National	both	5+	5+	34729	27849	
Mexico	2006	PREVENIMSS National Coverage Surveys	National	both	20+	20+	11315	8715	
Mexico	2009-2012	Encuesta Nacional Sobre Niveles de vida de los Hogares	National	both	5+	5+	4691	4912	
Mexico	2009-2010	SAGE	National	both	50+	50+	1236	796	
Mexico	2010	PREVENIMSS National Coverage Surveys	National	urban	20+	20+	6003	6238	
Mexico	2011-2012	Encuesta Nacional de Salud Y Nutricion	National	both	5+	5+	36894	31297	
Mexico	2012	The Mexican Health and Aging Study	National	both	50+	50+	1106	786	
Mexico	2016	Encuesta Nacional de Salud Y Nutricion	National	both	5+	5+	8429	5659	
Micronesia (Federated States of)	2002	STEPS	Subnational	both	25-64	25-64	892	591	
Micronesia (Federated States of)	2006	STEPS	Subnational	both	25-64	25-64	1160	628	
Micronesia (Federated States of)	2008	STEPS	Subnational	both	25-64	25-64	1264	876	
Micronesia (Federated States of)	2009	STEPS, Yap	Subnational	both	15-64	15-64	521	405	
Micronesia (Federated States of)	2009	STEPS, Kosrae	Subnational	both	15-64	15-64	411	208	
Moldova	2005	DHS	National	both	15-49		7076		
Moldova	2013	Childhood Obesity Surveillance Initiative 3	National	both	6-9	6-9	1264	1310	10
Moldova	2013	STEPS	National	both	18-69	18-69	2776	1712	
Mongolia	1999	National Nutrition Survey	National	both	35-65	35-65	1317	907	
Mongolia	2004	National Nutrition Survey	National	both	20-74	20-74	360	248	

Country	Data years	Survey/study name/citation	Level of representative-ness	Rural, urban, or both	Age range as used for global analysis		Sample size		Note
					Female	Male	Female	Male	
Mongolia	2005	STEPS	National	both	15-64	15-64	1717	1669	
Mongolia	2009	STEPS	National	both	15-64	15-64	3117	2197	
Mongolia	2013	Global School-based Student Health Survey	National	both	13-17	13-17	2360	2095	
Mongolia	2013	STEPS	National	both	15-64	15-64	3167	2698	
Montenegro	2016	Anthropometric parameters as an indicator of obesity at adolescents in Montenegrc	National	both	14-18	14-18	771	678	
Morocco	1992	DHS	National	both	20-49		2806		
Morocco	2000	National Survey 2000	National	both	20+	20+	1047	755	
Morocco	2003-2004	DHS	National	both	15-49		15944		
Morocco	2010	Global School-based Student Health Survey	National	both	13	13	335	281	
Morocco	2016	Global School-based Student Health Survey	National	both	13	13	485	475	
Mozambique	1997	DHS	National	both	20-49		2824		
Mozambique	2000	Growth of adolescents in Mozambique	Community	both	9-17	9-17	727	690	
Mozambique	2003	DHS	National	both	15-49		10535		
Mozambique	2005	STEPS	National	both	25-64	25-64	1684	1276	
Mozambique	2011	DHS	National	both	15-49		12201		
Mozambique	2014-2015	STEPS	National	both	15-64	15-64	1684	1147	
Myanmar	2003-2004	STEPS	Subnational	both	25-74	25-74	2449	1990	
Myanmar	2007	Global School-based Student Health Survey	National	both	13	13	294	231	
Myanmar	2009	STEPS	National	both	15-64	15-64	4419	2826	
Myanmar	2011	Underweight prevalence among young adults from rural areas, Salin Township, Magwe Region	Community	rural	15-35	15-35	233	156	
Myanmar	2013-2014	STEPS	Subnational	both	25-74	25-74	740	745	
Myanmar	2015-2016	DHS	National	both	15-49		12163		
Myanmar	2016	Global School-based Student Health Survey	National	both	13	13	283	234	
Namibia	1992	DHS	National	both	20-49		2062		
Namibia	2005	STEPS	National	both	24-64	24-64	1776	1390	
Namibia	2006-2007	DHS	National	both	15-49		8971		
Namibia	2009	Okambilimbili Survey	Community	urban	5+	5+	1167	962	
Namibia	2013	DHS	National	both	15-64		5111		
Namibia	2013	Global School-based Student Health Survey	National	both	13	13	305	168	
Nauru	1975-1976	Trends in the prevalence and incidence of non-insulin-dependent diabetes mellitus and impaired glucose tolerance	Subnational	both	10+	10+	322	297	
Nauru	1982	Trends in the prevalence and incidence of non-insulin-dependent diabetes mellitus and impaired glucose tolerance	National	both	20+	20+	773	701	
Nauru	1987	Trends in the prevalence and incidence of non-insulin-dependent diabetes mellitus and impaired glucose tolerance	National	both	20+	20+	667	555	
Nauru	1994	Trends in the prevalence and incidence of non-insulin-dependent diabetes mellitus and impaired glucose tolerance	National	both	25+	25+	731	647	
Nauru	2004	STEPS	National	both	15-64	15-64	1149	1082	
Nauru	2006	STEPS	National	both	16-65	16-65	236	255	
Nauru	2011	Global School-based Student Health Survey	National	both	13-17	13-17	259	189	
Nepal	1996	DHS	National	both	20-49		3068		
Nepal	1997	Ohno et al., Asia Pac J Public Health 18(3):20-9, 2006	Community	rural	20-75	20-75	41	36	
Nepal	2001	DHS	National	both	20-49		7216		
Nepal	2003	STEPS	Subnational	both	25-64	25-64	996	1010	
Nepal	2005	STEPS	Subnational	both	15-64	15-64	3994	3634	
Nepal	2006	DHS	National	both	15-49		10117		
Nepal	2006-2011	Early detection and management of Kidney disease, Hypertension, Diabetes and Cardiovascular disease (KHDC Nepal), Tarahara	Community	rural	18+	18+	2350	1175	
Nepal	2006-2011	Early detection and management of Kidney disease, Hypertension, Diabetes and Cardiovascular disease (KHDC Nepal), Damal	Community	urban	18+	18+	1577	1095	
Nepal	2006-2011	Early detection and management of Kidney disease, Hypertension, Diabetes and Cardiovascular disease (KHDC Nepal), Dharan	Community	urban	18+	18+	6126	4130	
Nepal	2007-2008	STEPS	National	both	15-64	15-64	2347	1889	
Nepal	2011	DHS	National	both	15-49		5848		
Nepal	2013	STEPS	National	both	15-69	15-69	2763	1326	
Nepal	2015	Global School-based Student Health Survey	National	both	13	13	569	467	
Netherlands	1985	INTERSALT	Community	urban	20-59	20-59	99	100	
Netherlands	1985	Zutphen Elderly Study	Community	urban		65+		886	
Netherlands	1989-1993	The Rotterdam Study, first subcohort	Community	urban	55+	55+	4103	2807	
Netherlands	1990	Zutphen Elderly Study	Community	urban		69-90		552	
Netherlands	1992-1993	The Longitudinal Aging Study Amsterdam (LASA)	Subnational	both	55-85	55-85	1308	1266	4
Netherlands	1993-1995	The Rotterdam Study, first subcohort	Community	urban	56+	56+	3105	2214	
Netherlands	1993-1997	EPIC Bilthoven	Community	urban	20-59	20-59	12021	9941	
Netherlands	1993-1997	EPIC Utrecht	Community	both	49-70		17335		
Netherlands	1995-1996	The Longitudinal Aging Study Amsterdam (LASA)	Subnational	both	58-88	58-88	765	716	4
Netherlands	1997-1999	The Rotterdam Study, first subcohort	Community	urban	61+	61+	2361	1718	
Netherlands	1998-2001	Regenboog Project	National	both	12-89	12-89	2643	2714	
Netherlands	1998-1999	The Longitudinal Aging Study Amsterdam (LASA)	Subnational	both	61-91	61-91	743	604	4

Country	Data years	Survey/study name/citation	Level of representative-ness	Rural, urban, or both	Age range as used for global analysis		Sample size		Note
					Female	Male	Female	Male	
Netherlands	2000-2001	The Rotterdam Study, second subcohort	Community	urban	55+	55+	1468	1210	
Netherlands	2001-2002	The Longitudinal Aging Study Amsterdam (LASA)	Subnational	both	64-94	64-94	690	577	4
Netherlands	2001-2003	Surinamese in the Netherlands: Study on Ethnicity and Health (SUNSET)	Community	urban	35-60	35-60	257	251	
Netherlands	2002-2003	The Longitudinal Aging Study Amsterdam (LASA)	Subnational	both	54-65	54-65	482	431	4
Netherlands	2002-2004	The Rotterdam Study, first subcohort	Community	urban	64+	64+	1708	1206	
Netherlands	2003-2007	Doetinchem Cohort Study (4th measurement)	Subnational	urban	36-74	36-74	2352	2125	
Netherlands	2004-2006	Prevention and Incidence of Asthma and Mite Allergy (PIAMA)	National	both	7-9	7-9	1104	1110	
Netherlands	2004-2005	The Rotterdam Study, second subcohort	Community	urban	58+	58+	1244	964	
Netherlands	2005-2006	The Longitudinal Aging Study Amsterdam (LASA)	Subnational	both	57-97	57-97	958	789	4
Netherlands	2006-2008	The Rotterdam Study, third subcohort	Community	urban	45+	45+	2029	1547	
Netherlands	2008-2011	Prevention and Incidence of Asthma and Mite Allergy (PIAMA)	National	both	12-13	12-13	769	739	
Netherlands	2008-2009	The Longitudinal Aging Study Amsterdam (LASA)	Subnational	both	60-100	60-100	789	642	4
Netherlands	2009-2010	Measuring the Netherlands (NL de Maat)	Subnational	both	30-70	30-70	2014	1781	
Netherlands	2009-2011	The Rotterdam Study, first subcohort	Community	urban	72+	72+	1006	690	
Netherlands	2011-2015	Healthy Life in an Urban Setting (HELIUS)	Community	urban	18-71	18-71	2473	2088	
Netherlands	2011-2012	The Rotterdam Study, second subcohort	Community	urban	65+	65+	934	735	
Netherlands	2011-2012	The Longitudinal Aging Study Amsterdam (LASA)	Subnational	both	63-104	63-104	653	532	4
Netherlands	2012-2014	Prevention and Incidence of Asthma and Mite Allergy (PIAMA)	National	both	15-17	15-17	414	386	
Netherlands	2012-2014	The Rotterdam Study, third subcohort	Community	urban	52+	52+	1639	1256	
New Zealand	1982	MONICA, Auckland	Community	urban	35-64	35-64	568	1019	
New Zealand	1989	The Life in New Zealand Survey	National	both	15+	15+	1571	1418	
New Zealand	1990-1993	Williams, N Z Med J 113(1114):308-11, 2000	Community	both	18-21	18-21	859	932	
New Zealand	1993-1994	MONICA, Auckland	Community	urban	35-64	35-64	674	723	
New Zealand	1996-1997	National Nutrition Survey	National	both	15+	15+	2522	1857	
New Zealand	2002	National Children's Nutrition Survey	National	both	5-15	5-15	1485	1564	
New Zealand	2002-2003	New Zealand Health Survey	National	both	15+	15+	6729	4594	
New Zealand	2006-2007	New Zealand Health Survey	National	both	5+	5+	8032	6766	
New Zealand	2008-2009	New Zealand Adult Nutrition Survey	National	both	15+	15+	2500	2003	
New Zealand	2011-2012	New Zealand Health Survey	National	both	5+	5+	7220	5783	
New Zealand	2012-2013	New Zealand Health Survey	National	both	5+	5+	7926	6409	
New Zealand	2013-2014	New Zealand Health Survey	National	both	5+	5+	8310	6971	
New Zealand	2014-2015	New Zealand Health Survey	National	both	5+	5+	8437	7128	
New Zealand	2015-2016	New Zealand Health Survey	National	both	5+	5+	8451	7258	
Nicaragua	1997-1998	DHS	National	both	15-49		12260		
Nicaragua	2001	DHS	National	both	15-49		11940		
Nicaragua	2003-2004	CAMDI	Community	urban	20+	20+	916	773	
Nicaragua	2003-2005	Sistema Integrado de Vigilancia de Intervenciones Nutricionales (SIVIN)	National	both	20-50		1115		
Nicaragua	2006-2007	Encuesta Nicaraguense de Demografia y Salud	National	both	15-49		13216		
Niger	1992	DHS	National	both	20-49		2993		
Niger	1998	DHS	National	both	20-49		2958		
Niger	2006	DHS	National	both	15-49		4151		
Niger	2007	STEPS	National	both	15-64	15-64	1215	1430	
Niger	2012	DHS	National	both	15-49		4431		
Nigeria	1990	Non-communicable diseases National Survey	National	rural	25+	25+	3682	3619	
Nigeria	1990	Non-communicable diseases National Survey	National	urban	25+	25+	1645	1617	
Nigeria	1991-1994	Cooper et al., Am J Public Health 87(2):160-68, 1997	Community	both	20-100	20-100	1080	910	
Nigeria	1999	DHS	National	both	20-49		2007		
Nigeria	1999-2009	Prostate cancer dietary risk factors study	Subnational	both		35+		627	
Nigeria	2003	DHS	National	both	15-49		6607		
Nigeria	2006	Senbanjo et al., West Afr J Med 30(6):425-31, 2011	Community	urban	5-19	5-19	274	296	
Nigeria	2007	Ibadan Study of Ageing	Subnational	both	60+	60+	914	642	
Nigeria	2008	DHS	National	both	15-49		28978		
Nigeria	2008	Ibadan Study of Ageing	Subnational	both	61+	61+	656	453	
Nigeria	2009	Community Health Plan - Kwara Central Survey	Community	rural	5+	5+	2350	2263	
Nigeria	2009	Ibadan Study of Ageing	Subnational	both	62+	62+	619	420	
Nigeria	2011	Community Health Plan - Kwara Central Survey	Community	rural	5+	5+	856	793	
Nigeria	2013	DHS	National	both	15-49		33946		
Nigeria	2013	Community Health Plan - Kwara Central Survey	Community	rural	5+	5+	754	714	
Niue	2010	Global School-based Student Health Survey	National	both	13-17	13-17	38	63	
Niue	2011	STEPS	National	both	15-100	15-100	478	407	
Norway	1972	Waaler. Acta Med Scand 215(S679):1-56, 1984.	National	both	7-89	7-89	76360	71964	1

Country	Data years	Survey/study name/citation	Level of representative-ness	Rural, urban, or both	Age range as used for global analysis		Sample size		Note
					Female	Male	Female	Male	
Norway	1973	Waaler. Acta Med Scand 215(S679):1-56, 1984.	National	both	7-89	7-89	45410	41607	1
Norway	1974	Waaler. Acta Med Scand 215(S679):1-56, 1984.	National	both	7-89	7-89	36580	34827	1
Norway	1975	Waaler. Acta Med Scand 215(S679):1-56, 1984.	National	both	7-89	7-89	32300	30022	
Norway	1979-1980	Jacobsen et al., Scand J Soc Med 16(2):101-4, 1988	Community	urban	20-49	20-54	7820	8309	
Norway	1979-1980	The Tromsø Study: Tromsø 2	Community	both	20-49	20-54	7823	8312	
Norway	1984-1986	HUNT1 Study	Subnational	rural	20+	20+	37811	36517	
Norway	1986-1987	The Tromsø Study: Tromsø 3	Community	both	20-56	20-61	9804	10374	
Norway	1994-1995	The Tromsø Study: Tromsø 4	Community	both	25+	25+	13835	12782	
Norway	1995-1997	Young-HUNT1 Study	Subnational	rural	12-21	12-21	4253	4203	
Norway	1995-1997	HUNT2 study	Subnational	rural	20+	20+	33599	30285	
Norway	1997-1999	HUSK	Community	urban	47-73	47-73	1670	1635	
Norway	1999-2000	European Youth Heart Study	Community	urban	9-15	9-15	369	364	
Norway	2000-2001	Young-HUNT2 Study	Subnational	rural	16-21	16-21	901	764	
Norway	2000-2003	the Oslo cohort (HUBBRO), the Oppland and Hedmark cohort (OPPHED), and the Troms and Finnmark cohort (TROFINN) of COHORT NORWAY	Subnational	both	30-76	30-76	20592	16825	
Norway	2001-2002	The Tromsø Study: Tromsø 5, Tromsø Study Panel	Community	both	30-89	30-89	3578	2525	
Norway	2005-2006	Physical Activity among Norwegian Children and Adolescents	National	both	8-16	8-16	1055	1186	
Norway	2006-2008	Young-HUNT3 Study	Subnational	rural	12-21	12-21	3792	3807	
Norway	2006-2008	HUNT3 Study	Subnational	rural	20+	20+	27553	22860	
Norway	2007-2008	The Tromsø Study: Tromsø 6	Community	both	30-87	30-87	6885	6048	
Norway	2008	Childhood Obesity Surveillance Initiative 1	National	both	6-9	6-9	1399	1435	10
Norway	2010	Childhood Obesity Surveillance Initiative 2	National	both	6-9	6-9	1286	1335	10
Norway	2012	Childhood Obesity Surveillance Initiative 3	National	both	6-9	6-9	1381	1492	10
Occupied Palestinian Territory	1996-1998	Kobar, rural	Community	rural	15-64	15-64	482	206	
Occupied Palestinian Territory	1996-1998	Old Ramallah, urban	Community	urban	15-64	15-64	493	182	
Occupied Palestinian Territory	1996	Stene et al., Eur J Clin Nutr 55(9):805-11, 2001	Community	rural	30-65	30-65	269	208	
Occupied Palestinian Territory	1999-2000	The First National Health and Nutrition Survey	National	both	18-64	18-64	1869	1736	
Occupied Palestinian Territory	2010	Global School-based Student Health Survey	National	both	13-17	13-17	1865	1823	
Occupied Palestinian Territory	2010	STEPS	National	both	15-64	15-64	3364	1851	
Oman	1991	Oman National Health survey	National	both	20+	20+	2958	2128	
Oman	2000	Oman National Health survey	National	both	20+	20+	3331	3069	
Oman	2001	Al-Lawati et al., Diabetes Care 26(6):1781-85, 2003	Community	urban	20+	20+	756	755	
Oman	2006	STEPS	Community	urban	20-59	20-59	732	540	
Oman	2008	The Oman World Health Survey	National	both	18+	18+	2228	2259	
Oman	2010	Global School-based Student Health Survey	National	both	13-17	13-17	300	251	
Oman	2015	Global School-based Student Health Survey	National	both	13-17	13-17	1551	1330	
Pakistan	1990-1994	MHS	Community	urban	18+	18+	478	432	
Pakistan	1990-1994	National Health Survey Of Pakistan 1990-1994	National	both	5+	5+	7406	7115	
Pakistan	1999	Shah et al., Trop Med Int Health 9(4):526-32, 2004	Community	both	18+	18+	2754	1391	
Pakistan	2004-2005	COBRA-1	Community	urban	40+	40+	1635	1500	
Pakistan	2005	STEPS	National	both	25-65	25-65	1071	787	
Pakistan	2011	National Nutrition Survey	National	both	5-49	5-49	48480	21451	
Pakistan	2012-2013	DHS	National	both	20-49		3980		
Pakistan	2014	STEPS	Subnational	both	18-69	18-69	3677	2966	
Palau	2013	STEPS	National	both	25-64	25-64	1124	1029	
Panama	2003	Second Living Standards Survey	National	both	18-75	18-75	7100	6844	
Panama	2010-2011	Prevalencia de factores de riesgo asociados a enfermedad cardiovascular 2010-2011	Subnational	both	18+	18+	2469	1067	
Papua New Guinea	1985-1986	INTERSALT	Community	rural	20-59	20-59	74	88	
Papua New Guinea	2007	STEPS	National	both	15-64	15-64	1440	1401	
Peru	1991-1992	DHS	National	both	15-49		4889		
Peru	1996	DHS	National	both	20-49		10126		
Peru	2000	DHS	National	both	15-49		25508		
Peru	2003-2007	FRENT	Subnational	urban	15+	15+	2147	1052	
Peru	2004-2006	DHS	National	both	15-49		5798		
Peru	2004-2005	CArdiovascular Risk factors Multiple Evaluation in Latin America	Community	urban	25-64	25-64	876	769	
Peru	2004-2005	Encuesta Nacional de Indicadores Nutricionales, Bioquímicos, Socioeconómicos y Culturales Relacionados con las Enfermedades Crónicas Degenerativas	National	both	20-95	20-95	2094	2087	
Peru	2007-2008	Monitoreo de Indicadores Nutricionales en la ENAHO 2007-2008	National	both	5+	5+	16302	15056	
Peru	2007-2008	DHS	National	both	15-49		20918		
Peru	2007-2008	PERU MIGRANT Study	Community	both	35+	35+	442	405	
Peru	2009	DHS	National	both	15-49		23036		
Peru	2009-2011	Monitoreo de Indicadores Nutricionales en la ENAHO 2009-2010	National	both	5+	5+	31274	27758	
Peru	2009-2012	CRONICAS Cohort Study	Subnational	both	35+	35+	1660	1557	

Country	Data years	Survey/study name/citation	Level of representative-ness	Rural, urban, or both	Age range as used for global analysis		Sample size		Note
					Female	Male	Female	Male	
Peru	2010	DHS	National	both	15-49		22425		
Peru	2010	Global School-based Student Health Survey	National	both	13-17	13-17	1306	1267	
Peru	2011	DHS	National	both	15-49		22215		
Peru	2011-2012	Monitoreo de Indicadores Nutricionales en la ENAHO 2011	National	both	5+	5+	8424	7424	
Peru	2012	DHS	National	both	15-49		23725		
Peru	2013	DHS	National	both	15-49		22676		
Peru	2014	DHS	National	both	15+	15+	28582	12670	
Peru	2015	DHS	National	both	15+	15+	38415	14744	
Philippines	1983-1984	Cebu Longitudinal Health and Nutrition Survey Baseline 2-Month Followup	Community	both	15-50		2866		
Philippines	1983-1984	Cebu Longitudinal Health and Nutrition Survey Baseline 4-Month Followup	Community	both	15-50		2728		
Philippines	1983-1984	Cebu Longitudinal Health and Nutrition Survey Baseline 6-Month Followup	Community	both	15-50		2601		
Philippines	1984-1985	Cebu Longitudinal Health and Nutrition Survey Baseline 8-Month Followup	Community	both	15-50		2473		
Philippines	1984-1985	Cebu Longitudinal Health and Nutrition Survey Baseline 10-Month Followup	Community	both	15-50		2348		
Philippines	1984-1985	Cebu Longitudinal Health and Nutrition Survey Baseline 12-Month Followup	Community	both	15-50		2263		
Philippines	1984-1985	Cebu Longitudinal Health and Nutrition Survey Baseline 14-Month Followup	Community	both	15-50		2193		
Philippines	1984-1985	Cebu Longitudinal Health and Nutrition Survey Baseline 16-Month Followup	Community	both	15-50		2129		
Philippines	1984-1985	Cebu Longitudinal Health and Nutrition Survey Baseline 18-Month Followup	Community	both	15-50		2079		
Philippines	1985-1986	Cebu Longitudinal Health and Nutrition Survey Baseline 20-Month Followup	Community	both	15-50		2047		
Philippines	1985-1986	Cebu Longitudinal Health and Nutrition Survey Baseline 22-Month Followup	Community	both	15-50		2017		
Philippines	1985-1986	Cebu Longitudinal Health and Nutrition Survey Baseline 24-Month Followup	Community	both	15-50		2022		
Philippines	1988	INCLEN	Community	rural		35-65		274	
Philippines	1991-1992	Cebu Longitudinal Health and Nutrition Survey 1991 Child Followup	Community	both	8	8	1076	1202	
Philippines	1991-1992	Cebu Longitudinal Health and Nutrition Survey 1991 Mother Followup	Community	both	22-55		2195		
Philippines	1993	4th National Nutrition Survey Philippine	National	both	20-70	20-70	4754	4383	
Philippines	1993	National Safe Motherhood Survey	National	both	20- 49		7181		
Philippines	1994-1995	Cebu Longitudinal Health and Nutrition Survey 1994-1995 Mother Followup	Community	both	15-59		2692		
Philippines	1998-1999	Cebu Longitudinal Health and Nutrition Survey 1998-1999 Child Followup	Community	both	14-16	14-16	999	1102	
Philippines	1998	5th National Nutrition Survey Philippine	National	both	20-60	20-60	1340	1323	
Philippines	1998-1999	Cebu Longitudinal Health and Nutrition Survey 1998-1999 Mother Followup	Community	both	15-59		1911		
Philippines	2002	Cebu Longitudinal Health and Nutrition Survey 2002 Child Followup	Community	both	17-19	17-19	907	1087	
Philippines	2002	Cebu Longitudinal Health and Nutrition Survey 2002 Mother Followup	Community	both	32-66		2080		
Philippines	2003	Global School-based Student Health Survey	National	both	13	13	291	206	
Philippines	2003-2004	National Nutrition and Health Survey	National	both	25+	25+	33295	30231	
Philippines	2005	Cebu Longitudinal Health and Nutrition Survey 2005 Child Followup	Community	both	20-22	20-22	831	1006	
Philippines	2005	Cebu Longitudinal Health and Nutrition Survey 2005 Mother Followup	Community	both	35-69		2001		
Philippines	2007	Global School-based Student Health Survey	National	both	13	13	254	141	
Philippines	2007	Cebu Longitudinal Health and Nutrition Survey 2007 Child Followup	Community	both	23-24	23-24	751	937	
Philippines	2007	Cebu Longitudinal Health and Nutrition Survey 2007 Mother Followup	Community	both	38-71		1925		
Philippines	2008	National Nutrition Survey	National	both	5+	5+	48652	45778	
Philippines	2009	Cebu Longitudinal Health and Nutrition Survey 2009 Child Followup	Community	both	24-26	24-26	718	864	
Philippines	2011	Global School-based Student Health Survey	National	both	13	13	540	345	
Philippines	2013-2014	8th National Nutrition Survey	National	both	5-18	5-18	21594	22930	
Philippines	2015	Global School-based Student Health Survey	National	both	13-17	13	3730	586	
Poland	1983-1984	MONICA, Tarnobrzeg Voivodship	Community	rural	35-64	35-64	1441	1236	
Poland	1983-1985	MONICA, Warsaw	Community	urban	35-64	35-64	1327	1297	
Poland	1986	Poland Conscripts 10% Sample Cohort	National	both		18-19		29421	
Poland	1986	INTERSALT, Krakow	Community	urban	20-59	20-59	100	100	
Poland	1986	INTERSALT, Warsaw	Community	urban	20-59	20-59	100	100	
Poland	1987-1988	MONICA, Tarnobrzeg Voivodship	Community	rural	35-64	35-64	672	616	
Poland	1988-1989	MONICA, Warsaw	Community	urban	35-64	35-64	713	705	
Poland	1989-1990	Polish Program CINDI (CINDI Lodz 1989-1990)	Community	urban	25-64	25-64	957	831	
Poland	1992-1993	MONICA, Tarnobrzeg Voivodship	Community	rural	35-64	35-64	692	618	
Poland	1993	MONICA, Warsaw	Community	urban	35-64	35-64	763	751	
Poland	1995	Poland Conscripts 10% Sample Cohort	National	both		18-19		31043	
Poland	1995-1996	Polish Program CINDI (CINDI Lodz 1995)	Community	urban	17-64	17-64	1459	997	
Poland	2000	The health status, risk factors of chronic diseases and health behaviors of residents of Torun (CINDI Torun 2000)	Community	urban	16-83	16-83	1054	989	
Poland	2000	Binkowska-Bury et al., Neuro Endocrinol Lett 34(8):814-20, 2013	Subnational	both		19-20		3003	
Poland	2000-2001	Household Food Consumption and Anthropometric Survey	National	both	5+	5+	2107	1766	
Poland	2001	Binkowska-Bury et al., Neuro Endocrinol Lett 34(8):814-20, 2013	Subnational	both		19-20		3420	
Poland	2001	Poland Conscripts 10% Sample Cohort	National	both		18-19		31213	
Poland	2001-2002	The health status, risk factors of chronic diseases and health behaviors of residents of Lodz (CINDI Lodz 2001)	Community	urban	18-64	18-64	840	1000	

Country	Data years	Survey/study name/citation	Level of representative-ness	Rural, urban, or both	Age range as used for global analysis		Sample size		Note
					Female	Male	Female	Male	
Poland	2002	Binkowska-Bury et al., Neuro Endocrinol Lett 34(8):814-20, 2013	Subnational	both		19-20		3544	
Poland	2002	NATPOL	National	both	18+	18+	1301	1018	
Poland	2002	The health status, risk factors of chronic diseases and health behaviors of residents of Lodz - seniors (CINDI Lodz 2002)	Community	urban	65+	65+	532	285	
Poland	2002-2005	Health, Alcohol and Psychosocial factors In Eastern Europe	Community	urban	45-69	45-69	4720	4454	
Poland	2003	Binkowska-Bury et al., Neuro Endocrinol Lett 34(8):814-20, 2013	Subnational	both		19-20		3633	
Poland	2003	The European Male Ageing Study	Community	both		40+		406	
Poland	2003-2005	National Multicenter Health Survey in Poland. Project WOBASZ	National	both	20-74	20-74	6907	6245	
Poland	2003-2013	Mogielica Human Ecology Study	Community	rural	21+	21+	896	353	
Poland	2004	Binkowska-Bury et al., Neuro Endocrinol Lett 34(8):814-20, 2013	Subnational	both		19-20		3538	
Poland	2005	Binkowska-Bury et al., Neuro Endocrinol Lett 34(8):814-20, 2013	Subnational	both		19-20		3308	
Poland	2006	The health, risk factors for chronic diseases, attitudes and behaviors of health residents of Torun (CINDI Torun 2006)	Community	urban	15-65	15-65	1147	790	
Poland	2006	Binkowska-Bury et al., Neuro Endocrinol Lett 34(8):814-20, 2013	Subnational	both		19-20		3701	
Poland	2006-2007	National Multicenter Health Survey in Poland. Project WOBASZ Senior	National	both	75+	75+	533	541	
Poland	2007	Binkowska-Bury et al., Neuro Endocrinol Lett 34(8):814-20, 2013	Subnational	both		19-20		3612	
Poland	2007-2009	Elaboration of the reference range of arterial blood pressure for the population of children and adolescents in Poland – PL0080 OLAF	National	both	6-18	6-18	9162	8383	
Poland	2007-2011	Medical, psychological and socioeconomic aspects of aging in Poland	National	both	55+	55+	2582	2750	
Poland	2008	Binkowska-Bury et al., Neuro Endocrinol Lett 34(8):814-20, 2013	Subnational	both		19-20		3435	
Poland	2008	The European Male Ageing Study	Community	both		40+		310	
Poland	2009	Binkowska-Bury et al., Neuro Endocrinol Lett 34(8):814-20, 2013	Subnational	both		19-20		3405	
Poland	2010	Binkowska-Bury et al., Neuro Endocrinol Lett 34(8):814-20, 2013	Subnational	both		19-20		3317	
Poland	2010-2012	Blood pressure references for Polish preschool children - the OLA study	National	both	5-6	5-6	904	926	
Poland	2011	NATPOL	National	both	18-79	18-79	1235	1158	
Poland	2013-2014	National Multicenter Health Survey in Poland. Project WOBASZ II	National	both	20+	20+	3196	2626	
Poland	2015-2016	LIPIDOGRAM2015 & LIPIDOGEN2015 Study - National epidemiological study of lipid disorders and selected risk factors of cardiovascular disease in primary	National	both	18+	18+	8690	5034	
Portugal	1985	Body Mass Index of Portuguese Conscripts	National	both		18-20		29420	
Portugal	1986	Body Mass Index of Portuguese Conscripts	National	both		18-20		70504	
Portugal	1986	INTERSALT	Community	both	20-59	20-59	99	99	
Portugal	1987	Body Mass Index of Portuguese Conscripts	National	both		18-20		68079	
Portugal	1988	Body Mass Index of Portuguese Conscripts	National	both		18-20		67573	
Portugal	1989	Body Mass Index of Portuguese Conscripts	National	both		18-20		68827	
Portugal	1990	Body Mass Index of Portuguese Conscripts	National	both		18-20		44359	
Portugal	1991	Body Mass Index of Portuguese Conscripts	National	both		18-20		19553	
Portugal	1992	Body Mass Index of Portuguese Conscripts	National	both		18-20		52393	
Portugal	1993	Body Mass Index of Portuguese Conscripts	National	both		18-20		59780	
Portugal	1994	Body Mass Index of Portuguese Conscripts	National	both		18-20		55511	
Portugal	1995	Body Mass Index of Portuguese Conscripts	National	both		18-20		68221	
Portugal	1996	The Portugese Conscription Database	National	both		18-21		106097	
Portugal	1997	The Portugese Conscription Database	National	both		18-21		61215	
Portugal	1998	The Portugese Conscription Database	National	both		18-21		41027	
Portugal	1998	European Youth Heart Study	Community	both	9-10; 15-16	9-10; 15-16	536	556	
Portugal	1999	The Portugese Conscription Database	National	both		18-21		54187	
Portugal	1999-2003	EPIPorto Study	Community	urban	18+	18+	1507	932	
Portugal	2000	The Portugese Conscription Database	National	both		18-21		53326	
Portugal	2003-2004	EPITeen - Epidemiological Health Investigation of Teenagers in Porto	Community	urban	13-14	13-14	1048	981	
Portugal	2003-2005	Estudo de Prevalência da Obesidade e Consumos Alimentares em Portugal	National	both	18-64	18-64	4320	3796	
Portugal	2004	Growth of adolescents in Coimbra	Community	both	9-16	9-16	408	265	
Portugal	2004	Growth of adolescents in Gouveia	Community	rural	10-19	10-19	246	238	
Portugal	2007	European Youth Heart Study	Community	both	8-10; 16-17	8-10; 16-17	311	315	
Portugal	2007	Growth of adolescents in Tondela	Community	rural	6-19	6-19	312	314	
Portugal	2007-2008	EPITeen - Epidemiological Health Investigation of Teenagers in Porto	Community	urban	16-17	16-17	1251	1186	
Portugal	2007-2009	Portuguese National Survey of Physical Activity and Physical Fitness	National	both	9+	9+	18025	14914	
Portugal	2007-2010	The Midland Adolescent Lifestyle Study	Subnational	both	13-16	13-16	203	163	
Portugal	2008	Azorean Physical Activity and Health Study II	Subnational	both	15-18	15-18	893	608	
Portugal	2008	Childhood Obesity Surveillance Initiative 1	National	both	6-9	6-9	904	911	10
Portugal	2008	PESSOA program	Community	both	9-16	9-16	1813	1931	
Portugal	2008-2013	Preschool Physical Activity, Body Composition and Lifestyle Study (PRESTYLE)	Community	urban	5-6	5-6	607	651	
Portugal	2009	Bracara Study	Community	urban	8-14	8-14	336	398	
Portugal	2009-2010	Portuguese Prevelance Study of Obesity in Childhood	National	both	5-10	5-10	7099	6805	
Portugal	2010	Childhood Obesity Surveillance Initiative 2	National	both	6-9	6-9	903	908	10
Portugal	2010-2012	Exercise for Elderly	Community	urban	60-84	60-84	104	48	
Portugal	2011	PESSOA program	Community	both	10-13	10-13	275	287	

Country	Data years	Survey/study name/citation	Level of representative-ness	Rural, urban, or both	Age range as used for global analysis		Sample size		Note
					Female	Male	Female	Male	
Portugal	2011-2012	The association of childhood obesity with asthma and rhinitis symptoms in 6-8years old children living in the Coimbra district, Portugal: the role of	Community	both		5-9		480	
Portugal	2011-2014	Longitudinal Analysis of Biomarkers and Environmental Determinants of Physical activity (LABMED Study)	Subnational	urban	12-18	12-18	471	546	
Portugal	2011-2013	EPITeen - Epidemiological Health Investigation of Teenagers in Portc	Community	urban	20-23	20-23	895	854	
Portugal	2013	Childhood Obesity Surveillance Initiative 3	National	both	6-9	6-9	1313	1336	10
Portugal	2013	Childhood obesity in Lousao	Community	rural	5-14	5-14	418	444	
Portugal	2013-2014	Cultural, social, economic, and environmental factors that can influence children's sport participation and obesity levels	Community	urban	6-10	6-10	408	385	
Puerto Rico	2002-2003	Puerto Rican Elderly: Health Conditions	National	both	60+	60+	2850	1914	5
Puerto Rico	2005-2007	Perez et al., Ethn Dis 18(4):434-41, 2008	Community	urban	25-84	25-84	529	275	
Puerto Rico	2006-2007	Puerto Rican Elderly: Health Conditions	National	both	60+	60+	1669	1056	5
Puerto Rico	2010-2013	HPV Infection in a Population-Based Sample of Puerto Rican Women	Subnational	both	16-64		563		
Qatar	2006	World Health Survey	National	both	18+	18+	2018	1859	
Qatar	2011	Global School-based Student Health Survey	National	both	13	13	126	102	
Qatar	2012	STEPS	National	both	18-64	18-64	1352	1034	
Romania	1986-1987	MONICA, Bucharest	Community	urban	35-64	35-64	873	702	
Romania	1997	Somatometria	National	both	25-75	25-75	4063	3142	
Romania	2006-2008	Hypertension in Romanian Children and Adolescents: A Cross-Sectional Survey	Subnational	both	5-17	5-17	2339	2313	
Romania	2008-2015	Healthy traditions for healthy children	Subnational	both	5-11	5-11	3273	3285	
Romania	2009-2011	Study on children in Dolj County, South Romania	Subnational	both	5-21	5-21	672	746	
Romania	2011-2012	Study for the Evaluation of Prevalence of Hypertension and cArdiovascular Risk among the Adult Population of Romania - SEPHAR I	National	both	18-80	18-80	927	1023	
Romania	2013	Childhood Obesity Surveillance Initiative 3	National	both	6-9	6-9	2173	2175	10
Romania	2013-2014	Auxological evaluation of school children in Mures County	Subnational	both	6-14	6-14	957	936	
Romania	2014	Timis County Study	Community	urban	6-19	6-19	205	238	
Romania	2015-2016	Study for the Evaluation of Prevalence of Hypertension and cArdiovascular Risk among the Adult Population of Romania - SEPHAR II	National	both	18-80	18-80	1034	936	
Russian Federation	1984-1986	MONICA, Moscow (control)	Community	urban	35-64	35-64	642	774	
Russian Federation	1984-1986	MONICA, Moscow, Leninsky district	Community	urban	35-64	35-64	622	553	
Russian Federation	1984-1986	MONICA, Moscow, Cheremushkinsky district	Community	urban	35-64	35-64	579	580	
Russian Federation	1985	MONICA, Novosibirsk (intervention)	Community	urban	25-64	25-64	818	797	
Russian Federation	1985-1986	MONICA, Novosibirsk, Kirowsky district	Community	urban	25-64	25-64	774	758	
Russian Federation	1985-1986	MONICA, Novosibirsk, Leninsky district	Community	urban	25-64	25-64	624	624	
Russian Federation	1986	INTERSALT	Community	urban	20-59	20-59	97	97	
Russian Federation	1988	MONICA, Novosibirsk (intervention)	Community	urban	25-64	25-64	852	837	
Russian Federation	1988-1989	MONICA, Moscow (control)	Community	urban	35-64	35-64	581	620	
Russian Federation	1988-1989	MONICA, Moscow, Leninsky district	Community	urban	35-64	35-64	612	597	
Russian Federation	1988-1989	MONICA, Novosibirsk, Kirowsky district	Community	urban	25-64	25-64	705	871	
Russian Federation	1992-1993	Russia Longitudinal Monitoring Survey-Higher School of Economics Round II	National	both	5+	5+	6348	4764	
Russian Federation	1992	CINDI	Community	rural	25-64	25-64	453	377	
Russian Federation	1992	Puska et al., Int J Epidemiol 22(6):1048-55, 1993	Community	both	25-64	25-64	458	379	
Russian Federation	1992	Russian Karelia Survey in Pitkaranta	Community	both	25-64	25-64	455	380	
Russian Federation	1992-1995	MONICA, Moscow (control)	Community	urban	35-64	35-64	527	556	
Russian Federation	1992-1995	MONICA, Moscow, Leninsky district	Community	urban	35-64	35-64	858	538	
Russian Federation	1993	Russia Longitudinal Monitoring Survey-Higher School of Economics Round III	National	both	5+	5+	7692	6013	
Russian Federation	1993-1994	Russia Longitudinal Monitoring Survey-Higher School of Economics Round IV	National	both	5+	5+	7099	5521	
Russian Federation	1994	Russia Longitudinal Monitoring Survey-Higher School of Economics Round V	National	both	5+	5+	5788	4726	
Russian Federation	1994-1995	MONICA, Novosibirsk (intervention)	Community	urban	25-64	25-64	860	820	
Russian Federation	1995	Russia Longitudinal Monitoring Survey-Higher School of Economics Round VI	National	both	5+	5+	5509	4463	
Russian Federation	1995	MONICA, Novosibirsk, Kirowsky district	Community	urban	25-64	25-64	787	771	
Russian Federation	1996	Russia Longitudinal Monitoring Survey-Higher School of Economics Round VII	National	both	5+	5+	5456	4377	
Russian Federation	1997	Russian Karelia Survey in Pitkaranta	Community	both	25-64	25-64	440	309	
Russian Federation	1998-1999	Russia Longitudinal Monitoring Survey-Higher School of Economics Round VIII	National	both	5+	5+	5590	4498	
Russian Federation	2000	Russia Longitudinal Monitoring Survey-Higher School of Economics Round IX	National	both	5+	5+	5761	4600	
Russian Federation	2001	Russia Longitudinal Monitoring Survey-Higher School of Economics Round X	National	both	5+	5+	6440	5023	
Russian Federation	2002	Russia Longitudinal Monitoring Survey-Higher School of Economics Round XI	National	both	5+	5+	6603	5198	
Russian Federation	2002	Russian Karelia Survey in Pitkaranta	Community	both	25-64	25-64	334	251	
Russian Federation	2002-2005	Health, Alcohol and Psychosocial factors In Eastern Europe	Community	urban	45-69	45-69	5040	4208	
Russian Federation	2003	Russia Longitudinal Monitoring Survey-Higher School of Economics Round XII	National	both	5+	5+	6674	5250	
Russian Federation	2004	Russia Longitudinal Monitoring Survey-Higher School of Economics Round XIII	National	both	5+	5+	6667	5219	
Russian Federation	2005	Russia Longitudinal Monitoring Survey-Higher School of Economics Round XIV	National	both	5+	5+	6452	5025	
Russian Federation	2007	Russian Karelia Survey in Pitkaranta	Community	both	25-64	25-64	276	176	
Russian Federation	2007-2010	SAGE	National	both	50+	50+	2251	1254	
Russian Federation	2015-2016	Ural Eye and Medical Study (UEMS)	Subnational	rural	40+	40+	1372	816	
Russian Federation	2015-2016	Ural Eye and Medical Study (UEMS)	Subnational	urban	40+	40+	1360	706	

Country	Data years	Survey/study name/citation	Level of representative-ness	Rural, urban, or both	Age range as used for global analysis		Sample size		Note
					Female	Male	Female	Male	
Rwanda	2000	DHS	National	both	15-49		9178		
Rwanda	2005	DHS	National	both	15-49		5212		
Rwanda	2010	DHS	National	both	15-49	15-59	6572	6472	
Rwanda	2012	STEPS	National	both	15-64	15-64	4241	2644	
Rwanda	2014-2015	DHS	National	both	15-49	15-59	6313	6366	
Saint Kitts and Nevis	2007	STEPS	Subnational	both	25-64	25-64	849	511	
Saint Kitts and Nevis	2011	Global School-based Student Health Survey	National	both	13-17	13-17	814	651	
Saint Lucia	1981	Population Study of Blood Pressure and Associated Factors in St Lucia, West Indies	National	both	15+	15+	191	168	
Saint Lucia	1991-1994	Cooper et al., Am J Public Health 87(2):160-68, 1997	Community	urban	25-100	25-100	593	491	
Saint Lucia	2012	STEPS	National	both	25-64	25-64	939	587	
Samoa	1979-1982	McGarvey, Am J Clin Nutr 53(6 Suppl):1586S-1594S, 1991	National	both	5+	5+	501	469	
Samoa	1991	McGarvey, Pac Health Dialog 8(1):157-62, 2001	National	both	25+	25+	381	347	
Samoa	1995	McGarvey, Pac Health Dialog 8(1):157-62, 2001	National	both	25+	25+	153	156	
Samoa	2002	STEPS	National	both	25-64	25-64	1334	1181	
Samoa	2010	Samoan Genome-Wide Association Study	National	both	24-65	24-65	2061	1402	
Samoa	2011	Global School-based Student Health Survey	National	both	13-16	13	912	129	
Samoa	2013	STEPS	National	both	18-64	18-64	918	605	
Sao Tome and Principe	2008-2009	DHS	National	both	15-49	15-59	2238	2173	
Sao Tome and Principe	2009	STEPS	National	both	25-64	25-64	1287	998	
Saudi Arabia	1985-1988	National Nutrition Survey	National	both	5-75	5-75	5944	4356	
Saudi Arabia	1989-1994	National Nutrition Survey	National	both	18-40	18-40	3294	2481	
Saudi Arabia	1990-1993	National Epidemiological Household Survey	National	both	20-60	20-60	4509	4882	
Saudi Arabia	1990-1993	Saudi National Survey	National	both	30-70	30-70	1648	1612	
Saudi Arabia	1992-1995	Saudi Health Information Survey	National	both	14-50	14-50	7707	4830	
Saudi Arabia	1995	National Household Survey	National	both	20-70	20-70	7074	7121	
Saudi Arabia	1995-2000	National Epidemiological Health Survey	National	both	30-70	30-70	9008	8215	
Saudi Arabia	2004-2005	Al-Baghli et al., Saudi Med J 29(9):1319-25, 2008	Subnational	both	30+	30+	97254	97254	
Saudi Arabia	2005	El Mouzan et al., Ann Saudi Med 30(3):203Ð208, 2010	National	both	5-18	5-18	9519	9853	
Saudi Arabia	2005	STEPS	National	both	15-64	15-64	2345	2245	
Saudi Arabia	2007	World Health Survey	National	both	18+	18+	87	102	
Saudi Arabia	2009-2010	Arab Teens Lifestyle Study (ATLS)	Subnational	urban	14-19	14-19	1479	1384	
Saudi Arabia	2011-2013	Jeddah City Study	Community	urban	5+	5+	867	957	
Saudi Arabia	2011-2012	Time for an Adolescent Health Surveillance System in Saudi Arabia: Findings from Jeeluna	National	both	10-19	10-19	5592	5858	
Saudi Arabia	2013	Saudi Health Information Survey	National	both	15+	15+	5249	5088	
Senegal	1984	Maire et al., Rev Epidemiol Sante Publique 40:252-58, 1992	National	rural	25-45		1628		
Senegal		Astagneau et al., J Hypertens 10(9):1095-101, 1992	Community	urban	15+	15+	707	651	
Senegal	1986	Maire et al., Rev Epidemiol Sante Publique 40:252-58, 1992	Community	urban	26-45		616		
Senegal	1992-1993	DHS	National	both	20-49		2713		
Senegal	2003	Holdsworth et al., Int J Obes Relat Metab Disord 28(12):1561-68, 2004	Community	urban	20-49		301		
Senegal	2005	DHS	National	both	15-49		4167		
Senegal	2010-2011	DHS	National	both	15-49	15-59	5498	4716	
Serbia	1984	MONICA, Novi Sad	Community	urban	25-64	25-64	777	798	
Serbia	1988-1989	MONICA, Novi Sad	Community	urban	25-64	25-64	791	778	
Serbia	1994-1995	MONICA, Novi Sad	Community	urban	25-64	25-64	670	600	
Serbia	2000	Health Status, Health Needs and Utilization of Health Care of the Population of Serbia	National	both	7+	7+	6189	5080	
Serbia	2006	The 2006 National Health Survey for the Population of Serbia	National	both	7+	7+	8558	7888	
Serbia	2013	The National Health Survey 2013	National	both	7+	7+	8140	7205	
Serbia	2013-2014	Stay Fit for Lifelong Health; the Prevalence of Lifestyle Health Conditions in Serbian Populatior	National	urban		18-65		1366	
Serbia	2015	Childhood Obesity Surveillance Initiative	National	both	6-9	6-9	2385	2475	10
Seychelles	1989	Seychelles Heart Survey I	National	both	25-64	25-64	568	513	
Seychelles	1994	Seychelles Heart Survey II	National	both	25-64	25-64	563	499	
Seychelles	1998	School Screening Program	National	both	5-19	5-19	1425	1567	
Seychelles	1999	School Screening Program	National	both	5-19	5-19	2786	2659	
Seychelles	2000	School Screening Program	National	both	5-19	5-19	1831	1783	
Seychelles	2001	School Screening Program	National	both	5-19	5-19	2595	2624	
Seychelles	2002	School Screening Program	National	both	5-19	5-19	2540	2498	
Seychelles	2003	School Screening Program	National	both	5-19	5-19	3449	3384	
Seychelles	2004	School Screening Program	National	both	5-19	5-19	2318	2360	
Seychelles	2004	Seychelles Heart Survey III	National	both	25-64	25-64	687	568	
Seychelles	2005	School Screening Program	National	both	5-19	5-19	2777	2742	
Seychelles	2006	School Screening Program	National	both	5-19	5-19	2620	2693	

Country	Data years	Survey/study name/citation	Level of representative-ness	Rural, urban, or both	Age range as used for global analysis		Sample size		Note
					Female	Male	Female	Male	
Seychelles	2007	Global School-based Student Health Survey	National	both	13-17	13-17	467	387	
Seychelles	2011	School Screening Program	National	both	5-19	5-19	2274	2311	
Seychelles	2012	School Screening Program	National	both	5-19	5-19	2331	2298	
Seychelles	2013	School Screening Program	National	both	5-19	5-19	2089	1938	
Seychelles	2013-2014	Seychelles Heart Survey IV	National	both	25-64	25-64	698	531	
Seychelles	2014	School Screening Program	National	both	5-19	5-19	2225	2148	
Seychelles	2015	Global School-based Student Health Survey	National	both	13-17	13-17	888	770	
Seychelles	2015	School Screening Program	National	both	5-19	5-19	2003	2002	
Sierra Leone	2008	DHS	National	both	15-49		3275		
Sierra Leone	2009	STEPS	National	both	25-64	25-64	2318	2200	
Sierra Leone	2013	DHS	National	both	15-49	15-59	7462	7039	
Singapore	1982-1985	Thyroid Heart Study	National	both	18-91	18-91	990	1030	
Singapore	1992	National Health Survey 1992	National	both	18-64	18-64	1704	1743	
Singapore	1993-1995	NUH Heart Study	National	both	26-89	26-89	484	498	
Singapore	1998	National Health Survey 1998	National	both	18-69	18-69	2265	2284	
Singapore	2004	National Health Survey 2004	National	both	18-74	18-74	2095	2059	
Singapore	2004-2007	Combined follow up of Singapore Cardiovascular Cohort study and Singapore Prospective study	National	both	24-95	24-95	2686	2471	
Singapore	2009	Social Isolation, Health and Lifestyles Survey (SIHLS) 2009	National	both	60+	60+	2382	2038	
Singapore	2009-2011	The Singapore Chinese Eye Study	Community	both	40+	40+	1679	1652	
Singapore	2012-2013	Singapore Health Study 2012	National	both	18-79	18-79	1026	956	
Slovakia	1993	Countrywide Integrated Noncommunicable Diseases Intervention Programme	National	both	15-64	15-64	1293	876	
Slovakia	1998	Countrywide Integrated Noncommunicable Diseases Intervention Programme	National	both	15-64	15-64	1122	923	
Slovakia	2003	Countrywide Integrated Noncommunicable Diseases Intervention Programme	National	both	15-64	15-64	905	664	
Slovakia	2008	Countrywide Integrated Noncommunicable Diseases Intervention Programme	National	both	15-64	15-64	584	412	
Slovakia	2011-2012	European Health Examination Survey	National	both	18-64	18-64	1080	884	
Slovenia	2008	Childhood Obesity Surveillance Initiative 1	National	both	6-9	6-9	5918	6022	10
Slovenia	2010	Childhood Obesity Surveillance Initiative 2	National	both	6-9	6-9	7736	8202	10
Slovenia	2013	Childhood Obesity Surveillance Initiative 3	National	both	6-9	6-9	11665	12181	10
Slovenia	2013-2014	Analysis of Children's Development in Slovenia (ACDSi), 10-15 year olds	National	both	6-15	6-15	1627	1665	
Slovenia	2014	Analysis of Children's Development in Slovenia (ACDSi), 15-19 year olds	National	urban	14-19	14-19	724	703	
Slovenia	2014	the SLOFIT monitoring system	National	both	18-21	18-21	6410	6460	
Solomon Islands	2006	STEPS	Subnational	both	15-64	15-64	1373	1031	
Solomon Islands	2009-2010	Furusawa et al., N Z Med J 124(1333):17-28, 2011	Subnational	rural	5-87	5-87	317	256	
Solomon Islands	2009-2010	Furusawa et al., N Z Med J 124(1333):17-28, 2011	Community	urban	5-70	5-70	95	60	
Solomon Islands	2015	STEPS	National	both	18-69	18-69	979	817	
South Africa	1989	Temple et al., Ethn Dis 11(3):431-7, 2001	Community	both	15+	15+	614	457	
South Africa	1990	Steyn et al., East Afr Med J 75(1):35-40, 1998	Community	urban	25-64	25-64	373	292	
South Africa	1996	Ellisras Longitudinal Study	Community	rural	5-10	5-10	535	583	
South Africa	1996	Temple et al., Ethn Dis 11(3):431-7, 2001	Community	both	15+	15+	406	302	
South Africa	1997	Ellisras Longitudinal Study	Community	rural	5-11	5-11	968	1046	
South Africa	1998	DHS	National	both	15+	15+	7759	5647	
South Africa	1998	Ellisras Longitudinal Study	Community	rural	5-12	5-12	856	958	
South Africa	1999	Ellisras Longitudinal Study	Community	rural	5-13	5-13	917	991	
South Africa	2000	Ellisras Longitudinal Study	Community	rural	5-14	5-14	877	936	
South Africa	2000-2001	Transition and Health during Urbanisation of South Africans: Children	Subnational	both	9-15	9-15	639	606	
South Africa	2001	Ellisras Longitudinal Study	Community	rural	6-15	6-15	904	962	
South Africa	2002	Ellisras Longitudinal Study	Community	rural	7-16	7-16	823	890	
South Africa	2002	The 1st South African National Youth Risk Behaviour Survey	National	both	14-18	14-18	4650	3892	
South Africa	2002-2003	SASPI	Community	rural	35+	35+	275	80	
South Africa	2003	DHS	National	both	15+	15+	4497	3200	
South Africa	2003	Ellisras Longitudinal Study	Community	rural	8-17	8-17	858	911	
South Africa	2003-2004	Africa Centre Biomeasure Survey	Community	rural	25-49	25-49	1693	778	
South Africa	2004-2006	Li et al., Curationis 30(4):79-87, 2007	Community	both	18-40	18-40	270	334	
South Africa	2007-2008	Cardiometabolic risk profile of South African Learners	Subnational	both	10-16	10-16	776	496	
South Africa	2007-2008	SAGE	National	both	50+	50+	2061	1543	
South Africa	2008	National Income Dynamics Study Wave I	National	both	5+	5+	10661	8135	
South Africa	2008	The 2nd South African National Youth Risk Behaviour Survey	National	both	14-18	14-18	4462	4090	
South Africa	2008-2009	Cape Town Bellville South Cohort Study - Baseline evaluation I	Community	urban	16+	16+	717	220	
South Africa	2010	Africa Centre Biomeasure Survey	Community	rural	15+	15+	6364	2934	
South Africa	2010-2011	National Income Dynamics Study Wave II	National	both	5+	5+	11100	8469	
South Africa	2012	National Income Dynamics Study Wave III	National	both	5+	5+	14136	10950	

Country	Data years	Survey/study name/citation	Level of representative-ness	Rural, urban, or both	Age range as used for global analysis		Sample size		Note
					Female	Male	Female	Male	
South Africa	2012	South African National Health and Nutrition Examination Survey	National	both	18+	18+	4320	2274	
South Africa	2014-2015	Health and Aging in Africa: A Longitudinal Study of an INDEPTH Community in South Africa (HAALSI)	Community	rural	40+	40+	2502	2141	
South Korea	1986	INTERSALT	Community	urban	20-59	20-59	98	100	
South Korea	1990	Korean National Blood Pressure Survey	National	both	30+	30+	12620	9734	
South Korea	1992-1993	Park et al., Diabetes Res Clin Pract 34 Suppl:S65-72, 1996	Subnational	both	30+	30-89	1392	1077	
South Korea	1997-1998	National Anthropometric Survey in Korean Children and Adolescents	National	both	5-19	5-19	26469	29318	
South Korea	1998	Korea National Health and Nutrition Examination Survey	National	both	10+	10+	5187	4514	
South Korea	2001	Korea National Health and Nutrition Examination Survey	National	both	5+	5+	4814	4151	
South Korea	2001	Kim et al., Br J Psychiatry 185:102-7, 2004	Community	both	65+	65+	432	300	
South Korea	2002-2003	Korean National Health Insurance	National	both	40+	40+	2505676	3001504	
South Korea	2004-2005	Korean National Health Insurance	National	both	40+	40+	3288531	3614273	
South Korea	2005	Korea National Health and Nutrition Examination Survey	National	both	5+	5+	3896	3183	
South Korea	2005	National Anthropometric Survey in Korean Children and Adolescents	National	both	5-19	5-19	39200	41727	
South Korea	2006-2007	Korean National Health Insurance	National	both	40+	40+	4402335	4359202	
South Korea	2007	Korea National Health and Nutrition Examination Survey	National	both	5+	5+	2174	1753	
South Korea	2008	Korea National Health and Nutrition Examination Survey	National	both	5+	5+	4824	3849	
South Korea	2008-2009	Korean National Health Insurance	National	both	40+	40+	5785629	5520401	
South Korea	2009	Korea National Health and Nutrition Examination Survey	National	both	5+	5+	5182	4288	
South Korea	2009	Korea National School Health Examination Survey (KNSHES)	National	both	6-20	6-20	89889	104137	
South Korea	2010	Korea National Health and Nutrition Examination Survey	National	both	5+	5+	4312	3583	
South Korea	2010-2011	Korean National Health Insurance	National	both	40+	40+	7148608	6688448	
South Korea	2011	Korea National Health and Nutrition Examination Survey	National	both	5+	5+	4193	3363	
South Korea	2011	Korea National School Health Examination Survey (KNSHES)	National	both	6-20	6-20	83052	97370	
South Korea	2012	Korea National Health and Nutrition Examination Survey	National	both	5+	5+	4030	3194	
South Korea	2012	Korea National School Health Examination Survey (KNSHES)	National	both	6-20	6-20	42005	45066	
South Korea	2012-2013	Korean National Health Insurance	National	both	40+	40+	7799780	7269769	
South Korea	2013	Korea National Health and Nutrition Examination Survey	National	both	5+	5+	3934	3211	
South Korea	2013	Korea National School Health Examination Survey (KNSHES)	National	both	6-20	6-20	40789	43679	
South Korea	2014	Korea National Health and Nutrition Examination Survey	National	both	5+	5+	3769	2966	
South Korea	2014	Korea National School Health Examination Survey (KNSHES)	National	both	6-20	6-20	39988	42577	
South Korea	2014-2015	Korean National Health Insurance	National	both	40+	40+	8369082	7880447	
South Korea	2015	Korea National Health and Nutrition Examination Survey	National	both	5+	5+	3640	3022	
South Korea	2015	Korea National School Health Examination Survey (KNSHES)	National	both	6-20	6-20	41648	43144	
South Korea	2016	Korea National School Health Examination Survey (KNSHES)	National	both	6-20	6-20	40634	42242	
Spain	1985	INTERSALT, Manresa	Community	urban	20-59	20-59	100	100	
Spain	1986	INTERSALT, Torrejo	Community	urban	20-59	20-59	100	100	
Spain	1986-1988	MONICA, Catalonia	Community	urban	25-64	25-64	1271	1251	
Spain	1989	Cardiovascular Risk Factors Study in Catalonia	Subnational	both	15+	15+	375	330	
Spain	1989-1994	SEEDO	Subnational	both	25-60	25-60	2855	2533	
Spain	1990	Banegas et al., Hypertension 32(6):998-1002, 1998	National	both	35-65	35-65	1203	810	
Spain	1990-1992	MONICA, Catalonia	Community	urban	25-64	25-64	1191	1719	
Spain	1990-2000	SEEDO	Subnational	both	25-60	25-60	5178	4707	
Spain	1991-1993	Encuesta de Factores de Riesgo Cardiovascular en la Región de Murcia (Cardiovascular Risk Factors Survey)	Subnational	both	18-69	18-69	1562	1512	
Spain	1992	CINDI	Subnational	both	25-64	25-64	1454	1194	
Spain	1992	ENCAT	Community	both	25-80	25-80	952	786	
Spain	1994-1995	Encuesta de Nutrición y Salud Comunidad Valenciana 1994-95 (ENCV)	Subnational	urban	15+	15+	960	830	
Spain	1994-1996	MONICA, Catalonia	Community	urban	25-64	25-64	1628	1800	
Spain	1996	Guía Study	Community	urban	30+	30+	384	305	
Spain	1996-2002	Castells et al., J Epidemiol Community Health 60(4):316-21, 2006	Community	urban	50-69		26963		
Spain		Soriguer et al., Eur J Epidemiol 19(1):33-40, 2004	Community	rural	18-65	18-65	613	613	
Spain	1998-2000	EnKID study	National	both	5-24	5-24	1730	1452	
Spain	1999-2000	ENIB	Community	both	20-60	20-60	702	498	
Spain	1999-2000	Factores de riesgo en las islas Baleares: Estudio CORSAIB	Subnational	both	35-74	35-74	864	811	
Spain	2000-2005	CDC of the Canary Islands	Community	both	18-75	18-75	3770	2883	
Spain	2000-2001	Regidor et al., J Hum Hypertens 20(1):73-82, 2006	National	both	60+	60+	2281	1318	
Spain	2000-2001	EUREYE Study	Subnational	both	65+	65+	324	274	
Spain	2001-2002	Catalan Health Interview Survey	Subnational	both	18-74	18-74	745	597	
Spain	2001-2003	DIabetes, Nutrición y Obesidad en la población adulta de la Región de Murcia (DINO)	Subnational	both	20+	20+	828	715	
Spain	2002-2003	ENCAT	Community	both	25-80	25-80	813	712	
Spain	2003	The European Male Ageing Study	Community	both		40+		405	
Spain	2003-2005	Registre Gironi del Cor (REGICOR)	Subnational	both	35-79	35-79	3266	2951	

Country	Data years	Survey/study name/citation	Level of representative-ness	Rural, urban, or both	Age range as used for global analysis		Sample size		Note
					Female	Male	Female	Male	
Spain	2004	Cardiovascular Risk Study in Castilla y León (RECCyL)	Subnational	both	15+	15+	2077	1903	
Spain	2004	Vioque J et al. Obesity 2008; 16: 664-670	Community	urban	24+	24+	115	87	
Spain	2004-2006	PREVICTUS	National	both	60+	60+	3640	3193	
Spain	2006-2008	Biblioni Mdel et al., Br J Nutr 103(1):99-106, 2010	Community	both	12-17	12-17	652	571	
Spain	2006-2007	HELENA	Community	urban	12-17	12-17	193	188	
Spain	2007-2009	Harmonizing Equation of Risk in Mediterraneon countries EXtremadura	Subnational	both	25-79	25-79	1498	1298	
Spain	2008-2010	Study on Nutrition and Cardiovascular Risk in Spain	National	both	18+	18+	6397	5756	
Spain	2008	The European Male Ageing Study	Community	both		40+		272	
Spain	2009	Cardiovascular Risk Study in Castilla y León (RECCyL)	Subnational	both	20+	20+	1590	1315	
Spain	2010-2011	Childhood Obesity Surveillance Initiative 2	National	both	6-9	6-9	3817	3839	10
Spain	2012-2013	Brain Development and Air Pollution Ultrafine Particles in School Children-BREATHE Project	Subnational	urban	7-12	7-12	1325	1338	
Spain	2012-2013	AMICS-INfancia y Medio Ambiente (Childhood and Environment) Project - Menorca	Subnational	both	14-15	14-15	165	162	
Spain	2012-2013	Infancia y Medio Ambiente (Childhood and Environment) Birth Cohort study - Sabadell	Subnational	urban	5-8	5-8	260	280	
Spain	2012-2013	Valencia cohort INMA Project	Subnational	both	7-8	7-8	232	229	
Spain	2013	Childhood Obesity Surveillance Initiative 3	National	both	6-9	6-9	1744	1682	10
Spain	2014	Cardiovascular Risk Study in Castilla y León (RECCyL)	Subnational	both	20+	20+	1475	1215	
Spain	2015-2016	Infancia y Medio Ambiente Gipuzkoa, 8 years	Subnational	both	7-8	7-8	189	192	
Spain	2015	National Study of Nutrition and Cardiovascular Risk (ENRICA)	National	both	65+	65+	770	712	
Sri Lanka	2003	Wijewardene et al., Ceylon Med J 50:62-70, 2005	Subnational	both	30-65	30-65	296	275	
Sri Lanka	2003	Wijewardene et al., Ceylon Med J 50:62-70, 2005	Subnational	both	30-65	30-65	192	139	
Sri Lanka	2003	Wijewardene et al., Ceylon Med J 50:62-70, 2005	Subnational	both	30-65	30-65	2410	1891	
Sri Lanka	2003	Wijewardene et al., Ceylon Med J 50:62-70, 2005	Subnational	both	30-65	30-65	457	387	
Sri Lanka	2006	STEPS	National	both	15-64	15-64	6211	6140	
Sri Lanka	2006-2007	DHS	National	both	20-49		12539		
Sri Lanka	2014	STEPS	National	both	18-69	18-69	2893	1863	
Sri Lanka	2016	Global School-based Student Health Survey	National	both	13-17	13-17	928	671	
Sudan	2005	STEPS	Subnational	both	25-64	25-64	881	626	
Sudan	2012	Global School-based Student Health Survey	National	both		13		61	
Suriname	2009	Global School-based Student Health Survey	National	both	13	13	133	95	
Suriname	2013-2015	The Healthy Life in Suriname Study (HELISUR)	Subnational	urban	18-70	18-70	722	424	
Suriname	2016	Global School-based Student Health Survey	National	both	13	13	189	171	
Swaziland	2006-2007	DHS	National	both	15-49	15-49	4714	4074	
Swaziland	2013	Global School-based Student Health Survey	National	both	13	13	165	103	
Swaziland	2014	STEPS	National	both	15-69	15-69	1977	1102	
Sweden	1972	The Swedish Conscription Database	National	both		17-19		50999	1
Sweden	1973	The Swedish Conscription Database	National	both		17-19		55792	1
Sweden	1974	The Swedish Conscription Database	National	both		17-19		57222	1
Sweden	1975	The Swedish Conscription Database	National	both		17-19		53371	
Sweden	1976	The Swedish Conscription Database	National	both		17-19		51624	
Sweden	1977	The Swedish Conscription Database	National	both		17-19		49996	
Sweden	1978	The Swedish Conscription Database	National	both		17-19		13073	
Sweden	1979	The Swedish Conscription Database	National	both		17-19		38307	
Sweden	1980	The Swedish Conscription Database	National	both		17-19		50786	
Sweden	1980-1984	Uppsala Longitudinal Study of Adult Men	Community	both		60		1841	
Sweden	1981	The Swedish Conscription Database	National	both		17-19		53750	
Sweden	1982	The Swedish Conscription Database	National	both		17-19		55802	
Sweden	1983	The Swedish Conscription Database	National	both		17-19		54597	
Sweden	1984	The Swedish Conscription Database	National	both		17-19		37472	
Sweden	1985	The Swedish Conscription Database	National	both		17-19		15120	
Sweden	1985	MONICA Gothenburg	Community	urban	25-64	25-64	702	666	
Sweden	1985-1989	Västerbotten Intervention Project	Subnational	both	25-64	25-64	1554	1676	
Sweden	1985-1996	EPIC Umea	Subnational	both	24-72	24-72	13217	12359	
Sweden	1986	The Swedish Conscription Database	National	both		17-19		49990	
Sweden	1986	MONICA, Northern Sweden	Subnational	urban	25-64	25-64	795	819	
Sweden	1987	The Swedish Conscription Database	National	both		17-19		50888	
Sweden	1988	The Swedish Conscription Database	National	both		17-19		49398	
Sweden	1989	The Swedish Conscription Database	National	both		17-19		49947	
Sweden	1990	The Swedish Conscription Database	National	both		17-19		50897	
Sweden	1990	MONICA, Northern Sweden	Subnational	urban	25-64	25-64	793	761	
Sweden	1990	MONICA Gothenburg	Community	urban	25-64	25-64	775	775	
Sweden	1990-1992	Västerbotten Intervention Project	Subnational	both	25-64	25-64	7804	7263	

Country	Data years	Survey/study name/citation	Level of representative-ness	Rural, urban, or both	Age range as used for global analysis		Sample size		Note
					Female	Male	Female	Male	
Sweden	1991	The Swedish Conscription Database	National	both		17-19		51060	
Sweden	1991-1995	Uppsala Longitudinal Study of Adult Men	Community	both		70		1215	
Sweden	1991-1996	Malmö Diet and Cancer	Community	urban	45-73	45-73	18293	12096	
Sweden	1992	The Swedish Conscription Database	National	both		17-19		49185	
Sweden	1993	The Swedish Conscription Database	National	both		17-19		48274	
Sweden	1993-1995	Västerbotten Intervention Project	Subnational	both	25-64	25-64	10727	9804	
Sweden	1994	The Swedish Conscription Database	National	both		17-19		45519	
Sweden	1994	Helicobacter Pylori	Community	urban	56-65	56-65	217	170	
Sweden	1994	MONICA, Northern Sweden	Subnational	urban	25-64	25-64	761	736	
Sweden	1994-1996	Kungsholmen Project	Community	urban	75+	75+	160	160	
Sweden	1995	The Swedish Conscription Database	National	both		17-19		45641	
Sweden	1995	MONICA Gothenburg	Community	urban	25-64	25-64	867	745	
Sweden	1996	The Swedish Conscription Database	National	both		17-19		45558	
Sweden	1996-1998	Västerbotten Intervention Project	Subnational	both	25-64	25-64	8893	8327	
Sweden	1997	The Swedish Conscription Database	National	both		17-19		37859	
Sweden	1997-2001	Uppsala Longitudinal Study of Adult Men	Community	both		77		783	
Sweden	1998	The Swedish Conscription Database	National	both		17-19		43902	
Sweden	1998-1999	European Youth Heart Study (EYHS) I	Subnational	both	8-16	8-16	602	525	
Sweden	1998-2001	The Kalixanda study	Community	both	20+	20+	483	508	
Sweden	1999	The Swedish Conscription Database	National	both		17-19		39202	
Sweden	1999	MONICA Northern Sweden	Subnational	both	25-74	25-74	930	889	
Sweden	1999-2003	Västerbotten Intervention Project	Subnational	both	25-64	25-64	6384	6354	
Sweden	2000	The Swedish Conscription Database	National	both		17-19		32719	
Sweden	2000-2002	The COMPASS study	Community	urban	14-16	14-16	1597	1718	
Sweden	2000-2001	H70 Study	Community	urban	70	70	270	242	
Sweden	2001	The Swedish Conscription Database	National	both		17-19		31153	
Sweden	2001-2003	Uppsala Longitudinal Study of Adult Men	Community	both		82		511	
Sweden	2001-2004	Swedish INTERGENE Cohort Study	Subnational	both	24-76	24-76	1906	1694	
Sweden	2001-2004	Prospective Investigation of the Vasculature in Uppsala Seniors (PIVUS)	Community	both	70	70	509	507	
Sweden	2002	The Swedish Conscription Database	National	both		17-19		25896	
Sweden	2003	The Swedish Conscription Database	National	both		17-19		30673	
Sweden	2003	The European Male Ageing Study	Community	both		40+		396	
Sweden	2003-2004	Welin et al., BMC Public Health 8:403, 2008	Community	urban	50	50	655	595	
Sweden	2003-2004	Welin et al., BMC Public Health 8:403, 2008	Community	urban		60		667	
Sweden	2004	The Swedish Conscription Database	National	both		17-19		29146	
Sweden	2004-2005	European Youth Heart Study (EYHS) II	Subnational	both	15-21	15-21	262	196	
Sweden	2004	MONICA Northern Sweden	Subnational	both	25-74	25-74	958	908	
Sweden	2004-2005	Population Study of Women in Gothenburg	Community	urban	38-50		494		
Sweden	2005	The Swedish Conscription Database	National	both		17-18		25836	
Sweden	2005-2006	H70 Study	Community	urban	75	75	422	320	
Sweden	2006-2007	HELENA	Community	urban	12-17	12-17	208	132	
Sweden	2006-2009	Prospective Investigation of the Vasculature in Uppsala Seniors (PIVUS)	Community	both	75	75	419	407	
Sweden	2007-2010	Identification and prevention of Dietary- and lifestyle-induced health EFfects In Children and infantS (IDEFICS)	Community	urban	5-9	5-9	557	557	
Sweden	2008	Childhood Obesity Surveillance Initiative 1	National	both	6-9	6-9	1795	1921	10
Sweden	2008	The European Male Ageing Study	Community	both		40+		353	
Sweden	2009	MONICA Northern Sweden	Subnational	both	25-74	25-74	861	849	
Sweden	2014	MONICA Northern Sweden	Subnational	both	25-74	25-74	801	753	
Switzerland	1984-1986	The Swiss MONICA Study Wave I	Subnational	both	25-74	25-74	1689	1744	
Switzerland	1988-1989	The Swiss MONICA Study Wave II	Subnational	both	25-74	25-74	1684	1778	
Switzerland	1992-1993	The Swiss MONICA Study Wave III	Subnational	both	25-74	25-74	1672	1577	
Switzerland	2002	Detection of overweight and obesity in a national sample of 6-12 year old children	National	both	6-13	6-13	1235	1196	
Switzerland	2003-2006	Cohorte Lausannoise	Community	urban	35-75	35-75	3536	3186	
Switzerland	2004	The Swiss Conscription Database	National	both		18-20		20491	
Switzerland	2005	The Swiss Conscription Database	National	both		18-20		32131	
Switzerland	2005	Kinder- und Jugendsportstudie (KISS)	Subnational	both	6-13	6-13	256	239	
Switzerland	2005-2006	Chiolero et al., J Hypertens 25(11):2209-17, 2007	Subnational	both	10-14	10-14	2586	2621	
Switzerland	2006	The Swiss Conscription Database	National	both		18-20		34530	
Switzerland	2006	Kinder- und Jugendsportstudie (KISS)	Subnational	both	7-14	7-14	103	100	
Switzerland	2007	Prevalence of overweight and obesity in 6-12 year old children in Switzerland: a 5-year follow-up study	National	both	6-13	6-13	1139	1082	
Switzerland	2007	The Swiss Conscription Database	National	both		18-20		36194	
Switzerland	2007-2012	Bus Santé Study	Subnational	both	20-80	20-80	1911	1884	

Country	Data years	Survey/study name/citation	Level of representative-ness	Rural, urban, or both	Age range as used for global analysis		Sample size		Note
					Female	Male	Female	Male	
Switzerland	2008	The Swiss Conscription Database	National	both		18-20		34497	
Switzerland	2009	The Swiss Conscription Database	National	both		18-20		34896	
Switzerland	2009	Kinder- und Jugendsportstudie (KISS)	Subnational	both	10-17	10-17	65	44	
Switzerland	2009-2012	Cohorte Lausannoise	Community	urban	40-81	40-81	2494	2176	
Switzerland	2010	The Swiss Conscription Database	National	both		18-20		37214	
Switzerland	2010-2013	BMI Monitoring for Switzerland	Subnational	both	5-15	5-15	12883	13558	
Switzerland	2011	The Swiss Conscription Database	National	both		18-20		38108	
Switzerland	2012	Prevalence of overweight and obesity in 6-12 year old children in Switzerland: a 5- and 10-year follow-up study	National	both	6-13	6-13	1464	1499	
Switzerland	2012	The Swiss Conscription Database	National	both		18-20		36938	
Switzerland	2013	The Swiss Conscription Database	National	both		18-20		35673	
Switzerland	2014	The Swiss Conscription Database	National	both		18-20		34914	
Switzerland	2014-2015	BMI Monitoring for Switzerland	Community	urban	5-15	5-15	6435	6626	
Switzerland	2015	The Swiss Conscription Database	National	both		18-20		34724	
Syrian Arab Republic	2002	National Survey on non-communicable diseases and factors affecting their development	National	both	20-64	20-64	4045	3155	
Syrian Arab Republic	2010	Global School-based Student Health Survey	National	both	13	13	508	349	
Taiwan	1985	INTERSALT	Community	rural	20-59	20-59	92	89	
Taiwan	1989-1991	Chiu et al., J Gerontol A Biol Sci Med Sci 55(11):M684-90, 2000	Subnational	both	65+	65+	1308	1322	
Taiwan	1993-1996	Nutrition and Health Survey in Taiwan 1993-1996	National	both	5+	5+	3217	2959	
Taiwan	1993-1994	The Kinmen Neurological Disorders Survey	Community	urban	50+	50+	593	672	
Taiwan	1999-2000	Nutrition and Health Survey in Taiwan 1999-2000	National	both	65+	65+	1202	1271	
Taiwan	2001-2002	Nutrition and Health Survey in Taiwan 2001-2002	National	both	6-12	6-12	1139	1334	
Taiwan	2002	Taiwanese Survey on Hypertension, Hyperglycemia and Hyperlipidemia	National	both	15+	15+	3471	3461	
Taiwan	2004-2005	TCHS	Community	urban	40+	40+	1212	1147	
Taiwan	2005-2008	Nutrition and Health Survey in Taiwan 2005-2008	National	both	19+	19+	1355	1311	
Taiwan	2007	Taiwanese Survey on Hypertension, Hyperglycemia and Hyperlipidemia	National	both	20+	20+	2469	2155	
Taiwan	2012	Global School-based Student Health Survey	National	both	13-17	13-17	2927	2998	
Taiwan	2013-2015	Nutrition and Health Survey in Taiwan	National	both	5-18	5-18	831	832	
Tajikistan	2003	Micronutrient Status Survey	National	both	15-49		2044		
Tajikistan	2012	DHS	National	both	15-49		8930		
Tajikistan	2016	STEPS	National	both	18-69	18-69	1554	1091	
Tanzania	1991-1992	DHS	National	both	20-49		4039		
Tanzania	1996	DHS	National	both	20-49		3514		
Tanzania	1996-1997	Aspray et al., Trans R Soc Trop Med Hyg 94:637-44, 2000	Community	rural	15+	15+	324	251	
Tanzania	1996-1997	Aspray et al., Trans R Soc Trop Med Hyg 94:637-44, 2000	Community	urban	15+	15+	118	117	
Tanzania	1998-1999	Bovet et al., Int J Epidemiol 31(1):240-7, 2002	Community	urban	25-64	25-64	5646	3593	
Tanzania	2004-2005	DHS	National	both	15-49		9160		
Tanzania	2010	DHS	National	both	15-49		9099		
Tanzania	2011	STEPS	Subnational	both	25-64	25-64	1517	1008	
Tanzania	2012	STEPS	National	both	25-64	25-64	2820	2581	
Tanzania	2014	Dar es Salaam Urban Cohort Hypertension Study	Community	urban	40+	40+	1266	965	
Tanzania	2015-2016	DHS	National	both	15-49		12036		
Thailand	1987	INCLEN	Community	rural		35-65		244	
Thailand	1989	INCLEN	Community	rural		35-65		209	
Thailand	1989	INCLEN	Community	urban		35-65		207	
Thailand	1991	Thailand National Health Examination Survey I	National	both	5+	5+	11021	8691	
Thailand	1995	The Fourth National Nutrition Survey of Thailand- 1995	National	both	20-60	20-60	3631	1405	
Thailand	1997	Thailand National Health Examination Survey II	National	both	5-59	5-59	4877	4118	
Thailand	2000	InterASIA	National	both	35+	35+	3211	2092	
Thailand	2003-2004	The Fifth National Nutrition Survey of Thailand	National	both	19+	19+	3366	1961	
Thailand	2004	Thailand National Health Examination Survey III	National	both	15+	15+	20137	18819	
Thailand	2009	Thailand National Health Examination Survey IV	National	both	5-98	5-98	13848	12972	
Thailand	2011	SEANUTS	National	both	5-12	5-12	939	922	
Timor-Leste	2009-2010	DHS	National	both	15-49		11984		
Timor-Leste	2009-2010	Timor-Leste Eye Health Survey	Subnational	both	40+	40+	247	245	
Timor-Leste	2014	STEPS	National	both	18-69	18-69	1437	1048	
Timor-Leste	2015	Global School-based Student Health Survey	National	both	13	13	171	97	
Togo	1998	DHS	National	both	20-49		3114		
Togo	2010	STEPS	National	both	15-64	15-64	2095	2063	
Togo	2013-2014	DHS	National	both	15-49		4398		
Tokelau	2005	STEPS	National	both	15-64	15-64	296	270	
Tokelau	2014	STEPS	National	both	18-64	18-64	276	261	

Country	Data years	Survey/study name/citation	Level of representative-ness	Rural, urban, or both	Age range as used for global analysis		Sample size		Note
					Female	Male	Female	Male	
Tonga	2004	STEPS	National	both	15-64	15-64	552	403	
Tonga	2005-2007	Pacific Obesity Prevention in Communities - Ma'alahi Youth Project	Subnational	rural	11-19	11-19	1445	1206	
Tonga	2007-2008	Pacific Obesity Prevention in Communities - Ma'alahi Youth Project	Subnational	rural	13-22	13-22	579	434	
Tonga	2010	Global School-based Student Health Survey	National	both	13-17	13-17	1069	927	
Tonga	2011	STEPS	National	both	15-64	15-64	1401	878	
Trinidad and Tobago	1985	INTERSALT	Community	urban	20-59	20-59	92	84	
Trinidad and Tobago	1999	Child Health Survey	National	both	5-9	5-9	3272	3060	
Trinidad and Tobago	2001	Adult Survey	National	rural	25+	25+	267	198	
Trinidad and Tobago	2003	Child Health Survey	National	both	5-9	5-9	1976	1834	
Trinidad and Tobago	2003	National Survey of Senior School Health	National	both	15-16	15-16	1112	828	
Trinidad and Tobago	2011	Global School-based Student Health Survey	National	both	13-15	13-15	782	914	
Tunisia	1996-1997	Tunisian National Nutrition Survey 1996-1997	National	both	5+	5+	4124	2725	
Tunisia	1996-1997	Ariana Healthy Project 1997	Community	both	35-65	35-65	2711	2664	
Tunisia	2005	Aounallah et al., Public Health 12(1):98, 2012	National	both	15-19	15-19	1566	1290	
Tunisia	2005	Tunisian National Survey	National	both	35-71	35-71	4590	3417	
Tunisia	2009-2010	ObeMaghreb	Subnational	urban	5-49	5-49	1601	1841	
Turkey	1990	Turkish Adult Risk Factor Study	National	both	20+	20+	1369	1338	
Turkey	1993	DHS	National	both	20-49		2294		
Turkey	1995	Turkish Adult Risk Factor Study	National	both	25+	25+	879	855	
Turkey	1998	DHS	National	both	20-49		2210		
Turkey	1998	Turkish Adult Risk Factor Study	National	both	28+	28+	909	877	
Turkey	1998-1999	Erem et al., Diabetes Res Clin Pract 54(3):203-08, 2001	Community	urban	20+	20+	1322	1324	
Turkey	2000	MDHS	Subnational	urban	25-49		1420		
Turkey	2000	Turkish Adult Risk Factor Study	National	both	30+	30+	938	890	
Turkey	2000-2002	The Healthy Nutrition for Healthy Heart Study	National	both	25-84	25-84	10631	4718	
Turkey	2001	Yumuk et al., Diabetes Res Clin Pract 70(2):151-58, 2005	Community	urban	20+	20+	1789	1042	
Turkey	2001-2002	Turkish Adult Risk Factor Study	National	both	32+	32+	1210	1098	
Turkey	2002	Onal et al., Blood Press 13(1):31-6, 2004	Subnational	urban	25+	25+	355	67	
Turkey	2003	Prevalence, awareness, treatment and control of hypertension in Turkey in 2003	National	both	18+	18+	2847	1988	
Turkey	2003	DHS	National	both	20-49		2934		
Turkey	2003-2004	Turkish Adult Risk Factor Study	National	both	34+	34+	1119	961	
Turkey	2004	Nationally Representative Cross-sectional Survey	National	both	20+	20+	2154	2110	
Turkey	2005-2006	Turkish Adult Risk Factor Study	National	both	33+	33+	1088	1029	
Turkey	2007	Natinal Household survey	National	both	20-85	20-85	1842	2263	
Turkey	2007-2008	Turkish Adult Risk Factor Study	National	both	35+	35+	1133	1101	
Turkey	2009-2010	Turkish Adult Risk Factor Study	National	both	37+	37+	507	466	
Turkey	2009-2012	Prevalence of diabetes and associated risk factors among adult population in Trabzon city	Subnational	both	20+	20+	2138	1583	
Turkey	2011	Chronic Diseases and Risk Factors Survey in Turkey	National	both	15+	15+	8924	8061	
Turkey	2012-2013	Turkish Adult Risk Factor Study	National	both	37+	37+	1087	1012	
Turkey	2013	Childhood Obesity Surveillance Initiative 3	National	both	6-9	6-9	2475	2483	10
Turkey	2014-2015	Turkish Adult Risk Factor Study	National	both	44+	44+	484	437	
Turkmenistan	2000	DHS	National	both	15-49		2084		
Turkmenistan	2013	STEPS	National	both	18-64	18-64	2858	1929	
Tuvalu	1976	The Funafuti Survey	Subnational	urban	10+	10+	377	345	
Tuvalu	2013	Global School-based Student Health Survey	National	both	13-17	13-17	215	211	
Uganda	1995	DHS	National	both	20-49		2833		
Uganda	2000-2001	DHS	National	both	15-49		5830		
Uganda	2006	DHS	National	both	15-49	15-54	2539	2476	
Uganda	2011	DHS	National	both	15-49	15-54	2502	2361	
Uganda	2011-2012	The Prevalence and Distribution of Non-communicable Diseases and Their Risk Factors in Kasese District, Uganda	Subnational	both	25-79	25-79	221	277	
Uganda	2014	STEPS	National	both	18-69	18-69	2120	1560	
Ukraine	2002	National Micronutrient Survey	National	both	20-50		816		
United Arab Emirates	1989-1990	el Mugamer et al., J Trop Med Hyg 98(6):407-15, 1995	Community	both	20+	20+	199	123	
United Arab Emirates	1999-2000	Emirates National Diabetes and Coronary Artery Disease Risk Factor Study	National	both	20-80	20-80	3743	2822	
United Arab Emirates	2000-2001	carter et al., J Health Popul Nutr 22(1):75-83, 2004	Community	both	20-79		521		
United Arab Emirates	2010	Global School-based Student Health Survey	National	both	13-17	13-17	1257	948	
United Kingdom	1974	National Child Development Study (1958 British Cohort Study)	National	both	16	16	5357	5705	1
United Kingdom	1982	MRC National Survey of Health and Development	National	both	36-37	36-37	1648	1632	
United Kingdom	1983-1984	MONICA, Belfast	Subnational	both	25-64	25-64	1183	1158	
United Kingdom	1984-1986	Scottish Heart Health Survey	Subnational	both	40-59	40-59	4464	4364	
United Kingdom	1985-1986	INTERSALT, Belfast	Community	urban	20-59	20-59	100	99	

Country	Data years	Survey/study name/citation	Level of representative-ness	Rural, urban, or both	Age range as used for global analysis		Sample size		Note
					Female	Male	Female	Male	
United Kingdom	1985	INTERSALT, Birmingham	Community	urban	20-59	20-59	100	100	
United Kingdom	1985	INTERSALT, Wales	Community	urban	20-59	20-59	99	100	
United Kingdom	1986-1987	Dietary and Nutritional Survey of British Adults 1986-1987	National	both	16-64	16-64	1161	1158	
United Kingdom	1986-1987	MONICA, Belfast	Subnational	both	25-64	25-64	1185	1155	
United Kingdom	1987-1988	Edinburgh Artery Study	Community	urban	54-75	54-75	783	808	
United Kingdom	1989	MRC National Survey of Health and Development	National	both	42-44	42-44	1608	1617	
United Kingdom	1991-1992	Health Survey for England	National	both	16+	16+	3428	3114	
United Kingdom	1991	National Child Development Study (1958 British Cohort Study)	National	both	33	33	5606	5426	
United Kingdom	1991-1992	MONICA, Belfast	Subnational	both	25-64	25-64	996	998	
United Kingdom	1992	MONICA, Glasgow	Community	urban	25-64	25-64	775	696	
United Kingdom	1992-1993	Whickham Survey	Community	urban	35+	35+	784	676	
United Kingdom	1992-1994	Edinburgh Artery Study	Community	urban	60-81	60-81	582	580	
United Kingdom	1993	Health Survey for England	National	both	16+	16+	8277	7461	
United Kingdom	1993-1997	EPIC Norfolk	Subnational	both	40-79	40-79	13995	11574	
United Kingdom	1993-2000	EPIC Oxford	Subnational	both	20-98	20-98	37605	10851	
United Kingdom	1994	Health Survey for England	National	both	16+	16+	7928	6825	
United Kingdom	1994-1995	National Diet and Nutrition Survey (NDNS)	National	both	65+	65+	687	701	
United Kingdom	1995	Health Survey for England	National	both	5+	5+	9013	8038	
United Kingdom	1995	Scottish Health Survey (SHeS)	Subnational	both	16-64	16-64	4001	3303	
United Kingdom	1995	MONICA, Glasgow	Community	urban	25-64	25-64	958	855	
United Kingdom	1996	Health Survey for England	National	both	5+	5+	9436	8469	
United Kingdom	1997	Health Survey for England	National	both	5+	5+	6824	6285	
United Kingdom	1997	National Diet and Nutrition Survey (NDNS)	National	both	5-18	5-18	896	933	
United Kingdom	1997-1999	INTERMAP, WestBromwich	Community	urban	40-59	40-59	138	141	
United Kingdom	1998	Health Survey for England	National	both	5+	5+	9023	7980	
United Kingdom	1998	Scottish Health Survey (SHeS)	Subnational	both	5-74	5-74	5897	5047	
United Kingdom	1998-1999	SportsLinx	Community	urban	9-10	9-10	1364	1429	
United Kingdom	1998-1999	INTERMAP, Belfast	Community	urban	40-59	40-59	97	125	
United Kingdom	1998-2000	The British Regional Heart Study	National	urban		60-79		4138	
United Kingdom	1999	Health Survey for England	National	both	5+	5+	4304	3880	
United Kingdom	1999-2000	SportsLinx	Community	urban	9-10	9-10	1439	1469	
United Kingdom	1999	MRC National Survey of Health and Development	National	both	53-54	53-54	1496	1452	
United Kingdom	1999-2001	British Women’s Heart and Health Study	National	both	60-79		3678		
United Kingdom	1999-2001	Edinburgh Artery Study	Community	urban	66-87	66-87	404	373	
United Kingdom	1999-2004	Hertfordshire Cohort Study	Subnational	both	59-73	59-73	1416	1571	
United Kingdom	2000	Health Survey for England	National	both	5+	5+	4601	4073	
United Kingdom	2000-2001	National Diet and Nutrition Survey 2000-2001	National	both	19-64	19-64	973	807	
United Kingdom	2000-2001	SportsLinx	Community	urban	9-10	9-10	1154	1166	
United Kingdom	2001	Health Survey for England	National	both	5+	5+	8635	7463	
United Kingdom	2001-2002	SportsLinx	Community	urban	9-10	9-10	743	866	
United Kingdom	2002	Health Survey for England	National	both	5+	5+	7555	6797	
United Kingdom	2002-2003	SportsLinx	Community	urban	9-10	9-10	749	725	
United Kingdom	2003	Health Survey for England	National	both	5+	5+	8242	7137	
United Kingdom	2003	Scottish Health Survey (SHeS)	Subnational	both	5+	5+	4678	3989	
United Kingdom	2003-2004	SportsLinx	Community	urban	9-10	9-10	1931	1907	
United Kingdom	2003	The European Male Ageing Study	Community	both		40+		394	
United Kingdom	2004	Health Survey for England	National	both	5+	5+	3608	2975	
United Kingdom	2004-2005	SportsLinx	Community	urban	9-10	9-10	1713	1724	
United Kingdom	2004-2005	English Longitudinal Study of Ageing Wave 2 2004-2005	National	both	52+	52+	3966	3259	
United Kingdom	2005	Health Survey for England	National	both	5+	5+	5488	4839	
United Kingdom	2005-2006	SportsLinx	Community	urban	9-10	9-10	1410	1456	
United Kingdom	2006	Health Survey for England	National	both	5+	5+	8898	8005	
United Kingdom	2006-2007	National Child Measurement Programme	National	both	5-11	5-11	358287	380595	
United Kingdom	2006-2007	SportsLinx	Community	urban	9-10	9-10	1683	1760	
United Kingdom	2006-2010	MRC National Survey of Health and Development	National	both	60-65	60-65	1156	1061	
United Kingdom	2007	Health Survey for England	National	both	5+	5+	5696	5354	
United Kingdom	2007	Welsh Health Survey (WHS)	Subnational	both	5-15	5-15	755	784	
United Kingdom	2007-2008	National Child Measurement Programme	National	both	5-11	5-11	374787	396801	
United Kingdom	2007-2008	SportsLinx	Community	urban	9-10	9-10	1815	1852	
United Kingdom	2008	Health Survey for England	National	both	5+	5+	9457	8317	
United Kingdom	2008	Scottish Health Survey (SHeS)	Subnational	both	5+	5+	3532	2970	

Country	Data years	Survey/study name/citation	Level of representative-ness	Rural, urban, or both	Age range as used for global analysis		Sample size		Note
					Female	Male	Female	Male	
United Kingdom	2008	Welsh Health Survey (WHS)	Subnational	both	5-15	5-15	625	740	
United Kingdom	2008-2009	National Child Measurement Programme	National	both	5-11	5-11	377149	398497	
United Kingdom	2008-2009	SportsLinx	Community	urban	9-10	9-10	1854	1824	
United Kingdom	2008-2012	National Diet and Nutrition Survey (NDNS)	National	both	5+	5+	1824	1591	
United Kingdom	2008	The European Male Ageing Study	Community	both		40+		301	
United Kingdom	2008-2009	English Longitudinal Study of Ageing Wave 4 2008-2009	National	both	50+	50+	4296	3540	
United Kingdom	2009	Health Survey for England	National	both	5+	5+	3377	3242	
United Kingdom	2009	Scottish Health Survey (SHeS)	Subnational	both	5+	5+	4164	3622	
United Kingdom	2009	Welsh Health Survey (WHS)	Subnational	both	5-15	5-15	858	909	
United Kingdom	2009-2010	National Child Measurement Programme	National	both	5-11	5-11	377962	398271	
United Kingdom	2009-2010	SportsLinx	Community	urban	9-10	9-10	1429	1493	
United Kingdom	2010	Health Survey for England	National	both	5+	5+	5539	4959	
United Kingdom	2010	Scottish Health Survey (SHeS)	Subnational	both	5+	5+	3765	3207	
United Kingdom	2010	Welsh Health Survey (WHS)	Subnational	both	5-15	5-15	860	896	
United Kingdom	2010-2011	National Child Measurement Programme	National	both	5-11	5-11	373473	393148	
United Kingdom	2010-2011	SportsLinx	Community	urban	9-10	9-10	1252	1332	
United Kingdom	2011	Health Survey for England	National	both	5+	5+	4394	3680	
United Kingdom	2011	Scottish Health Survey (SHeS)	Subnational	both	5+	5+	3860	3253	
United Kingdom	2011	Welsh Health Survey (WHS)	Subnational	both	5-15	5-15	839	949	
United Kingdom	2011-2012	National Child Measurement Programme	National	both	5-11	5-11	373068	390385	
United Kingdom	2011-2012	SportsLinx	Community	urban	9-10	9-10	1331	1286	
United Kingdom	2012	Health Survey for England	National	both	5+	5+	4269	3648	
United Kingdom	2012	Scottish Health Survey (SHeS)	Subnational	both	5+	5+	2734	2380	
United Kingdom	2012	Welsh Health Survey (WHS)	Subnational	both	5-15	5-15	712	805	
United Kingdom	2012-2013	National Child Measurement Programme	National	both	5-11	5-11	377113	396704	
United Kingdom	2012-2013	English Longitudinal Study of Ageing Wave 6 2012-2013	National	both	50+	50+	4015	3257	
United Kingdom	2013	Health Survey for England	National	both	5+	5+	4566	3910	
United Kingdom	2013	Scottish Health Survey (SHeS)	Subnational	both	5+	5+	2747	2340	
United Kingdom	2013-2014	National Child Measurement Programme	National	both	5-11	5-11	389424	407633	
United Kingdom	2013-2014	National Diet and Nutrition Survey (NDNS)	National	both	5+	5+	1194	940	
United Kingdom	2014	Health Survey for England	National	both	5+	5+	4323	3712	
United Kingdom	2014	Scottish Health Survey (SHeS)	Subnational	both	5+	5+	2675	2248	
United Kingdom	2014-2015	National Child Measurement Programme	National	both	5-11	5-11	395006	412685	
United Kingdom	2015	Scottish Health Survey (SHeS)	Subnational	both	5+	5+	2531	2264	
United Kingdom	2015-2016	National Child Measurement Programme	National	both	5-11	5-11	410085	427039	
United States of America	1971-1975	US NHANES I	National	both	5-74	5-74	10587	7613	1
United States of America	1976-1980	US NHANES II	National	both	5-74	5-74	7508	6952	
United States of America	1979-1980	MONICA, Stanford	Subnational	urban	25-64	25-64	806	703	6
United States of America	1980-1982	The Minnesota Heart Survey	Community	both	25-75	25-75	1837	1611	
United States of America	1985-1986	INTERSALT, Chicago	Community	urban	20-59	20-59	99	97	
United States of America	1985-1987	The Minnesota Heart Survey	Community	both	25-75	25-75	2421	5220	
United States of America	1985-1986	MONICA, Stanford	Subnational	urban	25-64	25-64	848	713	6
United States of America	1986	INTERSALT, Goodman	Community	urban	20-59	20-59	192	192	
United States of America	1986	INTERSALT, Jackson	Community	urban	20-59	20-59	199	184	
United States of America	1988-1994	US NHANES III	National	both	5+	5+	12523	11567	
United States of America	1989-1990	MONICA, Stanford	Subnational	urban	25-64	25-64	842	720	6
United States of America	1996	National Longitudinal Study of Adolescent Health Wave II	National	both	11-21	11-21	2460	2285	7
United States of America	1996-1997	INTERMAP, Baltimore	Community	urban	40-59	40-59	134	146	
United States of America	1996-1997	INTERMAP, Jackson	Community	urban	40-59	40-59	134	132	
United States of America	1996-1998	INTERMAP, Minneapolis	Community	urban	40-59	40-59	130	130	
United States of America	1996-1997	INTERMAP, Pittsburgh	Community	urban	40-59	40-59	128	132	
United States of America	1996-1997	Study of Women's Health Across the Nation	National	both	40-55		3199		8
United States of America	1997-1998	INTERMAP, CC	Community	urban	40-59	40-59	276	271	
United States of America	1997-1998	INTERMAP, Chicago	Community	urban	40-59	40-59	159	156	
United States of America	1997-1999	Study of Women's Health Across the Nation	National	both	40-55		2760		8
United States of America	1998-1999	Coronary Artery Risk Detection in Appalachian Communities (CARDIAC), 5th Grade	Subnational	both	10-12	10-12	461	541	
United States of America	1998-2000	Study of Women's Health Across the Nation	National	both	40-55		2596		8
United States of America	1999-2000	US NHANES 1999-2000	National	both	5+	5+	3788	3809	
United States of America	1999-2001	Study of Women's Health Across the Nation	National	both	40-56		2507		8
United States of America	2000-2001	Coronary Artery Risk Detection in Appalachian Communities (CARDIAC), 5th Grade	Subnational	both	10-12	10-12	569	639	
United States of America	2000-2002	Study of Women's Health Across the Nation	National	both	40-57		2440		8

Country	Data years	Survey/study name/citation	Level of representative-ness	Rural, urban, or both	Age range as used for global analysis		Sample size		Note
					Female	Male	Female	Male	
United States of America	2001	Coronary Artery Risk Detection in Appalachian Communities (CARDIAC), 5th Grade	Subnational	both	10-12	10-12	1635	1732	
United States of America	2001-2002	National Longitudinal Study of Adolescent Health Wave III	National	both	18-28	18-28	2443	2139	7
United States of America	2001-2002	US NHANES 2001-2002	National	both	5+	5+	4006	4045	
United States of America	2002-2003	Coronary Artery Risk Detection in Appalachian Communities (CARDIAC), 5th Grade	Subnational	both	10-12	10-12	2595	2639	
United States of America	2003-2004	Coronary Artery Risk Detection in Appalachian Communities (CARDIAC), 5th Grade	Subnational	both	10-12	10-12	4062	4582	
United States of America	2003-2004	US NHANES 2003-2004	National	both	5+	5+	3838	3940	
United States of America	2004-2005	Coronary Artery Risk Detection in Appalachian Communities (CARDIAC), 5th Grade	Subnational	both	10-12	10-12	4277	4343	
United States of America	2005-2006	Coronary Artery Risk Detection in Appalachian Communities (CARDIAC), 2nd Grade	Subnational	both	7-9	7-9	270	327	
United States of America	2005-2006	Coronary Artery Risk Detection in Appalachian Communities (CARDIAC), 5th Grade	Subnational	both	10-12	10-12	4206	4859	
United States of America	2005-2006	US NHANES 2005-2006	National	both	5+	5+	3835	3985	
United States of America	2005-2006	National Social Life Health and Aging Project	National	both	57-85	57-85	1435	1355	9
United States of America	2006-2007	Coronary Artery Risk Detection in Appalachian Communities (CARDIAC), 2nd Grade	Subnational	both	7-9	7-9	799	845	
United States of America	2006-2007	Coronary Artery Risk Detection in Appalachian Communities (CARDIAC), 5th Grade	Subnational	both	10-12	10-12	3420	4041	
United States of America	2007-2008	Coronary Artery Risk Detection in Appalachian Communities (CARDIAC), 2nd Grade	Subnational	both	7-9	7-9	3976	3873	
United States of America	2007-2008	Coronary Artery Risk Detection in Appalachian Communities (CARDIAC), 5th Grade	Subnational	both	10-12	10-12	3635	4049	
United States of America	2007-2008	US NHANES 2007-2008	National	both	5+	5+	4038	4086	
United States of America	2008-2009	Coronary Artery Risk Detection in Appalachian Communities (CARDIAC), 2nd Grade	Subnational	both	7-9	7-9	5180	4910	
United States of America	2008-2009	Coronary Artery Risk Detection in Appalachian Communities (CARDIAC), 5th Grade	Subnational	both	10-12	10-12	3594	4152	
United States of America	2008-2009	National Longitudinal Study of Adolescent Health Wave IV	National	both	24-34	24-34	2725	2317	7
United States of America	2009-2010	Coronary Artery Risk Detection in Appalachian Communities (CARDIAC), 2nd Grade	Subnational	both	7-9	7-9	5566	5221	
United States of America	2009-2010	Coronary Artery Risk Detection in Appalachian Communities (CARDIAC), 5th Grade	Subnational	both	10-12	10-12	3513	3990	
United States of America	2009-2010	US NHANES 2009-2010	National	both	5+	5+	4332	4291	
United States of America	2010-2011	Coronary Artery Risk Detection in Appalachian Communities (CARDIAC), 2nd Grade	Subnational	both	7-9	7-9	5289	4994	
United States of America	2010-2011	Coronary Artery Risk Detection in Appalachian Communities (CARDIAC), 5th Grade	Subnational	both	10-12	10-12	2819	3313	
United States of America	2010-2011	National Social Life Health and Aging Project	National	both	36-99	36-99	1738	1452	9
United States of America	2011-2012	Coronary Artery Risk Detection in Appalachian Communities (CARDIAC), 2nd Grade	Subnational	both	7-9	7-9	4415	4124	
United States of America	2011-2012	Coronary Artery Risk Detection in Appalachian Communities (CARDIAC), 5th Grade	Subnational	both	10-12	10-12	2112	2644	
United States of America	2011-2012	US NHANES 2011-2012	National	both	5+	5+	3888	3952	
United States of America	2012-2013	Coronary Artery Risk Detection in Appalachian Communities (CARDIAC), 2nd Grade	Subnational	both	7-9	7-9	5190	4568	
United States of America	2012-2013	Coronary Artery Risk Detection in Appalachian Communities (CARDIAC), 5th Grade	Subnational	both	10-12	10-12	2332	2796	
United States of America	2013-2014	Coronary Artery Risk Detection in Appalachian Communities (CARDIAC), 2nd Grade	Subnational	both	7-9	7-9	5420	4998	
United States of America	2013-2014	Coronary Artery Risk Detection in Appalachian Communities (CARDIAC), 5th Grade	Subnational	both	10-12	10-12	1987	2408	
United States of America	2013-2014	US NHANES 2013-2014	National	both	5+	5+	4226	4105	
United States of America	2014-2015	Coronary Artery Risk Detection in Appalachian Communities (CARDIAC), 2nd Grade	Subnational	both	7-9	7-9	5497	5184	
United States of America	2014-2015	Coronary Artery Risk Detection in Appalachian Communities (CARDIAC), 5th Grade	Subnational	both	10-12	10-12	1889	2286	
Uruguay	1999-2000	The Survey on Health, Well-Being, and Aging in Latin America and the	Community	urban	60+	60+	828	492	2
Uruguay	2006	STEPS	National	both	25-64	25-64	641	261	
Uruguay	2011-2012	CESCAS Study	Community	urban	35-74	35-74	927	650	
Uruguay	2012	Global School-based Student Health Survey	National	both	13-15	13-15	1377	1205	
Uruguay	2012-2016	Genotype, Phenotype and Environment of Hypertension in Uruguay (GEFA-HT-UY)	Community	urban	19+	19+	187	124	
Uzbekistan	1996	DHS	National	both	15-49		4082		
Uzbekistan	2002	DHS	National	both	15-49	15-59	5275	2331	
Uzbekistan	2014	STEPS	National	both	18-64	18-64	2163	1533	
Vanuatu	1996	Second National Nutrition Survey	National	both	20-50		1353		
Vanuatu	1998	Vanuatu Non-comunicable Disease Survey	National	both	20-60	20-60	730	533	
Vanuatu	2005	STEPS	Subnational	both	15-60	15-60	759	626	
Vanuatu	2011	Global School-based Student Health Survey	National	both	13	13	102	67	
Vanuatu	2011	STEPS	National	both	25-64	25-64	2181	2251	
Venezuela	1999-2001	Florez et al., Diabetes Res Clin Pract 69(1):63-77, 2005	Subnational	both	15+	15+	2599	1134	
Venezuela	2000	Diaz et al., Invest Clin 46(2):111-19, 2005	Community	urban	60+	60+	59	42	
Venezuela	2004-2005	CArdiovascular Risk factors Multiple Evaluation in Latin America	Community	urban	25-64	25-64	1123	713	
Venezuela	2005-2006	Brajkovich et al., Rev Ven Endoc Metab 4(3):31-32, 2006	Community	urban	20-65	20-65	439	205	
Venezuela	2007-2008	Venezuelan Study of Metabolic Syndrome, Obesity and Lifestyle (VEMSOLS)	Community	urban	20+	20+	230	107	
Venezuela	2008-2009	Venezuelan Study of Metabolic Syndrome, Obesity and Lifestyle (VEMSOLS)	Community	rural	20+	20+	89	51	
Venezuela	2010-2011	Venezuelan Study of Metabolic Syndrome, Obesity and Lifestyle (VEMSOLS)	Community	urban	20+	20+	193	66	
Venezuela	2015-2017	Cardio-Metabolic Health Venezuelan Study (EVESCAM)	National	both	20+	20+	2346	1056	
Viet Nam	1981-1985	National Nutrition Survey	Subnational	rural	18+	18+	7985	4815	
Viet Nam	1987-1989	General Nutrition Survey	National	both	15-70	15-70	19574	16012	
Viet Nam	1992-1993	Living Standard Survey	National	both	5+	5+	10206	9417	
Viet Nam	1997-1998	Living Standard Survey	National	both	5+	5+	13123	12057	
Viet Nam	2000	National Nutrition Survey	National	both	20+	20+	9464	8985	

Country	Data years	Survey/study name/citation	Level of representative-ness	Rural, urban, or both	Age range as used for global analysis		Sample size		Note
					Female	Male	Female	Male	
Viet Nam	2001-2002	Viet Nam National Health Survey 2001-2002	National	both	5+	5+	71614	66722	
Viet Nam	2004	Cuong et al., Eur J Clin Nutr 61(5):673-81, 2007	Community	urban	20-60	20-60	771	717	
Viet Nam	2005	STEPS Bavi district	Subnational	rural	25-64	25-64	997	987	
Viet Nam	2005	National Adult Overweight Survey	National	both	25-64	25-64	8725	8474	
Viet Nam	2005	Non-communicable disease risk factors in Ho Chi Minh City	Community	urban	25-64	25-64	1063	908	
Viet Nam	2006	Qualitative and quantitative assessment of nutritional status and lifestyles of Vietnamese adolescents, rura	Subnational	rural	15-19	15-19	363	252	
Viet Nam	2006	Qualitative and quantitative assessment of nutritional status and lifestyles of Vietnamese adolescents, urbar	Subnational	urban	15-19	15-19	334	254	
Viet Nam	2008-2009	The survey on diabetes and its risk factors in 2 northern provinces of Vietnam (DM-S)	Subnational	both	25+	25+	1446	830	
Viet Nam	2009-2010	Vietnam National Nutrition Survey 2009-2010	National	both	5-19	5-19	5151	5221	
Viet Nam	2009	STEPS	National	both	25-64	25-64	7804	6738	
Viet Nam	2009-2010	General Nutrition Survey	National	both	20+	20+	11729	10810	
Viet Nam	2011	SEANUTS	National	both	5-11	5-11	980	974	
Viet Nam	2012	National Survey of Diabetes in Vietnam	National	both	30-69	30-69	5858	5324	
Viet Nam	2013	Global School-based Student Health Survey	National	both	13-17	13-17	1578	1368	
Viet Nam	2015	STEPS	National	both	18-69	18-69	1722	1316	
Yemen	1997	DHS	National	both	20-49		5123		
Yemen	2005-2006	Yemen Household Budget Survey 2005-2006	National	both	5+	5+	3306	3287	
Yemen	2007-2009	Hypertension and Diabetes in Yemen (HYDY)	National	rural	6-70	6-70	3065	3023	
Yemen	2007-2009	Hypertension and Diabetes in Yemen (HYDY)	National	urban	6-70	6-70	3077	2996	
Yemen	2013	DHS	National	both	15-49		22530		
Yemen	2014	Global School-based Student Health Survey	National	both	13	13-17	175	832	
Zambia	1992	DHS	National	both	20-49		2829		
Zambia	1996	DHS	National	both	20-49		3485		
Zambia	2001-2002	DHS	National	both	15-49		6732		
Zambia	2003	Kelly et al., Am J Clin Nut 88(4):1010-17, 2008	Community	urban	25-84	25-74	217	132	
Zambia	2007	DHS	National	both	15-49		6381		
Zambia	2008	STEPS	Community	urban	25+	25+	1214	626	
Zambia	2013-2014	DHS	National	both	15-49		14837		
Zimbabwe	1985-1986	INTERSALT	Community	urban	20-59	20-59	95	100	
Zimbabwe	1991	Zinyowera et al., Cent Afr J Med 40(2):33-8, 1994	Community	both	18+	18+	734	775	
Zimbabwe	1994	DHS	National	both	20-49		1776		
Zimbabwe	1995	Mufunda et al., J Hum Hypertens 14(1):65-73, 2000	Community	urban	25+	25+	391	384	
Zimbabwe	1999	DHS	National	both	15-49		5169		
Zimbabwe	2005-2006	DHS	National	both	15-49		8186		
Zimbabwe	2005	STEPS	National	both	25-64	25-64	1556	431	
Zimbabwe	2010-2011	DHS	National	both	15-49	15-54	8329	7383	
Zimbabwe	2015	DHS	National	both	15-49	15-54	9396	8386	

1. National studies for the 3 years prior to 1975 were assigned to 1975 so that they can inform the estimates in countries with older national data.

2. The bibliographic citation for this data source is: Pelaez, Martha, Alberto Palloni, Cecilia Albala, Juan C. Alfonso, Roberto Ham-Chande, Anselm Hennis, Maria Lucia Lebrao, Esther Lesn-Diaz, Edith Pantelides, and Omar Prats. SABE - SURVEY ON HEALTH, WELL-BEING, AND AGING IN LATIN AMERICA AND THE CARIBBEAN, 2000 [Computer file]. ICPSR version. Washington, D.C.: Pan American Health Organization/World Health Organization (PAHO/WHO) [producers], 2004. Ann Arbor, MI: Inter-university Consortium for Political and Social Research [distributor], 2005.

3. This research uses data from China Health and Nutrition Survey (CHNS). We thank the National Institute of Nutrition and Food Safety, China Center for Disease Control and Prevention, Carolina Population Center (5 R24 HD050924), the University of North Carolina at Chapel Hill, the NIH (R01-HD30880, DK056350, R24HD050924, and R01-HD38700) and the Fogarty International Center, NIH for financial support for the CHNS data collection and analysis files from 1989 to 2011 and future surveys, and the China -Japan Friendship Hospital, Ministry of Health for support for CHNS 2009.

4. The Longitudinal Aging Study Amsterdam is supported by a grant from the Netherlands Ministry of Health Welfare and Sports, Directorate of Long-Term Care.

5. The bibliographic citation for this data source is: Palloni, Alberto, Ana Luisa Davila, and Melba Sanchez-Ayendez. Puerto Rican Elderly: Health Conditions (PREHCO) Project, 2002-2003, 2006-2007. ICPSR34596-v1. Ann Arbor, MI: Inter-university Consortium for Political and Social Research[distributor], 2013-09-13. doi:10.3886/ICPSR34596.v1

6. We thank Prof Stephen Fortmann for data from the Stanford Five-City Project.

7. This research uses data from Add Health, a program project designed by J. Richard Udry, Peter S. Bearman, and Kathleen Mullan Harris, and funded by a grant P01-HD31921 from the Eunice Kennedy Shriver National Institute of Child Health and Human Development, with cooperative funding from 17 other agencies. Special acknowledgment is due Ronald R. Rindfuss and Barbara Entwisle for assistance in the original design. Persons interested in obtaining data files from Add Health should contact Add Health, Carolina Population Center, 123 W. Franklin Street, Chapel Hill, NC 27516-2524 (addhealth@unc.edu). No direct support was received from grant P01-HD31921 for this analysis.

8. The bibliographic citation for this data source is: Sutton-Tyrrell, Kim, Faith Selzer, MaryFran Sowers, Robert Neer, Lynda Powell, Ellen Gold, Gail Greendale, Gerson Weiss, Karen Matthews, and Sonja McKinlay. Study of Women's Health Across the Nation (SWAN), 1996-1997: Baseline Dataset. ICPSR28762-v2. Ann Arbor, MI: Inter-university Consortium for Political and Social Research[distributor], 2014-02-04. http://doi.org/10.3886/ICPSR28762.v2

9. The bibliographic citation for this data source is: Waite, Linda J., Kathleen Cagney, William Dale, Elbert Huang, Edward O. Laumann, Martha McClintock, Colm A. O'Muircheartaigh, L. Phillip Schumm, and Benjamin Cornwell. National Social Life, Health, and Aging Project (NSHAP): Wave 2 and Partner Data Collection. ICPSR34921-v1. Ann Arbor, MI: Inter-university Consortium for Political and Social Research [distributor], 2014-04-29. https://doi.org/10.3886/ICPSR34921.v1

10. We thank Jelena Jakovljevic, Henrique Loyola, Harry Rutter and Joop van Raaij for data from the COSI Project.

Appendix Table 3: Number of data sources and number of participants used in the analysis by age.

	Male		Female	
**Age group (years)**	**Number of data sources**	**Number of participants**	**Number of data sources**	**Number of participants**
5	299 (13.8)	1,824,144 (2.7)	299 (13.6)	1,752,448 (2.8)
6	368 (17.0)	677,023 (1.0)	367 (16.6)	661,232 (1.1)
7	409 (18.9)	679,952 (1.0)	409 (18.5)	653,677 (1.1)
8	431 (19.9)	623,187 (0.9)	429 (19.4)	610,081 (1.0)
9	443 (20.5)	528,093 (0.8)	443 (20.1)	515,329 (0.8)
10	464 (21.4)	1,912,376 (2.8)	466 (21.1)	1,836,778 (3.0)
11	434 (20.0)	1,754,945 (2.6)	441 (20.0)	1,679,900 (2.7)
12	452 (20.9)	640,951 (1.0)	456 (20.7)	639,203 (1.0)
13	509 (23.5)	650,785 (1.0)	513 (23.3)	654,615 (1.1)
14	466 (21.5)	665,152 (1.0)	479 (21.7)	676,662 (1.1)
15	589 (27.2)	562,233 (0.8)	711 (32.2)	625,959 (1.0)
16	569 (26.3)	443,321 (0.7)	698 (31.6)	496,043 (0.8)
17	596 (27.5)	1,846,765 (2.7)	653 (29.6)	459,382 (0.7)
18	644 (29.7)	1,632,467 (2.4)	697 (31.6)	251,215 (0.4)
19	614 (28.4)	5,353,528 (8.0)	646 (29.3)	146,899 (0.2)
20-29	1,053 (48.6)	1,643,433 (2.4)	1,218 (55.2)	1,974,427 (3.2)
30-39	1,184 (54.7)	1,603,352 (2.4)	1,407 (63.8)	2,236,492 (3.6)
40-49	1,285 (59.4)	16,719,643 (24.9)	1,493 (67.7)	15,749,804 (25.5)
50-59	1,325 (61.2)	13,712,232 (20.4)	1,285 (58.3)	14,328,800 (23.2)
60-69	1,253 (57.9)	8,639,107 (12.9)	1,226 (55.6)	9,548,063 (15.5)
70-79	805 (37.2)	4,256,697 (6.3)	780 (35.4)	5,120,258 (8.3)
80+	649 (30.0)	790,746 (1.2)	622 (28.2)	1,102,015 (1.8)

Appendix Table 4: Model specifications and regression coefficients to estimate the primary outcomes (mean BMI or prevalence of various BMI categories) from other metrics for children and adolescents (aged 5-19 years).

The dependent variable in all regressions was the inverse of the mean or a prevalence, fitted using a linear (mixed) model for mean and a generalised linear (mixed) model fitted with a probit link function for prevalence. Random effects for regions in regression are presented after the table of coefficients.

* denotes statistical interaction. CI: confidence interval.

**Dependent variable: Inverse mean BMI**	
**Independent variable: Prevalence (BMI ≥25 kg/m^2)**	
**Variables**	**Coefficients (95% CI)**
Intercept	0.055 (0.054, 0.056)
Probit-transformed prevalence (BMI ≥25 kg/m^2)	-0.0066 (-0.0069, -0.0064)
Mean age of age group	-0.00072 (-0.00075, -0.00069)
Male sex	0.00053 (0.00035, 0.00072)
Study mid-year (per one more recent year since 1975)	-3.2e-05 (-3.7e-05, -2.7e-05)
Natural logarithm of per-capita gross domestic product	-6.3e-05 (-0.00013, 6.8e-06)
Probit-transformed prevalence (BMI ≥25 kg/m^2) * mean age of age group	0.00015 (0.00013, 0.00016)
Probit-transformed prevalence (BMI ≥25 kg/m^2) * male sex	8.4e-05 (-2.7e-05, 0.00020)
Random effects for regions	Yes
**Number of data points used to fit the model = 7,729**	

Traditional R^2 is not clearly defined for mixed-effect models. The conditional R^2 for the model, which describes the proportion of variance explained by both fixed and random factors,[11] was 0.944.

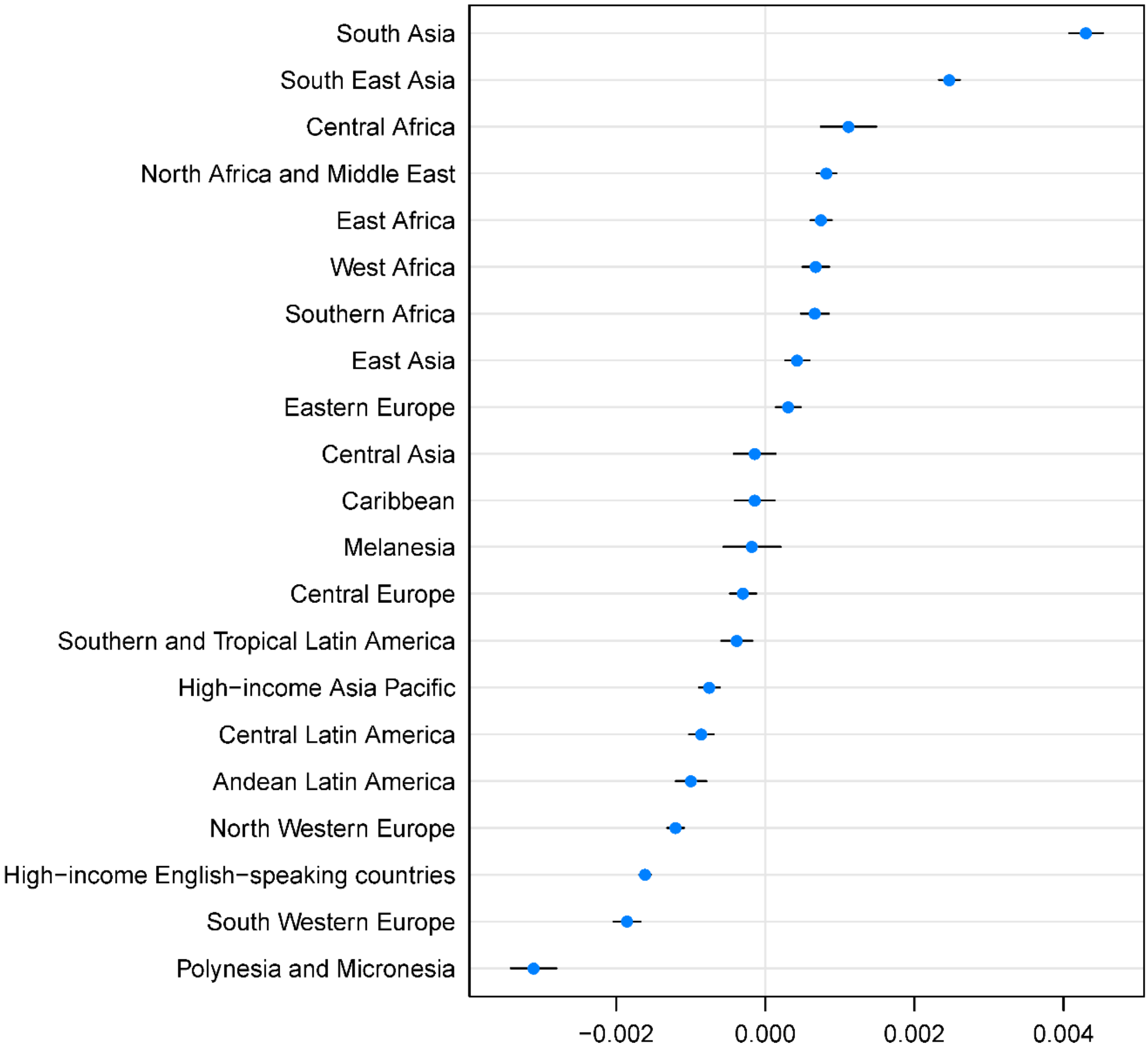

South Asia
South East Asia
Central Africa
North Africa and Middle East
East Africa
West Africa
Southern Africa
East Asia
Eastern Europe
Central Asia
Caribbean
Melanesia
Central Europe
Southern and Tropical Latin America
High−income Asia Pacific
Central Latin America
Andean Latin America
North Western Europe
High−income English−speaking countries
South Western Europe
Polynesia and Micronesia
−0.002
0.000
0.002
0.004

**Dependent variable: Inverse mean BMI**	
**Independent variable: Prevalence (BMI ≥30 kg/m^2)**	
**Variables**	**Coefficients (95% CI)**
Intercept	0.056 (0.055, 0.058)
Probit-transformed prevalence (BMI ≥30 kg/m^2)	-0.0062 (-0.0066, -0.0059)
Mean age of age group	-0.00070 (-0.00075, -0.00065)
Male sex	0.00042 (5.6e-05, 0.00078)
Study mid-year (per one more recent year since 1975)	-5.6e-05 (-6.3e-05, -4.9e-05)
Natural logarithm of per-capita gross domestic product	-0.00026 (-0.00035, -0.00016)
Probit-transformed prevalence (BMI ≥30 kg/m^2) * mean age of age group	0.00020 (0.00018, 0.00023)
Probit-transformed prevalence (BMI ≥30 kg/m^2) * male sex	-0.00012 (-0.00029, 5.2e-05)
Random effects for regions	Yes
**Number of data points used to fit the model = 5,745**	

Traditional R^2 is not clearly defined for mixed-effect models. The conditional R^2 for the model, which describes the proportion of variance explained by both fixed and random factors,[11] was 0.916.

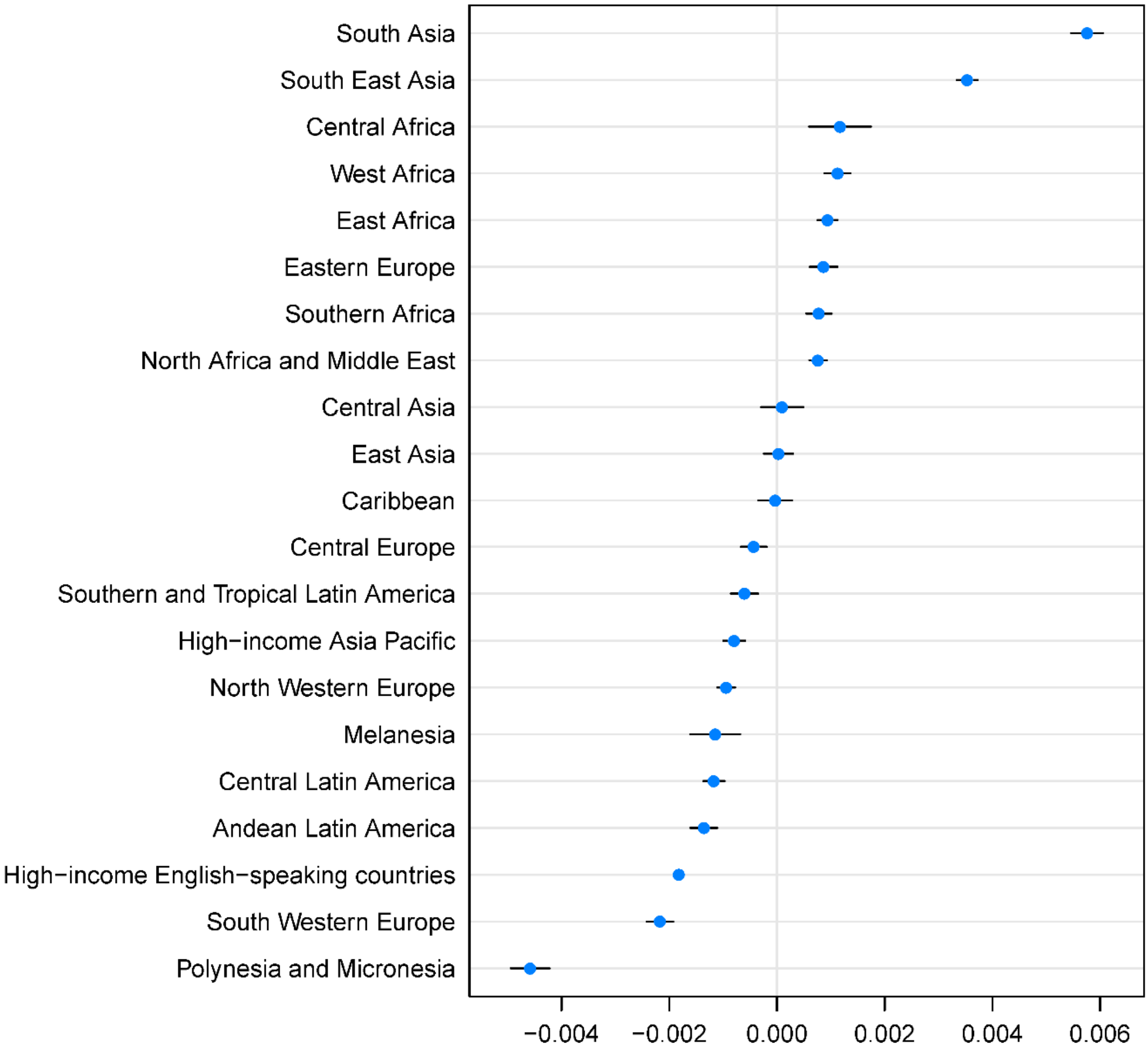
South Asia
South East Asia
Central Africa
West Africa
East Africa
Eastern Europe
Southern Africa
North Africa and Middle East
Central Asia
East Asia
Caribbean
Central Europe
Southern and Tropical Latin America
High−income Asia Pacific
North Western Europe
Melanesia
Central Latin America
Andean Latin America
High−income English−speaking countries
South Western Europe
Polynesia and Micronesia
−0.004
−0.002
0.000
0.002
0.004
0.006

Dependent variable: Inverse mean BMI	
**Independent variable: Prevalence (BMI ≥25 kg/m^2) and prevalence (BMI ≥30 kg/m^2)**	
**Variables**	**Coefficients (95% CI)**
Intercept	0.051 (0.050, 0.053)
Probit-transformed prevalence (BMI ≥25 kg/m^2)	-0.011 (-0.012, -0.011)
Probit-transformed prevalence (BMI ≥30 kg/m^2)	0.0033 (0.0027, 0.0038)
Mean age of age group	-0.00072 (-0.00077, -0.00068)
Male sex	-0.00028 (-0.00059, 2.7e-05)
Study mid-year (per one more recent year since 1975)	-2.4e-05 (-2.9e-05, -1.8e-05)
Natural logarithm of per-capita gross domestic product	0.00016 (8.8e-05, 0.00023)
Probit-transformed prevalence (BMI ≥25 kg/m^2) * mean age of age group	0.00043 (0.00040, 0.00047)
Probit-transformed prevalence (BMI ≥30 kg/m^2) * mean age of age group	-0.00024 (-0.00028, -0.00020)
Probit-transformed prevalence (BMI ≥25 kg/m^2) * male sex	0.00043 (0.00016, 0.00069)
Probit-transformed prevalence (BMI ≥30 kg/m^2) * male sex	-0.00060 (-0.00088, -0.00033)
Random effects for regions	Yes
**Number of data points used to fit the model = 5,615**	

Traditional R^2 is not clearly defined for mixed-effect models. The conditional R^2 for the model, which describes the proportion of variance explained by both fixed and random factors,[11] was 0.951.

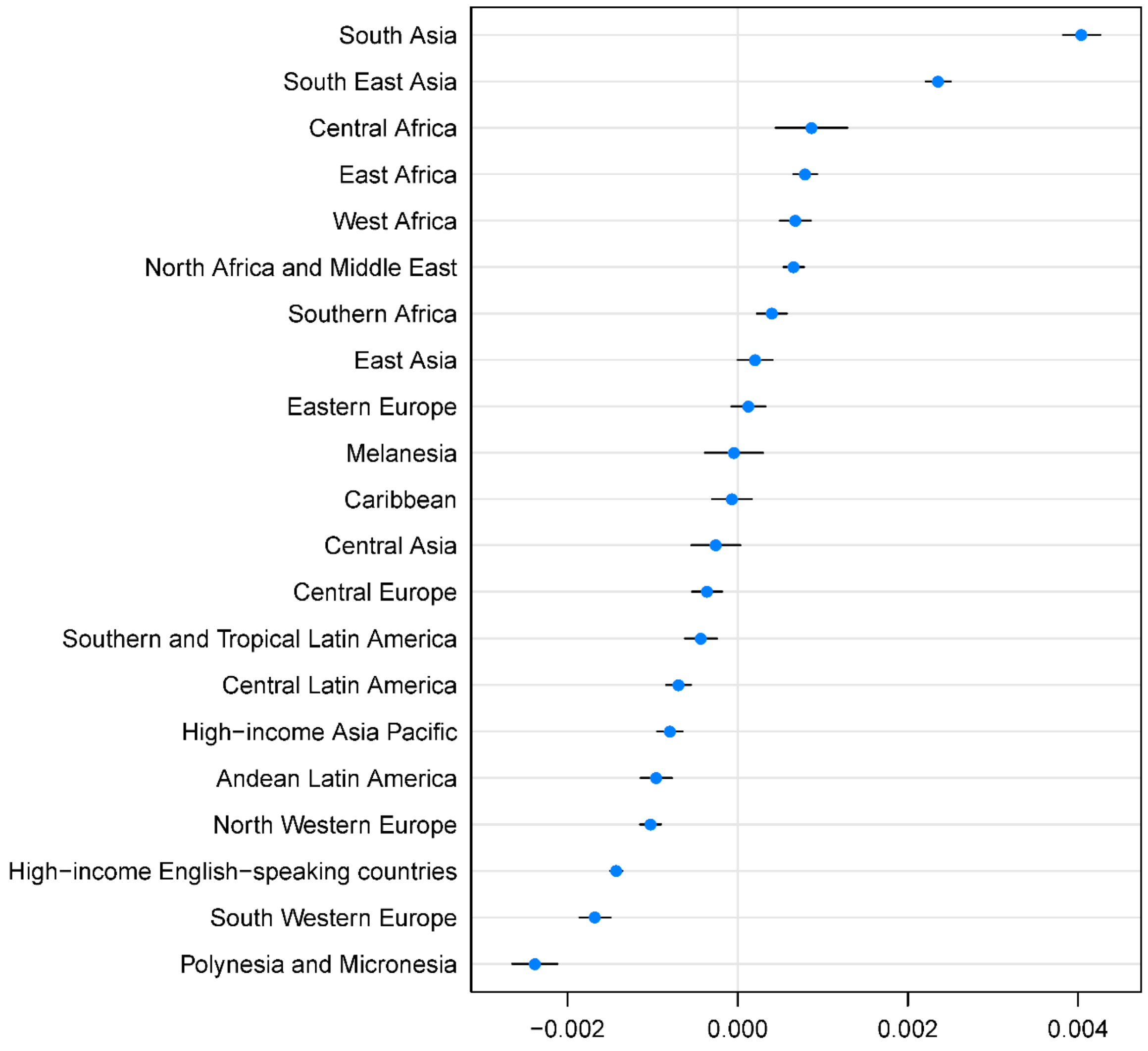
South Asia
South East Asia
Central Africa
East Africa
West Africa
North Africa and Middle East
Southern Africa
East Asia
Eastern Europe
Melanesia
Caribbean
Central Asia
Central Europe
Southern and Tropical Latin America
Central Latin America
High−income Asia Pacific
Andean Latin America
North Western Europe
High−income English−speaking countries
South Western Europe
Polynesia and Micronesia
−0.002
0.000
0.002
0.004

Dependent variable: Inverse mean BMI	
**Independent variable: Prevalence (BMI > +1SD), prevalence (BMI > +2SD) and prevalence (BMI < -1SD)**	
**Variables**	**Coefficients (95% CI)**
Intercept	0.073 (0.072, 0.073)
Probit-transformed prevalence (BMI > +1SD)	-0.0041 (-0.0046, -0.0036)
Probit-transformed prevalence (BMI > +2SD)	-0.0013 (-0.0017, -0.00090)
Probit-transformed prevalence (BMI < -1SD)	0.0032 (0.0029, 0.0034)
Mean age of age group	-0.0017 (-0.0018, -0.0017)
Male sex	0.00065 (0.00036, 0.00094)
Study mid-year (per one more recent year since 1975)	-6.8e-07 (-4.0e-06, 2.6e-06)
Natural logarithm of per-capita gross domestic product	-0.00020 (-0.00026, -0.00015)
Probit-transformed prevalence (BMI > +1SD) * mean age of age group	-2.4e-05 (-5.6e-05, 9.2e-06)
Probit-transformed prevalence (BMI > +2SD) * mean age of age group	3.6e-05 (8.7e-06, 6.2e-05)
Probit-transformed prevalence (BMI < -1SD) * mean age of age group	-0.00010 (-0.00012, -8.3e-05)
Probit-transformed prevalence (BMI > +1SD) * male sex	0.0010 (0.00076, 0.0013)
Probit-transformed prevalence (BMI > +2SD) * male sex	-0.00073 (-0.00095, -0.00051)
Probit-transformed prevalence (BMI < -1SD) * male sex	0.00040 (0.00026, 0.00055)
Random effects for regions	Yes
**Number of data points used to fit the model = 10,671**	

Traditional R^2 is not clearly defined for mixed-effect models. The conditional R^2 for the model, which describes the proportion of variance explained by both fixed and random factors,[11] was 0.967.

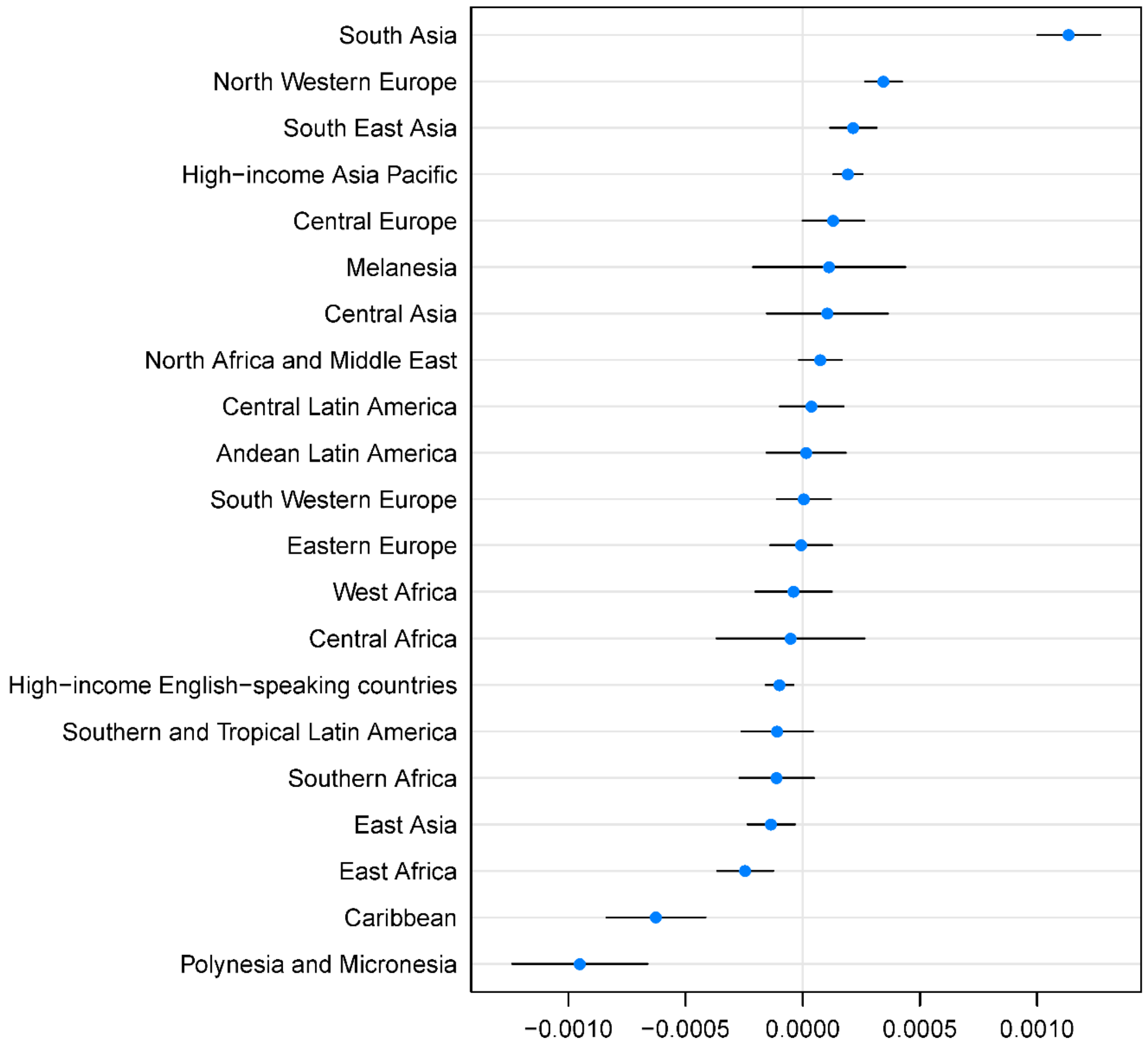
South Asia
North Western Europe
South East Asia
High−income Asia Pacific
Central Europe
Melanesia
Central Asia
North Africa and Middle East
Central Latin America
Andean Latin America
South Western Europe
Eastern Europe
West Africa
Central Africa
High−income English−speaking countries
Southern and Tropical Latin America
Southern Africa
East Asia
East Africa
Caribbean
Polynesia and Micronesia
−0.0010
−0.0005
0.0000
0.0005
0.0010

**Dependent variable: Inverse mean BMI**	
**Independent variable: Prevalence (BMI > +1SD), prevalence (BMI > +2SD) and prevalence (BMI < -2SD)**	
**Variables**	**Coefficients (95% CI)**
Intercept	0.072 (0.072, 0.073)
Probit-transformed prevalence (BMI > +1SD)	-0.0052 (-0.0057, -0.0047)
Probit-transformed prevalence (BMI > +2SD)	-0.0011 (-0.0016, -0.00064)
Probit-transformed prevalence (BMI < -2SD)	0.0024 (0.0022, 0.0026)
Mean age of age group	-0.0018 (-0.0018, -0.0018)
Male sex	0.0011 (0.00072, 0.0014)
Study mid-year (per one more recent year since 1975)	-1.3e-06 (-5.0e-06, 2.3e-06)
Natural logarithm of per-capita gross domestic product	-0.00010 (-0.00016, -4.1e-05)
Probit-transformed prevalence (BMI > +1SD) * mean age of age group	-1.6e-05 (-5.1e-05, 2.0e-05)
Probit-transformed prevalence (BMI > +2SD) * mean age of age group	5.1e-05 (1.9e-05, 8.2e-05)
Probit-transformed prevalence (BMI < -2SD) * mean age of age group	-0.00011 (-0.00012, -8.9e-05)
Probit-transformed prevalence (BMI > +1SD) * male sex	0.0013 (0.00099, 0.0016)
Probit-transformed prevalence (BMI > +2SD) * male sex	-0.0010 (-0.0013, -0.00077)
Probit-transformed prevalence (BMI < -2SD) * male sex	0.00056 (0.00042, 0.00070)
Random effects for regions	Yes
**Number of data points used to fit the model = 9,096**	

Traditional R^2 is not clearly defined for mixed-effect models. The conditional R^2 for the model, which describes the proportion of variance explained by both fixed and random factors,[11] was 0.964.

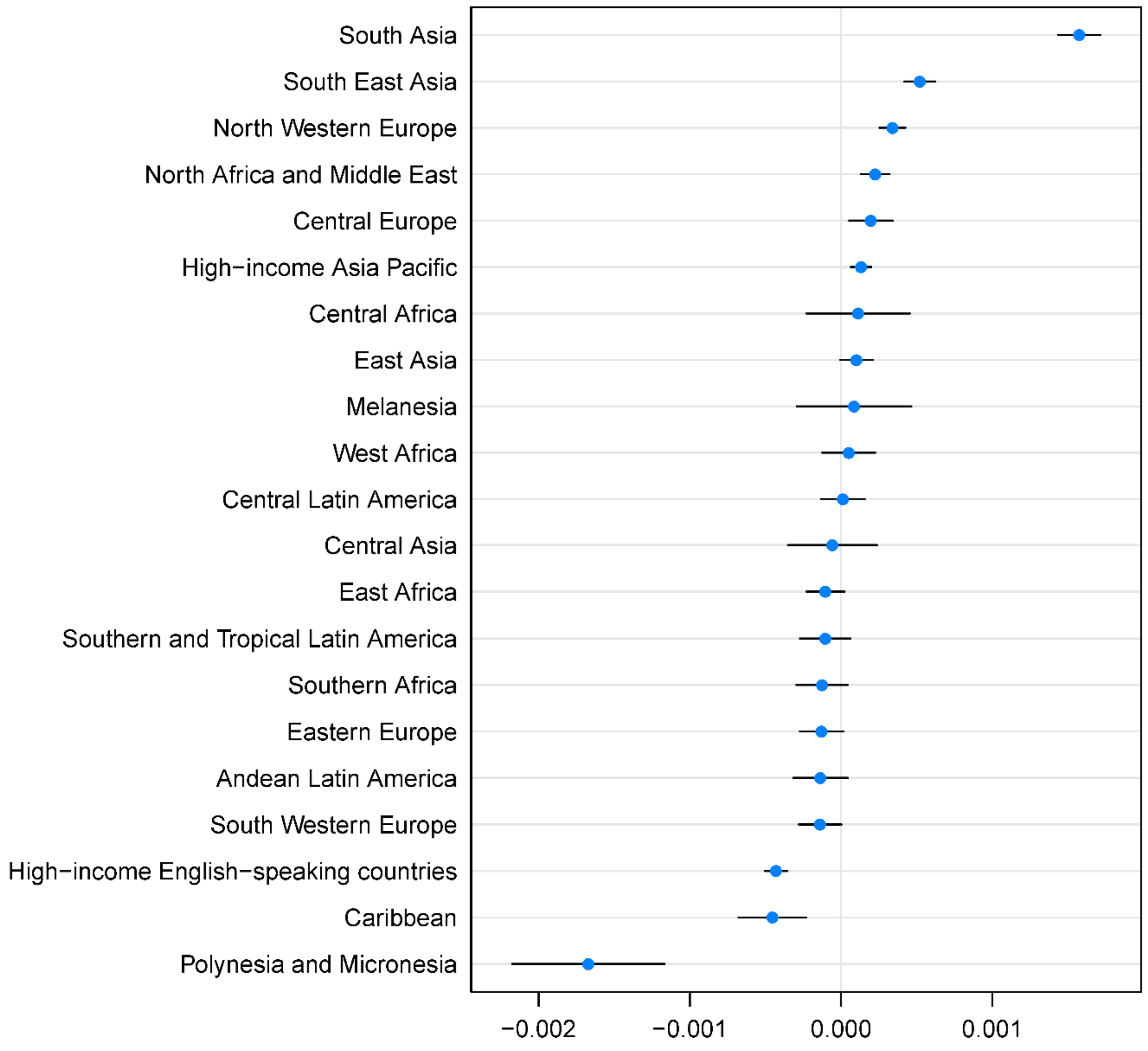
South Asia
South East Asia
North Western Europe
North Africa and Middle East
Central Europe
High−income Asia Pacific
Central Africa
East Asia
Melanesia
West Africa
Central Latin America
Central Asia
East Africa
Southern and Tropical Latin America
Southern Africa
Eastern Europe
Andean Latin America
South Western Europe
High−income English−speaking countries
Caribbean
Polynesia and Micronesia
−0.002
−0.001
0.000
0.001

**Dependent variable: Inverse mean BMI**	
**Independent variable: Prevalence (BMI > +1SD) and prevalence (BMI > +2SD)**	
**Variables**	**Coefficients (95% CI)**
Intercept	0.067 (0.066, 0.068)
Probit-transformed prevalence (BMI > +1SD)	-0.0066 (-0.0070, -0.0062)
Probit-transformed prevalence (BMI > +2SD)	-0.00085 (-0.0013, -0.00044)
Mean age of age group	-0.0016 (-0.0016, -0.0015)
Male sex	0.00020 (-2.7e-05, 0.00042)
Study mid-year (per one more recent year since 1975)	-3.7e-06 (-7.2e-06, -8.0e-08)
Natural logarithm of per-capita gross domestic product	-6.6e-05 (-0.00013, -6.9e-06)
Probit-transformed prevalence (BMI > +1SD) * mean age of age group	9.8e-05 (7.0e-05, 0.00013)
Probit-transformed prevalence (BMI > +2SD) * mean age of age group	1.7e-05 (-1.1e-05, 4.5e-05)
Probit-transformed prevalence (BMI > +1SD) * male sex	0.00043 (0.00019, 0.00067)
Probit-transformed prevalence (BMI > +2SD) * male sex	-0.00059 (-0.00082, -0.00036)
Random effects for regions	Yes
**Number of data points used to fit the model = 10,808**	

Traditional R^2 is not clearly defined for mixed-effect models. The conditional R^2 for the model, which describes the proportion of variance explained by both fixed and random factors,[11] was 0.960.

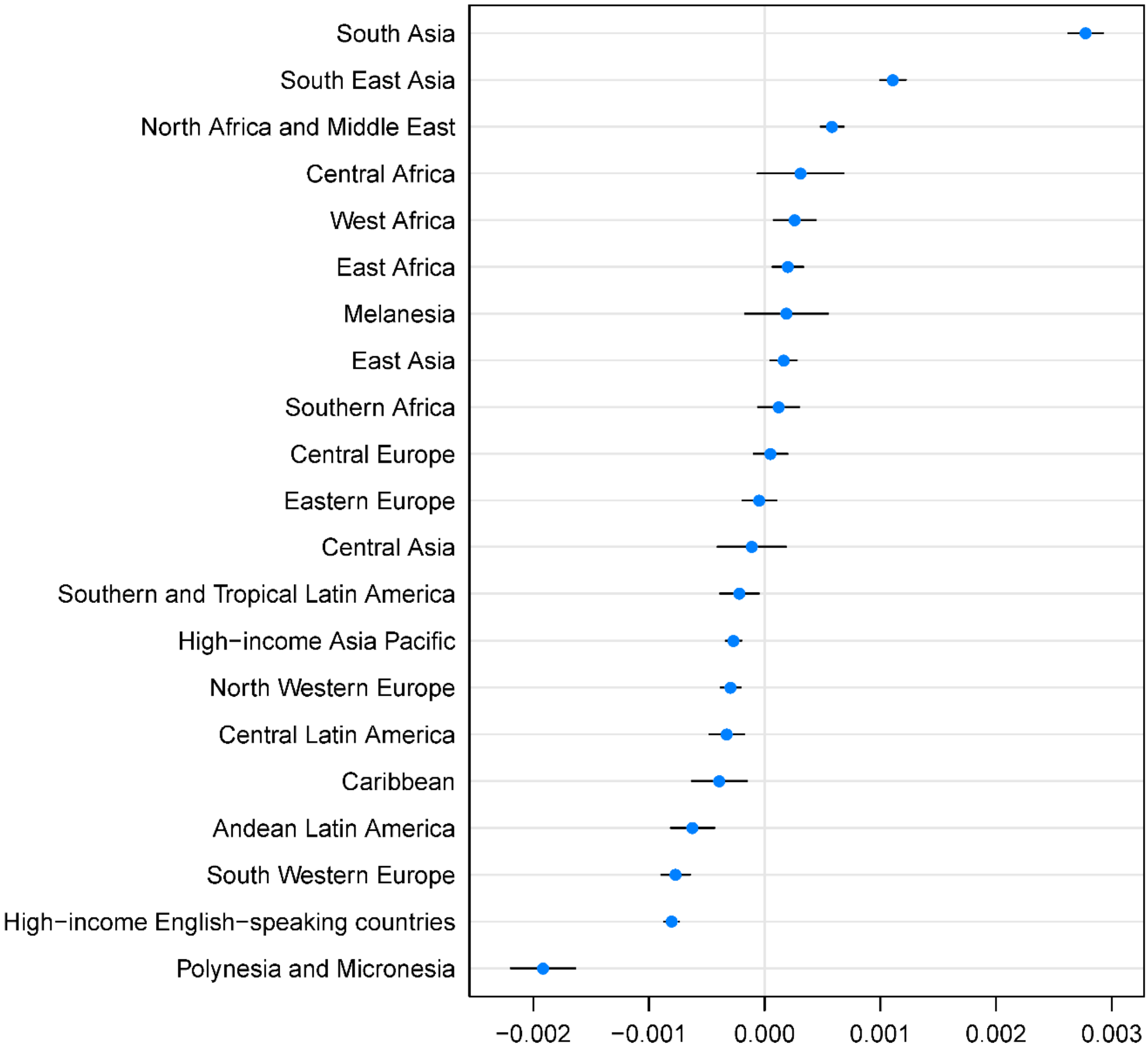
South Asia
South East Asia
North Africa and Middle East
Central Africa
West Africa
East Africa
Melanesia
East Asia
Southern Africa
Central Europe
Eastern Europe
Central Asia
Southern and Tropical Latin America
High−income Asia Pacific
North Western Europe
Central Latin America
Caribbean
Andean Latin America
South Western Europe
High−income English−speaking countries
Polynesia and Micronesia
−0.002
−0.001
0.000
0.001
0.002
0.003

Dependent variable: Inverse mean BMI	
**Independent variable: Prevalence (BMI > +1SD) and prevalence (BMI < -2SD)**	
**Variables**	**Coefficients (95% CI)**
Intercept	0.074 (0.073, 0.074)
Probit-transformed prevalence (BMI > +1SD)	-0.0063 (-0.0066, -0.0061)
Probit-transformed prevalence (BMI < -2SD)	0.0024 (0.0022, 0.0026)
Mean age of age group	-0.0018 (-0.0018, -0.0017)
Male sex	0.0017 (0.0014, 0.0020)
Study mid-year (per one more recent year since 1975)	-8.6e-06 (-1.2e-05, -5.0e-06)
Natural logarithm of per-capita gross domestic product	-0.00015 (-0.00021, -9.8e-05)
Probit-transformed prevalence (BMI > +1SD) * mean age of age group	6.4e-05 (4.8e-05, 8.1e-05)
Probit-transformed prevalence (BMI < -2SD) * mean age of age group	-8.8e-05 (-0.00010, -7.2e-05)
Probit-transformed prevalence (BMI > +1SD) * male sex	0.00038 (0.00025, 0.00051)
Probit-transformed prevalence (BMI < -2SD) * male sex	0.00051 (0.00038, 0.00064)
Random effects for regions	Yes
**Number of data points used to fit the model = 9,928**	

Traditional R^2 is not clearly defined for mixed-effect models. The conditional R^2 for the model, which describes the proportion of variance explained by both fixed and random factors,[11] was 0.960.

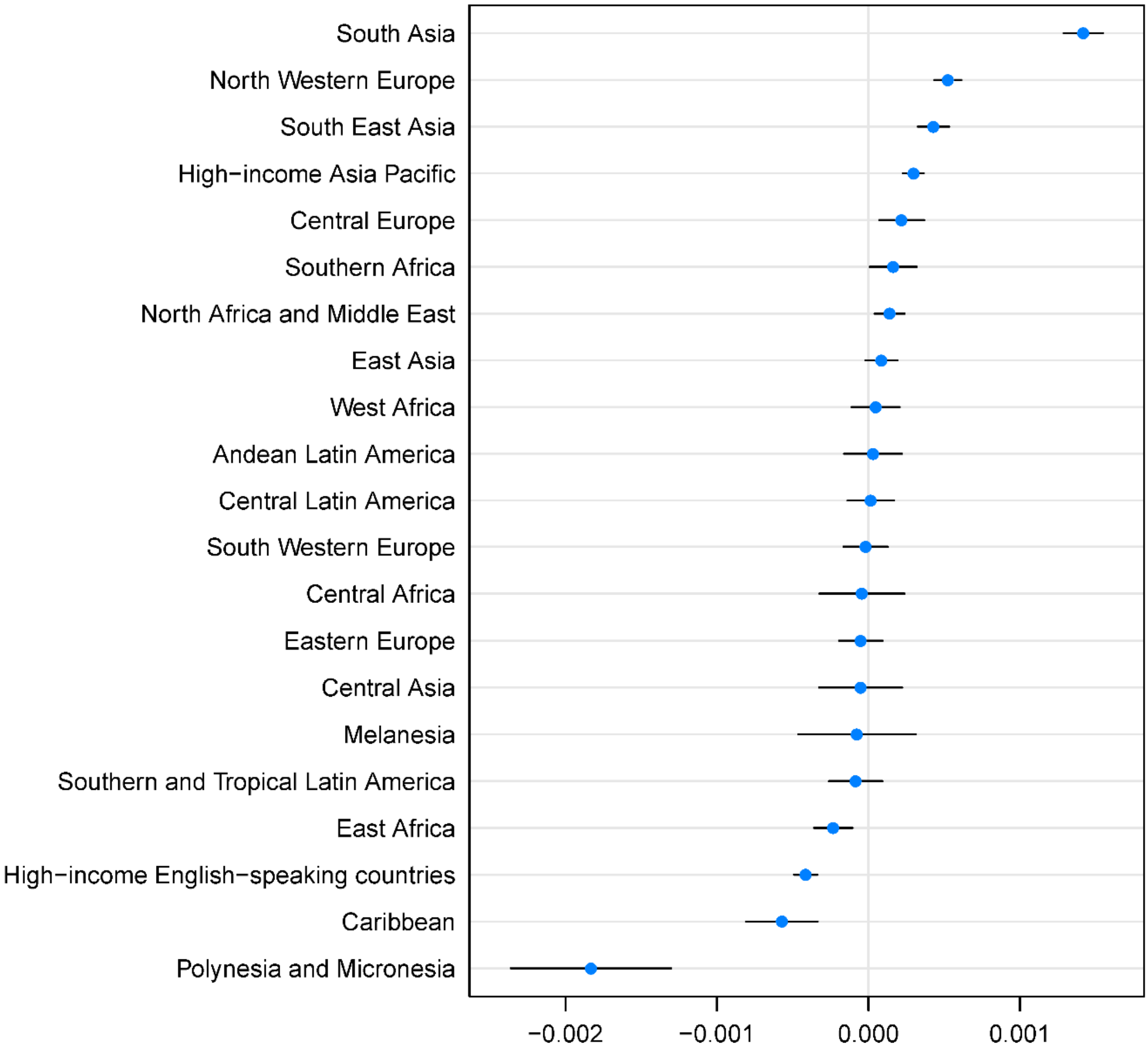
South Asia
North Western Europe
South East Asia
High−income Asia Pacific
Central Europe
Southern Africa
North Africa and Middle East
East Asia
West Africa
Andean Latin America
Central Latin America
South Western Europe
Central Africa
Eastern Europe
Central Asia
Melanesia
Southern and Tropical Latin America
East Africa
High−income English−speaking countries
Caribbean
Polynesia and Micronesia
−0.002
−0.001
0.000
0.001

**Dependent variable: Prevalence (BMI > +1SD)**	
**Independent variable: Inverse mean BMI**	
**Variables**	**Coefficients (95% CI)**
Intercept	6.70 (6.63, 6.77)
Inverse mean BMI	-126.00 (-126.00, -125.00)
Mean age of age group	-0.32 (-0.32, -0.32)
Male sex	0.52 (0.51, 0.53)
Study mid-year (per one more recent year since 1975)	0.0061 (0.0059, 0.0062)
Natural logarithm of per-capita gross domestic product	0.11 (0.11, 0.11)
Inverse mean BMI * mean age of age group	2.85 (2.83, 2.88)
Inverse mean BMI * male sex	-7.14 (-7.34, -6.94)
Random effects for regions	Yes
**Number of data points used to fit the model = 11,888**	

Traditional R^2 is not clearly defined for mixed-effect models. The pseudo-R^2 for the model, which describes the proportion of variance explained by both fixed and random factors,[12] was 0.879.

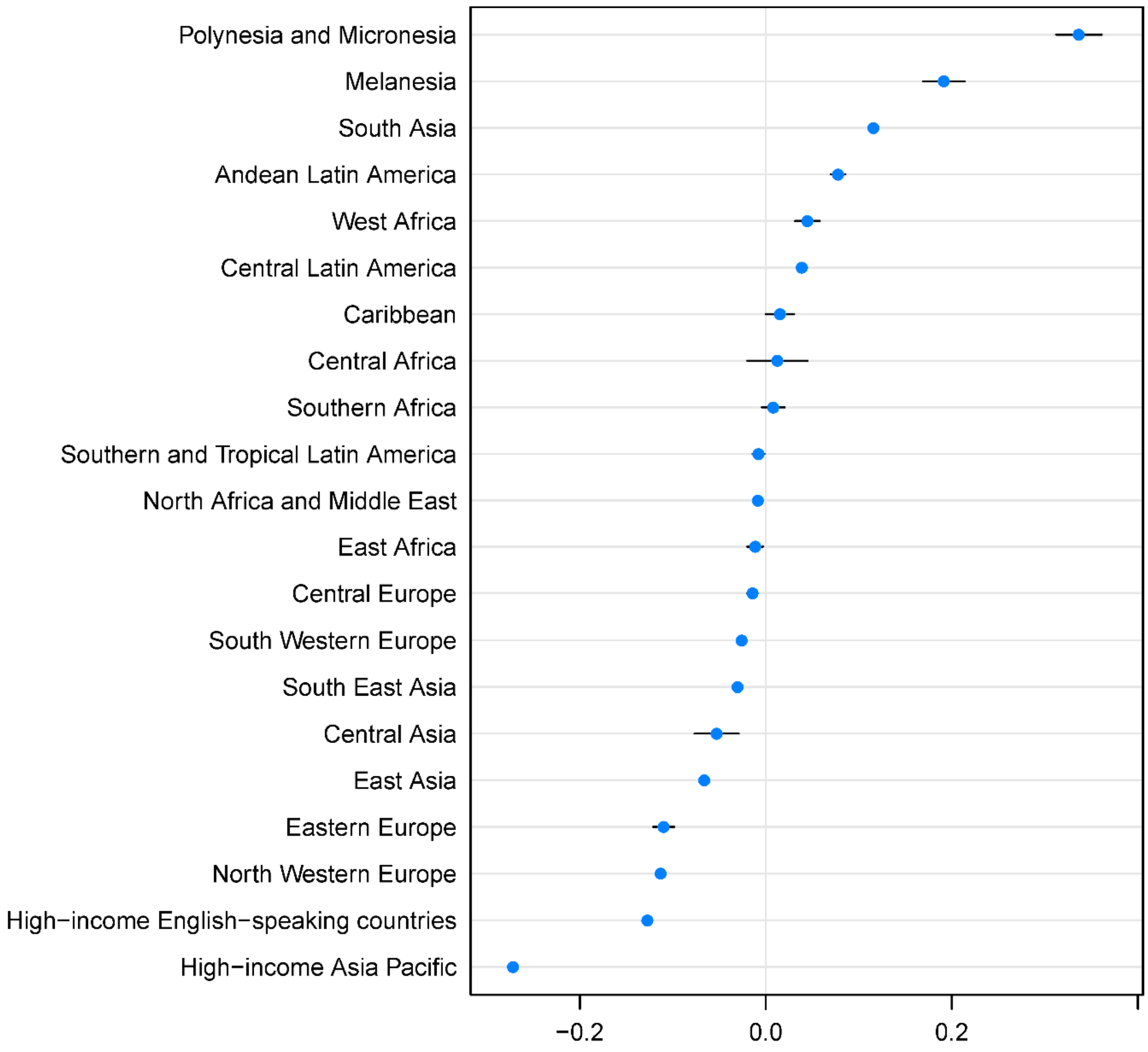

Polynesia and Micronesia
Melanesia
South Asia
Andean Latin America
West Africa
Central Latin America
Caribbean
Central Africa
Southern Africa
Southern and Tropical Latin America
North Africa and Middle East
East Africa
Central Europe
South Western Europe
South East Asia
Central Asia
East Asia
Eastern Europe
North Western Europe
High−income English−speaking countries
High−income Asia Pacific
−0.2
0.0
0.2

Dependent variable: Prevalence (BMI > +1SD)	
**Independent variable: Prevalence (BMI ≥30 kg/m^2)**	
**Variables**	**Coefficients (95% CI)**
Intercept	0.37 (0.27, 0.47)
Probit-transformed prevalence (BMI ≥30 kg/m^2)	0.60 (0.59, 0.60)
Mean age of age group	-0.092 (-0.092, -0.091)
Male sex	-0.0013 (-0.0078, 0.0051)
Study mid-year (per one more recent year since 1975)	0.0099 (0.0098, 0.010)
Natural logarithm of per-capita gross domestic product	0.086 (0.083, 0.090)
Probit-transformed prevalence (BMI ≥30 kg/m^2) * mean age of age group	-0.0069 (-0.0072, -0.0065)
Probit-transformed prevalence (BMI ≥30 kg/m^2) * male sex	-0.029 (-0.031, -0.026)
Random effects for regions	Yes
**Number of data points used to fit the model = 6,848**	

Traditional R^2 is not clearly defined for mixed-effect models. The pseudo-R^2 for the model, which describes the proportion of variance explained by both fixed and random factors,[12] was 0.868.

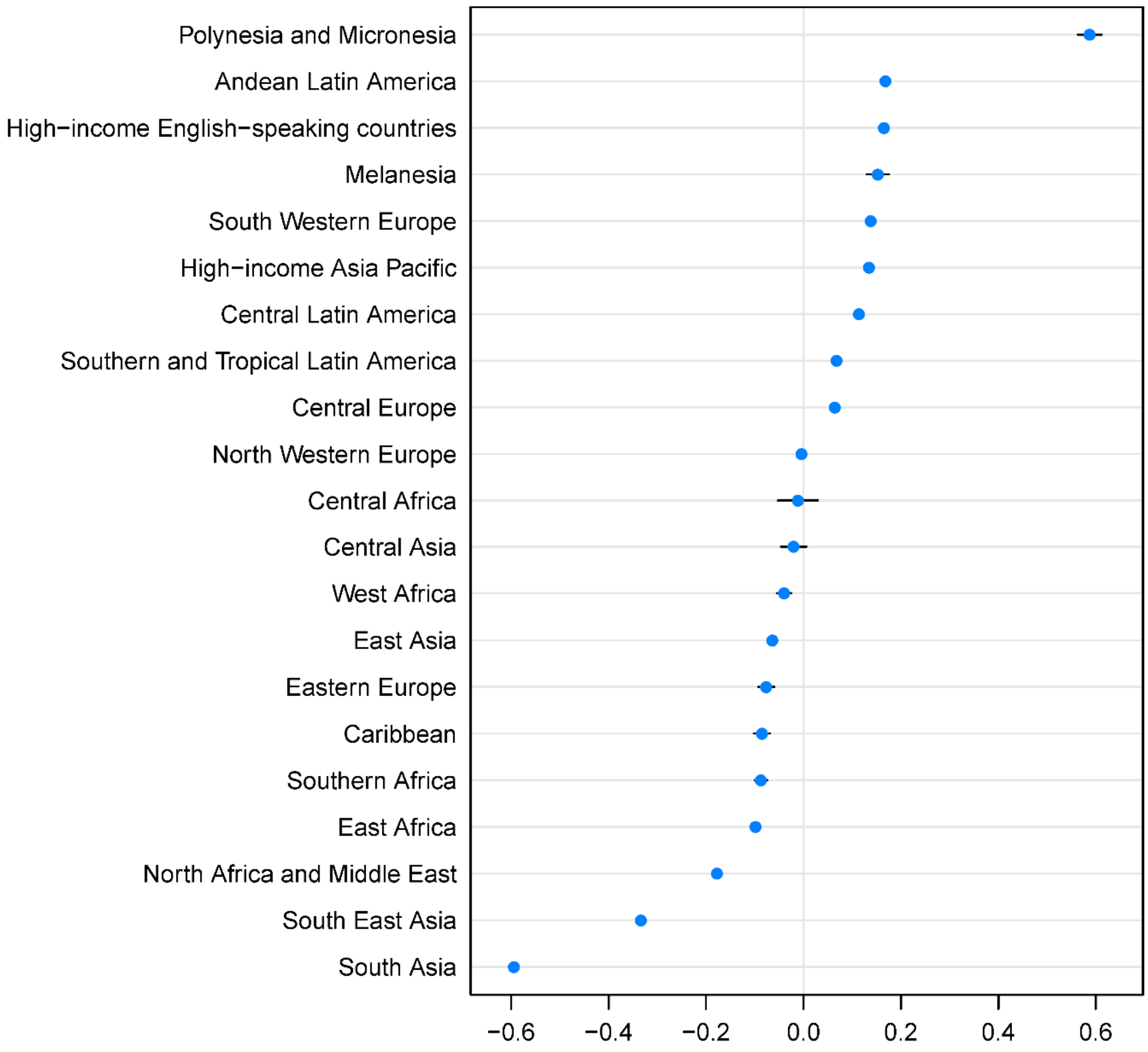
Polynesia and Micronesia
Andean Latin America
High−income English−speaking countries
Melanesia
South Western Europe
High−income Asia Pacific
Central Latin America
Southern and Tropical Latin America
Central Europe
North Western Europe
Central Africa
Central Asia
West Africa
East Asia
Eastern Europe
Caribbean
Southern Africa
East Africa
North Africa and Middle East
South East Asia
South Asia
−0.6
−0.4
−0.2
0.0
0.2
0.4
0.6

Dependent variable: Prevalence (BMI > +1SD)	
**Independent variable: Prevalence (BMI ≥25 kg/m^2) and prevalence (BMI ≥30 kg/m^2)**	
**Variables**	**Coefficients (95% CI)**
Intercept	0.27 (0.21, 0.33)
Probit-transformed prevalence (BMI ≥25 kg/m^2)	1.72 (1.70, 1.74)
Probit-transformed prevalence (BMI ≥30 kg/m^2)	-1.19 (-1.21, -1.18)
Mean age of age group	-0.029 (-0.031, -0.028)
Male sex	0.13 (0.12, 0.14)
Study mid-year (per one more recent year since 1975)	0.0027 (0.0025, 0.0028)
Natural logarithm of per-capita gross domestic product	0.016 (0.013, 0.020)
Probit-transformed prevalence (BMI ≥25 kg/m^2) * mean age of age group	-0.061 (-0.062, -0.060)
Probit-transformed prevalence (BMI ≥30 kg/m^2) * mean age of age group	0.079 (0.078, 0.080)
Probit-transformed prevalence (BMI ≥25 kg/m^2) * male sex	-0.062 (-0.072, -0.052)
Probit-transformed prevalence (BMI ≥30 kg/m^2) * male sex	0.061 (0.050, 0.072)
Random effects for regions	Yes
**Number of data points used to fit the model = 6,667**	

Traditional R^2 is not clearly defined for mixed-effect models. The pseudo-R^2 for the model, which describes the proportion of variance explained by both fixed and random factors,[12] was 0.926.

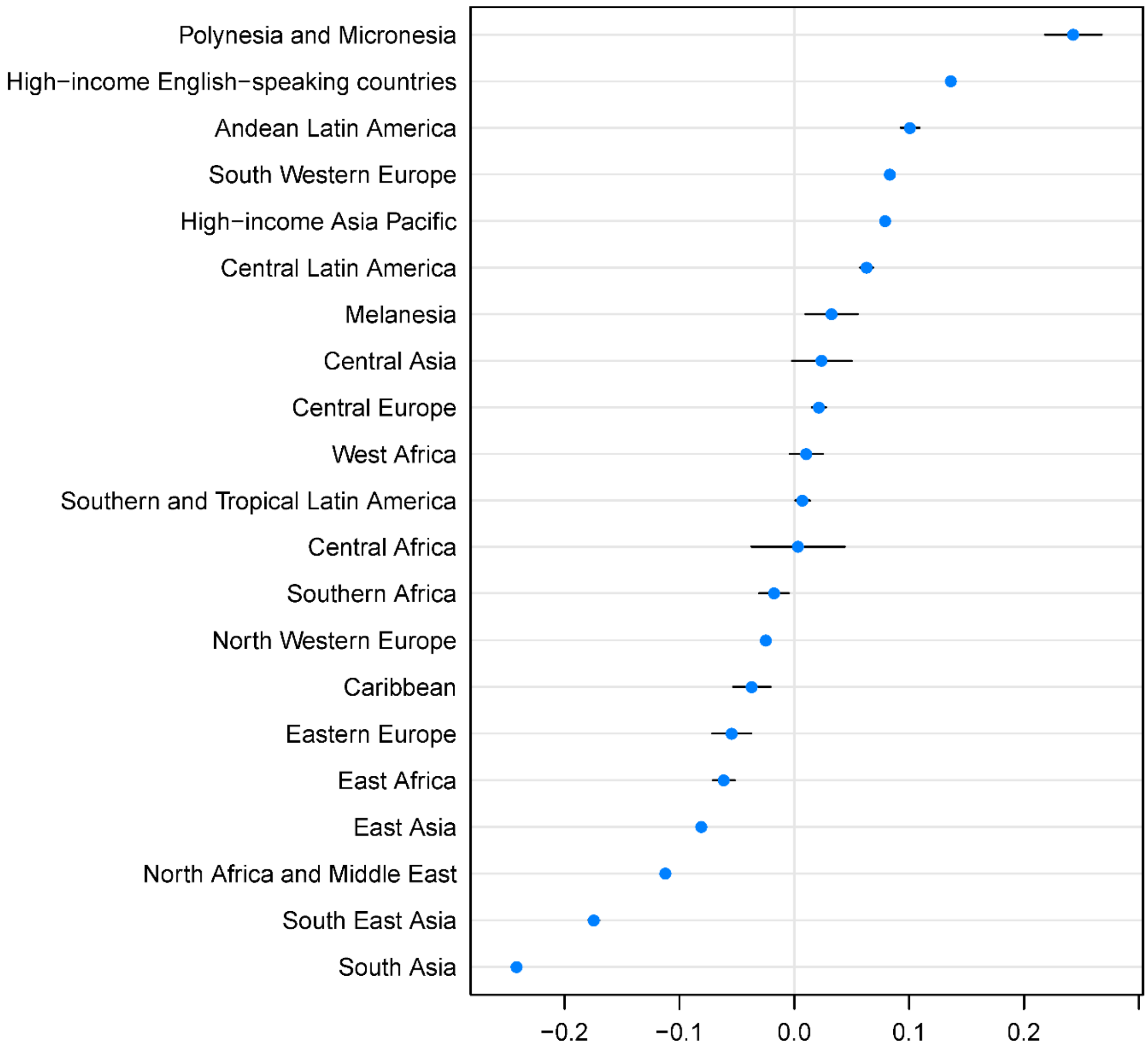
Polynesia and Micronesia
High−income English−speaking countries
Andean Latin America
South Western Europe
High−income Asia Pacific
Central Latin America
Melanesia
Central Asia
Central Europe
West Africa
Southern and Tropical Latin America
Central Africa
Southern Africa
North Western Europe
Caribbean
Eastern Europe
East Africa
East Asia
North Africa and Middle East
South East Asia
South Asia
−0.2
−0.1
0.0
0.1
0.2

**Dependent variable: Prevalence (BMI > +1SD)**	
**Independent variable: Inverse mean BMI, prevalence (BMI ≥25 kg/m^2) and prevalence (BMI ≥30 kg/m^2)**	
**Variables**	**Coefficients (95% CI)**
Intercept	5.49 (5.41, 5.57)
Inverse mean BMI	-91.60 (-92.70, -90.40)
Probit-transformed prevalence (BMI ≥25 kg/m^2)	0.31 (0.29, 0.34)
Probit-transformed prevalence (BMI ≥30 kg/m^2)	-0.31 (-0.33, -0.29)
Mean age of age group	-0.35 (-0.36, -0.35)
Male sex	0.22 (0.19, 0.26)
Study mid-year (per one more recent year since 1975)	0.0024 (0.0022, 0.0026)
Natural logarithm of per-capita gross domestic product	-9.9e-05 (-0.0039, 0.0037)
Inverse mean BMI * mean age of age group	5.66 (5.55, 5.78)
Probit-transformed prevalence (BMI ≥25 kg/m^2) * mean age of age group	0.044 (0.042, 0.047)
Probit-transformed prevalence (BMI ≥30 kg/m^2) * mean age of age group	0.0087 (0.0071, 0.010)
Inverse mean BMI * male sex	-2.02 (-2.71, -1.34)
Probit-transformed prevalence (BMI ≥25 kg/m^2) * male sex	-0.0078 (-0.022, 0.0065)
Probit-transformed prevalence (BMI ≥30 kg/m^2) * male sex	0.011 (-0.0012, 0.023)
Random effects for regions	Yes
**Number of data points used to fit the model = 6,667**	

Traditional R^2 is not clearly defined for mixed-effect models. The pseudo-R^2 for the model, which describes the proportion of variance explained by both fixed and random factors,[12] was 0.948.

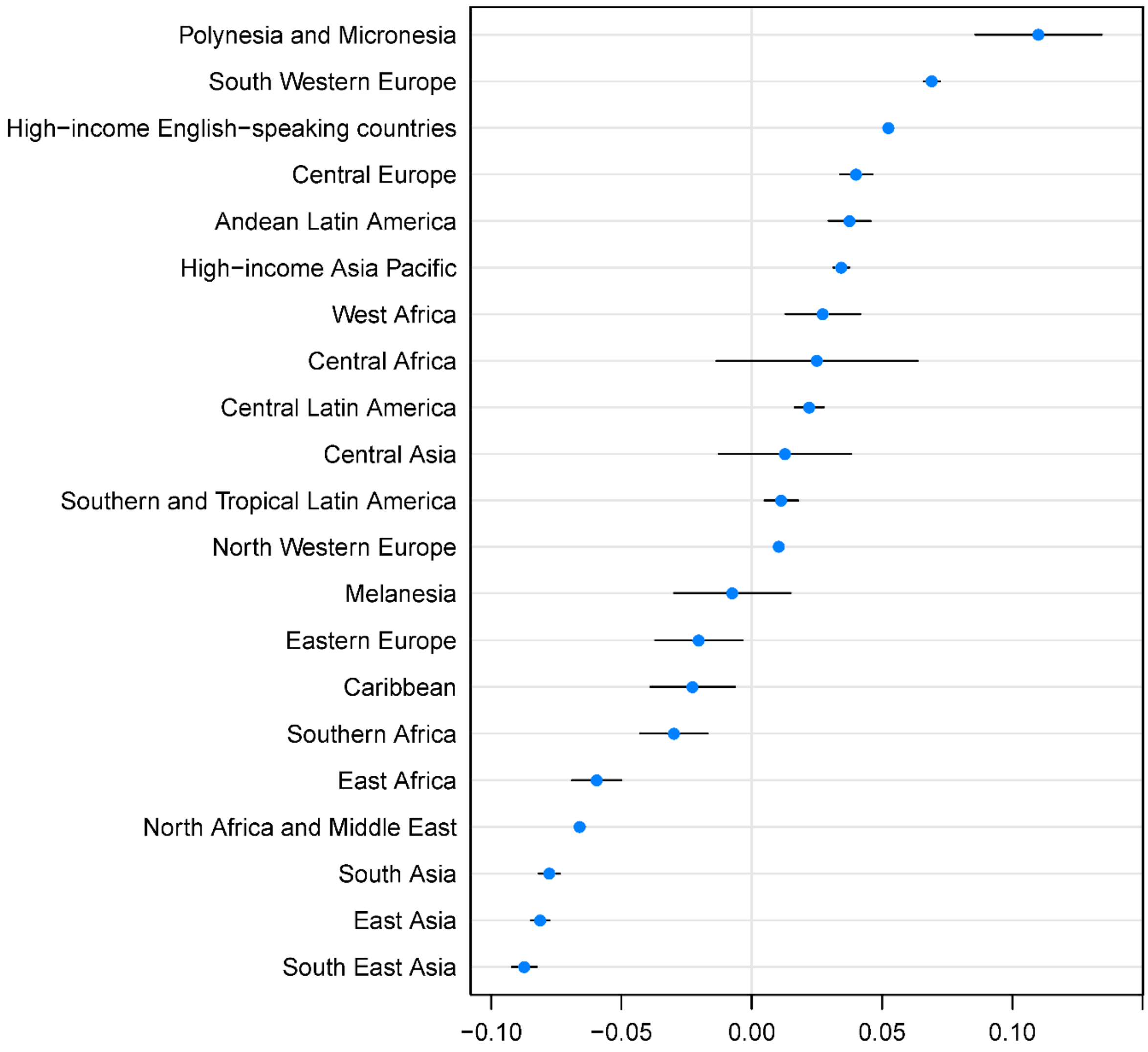
Polynesia and Micronesia
South Western Europe
High−income English−speaking countries
Central Europe
Andean Latin America
High−income Asia Pacific
West Africa
Central Africa
Central Latin America
Central Asia
Southern and Tropical Latin America
North Western Europe
Melanesia
Eastern Europe
Caribbean
Southern Africa
East Africa
North Africa and Middle East
South Asia
East Asia
South East Asia
−0.10
−0.05
0.00
0.05
0.10

Dependent variable: Prevalence (BMI > +1SD)	
**Independent variable: Inverse mean BMI, prevalence (20 kg/m^2 ≤ BMI <25 kg/m^2) and prevalence (25 kg/m^2 ≤ BMI <30 kg/m^2)**	
**Variables**	**Coefficients (95% CI)**
Intercept	3.62 (3.54, 3.70)
Inverse mean BMI	-55.40 (-56.80, -54.00)
Probit-transformed prevalence (20 kg/m^2 ≤ BMI <25 kg/m^2)	0.88 (0.86, 0.90)
Probit-transformed prevalence (25 kg/m^2 ≤ BMI <30 kg/m^2)	-0.45 (-0.46, -0.44)
Mean age of age group	-0.17 (-0.18, -0.17)
Male sex	0.54 (0.50, 0.58)
Study mid-year (per one more recent year since 1975)	0.0012 (0.0010, 0.0013)
Natural logarithm of per-capita gross domestic product	0.030 (0.026, 0.033)
Inverse mean BMI * mean age of age group	2.04 (1.90, 2.18)
Probit-transformed prevalence (20 kg/m^2 ≤ BMI <25 kg/m^2) * mean age of age group	-0.054 (-0.055, -0.053)
Probit-transformed prevalence (25 kg/m^2 ≤ BMI <30 kg/m^2) * mean age of age group	0.079 (0.078, 0.081)
Inverse mean BMI * male sex	-9.10 (-9.94, -8.26)
Probit-transformed prevalence (20 kg/m^2 ≤ BMI <25 kg/m^2) * male sex	-0.074 (-0.084, -0.065)
Probit-transformed prevalence (25 kg/m^2 ≤ BMI <30 kg/m^2) * male sex	-0.0049 (-0.012, 0.0023)
Random effects for regions	Yes
**Number of data points used to fit the model = 9,419**	

Traditional R^2 is not clearly defined for mixed-effect models. The pseudo-R^2 for the model, which describes the proportion of variance explained by both fixed and random factors,[12] was 0.943.

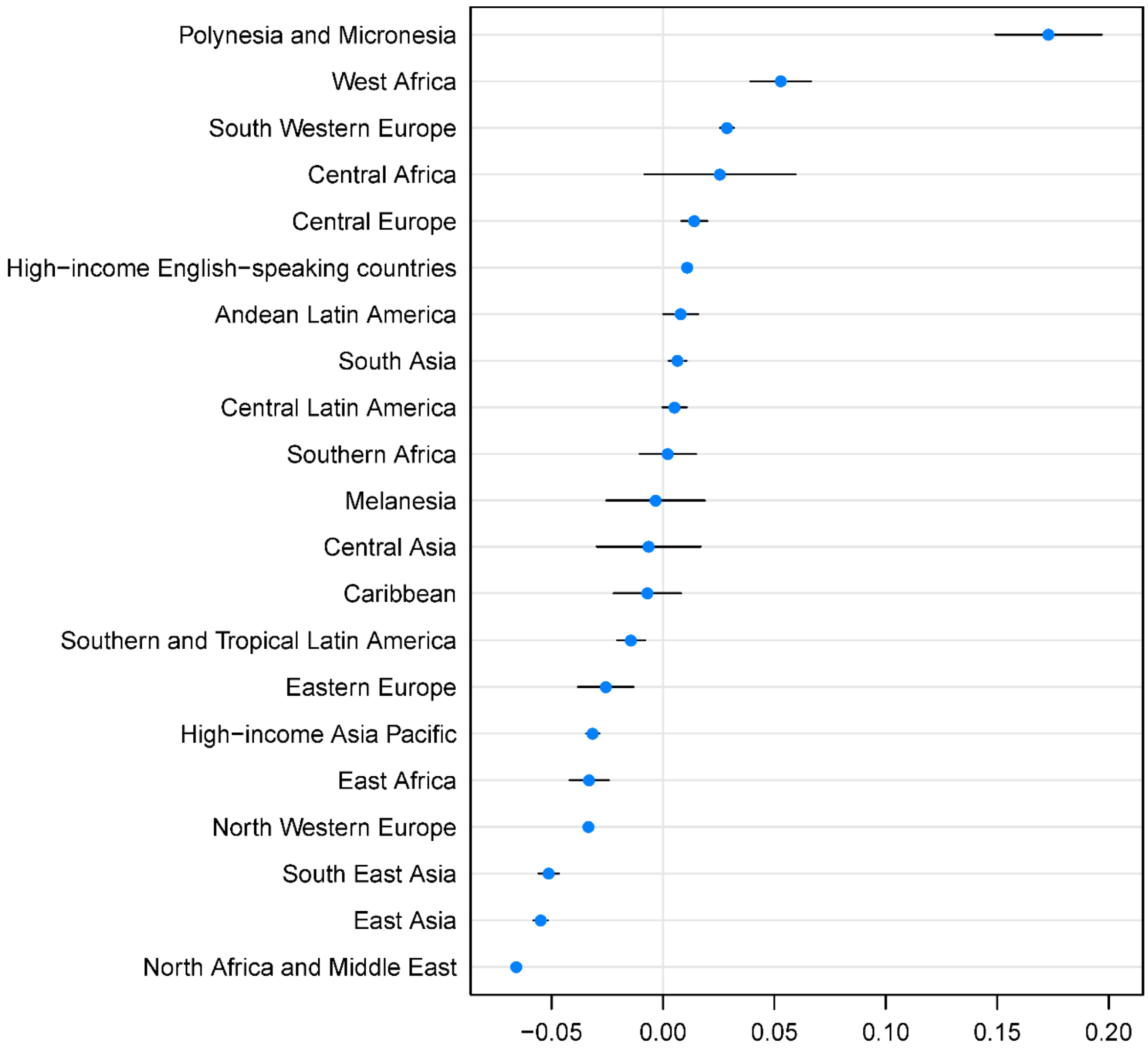
Polynesia and Micronesia
West Africa
South Western Europe
Central Africa
Central Europe
High−income English−speaking countries
Andean Latin America
South Asia
Central Latin America
Southern Africa
Melanesia
Central Asia
Caribbean
Southern and Tropical Latin America
Eastern Europe
High−income Asia Pacific
East Africa
North Western Europe
South East Asia
East Asia
North Africa and Middle East
−0.05
0.00
0.05
0.10
0.15
0.20

**Dependent variable: Prevalence (BMI > +1SD)**	
**Independent variable: Inverse mean BMI and prevalence (BMI ≥25 kg/m²)**	
**Variables**	**Coefficients (95% CI)**
Intercept	6.17 (6.10, 6.23)
Inverse mean BMI	-105.00 (-106.00, -104.00)
Probit-transformed prevalence (BMI ≥25 kg/m²)	-0.057 (-0.065, -0.049)
Mean age of age group	-0.39 (-0.39, -0.38)
Male sex	0.11 (0.088, 0.14)
Study mid-year (per one more recent year since 1975)	0.0029 (0.0027, 0.0030)
Natural logarithm of per-capita gross domestic product	0.017 (0.014, 0.020)
Inverse mean BMI * mean age of age group	6.16 (6.07, 6.26)
Probit-transformed prevalence (BMI ≥25 kg/m²) * mean age of age group	0.052 (0.051, 0.053)
Inverse mean BMI * male sex	0.73 (0.13, 1.32)
Probit-transformed prevalence (BMI ≥25 kg/m²) * male sex	0.031 (0.025, 0.036)
Random effects for regions	Yes
**Number of data points used to fit the model = 9,619**	

Traditional R^2 is not clearly defined for mixed-effect models. The pseudo-R^2 for the model, which describes the proportion of variance explained by both fixed and random factors,[12] was 0.937.

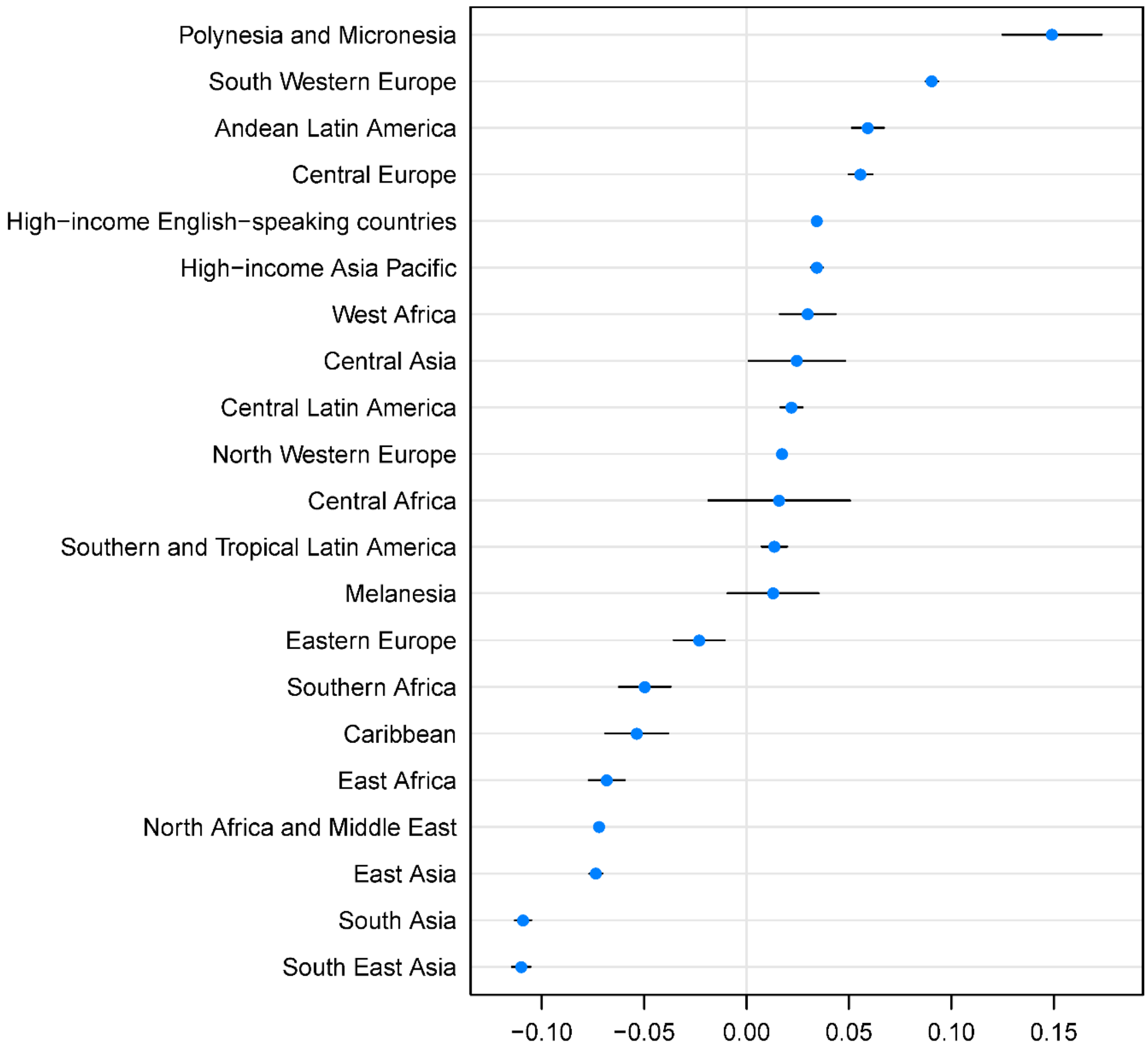
Polynesia and Micronesia
South Western Europe
Andean Latin America
Central Europe
High−income English−speaking countries
High−income Asia Pacific
West Africa
Central Asia
Central Latin America
North Western Europe
Central Africa
Southern and Tropical Latin America
Melanesia
Eastern Europe
Southern Africa
Caribbean
East Africa
North Africa and Middle East
East Asia
South Asia
South East Asia
−0.10
−0.05
0.00
0.05
0.10
0.15

Dependent variable: Prevalence (BMI > +1SD)	
**Independent variable: Inverse mean BMI, prevalence (15 kg/m^2 ≤ BMI <18.5 kg/m^2), prevalence (18.5 kg/m^2 ≤ BMI <20 kg/m^2), prevalence (20 kg/m^2 ≤ BMI <25 kg/m^2) and prevalence (BMI ≥25 kg/m^2)**	
**Variables**	**Coefficients (95% CI)**
Intercept	3.59 (3.49, 3.69)
Inverse mean BMI	-34.20 (-36.30, -32.20)
Probit-transformed prevalence (15 kg/m^2 ≤ BMI <18.5 kg/m^2)	0.23 (0.22, 0.25)
Probit-transformed prevalence (18.5 kg/m^2 ≤ BMI <20 kg/m^2)	0.50 (0.47, 0.53)
Probit-transformed prevalence (20 kg/m^2 ≤ BMI <25 kg/m^2)	0.78 (0.76, 0.81)
Probit-transformed prevalence (BMI ≥25 kg/m^2)	-0.25 (-0.26, -0.23)
Mean age of age group	-0.23 (-0.24, -0.22)
Male sex	0.54 (0.50, 0.58)
Study mid-year (per one more recent year since 1975)	0.00039 (0.00024, 0.00054)
Natural logarithm of per-capita gross domestic product	0.019 (0.016, 0.023)
Inverse mean BMI * mean age of age group	1.40 (1.21, 1.59)
Probit-transformed prevalence (15 kg/m^2 ≤ BMI <18.5 kg/m^2) * mean age of age group	-0.015 (-0.017, -0.014)
Probit-transformed prevalence (18.5 kg/m^2 ≤ BMI <20 kg/m^2) * mean age of age group	-0.049 (-0.051, -0.047)
Probit-transformed prevalence (20 kg/m^2 ≤ BMI <25 kg/m^2) * mean age of age group	-0.031 (-0.033, -0.029)
Probit-transformed prevalence (BMI ≥25 kg/m^2) * mean age of age group	0.059 (0.057, 0.060)
Inverse mean BMI * male sex	-5.63 (-6.55, -4.72)
Probit-transformed prevalence (15 kg/m^2 ≤ BMI <18.5 kg/m^2) * male sex	0.054 (0.047, 0.061)
Probit-transformed prevalence (18.5 kg/m^2 ≤ BMI <20 kg/m^2) * male sex	0.15 (0.14, 0.16)
Probit-transformed prevalence (20 kg/m^2 ≤ BMI <25 kg/m^2) * male sex	-0.15 (-0.16, -0.13)
Probit-transformed prevalence (BMI ≥25 kg/m^2) * male sex	0.038 (0.031, 0.044)
Random effects for regions	Yes
**Number of data points used to fit the model = 9,473**	

Traditional R^2 is not clearly defined for mixed-effect models. The pseudo-R^2 for the model, which describes the proportion of variance explained by both fixed and random factors,[12] was 0.952.

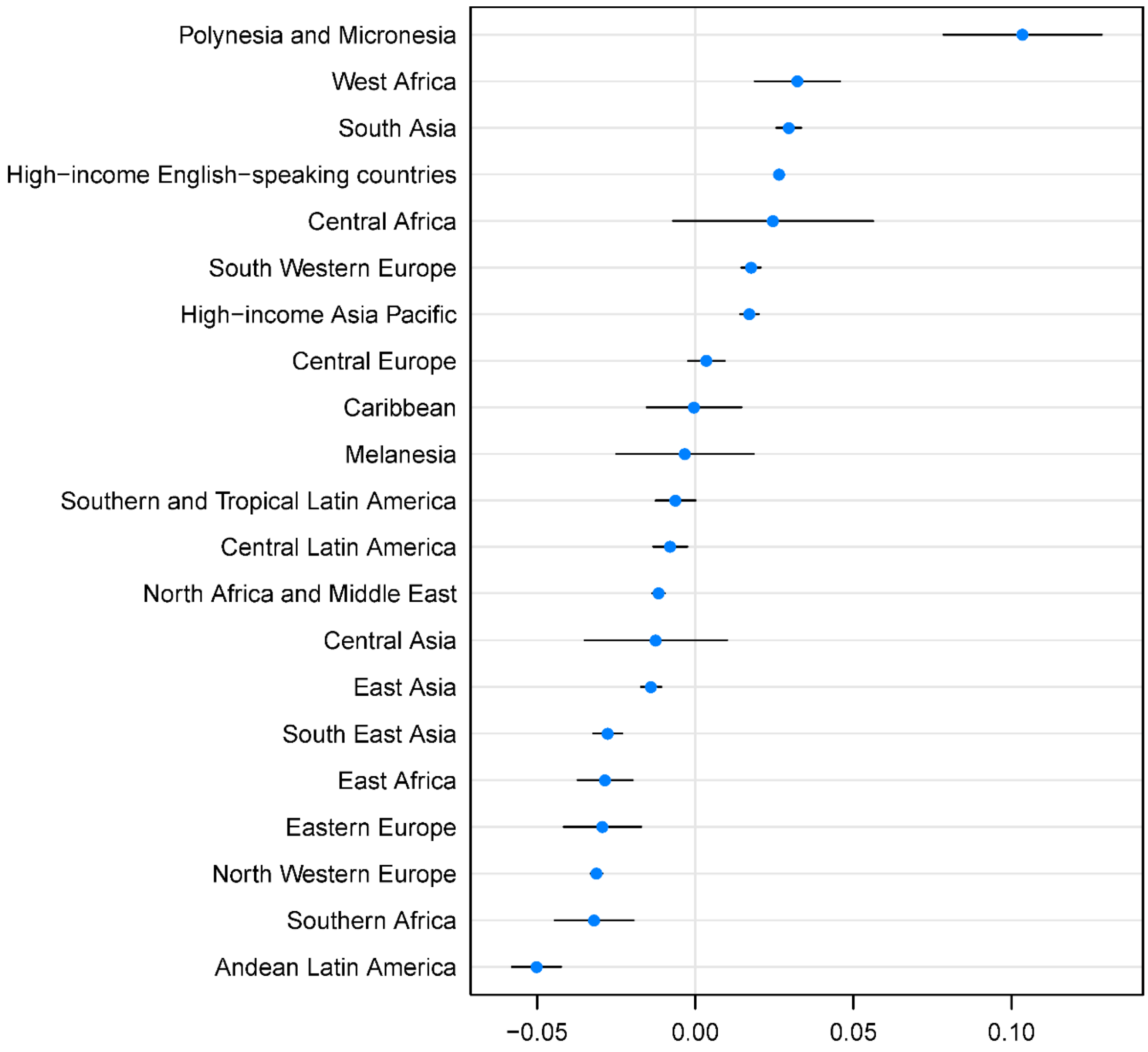
Polynesia and Micronesia
West Africa
South Asia
High−income English−speaking countries
Central Africa
South Western Europe
High−income Asia Pacific
Central Europe
Caribbean
Melanesia
Southern and Tropical Latin America
Central Latin America
North Africa and Middle East
Central Asia
East Asia
South East Asia
East Africa
Eastern Europe
North Western Europe
Southern Africa
Andean Latin America
−0.05
0.00
0.05
0.10

Dependent variable: Prevalence (BMI > +1SD)	
**Independent variable: Inverse mean BMI and prevalence (20 kg/m^2 ≤ BMI <25 kg/m^2)**	
**Variables**	**Coefficients (95% CI)**
Intercept	1.78 (1.71, 1.86)
Inverse mean BMI	-17.00 (-18.20, -15.70)
Probit-transformed prevalence (20 kg/m^2 ≤ BMI <25 kg/m^2)	0.97 (0.96, 0.98)
Mean age of age group	0.079 (0.074, 0.084)
Male sex	0.44 (0.41, 0.48)
Study mid-year (per one more recent year since 1975)	0.0037 (0.0036, 0.0038)
Natural logarithm of per-capita gross domestic product	0.10 (0.099, 0.10)
Inverse mean BMI * mean age of age group	-5.70 (-5.80, -5.60)
Probit-transformed prevalence (20 kg/m^2 ≤ BMI <25 kg/m^2) * mean age of age group	-0.070 (-0.071, -0.069)
Inverse mean BMI * male sex	-6.36 (-7.17, -5.55)
Probit-transformed prevalence (20 kg/m^2 ≤ BMI <25 kg/m^2) * male sex	-0.040 (-0.048, -0.033)
Random effects for regions	Yes
**Number of data points used to fit the model = 10,719**	

Traditional R^2 is not clearly defined for mixed-effect models. The pseudo-R^2 for the model, which describes the proportion of variance explained by both fixed and random factors,[12] was 0.921.

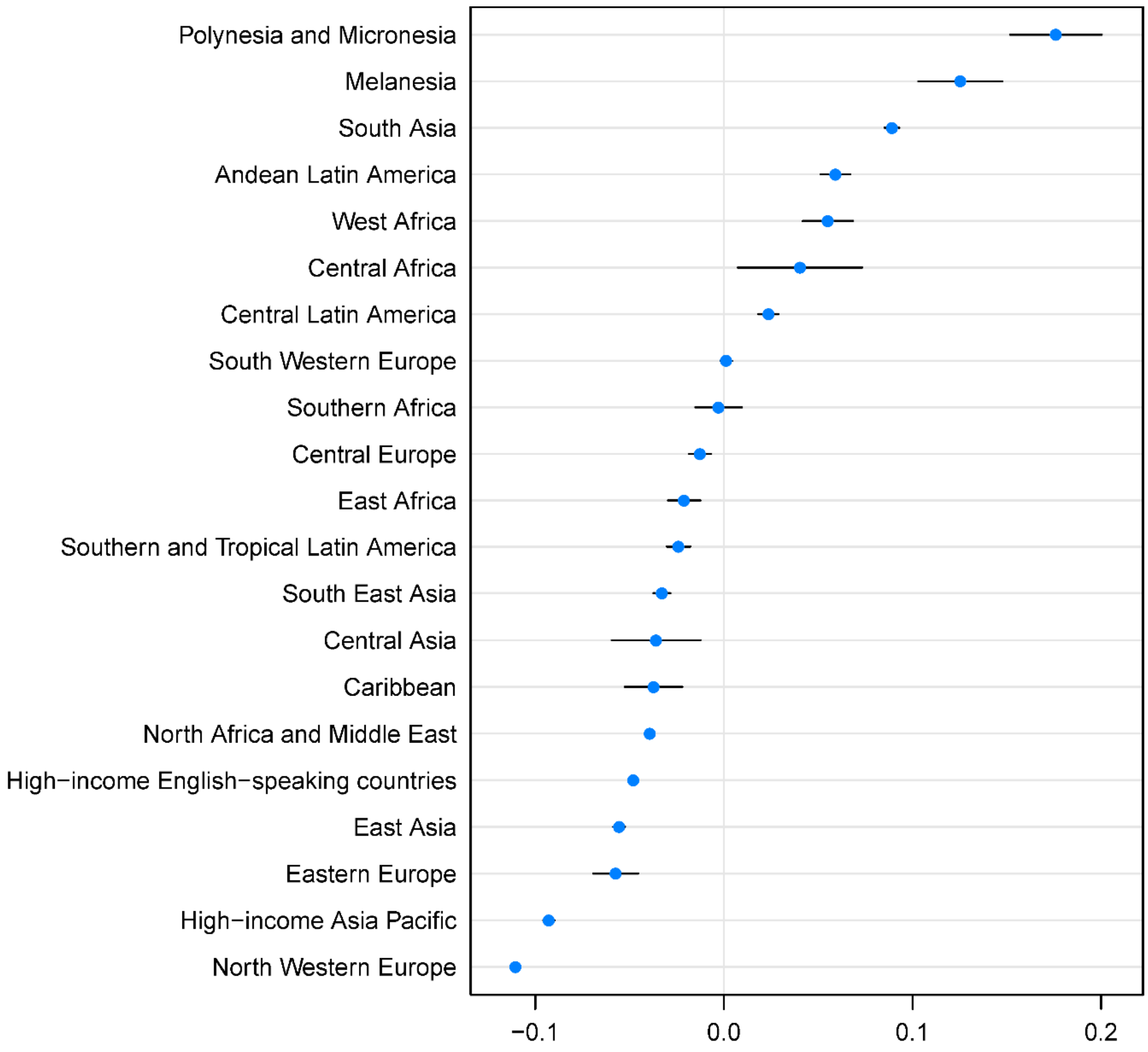
Polynesia and Micronesia
Melanesia
South Asia
Andean Latin America
West Africa
Central Africa
Central Latin America
South Western Europe
Southern Africa
Central Europe
East Africa
Southern and Tropical Latin America
South East Asia
Central Asia
Caribbean
North Africa and Middle East
High−income English−speaking countries
East Asia
Eastern Europe
High−income Asia Pacific
North Western Europe
−0.1
0.0
0.1
0.2

Dependent variable: Prevalence (BMI > +2SD)	
**Independent variable: Inverse mean BMI**	
**Variables**	**Coefficients (95% CI)**
Intercept	5.87 (5.78, 5.97)
Inverse mean BMI	-132.00 (-133.00, -132.00)
Mean age of age group	-0.39 (-0.39, -0.38)
Male sex	0.84 (0.82, 0.85)
Study mid-year (per one more recent year since 1975)	0.0077 (0.0075, 0.0079)
Natural logarithm of per-capita gross domestic product	0.15 (0.14, 0.15)
Inverse mean BMI * mean age of age group	4.09 (4.05, 4.13)
Inverse mean BMI * male sex	-11.00 (-11.30, -10.70)
Random effects for regions	Yes
**Number of data points used to fit the model = 10,808**	

Traditional R^2 is not clearly defined for mixed-effect models. The pseudo-R^2 for the model, which describes the proportion of variance explained by both fixed and random factors,[12] was 0.795.

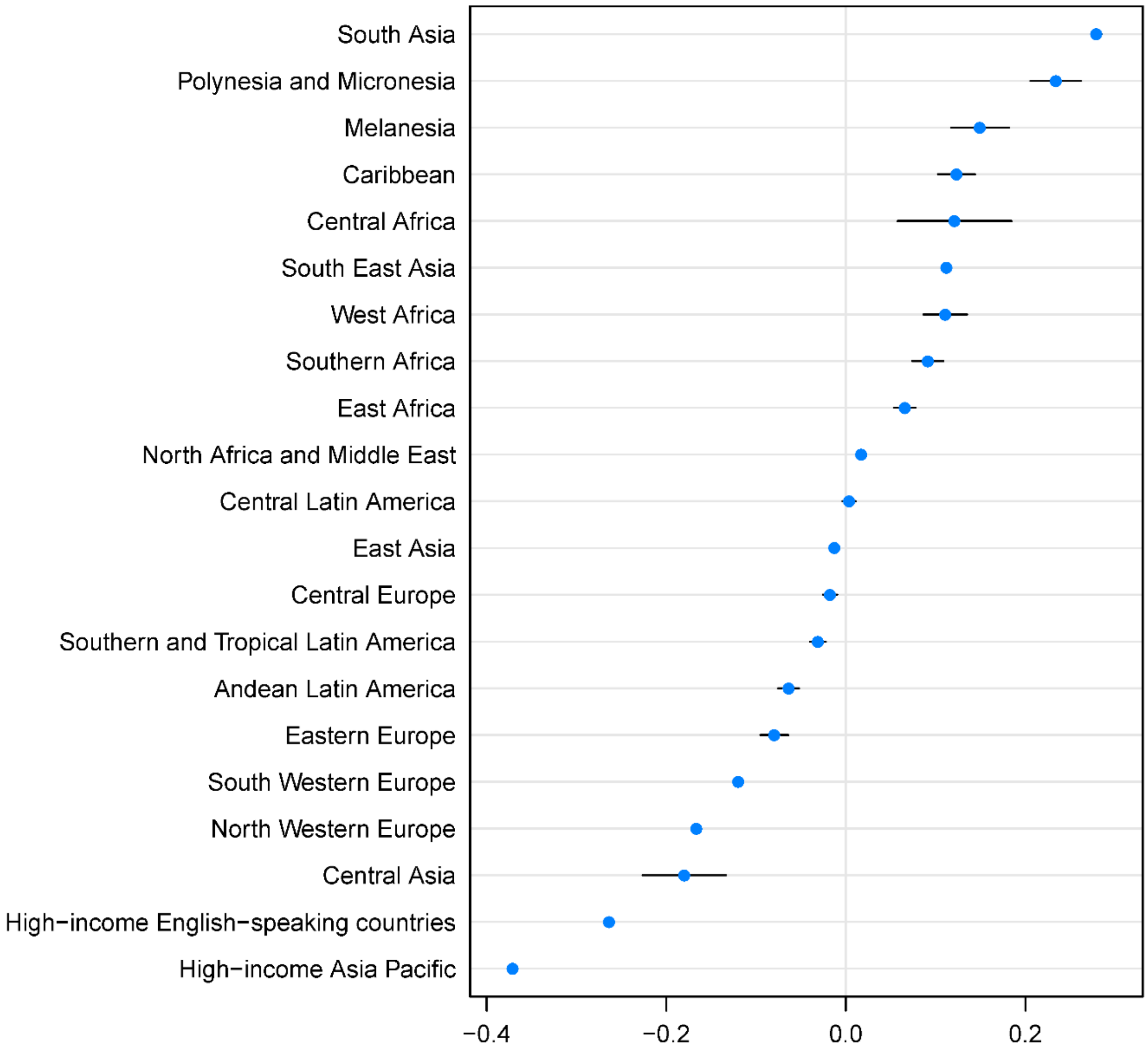
South Asia
Polynesia and Micronesia
Melanesia
Caribbean
Central Africa
South East Asia
West Africa
Southern Africa
East Africa
North Africa and Middle East
Central Latin America
East Asia
Central Europe
Southern and Tropical Latin America
Andean Latin America
Eastern Europe
South Western Europe
North Western Europe
Central Asia
High−income English−speaking countries
High−income Asia Pacific
−0.4
−0.2
0.0
0.2

**Dependent variable: Prevalence (BMI > +2SD)**	
**Independent variable: Prevalence (BMI ≥25 kg/m^2)**	
**Variables**	**Coefficients (95% CI)**
Intercept	0.19 (0.13, 0.25)
Probit-transformed prevalence (BMI ≥25 kg/m^2)	0.78 (0.78, 0.78)
Mean age of age group	-0.14 (-0.14, -0.14)
Male sex	0.28 (0.28, 0.29)
Study mid-year (per one more recent year since 1975)	0.0055 (0.0053, 0.0057)
Natural logarithm of per-capita gross domestic product	0.096 (0.091, 0.10)
Probit-transformed prevalence (BMI ≥25 kg/m^2) * mean age of age group	-0.0036 (-0.0040, -0.0031)
Probit-transformed prevalence (BMI ≥25 kg/m^2) * male sex	0.059 (0.056, 0.062)
Random effects for regions	Yes
**Number of data points used to fit the model = 8,961**	

Traditional R^2 is not clearly defined for mixed-effect models. The pseudo-R^2 for the model, which describes the proportion of variance explained by both fixed and random factors,[12] was 0.891.

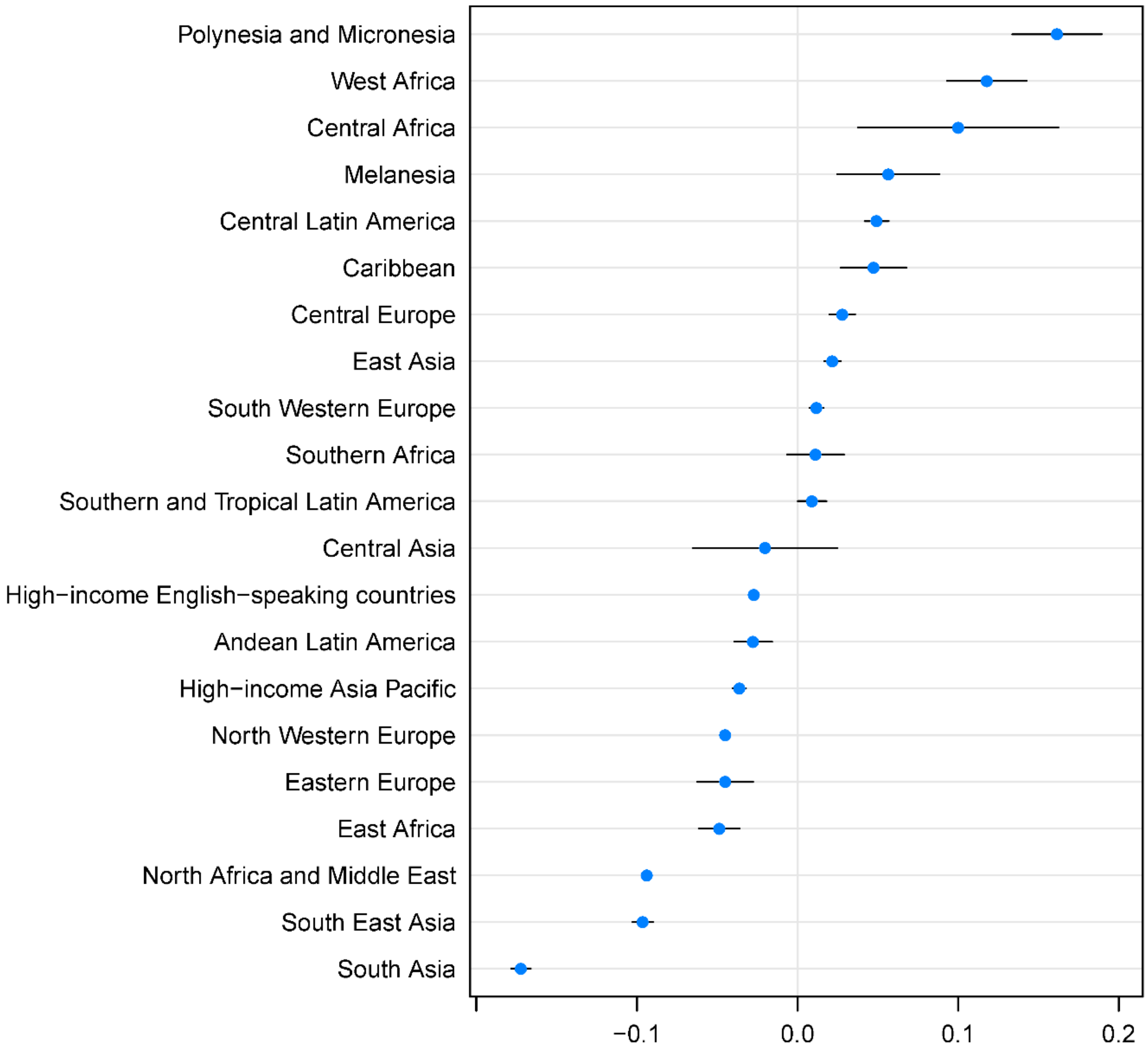
Polynesia and Micronesia
West Africa
Central Africa
Melanesia
Central Latin America
Caribbean
Central Europe
East Asia
South Western Europe
Southern Africa
Southern and Tropical Latin America
Central Asia
High−income English−speaking countries
Andean Latin America
High−income Asia Pacific
North Western Europe
Eastern Europe
East Africa
North Africa and Middle East
South East Asia
South Asia
−0.1
0.0
0.1
0.2

Dependent variable: Prevalence (BMI > +2SD)	
**Independent variable: Prevalence (BMI ≥25 kg/m^2) and prevalence (BMI ≥30 kg/m^2)**	
**Variables**	**Coefficients (95% CI)**
Intercept	0.21 (0.14, 0.28)
Probit-transformed prevalence (BMI ≥25 kg/m^2)	1.81 (1.78, 1.83)
Probit-transformed prevalence (BMI ≥30 kg/m^2)	-1.06 (-1.08, -1.03)
Mean age of age group	-0.054 (-0.056, -0.052)
Male sex	0.13 (0.12, 0.15)
Study mid-year (per one more recent year since 1975)	0.0022 (0.0020, 0.0024)
Natural logarithm of per-capita gross domestic product	0.029 (0.024, 0.035)
Probit-transformed prevalence (BMI ≥25 kg/m^2) * mean age of age group	-0.096 (-0.097, -0.094)
Probit-transformed prevalence (BMI ≥30 kg/m^2) * mean age of age group	0.10 (0.10, 0.11)
Probit-transformed prevalence (BMI ≥25 kg/m^2) * male sex	0.19 (0.17, 0.20)
Probit-transformed prevalence (BMI ≥30 kg/m^2) * male sex	-0.15 (-0.17, -0.14)
Random effects for regions	Yes
**Number of data points used to fit the model = 6,665**	

Traditional R^2 is not clearly defined for mixed-effect models. The pseudo-R^2 for the model, which describes the proportion of variance explained by both fixed and random factors,[12] was 0.922.

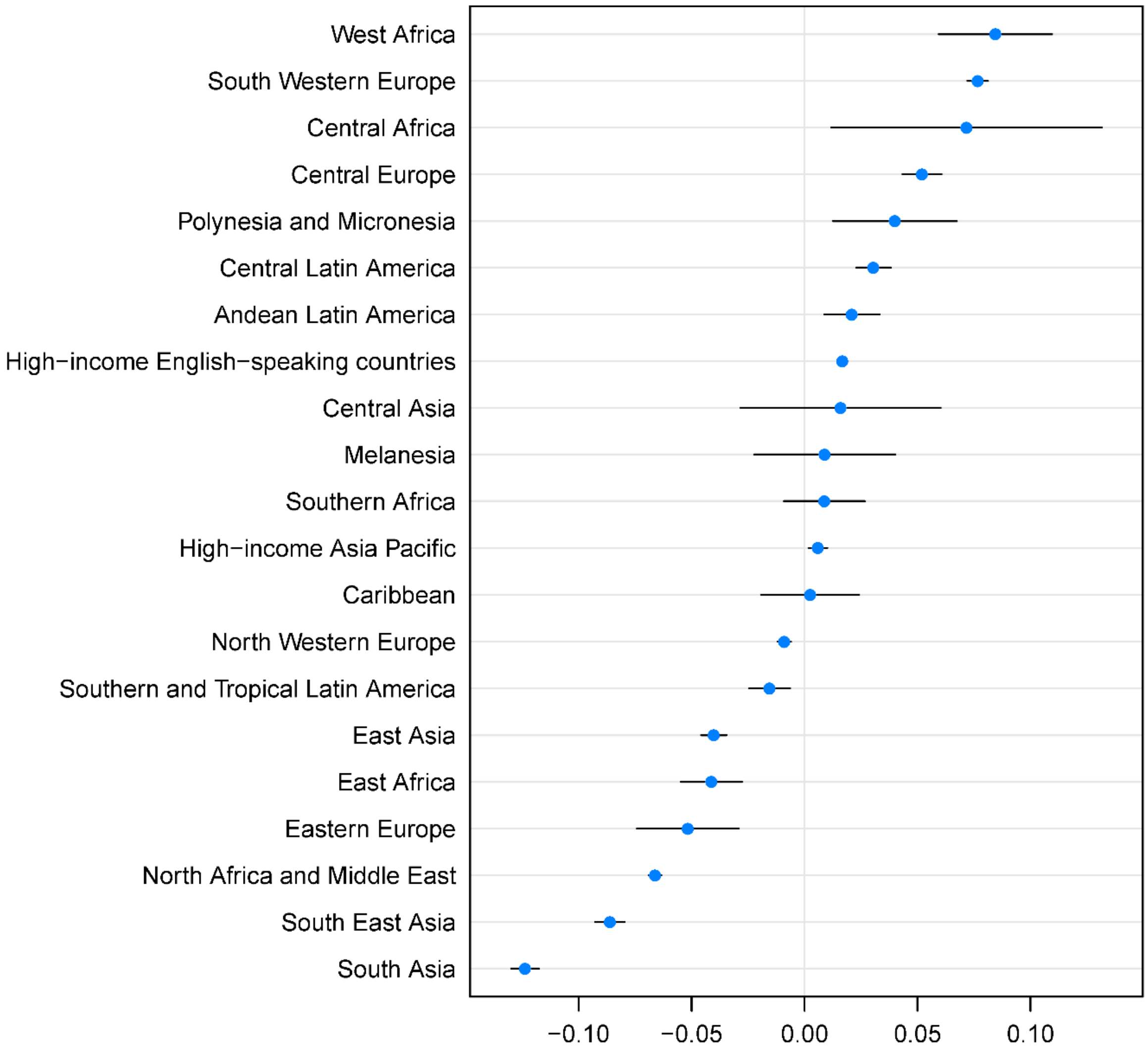
West Africa
South Western Europe
Central Africa
Central Europe
Polynesia and Micronesia
Central Latin America
Andean Latin America
High−income English−speaking countries
Central Asia
Melanesia
Southern Africa
High−income Asia Pacific
Caribbean
North Western Europe
Southern and Tropical Latin America
East Asia
East Africa
Eastern Europe
North Africa and Middle East
South East Asia
South Asia
−0.10
−0.05
0.00
0.05
0.10

Dependent variable: Prevalence (BMI > +2SD)	
**Independent variable: Inverse mean BMI, prevalence (BMI ≥25 kg/m^2) and prevalence (BMI ≥30 kg/m^2)**	
**Variables**	**Coefficients (95% CI)**
Intercept	1.00 (0.89, 1.11)
Inverse mean BMI	-14.20 (-15.80, -12.70)
Probit-transformed prevalence (BMI ≥25 kg/m^2)	1.58 (1.55, 1.62)
Probit-transformed prevalence (BMI ≥30 kg/m^2)	-0.92 (-0.95, -0.90)
Mean age of age group	-0.10 (-0.11, -0.093)
Male sex	-0.0074 (-0.054, 0.039)
Study mid-year (per one more recent year since 1975)	0.0021 (0.0018, 0.0023)
Natural logarithm of per-capita gross domestic product	0.026 (0.020, 0.032)
Inverse mean BMI * mean age of age group	0.82 (0.65, 0.99)
Probit-transformed prevalence (BMI ≥25 kg/m^2) * mean age of age group	-0.080 (-0.083, -0.077)
Probit-transformed prevalence (BMI ≥30 kg/m^2) * mean age of age group	0.094 (0.091, 0.096)
Inverse mean BMI * male sex	3.07 (2.11, 4.03)
Probit-transformed prevalence (BMI ≥25 kg/m^2) * male sex	0.24 (0.22, 0.26)
Probit-transformed prevalence (BMI ≥30 kg/m^2) * male sex	-0.18 (-0.19, -0.16)
Random effects for regions	Yes
**Number of data points used to fit the model = 6,665**	

Traditional R^2 is not clearly defined for mixed-effect models. The pseudo-R^2 for the model, which describes the proportion of variance explained by both fixed and random factors,[12] was 0.923.

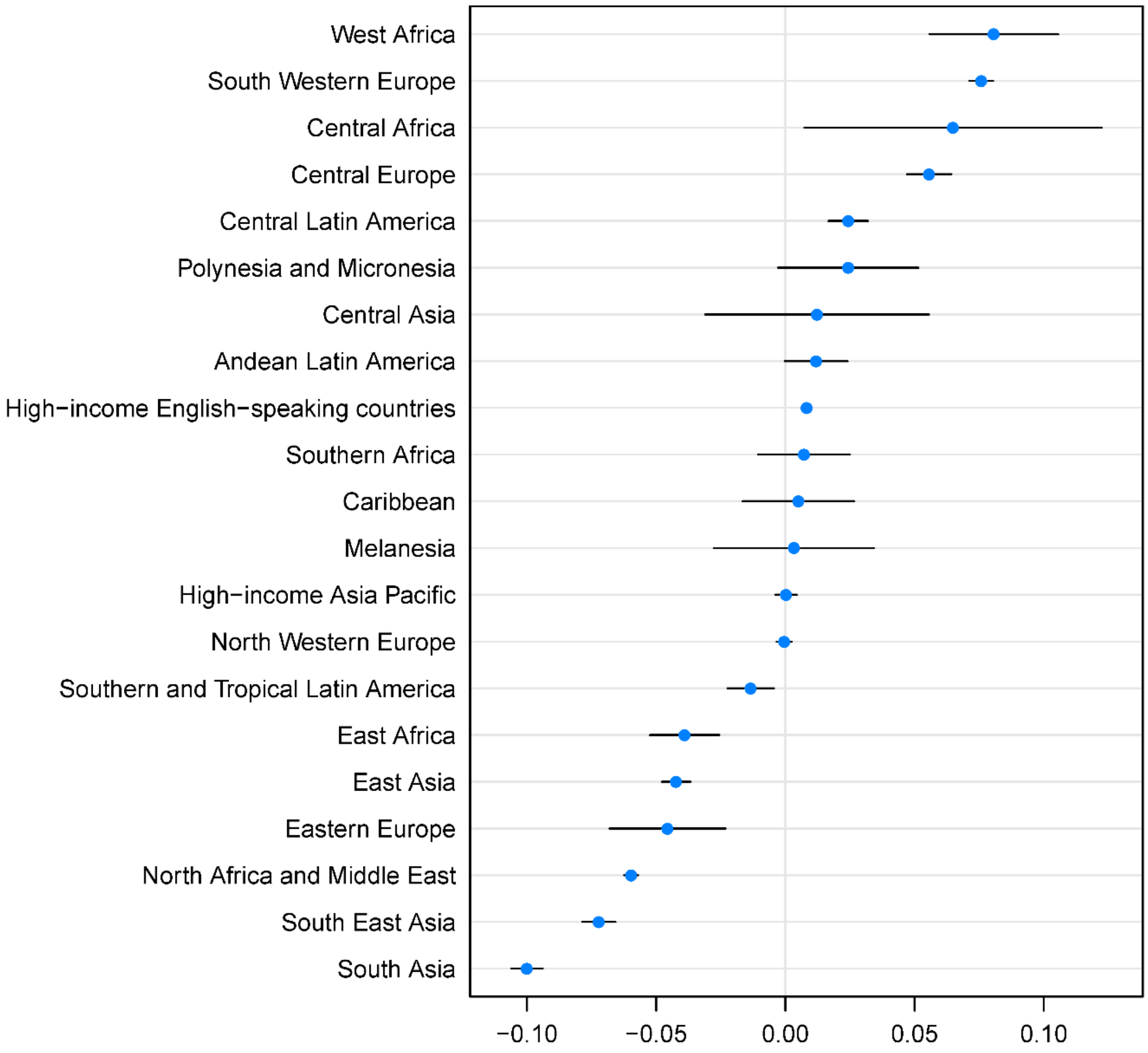
West Africa
South Western Europe
Central Africa
Central Europe
Central Latin America
Polynesia and Micronesia
Central Asia
Andean Latin America
High−income English−speaking countries
Southern Africa
Caribbean
Melanesia
High−income Asia Pacific
North Western Europe
Southern and Tropical Latin America
East Africa
East Asia
Eastern Europe
North Africa and Middle East
South East Asia
South Asia
−0.10
−0.05
0.00
0.05
0.10

**Dependent variable: Prevalence (BMI > +2SD)**	
**Independent variable: Inverse mean BMI and prevalence (BMI > +1SD)**	
**Variables**	**Coefficients (95% CI)**
Intercept	-2.48 (-2.58, -2.38)
Inverse mean BMI	14.10 (12.90, 15.30)
Probit-transformed prevalence (BMI > +1SD)	1.07 (1.05, 1.09)
Mean age of age group	-0.0030 (-0.0063, 0.00024)
Male sex	0.24 (0.22, 0.26)
Study mid-year (per one more recent year since 1975)	0.00056 (0.00039, 0.00074)
Natural logarithm of per-capita gross domestic product	0.075 (0.070, 0.079)
Inverse mean BMI * mean age of age group	0.69 (0.64, 0.74)
Probit-transformed prevalence (BMI > +1SD) * mean age of age group	0.0099 (0.0090, 0.011)
Inverse mean BMI * male sex	-3.70 (-4.01, -3.39)
Probit-transformed prevalence (BMI > +1SD) * male sex	-0.078 (-0.083, -0.072)
Random effects for regions	Yes
**Number of data points used to fit the model = 10,808**	

Traditional R^2 is not clearly defined for mixed-effect models. The pseudo-R^2 for the model, which describes the proportion of variance explained by both fixed and random factors,[12] was 0.899.

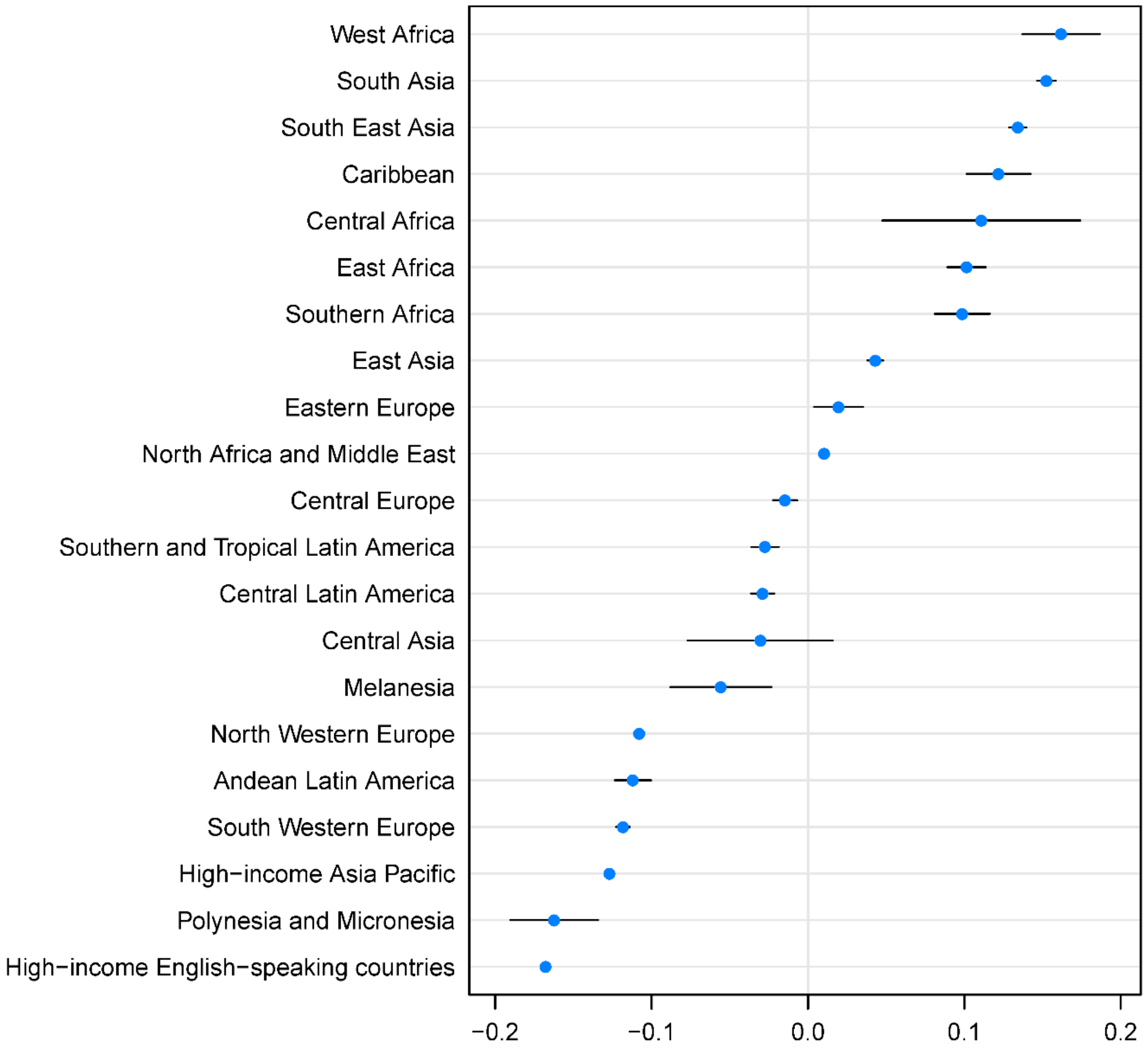
West Africa
South Asia
South East Asia
Caribbean
Central Africa
East Africa
Southern Africa
East Asia
Eastern Europe
North Africa and Middle East
Central Europe
Southern and Tropical Latin America
Central Latin America
Central Asia
Melanesia
North Western Europe
Andean Latin America
South Western Europe
High−income Asia Pacific
Polynesia and Micronesia
High−income English−speaking countries
−0.2
−0.1
0.0
0.1
0.2

**Dependent variable: Prevalence (BMI > +2SD)**	
**Independent variable: Inverse mean BMI and prevalence (20 kg/m^2 ≤ BMI < 25 kg/m^2)**	
**Variables**	**Coefficients (95% CI)**
Intercept	-2.15 (-2.25, -2.04)
Inverse mean BMI	52.00 (50.30, 53.70)
Probit-transformed prevalence (20 kg/m^2 ≤ BMI < 25 kg/m^2)	1.70 (1.68, 1.71)
Mean age of age group	0.32 (0.31, 0.32)
Male sex	0.050 (-8.4e-05, 0.10)
Study mid-year (per one more recent year since 1975)	0.0029 (0.0027, 0.0031)
Natural logarithm of per-capita gross domestic product	0.12 (0.12, 0.13)
Inverse mean BMI * mean age of age group	-11.30 (-11.40, -11.20)
Probit-transformed prevalence (20 kg/m^2 ≤ BMI < 25 kg/m^2) * mean age of age group	-0.13 (-0.13, -0.13)
Inverse mean BMI * male sex	5.26 (4.18, 6.34)
Probit-transformed prevalence (20 kg/m^2 ≤ BMI < 25 kg/m^2) * male sex	0.11 (0.096, 0.12)
Random effects for regions	Yes
**Number of data points used to fit the model = 9,844**	

Traditional R^2 is not clearly defined for mixed-effect models. The pseudo-R^2 for the model, which describes the proportion of variance explained by both fixed and random factors,[12] was 0.891.

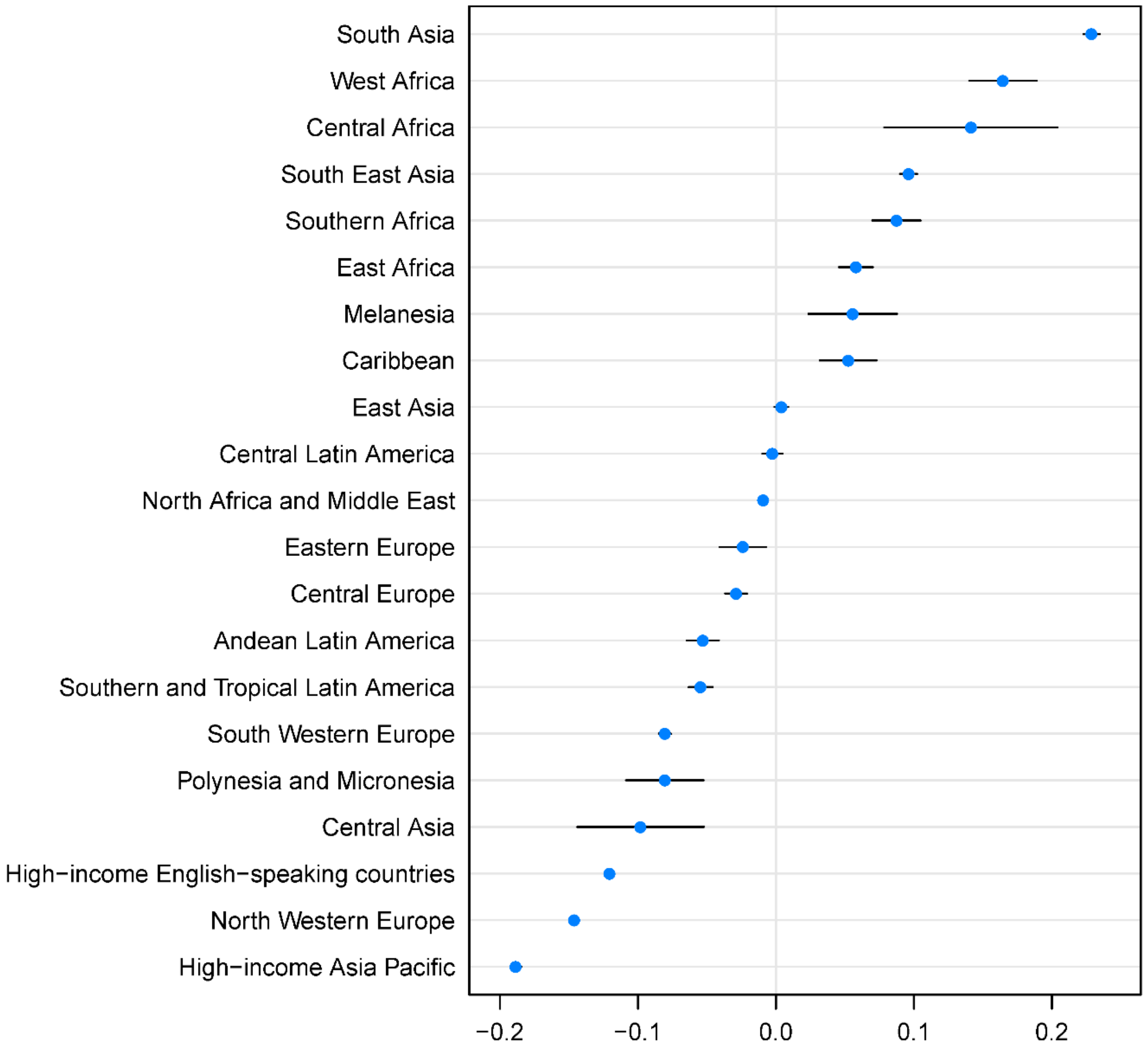
South Asia
West Africa
Central Africa
South East Asia
Southern Africa
East Africa
Melanesia
Caribbean
East Asia
Central Latin America
North Africa and Middle East
Eastern Europe
Central Europe
Andean Latin America
Southern and Tropical Latin America
South Western Europe
Polynesia and Micronesia
Central Asia
High−income English−speaking countries
North Western Europe
High−income Asia Pacific
−0.2
−0.1
0.0
0.1
0.2

**Dependent variable: Prevalence (BMI > +2SD)**	
**Independent variable: Inverse mean BMI, prevalence (20 kg/m^2 ≤ BMI < 25 kg/m^2) and prevalence (25 kg/m^2 ≤ BMI < 30 kg/m^2)**	
**Variables**	**Coefficients (95% CI)**
Intercept	-0.32 (-0.43, -0.21)
Inverse mean BMI	22.40 (20.60, 24.30)
Probit-transformed prevalence (20 kg/m^2 ≤ BMI < 25 kg/m^2)	0.99 (0.97, 1.02)
Probit-transformed prevalence (25 kg/m^2 ≤ BMI < 30 kg/m^2)	0.28 (0.26, 0.30)
Mean age of age group	0.14 (0.13, 0.14)
Male sex	-0.068 (-0.12, -0.017)
Study mid-year (per one more recent year since 1975)	0.0025 (0.0023, 0.0027)
Natural logarithm of per-capita gross domestic product	0.079 (0.074, 0.084)
Inverse mean BMI * mean age of age group	-6.55 (-6.74, -6.36)
Probit-transformed prevalence (20 kg/m^2 ≤ BMI < 25 kg/m^2) * mean age of age group	-0.087 (-0.089, -0.085)
Probit-transformed prevalence (25 kg/m^2 ≤ BMI < 30 kg/m^2) * mean age of age group	0.015 (0.013, 0.017)
Inverse mean BMI * male sex	6.48 (5.37, 7.58)
Probit-transformed prevalence (20 kg/m^2 ≤ BMI < 25 kg/m^2) * male sex	0.17 (0.16, 0.19)
Probit-transformed prevalence (25 kg/m^2 ≤ BMI < 30 kg/m^2) * male sex	-0.048 (-0.058, -0.037)
Random effects for regions	Yes
**Number of data points used to fit the model = 8,762**	

Traditional R^2 is not clearly defined for mixed-effect models. The pseudo-R^2 for the model, which describes the proportion of variance explained by both fixed and random factors,[12] was 0.908.

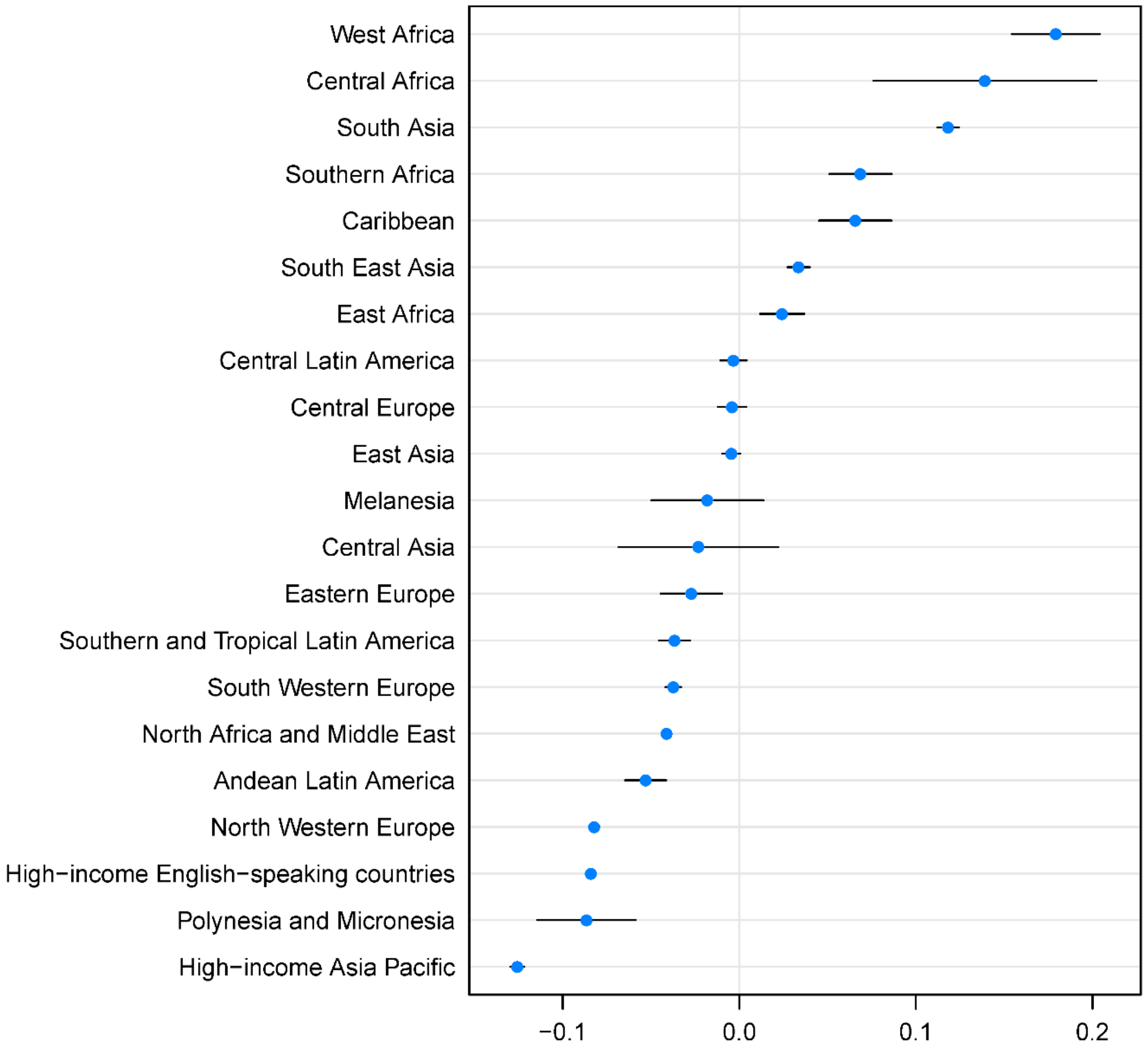

West Africa
Central Africa
South Asia
Southern Africa
Caribbean
South East Asia
East Africa
Central Latin America
Central Europe
East Asia
Melanesia
Central Asia
Eastern Europe
Southern and Tropical Latin America
South Western Europe
North Africa and Middle East
Andean Latin America
North Western Europe
High−income English−speaking countries
Polynesia and Micronesia
High−income Asia Pacific
−0.1
0.0
0.1
0.2

**Dependent variable: Prevalence (BMI > +2SD)**	
**Independent variable: Inverse mean BMI and prevalence (BMI ≥25 kg/m^2)**	
**Variables**	**Coefficients (95% CI)**
Intercept	3.09 (3.00, 3.18)
Inverse mean BMI	-54.10 (-55.40, -52.80)
Probit-transformed prevalence (BMI ≥25 kg/m^2)	0.42 (0.40, 0.43)
Mean age of age group	-0.30 (-0.30, -0.29)
Male sex	0.17 (0.13, 0.20)
Study mid-year (per one more recent year since 1975)	0.0038 (0.0036, 0.0040)
Natural logarithm of per-capita gross domestic product	0.061 (0.056, 0.066)
Inverse mean BMI * mean age of age group	3.53 (3.38, 3.68)
Probit-transformed prevalence (BMI ≥25 kg/m^2) * mean age of age group	0.025 (0.024, 0.027)
Inverse mean BMI * male sex	3.54 (2.69, 4.39)
Probit-transformed prevalence (BMI ≥25 kg/m^2) * male sex	0.099 (0.091, 0.11)
Random effects for regions	Yes
**Number of data points used to fit the model = 8,961**	

Traditional R^2 is not clearly defined for mixed-effect models. The pseudo-R^2 for the model, which describes the proportion of variance explained by both fixed and random factors,[12] was 0.901.

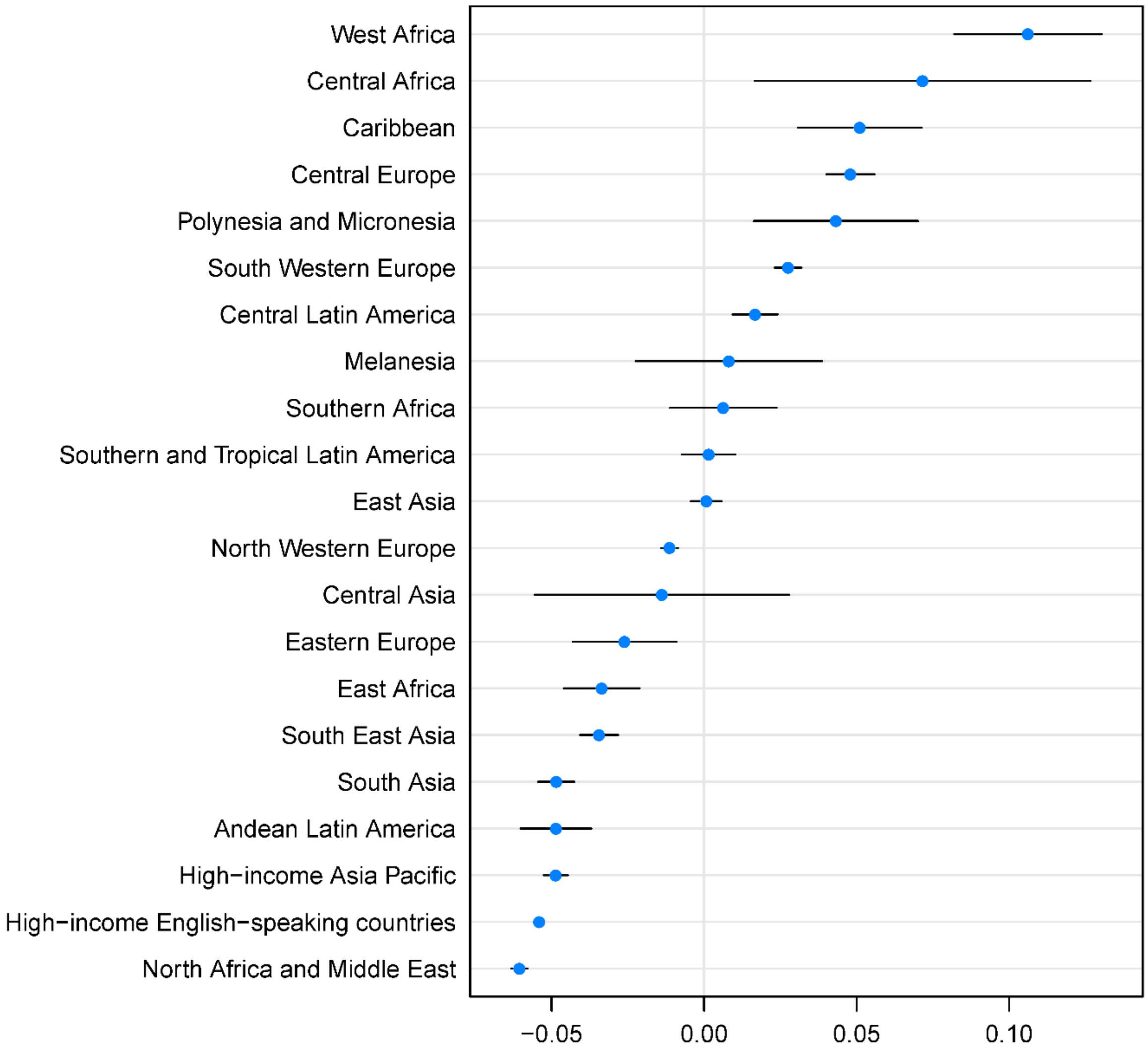

West Africa
Central Africa
Caribbean
Central Europe
Polynesia and Micronesia
South Western Europe
Central Latin America
Melanesia
Southern Africa
Southern and Tropical Latin America
East Asia
North Western Europe
Central Asia
Eastern Europe
East Africa
South East Asia
South Asia
Andean Latin America
High−income Asia Pacific
High−income English−speaking countries
North Africa and Middle East
−0.05
0.00
0.05
0.10

Dependent variable: Prevalence (BMI > +2SD)	
**Independent variable: Inverse mean BMI, prevalence (15 kg/m^2 ≤ BMI <18.5 kg/m^2), prevalence (18.5 kg/m^2 ≤ BMI <20 kg/m^2), prevalence (20 kg/m^2 ≤ BMI <25 kg/m^2), prevalence (25 kg/m^2 ≤ BMI <30 kg/m^2) and prevalence (BMI ≥30 kg/m^2)**	
**Variables**	**Coefficients (95% CI)**
Intercept	-0.20 (-0.36, -0.049)
Inverse mean BMI	38.40 (35.30, 41.50)
Probit-transformed prevalence (15 kg/m^2 ≤ BMI <18.5 kg/m^2)	0.41 (0.38, 0.43)
Probit-transformed prevalence (18.5 kg/m^2 ≤ BMI <20 kg/m^2)	-0.0058 (-0.058, 0.046)
Probit-transformed prevalence (20 kg/m^2 ≤ BMI <25 kg/m^2)	0.84 (0.79, 0.89)
Probit-transformed prevalence (25 kg/m^2 ≤ BMI <30 kg/m^2)	1.13 (1.09, 1.16)
Probit-transformed prevalence (BMI ≥30 kg/m^2)	-0.33 (-0.36, -0.31)
Mean age of age group	0.060 (0.049, 0.072)
Male sex	0.011 (-0.048, 0.069)
Study mid-year (per one more recent year since 1975)	0.00096 (0.00071, 0.0012)
Natural logarithm of per-capita gross domestic product	0.043 (0.037, 0.049)
Inverse mean BMI * mean age of age group	-5.45 (-5.74, -5.15)
Probit-transformed prevalence (15 kg/m^2 ≤ BMI <18.5 kg/m^2) * mean age of age group	-0.031 (-0.033, -0.029)
Probit-transformed prevalence (18.5 kg/m^2 ≤ BMI <20 kg/m^2) * mean age of age group	-0.017 (-0.021, -0.014)
Probit-transformed prevalence (20 kg/m^2 ≤ BMI <25 kg/m^2) * mean age of age group	-0.060 (-0.064, -0.057)
Probit-transformed prevalence (25 kg/m^2 ≤ BMI <30 kg/m^2) * mean age of age group	-0.068 (-0.071, -0.065)
Probit-transformed prevalence (BMI ≥30 kg/m^2) * mean age of age group	0.054 (0.052, 0.056)
Inverse mean BMI * male sex	7.35 (6.09, 8.61)
Probit-transformed prevalence (15 kg/m^2 ≤ BMI <18.5 kg/m^2) * male sex	0.066 (0.054, 0.078)
Probit-transformed prevalence (18.5 kg/m^2 ≤ BMI <20 kg/m^2) * male sex	0.090 (0.066, 0.11)
Probit-transformed prevalence (20 kg/m^2 ≤ BMI <25 kg/m^2) * male sex	0.081 (0.056, 0.11)
Probit-transformed prevalence (25 kg/m^2 ≤ BMI <30 kg/m^2) * male sex	0.068 (0.049, 0.088)
Probit-transformed prevalence (BMI ≥30 kg/m^2) * male sex	-0.036 (-0.050, -0.022)
Random effects for regions	Yes
**Number of data points used to fit the model = 6,581**	

Traditional R^2 is not clearly defined for mixed-effect models. The pseudo-R^2 for the model, which describes the proportion of variance explained by both fixed and random factors,[12] was 0.934.

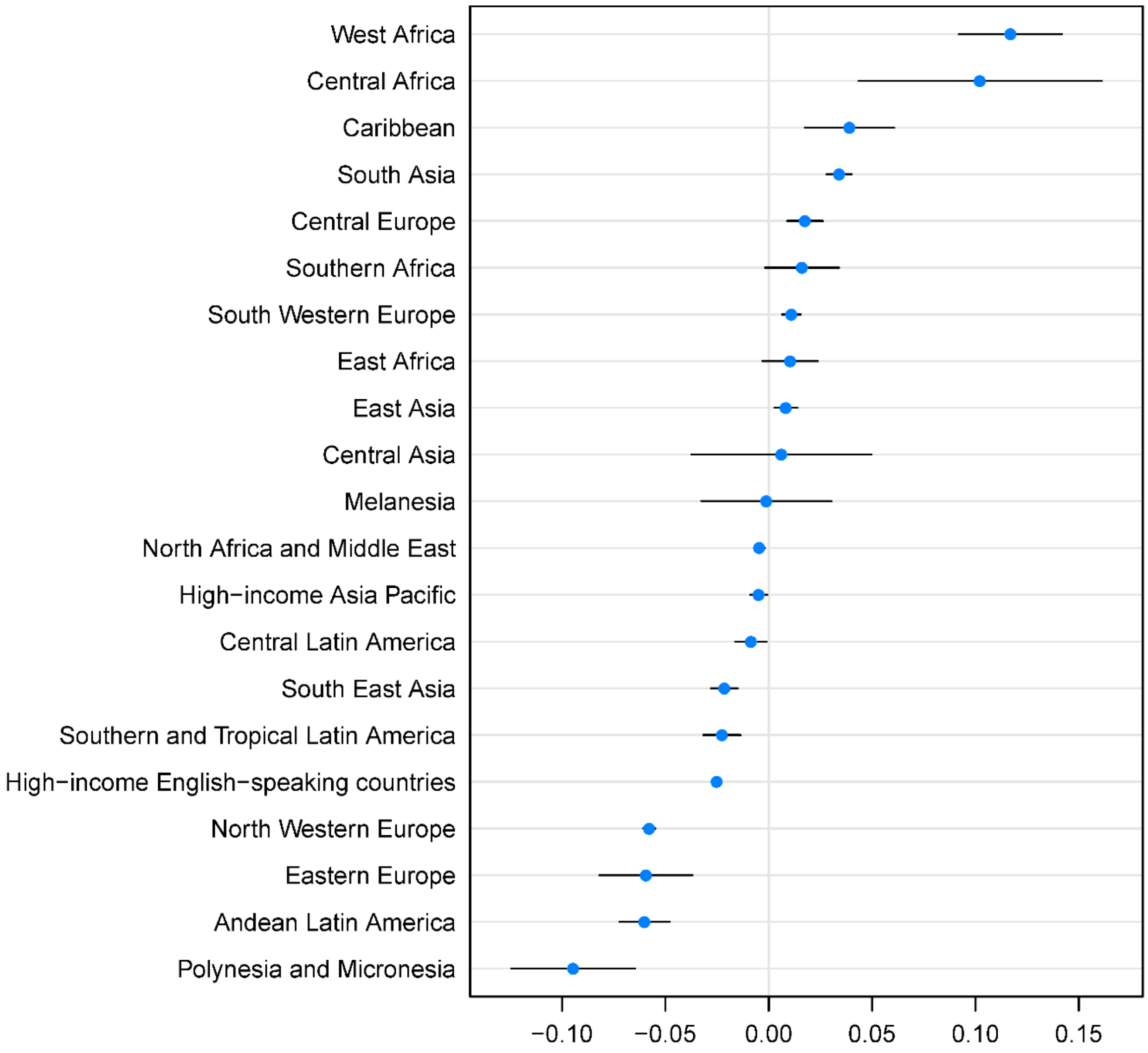

West Africa
Central Africa
Caribbean
South Asia
Central Europe
Southern Africa
South Western Europe
East Africa
East Asia
Central Asia
Melanesia
North Africa and Middle East
High−income Asia Pacific
Central Latin America
South East Asia
Southern and Tropical Latin America
High−income English−speaking countries
North Western Europe
Eastern Europe
Andean Latin America
Polynesia and Micronesia
−0.10
−0.05
0.00
0.05
0.10
0.15

**Dependent variable: Prevalence (BMI < -1SD)**	
**Independent variable: Inverse mean BMI**	
**Variables**	**Coefficients (95% CI)**
Intercept	-5.73 (-5.84, -5.62)
Inverse mean BMI	51.60 (51.10, 52.00)
Mean age of age group	-0.013 (-0.014, -0.011)
Male sex	0.16 (0.15, 0.18)
Study mid-year (per one more recent year since 1975)	-0.0014 (-0.0015, -0.0013)
Natural logarithm of per-capita gross domestic product	0.056 (0.054, 0.059)
Inverse mean BMI * mean age of age group	2.72 (2.69, 2.74)
Inverse mean BMI * male sex	-2.40 (-2.60, -2.21)
Random effects for regions	Yes
**Number of data points used to fit the model = 11,875**	

Traditional R^2 is not clearly defined for mixed-effect models. The pseudo-R^2 for the model, which describes the proportion of variance explained by both fixed and random factors,[12] was 0.905.

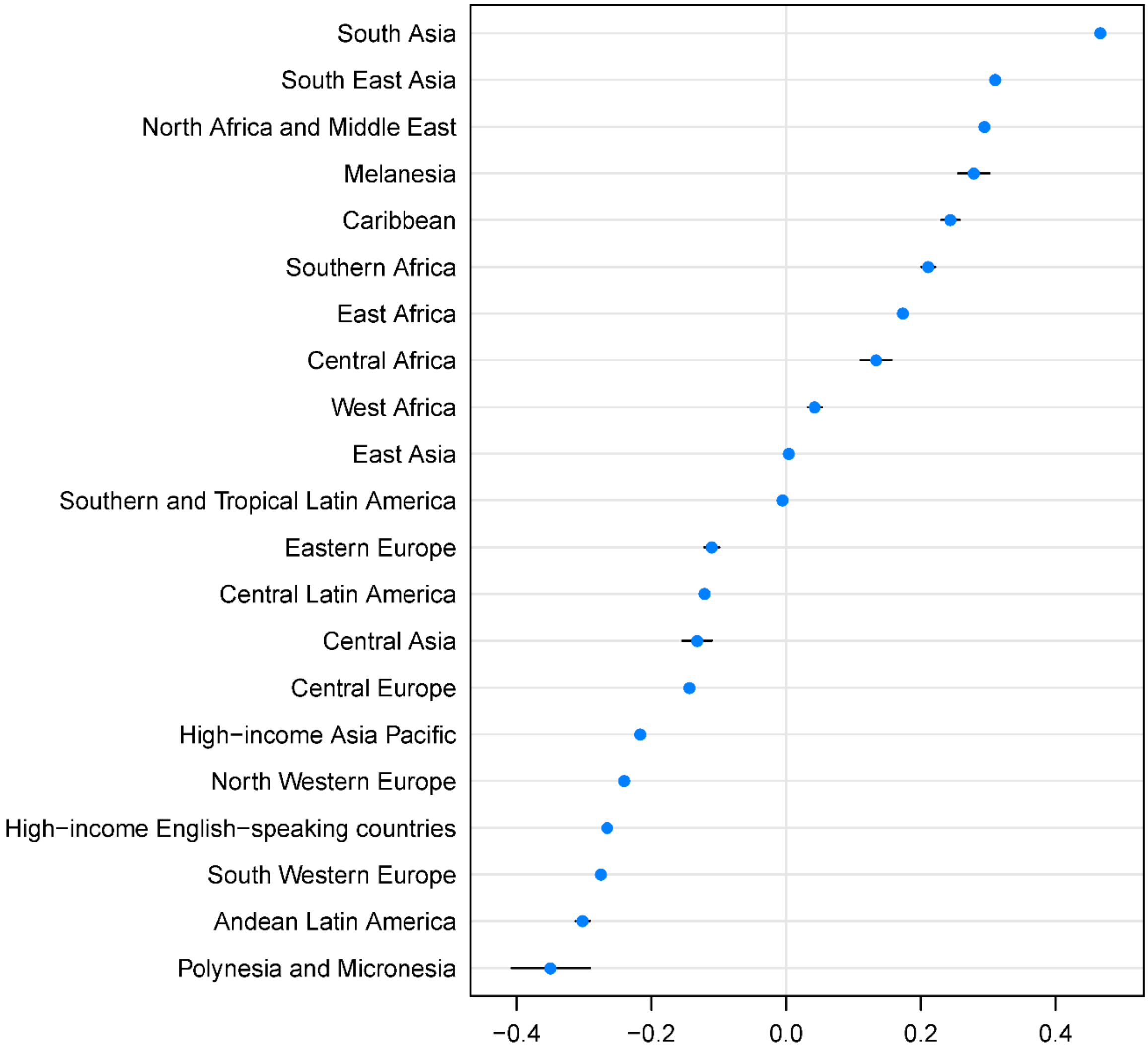
South Asia
South East Asia
North Africa and Middle East
Melanesia
Caribbean
Southern Africa
East Africa
Central Africa
West Africa
East Asia
Southern and Tropical Latin America
Eastern Europe
Central Latin America
Central Asia
Central Europe
High−income Asia Pacific
North Western Europe
High−income English−speaking countries
South Western Europe
Andean Latin America
Polynesia and Micronesia
−0.4
−0.2
0.0
0.2
0.4

**Dependent variable: Prevalence (BMI < -1SD)**	
**Independent variable: Prevalence (BMI ≥25 kg/m^2)**	
**Variables**	**Coefficients (95% CI)**
Intercept	-0.56 (-0.72, -0.40)
Probit-transformed prevalence (BMI ≥25 kg/m^2)	0.14 (0.14, 0.14)
Mean age of age group	-0.035 (-0.035, -0.034)
Male sex	0.17 (0.17, 0.18)
Study mid-year (per one more recent year since 1975)	-0.0059 (-0.0060, -0.0058)
Natural logarithm of per-capita gross domestic product	-0.0045 (-0.0072, -0.0018)
Probit-transformed prevalence (BMI ≥25 kg/m^2) * mean age of age group	-0.027 (-0.027, -0.026)
Probit-transformed prevalence (BMI ≥25 kg/m^2) * male sex	0.063 (0.061, 0.066)
Random effects for regions	Yes
**Number of data points used to fit the model = 9,548**	

Traditional R^2 is not clearly defined for mixed-effect models. The pseudo-R^2 for the model, which describes the proportion of variance explained by both fixed and random factors,[12] was 0.858.

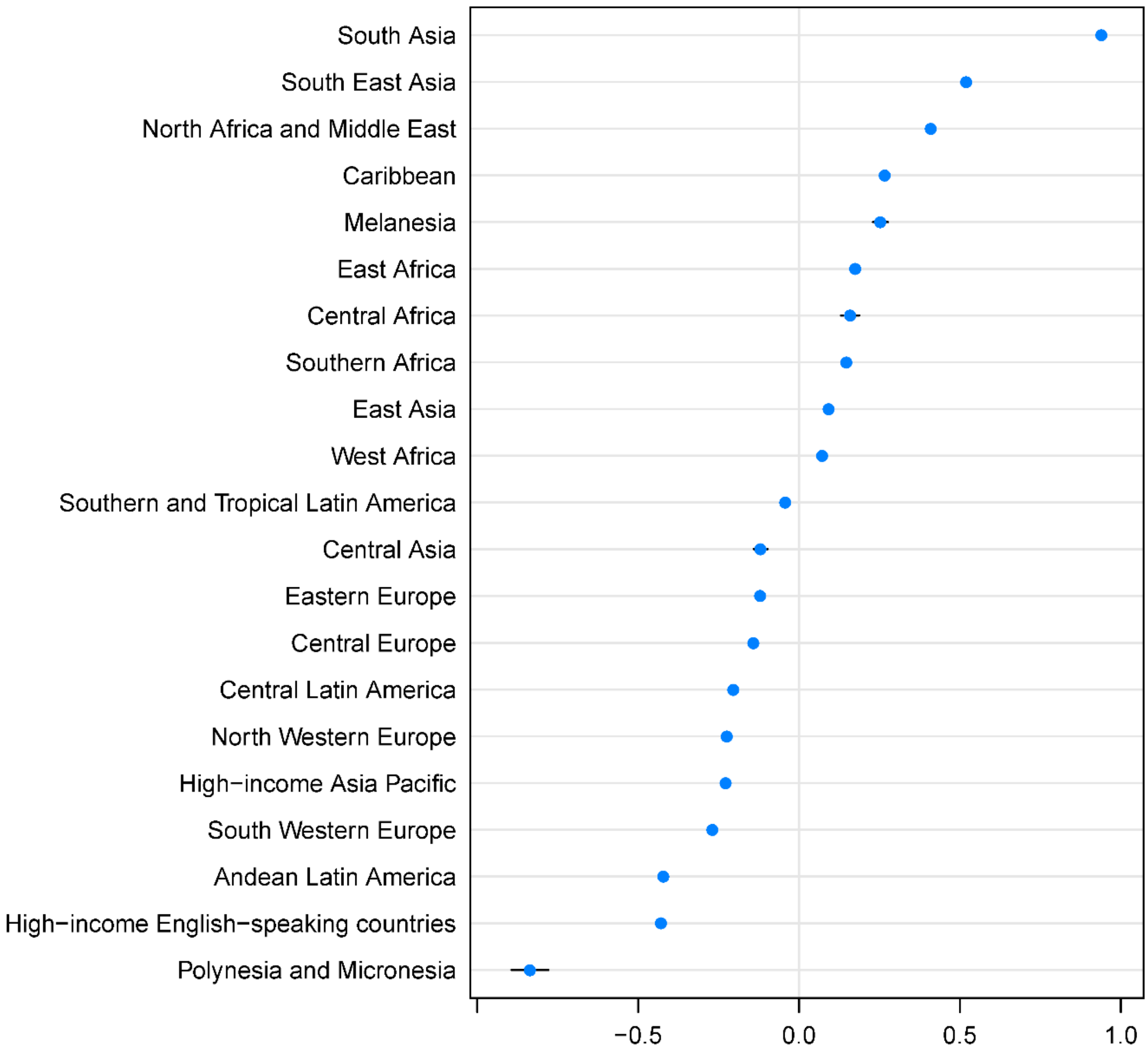
South Asia
South East Asia
North Africa and Middle East
Caribbean
Melanesia
East Africa
Central Africa
Southern Africa
East Asia
West Africa
Southern and Tropical Latin America
Central Asia
Eastern Europe
Central Europe
Central Latin America
North Western Europe
High−income Asia Pacific
South Western Europe
Andean Latin America
High−income English−speaking countries
Polynesia and Micronesia
−0.5
0.0
0.5
1.0

Dependent variable: Prevalence (BMI < -1SD)	
**Independent variable: Prevalence (BMI ≥30 kg/m^2)**	
**Variables**	**Coefficients (95% CI)**
Intercept	-0.37 (-0.54, -0.20)
Probit-transformed prevalence (BMI ≥30 kg/m^2)	0.23 (0.23, 0.23)
Mean age of age group	-0.070 (-0.070, -0.068)
Male sex	0.25 (0.24, 0.26)
Study mid-year (per one more recent year since 1975)	-0.0065 (-0.0066, -0.0063)
Natural logarithm of per-capita gross domestic product	0.025 (0.022, 0.028)
Probit-transformed prevalence (BMI ≥30 kg/m^2) * mean age of age group	-0.028 (-0.029, -0.028)
Probit-transformed prevalence (BMI ≥30 kg/m^2) * male sex	0.078 (0.075, 0.081)
Random effects for regions	Yes
**Number of data points used to fit the model = 6,786**	

Traditional R^2 is not clearly defined for mixed-effect models. The pseudo-R^2 for the model, which describes the proportion of variance explained by both fixed and random factors,[12] was 0.866.

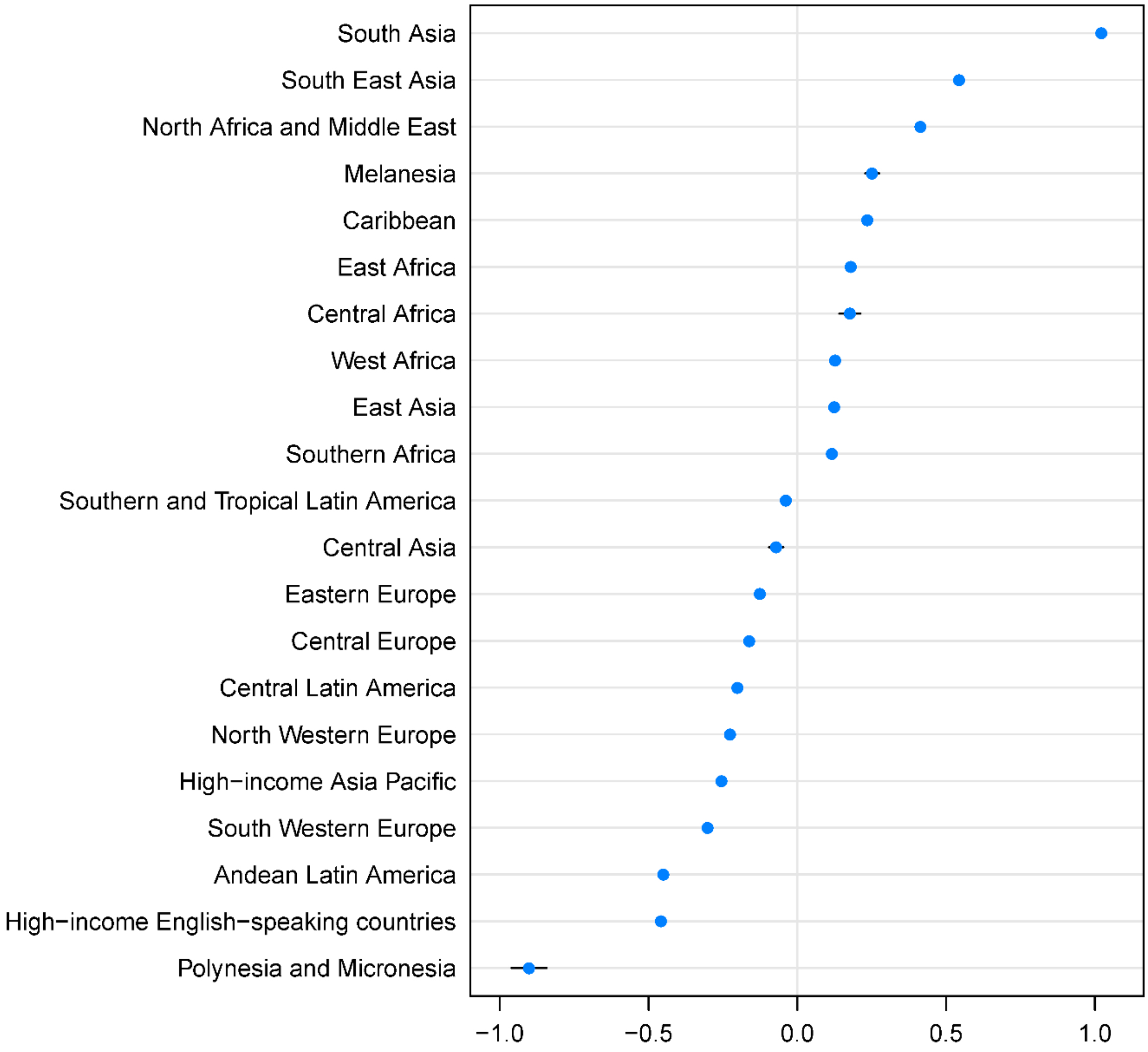
South Asia
South East Asia
North Africa and Middle East
Melanesia
Caribbean
East Africa
Central Africa
West Africa
East Asia
Southern Africa
Southern and Tropical Latin America
Central Asia
Eastern Europe
Central Europe
Central Latin America
North Western Europe
High−income Asia Pacific
South Western Europe
Andean Latin America
High−income English−speaking countries
Polynesia and Micronesia
−1.0
−0.5
0.0
0.5
1.0

**Dependent variable: Prevalence (BMI < -1SD)**	
**Independent variable: Prevalence (BMI ≥25 kg/m^2) and prevalence (BMI ≥30 kg/m^2)**	
**Variables**	**Coefficients (95% CI)**
Intercept	-0.33 (-0.47, -0.18)
Probit-transformed prevalence (BMI ≥25 kg/m^2)	-0.88 (-0.90, -0.86)
Probit-transformed prevalence (BMI ≥30 kg/m^2)	1.16 (1.14, 1.17)
Mean age of age group	-0.090 (-0.092, -0.089)
Male sex	0.16 (0.15, 0.17)
Study mid-year (per one more recent year since 1975)	-0.0027 (-0.0029, -0.0026)
Natural logarithm of per-capita gross domestic product	0.058 (0.055, 0.062)
Probit-transformed prevalence (BMI ≥25 kg/m^2) * mean age of age group	0.016 (0.014, 0.017)
Probit-transformed prevalence (BMI ≥30 kg/m^2) * mean age of age group	-0.058 (-0.060, -0.057)
Probit-transformed prevalence (BMI ≥25 kg/m^2) * male sex	0.067 (0.058, 0.076)
Probit-transformed prevalence (BMI ≥30 kg/m^2) * male sex	-4.6e-05 (-0.0098, 0.0097)
Random effects for regions	Yes
**Number of data points used to fit the model = 6,605**	

Traditional R^2 is not clearly defined for mixed-effect models. The pseudo-R^2 for the model, which describes the proportion of variance explained by both fixed and random factors,[12] was 0.885.

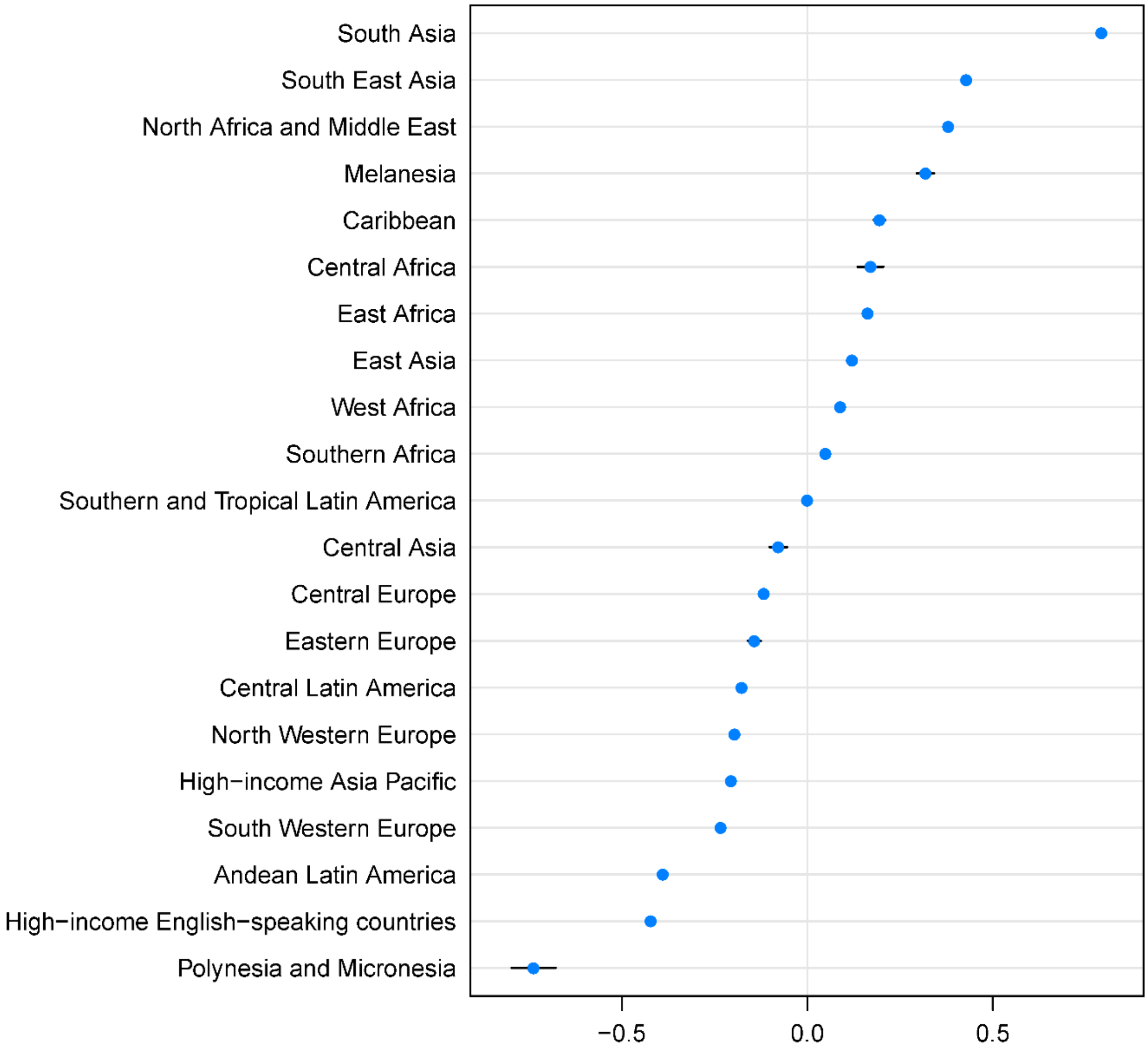
South Asia
South East Asia
North Africa and Middle East
Melanesia
Caribbean
Central Africa
East Africa
East Asia
West Africa
Southern Africa
Southern and Tropical Latin America
Central Asia
Central Europe
Eastern Europe
Central Latin America
North Western Europe
High−income Asia Pacific
South Western Europe
Andean Latin America
High−income English−speaking countries
Polynesia and Micronesia
−0.5
0.0
0.5

**Dependent variable: Prevalence (BMI < -1SD)**	
**Independent variable: Inverse mean BMI and prevalence (BMI > +1SD)**	
**Variables**	**Coefficients (95% CI)**
Intercept	-5.02 (-5.14, -4.91)
Inverse mean BMI	32.20 (31.50, 33.00)
Probit-transformed prevalence (BMI > +1SD)	-0.46 (-0.47, -0.45)
Mean age of age group	-0.034 (-0.036, -0.032)
Male sex	0.12 (0.11, 0.13)
Study mid-year (per one more recent year since 1975)	-0.00076 (-0.00088, -0.00064)
Natural logarithm of per-capita gross domestic product	0.072 (0.069, 0.074)
Inverse mean BMI * mean age of age group	3.35 (3.32, 3.38)
Probit-transformed prevalence (BMI > +1SD) * mean age of age group	0.035 (0.034, 0.035)
Inverse mean BMI * male sex	-3.84 (-4.05, -3.64)
Probit-transformed prevalence (BMI > +1SD) * male sex	-0.15 (-0.16, -0.15)
Random effects for regions	Yes
**Number of data points used to fit the model = 11,712**	

Traditional R^2 is not clearly defined for mixed-effect models. The pseudo-R^2 for the model, which describes the proportion of variance explained by both fixed and random factors,[12] was 0.921.

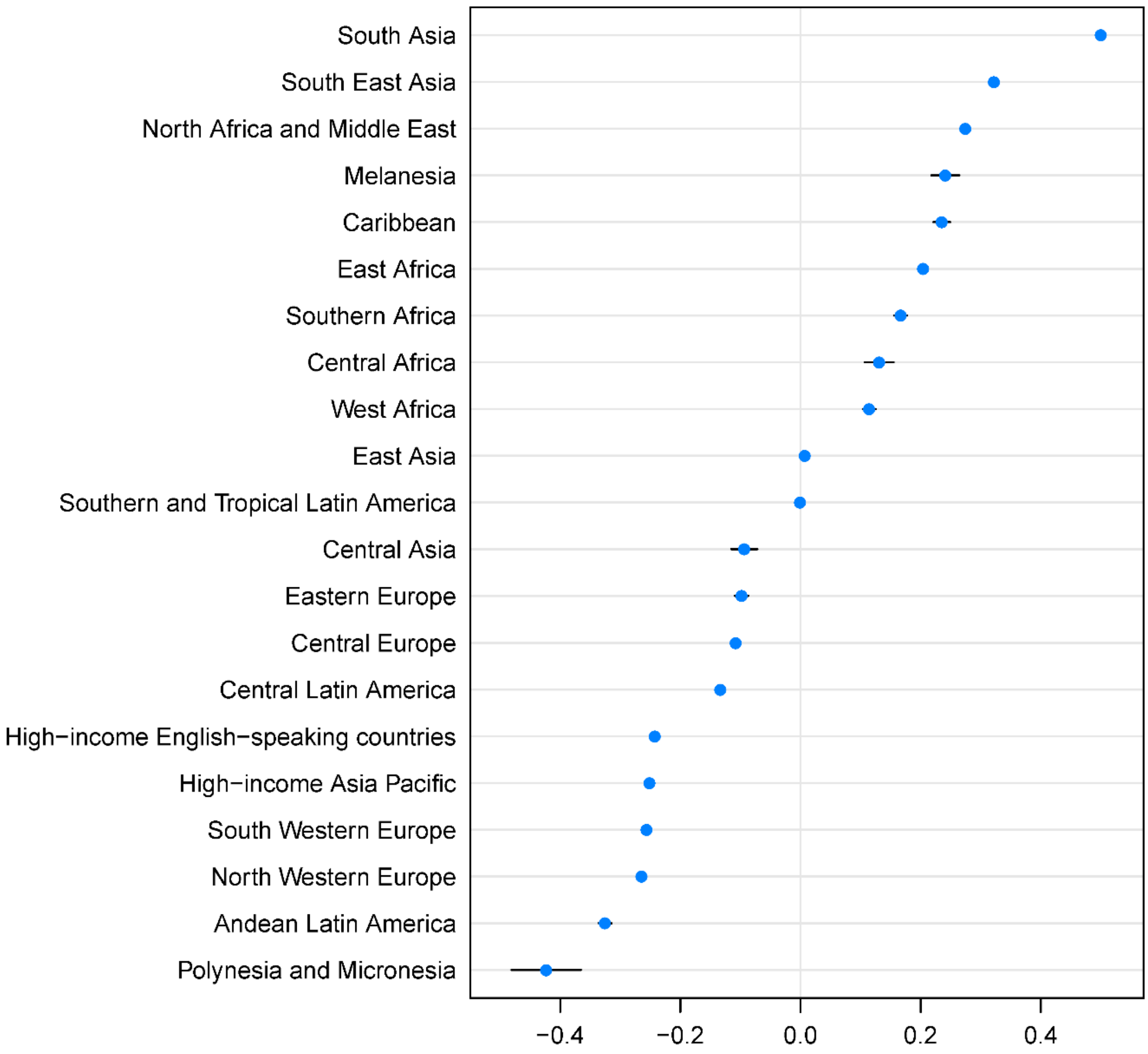
South Asia
South East Asia
North Africa and Middle East
Melanesia
Caribbean
East Africa
Southern Africa
Central Africa
West Africa
East Asia
Southern and Tropical Latin America
Central Asia
Eastern Europe
Central Europe
Central Latin America
High−income English−speaking countries
High−income Asia Pacific
South Western Europe
North Western Europe
Andean Latin America
Polynesia and Micronesia
−0.4
−0.2
0.0
0.2
0.4

Dependent variable: Prevalence (BMI < -1SD)	
**Independent variable: Inverse mean BMI, prevalence (BMI > +1SD) and prevalence (BMI > +2SD)**	
**Variables**	**Coefficients (95% CI)**
Intercept	-3.82 (-3.93, -3.71)
Inverse mean BMI	23.00 (22.30, 23.80)
Probit-transformed prevalence (BMI > +1SD)	-1.06 (-1.07, -1.04)
Probit-transformed prevalence (BMI > +2SD)	0.62 (0.60, 0.63)
Mean age of age group	-0.083 (-0.086, -0.081)
Male sex	0.31 (0.29, 0.32)
Study mid-year (per one more recent year since 1975)	-0.0012 (-0.0013, -0.0010)
Natural logarithm of per-capita gross domestic product	0.066 (0.063, 0.068)
Inverse mean BMI * mean age of age group	3.43 (3.40, 3.47)
Probit-transformed prevalence (BMI > +1SD) * mean age of age group	0.065 (0.064, 0.067)
Probit-transformed prevalence (BMI > +2SD) * mean age of age group	-0.036 (-0.037, -0.034)
Inverse mean BMI * male sex	-6.00 (-6.23, -5.77)
Probit-transformed prevalence (BMI > +1SD) * male sex	-0.27 (-0.28, -0.26)
Probit-transformed prevalence (BMI > +2SD) * male sex	0.12 (0.11, 0.13)
Random effects for regions	Yes
**Number of data points used to fit the model = 10,671**	

Traditional R^2 is not clearly defined for mixed-effect models. The pseudo-R^2 for the model, which describes the proportion of variance explained by both fixed and random factors,[12] was 0.927.

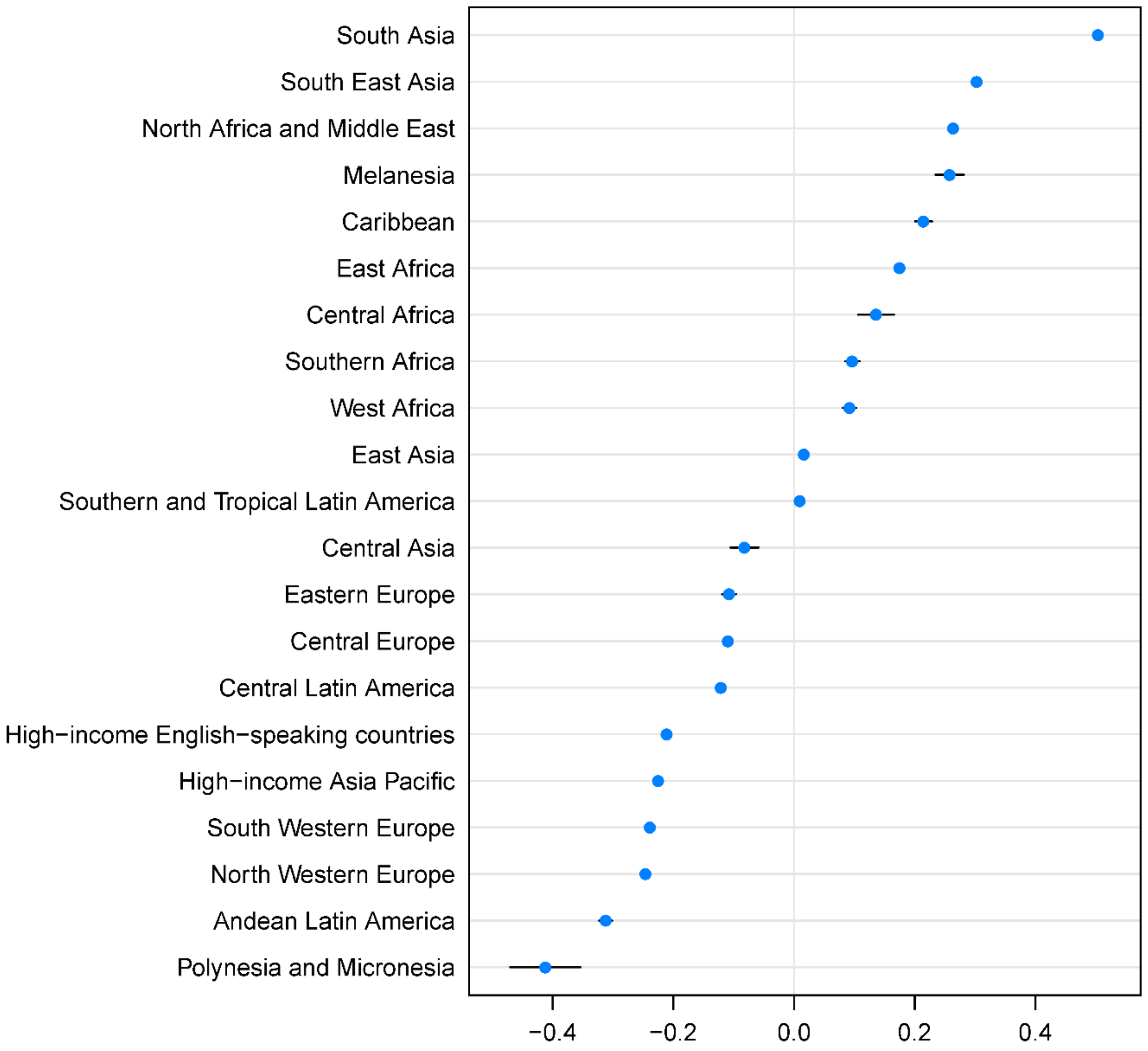
South Asia
South East Asia
North Africa and Middle East
Melanesia
Caribbean
East Africa
Central Africa
Southern Africa
West Africa
East Asia
Southern and Tropical Latin America
Central Asia
Eastern Europe
Central Europe
Central Latin America
High−income English−speaking countries
High−income Asia Pacific
South Western Europe
North Western Europe
Andean Latin America
Polynesia and Micronesia
−0.4
−0.2
0.0
0.2
0.4

**Dependent variable: Prevalence (BMI < -1SD)**	
**Independent variable: Prevalence (BMI > +1SD) and prevalence (BMI > +2SD)**	
**Variables**	**Coefficients (95% CI)**
Intercept	-1.21 (-1.34, -1.07)
Probit-transformed prevalence (BMI > +1SD)	-1.27 (-1.29, -1.26)
Probit-transformed prevalence (BMI > +2SD)	0.67 (0.66, 0.68)
Mean age of age group	0.017 (0.016, 0.018)
Male sex	-0.041 (-0.049, -0.033)
Study mid-year (per one more recent year since 1975)	-0.0033 (-0.0034, -0.0032)
Natural logarithm of per-capita gross domestic product	0.022 (0.020, 0.025)
Probit-transformed prevalence (BMI > +1SD) * mean age of age group	0.049 (0.048, 0.050)
Probit-transformed prevalence (BMI > +2SD) * mean age of age group	-0.022 (-0.023, -0.021)
Probit-transformed prevalence (BMI > +1SD) * male sex	-0.17 (-0.18, -0.16)
Probit-transformed prevalence (BMI > +2SD) * male sex	0.058 (0.048, 0.067)
Random effects for regions	Yes
**Number of data points used to fit the model = 10,699**	

Traditional R^2 is not clearly defined for mixed-effect models. The pseudo-R^2 for the model, which describes the proportion of variance explained by both fixed and random factors,[12] was 0.883.

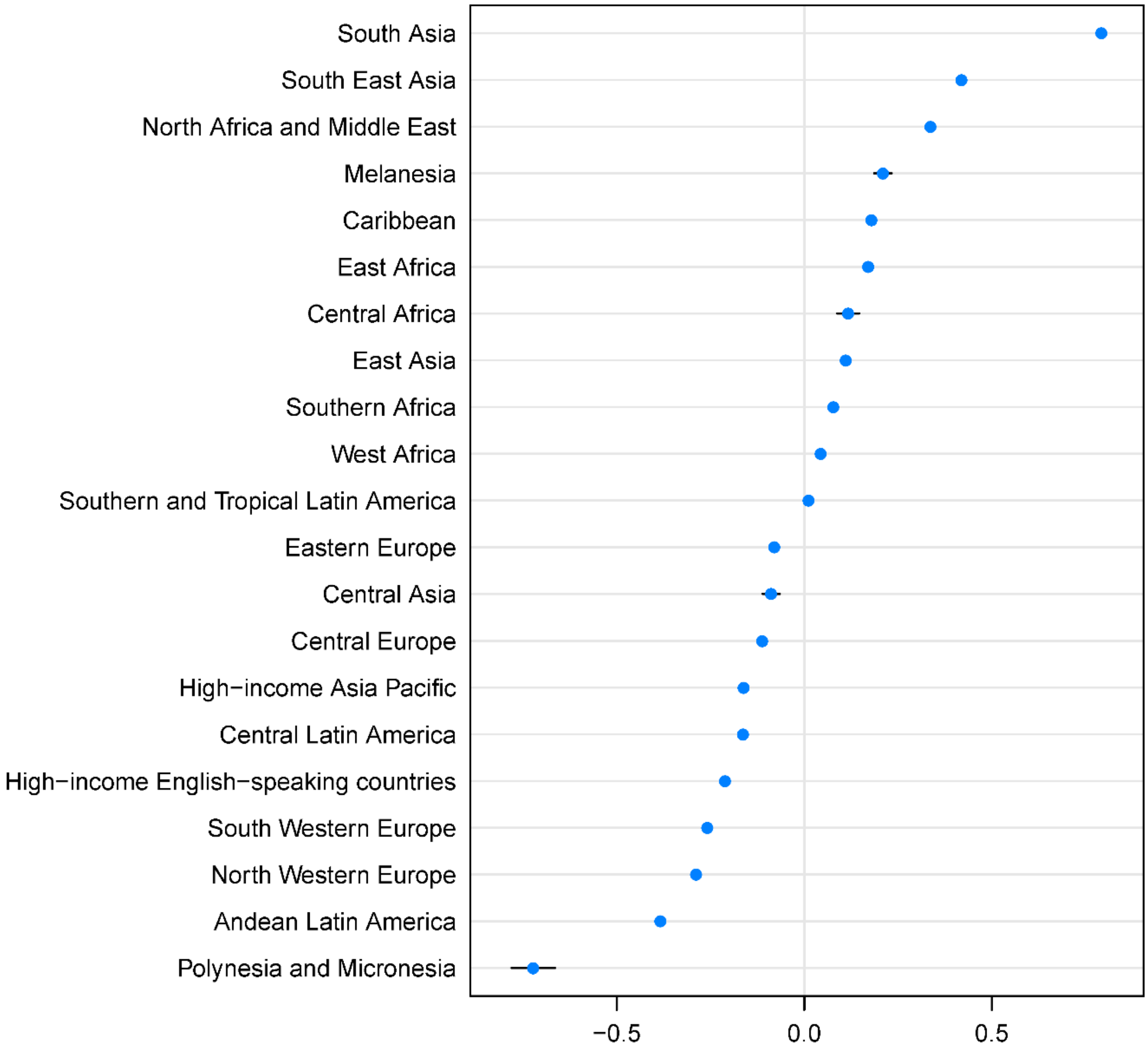

South Asia
South East Asia
North Africa and Middle East
Melanesia
Caribbean
East Africa
Central Africa
East Asia
Southern Africa
West Africa
Southern and Tropical Latin America
Eastern Europe
Central Asia
Central Europe
High−income Asia Pacific
Central Latin America
High−income English−speaking countries
South Western Europe
North Western Europe
Andean Latin America
Polynesia and Micronesia
−0.5
0.0
0.5

Dependent variable: Prevalence (BMI < -1SD)	
**Independent variable: Prevalence (BMI > +1SD) and prevalence (BMI < -2SD)**	
**Variables**	**Coefficients (95% CI)**
Intercept	-0.057 (-0.095, -0.019)
Probit-transformed prevalence (BMI > +1SD)	-0.29 (-0.30, -0.28)
Probit-transformed prevalence (BMI < -2SD)	0.78 (0.77, 0.78)
Mean age of age group	-0.016 (-0.018, -0.015)
Male sex	-0.022 (-0.031, -0.014)
Study mid-year (per one more recent year since 1975)	-0.00040 (-0.00052, -0.00028)
Natural logarithm of per-capita gross domestic product	0.034 (0.031, 0.036)
Probit-transformed prevalence (BMI > +1SD) * mean age of age group	0.0017 (0.0012, 0.0022)
Probit-transformed prevalence (BMI < -2SD) * mean age of age group	-0.0086 (-0.0090, -0.0082)
Probit-transformed prevalence (BMI > +1SD) * male sex	-0.030 (-0.033, -0.026)
Probit-transformed prevalence (BMI < -2SD) * male sex	0.0048 (0.0017, 0.0078)
Random effects for regions	Yes
**Number of data points used to fit the model = 9,943**	

Traditional R^2 is not clearly defined for mixed-effect models. The pseudo-R^2 for the model, which describes the proportion of variance explained by both fixed and random factors,[12] was 0.954.

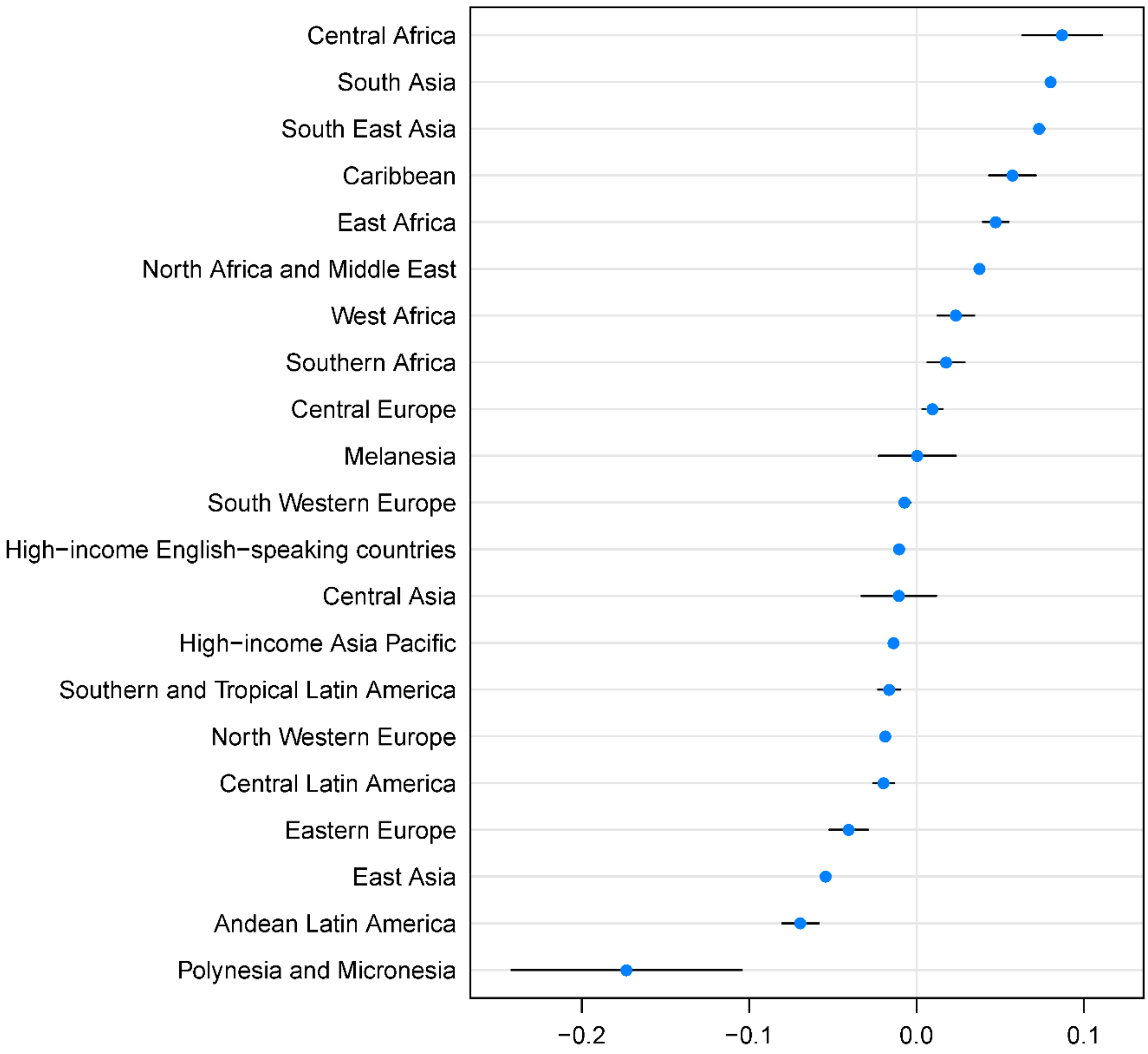

Central Africa
South Asia
South East Asia
Caribbean
East Africa
North Africa and Middle East
West Africa
Southern Africa
Central Europe
Melanesia
South Western Europe
High−income English−speaking countries
Central Asia
High−income Asia Pacific
Southern and Tropical Latin America
North Western Europe
Central Latin America
Eastern Europe
East Asia
Andean Latin America
Polynesia and Micronesia
−0.2
−0.1
0.0
0.1

Dependent variable: Prevalence (BMI < -1SD)	
**Independent variable: Prevalence (BMI > +1SD), prevalence (BMI > +2SD) and prevalence (BMI < -2SD)**	
**Variables**	**Coefficients (95% CI)**
Intercept	0.15 (0.11, 0.19)
Probit-transformed prevalence (BMI > +1SD)	-0.52 (-0.55, -0.50)
Probit-transformed prevalence (BMI > +2SD)	0.24 (0.22, 0.26)
Probit-transformed prevalence (BMI < -2SD)	0.72 (0.72, 0.73)
Mean age of age group	-0.014 (-0.015, -0.012)
Male sex	-0.057 (-0.066, -0.048)
Study mid-year (per one more recent year since 1975)	-0.00064 (-0.00077, -0.00052)
Natural logarithm of per-capita gross domestic product	0.021 (0.018, 0.023)
Probit-transformed prevalence (BMI > +1SD) * mean age of age group	0.0061 (0.0043, 0.0079)
Probit-transformed prevalence (BMI > +2SD) * mean age of age group	-0.0047 (-0.0063, -0.0032)
Probit-transformed prevalence (BMI < -2SD) * mean age of age group	-0.0060 (-0.0066, -0.0055)
Probit-transformed prevalence (BMI > +1SD) * male sex	0.074 (0.061, 0.087)
Probit-transformed prevalence (BMI > +2SD) * male sex	-0.090 (-0.10, -0.078)
Probit-transformed prevalence (BMI < -2SD) * male sex	0.025 (0.021, 0.028)
Random effects for regions	Yes
**Number of data points used to fit the model = 9,111**	

Traditional R^2 is not clearly defined for mixed-effect models. The pseudo-R^2 for the model, which describes the proportion of variance explained by both fixed and random factors,[12] was 0.956.

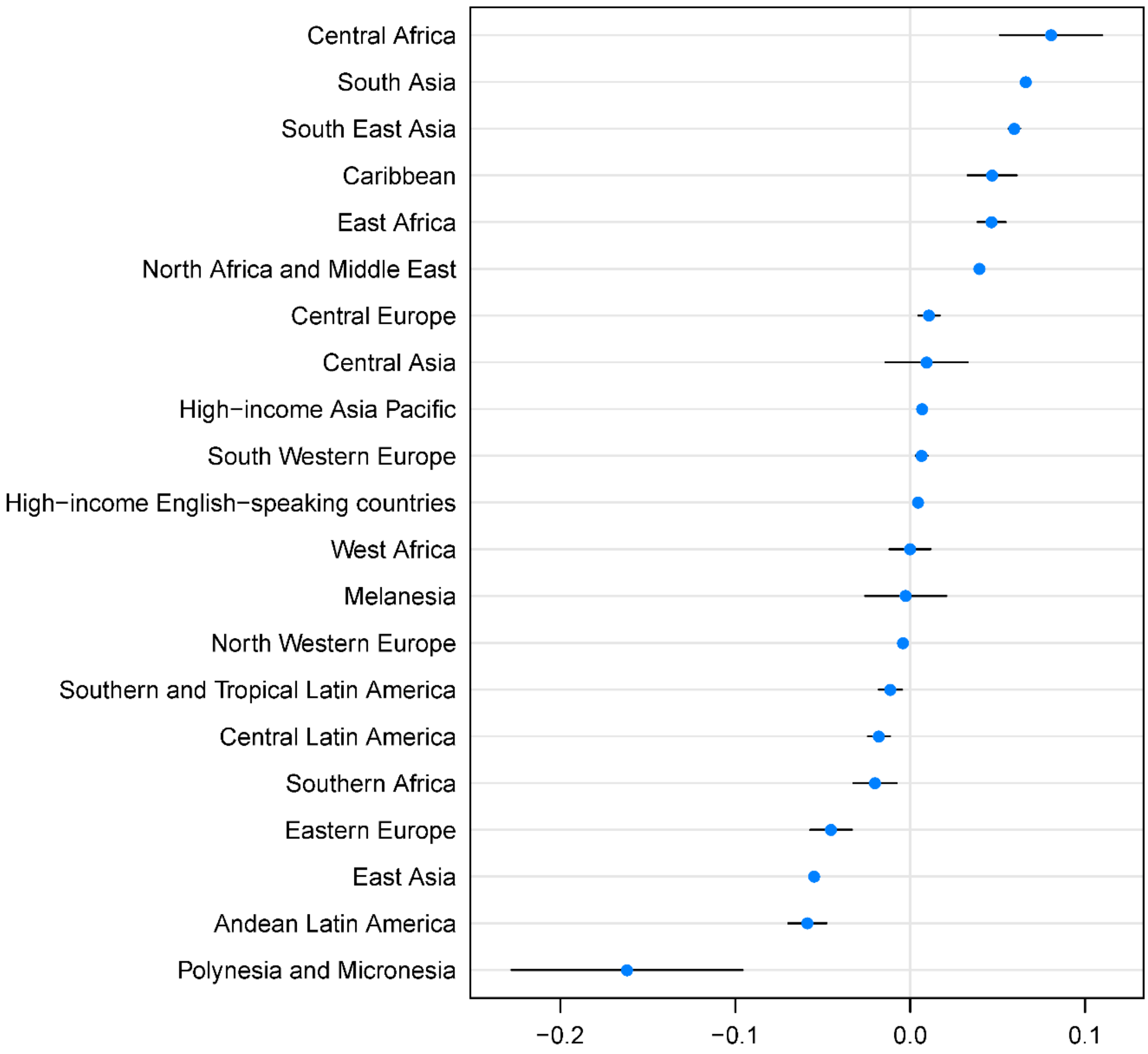
Central Africa
South Asia
South East Asia
Caribbean
East Africa
North Africa and Middle East
Central Europe
Central Asia
High−income Asia Pacific
South Western Europe
High−income English−speaking countries
West Africa
Melanesia
North Western Europe
Southern and Tropical Latin America
Central Latin America
Southern Africa
Eastern Europe
East Asia
Andean Latin America
Polynesia and Micronesia
−0.2
−0.1
0.0
0.1

**Dependent variable: Prevalence (BMI < -1SD)**	
**Independent variable: Inverse mean BMI, prevalence (17 kg/m^2 ≤ BMI < 18.5 kg/m^2) and prevalence (18.5 kg/m^2 ≤ BMI < 25 kg/m^2)**	
**Variables**	**Coefficients (95% CI)**
Intercept	-7.77 (-7.90, -7.64)
Inverse mean BMI	74.20 (72.10, 76.30)
Probit-transformed prevalence (17 kg/m^2 ≤ BMI < 18.5 kg/m^2)	-1.01 (-1.02, -0.99)
Probit-transformed prevalence (18.5 kg/m^2 ≤ BMI < 25 kg/m^2)	0.43 (0.42, 0.45)
Mean age of age group	0.22 (0.22, 0.23)
Male sex	-0.56 (-0.61, -0.51)
Study mid-year (per one more recent year since 1975)	-0.0013 (-0.0015, -0.0012)
Natural logarithm of per-capita gross domestic product	0.041 (0.037, 0.044)
Inverse mean BMI * mean age of age group	-0.23 (-0.38, -0.084)
Probit-transformed prevalence (17 kg/m^2 ≤ BMI < 18.5 kg/m^2) * mean age of age group	0.080 (0.080, 0.082)
Probit-transformed prevalence (18.5 kg/m^2 ≤ BMI < 25 kg/m^2) * mean age of age group	-0.049 (-0.050, -0.048)
Inverse mean BMI * male sex	6.38 (5.43, 7.33)
Probit-transformed prevalence (17 kg/m^2 ≤ BMI < 18.5 kg/m^2) * male sex	-0.30 (-0.30, -0.29)
Probit-transformed prevalence (18.5 kg/m^2 ≤ BMI < 25 kg/m^2) * male sex	0.15 (0.14, 0.16)
Random effects for regions	Yes
**Number of data points used to fit the model = 8,227**	

Traditional R^2 is not clearly defined for mixed-effect models. The pseudo-R^2 for the model, which describes the proportion of variance explained by both fixed and random factors,[12] was 0.949.

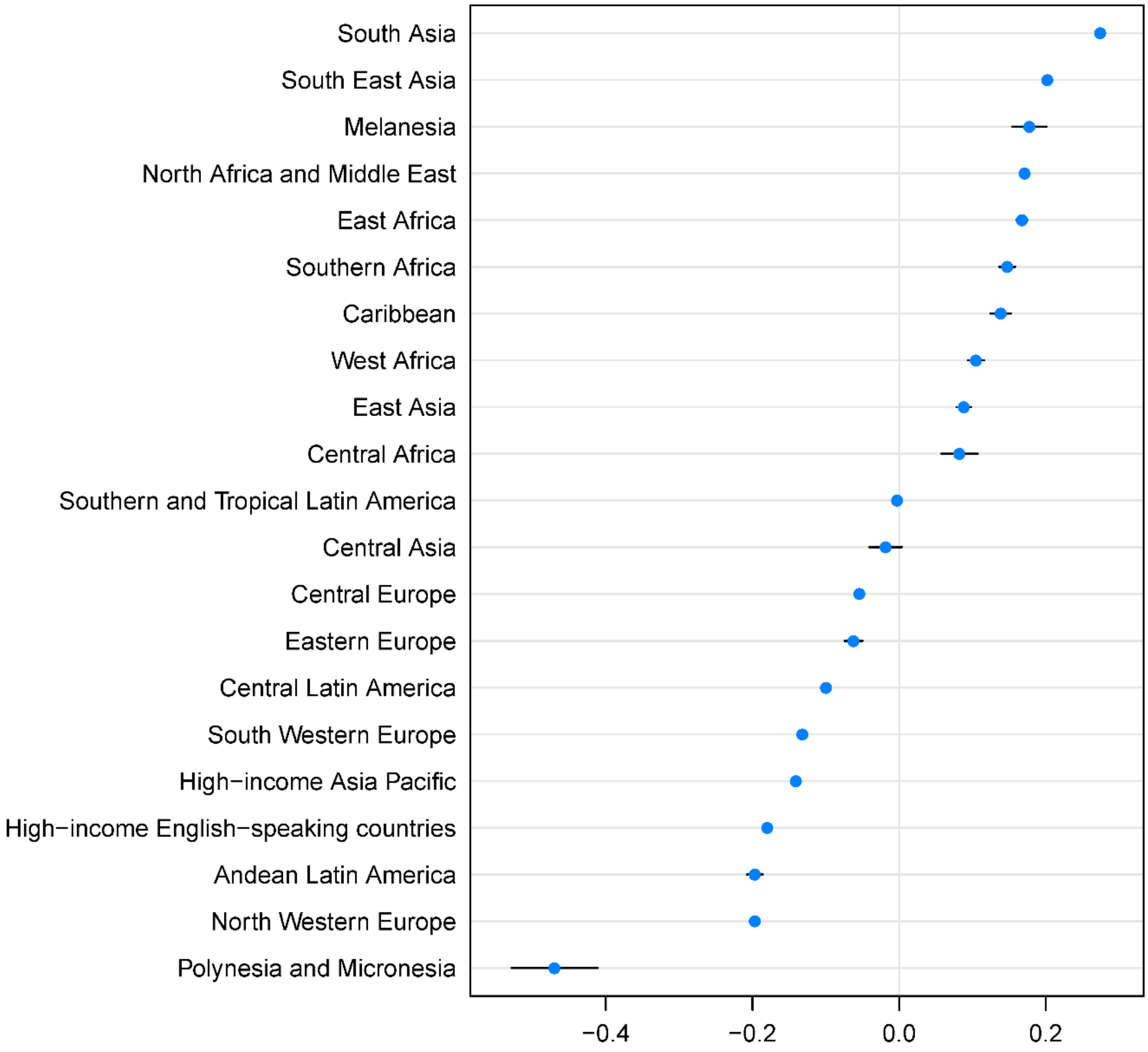
South Asia
South East Asia
Melanesia
North Africa and Middle East
East Africa
Southern Africa
Caribbean
West Africa
East Asia
Central Africa
Southern and Tropical Latin America
Central Asia
Central Europe
Eastern Europe
Central Latin America
South Western Europe
High−income Asia Pacific
High−income English−speaking countries
Andean Latin America
North Western Europe
Polynesia and Micronesia
−0.4
−0.2
0.0
0.2

Dependent variable: Prevalence (BMI < -1SD)	
**Independent variable: Inverse mean BMI and prevalence (BMI <18.5 kg/m^2)**	
**Variables**	**Coefficients (95% CI)**
Intercept	-11.90 (-12.00, -11.80)
Inverse mean BMI	171.00 (169.00, 172.00)
Probit-transformed prevalence (BMI <18.5 kg/m^2)	-0.80 (-0.81, -0.79)
Mean age of age group	0.66 (0.65, 0.67)
Male sex	-2.18 (-2.22, -2.13)
Study mid-year (per one more recent year since 1975)	-0.0026 (-0.0027, -0.0025)
Natural logarithm of per-capita gross domestic product	0.027 (0.025, 0.030)
Inverse mean BMI * mean age of age group	-9.68 (-9.81, -9.54)
Probit-transformed prevalence (BMI <18.5 kg/m^2) * mean age of age group	0.089 (0.088, 0.090)
Inverse mean BMI * male sex	42.00 (41.10, 42.90)
Probit-transformed prevalence (BMI <18.5 kg/m^2) * male sex	-0.35 (-0.36, -0.34)
Random effects for regions	Yes
**Number of data points used to fit the model = 10,844**	

Traditional R^2 is not clearly defined for mixed-effect models. The pseudo-R^2 for the model, which describes the proportion of variance explained by both fixed and random factors,[12] was 0.935.

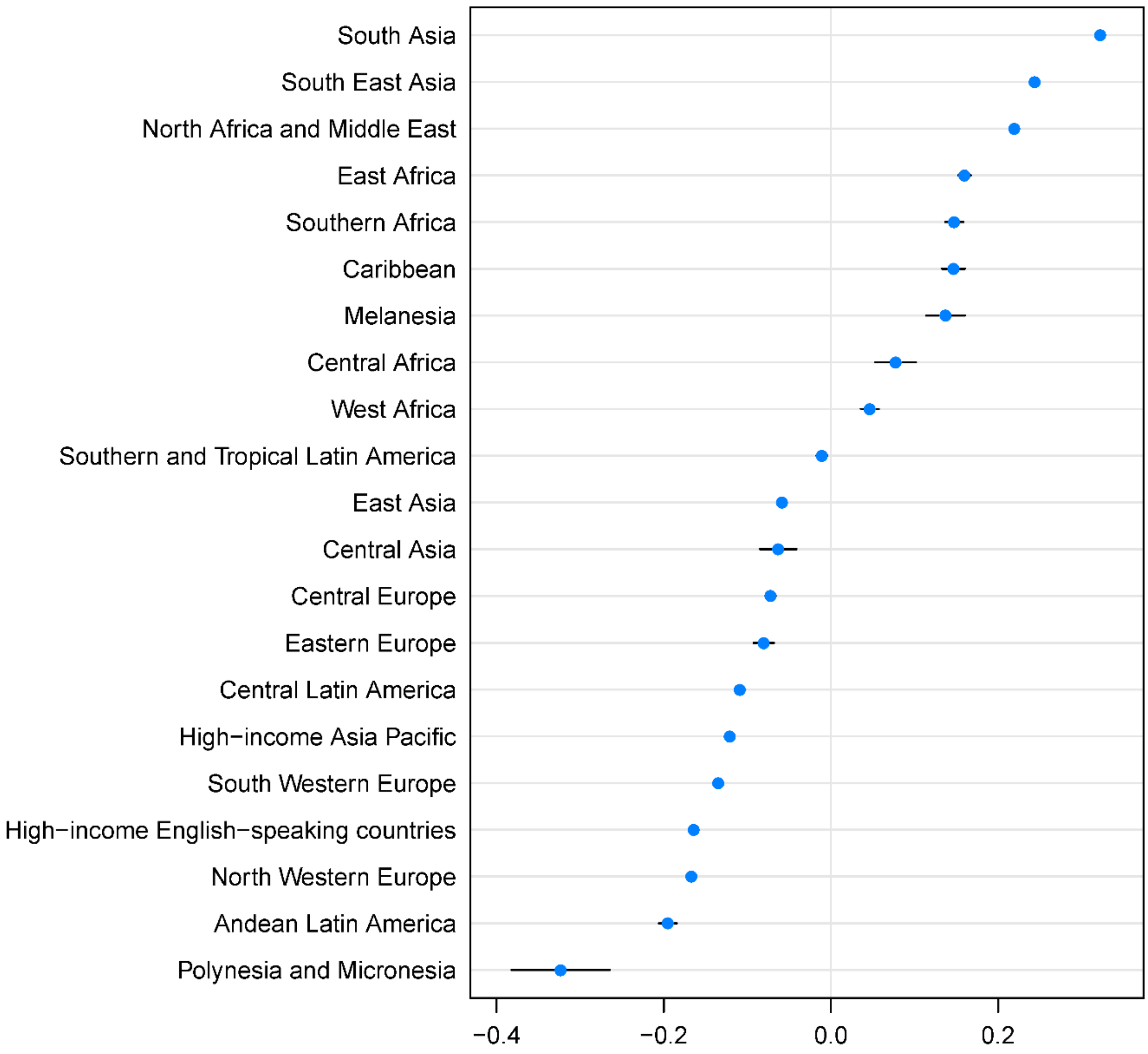

South Asia
South East Asia
North Africa and Middle East
East Africa
Southern Africa
Caribbean
Melanesia
Central Africa
West Africa
Southern and Tropical Latin America
East Asia
Central Asia
Central Europe
Eastern Europe
Central Latin America
High−income Asia Pacific
South Western Europe
High−income English−speaking countries
North Western Europe
Andean Latin America
Polynesia and Micronesia
−0.4
−0.2
0.0
0.2

Dependent variable: Prevalence (BMI < -1SD)	
**Independent variable: Inverse mean BMI, prevalence (BMI <15 kg/m²) and prevalence (15 kg/m² ≤ BMI < 18.5 kg/m²)**	
**Variables**	**Coefficients (95% CI)**
Intercept	-5.11 (-5.26, -4.96)
Inverse mean BMI	46.00 (44.10, 47.80)
Probit-transformed prevalence (BMI <15 kg/m²)	0.26 (0.24, 0.28)
Probit-transformed prevalence (15 kg/m² ≤ BMI < 18.5 kg/m²)	-0.53 (-0.54, -0.52)
Mean age of age group	0.11 (0.10, 0.12)
Male sex	-1.69 (-1.73, -1.64)
Study mid-year (per one more recent year since 1975)	-0.00066 (-0.00080, -0.00053)
Natural logarithm of per-capita gross domestic product	0.035 (0.033, 0.038)
Inverse mean BMI * mean age of age group	0.78 (0.66, 0.90)
Probit-transformed prevalence (BMI <15 kg/m²) * mean age of age group	-0.0050 (-0.0061, -0.0039)
Probit-transformed prevalence (15 kg/m² ≤ BMI < 18.5 kg/m²) * mean age of age group	0.050 (0.049, 0.050)
Inverse mean BMI * male sex	26.40 (25.60, 27.10)
Probit-transformed prevalence (BMI <15 kg/m²) * male sex	-0.18 (-0.19, -0.18)
Probit-transformed prevalence (15 kg/m² ≤ BMI < 18.5 kg/m²) * male sex	-0.15 (-0.15, -0.14)
Random effects for regions	Yes
**Number of data points used to fit the model = 8,060**	

Traditional R^2 is not clearly defined for mixed-effect models. The pseudo-R^2 for the model, which describes the proportion of variance explained by both fixed and random factors,[12] was 0.953.

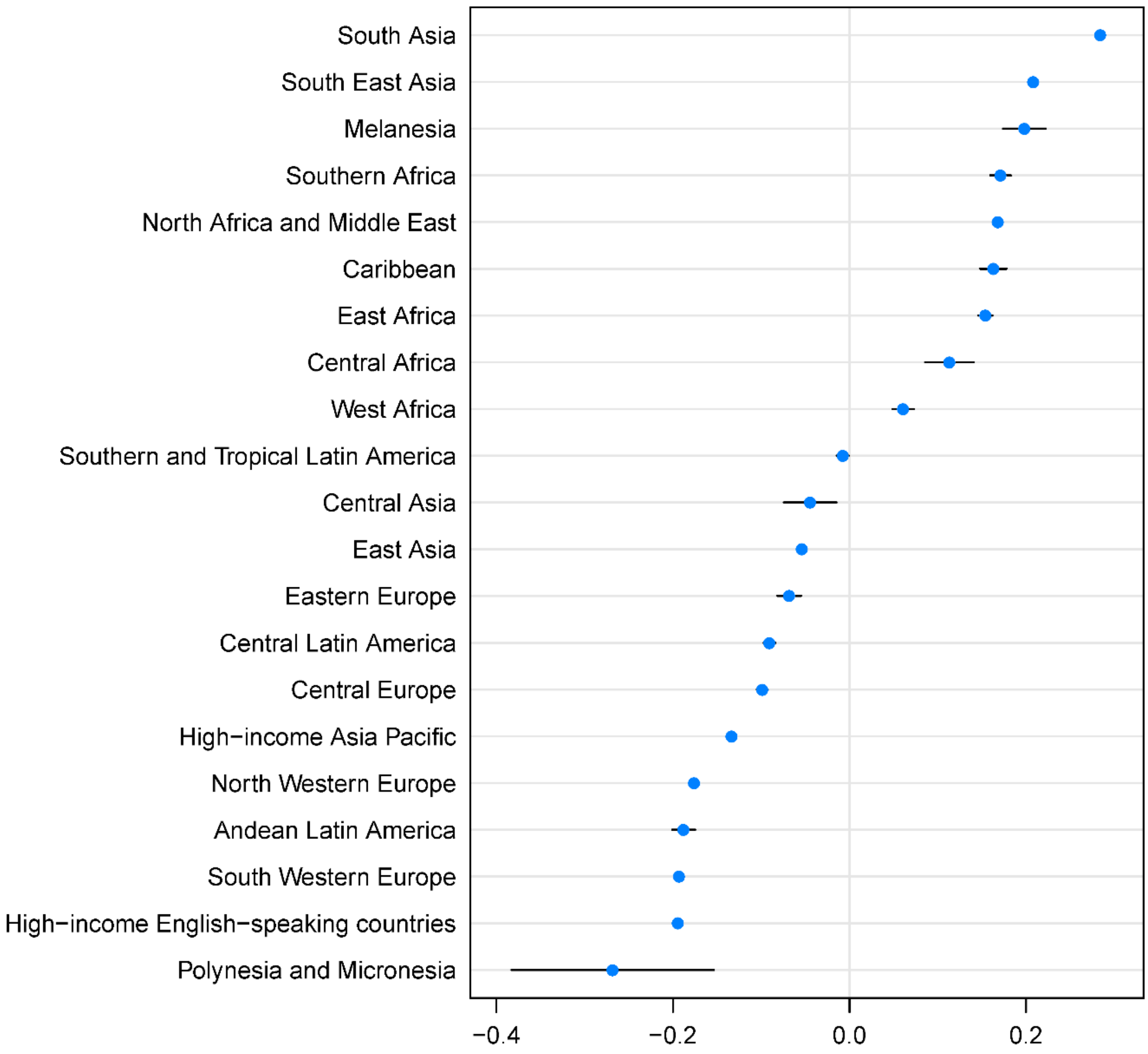
South Asia
South East Asia
Melanesia
Southern Africa
North Africa and Middle East
Caribbean
East Africa
Central Africa
West Africa
Southern and Tropical Latin America
Central Asia
East Asia
Eastern Europe
Central Latin America
Central Europe
High−income Asia Pacific
North Western Europe
Andean Latin America
South Western Europe
High−income English−speaking countries
Polynesia and Micronesia
−0.4
−0.2
0.0
0.2

**Dependent variable: Prevalence (BMI < -2SD)**	
**Independent variable: Inverse mean BMI**	
**Variables**	**Coefficients (95% CI)**
Intercept	-4.89 (-5.05, -4.74)
Inverse mean BMI	24.70 (24.00, 25.40)
Mean age of age group	-0.098 (-0.10, -0.095)
Male sex	0.25 (0.23, 0.27)
Study mid-year (per one more recent year since 1975)	-0.00084 (-0.0010, -0.00064)
Natural logarithm of per-capita gross domestic product	0.080 (0.077, 0.084)
Inverse mean BMI * mean age of age group	3.57 (3.53, 3.62)
Inverse mean BMI * male sex	-2.43 (-2.73, -2.13)
Random effects for regions	Yes
**Number of data points used to fit the model = 10,081**	

Traditional R^2 is not clearly defined for mixed-effect models. The pseudo-R^2 for the model, which describes the proportion of variance explained by both fixed and random factors,[12] was 0.910.

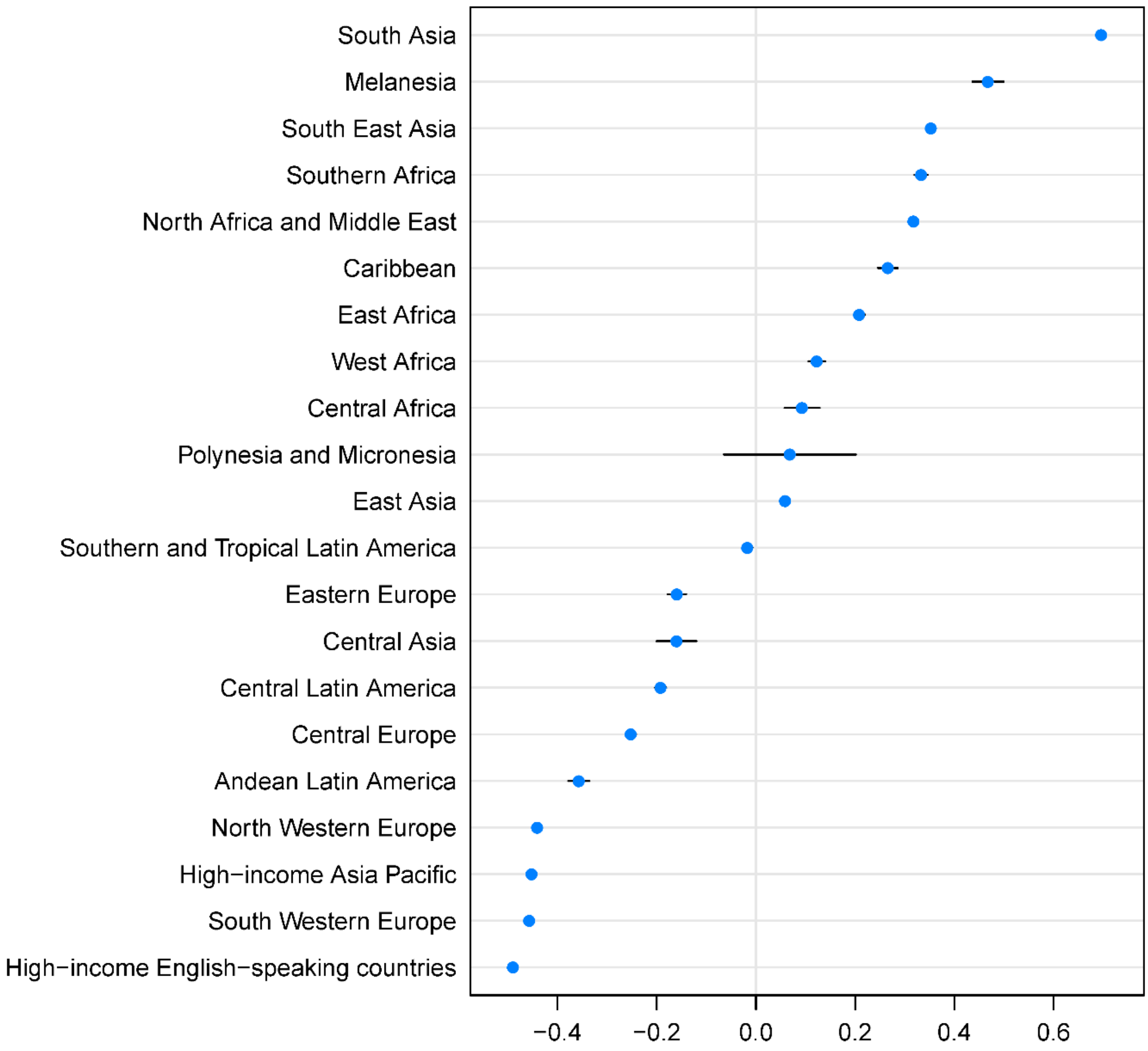
South Asia
Melanesia
South East Asia
Southern Africa
North Africa and Middle East
Caribbean
East Africa
West Africa
Central Africa
Polynesia and Micronesia
East Asia
Southern and Tropical Latin America
Eastern Europe
Central Asia
Central Latin America
Central Europe
Andean Latin America
North Western Europe
High−income Asia Pacific
South Western Europe
High−income English−speaking countries
−0.4
−0.2
0.0
0.2
0.4
0.6

**Dependent variable: Prevalence (BMI < -2SD)**	
**Independent variable: Prevalence (BMI ≥25 kg/m^2)**	
**Variables**	**Coefficients (95% CI)**
Intercept	-1.00 (-1.18, -0.82)
Probit-transformed prevalence (BMI ≥25 kg/m^2)	0.29 (0.29, 0.30)
Mean age of age group	-0.071 (-0.072, -0.070)
Male sex	0.26 (0.26, 0.27)
Study mid-year (per one more recent year since 1975)	-0.0049 (-0.0051, -0.0047)
Natural logarithm of per-capita gross domestic product	0.010 (0.0060, 0.014)
Probit-transformed prevalence (BMI ≥25 kg/m^2) * mean age of age group	-0.033 (-0.034, -0.033)
Probit-transformed prevalence (BMI ≥25 kg/m^2) * male sex	0.068 (0.064, 0.072)
Random effects for regions	Yes
**Number of data points used to fit the model = 8,160**	

Traditional R^2 is not clearly defined for mixed-effect models. The pseudo-R^2 for the model, which describes the proportion of variance explained by both fixed and random factors,[12] was 0.884.

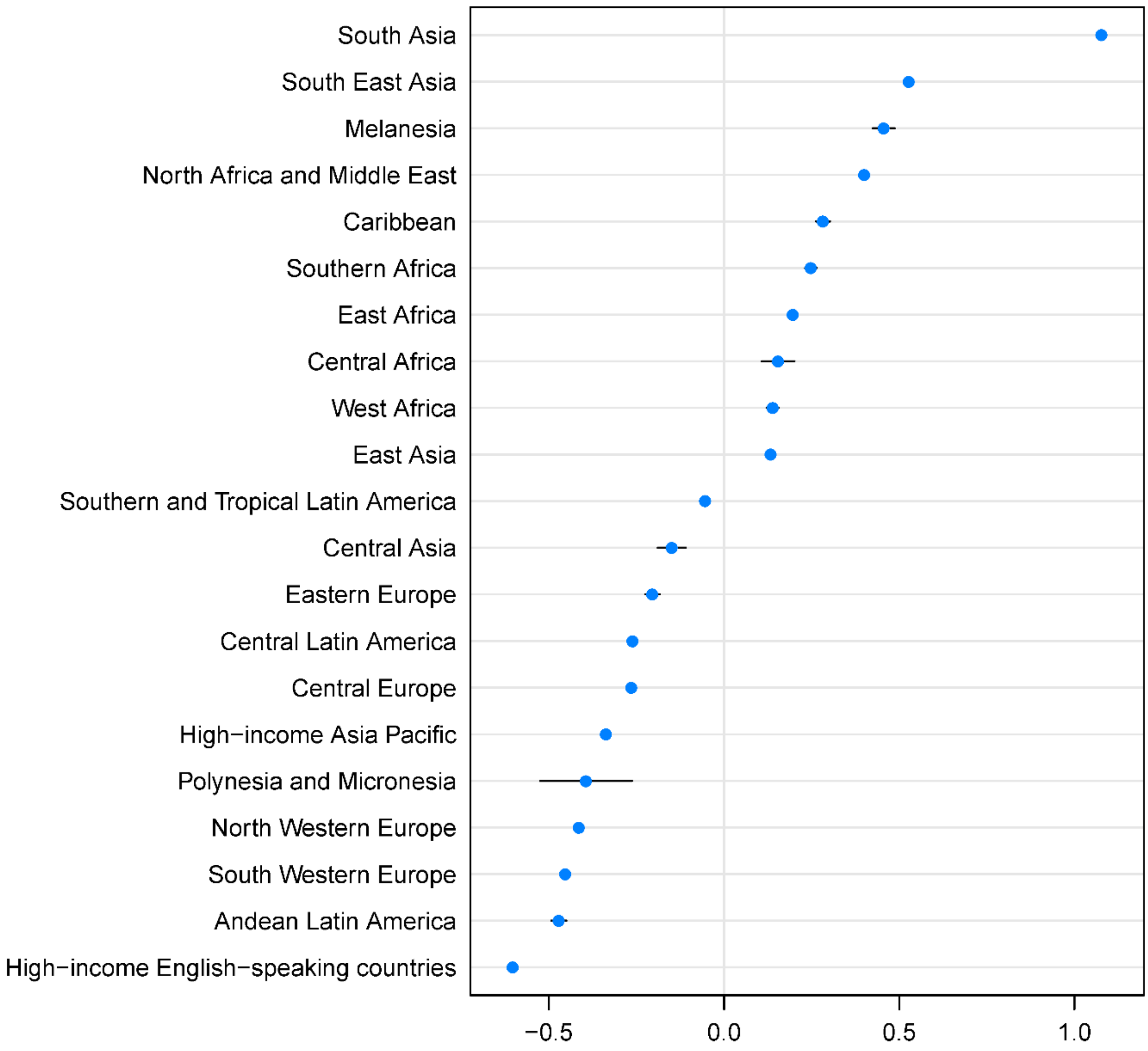

South Asia
South East Asia
Melanesia
North Africa and Middle East
Caribbean
Southern Africa
East Africa
Central Africa
West Africa
East Asia
Southern and Tropical Latin America
Central Asia
Eastern Europe
Central Latin America
Central Europe
High−income Asia Pacific
Polynesia and Micronesia
North Western Europe
South Western Europe
Andean Latin America
High−income English−speaking countries
−0.5
0.0
0.5
1.0

Dependent variable: Prevalence (BMI < -2SD)	
**Independent variable: Prevalence (BMI ≥30 kg/m^2)**	
**Variables**	**Coefficients (95% CI)**
Intercept	-0.64 (-0.83, -0.46)
Probit-transformed prevalence (BMI ≥30 kg/m^2)	0.37 (0.36, 0.38)
Mean age of age group	-0.10 (-0.10, -0.10)
Male sex	0.35 (0.33, 0.36)
Study mid-year (per one more recent year since 1975)	-0.0046 (-0.0049, -0.0044)
Natural logarithm of per-capita gross domestic product	0.021 (0.016, 0.026)
Probit-transformed prevalence (BMI ≥30 kg/m^2) * mean age of age group	-0.033 (-0.033, -0.032)
Probit-transformed prevalence (BMI ≥30 kg/m^2) * male sex	0.086 (0.081, 0.090)
Random effects for regions	Yes
**Number of data points used to fit the model = 5,879**	

Traditional R^2 is not clearly defined for mixed-effect models. The pseudo-R^2 for the model, which describes the proportion of variance explained by both fixed and random factors,[12] was 0.895.

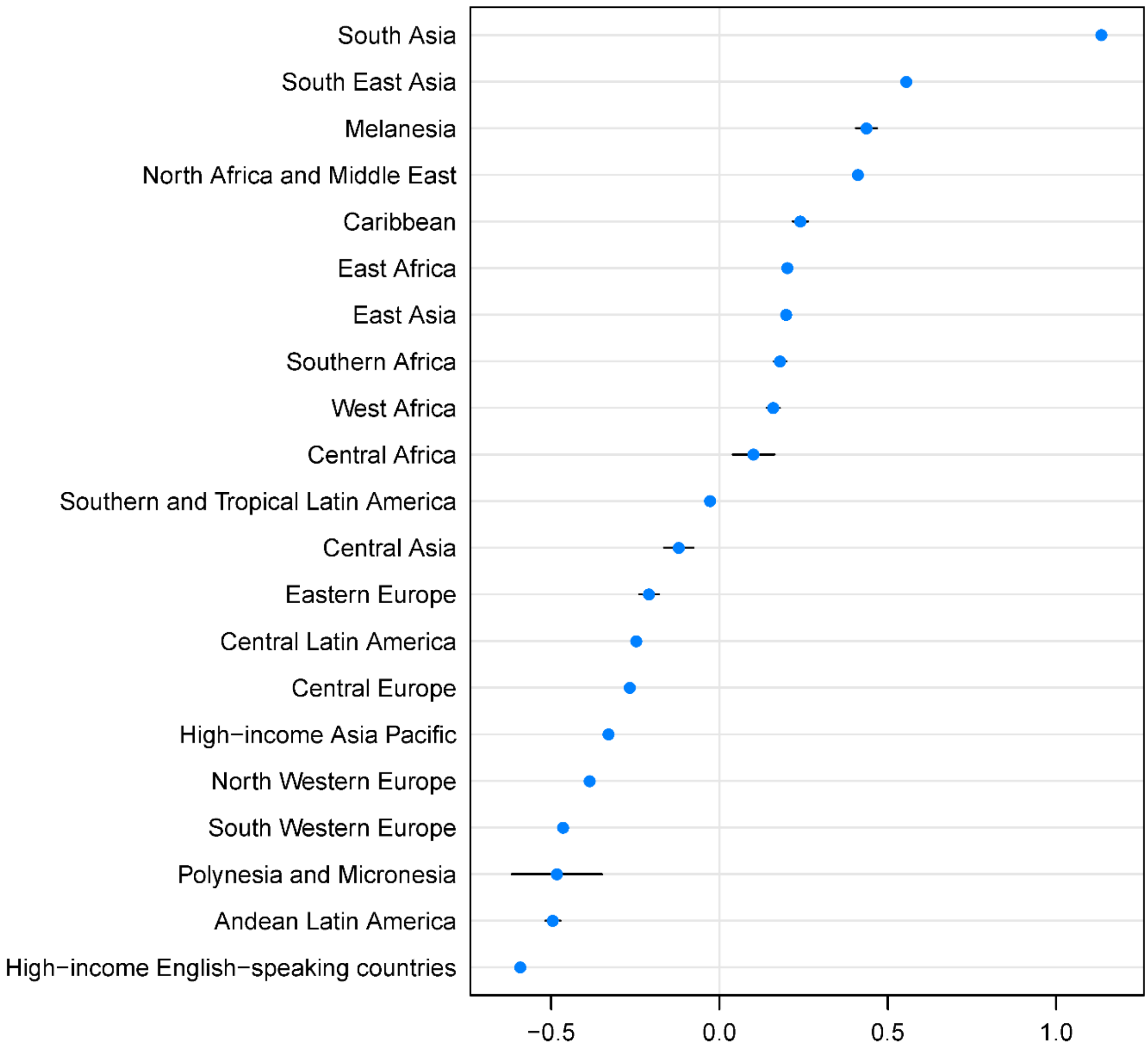

South Asia
South East Asia
Melanesia
North Africa and Middle East
Caribbean
East Africa
East Asia
Southern Africa
West Africa
Central Africa
Southern and Tropical Latin America
Central Asia
Eastern Europe
Central Latin America
Central Europe
High−income Asia Pacific
North Western Europe
South Western Europe
Polynesia and Micronesia
Andean Latin America
High−income English−speaking countries
−0.5
0.0
0.5
1.0

Dependent variable: Prevalence (BMI < -2SD)	
**Independent variable: Prevalence (BMI ≥25 kg/m^2) and prevalence (BMI ≥30 kg/m^2)**	
**Variables**	**Coefficients (95% CI)**
Intercept	-0.60 (-0.76, -0.44)
Probit-transformed prevalence (BMI ≥25 kg/m^2)	-0.41 (-0.44, -0.39)
Probit-transformed prevalence (BMI ≥30 kg/m^2)	0.83 (0.80, 0.85)
Mean age of age group	-0.097 (-0.099, -0.095)
Male sex	0.34 (0.33, 0.36)
Study mid-year (per one more recent year since 1975)	-0.0027 (-0.0030, -0.0025)
Natural logarithm of per-capita gross domestic product	0.033 (0.028, 0.038)
Probit-transformed prevalence (BMI ≥25 kg/m^2) * mean age of age group	-0.019 (-0.021, -0.017)
Probit-transformed prevalence (BMI ≥30 kg/m^2) * mean age of age group	-0.025 (-0.027, -0.023)
Probit-transformed prevalence (BMI ≥25 kg/m^2) * male sex	-0.030 (-0.042, -0.017)
Probit-transformed prevalence (BMI ≥30 kg/m^2) * male sex	0.11 (0.098, 0.12)
Random effects for regions	Yes
**Number of data points used to fit the model = 5,744**	

Traditional R^2 is not clearly defined for mixed-effect models. The pseudo-R^2 for the model, which describes the proportion of variance explained by both fixed and random factors,[12] was 0.909.

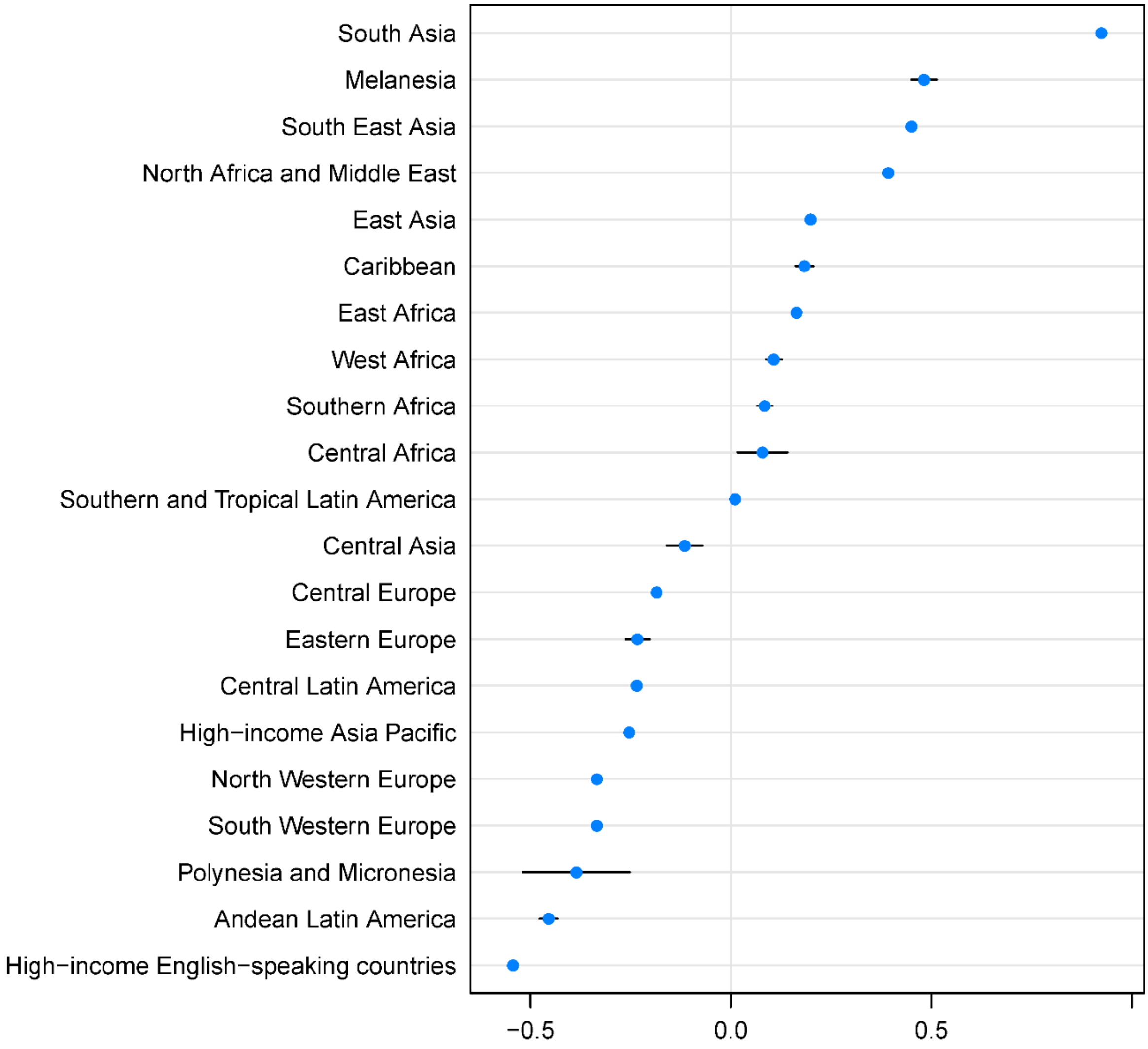
South Asia
Melanesia
South East Asia
North Africa and Middle East
East Asia
Caribbean
East Africa
West Africa
Southern Africa
Central Africa
Southern and Tropical Latin America
Central Asia
Central Europe
Eastern Europe
Central Latin America
High−income Asia Pacific
North Western Europe
South Western Europe
Polynesia and Micronesia
Andean Latin America
High−income English−speaking countries
−0.5
0.0
0.5

**Dependent variable: Prevalence (BMI < -2SD)**	
**Independent variable: Inverse mean BMI and prevalence (BMI > +1SD)**	
**Variables**	**Coefficients (95% CI)**
Intercept	-5.09 (-5.24, -4.93)
Inverse mean BMI	22.30 (21.20, 23.40)
Probit-transformed prevalence (BMI > +1SD)	-0.33 (-0.34, -0.32)
Mean age of age group	-0.079 (-0.082, -0.076)
Male sex	0.18 (0.16, 0.20)
Study mid-year (per one more recent year since 1975)	-0.00026 (-0.00046, -5.8e-05)
Natural logarithm of per-capita gross domestic product	0.065 (0.061, 0.069)
Inverse mean BMI * mean age of age group	4.14 (4.10, 4.19)
Probit-transformed prevalence (BMI > +1SD) * mean age of age group	0.038 (0.037, 0.039)
Inverse mean BMI * male sex	-4.05 (-4.37, -3.73)
Probit-transformed prevalence (BMI > +1SD) * male sex	-0.15 (-0.16, -0.15)
Random effects for regions	Yes
**Number of data points used to fit the model = 9,928**	

Traditional R^2 is not clearly defined for mixed-effect models. The pseudo-R^2 for the model, which describes the proportion of variance explained by both fixed and random factors,[12] was 0.923.

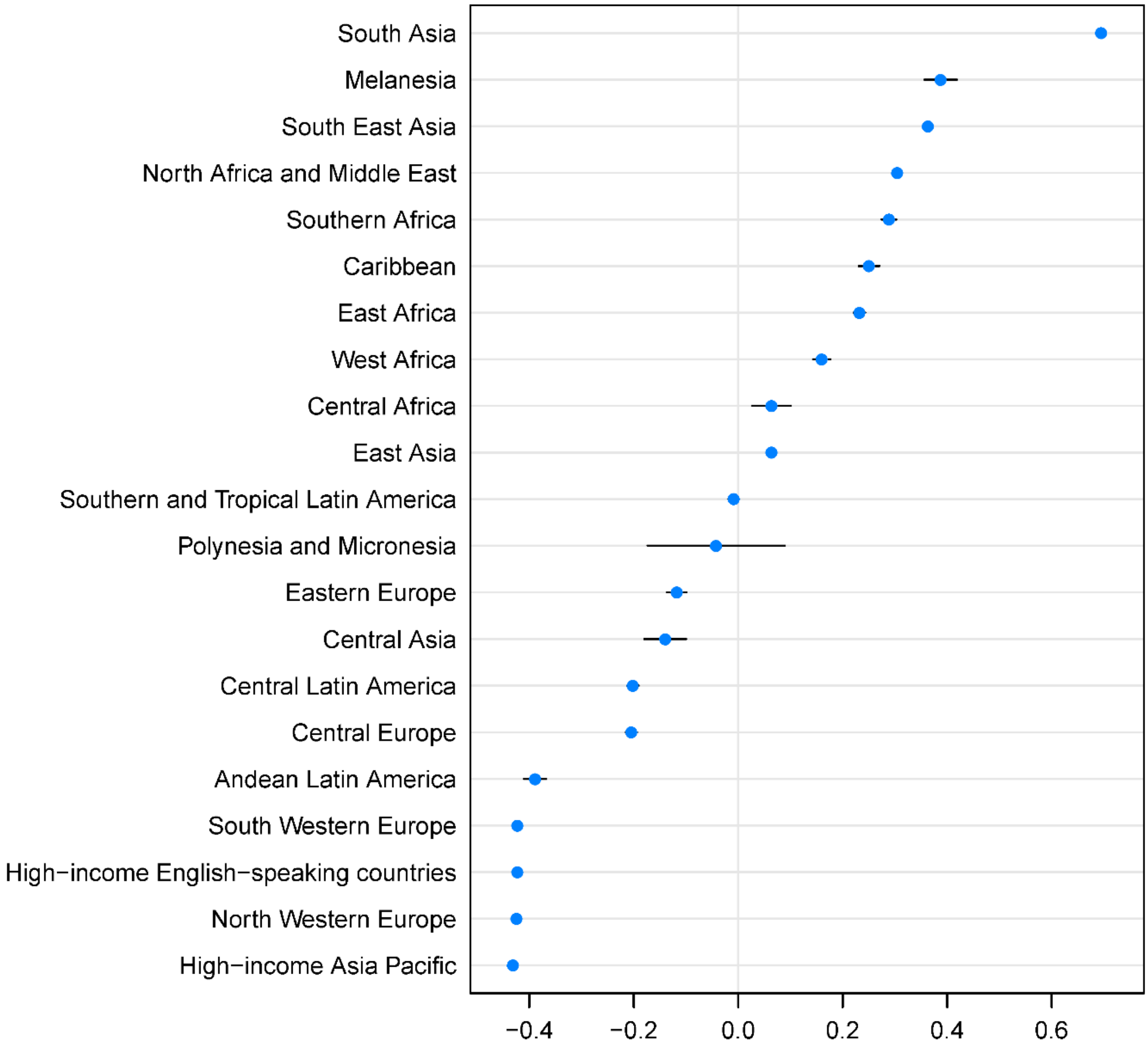
South Asia
Melanesia
South East Asia
North Africa and Middle East
Southern Africa
Caribbean
East Africa
West Africa
Central Africa
East Asia
Southern and Tropical Latin America
Polynesia and Micronesia
Eastern Europe
Central Asia
Central Latin America
Central Europe
Andean Latin America
South Western Europe
High−income English−speaking countries
North Western Europe
High−income Asia Pacific
−0.4
−0.2
0.0
0.2
0.4
0.6

**Dependent variable: Prevalence (BMI < -2SD)**	
**Independent variable: Inverse mean BMI, prevalence (BMI > +1SD) and prevalence (BMI > +2SD)**	
**Variables**	**Coefficients (95% CI)**
Intercept	-4.04 (-4.19, -3.88)
Inverse mean BMI	13.70 (12.50, 14.90)
Probit-transformed prevalence (BMI > +1SD)	-0.86 (-0.89, -0.83)
Probit-transformed prevalence (BMI > +2SD)	0.55 (0.53, 0.58)
Mean age of age group	-0.14 (-0.14, -0.14)
Male sex	0.60 (0.57, 0.63)
Study mid-year (per one more recent year since 1975)	-0.00061 (-0.00081, -0.00040)
Natural logarithm of per-capita gross domestic product	0.059 (0.055, 0.063)
Inverse mean BMI * mean age of age group	4.57 (4.52, 4.62)
Probit-transformed prevalence (BMI > +1SD) * mean age of age group	0.074 (0.072, 0.076)
Probit-transformed prevalence (BMI > +2SD) * mean age of age group	-0.039 (-0.041, -0.037)
Inverse mean BMI * male sex	-8.48 (-8.87, -8.10)
Probit-transformed prevalence (BMI > +1SD) * male sex	-0.43 (-0.45, -0.42)
Probit-transformed prevalence (BMI > +2SD) * male sex	0.28 (0.26, 0.30)
Random effects for regions	Yes
**Number of data points used to fit the model = 9,096**	

Traditional R^2 is not clearly defined for mixed-effect models. The pseudo-R^2 for the model, which describes the proportion of variance explained by both fixed and random factors,[12] was 0.927.

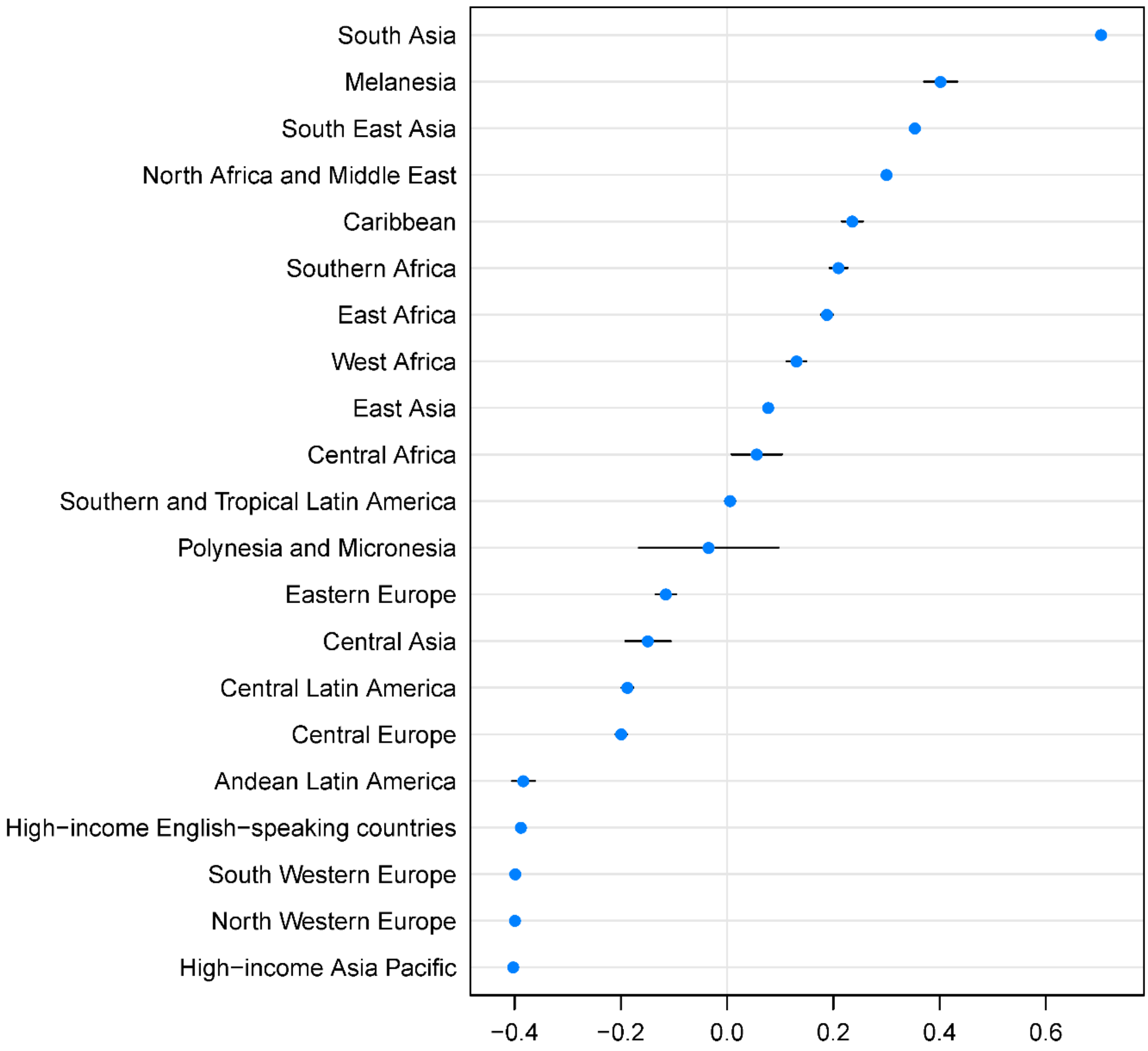

South Asia
Melanesia
South East Asia
North Africa and Middle East
Caribbean
Southern Africa
East Africa
West Africa
East Asia
Central Africa
Southern and Tropical Latin America
Polynesia and Micronesia
Eastern Europe
Central Asia
Central Latin America
Central Europe
Andean Latin America
High−income English−speaking countries
South Western Europe
North Western Europe
High−income Asia Pacific
−0.4
−0.2
0.0
0.2
0.4
0.6

Dependent variable: Prevalence (BMI < -2SD)	
**Independent variable: Prevalence (BMI > +1SD) and prevalence (BMI > +2SD)**	
**Variables**	**Coefficients (95% CI)**
Intercept	-1.99 (-2.14, -1.83)
Probit-transformed prevalence (BMI > +1SD)	-0.53 (-0.55, -0.51)
Probit-transformed prevalence (BMI > +2SD)	0.066 (0.044, 0.089)
Mean age of age group	0.042 (0.040, 0.044)
Male sex	0.087 (0.075, 0.099)
Study mid-year (per one more recent year since 1975)	-0.00026 (-0.00045, -6.8e-05)
Natural logarithm of per-capita gross domestic product	-0.026 (-0.030, -0.022)
Probit-transformed prevalence (BMI > +1SD) * mean age of age group	0.014 (0.013, 0.016)
Probit-transformed prevalence (BMI > +2SD) * mean age of age group	0.014 (0.013, 0.016)
Probit-transformed prevalence (BMI > +1SD) * male sex	-0.24 (-0.25, -0.22)
Probit-transformed prevalence (BMI > +2SD) * male sex	0.13 (0.12, 0.14)
Random effects for regions	Yes
**Number of data points used to fit the model = 9,426**	

Traditional R^2 is not clearly defined for mixed-effect models. The pseudo-R^2 for the model, which describes the proportion of variance explained by both fixed and random factors,[12] was 0.886.

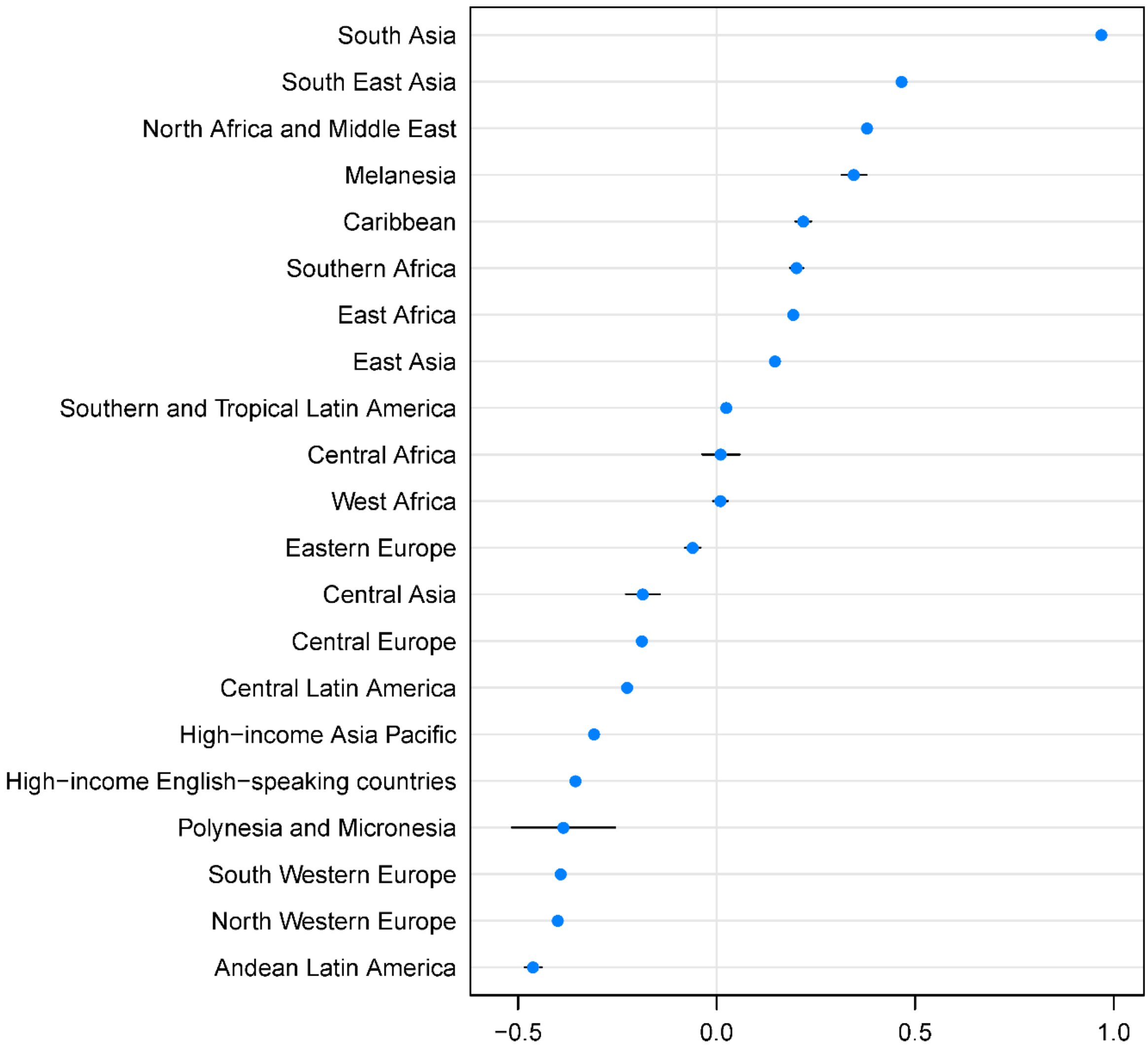
South Asia
South East Asia
North Africa and Middle East
Melanesia
Caribbean
Southern Africa
East Africa
East Asia
Southern and Tropical Latin America
Central Africa
West Africa
Eastern Europe
Central Asia
Central Europe
Central Latin America
High−income Asia Pacific
High−income English−speaking countries
Polynesia and Micronesia
South Western Europe
North Western Europe
Andean Latin America
−0.5
0.0
0.5
1.0

Dependent variable: Prevalence (BMI < -2SD)	
**Independent variable: Inverse mean BMI, prevalence (BMI > +1SD) and prevalence (BMI < -1SD)**	
**Variables**	**Coefficients (95% CI)**
Intercept	0.36 (0.26, 0.46)
Inverse mean BMI	-19.10 (-20.30, -17.80)
Probit-transformed prevalence (BMI > +1SD)	0.13 (0.11, 0.14)
Probit-transformed prevalence (BMI < -1SD)	1.07 (1.05, 1.08)
Mean age of age group	-0.090 (-0.094, -0.085)
Male sex	0.035 (0.010, 0.060)
Study mid-year (per one more recent year since 1975)	0.0014 (0.0012, 0.0016)
Natural logarithm of per-capita gross domestic product	-0.00070 (-0.0047, 0.0033)
Inverse mean BMI * mean age of age group	1.57 (1.51, 1.63)
Probit-transformed prevalence (BMI > +1SD) * mean age of age group	0.0034 (0.0024, 0.0042)
Probit-transformed prevalence (BMI < -1SD) * mean age of age group	-0.0062 (-0.0070, -0.0053)
Inverse mean BMI * male sex	0.16 (-0.20, 0.53)
Probit-transformed prevalence (BMI > +1SD) * male sex	-0.0070 (-0.013, -0.00079)
Probit-transformed prevalence (BMI < -1SD) * male sex	0.00015 (-0.0064, 0.0067)
Random effects for regions	Yes
**Number of data points used to fit the model = 9,925**	

Traditional R^2 is not clearly defined for mixed-effect models. The pseudo-R^2 for the model, which describes the proportion of variance explained by both fixed and random factors,[12] was 0.953.

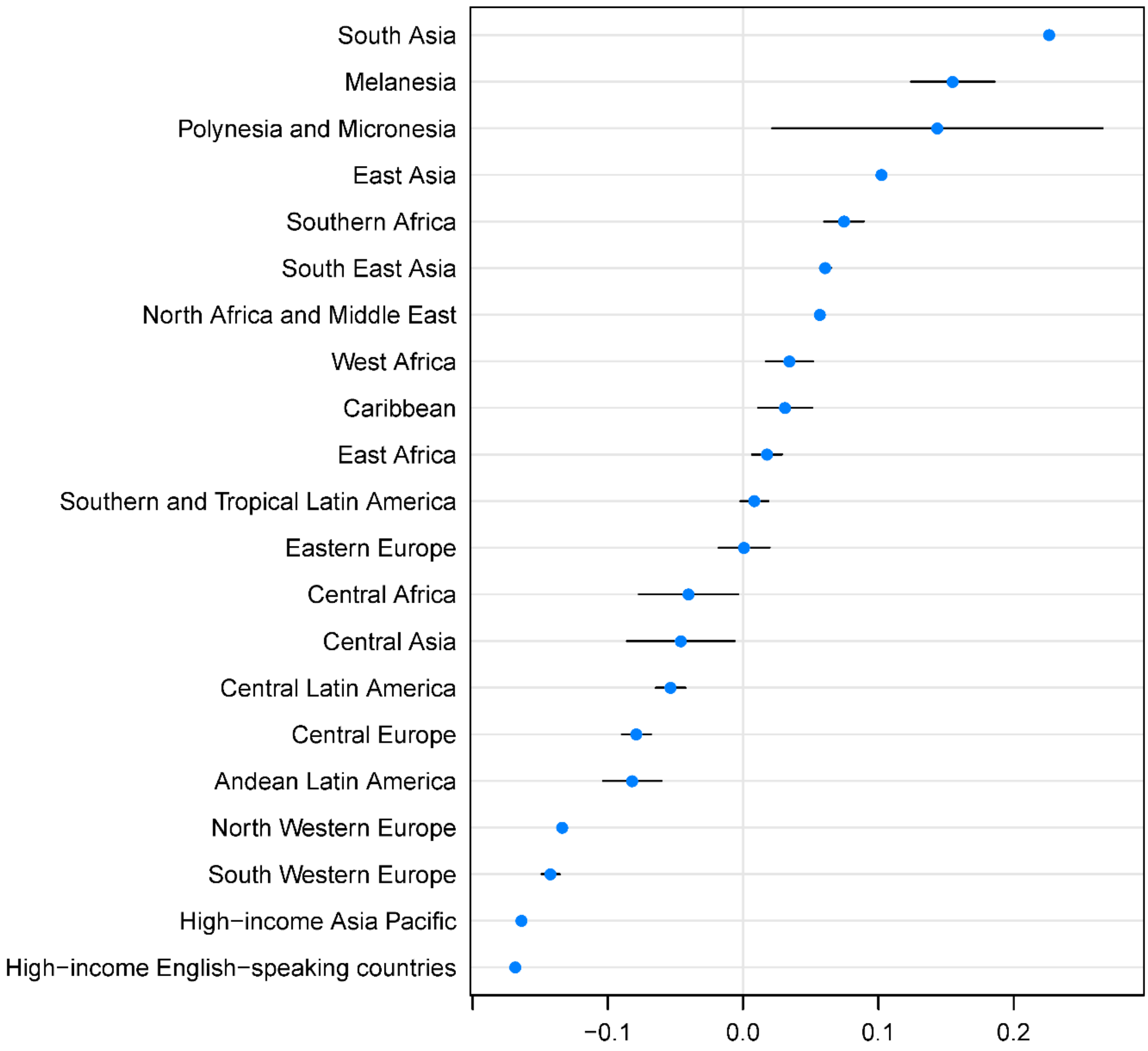
South Asia
Melanesia
Polynesia and Micronesia
East Asia
Southern Africa
South East Asia
North Africa and Middle East
West Africa
Caribbean
East Africa
Southern and Tropical Latin America
Eastern Europe
Central Africa
Central Asia
Central Latin America
Central Europe
Andean Latin America
North Western Europe
South Western Europe
High−income Asia Pacific
High−income English−speaking countries
−0.1
0.0
0.1
0.2

**Dependent variable: Prevalence (BMI < -2SD)**	
**Independent variable: Inverse mean BMI, prevalence (BMI > +1SD), prevalence (BMI > +2SD) and prevalence (BMI < -1SD)**	
**Variables**	**Coefficients (95% CI)**
Intercept	0.20 (0.090, 0.31)
Inverse mean BMI	-18.50 (-19.80, -17.20)
Probit-transformed prevalence (BMI > +1SD)	0.49 (0.45, 0.53)
Probit-transformed prevalence (BMI > +2SD)	-0.33 (-0.36, -0.29)
Probit-transformed prevalence (BMI < -1SD)	1.16 (1.15, 1.18)
Mean age of age group	-0.078 (-0.083, -0.074)
Male sex	0.067 (0.037, 0.097)
Study mid-year (per one more recent year since 1975)	0.0013 (0.0011, 0.0015)
Natural logarithm of per-capita gross domestic product	0.0038 (-0.00040, 0.0079)
Inverse mean BMI * mean age of age group	1.42 (1.35, 1.48)
Probit-transformed prevalence (BMI > +1SD) * mean age of age group	-0.018 (-0.021, -0.015)
Probit-transformed prevalence (BMI > +2SD) * mean age of age group	0.018 (0.016, 0.021)
Probit-transformed prevalence (BMI < -1SD) * mean age of age group	-0.011 (-0.012, -0.010)
Inverse mean BMI * male sex	0.26 (-0.14, 0.67)
Probit-transformed prevalence (BMI > +1SD) * male sex	-0.078 (-0.10, -0.055)
Probit-transformed prevalence (BMI > +2SD) * male sex	0.066 (0.047, 0.086)
Probit-transformed prevalence (BMI < -1SD) * male sex	-0.019 (-0.027, -0.012)
Random effects for regions	Yes
**Number of data points used to fit the model = 9,093**	

Traditional R^2 is not clearly defined for mixed-effect models. The pseudo-R^2 for the model, which describes the proportion of variance explained by both fixed and random factors,[12] was 0.955.

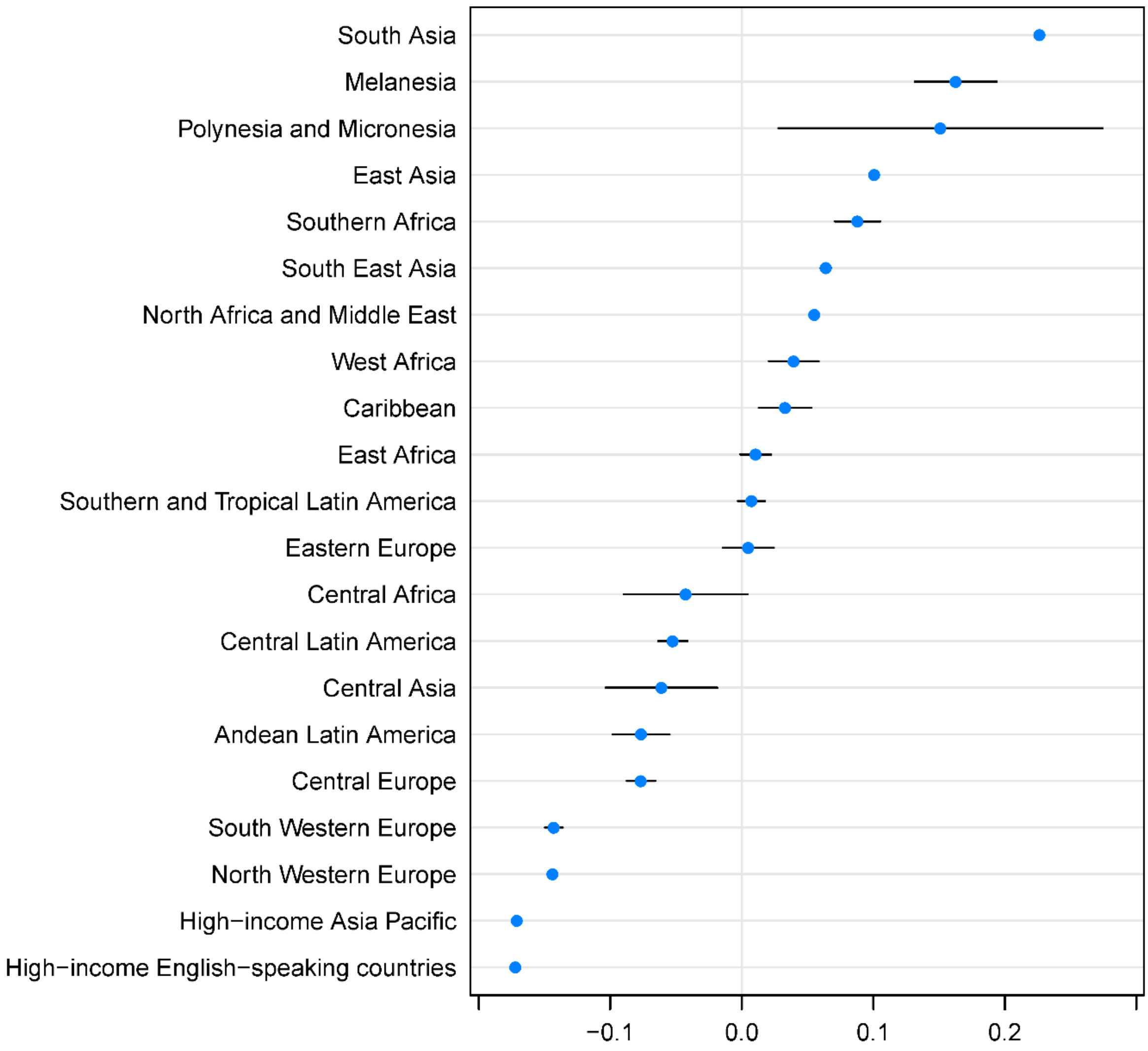
South Asia
Melanesia
Polynesia and Micronesia
East Asia
Southern Africa
South East Asia
North Africa and Middle East
West Africa
Caribbean
East Africa
Southern and Tropical Latin America
Eastern Europe
Central Africa
Central Latin America
Central Asia
Andean Latin America
Central Europe
South Western Europe
North Western Europe
High−income Asia Pacific
High−income English−speaking countries
−0.1
0.0
0.1
0.2

**Dependent variable: Prevalence (BMI < -2SD)**	
**Independent variable: Inverse mean BMI, prevalence (17 kg/m^2 ≤ BMI < 18.5 kg/m^2) and prevalence (18.5 kg/m^2 ≤ BMI < 25 kg/m^2)**	
**Variables**	**Coefficients (95% CI)**
Intercept	-9.52 (-9.71, -9.33)
Inverse mean BMI	109.00 (106.00, 112.00)
Probit-transformed prevalence (17 kg/m^2 ≤ BMI < 18.5 kg/m^2)	-0.68 (-0.70, -0.65)
Probit-transformed prevalence (18.5 kg/m^2 ≤ BMI < 25 kg/m^2)	1.12 (1.09, 1.14)
Mean age of age group	0.35 (0.34, 0.36)
Male sex	-1.03 (-1.10, -0.96)
Study mid-year (per one more recent year since 1975)	-0.0012 (-0.0015, -0.00094)
Natural logarithm of per-capita gross domestic product	0.052 (0.047, 0.057)
Inverse mean BMI * mean age of age group	-4.13 (-4.34, -3.92)
Probit-transformed prevalence (17 kg/m^2 ≤ BMI < 18.5 kg/m^2) * mean age of age group	0.053 (0.052, 0.055)
Probit-transformed prevalence (18.5 kg/m^2 ≤ BMI < 25 kg/m^2) * mean age of age group	-0.10 (-0.10, -0.10)
Inverse mean BMI * male sex	17.30 (15.90, 18.80)
Probit-transformed prevalence (17 kg/m^2 ≤ BMI < 18.5 kg/m^2) * male sex	-0.30 (-0.31, -0.28)
Probit-transformed prevalence (18.5 kg/m^2 ≤ BMI < 25 kg/m^2) * male sex	0.27 (0.25, 0.28)
Random effects for regions	Yes
**Number of data points used to fit the model = 7,039**	

Traditional R^2 is not clearly defined for mixed-effect models. The pseudo-R^2 for the model, which describes the proportion of variance explained by both fixed and random factors,[12] was 0.944.

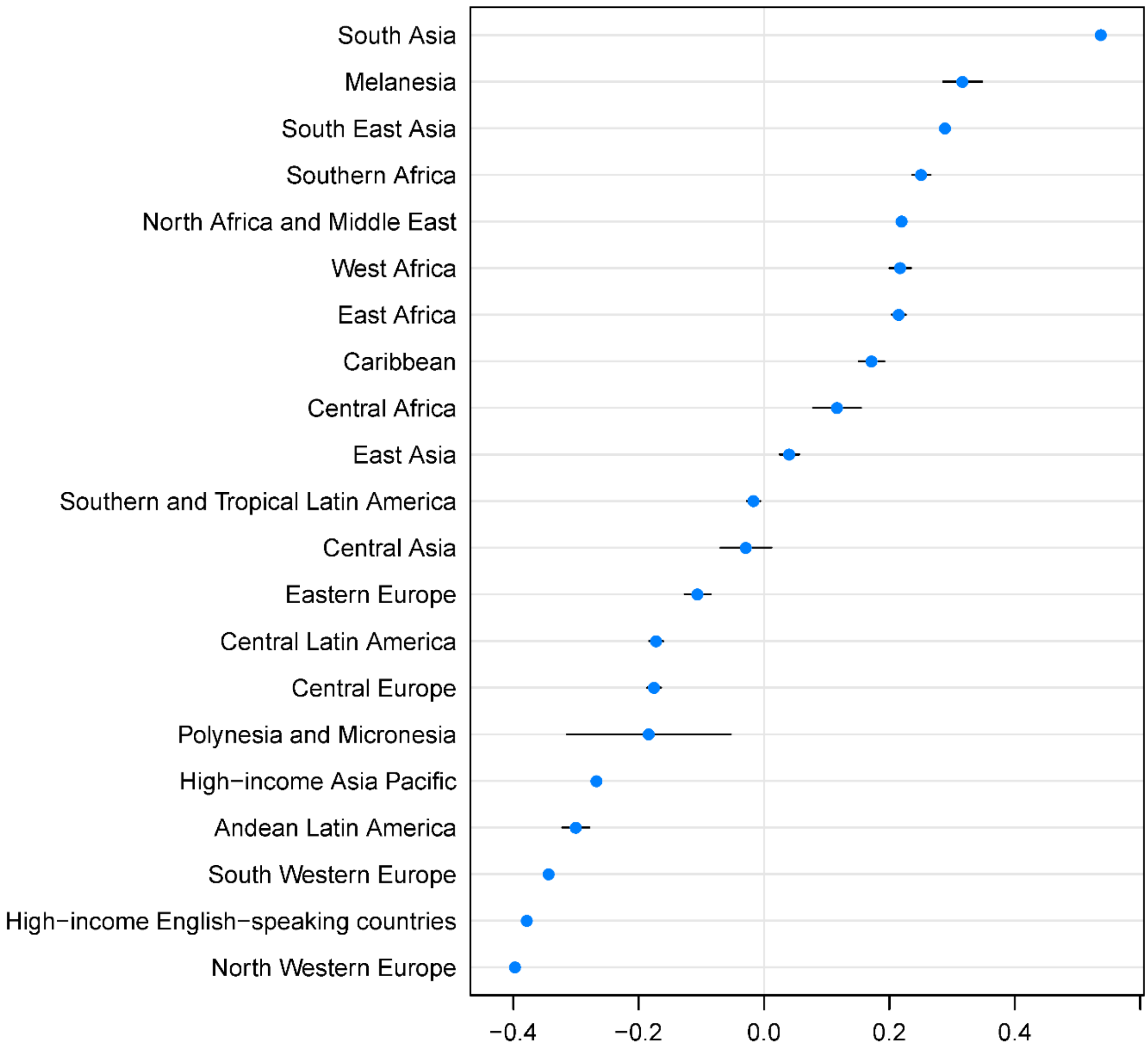

South Asia
Melanesia
South East Asia
Southern Africa
North Africa and Middle East
West Africa
East Africa
Caribbean
Central Africa
East Asia
Southern and Tropical Latin America
Central Asia
Eastern Europe
Central Latin America
Central Europe
Polynesia and Micronesia
High−income Asia Pacific
Andean Latin America
South Western Europe
High−income English−speaking countries
North Western Europe
−0.4
−0.2
0.0
0.2
0.4

Dependent variable: Prevalence (BMI < -2SD)	
**Independent variable: Inverse mean BMI and prevalence (BMI <18.5 kg/m^2)**	
**Variables**	**Coefficients (95% CI)**
Intercept	-12.20 (-12.40, -12.10)
Inverse mean BMI	165.00 (162.00, 167.00)
Probit-transformed prevalence (BMI <18.5 kg/m^2)	-1.05 (-1.06, -1.03)
Mean age of age group	0.66 (0.65, 0.67)
Male sex	-2.43 (-2.50, -2.36)
Study mid-year (per one more recent year since 1975)	-0.0020 (-0.0023, -0.0018)
Natural logarithm of per-capita gross domestic product	0.054 (0.050, 0.058)
Inverse mean BMI * mean age of age group	-10.30 (-10.50, -10.20)
Probit-transformed prevalence (BMI <18.5 kg/m^2) * mean age of age group	0.11 (0.11, 0.11)
Inverse mean BMI * male sex	48.20 (46.90, 49.50)
Probit-transformed prevalence (BMI <18.5 kg/m^2) * male sex	-0.42 (-0.43, -0.41)
Random effects for regions	Yes
**Number of data points used to fit the model = 9,210**	

Traditional R^2 is not clearly defined for mixed-effect models. The pseudo-R^2 for the model, which describes the proportion of variance explained by both fixed and random factors,[12] was 0.933.

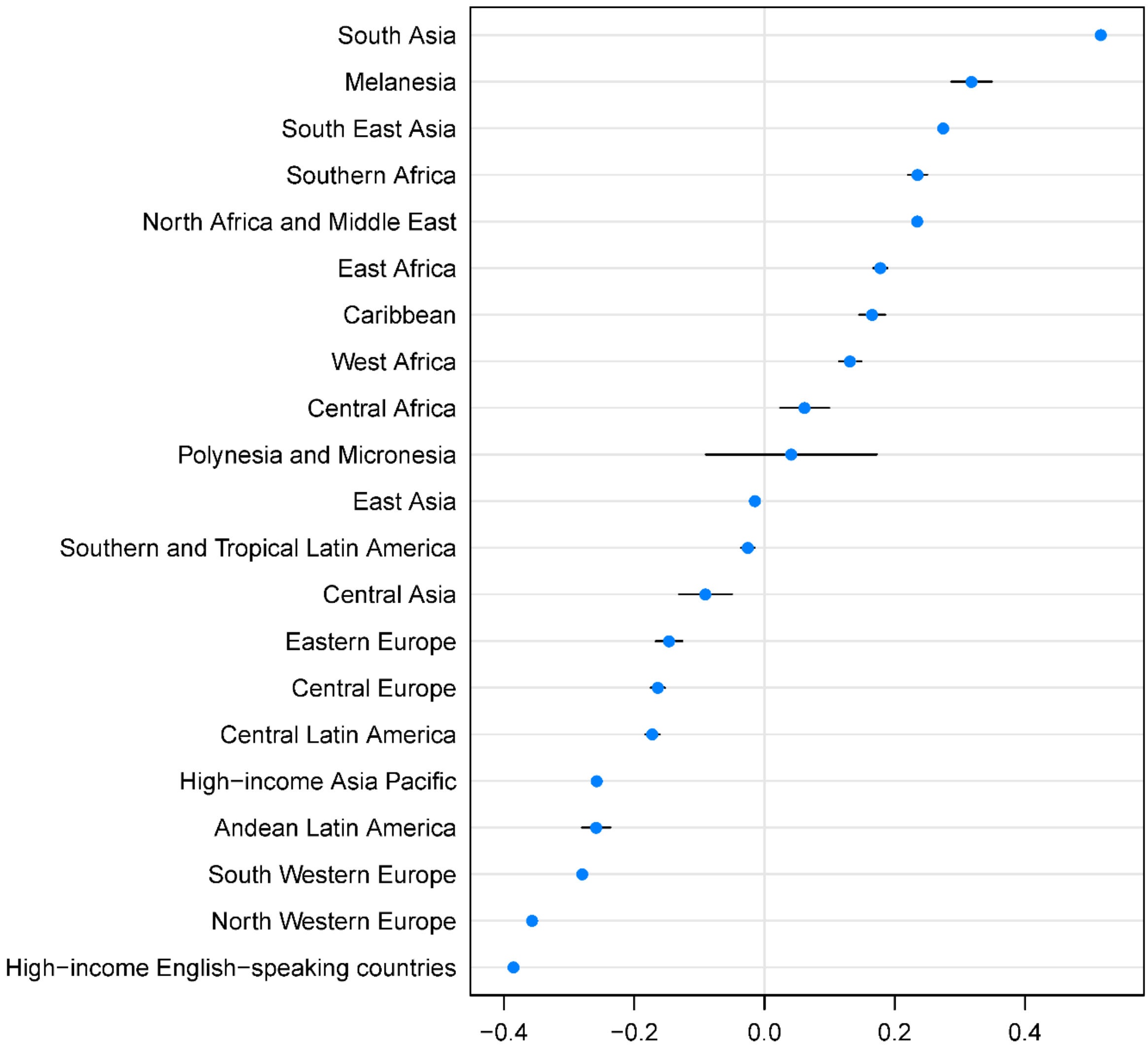
South Asia
Melanesia
South East Asia
Southern Africa
North Africa and Middle East
East Africa
Caribbean
West Africa
Central Africa
Polynesia and Micronesia
East Asia
Southern and Tropical Latin America
Central Asia
Eastern Europe
Central Europe
Central Latin America
High−income Asia Pacific
Andean Latin America
South Western Europe
North Western Europe
High−income English−speaking countries
−0.4
−0.2
0.0
0.2
0.4

**Dependent variable: Prevalence (BMI < -2SD)**	
**Independent variable: Inverse mean BMI, prevalence (15 kg/m^2 ≤ BMI < 18.5 kg/m^2) and prevalence (BMI <18.5 kg/m^2)**	
**Variables**	**Coefficients (95% CI)**
Intercept	-10.80 (-11.00, -10.60)
Inverse mean BMI	140.00 (137.00, 143.00)
Probit-transformed prevalence (15 kg/m^2 ≤ BMI < 18.5 kg/m^2)	-1.01 (-1.04, -0.99)
Probit-transformed prevalence (BMI <18.5 kg/m^2)	-0.15 (-0.16, -0.13)
Mean age of age group	0.51 (0.50, 0.52)
Male sex	-0.78 (-0.86, -0.69)
Study mid-year (per one more recent year since 1975)	-0.00096 (-0.0012, -0.00074)
Natural logarithm of per-capita gross domestic product	0.051 (0.047, 0.055)
Inverse mean BMI * mean age of age group	-7.69 (-7.96, -7.43)
Probit-transformed prevalence (15 kg/m^2 ≤ BMI < 18.5 kg/m^2) * mean age of age group	0.10 (0.10, 0.10)
Probit-transformed prevalence (BMI <18.5 kg/m^2) * mean age of age group	0.0059 (0.0046, 0.0072)
Inverse mean BMI * male sex	15.90 (14.30, 17.60)
Probit-transformed prevalence (15 kg/m^2 ≤ BMI < 18.5 kg/m^2) * male sex	-0.13 (-0.14, -0.12)
Probit-transformed prevalence (BMI <18.5 kg/m^2) * male sex	-0.21 (-0.21, -0.20)
Random effects for regions	Yes
**Number of data points used to fit the model = 7,392**	

Traditional R^2 is not clearly defined for mixed-effect models. The pseudo-R^2 for the model, which describes the proportion of variance explained by both fixed and random factors,[12] was 0.944.

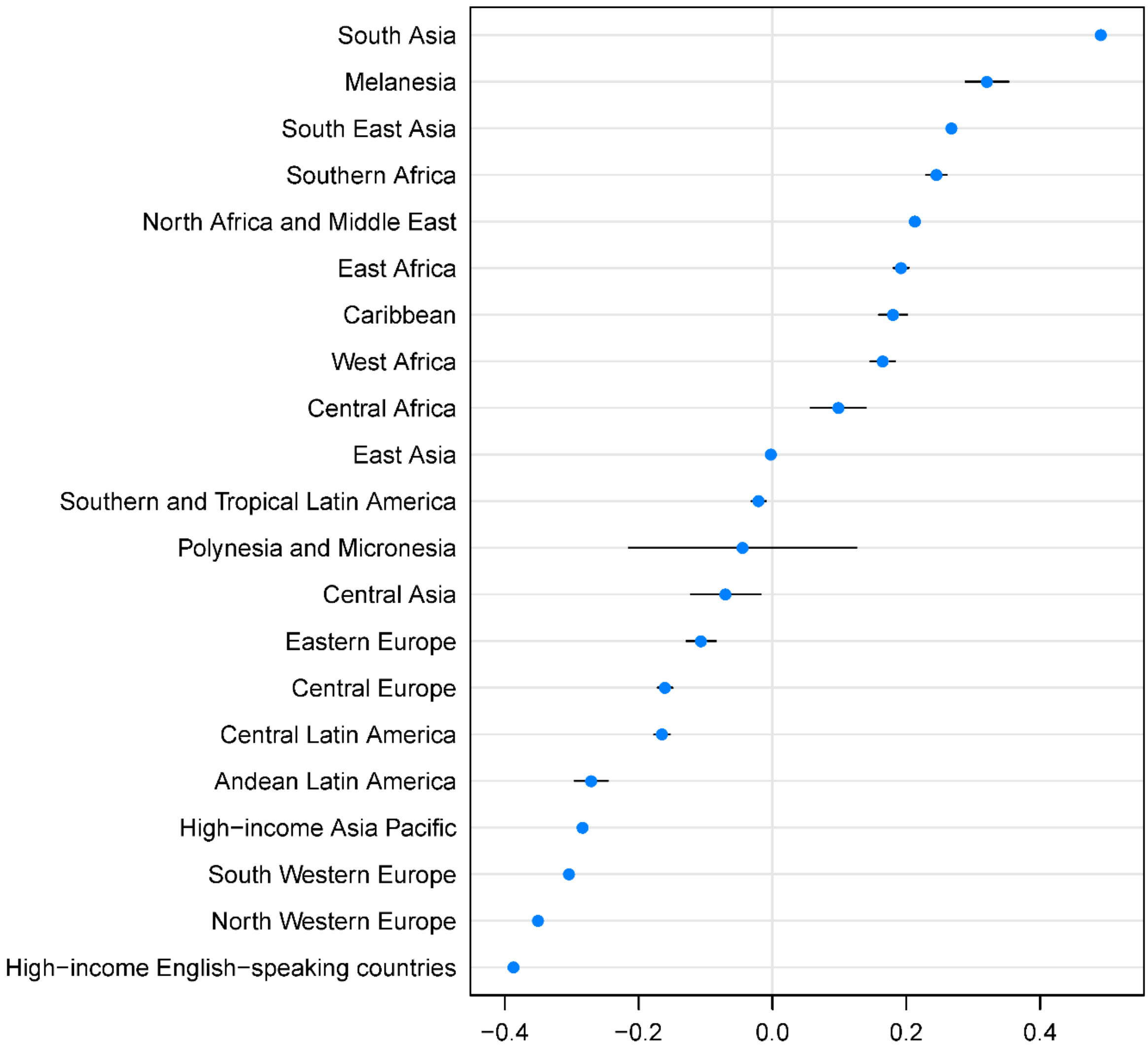
South Asia
Melanesia
South East Asia
Southern Africa
North Africa and Middle East
East Africa
Caribbean
West Africa
Central Africa
East Asia
Southern and Tropical Latin America
Polynesia and Micronesia
Central Asia
Eastern Europe
Central Europe
Central Latin America
Andean Latin America
High−income Asia Pacific
South Western Europe
North Western Europe
High−income English−speaking countries
−0.4
−0.2
0.0
0.2
0.4

Appendix Table 5: Model specifications and regression coefficients to estimate the primary outcomes (mean BMI or prevalence of various BMI categories) from other metrics for adults (aged 20 years and above).

The dependent variable in all regressions was the inverse of the mean or a prevalence, fitted using a linear (mixed) model for mean and a generalised linear (mixed) model fitted with a probit link function for prevalence. Random effects for regions in regression are presented after the table of coefficients.

* denotes statistical interaction. CI: confidence interval.

**Dependent variable: Inverse mean BMI**	
**Age range: 18 years and older**	
**Independent variable: Prevalence (BMI ≥22 kg/m^2)**	
**Variables**	**Coefficients (95% CI)**
Intercept	0.048 (0.047 – 0.048)
Probit-transformed prevalence	-0.0062 (-0.0062 – -0.0061)
Mean age of age group	-2.08 x 10^{-6} (-3.64 x 10^{-6} – -5.21 x 10^{-7})
Male sex	0.00051 (0.00045 – 0.00057)
Study mid-year (per one more recent year since 1975)	-2.14 x 10^{-5} (-2.40 x 10^{-5} – -1.87 x 10^{-5})
Natural logarithm of per-capita gross domestic product	-0.00036 (-0.00040 – -0.00032)
Probit-transformed prevalence * mean age of age group	2.31 x 10^{-6} (7.16 x 10^{-7} – 3.90 x 10^{-6})
Probit-transformed prevalence * male sex	0.00098 (0.00093 – 0.0010)
**Number of data points used to fit the model = 9,752**	

Traditional R^2 is not clearly defined for mixed-effect models. The conditional R^2 for the model, which describes the proportion of variance explained by both fixed and random factors,[11] was 0.9476.

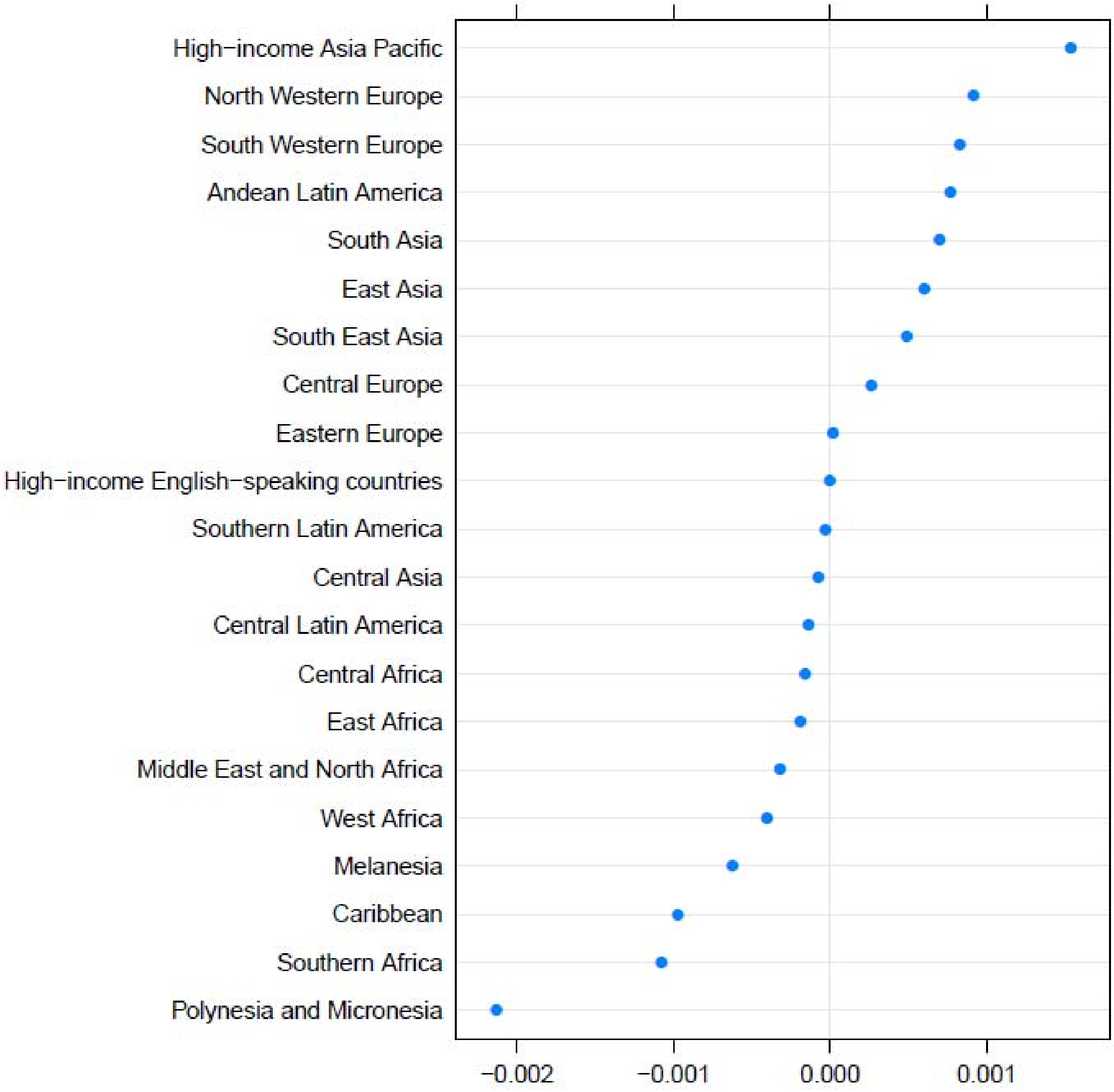
High−income Asia Pacific
North Western Europe
South Western Europe
Andean Latin America
South Asia
East Asia
South East Asia
Central Europe
Eastern Europe
High−income English−speaking countries
Southern Latin America
Central Asia
Central Latin America
Central Africa
East Africa
Middle East and North Africa
West Africa
Melanesia
Caribbean
Southern Africa
Polynesia and Micronesia
−0.002
−0.001
0.000
0.001

Dependent variable: Inverse mean BMI	
**Age range: 18 years and older**	
**Independent variable: Prevalence (BMI ≥23 kg/m^2)**	
**Variables**	**Coefficients (95% CI)**
Intercept	0.044 (0.044 – 0.045)
Probit-transformed prevalence	-0.0061 (-0.0061 – -0.0060)
Mean age of age group	6.28×10^{-6} (5.04×10^{-6} – 7.53×10^{-6})
Male sex	0.00064 (0.00059 – 0.00068)
Study mid-year (per one more recent year since 1975)	-1.66×10^{-5} (-1.89×10^{-5} – -1.42×10^{-5})
Natural logarithm of per-capita gross domestic product	-0.00024 (-0.00028 – -0.00020)
Probit-transformed prevalence * mean age of age group	-1.02×10^{-6} (-2.43×10^{-6} – 3.84×10^{-7})
Probit-transformed prevalence * male sex	0.00091 (0.00086 – 0.00096)
**Number of data points used to fit the model = 9,785**	

Traditional R^2 is not clearly defined for mixed-effect models. The conditional R^2 for the model, which describes the proportion of variance explained by both fixed and random factors,[11] was 0.9573.

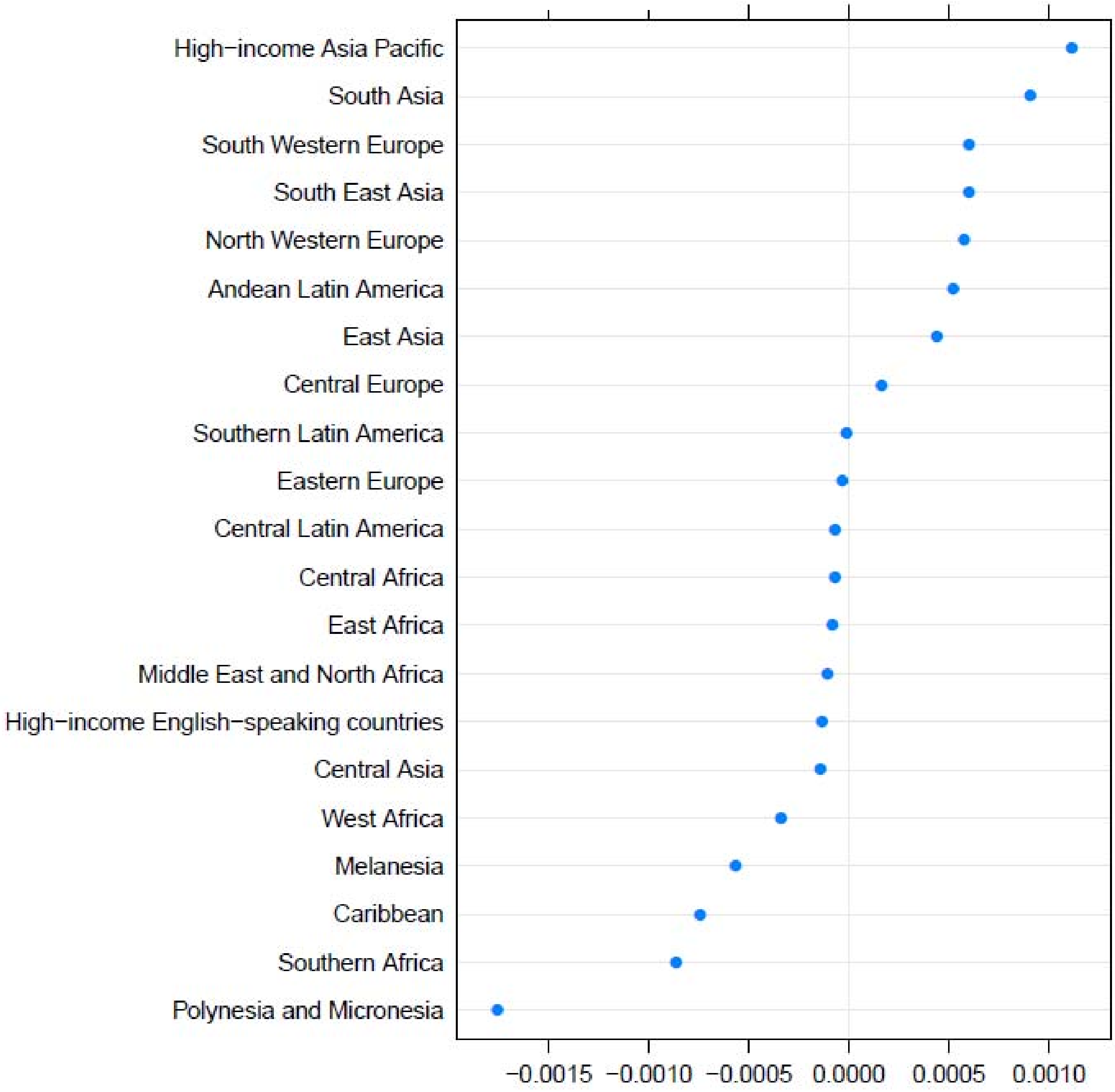
High−income Asia Pacific
South Asia
South Western Europe
South East Asia
North Western Europe
Andean Latin America
East Asia
Central Europe
Southern Latin America
Eastern Europe
Central Latin America
Central Africa
East Africa
Middle East and North Africa
High−income English−speaking countries
Central Asia
West Africa
Melanesia
Caribbean
Southern Africa
Polynesia and Micronesia
−0.0015
−0.0010
−0.0005
0.0000
0.0005
0.0010

**Dependent variable: Inverse mean BMI**	
**Age range: 18 years and older**	
**Independent variable: Prevalence (BMI ≥24 kg/m^2)**	
**Variables**	**Coefficients (95% CI)**
Intercept	0.042 (0.042 – 0.042)
Probit-transformed prevalence	-0.0058 (-0.0059 – -0.0058)
Mean age of age group	1.02 x 10^{-5} (9.08 x 10^{-6} – 1.14 x 10^{-5})
Male sex	0.00068 (0.00064 – 0.00071)
Study mid-year (per one more recent year since 1975)	-1.22 x 10^{-5} (-1.45 x 10^{-5} – -1.00 x 10^{-5})
Natural logarithm of per-capita gross domestic product	-0.00017 (-0.00020 – -0.00013)
Probit-transformed prevalence * mean age of age group	-6.05E x 10^{-6} (-7.37 x 10^{-6} – -4.73 x 10^{-6})
Probit-transformed prevalence * male sex	0.00086 (0.00081 – 0.00091)
**Number of data points used to fit the model = 9,788**	

Traditional R^2 is not clearly defined for mixed-effect models. The conditional R^2 for the model, which describes the proportion of variance explained by both fixed and random factors,[11] was 0.9614.

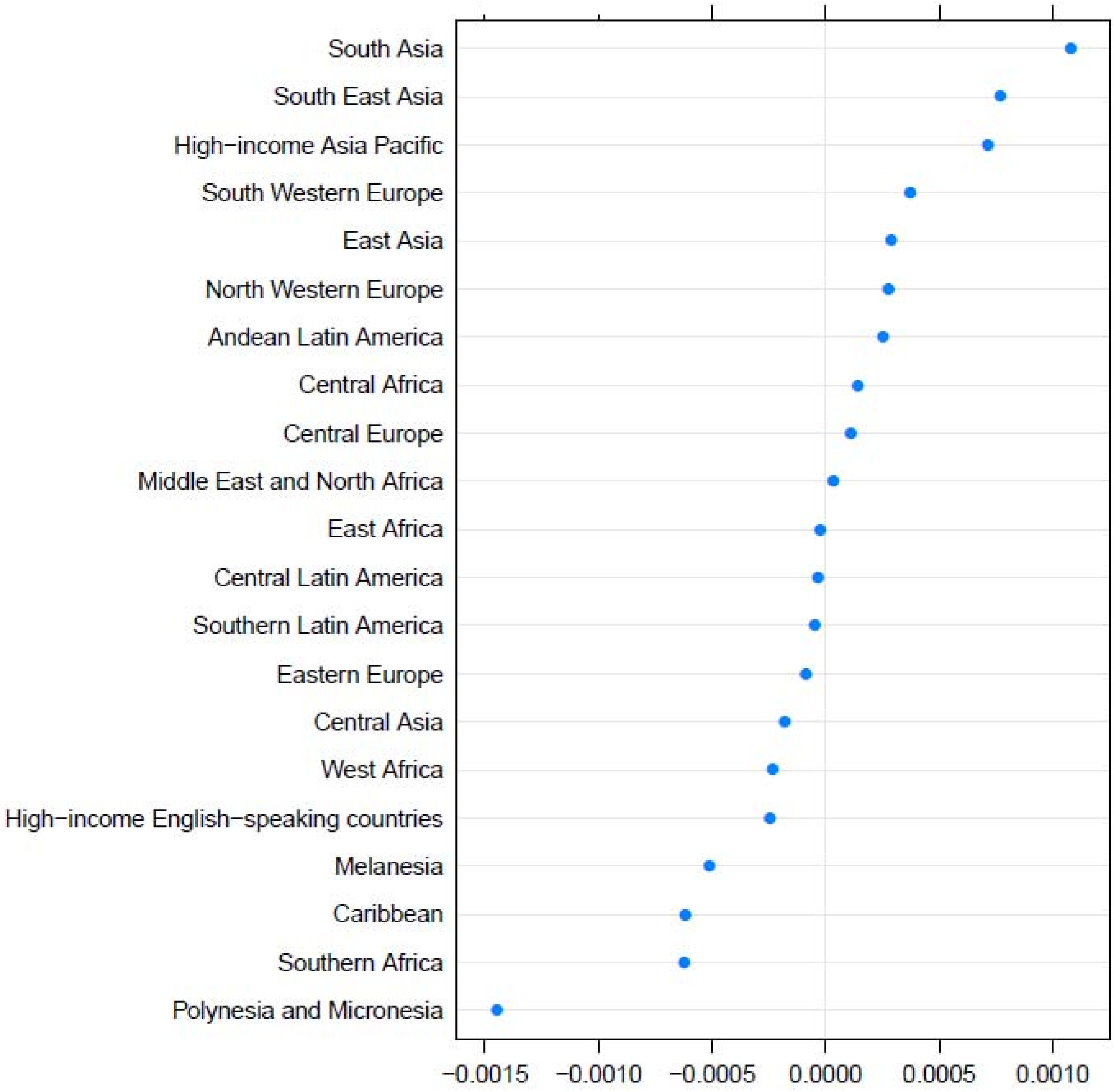

South Asia
South East Asia
High−income Asia Pacific
South Western Europe
East Asia
North Western Europe
Andean Latin America
Central Africa
Central Europe
Middle East and North Africa
East Africa
Central Latin America
Southern Latin America
Eastern Europe
Central Asia
West Africa
High−income English−speaking countries
Melanesia
Caribbean
Southern Africa
Polynesia and Micronesia
−0.0015
−0.0010
−0.0005
0.0000
0.0005
0.0010

Dependent variable: Inverse mean BMI	
**Age range: 18 years and older**	
**Independent variable: Prevalence (BMI ≥25 kg/m^2)**	
**Variables**	**Coefficients (95% CI)**
Intercept	0.040 (0.039 – 0.040)
Probit-transformed prevalence	-0.0057 (-0.0058 – -0.0056)
Mean age of age group	9.76 x 10^{-6} (8.60 x 10^{-6} – 1.09 x 10^{-5})
Male sex	0.00063 (0.00060 – 0.00067)
Study mid-year (per one more recent year since 1975)	-8.34 x 10^{-6} (-1.05 x 10^{-5} – -6.16 x 10^{-6})
Natural logarithm of per-capita gross domestic product	-9.38 x 10^{-5} (-0.000127 – -6.05 x 10^{-5})
Probit-transformed prevalence * mean age of age group	-9.67 x 10^{-6} (-1.10 x 10^{-5} – -8.37 x 10^{-6})
Probit-transformed prevalence * male sex	0.00081 (0.00076 – 0.00085)
**Number of data points used to fit the model = 9,778**	

Traditional R^2 is not clearly defined for mixed-effect models. The conditional R^2 for the model, which describes the proportion of variance explained by both fixed and random factors,[11] was 0.9627.

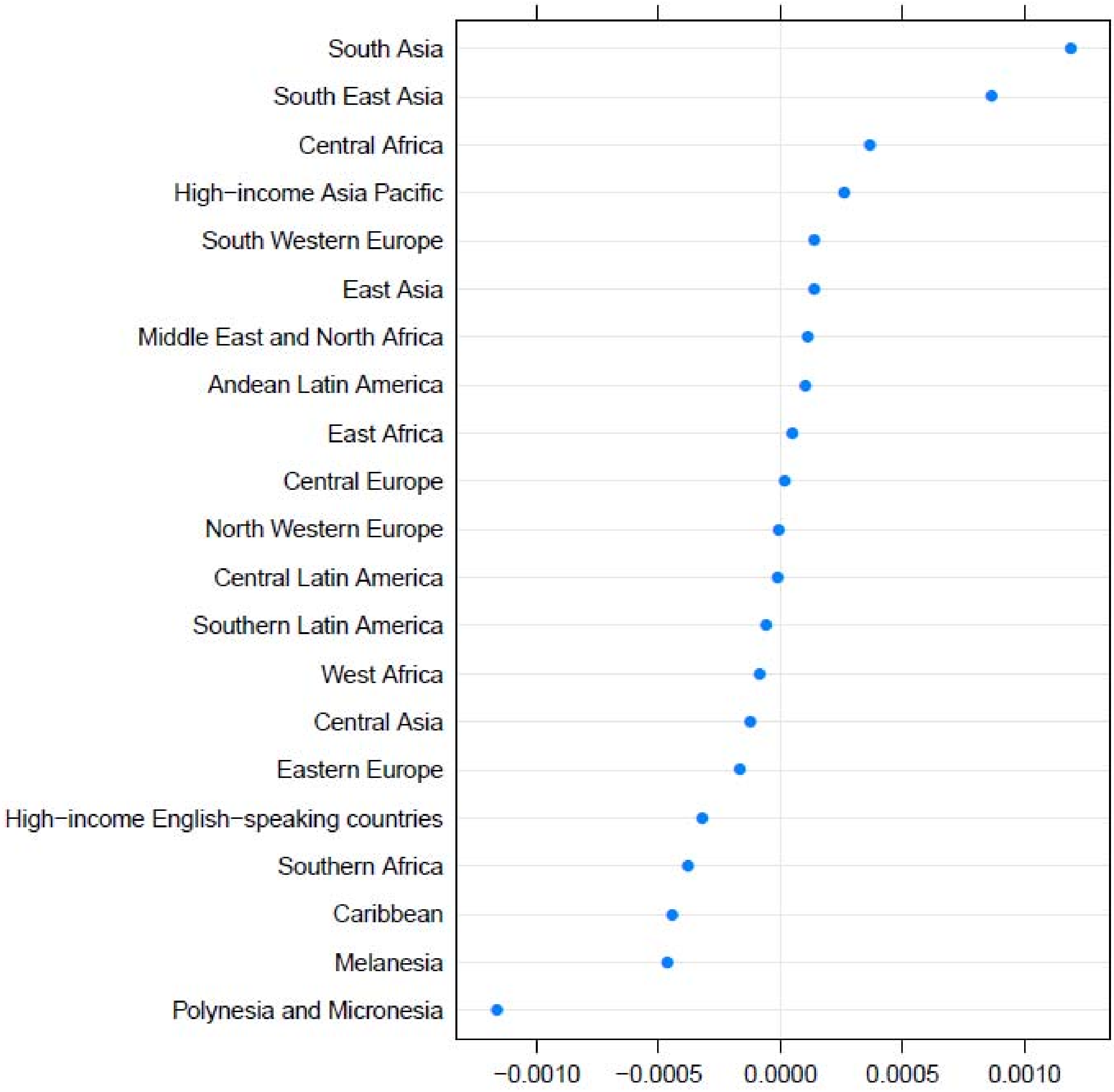
South Asia
South East Asia
Central Africa
High−income Asia Pacific
South Western Europe
East Asia
Middle East and North Africa
Andean Latin America
East Africa
Central Europe
North Western Europe
Central Latin America
Southern Latin America
West Africa
Central Asia
Eastern Europe
High−income English−speaking countries
Southern Africa
Caribbean
Melanesia
Polynesia and Micronesia
−0.0010
−0.0005
0.0000
0.0005
0.0010

**Dependent variable: Inverse mean BMI**	
**Age range: 18 years and older**	
**Independent variable: Prevalence (BMI ≥27 kg/m^2)**	
**Variables**	**Coefficients (95% CI)**
Intercept	0.037 (0.036 – 0.037)
Probit-transformed prevalence	-0.0055 (-0.0056 – -0.0055)
Mean age of age group	-7.67×10^{-7} (-2.35×10^{-6} – 8.13×10^{-7})
Male sex	0.00033 (0.00028 – 0.00038)
Study mid-year (per one more recent year since 1975)	3.02×10^{-6} (6.27×10^{-7} – 5.41×10^{-6})
Natural logarithm of per-capita gross domestic product	-3.13×10^{-5} (-6.80×10^{-5} – 5.34×10^{-6})
Probit-transformed prevalence * mean age of age group	-1.21×10^{-5} (-1.36×10^{-5} – -1.06×10^{-5})
Probit-transformed prevalence * male sex	0.00057 (0.00052 – 0.00062)
**Number of data points used to fit the model = 9,669**	

Traditional R^2 is not clearly defined for mixed-effect models. The conditional R^2 for the model, which describes the proportion of variance explained by both fixed and random factors,[11] was 0.9544.

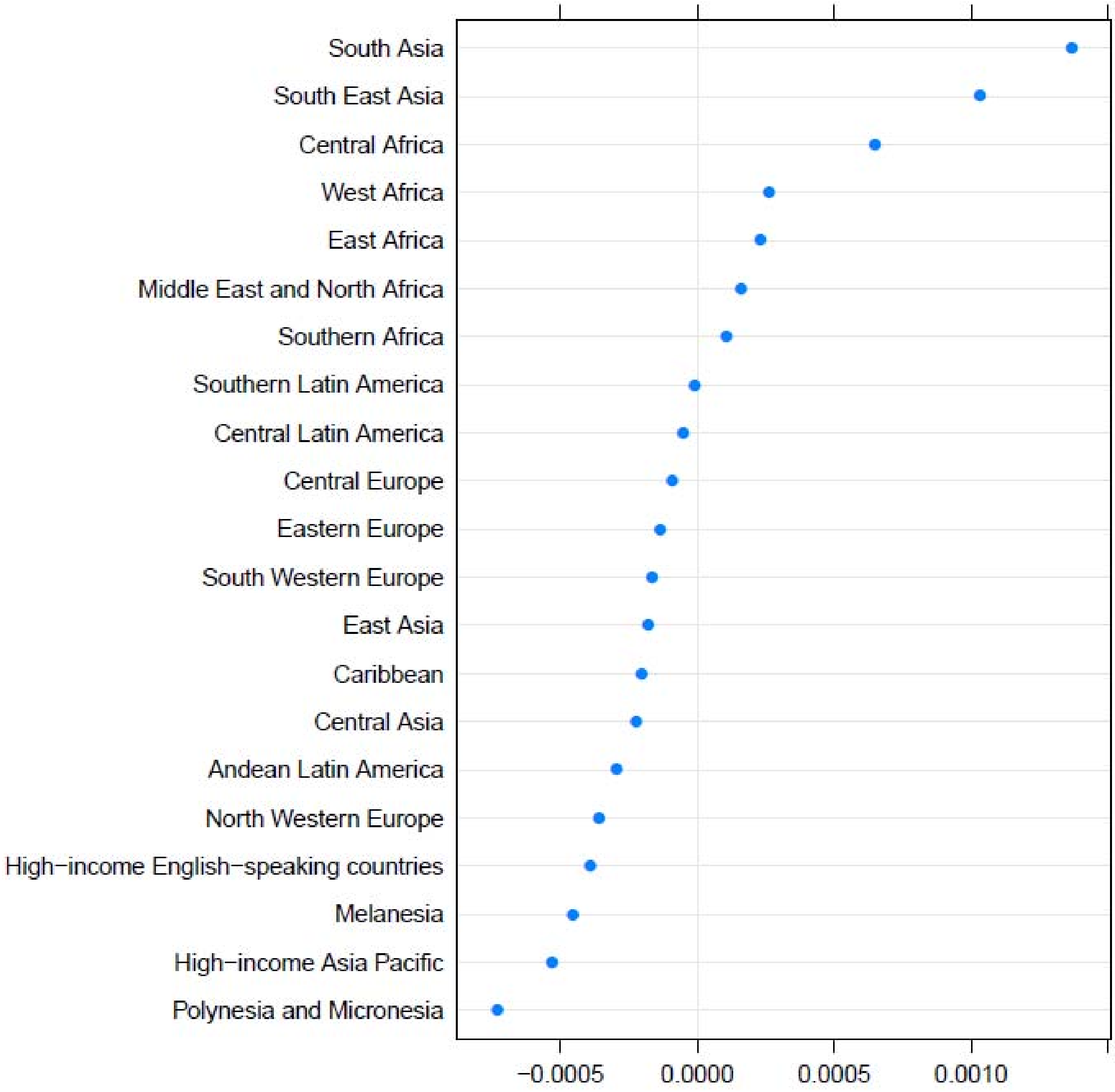
South Asia
South East Asia
Central Africa
West Africa
East Africa
Middle East and North Africa
Southern Africa
Southern Latin America
Central Latin America
Central Europe
Eastern Europe
South Western Europe
East Asia
Caribbean
Central Asia
Andean Latin America
North Western Europe
High−income English−speaking countries
Melanesia
High−income Asia Pacific
Polynesia and Micronesia
−0.0005
0.0000
0.0005
0.0010

**Dependent variable: Inverse mean BMI**	
**Age range: 18 years and older**	
**Independent variable: Prevalence (BMI ≥27.5 kg/m^2)**	
**Variables**	**Coefficients (95% CI)**
Intercept	0.036 (0.036 – 0.036)
Probit-transformed prevalence	-0.0055 (-0.0056 – -0.0054)
Mean age of age group	-4.20 x 10^{-6} (-5.95 x 10^{-6} – -2.45 x 10^{-6})
Male sex	0.00020 (0.00015 – 0.00026)
Study mid-year (per one more recent year since 1975)	5.26 x 10^{-6} (2.78 x 10^{-6} – 7.74 x 10^{-6})
Natural logarithm of per-capita gross domestic product	-9.65 x 10^{-6} (-4.77 x 10^{-5} – 2.84 x 10^{-5})
Probit-transformed prevalence * mean age of age group	-1.27 x 10^{-5} (-1.43 x 10^{-5} – -1.11 x 10^{-5})
Probit-transformed prevalence * male sex	0.00050 (0.00045 – 0.00056)
**Number of data points used to fit the model = 9,627**	

Traditional R^2 is not clearly defined for mixed-effect models. The conditional R^2 for the model, which describes the proportion of variance explained by both fixed and random factors,[11] was 0.9509.

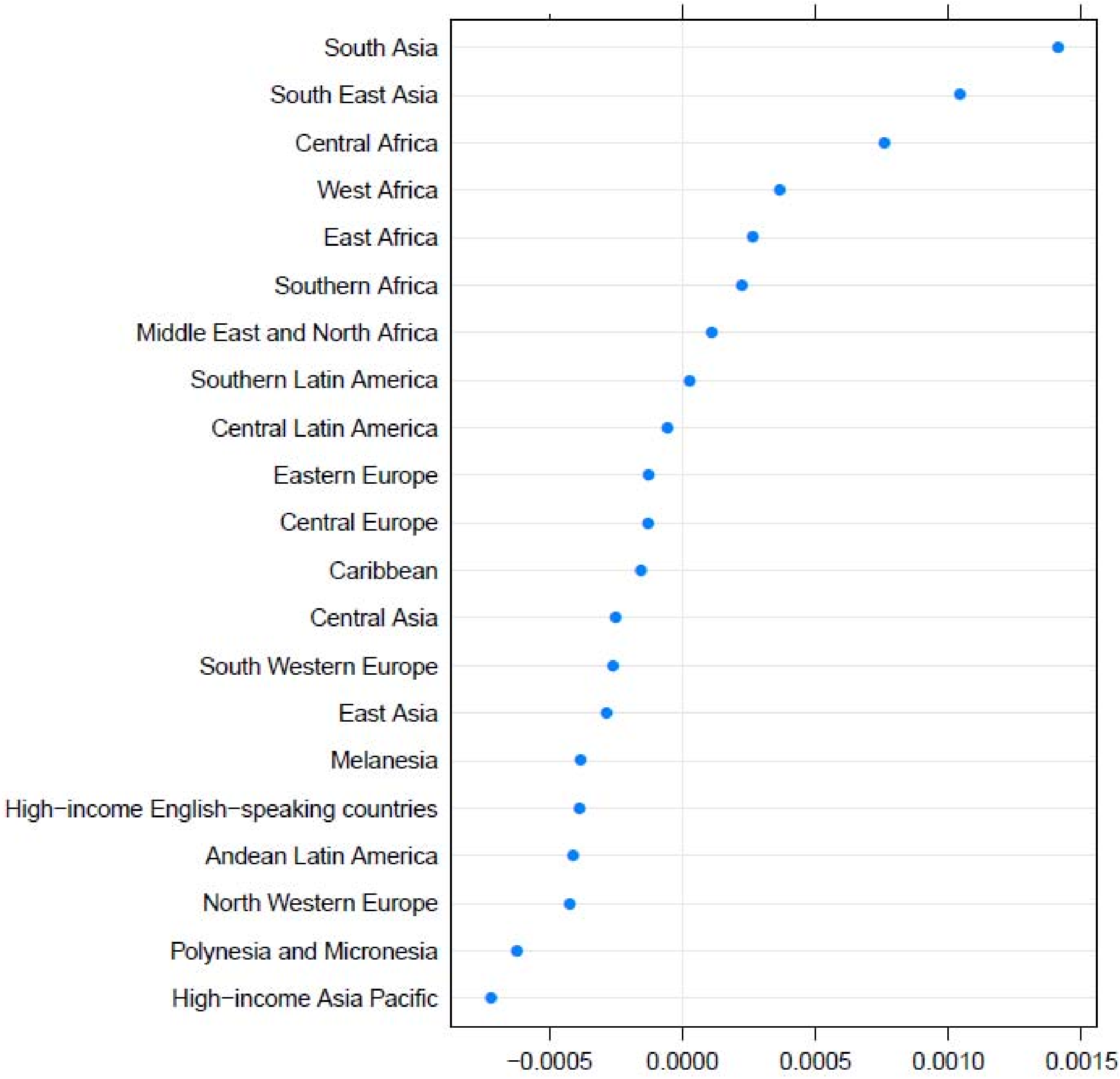
South Asia
South East Asia
Central Africa
West Africa
East Africa
Southern Africa
Middle East and North Africa
Southern Latin America
Central Latin America
Eastern Europe
Central Europe
Caribbean
Central Asia
South Western Europe
East Asia
Melanesia
High−income English−speaking countries
Andean Latin America
North Western Europe
Polynesia and Micronesia
High−income Asia Pacific
−0.0005
0.0000
0.0005
0.0010
0.0015

**Dependent variable: Inverse mean BMI**	
**Age range: 18 years and older**	
**Independent variable: Prevalence (BMI ≥28 kg/m^2)**	
**Variables**	**Coefficients (95% CI)**
Intercept	0.036 (0.035 – 0.036)
Probit-transformed prevalence	-0.0055 (-0.0056 – -0.0054)
Mean age of age group	-7.58 x 10^{-6} (-9.52 x 10^{-6} – -5.64 x 10^{-6})
Male sex	8.69 x 10^{-5} (2.43 x 10^{-5} – 0.00015)
Study mid-year (per one more recent year since 1975)	6.48 x 10^{-6} (3.90 x 10^{-6} – 9.05 x 10^{-6})
Natural logarithm of per-capita gross domestic product	-2.05 x 10^{-6} (-4.16 x 10^{-5} – 3.75 x 10^{-5})
Probit-transformed prevalence * mean age of age group	-1.27 x 10^{-5} (-1.43 x 10^{-5} – -1.10 x 10^{-5})
Probit-transformed prevalence * male sex	0.00045 (0.00039 – 0.00051)
**Number of data points used to fit the model = 9,578**	

Traditional R^2 is not clearly defined for mixed-effect models. The conditional R^2 for the model, which describes the proportion of variance explained by both fixed and random factors,[11] was 0.9473.

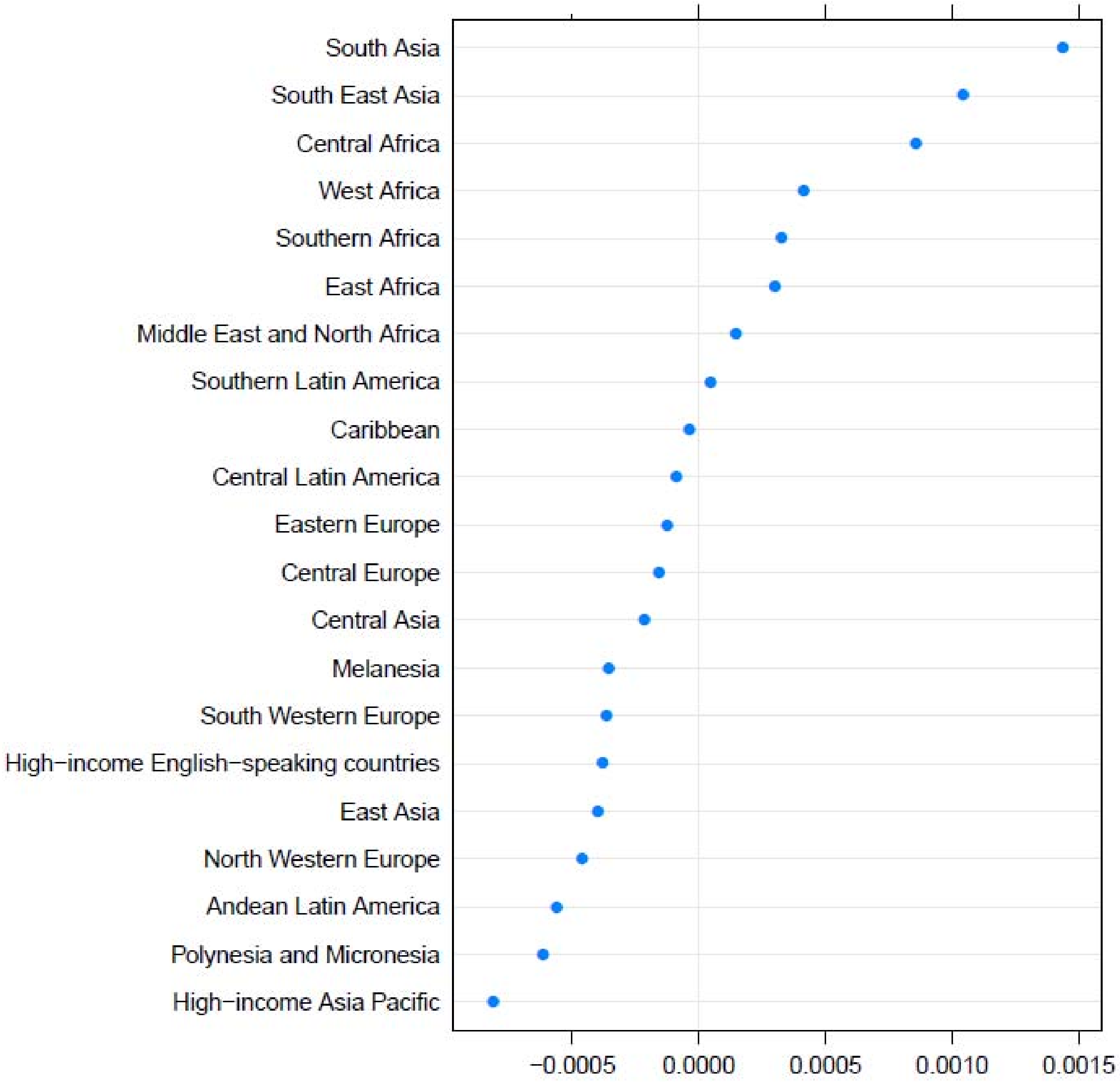
South Asia
South East Asia
Central Africa
West Africa
Southern Africa
East Africa
Middle East and North Africa
Southern Latin America
Caribbean
Central Latin America
Eastern Europe
Central Europe
Central Asia
Melanesia
South Western Europe
High−income English−speaking countries
East Asia
North Western Europe
Andean Latin America
Polynesia and Micronesia
High−income Asia Pacific
−0.0005
0.0000
0.0005
0.0010
0.0015

**Dependent variable: Inverse mean BMI**	
**Age range: 18 years and older**	
**Independent variable: Prevalence (BMI ≥30 kg/m^2)**	
**Variables**	**Coefficients (95% CI)**
Intercept	0.034 (0.034 – 0.035)
Probit-transformed prevalence	-0.0056 (-0.0057 – -0.0055)
Mean age of age group	-2.28 x 10^{-5} (-2.58 x 10^{-5} – -1.99 x 10^{-5})
Male sex	-0.00031 (-0.00041 – -0.00021)
Study mid-year (per one more recent year since 1975)	1.09 x 10^{-5} (7.84 x 10^{-6} – 1.40 x 10^{-5})
Natural logarithm of per-capita gross domestic product	-8.55 x 10^{-6} (-5.56 x 10^{-5} – 3.84 x 10^{-5})
Probit-transformed prevalence * mean age of age group	-1.07 x 10^{-5} (-1.28 x 10^{-5} – -8.64 x 10^{-6})
Probit-transformed prevalence * male sex	0.00037 (0.00029 – 0.00044)
**Number of data points used to fit the model = 9,318**	

Traditional R^2 is not clearly defined for mixed-effect models. The conditional R^2 for the model, which describes the proportion of variance explained by both fixed and random factors,[11] was 0.9260.

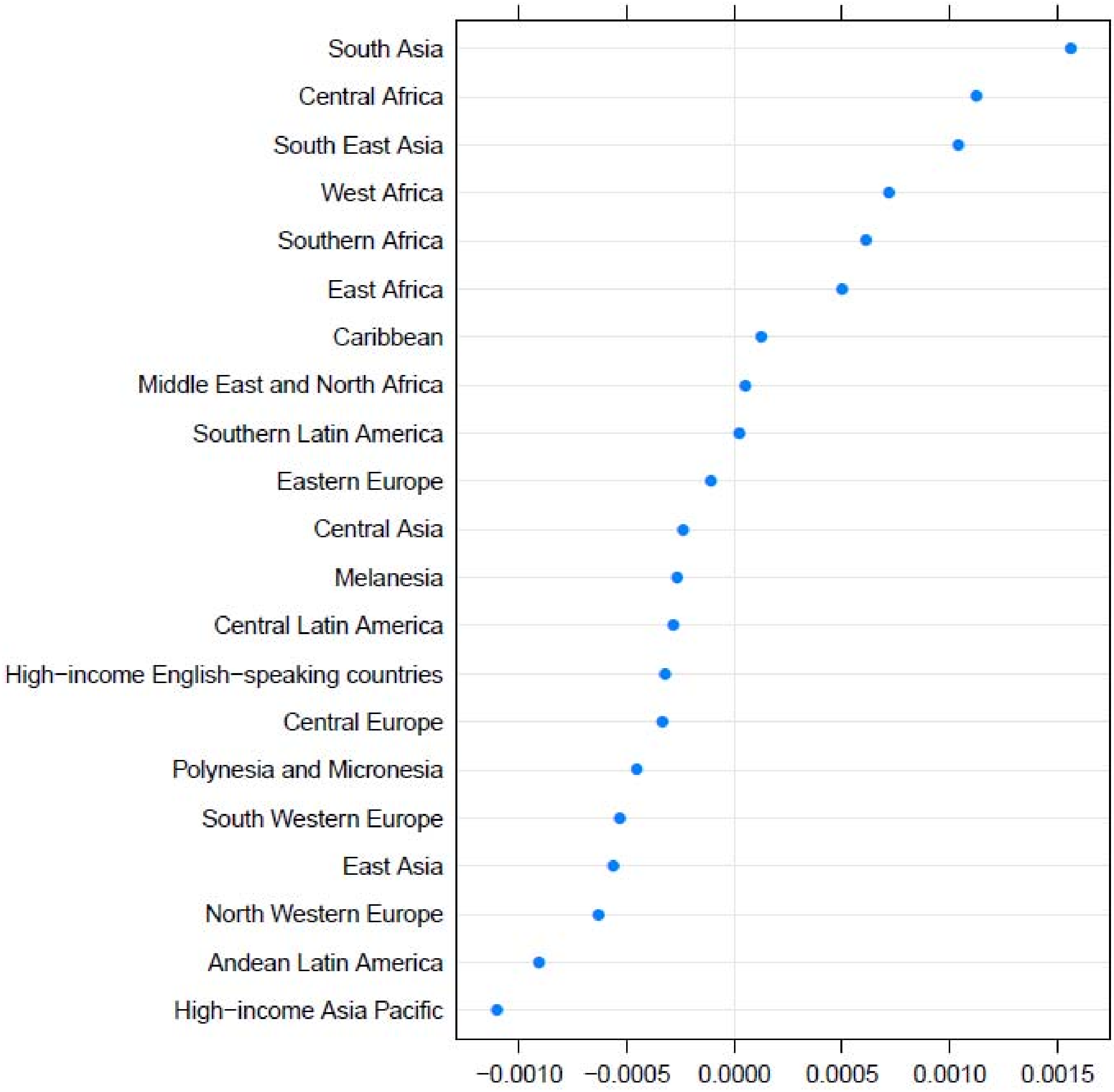
South Asia
Central Africa
South East Asia
West Africa
Southern Africa
East Africa
Caribbean
Middle East and North Africa
Southern Latin America
Eastern Europe
Central Asia
Melanesia
Central Latin America
High−income English−speaking countries
Central Europe
Polynesia and Micronesia
South Western Europe
East Asia
North Western Europe
Andean Latin America
High−income Asia Pacific
−0.0010
−0.0005
0.0000
0.0005
0.0010
0.0015

**Dependent variable: Inverse mean BMI**	
**Age range: 18 years and older**	
**Independent variable: Prevalence (BMI ≥40 kg/m^2)**	
**Variables**	**Coefficients (95% CI)**
Intercep	0.035 (0.034 – 0.037)
Probit-transformed prevalence	-0.0051 (-0.0054 – -0.0048)
Mean age of age group	-5.15 x 10^{-5} (-6.48 x 10^{-5} – -3.82 x 10^{-5})
Male sex	0.0020 (0.0015 – 0.0024)
Study mid-year (per one more recent year since 1975)	4.46 x 10^{-6} (-2.01 x 10^{-6} – 1.09 x 10^{-5})
Natural logarithm of per-capita gross domestic product	-0.00051 (-0.00061 – -0.00042)
Probit-transformed prevalence * mean age of age group	5.78 x 10^{-6} (7.80 x 10^{-8} – 1.15 x 10^{-5})
Probit-transformed prevalence * male sex	0.00110 (0.00089 – 0.00130)
**Number of data points used to fit the model = 6,157**	

Traditional R^2 is not clearly defined for mixed-effect models. The conditional R^2 for the model, which describes the proportion of variance explained by both fixed and random factors,[11] was 0.7509.

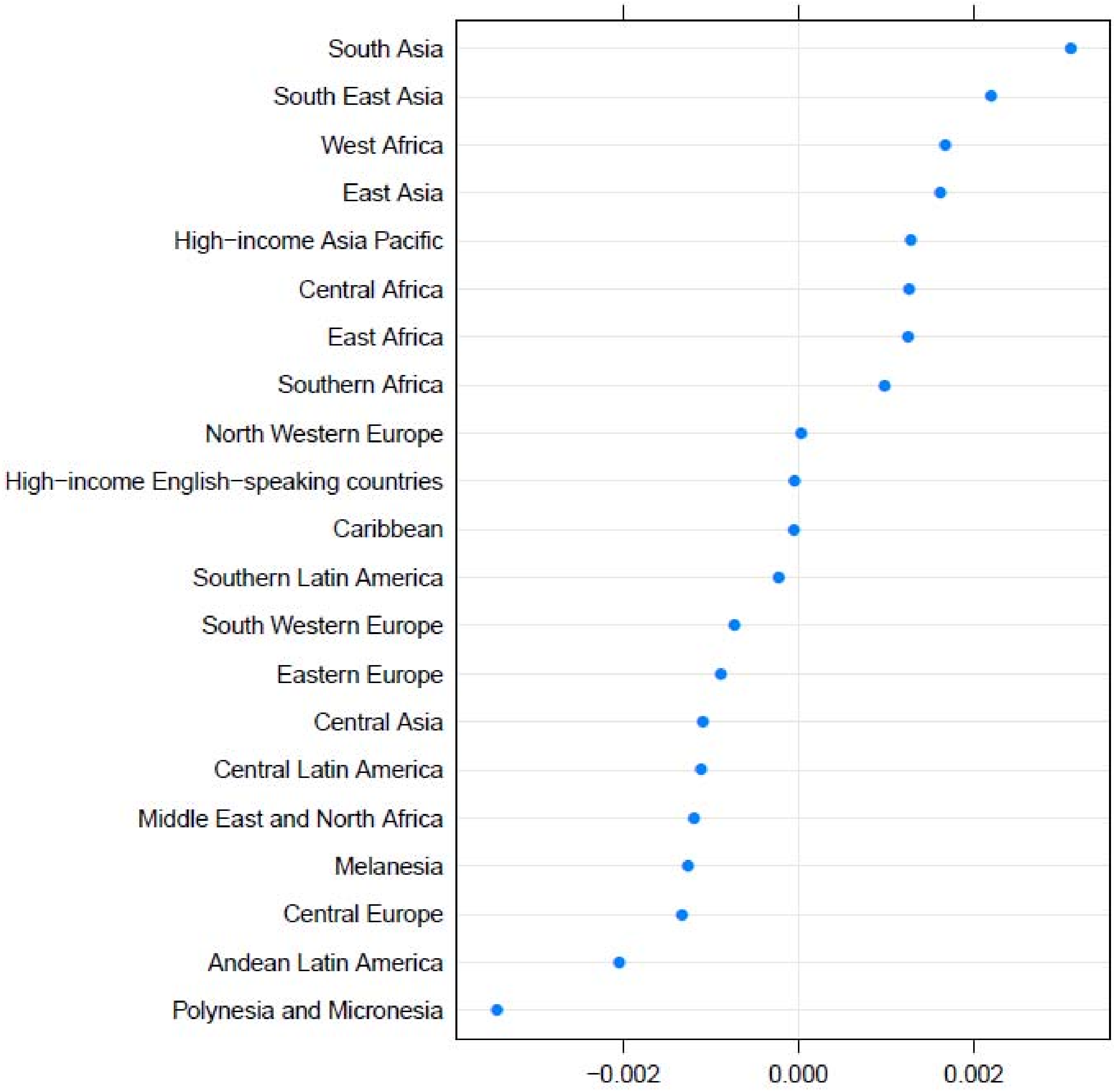

South Asia
South East Asia
West Africa
East Asia
High−income Asia Pacific
Central Africa
East Africa
Southern Africa
North Western Europe
High−income English−speaking countries
Caribbean
Southern Latin America
South Western Europe
Eastern Europe
Central Asia
Central Latin America
Middle East and North Africa
Melanesia
Central Europe
Andean Latin America
Polynesia and Micronesia
−0.002
0.000
0.002

**Dependent variable: Prevalence (BMI <18.5 kg/m^2)**	
**Age range: 18 years and older**	
**Independent variable: Inverse mean BMI**	
**Variables**	**Coefficients (95% CI)**
Intercept	-6.84 (-6.94 – -6.74)
Inverse mean BMI	114 (114 – 115)
Mean age of age group	-0.0066 (-0.0073 – -0.0059)
Male sex	-0.501 (-0.525 – -0.476)
Study mid-year (per one more recent year since 1975)	0.00051 (0.00038 – 0.00065)
Natural logarithm of per-capita gross domestic product	0.049 (0.046 – 0.051)
Inverse mean BMI * mean age of age group	0.194 (0.179 – 0.210)
Inverse mean BMI * male sex	8.46 (7.91 – 9.01)
**Number of data points used to fit the model = 11,654**	

Traditional R^2 is not clearly defined for mixed-effect models. The conditional R^2 for the model, which describes the proportion of variance explained by both fixed and random factors,[11] was 0.9077.

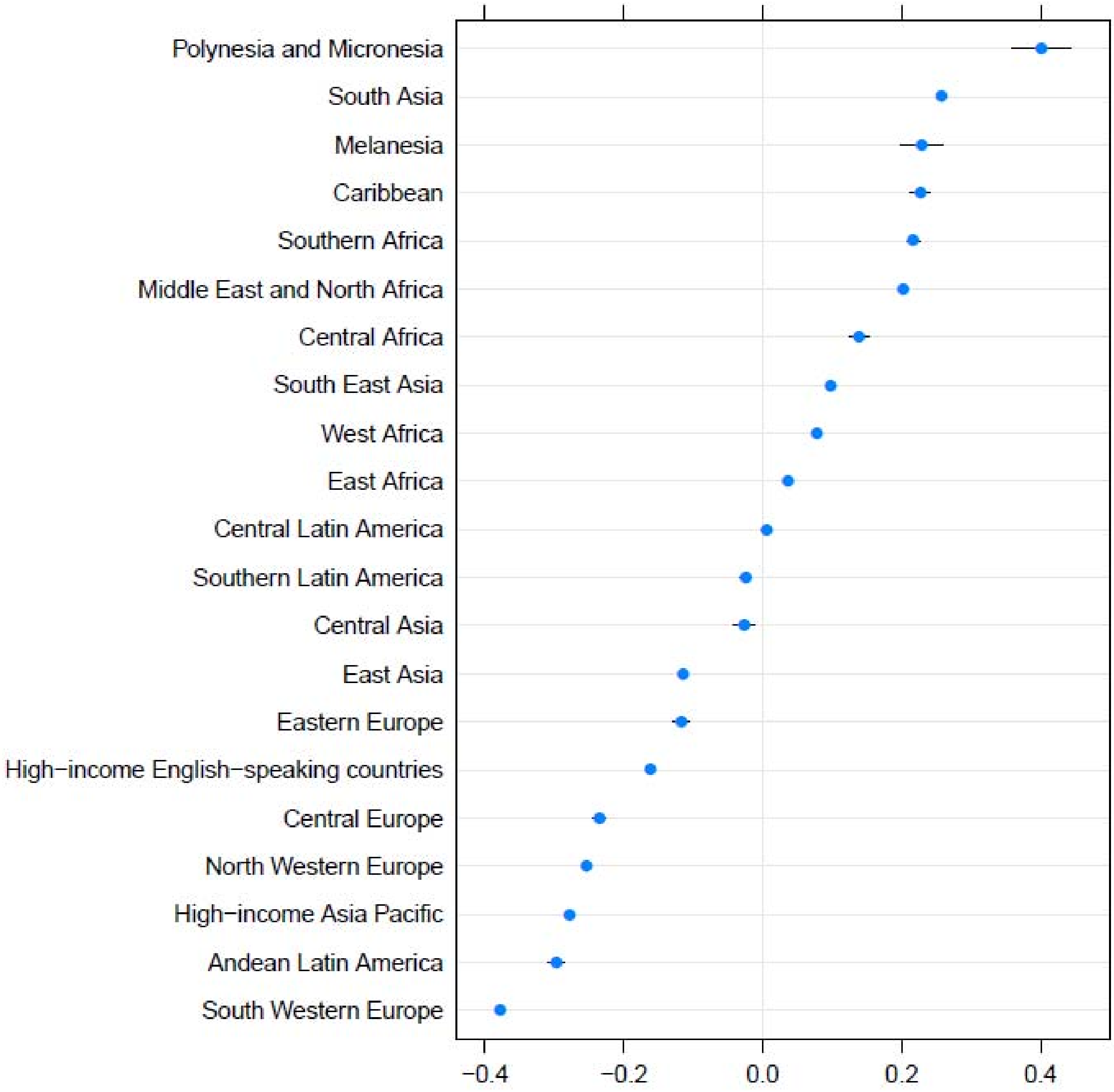
Polynesia and Micronesia
South Asia
Melanesia
Caribbean
Southern Africa
Middle East and North Africa
Central Africa
South East Asia
West Africa
East Africa
Central Latin America
Southern Latin America
Central Asia
East Asia
Eastern Europe
High−income English−speaking countries
Central Europe
North Western Europe
High−income Asia Pacific
Andean Latin America
South Western Europe
−0.4
−0.2
0.0
0.2
0.4

**Dependent variable: Prevalence (BMI ≥18.5 kg/m^2 and <20 kg/m^2)**	
**Age range: 18 years and older**	
**Independent variable: Inverse mean BMI**	
**Variables**	**Coefficients (95% CI)**
Intercept	-0.406 (-0.444 – -0.367)
Inverse (mean BMI – 19.25)	-77.8 (-78.6 – -77.1)
Mean age of age group	-0.0017 (-0.0018 – -0.0016)
Male sex	0.121 (0.117 – 0.125)
Study mid-year (per one more recent year since 1975)	0.000092 (-0.000019 – 0.00020)
Natural logarithm of per-capita gross domestic product	0.0048 (0.0026 – 0.0071)
[Inverse (mean BMI – 19.25)] * mean age of age group	-0.277 (-0.291 – -0.263)
[Inverse (mean BMI – 19.25)] * male sex	-21.5 (-22 – -21)
**Number of data points used to fit the model = 12,650**	

Traditional R^2 is not clearly defined for mixed-effect models. The conditional R^2 for the model, which describes the proportion of variance explained by both fixed and random factors,[11] was 0.8623.

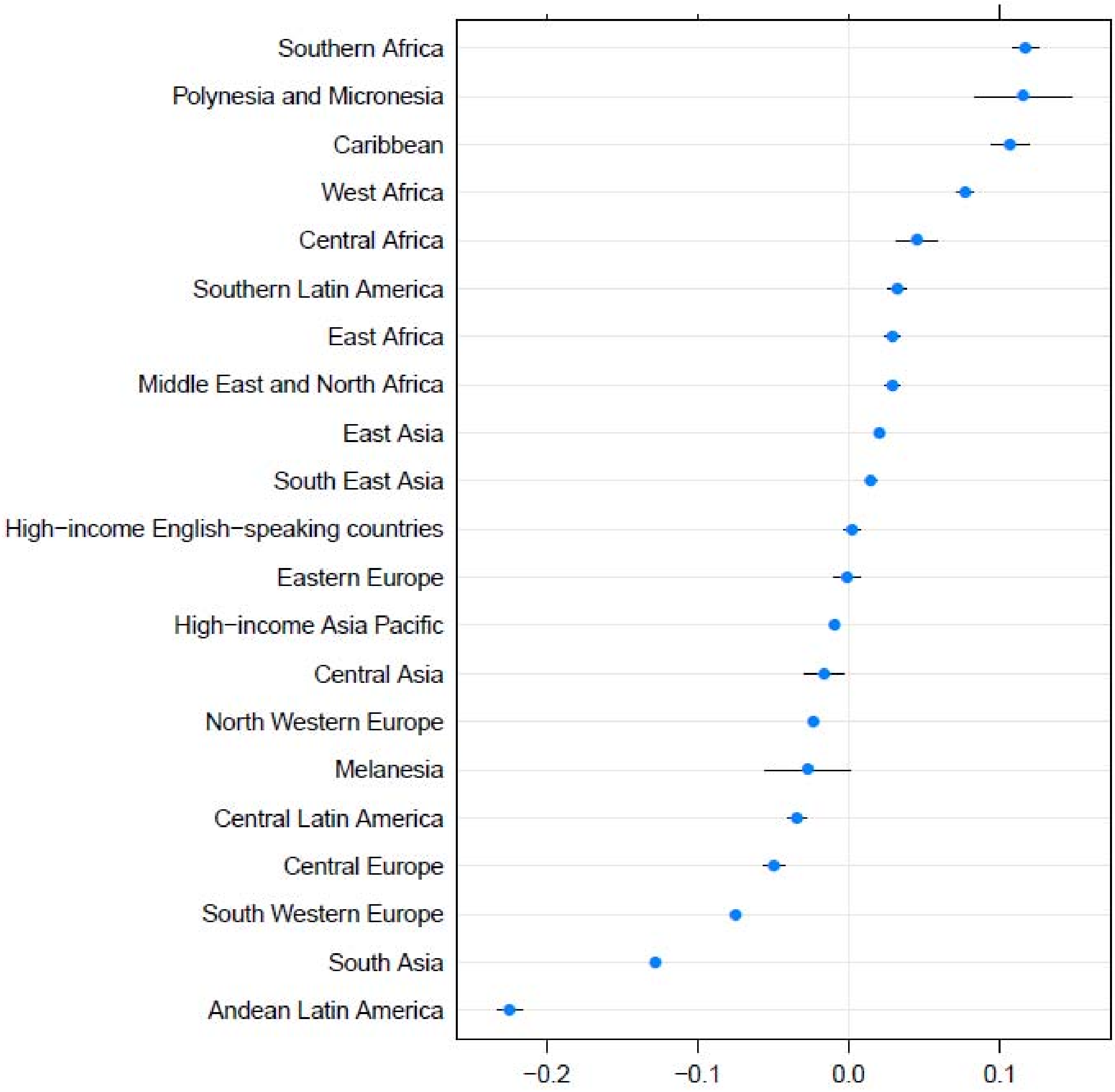
Southern Africa
Polynesia and Micronesia
Caribbean
West Africa
Central Africa
Southern Latin America
East Africa
Middle East and North Africa
East Asia
South East Asia
High−income English−speaking countries
Eastern Europe
High−income Asia Pacific
Central Asia
North Western Europe
Melanesia
Central Latin America
Central Europe
South Western Europe
South Asia
Andean Latin America
−0.2
−0.1
0.0
0.1

Dependent variable: Prevalence (BMI ≥20 kg/m^2 and <25 kg/m^2)	
**Age range: 18 years and older**	
**Independent variable: Inverse mean BMI**	
**Variables**	**Coefficients (95% CI)**
Intercept	0.319 (0.281 – 0.357)
Inverse (mean BMI – 22.5)	-56.1 (-56.9 – -55.4)
Mean age of age group	0.00052 (0.00045 – 0.00059)
Male sex	0.106 (0.104 – 0.108)
Study mid-year (per one more recent year since 1975)	-0.00076 (-0.00084 – -0.00068)
Natural logarithm of per-capita gross domestic product	-0.011 (-0.013 – -0.0094)
[Inverse (mean BMI – 22.5)] * mean age of age group	-0.706 (-0.722 – -0.691)
[Inverse (mean BMI – 22.5)] * male sex	-17.3 (-17.8 – -16.8)
**Number of data points used to fit the model = 13,481**	

Traditional R^2 is not clearly defined for mixed-effect models. The conditional R^2 for the model, which describes the proportion of variance explained by both fixed and random factors,[11] was 0.8381.

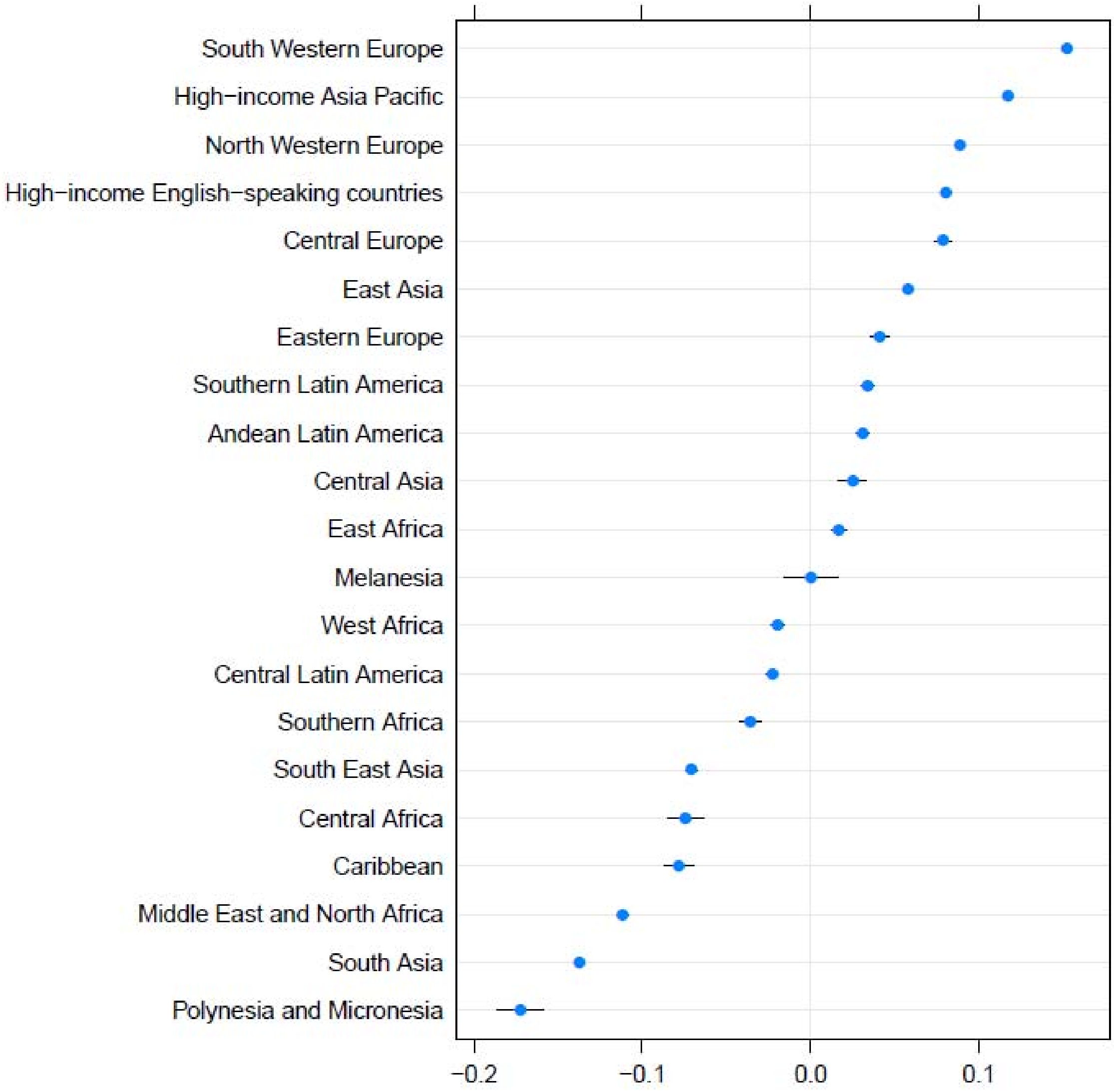
South Western Europe
High−income Asia Pacific
North Western Europe
High−income English−speaking countries
Central Europe
East Asia
Eastern Europe
Southern Latin America
Andean Latin America
Central Asia
East Africa
Melanesia
West Africa
Central Latin America
Southern Africa
South East Asia
Central Africa
Caribbean
Middle East and North Africa
South Asia
Polynesia and Micronesia
−0.2
−0.1
0.0
0.1

Dependent variable: Prevalence (BMI ≥25 kg/m^2 and <30 kg/m^2)	
**Age range: 18 years and older**	
**Independent variable: Inverse mean BMI**	
**Variables**	**Coefficients (95% CI)**
Intercept	-0.181 (-0.211 – -0.150)
Inverse (mean BMI – 27.5)	-121 (-121 – -120)
Mean age of age group	0.0013 (0.0013 – 0.0014)
Male sex	0.286 (0.283 – 0.289)
Study mid-year (per one more recent year since 1975)	-0.0012 (-0.0013 – -0.0011)
Natural logarithm of per-capita gross domestic product	-0.000049 (-0.0019 – 0.0018)
[Inverse (mean BMI – 27.5)] * mean age of age group	0.329 (0.316 – 0.342)
[Inverse (mean BMI – 27.5)] * male sex	-35.2 (-35.7 – -34.8)
**Number of data points used to fit the model = 13,408**	

Traditional R^2 is not clearly defined for mixed-effect models. The conditional R^2 for the model, which describes the proportion of variance explained by both fixed and random factors,[11] was 0.9182.

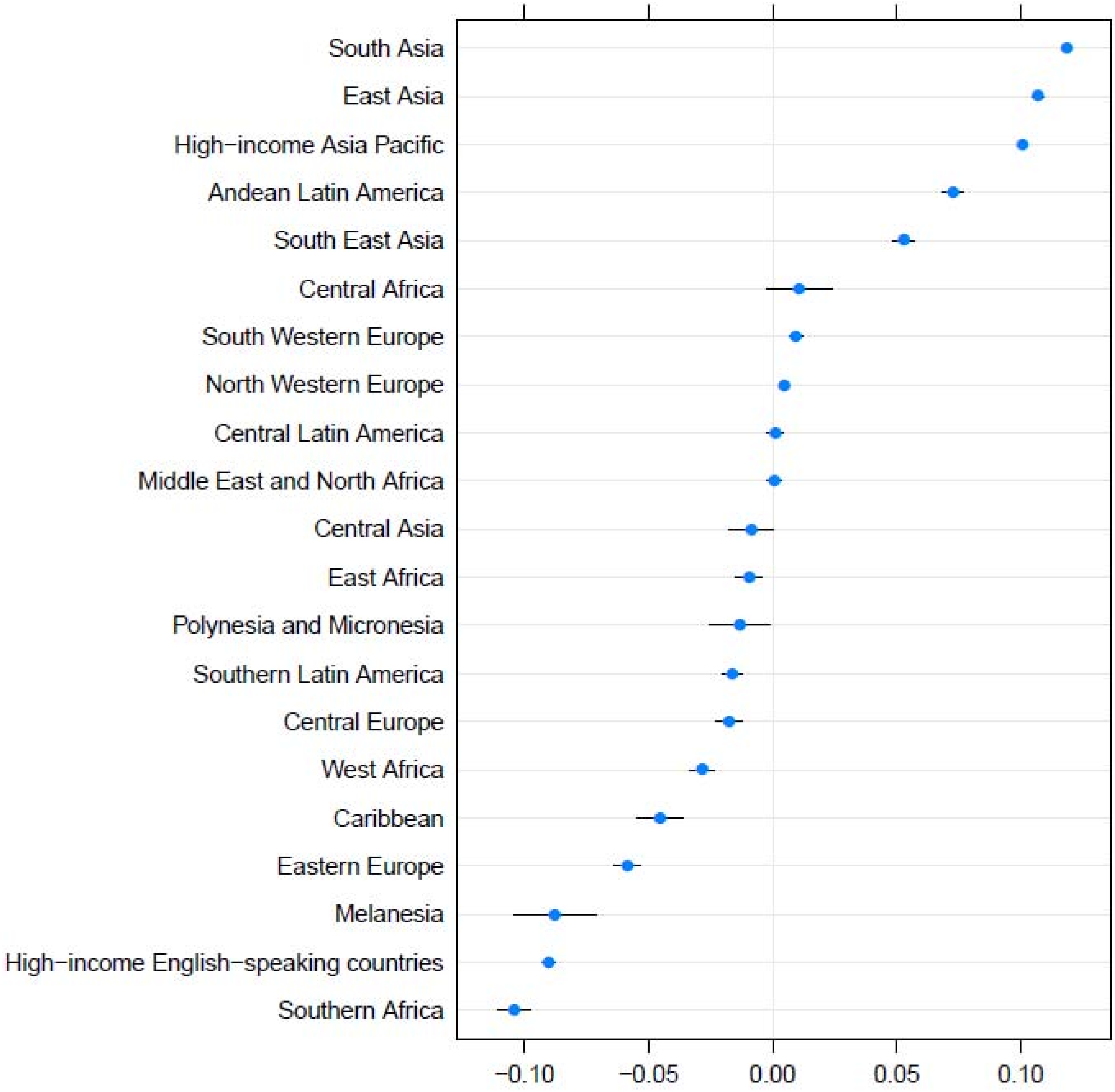
South Asia
East Asia
High−income Asia Pacific
Andean Latin America
South East Asia
Central Africa
South Western Europe
North Western Europe
Central Latin America
Middle East and North Africa
Central Asia
East Africa
Polynesia and Micronesia
Southern Latin America
Central Europe
West Africa
Caribbean
Eastern Europe
Melanesia
High−income English−speaking countries
Southern Africa
−0.10
−0.05
0.00
0.05
0.10

**Dependent variable: Prevalence (BMI ≥30 kg/m^2 and <35 kg/m^2)**	
**Age range: 18 years and older**	
**Independent variable: Inverse mean BMI**	
**Variables**	**Coefficients (95% CI)**
Intercept	-0.684 (-0.725 – -0.644)
Inverse (mean BMI – 32.5)	-101 (-102 – -100)
Mean age of age group	0.0014 (0.0012 – 0.0016)
Male sex	0.107 (0.102 – 0.113)
Study mid-year (per one more recent year since 1975)	0.0020 (0.0019 – 0.0021)
Natural logarithm of per-capita gross domestic product	0.024 (0.021 – 0.027)
[Inverse (mean BMI – 32.5)] * mean age of age group	-0.14 (-0.16 – -0.12)
[Inverse (mean BMI – 32.5)] * male sex	-20.2 (-20.8 – -19.7)
**Number of data points used to fit the model = 12,594**	

Traditional R^2 is not clearly defined for mixed-effect models. The conditional R^2 for the model, which describes the proportion of variance explained by both fixed and random factors,[11] was 0.9065.

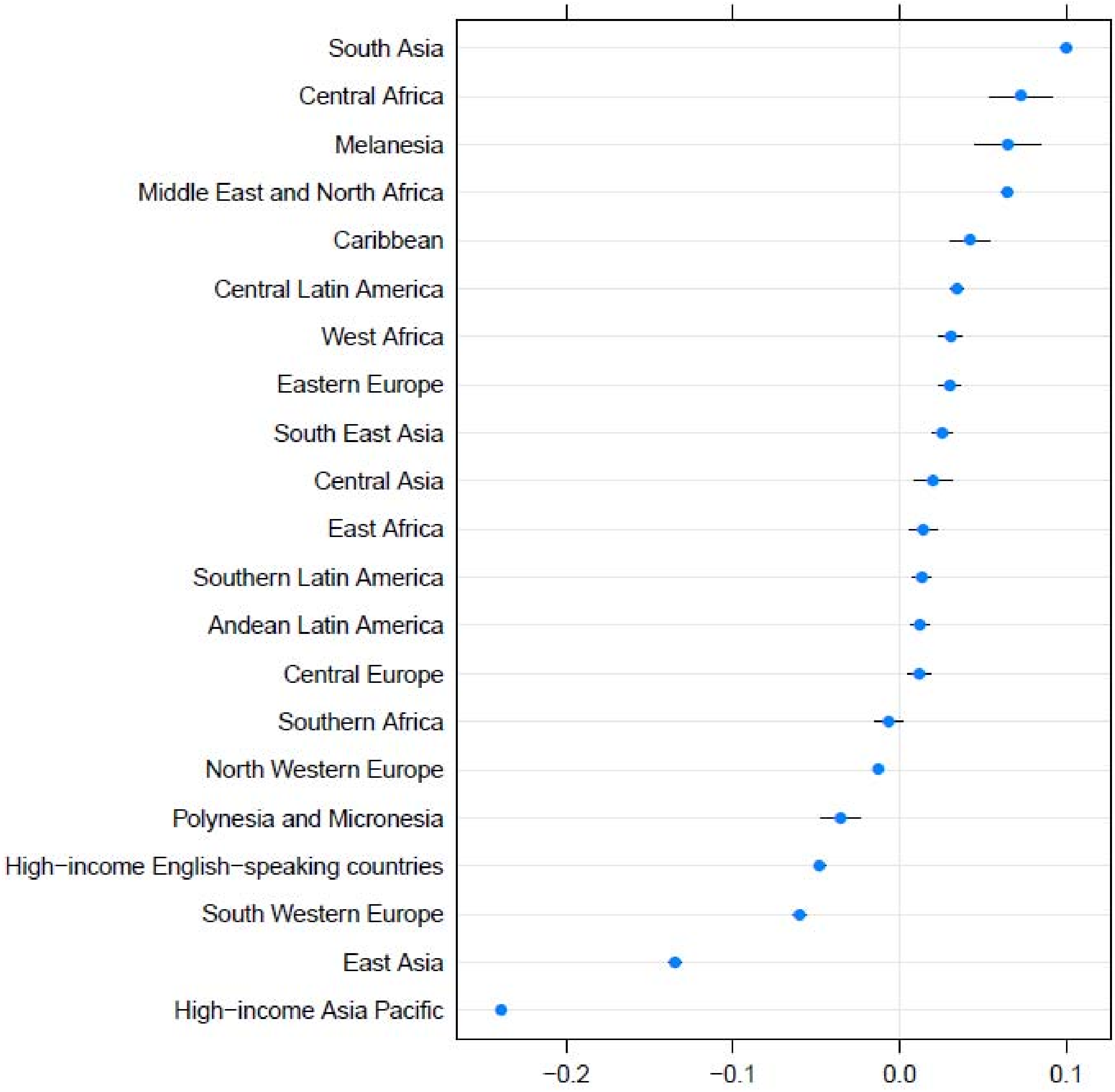
South Asia
Central Africa
Melanesia
Middle East and North Africa
Caribbean
Central Latin America
West Africa
Eastern Europe
South East Asia
Central Asia
East Africa
Southern Latin America
Andean Latin America
Central Europe
Southern Africa
North Western Europe
Polynesia and Micronesia
High−income English−speaking countries
South Western Europe
East Asia
High−income Asia Pacific
−0.2
−0.1
0.0
0.1

**Dependent variable: Prevalence (BMI ≥35 kg/m^2 and <40 kg/m^2)**	
**Age range: 18 years and older**	
**Independent variable: Inverse mean BMI**	
**Variables**	**Coefficients (95% CI)**
Intercept	-1.08 (-1.15 – -1.01)
Inverse (mean BMI – 37.5)	-92.7 (-94.2 – -91.3)
Mean age of age group	0.0017 (0.0013 – 0.0021)
Male sex	-0.237 (-0.250 – -0.225)
Study mid-year (per one more recent year since 1975)	0.0057 (0.0055 – 0.0059)
Natural logarithm of per-capita gross domestic product	0.032 (0.027 – 0.036)
[Inverse (mean BMI – 37.5)] * mean age of age group	-0.397 (-0.428 – -0.367)
[Inverse (mean BMI – 37.5)] * male sex	1.62 (0.70 – 2.54)
**Number of data points used to fit the model = 10,491**	

Traditional R^2 is not clearly defined for mixed-effect models. The conditional R^2 for the model, which describes the proportion of variance explained by both fixed and random factors,[11] was 0.8581.

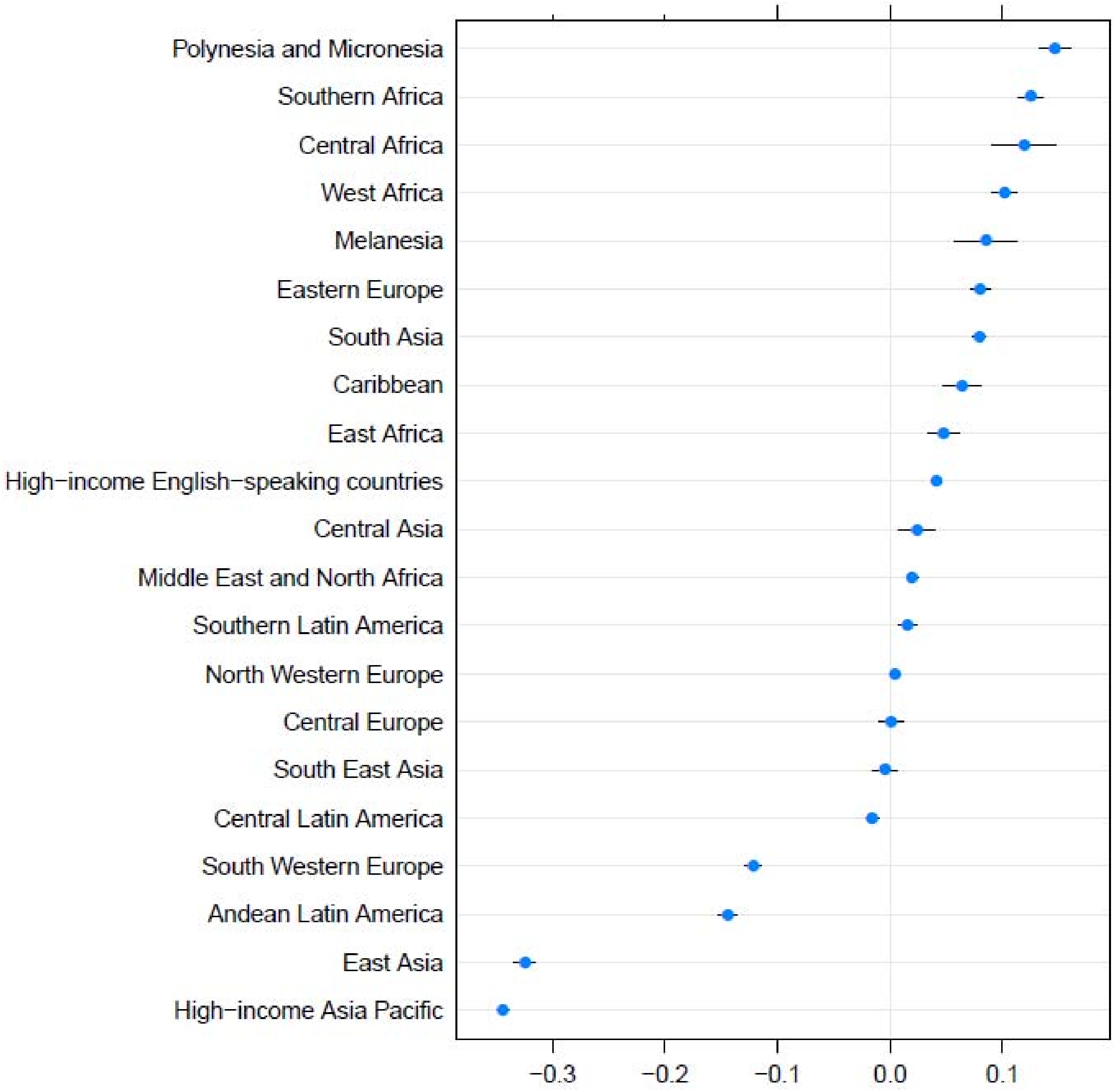
Polynesia and Micronesia
Southern Africa
Central Africa
West Africa
Melanesia
Eastern Europe
South Asia
Caribbean
East Africa
High−income English−speaking countries
Central Asia
Middle East and North Africa
Southern Latin America
North Western Europe
Central Europe
South East Asia
Central Latin America
South Western Europe
Andean Latin America
East Asia
High−income Asia Pacific
−0.3
−0.2
−0.1
0.0
0.1

**Dependent variable: Prevalence (BMI ≥40 kg/m^2)**	
**Age range: 18 years and older**	
**Independent variable: Inverse mean BMI**	
**Variables**	**Coefficients (95% CI)**
Intercept	1.07 (0.93 – 1.20)
Inverse mean BMI	-102 (-104 – -99.9)
Mean age of age group	0.0025 (0.00079 – 0.0042)
Male sex	-1.05 (-1.11 – -1.00)
Study mid-year (per one more recent year since 1975)	0.0078 (0.0075 – 0.0081)
Natural logarithm of per-capita gross domestic product	0.065 (0.059 – 0.071)
Inverse mean BMI * mean age of age group	-0.199 (-0.244 – -0.155)
Inverse mean BMI * male sex	20.6 (19.3 – 22.0)
**Number of data points used to fit the model = 8,113**	

Traditional R^2 is not clearly defined for mixed-effect models. The conditional R^2 for the model, which describes the proportion of variance explained by both fixed and random factors,[11] was 0.8067.

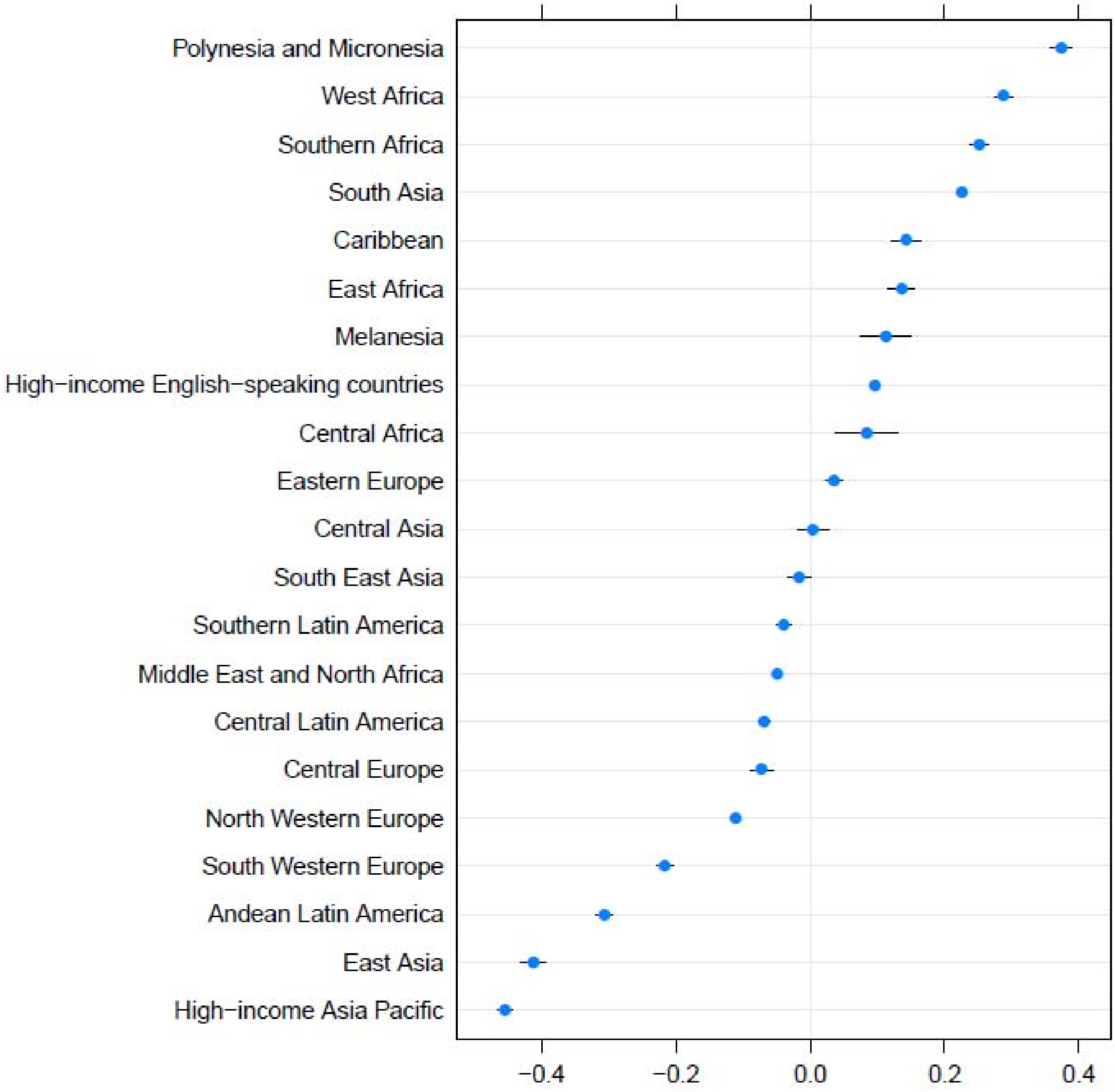
Polynesia and Micronesia
West Africa
Southern Africa
South Asia
Caribbean
East Africa
Melanesia
High−income English−speaking countries
Central Africa
Eastern Europe
Central Asia
South East Asia
Southern Latin America
Middle East and North Africa
Central Latin America
Central Europe
North Western Europe
South Western Europe
Andean Latin America
East Asia
High−income Asia Pacific
−0.4
−0.2
0.0
0.2
0.4

Appendix Figure 1: Number of data sources by country.

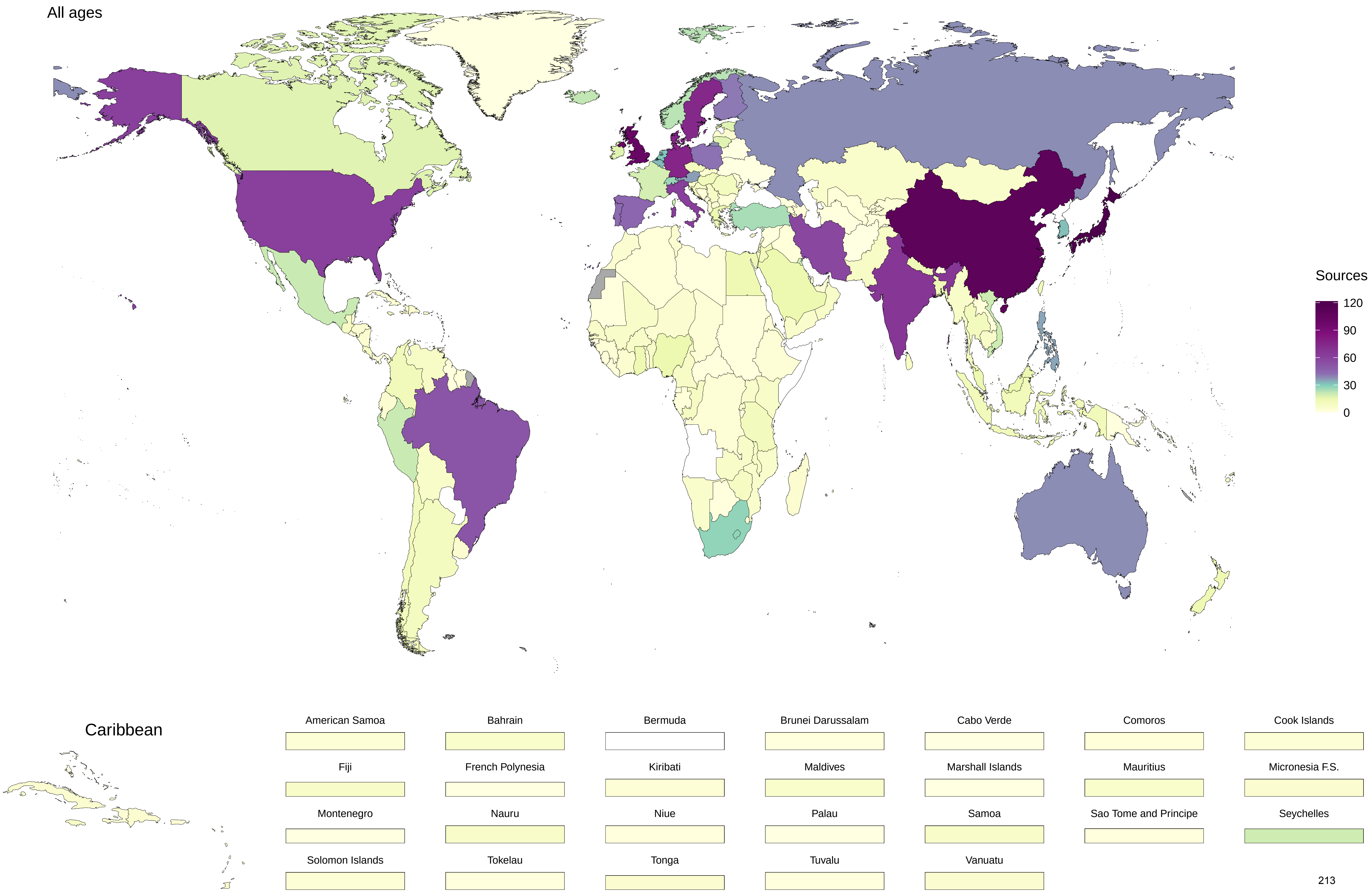
All ages
Sources
120
90
60
30
0
Caribbean
American Samoa
Bahrain
Bermuda
Brunei Darussalam
Cabo Verde
Comoros
Cook Islands
Fiji
French Polynesia
Kiribati
Maldives
Marshall Islands
Mauritius
Micronesia F.S.
Montenegro
Nauru
Niue
Palau
Samoa
Sao Tome and Principe
Seychelles
Solomon Islands
Tokelau
Tonga
Tuvalu
Vanuatu

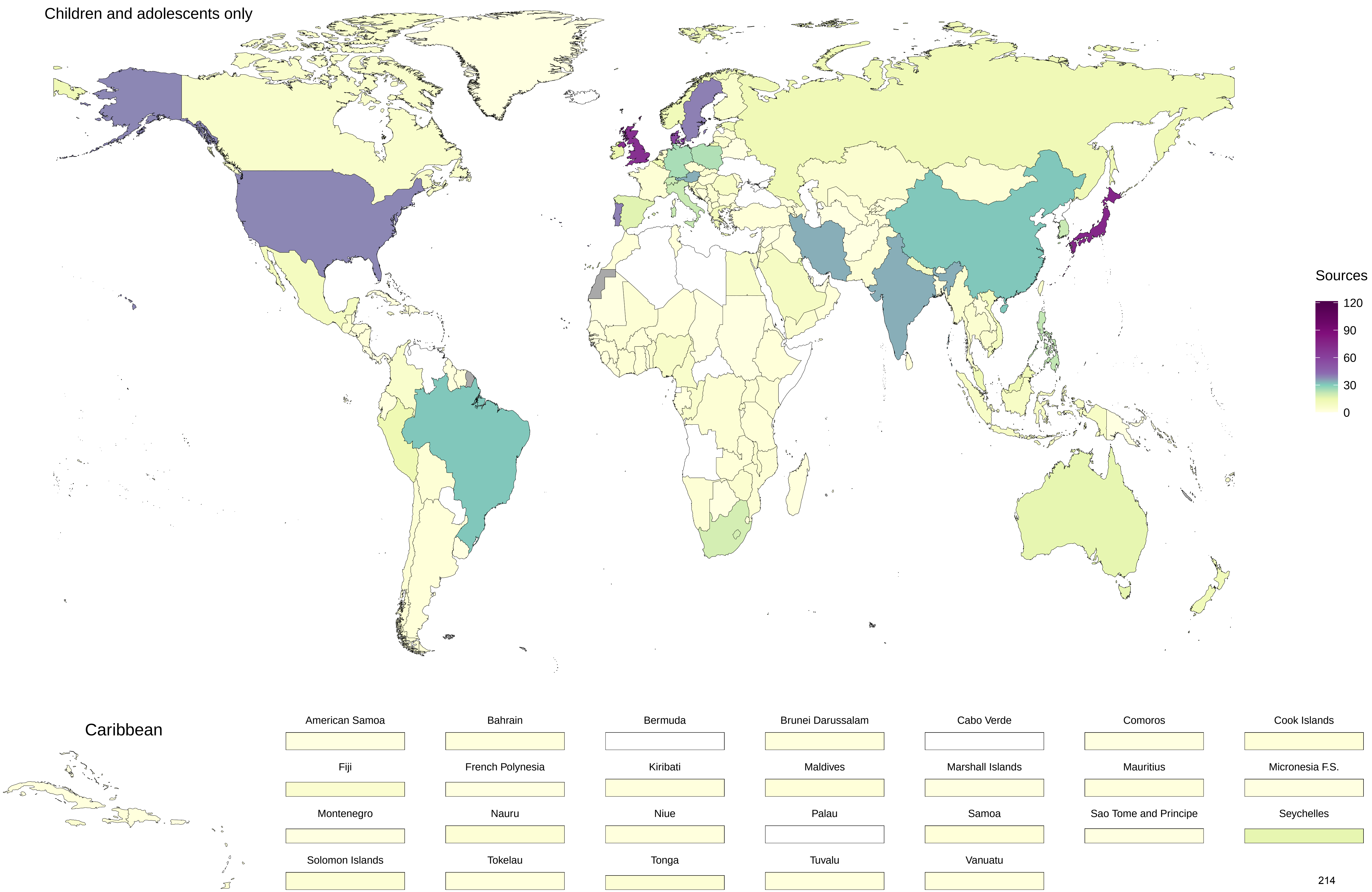

Children and adolescents only
Sources
120
90
60
30
0
Caribbean
American Samoa
Bahrain
Bermuda
Brunei Darussalam
Cabo Verde
Comoros
Cook Islands
Fiji
French Polynesia
Kiribati
Maldives
Marshall Islands
Mauritius
Micronesia F.S.
Montenegro
Nauru
Niue
Palau
Samoa
Sao Tome and Principe
Seychelles
Solomon Islands
Tokelau
Tonga
Tuvalu
Vanuatu

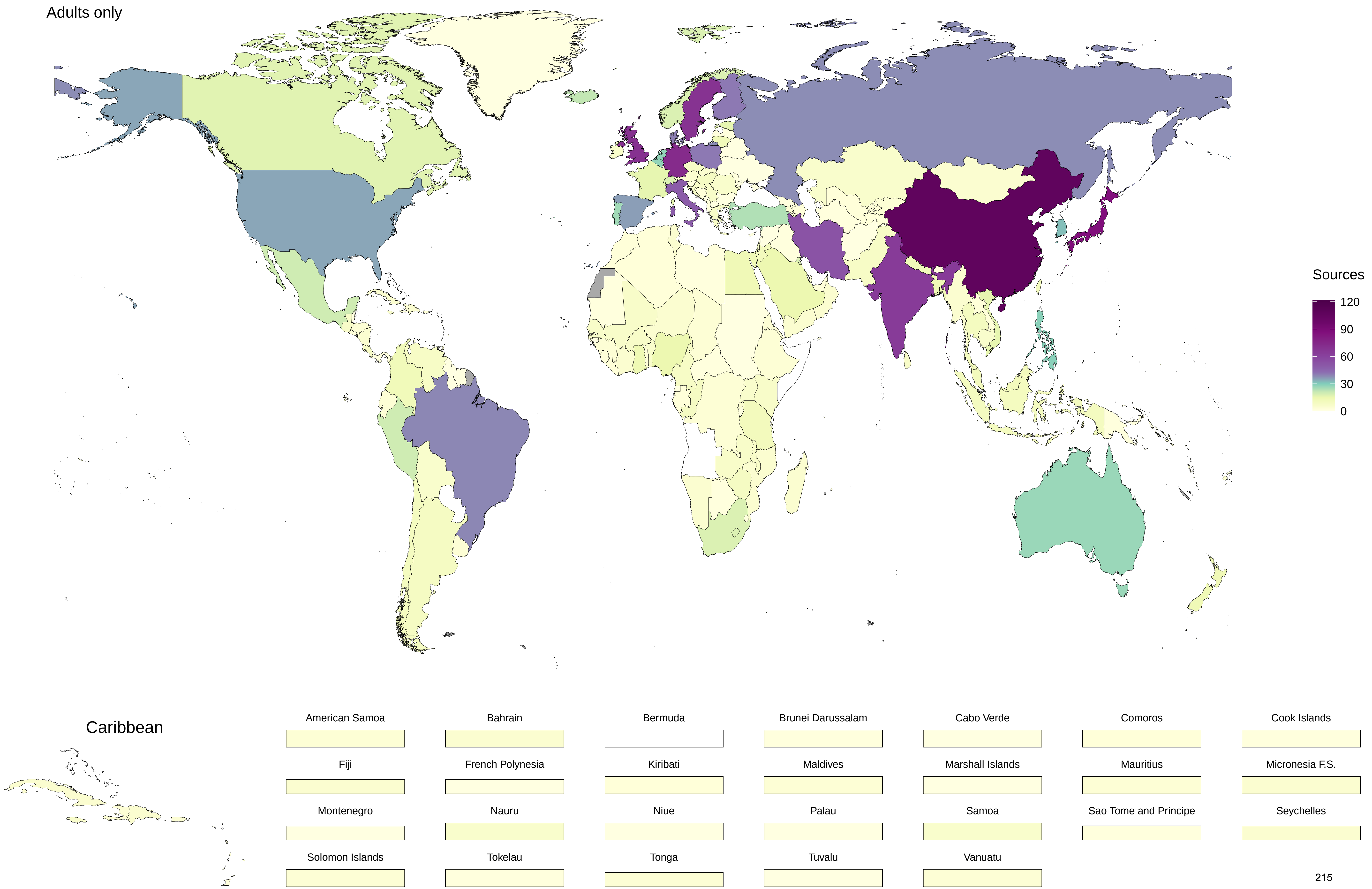
Adults only
Sources
120
90
60
30
0
Caribbean
American Samoa
Bahrain
Bermuda
Brunei Darussalam
Cabo Verde
Comoros
Cook Islands
Fiji
French Polynesia
Kiribati
Maldives
Marshall Islands
Mauritius
Micronesia F.S.
Montenegro
Nauru
Niue
Palau
Samoa
Sao Tome and Principe
Seychelles
Solomon Islands
Tokelau
Tonga
Tuvalu
Vanuatu

Appendix Figure 2: Number of data sources by region and year divided by whether they had measured height and weight in samples of a national, sub-national (i.e. covering one or more sub-national regions, or more than three communities) or community (one or a small number of communities) population.

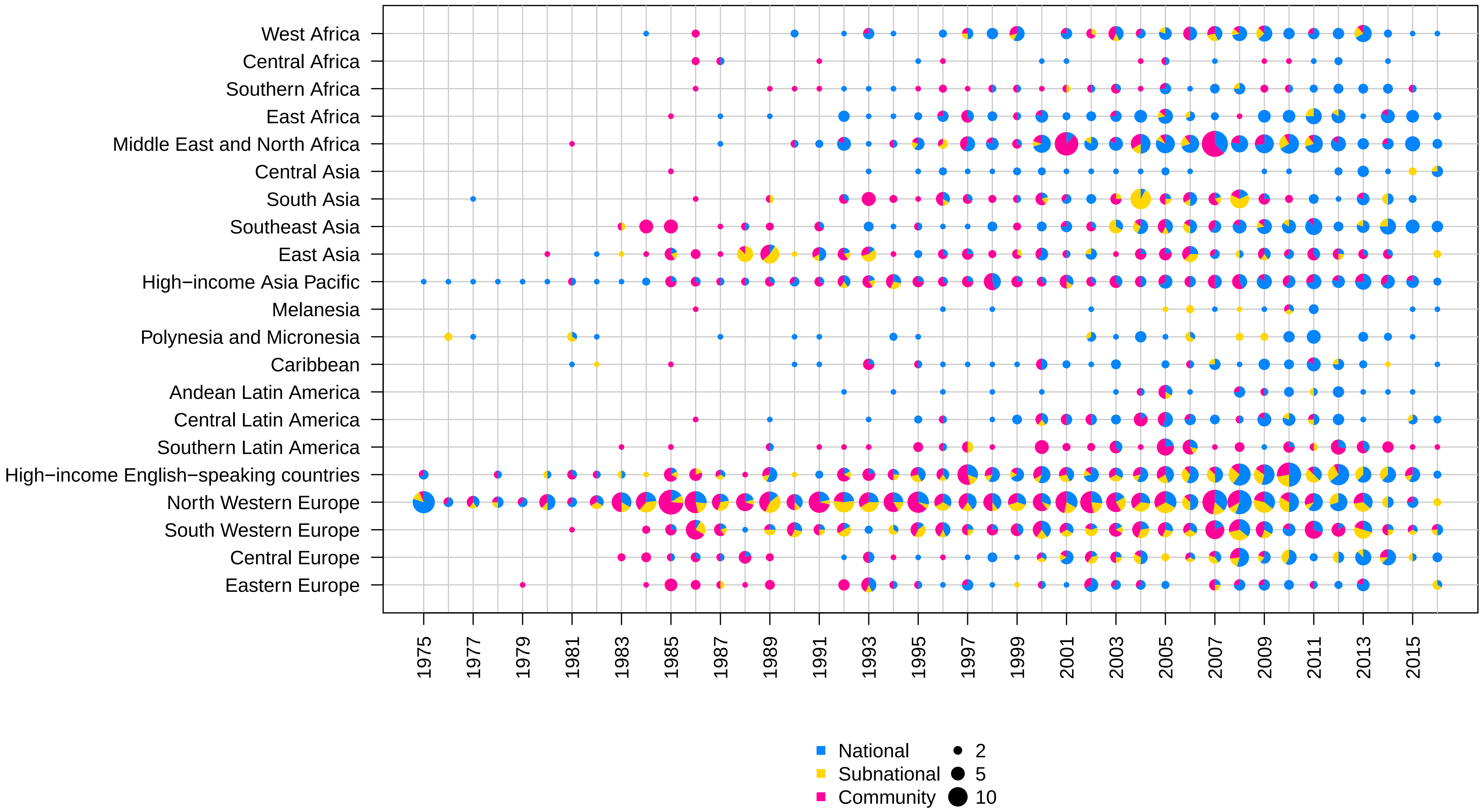
West Africa
Central Africa
Southern Africa
East Africa
Middle East and North Africa
Central Asia
South Asia
Southeast Asia
East Asia
High−income Asia Pacific
Melanesia
Polynesia and Micronesia
Caribbean
Andean Latin America
Central Latin America
Southern Latin America
High−income English−speaking countries
North Western Europe
South Western Europe
Central Europe
Eastern Europe
1975
1977
1979
1981
1983
1985
1987
1989
1991
1993
1995
1997
1999
2001
2003
2005
2007
2009
2011
2013
2015
National
Subnational
Community
2
5
10

Appendix Figure 3: Number of data sources by region and year by participants' age. Note that the same data source can have participants aged 5-9 years, 10-19 years, and 20 years and older. The same source may have data on more than one of these age groups and hence may appear in the segment for multiple age groups.

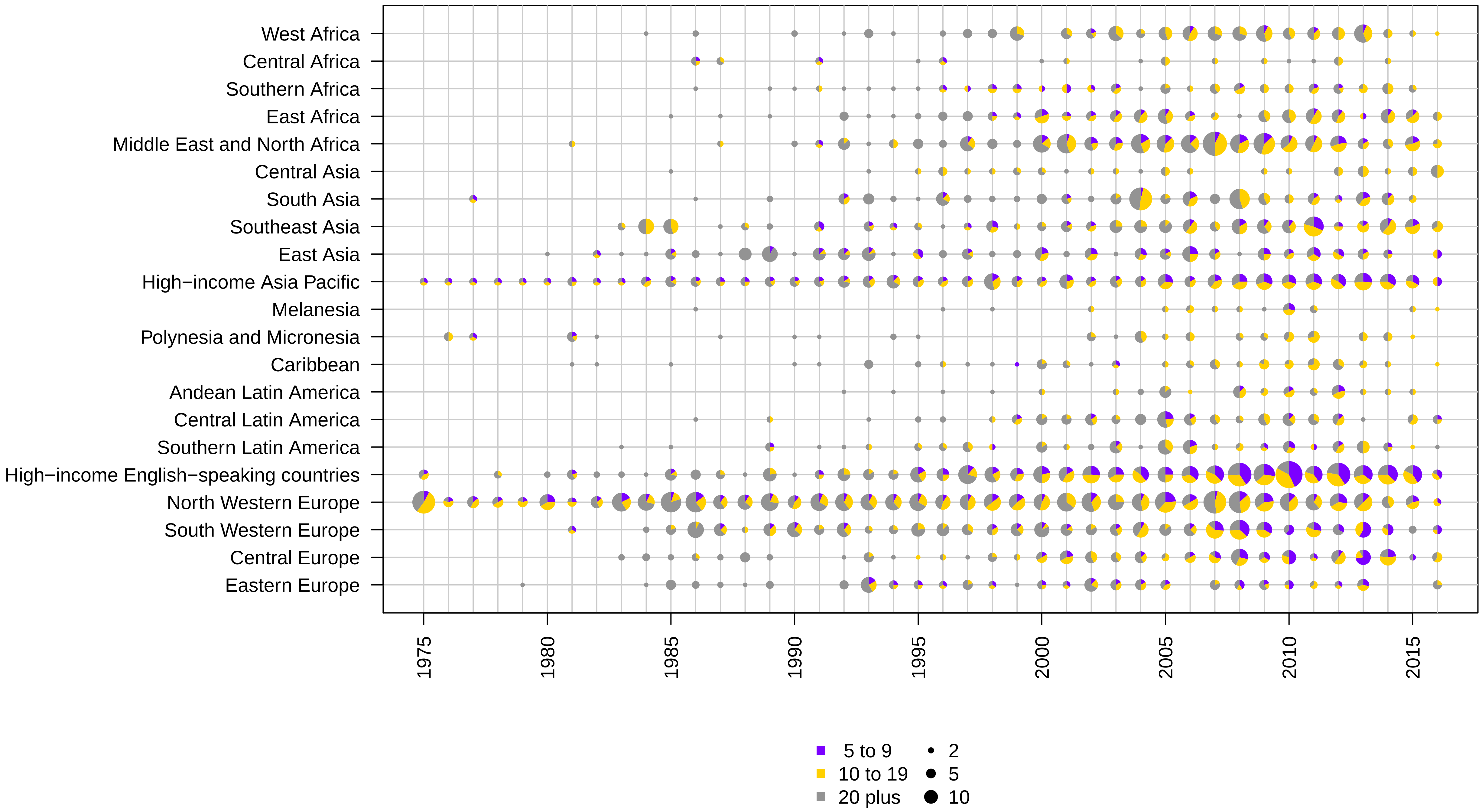
West Africa
Central Africa
Southern Africa
East Africa
Middle East and North Africa
Central Asia
South Asia
Southeast Asia
East Asia
High−income Asia Pacific
Melanesia
Polynesia and Micronesia
Caribbean
Andean Latin America
Central Latin America
Southern Latin America
High−income English−speaking countries
North Western Europe
South Western Europe
Central Europe
Eastern Europe
1975
1980
1985
1990
1995
2000
2005
2010
2015
5 to 9
10 to 19
20 plus
2
5
10

Appendix Figure 4: Age-standardised mean body mass index (BMI) in 2016 by region for children (aged 5-9 years) and adolescents (aged 10-19 years). The regions are ranked by their age-standardised mean BMI.

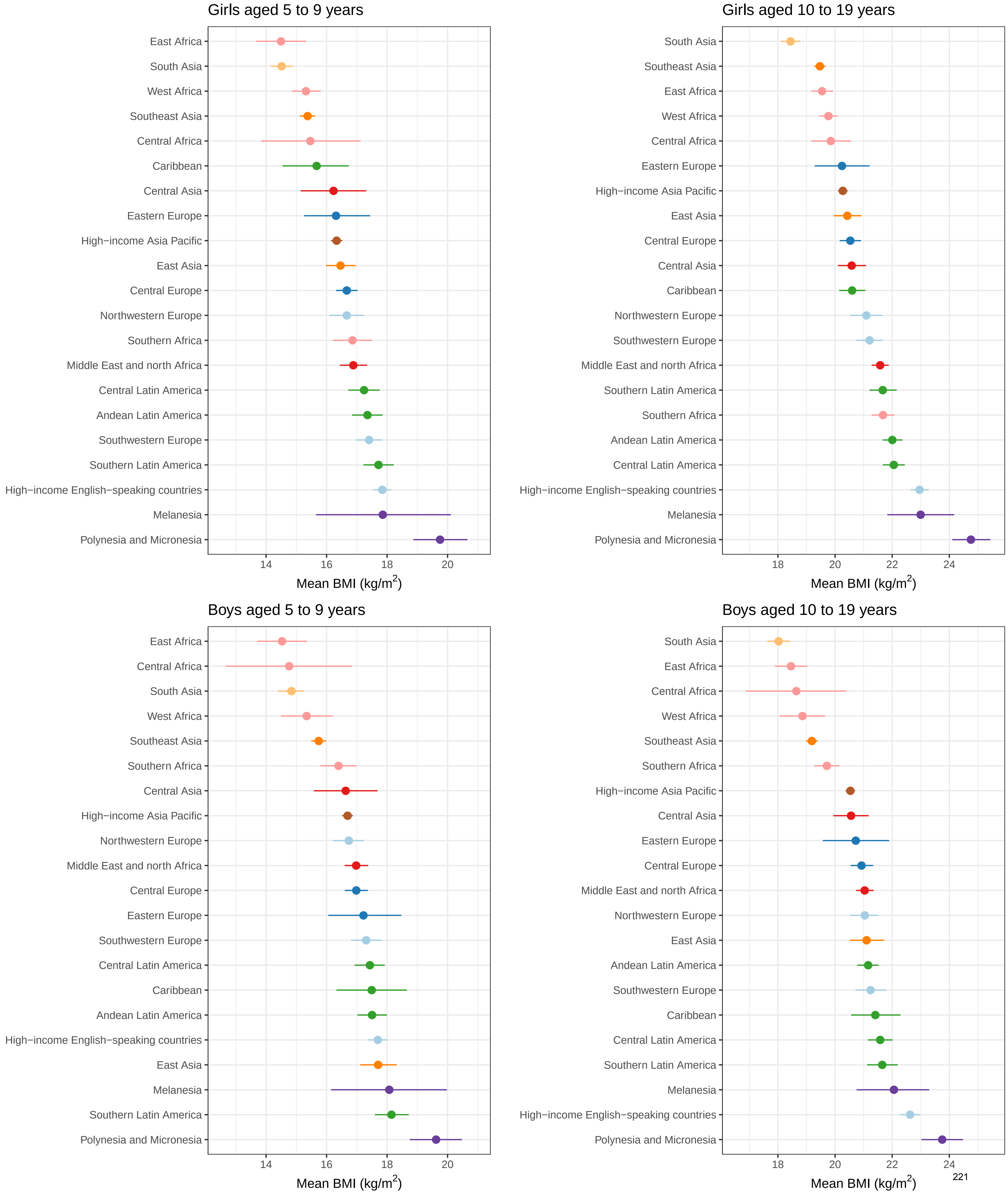
Girls aged 5 to 9 years
East Africa
South Asia
West Africa
Southeast Asia
Central Africa
Caribbean
Central Asia
Eastern Europe
High−income Asia Pacific
East Asia
Central Europe
Northwestern Europe
Southern Africa
Middle East and north Africa
Central Latin America
Andean Latin America
Southwestern Europe
Southern Latin America
High−income English−speaking countries
Melanesia
Polynesia and Micronesia
14
16
18
20
Mean BMI (kg/m^2)
Girls aged 10 to 19 years
South Asia
Southeast Asia
East Africa
West Africa
Central Africa
Eastern Europe
High−income Asia Pacific
East Asia
Central Europe
Central Asia
Caribbean
Northwestern Europe
Southwestern Europe
Middle East and north Africa
Southern Latin America
Southern Africa
Andean Latin America
Central Latin America
High−income English−speaking countries
Melanesia
Polynesia and Micronesia
18
20
22
24
Mean BMI (kg/m^2)
Boys aged 5 to 9 years
East Africa
Central Africa
South Asia
West Africa
Southeast Asia
Southern Africa
Central Asia
High−income Asia Pacific
Northwestern Europe
Middle East and north Africa
Central Europe
Eastern Europe
Southwestern Europe
Central Latin America
Caribbean
Andean Latin America
High−income English−speaking countries
East Asia
Melanesia
Southern Latin America
Polynesia and Micronesia
14
16
18
20
Mean BMI (kg/m^2)
Boys aged 10 to 19 years
South Asia
East Africa
Central Africa
West Africa
Southeast Asia
Southern Africa
High−income Asia Pacific
Central Asia
Eastern Europe
Central Europe
Middle East and north Africa
Northwestern Europe
East Asia
Andean Latin America
Southwestern Europe
Caribbean
Central Latin America
Southern Latin America
Melanesia
High−income English−speaking countries
Polynesia and Micronesia
18
20
22
24
Mean BMI (kg/m^2)

Appendix Figure 5: Trends in the number of obese school-aged children and adolescents (aged 5-19 years), decomposed into the contributions of population growth and changes in age structure, change in prevalence, and interaction of the two for the world as a whole.

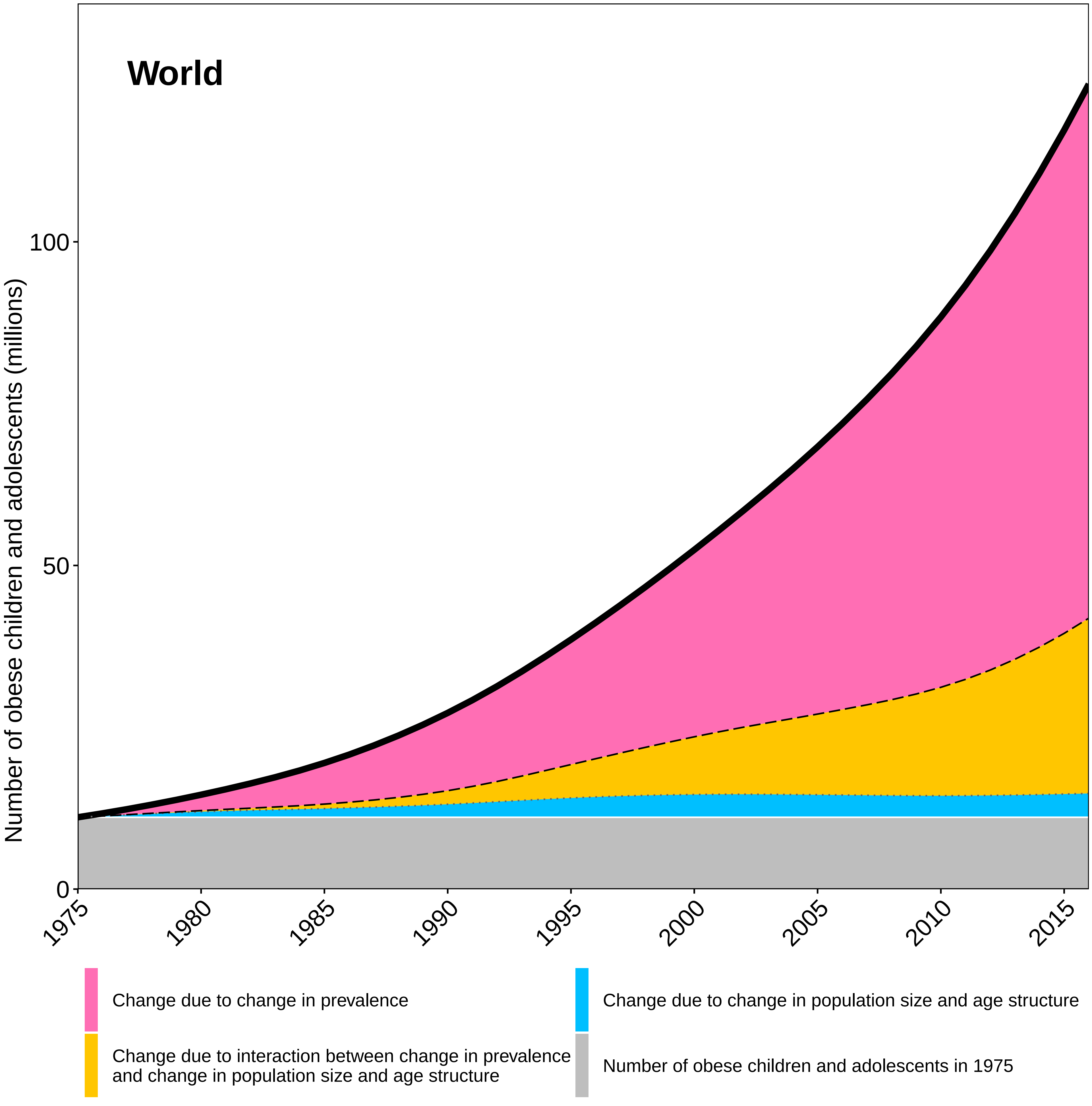
World
Number of obese children and adolescents (millions)
0
50
100
1975
1980
1985
1990
1995
2000
2005
2010
2015
Change due to change in prevalence
Change due to change in population size and age structure
Change due to interaction between change in prevalence and change in population size and age structure
Number of obese children and adolescents in 1975

Appendix Figure 6: Trends in age-standardised mean body mass index (BMI) and age-standardised prevalence of BMI categories by sex and country in school-aged children and adolescents (aged 5-19 years) and adults (aged 20 years and older) by country. In plots for mean BMI, the lines show the posterior mean estimates and the shaded area shows the 95% credible interval. The number of data sources (all data as well as nationally representative data) are shown for each country.

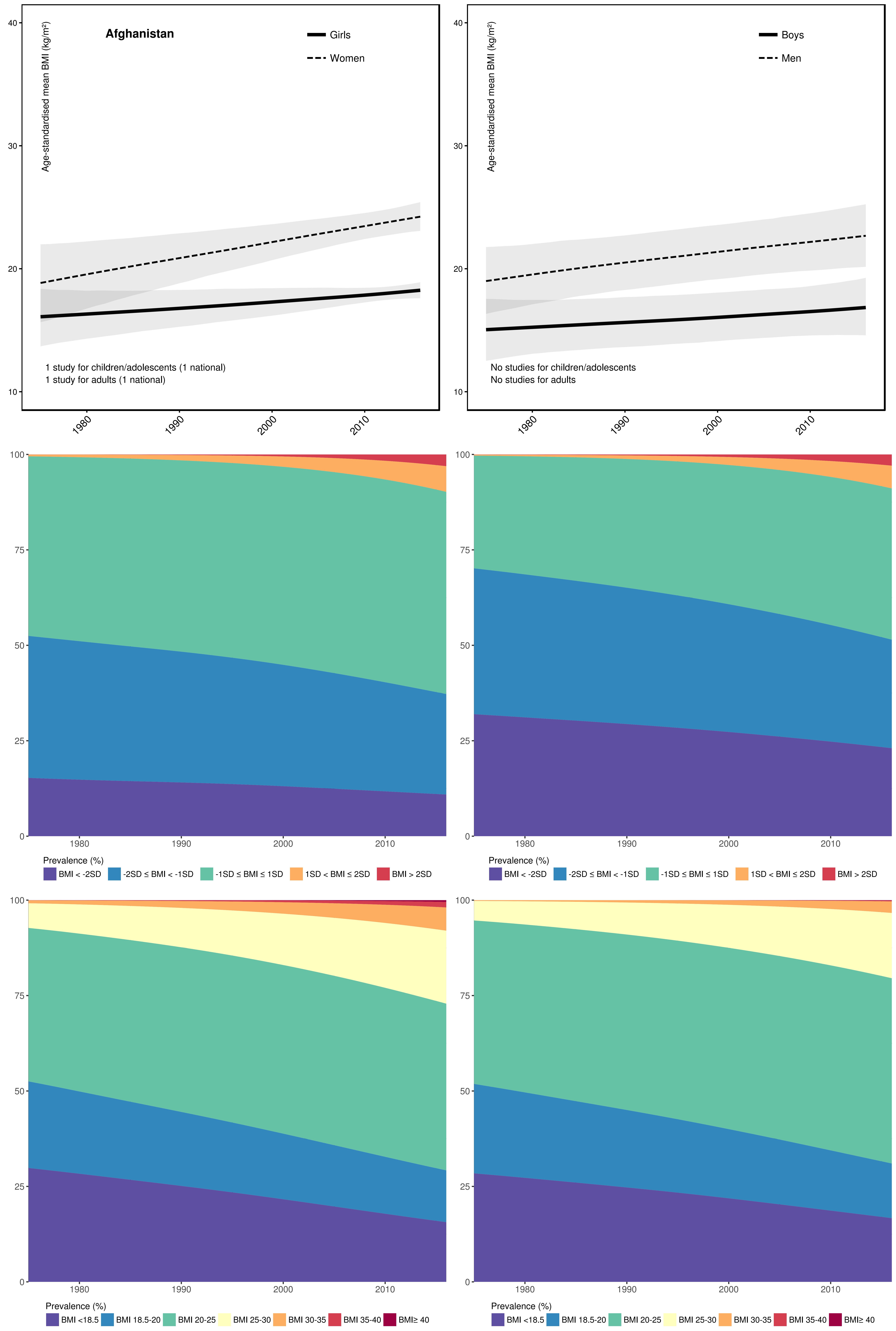

Afghanistan
Girls
Women
Boys
Men
Age-standardised mean BMI (kg/m²)
1 study for children/adolescents (1 national)
1 study for adults (1 national)
No studies for children/adolescents
No studies for adults
1980
1990
2000
2010
Prevalence (%)
BMI < -2SD
-2SD ≤ BMI < -1SD
-1SD ≤ BMI ≤ 1SD
1SD < BMI ≤ 2SD
BMI > 2SD
BMI <18.5
BMI 18.5-20
BMI 20-25
BMI 25-30
BMI 30-35
BMI 35-40
BMI≥ 40

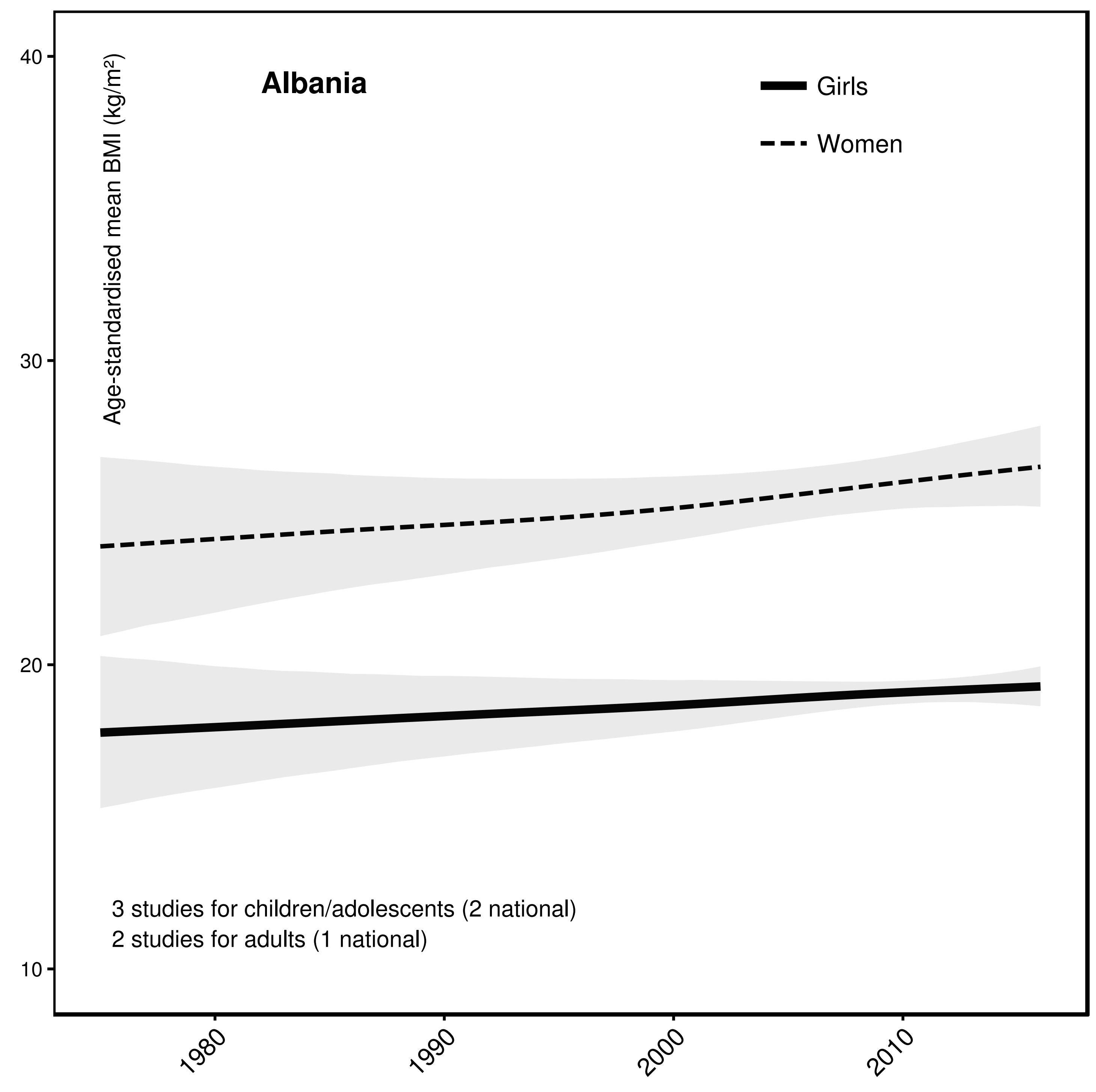
Albania
Girls
Women
Age-standardised mean BMI (kg/m²)
40
30
20
10
3 studies for children/adolescents (2 national)
2 studies for adults (1 national)
1980
1990
2000
2010

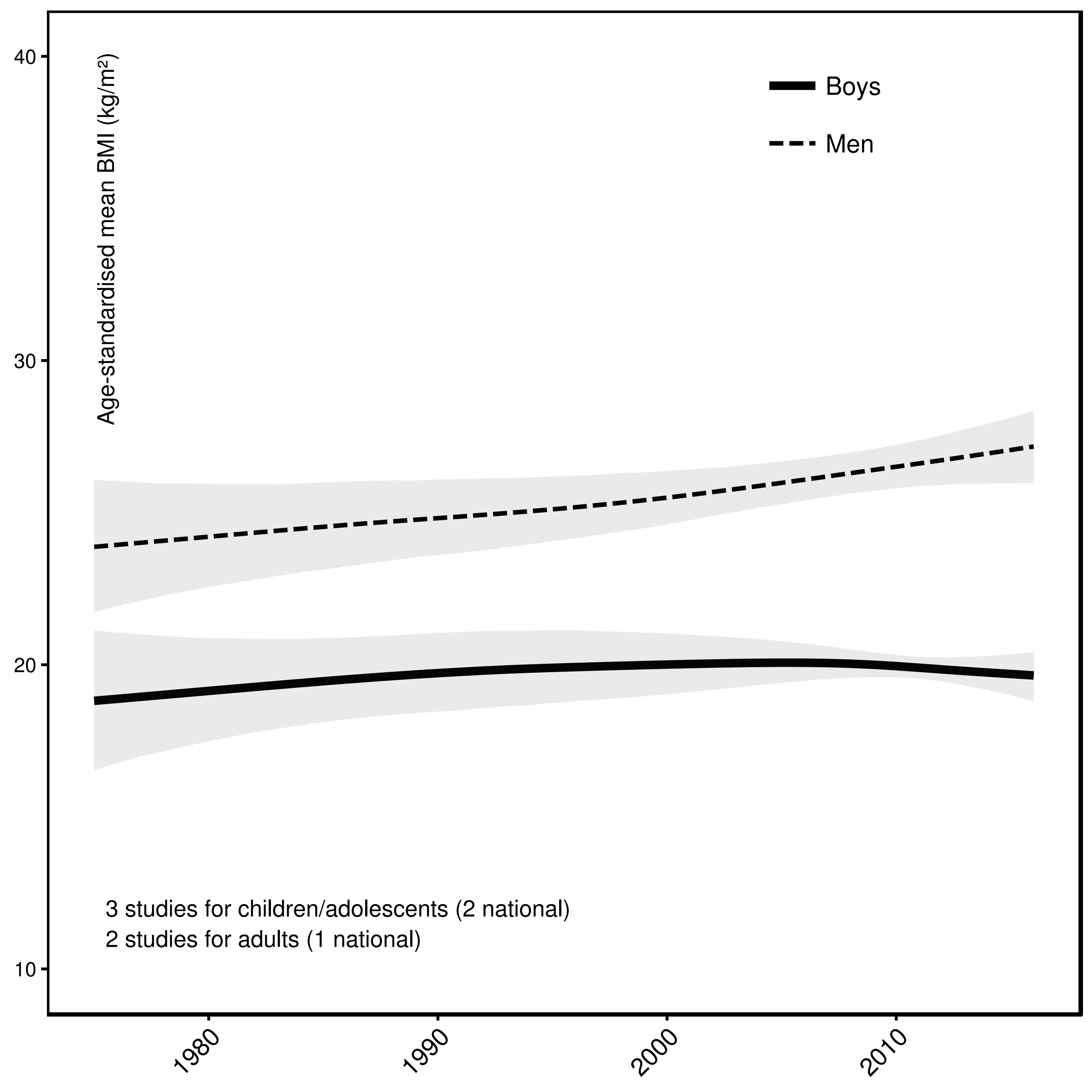
Boys
Men
Age-standardised mean BMI (kg/m²)
40
30
20
10
3 studies for children/adolescents (2 national)
2 studies for adults (1 national)
1980
1990
2000
2010

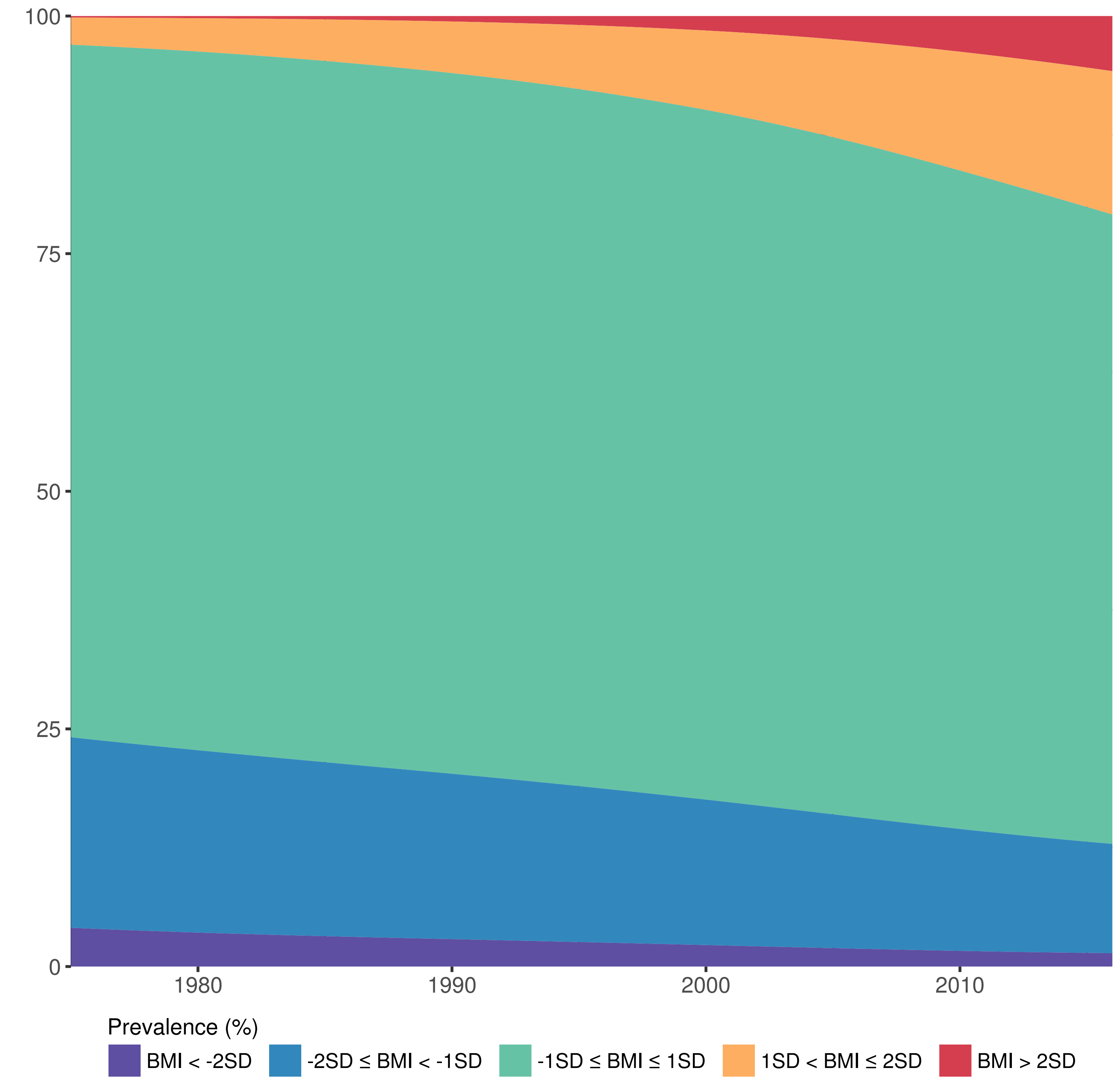
100
75
50
25
0
1980
1990
2000
2010
Prevalence (%)
BMI < -2SD
-2SD ≤ BMI < -1SD
-1SD ≤ BMI ≤ 1SD
1SD < BMI ≤ 2SD
BMI > 2SD

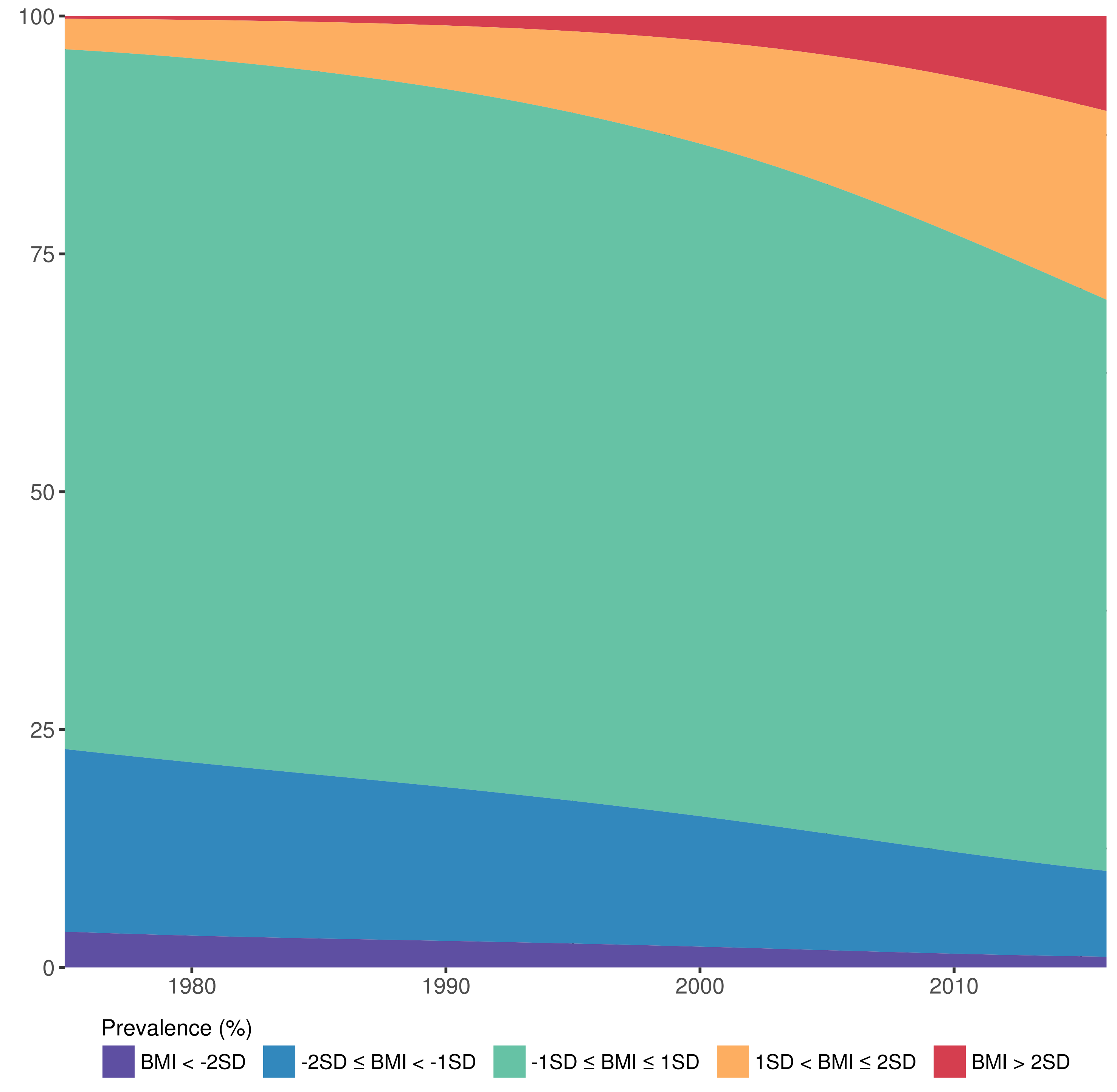
100
75
50
25
0
1980
1990
2000
2010
Prevalence (%)
BMI < -2SD
-2SD ≤ BMI < -1SD
-1SD ≤ BMI ≤ 1SD
1SD < BMI ≤ 2SD
BMI > 2SD

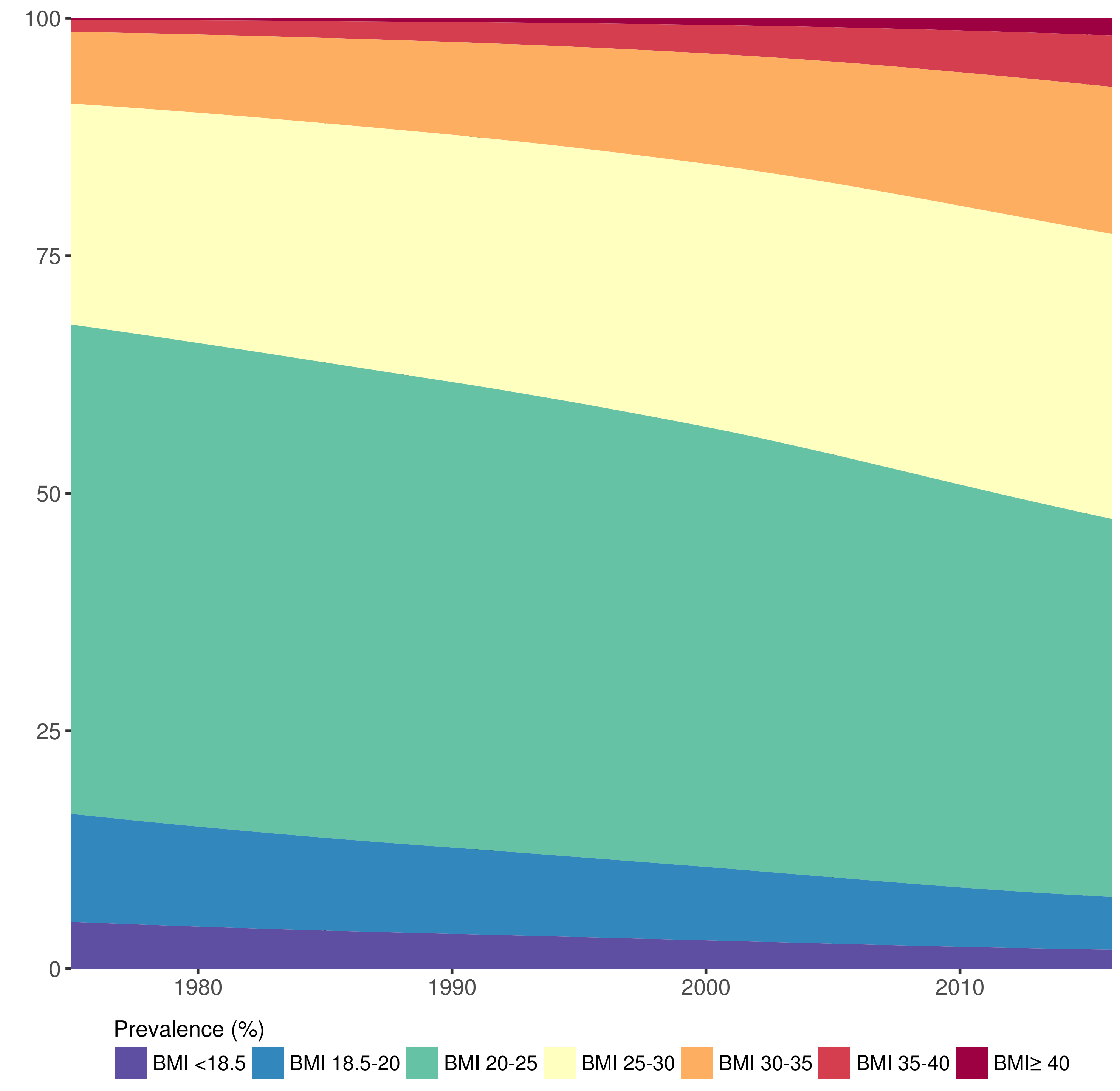
100
75
50
25
0
1980
1990
2000
2010
Prevalence (%)
BMI <18.5
BMI 18.5-20
BMI 20-25
BMI 25-30
BMI 30-35
BMI 35-40
BMI≥ 40

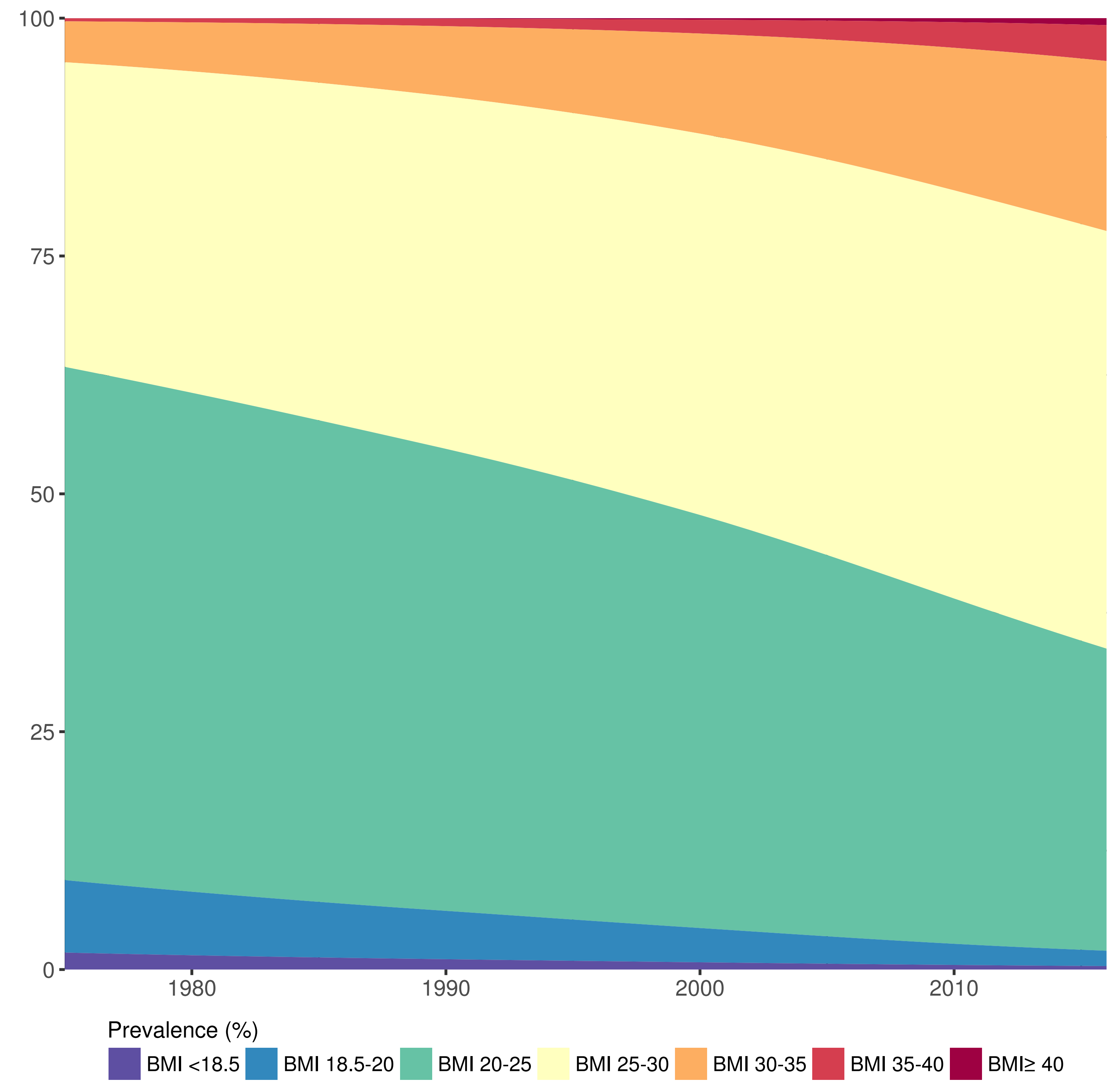
100
75
50
25
0
1980
1990
2000
2010
Prevalence (%)
BMI <18.5
BMI 18.5-20
BMI 20-25
BMI 25-30
BMI 30-35
BMI 35-40
BMI≥ 40

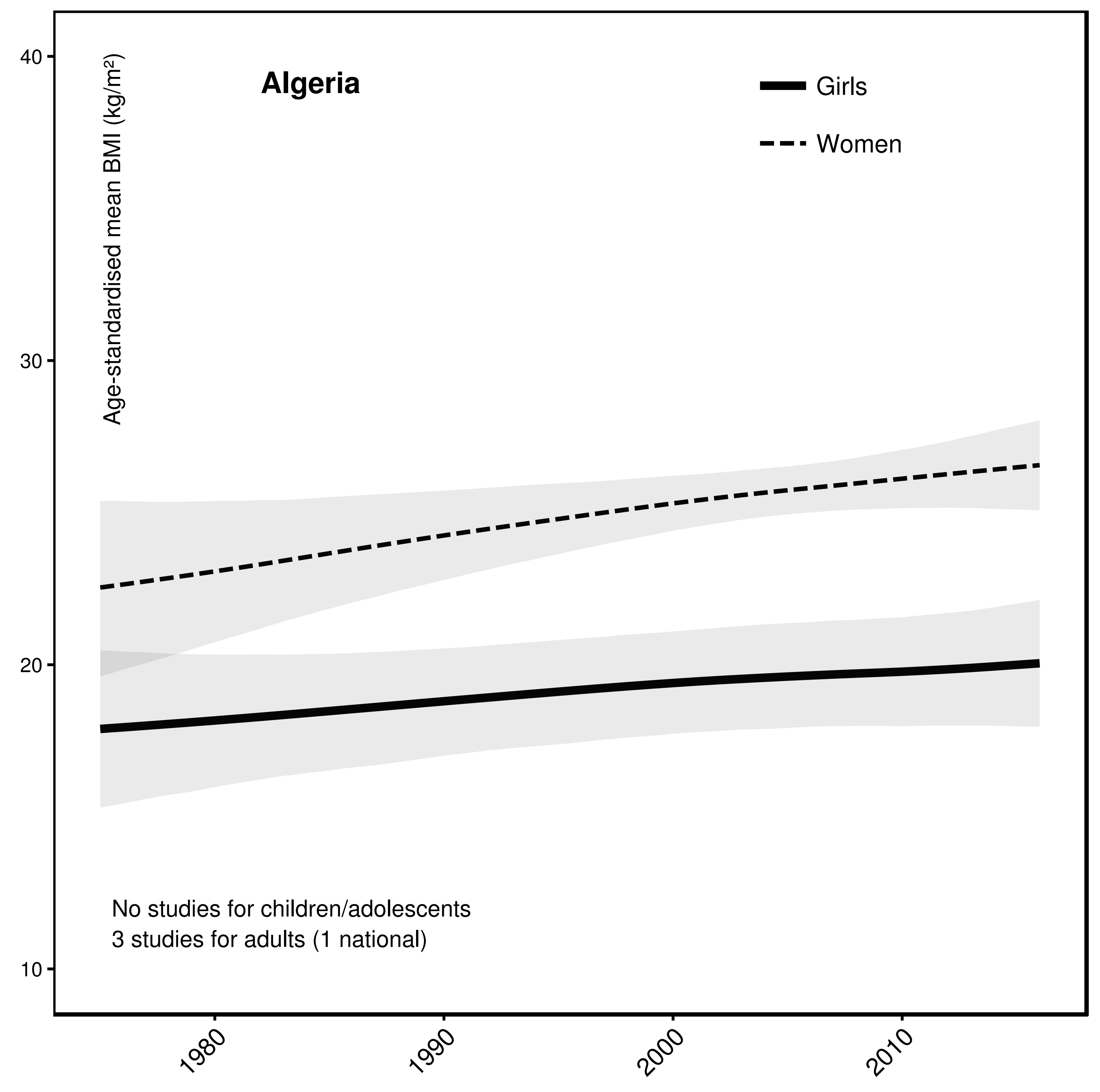
Algeria
Girls
Women
Age-standardised mean BMI (kg/m²)
40
30
20
10
No studies for children/adolescents
3 studies for adults (1 national)
1980
1990
2000
2010

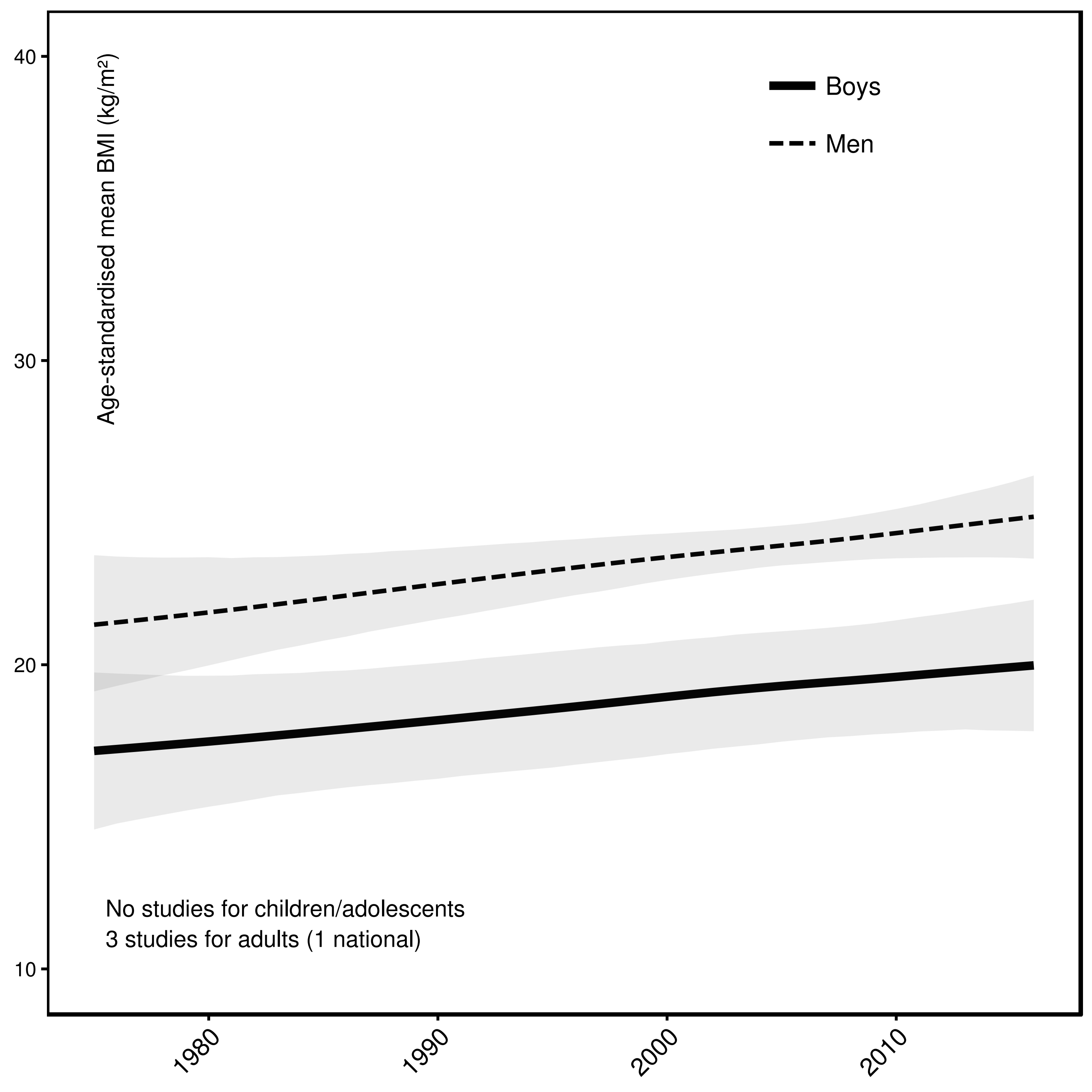
Boys
Men
Age-standardised mean BMI (kg/m²)
40
30
20
10
No studies for children/adolescents
3 studies for adults (1 national)
1980
1990
2000
2010

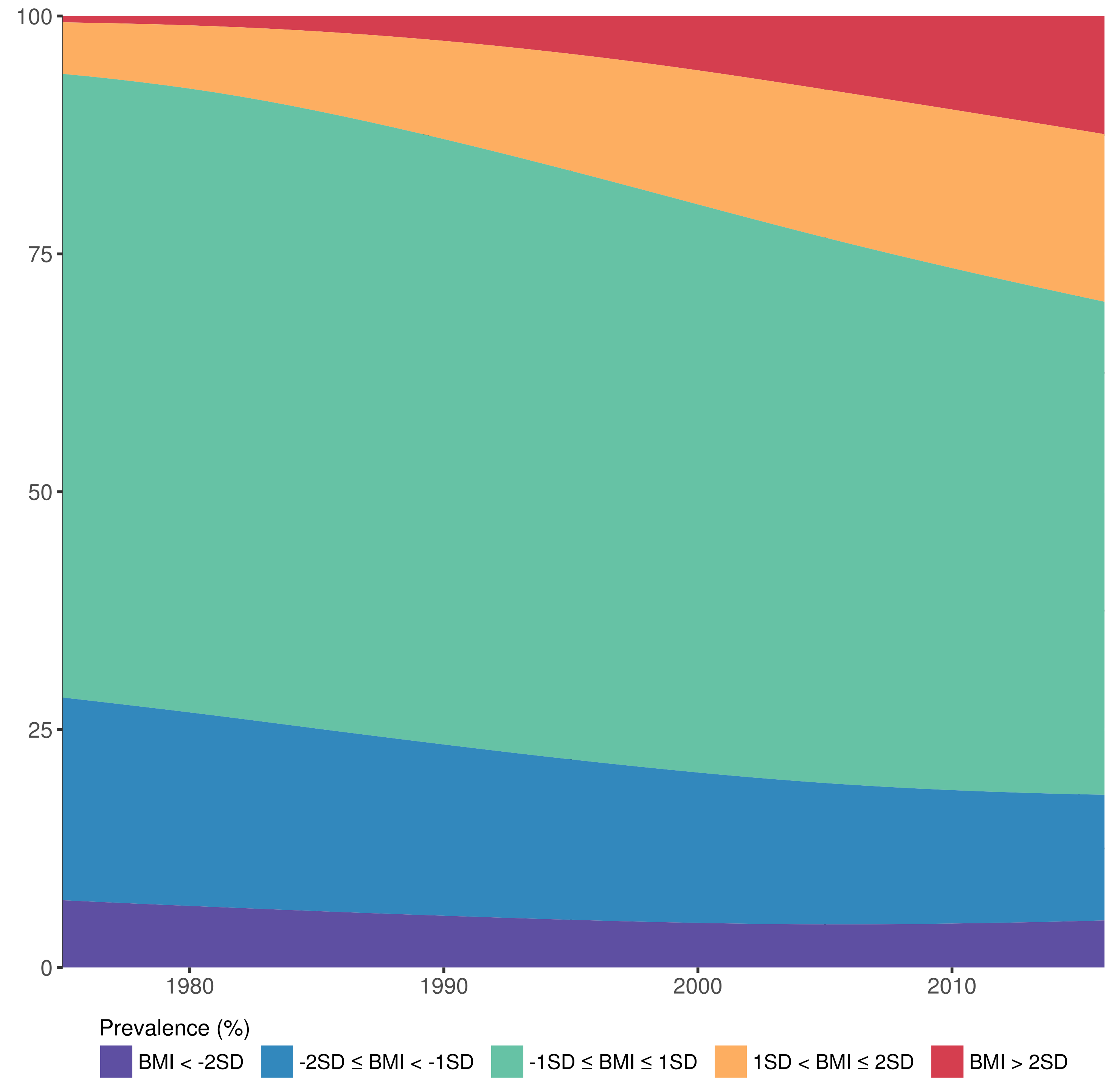
100
75
50
25
0
1980
1990
2000
2010
Prevalence (%)
BMI < -2SD
-2SD ≤ BMI < -1SD
-1SD ≤ BMI ≤ 1SD
1SD < BMI ≤ 2SD
BMI > 2SD

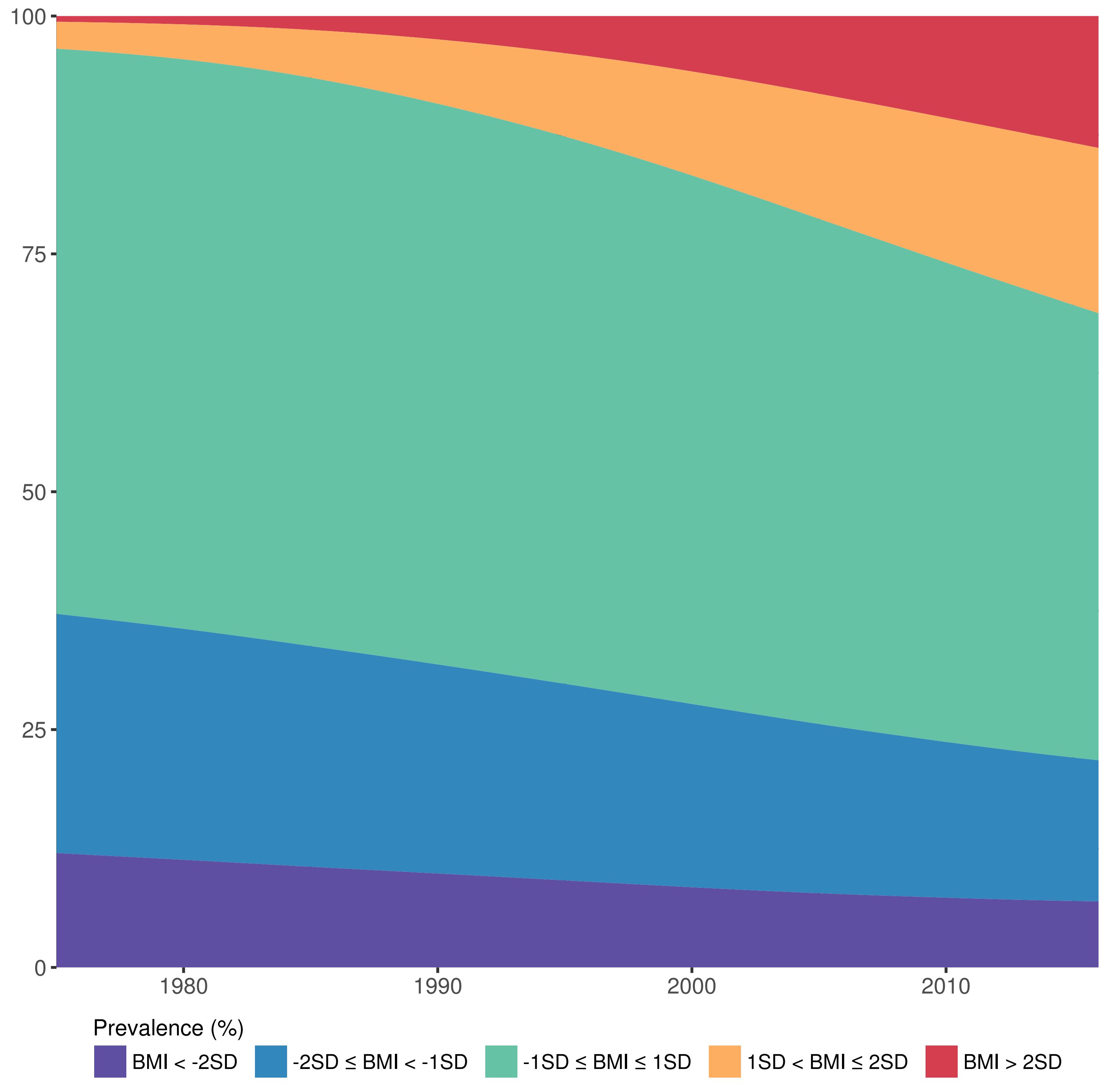
100
75
50
25
0
1980
1990
2000
2010
Prevalence (%)
BMI < -2SD
-2SD ≤ BMI < -1SD
-1SD ≤ BMI ≤ 1SD
1SD < BMI ≤ 2SD
BMI > 2SD

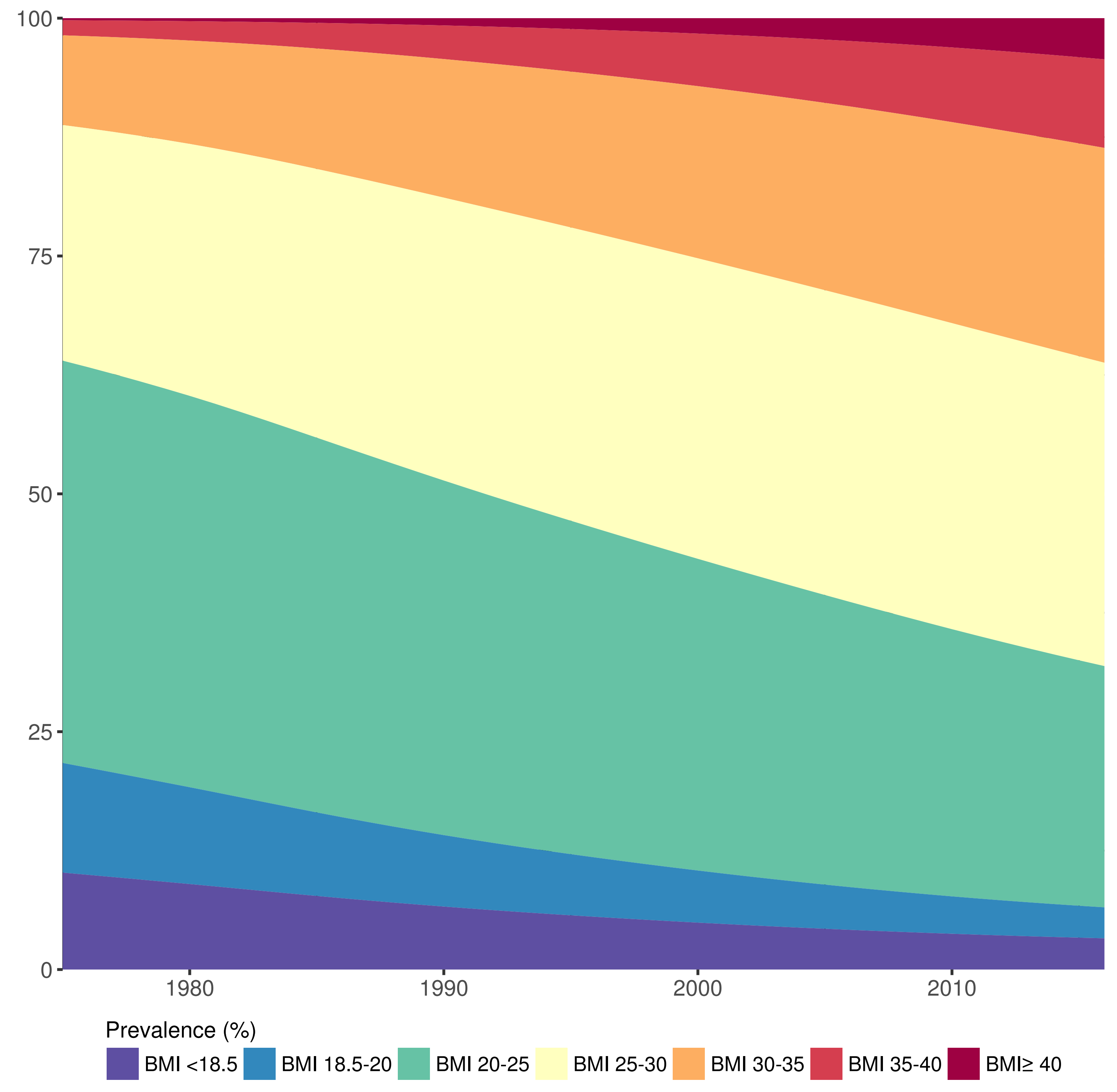
100
75
50
25
0
1980
1990
2000
2010
Prevalence (%)
BMI <18.5
BMI 18.5-20
BMI 20-25
BMI 25-30
BMI 30-35
BMI 35-40
BMI≥ 40

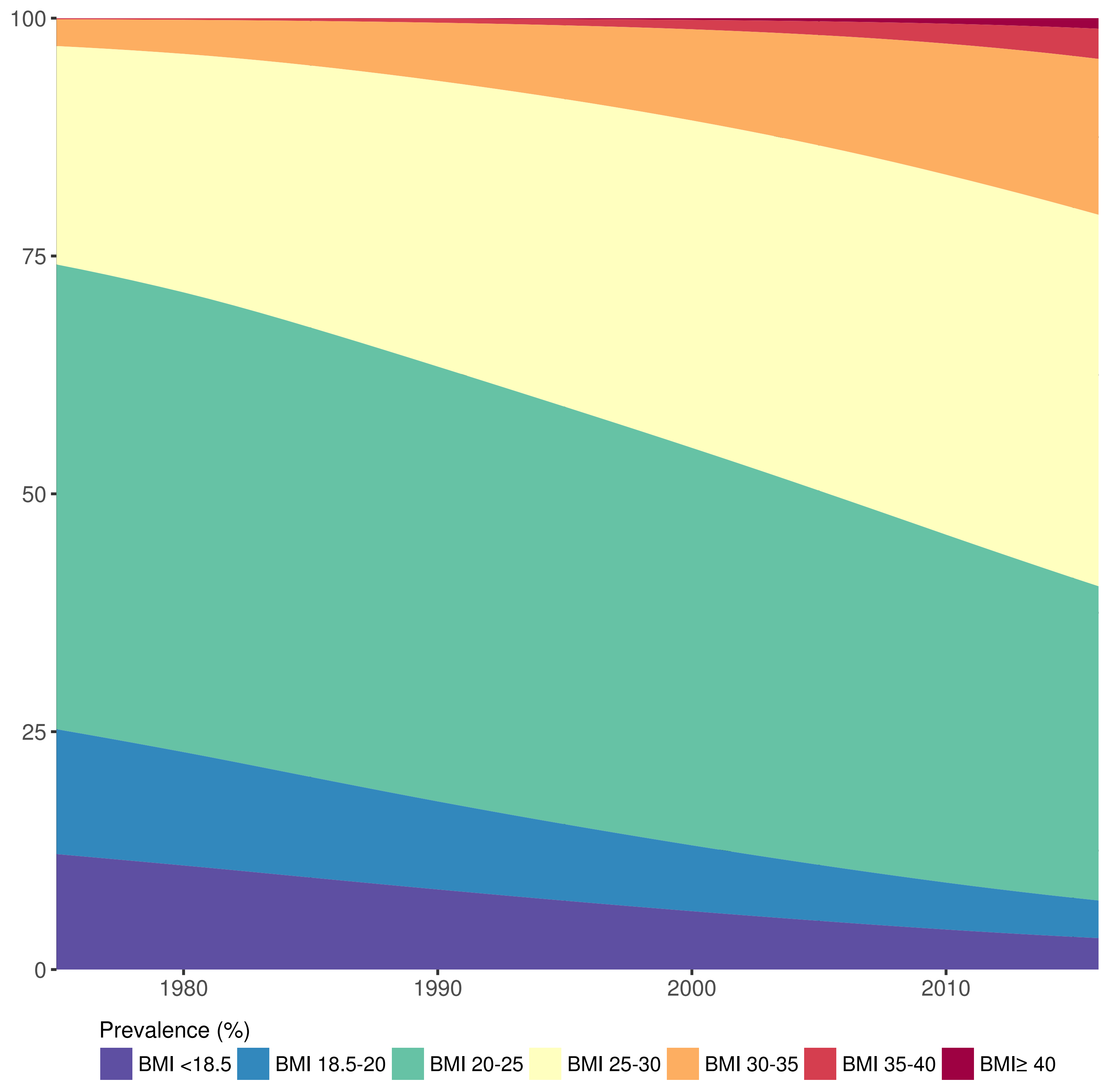
100
75
50
25
0
1980
1990
2000
2010
Prevalence (%)
BMI <18.5
BMI 18.5-20
BMI 20-25
BMI 25-30
BMI 30-35
BMI 35-40
BMI≥ 40

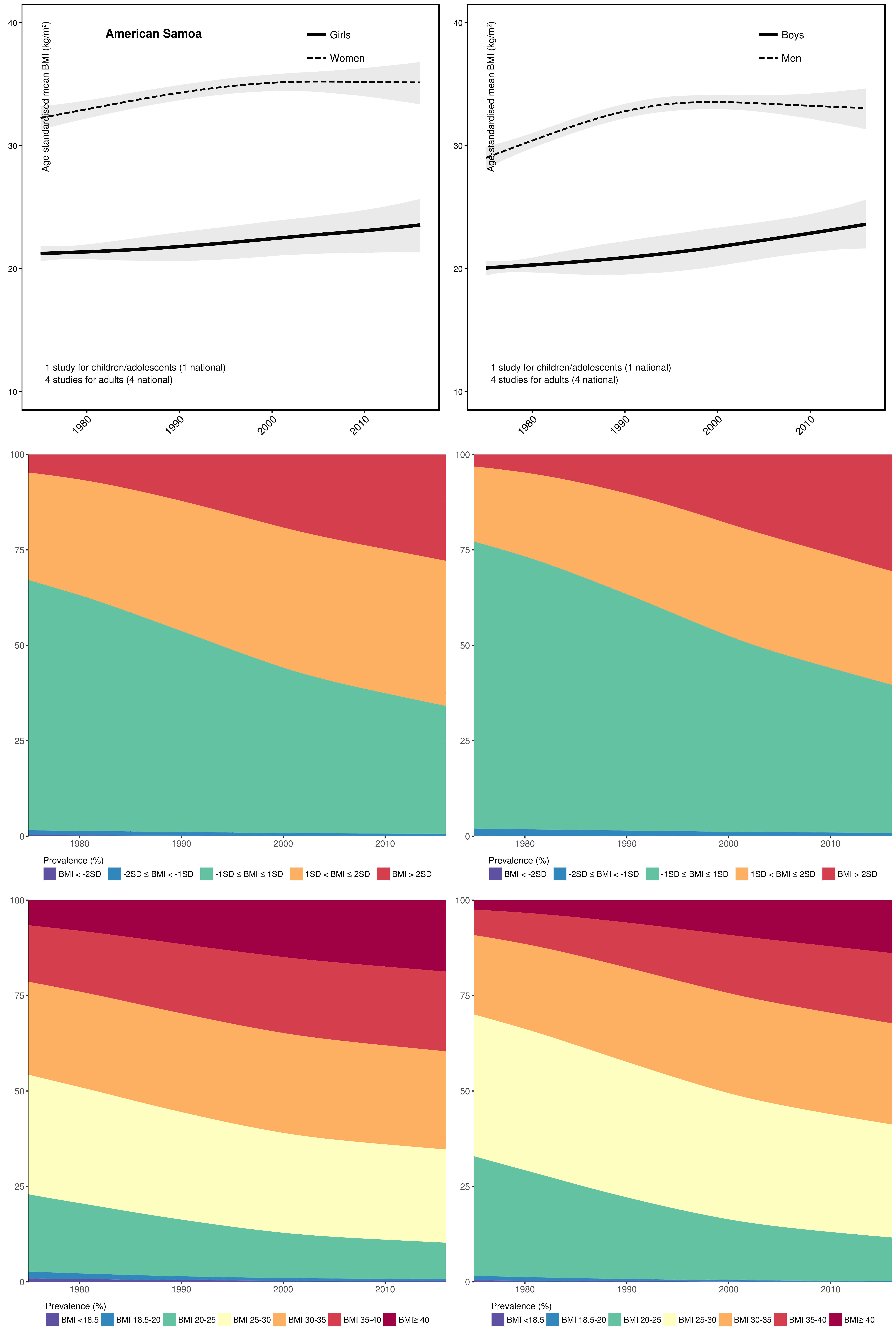
American Samoa
Girls
Women
Boys
Men
Age-standardised mean BMI (kg/m²)
1 study for children/adolescents (1 national)
4 studies for adults (4 national)
1980
1990
2000
2010
Prevalence (%)
BMI < -2SD
-2SD ≤ BMI < -1SD
-1SD ≤ BMI ≤ 1SD
1SD < BMI ≤ 2SD
BMI > 2SD
BMI <18.5
BMI 18.5-20
BMI 20-25
BMI 25-30
BMI 30-35
BMI 35-40
BMI≥ 40

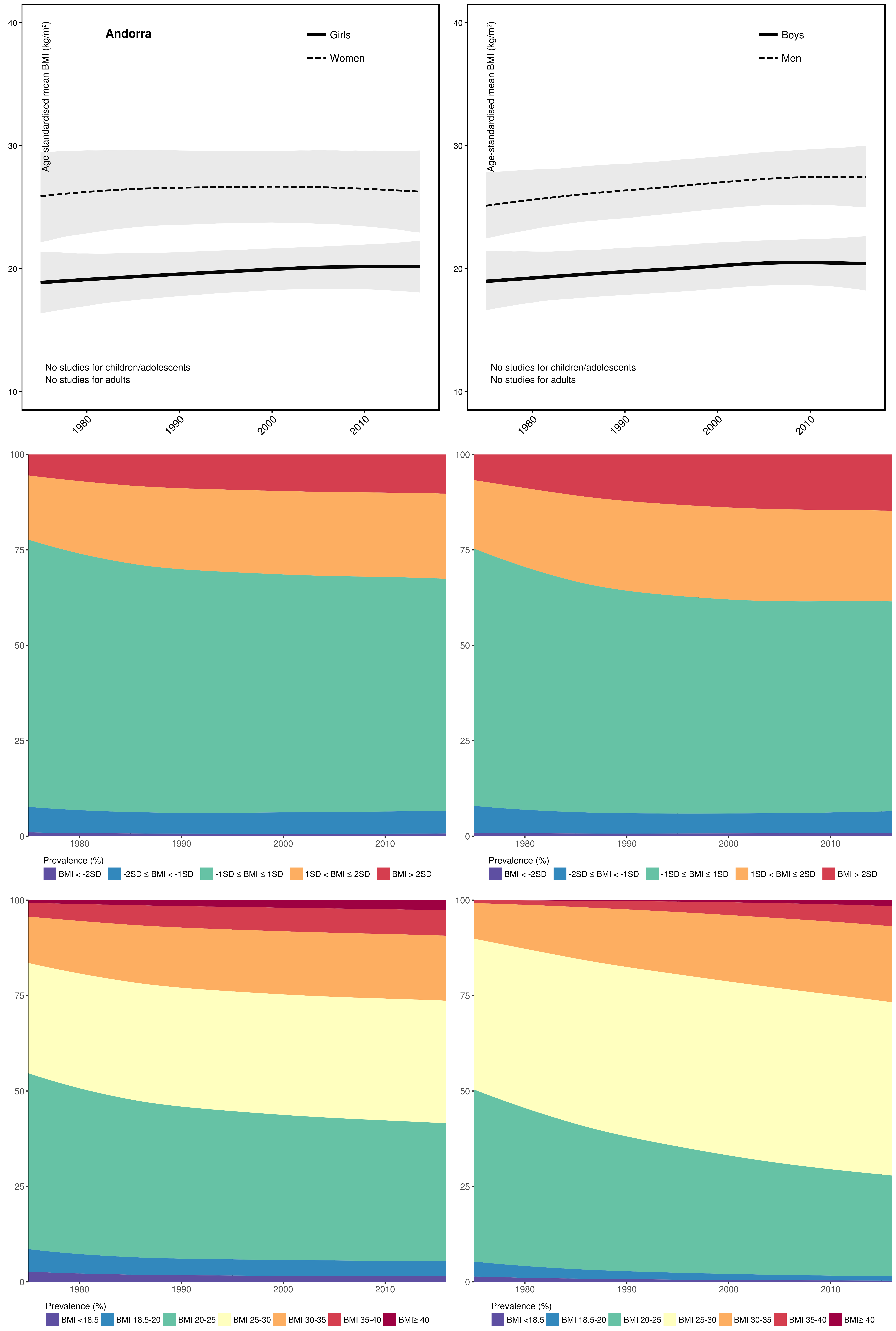
Andorra
Girls
Women
Boys
Men
Age-standardised mean BMI (kg/m²)
No studies for children/adolescents
No studies for adults
Prevalence (%)
BMI < -2SD
-2SD ≤ BMI < -1SD
-1SD ≤ BMI ≤ 1SD
1SD < BMI ≤ 2SD
BMI > 2SD
BMI <18.5
BMI 18.5-20
BMI 20-25
BMI 25-30
BMI 30-35
BMI 35-40
BMI≥ 40

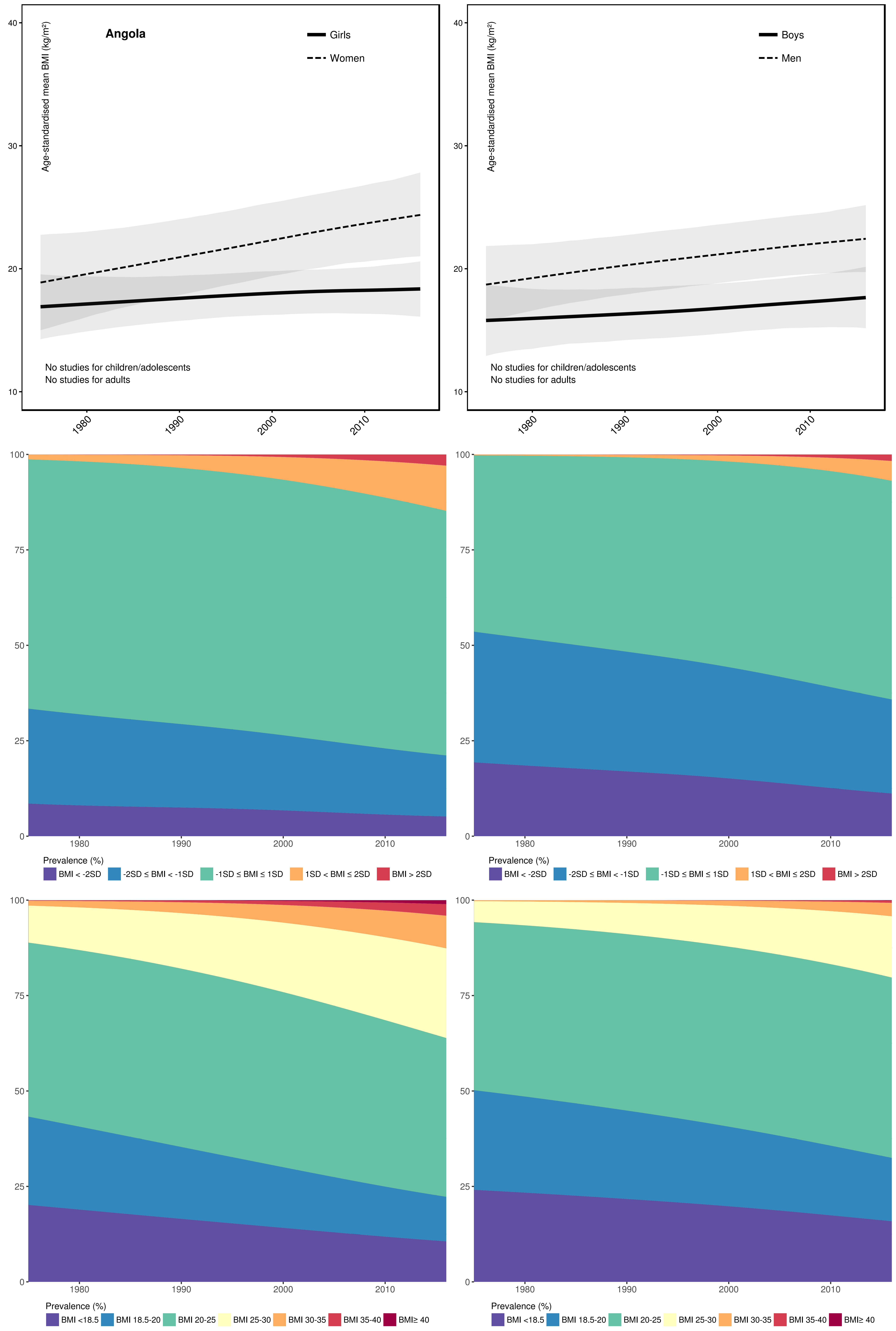
Angola
Girls
Women
Boys
Men
Age-standardised mean BMI (kg/m²)
No studies for children/adolescents
No studies for adults
40
30
20
10
1980
1990
2000
2010
100
75
50
25
0
Prevalence (%)
BMI < -2SD
-2SD ≤ BMI < -1SD
-1SD ≤ BMI ≤ 1SD
1SD < BMI ≤ 2SD
BMI > 2SD
BMI <18.5
BMI 18.5-20
BMI 20-25
BMI 25-30
BMI 30-35
BMI 35-40
BMI≥ 40

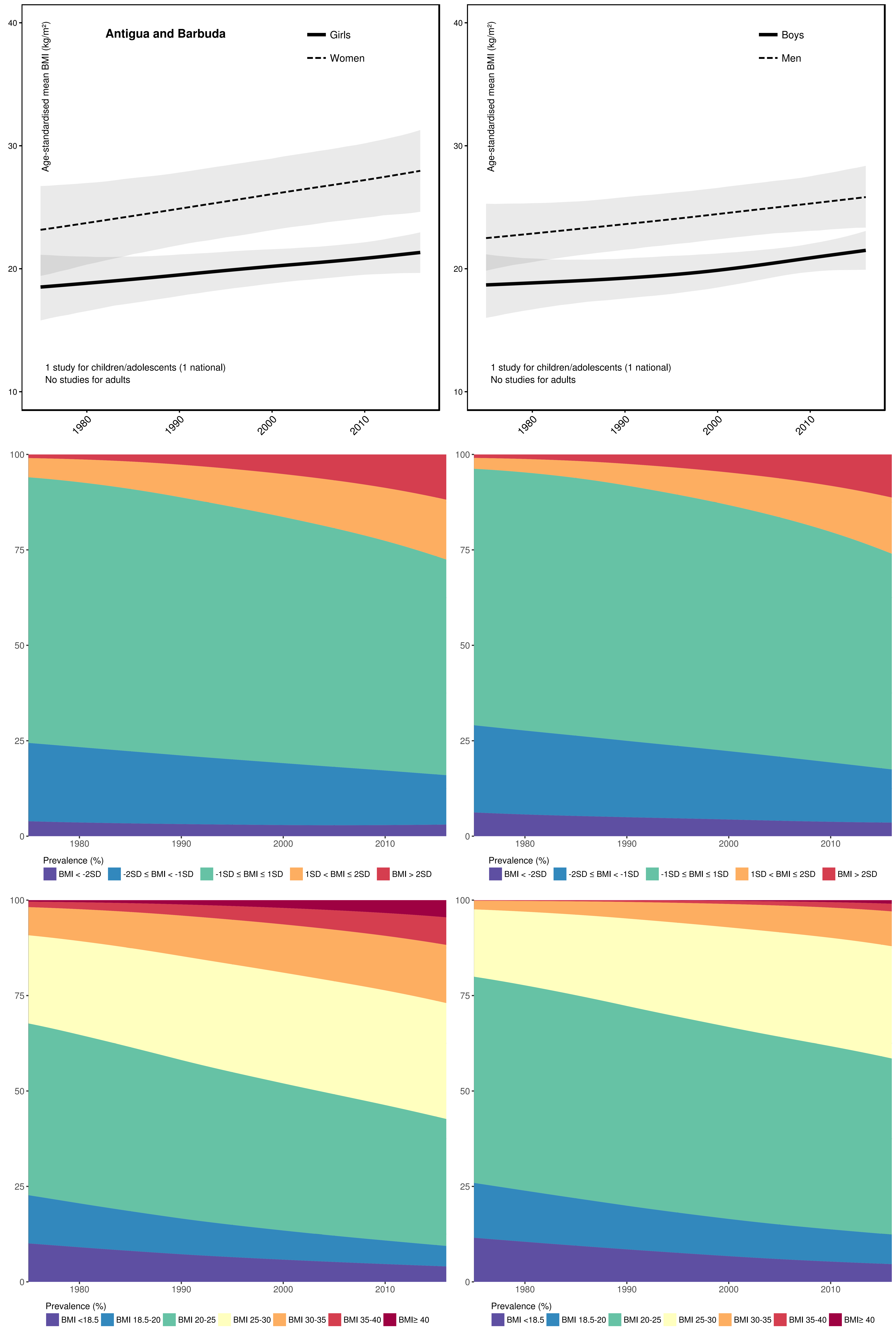
Antigua and Barbuda
Girls
Women
Boys
Men
Age-standardised mean BMI (kg/m²)
1 study for children/adolescents (1 national)
No studies for adults
1980
1990
2000
2010
10
20
30
40
0
25
50
75
100
Prevalence (%)
BMI < -2SD
-2SD ≤ BMI < -1SD
-1SD ≤ BMI ≤ 1SD
1SD < BMI ≤ 2SD
BMI > 2SD
BMI <18.5
BMI 18.5-20
BMI 20-25
BMI 25-30
BMI 30-35
BMI 35-40
BMI≥ 40

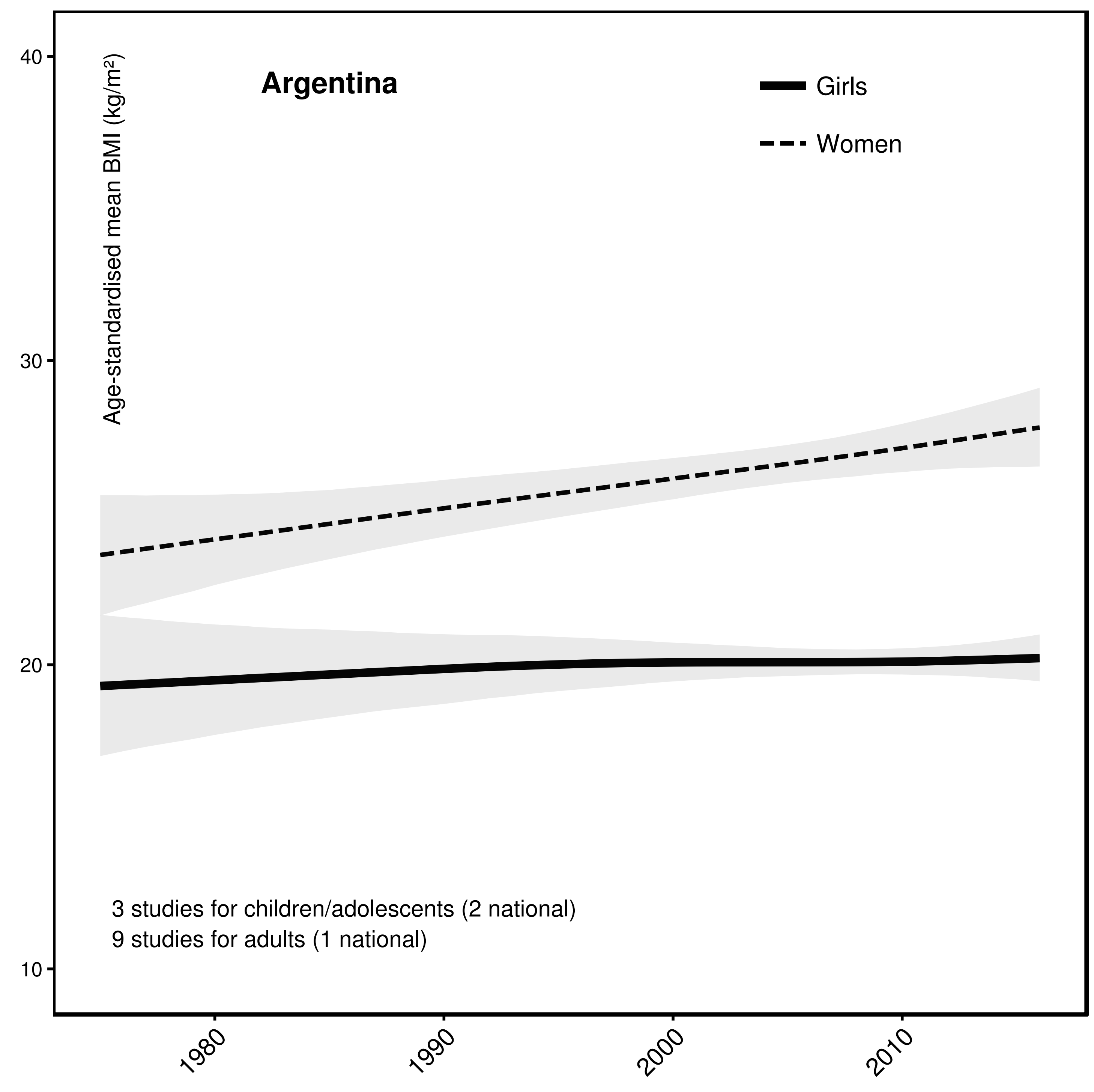
Argentina
Girls
Women
Age-standardised mean BMI (kg/m²)
40
30
20
10
3 studies for children/adolescents (2 national)
9 studies for adults (1 national)
1980
1990
2000
2010

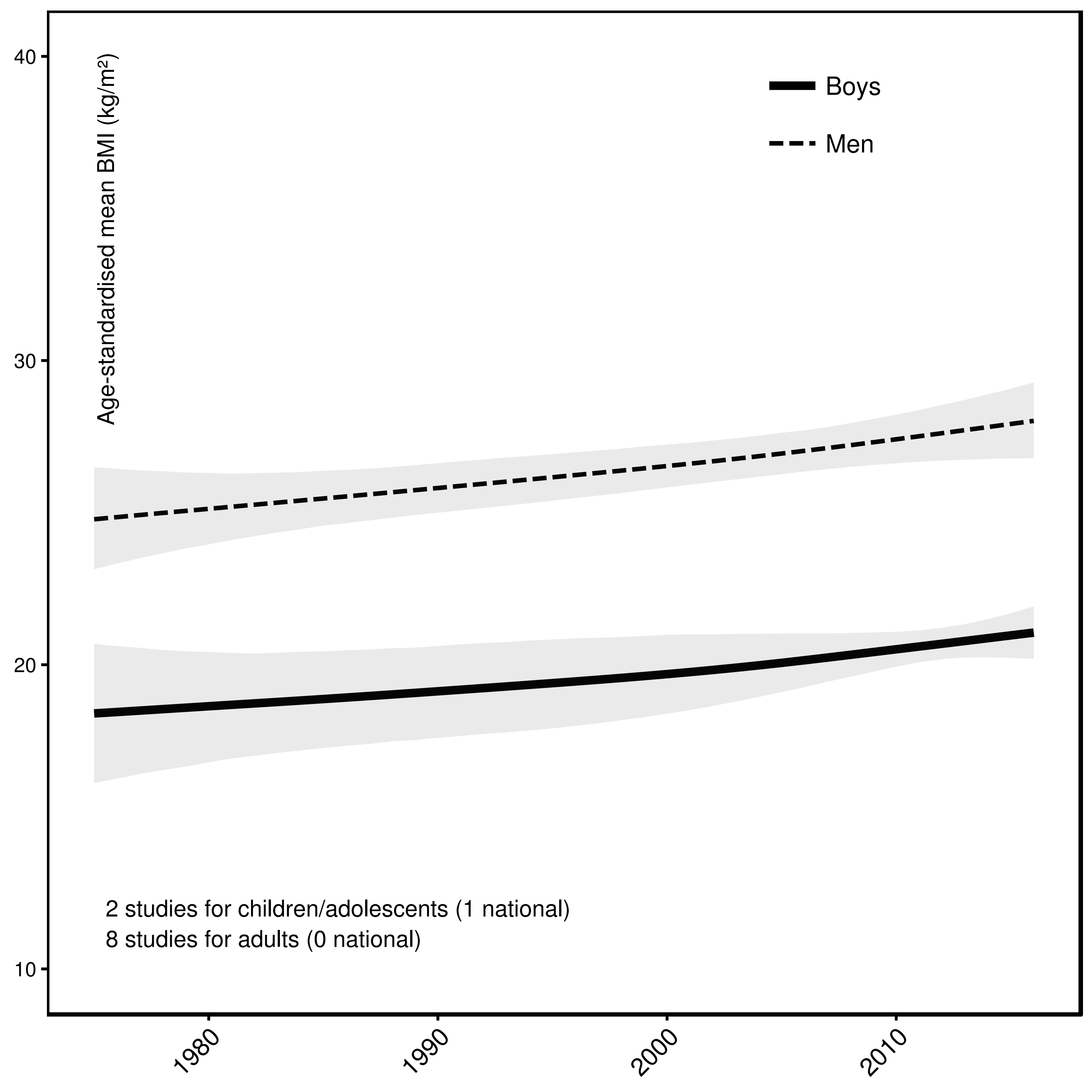
Boys
Men
Age-standardised mean BMI (kg/m²)
40
30
20
10
2 studies for children/adolescents (1 national)
8 studies for adults (0 national)
1980
1990
2000
2010

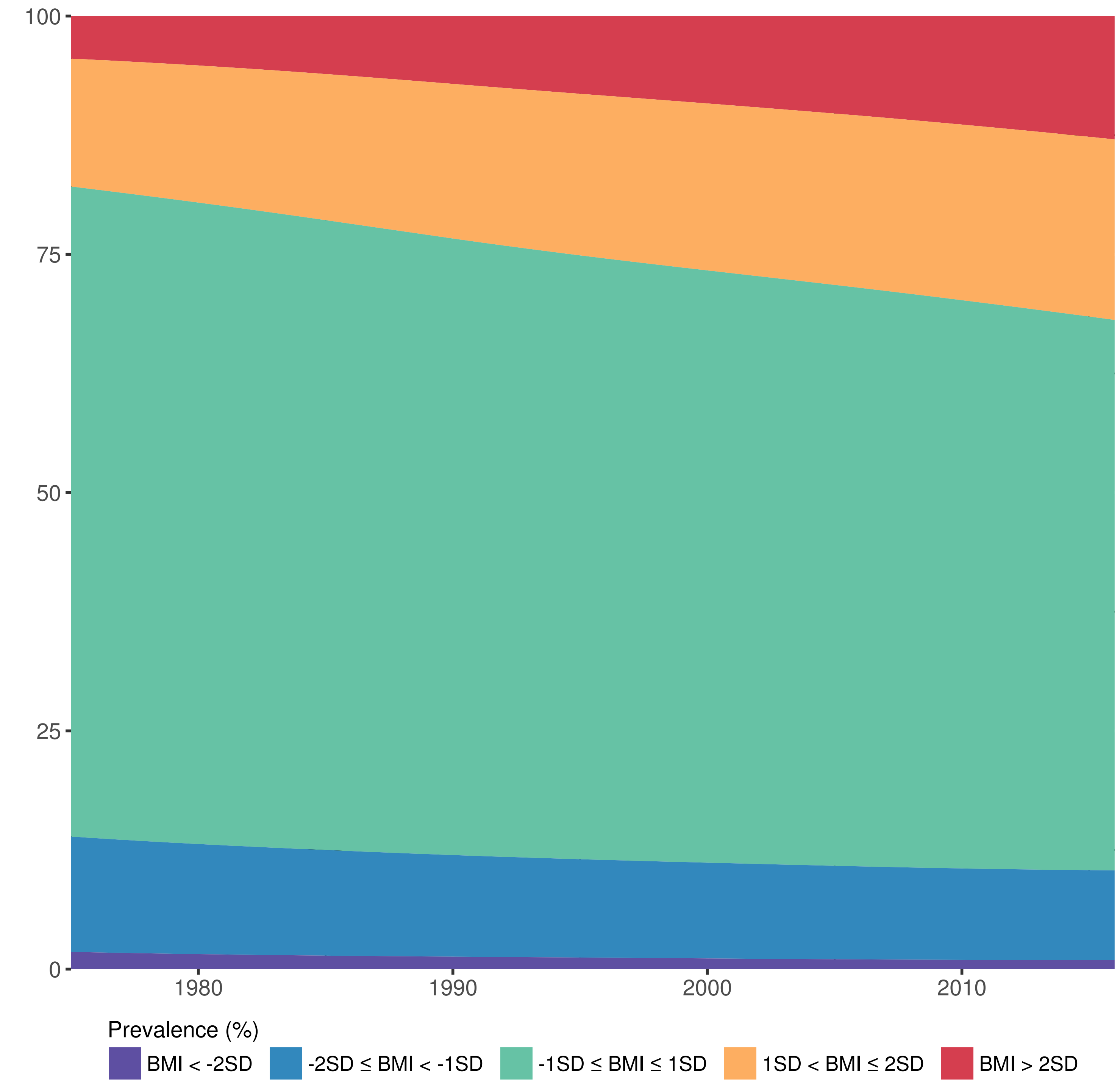
100
75
50
25
0
1980
1990
2000
2010
Prevalence (%)
BMI < -2SD
-2SD ≤ BMI < -1SD
-1SD ≤ BMI ≤ 1SD
1SD < BMI ≤ 2SD
BMI > 2SD

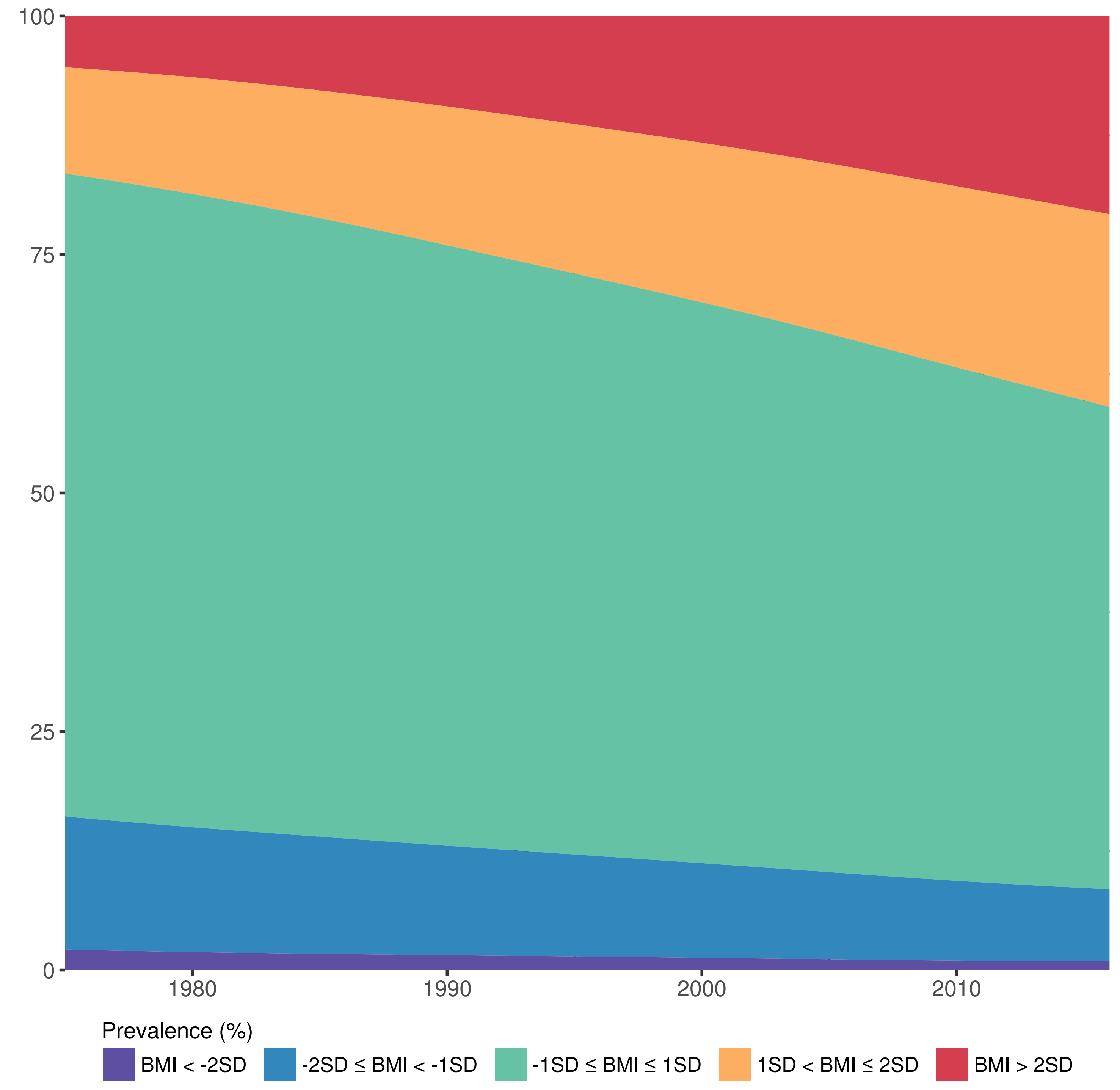
100
75
50
25
0
1980
1990
2000
2010
Prevalence (%)
BMI < -2SD
-2SD ≤ BMI < -1SD
-1SD ≤ BMI ≤ 1SD
1SD < BMI ≤ 2SD
BMI > 2SD

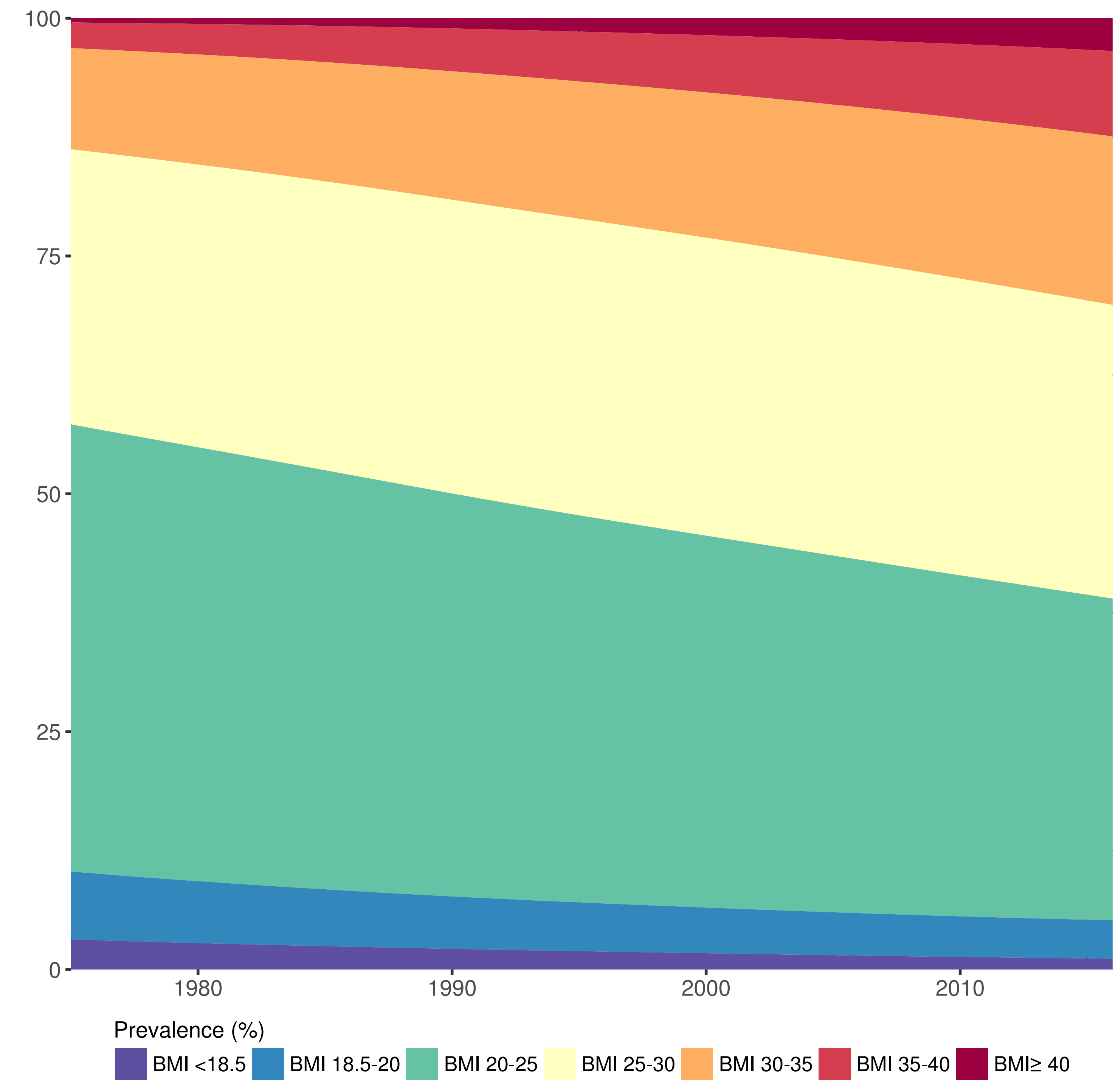
100
75
50
25
0
1980
1990
2000
2010
Prevalence (%)
BMI <18.5
BMI 18.5-20
BMI 20-25
BMI 25-30
BMI 30-35
BMI 35-40
BMI≥ 40

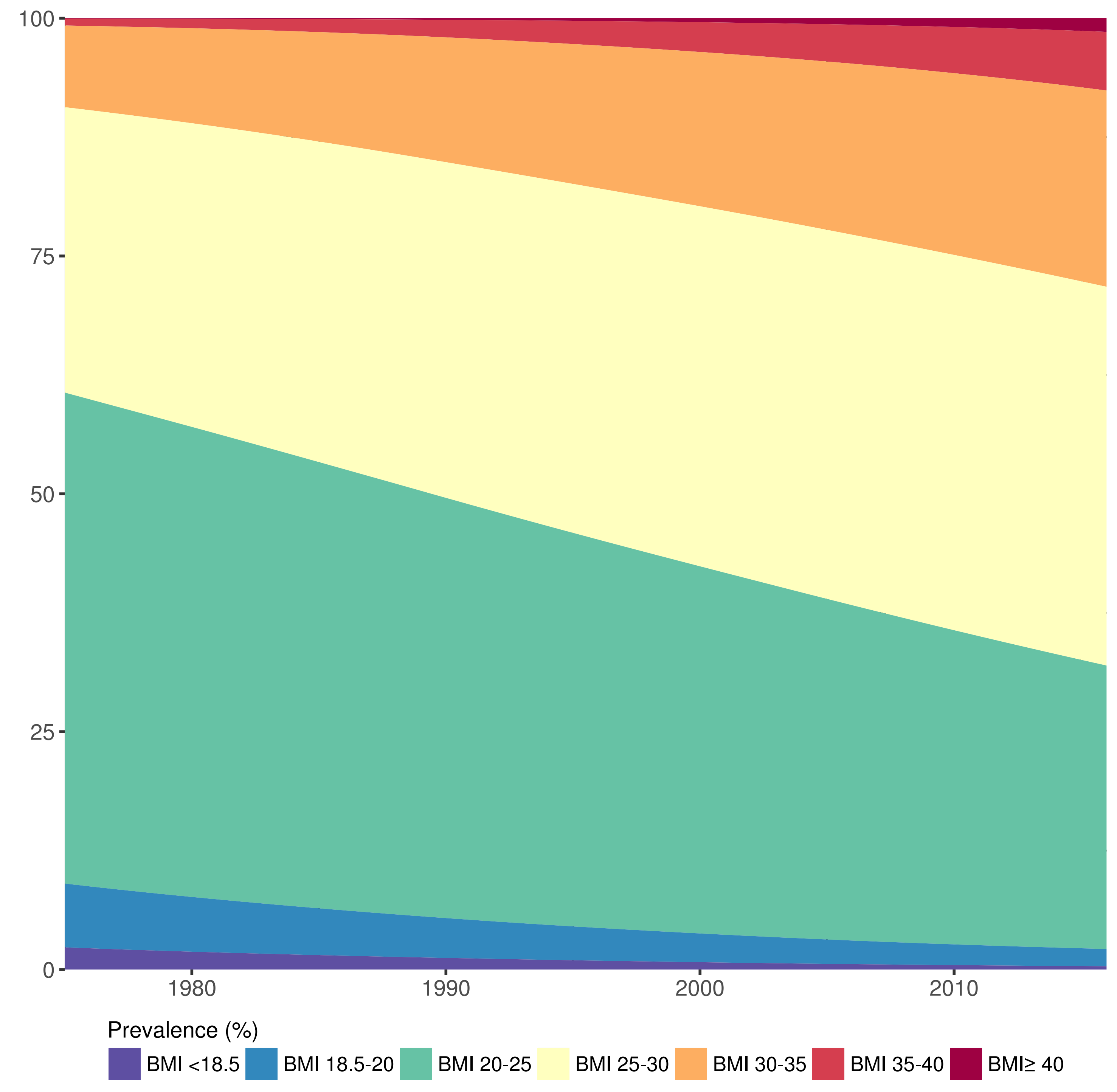
100
75
50
25
0
1980
1990
2000
2010
Prevalence (%)
BMI <18.5
BMI 18.5-20
BMI 20-25
BMI 25-30
BMI 30-35
BMI 35-40
BMI≥ 40

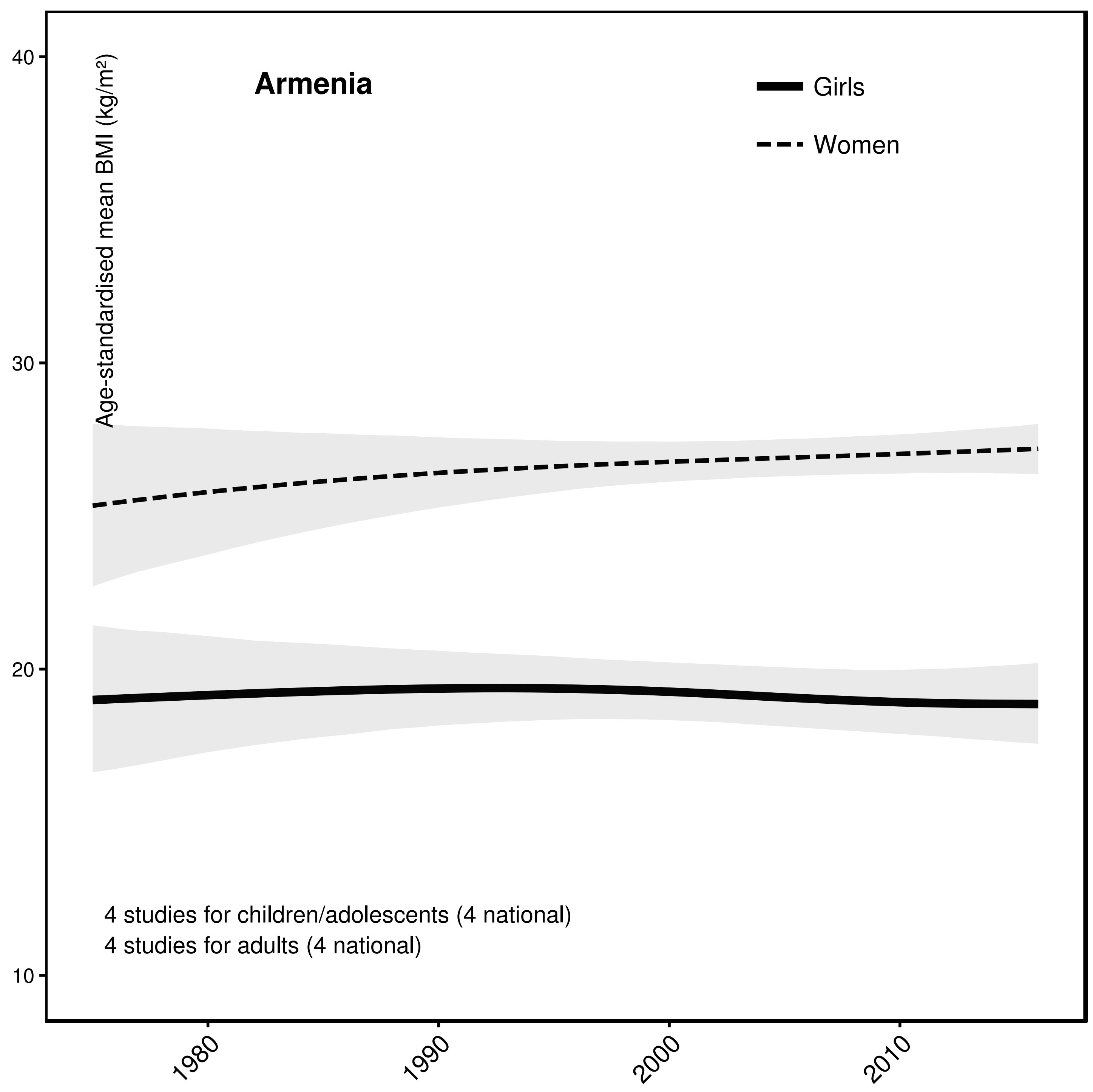
Armenia
Girls
Women
Age-standardised mean BMI (kg/m²)
40
30
20
10
4 studies for children/adolescents (4 national)
4 studies for adults (4 national)
1980
1990
2000
2010

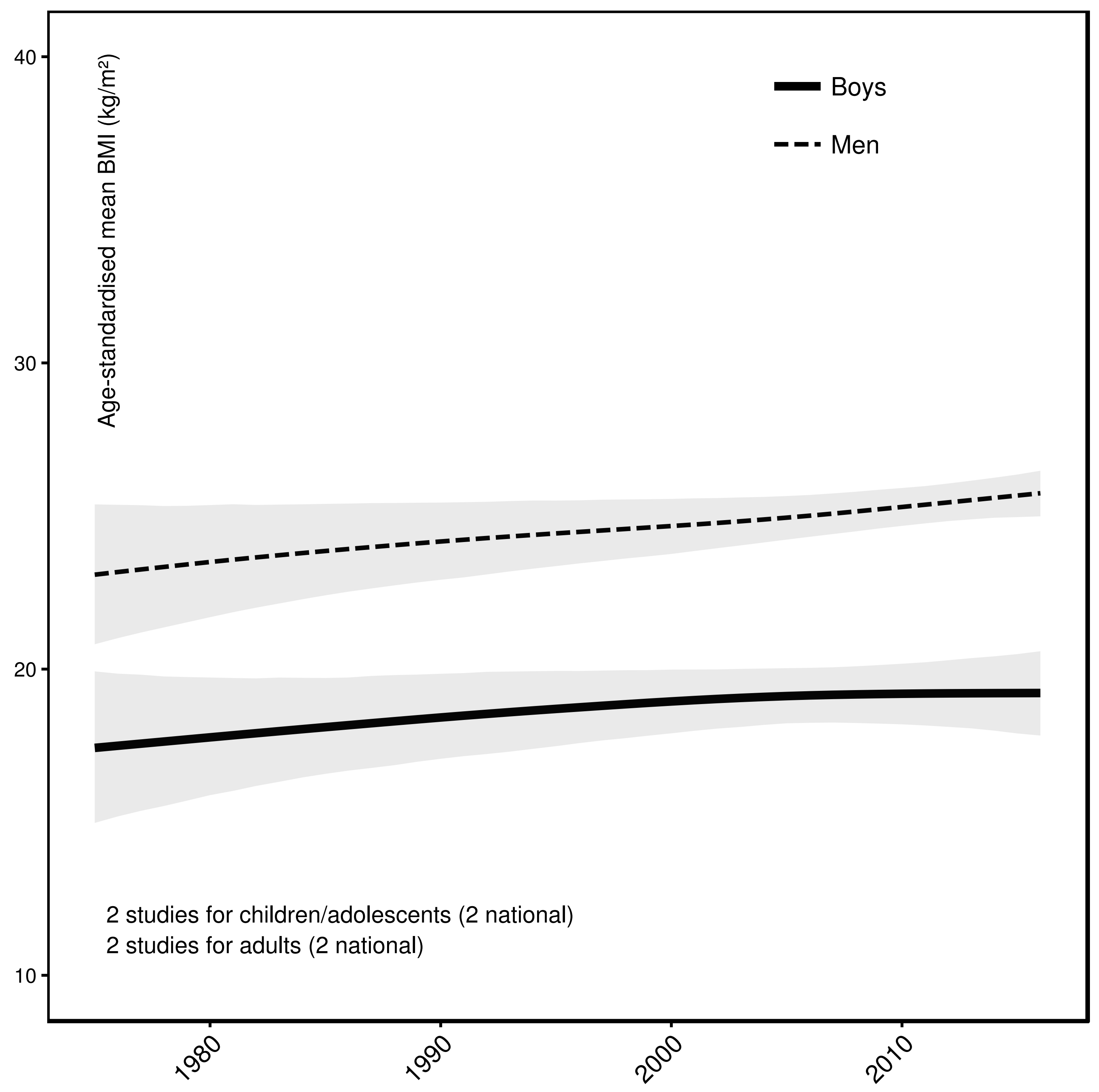
Boys
Men
Age-standardised mean BMI (kg/m²)
40
30
20
10
2 studies for children/adolescents (2 national)
2 studies for adults (2 national)
1980
1990
2000
2010

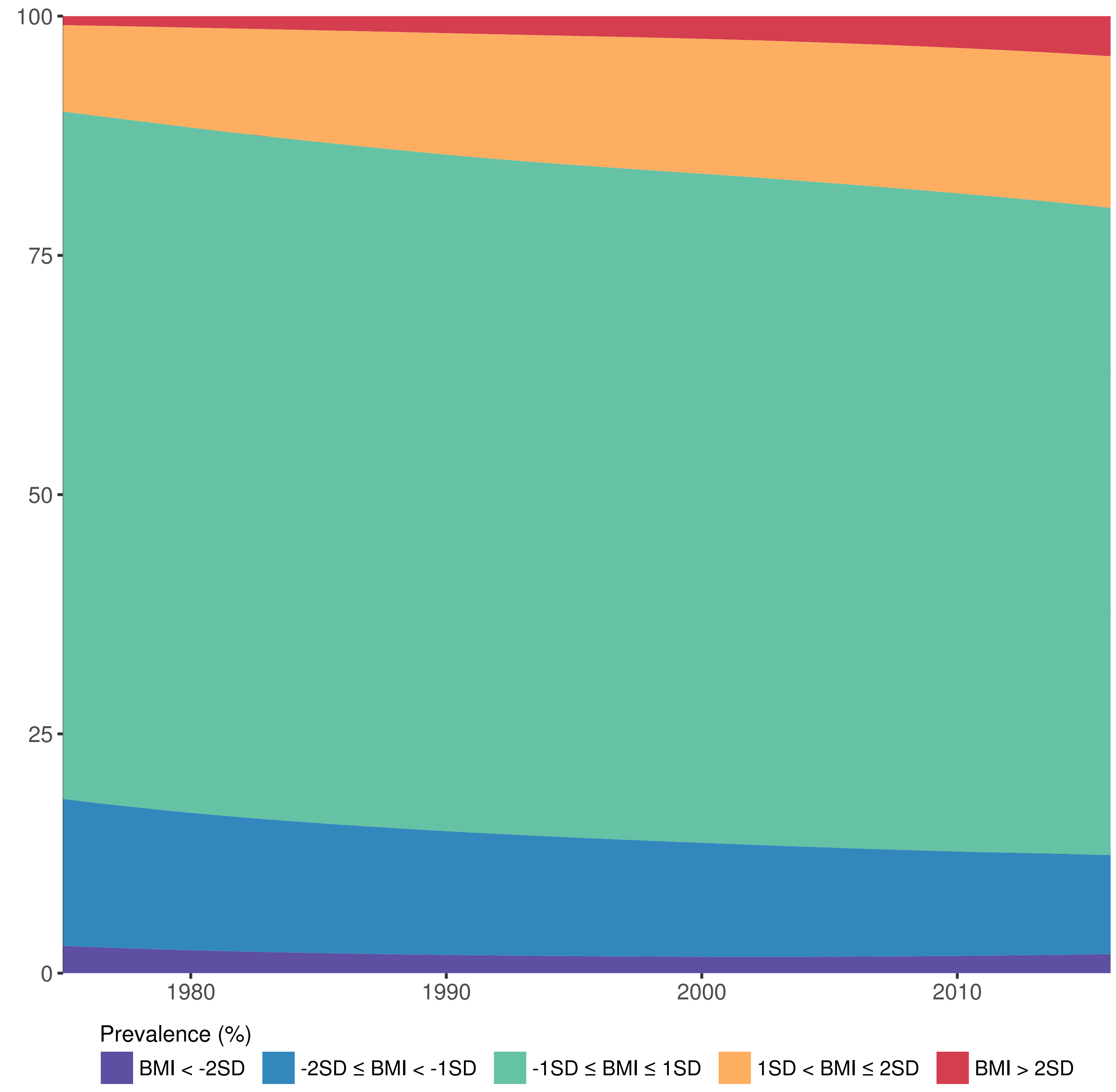
100
75
50
25
0
1980
1990
2000
2010
Prevalence (%)
BMI < -2SD
-2SD ≤ BMI < -1SD
-1SD ≤ BMI ≤ 1SD
1SD < BMI ≤ 2SD
BMI > 2SD

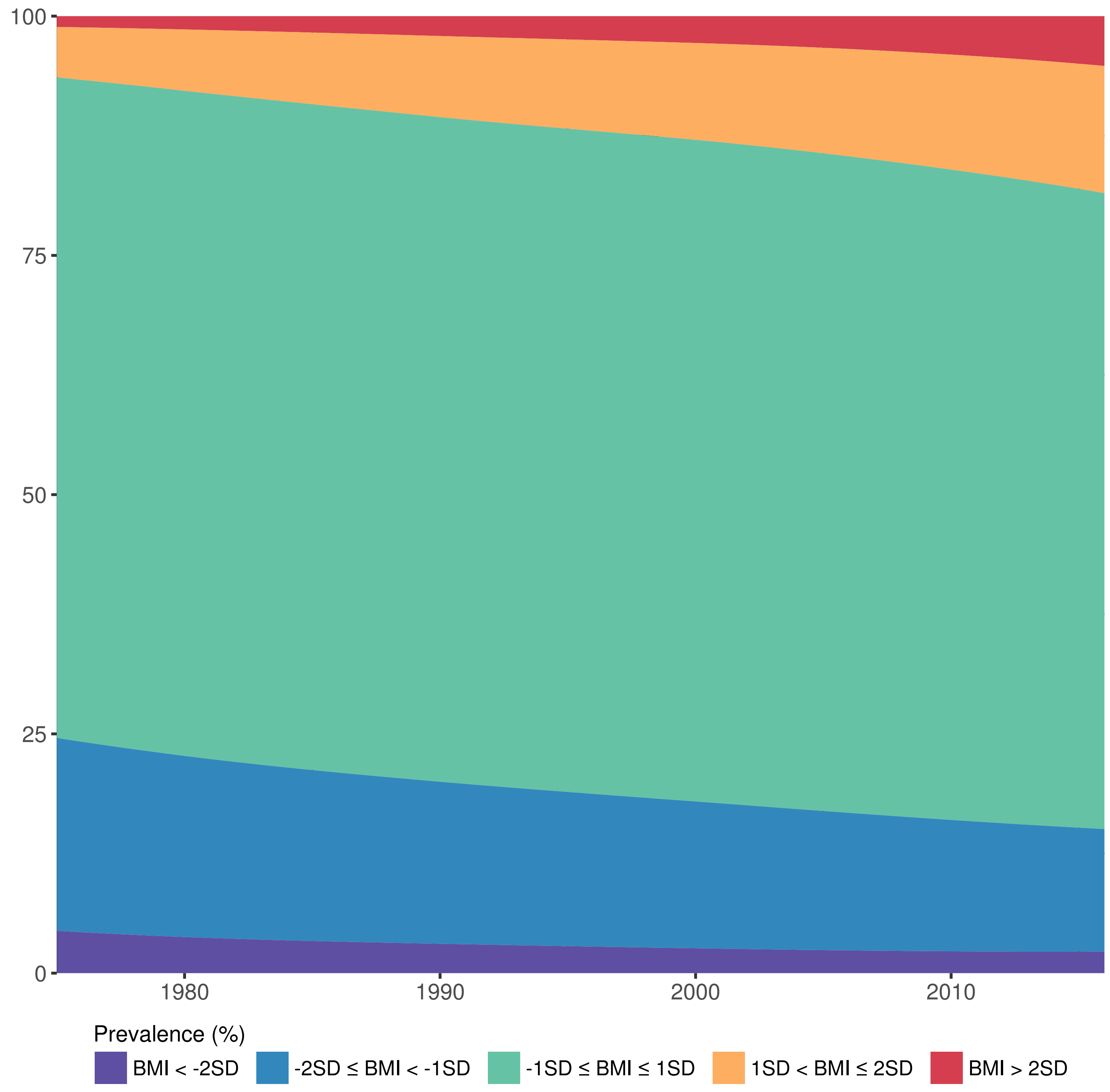
100
75
50
25
0
1980
1990
2000
2010
Prevalence (%)
BMI < -2SD
-2SD ≤ BMI < -1SD
-1SD ≤ BMI ≤ 1SD
1SD < BMI ≤ 2SD
BMI > 2SD

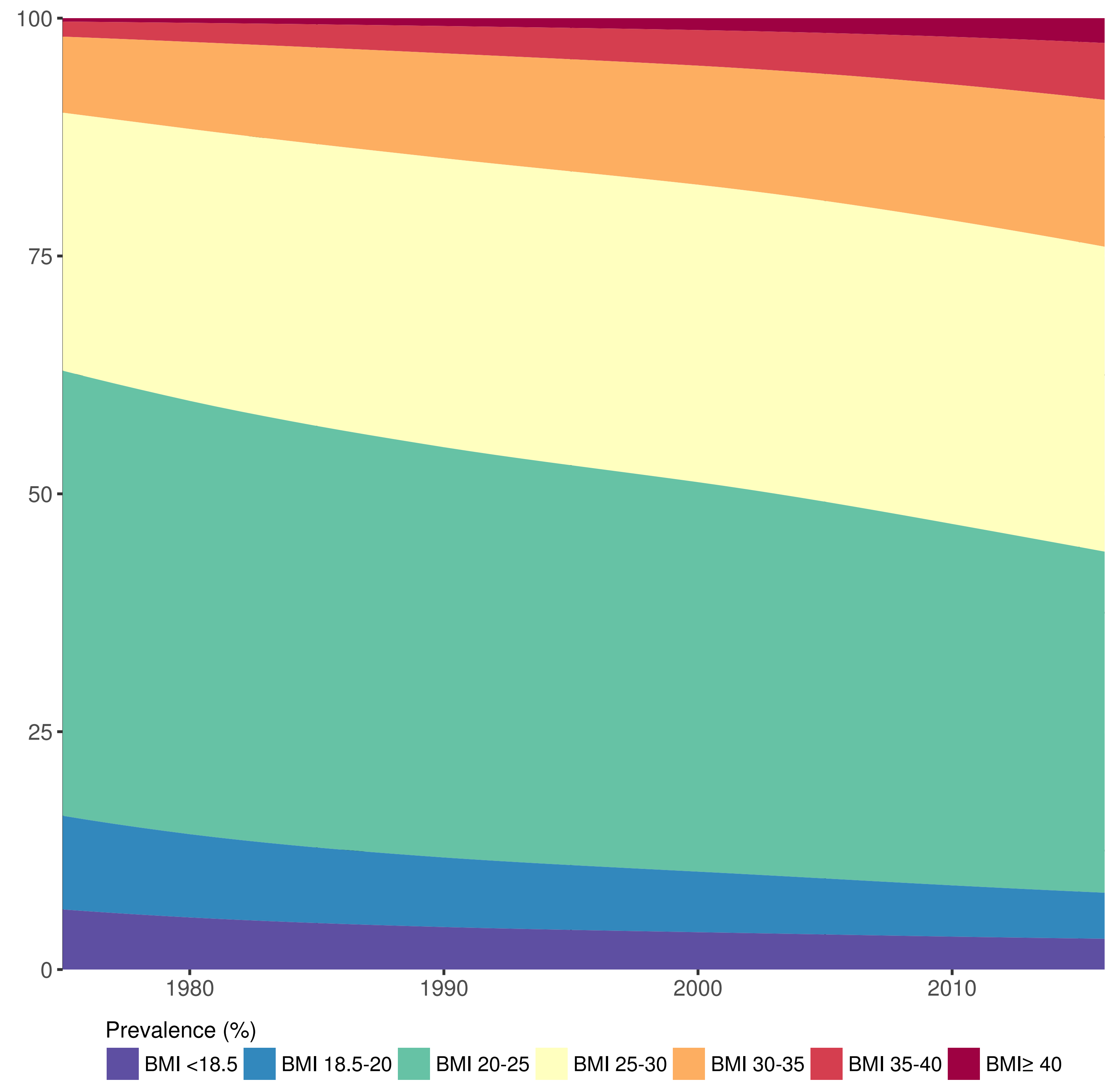
100
75
50
25
0
1980
1990
2000
2010
Prevalence (%)
BMI <18.5
BMI 18.5-20
BMI 20-25
BMI 25-30
BMI 30-35
BMI 35-40
BMI≥ 40

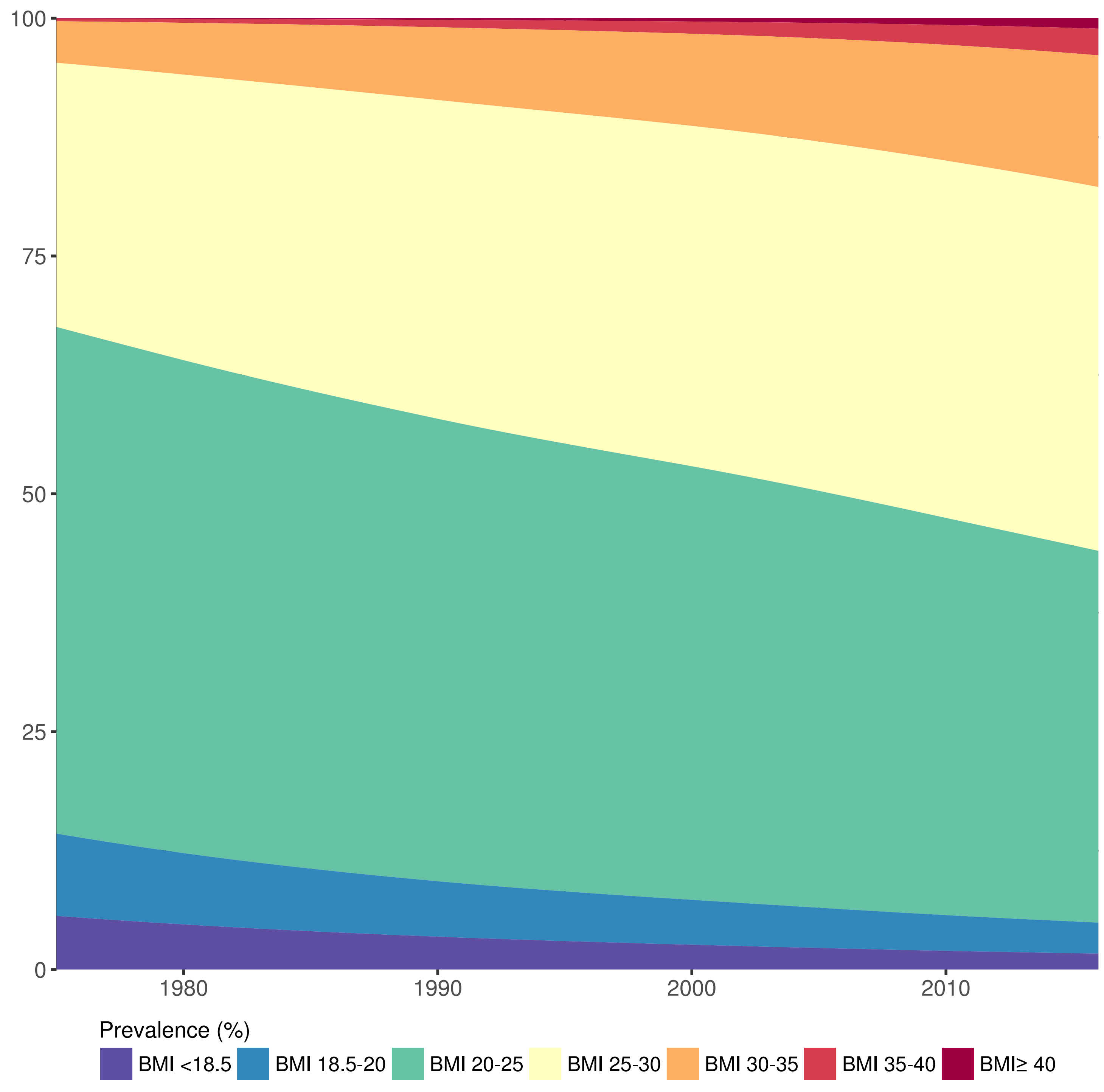
100
75
50
25
0
1980
1990
2000
2010
Prevalence (%)
BMI <18.5
BMI 18.5-20
BMI 20-25
BMI 25-30
BMI 30-35
BMI 35-40
BMI≥ 40

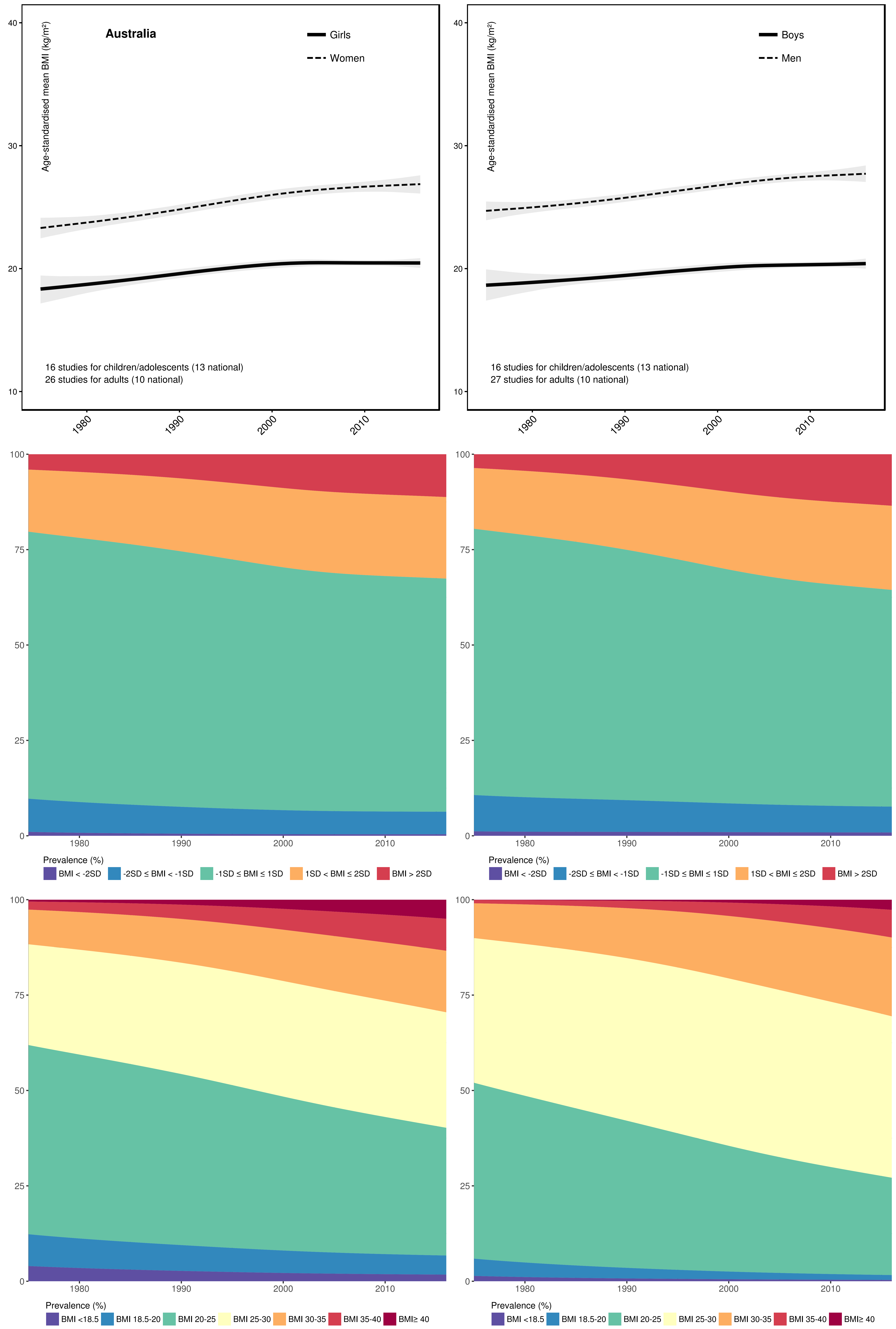
Australia
Girls
Women
Boys
Men
Age-standardised mean BMI (kg/m²)
16 studies for children/adolescents (13 national)
26 studies for adults (10 national)
16 studies for children/adolescents (13 national)
27 studies for adults (10 national)
1980
1990
2000
2010
Prevalence (%)
BMI < -2SD
-2SD ≤ BMI < -1SD
-1SD ≤ BMI ≤ 1SD
1SD < BMI ≤ 2SD
BMI > 2SD
BMI <18.5
BMI 18.5-20
BMI 20-25
BMI 25-30
BMI 30-35
BMI 35-40
BMI≥ 40

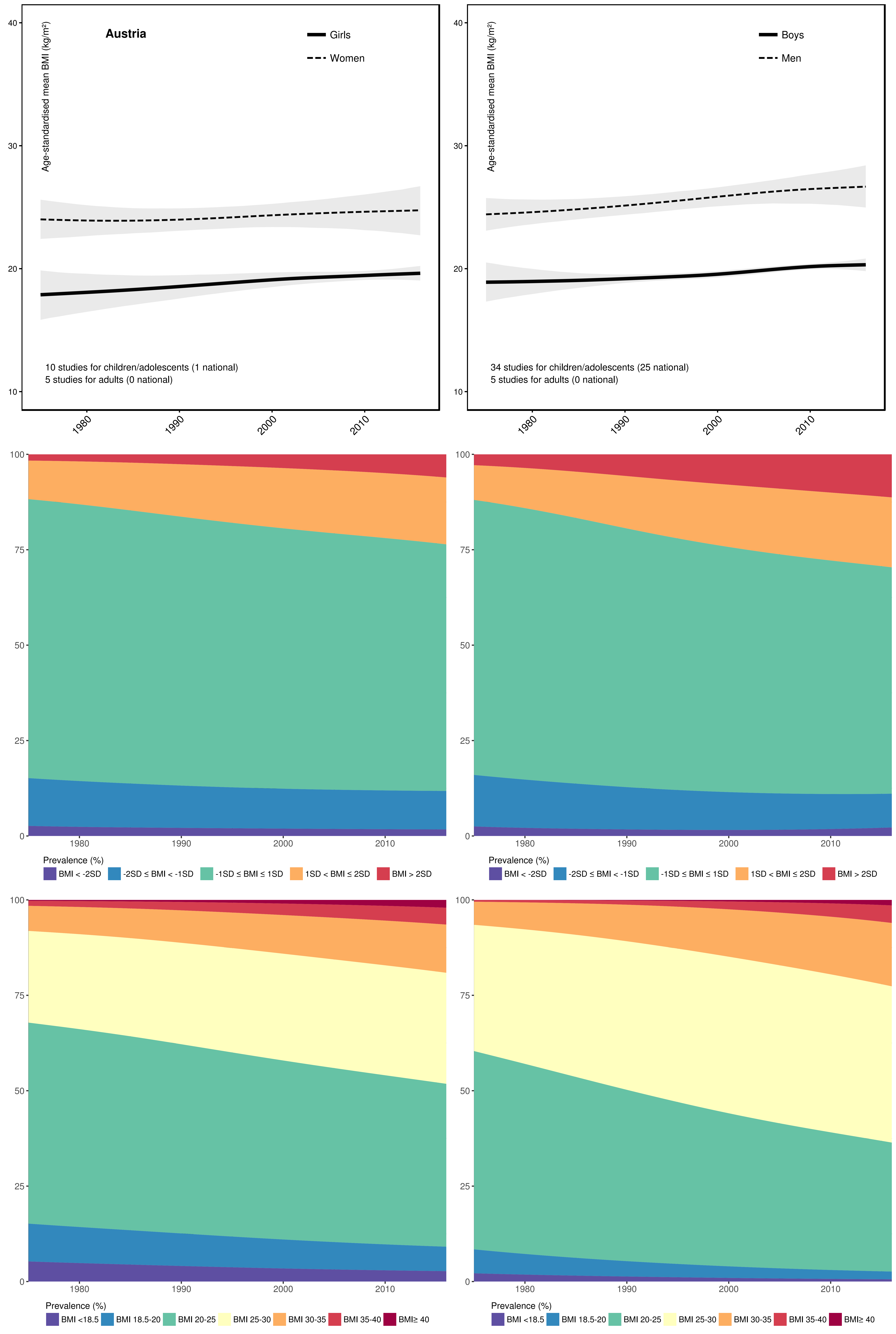
Austria
Girls
Women
Boys
Men
Age-standardised mean BMI (kg/m²)
10 studies for children/adolescents (1 national)
5 studies for adults (0 national)
34 studies for children/adolescents (25 national)
5 studies for adults (0 national)
Prevalence (%)
BMI < -2SD
-2SD ≤ BMI < -1SD
-1SD ≤ BMI ≤ 1SD
1SD < BMI ≤ 2SD
BMI > 2SD
BMI <18.5
BMI 18.5-20
BMI 20-25
BMI 25-30
BMI 30-35
BMI 35-40
BMI≥ 40

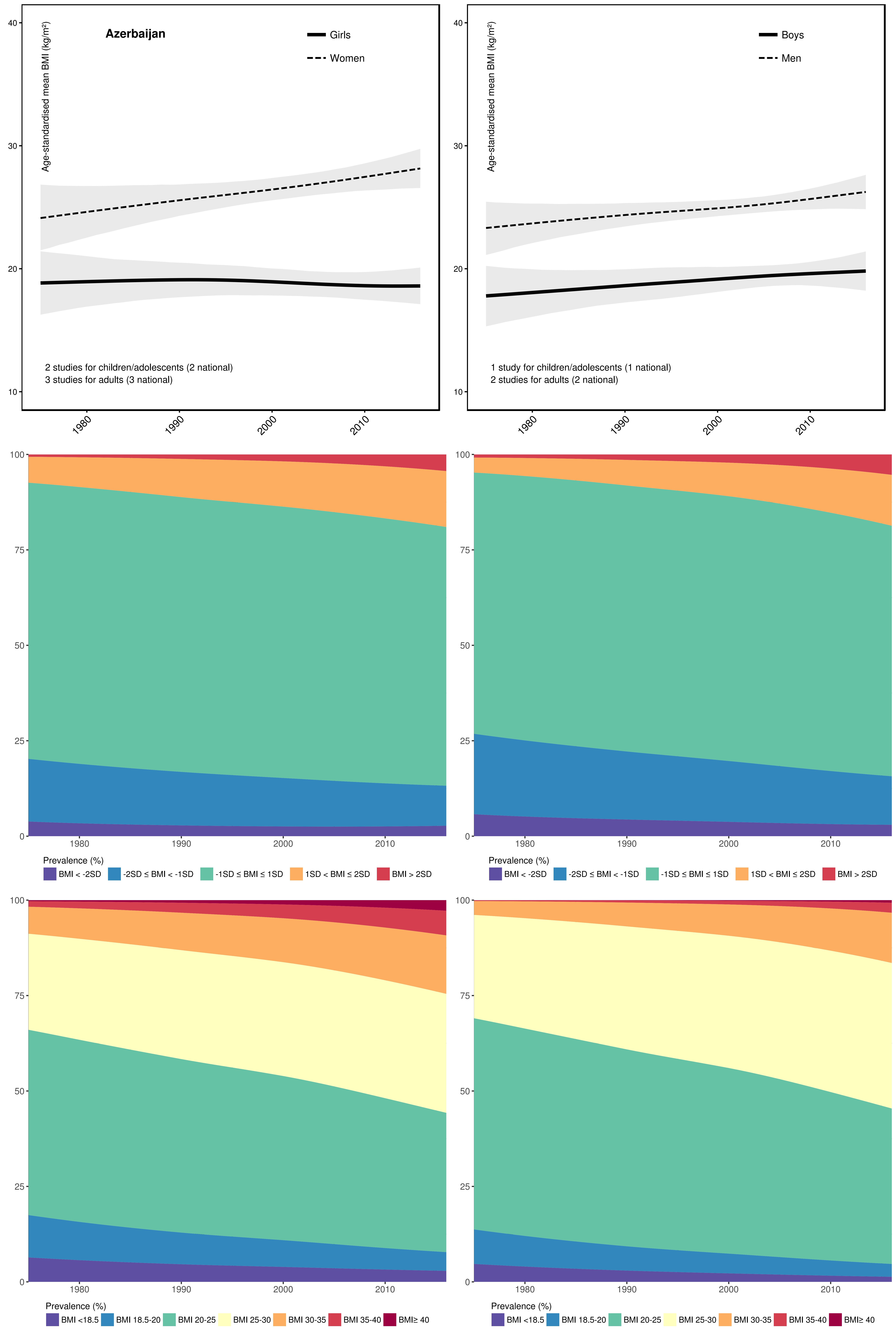

Azerbaijan
Girls
Women
Age-standardised mean BMI (kg/m²)
2 studies for children/adolescents (2 national)
3 studies for adults (3 national)
Boys
Men
1 study for children/adolescents (1 national)
2 studies for adults (2 national)
Prevalence (%)
BMI < -2SD
-2SD ≤ BMI < -1SD
-1SD ≤ BMI ≤ 1SD
1SD < BMI ≤ 2SD
BMI > 2SD
BMI <18.5
BMI 18.5-20
BMI 20-25
BMI 25-30
BMI 30-35
BMI 35-40
BMI≥ 40

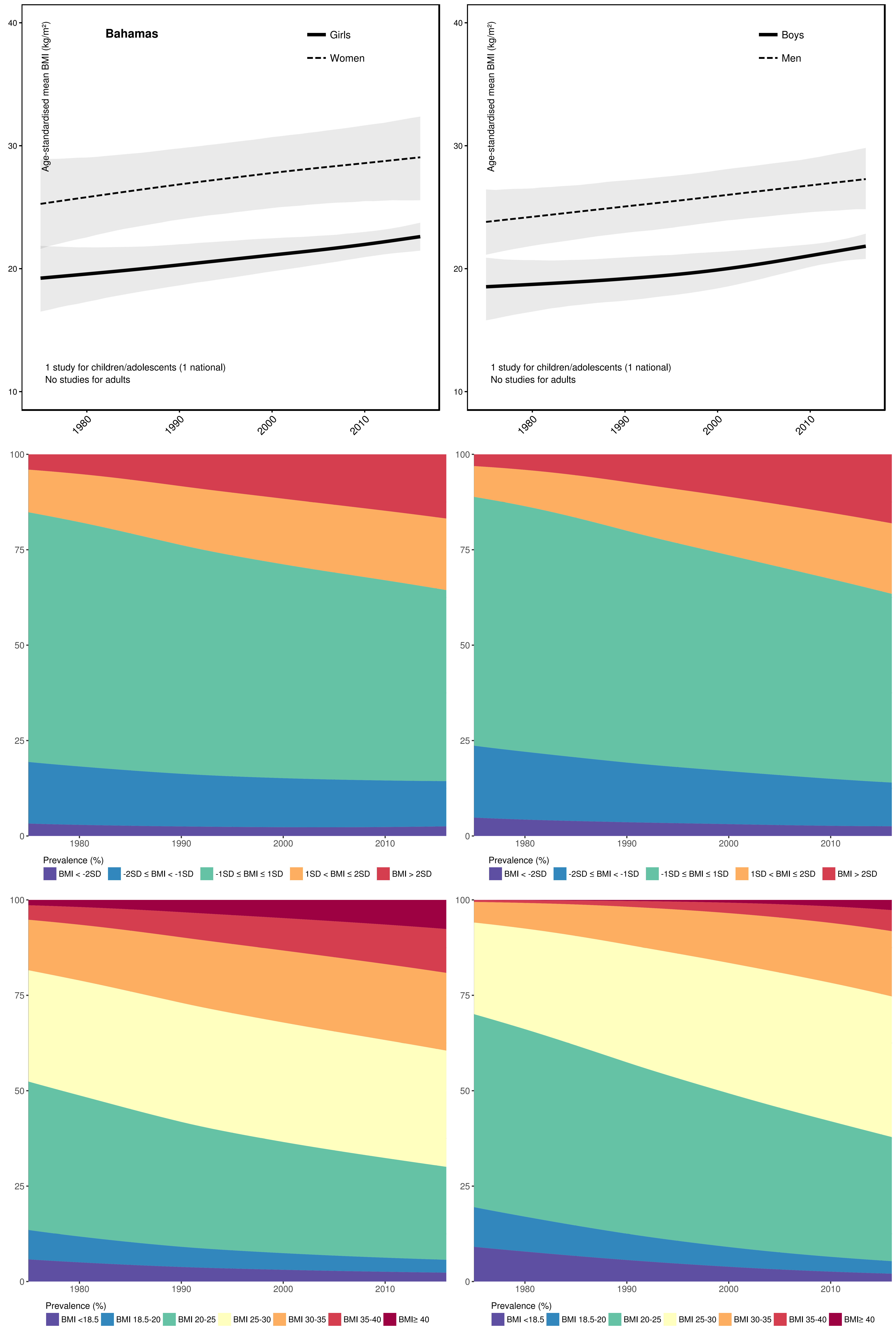
Bahamas
Girls
Women
Boys
Men
Age-standardised mean BMI (kg/m²)
1 study for children/adolescents (1 national)
No studies for adults
1980
1990
2000
2010
Prevalence (%)
BMI < -2SD
-2SD ≤ BMI < -1SD
-1SD ≤ BMI ≤ 1SD
1SD < BMI ≤ 2SD
BMI > 2SD
BMI <18.5
BMI 18.5-20
BMI 20-25
BMI 25-30
BMI 30-35
BMI 35-40
BMI≥ 40

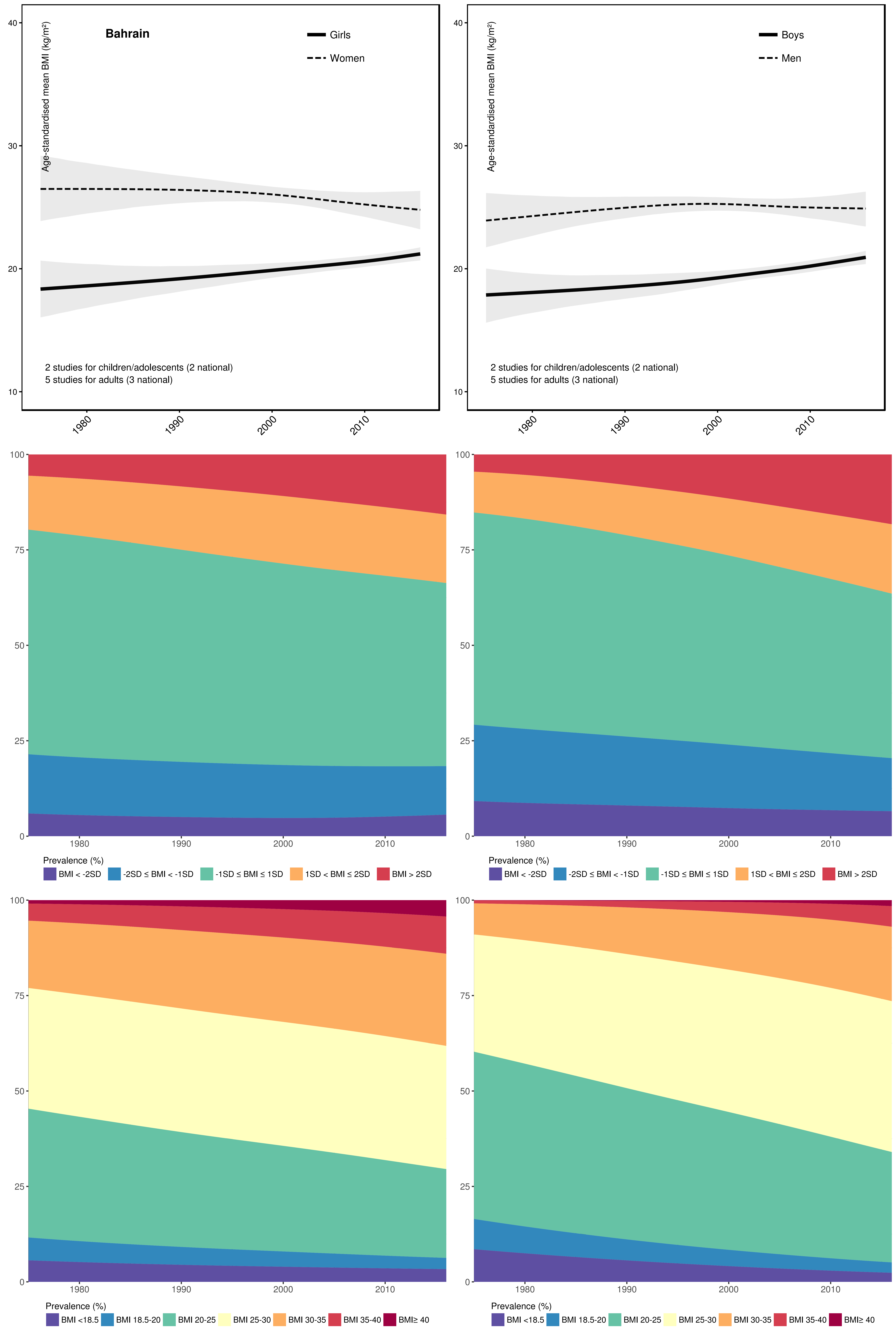
Bahrain
Girls
Women
Boys
Men
Age-standardised mean BMI (kg/m²)
2 studies for children/adolescents (2 national)
5 studies for adults (3 national)
Prevalence (%)
BMI < -2SD
-2SD ≤ BMI < -1SD
-1SD ≤ BMI ≤ 1SD
1SD < BMI ≤ 2SD
BMI > 2SD
BMI <18.5
BMI 18.5-20
BMI 20-25
BMI 25-30
BMI 30-35
BMI 35-40
BMI≥ 40

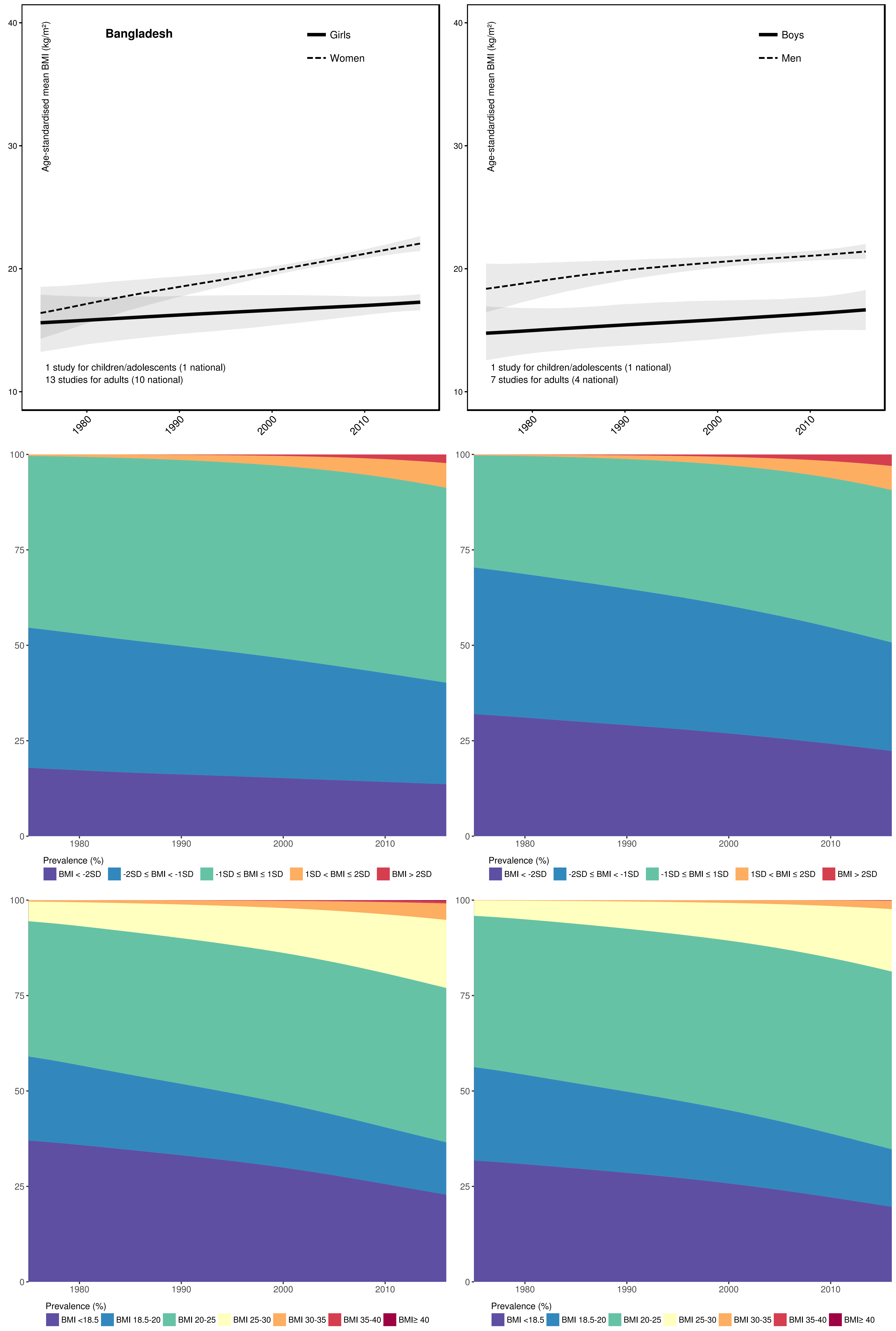
Bangladesh
Girls
Women
Boys
Men
Age-standardised mean BMI (kg/m²)
1 study for children/adolescents (1 national)
13 studies for adults (10 national)
1 study for children/adolescents (1 national)
7 studies for adults (4 national)
1980
1990
2000
2010
Prevalence (%)
BMI < -2SD
-2SD ≤ BMI < -1SD
-1SD ≤ BMI ≤ 1SD
1SD < BMI ≤ 2SD
BMI > 2SD
BMI <18.5
BMI 18.5-20
BMI 20-25
BMI 25-30
BMI 30-35
BMI 35-40
BMI≥ 40

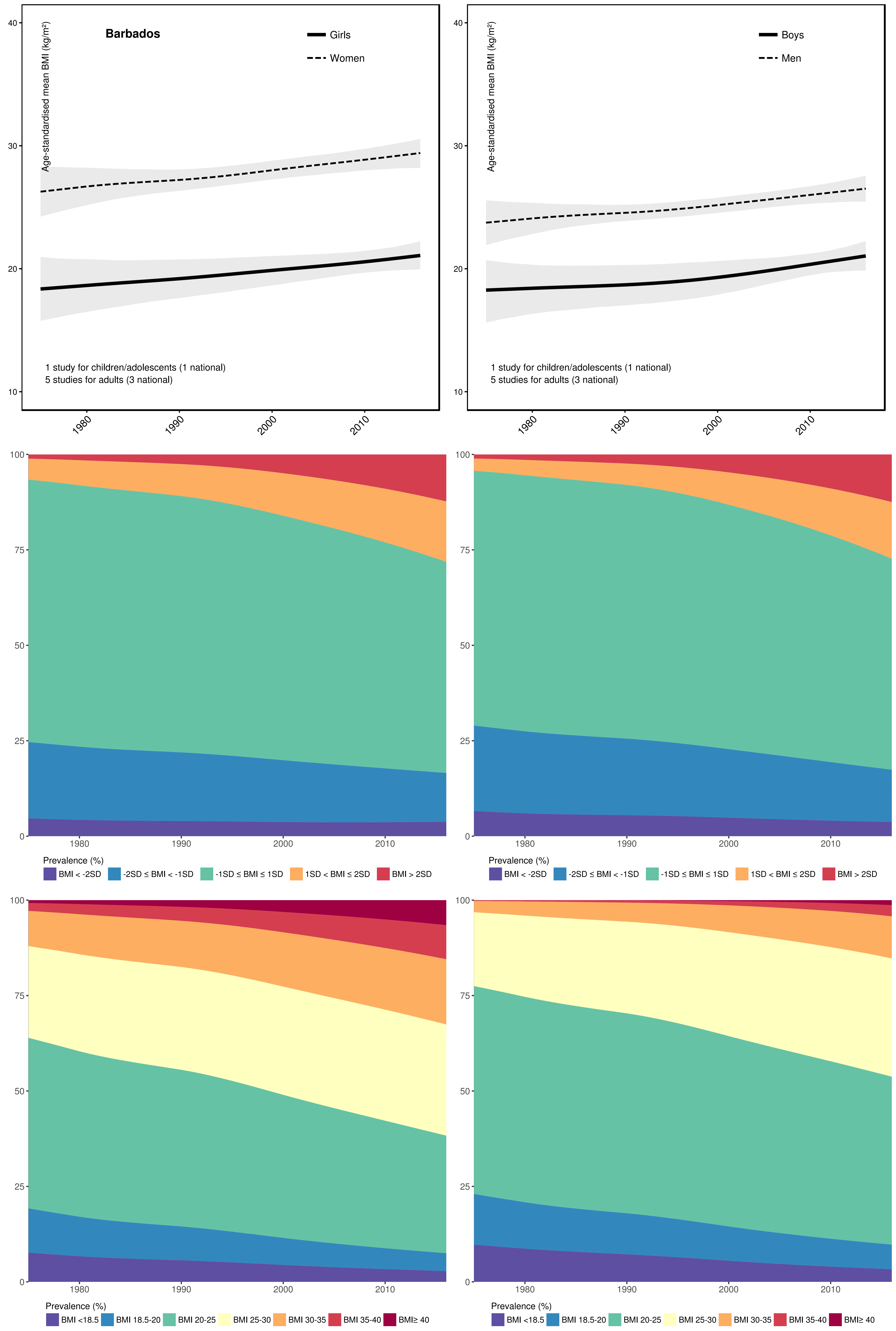
Barbados
Girls
Women
Boys
Men
Age-standardised mean BMI (kg/m²)
1 study for children/adolescents (1 national)
5 studies for adults (3 national)
1 study for children/adolescents (1 national)
5 studies for adults (3 national)
Prevalence (%)
BMI < -2SD
-2SD ≤ BMI < -1SD
-1SD ≤ BMI ≤ 1SD
1SD < BMI ≤ 2SD
BMI > 2SD
Prevalence (%)
BMI <18.5
BMI 18.5-20
BMI 20-25
BMI 25-30
BMI 30-35
BMI 35-40
BMI≥ 40

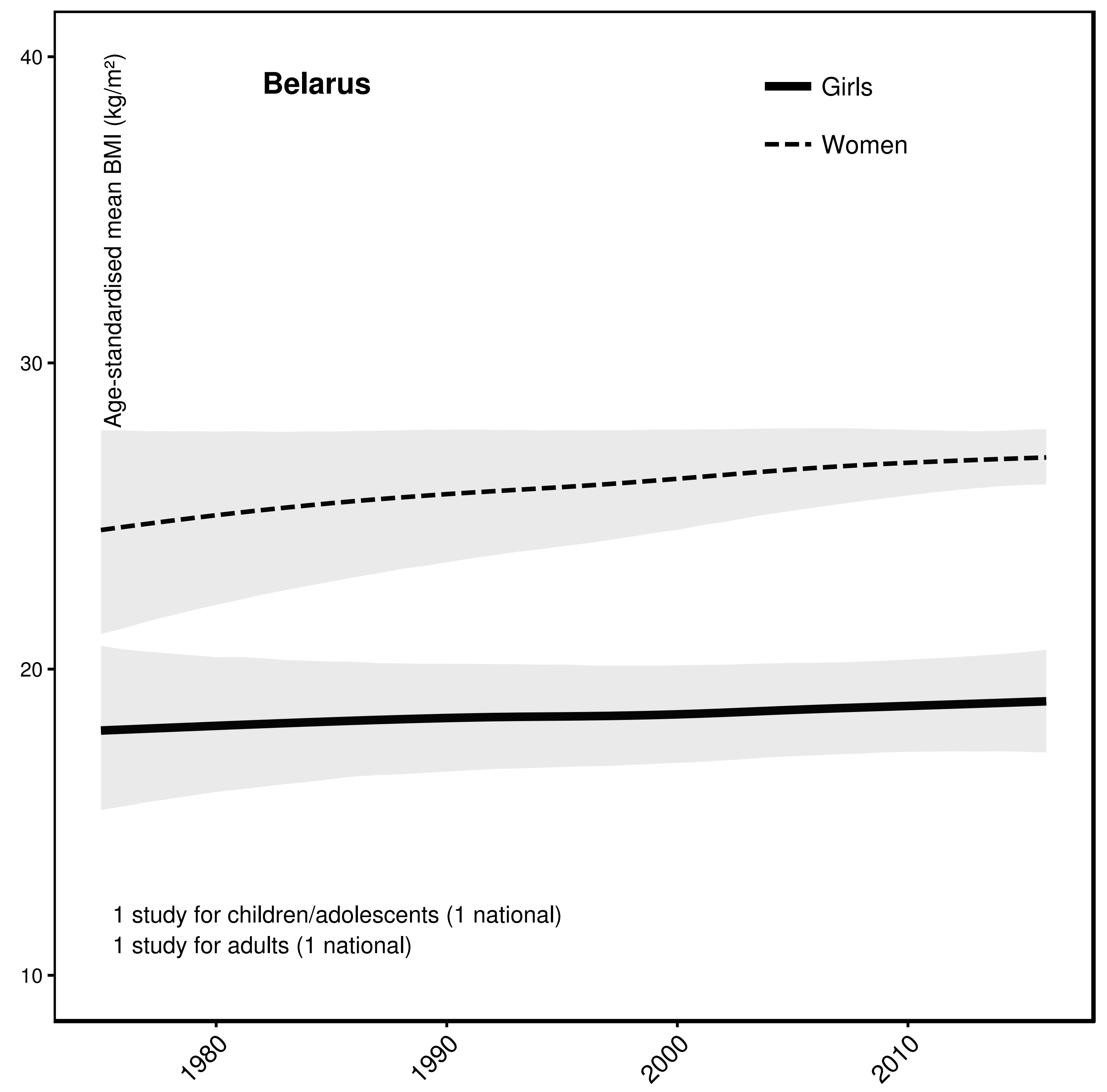
Belarus
Girls
Women
Age-standardised mean BMI (kg/m²)
1 study for children/adolescents (1 national)
1 study for adults (1 national)
40
30
20
10
1980
1990
2000
2010

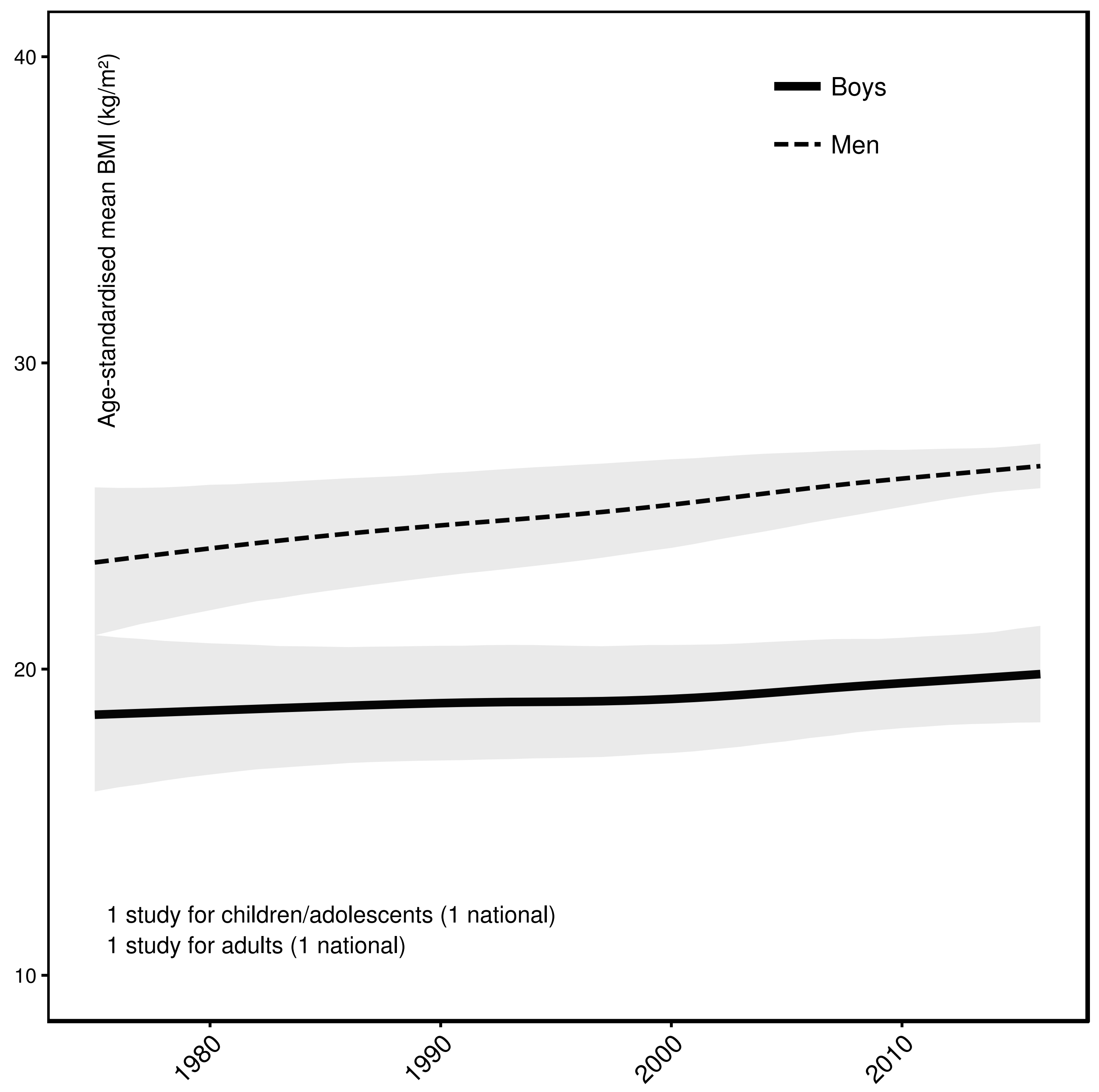
Boys
Men
Age-standardised mean BMI (kg/m²)
1 study for children/adolescents (1 national)
1 study for adults (1 national)
40
30
20
10
1980
1990
2000
2010

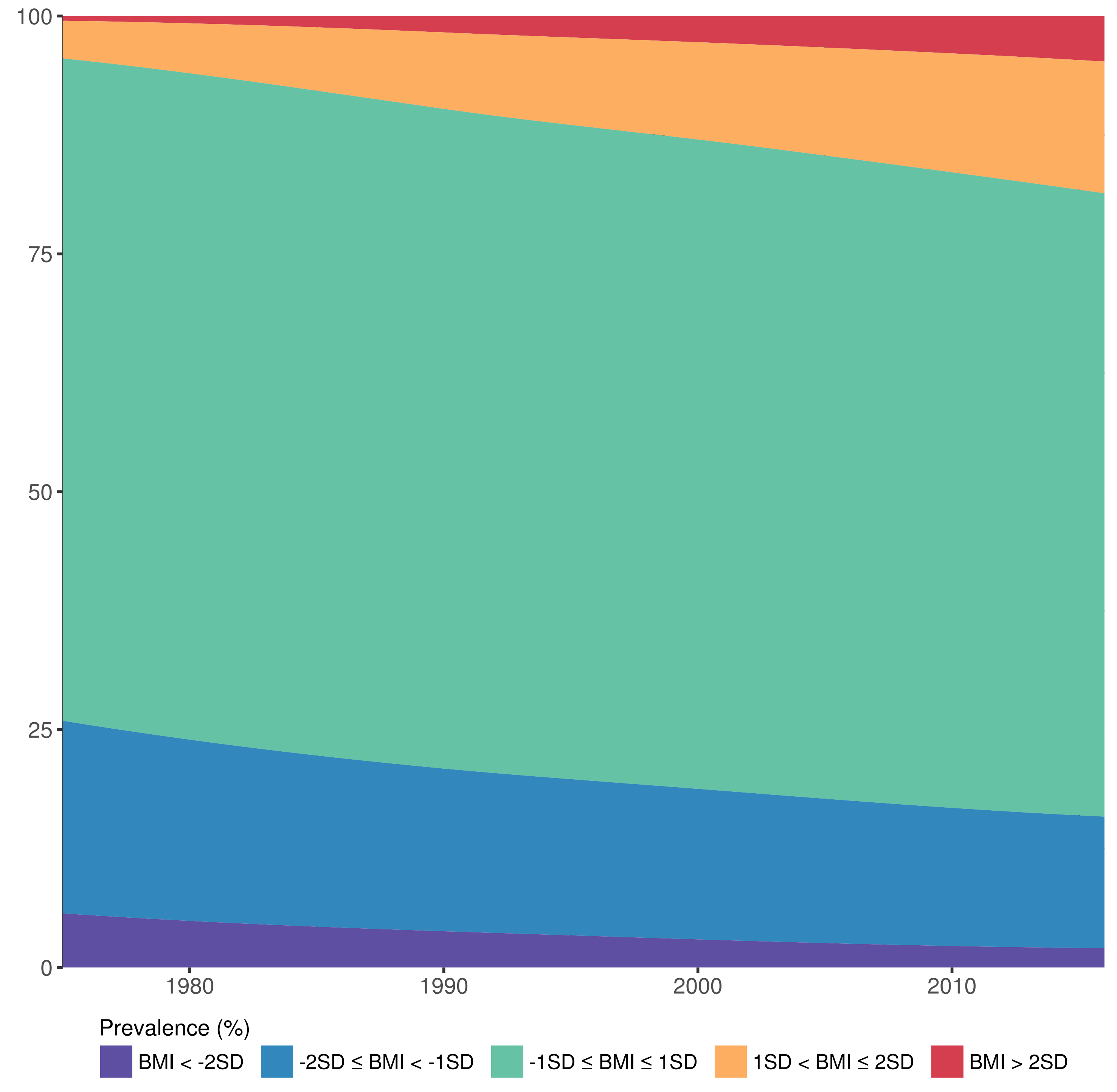
100
75
50
25
0
1980
1990
2000
2010
Prevalence (%)
BMI < -2SD
-2SD ≤ BMI < -1SD
-1SD ≤ BMI ≤ 1SD
1SD < BMI ≤ 2SD
BMI > 2SD

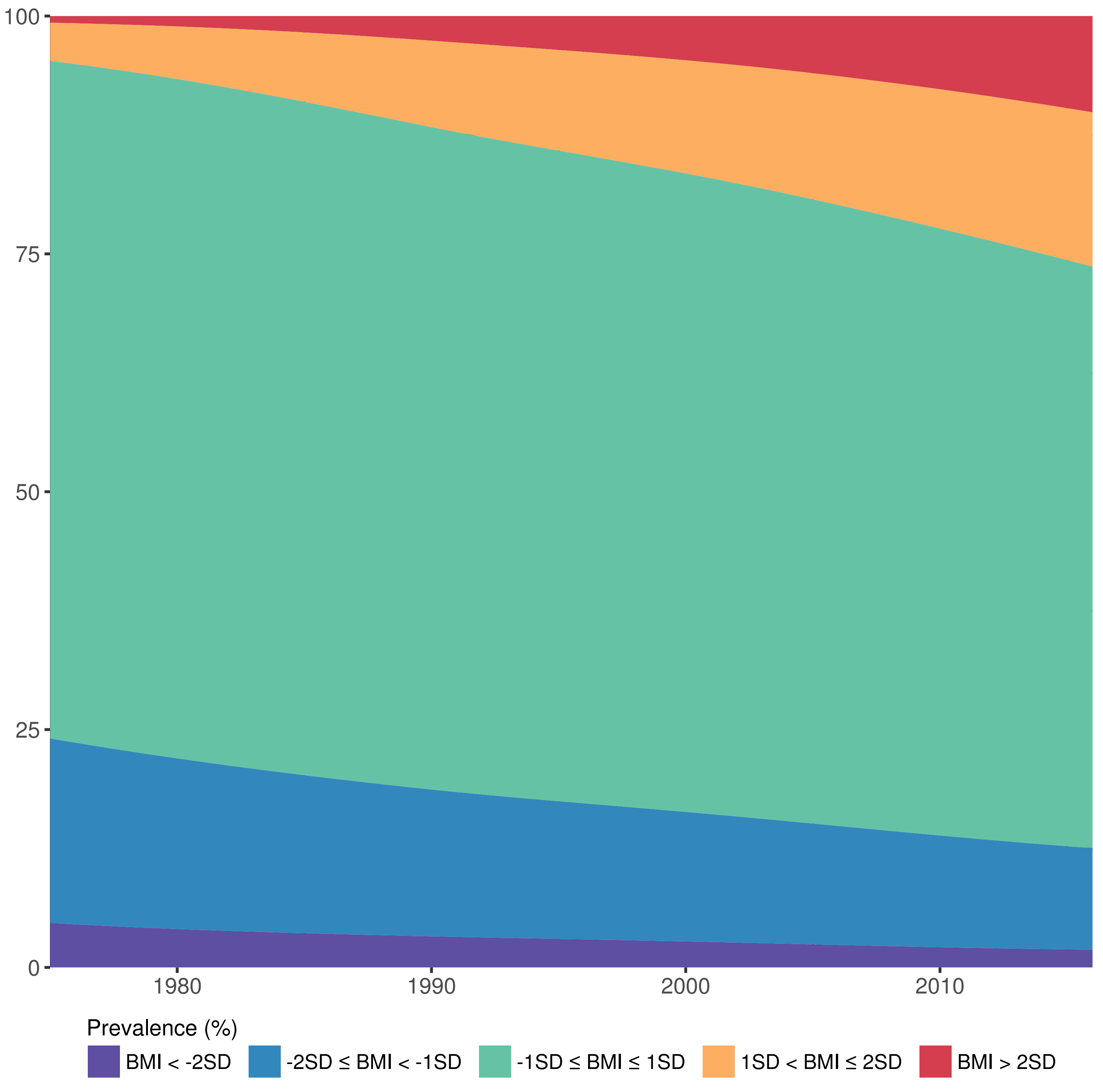
100
75
50
25
0
1980
1990
2000
2010
Prevalence (%)
BMI < -2SD
-2SD ≤ BMI < -1SD
-1SD ≤ BMI ≤ 1SD
1SD < BMI ≤ 2SD
BMI > 2SD

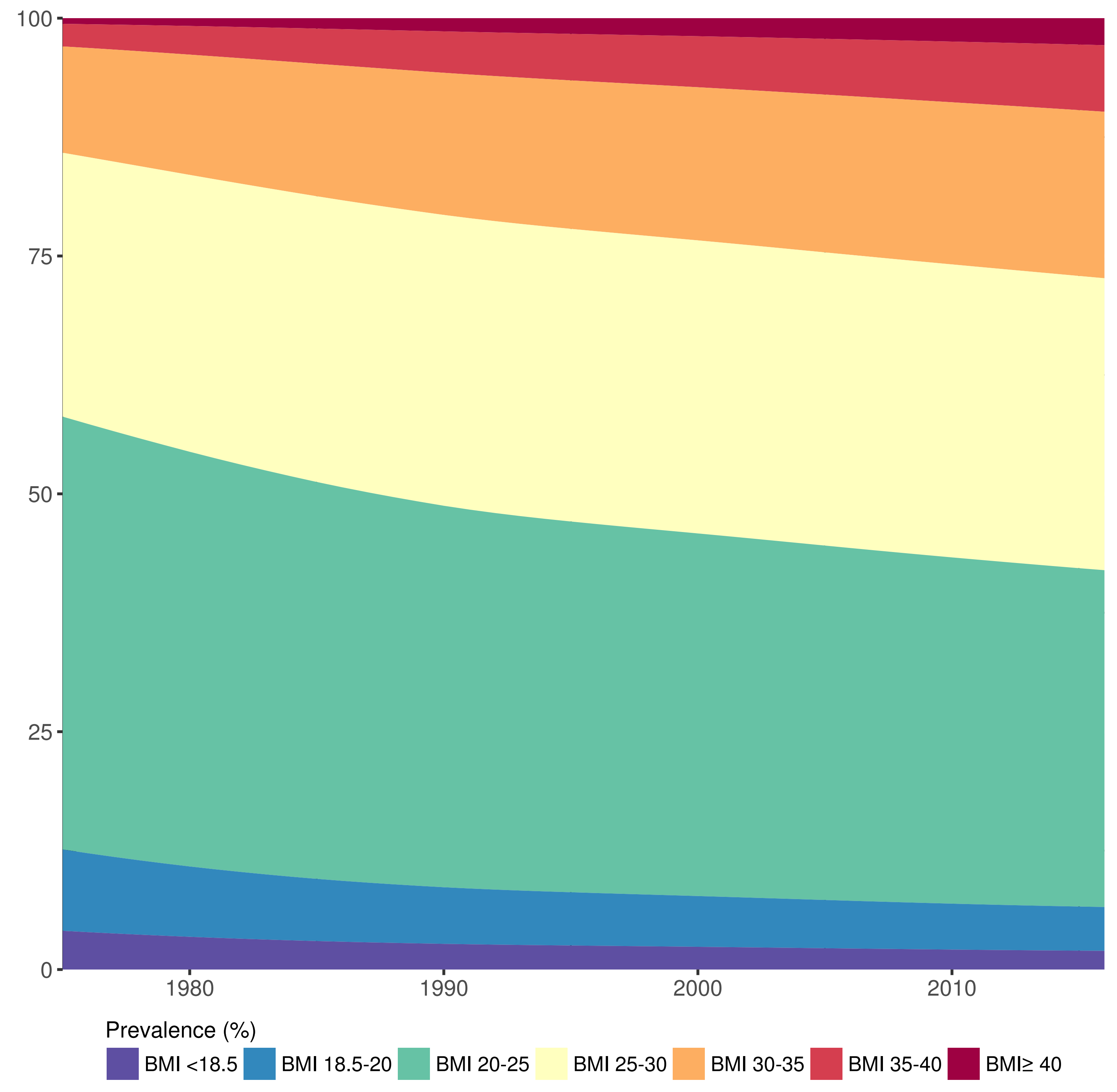
100
75
50
25
0
1980
1990
2000
2010
Prevalence (%)
BMI <18.5
BMI 18.5-20
BMI 20-25
BMI 25-30
BMI 30-35
BMI 35-40
BMI≥ 40

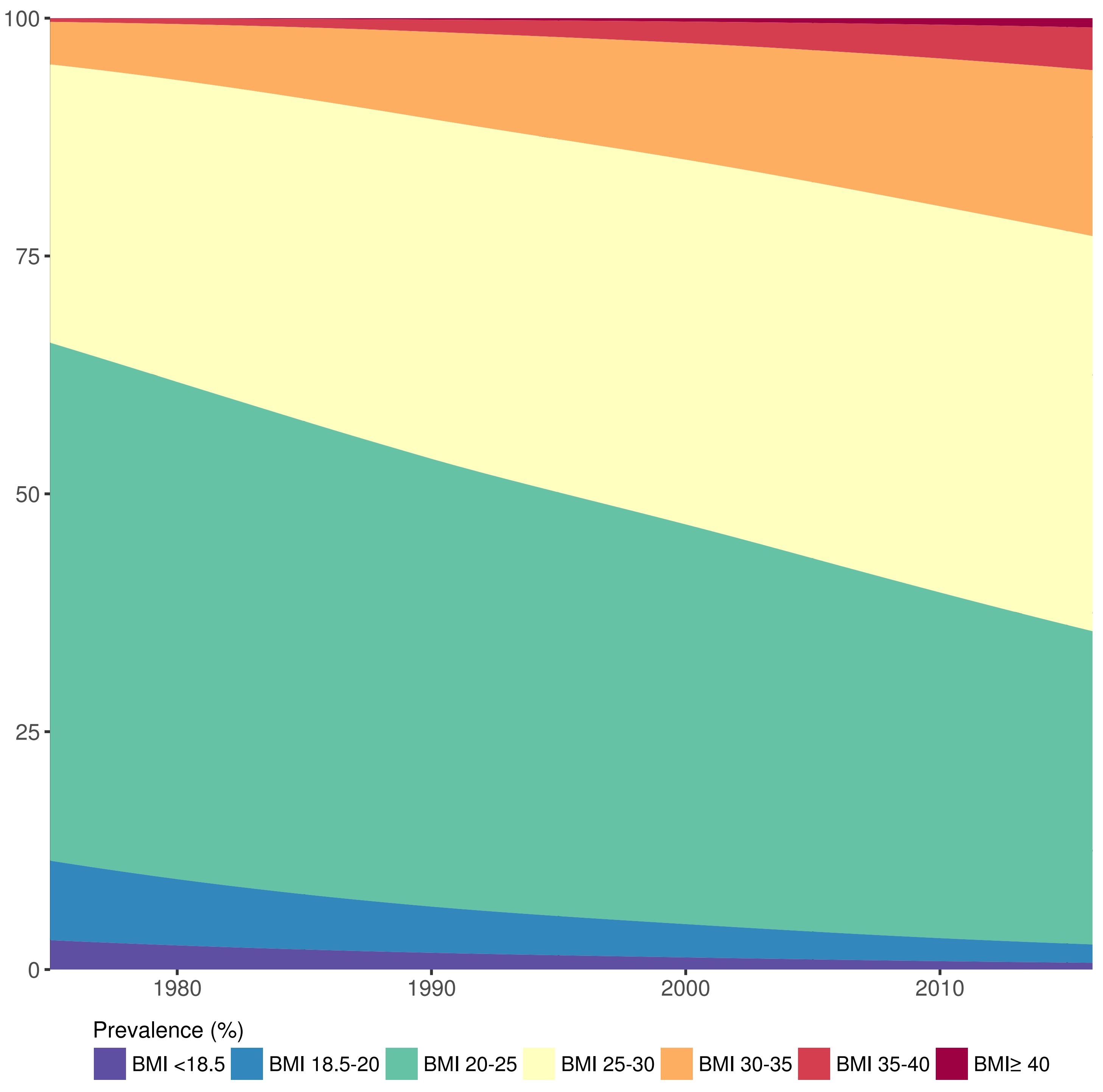
100
75
50
25
0
1980
1990
2000
2010
Prevalence (%)
BMI <18.5
BMI 18.5-20
BMI 20-25
BMI 25-30
BMI 30-35
BMI 35-40
BMI≥ 40

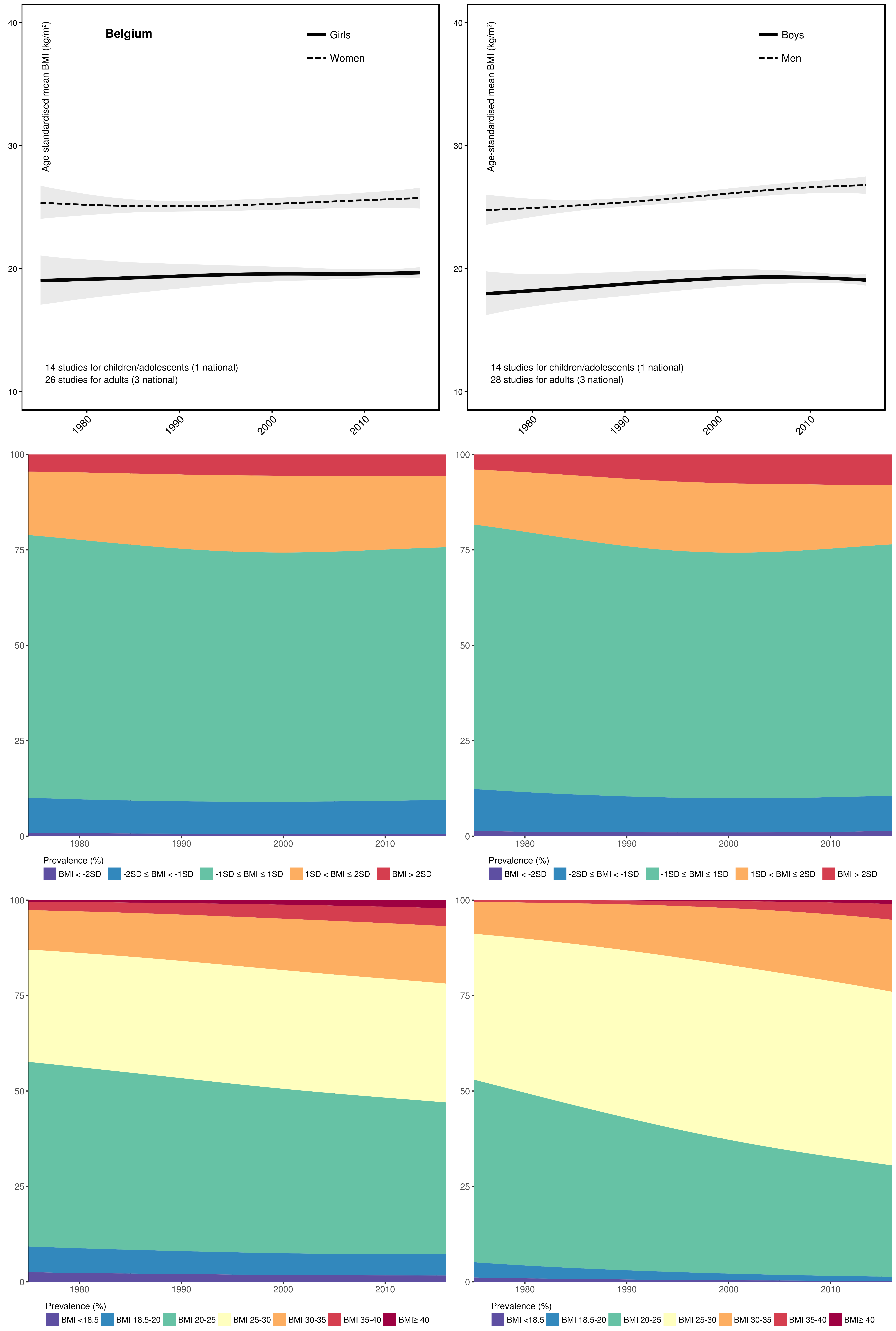
Belgium
Girls
Women
Boys
Men
Age-standardised mean BMI (kg/m²)
14 studies for children/adolescents (1 national)
26 studies for adults (3 national)
14 studies for children/adolescents (1 national)
28 studies for adults (3 national)
Prevalence (%)
BMI < -2SD
-2SD ≤ BMI < -1SD
-1SD ≤ BMI ≤ 1SD
1SD < BMI ≤ 2SD
BMI > 2SD
BMI <18.5
BMI 18.5-20
BMI 20-25
BMI 25-30
BMI 30-35
BMI 35-40
BMI≥ 40

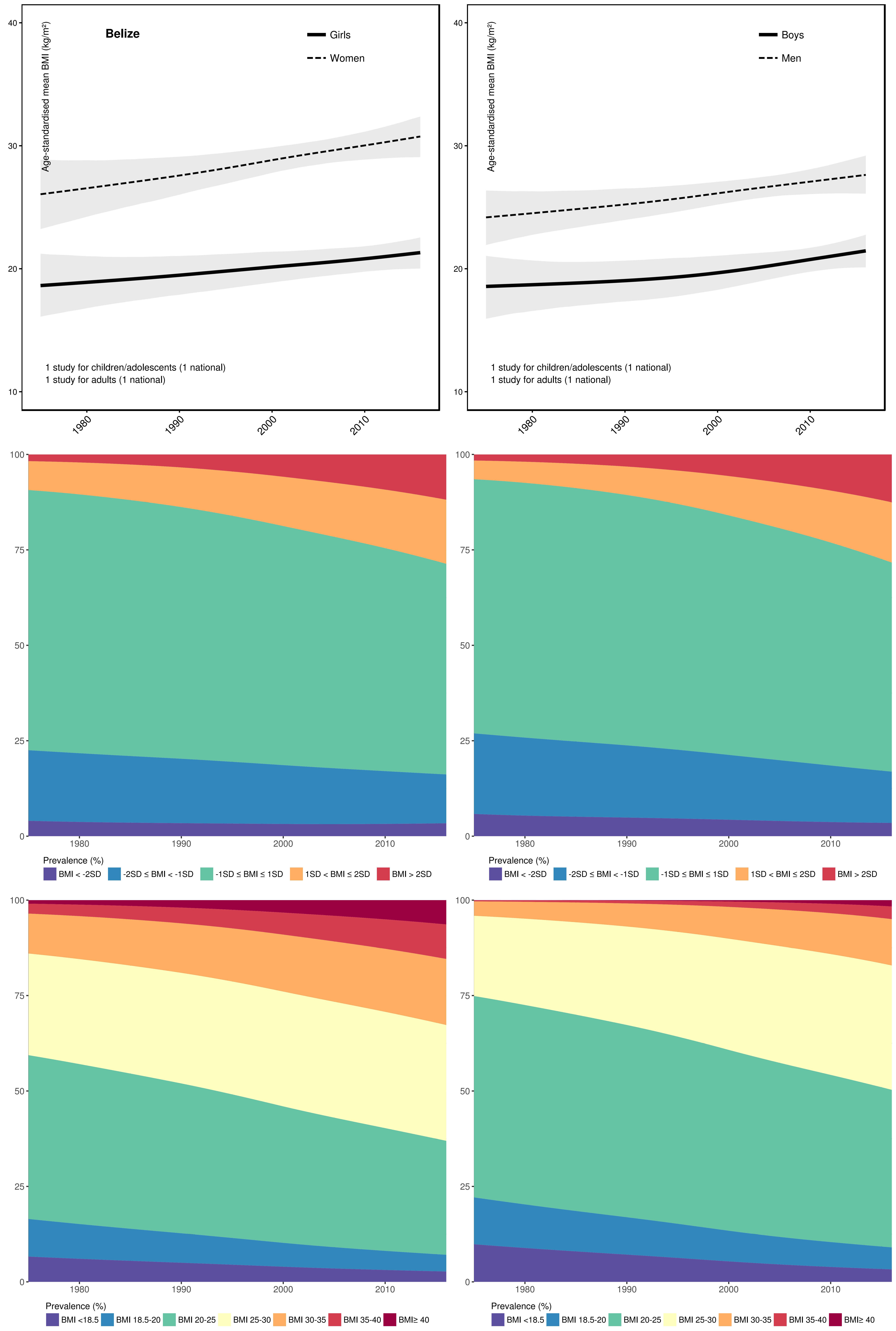
Belize
Girls
Women
Age-standardised mean BMI (kg/m²)
1 study for children/adolescents (1 national)
1 study for adults (1 national)
Boys
Men
Age-standardised mean BMI (kg/m²)
1 study for children/adolescents (1 national)
1 study for adults (1 national)
Prevalence (%)
BMI < -2SD
-2SD ≤ BMI < -1SD
-1SD ≤ BMI ≤ 1SD
1SD < BMI ≤ 2SD
BMI > 2SD
Prevalence (%)
BMI < -2SD
-2SD ≤ BMI < -1SD
-1SD ≤ BMI ≤ 1SD
1SD < BMI ≤ 2SD
BMI > 2SD
Prevalence (%)
BMI <18.5
BMI 18.5-20
BMI 20-25
BMI 25-30
BMI 30-35
BMI 35-40
BMI≥ 40
Prevalence (%)
BMI <18.5
BMI 18.5-20
BMI 20-25
BMI 25-30
BMI 30-35
BMI 35-40
BMI≥ 40

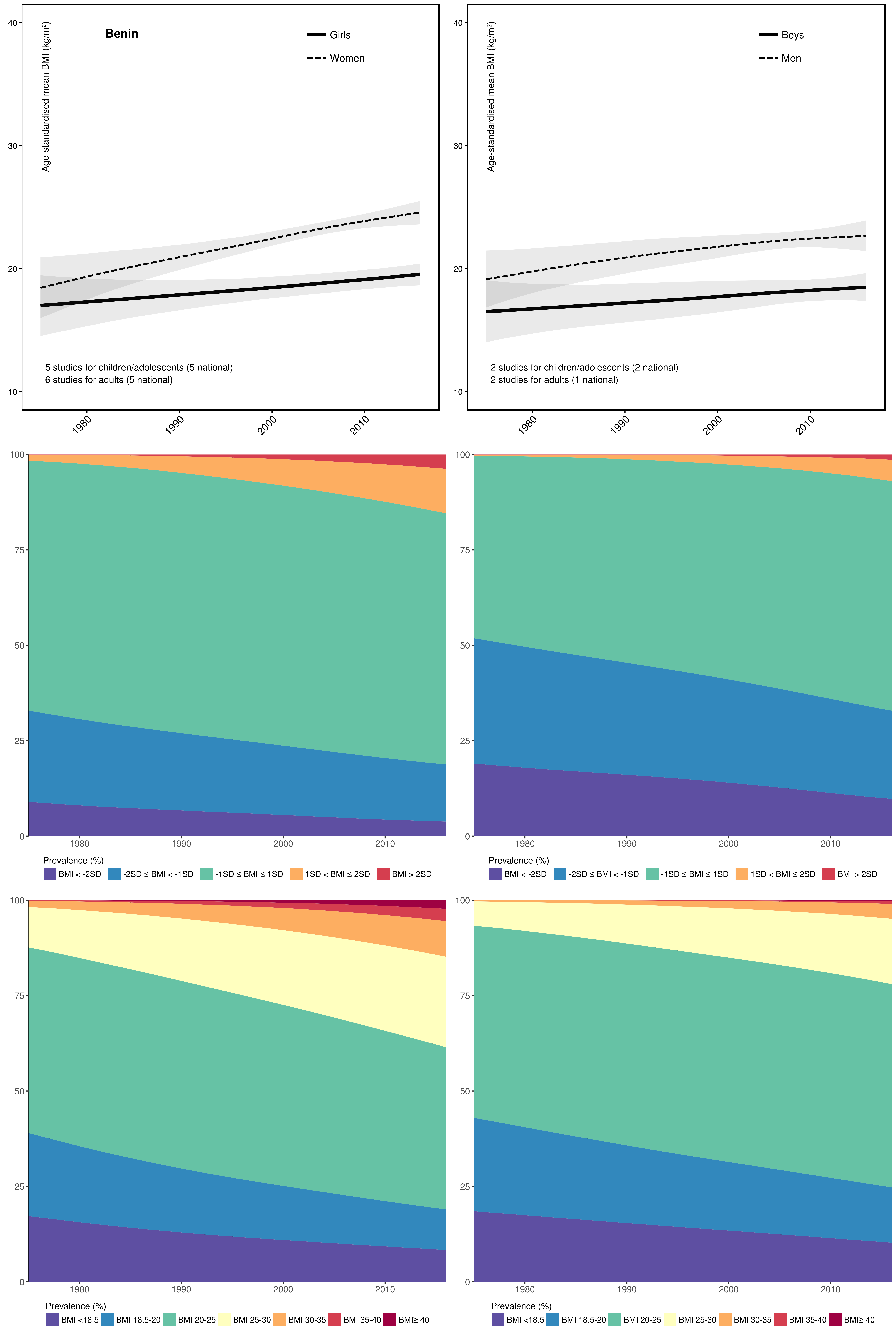

Benin
Girls
Women
Boys
Men
Age-standardised mean BMI (kg/m²)
5 studies for children/adolescents (5 national)
6 studies for adults (5 national)
2 studies for children/adolescents (2 national)
2 studies for adults (1 national)
Prevalence (%)
BMI < -2SD
-2SD ≤ BMI < -1SD
-1SD ≤ BMI ≤ 1SD
1SD < BMI ≤ 2SD
BMI > 2SD
BMI <18.5
BMI 18.5-20
BMI 20-25
BMI 25-30
BMI 30-35
BMI 35-40
BMI≥ 40

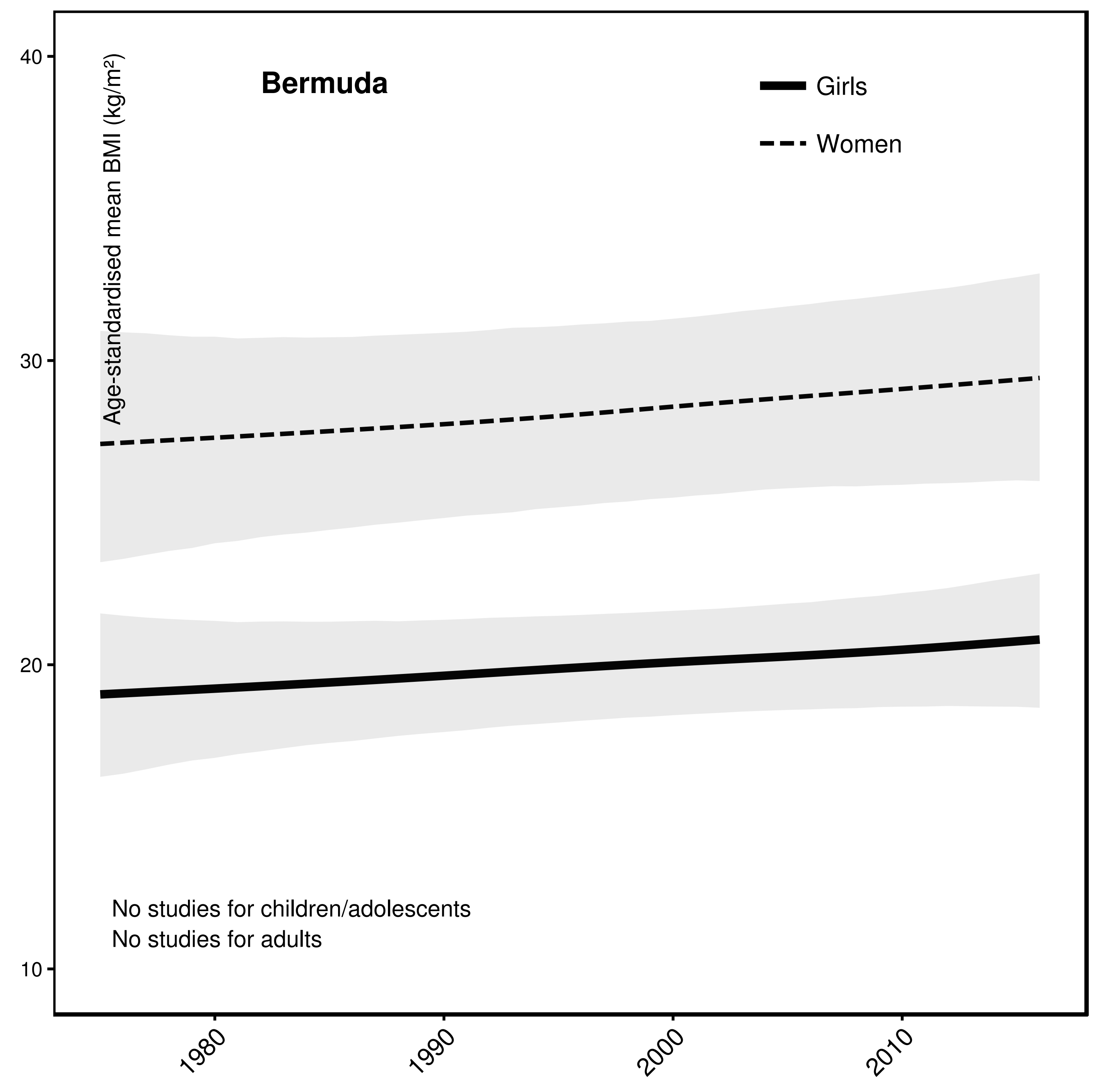
Bermuda
Girls
Women
Age-standardised mean BMI (kg/m²)
40
30
20
10
1980
1990
2000
2010
No studies for children/adolescents
No studies for adults

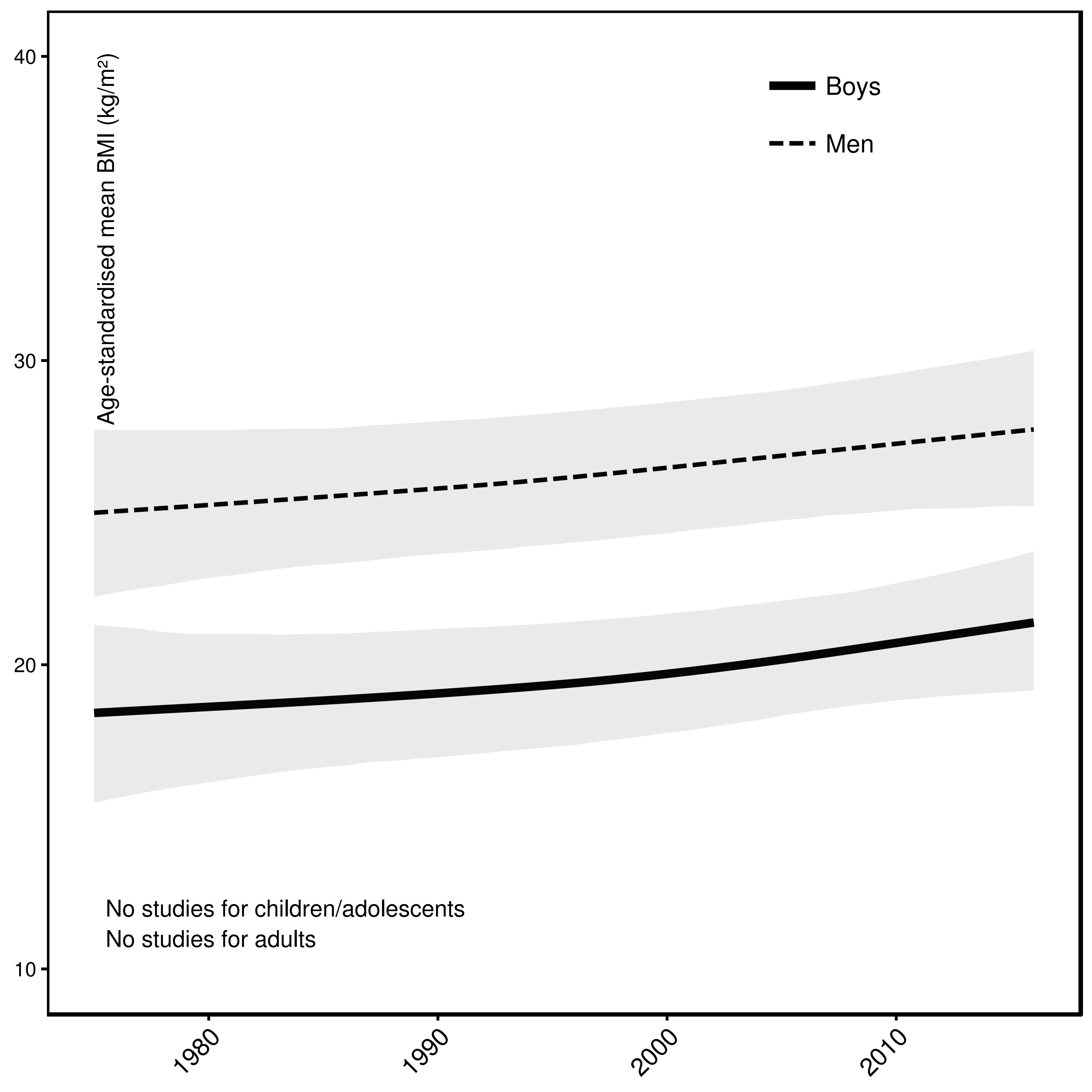
Boys
Men
Age-standardised mean BMI (kg/m²)
40
30
20
10
1980
1990
2000
2010
No studies for children/adolescents
No studies for adults

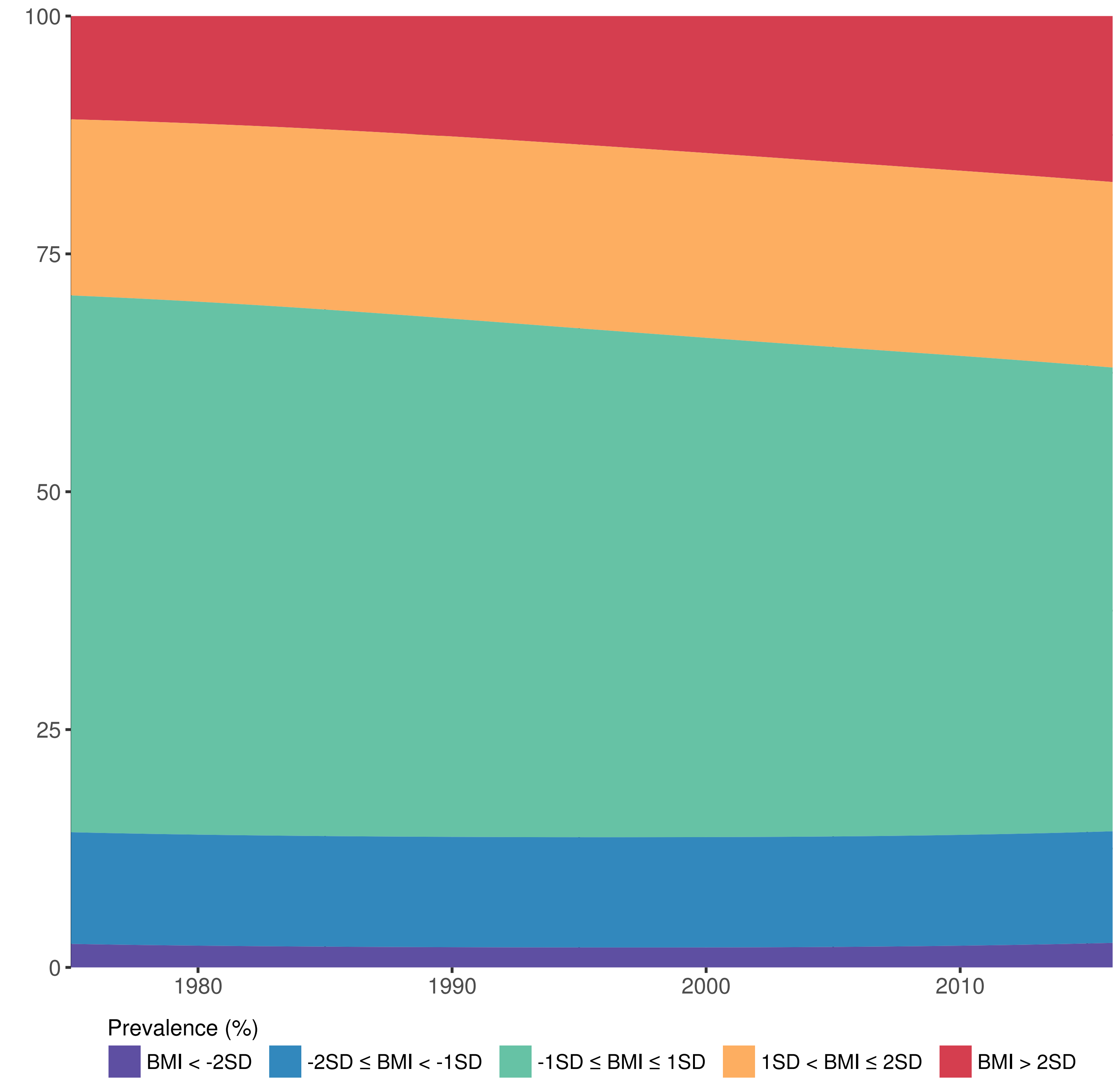
100
75
50
25
0
1980
1990
2000
2010
Prevalence (%)
BMI < -2SD
-2SD ≤ BMI < -1SD
-1SD ≤ BMI ≤ 1SD
1SD < BMI ≤ 2SD
BMI > 2SD

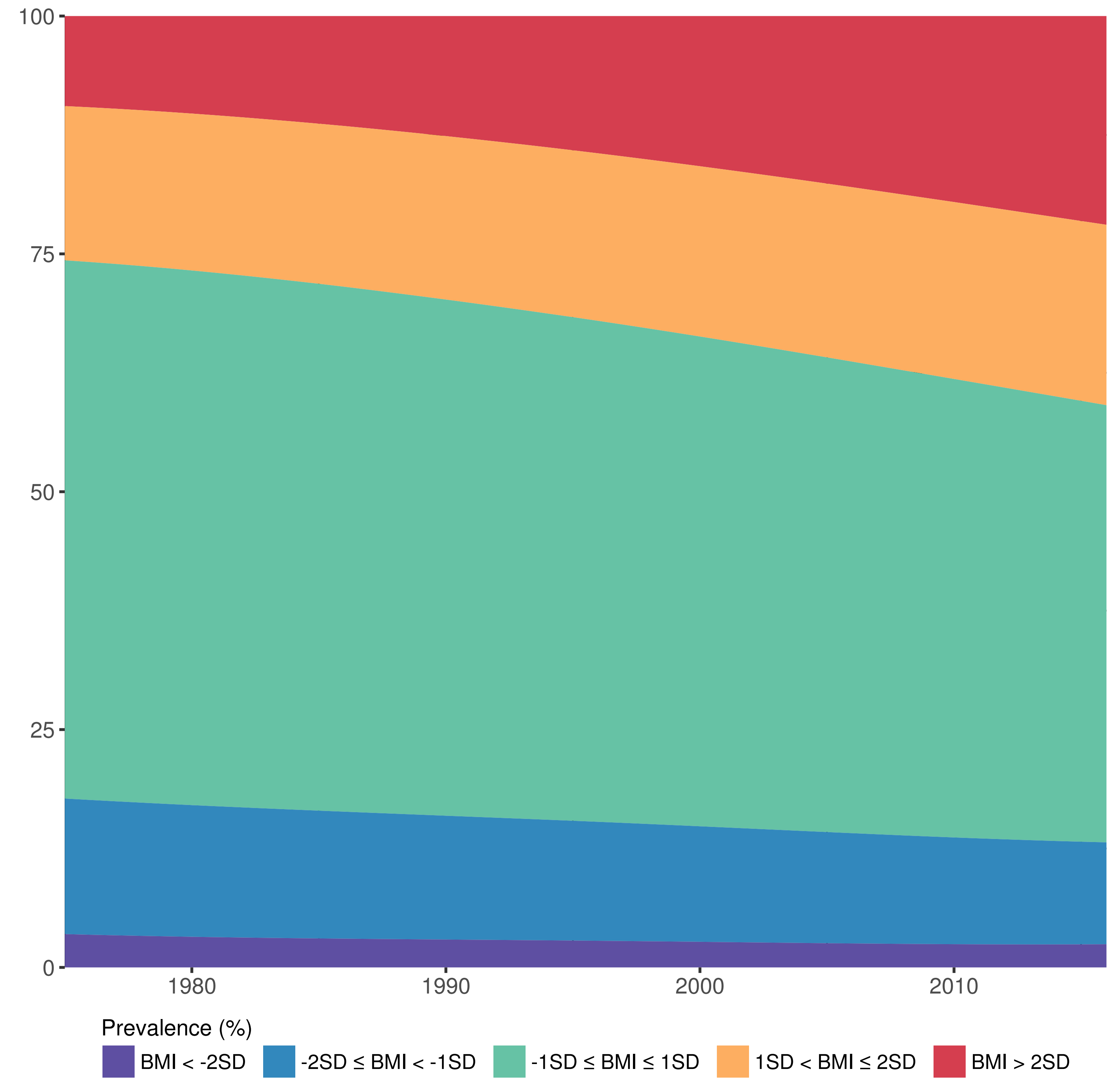
100
75
50
25
0
1980
1990
2000
2010
Prevalence (%)
BMI < -2SD
-2SD ≤ BMI < -1SD
-1SD ≤ BMI ≤ 1SD
1SD < BMI ≤ 2SD
BMI > 2SD

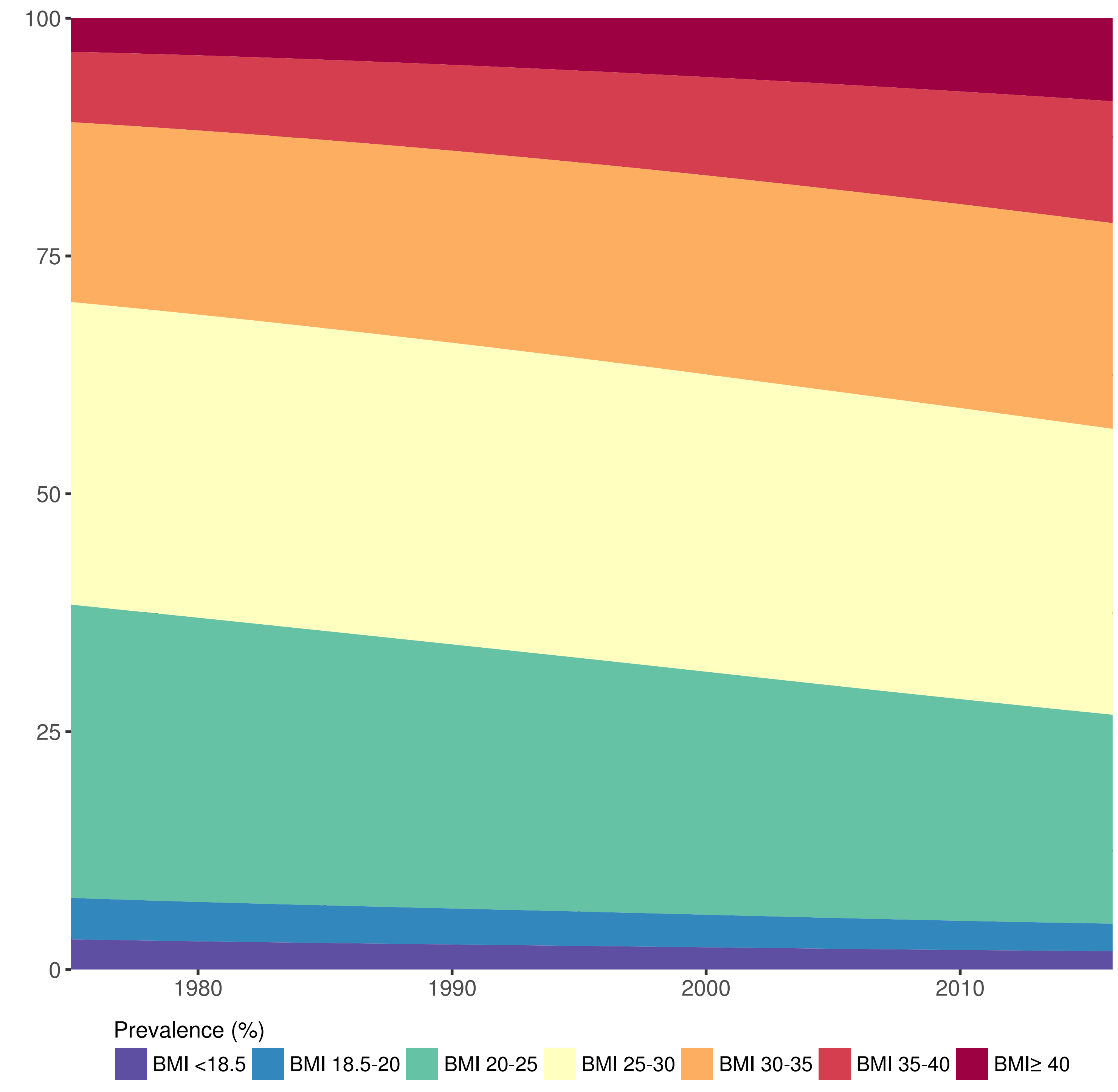
100
75
50
25
0
1980
1990
2000
2010
Prevalence (%)
BMI <18.5
BMI 18.5-20
BMI 20-25
BMI 25-30
BMI 30-35
BMI 35-40
BMI≥ 40

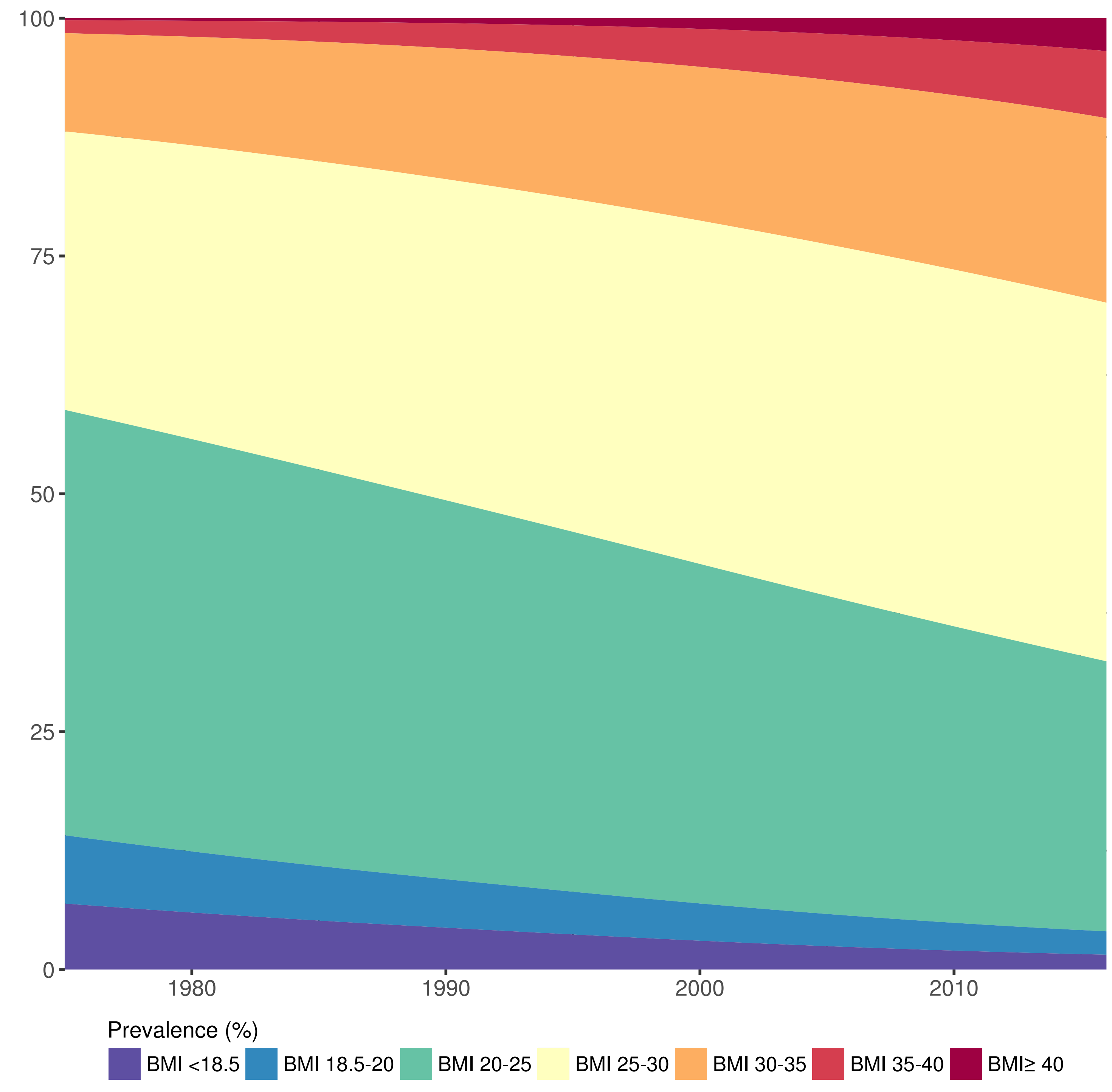
100
75
50
25
0
1980
1990
2000
2010
Prevalence (%)
BMI <18.5
BMI 18.5-20
BMI 20-25
BMI 25-30
BMI 30-35
BMI 35-40
BMI≥ 40

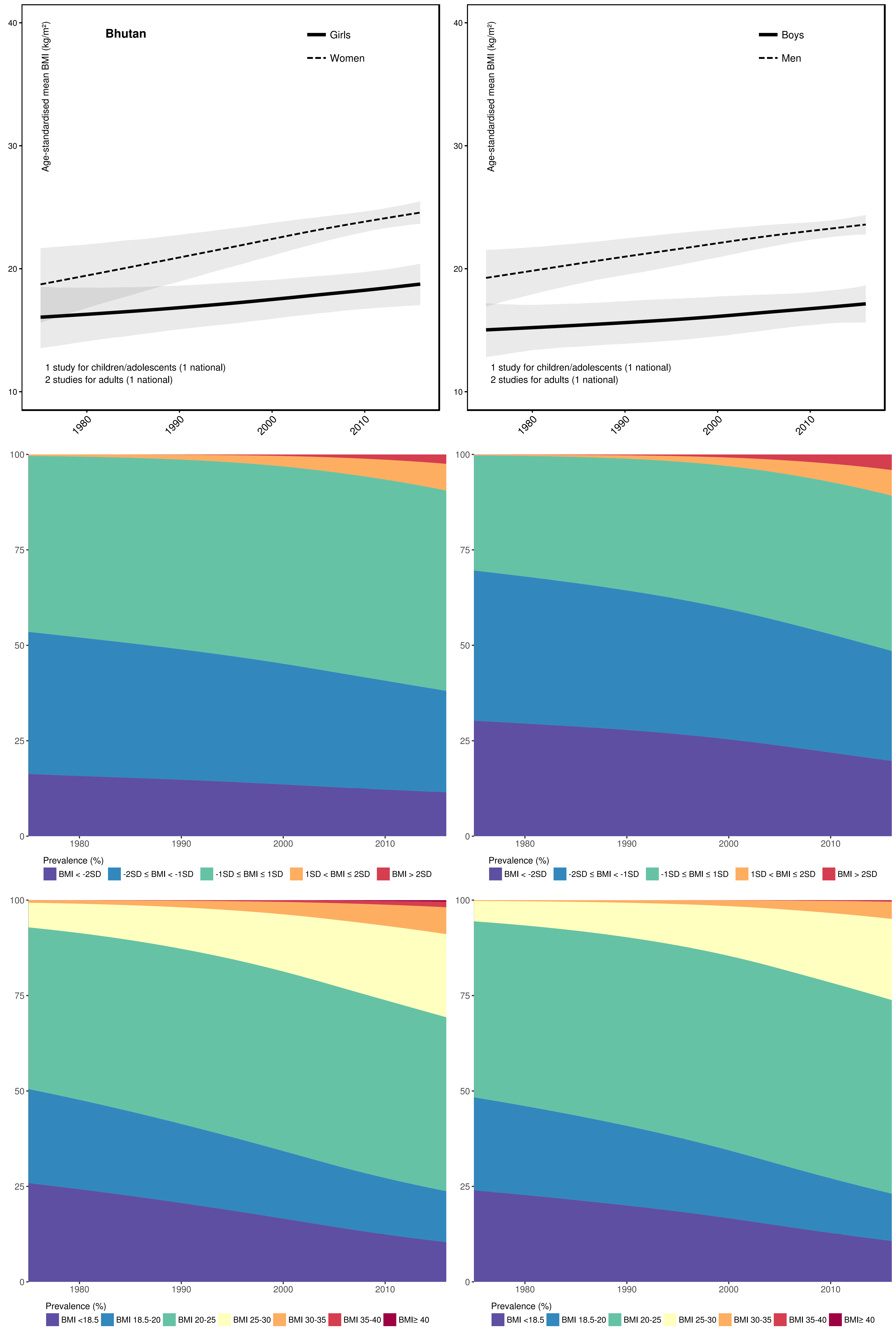
Bhutan
Girls
Women
Boys
Men
Age-standardised mean BMI (kg/m²)
1 study for children/adolescents (1 national)
2 studies for adults (1 national)
40
30
20
10
1980
1990
2000
2010
100
75
50
25
0
Prevalence (%)
BMI < -2SD
-2SD ≤ BMI < -1SD
-1SD ≤ BMI ≤ 1SD
1SD < BMI ≤ 2SD
BMI > 2SD
BMI <18.5
BMI 18.5-20
BMI 20-25
BMI 25-30
BMI 30-35
BMI 35-40
BMI≥ 40

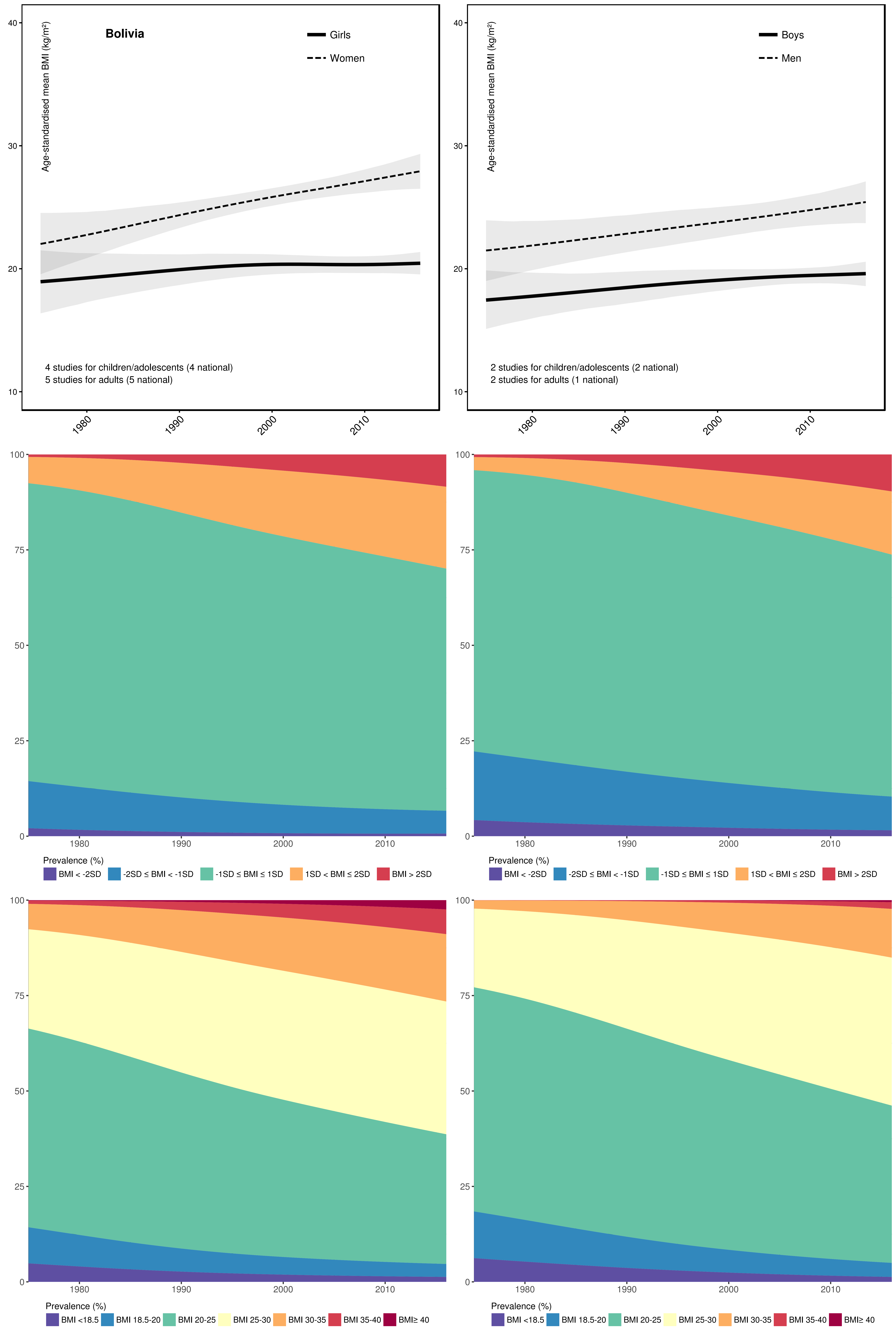
Bolivia
Girls
Women
Boys
Men
Age-standardised mean BMI (kg/m²)
4 studies for children/adolescents (4 national)
5 studies for adults (5 national)
2 studies for children/adolescents (2 national)
2 studies for adults (1 national)
1980
1990
2000
2010
Prevalence (%)
BMI < -2SD
-2SD ≤ BMI < -1SD
-1SD ≤ BMI ≤ 1SD
1SD < BMI ≤ 2SD
BMI > 2SD
BMI <18.5
BMI 18.5-20
BMI 20-25
BMI 25-30
BMI 30-35
BMI 35-40
BMI≥ 40

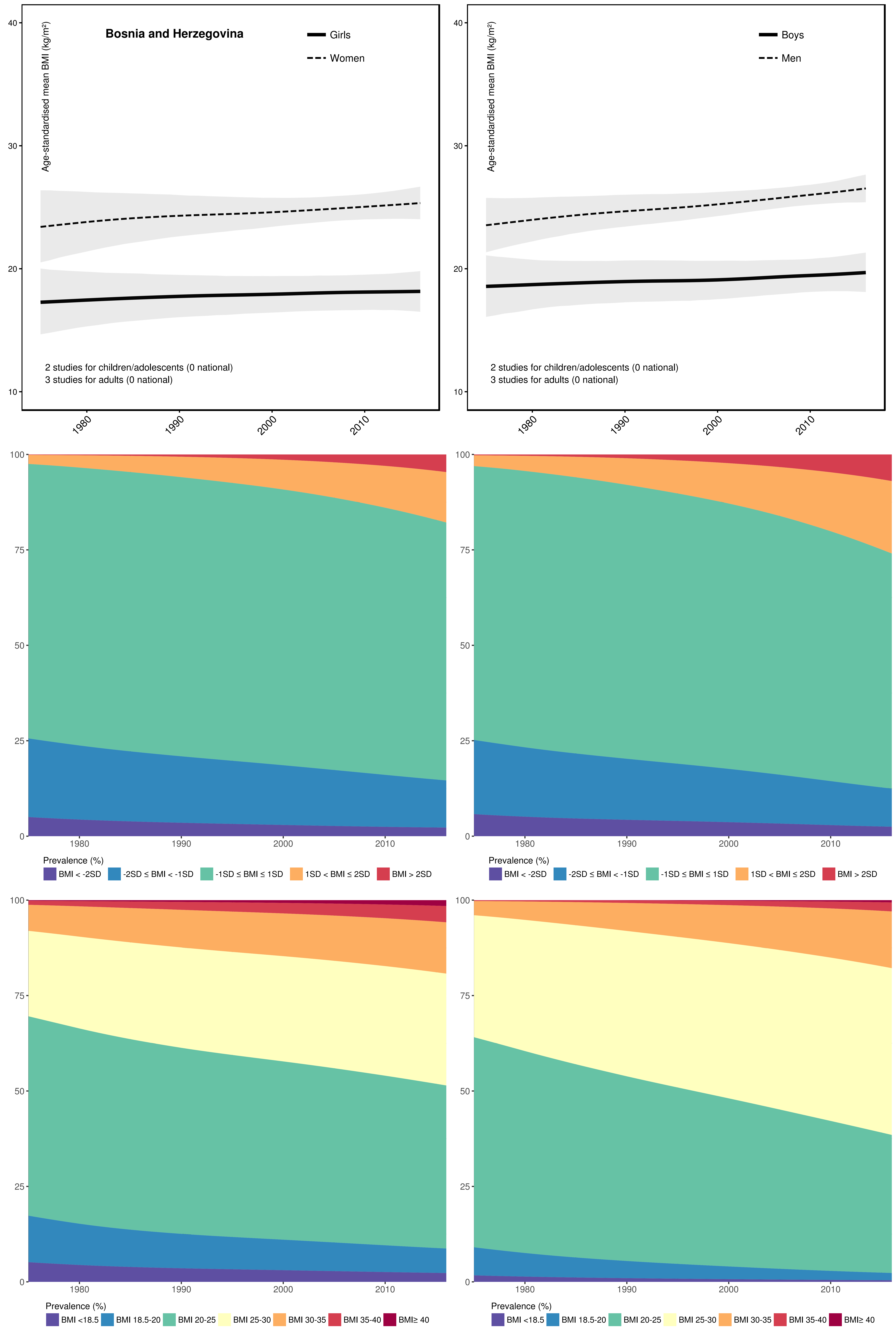
Bosnia and Herzegovina
Girls
Women
Boys
Men
Age-standardised mean BMI (kg/m²)
2 studies for children/adolescents (0 national)
3 studies for adults (0 national)
10
20
30
40
1980
1990
2000
2010
0
25
50
75
100
Prevalence (%)
BMI < -2SD
-2SD ≤ BMI < -1SD
-1SD ≤ BMI ≤ 1SD
1SD < BMI ≤ 2SD
BMI > 2SD
BMI <18.5
BMI 18.5-20
BMI 20-25
BMI 25-30
BMI 30-35
BMI 35-40
BMI≥ 40

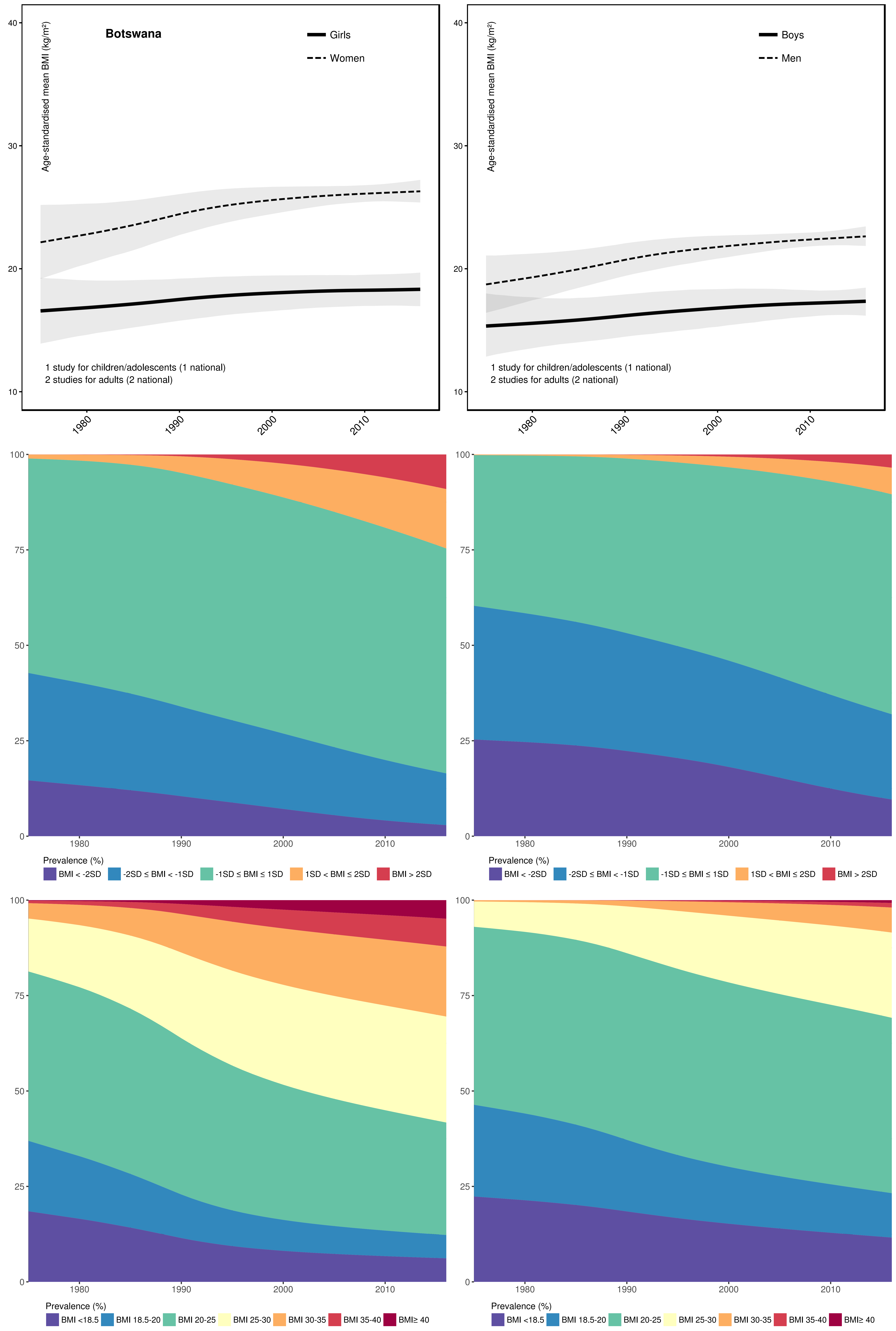
Botswana
Girls
Women
Boys
Men
Age-standardised mean BMI (kg/m²)
1 study for children/adolescents (1 national)
2 studies for adults (2 national)
1980
1990
2000
2010
Prevalence (%)
BMI < -2SD
-2SD ≤ BMI < -1SD
-1SD ≤ BMI ≤ 1SD
1SD < BMI ≤ 2SD
BMI > 2SD
BMI <18.5
BMI 18.5-20
BMI 20-25
BMI 25-30
BMI 30-35
BMI 35-40
BMI≥ 40

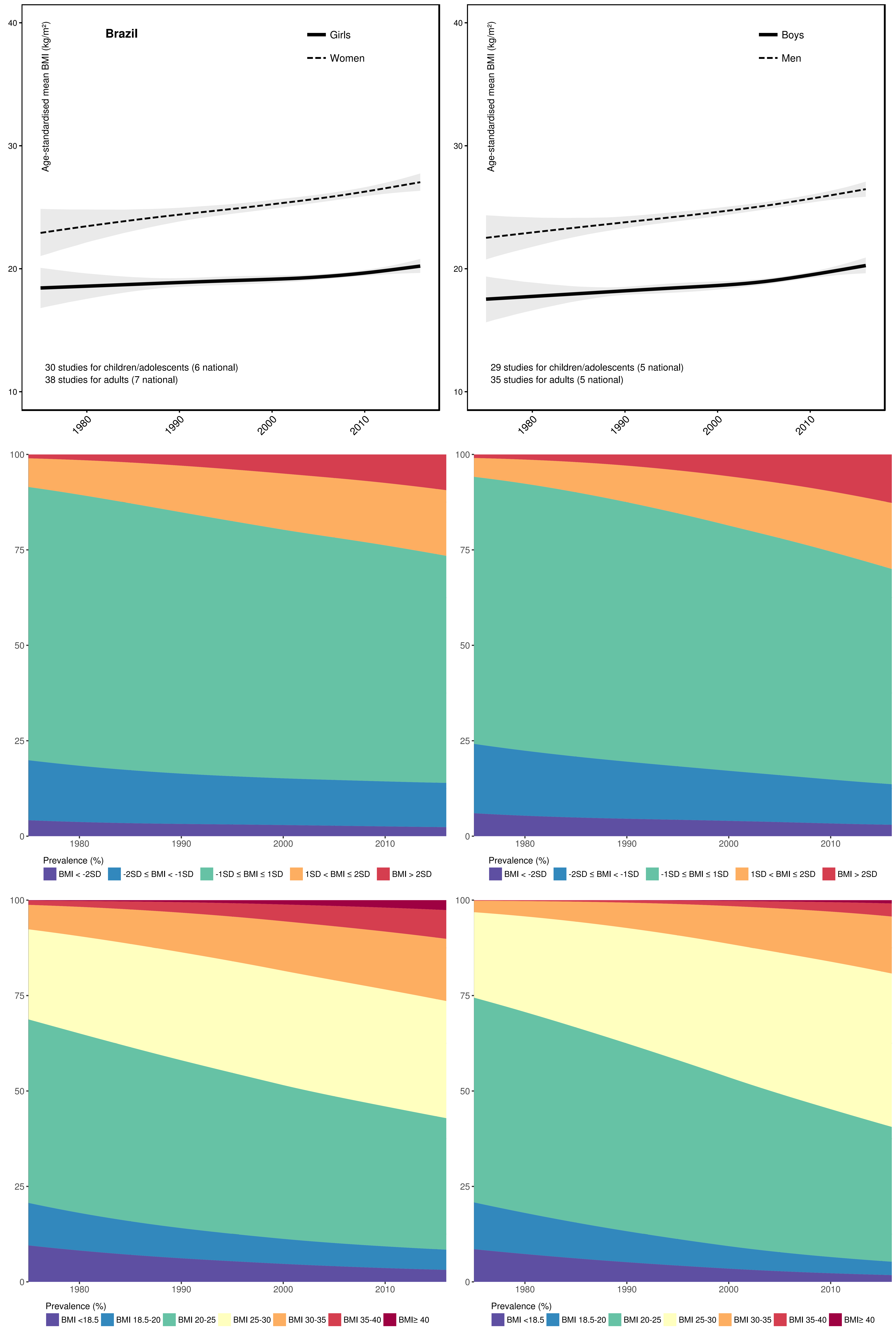
Brazil
Girls
Women
Boys
Men
Age-standardised mean BMI (kg/m²)
30 studies for children/adolescents (6 national)
38 studies for adults (7 national)
29 studies for children/adolescents (5 national)
35 studies for adults (5 national)
Prevalence (%)
BMI < -2SD
-2SD ≤ BMI < -1SD
-1SD ≤ BMI ≤ 1SD
1SD < BMI ≤ 2SD
BMI > 2SD
BMI <18.5
BMI 18.5-20
BMI 20-25
BMI 25-30
BMI 30-35
BMI 35-40
BMI≥ 40

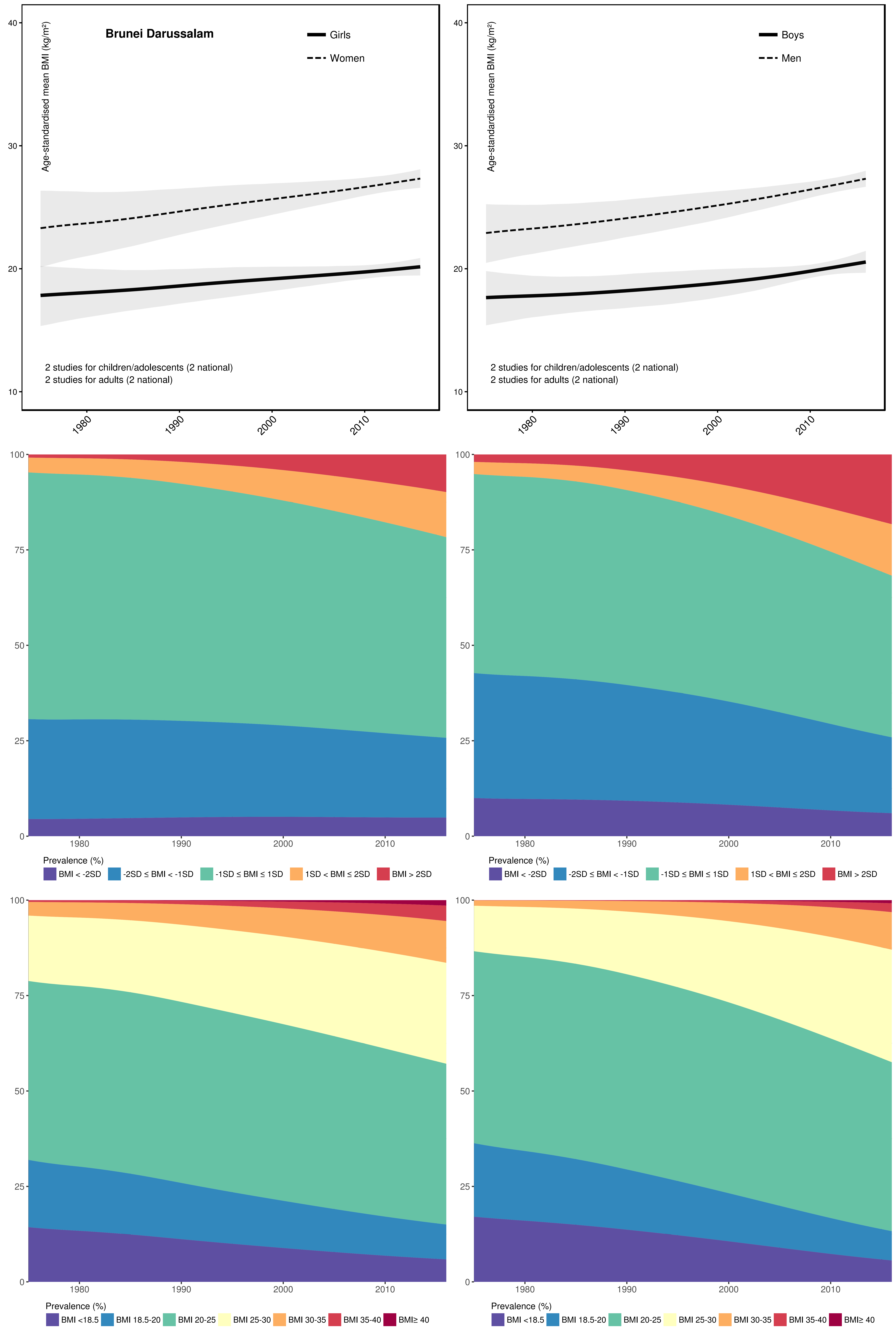
Brunei Darussalam
Girls
Women
Boys
Men
Age-standardised mean BMI (kg/m²)
2 studies for children/adolescents (2 national)
2 studies for adults (2 national)
Prevalence (%)
BMI < -2SD
-2SD ≤ BMI < -1SD
-1SD ≤ BMI ≤ 1SD
1SD < BMI ≤ 2SD
BMI > 2SD
BMI <18.5
BMI 18.5-20
BMI 20-25
BMI 25-30
BMI 30-35
BMI 35-40
BMI≥ 40

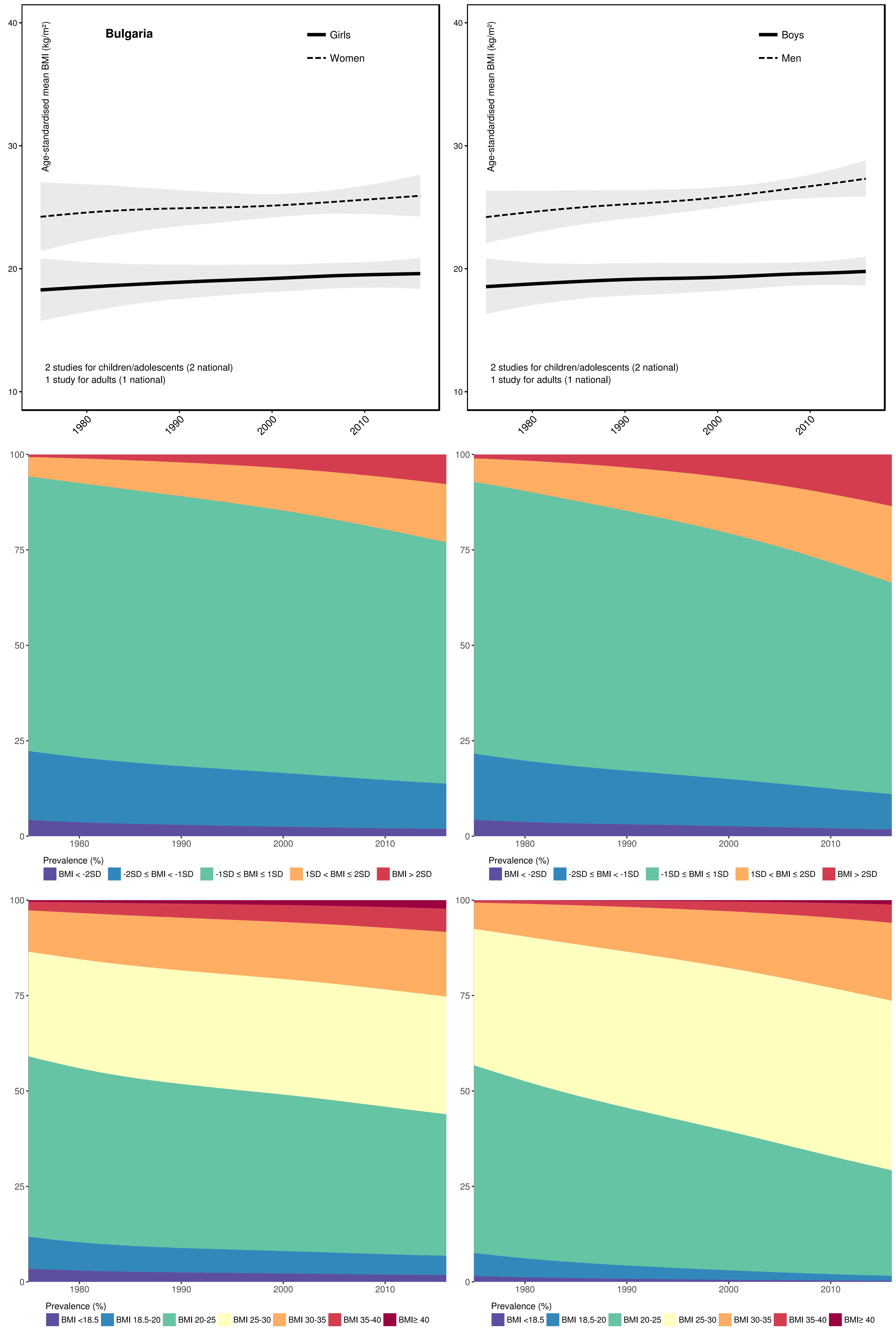
Bulgaria
Girls
Women
Boys
Men
Age-standardised mean BMI (kg/m²)
2 studies for children/adolescents (2 national)
1 study for adults (1 national)
Prevalence (%)
BMI < -2SD
-2SD ≤ BMI < -1SD
-1SD ≤ BMI ≤ 1SD
1SD < BMI ≤ 2SD
BMI > 2SD
BMI <18.5
BMI 18.5-20
BMI 20-25
BMI 25-30
BMI 30-35
BMI 35-40
BMI≥ 40

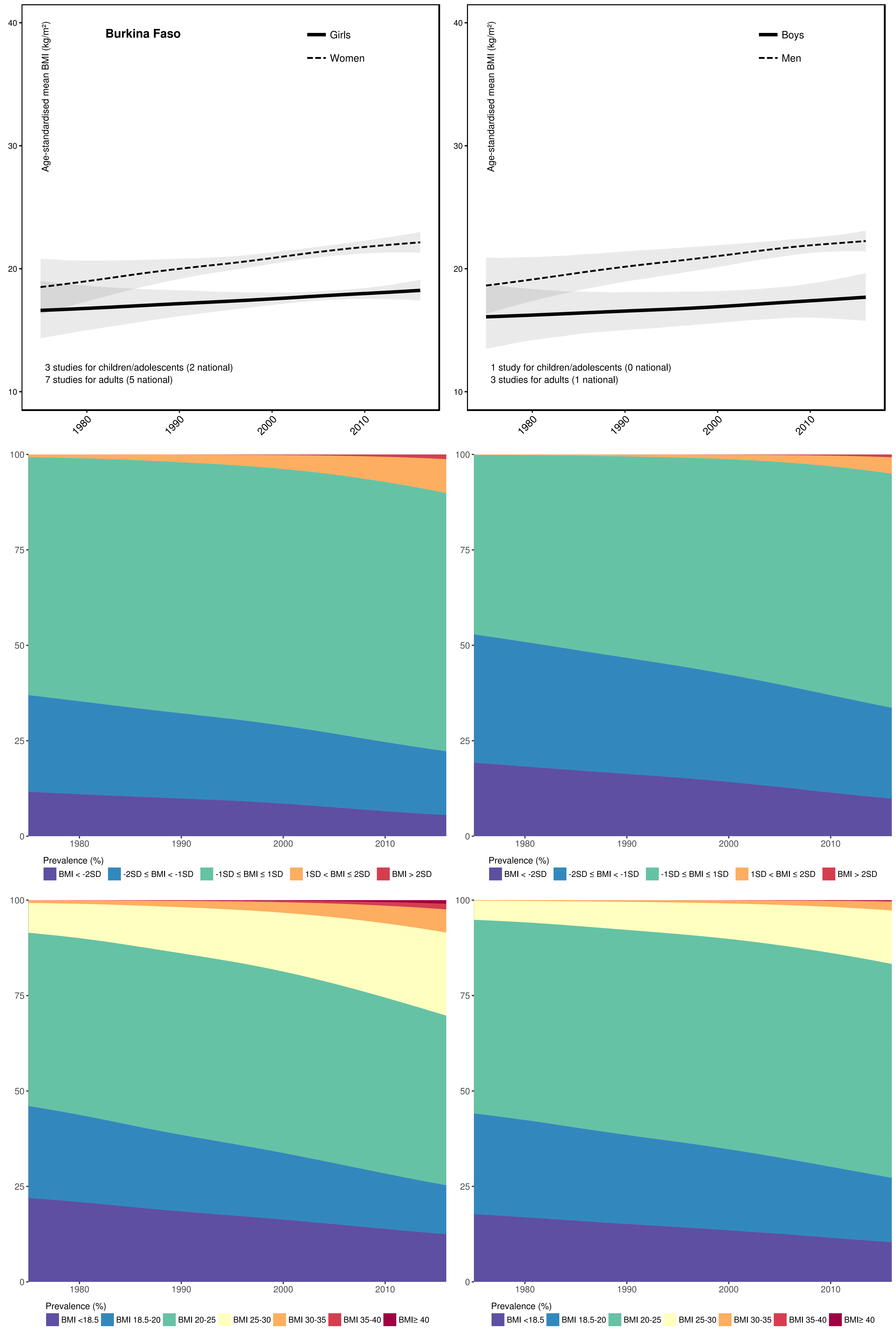
Burkina Faso
Girls
Women
Boys
Men
Age-standardised mean BMI (kg/m²)
3 studies for children/adolescents (2 national)
7 studies for adults (5 national)
1 study for children/adolescents (0 national)
3 studies for adults (1 national)
Prevalence (%)
BMI < -2SD
-2SD ≤ BMI < -1SD
-1SD ≤ BMI ≤ 1SD
1SD < BMI ≤ 2SD
BMI > 2SD
BMI <18.5
BMI 18.5-20
BMI 20-25
BMI 25-30
BMI 30-35
BMI 35-40
BMI≥ 40

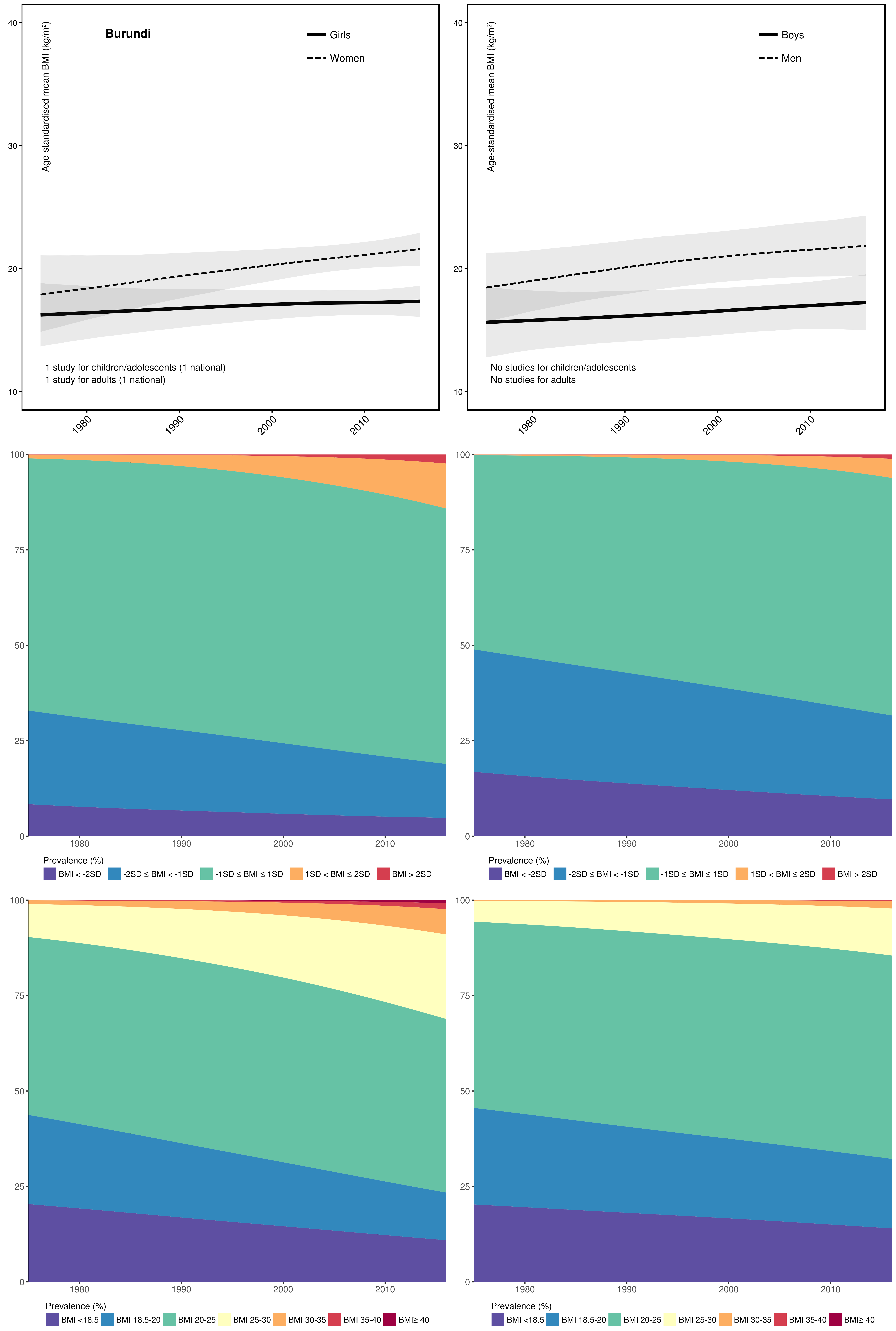

Burundi
Girls
Women
Boys
Men
Age-standardised mean BMI (kg/m²)
1 study for children/adolescents (1 national)
1 study for adults (1 national)
No studies for children/adolescents
No studies for adults
Prevalence (%)
BMI < -2SD
-2SD ≤ BMI < -1SD
-1SD ≤ BMI ≤ 1SD
1SD < BMI ≤ 2SD
BMI > 2SD
BMI <18.5
BMI 18.5-20
BMI 20-25
BMI 25-30
BMI 30-35
BMI 35-40
BMI≥ 40

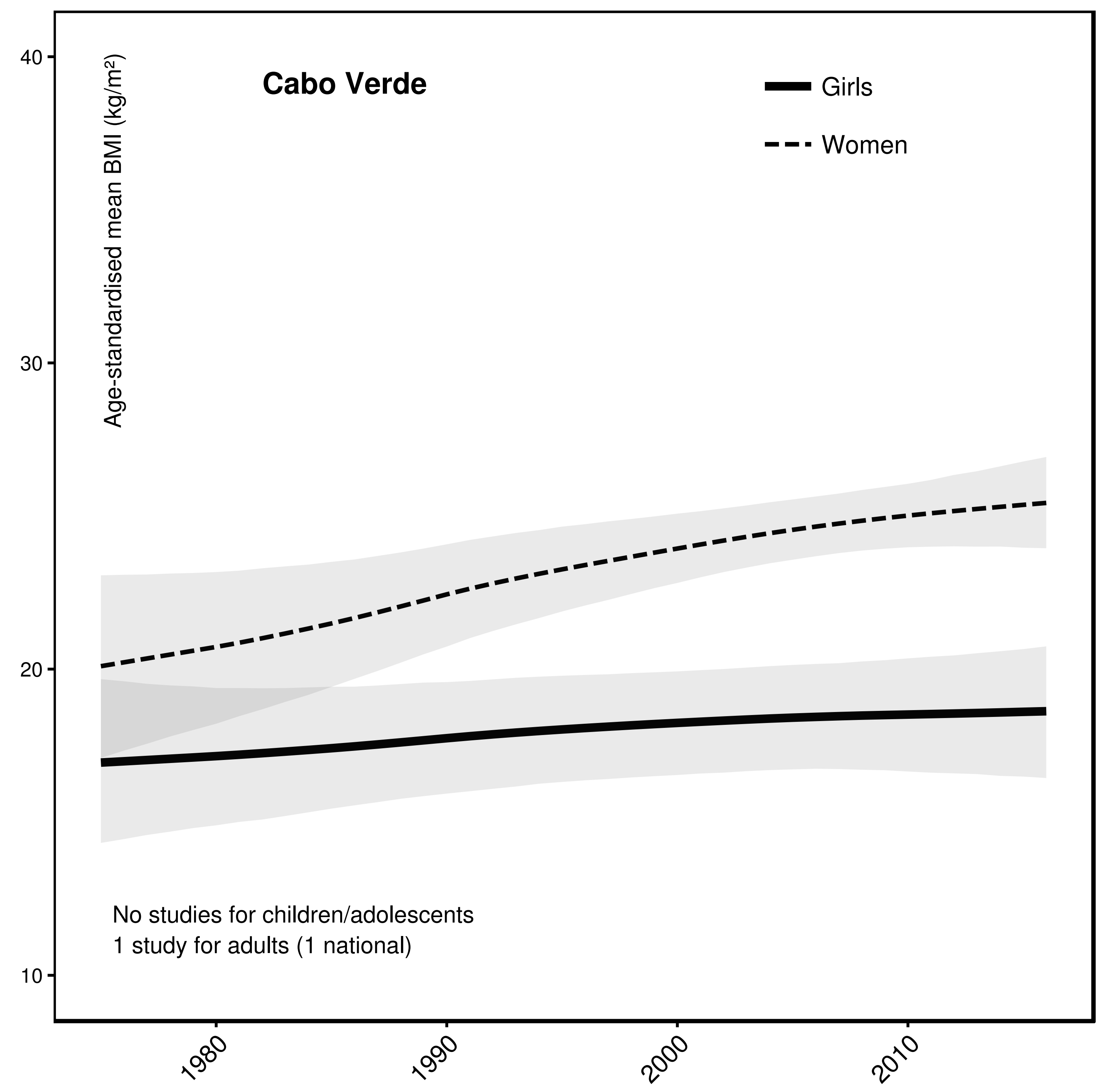
Cabo Verde
Girls
Women
Age-standardised mean BMI (kg/m²)
40
30
20
10
No studies for children/adolescents
1 study for adults (1 national)
1980
1990
2000
2010

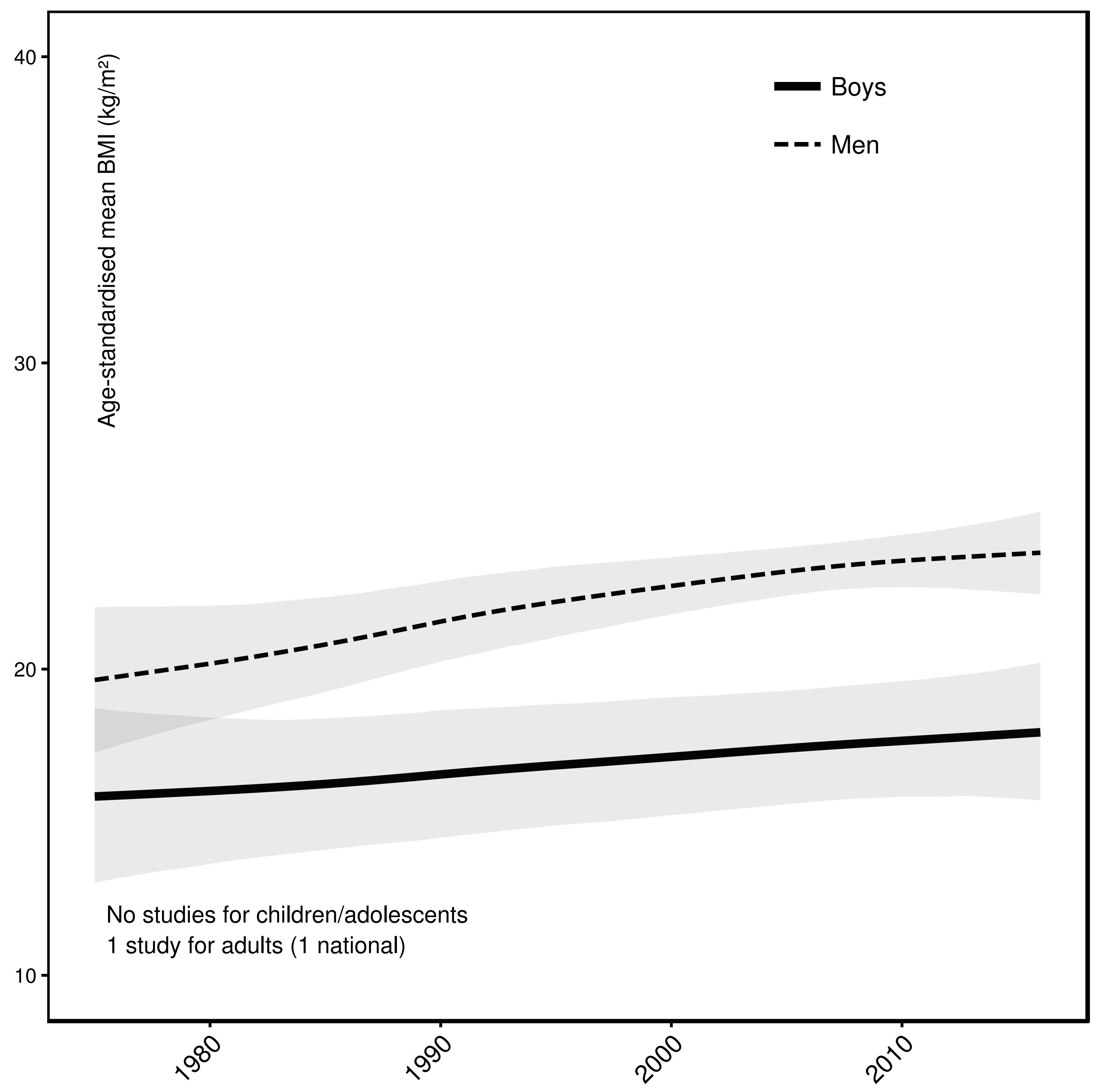
Boys
Men
Age-standardised mean BMI (kg/m²)
40
30
20
10
No studies for children/adolescents
1 study for adults (1 national)
1980
1990
2000
2010

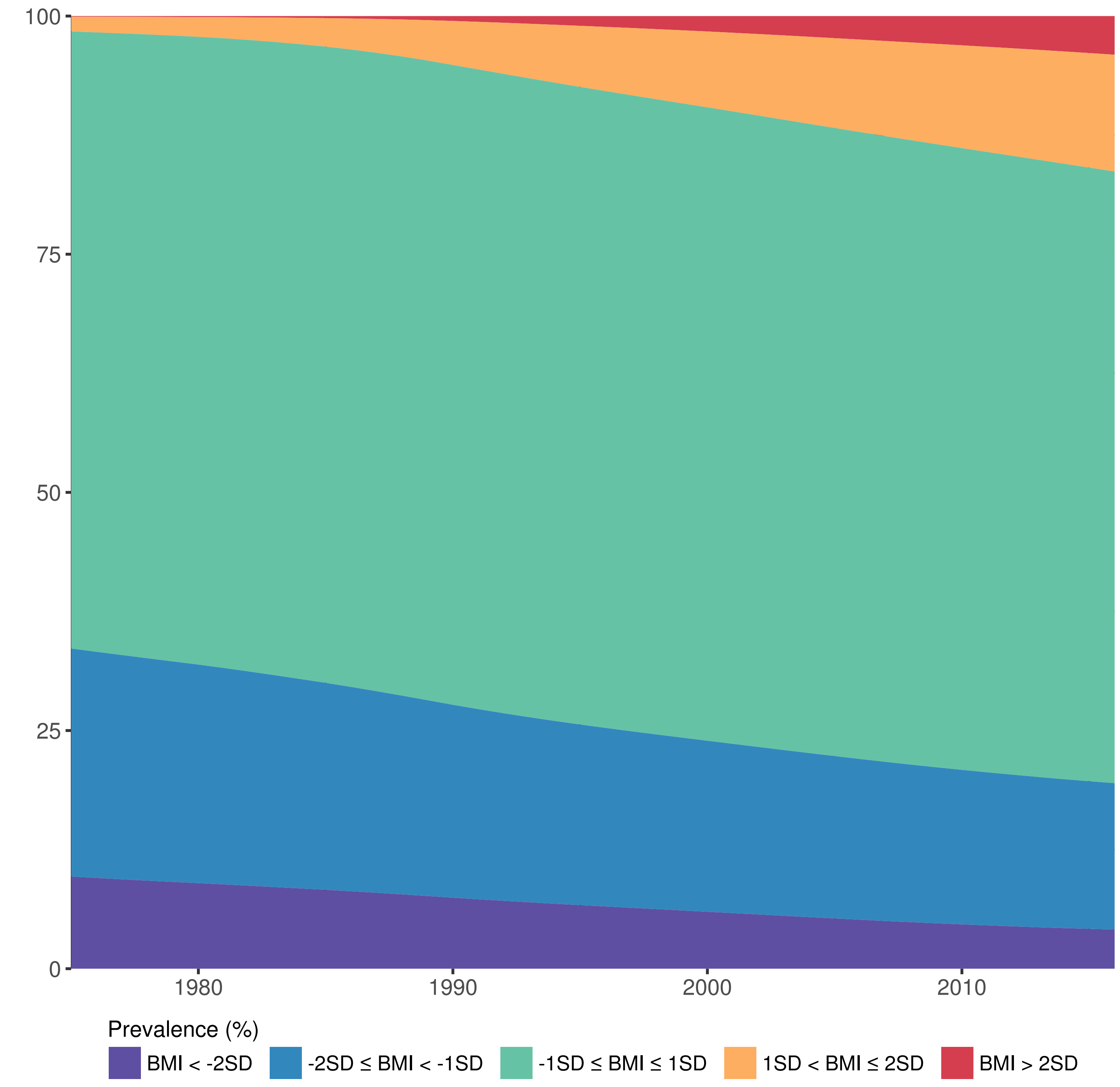
100
75
50
25
0
1980
1990
2000
2010
Prevalence (%)
BMI < -2SD
-2SD ≤ BMI < -1SD
-1SD ≤ BMI ≤ 1SD
1SD < BMI ≤ 2SD
BMI > 2SD

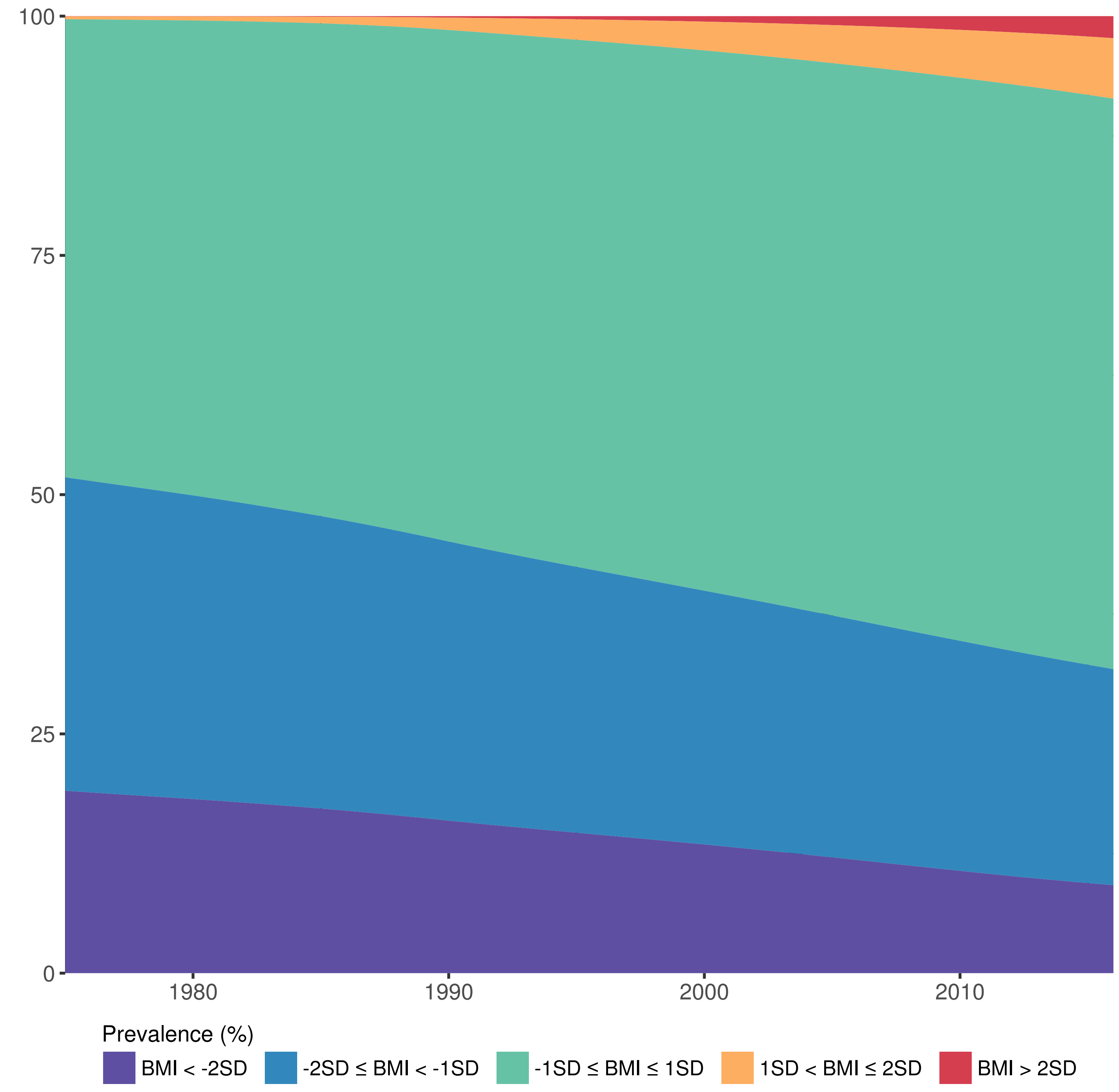
100
75
50
25
0
1980
1990
2000
2010
Prevalence (%)
BMI < -2SD
-2SD ≤ BMI < -1SD
-1SD ≤ BMI ≤ 1SD
1SD < BMI ≤ 2SD
BMI > 2SD

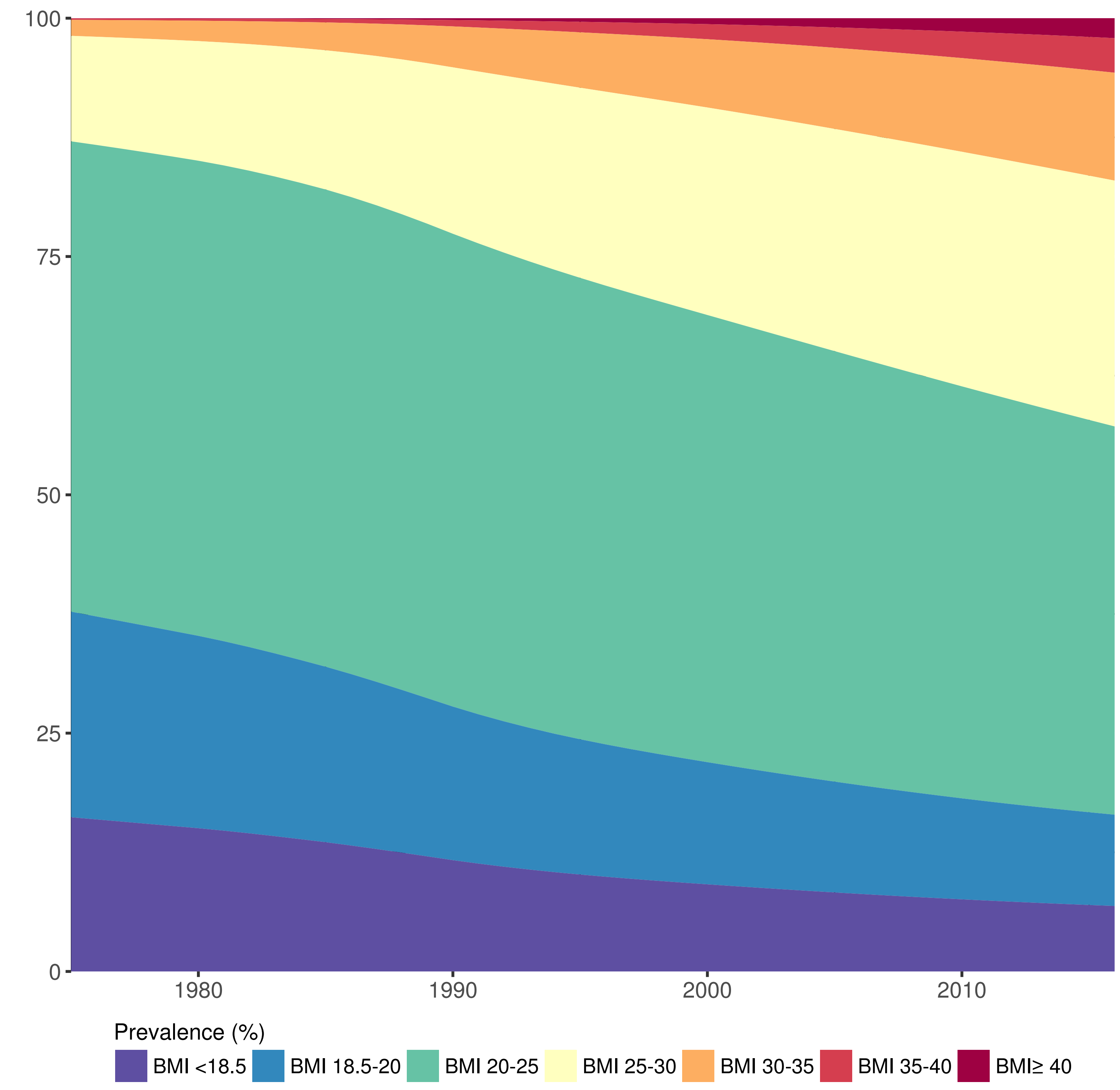
100
75
50
25
0
1980
1990
2000
2010
Prevalence (%)
BMI <18.5
BMI 18.5-20
BMI 20-25
BMI 25-30
BMI 30-35
BMI 35-40
BMI≥ 40

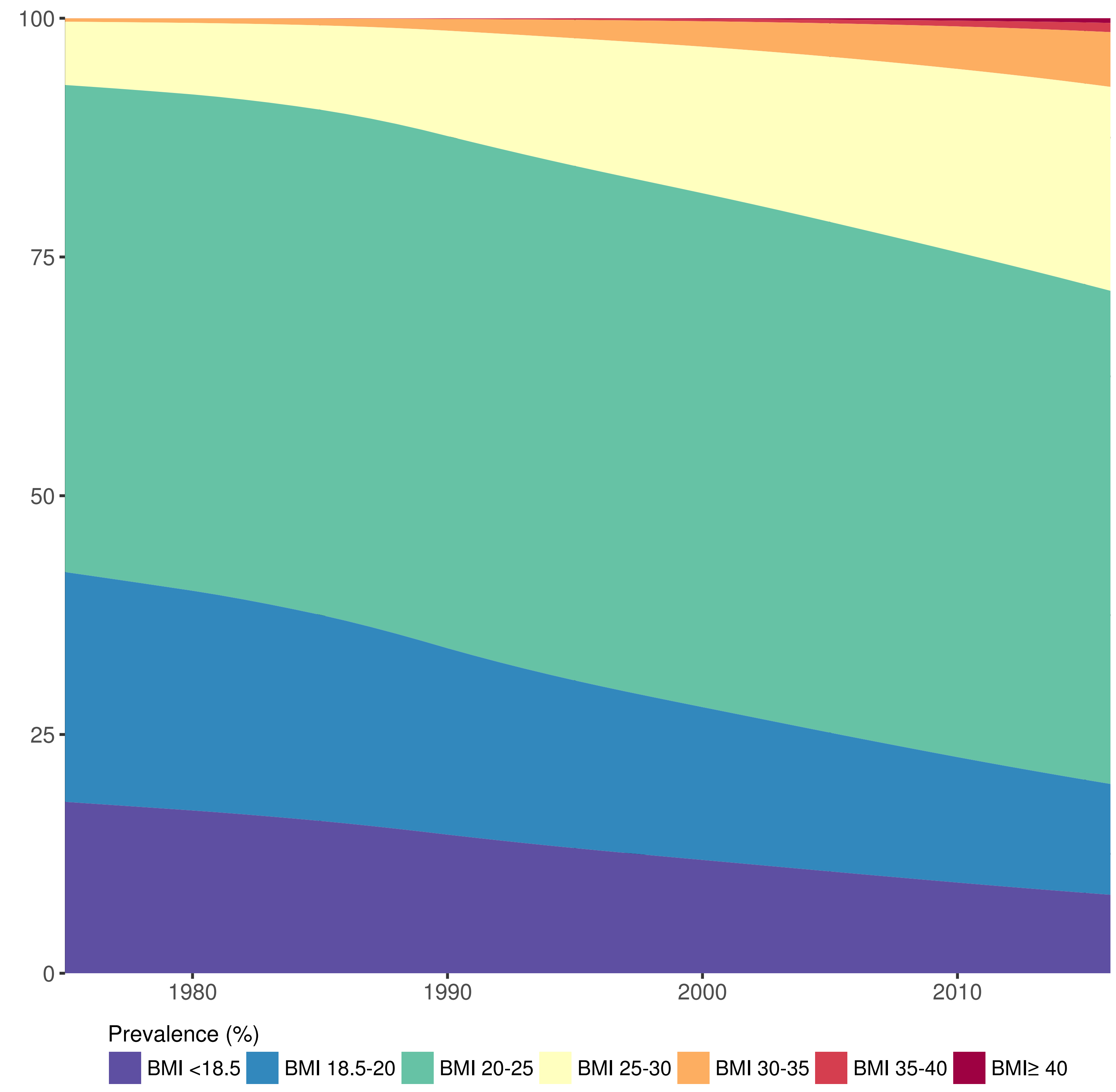
100
75
50
25
0
1980
1990
2000
2010
Prevalence (%)
BMI <18.5
BMI 18.5-20
BMI 20-25
BMI 25-30
BMI 30-35
BMI 35-40
BMI≥ 40

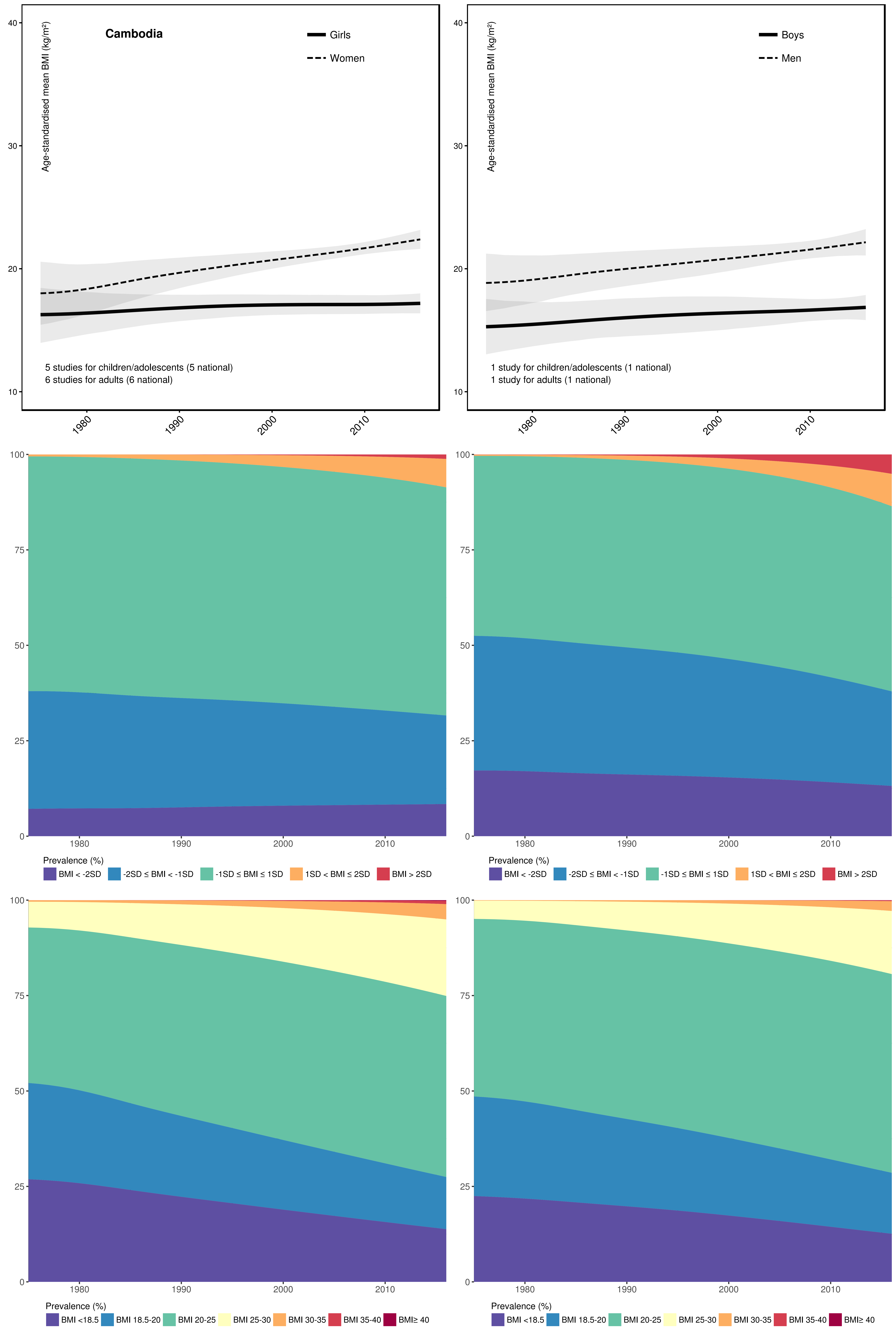
Cambodia
Girls
Women
Boys
Men
Age-standardised mean BMI (kg/m²)
5 studies for children/adolescents (5 national)
6 studies for adults (6 national)
1 study for children/adolescents (1 national)
1 study for adults (1 national)
Prevalence (%)
BMI < -2SD
-2SD ≤ BMI < -1SD
-1SD ≤ BMI ≤ 1SD
1SD < BMI ≤ 2SD
BMI > 2SD
BMI <18.5
BMI 18.5-20
BMI 20-25
BMI 25-30
BMI 30-35
BMI 35-40
BMI≥ 40

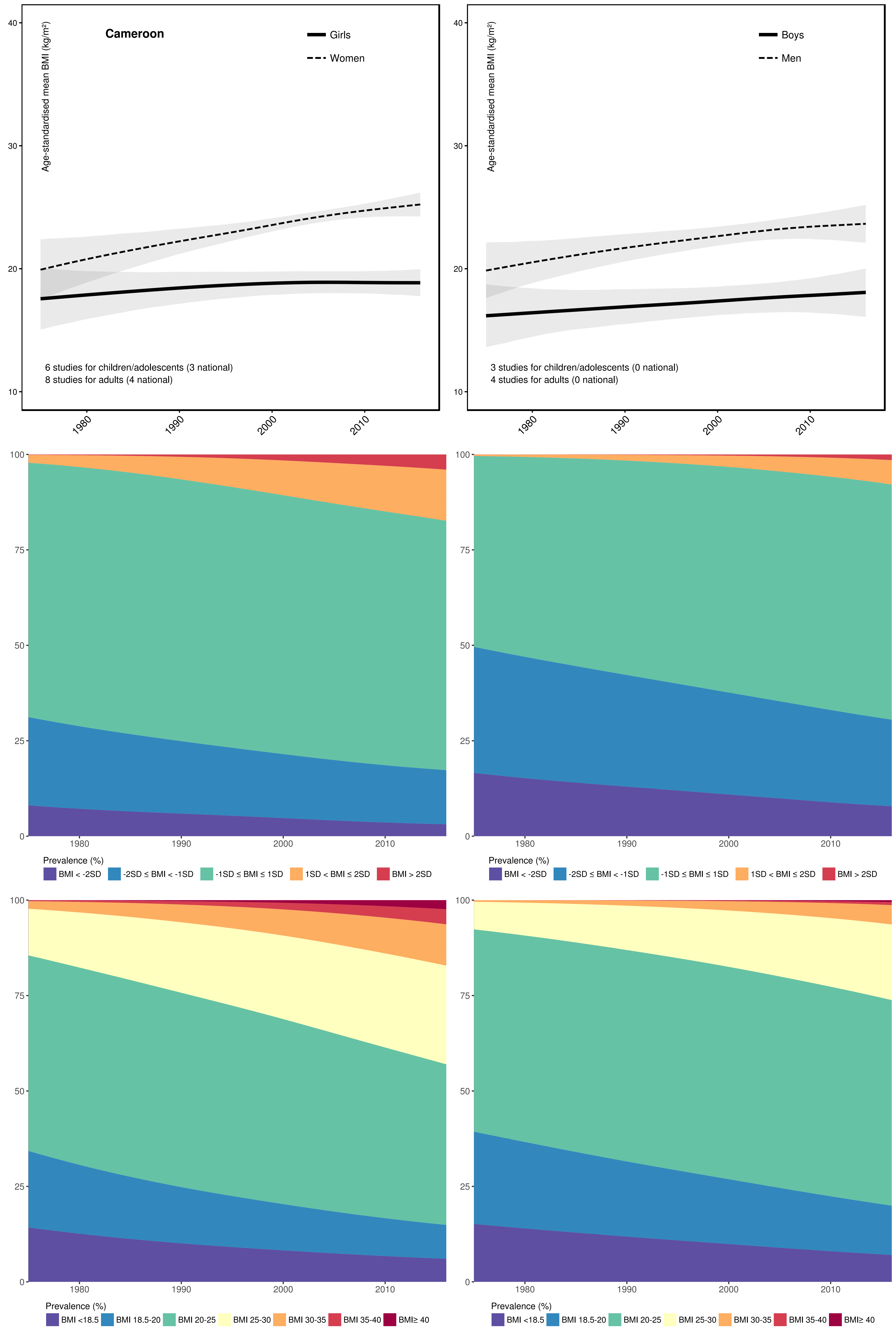
Cameroon
Girls
Women
Boys
Men
Age-standardised mean BMI (kg/m²)
6 studies for children/adolescents (3 national)
8 studies for adults (4 national)
3 studies for children/adolescents (0 national)
4 studies for adults (0 national)
Prevalence (%)
BMI < -2SD
-2SD ≤ BMI < -1SD
-1SD ≤ BMI ≤ 1SD
1SD < BMI ≤ 2SD
BMI > 2SD
BMI <18.5
BMI 18.5-20
BMI 20-25
BMI 25-30
BMI 30-35
BMI 35-40
BMI≥ 40

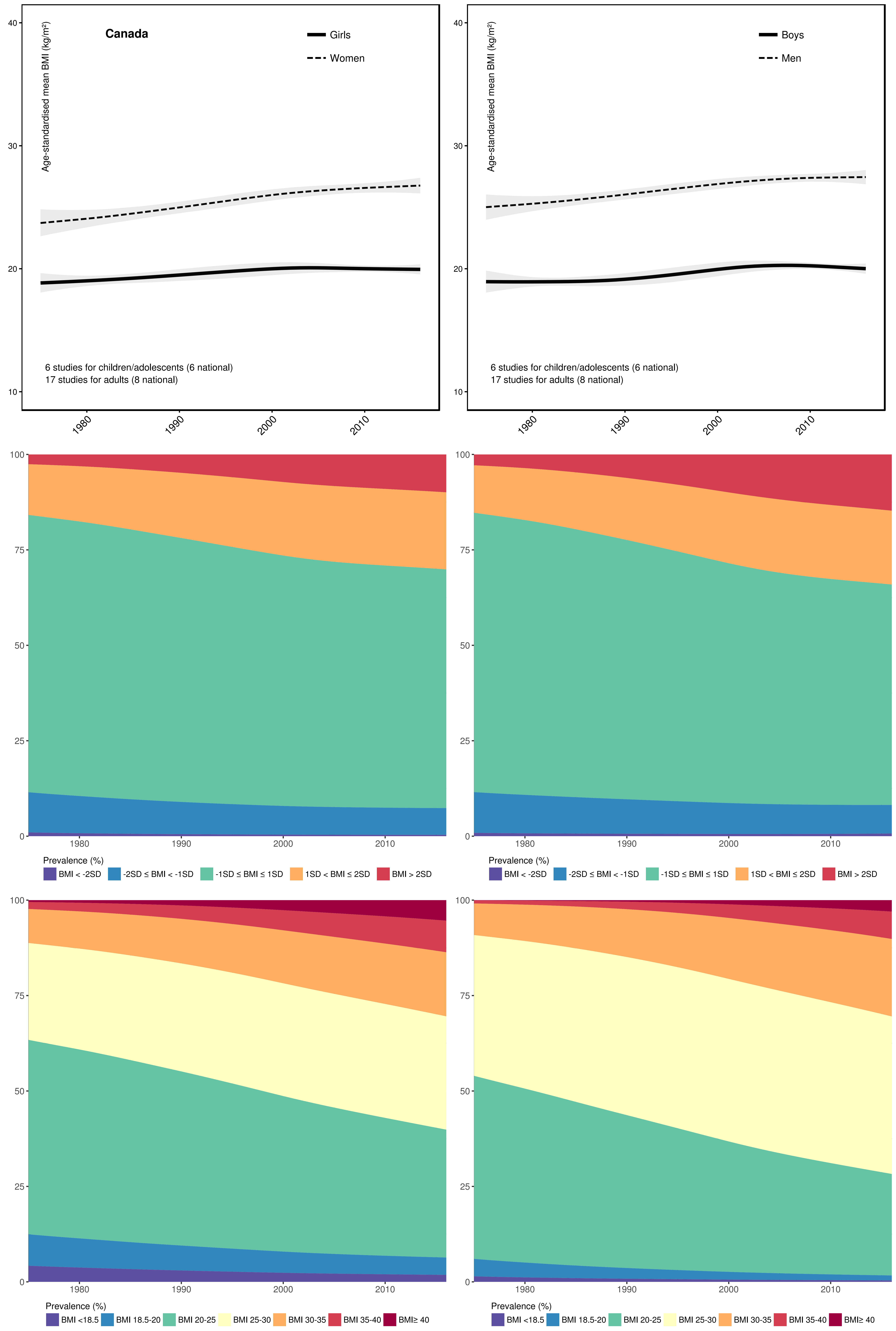
Canada
Girls
Women
Boys
Men
Age-standardised mean BMI (kg/m²)
6 studies for children/adolescents (6 national)
17 studies for adults (8 national)
Prevalence (%)
BMI < -2SD
-2SD ≤ BMI < -1SD
-1SD ≤ BMI ≤ 1SD
1SD < BMI ≤ 2SD
BMI > 2SD
BMI <18.5
BMI 18.5-20
BMI 20-25
BMI 25-30
BMI 30-35
BMI 35-40
BMI≥ 40

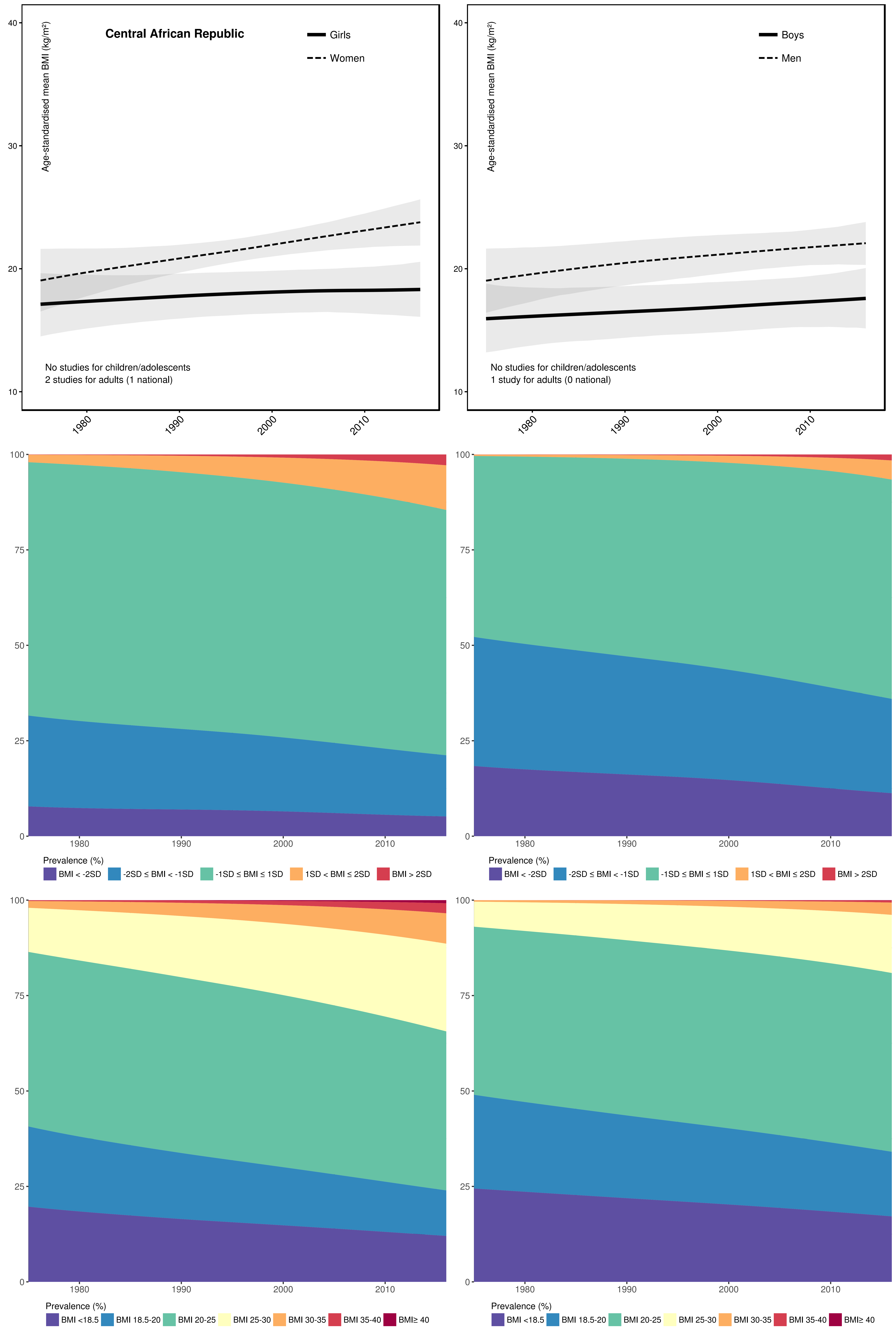
Central African Republic
Girls
Women
Age-standardised mean BMI (kg/m²)
No studies for children/adolescents
2 studies for adults (1 national)
Boys
Men
No studies for children/adolescents
1 study for adults (0 national)
Prevalence (%)
BMI < -2SD
-2SD ≤ BMI < -1SD
-1SD ≤ BMI ≤ 1SD
1SD < BMI ≤ 2SD
BMI > 2SD
BMI <18.5
BMI 18.5-20
BMI 20-25
BMI 25-30
BMI 30-35
BMI 35-40
BMI≥ 40

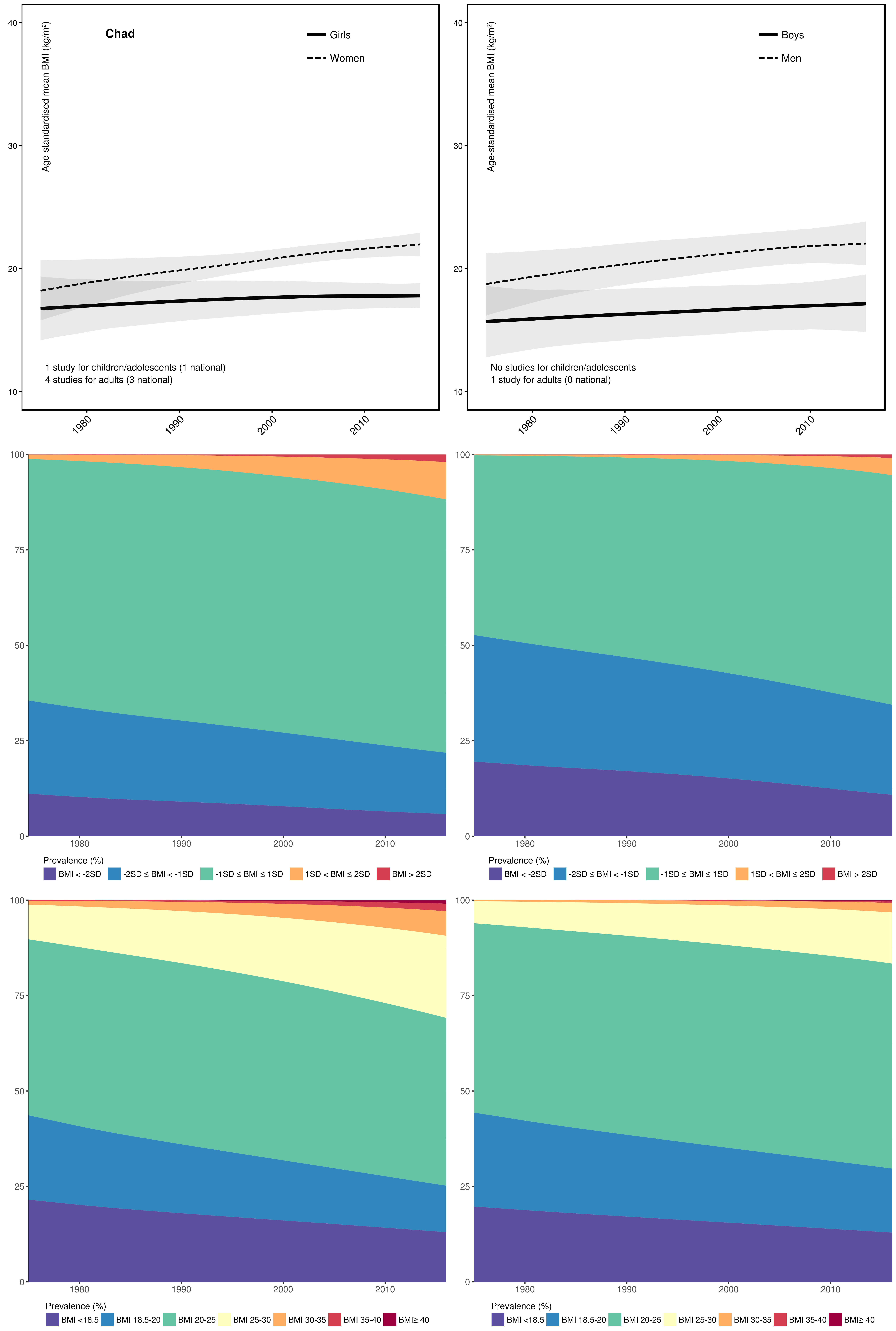

Chad
Girls
Women
Age-standardised mean BMI (kg/m²)
1 study for children/adolescents (1 national)
4 studies for adults (3 national)
Boys
Men
Age-standardised mean BMI (kg/m²)
No studies for children/adolescents
1 study for adults (0 national)
Prevalence (%)
BMI < -2SD
-2SD ≤ BMI < -1SD
-1SD ≤ BMI ≤ 1SD
1SD < BMI ≤ 2SD
BMI > 2SD
Prevalence (%)
BMI <18.5
BMI 18.5-20
BMI 20-25
BMI 25-30
BMI 30-35
BMI 35-40
BMI≥ 40

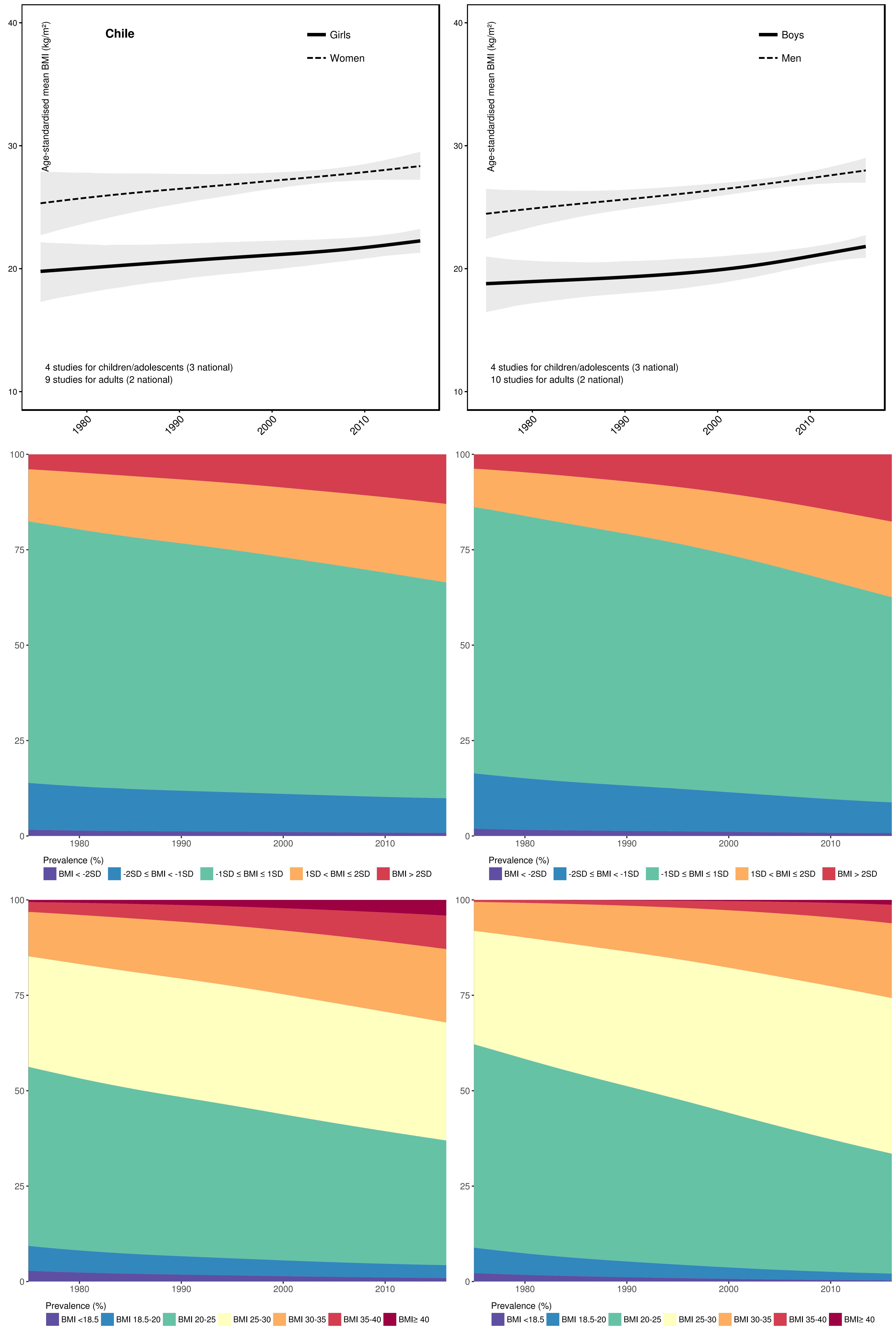
Chile
Girls
Women
Age-standardised mean BMI (kg/m²)
4 studies for children/adolescents (3 national)
9 studies for adults (2 national)
Boys
Men
Age-standardised mean BMI (kg/m²)
4 studies for children/adolescents (3 national)
10 studies for adults (2 national)
Prevalence (%)
BMI < -2SD
-2SD ≤ BMI < -1SD
-1SD ≤ BMI ≤ 1SD
1SD < BMI ≤ 2SD
BMI > 2SD
Prevalence (%)
BMI <18.5
BMI 18.5-20
BMI 20-25
BMI 25-30
BMI 30-35
BMI 35-40
BMI≥ 40

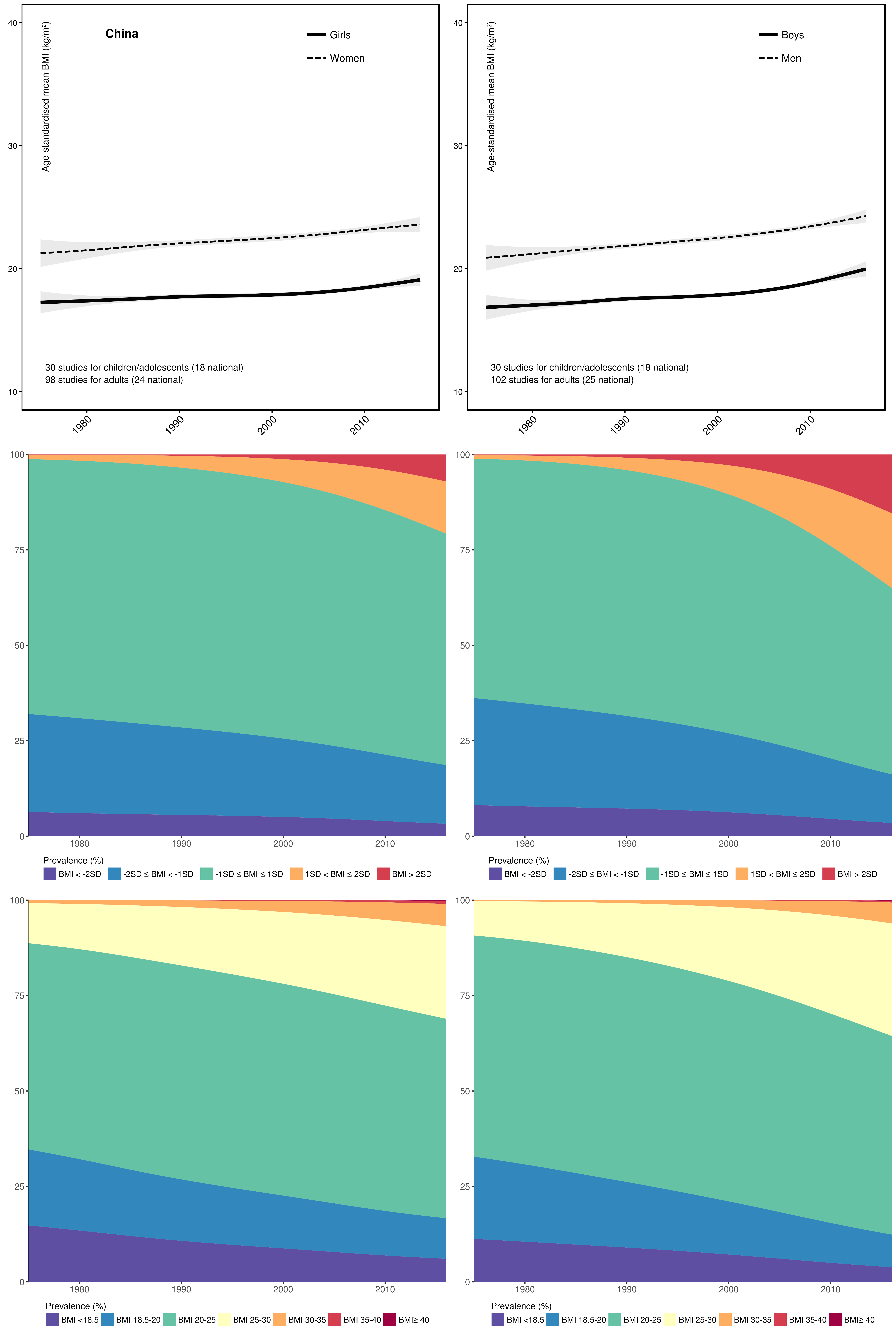
China
Girls
Women
Boys
Men
Age-standardised mean BMI (kg/m²)
30 studies for children/adolescents (18 national)
98 studies for adults (24 national)
30 studies for children/adolescents (18 national)
102 studies for adults (25 national)
1980
1990
2000
2010
Prevalence (%)
BMI < -2SD
-2SD ≤ BMI < -1SD
-1SD ≤ BMI ≤ 1SD
1SD < BMI ≤ 2SD
BMI > 2SD
BMI <18.5
BMI 18.5-20
BMI 20-25
BMI 25-30
BMI 30-35
BMI 35-40
BMI≥ 40

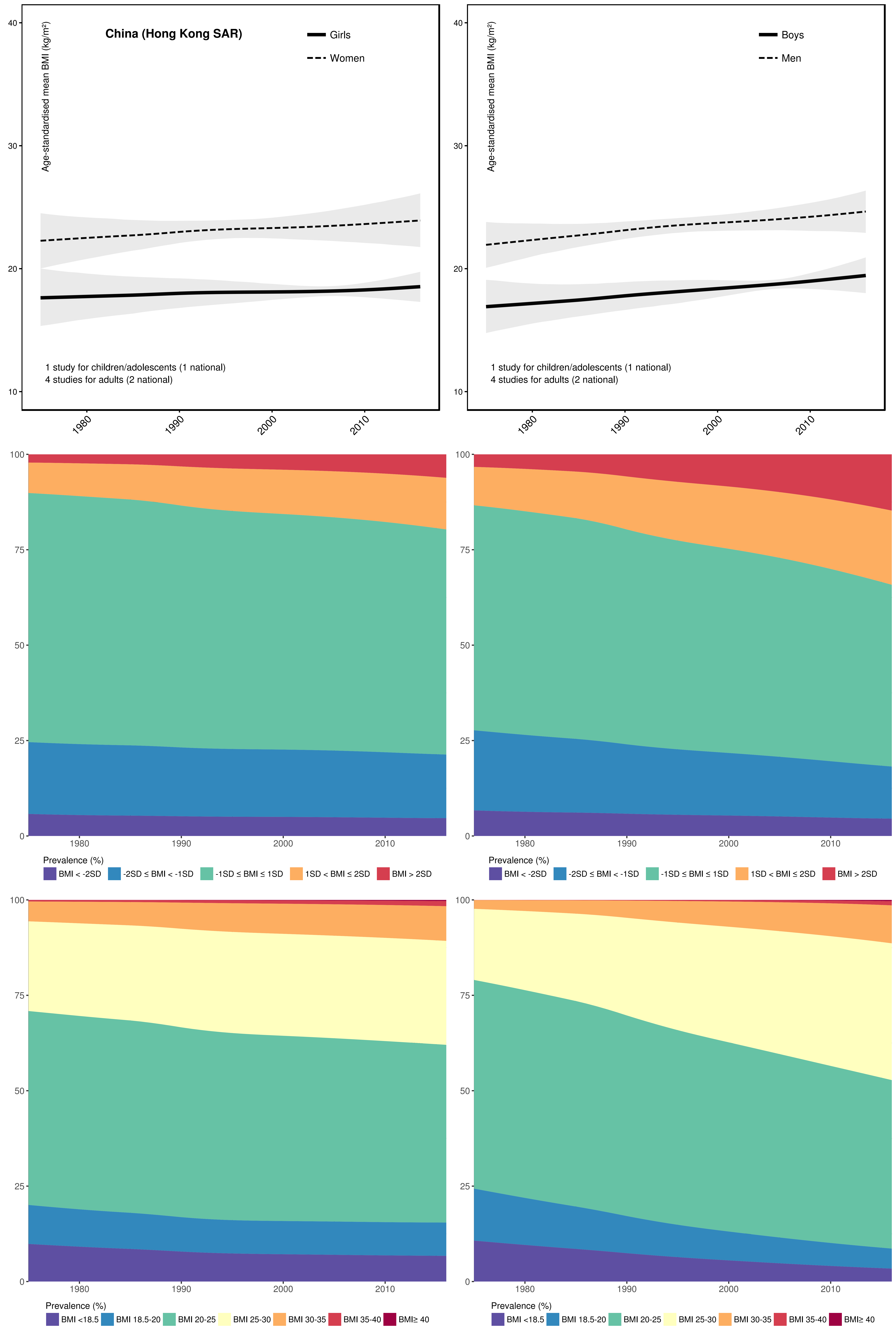
China (Hong Kong SAR)
Girls
Women
Boys
Men
Age-standardised mean BMI (kg/m²)
1 study for children/adolescents (1 national)
4 studies for adults (2 national)
Prevalence (%)
BMI < -2SD
-2SD ≤ BMI < -1SD
-1SD ≤ BMI ≤ 1SD
1SD < BMI ≤ 2SD
BMI > 2SD
BMI <18.5
BMI 18.5-20
BMI 20-25
BMI 25-30
BMI 30-35
BMI 35-40
BMI≥ 40

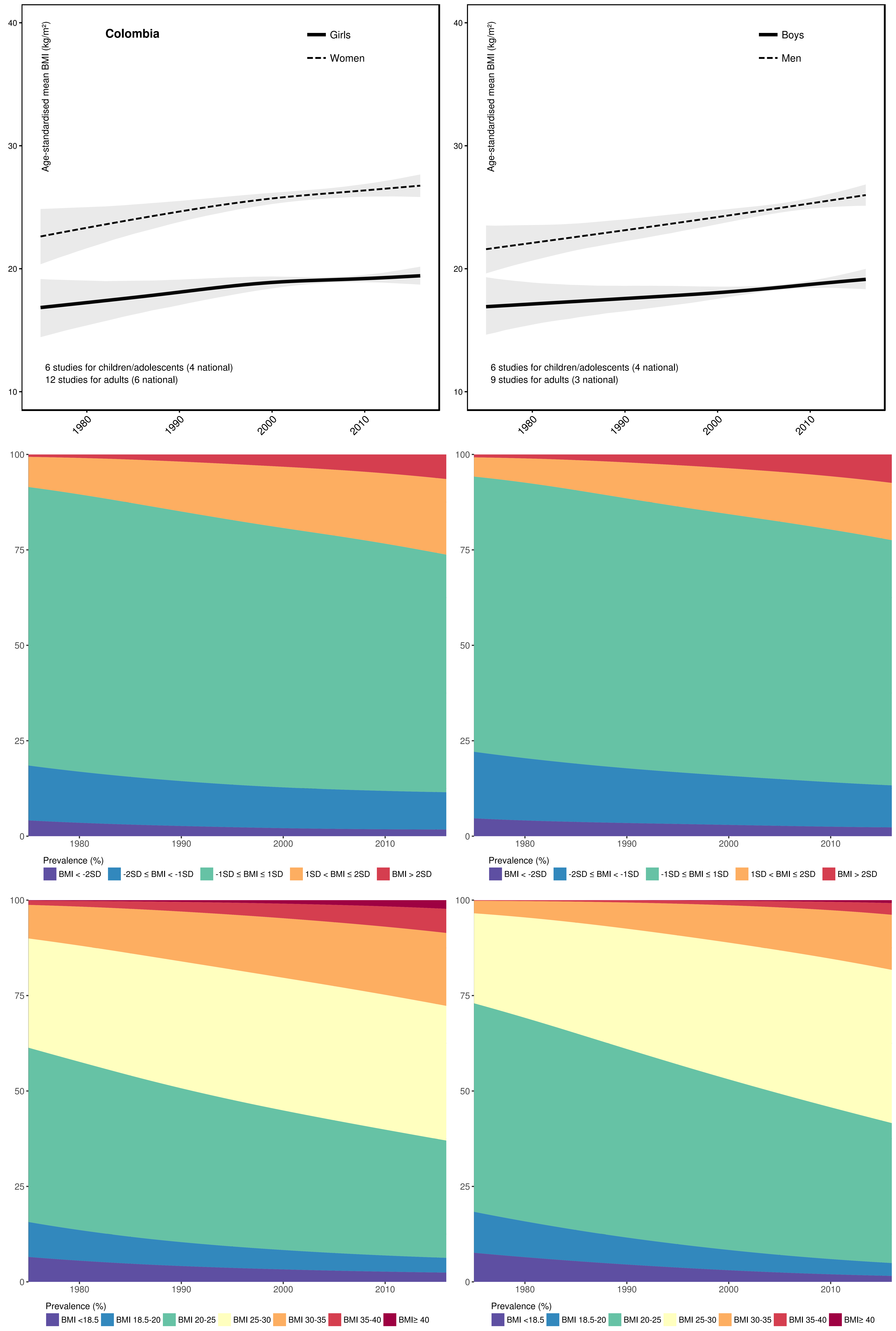
Colombia
Girls
Women
Boys
Men
Age-standardised mean BMI (kg/m²)
6 studies for children/adolescents (4 national)
12 studies for adults (6 national)
6 studies for children/adolescents (4 national)
9 studies for adults (3 national)
Prevalence (%)
BMI < -2SD
-2SD ≤ BMI < -1SD
-1SD ≤ BMI ≤ 1SD
1SD < BMI ≤ 2SD
BMI > 2SD
BMI <18.5
BMI 18.5-20
BMI 20-25
BMI 25-30
BMI 30-35
BMI 35-40
BMI≥ 40

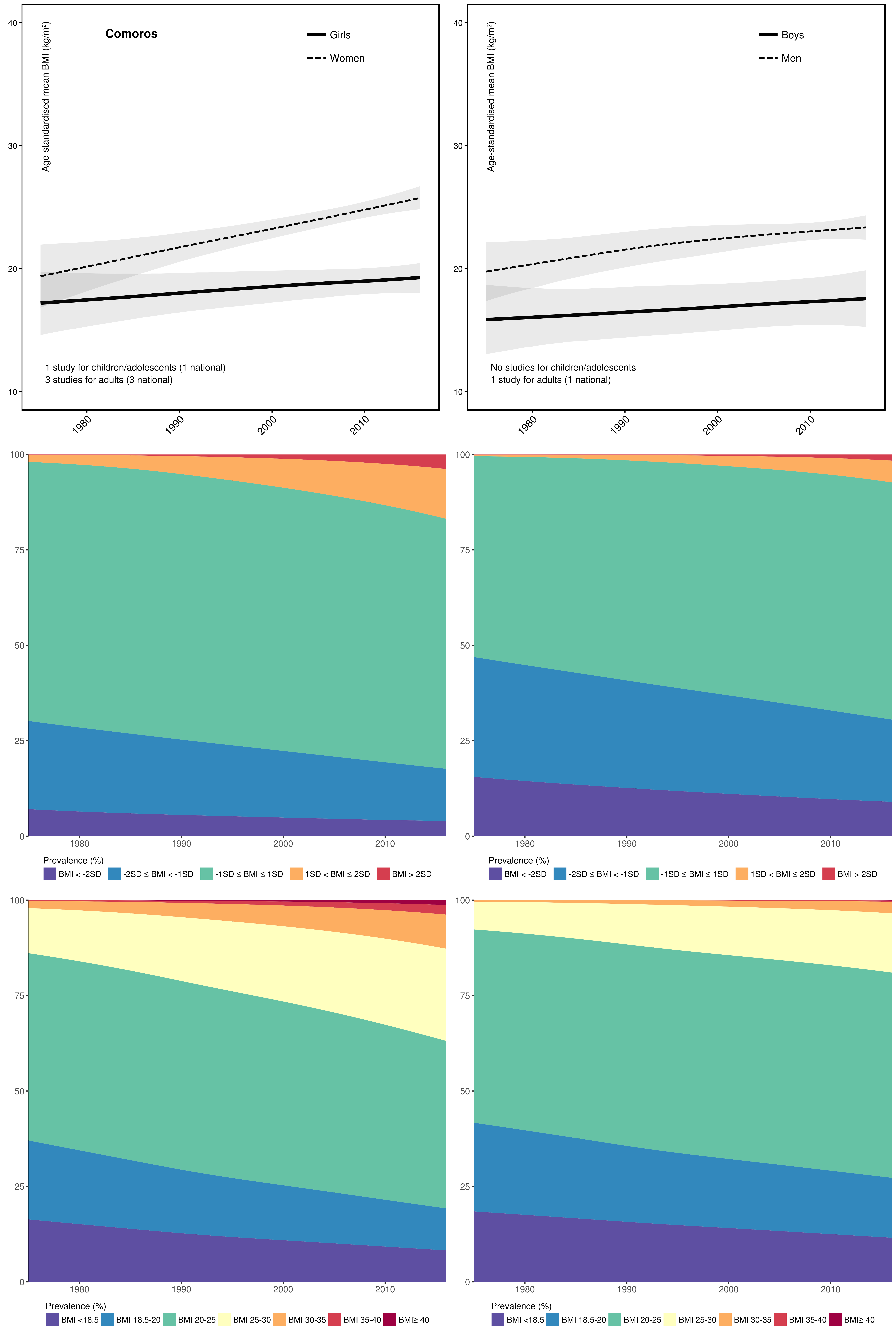
Comoros
Girls
Women
Boys
Men
Age-standardised mean BMI (kg/m²)
1 study for children/adolescents (1 national)
3 studies for adults (3 national)
No studies for children/adolescents
1 study for adults (1 national)
Prevalence (%)
BMI < -2SD
-2SD ≤ BMI < -1SD
-1SD ≤ BMI ≤ 1SD
1SD < BMI ≤ 2SD
BMI > 2SD
BMI <18.5
BMI 18.5-20
BMI 20-25
BMI 25-30
BMI 30-35
BMI 35-40
BMI≥ 40

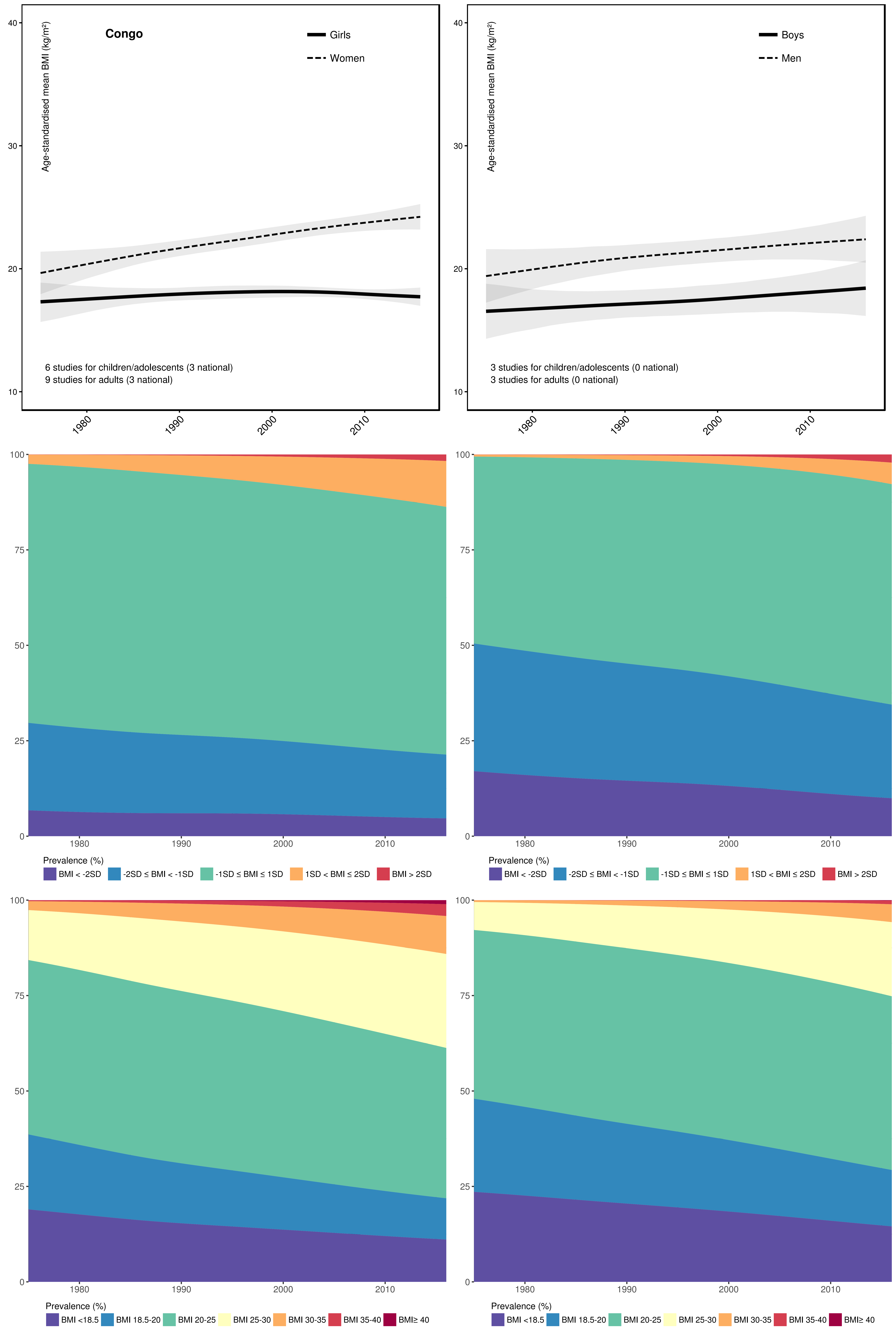
Congo
Girls
Women
Boys
Men
Age-standardised mean BMI (kg/m²)
6 studies for children/adolescents (3 national)
9 studies for adults (3 national)
3 studies for children/adolescents (0 national)
3 studies for adults (0 national)
Prevalence (%)
BMI < -2SD
-2SD ≤ BMI < -1SD
-1SD ≤ BMI ≤ 1SD
1SD < BMI ≤ 2SD
BMI > 2SD
BMI <18.5
BMI 18.5-20
BMI 20-25
BMI 25-30
BMI 30-35
BMI 35-40
BMI≥ 40

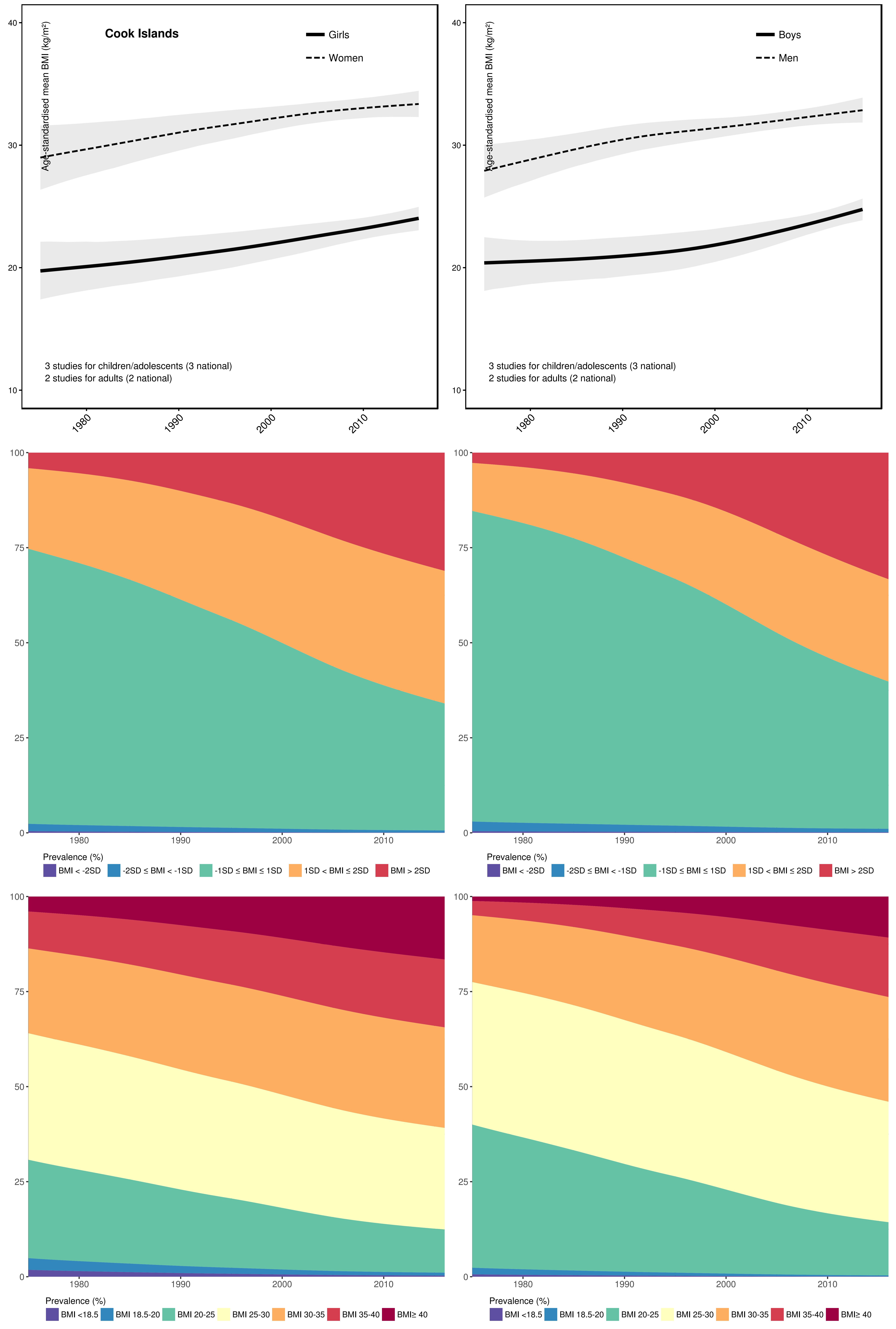
Cook Islands
Girls
Women
Boys
Men
Age-standardised mean BMI (kg/m²)
3 studies for children/adolescents (3 national)
2 studies for adults (2 national)
3 studies for children/adolescents (3 national)
2 studies for adults (2 national)
Prevalence (%)
BMI < -2SD
-2SD ≤ BMI < -1SD
-1SD ≤ BMI ≤ 1SD
1SD < BMI ≤ 2SD
BMI > 2SD
Prevalence (%)
BMI <18.5
BMI 18.5-20
BMI 20-25
BMI 25-30
BMI 30-35
BMI 35-40
BMI≥ 40

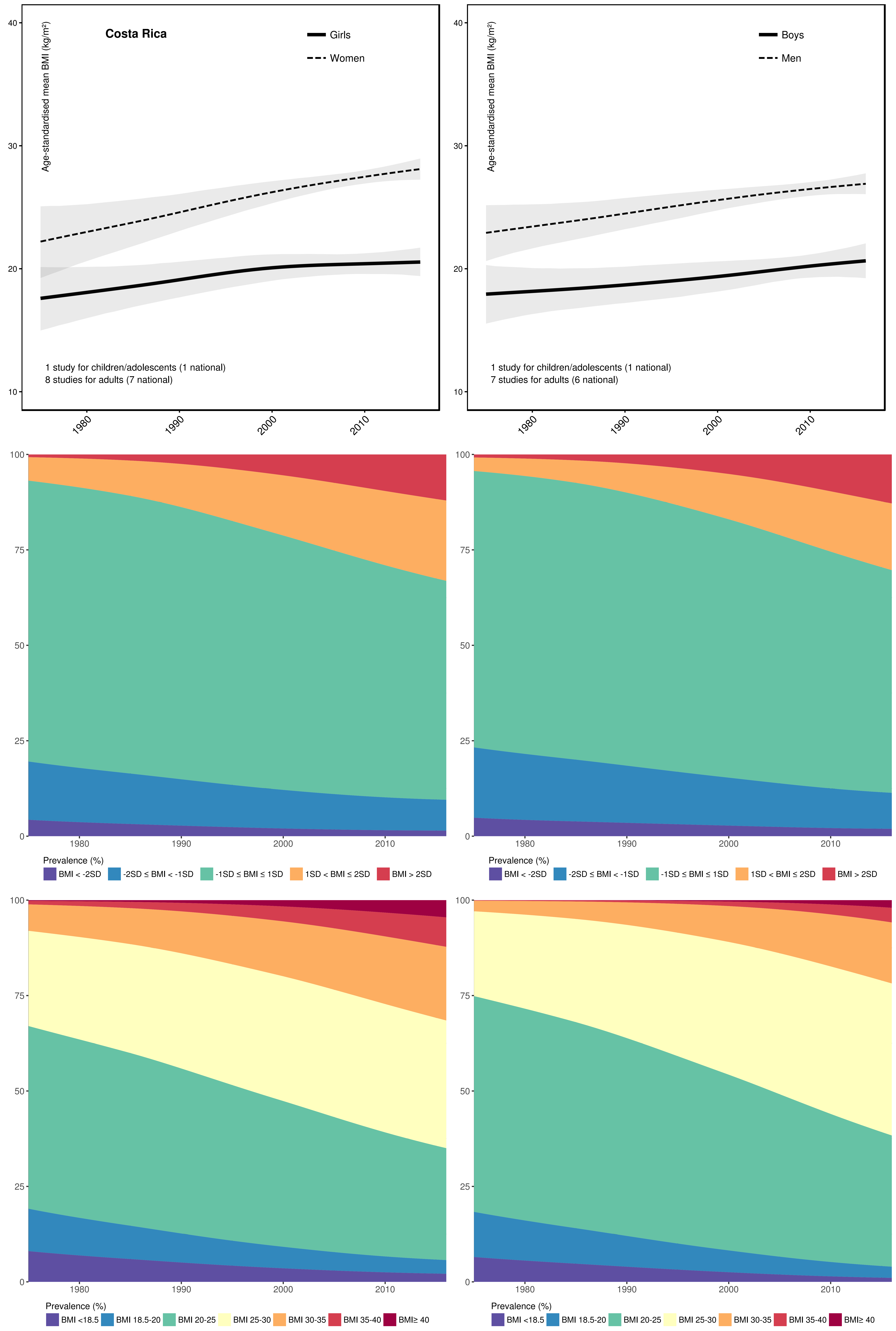
Costa Rica
Girls
Women
Boys
Men
Age-standardised mean BMI (kg/m²)
1 study for children/adolescents (1 national)
8 studies for adults (7 national)
1 study for children/adolescents (1 national)
7 studies for adults (6 national)
1980
1990
2000
2010
Prevalence (%)
BMI < -2SD
-2SD ≤ BMI < -1SD
-1SD ≤ BMI ≤ 1SD
1SD < BMI ≤ 2SD
BMI > 2SD
BMI <18.5
BMI 18.5-20
BMI 20-25
BMI 25-30
BMI 30-35
BMI 35-40
BMI≥ 40

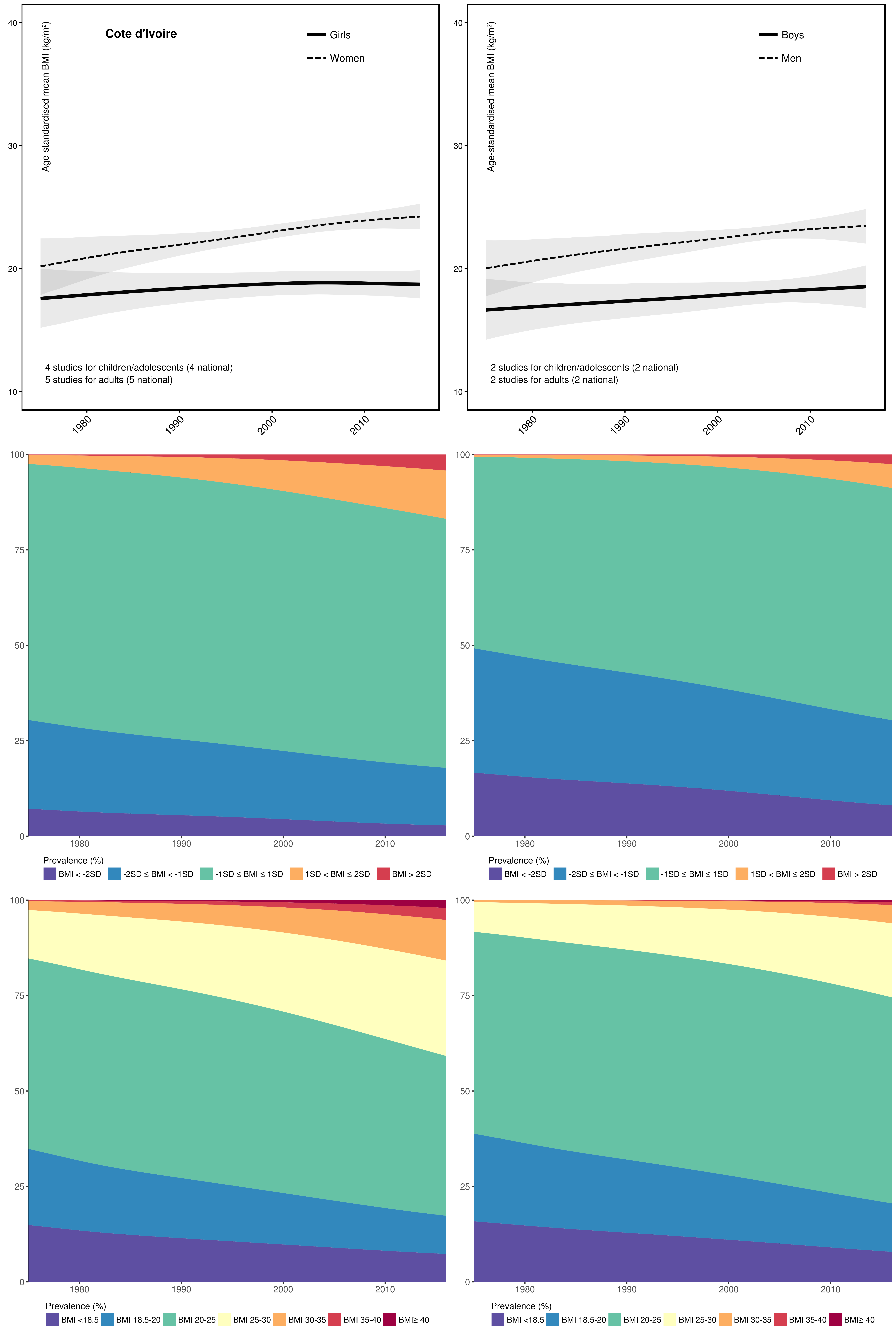
Cote d'Ivoire
Girls
Women
Boys
Men
Age-standardised mean BMI (kg/m²)
4 studies for children/adolescents (4 national)
5 studies for adults (5 national)
2 studies for children/adolescents (2 national)
2 studies for adults (2 national)
40
30
20
10
1980
1990
2000
2010
100
75
50
25
0
Prevalence (%)
BMI < -2SD
-2SD ≤ BMI < -1SD
-1SD ≤ BMI ≤ 1SD
1SD < BMI ≤ 2SD
BMI > 2SD
BMI <18.5
BMI 18.5-20
BMI 20-25
BMI 25-30
BMI 30-35
BMI 35-40
BMI≥ 40

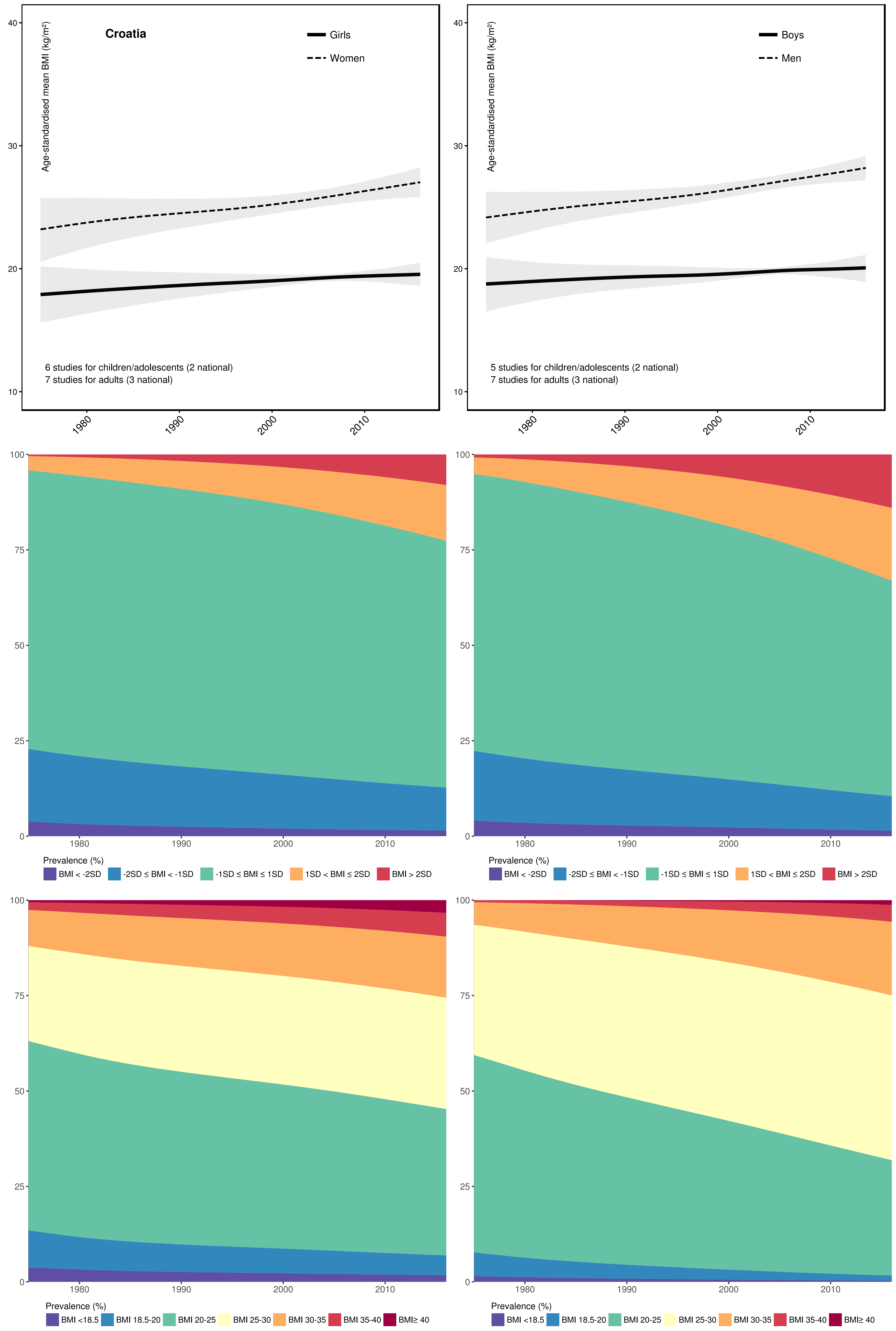
Croatia
Girls
Women
Age-standardised mean BMI (kg/m²)
6 studies for children/adolescents (2 national)
7 studies for adults (3 national)
Boys
Men
5 studies for children/adolescents (2 national)
7 studies for adults (3 national)
1980
1990
2000
2010
Prevalence (%)
BMI < -2SD
-2SD ≤ BMI < -1SD
-1SD ≤ BMI ≤ 1SD
1SD < BMI ≤ 2SD
BMI > 2SD
BMI <18.5
BMI 18.5-20
BMI 20-25
BMI 25-30
BMI 30-35
BMI 35-40
BMI≥ 40

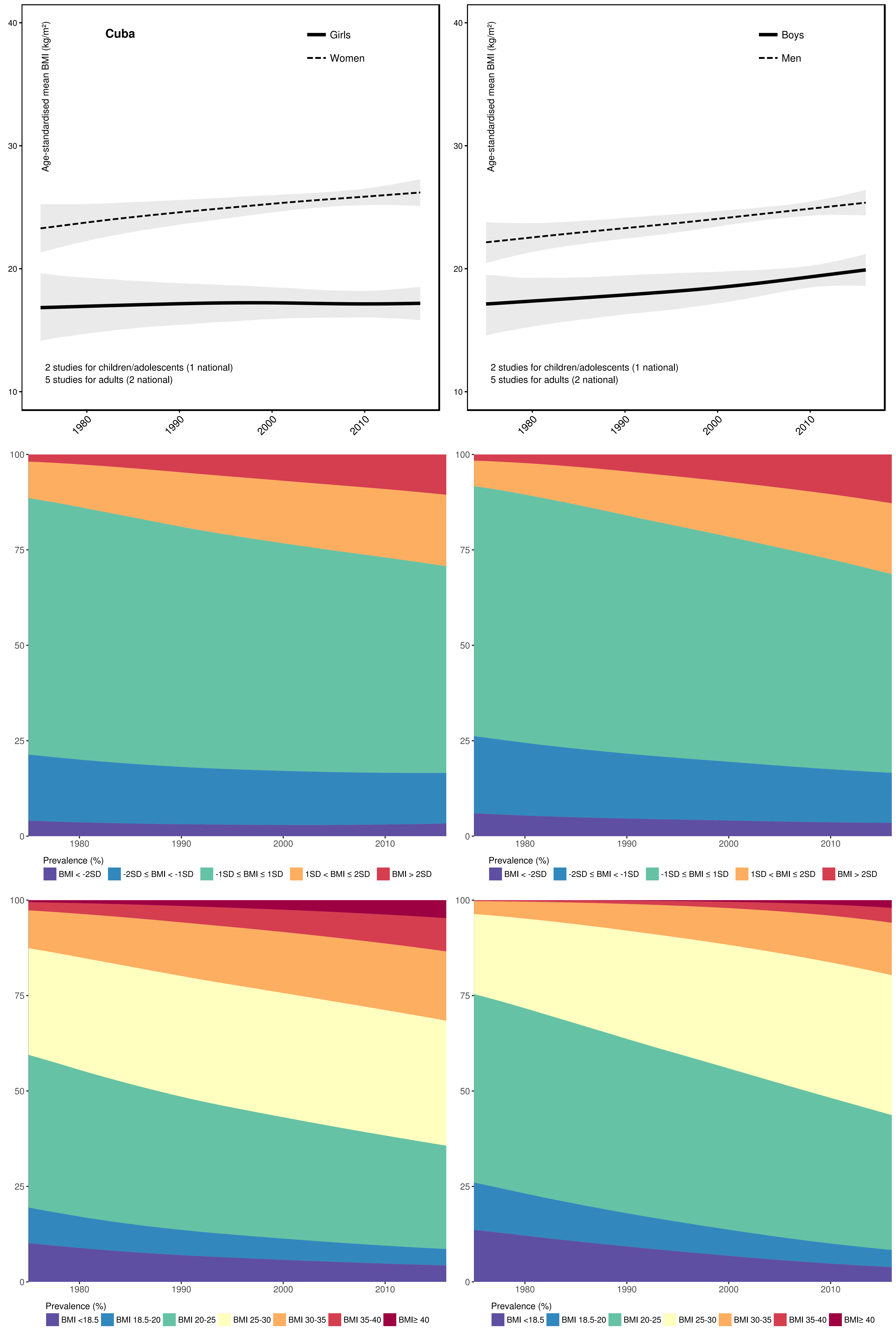
Cuba
Girls
Women
Boys
Men
Age-standardised mean BMI (kg/m²)
2 studies for children/adolescents (1 national)
5 studies for adults (2 national)
Prevalence (%)
BMI < -2SD
-2SD ≤ BMI < -1SD
-1SD ≤ BMI ≤ 1SD
1SD < BMI ≤ 2SD
BMI > 2SD
BMI <18.5
BMI 18.5-20
BMI 20-25
BMI 25-30
BMI 30-35
BMI 35-40
BMI≥ 40

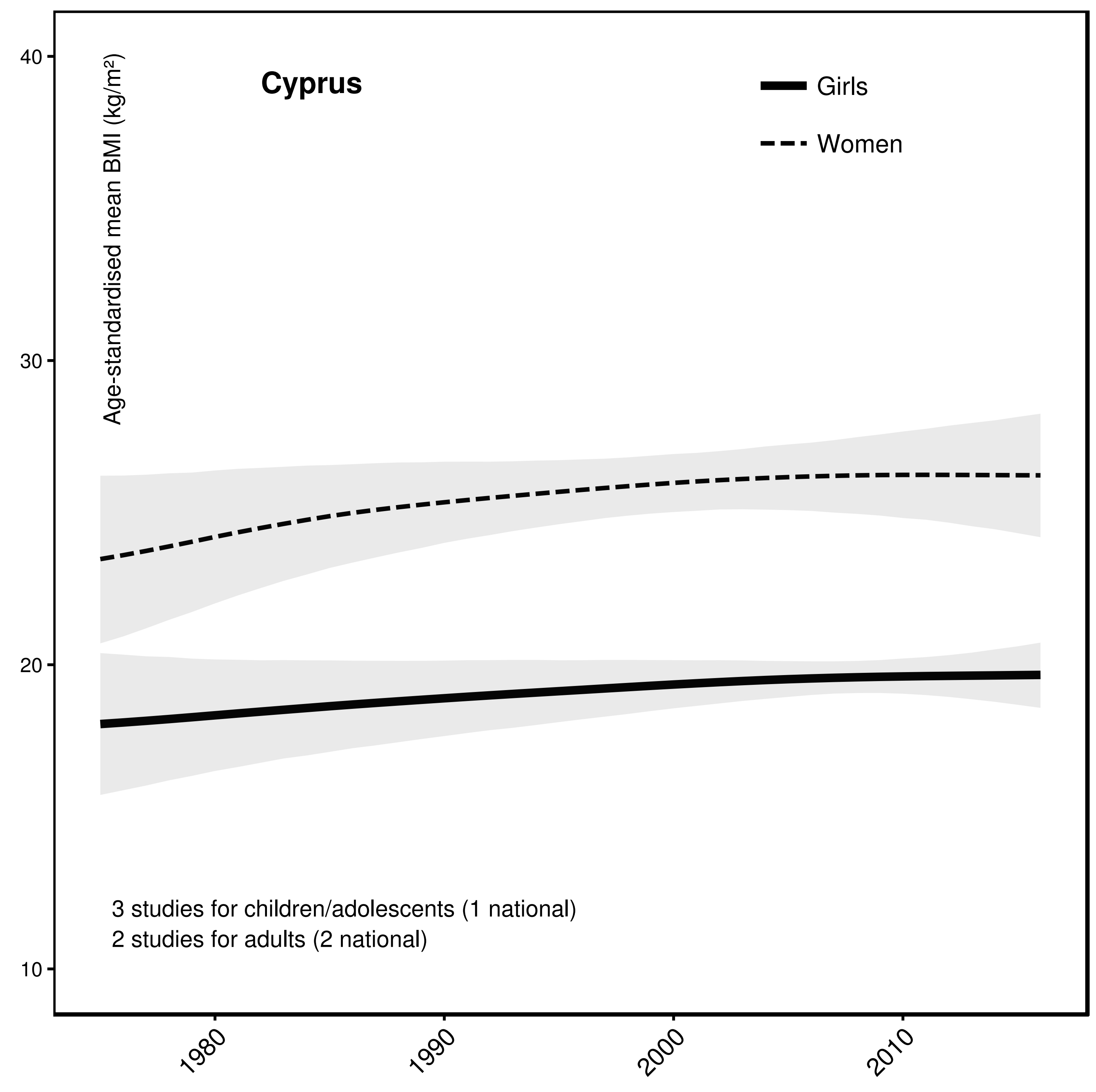
Cyprus
Girls
Women
Age-standardised mean BMI (kg/m²)
40
30
20
10
3 studies for children/adolescents (1 national)
2 studies for adults (2 national)
1980
1990
2000
2010

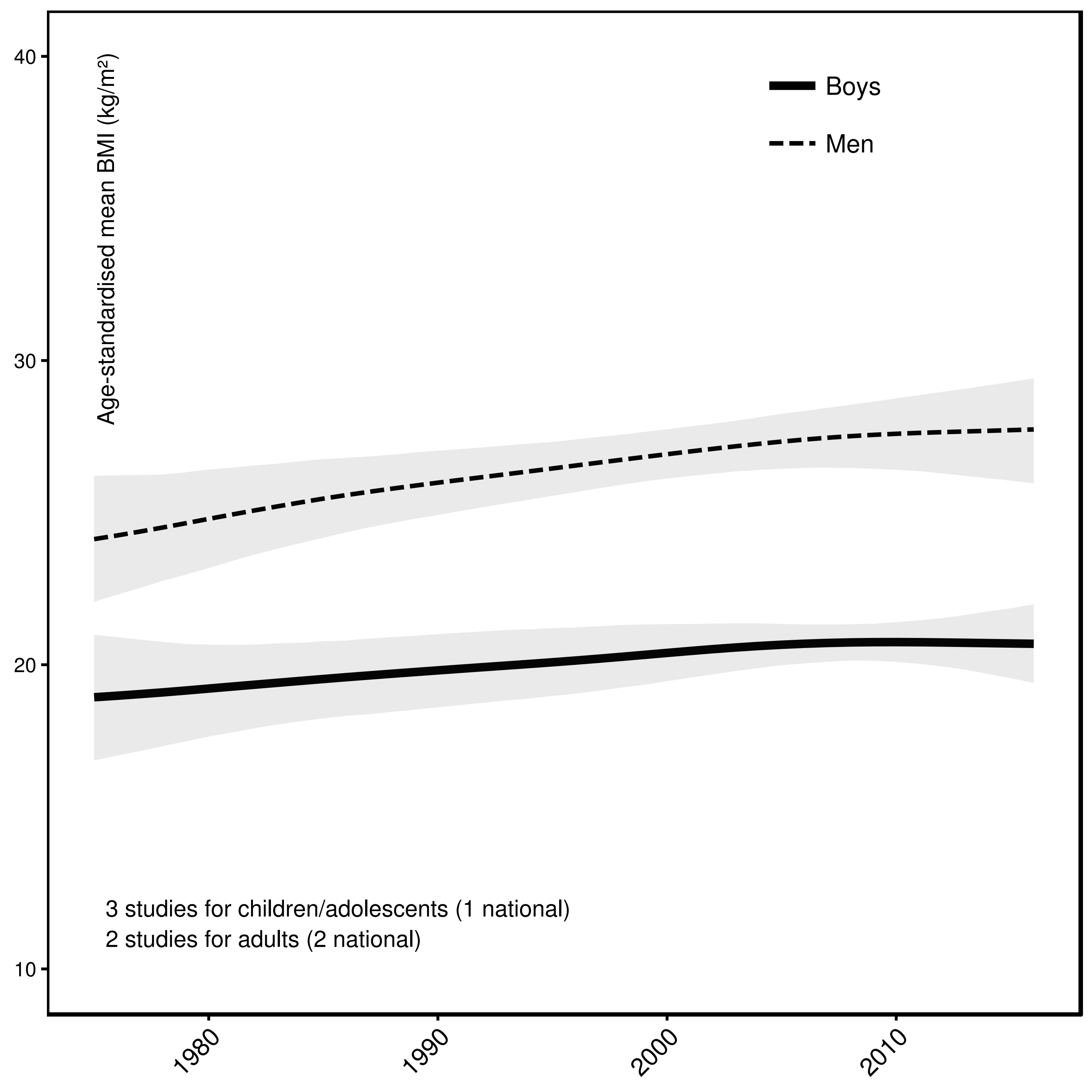
Boys
Men
Age-standardised mean BMI (kg/m²)
40
30
20
10
3 studies for children/adolescents (1 national)
2 studies for adults (2 national)
1980
1990
2000
2010

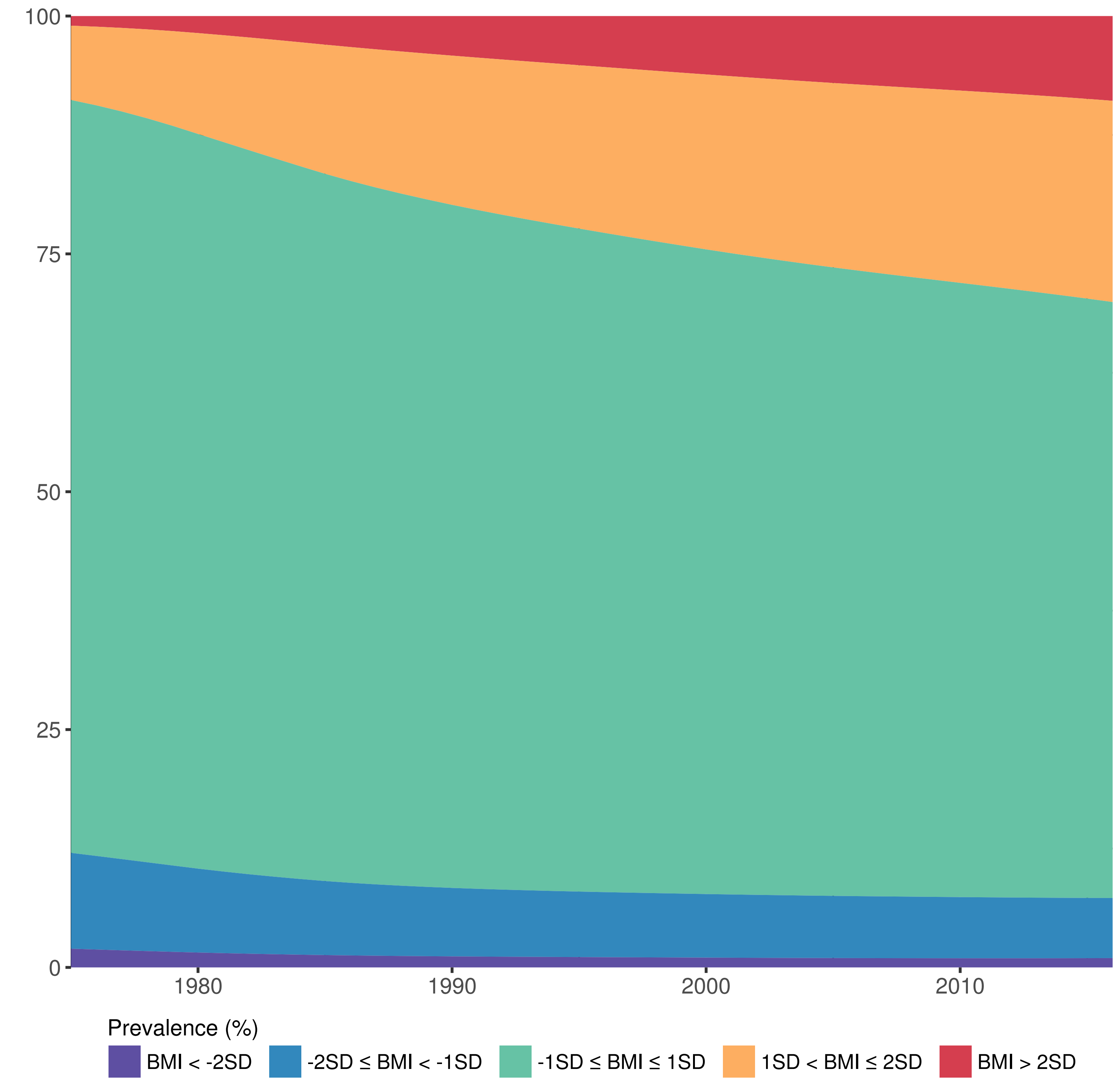
100
75
50
25
0
1980
1990
2000
2010
Prevalence (%)
BMI < -2SD
-2SD ≤ BMI < -1SD
-1SD ≤ BMI ≤ 1SD
1SD < BMI ≤ 2SD
BMI > 2SD

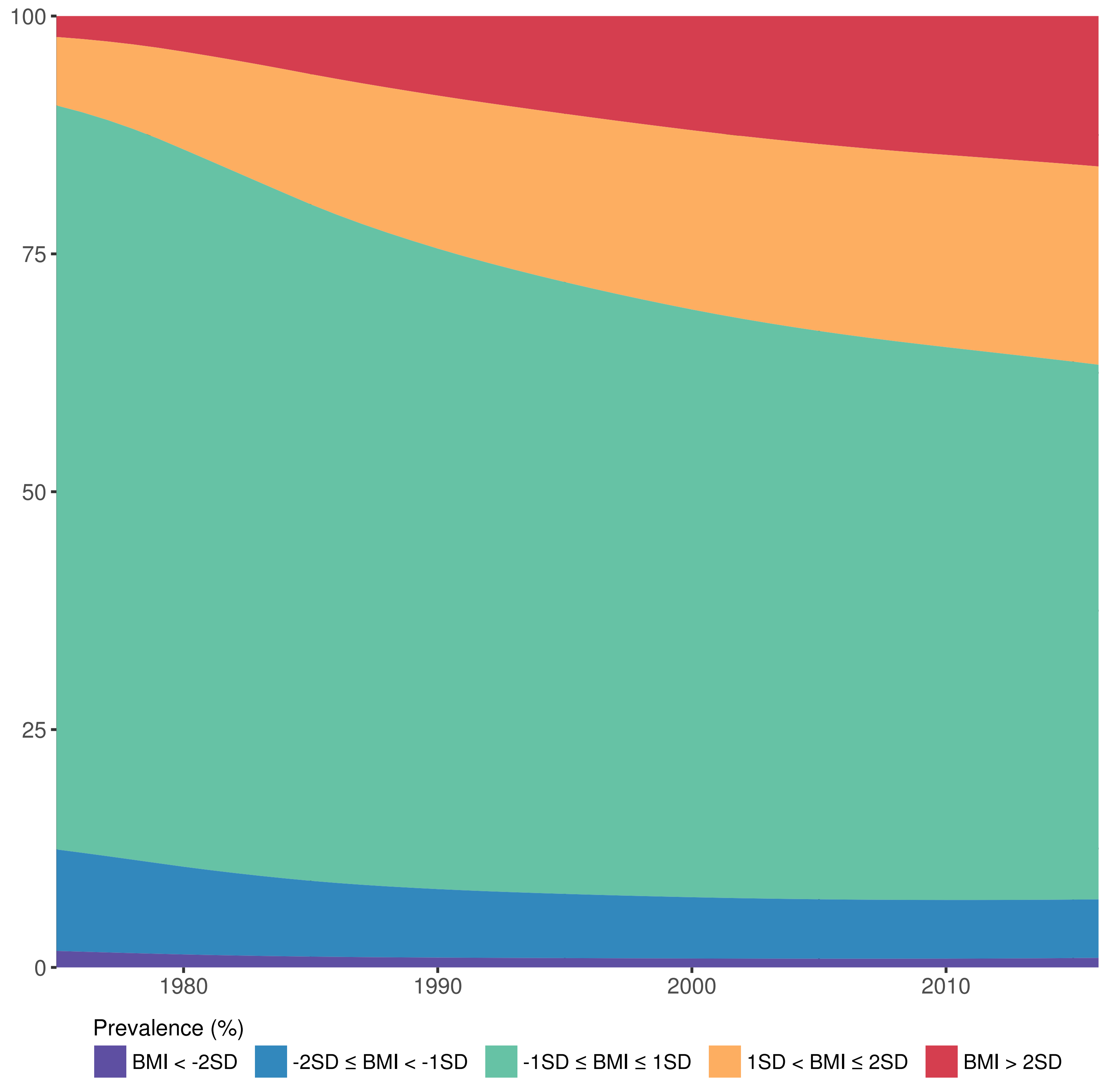
100
75
50
25
0
1980
1990
2000
2010
Prevalence (%)
BMI < -2SD
-2SD ≤ BMI < -1SD
-1SD ≤ BMI ≤ 1SD
1SD < BMI ≤ 2SD
BMI > 2SD

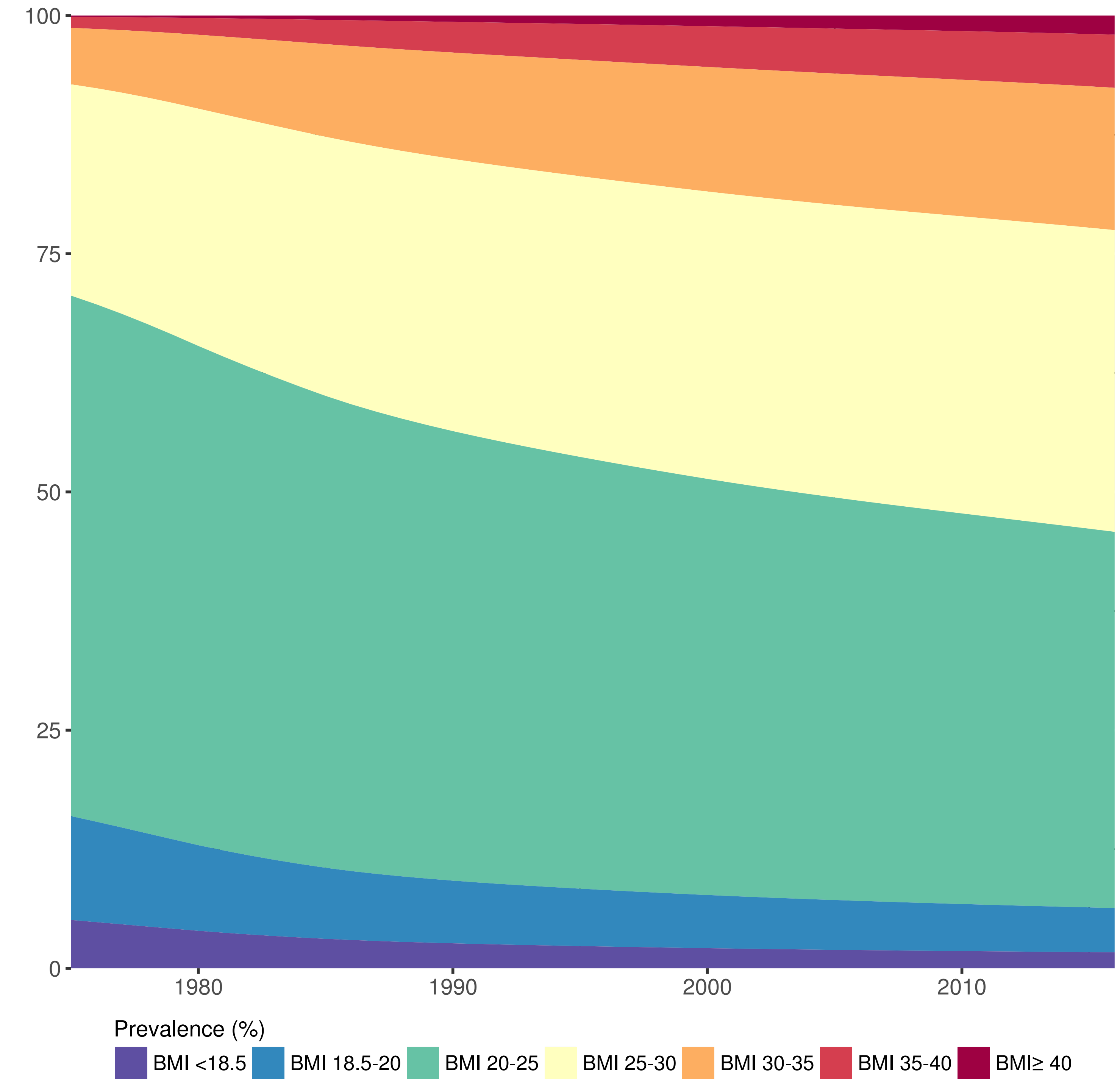
100
75
50
25
0
1980
1990
2000
2010
Prevalence (%)
BMI <18.5
BMI 18.5-20
BMI 20-25
BMI 25-30
BMI 30-35
BMI 35-40
BMI≥ 40

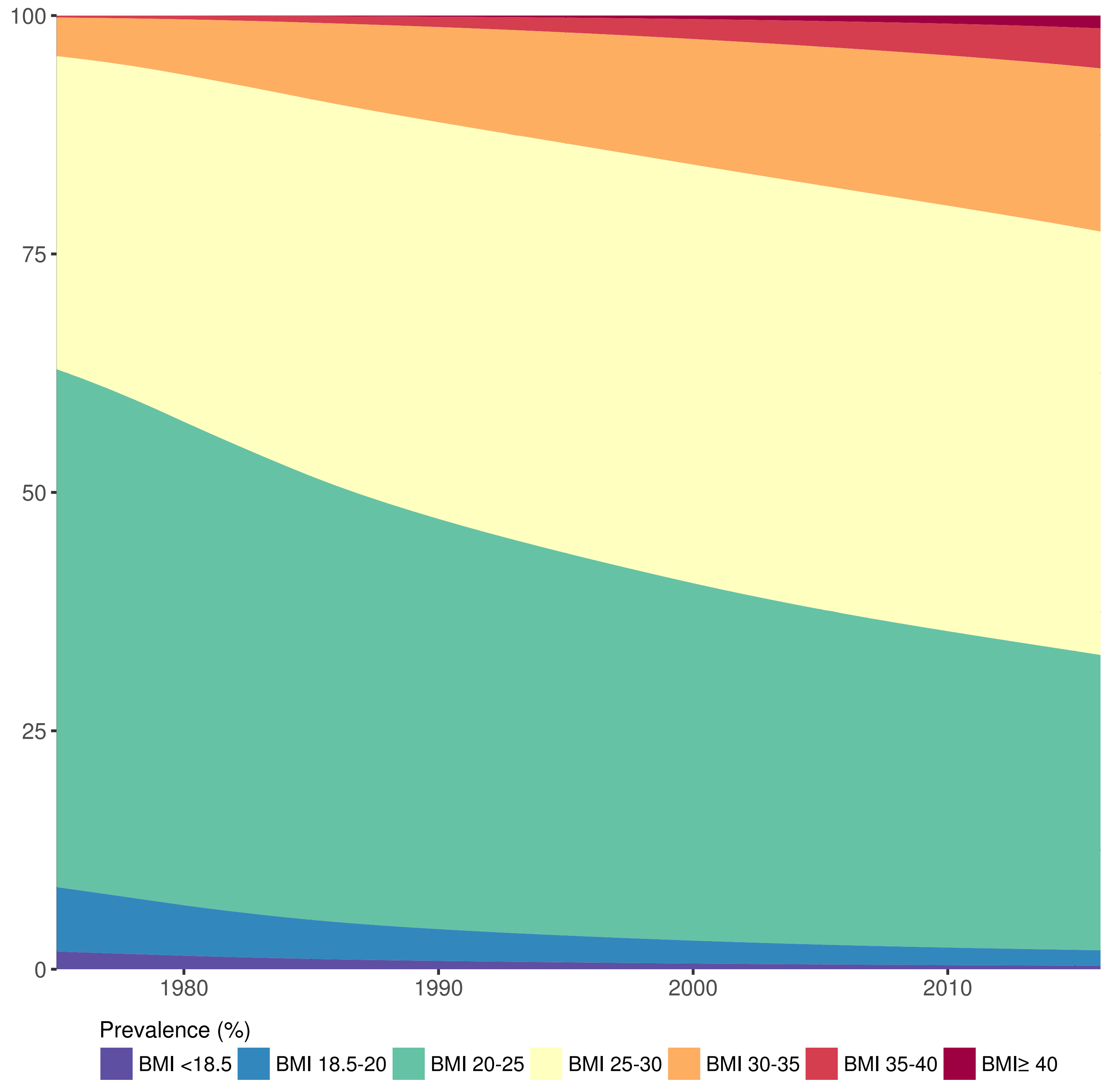
100
75
50
25
0
1980
1990
2000
2010
Prevalence (%)
BMI <18.5
BMI 18.5-20
BMI 20-25
BMI 25-30
BMI 30-35
BMI 35-40
BMI≥ 40

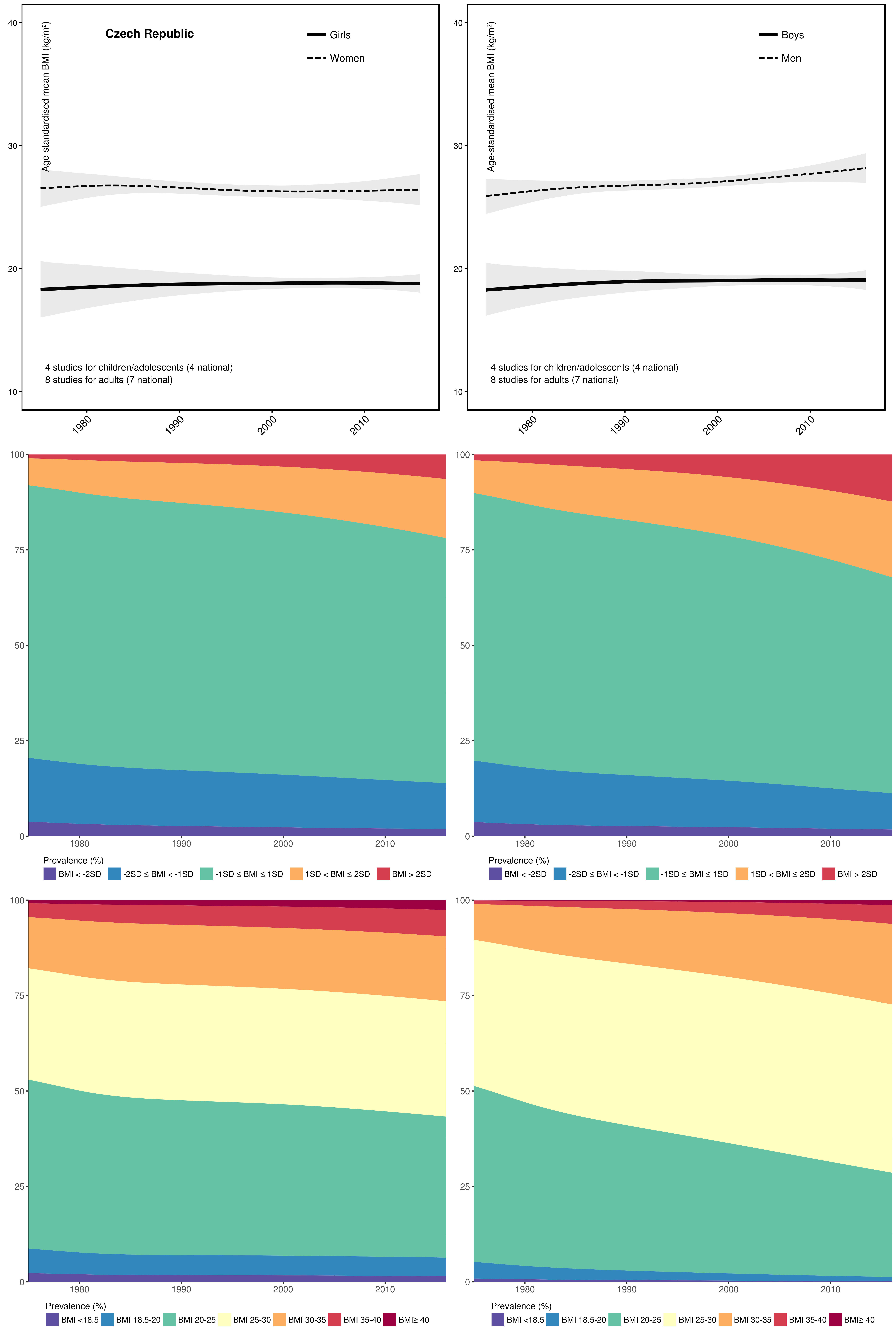
Czech Republic
Girls
Women
Boys
Men
Age-standardised mean BMI (kg/m²)
4 studies for children/adolescents (4 national)
8 studies for adults (7 national)
Prevalence (%)
BMI < -2SD
-2SD ≤ BMI < -1SD
-1SD ≤ BMI ≤ 1SD
1SD < BMI ≤ 2SD
BMI > 2SD
BMI <18.5
BMI 18.5-20
BMI 20-25
BMI 25-30
BMI 30-35
BMI 35-40
BMI≥ 40

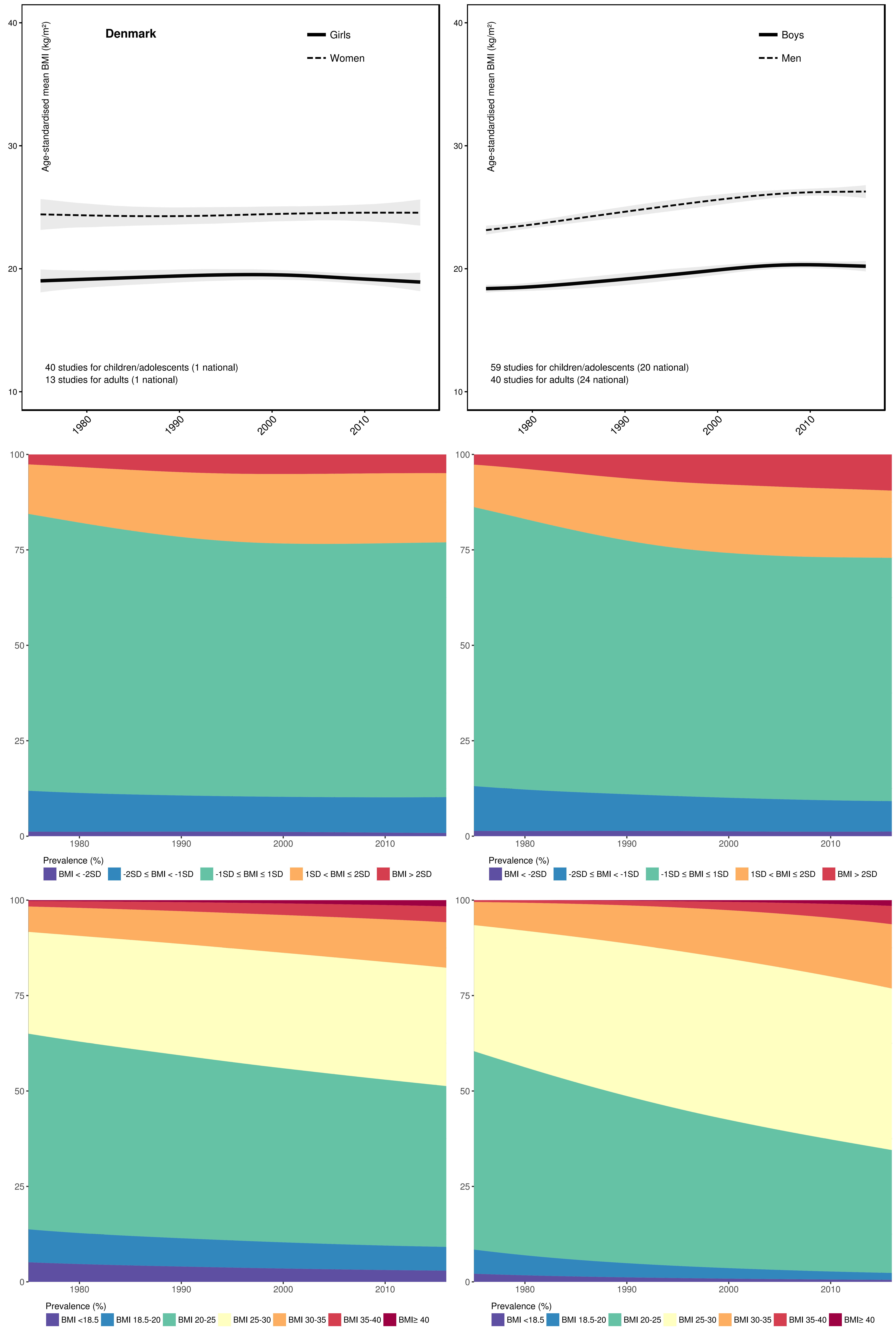
Denmark
Girls
Women
Boys
Men
Age-standardised mean BMI (kg/m²)
40 studies for children/adolescents (1 national)
13 studies for adults (1 national)
59 studies for children/adolescents (20 national)
40 studies for adults (24 national)
Prevalence (%)
BMI < -2SD
-2SD ≤ BMI < -1SD
-1SD ≤ BMI ≤ 1SD
1SD < BMI ≤ 2SD
BMI > 2SD
BMI <18.5
BMI 18.5-20
BMI 20-25
BMI 25-30
BMI 30-35
BMI 35-40
BMI≥ 40

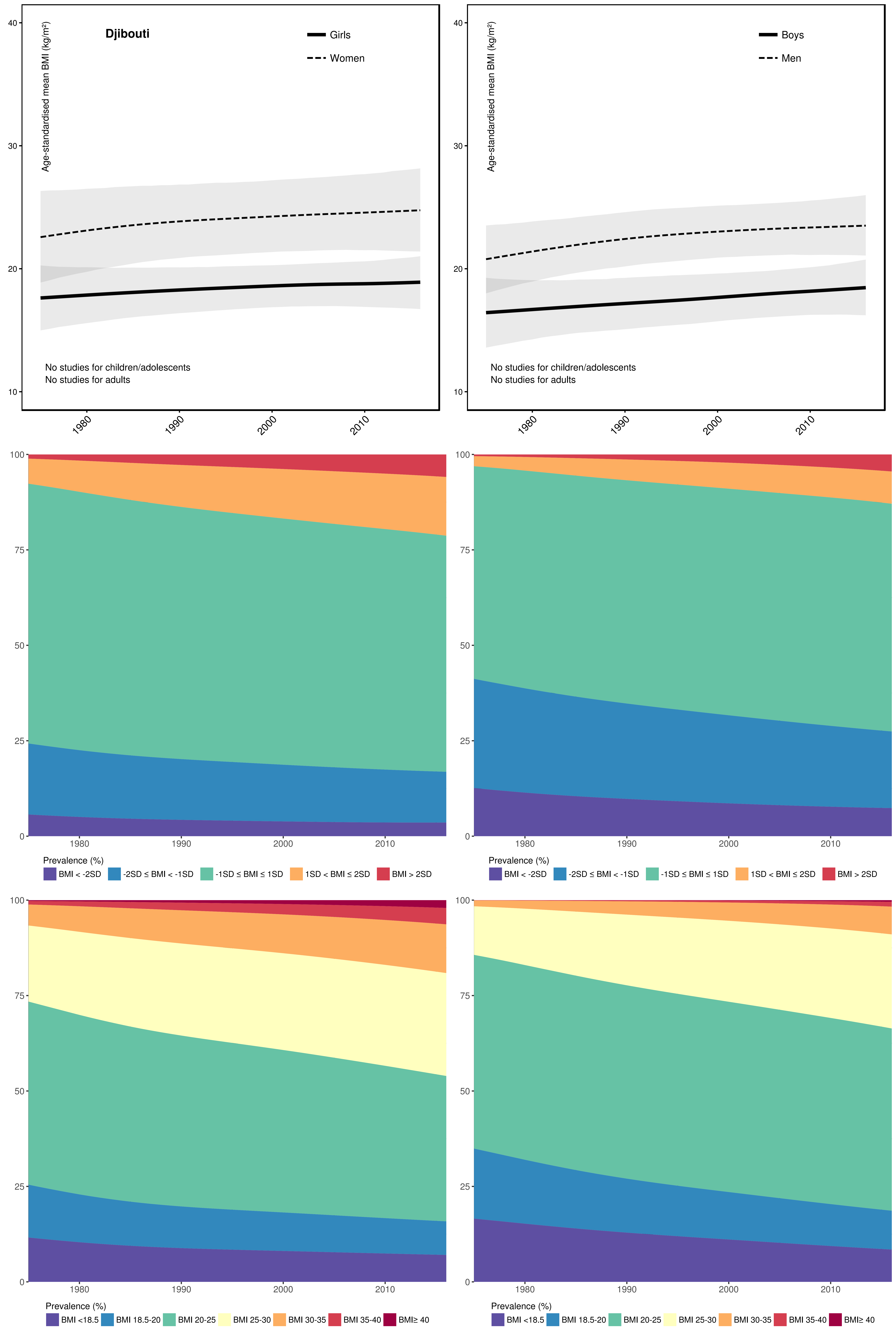
Djibouti
Girls
Women
Boys
Men
Age-standardised mean BMI (kg/m²)
No studies for children/adolescents
No studies for adults
Prevalence (%)
BMI < -2SD
-2SD ≤ BMI < -1SD
-1SD ≤ BMI ≤ 1SD
1SD < BMI ≤ 2SD
BMI > 2SD
BMI <18.5
BMI 18.5-20
BMI 20-25
BMI 25-30
BMI 30-35
BMI 35-40
BMI≥ 40

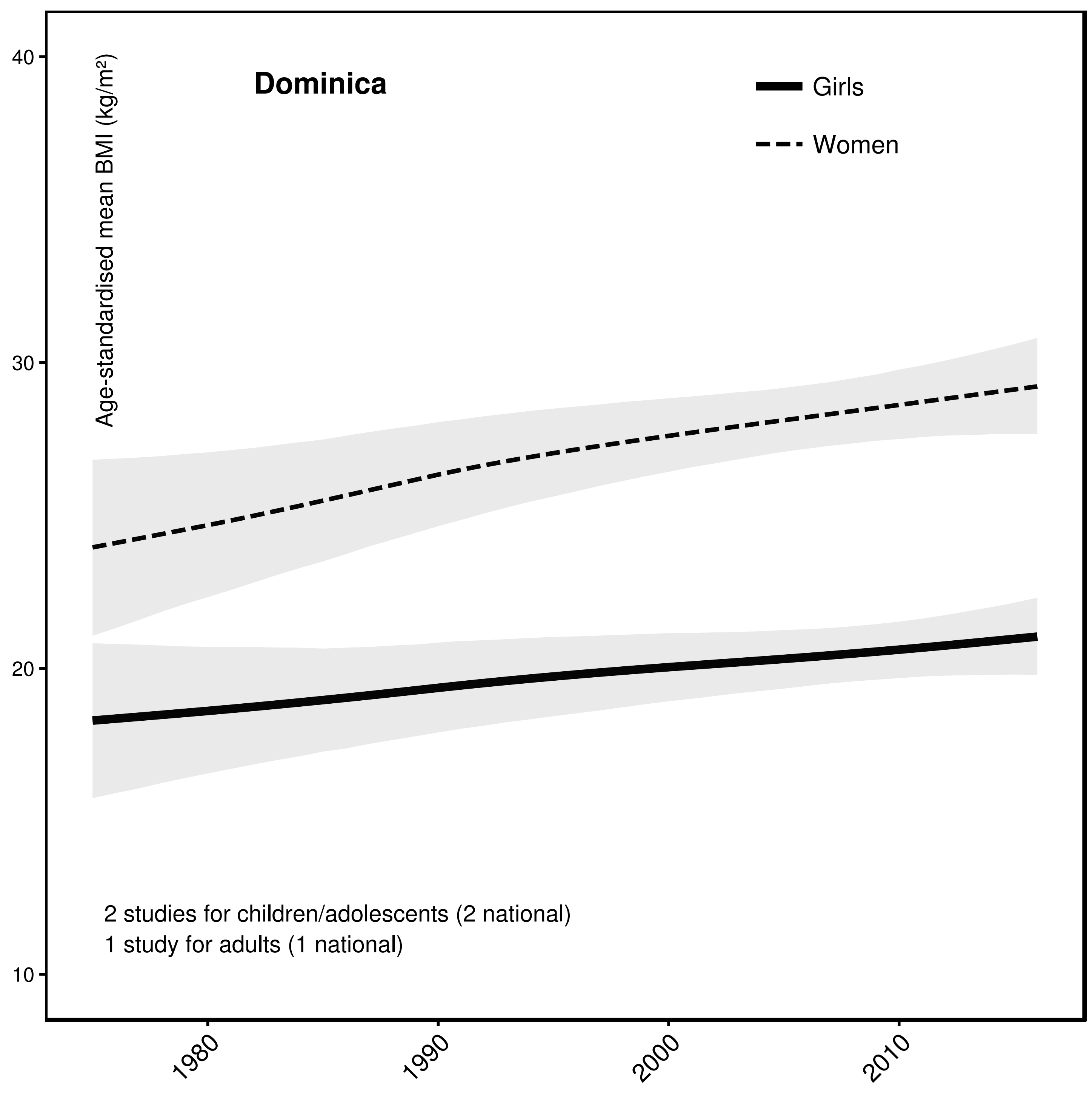
Dominica
Girls
Women
Age-standardised mean BMI (kg/m²)
2 studies for children/adolescents (2 national)
1 study for adults (1 national)
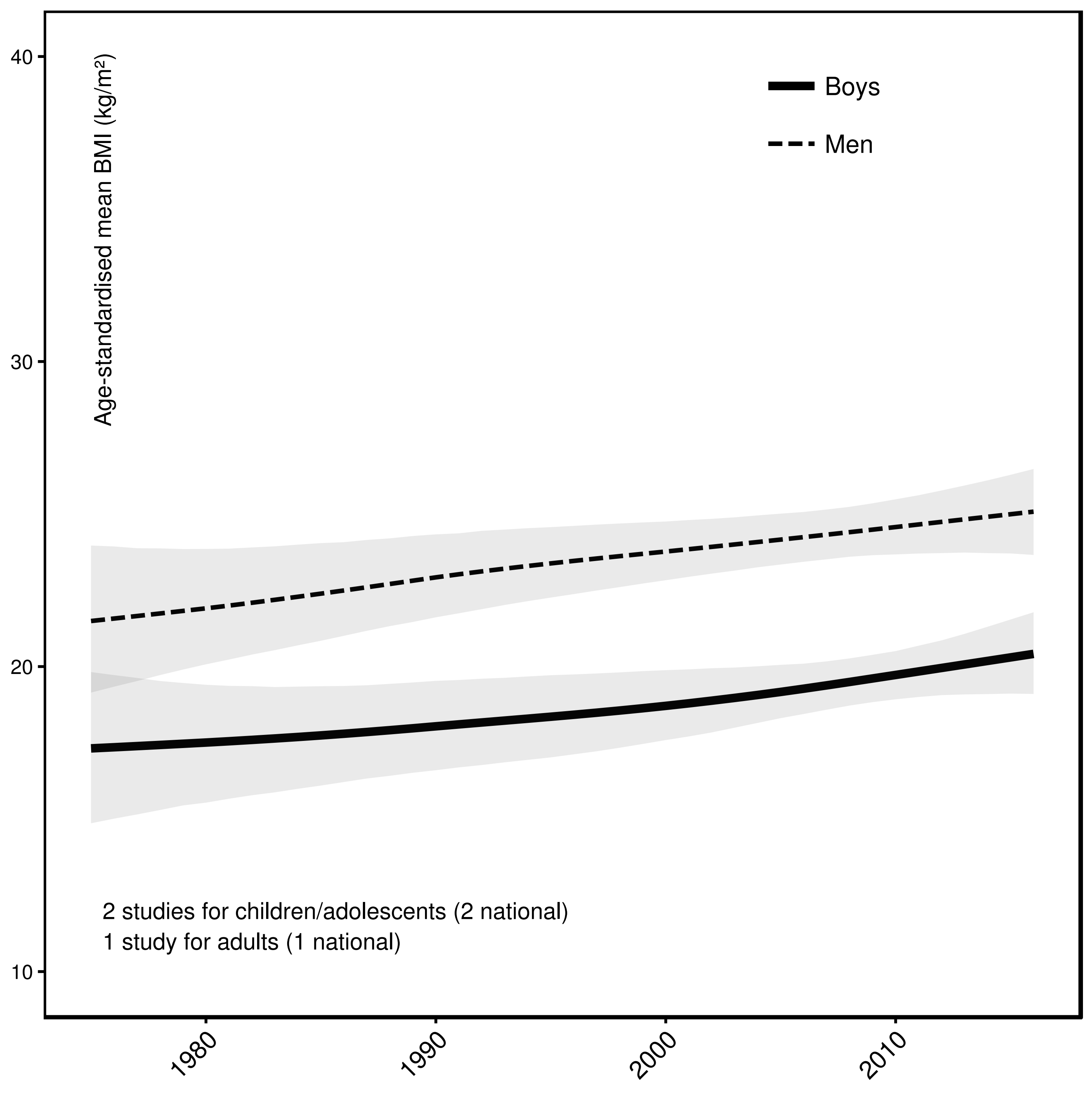
Boys
Men
Age-standardised mean BMI (kg/m²)
2 studies for children/adolescents (2 national)
1 study for adults (1 national)
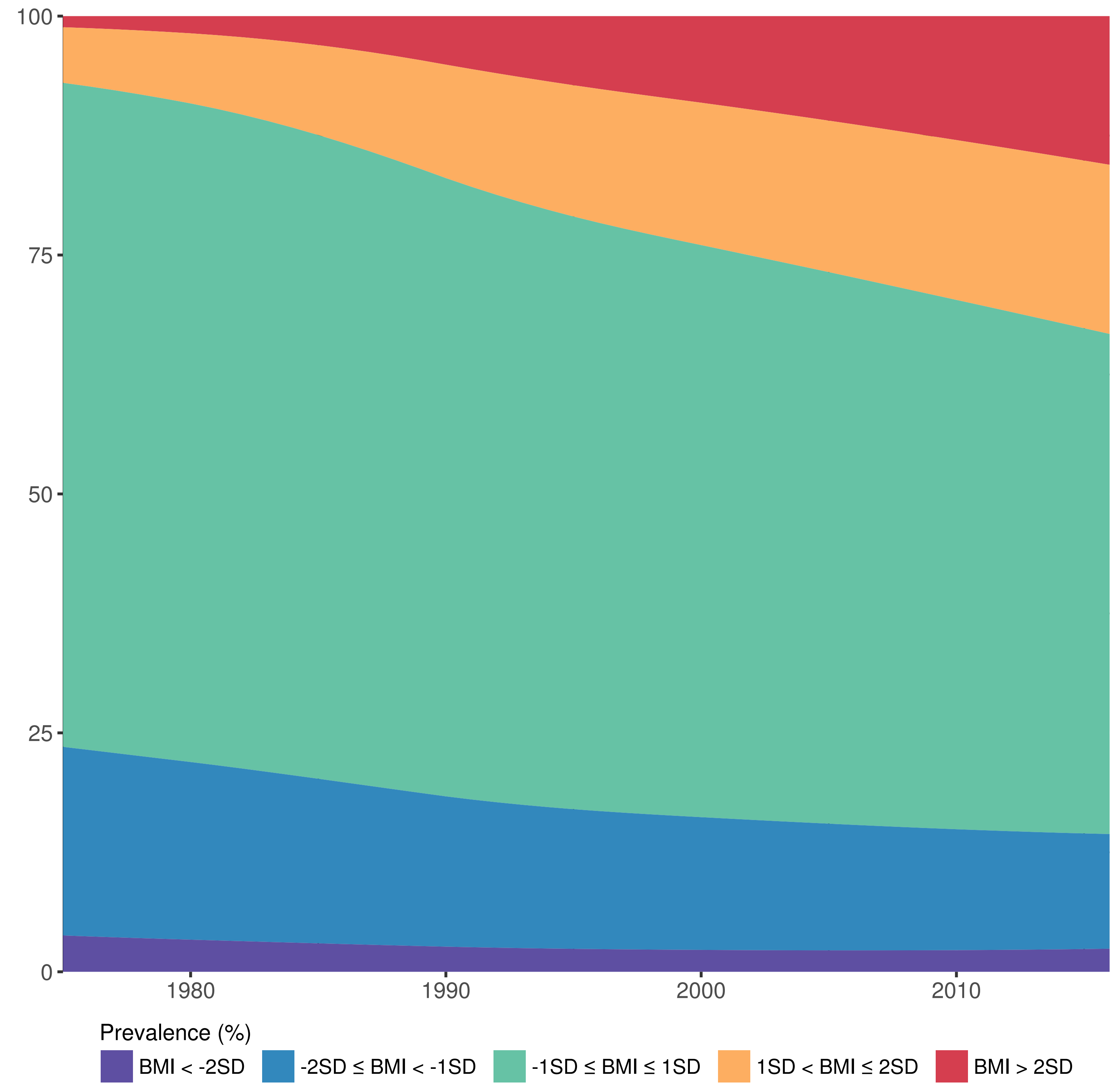
Prevalence (%)
BMI < -2SD
-2SD ≤ BMI < -1SD
-1SD ≤ BMI ≤ 1SD
1SD < BMI ≤ 2SD
BMI > 2SD
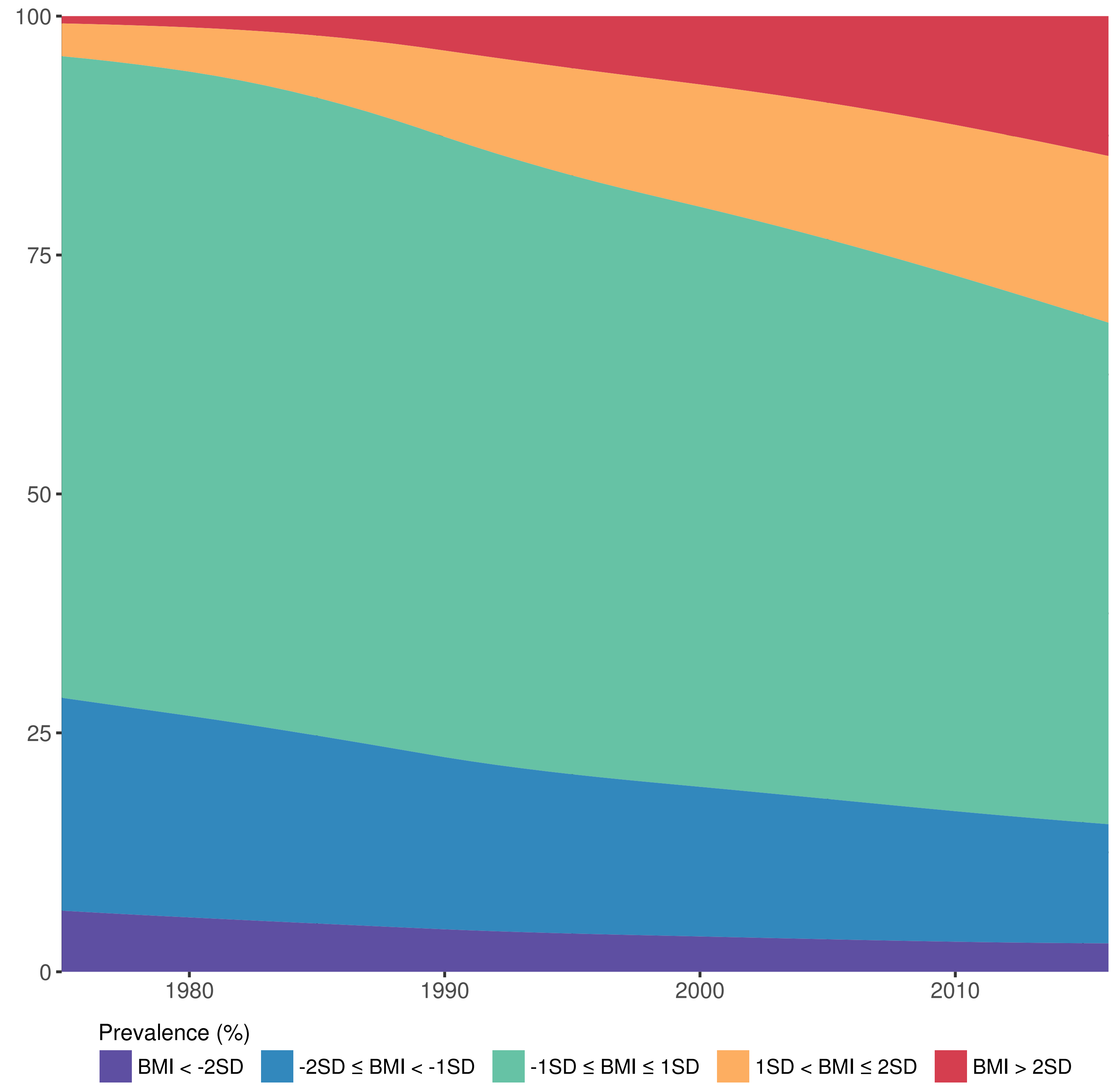
Prevalence (%)
BMI < -2SD
-2SD ≤ BMI < -1SD
-1SD ≤ BMI ≤ 1SD
1SD < BMI ≤ 2SD
BMI > 2SD
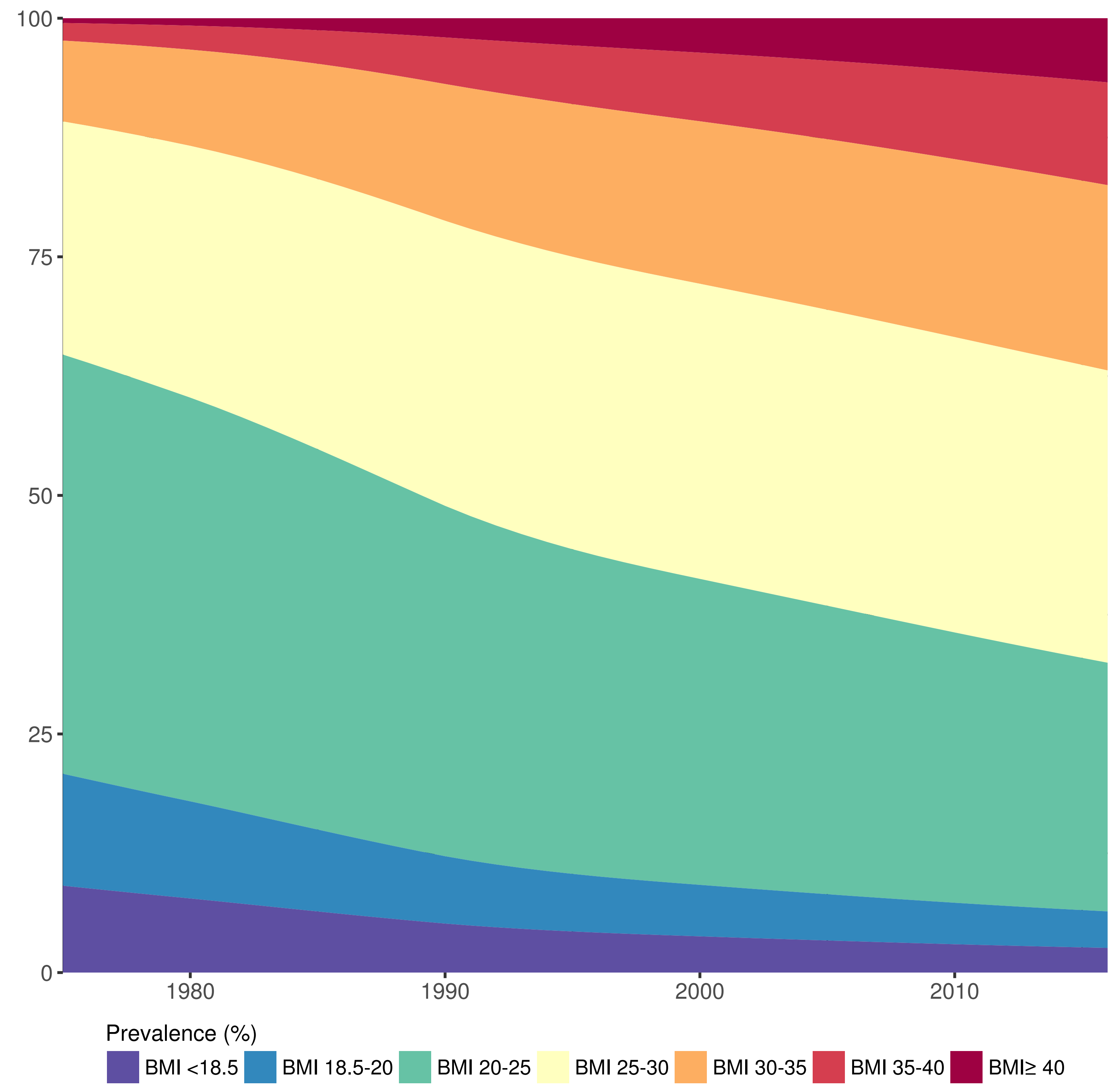
Prevalence (%)
BMI <18.5
BMI 18.5-20
BMI 20-25
BMI 25-30
BMI 30-35
BMI 35-40
BMI≥ 40
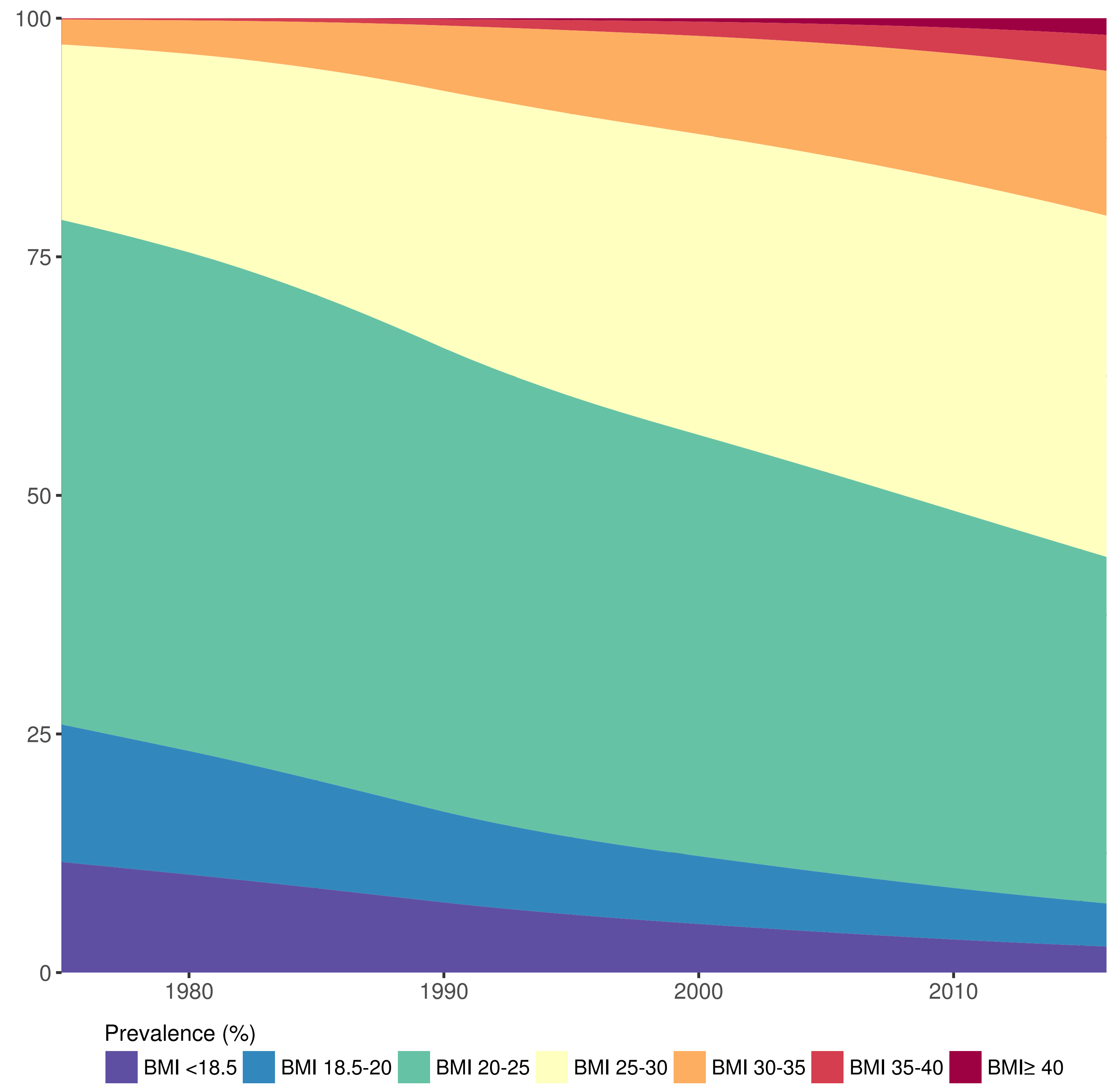
Prevalence (%)
BMI <18.5
BMI 18.5-20
BMI 20-25
BMI 25-30
BMI 30-35
BMI 35-40
BMI≥ 40

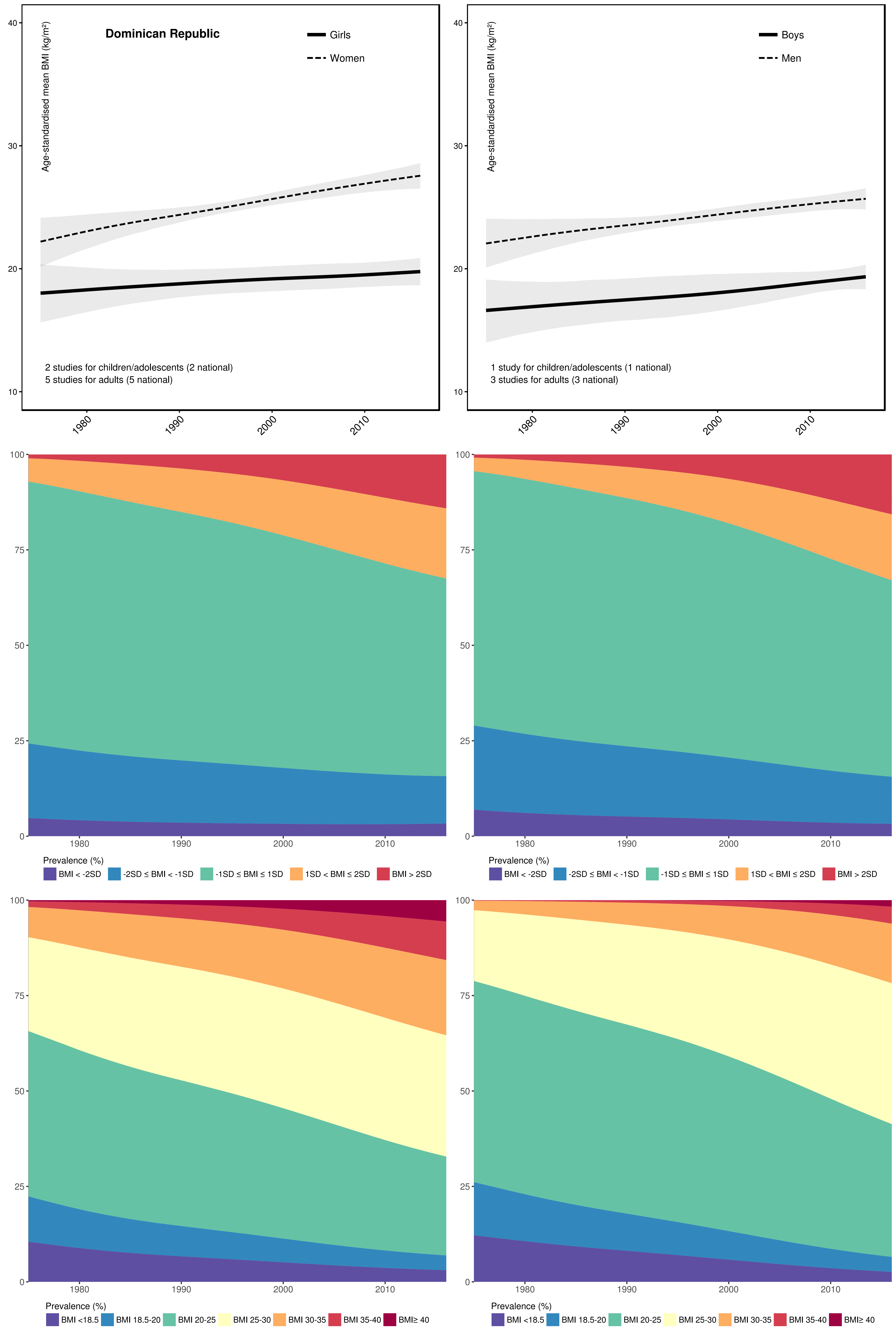
Dominican Republic
Girls
Women
Boys
Men
Age-standardised mean BMI (kg/m²)
2 studies for children/adolescents (2 national)
5 studies for adults (5 national)
1 study for children/adolescents (1 national)
3 studies for adults (3 national)
Prevalence (%)
BMI < -2SD
-2SD ≤ BMI < -1SD
-1SD ≤ BMI ≤ 1SD
1SD < BMI ≤ 2SD
BMI > 2SD
BMI <18.5
BMI 18.5-20
BMI 20-25
BMI 25-30
BMI 30-35
BMI 35-40
BMI≥ 40

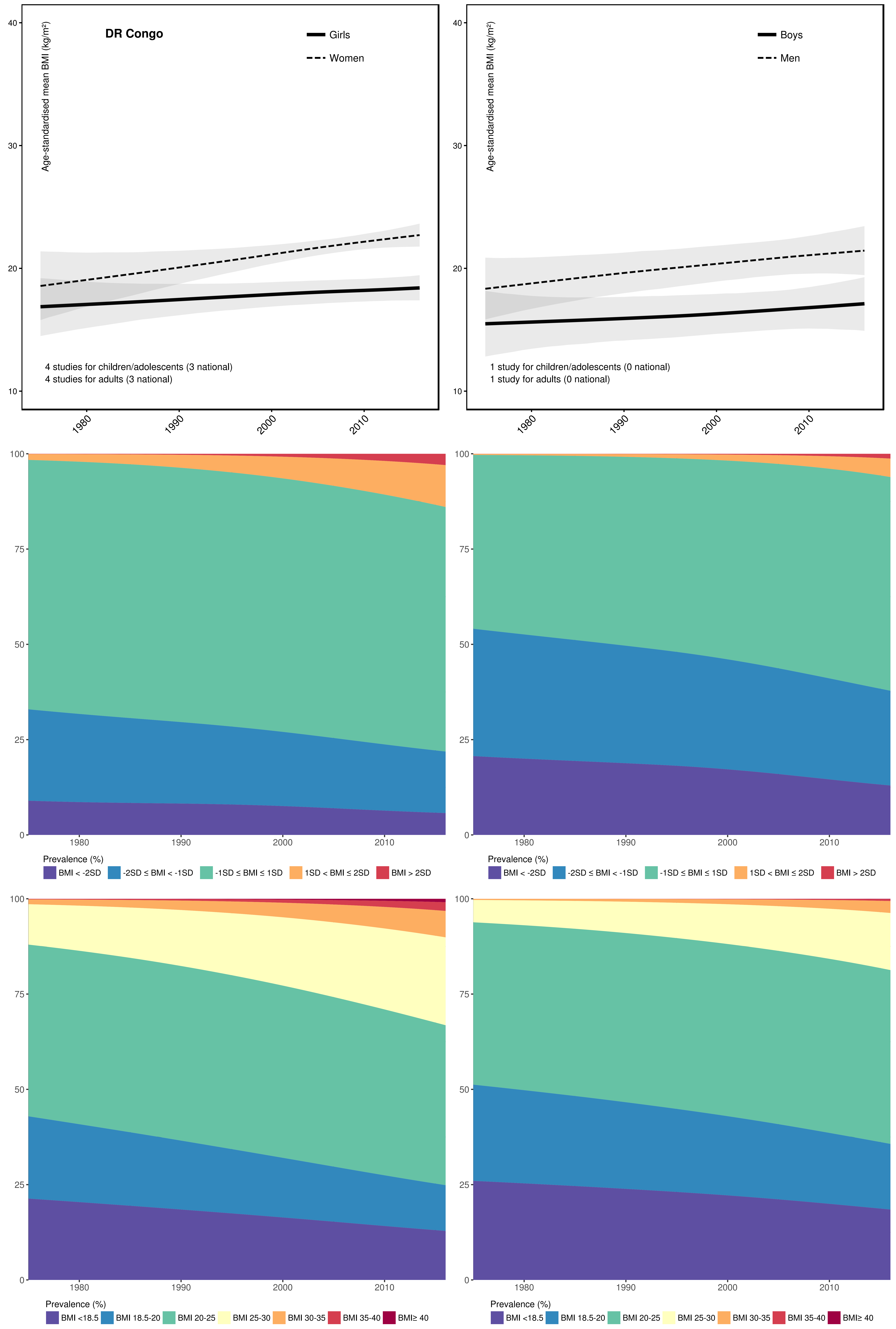
DR Congo
Girls
Women
Boys
Men
Age-standardised mean BMI (kg/m²)
4 studies for children/adolescents (3 national)
4 studies for adults (3 national)
1 study for children/adolescents (0 national)
1 study for adults (0 national)
Prevalence (%)
BMI < -2SD
-2SD ≤ BMI < -1SD
-1SD ≤ BMI ≤ 1SD
1SD < BMI ≤ 2SD
BMI > 2SD
BMI <18.5
BMI 18.5-20
BMI 20-25
BMI 25-30
BMI 30-35
BMI 35-40
BMI≥ 40

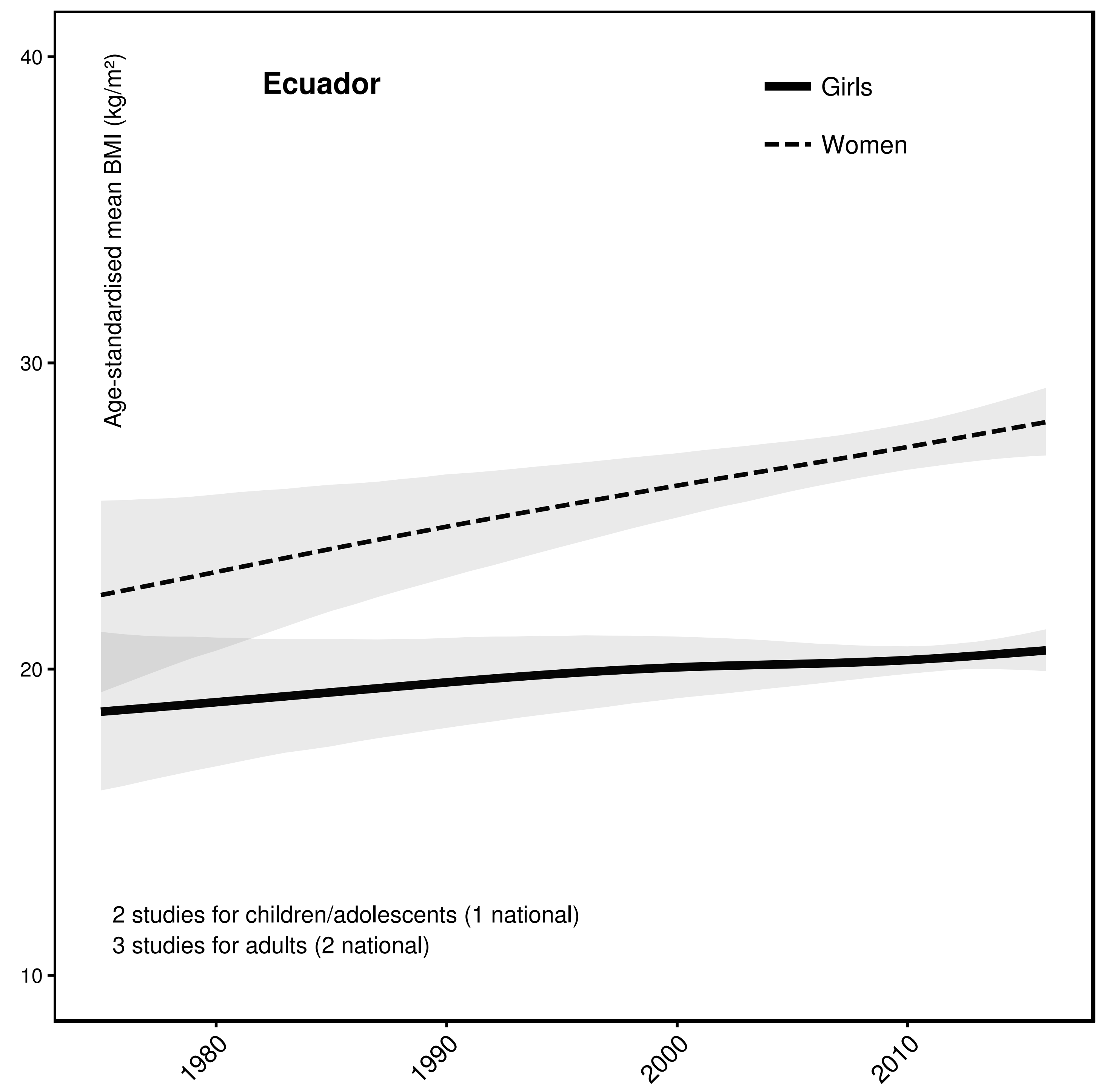
Ecuador
Girls
Women
Age-standardised mean BMI (kg/m²)
40
30
20
10
2 studies for children/adolescents (1 national)
3 studies for adults (2 national)
1980
1990
2000
2010

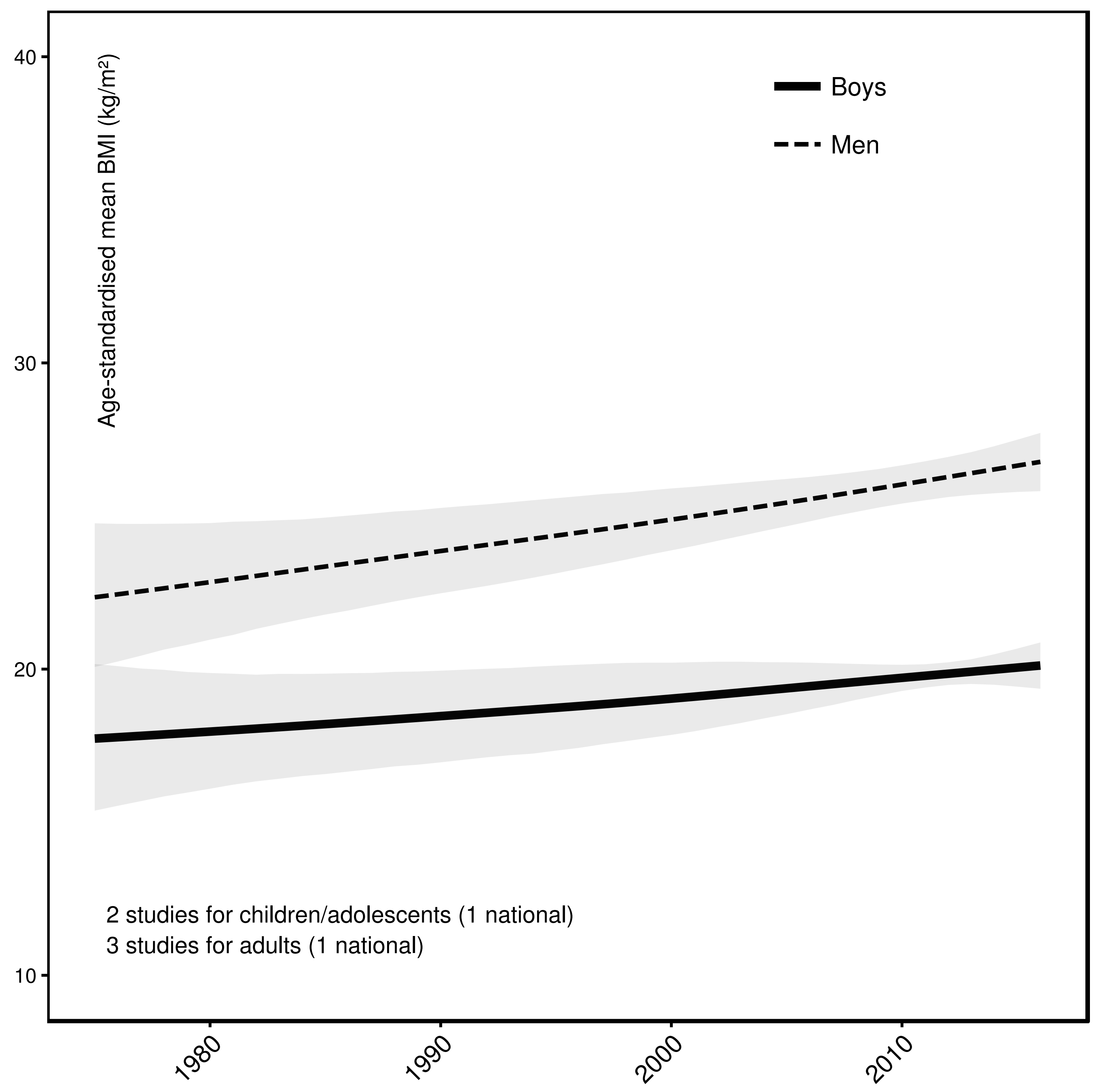
Boys
Men
Age-standardised mean BMI (kg/m²)
40
30
20
10
2 studies for children/adolescents (1 national)
3 studies for adults (1 national)
1980
1990
2000
2010

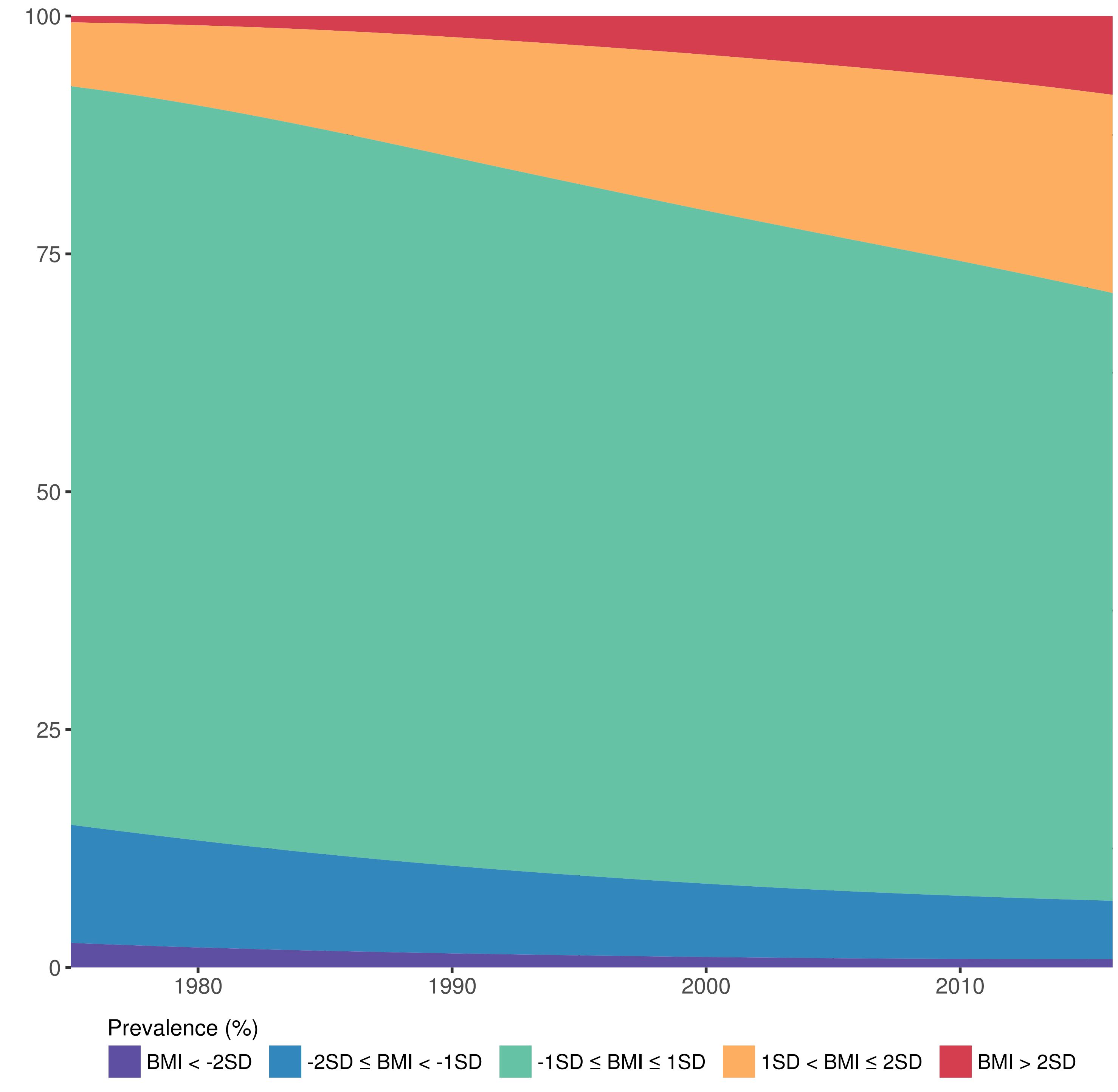
100
75
50
25
0
1980
1990
2000
2010
Prevalence (%)
BMI < -2SD
-2SD ≤ BMI < -1SD
-1SD ≤ BMI ≤ 1SD
1SD < BMI ≤ 2SD
BMI > 2SD

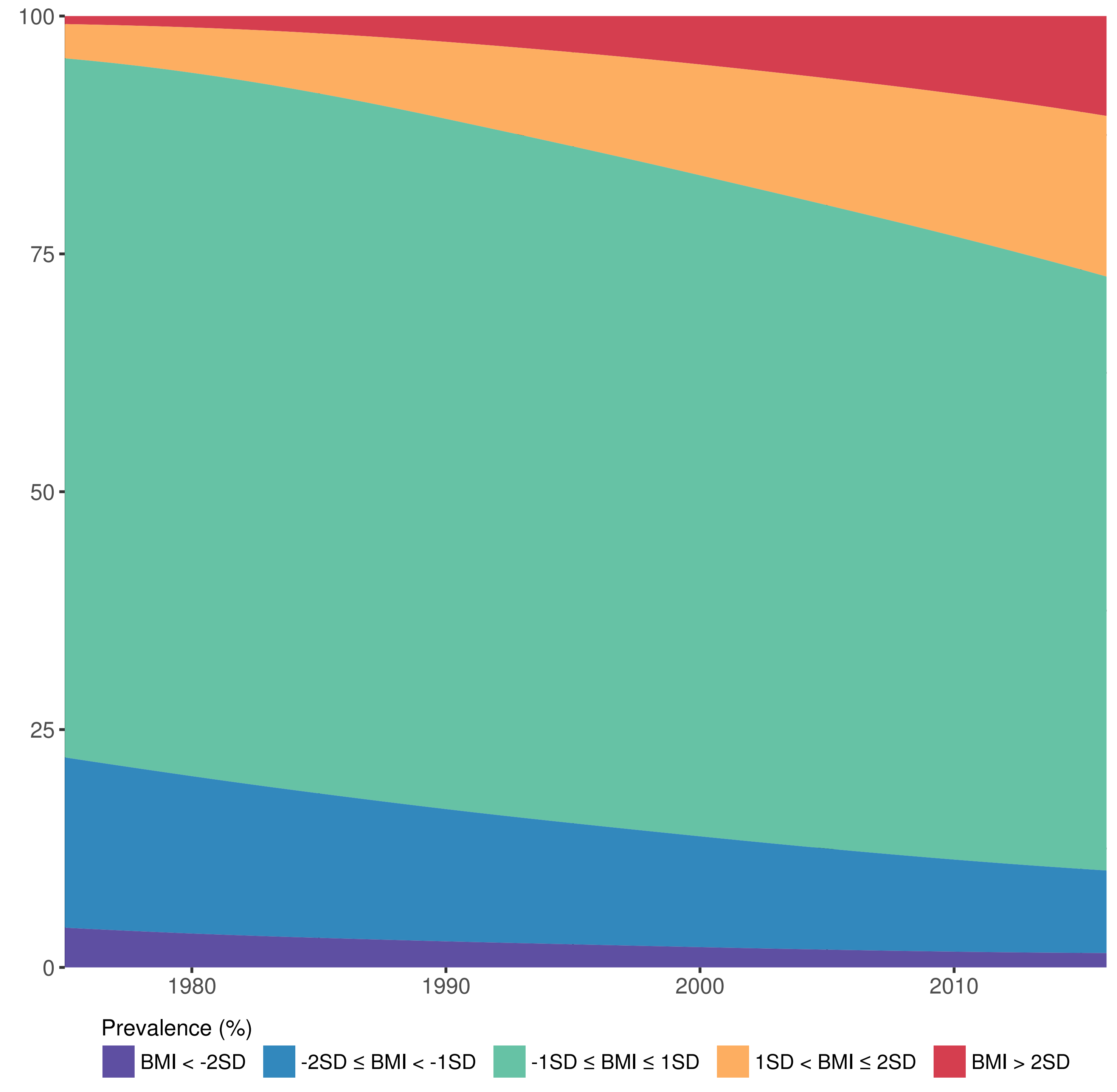
100
75
50
25
0
1980
1990
2000
2010
Prevalence (%)
BMI < -2SD
-2SD ≤ BMI < -1SD
-1SD ≤ BMI ≤ 1SD
1SD < BMI ≤ 2SD
BMI > 2SD

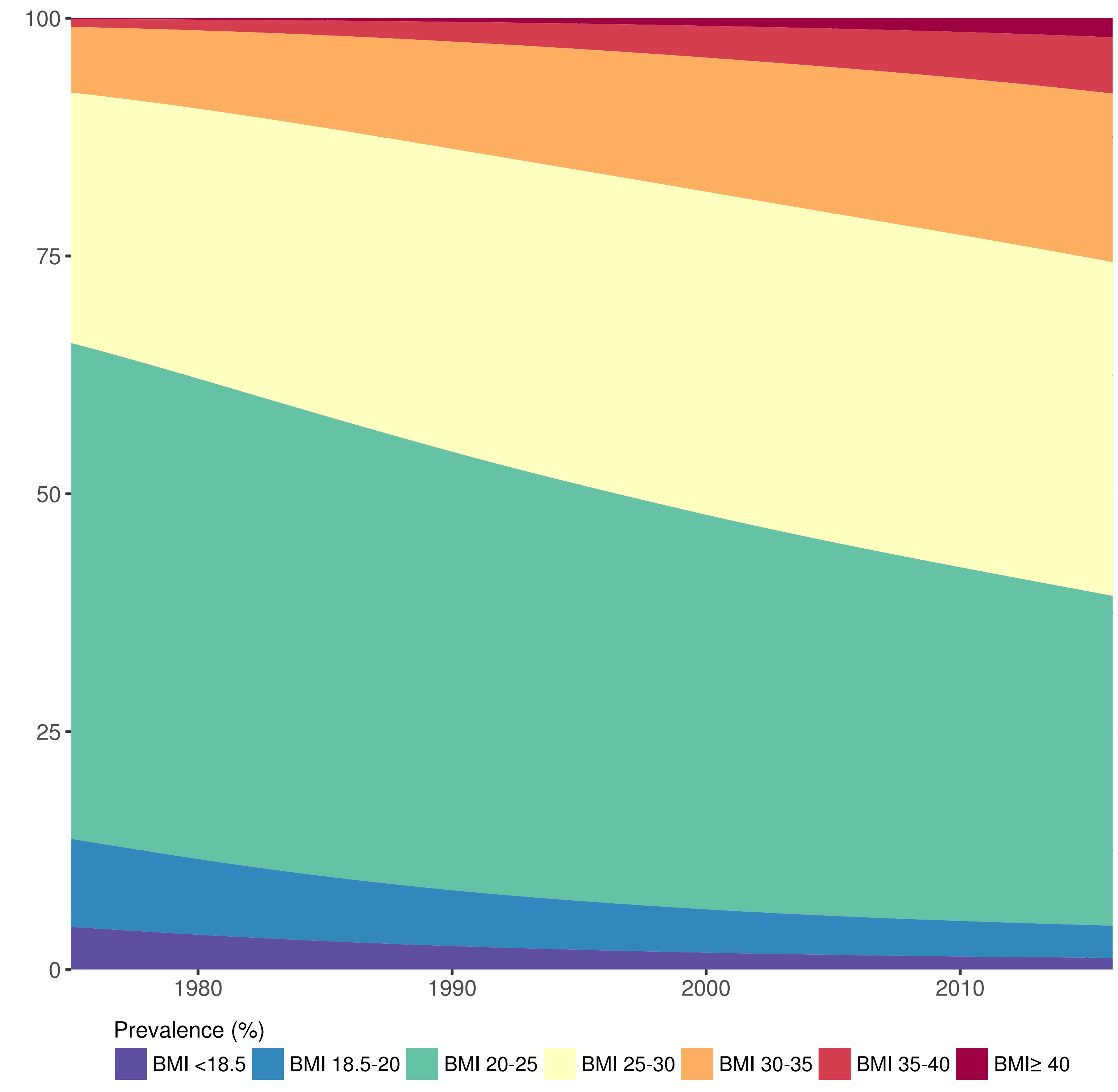
100
75
50
25
0
1980
1990
2000
2010
Prevalence (%)
BMI <18.5
BMI 18.5-20
BMI 20-25
BMI 25-30
BMI 30-35
BMI 35-40
BMI≥ 40

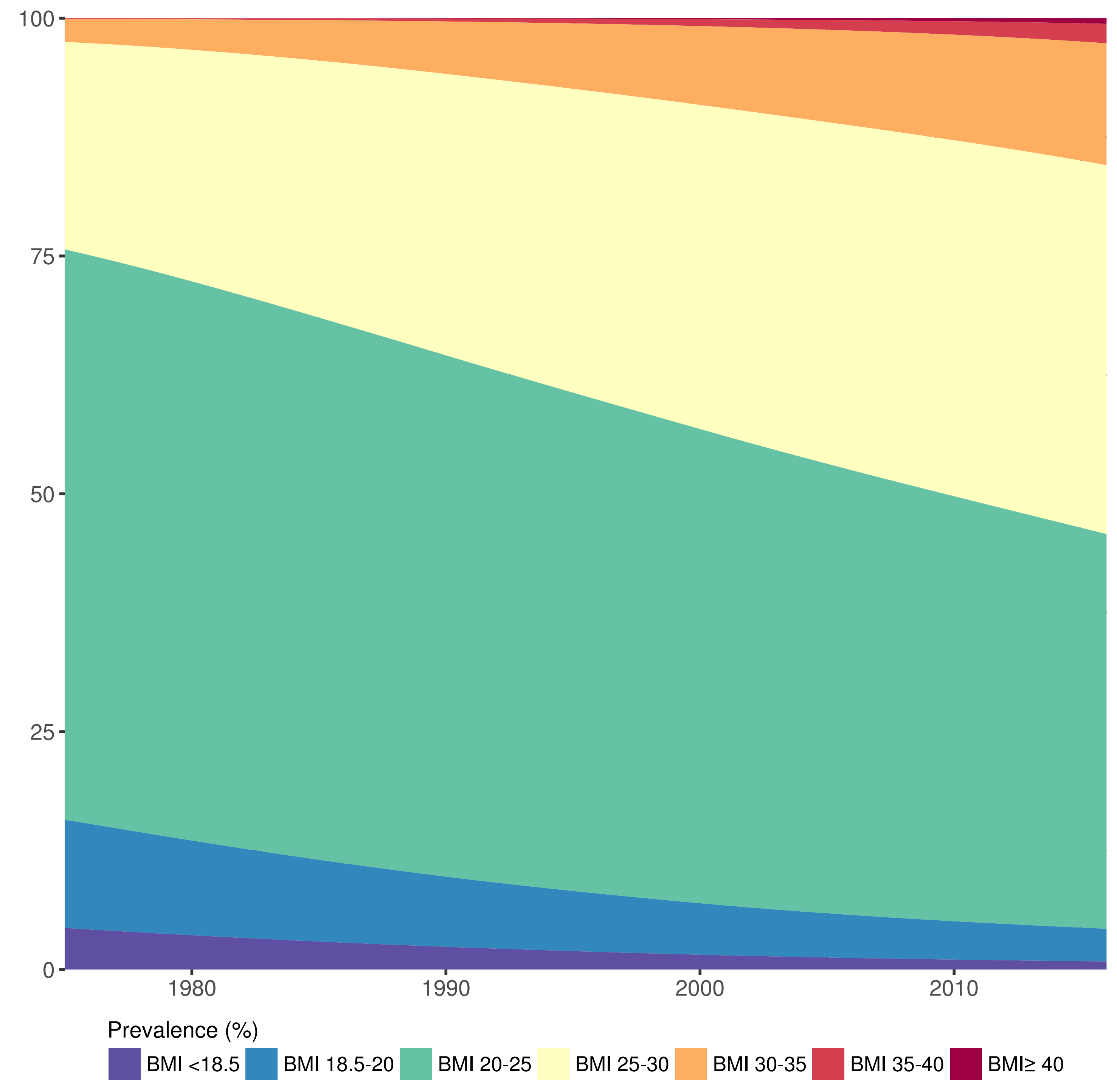
100
75
50
25
0
1980
1990
2000
2010
Prevalence (%)
BMI <18.5
BMI 18.5-20
BMI 20-25
BMI 25-30
BMI 30-35
BMI 35-40
BMI≥ 40

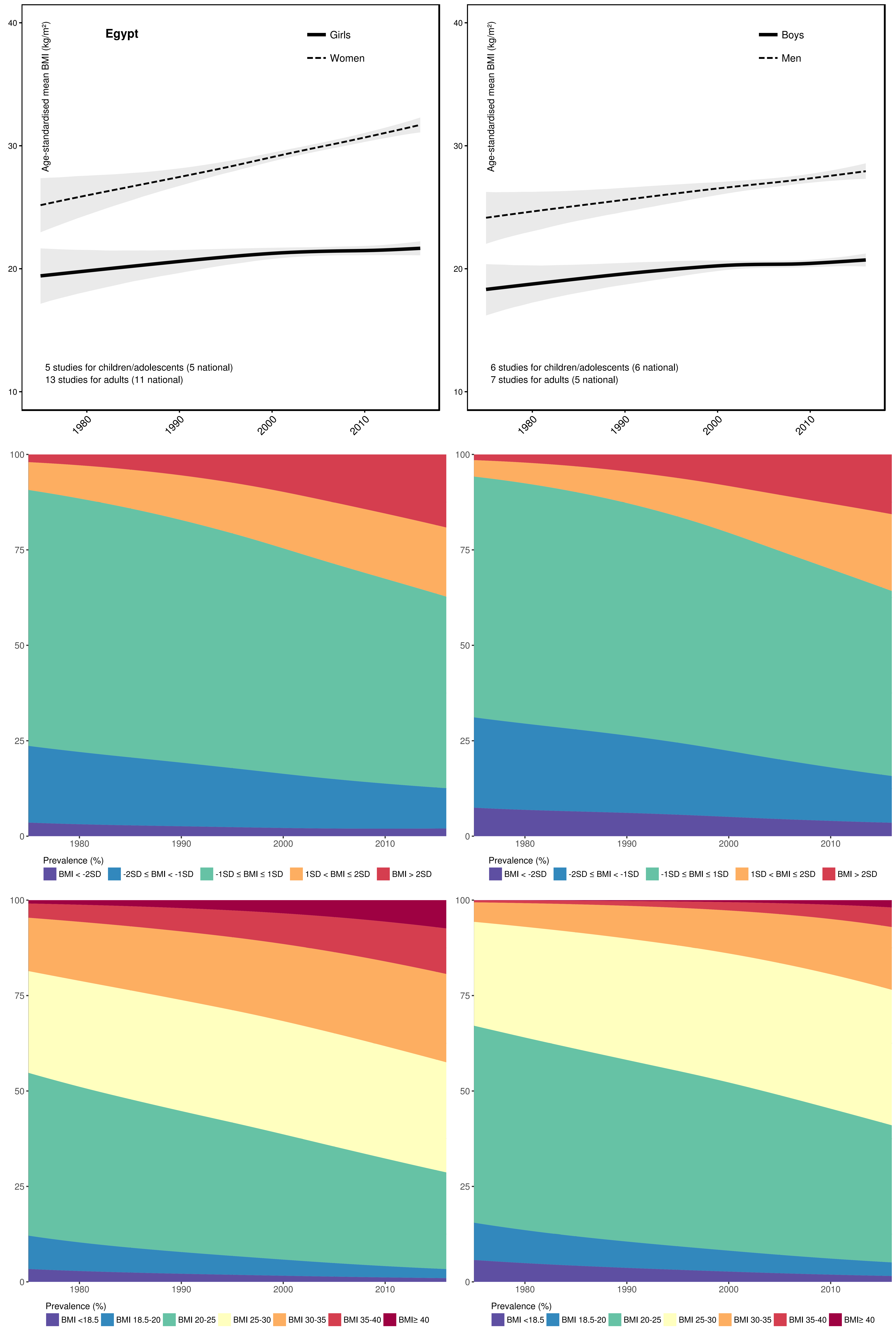
Egypt
Girls
Women
Boys
Men
Age-standardised mean BMI (kg/m²)
5 studies for children/adolescents (5 national)
13 studies for adults (11 national)
6 studies for children/adolescents (6 national)
7 studies for adults (5 national)
10
20
30
40
1980
1990
2000
2010
0
25
50
75
100
Prevalence (%)
BMI < -2SD
-2SD ≤ BMI < -1SD
-1SD ≤ BMI ≤ 1SD
1SD < BMI ≤ 2SD
BMI > 2SD
BMI <18.5
BMI 18.5-20
BMI 20-25
BMI 25-30
BMI 30-35
BMI 35-40
BMI≥ 40

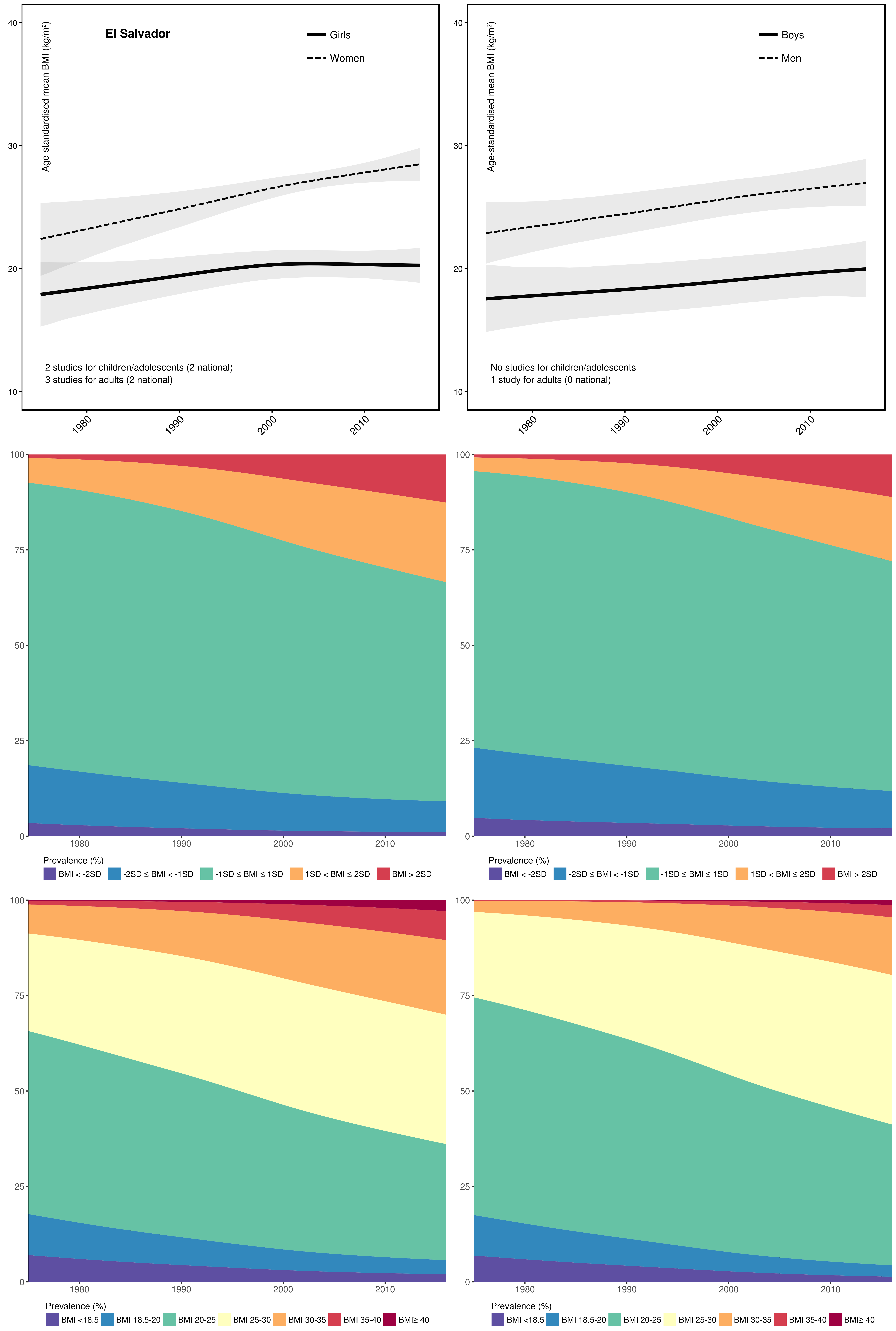
El Salvador
Girls
Women
Boys
Men
Age-standardised mean BMI (kg/m²)
2 studies for children/adolescents (2 national)
3 studies for adults (2 national)
No studies for children/adolescents
1 study for adults (0 national)
Prevalence (%)
BMI < -2SD
-2SD ≤ BMI < -1SD
-1SD ≤ BMI ≤ 1SD
1SD < BMI ≤ 2SD
BMI > 2SD
BMI <18.5
BMI 18.5-20
BMI 20-25
BMI 25-30
BMI 30-35
BMI 35-40
BMI≥ 40

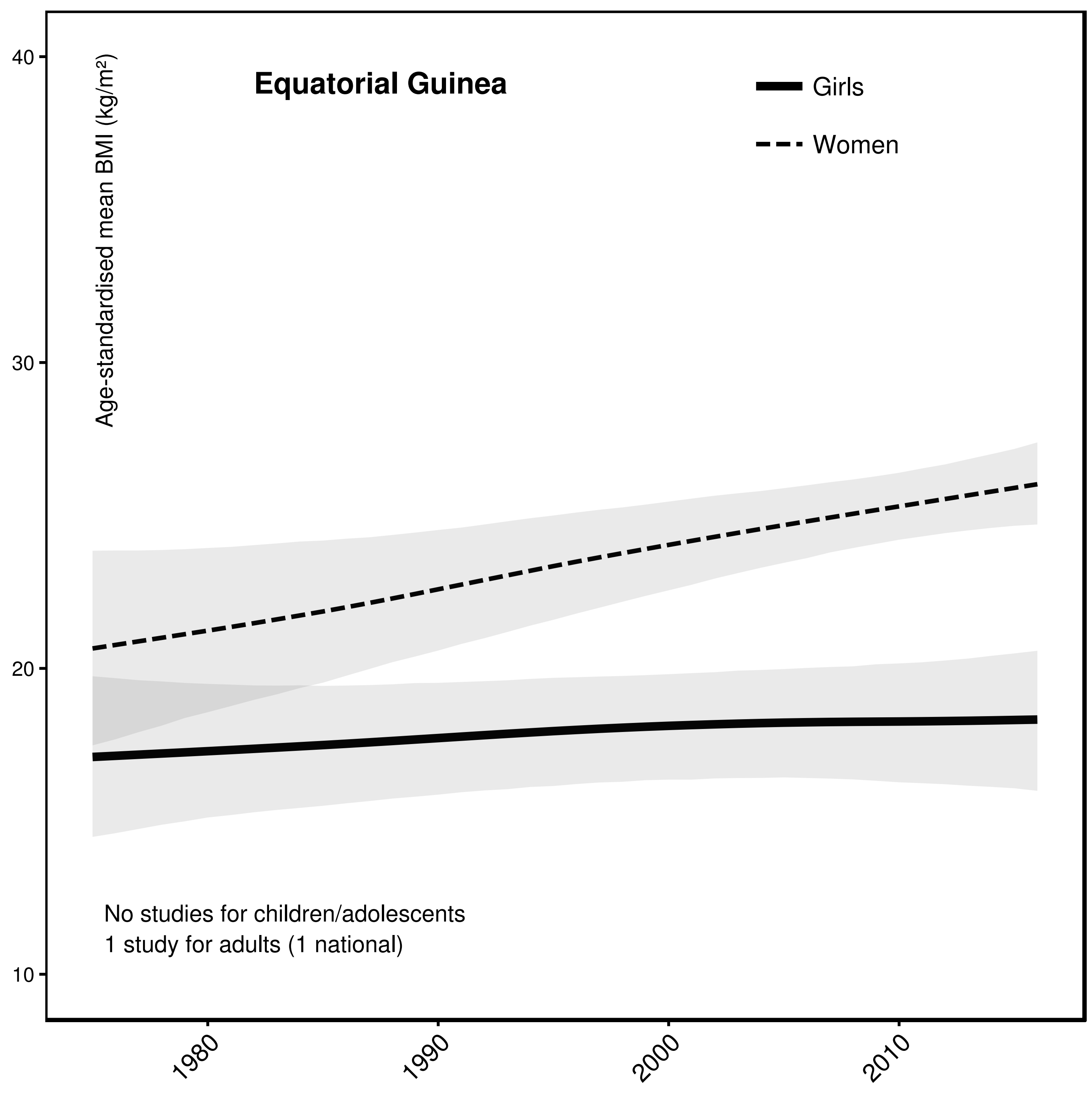
Equatorial Guinea
Girls
Women
Age-standardised mean BMI (kg/m²)
40
30
20
10
No studies for children/adolescents
1 study for adults (1 national)
1980
1990
2000
2010

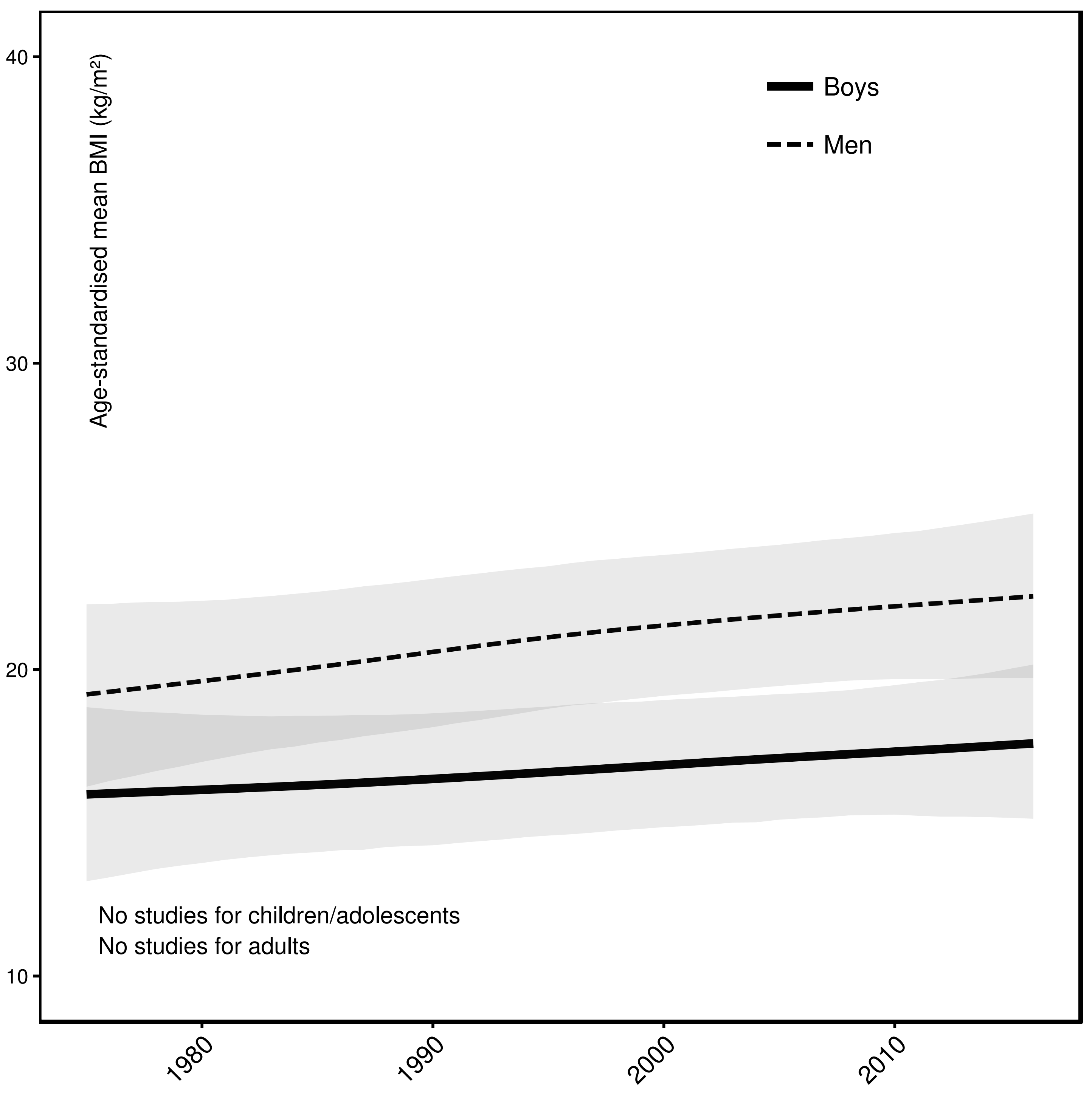
Boys
Men
Age-standardised mean BMI (kg/m²)
40
30
20
10
No studies for children/adolescents
No studies for adults
1980
1990
2000
2010

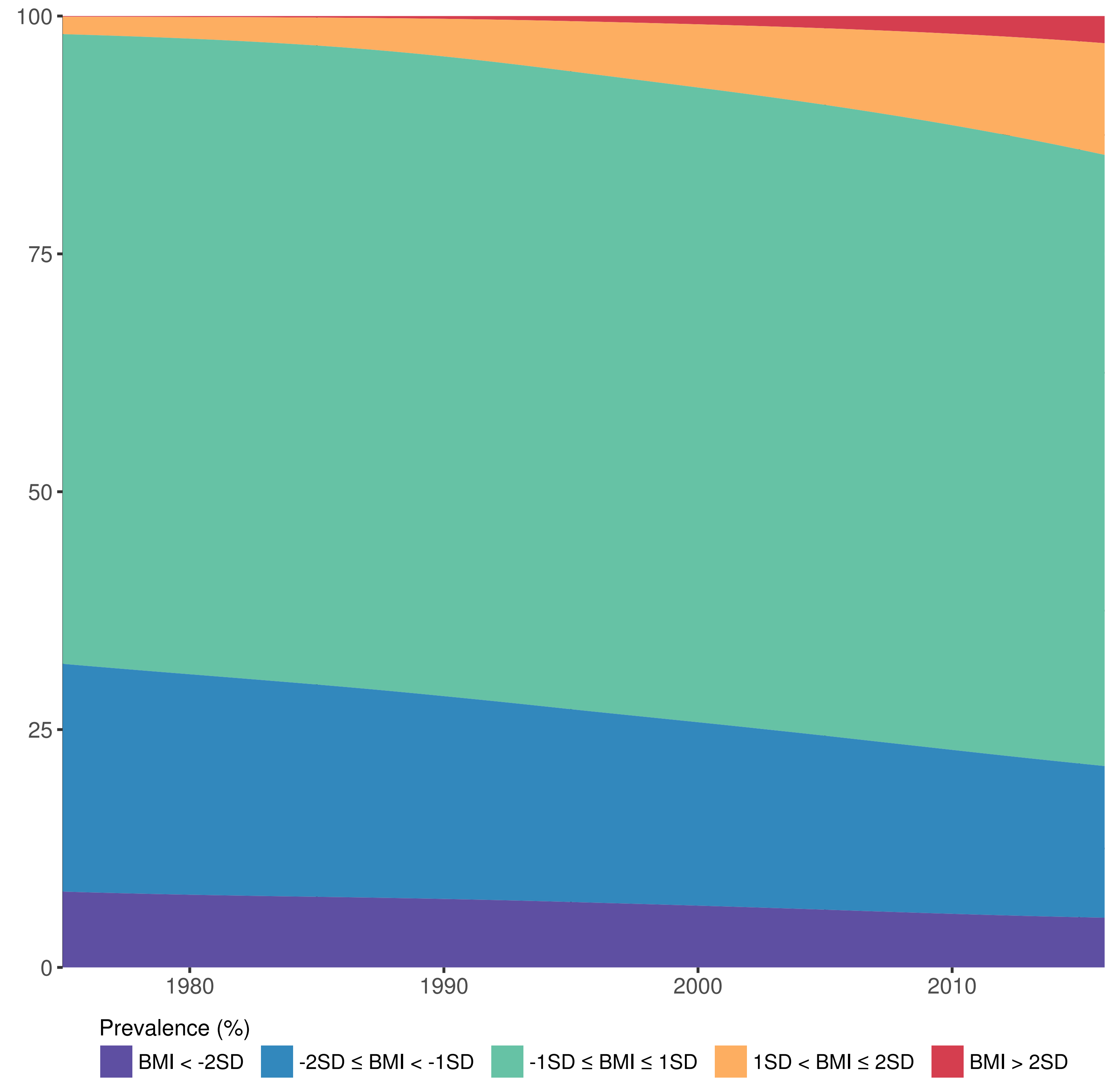
100
75
50
25
0
1980
1990
2000
2010
Prevalence (%)
BMI < -2SD
-2SD ≤ BMI < -1SD
-1SD ≤ BMI ≤ 1SD
1SD < BMI ≤ 2SD
BMI > 2SD

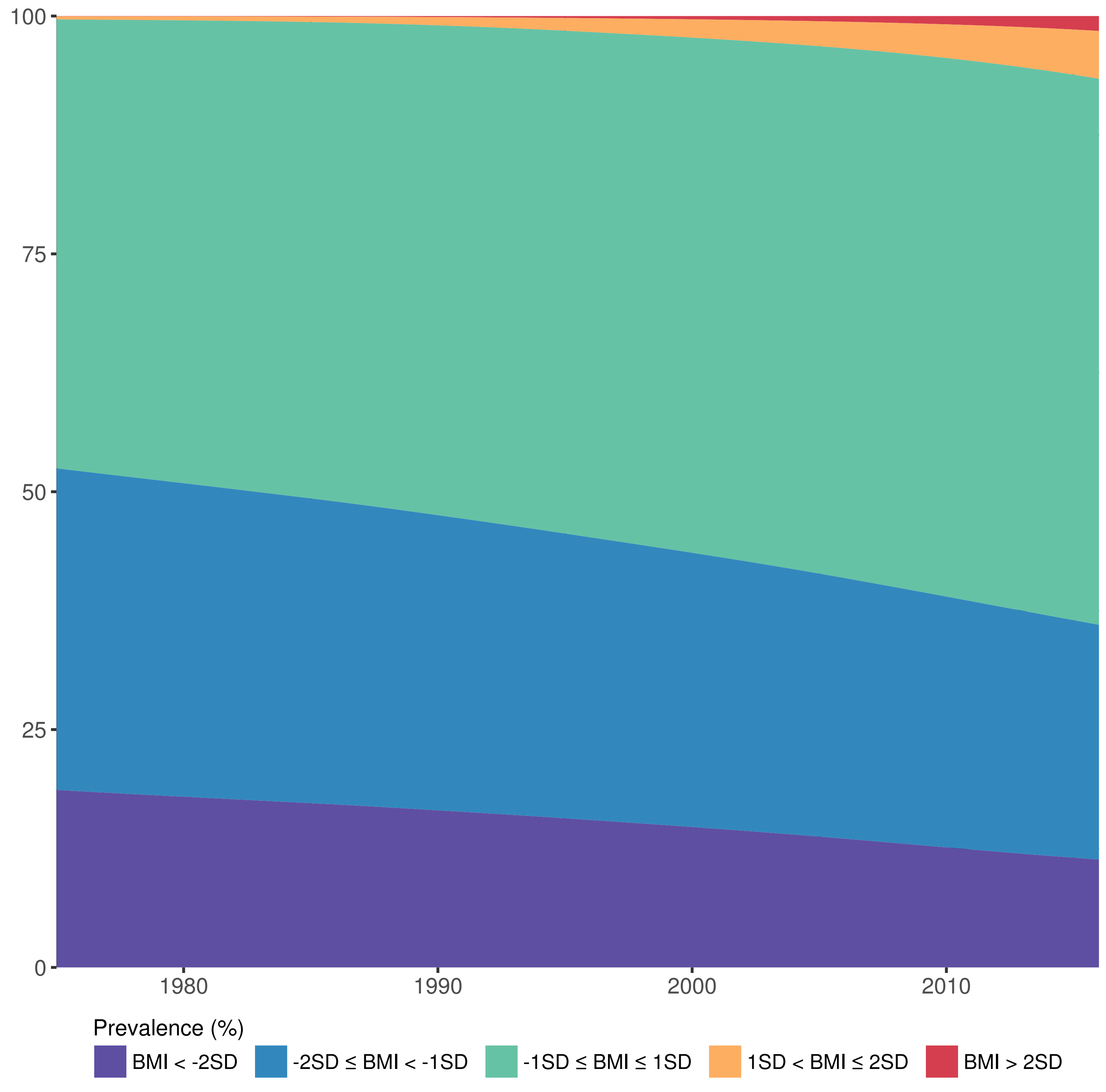
100
75
50
25
0
1980
1990
2000
2010
Prevalence (%)
BMI < -2SD
-2SD ≤ BMI < -1SD
-1SD ≤ BMI ≤ 1SD
1SD < BMI ≤ 2SD
BMI > 2SD

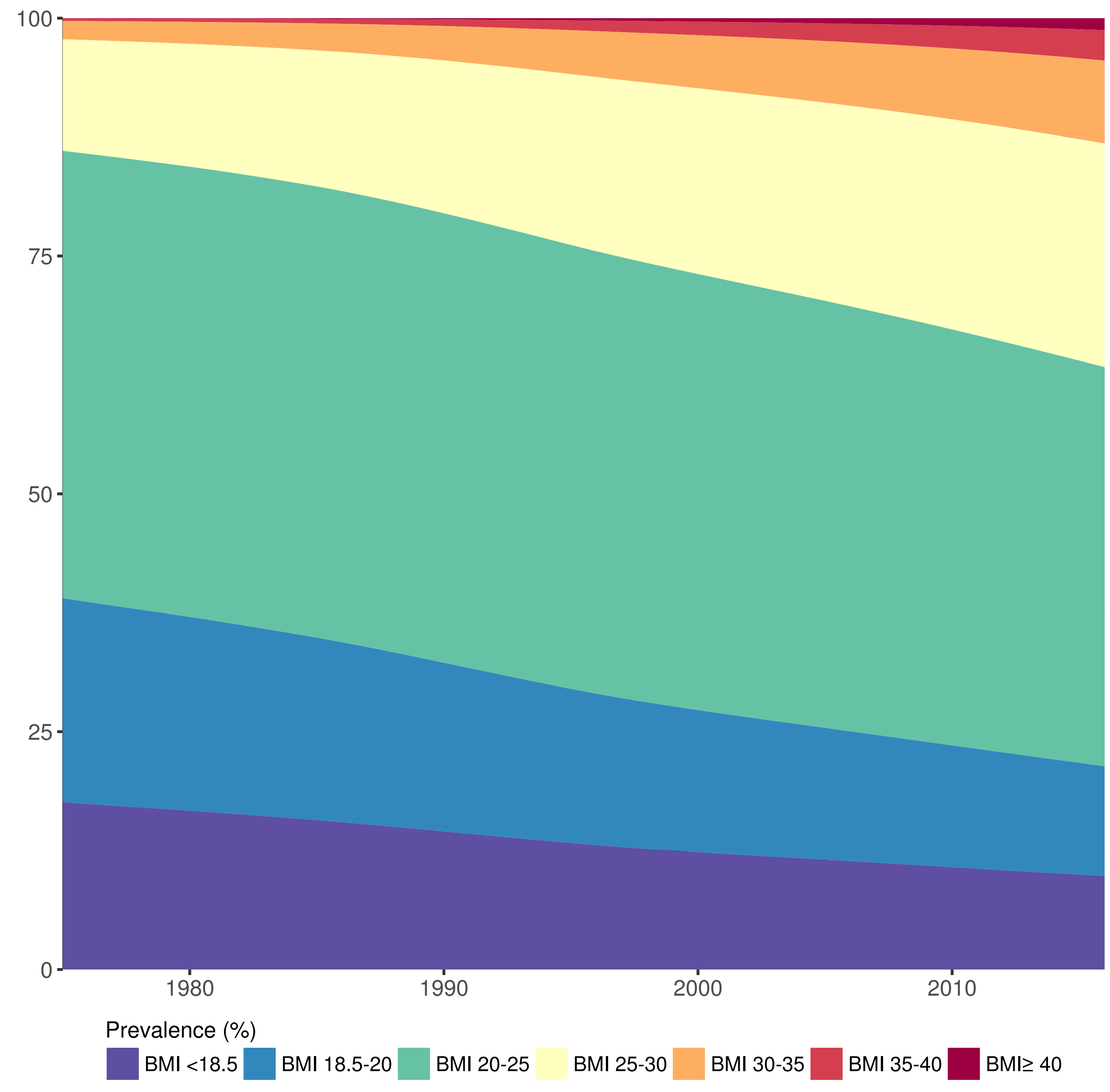
100
75
50
25
0
1980
1990
2000
2010
Prevalence (%)
BMI <18.5
BMI 18.5-20
BMI 20-25
BMI 25-30
BMI 30-35
BMI 35-40
BMI≥ 40

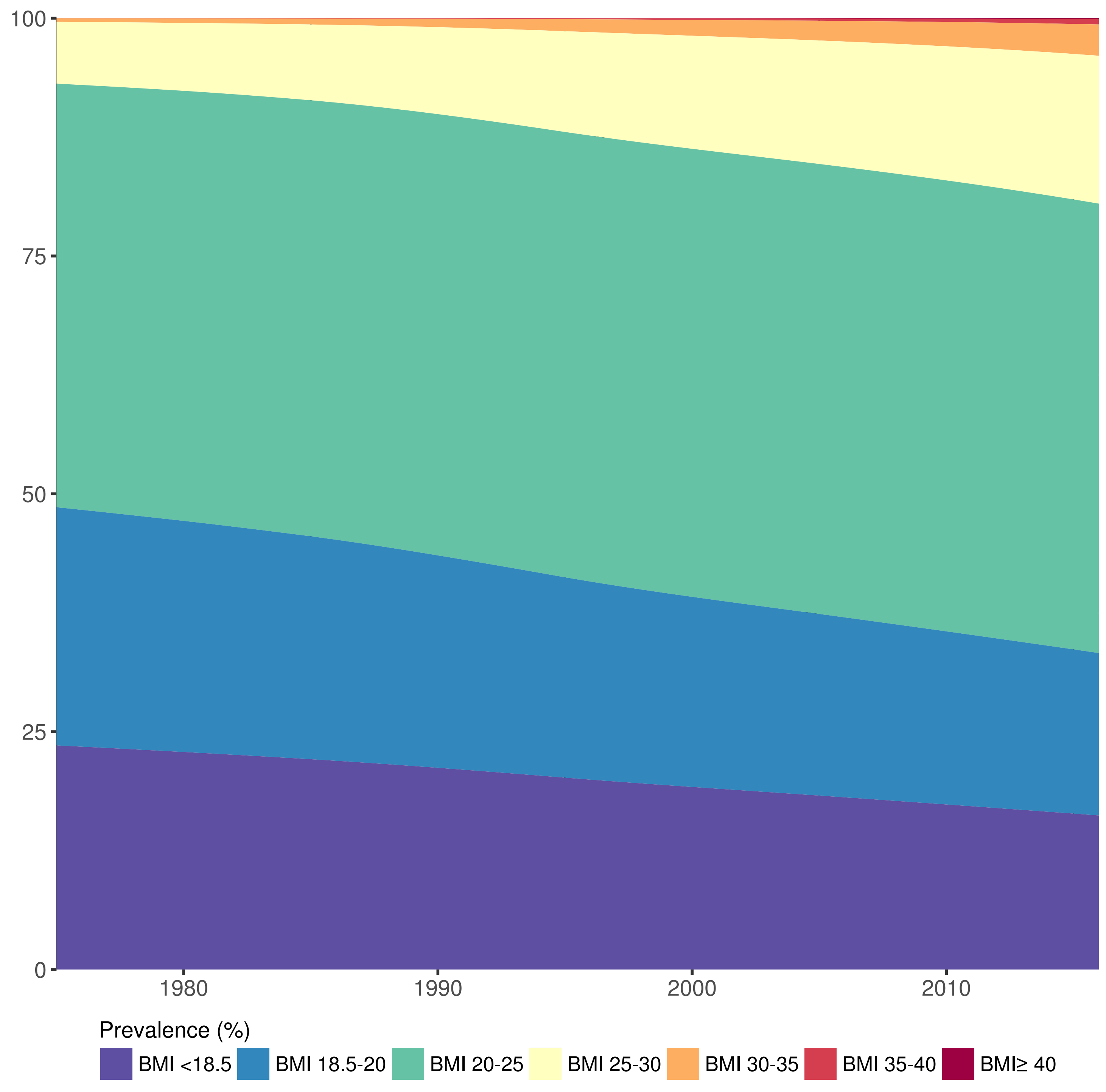
100
75
50
25
0
1980
1990
2000
2010
Prevalence (%)
BMI <18.5
BMI 18.5-20
BMI 20-25
BMI 25-30
BMI 30-35
BMI 35-40
BMI≥ 40

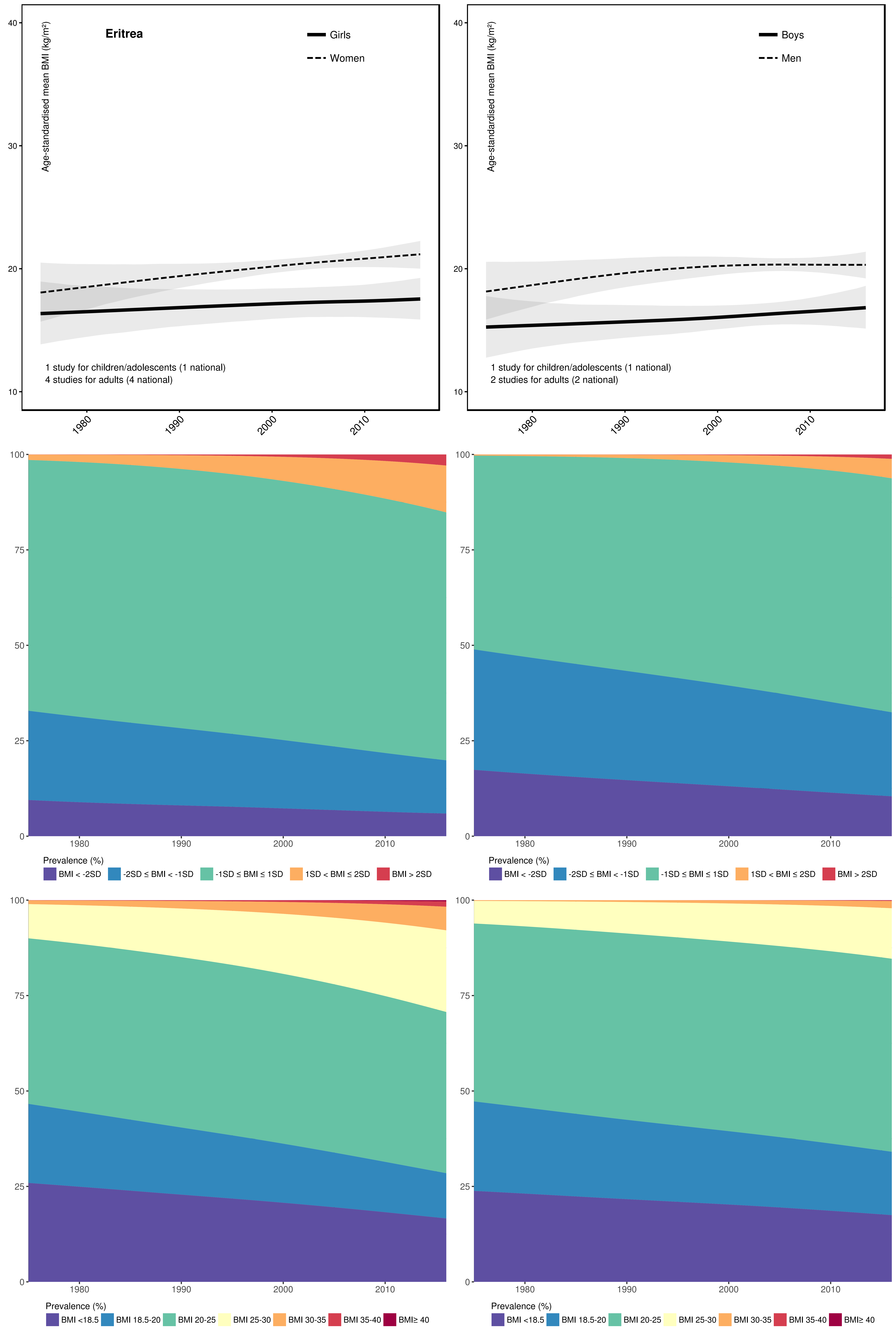
Eritrea
Girls
Women
Age-standardised mean BMI (kg/m²)
1 study for children/adolescents (1 national)
4 studies for adults (4 national)
Boys
Men
1 study for children/adolescents (1 national)
2 studies for adults (2 national)
1980
1990
2000
2010
Prevalence (%)
BMI < -2SD
-2SD ≤ BMI < -1SD
-1SD ≤ BMI ≤ 1SD
1SD < BMI ≤ 2SD
BMI > 2SD
BMI <18.5
BMI 18.5-20
BMI 20-25
BMI 25-30
BMI 30-35
BMI 35-40
BMI≥ 40

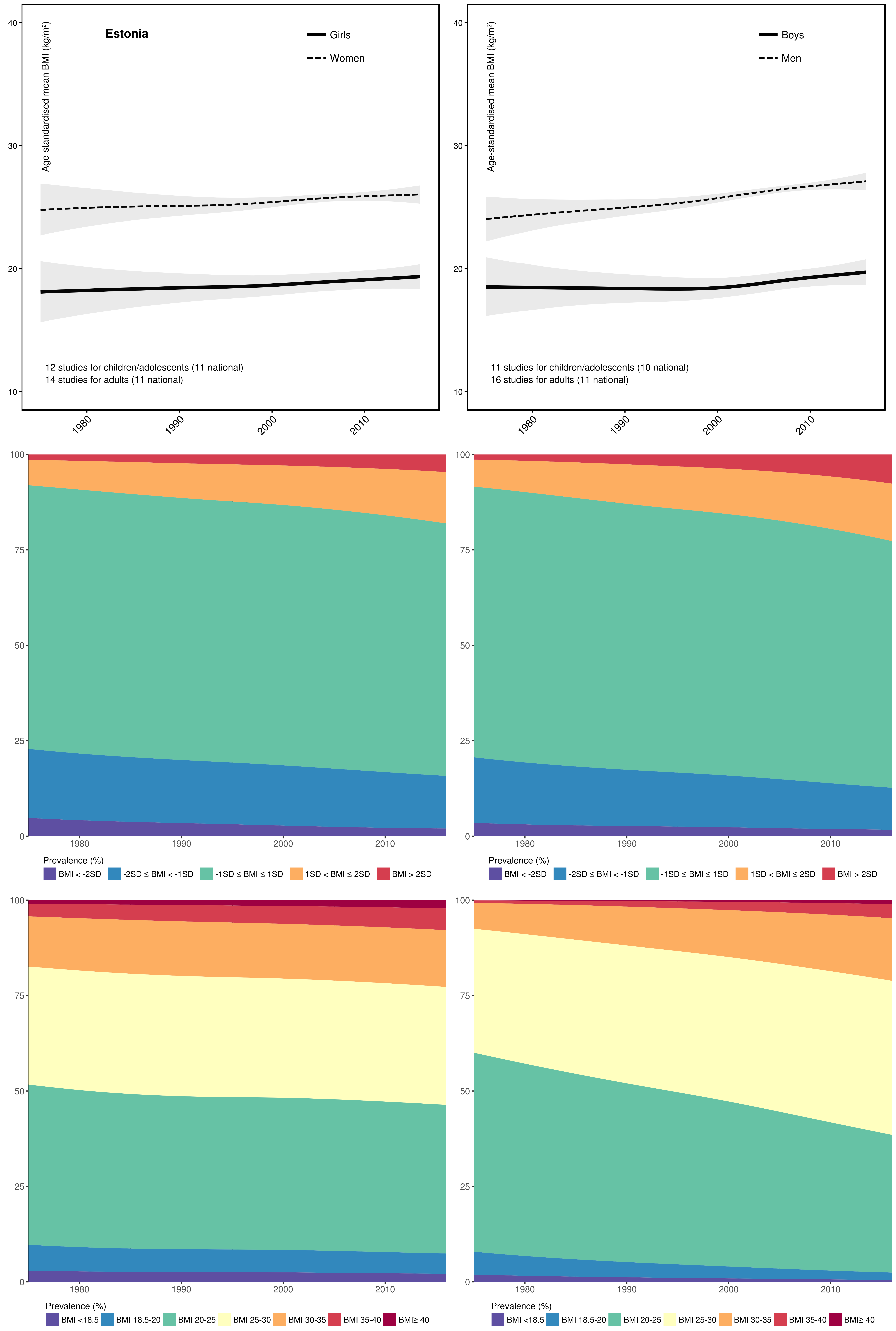
Estonia
Girls
Women
Boys
Men
Age-standardised mean BMI (kg/m²)
12 studies for children/adolescents (11 national)
14 studies for adults (11 national)
11 studies for children/adolescents (10 national)
16 studies for adults (11 national)
Prevalence (%)
BMI < -2SD
-2SD ≤ BMI < -1SD
-1SD ≤ BMI ≤ 1SD
1SD < BMI ≤ 2SD
BMI > 2SD
BMI <18.5
BMI 18.5-20
BMI 20-25
BMI 25-30
BMI 30-35
BMI 35-40
BMI≥ 40

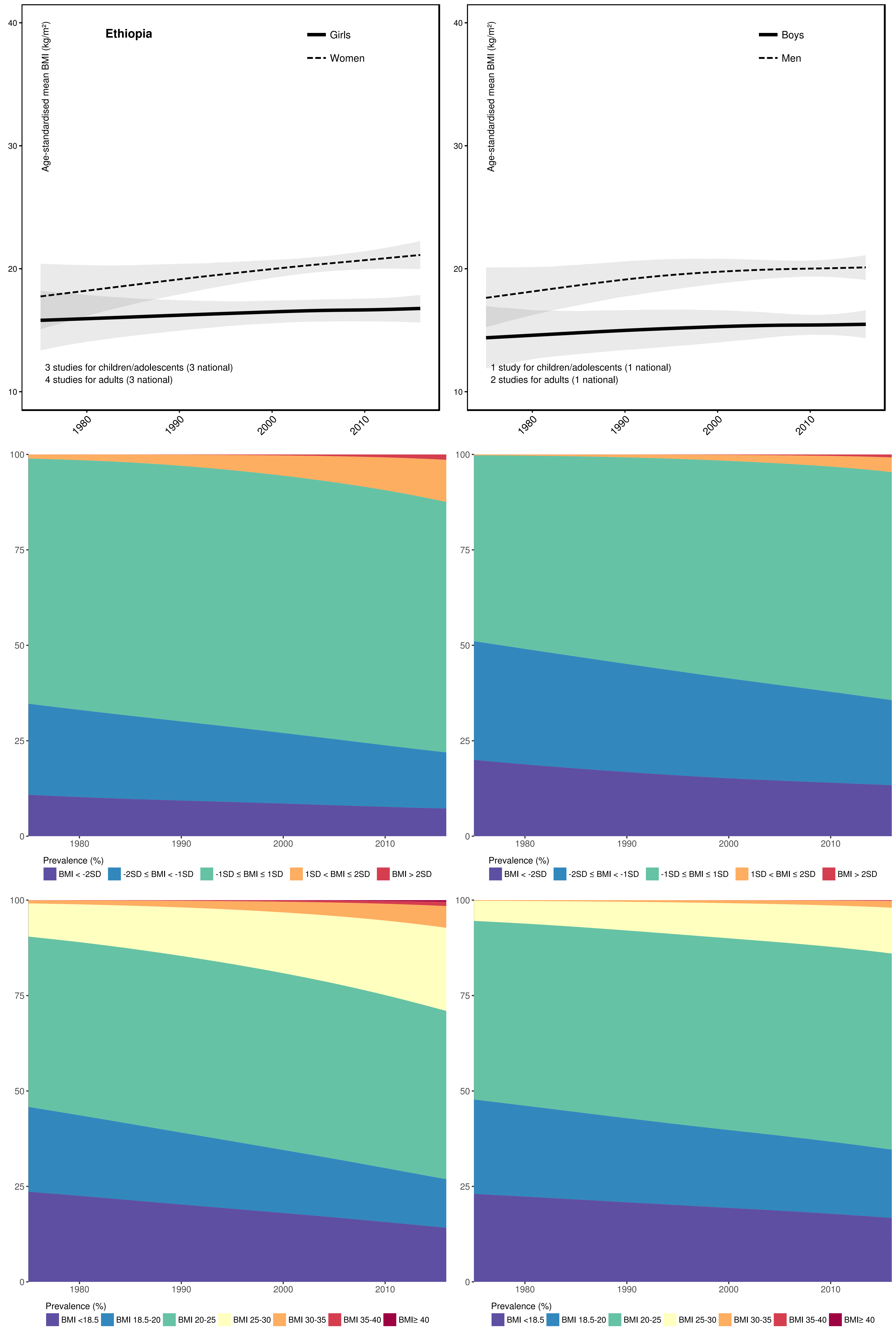
Ethiopia
Girls
Women
Boys
Men
Age-standardised mean BMI (kg/m²)
3 studies for children/adolescents (3 national)
4 studies for adults (3 national)
1 study for children/adolescents (1 national)
2 studies for adults (1 national)
1980
1990
2000
2010
Prevalence (%)
BMI < -2SD
-2SD ≤ BMI < -1SD
-1SD ≤ BMI ≤ 1SD
1SD < BMI ≤ 2SD
BMI > 2SD
BMI <18.5
BMI 18.5-20
BMI 20-25
BMI 25-30
BMI 30-35
BMI 35-40
BMI≥ 40

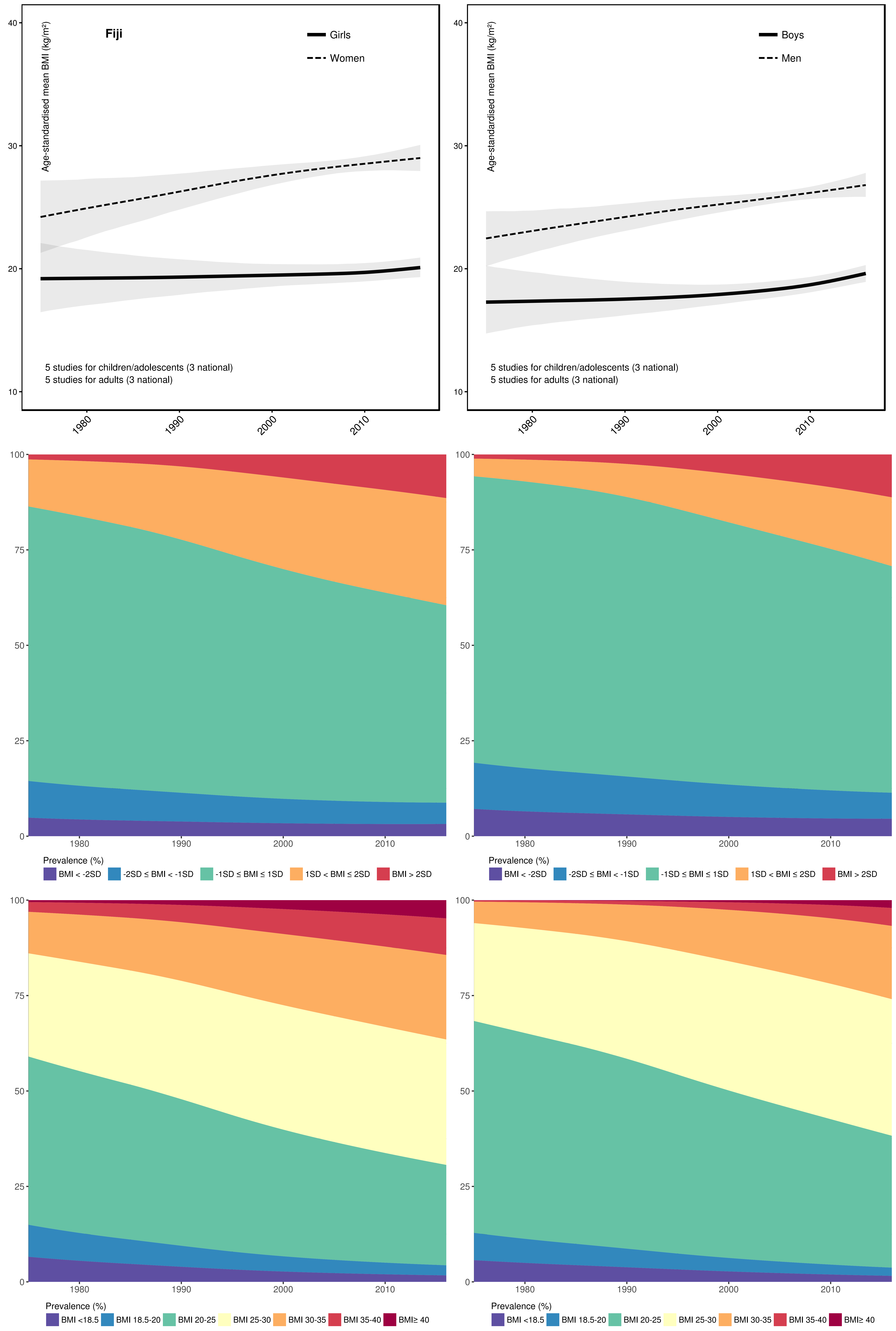

Fiji
Girls
Women
Age-standardised mean BMI (kg/m²)
5 studies for children/adolescents (3 national)
5 studies for adults (3 national)
Boys
Men
Age-standardised mean BMI (kg/m²)
5 studies for children/adolescents (3 national)
5 studies for adults (3 national)
Prevalence (%)
BMI < -2SD
-2SD ≤ BMI < -1SD
-1SD ≤ BMI ≤ 1SD
1SD < BMI ≤ 2SD
BMI > 2SD
Prevalence (%)
BMI < -2SD
-2SD ≤ BMI < -1SD
-1SD ≤ BMI ≤ 1SD
1SD < BMI ≤ 2SD
BMI > 2SD
Prevalence (%)
BMI <18.5
BMI 18.5-20
BMI 20-25
BMI 25-30
BMI 30-35
BMI 35-40
BMI≥ 40
Prevalence (%)
BMI <18.5
BMI 18.5-20
BMI 20-25
BMI 25-30
BMI 30-35
BMI 35-40
BMI≥ 40

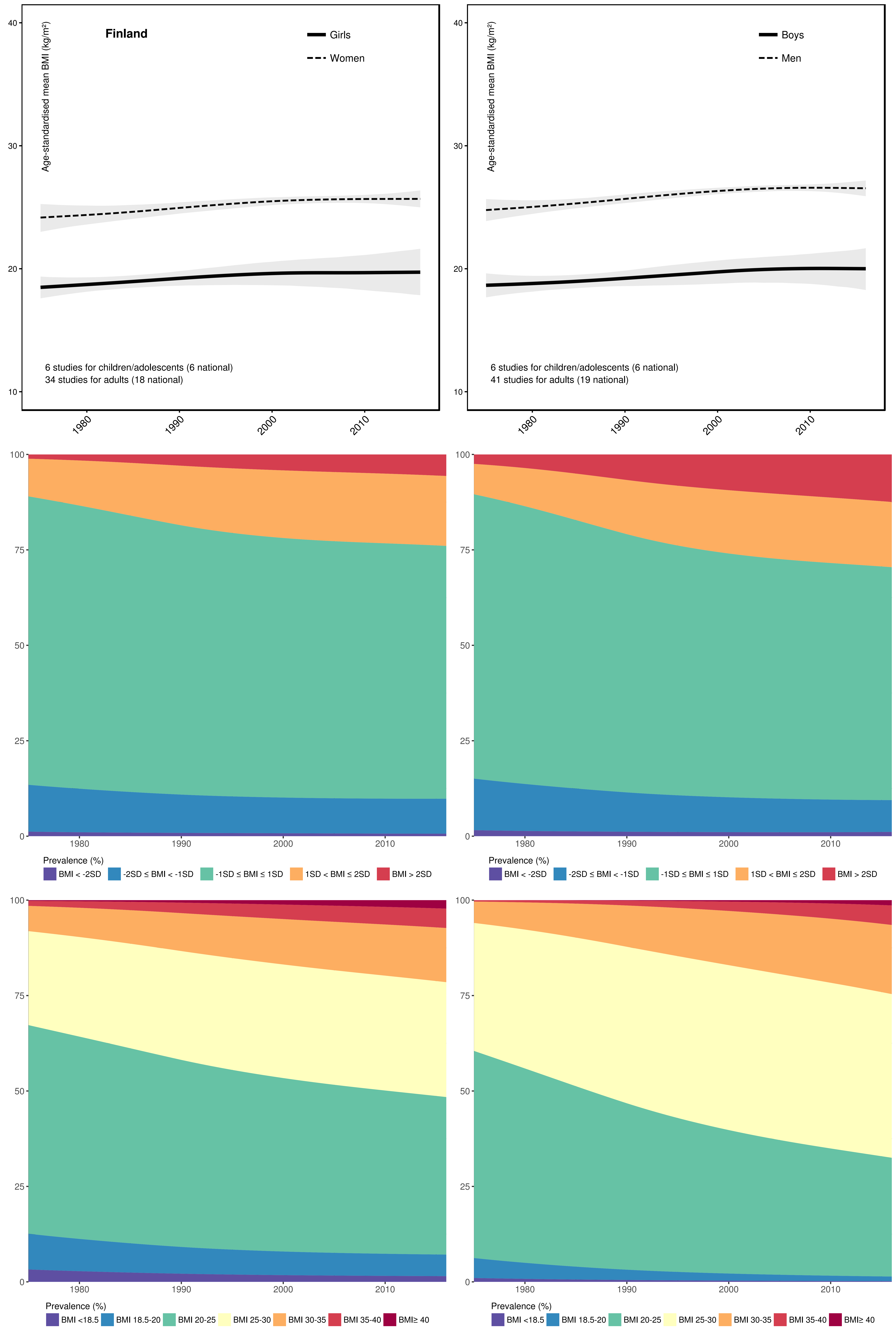
Finland
Girls
Women
Boys
Men
Age-standardised mean BMI (kg/m²)
6 studies for children/adolescents (6 national)
34 studies for adults (18 national)
6 studies for children/adolescents (6 national)
41 studies for adults (19 national)
Prevalence (%)
BMI < -2SD
-2SD ≤ BMI < -1SD
-1SD ≤ BMI ≤ 1SD
1SD < BMI ≤ 2SD
BMI > 2SD
BMI <18.5
BMI 18.5-20
BMI 20-25
BMI 25-30
BMI 30-35
BMI 35-40
BMI≥ 40

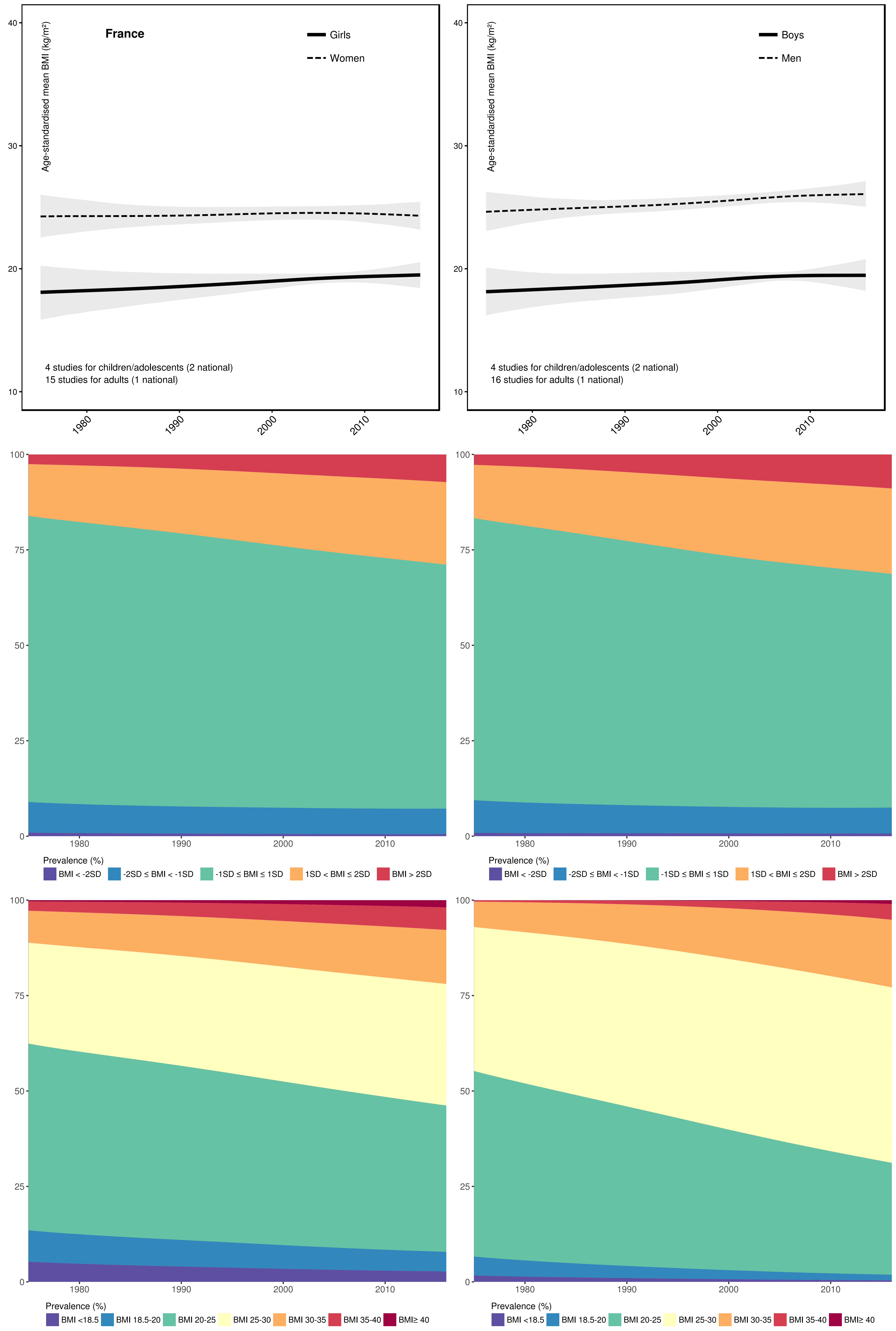
France
Girls
Women
Boys
Men
Age-standardised mean BMI (kg/m²)
4 studies for children/adolescents (2 national)
15 studies for adults (1 national)
4 studies for children/adolescents (2 national)
16 studies for adults (1 national)
Prevalence (%)
BMI < -2SD
-2SD ≤ BMI < -1SD
-1SD ≤ BMI ≤ 1SD
1SD < BMI ≤ 2SD
BMI > 2SD
BMI <18.5
BMI 18.5-20
BMI 20-25
BMI 25-30
BMI 30-35
BMI 35-40
BMI≥ 40

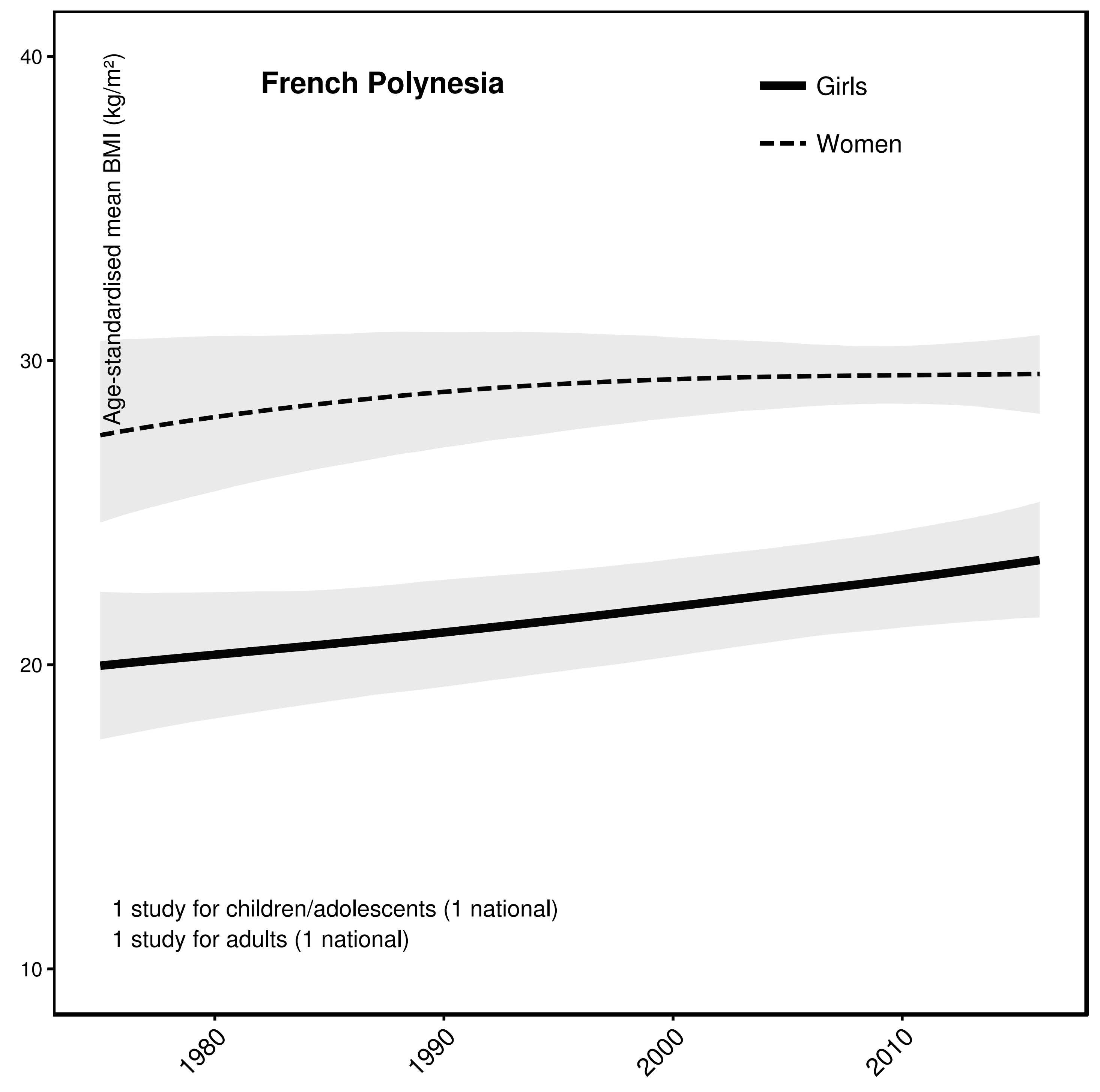
French Polynesia
Girls
Women
Age-standardised mean BMI (kg/m²)
40
30
20
10
1980
1990
2000
2010
1 study for children/adolescents (1 national)
1 study for adults (1 national)

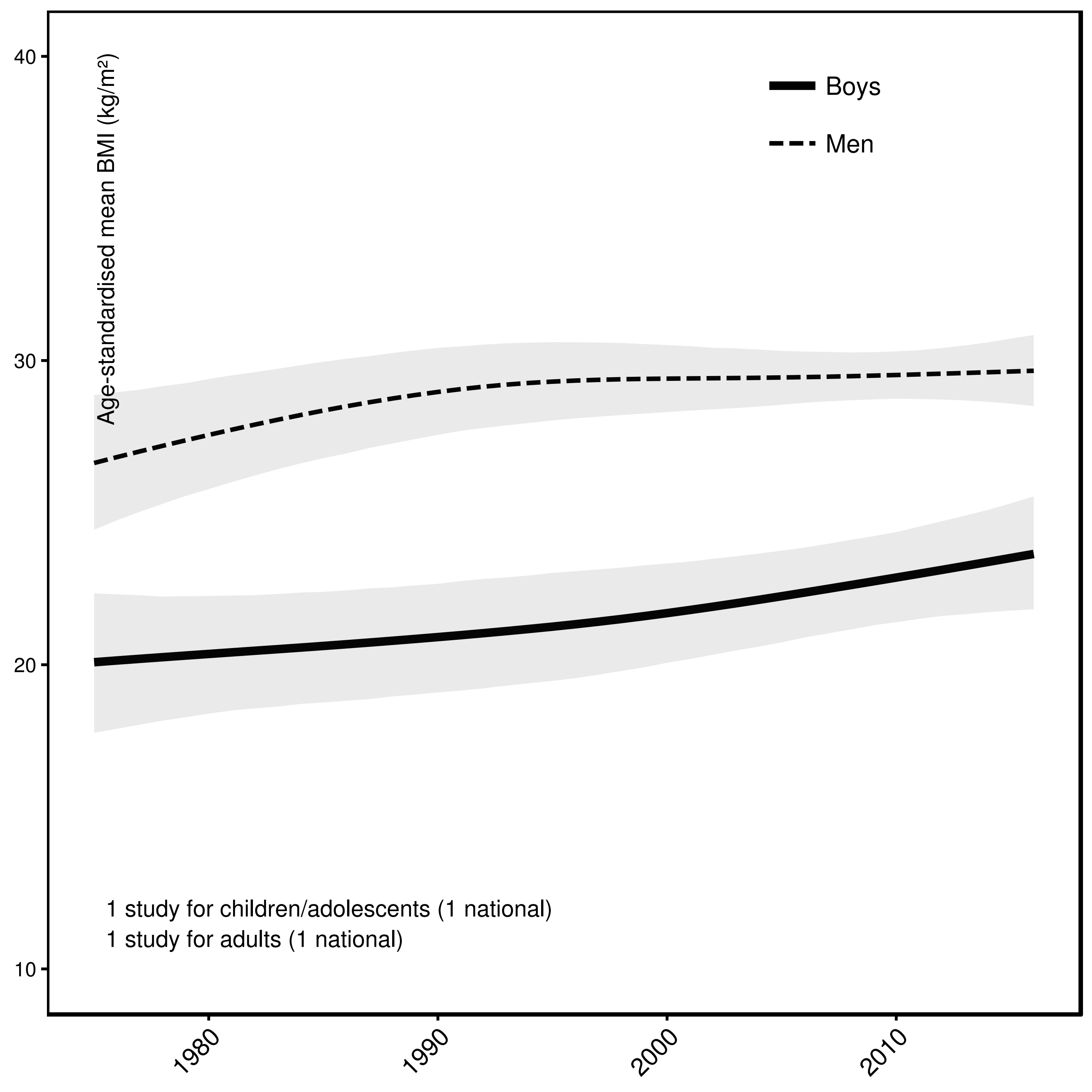
Boys
Men
Age-standardised mean BMI (kg/m²)
40
30
20
10
1980
1990
2000
2010
1 study for children/adolescents (1 national)
1 study for adults (1 national)

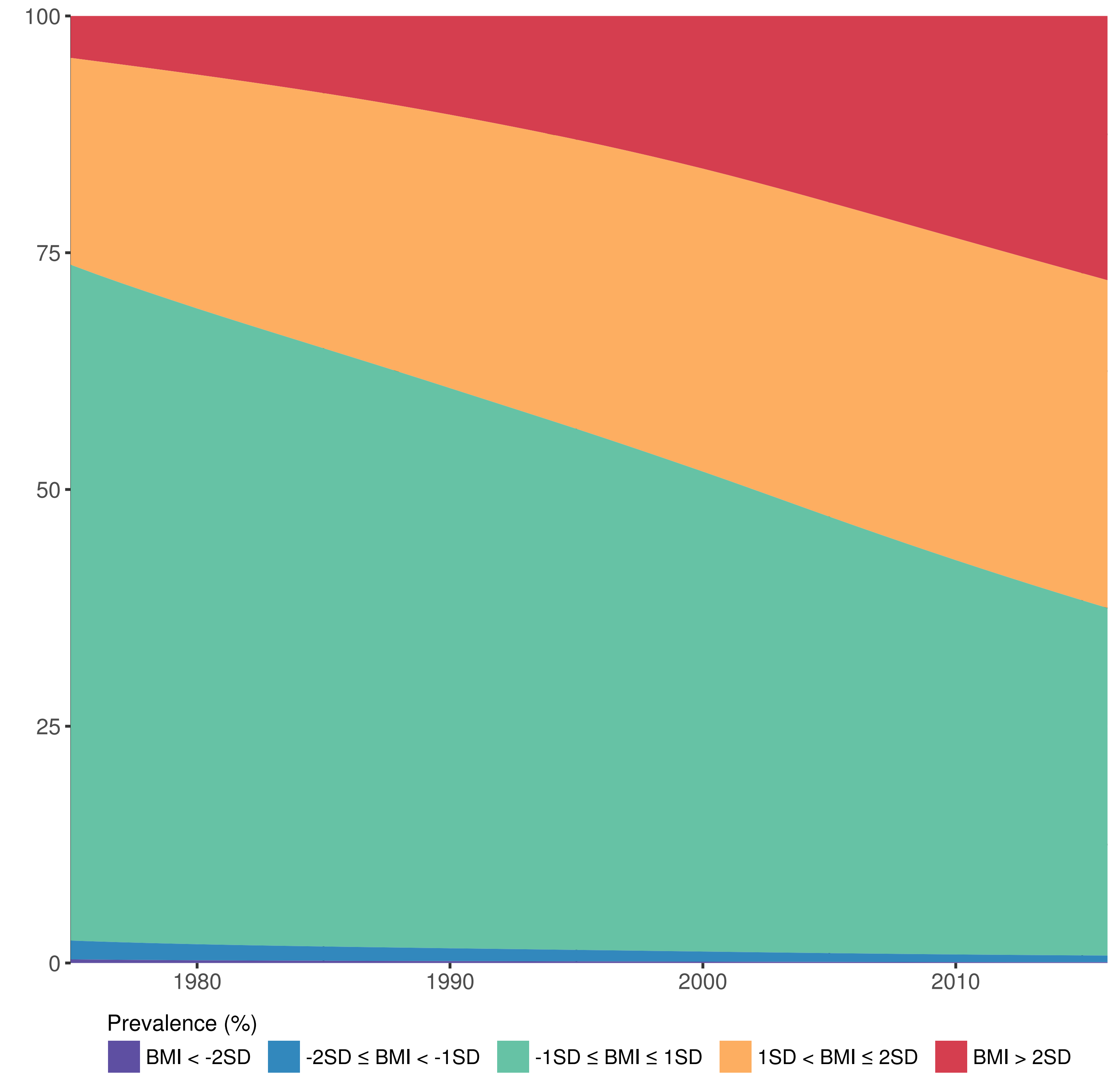
100
75
50
25
0
1980
1990
2000
2010
Prevalence (%)
BMI < -2SD
-2SD ≤ BMI < -1SD
-1SD ≤ BMI ≤ 1SD
1SD < BMI ≤ 2SD
BMI > 2SD

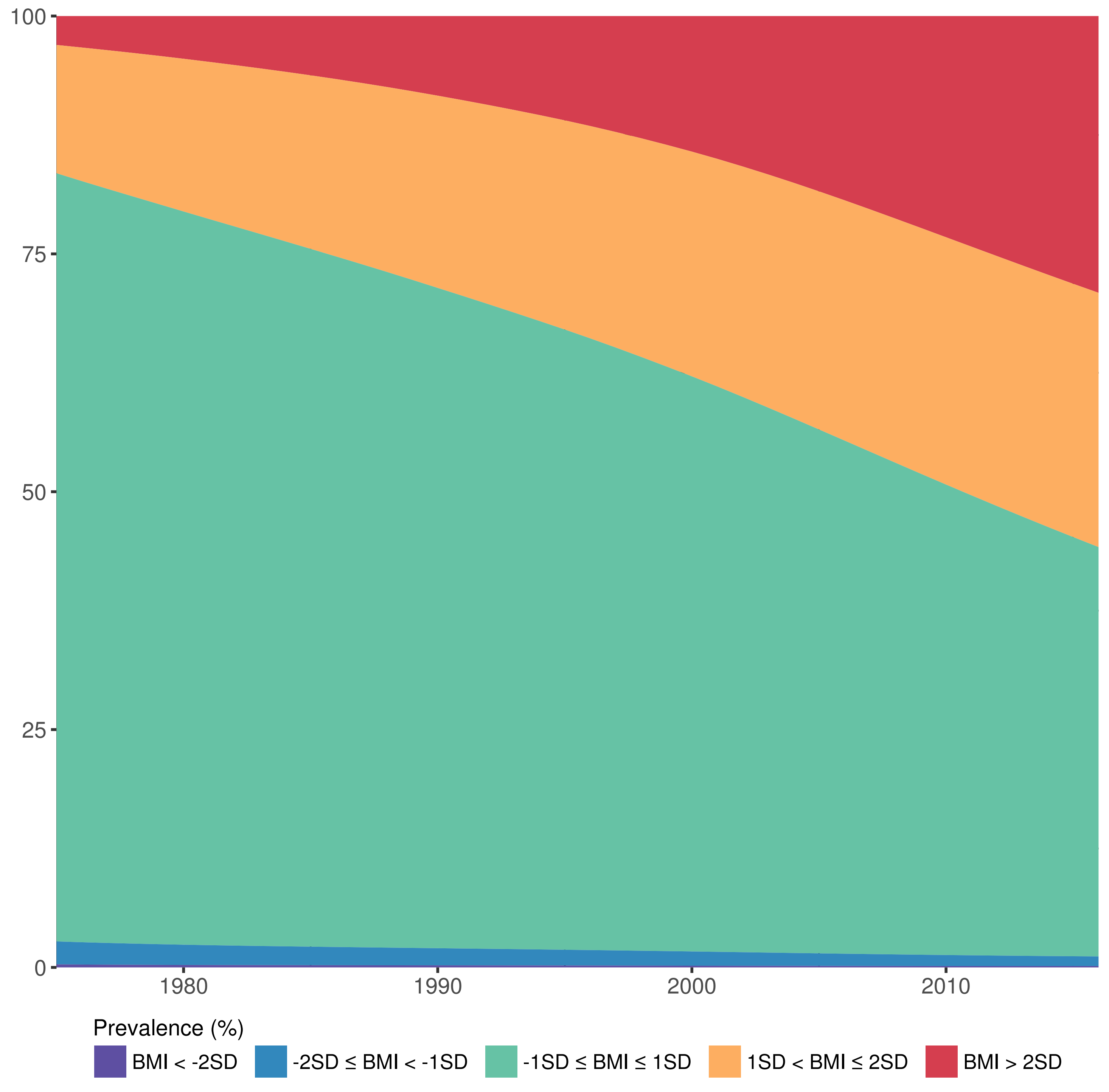
100
75
50
25
0
1980
1990
2000
2010
Prevalence (%)
BMI < -2SD
-2SD ≤ BMI < -1SD
-1SD ≤ BMI ≤ 1SD
1SD < BMI ≤ 2SD
BMI > 2SD

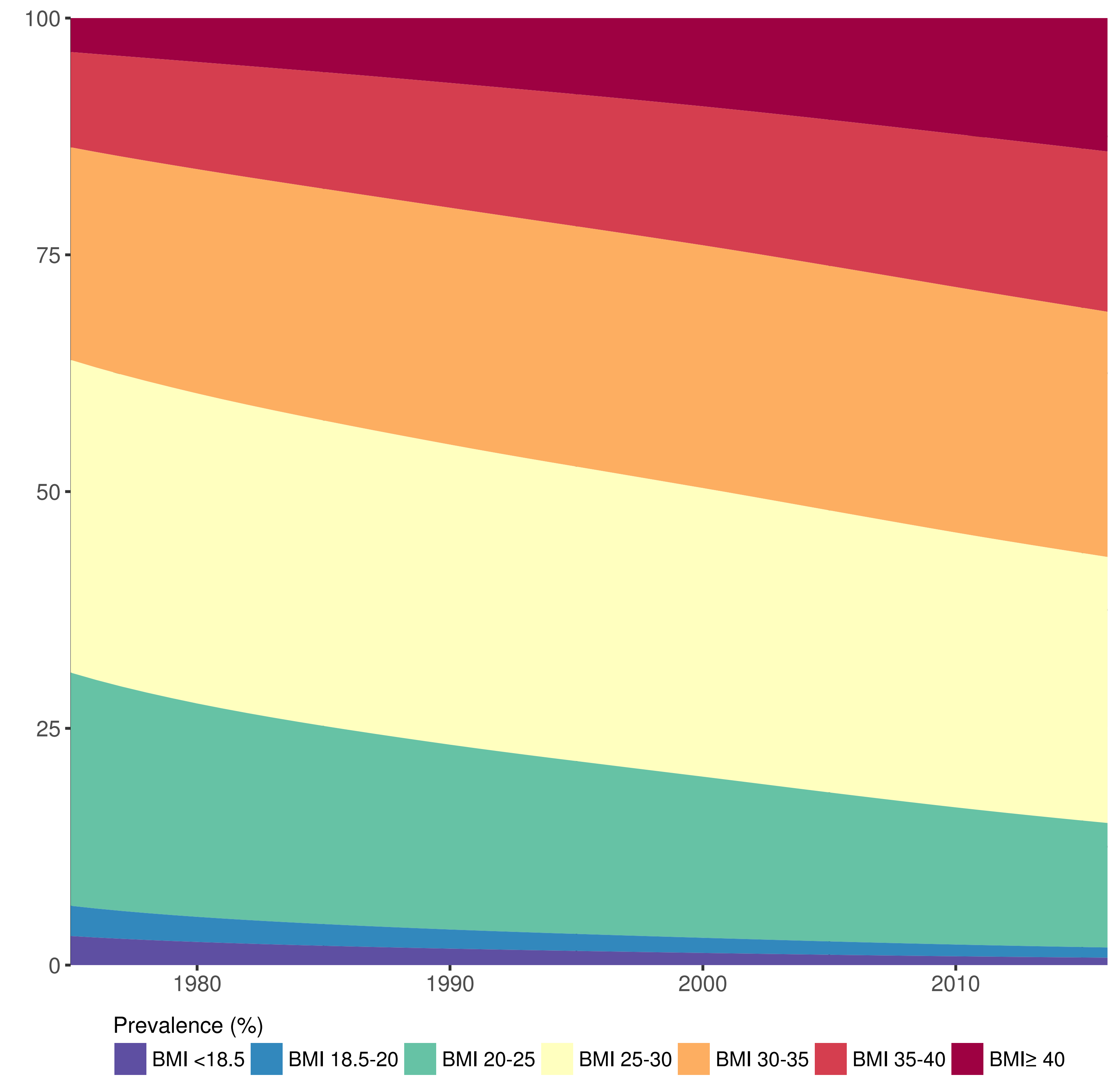
100
75
50
25
0
1980
1990
2000
2010
Prevalence (%)
BMI <18.5
BMI 18.5-20
BMI 20-25
BMI 25-30
BMI 30-35
BMI 35-40
BMI≥ 40

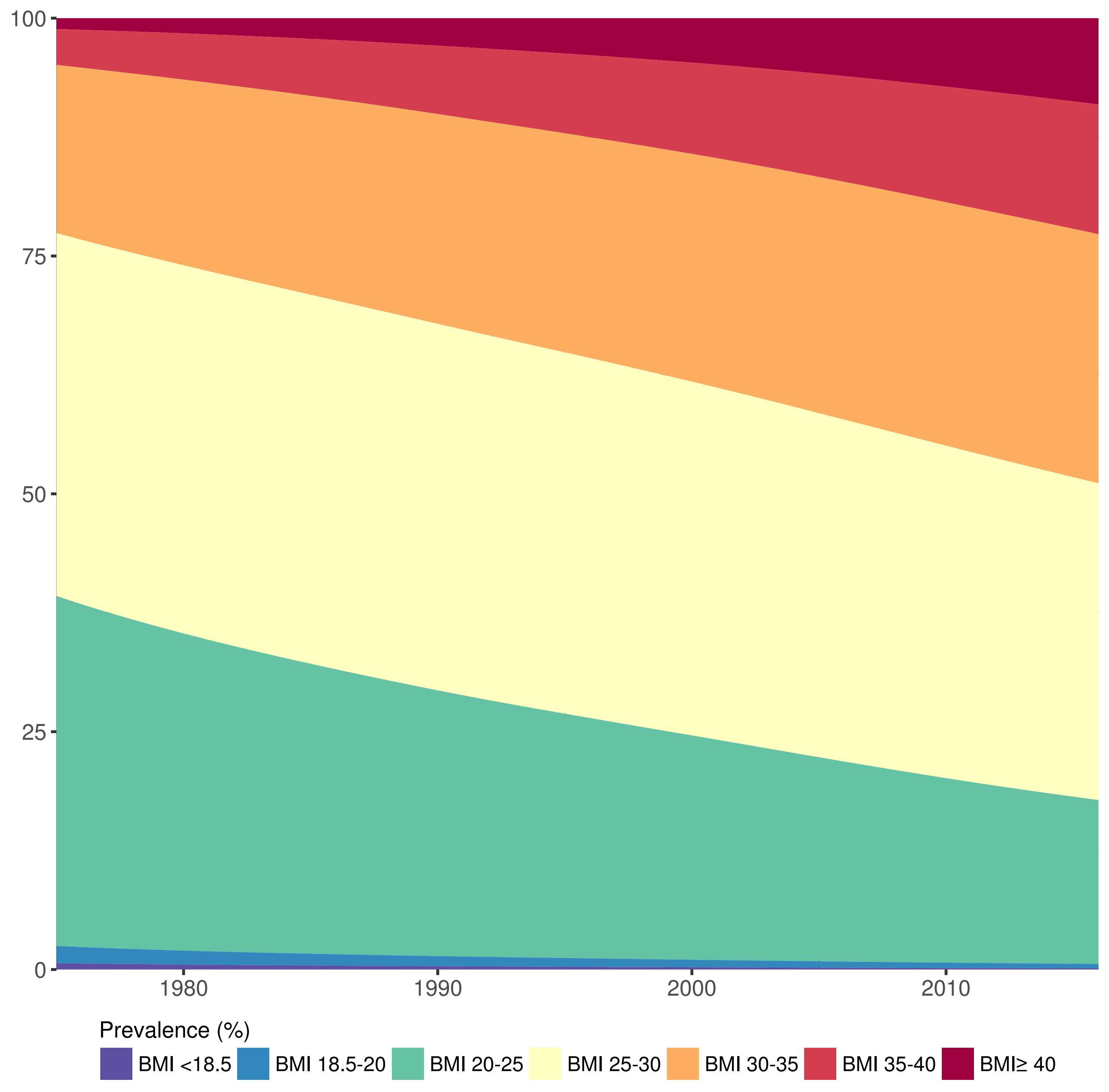
100
75
50
25
0
1980
1990
2000
2010
Prevalence (%)
BMI <18.5
BMI 18.5-20
BMI 20-25
BMI 25-30
BMI 30-35
BMI 35-40
BMI≥ 40

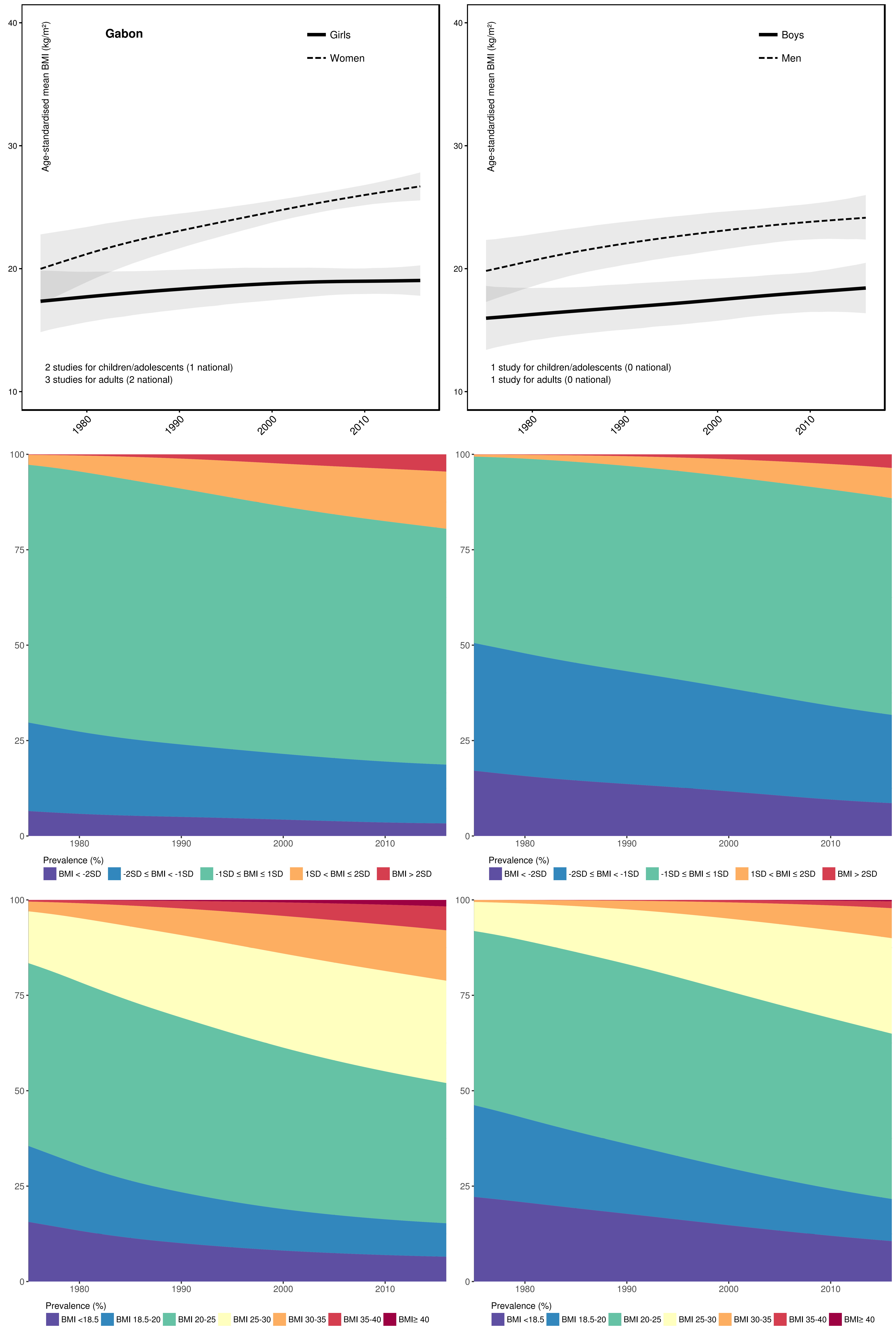
Gabon
Girls
Women
Boys
Men
Age-standardised mean BMI (kg/m²)
2 studies for children/adolescents (1 national)
3 studies for adults (2 national)
1 study for children/adolescents (0 national)
1 study for adults (0 national)
Prevalence (%)
BMI < -2SD
-2SD ≤ BMI < -1SD
-1SD ≤ BMI ≤ 1SD
1SD < BMI ≤ 2SD
BMI > 2SD
BMI <18.5
BMI 18.5-20
BMI 20-25
BMI 25-30
BMI 30-35
BMI 35-40
BMI≥ 40

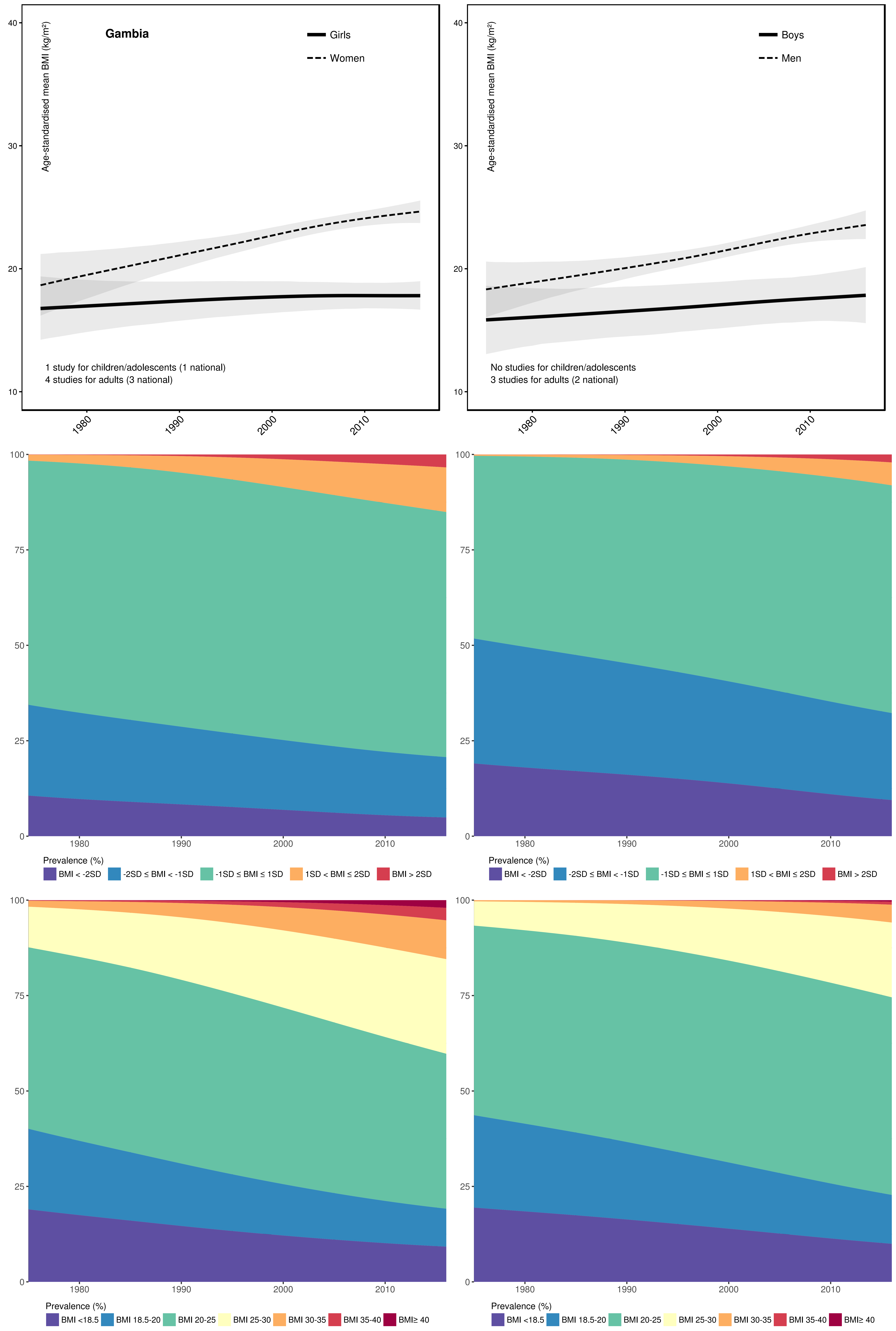
Gambia
Girls
Women
Age-standardised mean BMI (kg/m²)
1 study for children/adolescents (1 national)
4 studies for adults (3 national)
Boys
Men
Age-standardised mean BMI (kg/m²)
No studies for children/adolescents
3 studies for adults (2 national)
Prevalence (%)
BMI < -2SD
-2SD ≤ BMI < -1SD
-1SD ≤ BMI ≤ 1SD
1SD < BMI ≤ 2SD
BMI > 2SD
Prevalence (%)
BMI <18.5
BMI 18.5-20
BMI 20-25
BMI 25-30
BMI 30-35
BMI 35-40
BMI≥ 40

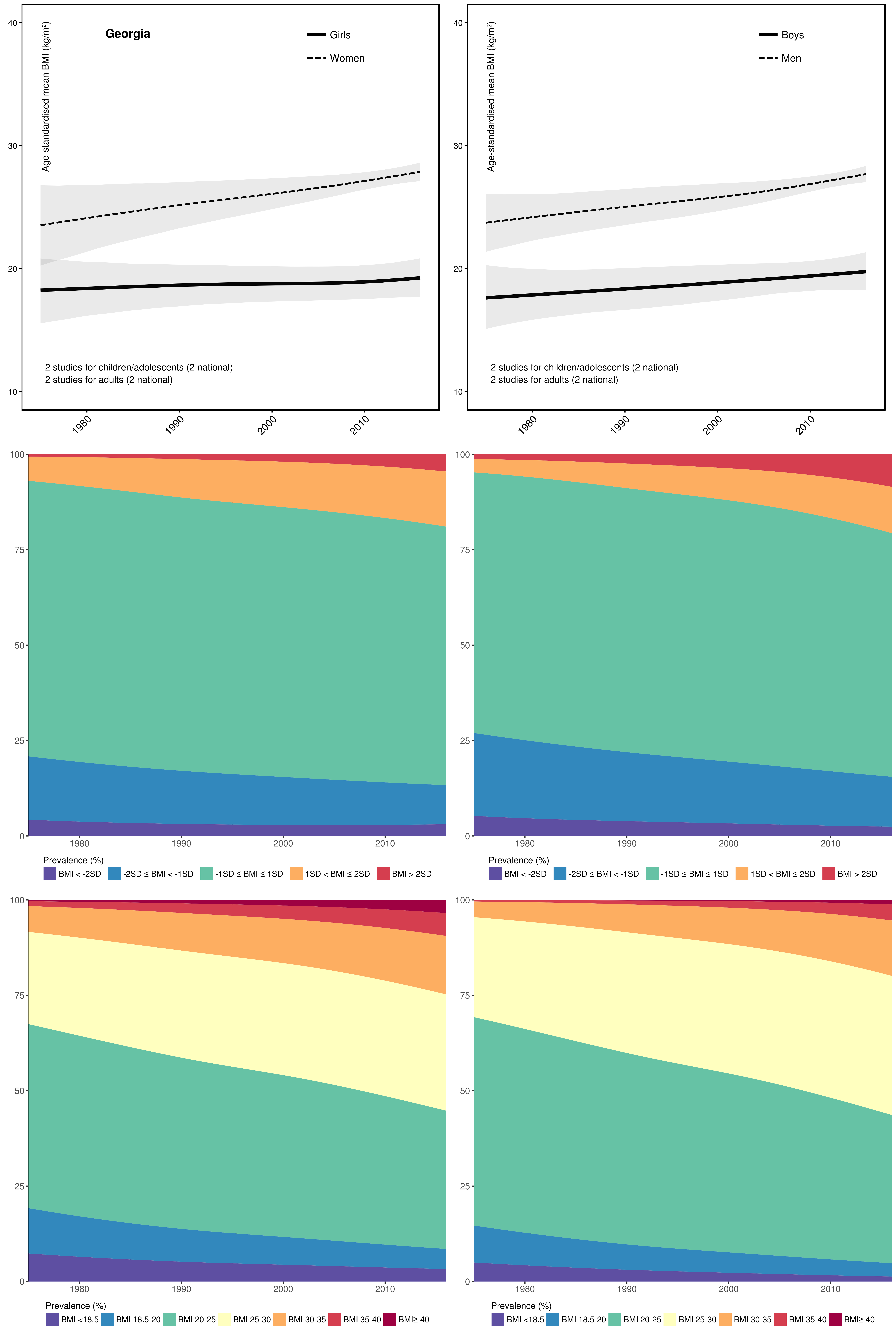
Georgia
Girls
Women
Boys
Men
Age-standardised mean BMI (kg/m²)
2 studies for children/adolescents (2 national)
2 studies for adults (2 national)
1980
1990
2000
2010
Prevalence (%)
BMI < -2SD
-2SD ≤ BMI < -1SD
-1SD ≤ BMI ≤ 1SD
1SD < BMI ≤ 2SD
BMI > 2SD
BMI <18.5
BMI 18.5-20
BMI 20-25
BMI 25-30
BMI 30-35
BMI 35-40
BMI≥ 40

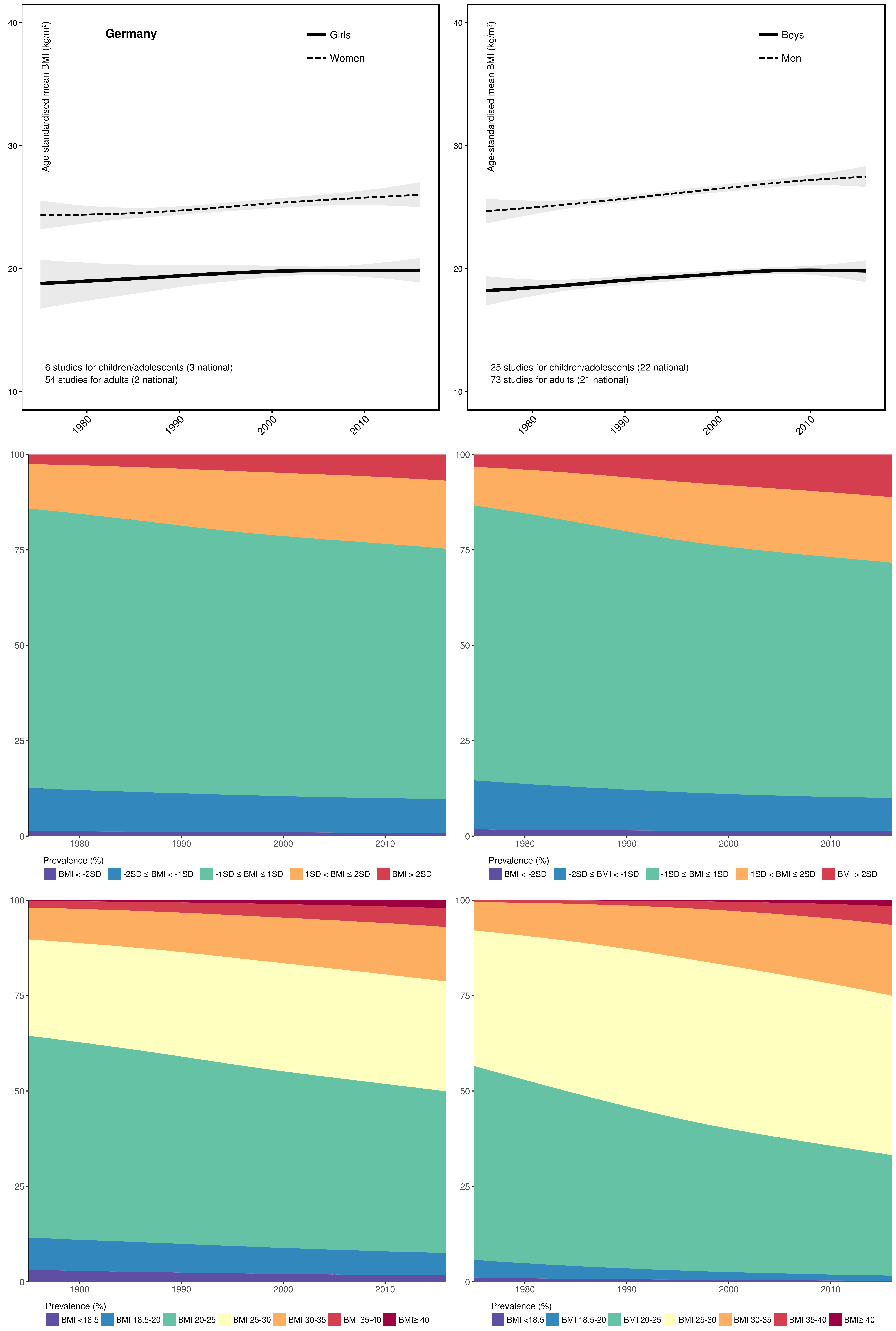
Germany
Girls
Women
Boys
Men
Age-standardised mean BMI (kg/m²)
6 studies for children/adolescents (3 national)
54 studies for adults (2 national)
25 studies for children/adolescents (22 national)
73 studies for adults (21 national)
Prevalence (%)
BMI < -2SD
-2SD ≤ BMI < -1SD
-1SD ≤ BMI ≤ 1SD
1SD < BMI ≤ 2SD
BMI > 2SD
BMI <18.5
BMI 18.5-20
BMI 20-25
BMI 25-30
BMI 30-35
BMI 35-40
BMI≥ 40

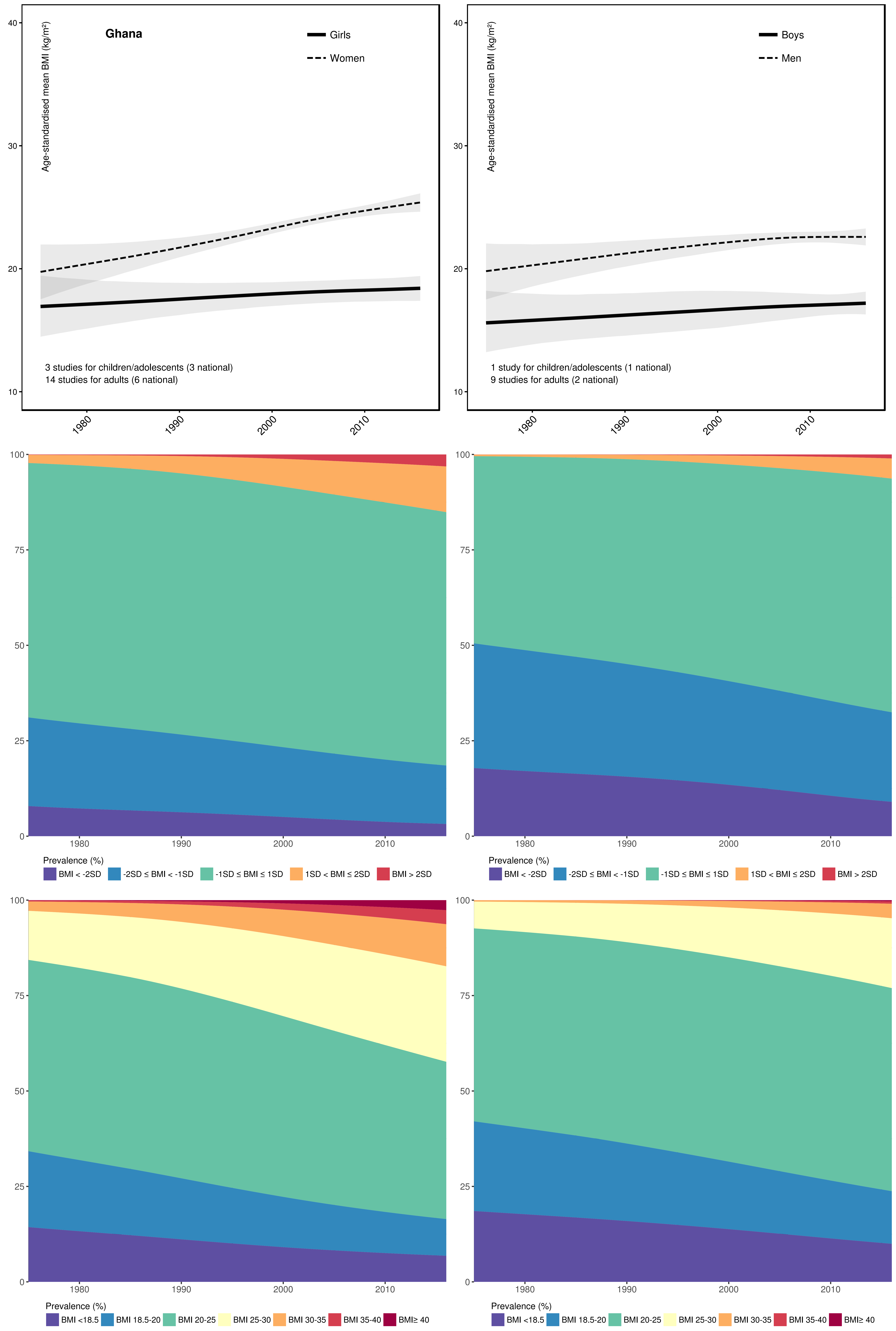
Ghana
Age-standardised mean BMI (kg/m²)
Girls
Women
Boys
Men
3 studies for children/adolescents (3 national)
14 studies for adults (6 national)
1 study for children/adolescents (1 national)
9 studies for adults (2 national)
1980
1990
2000
2010
Prevalence (%)
BMI < -2SD
-2SD ≤ BMI < -1SD
-1SD ≤ BMI ≤ 1SD
1SD < BMI ≤ 2SD
BMI > 2SD
BMI <18.5
BMI 18.5-20
BMI 20-25
BMI 25-30
BMI 30-35
BMI 35-40
BMI≥ 40

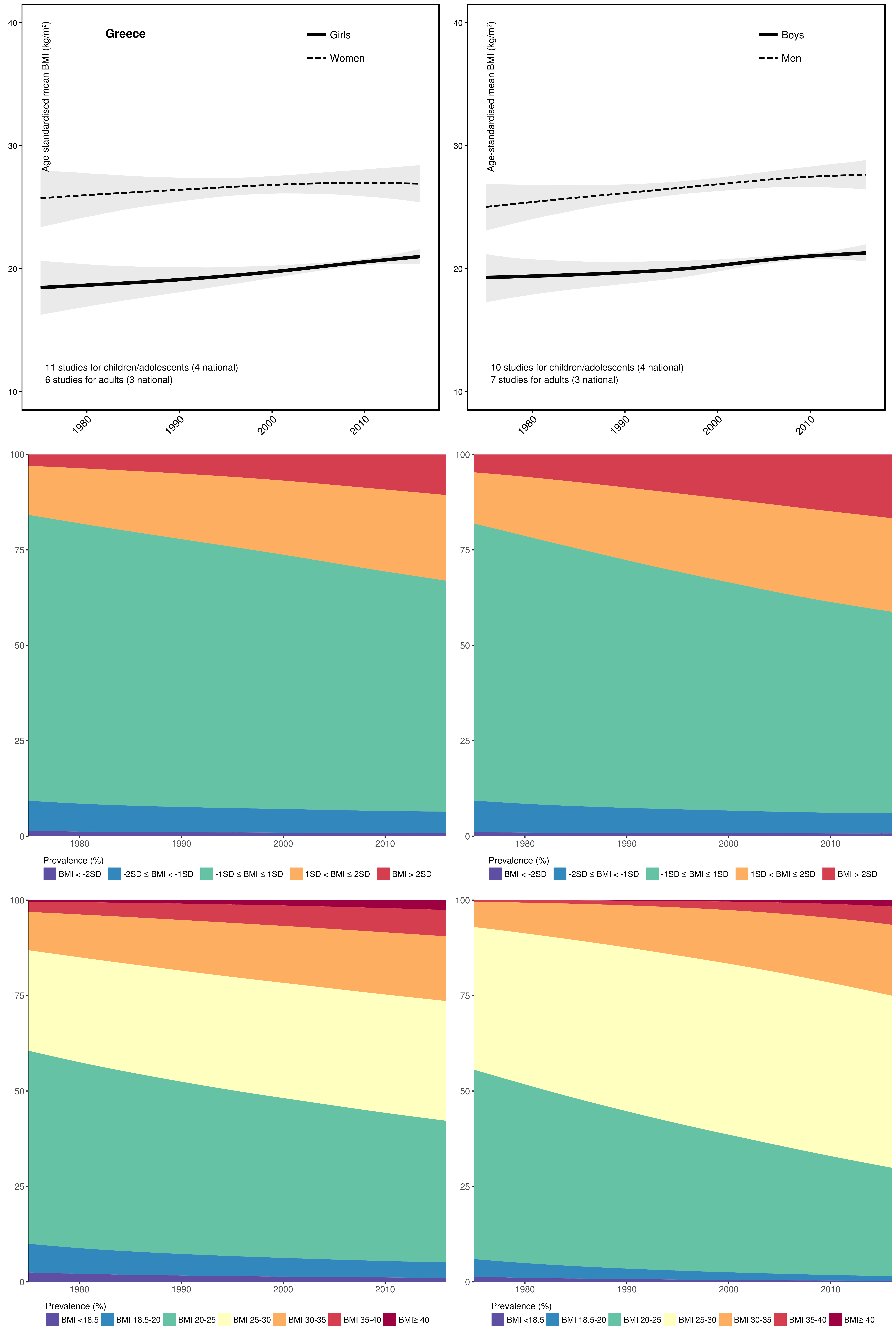
Greece
Girls
Women
Boys
Men
Age-standardised mean BMI (kg/m²)
11 studies for children/adolescents (4 national)
6 studies for adults (3 national)
10 studies for children/adolescents (4 national)
7 studies for adults (3 national)
Prevalence (%)
BMI < -2SD
-2SD ≤ BMI < -1SD
-1SD ≤ BMI ≤ 1SD
1SD < BMI ≤ 2SD
BMI > 2SD
BMI <18.5
BMI 18.5-20
BMI 20-25
BMI 25-30
BMI 30-35
BMI 35-40
BMI≥ 40

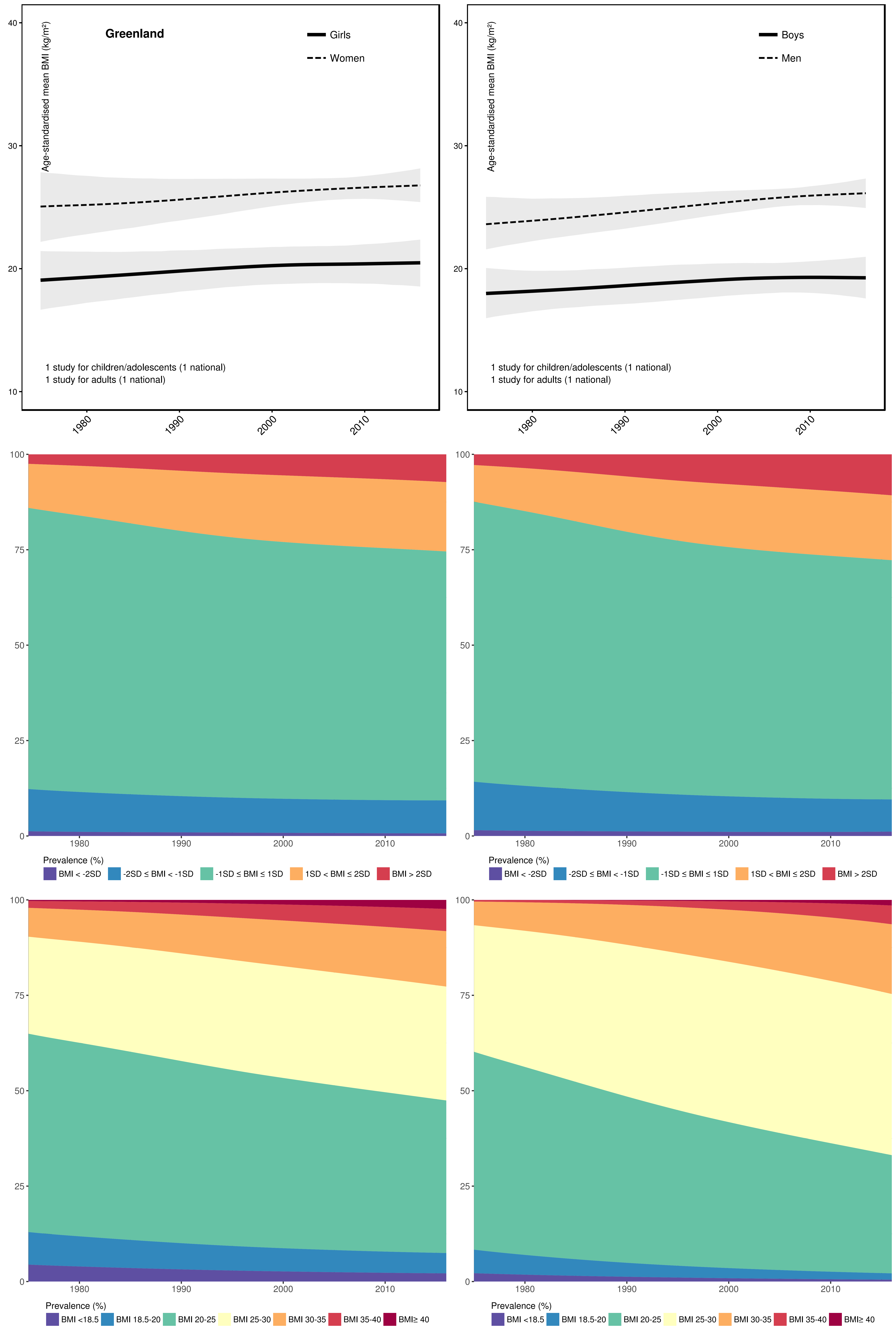
Greenland
Girls
Women
Boys
Men
Age-standardised mean BMI (kg/m²)
1 study for children/adolescents (1 national)
1 study for adults (1 national)
1980
1990
2000
2010
Prevalence (%)
BMI < -2SD
-2SD ≤ BMI < -1SD
-1SD ≤ BMI ≤ 1SD
1SD < BMI ≤ 2SD
BMI > 2SD
BMI <18.5
BMI 18.5-20
BMI 20-25
BMI 25-30
BMI 30-35
BMI 35-40
BMI≥ 40

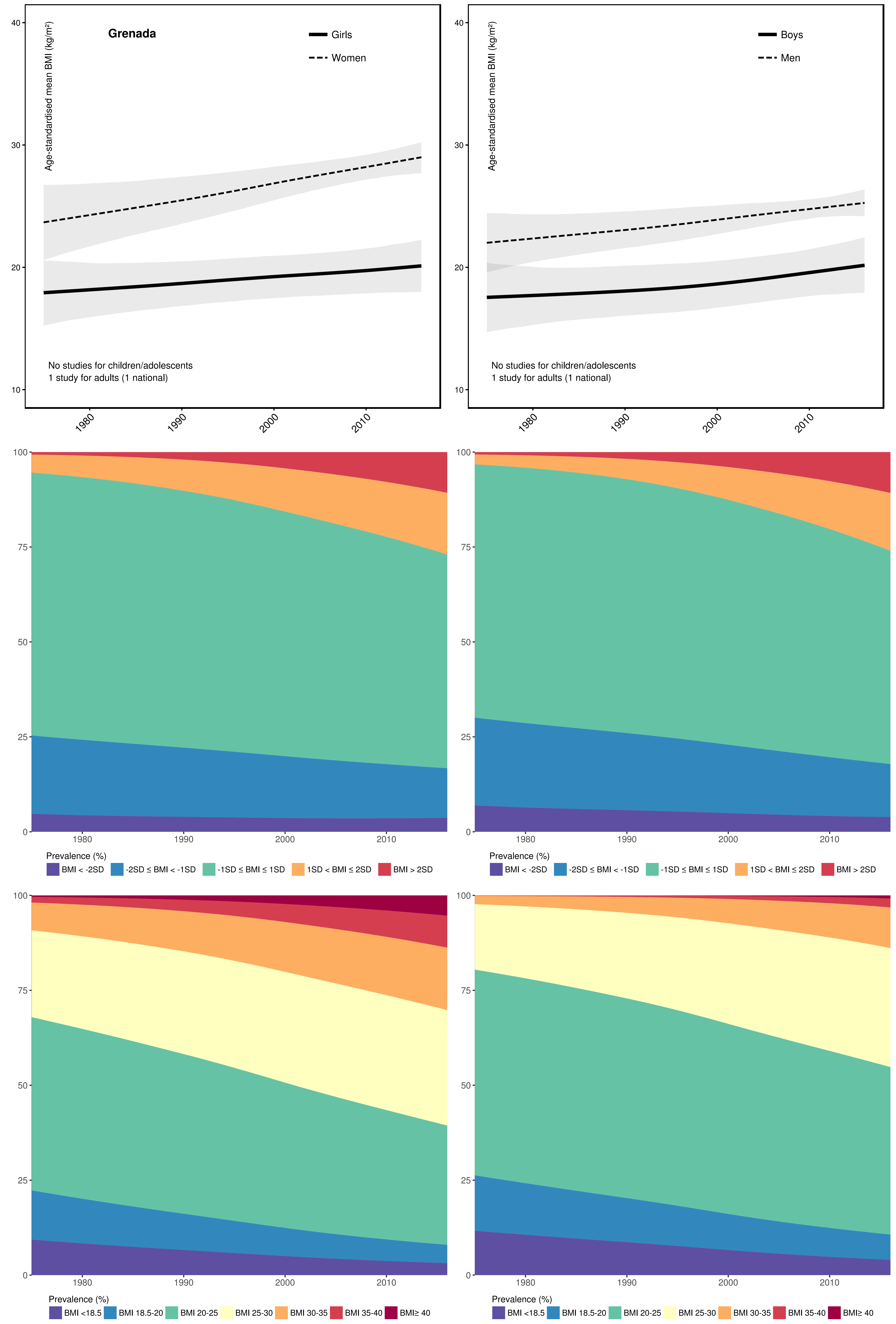
Grenada
Girls
Women
Age-standardised mean BMI (kg/m²)
No studies for children/adolescents
1 study for adults (1 national)
Boys
Men
Age-standardised mean BMI (kg/m²)
No studies for children/adolescents
1 study for adults (1 national)
Prevalence (%)
BMI < -2SD
-2SD ≤ BMI < -1SD
-1SD ≤ BMI ≤ 1SD
1SD < BMI ≤ 2SD
BMI > 2SD
Prevalence (%)
BMI < -2SD
-2SD ≤ BMI < -1SD
-1SD ≤ BMI ≤ 1SD
1SD < BMI ≤ 2SD
BMI > 2SD
Prevalence (%)
BMI <18.5
BMI 18.5-20
BMI 20-25
BMI 25-30
BMI 30-35
BMI 35-40
BMI≥ 40
Prevalence (%)
BMI <18.5
BMI 18.5-20
BMI 20-25
BMI 25-30
BMI 30-35
BMI 35-40
BMI≥ 40

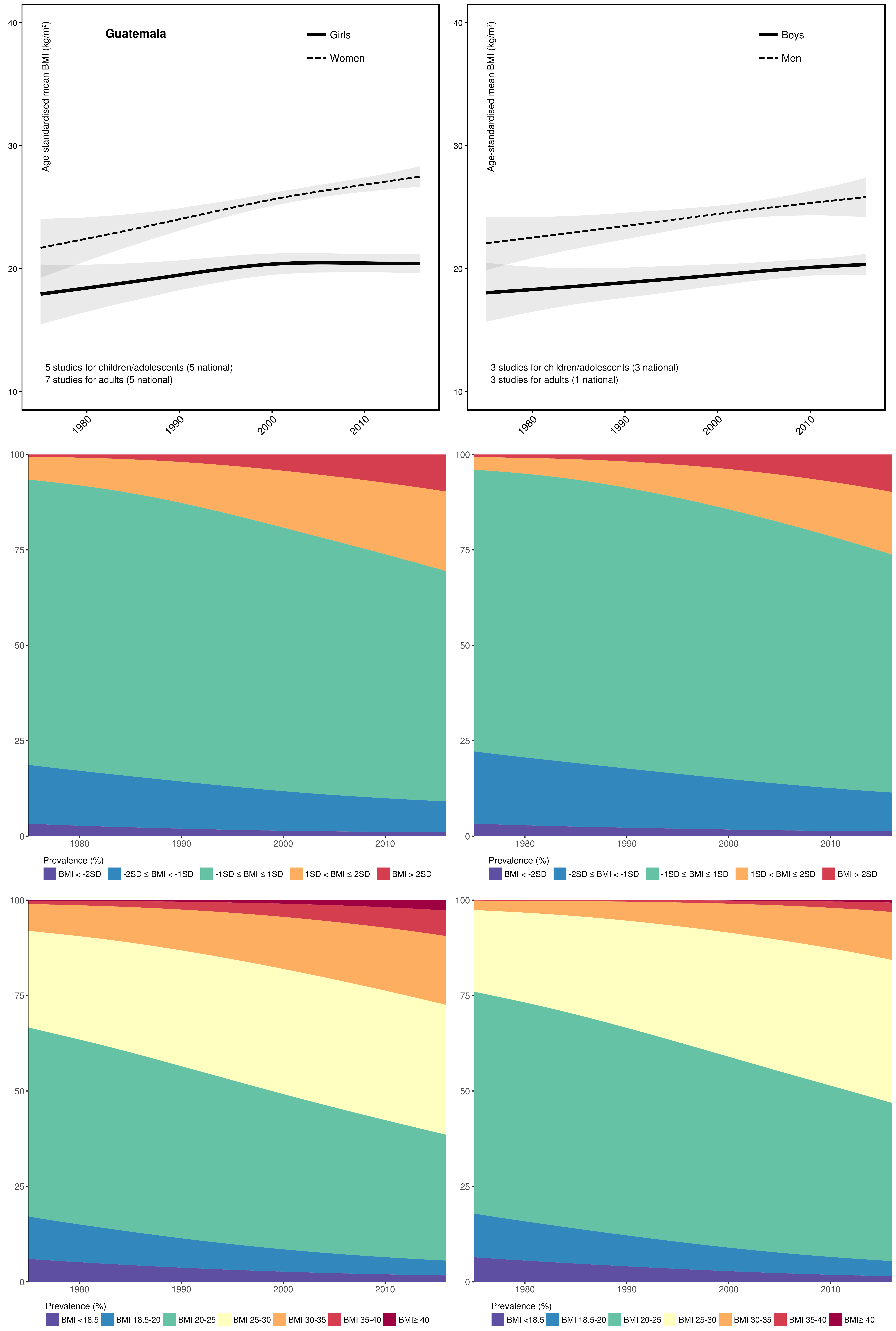
Guatemala
Girls
Women
Boys
Men
Age-standardised mean BMI (kg/m²)
40
30
20
10
1980
1990
2000
2010
5 studies for children/adolescents (5 national)
7 studies for adults (5 national)
3 studies for children/adolescents (3 national)
3 studies for adults (1 national)
100
75
50
25
0
Prevalence (%)
BMI < -2SD
-2SD ≤ BMI < -1SD
-1SD ≤ BMI ≤ 1SD
1SD < BMI ≤ 2SD
BMI > 2SD
BMI <18.5
BMI 18.5-20
BMI 20-25
BMI 25-30
BMI 30-35
BMI 35-40
BMI≥ 40

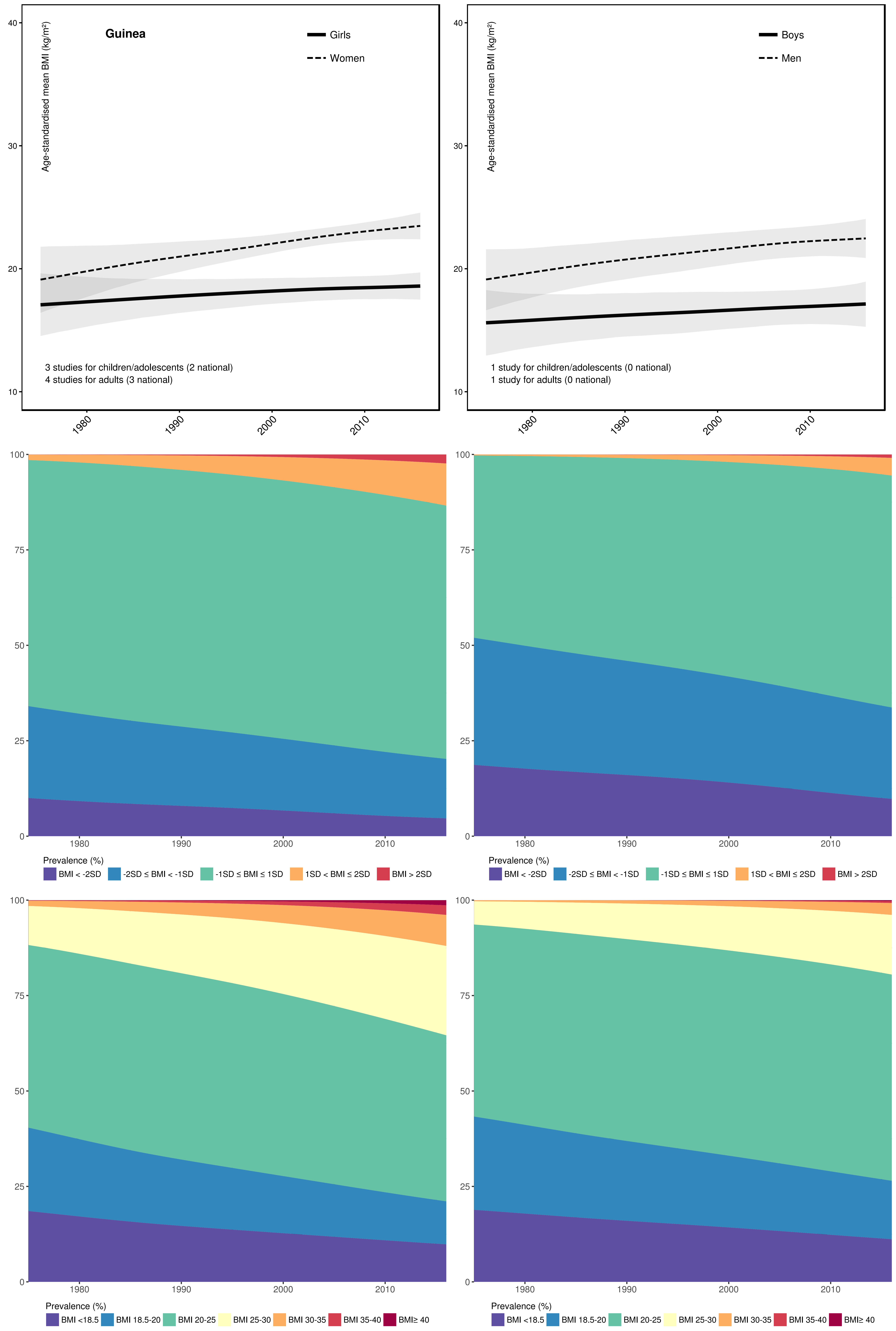
Guinea
Girls
Women
Boys
Men
Age-standardised mean BMI (kg/m²)
40
30
20
10
1980
1990
2000
2010
3 studies for children/adolescents (2 national)
4 studies for adults (3 national)
1 study for children/adolescents (0 national)
1 study for adults (0 national)
100
75
50
25
0
Prevalence (%)
BMI < -2SD
-2SD ≤ BMI < -1SD
-1SD ≤ BMI ≤ 1SD
1SD < BMI ≤ 2SD
BMI > 2SD
BMI <18.5
BMI 18.5-20
BMI 20-25
BMI 25-30
BMI 30-35
BMI 35-40
BMI≥ 40

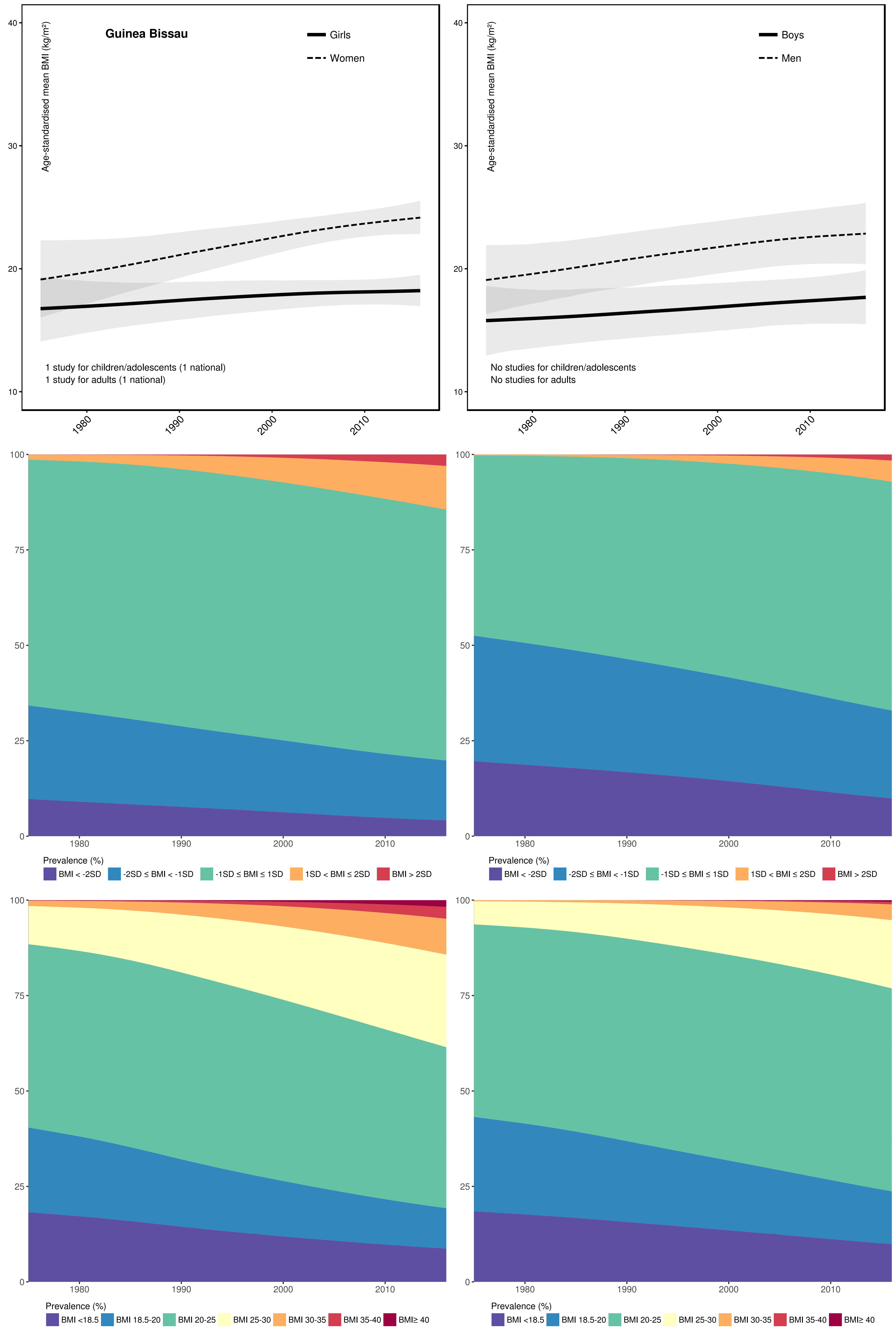
Guinea Bissau
Girls
Women
Boys
Men
Age-standardised mean BMI (kg/m²)
1 study for children/adolescents (1 national)
1 study for adults (1 national)
No studies for children/adolescents
No studies for adults
1980
1990
2000
2010
Prevalence (%)
BMI < -2SD
-2SD ≤ BMI < -1SD
-1SD ≤ BMI ≤ 1SD
1SD < BMI ≤ 2SD
BMI > 2SD
BMI <18.5
BMI 18.5-20
BMI 20-25
BMI 25-30
BMI 30-35
BMI 35-40
BMI≥ 40

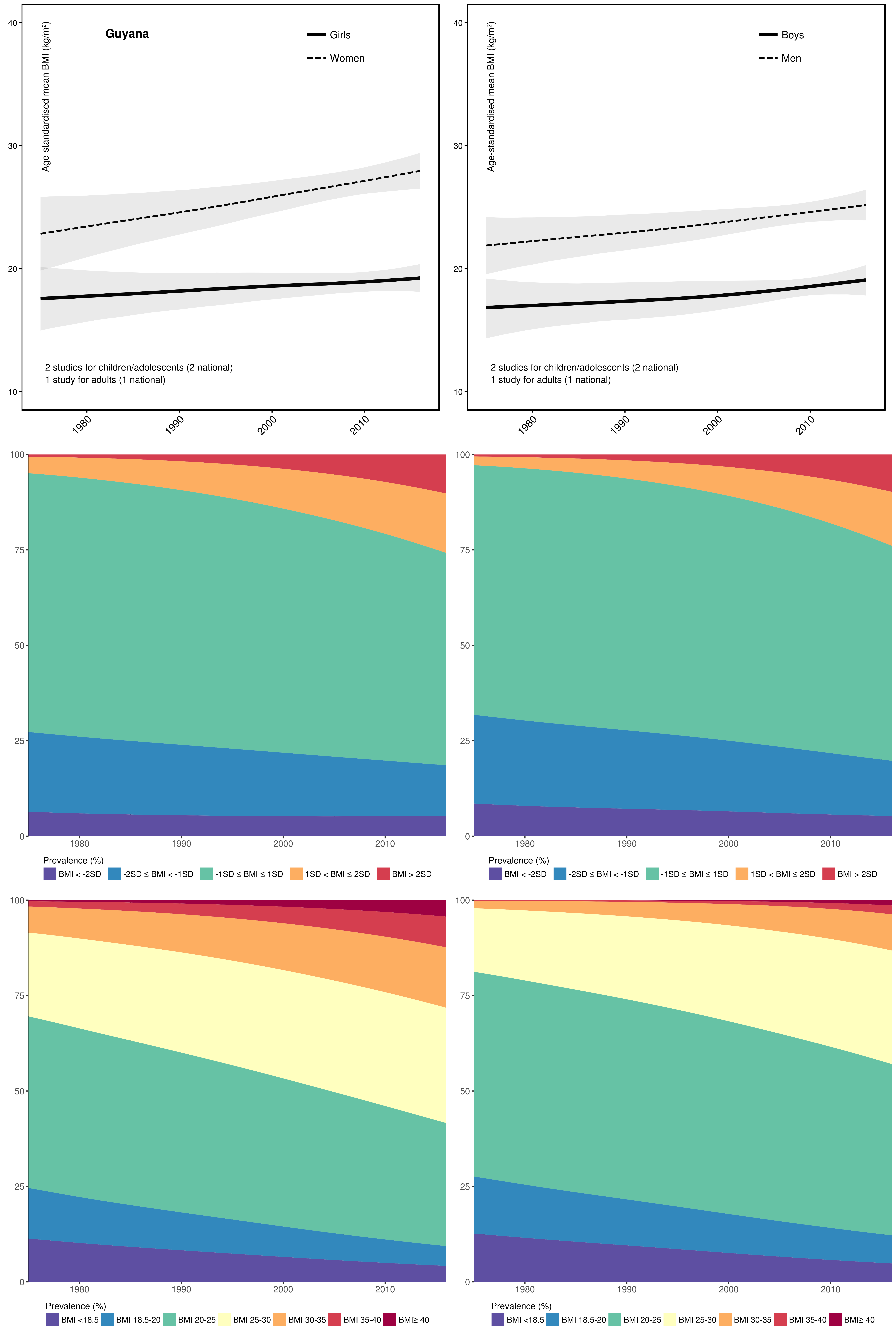
Guyana
Girls
Women
Age-standardised mean BMI (kg/m²)
2 studies for children/adolescents (2 national)
1 study for adults (1 national)
Boys
Men
Age-standardised mean BMI (kg/m²)
2 studies for children/adolescents (2 national)
1 study for adults (1 national)
Prevalence (%)
BMI < -2SD
-2SD ≤ BMI < -1SD
-1SD ≤ BMI ≤ 1SD
1SD < BMI ≤ 2SD
BMI > 2SD
Prevalence (%)
BMI <18.5
BMI 18.5-20
BMI 20-25
BMI 25-30
BMI 30-35
BMI 35-40
BMI≥ 40

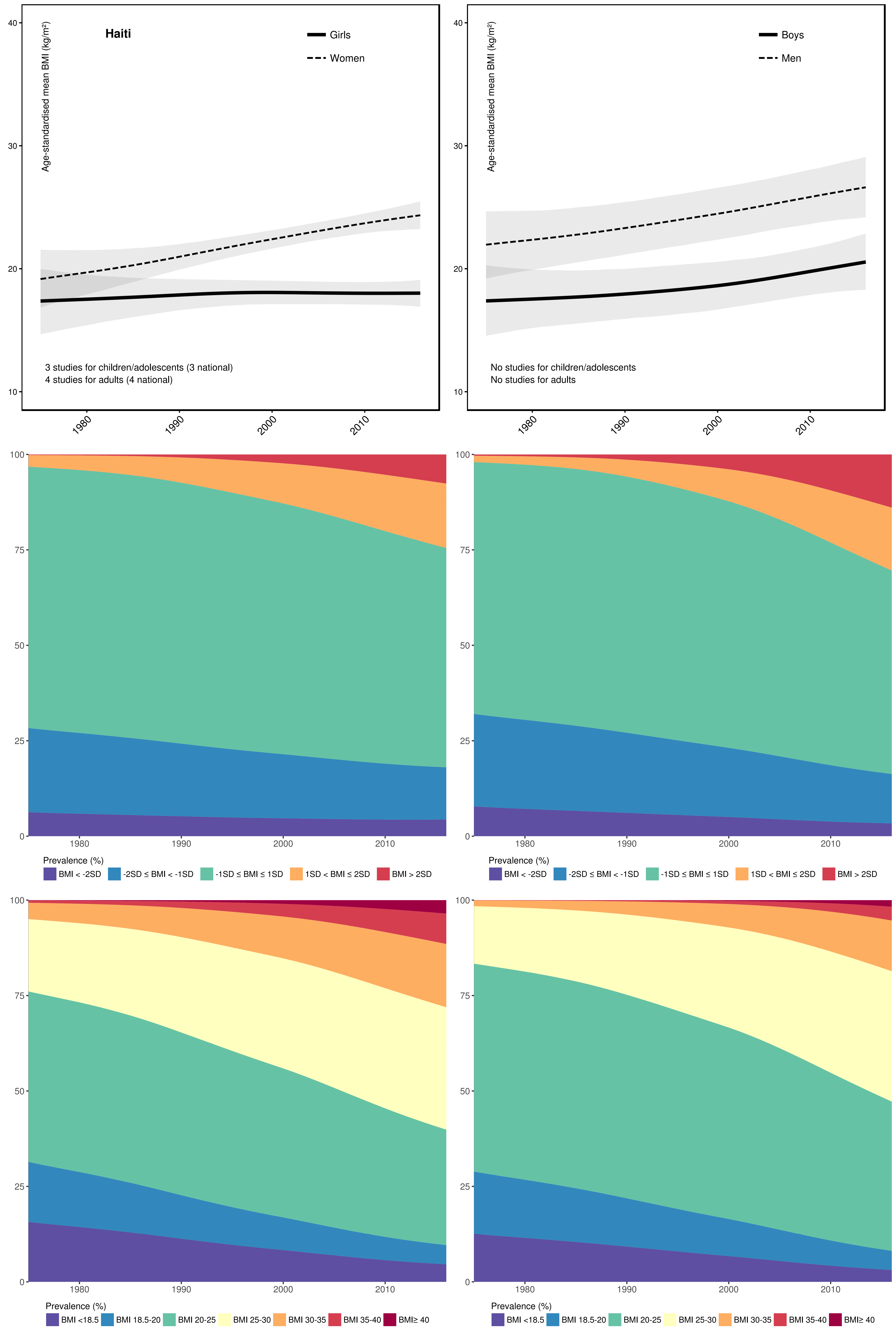
Haiti
Girls
Women
Boys
Men
Age-standardised mean BMI (kg/m²)
3 studies for children/adolescents (3 national)
4 studies for adults (4 national)
No studies for children/adolescents
No studies for adults
Prevalence (%)
BMI < -2SD
-2SD ≤ BMI < -1SD
-1SD ≤ BMI ≤ 1SD
1SD < BMI ≤ 2SD
BMI > 2SD
BMI <18.5
BMI 18.5-20
BMI 20-25
BMI 25-30
BMI 30-35
BMI 35-40
BMI≥ 40

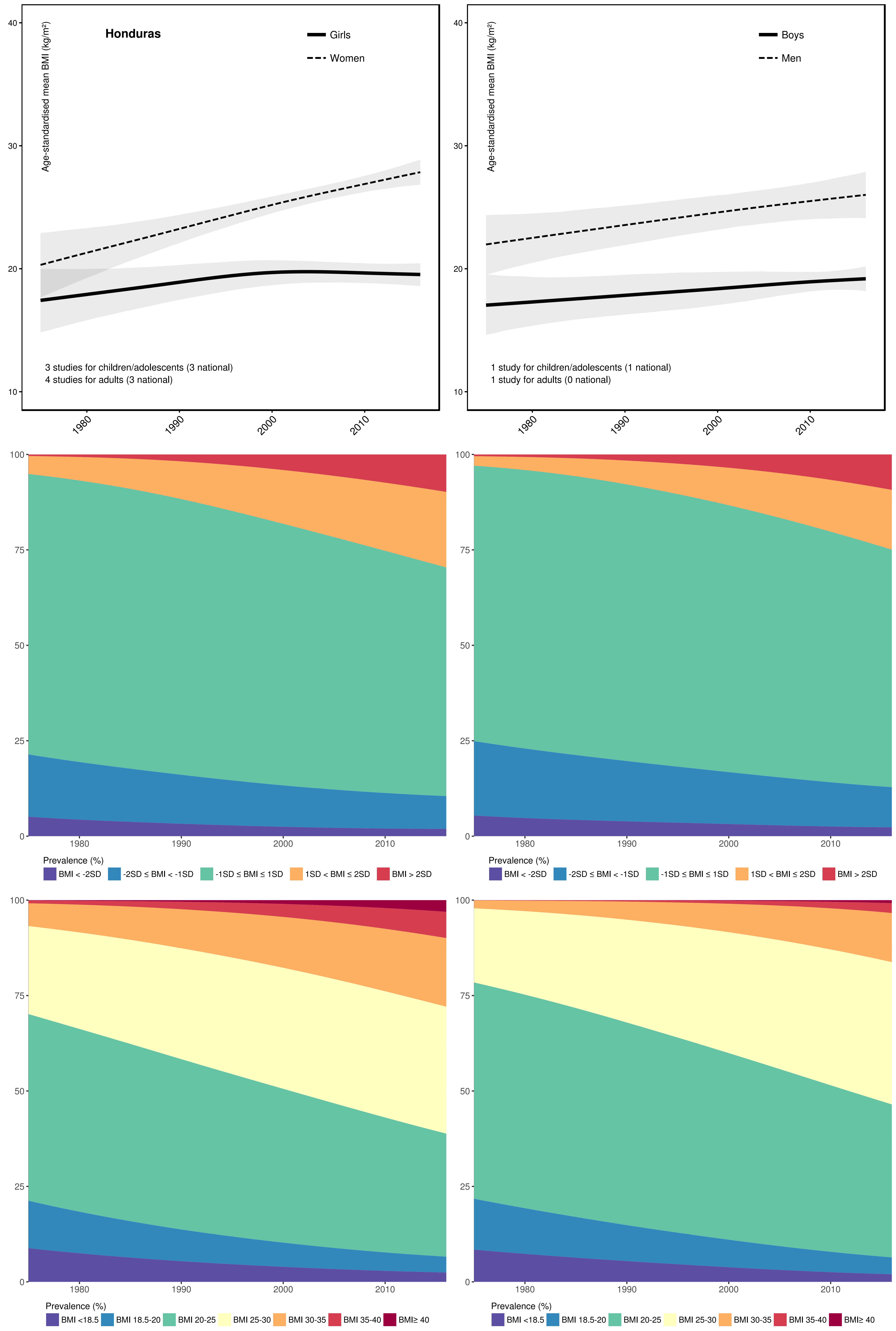
Honduras
Girls
Women
Boys
Men
Age-standardised mean BMI (kg/m²)
3 studies for children/adolescents (3 national)
4 studies for adults (3 national)
1 study for children/adolescents (1 national)
1 study for adults (0 national)
Prevalence (%)
BMI < -2SD
-2SD ≤ BMI < -1SD
-1SD ≤ BMI ≤ 1SD
1SD < BMI ≤ 2SD
BMI > 2SD
BMI <18.5
BMI 18.5-20
BMI 20-25
BMI 25-30
BMI 30-35
BMI 35-40
BMI≥ 40

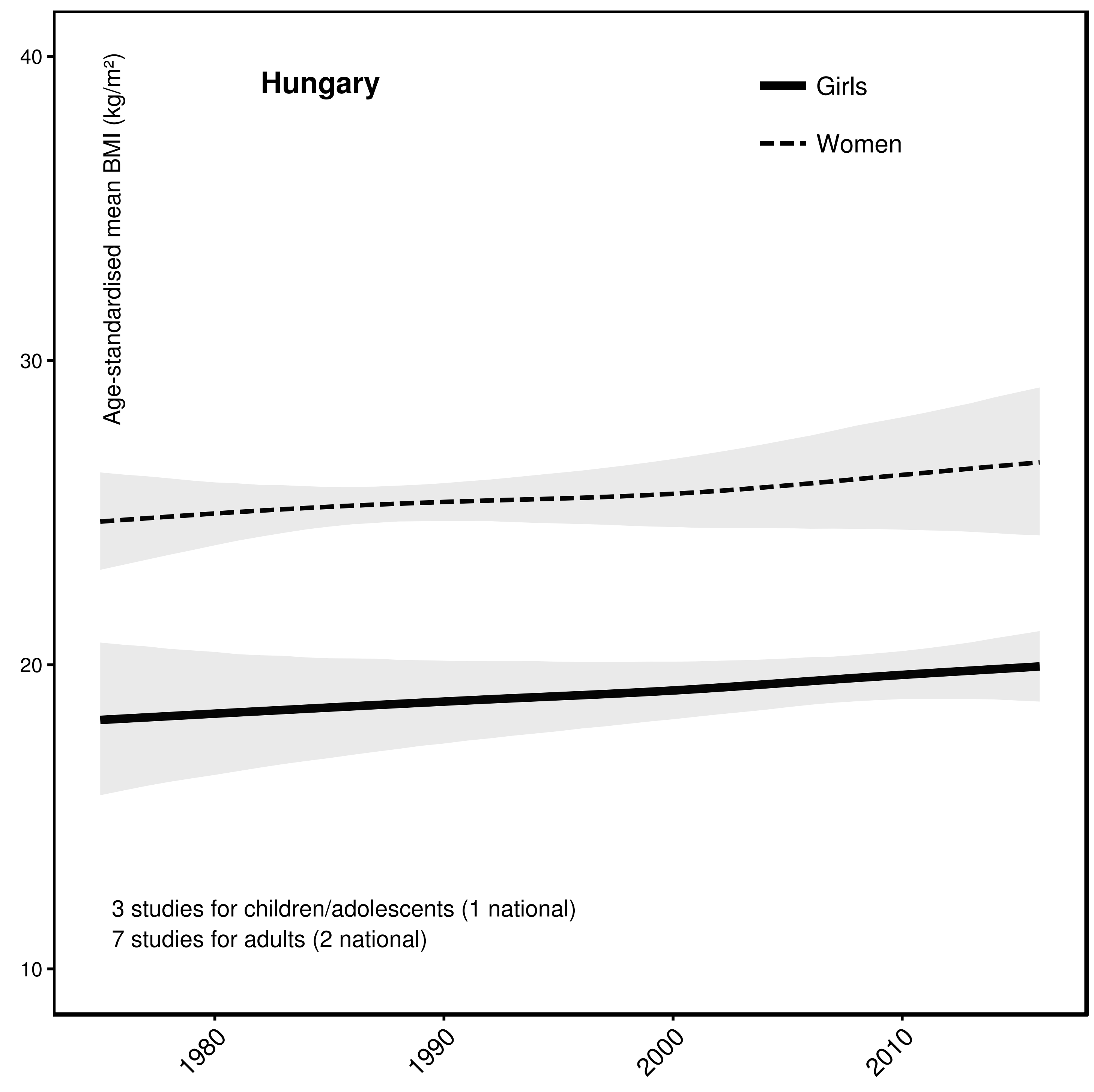
Hungary
Girls
Women
Age-standardised mean BMI (kg/m²)
3 studies for children/adolescents (1 national)
7 studies for adults (2 national)

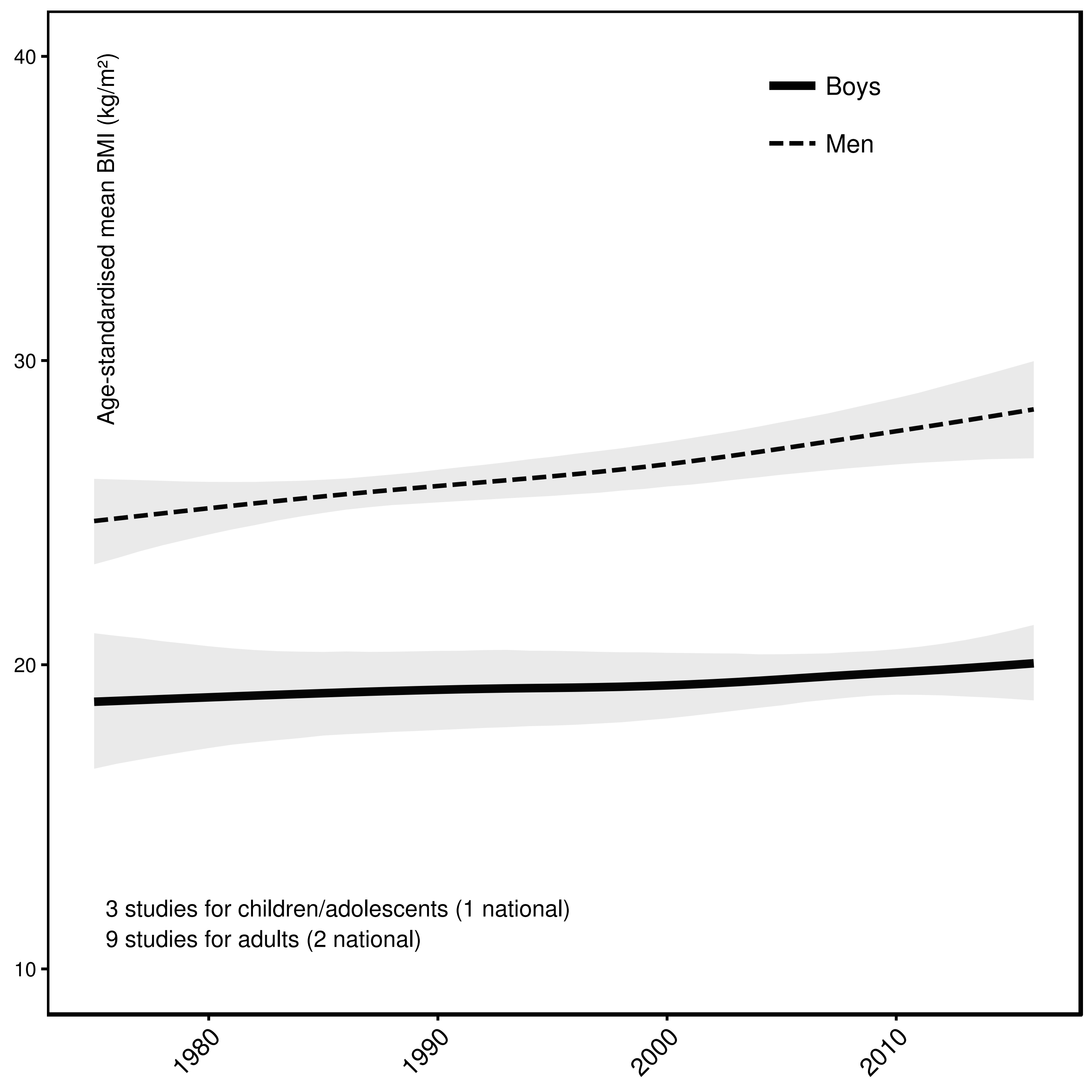
Boys
Men
Age-standardised mean BMI (kg/m²)
3 studies for children/adolescents (1 national)
9 studies for adults (2 national)

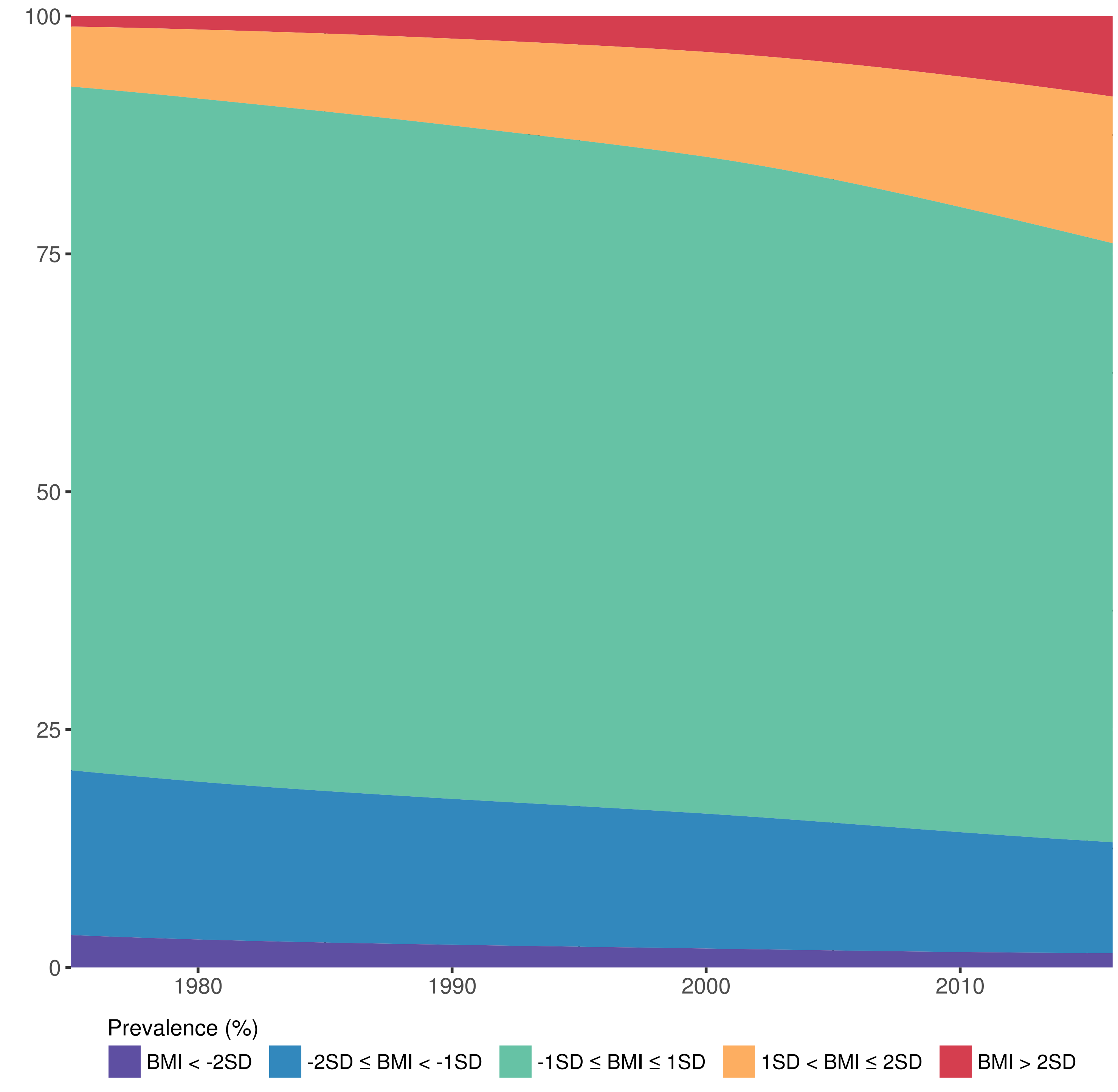
Prevalence (%)
BMI < -2SD
-2SD ≤ BMI < -1SD
-1SD ≤ BMI ≤ 1SD
1SD < BMI ≤ 2SD
BMI > 2SD

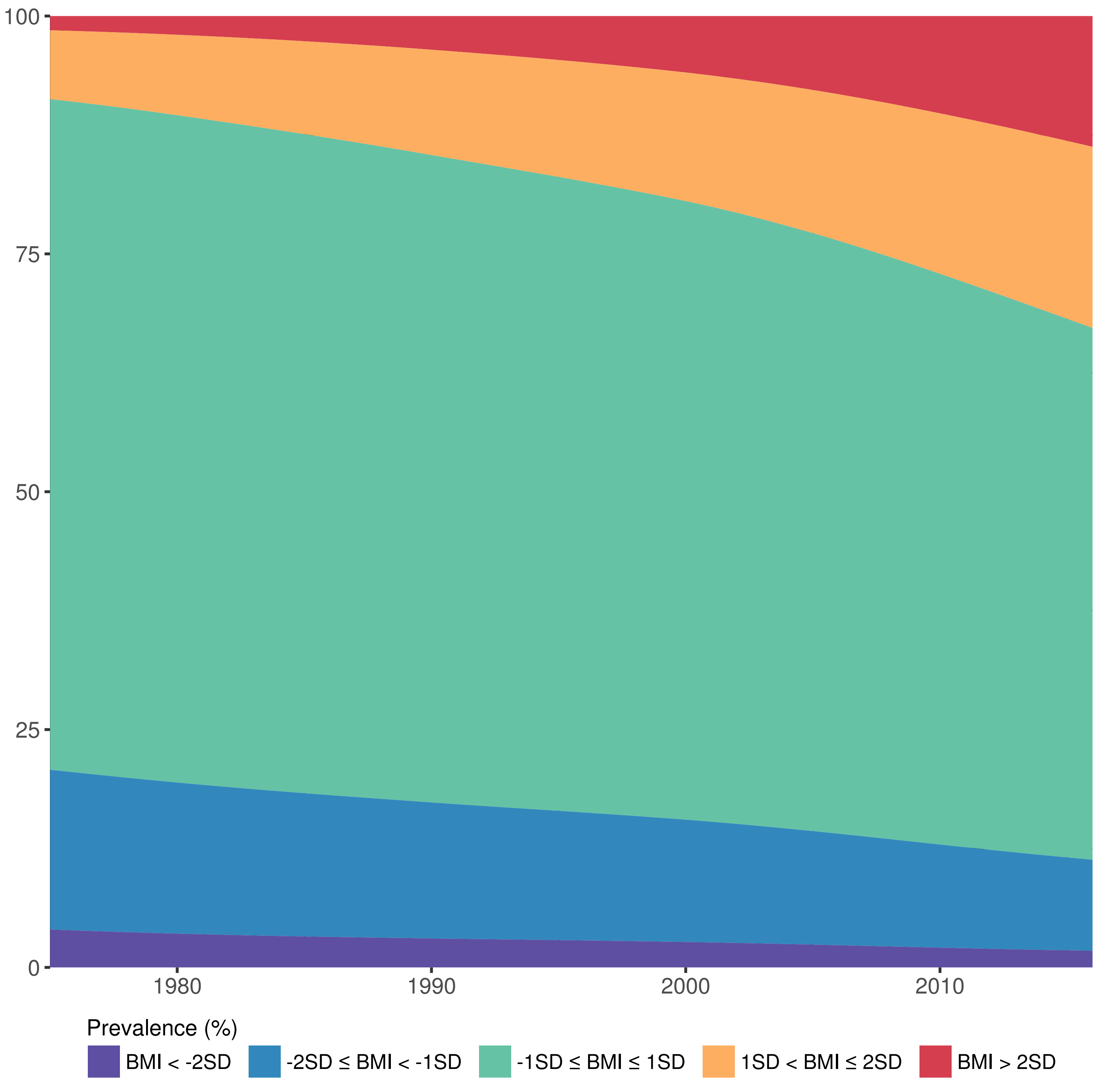
Prevalence (%)
BMI < -2SD
-2SD ≤ BMI < -1SD
-1SD ≤ BMI ≤ 1SD
1SD < BMI ≤ 2SD
BMI > 2SD

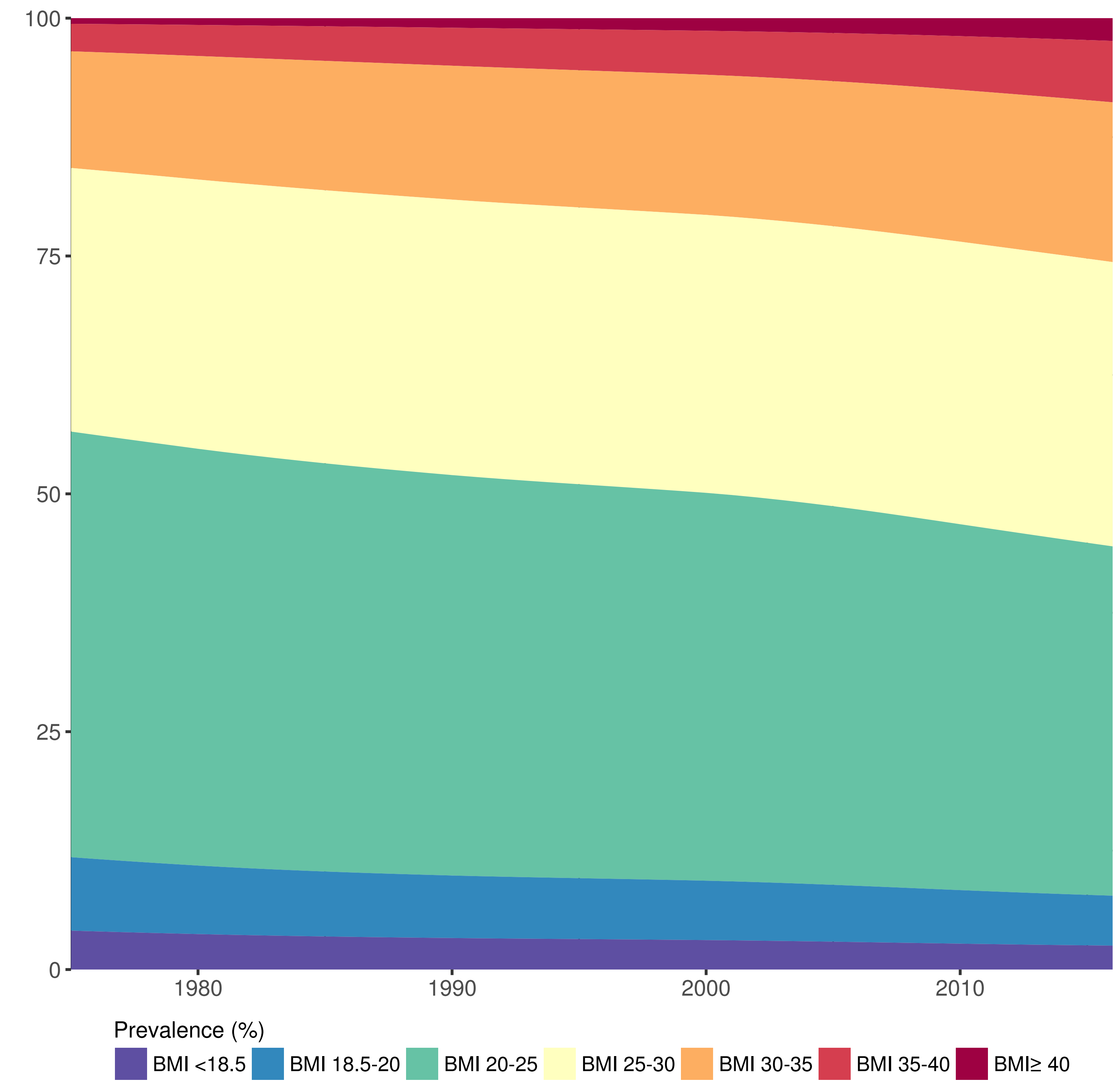
Prevalence (%)
BMI <18.5
BMI 18.5-20
BMI 20-25
BMI 25-30
BMI 30-35
BMI 35-40
BMI≥ 40

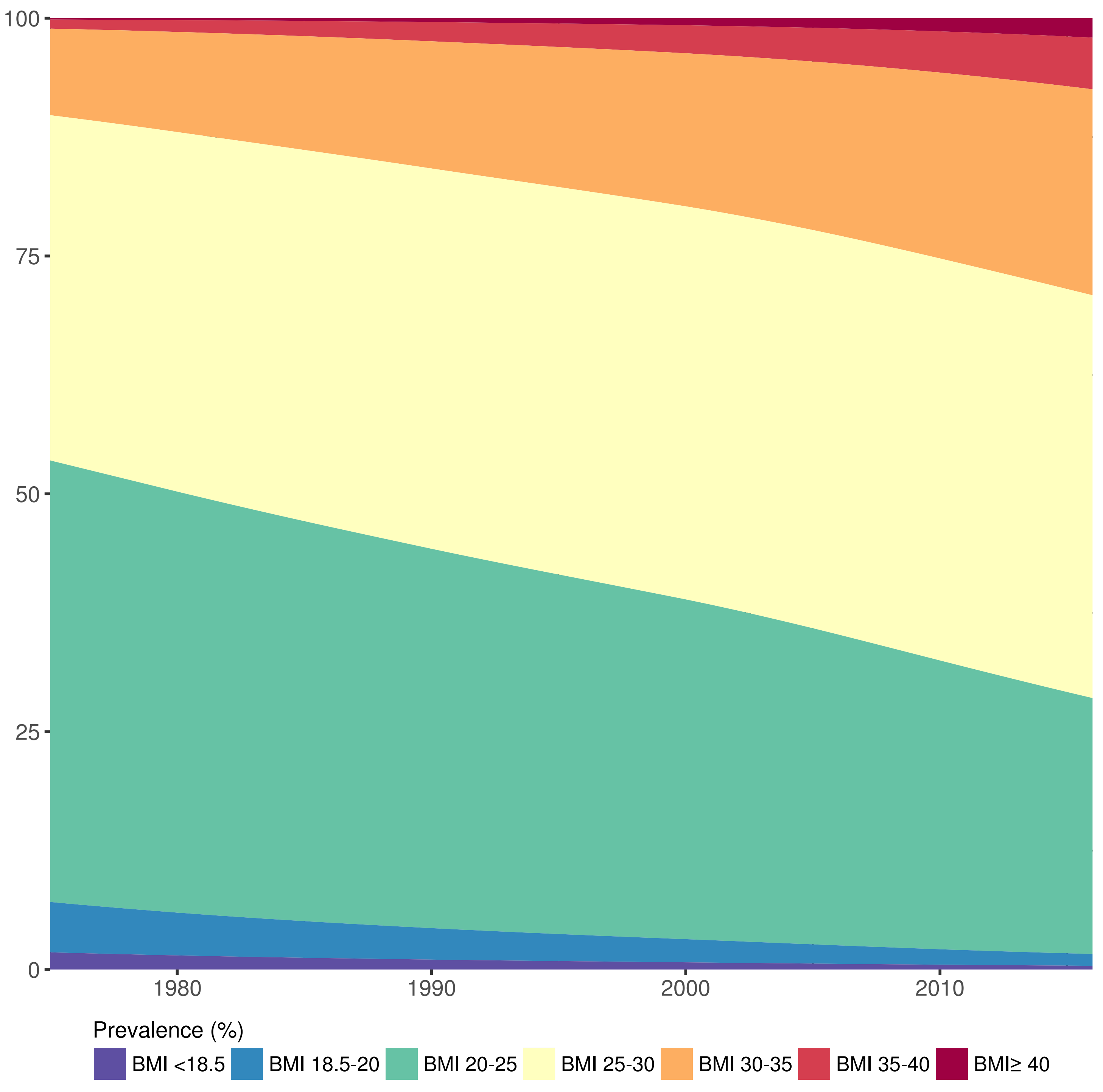
Prevalence (%)
BMI <18.5
BMI 18.5-20
BMI 20-25
BMI 25-30
BMI 30-35
BMI 35-40
BMI≥ 40

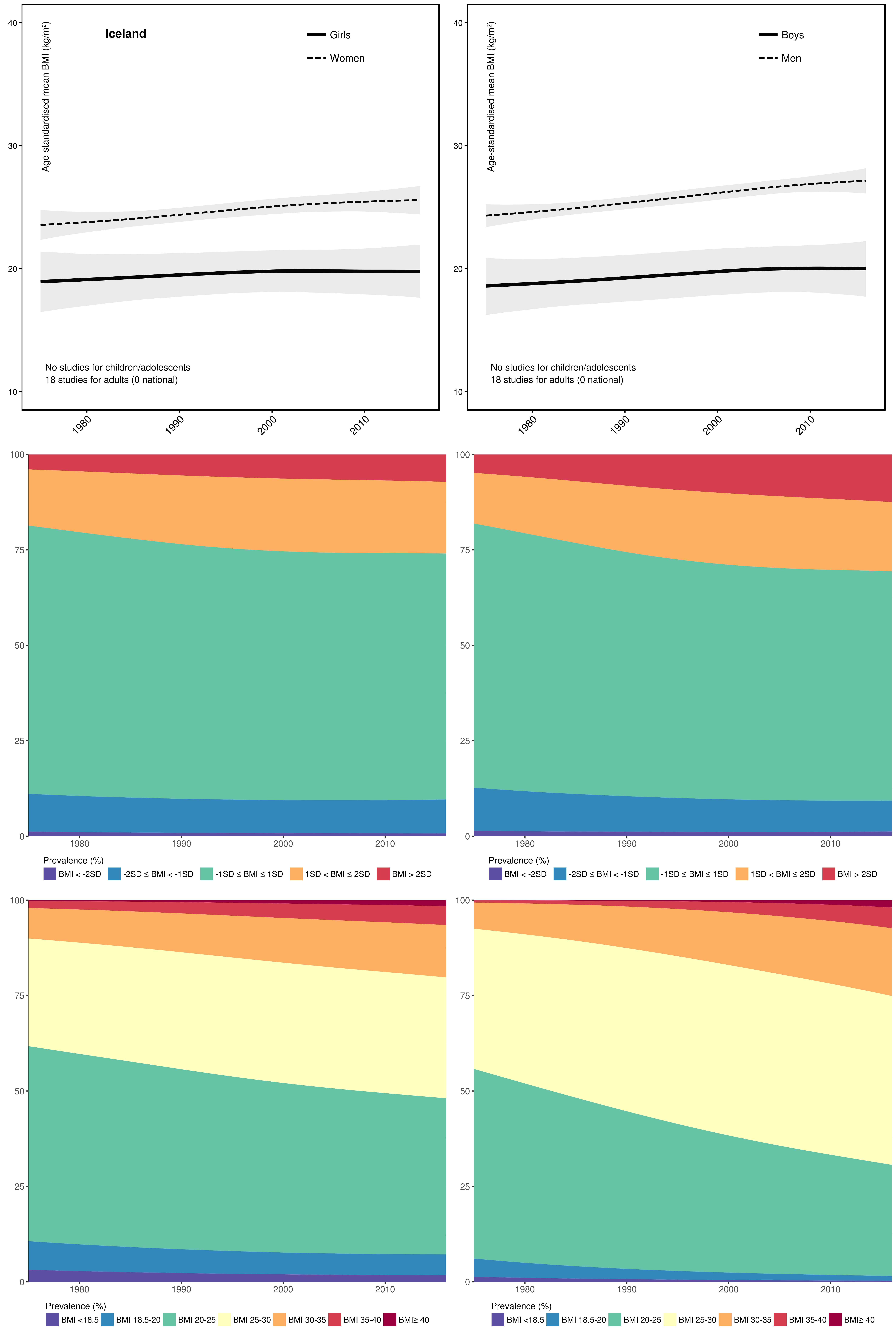
Iceland
Age-standardised mean BMI (kg/m²)
Girls
Women
Boys
Men
No studies for children/adolescents
18 studies for adults (0 national)
No studies for children/adolescents
18 studies for adults (0 national)
1980
1990
2000
2010
Prevalence (%)
BMI < -2SD
-2SD ≤ BMI < -1SD
-1SD ≤ BMI ≤ 1SD
1SD < BMI ≤ 2SD
BMI > 2SD
Prevalence (%)
BMI <18.5
BMI 18.5-20
BMI 20-25
BMI 25-30
BMI 30-35
BMI 35-40
BMI≥ 40

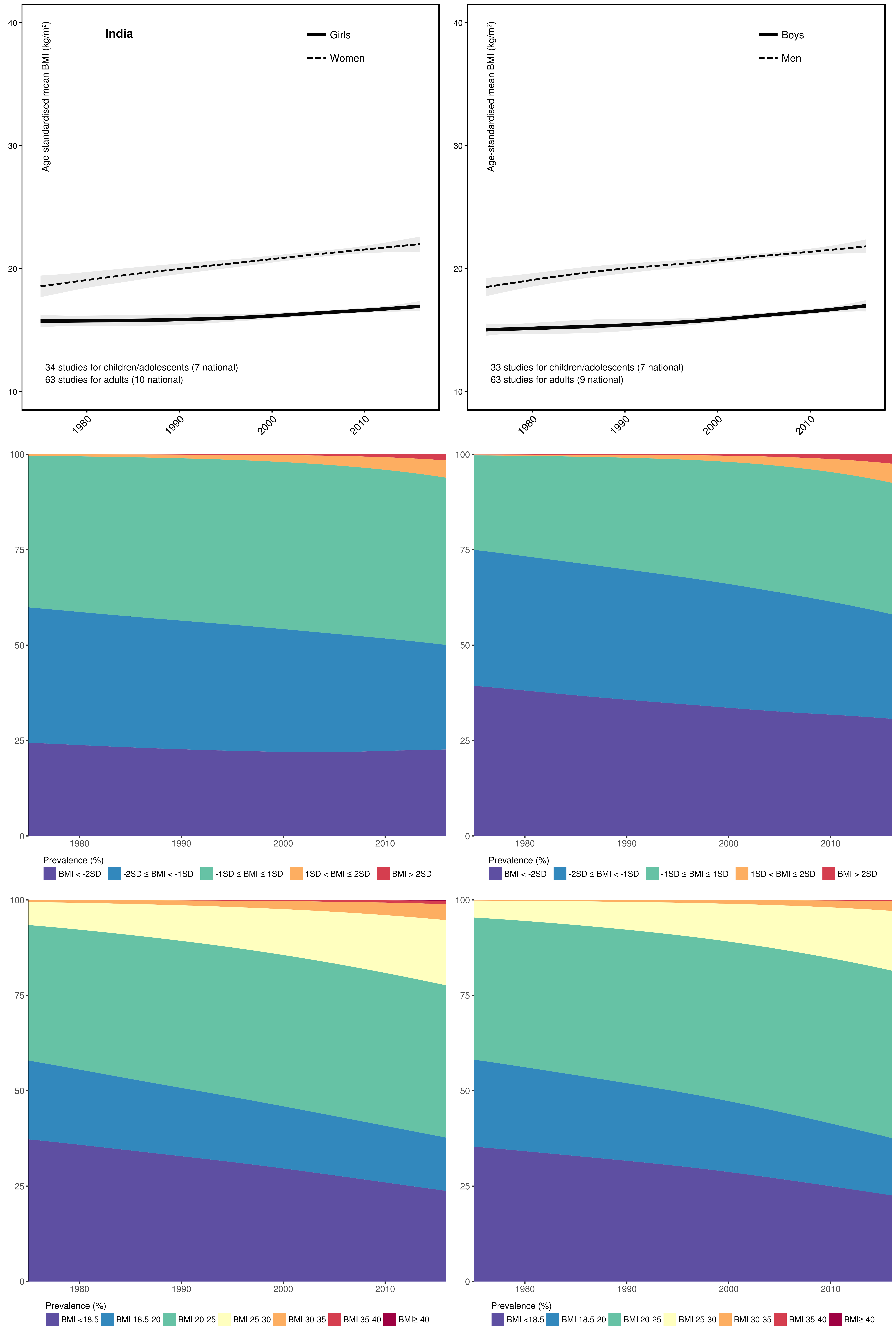
India
Girls
Women
Boys
Men
Age-standardised mean BMI (kg/m²)
34 studies for children/adolescents (7 national)
63 studies for adults (10 national)
33 studies for children/adolescents (7 national)
63 studies for adults (9 national)
Prevalence (%)
BMI < -2SD
-2SD ≤ BMI < -1SD
-1SD ≤ BMI ≤ 1SD
1SD < BMI ≤ 2SD
BMI > 2SD
BMI <18.5
BMI 18.5-20
BMI 20-25
BMI 25-30
BMI 30-35
BMI 35-40
BMI≥ 40

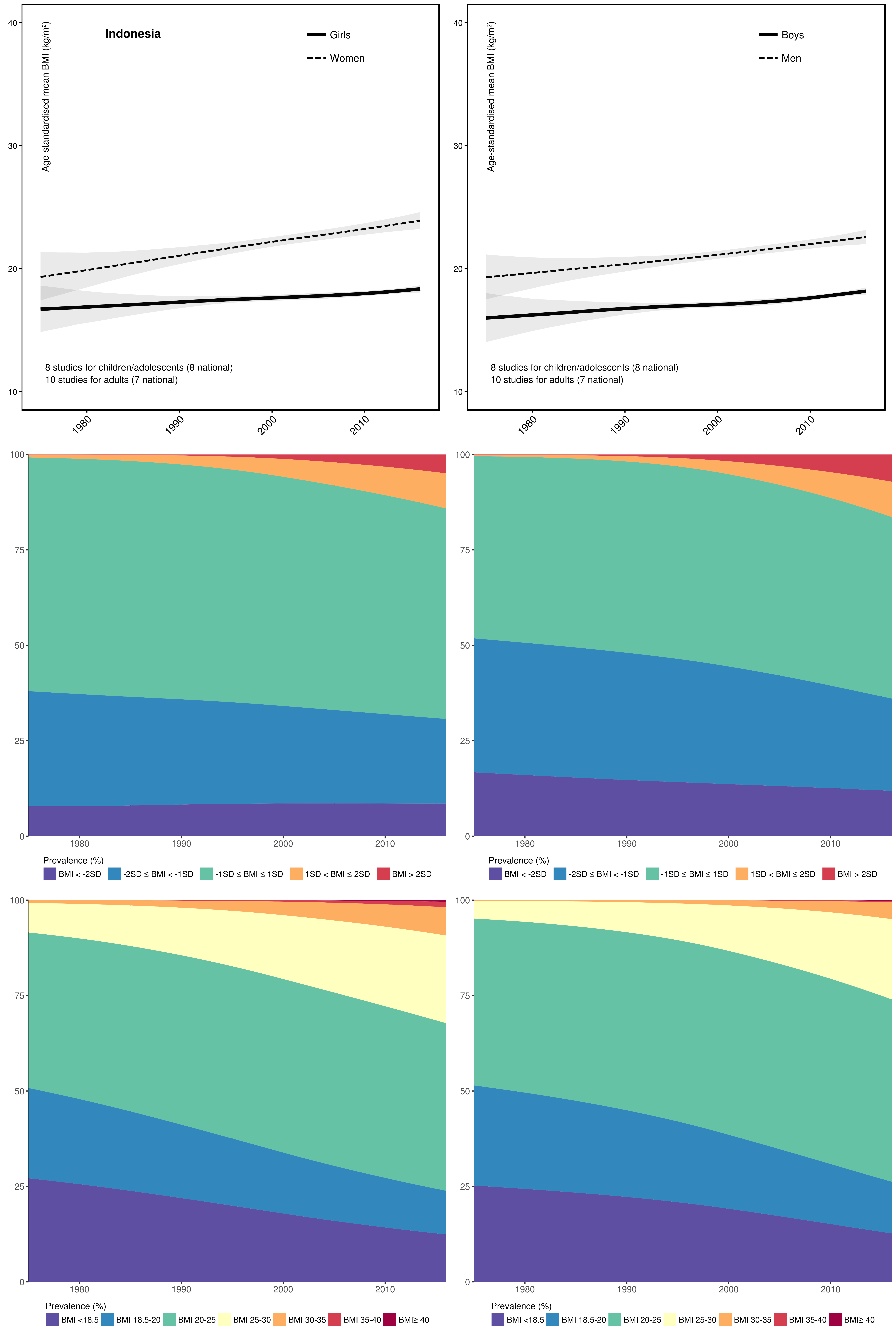
Indonesia
Girls
Women
Boys
Men
Age-standardised mean BMI (kg/m²)
8 studies for children/adolescents (8 national)
10 studies for adults (7 national)
Prevalence (%)
BMI < -2SD
-2SD ≤ BMI < -1SD
-1SD ≤ BMI ≤ 1SD
1SD < BMI ≤ 2SD
BMI > 2SD
BMI <18.5
BMI 18.5-20
BMI 20-25
BMI 25-30
BMI 30-35
BMI 35-40
BMI≥ 40

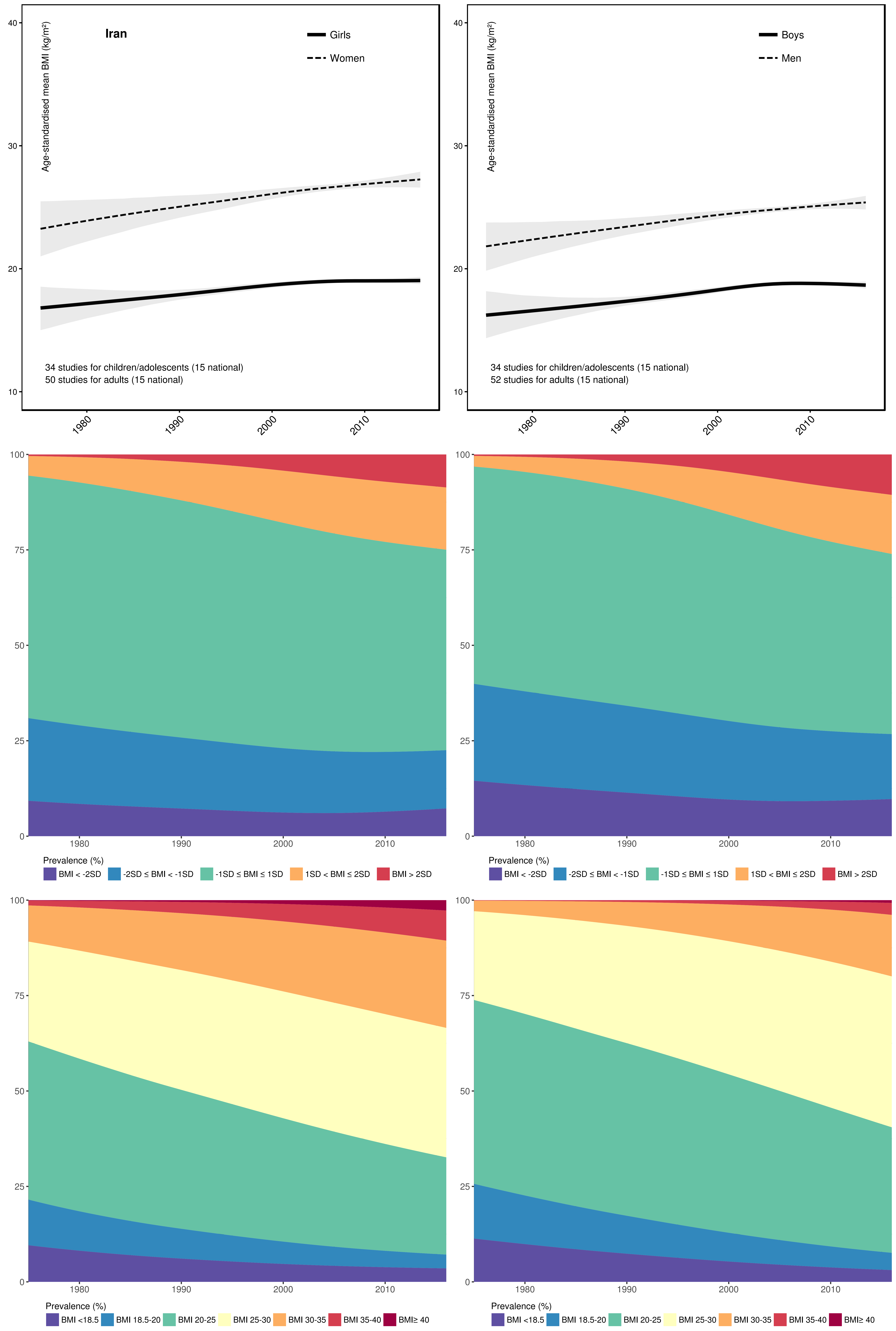
Iran
Girls
Women
Boys
Men
Age-standardised mean BMI (kg/m²)
34 studies for children/adolescents (15 national)
50 studies for adults (15 national)
34 studies for children/adolescents (15 national)
52 studies for adults (15 national)
Prevalence (%)
BMI < -2SD
-2SD ≤ BMI < -1SD
-1SD ≤ BMI ≤ 1SD
1SD < BMI ≤ 2SD
BMI > 2SD
BMI <18.5
BMI 18.5-20
BMI 20-25
BMI 25-30
BMI 30-35
BMI 35-40
BMI≥ 40

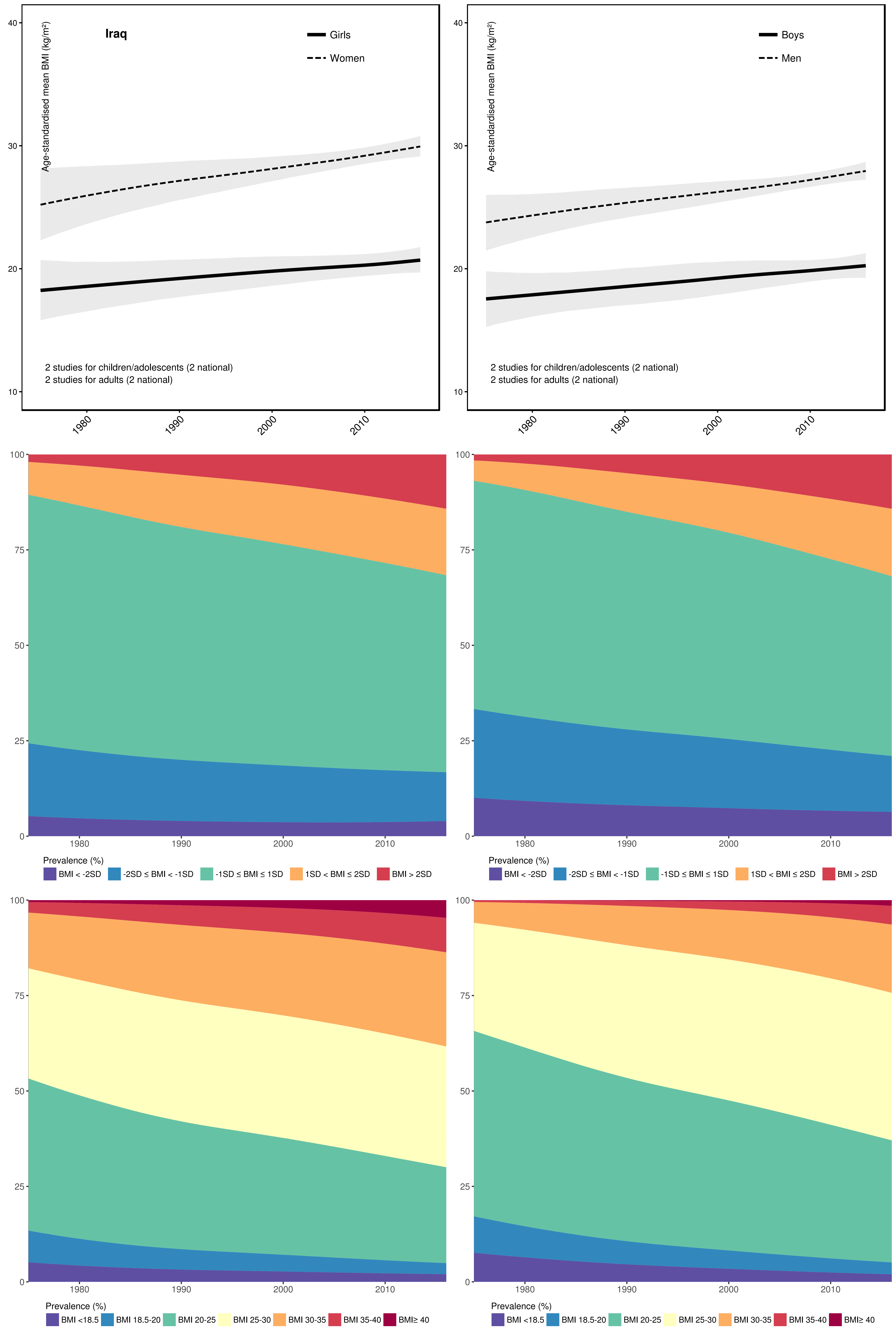
Iraq
Girls
Women
Boys
Men
Age-standardised mean BMI (kg/m²)
2 studies for children/adolescents (2 national)
2 studies for adults (2 national)
Prevalence (%)
BMI < -2SD
-2SD ≤ BMI < -1SD
-1SD ≤ BMI ≤ 1SD
1SD < BMI ≤ 2SD
BMI > 2SD
BMI <18.5
BMI 18.5-20
BMI 20-25
BMI 25-30
BMI 30-35
BMI 35-40
BMI≥ 40

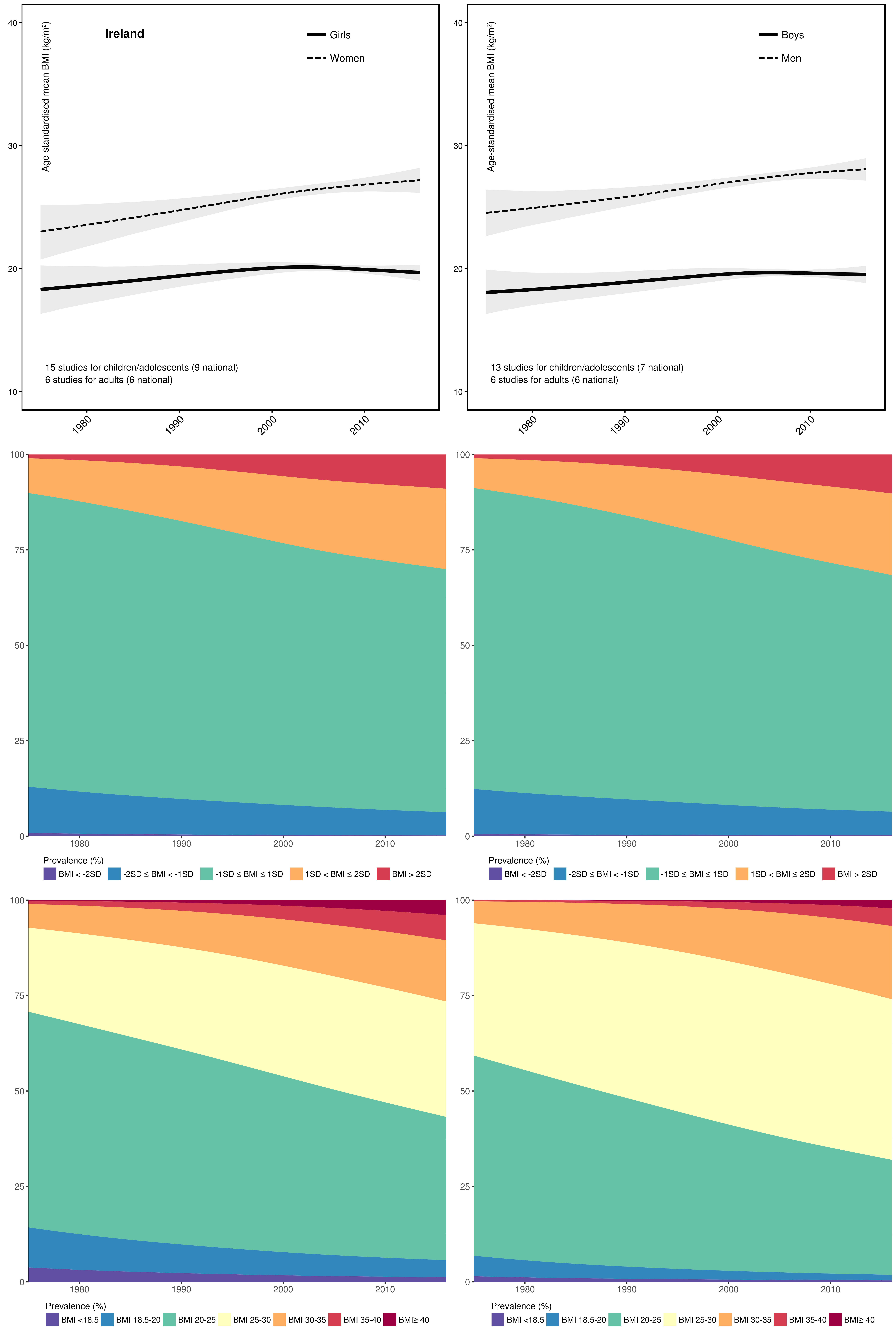
Ireland
Girls
Women
Boys
Men
Age-standardised mean BMI (kg/m²)
15 studies for children/adolescents (9 national)
6 studies for adults (6 national)
13 studies for children/adolescents (7 national)
6 studies for adults (6 national)
Prevalence (%)
BMI < -2SD
-2SD ≤ BMI < -1SD
-1SD ≤ BMI ≤ 1SD
1SD < BMI ≤ 2SD
BMI > 2SD
BMI <18.5
BMI 18.5-20
BMI 20-25
BMI 25-30
BMI 30-35
BMI 35-40
BMI≥ 40

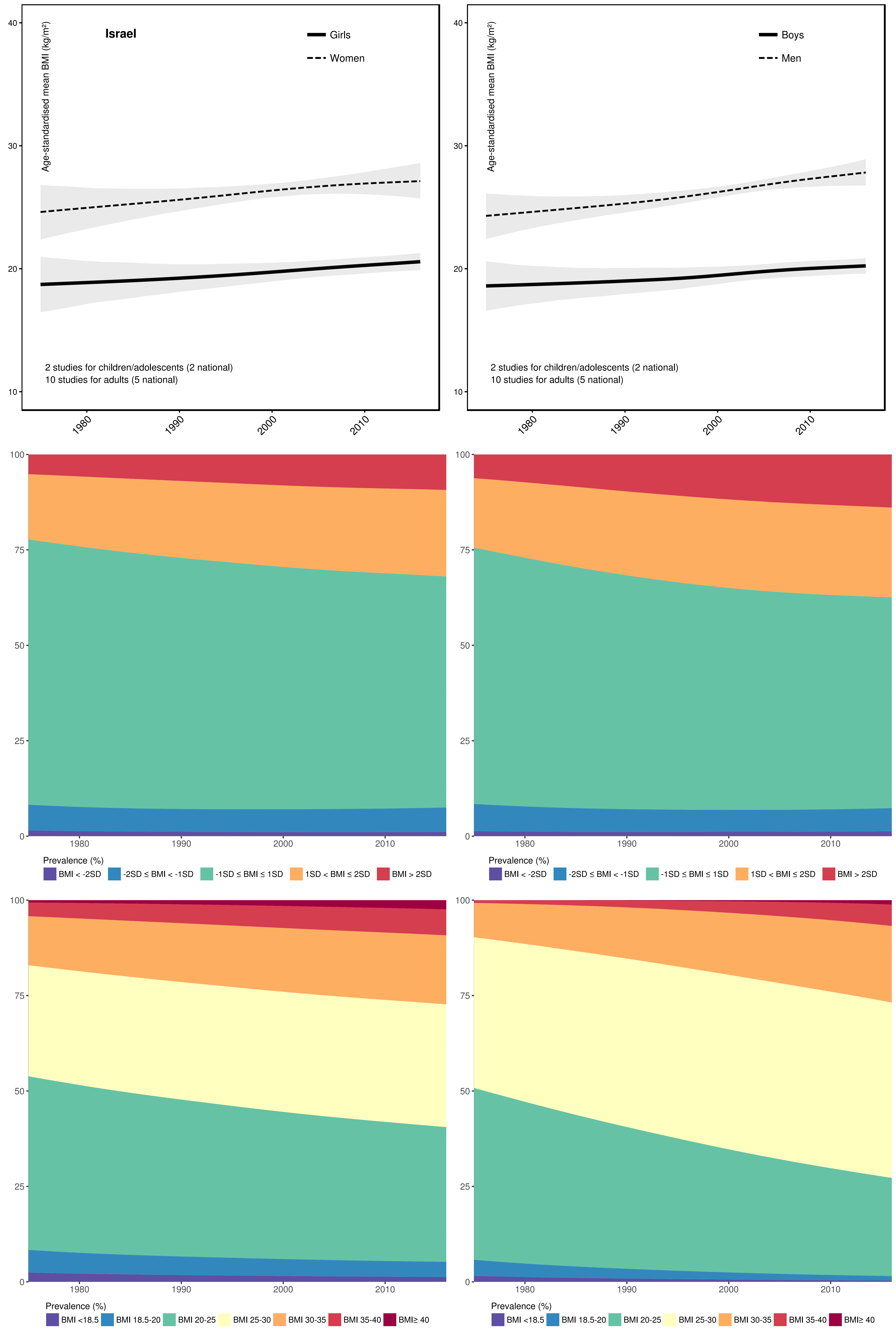
Israel
Girls
Women
Boys
Men
Age-standardised mean BMI (kg/m²)
2 studies for children/adolescents (2 national)
10 studies for adults (5 national)
Prevalence (%)
BMI < -2SD
-2SD ≤ BMI < -1SD
-1SD ≤ BMI ≤ 1SD
1SD < BMI ≤ 2SD
BMI > 2SD
BMI <18.5
BMI 18.5-20
BMI 20-25
BMI 25-30
BMI 30-35
BMI 35-40
BMI≥ 40

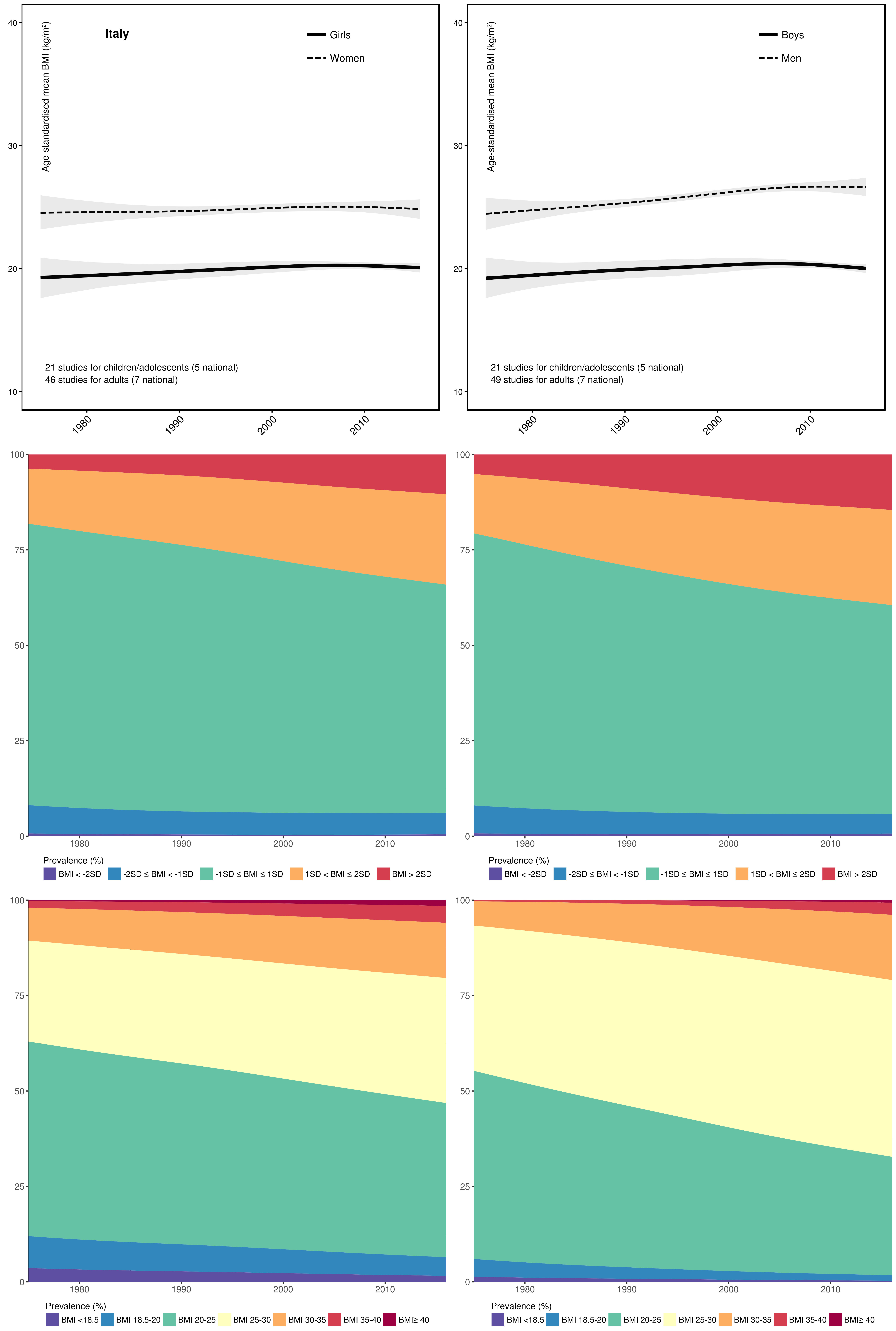
Italy
Girls
Women
Boys
Men
Age-standardised mean BMI (kg/m²)
21 studies for children/adolescents (5 national)
46 studies for adults (7 national)
21 studies for children/adolescents (5 national)
49 studies for adults (7 national)
Prevalence (%)
BMI < -2SD
-2SD ≤ BMI < -1SD
-1SD ≤ BMI ≤ 1SD
1SD < BMI ≤ 2SD
BMI > 2SD
BMI <18.5
BMI 18.5-20
BMI 20-25
BMI 25-30
BMI 30-35
BMI 35-40
BMI≥ 40

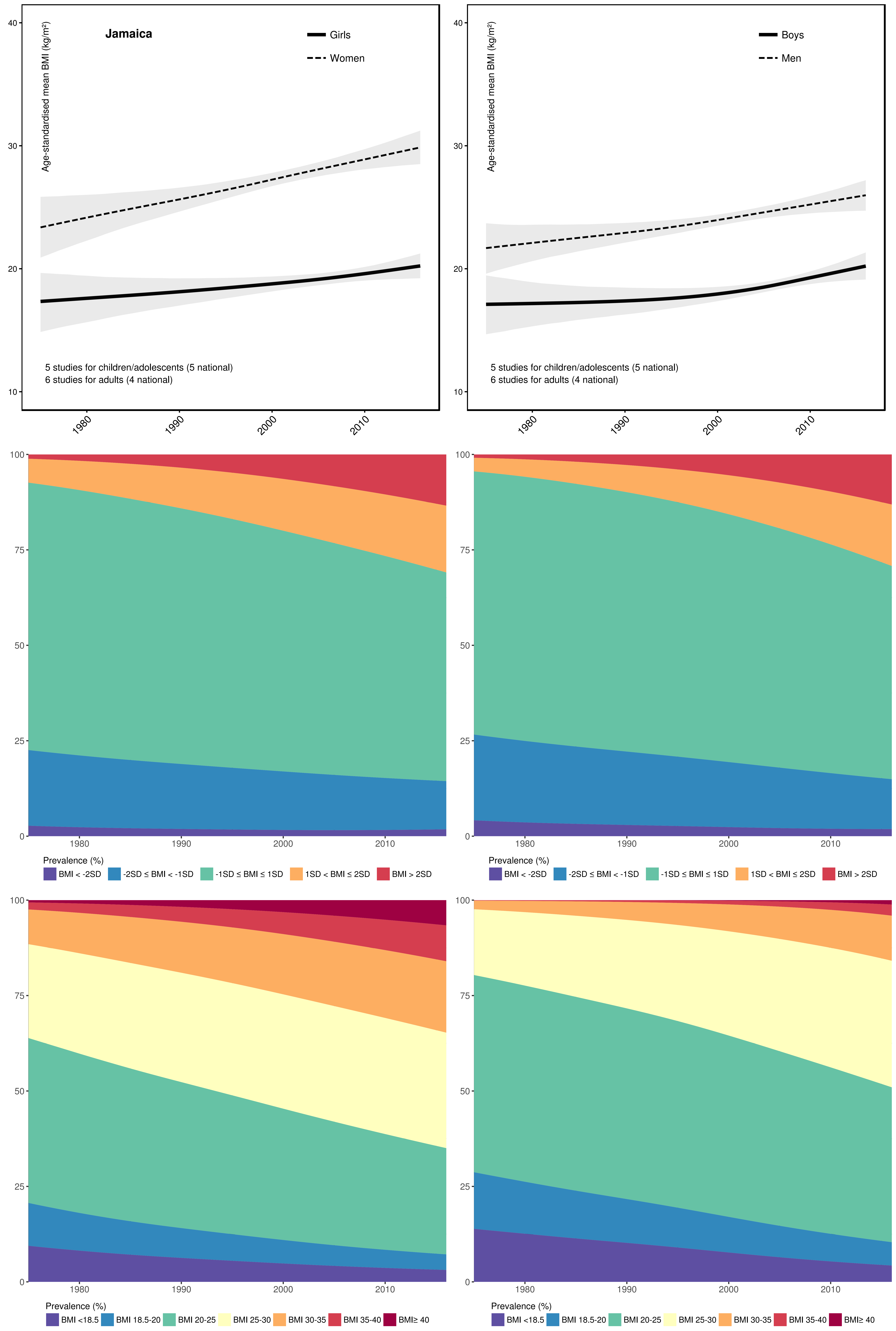
Jamaica
Girls
Women
Boys
Men
Age-standardised mean BMI (kg/m²)
5 studies for children/adolescents (5 national)
6 studies for adults (4 national)
5 studies for children/adolescents (5 national)
6 studies for adults (4 national)
Prevalence (%)
BMI < -2SD
-2SD ≤ BMI < -1SD
-1SD ≤ BMI ≤ 1SD
1SD < BMI ≤ 2SD
BMI > 2SD
Prevalence (%)
BMI <18.5
BMI 18.5-20
BMI 20-25
BMI 25-30
BMI 30-35
BMI 35-40
BMI≥ 40

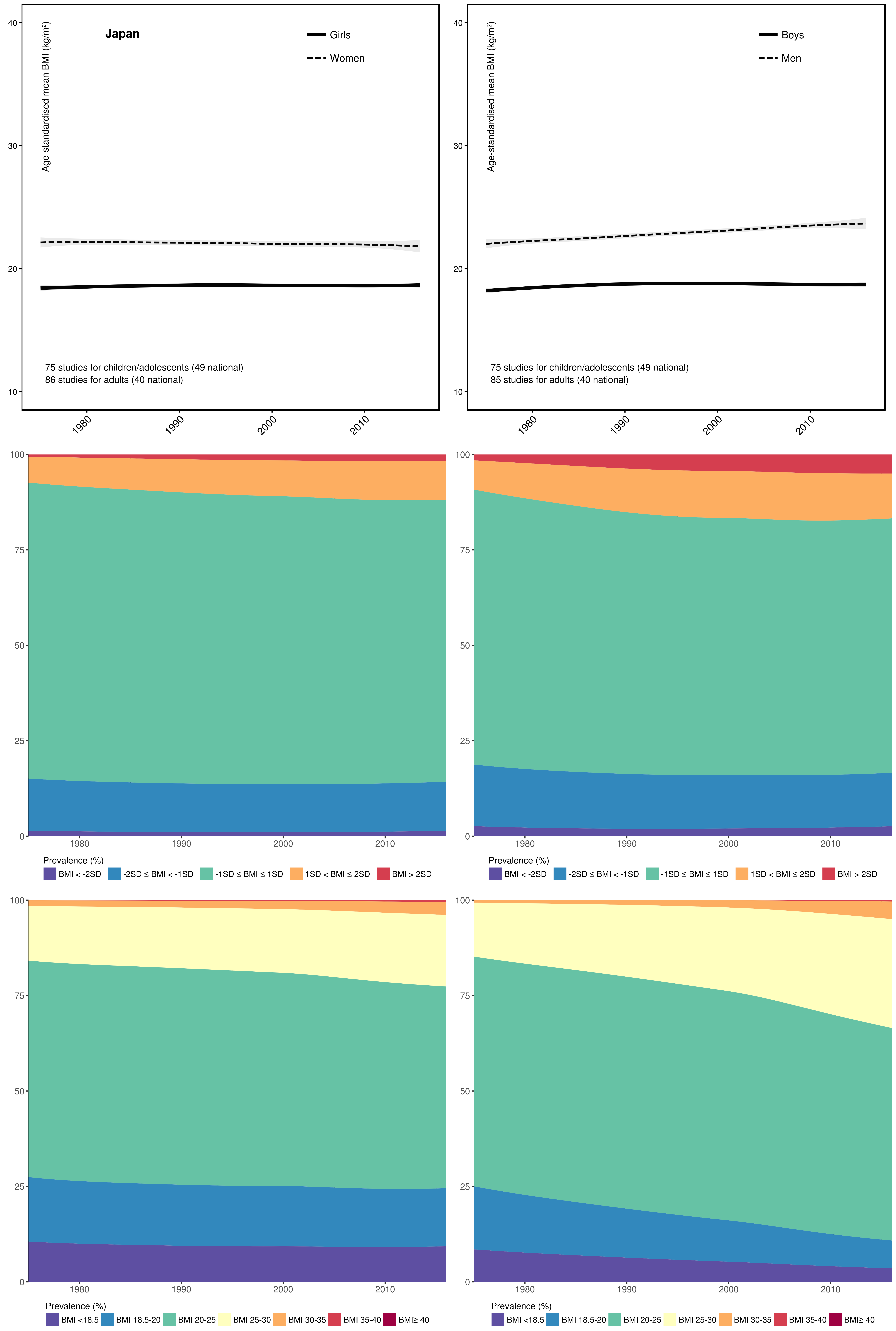
Japan
Girls
Women
Boys
Men
Age-standardised mean BMI (kg/m²)
75 studies for children/adolescents (49 national)
86 studies for adults (40 national)
75 studies for children/adolescents (49 national)
85 studies for adults (40 national)
Prevalence (%)
BMI < -2SD
-2SD ≤ BMI < -1SD
-1SD ≤ BMI ≤ 1SD
1SD < BMI ≤ 2SD
BMI > 2SD
BMI <18.5
BMI 18.5-20
BMI 20-25
BMI 25-30
BMI 30-35
BMI 35-40
BMI≥ 40

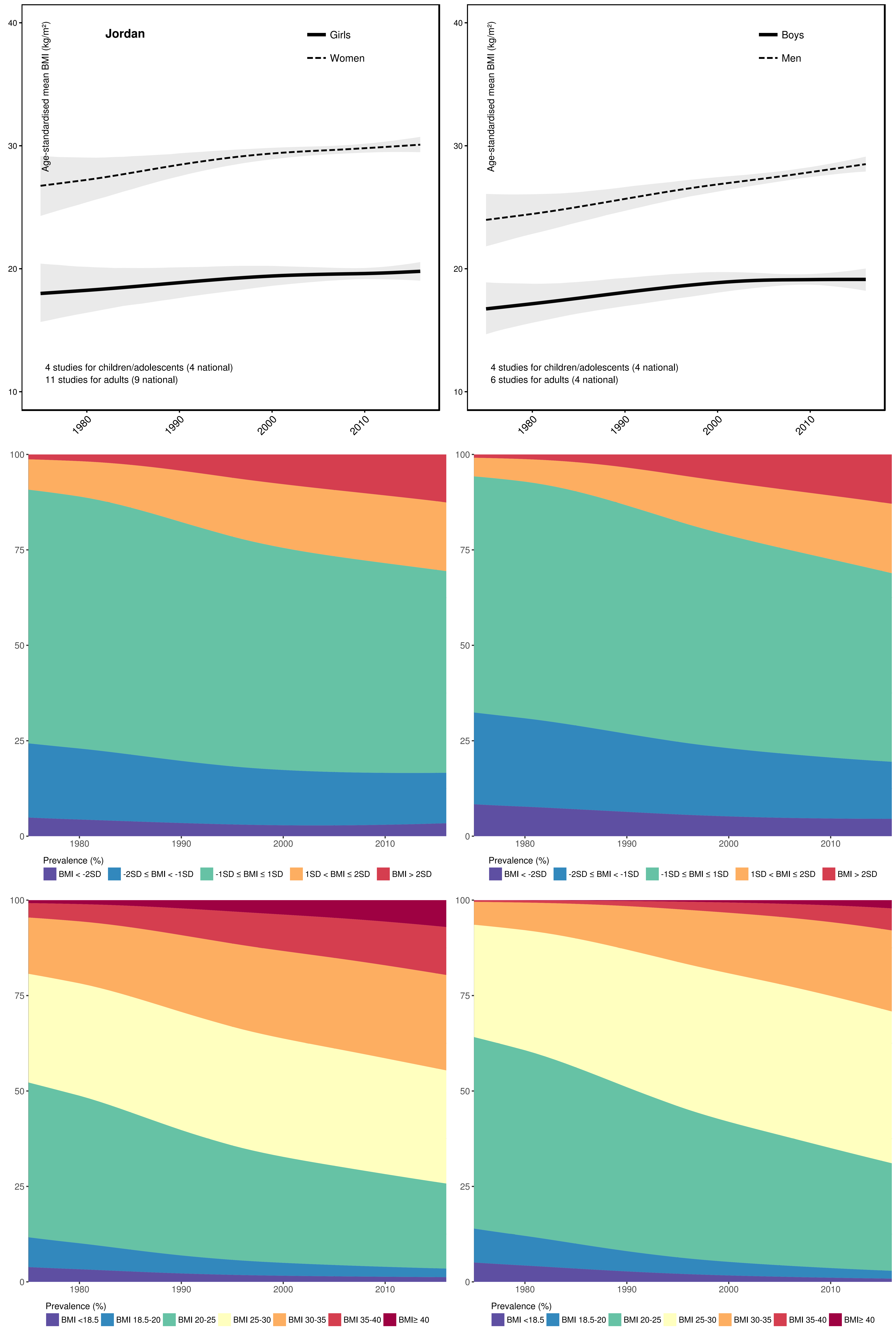
Jordan
Girls
Women
Boys
Men
Age-standardised mean BMI (kg/m²)
4 studies for children/adolescents (4 national)
11 studies for adults (9 national)
4 studies for children/adolescents (4 national)
6 studies for adults (4 national)
Prevalence (%)
BMI < -2SD
-2SD ≤ BMI < -1SD
-1SD ≤ BMI ≤ 1SD
1SD < BMI ≤ 2SD
BMI > 2SD
BMI <18.5
BMI 18.5-20
BMI 20-25
BMI 25-30
BMI 30-35
BMI 35-40
BMI≥ 40

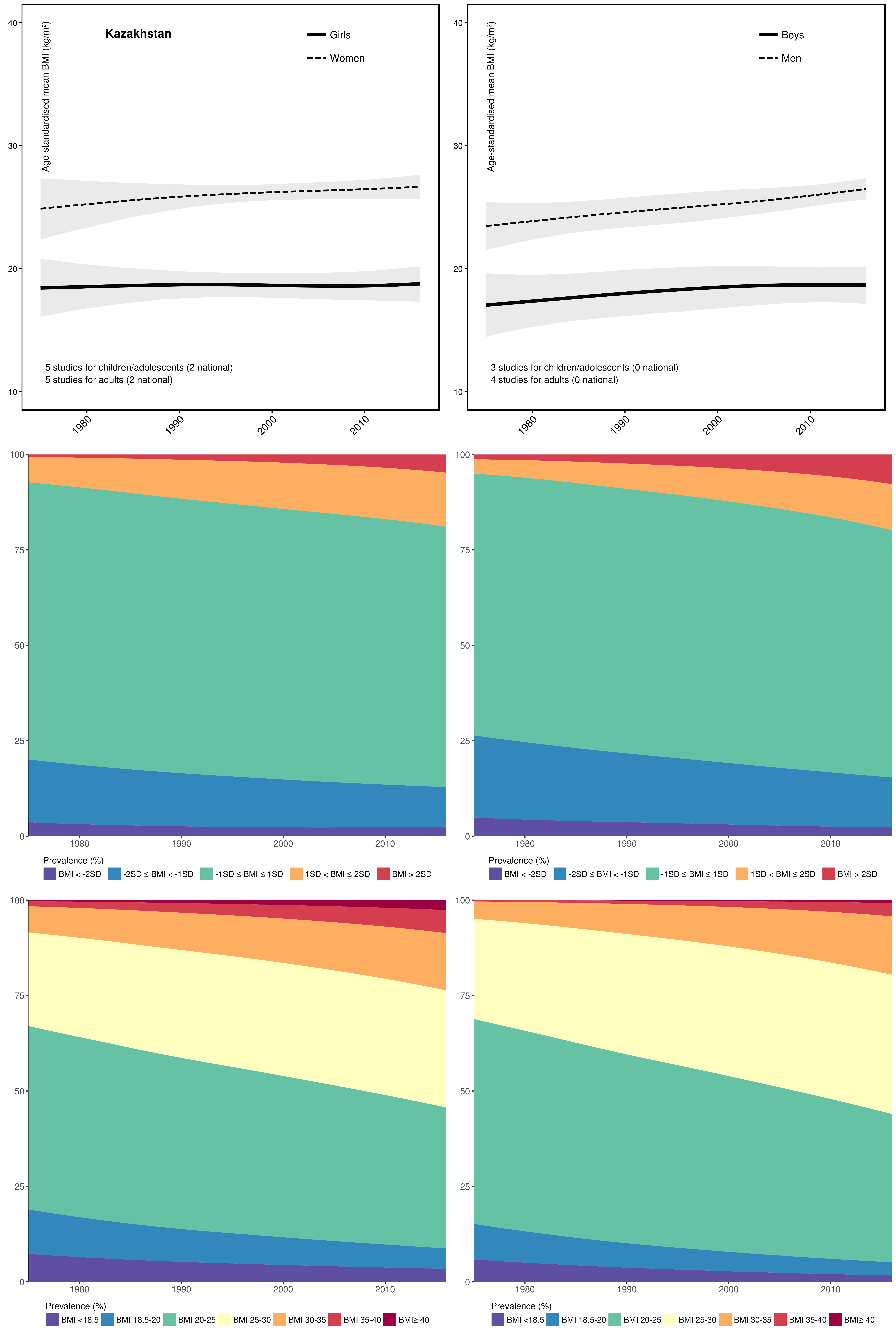
Kazakhstan
Girls
Women
Age-standardised mean BMI (kg/m²)
5 studies for children/adolescents (2 national)
5 studies for adults (2 national)
Boys
Men
3 studies for children/adolescents (0 national)
4 studies for adults (0 national)
40
30
20
10
1980
1990
2000
2010
100
75
50
25
0
Prevalence (%)
BMI < -2SD
-2SD ≤ BMI < -1SD
-1SD ≤ BMI ≤ 1SD
1SD < BMI ≤ 2SD
BMI > 2SD
BMI <18.5
BMI 18.5-20
BMI 20-25
BMI 25-30
BMI 30-35
BMI 35-40
BMI≥ 40

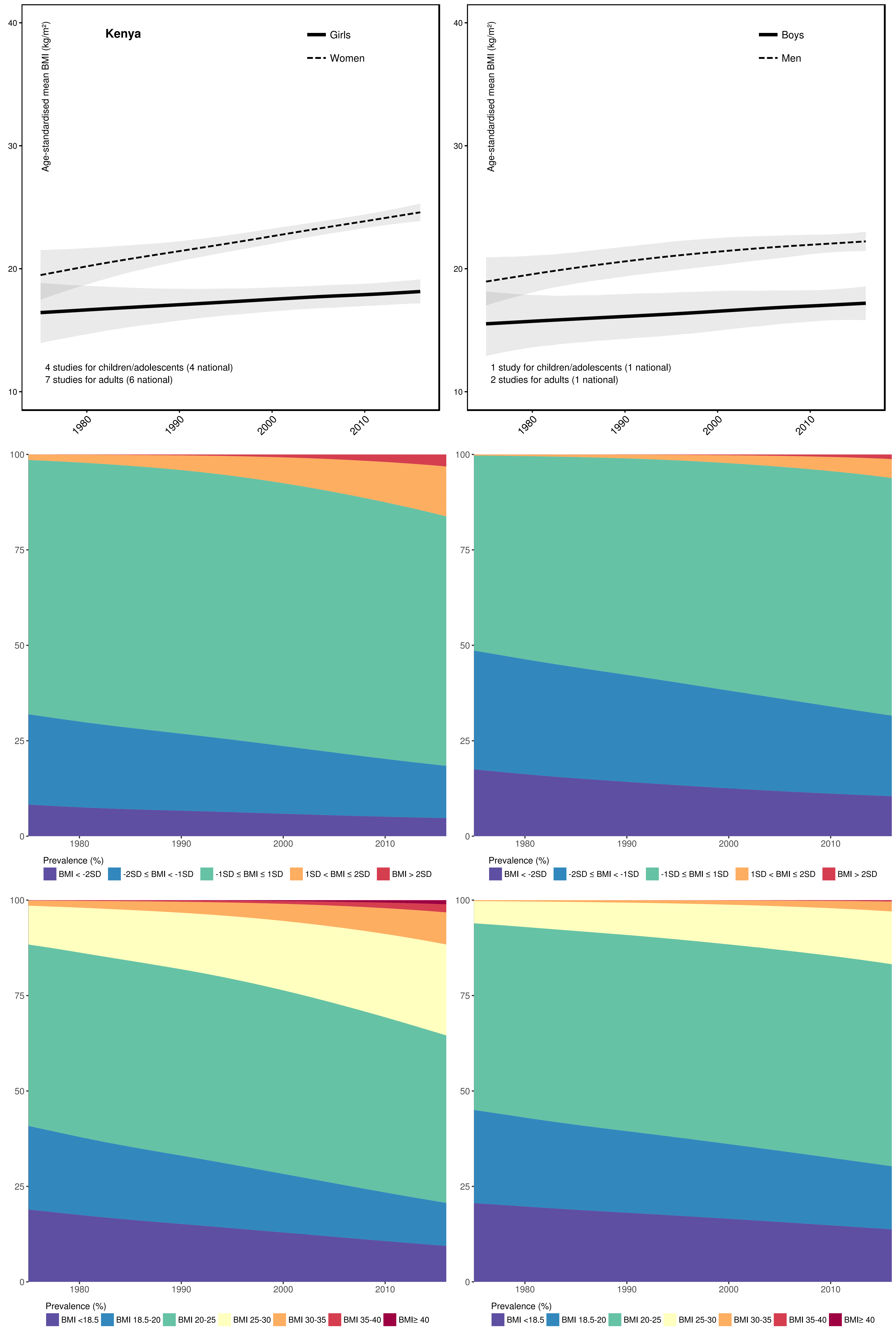
Kenya
Girls
Women
Boys
Men
Age-standardised mean BMI (kg/m²)
4 studies for children/adolescents (4 national)
7 studies for adults (6 national)
1 study for children/adolescents (1 national)
2 studies for adults (1 national)
Prevalence (%)
BMI < -2SD
-2SD ≤ BMI < -1SD
-1SD ≤ BMI ≤ 1SD
1SD < BMI ≤ 2SD
BMI > 2SD
BMI <18.5
BMI 18.5-20
BMI 20-25
BMI 25-30
BMI 30-35
BMI 35-40
BMI≥ 40

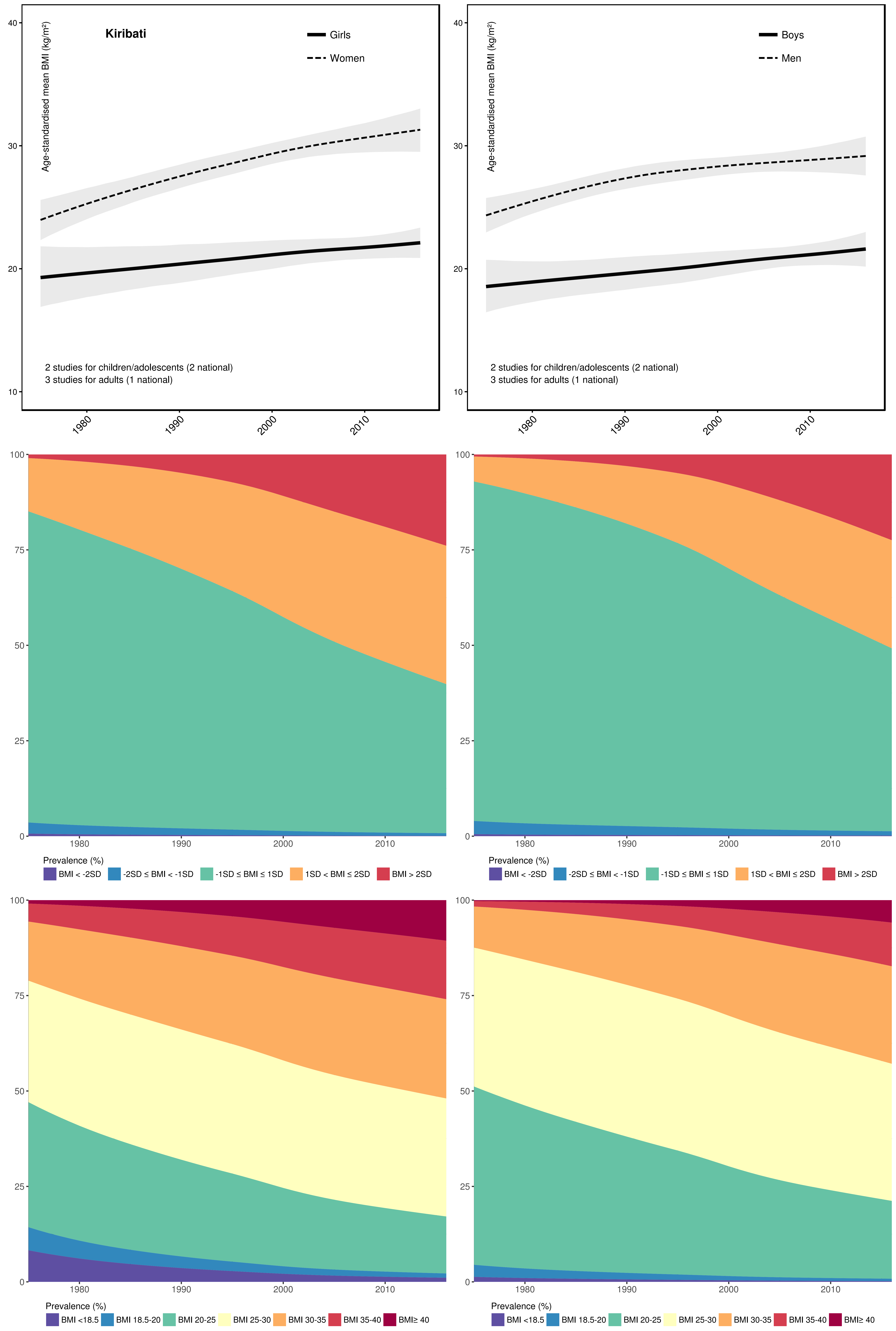
Kiribati
Girls
Women
Boys
Men
Age-standardised mean BMI (kg/m²)
2 studies for children/adolescents (2 national)
3 studies for adults (1 national)
Prevalence (%)
BMI < -2SD
-2SD ≤ BMI < -1SD
-1SD ≤ BMI ≤ 1SD
1SD < BMI ≤ 2SD
BMI > 2SD
BMI <18.5
BMI 18.5-20
BMI 20-25
BMI 25-30
BMI 30-35
BMI 35-40
BMI≥ 40

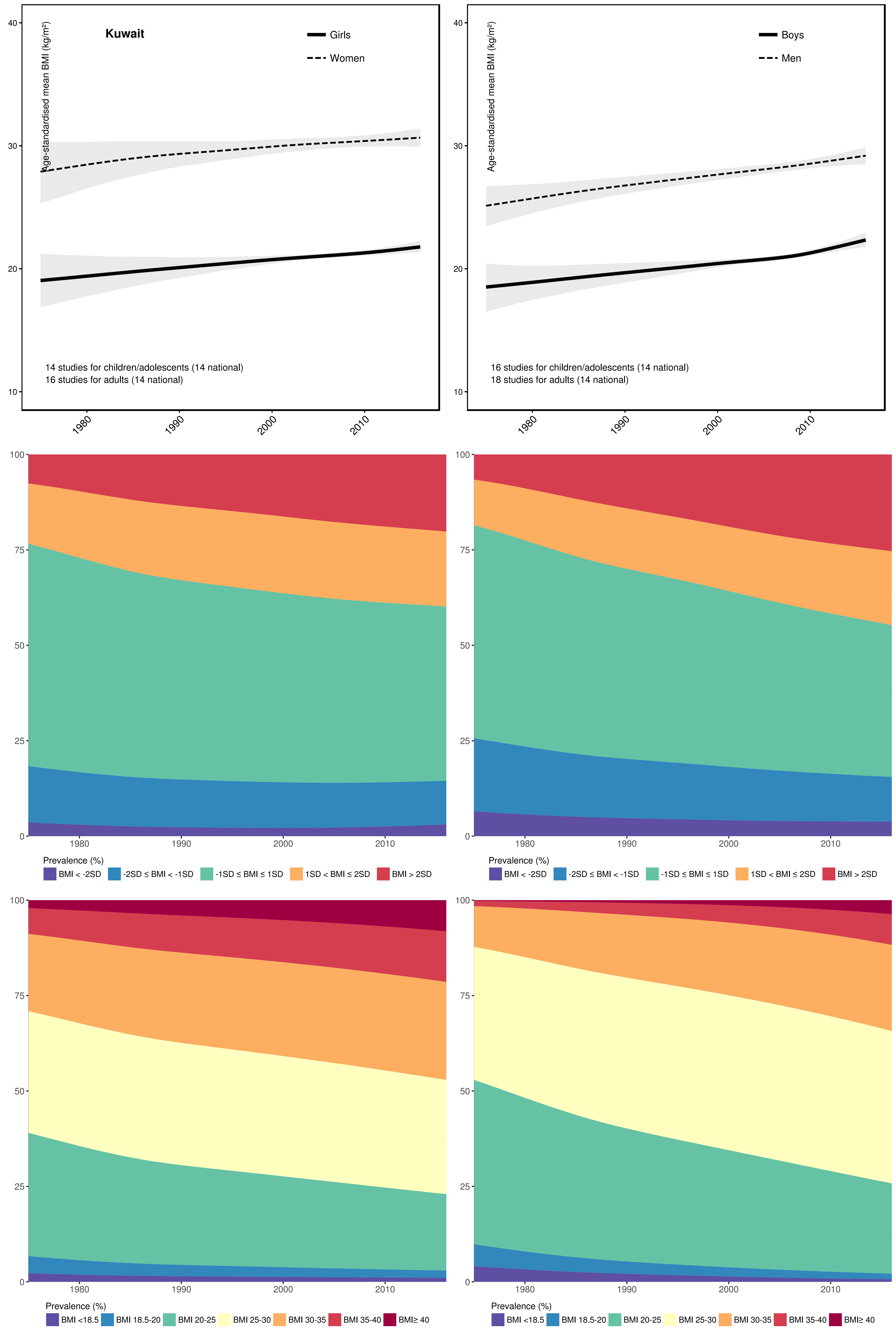
Kuwait
Girls
Women
Boys
Men
Age-standardised mean BMI (kg/m²)
14 studies for children/adolescents (14 national)
16 studies for adults (14 national)
16 studies for children/adolescents (14 national)
18 studies for adults (14 national)
Prevalence (%)
BMI < -2SD
-2SD ≤ BMI < -1SD
-1SD ≤ BMI ≤ 1SD
1SD < BMI ≤ 2SD
BMI > 2SD
BMI <18.5
BMI 18.5-20
BMI 20-25
BMI 25-30
BMI 30-35
BMI 35-40
BMI≥ 40

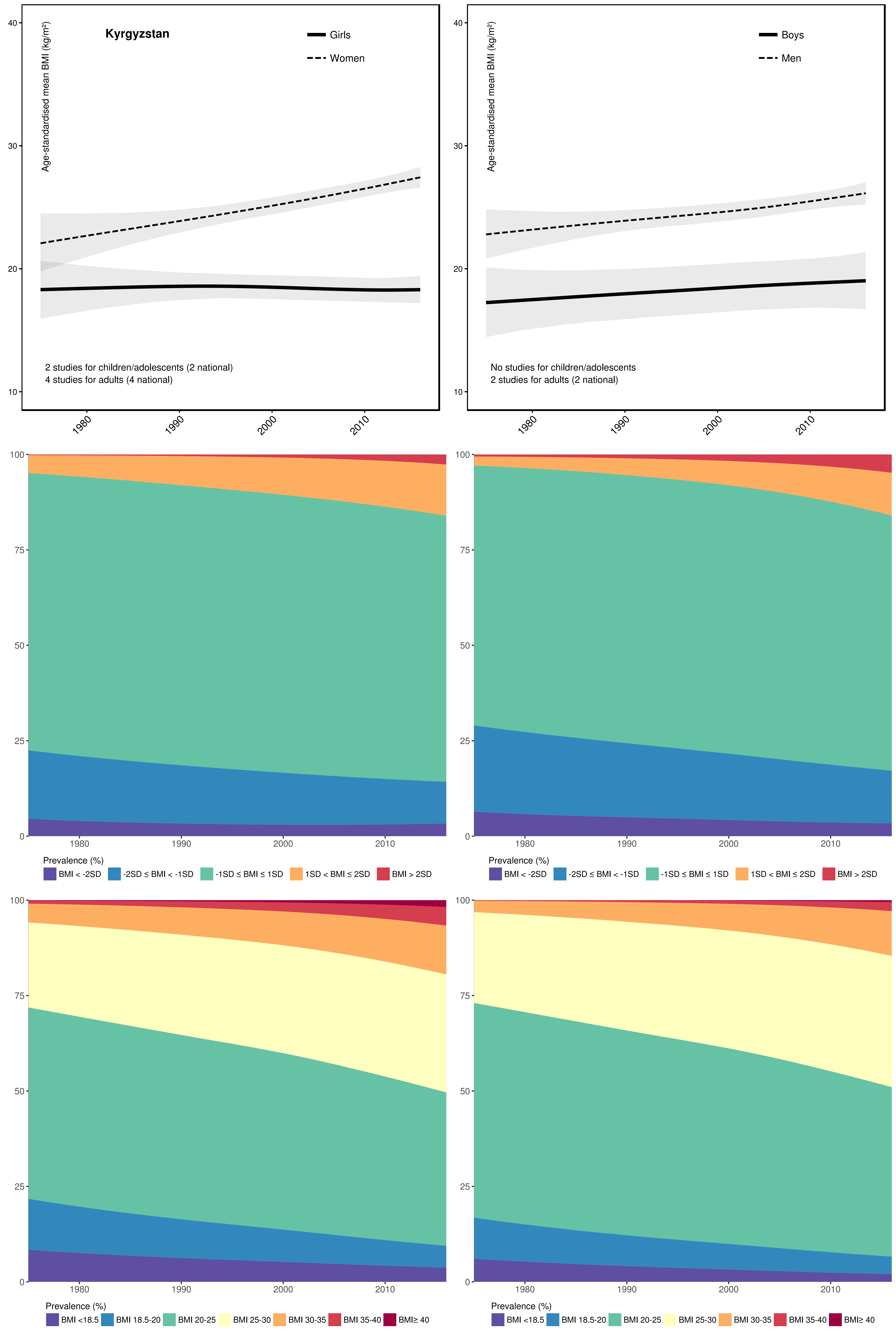
Kyrgyzstan
Girls
Women
Boys
Men
Age-standardised mean BMI (kg/m²)
2 studies for children/adolescents (2 national)
4 studies for adults (4 national)
No studies for children/adolescents
2 studies for adults (2 national)
Prevalence (%)
BMI < -2SD
-2SD ≤ BMI < -1SD
-1SD ≤ BMI ≤ 1SD
1SD < BMI ≤ 2SD
BMI > 2SD
BMI <18.5
BMI 18.5-20
BMI 20-25
BMI 25-30
BMI 30-35
BMI 35-40
BMI≥ 40

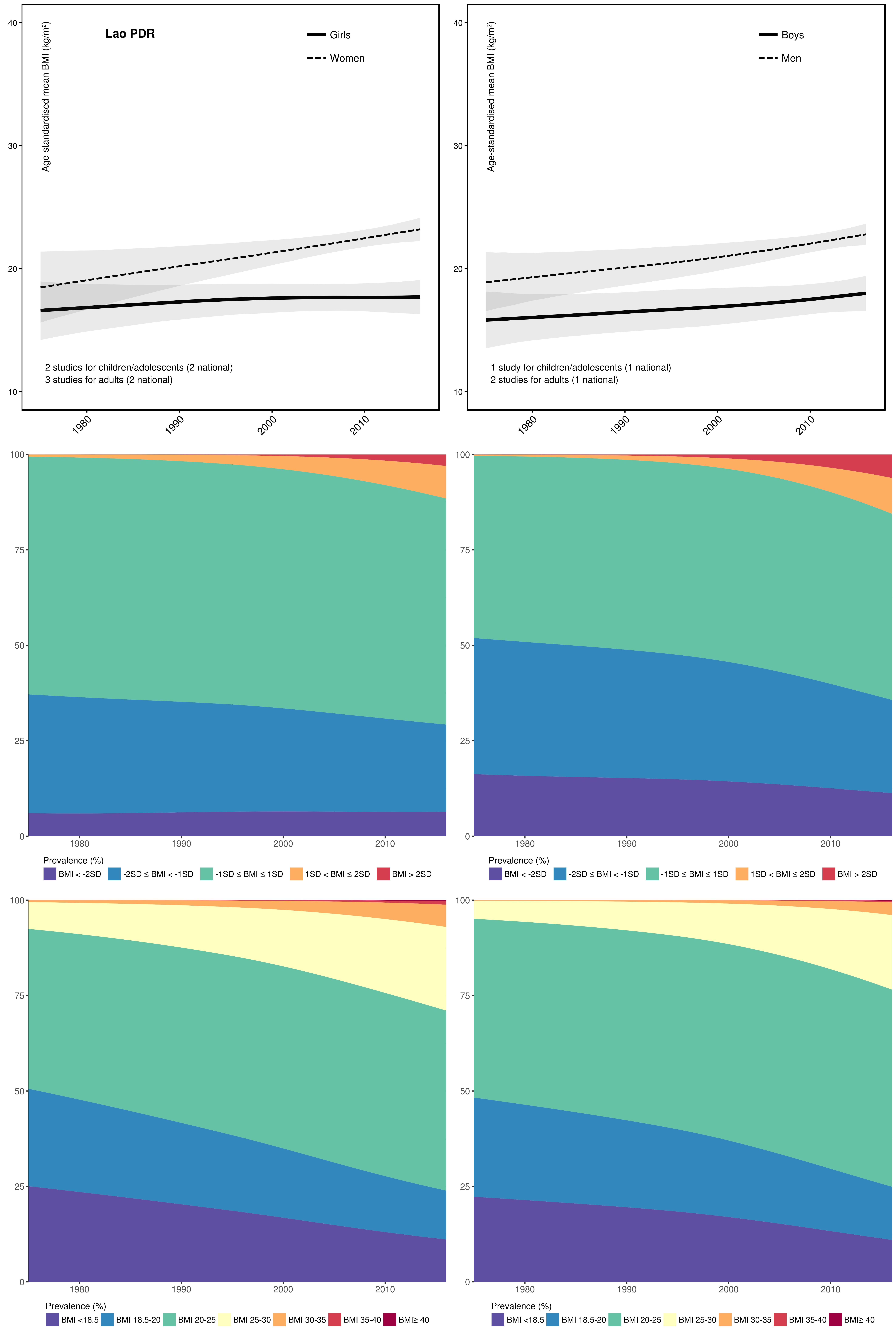

Lao PDR
Girls
Women
Boys
Men
Age-standardised mean BMI (kg/m²)
2 studies for children/adolescents (2 national)
3 studies for adults (2 national)
1 study for children/adolescents (1 national)
2 studies for adults (1 national)
1980
1990
2000
2010
Prevalence (%)
BMI < -2SD
-2SD ≤ BMI < -1SD
-1SD ≤ BMI ≤ 1SD
1SD < BMI ≤ 2SD
BMI > 2SD
BMI <18.5
BMI 18.5-20
BMI 20-25
BMI 25-30
BMI 30-35
BMI 35-40
BMI≥ 40

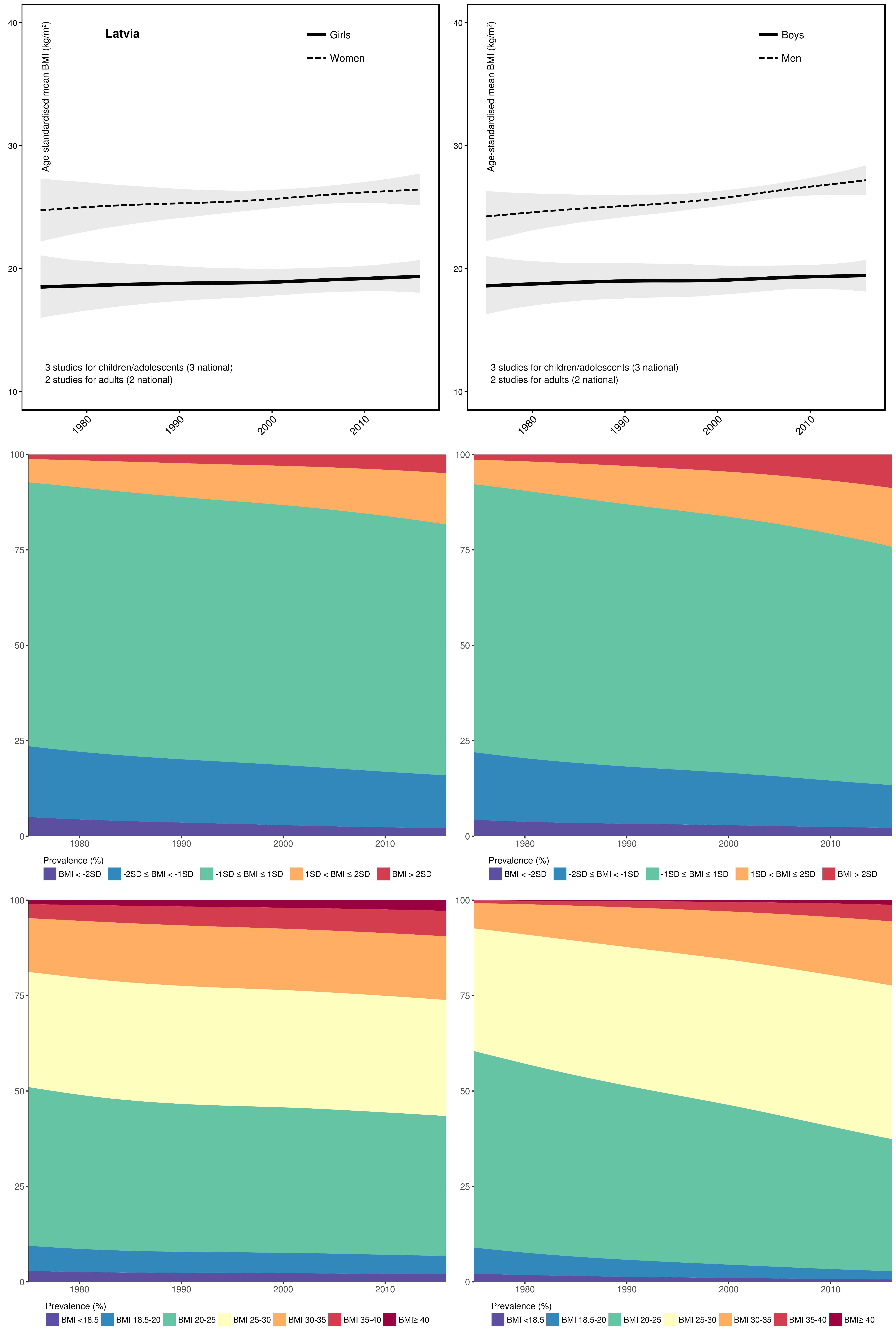
Latvia
Girls
Women
Boys
Men
Age-standardised mean BMI (kg/m²)
3 studies for children/adolescents (3 national)
2 studies for adults (2 national)
3 studies for children/adolescents (3 national)
2 studies for adults (2 national)
Prevalence (%)
BMI < -2SD
-2SD ≤ BMI < -1SD
-1SD ≤ BMI ≤ 1SD
1SD < BMI ≤ 2SD
BMI > 2SD
Prevalence (%)
BMI <18.5
BMI 18.5-20
BMI 20-25
BMI 25-30
BMI 30-35
BMI 35-40
BMI≥ 40

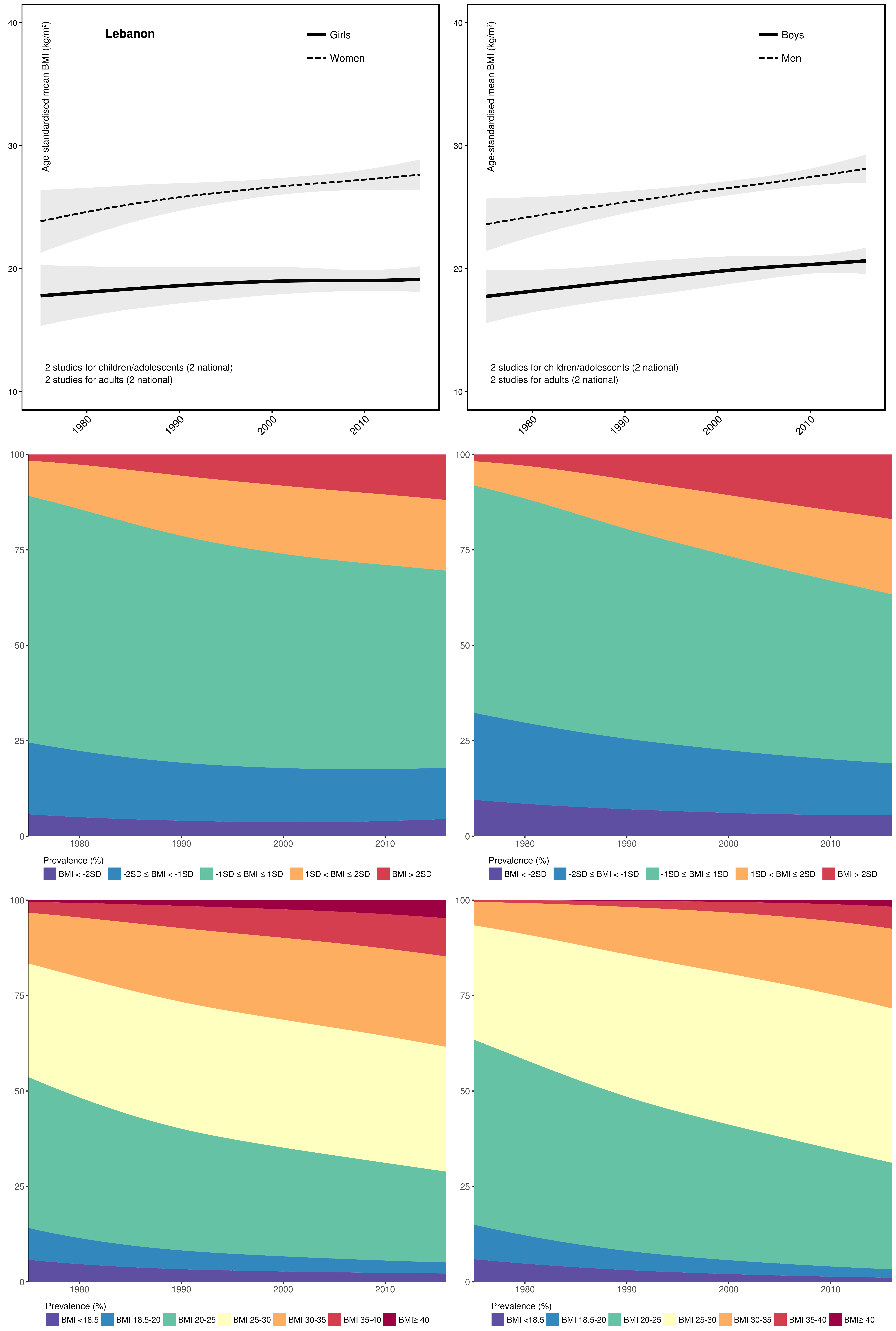
Lebanon
Girls
Women
Boys
Men
Age-standardised mean BMI (kg/m²)
2 studies for children/adolescents (2 national)
2 studies for adults (2 national)
1980
1990
2000
2010
Prevalence (%)
BMI < -2SD
-2SD ≤ BMI < -1SD
-1SD ≤ BMI ≤ 1SD
1SD < BMI ≤ 2SD
BMI > 2SD
BMI <18.5
BMI 18.5-20
BMI 20-25
BMI 25-30
BMI 30-35
BMI 35-40
BMI≥ 40

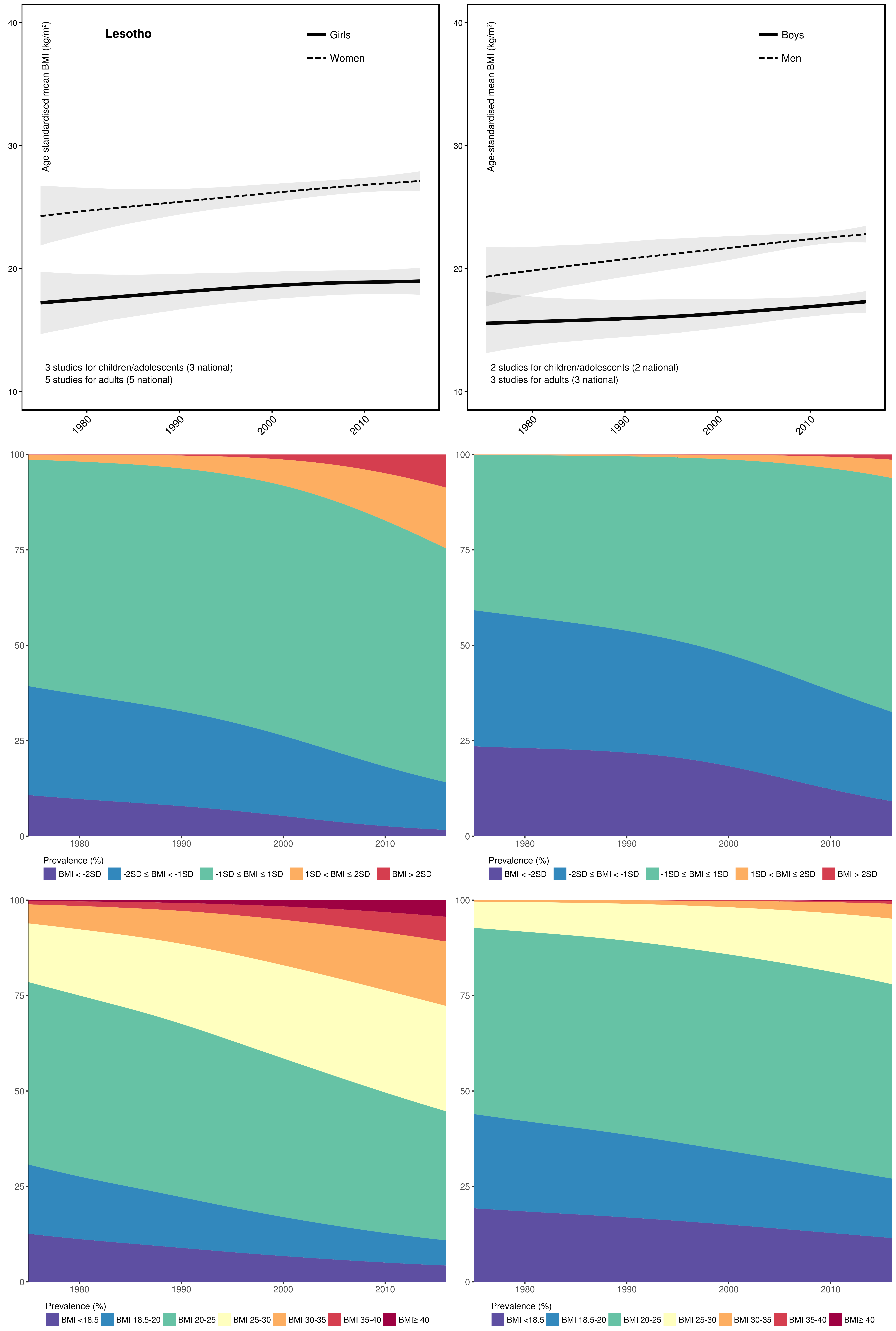
Lesotho
Girls
Women
Age-standardised mean BMI (kg/m²)
3 studies for children/adolescents (3 national)
5 studies for adults (5 national)
Boys
Men
2 studies for children/adolescents (2 national)
3 studies for adults (3 national)
Prevalence (%)
BMI < -2SD
-2SD ≤ BMI < -1SD
-1SD ≤ BMI ≤ 1SD
1SD < BMI ≤ 2SD
BMI > 2SD
Prevalence (%)
BMI <18.5
BMI 18.5-20
BMI 20-25
BMI 25-30
BMI 30-35
BMI 35-40
BMI≥ 40

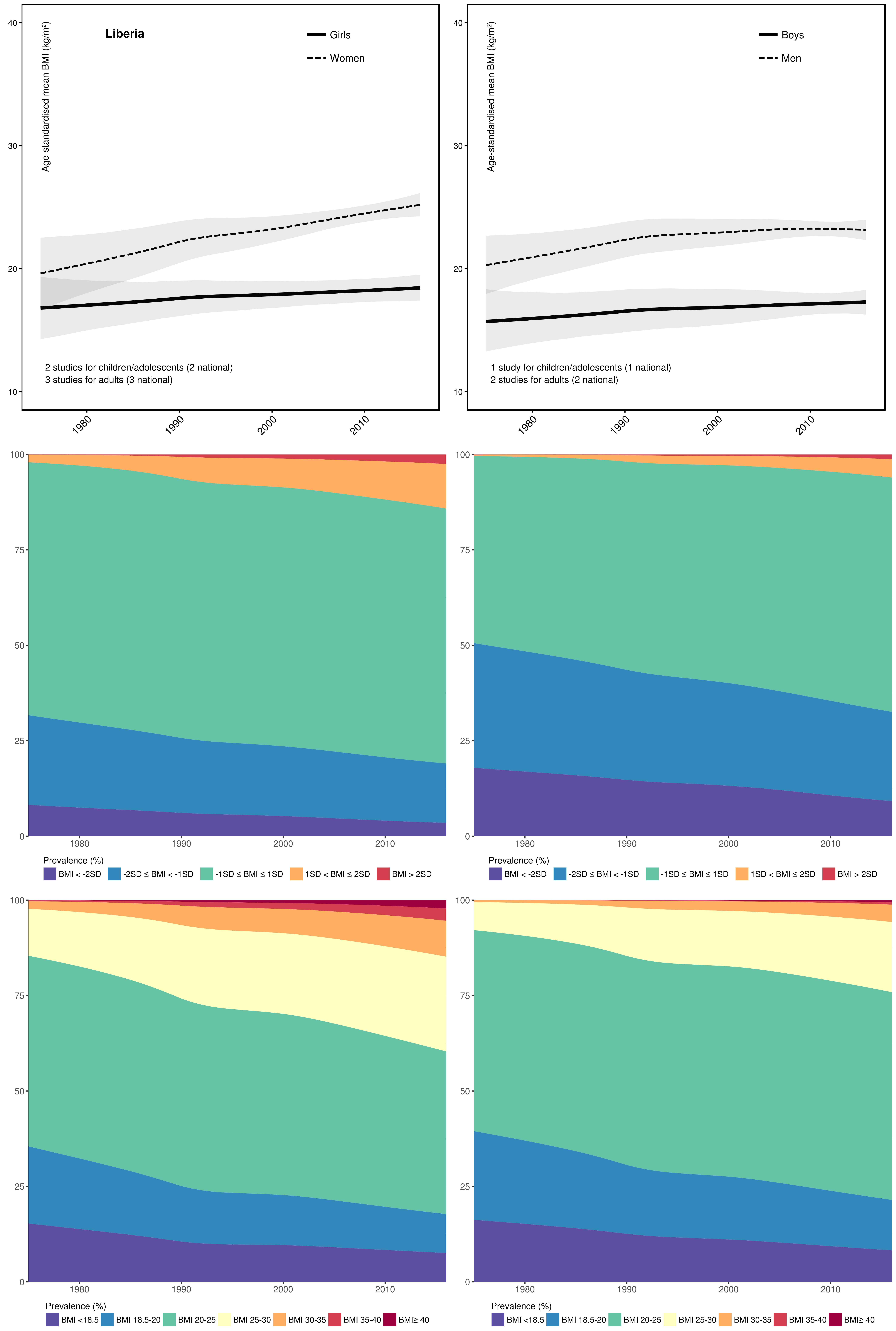
Liberia
Girls
Women
Boys
Men
Age-standardised mean BMI (kg/m²)
2 studies for children/adolescents (2 national)
3 studies for adults (3 national)
1 study for children/adolescents (1 national)
2 studies for adults (2 national)
Prevalence (%)
BMI < -2SD
-2SD ≤ BMI < -1SD
-1SD ≤ BMI ≤ 1SD
1SD < BMI ≤ 2SD
BMI > 2SD
BMI <18.5
BMI 18.5-20
BMI 20-25
BMI 25-30
BMI 30-35
BMI 35-40
BMI≥ 40

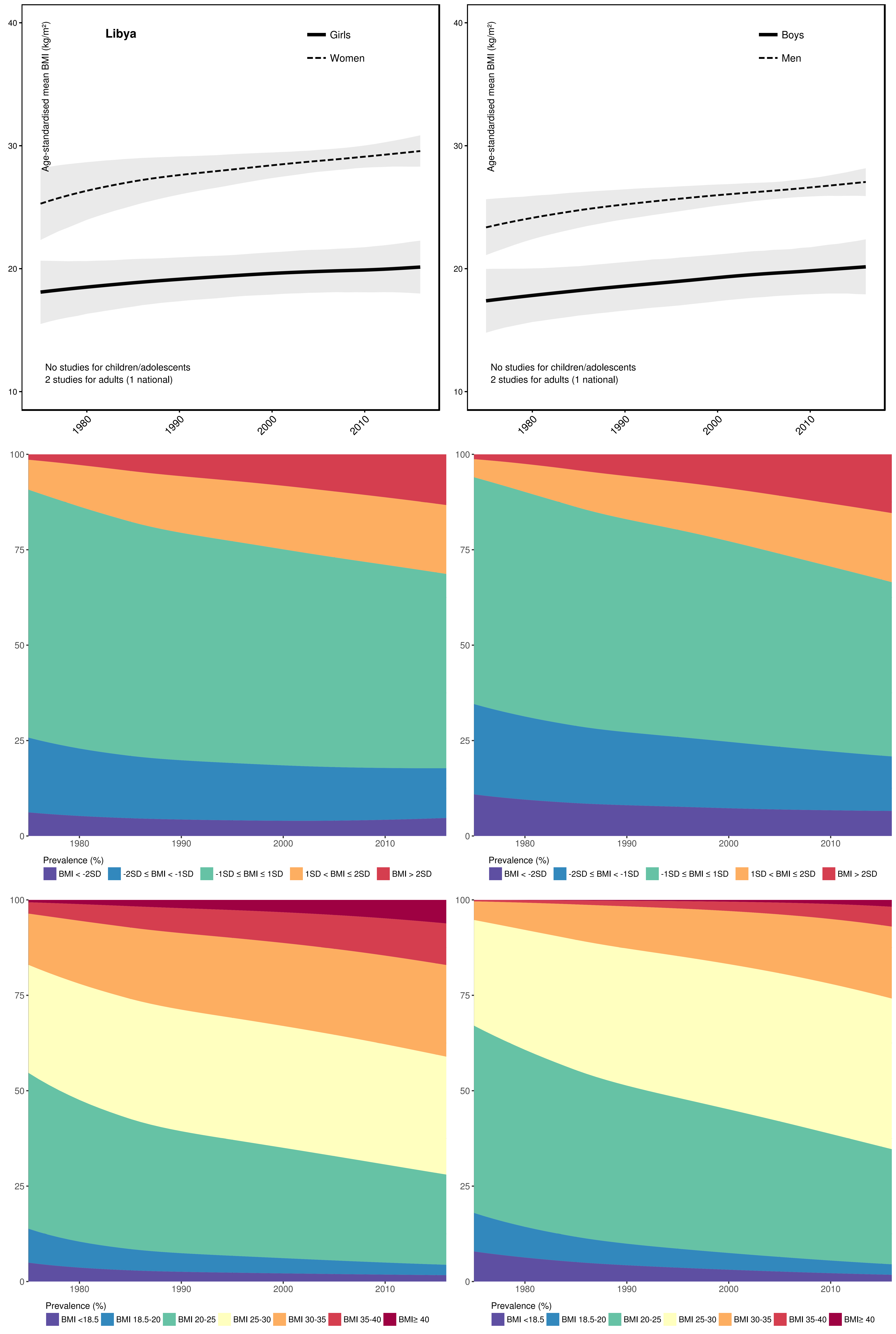
Libya
Girls
Women
Boys
Men
Age-standardised mean BMI (kg/m²)
No studies for children/adolescents
2 studies for adults (1 national)
1980
1990
2000
2010
Prevalence (%)
BMI < -2SD
-2SD ≤ BMI < -1SD
-1SD ≤ BMI ≤ 1SD
1SD < BMI ≤ 2SD
BMI > 2SD
BMI <18.5
BMI 18.5-20
BMI 20-25
BMI 25-30
BMI 30-35
BMI 35-40
BMI≥ 40

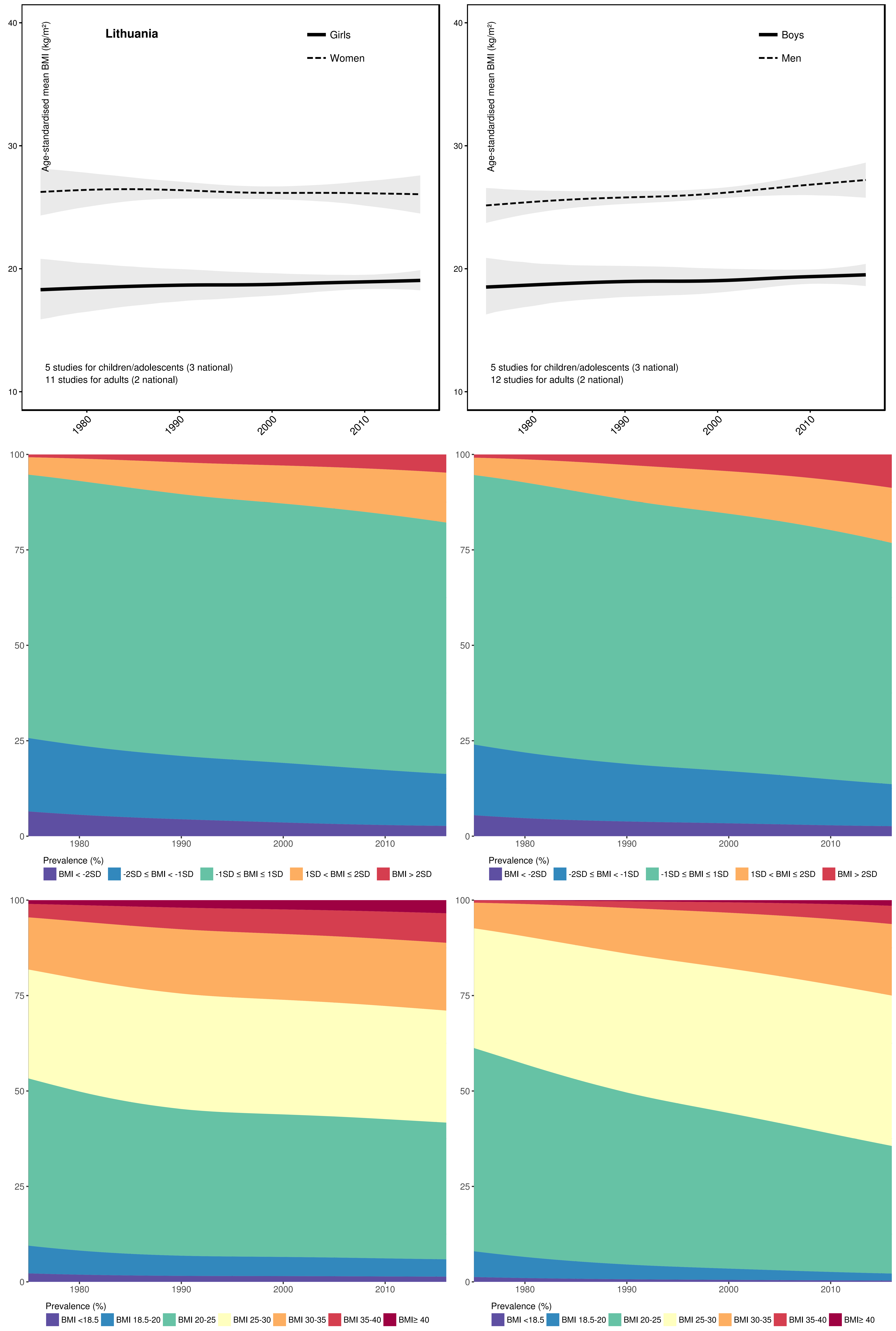
Lithuania
Girls
Women
Boys
Men
Age-standardised mean BMI (kg/m²)
5 studies for children/adolescents (3 national)
11 studies for adults (2 national)
5 studies for children/adolescents (3 national)
12 studies for adults (2 national)
Prevalence (%)
BMI < -2SD
-2SD ≤ BMI < -1SD
-1SD ≤ BMI ≤ 1SD
1SD < BMI ≤ 2SD
BMI > 2SD
BMI <18.5
BMI 18.5-20
BMI 20-25
BMI 25-30
BMI 30-35
BMI 35-40
BMI≥ 40

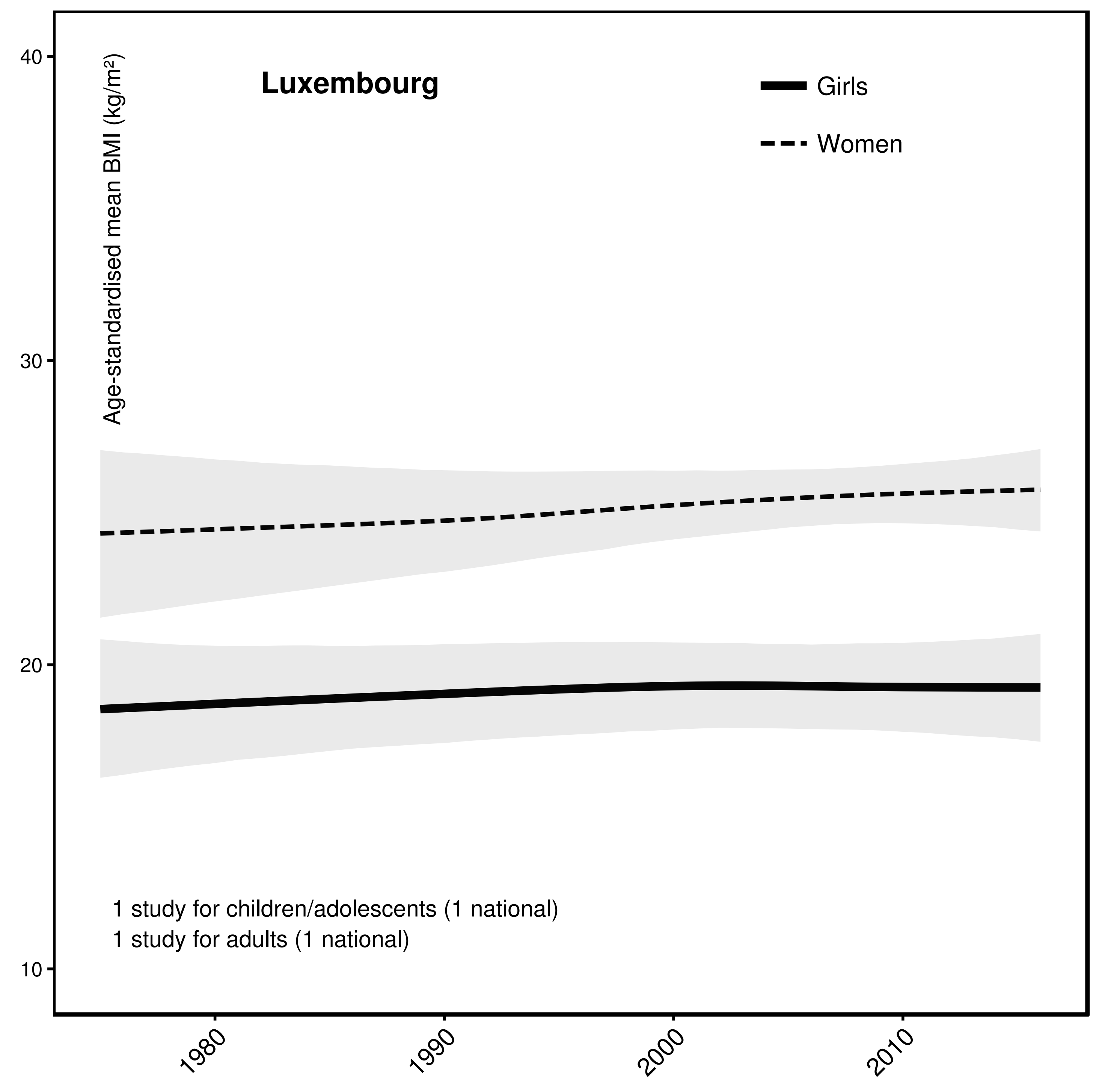
Luxembourg
Girls
Women
Age-standardised mean BMI (kg/m²)
40
30
20
10
1 study for children/adolescents (1 national)
1 study for adults (1 national)
1980
1990
2000
2010

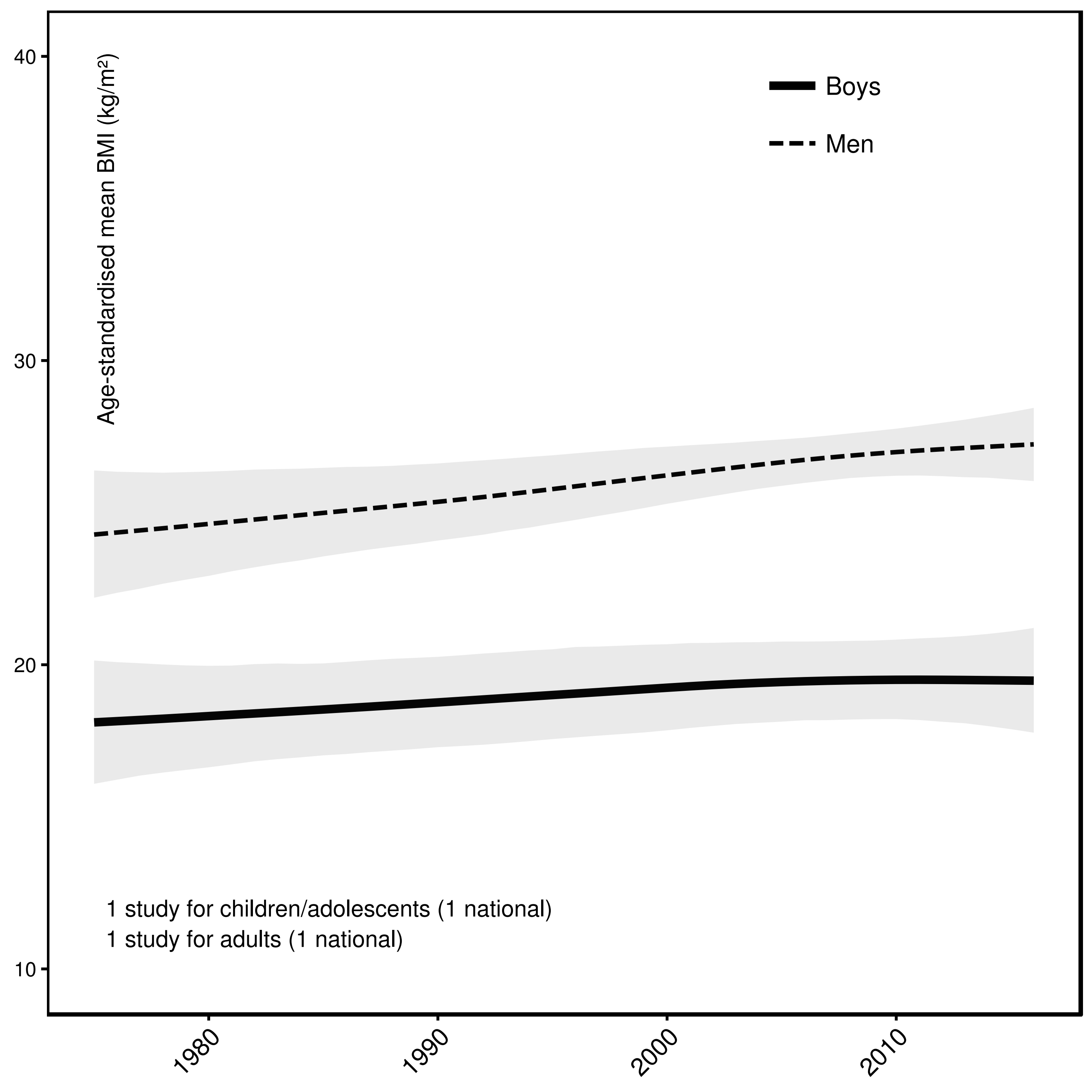
Boys
Men
Age-standardised mean BMI (kg/m²)
40
30
20
10
1 study for children/adolescents (1 national)
1 study for adults (1 national)
1980
1990
2000
2010

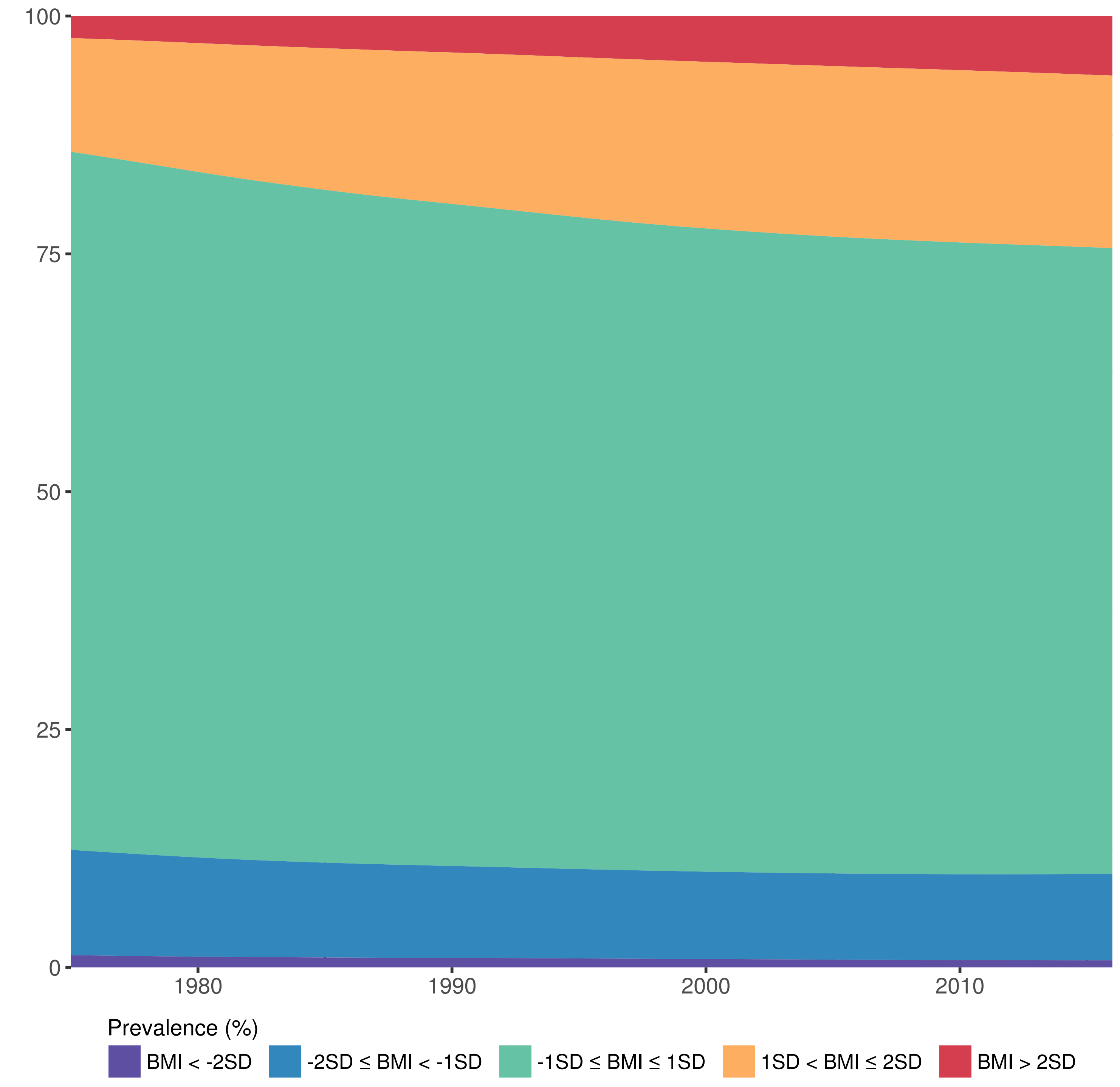
100
75
50
25
0
1980
1990
2000
2010
Prevalence (%)
BMI < -2SD
-2SD ≤ BMI < -1SD
-1SD ≤ BMI ≤ 1SD
1SD < BMI ≤ 2SD
BMI > 2SD

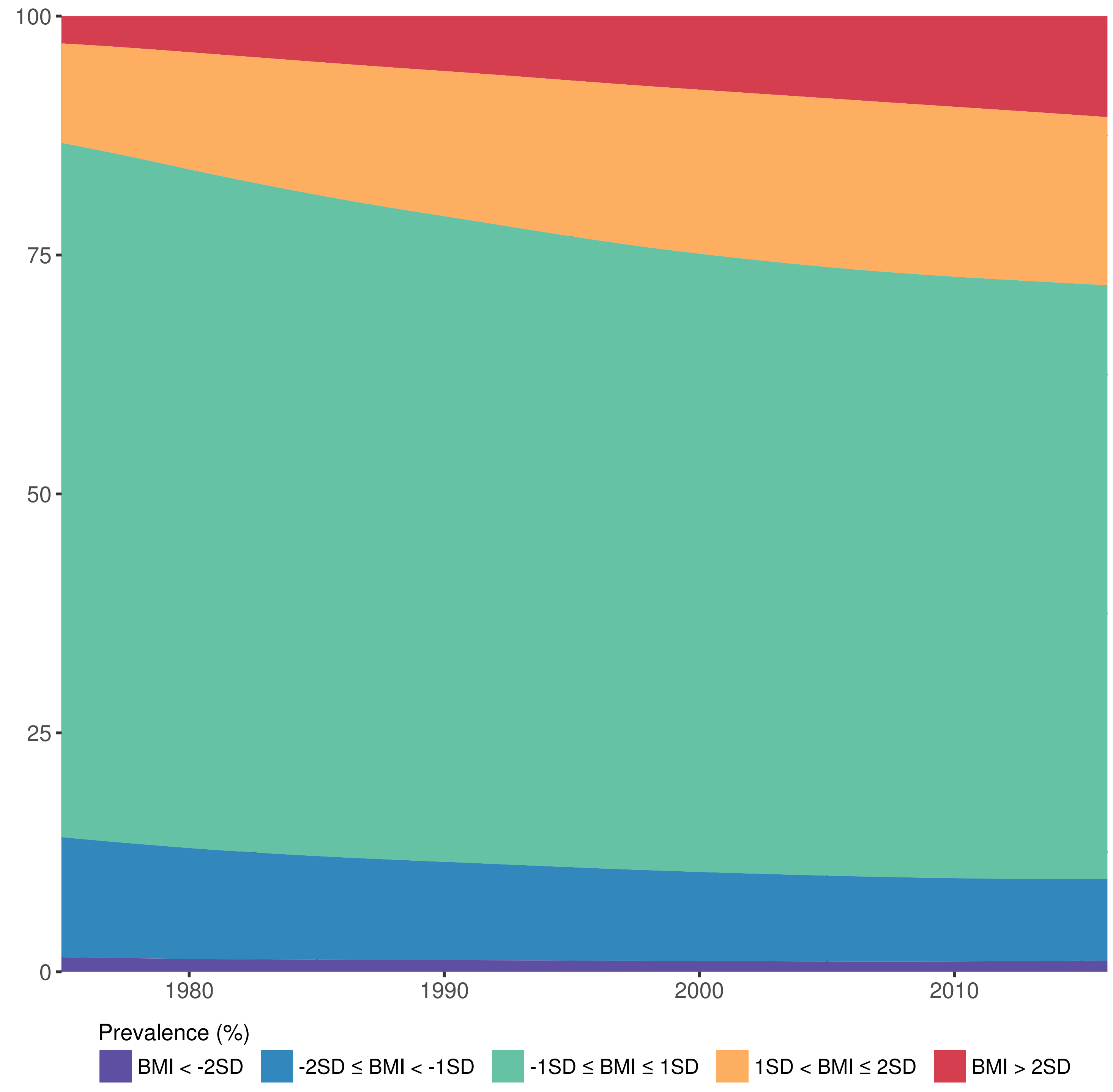
100
75
50
25
0
1980
1990
2000
2010
Prevalence (%)
BMI < -2SD
-2SD ≤ BMI < -1SD
-1SD ≤ BMI ≤ 1SD
1SD < BMI ≤ 2SD
BMI > 2SD

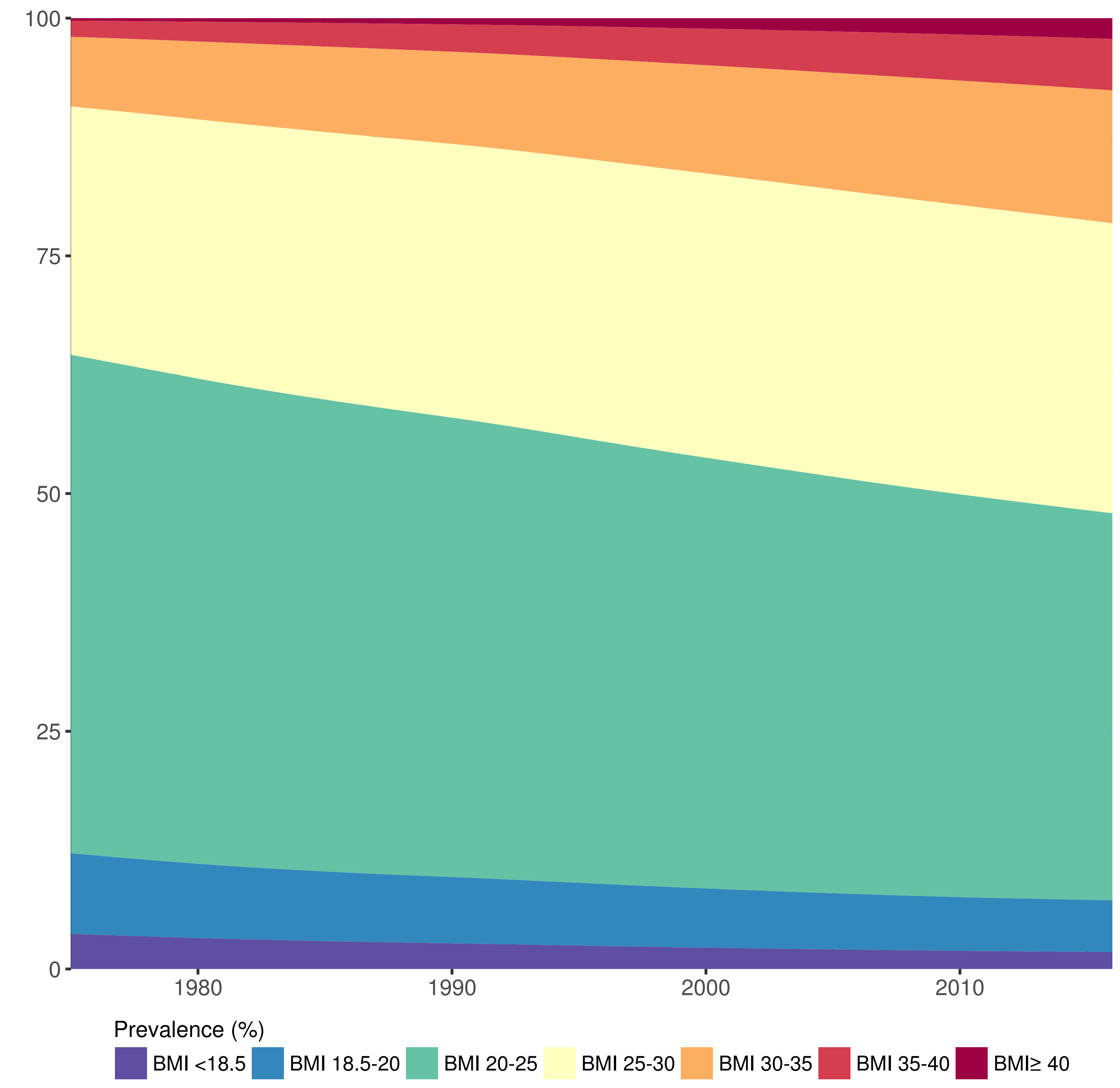
100
75
50
25
0
1980
1990
2000
2010
Prevalence (%)
BMI <18.5
BMI 18.5-20
BMI 20-25
BMI 25-30
BMI 30-35
BMI 35-40
BMI≥ 40

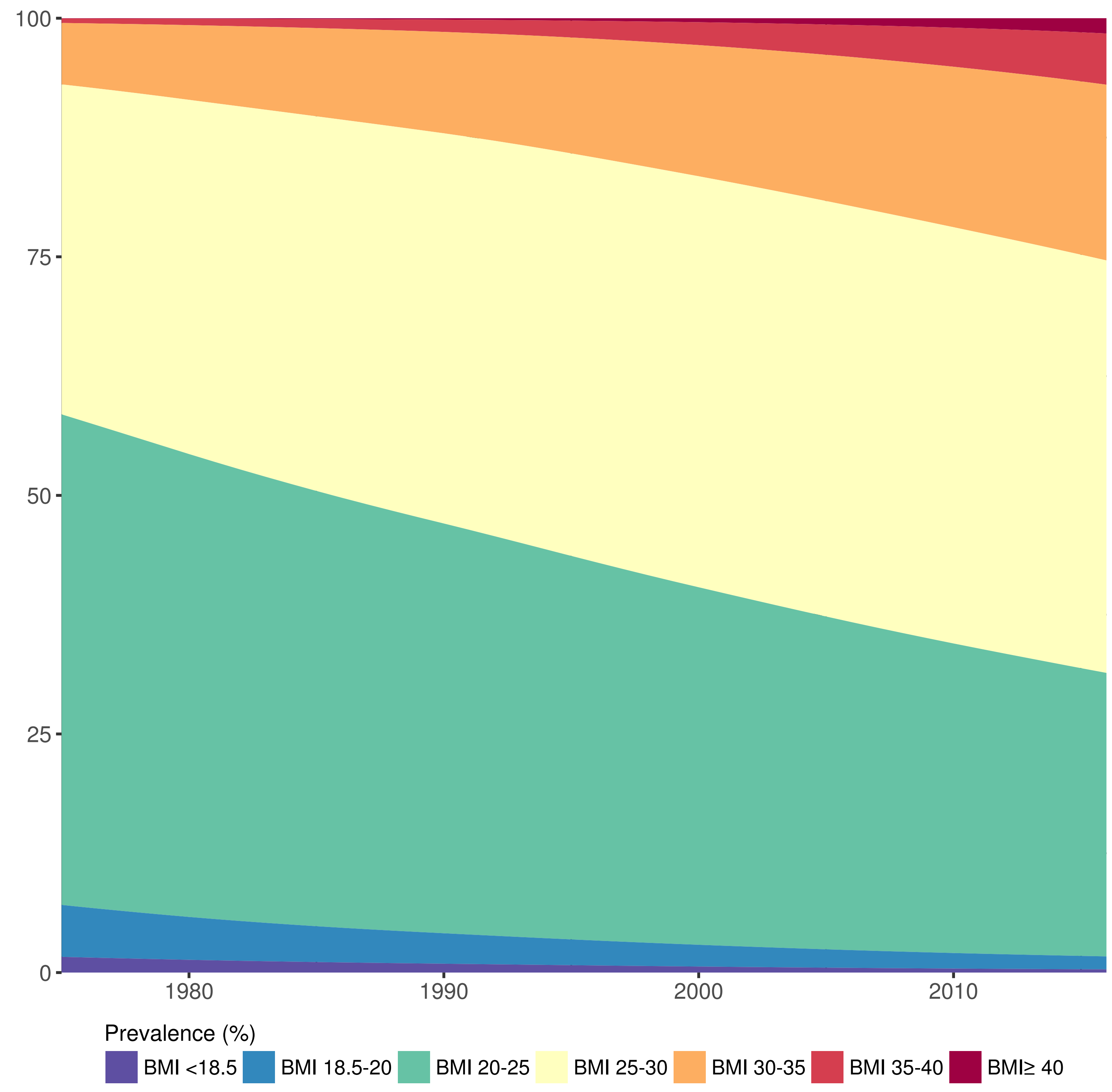
100
75
50
25
0
1980
1990
2000
2010
Prevalence (%)
BMI <18.5
BMI 18.5-20
BMI 20-25
BMI 25-30
BMI 30-35
BMI 35-40
BMI≥ 40

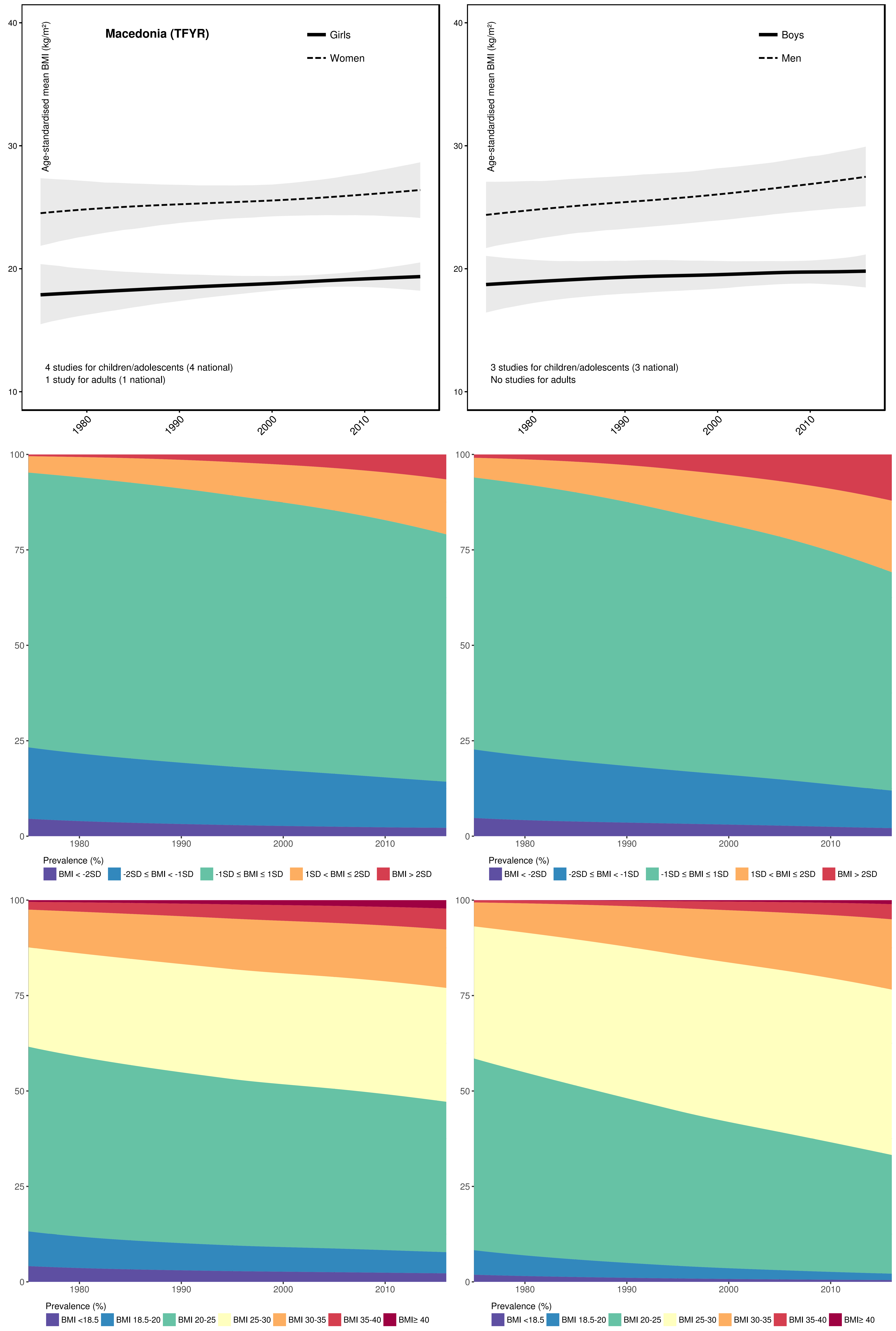

Macedonia (TFYR)
Girls
Women
Boys
Men
Age-standardised mean BMI (kg/m²)
4 studies for children/adolescents (4 national)
1 study for adults (1 national)
3 studies for children/adolescents (3 national)
No studies for adults
Prevalence (%)
BMI < -2SD
-2SD ≤ BMI < -1SD
-1SD ≤ BMI ≤ 1SD
1SD < BMI ≤ 2SD
BMI > 2SD
BMI <18.5
BMI 18.5-20
BMI 20-25
BMI 25-30
BMI 30-35
BMI 35-40
BMI≥ 40

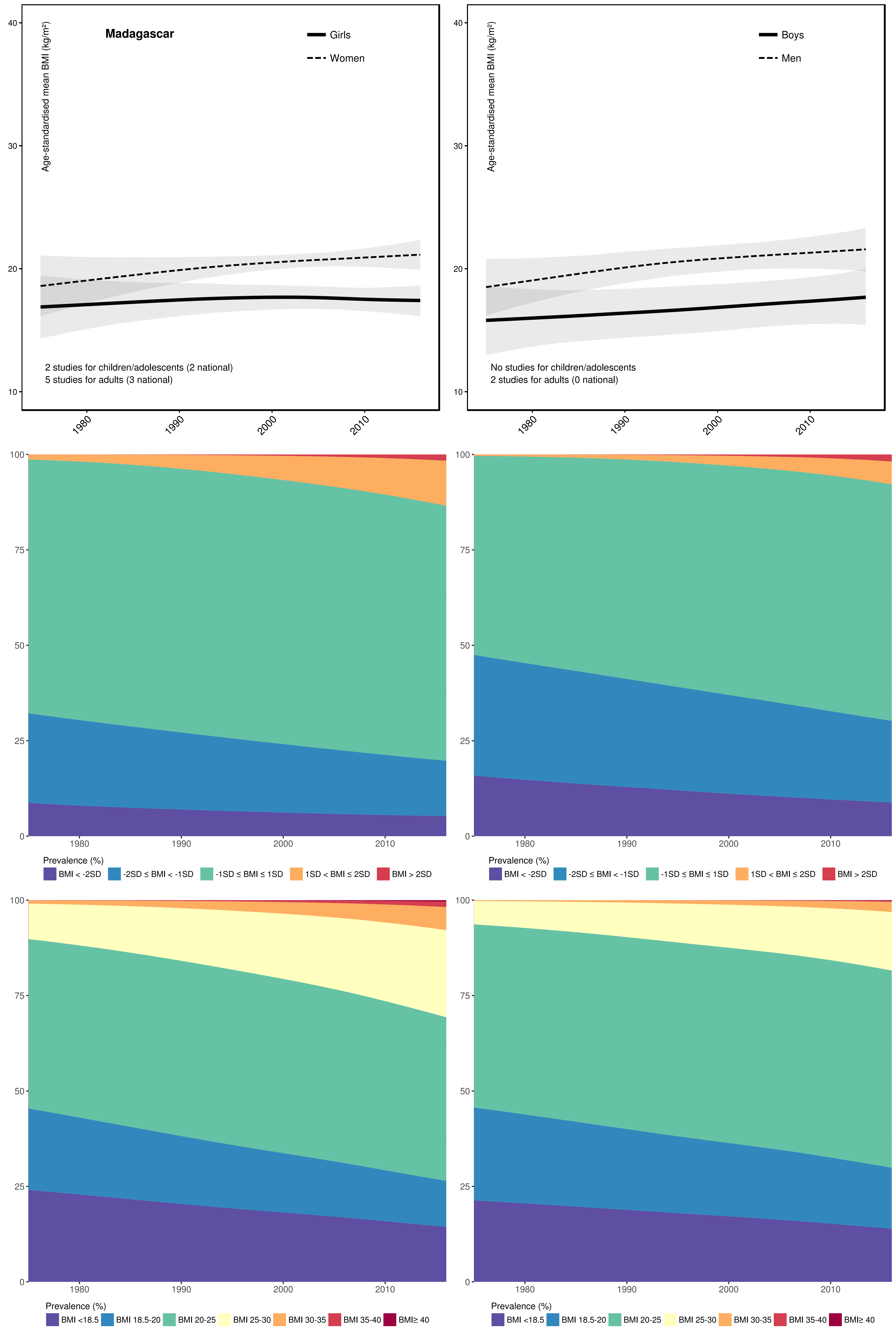
Madagascar
Girls
Women
Boys
Men
Age-standardised mean BMI (kg/m²)
40
30
20
10
2 studies for children/adolescents (2 national)
5 studies for adults (3 national)
No studies for children/adolescents
2 studies for adults (0 national)
1980
1990
2000
2010
100
75
50
25
0
Prevalence (%)
BMI < -2SD
-2SD ≤ BMI < -1SD
-1SD ≤ BMI ≤ 1SD
1SD < BMI ≤ 2SD
BMI > 2SD
BMI <18.5
BMI 18.5-20
BMI 20-25
BMI 25-30
BMI 30-35
BMI 35-40
BMI≥ 40

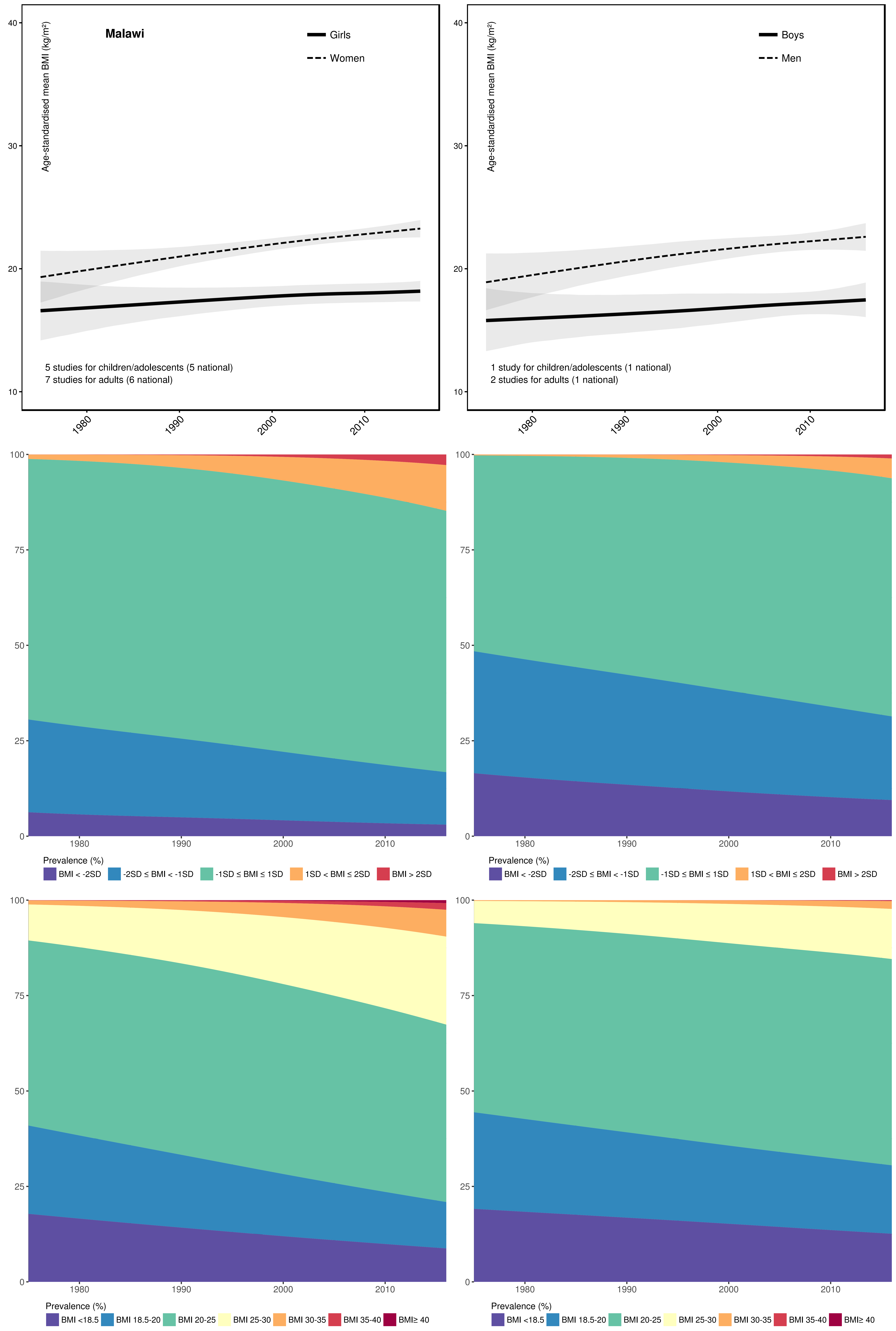
Malawi
Girls
Women
Boys
Men
Age-standardised mean BMI (kg/m²)
5 studies for children/adolescents (5 national)
7 studies for adults (6 national)
1 study for children/adolescents (1 national)
2 studies for adults (1 national)
Prevalence (%)
BMI < -2SD
-2SD ≤ BMI < -1SD
-1SD ≤ BMI ≤ 1SD
1SD < BMI ≤ 2SD
BMI > 2SD
BMI <18.5
BMI 18.5-20
BMI 20-25
BMI 25-30
BMI 30-35
BMI 35-40
BMI≥ 40

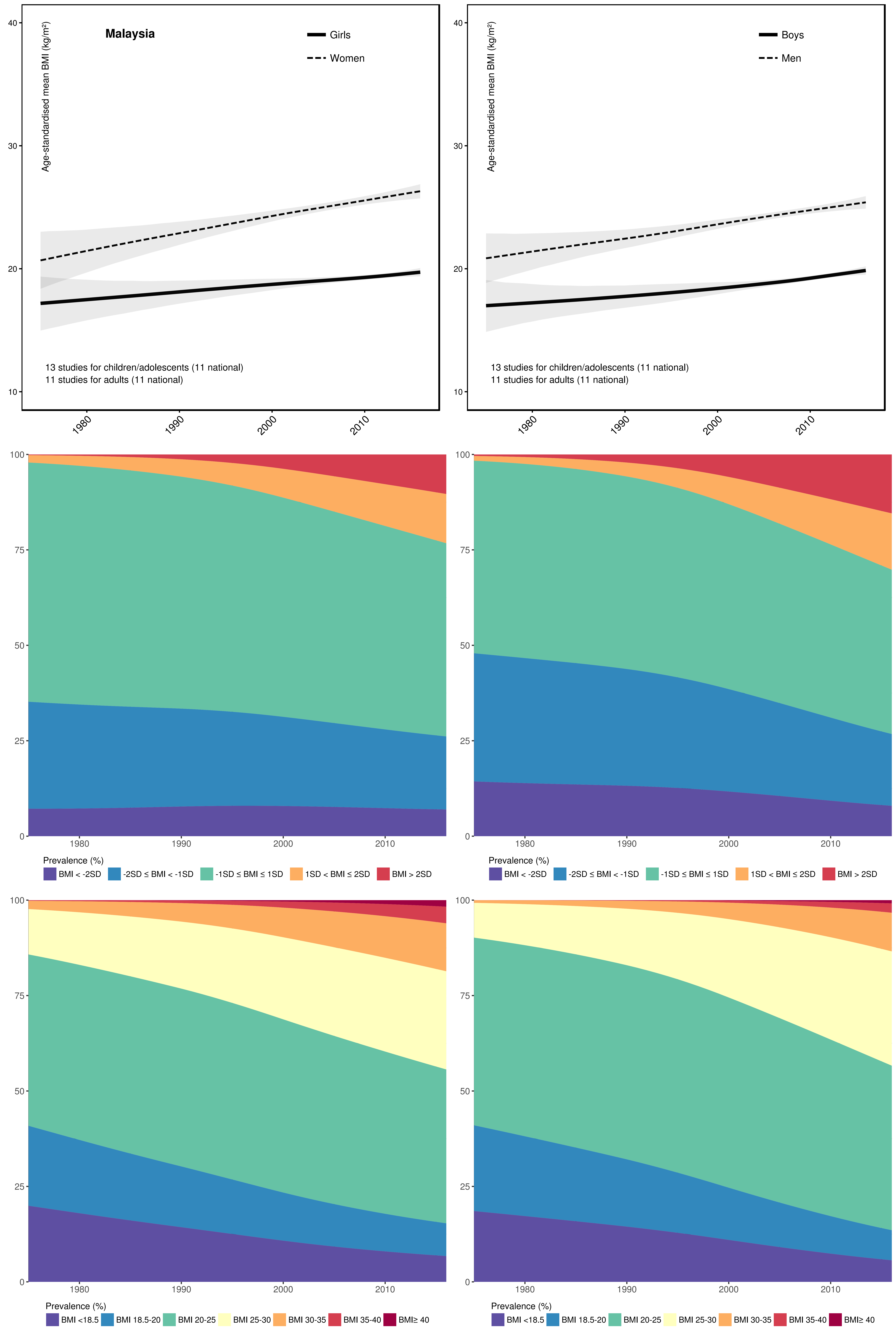
Malaysia
Girls
Women
Boys
Men
Age-standardised mean BMI (kg/m²)
13 studies for children/adolescents (11 national)
11 studies for adults (11 national)
1980
1990
2000
2010
Prevalence (%)
BMI < -2SD
-2SD ≤ BMI < -1SD
-1SD ≤ BMI ≤ 1SD
1SD < BMI ≤ 2SD
BMI > 2SD
BMI <18.5
BMI 18.5-20
BMI 20-25
BMI 25-30
BMI 30-35
BMI 35-40
BMI≥ 40

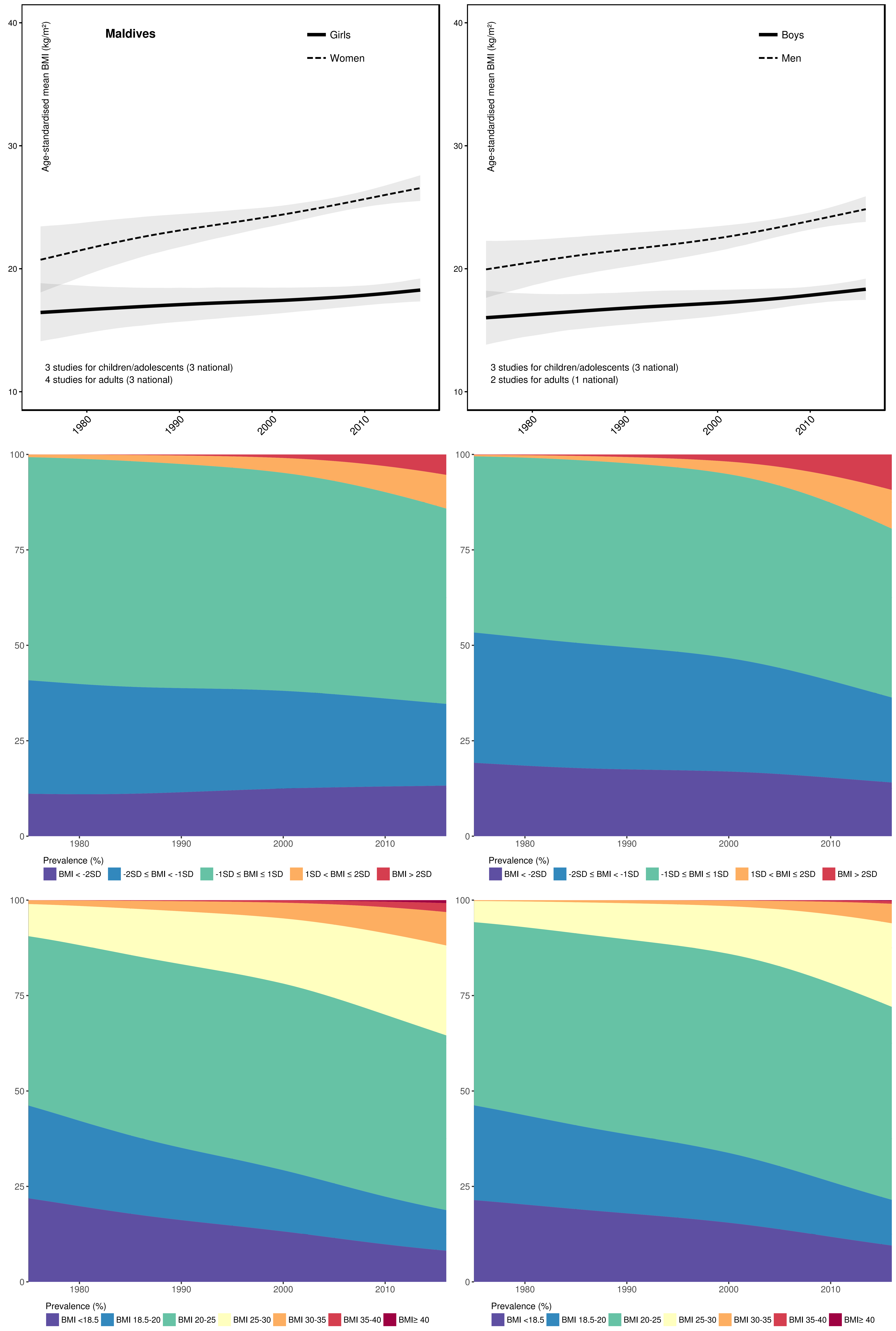
Maldives
Girls
Women
Boys
Men
Age-standardised mean BMI (kg/m²)
3 studies for children/adolescents (3 national)
4 studies for adults (3 national)
3 studies for children/adolescents (3 national)
2 studies for adults (1 national)
Prevalence (%)
BMI < -2SD
-2SD ≤ BMI < -1SD
-1SD ≤ BMI ≤ 1SD
1SD < BMI ≤ 2SD
BMI > 2SD
BMI <18.5
BMI 18.5-20
BMI 20-25
BMI 25-30
BMI 30-35
BMI 35-40
BMI≥ 40

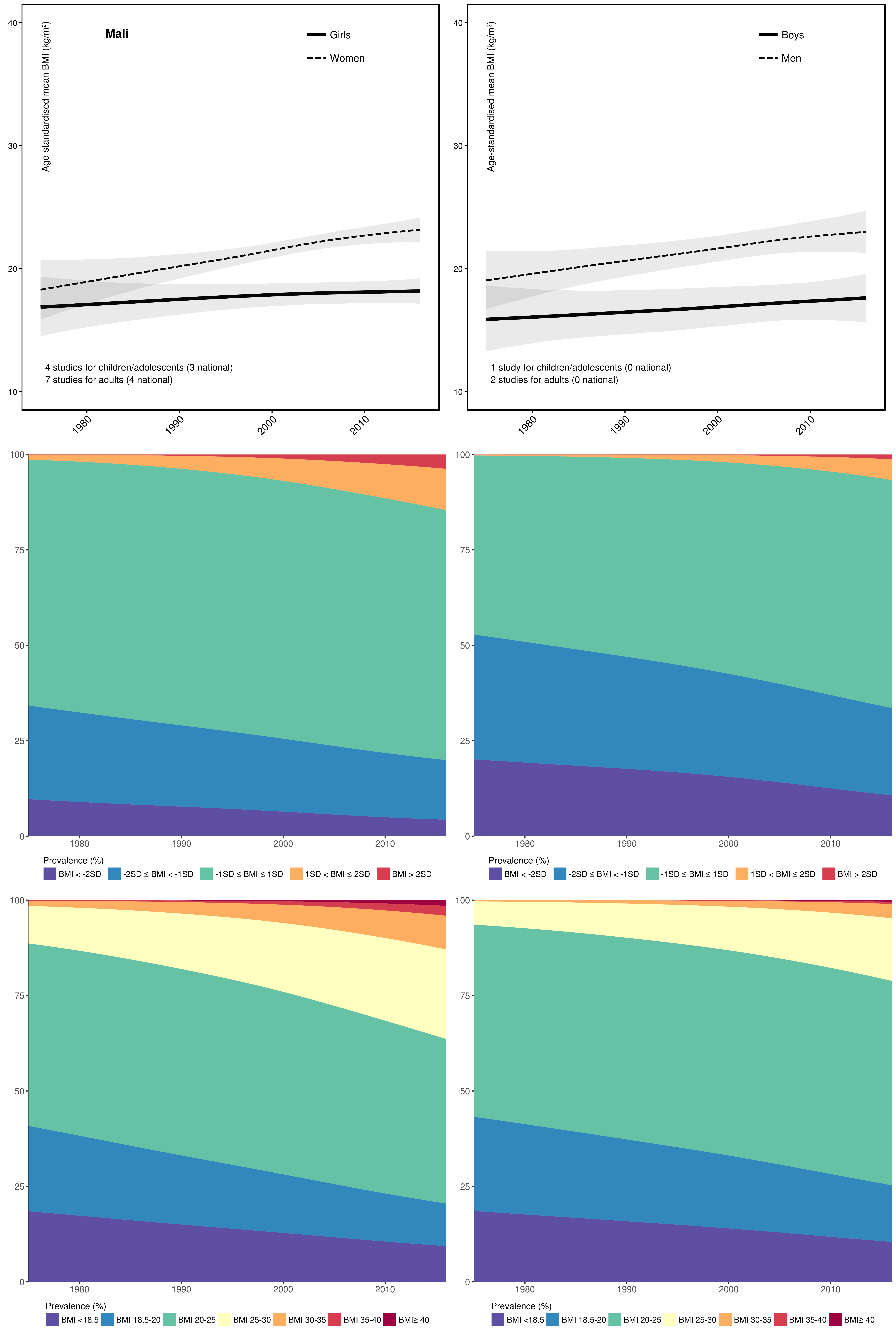
Mali
Girls
Women
Age-standardised mean BMI (kg/m²)
4 studies for children/adolescents (3 national)
7 studies for adults (4 national)
Boys
Men
1 study for children/adolescents (0 national)
2 studies for adults (0 national)
Prevalence (%)
BMI < -2SD
-2SD ≤ BMI < -1SD
-1SD ≤ BMI ≤ 1SD
1SD < BMI ≤ 2SD
BMI > 2SD
Prevalence (%)
BMI <18.5
BMI 18.5-20
BMI 20-25
BMI 25-30
BMI 30-35
BMI 35-40
BMI≥ 40

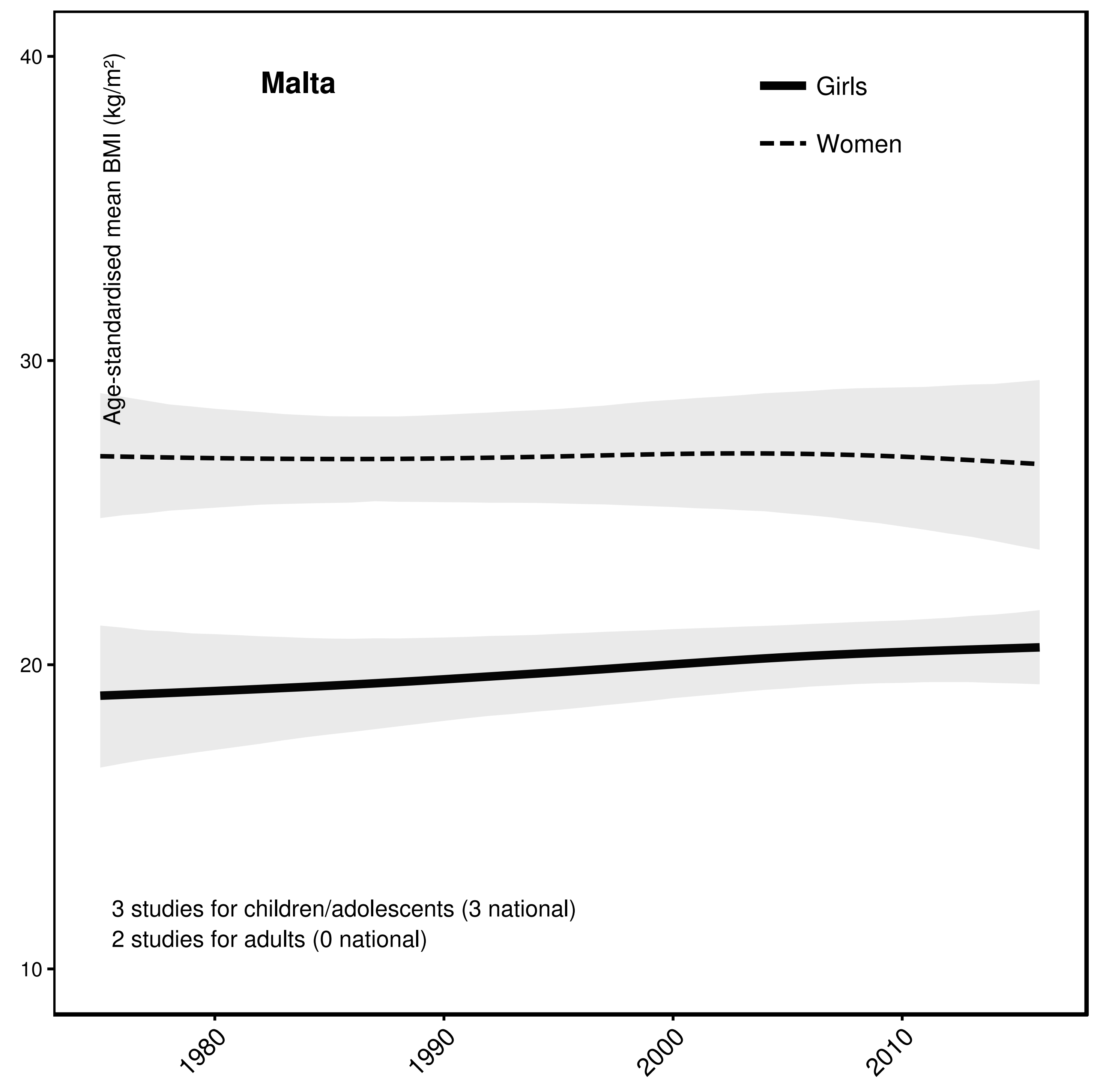
Malta
Girls
Women
Age-standardised mean BMI (kg/m²)
3 studies for children/adolescents (3 national)
2 studies for adults (0 national)
10
20
30
40
1980
1990
2000
2010

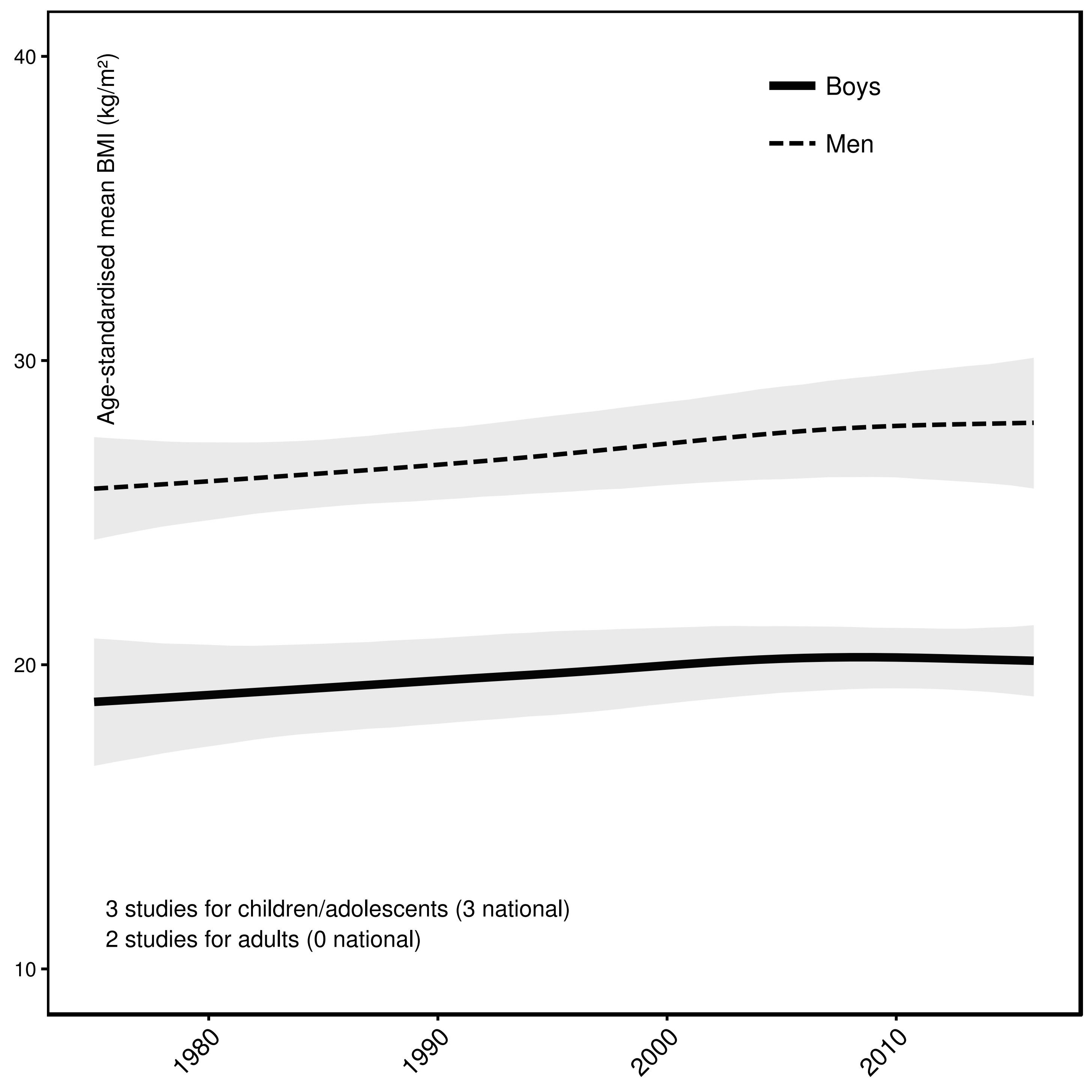
Boys
Men
Age-standardised mean BMI (kg/m²)
3 studies for children/adolescents (3 national)
2 studies for adults (0 national)
10
20
30
40
1980
1990
2000
2010

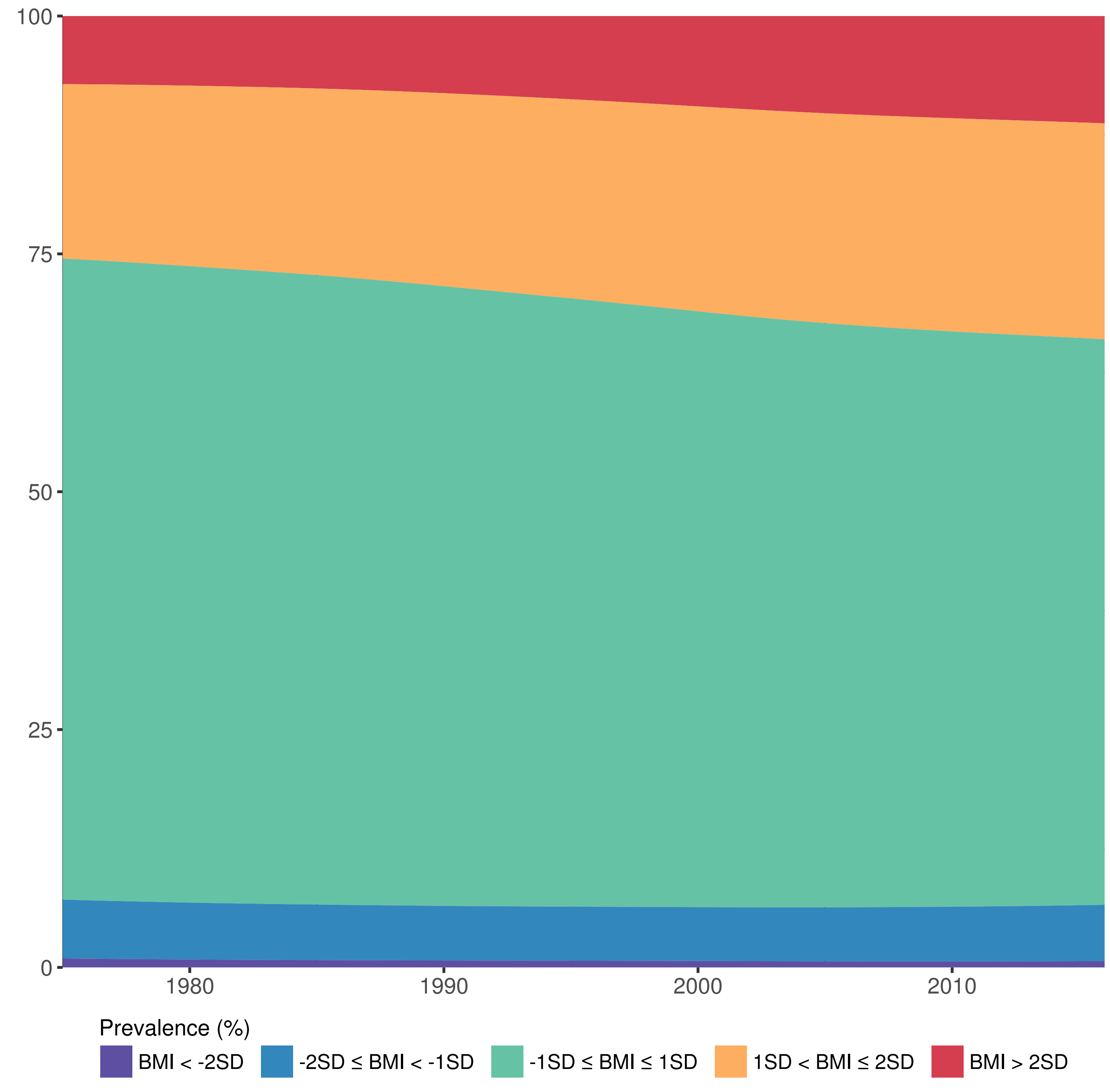
0
25
50
75
100
1980
1990
2000
2010
Prevalence (%)
BMI < -2SD
-2SD ≤ BMI < -1SD
-1SD ≤ BMI ≤ 1SD
1SD < BMI ≤ 2SD
BMI > 2SD

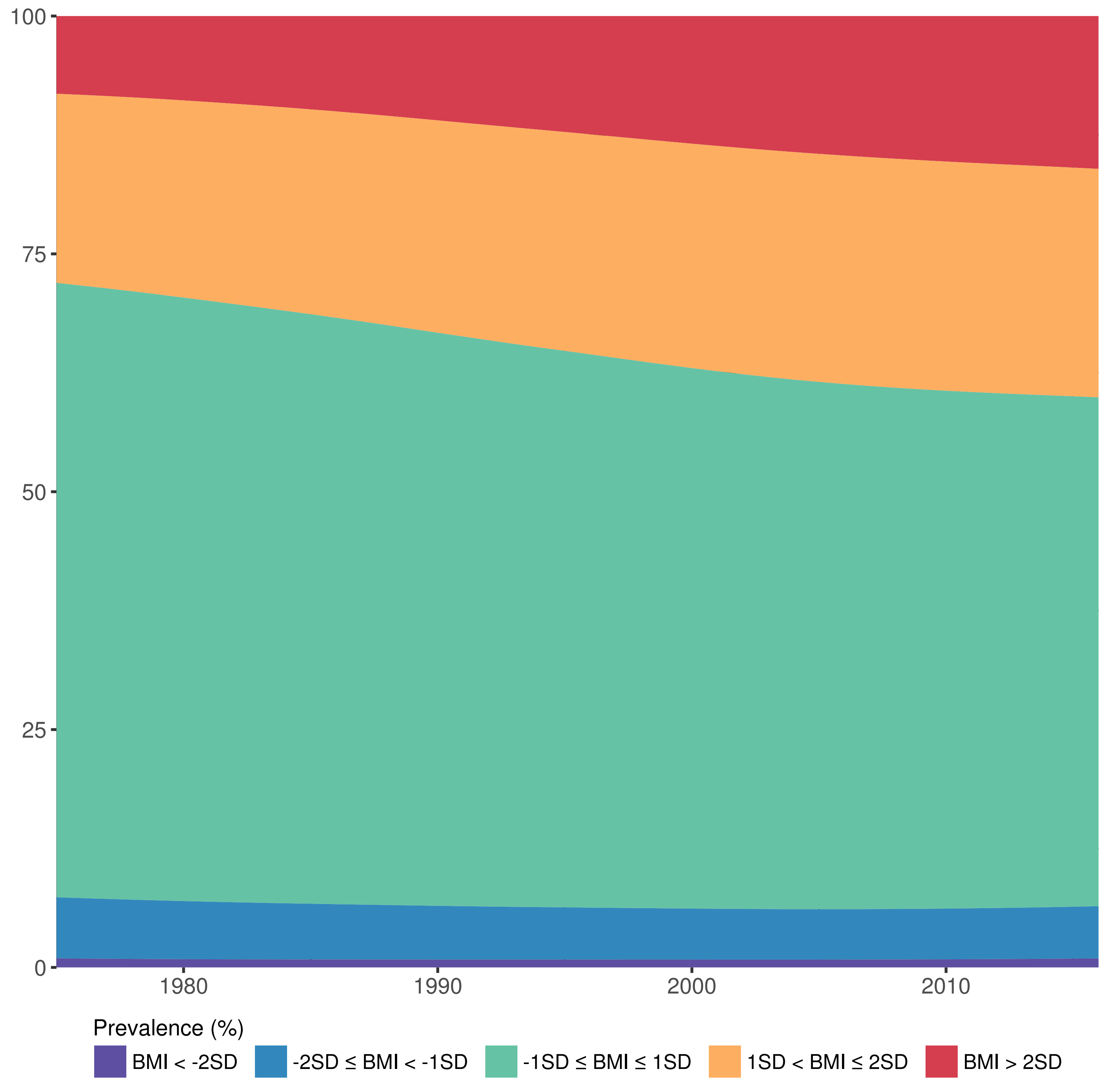
0
25
50
75
100
1980
1990
2000
2010
Prevalence (%)
BMI < -2SD
-2SD ≤ BMI < -1SD
-1SD ≤ BMI ≤ 1SD
1SD < BMI ≤ 2SD
BMI > 2SD

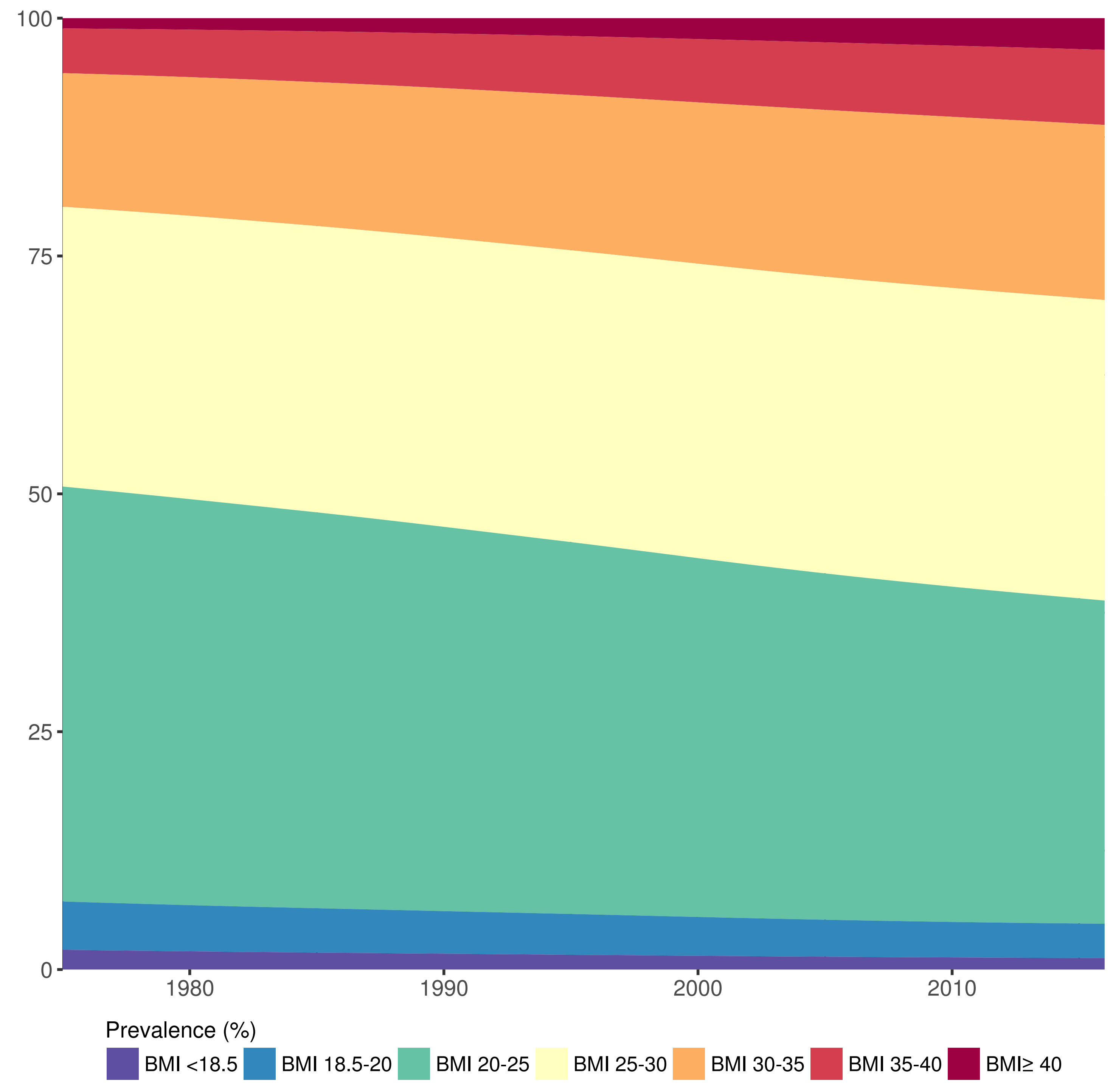
0
25
50
75
100
1980
1990
2000
2010
Prevalence (%)
BMI <18.5
BMI 18.5-20
BMI 20-25
BMI 25-30
BMI 30-35
BMI 35-40
BMI≥ 40

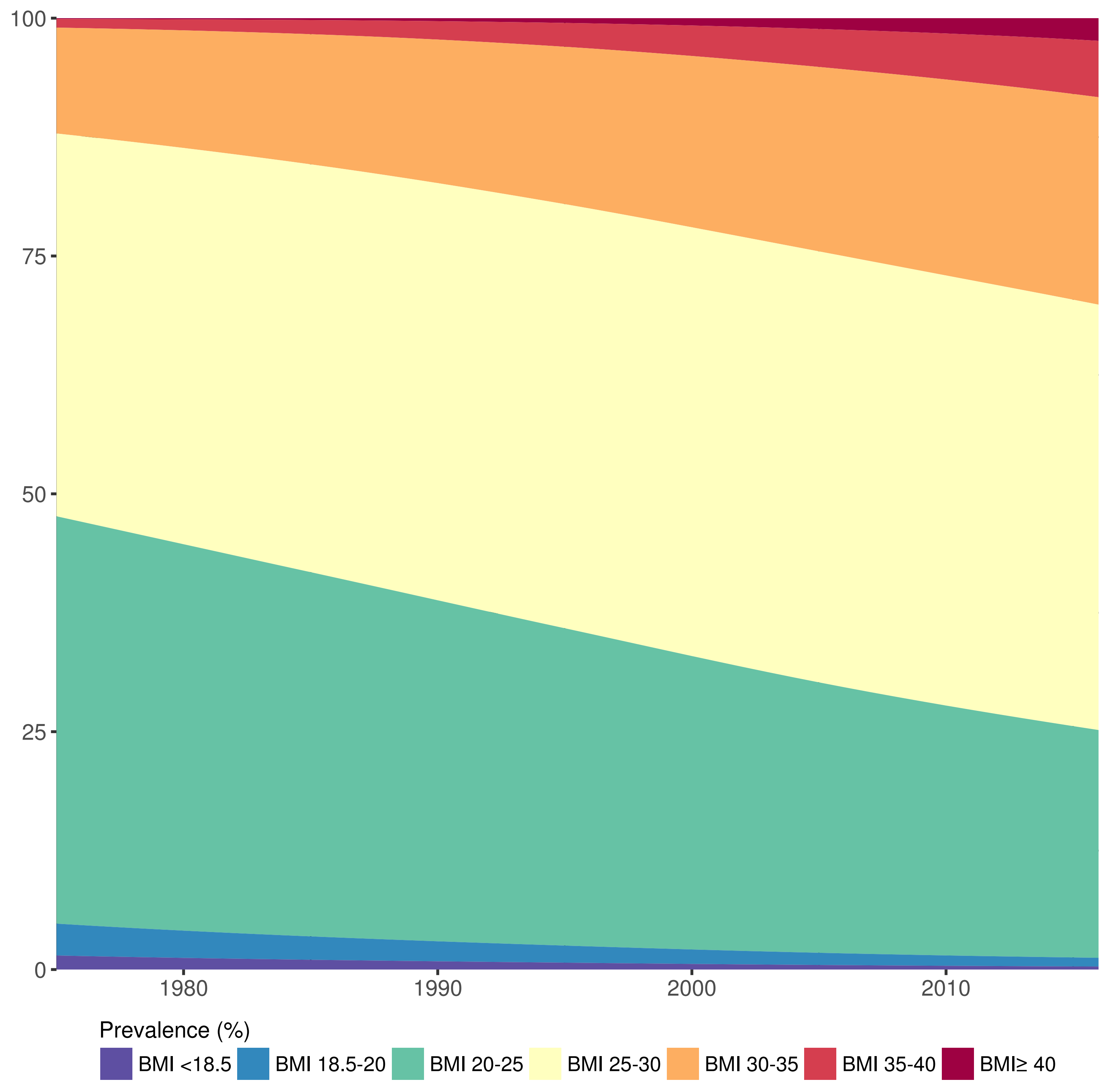
0
25
50
75
100
1980
1990
2000
2010
Prevalence (%)
BMI <18.5
BMI 18.5-20
BMI 20-25
BMI 25-30
BMI 30-35
BMI 35-40
BMI≥ 40

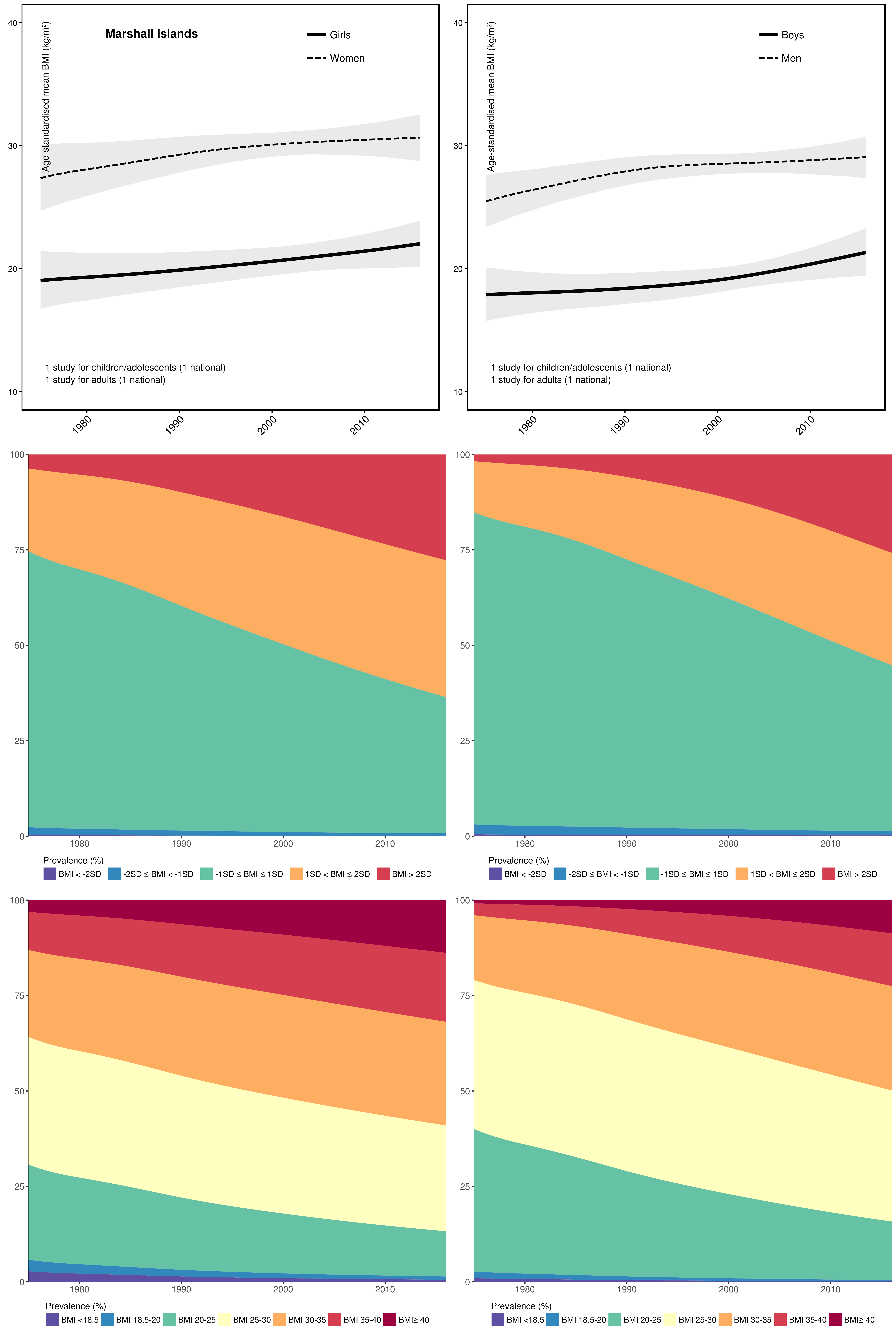
Marshall Islands
Girls
Women
Boys
Men
Age-standardised mean BMI (kg/m²)
1 study for children/adolescents (1 national)
1 study for adults (1 national)
1980
1990
2000
2010
Prevalence (%)
BMI < -2SD
-2SD ≤ BMI < -1SD
-1SD ≤ BMI ≤ 1SD
1SD < BMI ≤ 2SD
BMI > 2SD
BMI <18.5
BMI 18.5-20
BMI 20-25
BMI 25-30
BMI 30-35
BMI 35-40
BMI≥ 40

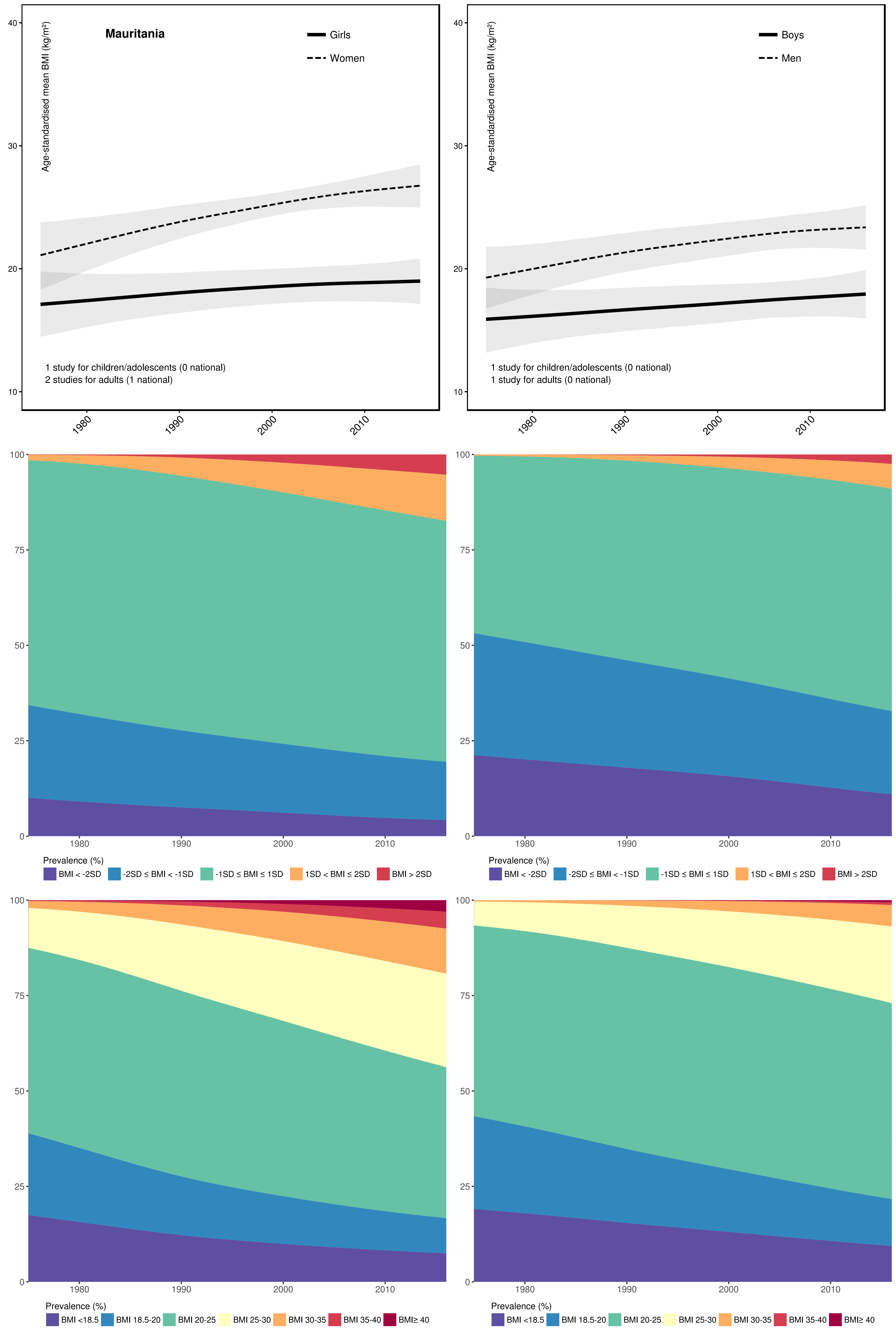
Mauritania
Girls
Women
Boys
Men
Age-standardised mean BMI (kg/m²)
1 study for children/adolescents (0 national)
2 studies for adults (1 national)
1 study for children/adolescents (0 national)
1 study for adults (0 national)
Prevalence (%)
BMI < -2SD
-2SD ≤ BMI < -1SD
-1SD ≤ BMI ≤ 1SD
1SD < BMI ≤ 2SD
BMI > 2SD
BMI <18.5
BMI 18.5-20
BMI 20-25
BMI 25-30
BMI 30-35
BMI 35-40
BMI≥ 40

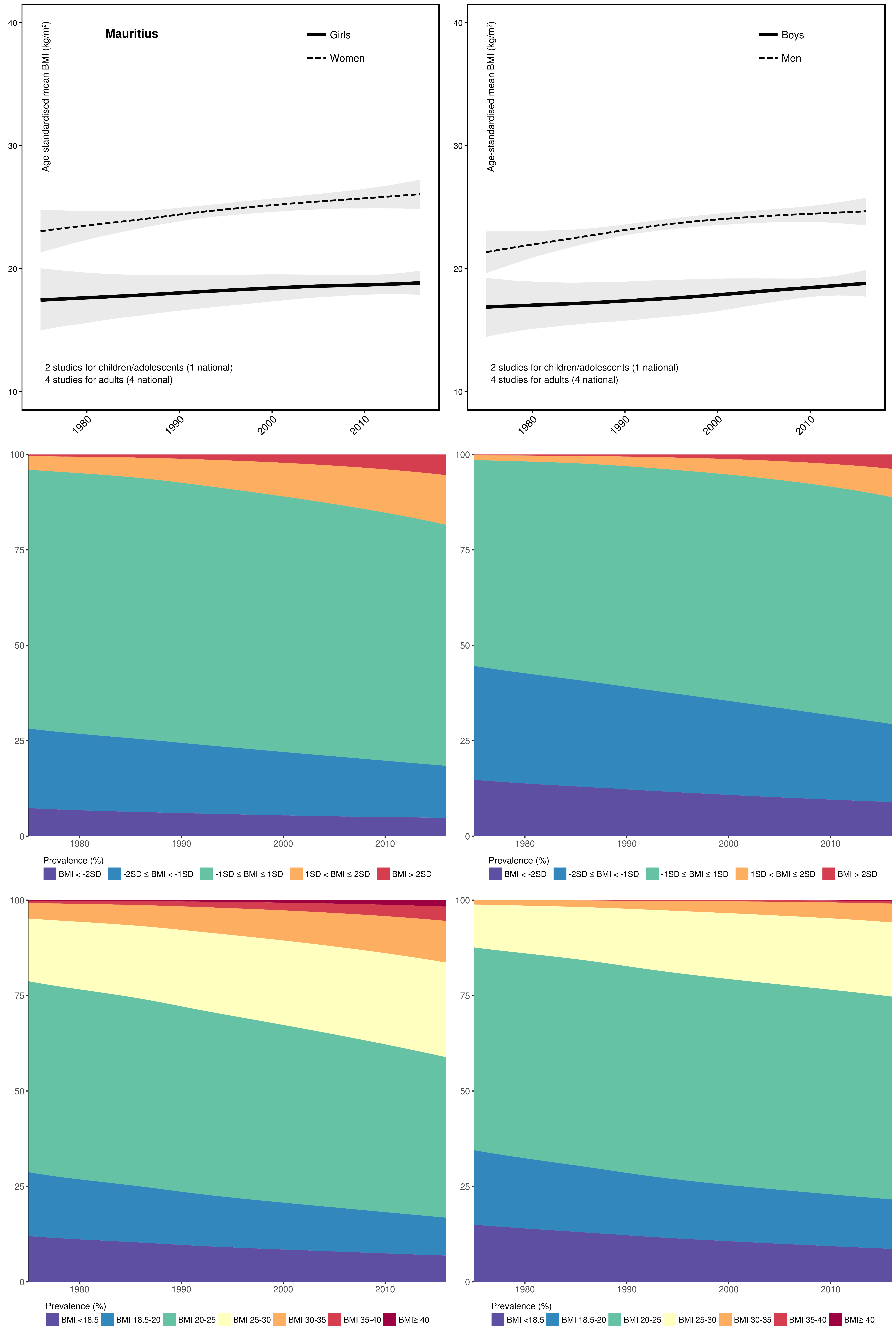
Mauritius
Girls
Women
Boys
Men
Age-standardised mean BMI (kg/m²)
2 studies for children/adolescents (1 national)
4 studies for adults (4 national)
Prevalence (%)
BMI < -2SD
-2SD ≤ BMI < -1SD
-1SD ≤ BMI ≤ 1SD
1SD < BMI ≤ 2SD
BMI > 2SD
BMI <18.5
BMI 18.5-20
BMI 20-25
BMI 25-30
BMI 30-35
BMI 35-40
BMI≥ 40

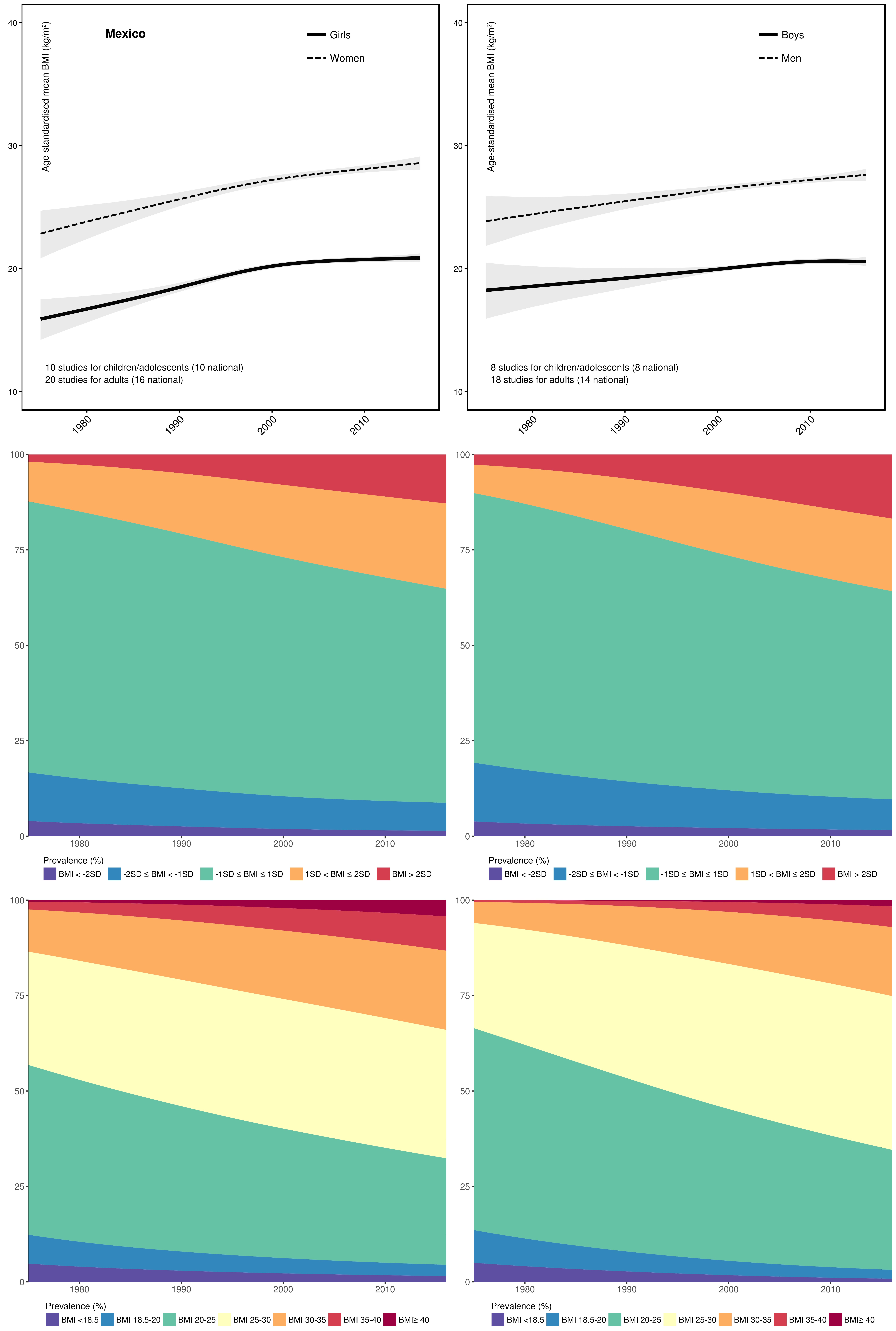
Mexico
Girls
Women
Boys
Men
Age-standardised mean BMI (kg/m²)
10 studies for children/adolescents (10 national)
20 studies for adults (16 national)
8 studies for children/adolescents (8 national)
18 studies for adults (14 national)
Prevalence (%)
BMI < -2SD
-2SD ≤ BMI < -1SD
-1SD ≤ BMI ≤ 1SD
1SD < BMI ≤ 2SD
BMI > 2SD
BMI <18.5
BMI 18.5-20
BMI 20-25
BMI 25-30
BMI 30-35
BMI 35-40
BMI≥ 40

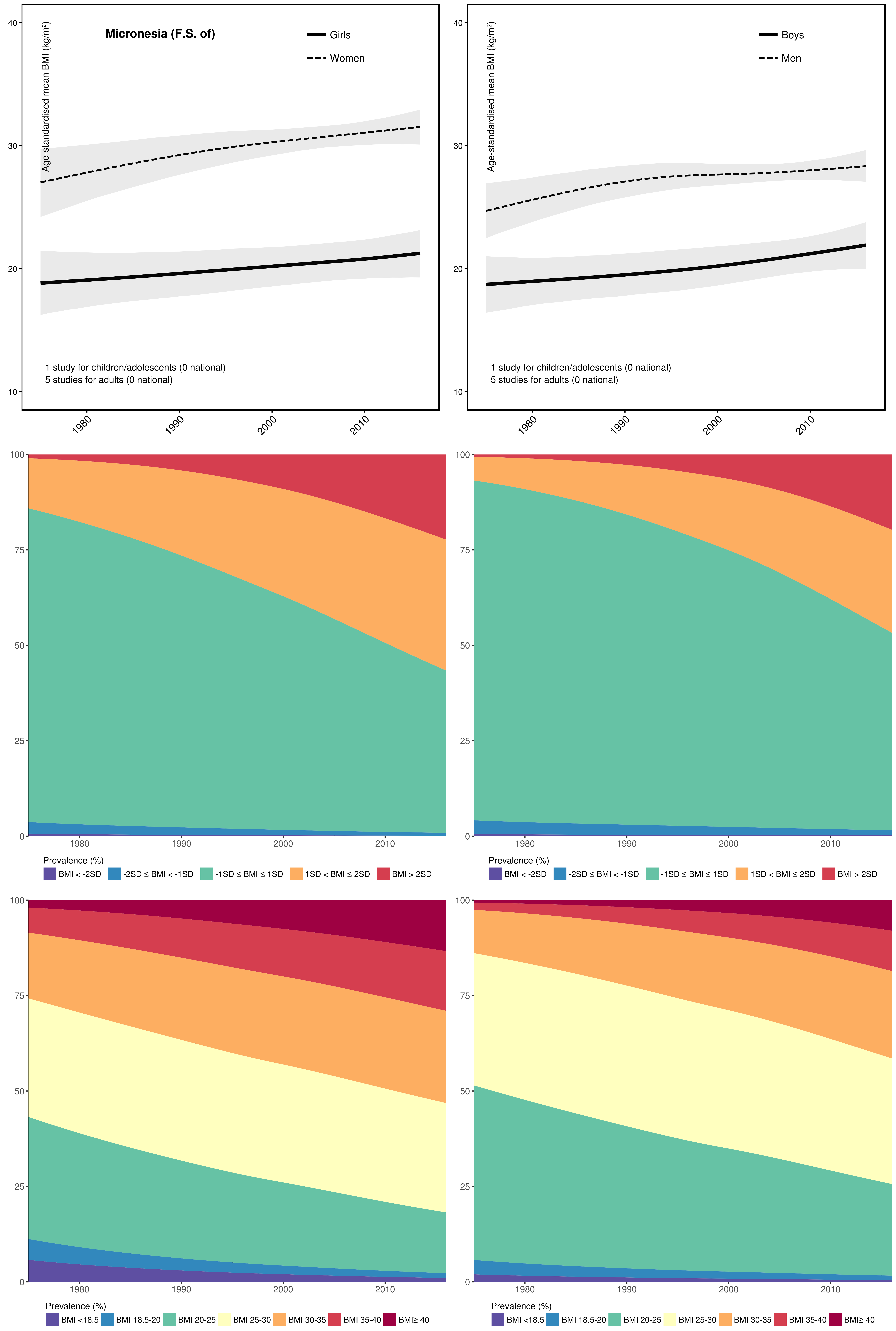
Micronesia (F.S. of)
Girls
Women
Boys
Men
Age-standardised mean BMI (kg/m²)
1 study for children/adolescents (0 national)
5 studies for adults (0 national)
Prevalence (%)
BMI < -2SD
-2SD ≤ BMI < -1SD
-1SD ≤ BMI ≤ 1SD
1SD < BMI ≤ 2SD
BMI > 2SD
BMI <18.5
BMI 18.5-20
BMI 20-25
BMI 25-30
BMI 30-35
BMI 35-40
BMI≥ 40

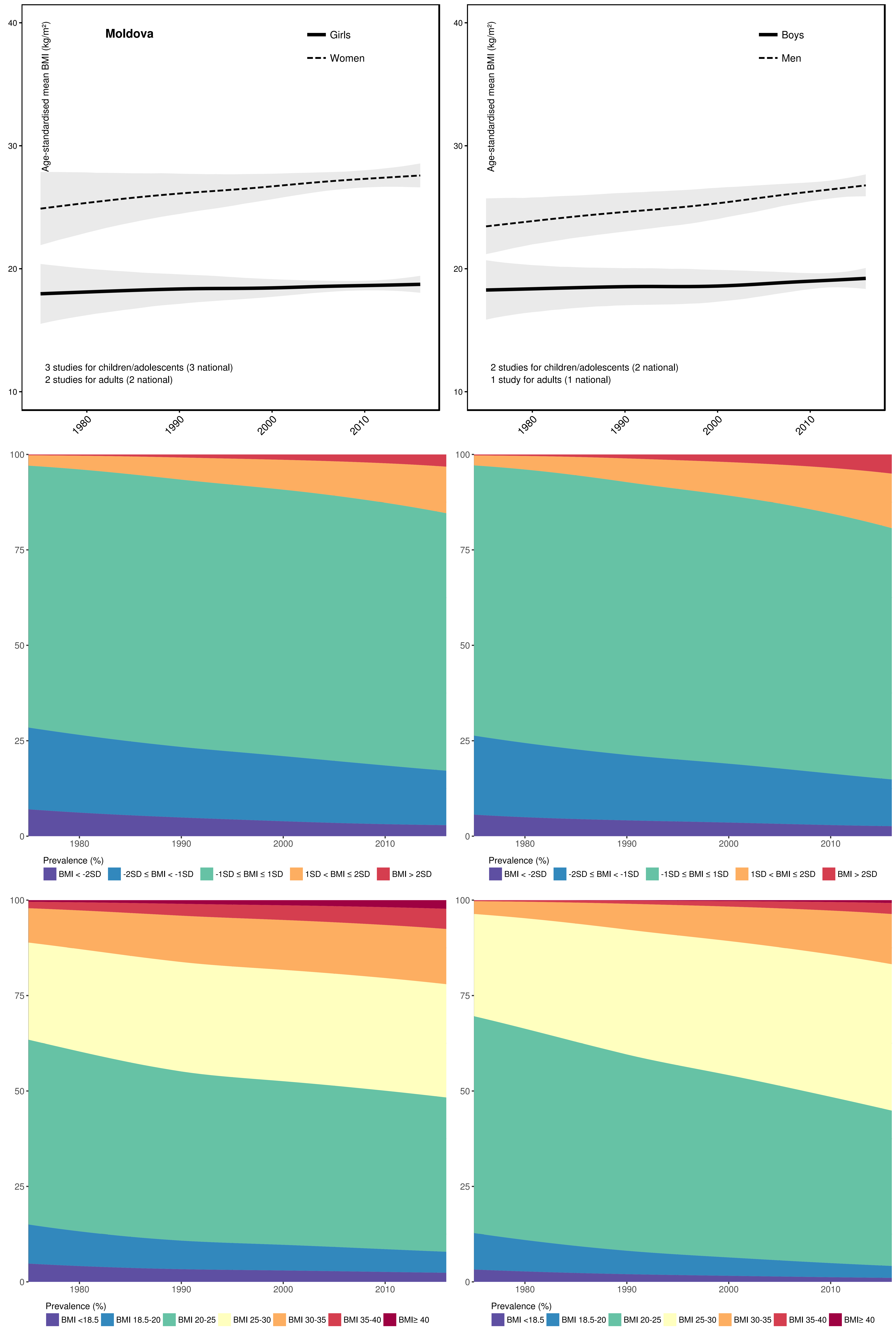
Moldova
Girls
Women
Boys
Men
Age-standardised mean BMI (kg/m²)
40
30
20
10
1980
1990
2000
2010
3 studies for children/adolescents (3 national)
2 studies for adults (2 national)
2 studies for children/adolescents (2 national)
1 study for adults (1 national)
100
75
50
25
0
Prevalence (%)
BMI < -2SD
-2SD ≤ BMI < -1SD
-1SD ≤ BMI ≤ 1SD
1SD < BMI ≤ 2SD
BMI > 2SD
BMI <18.5
BMI 18.5-20
BMI 20-25
BMI 25-30
BMI 30-35
BMI 35-40
BMI≥ 40

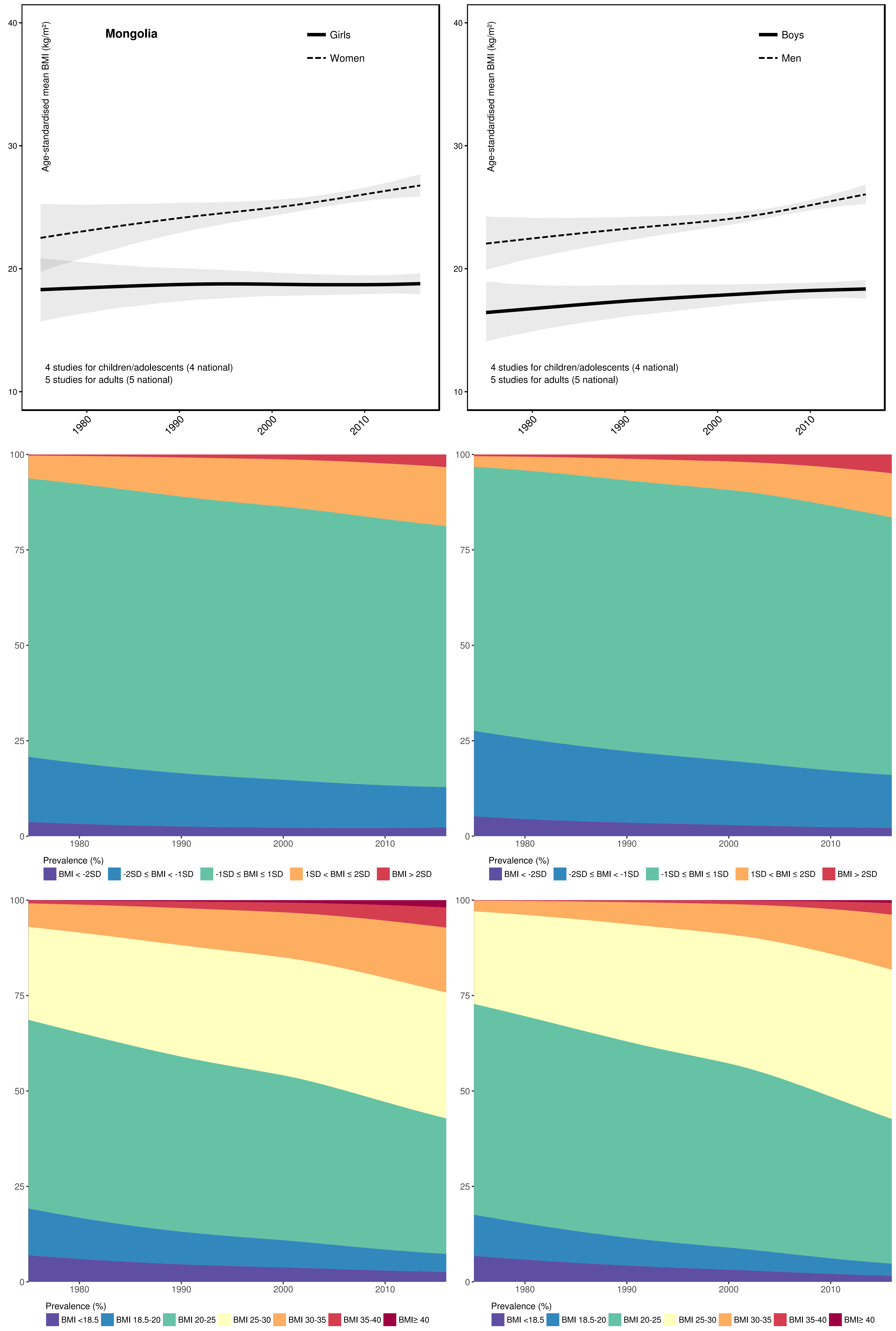
Mongolia
Girls
Women
Boys
Men
Age-standardised mean BMI (kg/m²)
4 studies for children/adolescents (4 national)
5 studies for adults (5 national)
Prevalence (%)
BMI < -2SD
-2SD ≤ BMI < -1SD
-1SD ≤ BMI ≤ 1SD
1SD < BMI ≤ 2SD
BMI > 2SD
BMI <18.5
BMI 18.5-20
BMI 20-25
BMI 25-30
BMI 30-35
BMI 35-40
BMI≥ 40

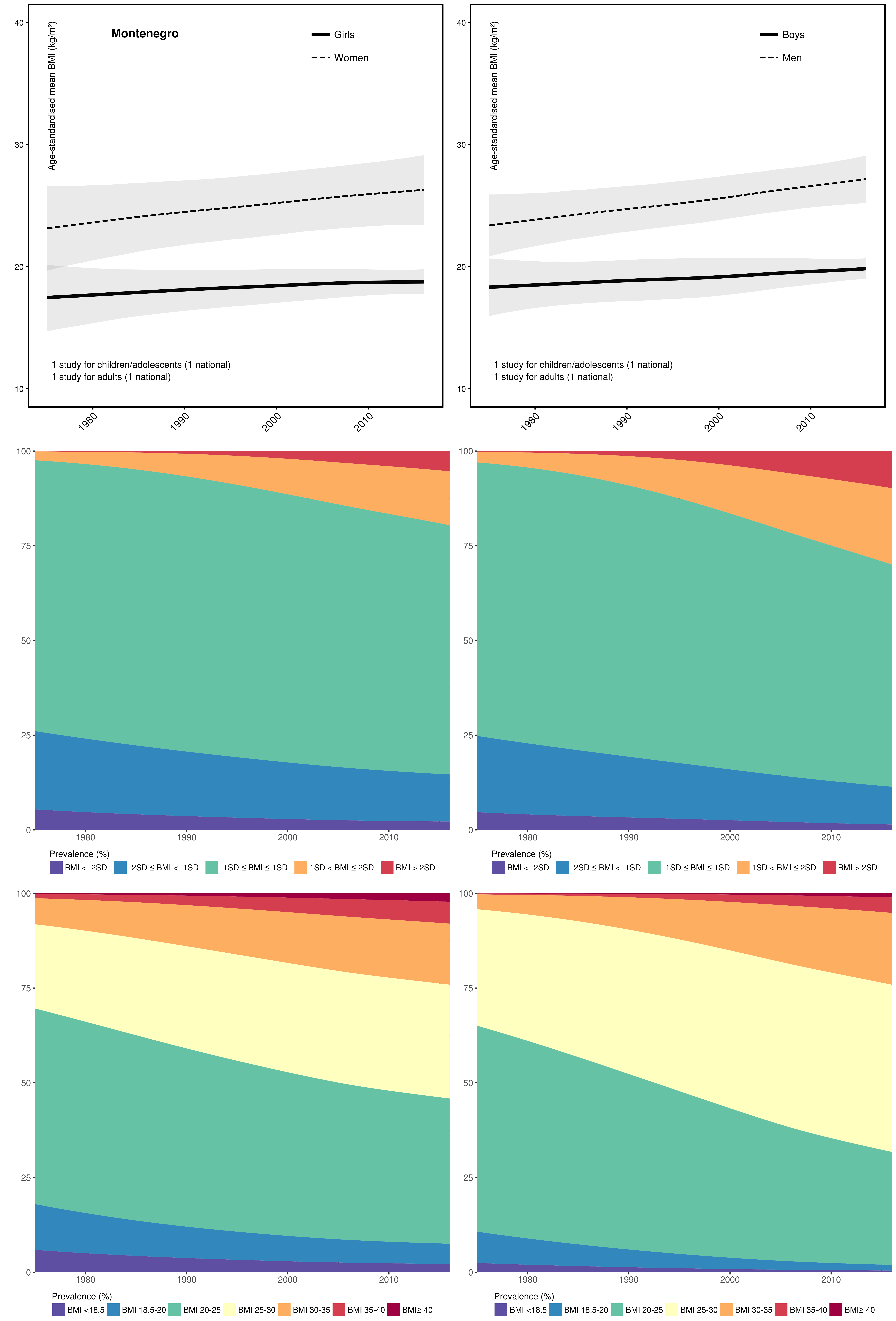
Montenegro
Girls
Women
Boys
Men
Age-standardised mean BMI (kg/m²)
1 study for children/adolescents (1 national)
1 study for adults (1 national)
1980
1990
2000
2010
Prevalence (%)
BMI < -2SD
-2SD ≤ BMI < -1SD
-1SD ≤ BMI ≤ 1SD
1SD < BMI ≤ 2SD
BMI > 2SD
BMI <18.5
BMI 18.5-20
BMI 20-25
BMI 25-30
BMI 30-35
BMI 35-40
BMI≥ 40

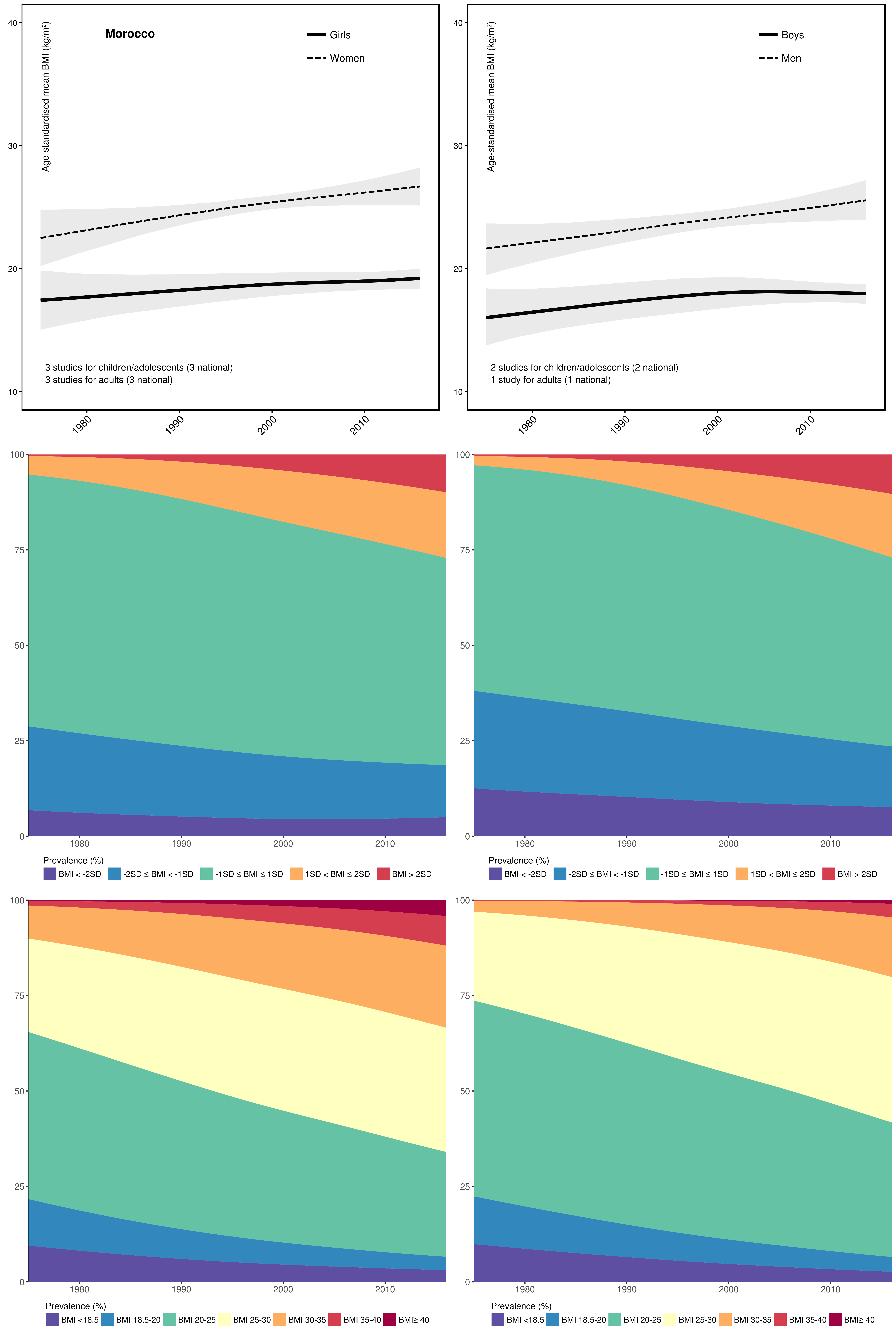

Morocco
Girls
Women
Boys
Men
Age-standardised mean BMI (kg/m²)
3 studies for children/adolescents (3 national)
3 studies for adults (3 national)
2 studies for children/adolescents (2 national)
1 study for adults (1 national)
Prevalence (%)
BMI < -2SD
-2SD ≤ BMI < -1SD
-1SD ≤ BMI ≤ 1SD
1SD < BMI ≤ 2SD
BMI > 2SD
BMI <18.5
BMI 18.5-20
BMI 20-25
BMI 25-30
BMI 30-35
BMI 35-40
BMI≥ 40

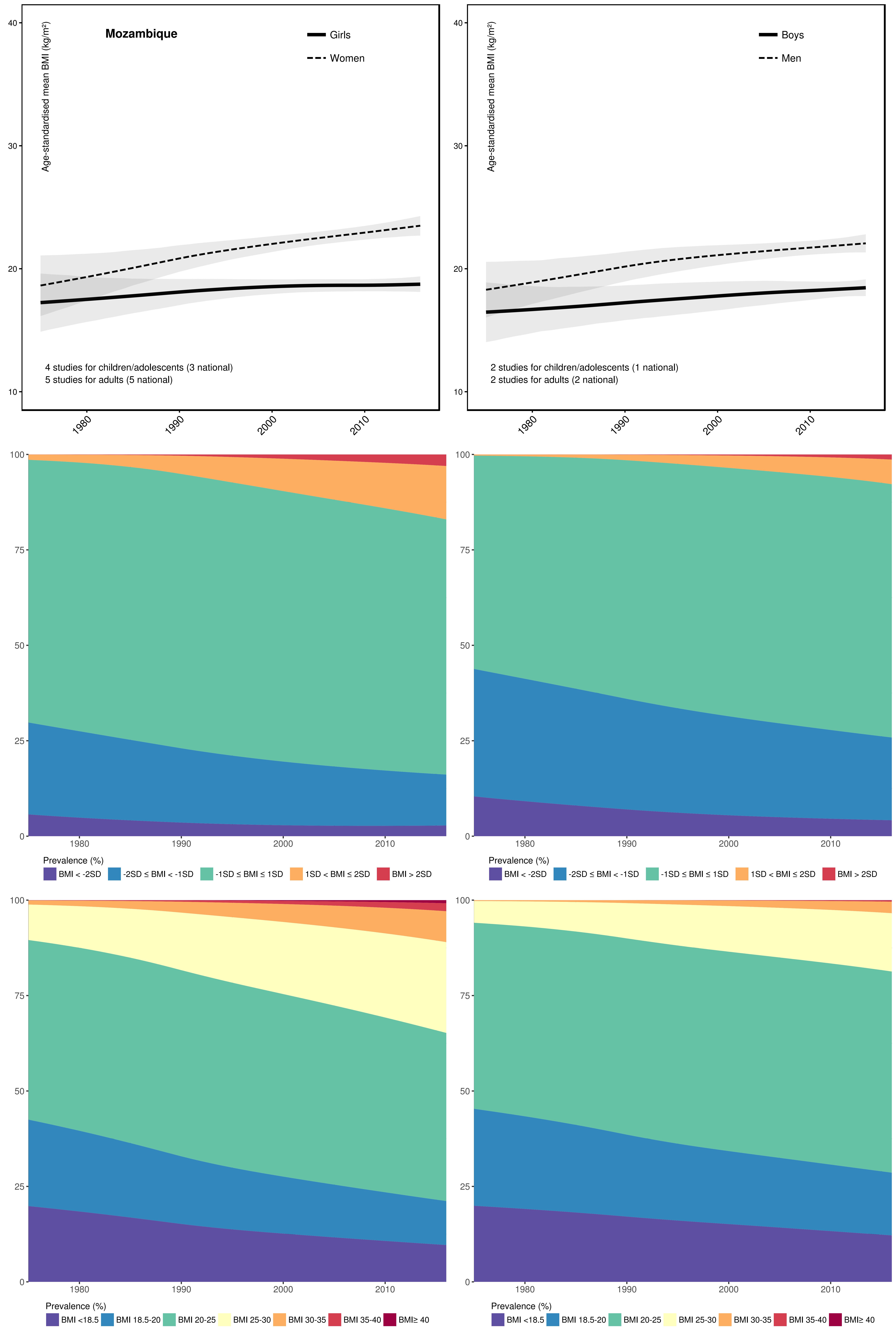
Mozambique
Girls
Women
Boys
Men
Age-standardised mean BMI (kg/m²)
4 studies for children/adolescents (3 national)
5 studies for adults (5 national)
2 studies for children/adolescents (1 national)
2 studies for adults (2 national)
Prevalence (%)
BMI < -2SD
-2SD ≤ BMI < -1SD
-1SD ≤ BMI ≤ 1SD
1SD < BMI ≤ 2SD
BMI > 2SD
BMI <18.5
BMI 18.5-20
BMI 20-25
BMI 25-30
BMI 30-35
BMI 35-40
BMI≥ 40

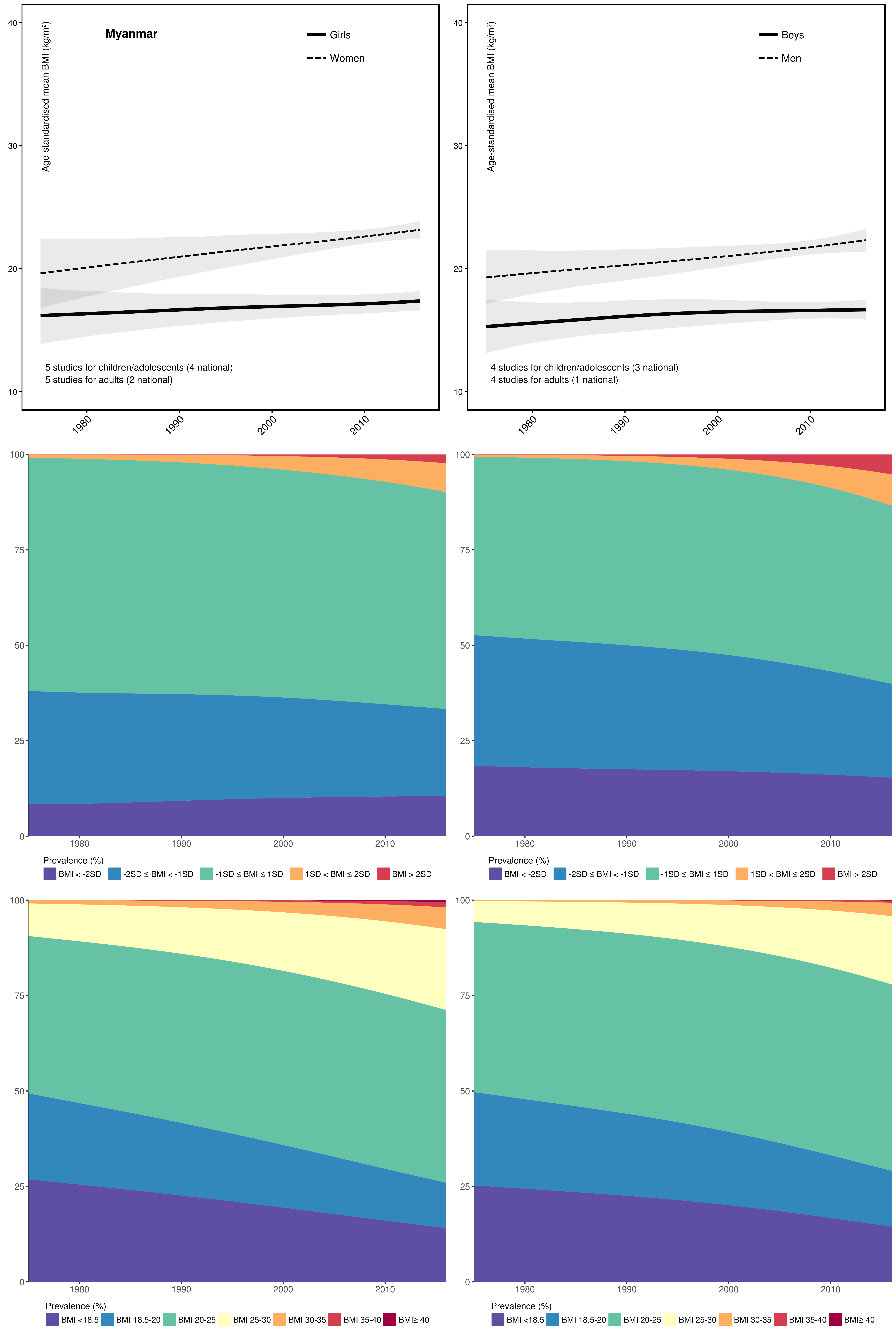
Myanmar
Girls
Women
Boys
Men
Age-standardised mean BMI (kg/m²)
5 studies for children/adolescents (4 national)
5 studies for adults (2 national)
4 studies for children/adolescents (3 national)
4 studies for adults (1 national)
Prevalence (%)
BMI < -2SD
-2SD ≤ BMI < -1SD
-1SD ≤ BMI ≤ 1SD
1SD < BMI ≤ 2SD
BMI > 2SD
BMI <18.5
BMI 18.5-20
BMI 20-25
BMI 25-30
BMI 30-35
BMI 35-40
BMI≥ 40

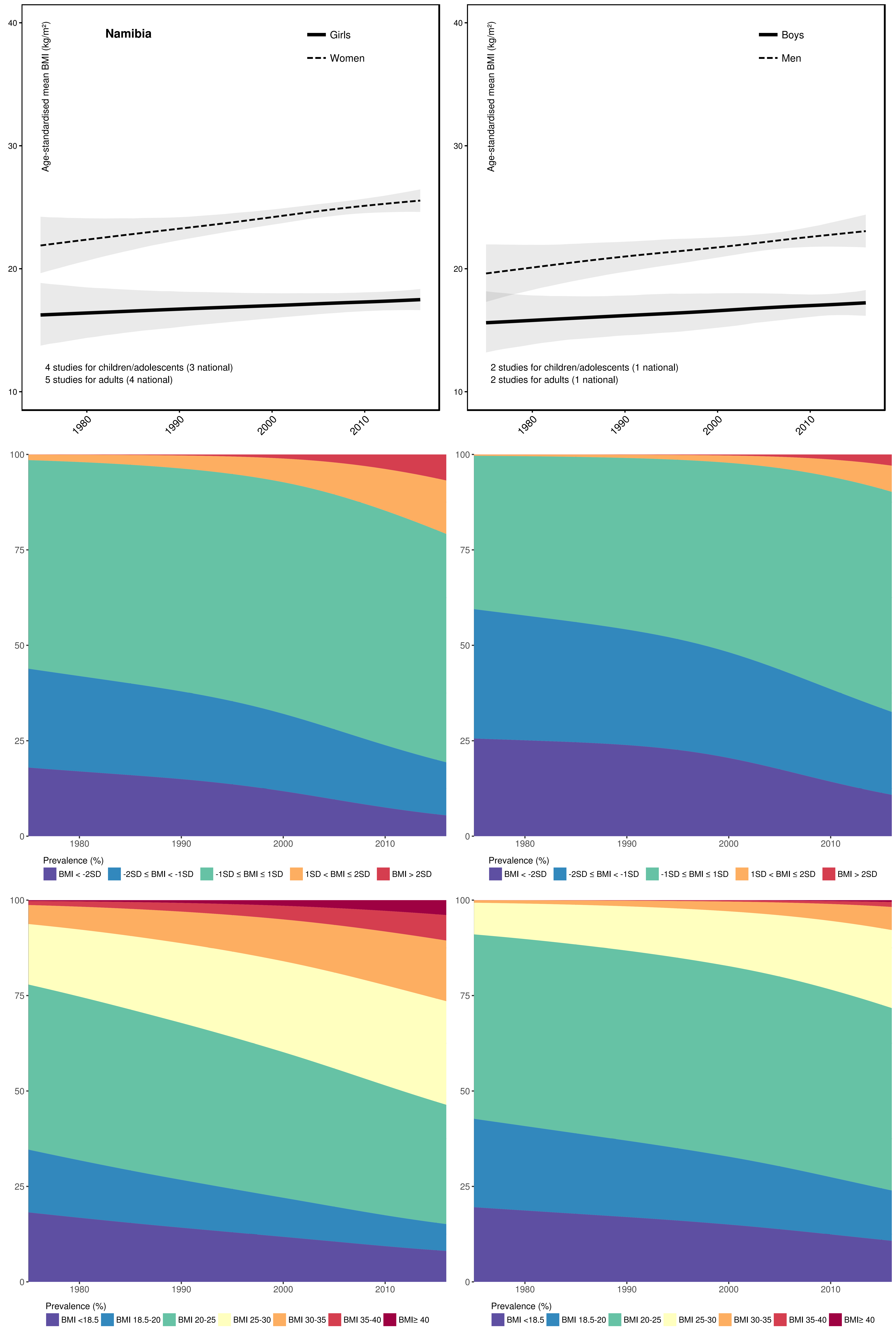
Namibia
Girls
Women
Boys
Men
Age-standardised mean BMI (kg/m²)
4 studies for children/adolescents (3 national)
5 studies for adults (4 national)
2 studies for children/adolescents (1 national)
2 studies for adults (1 national)
Prevalence (%)
BMI < -2SD
-2SD ≤ BMI < -1SD
-1SD ≤ BMI ≤ 1SD
1SD < BMI ≤ 2SD
BMI > 2SD
BMI <18.5
BMI 18.5-20
BMI 20-25
BMI 25-30
BMI 30-35
BMI 35-40
BMI≥ 40

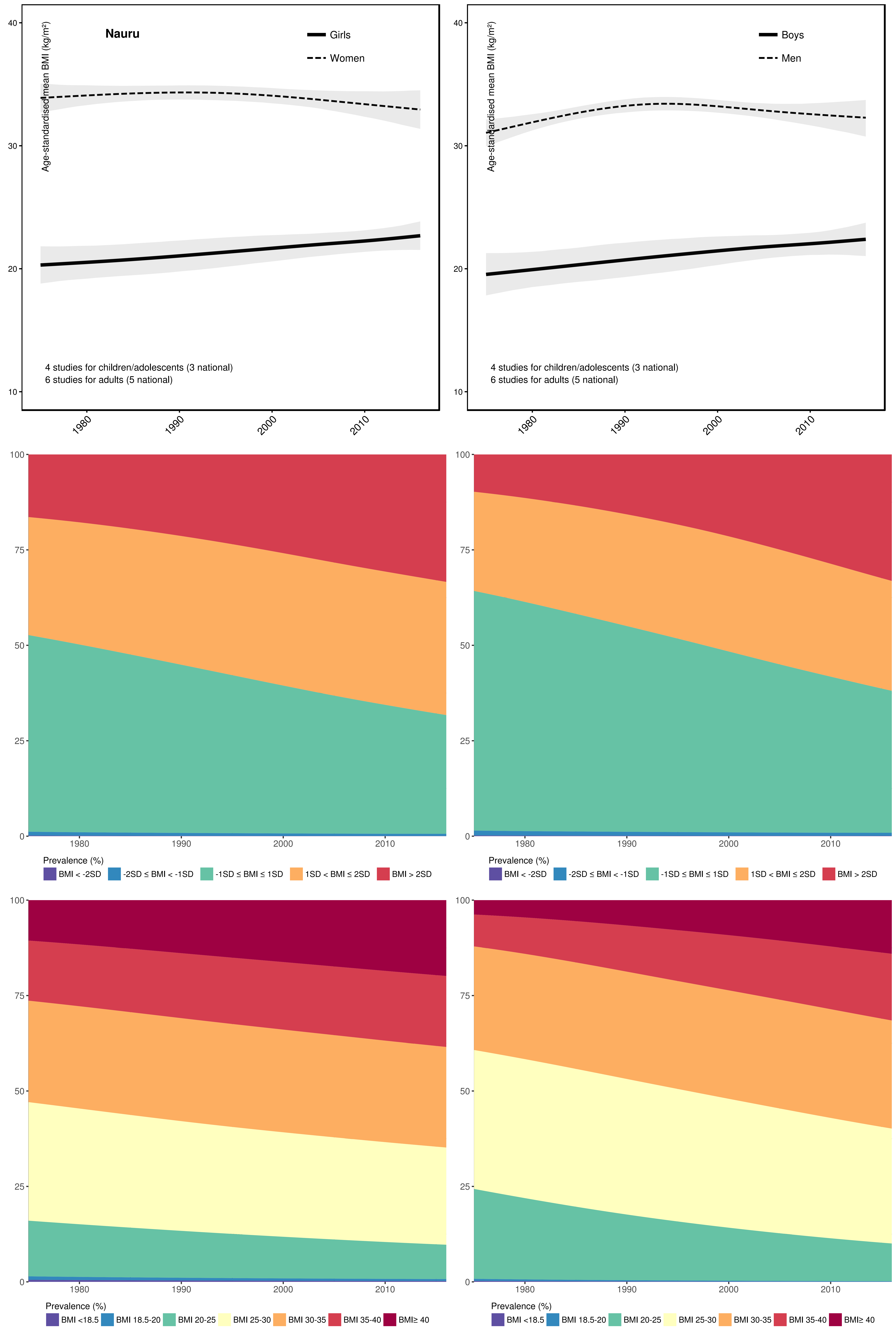
Nauru
Girls
Women
Boys
Men
Age-standardised mean BMI (kg/m²)
4 studies for children/adolescents (3 national)
6 studies for adults (5 national)
Prevalence (%)
BMI < -2SD
-2SD ≤ BMI < -1SD
-1SD ≤ BMI ≤ 1SD
1SD < BMI ≤ 2SD
BMI > 2SD
BMI <18.5
BMI 18.5-20
BMI 20-25
BMI 25-30
BMI 30-35
BMI 35-40
BMI≥ 40

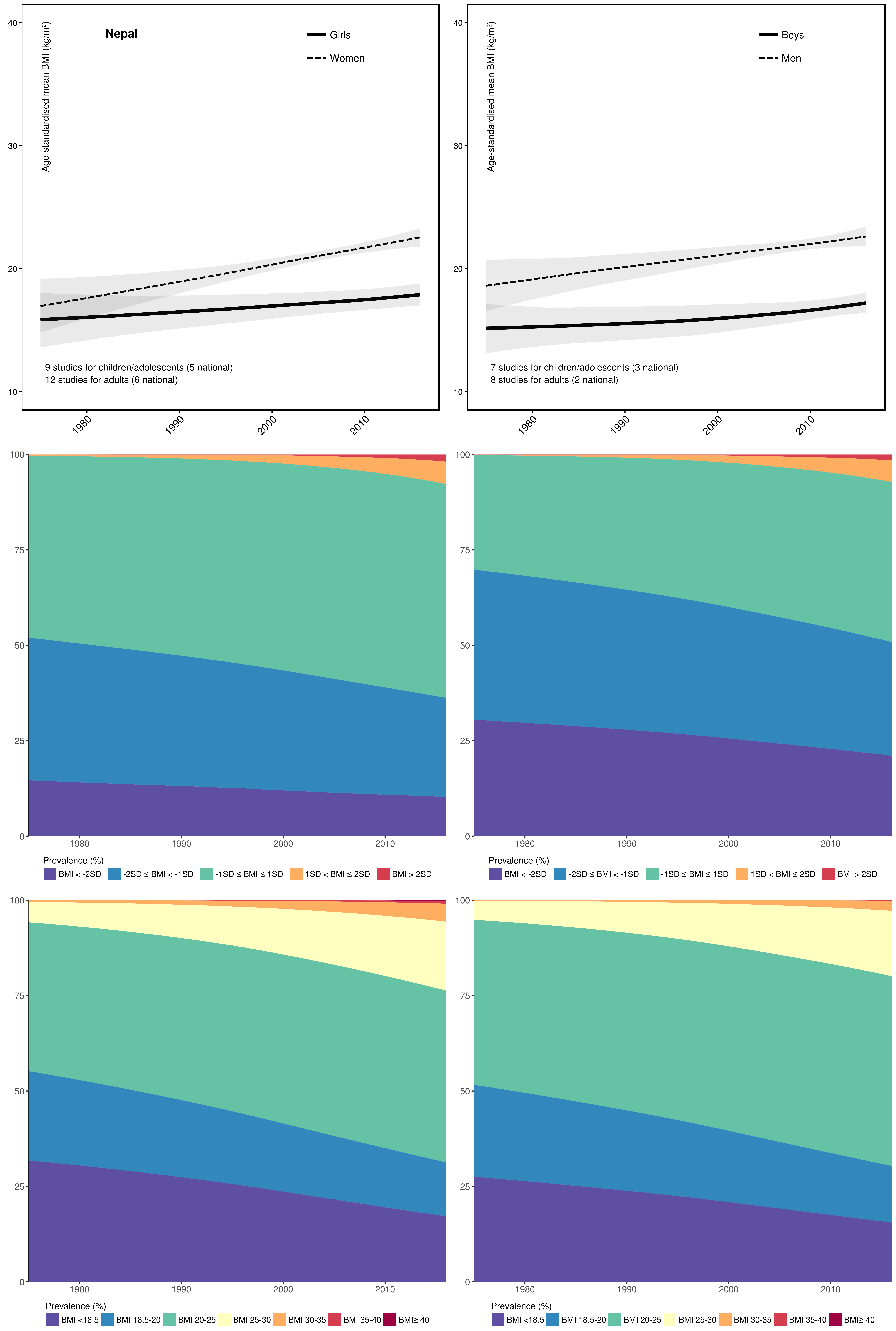

Nepal
Girls
Women
Boys
Men
Age-standardised mean BMI (kg/m²)
9 studies for children/adolescents (5 national)
12 studies for adults (6 national)
7 studies for children/adolescents (3 national)
8 studies for adults (2 national)
Prevalence (%)
BMI < -2SD
-2SD ≤ BMI < -1SD
-1SD ≤ BMI ≤ 1SD
1SD < BMI ≤ 2SD
BMI > 2SD
BMI <18.5
BMI 18.5-20
BMI 20-25
BMI 25-30
BMI 30-35
BMI 35-40
BMI≥ 40
1980
1990
2000
2010

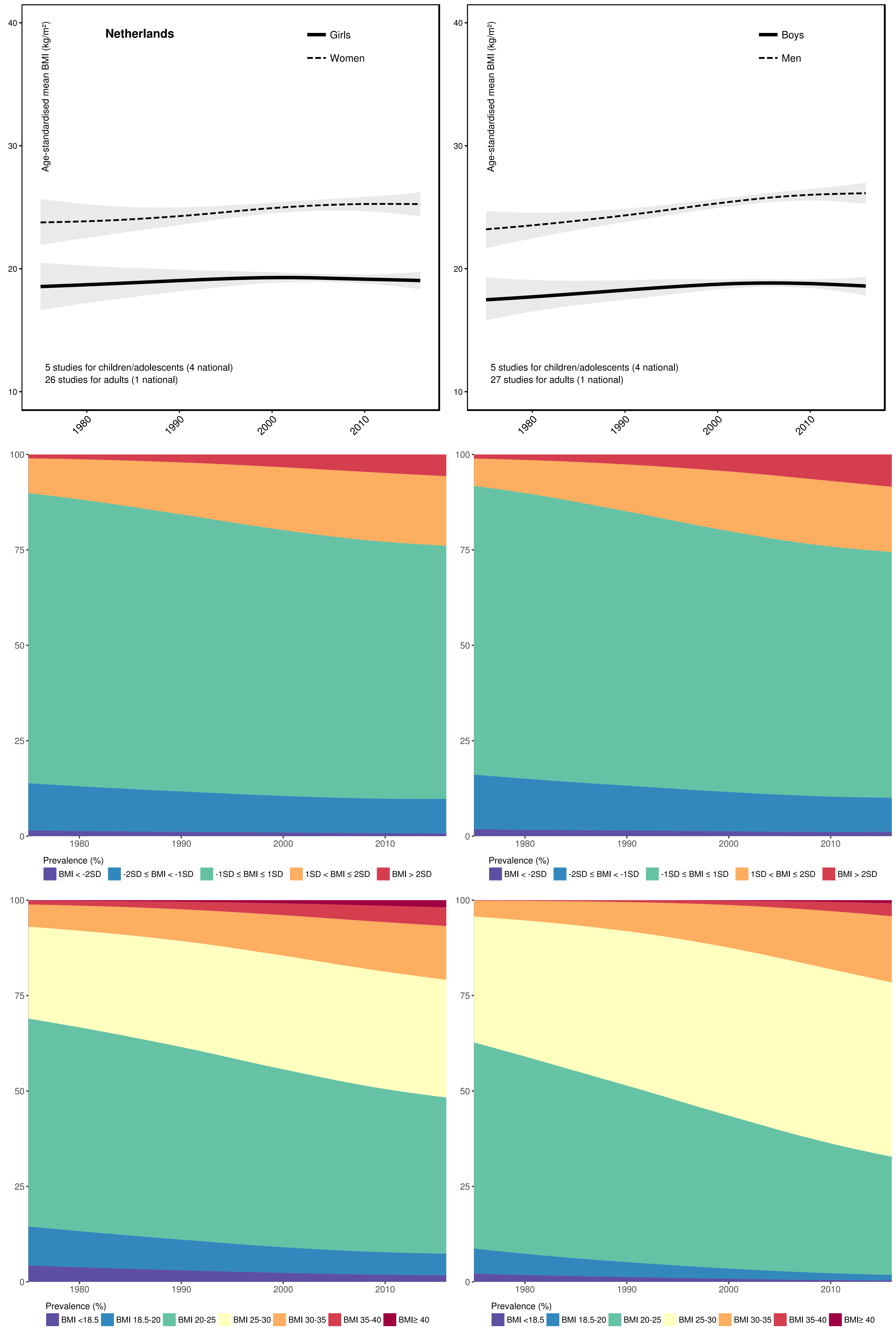
Netherlands
Girls
Women
Boys
Men
Age-standardised mean BMI (kg/m²)
5 studies for children/adolescents (4 national)
26 studies for adults (1 national)
5 studies for children/adolescents (4 national)
27 studies for adults (1 national)
Prevalence (%)
BMI < -2SD
-2SD ≤ BMI < -1SD
-1SD ≤ BMI ≤ 1SD
1SD < BMI ≤ 2SD
BMI > 2SD
BMI <18.5
BMI 18.5-20
BMI 20-25
BMI 25-30
BMI 30-35
BMI 35-40
BMI≥ 40

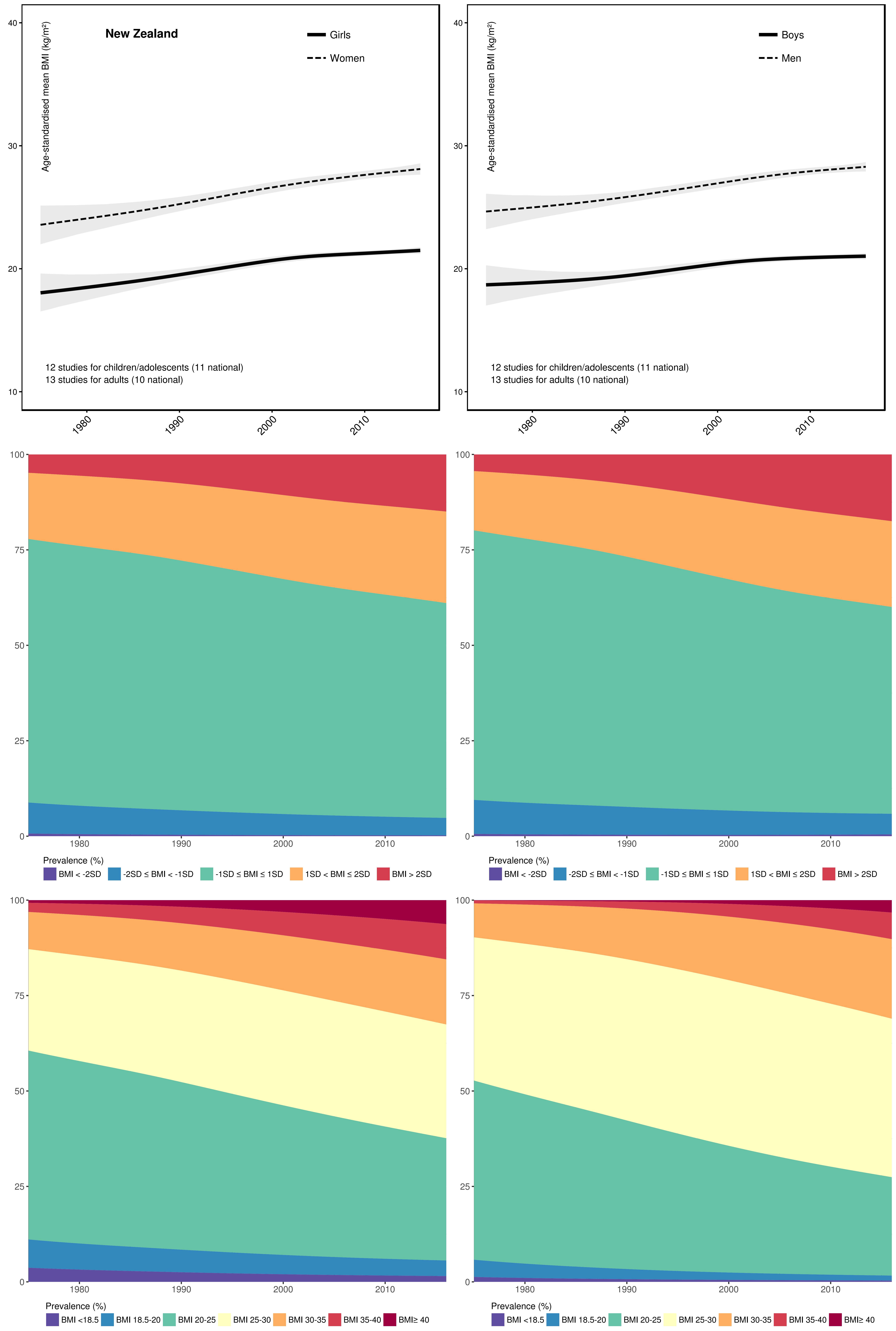
New Zealand
Girls
Women
Boys
Men
Age-standardised mean BMI (kg/m²)
12 studies for children/adolescents (11 national)
13 studies for adults (10 national)
Prevalence (%)
BMI < -2SD
-2SD ≤ BMI < -1SD
-1SD ≤ BMI ≤ 1SD
1SD < BMI ≤ 2SD
BMI > 2SD
BMI <18.5
BMI 18.5-20
BMI 20-25
BMI 25-30
BMI 30-35
BMI 35-40
BMI≥ 40

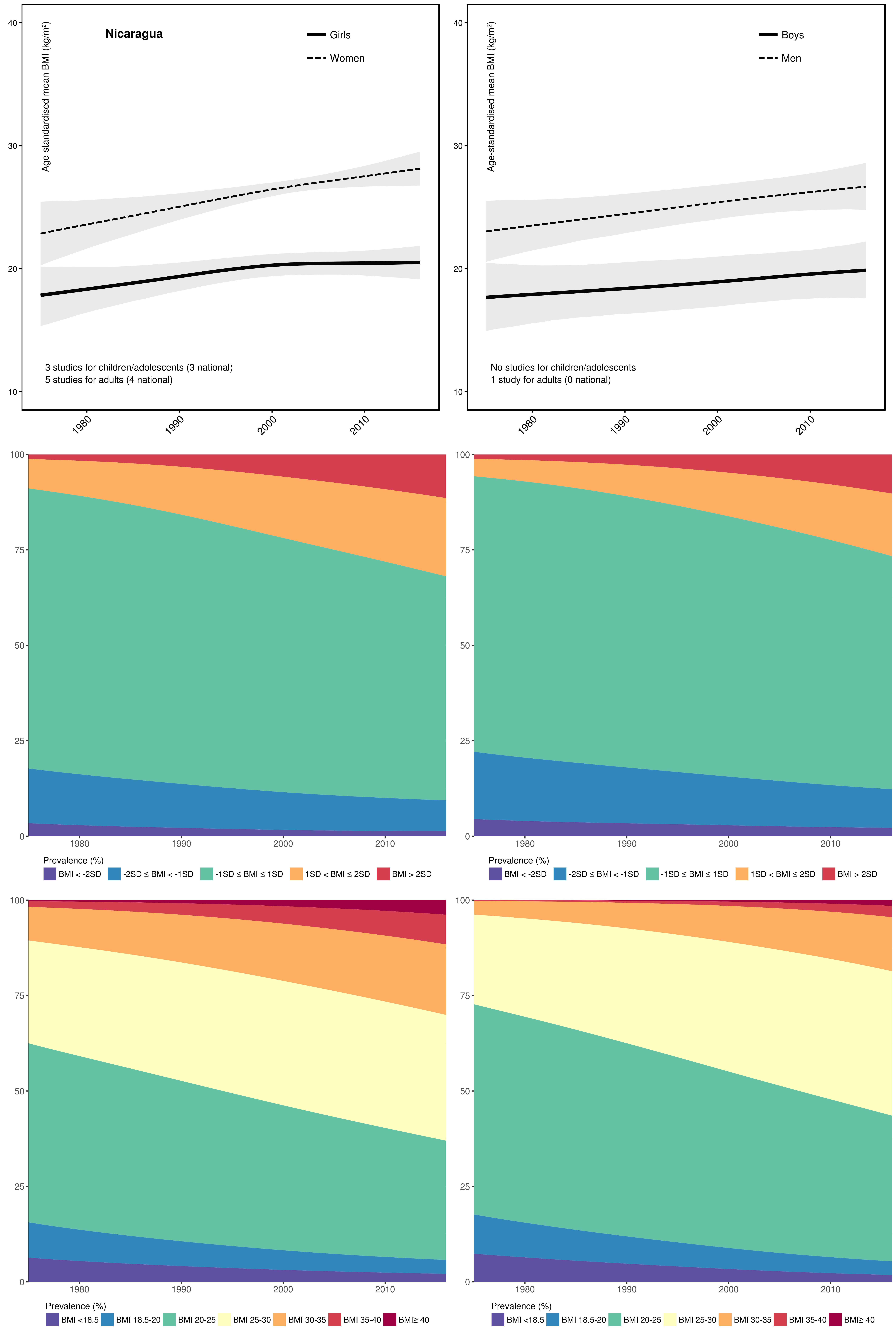

Nicaragua
Girls
Women
Age-standardised mean BMI (kg/m²)
3 studies for children/adolescents (3 national)
5 studies for adults (4 national)
Boys
Men
No studies for children/adolescents
1 study for adults (0 national)
Prevalence (%)
BMI < -2SD
-2SD ≤ BMI < -1SD
-1SD ≤ BMI ≤ 1SD
1SD < BMI ≤ 2SD
BMI > 2SD
BMI <18.5
BMI 18.5-20
BMI 20-25
BMI 25-30
BMI 30-35
BMI 35-40
BMI≥ 40

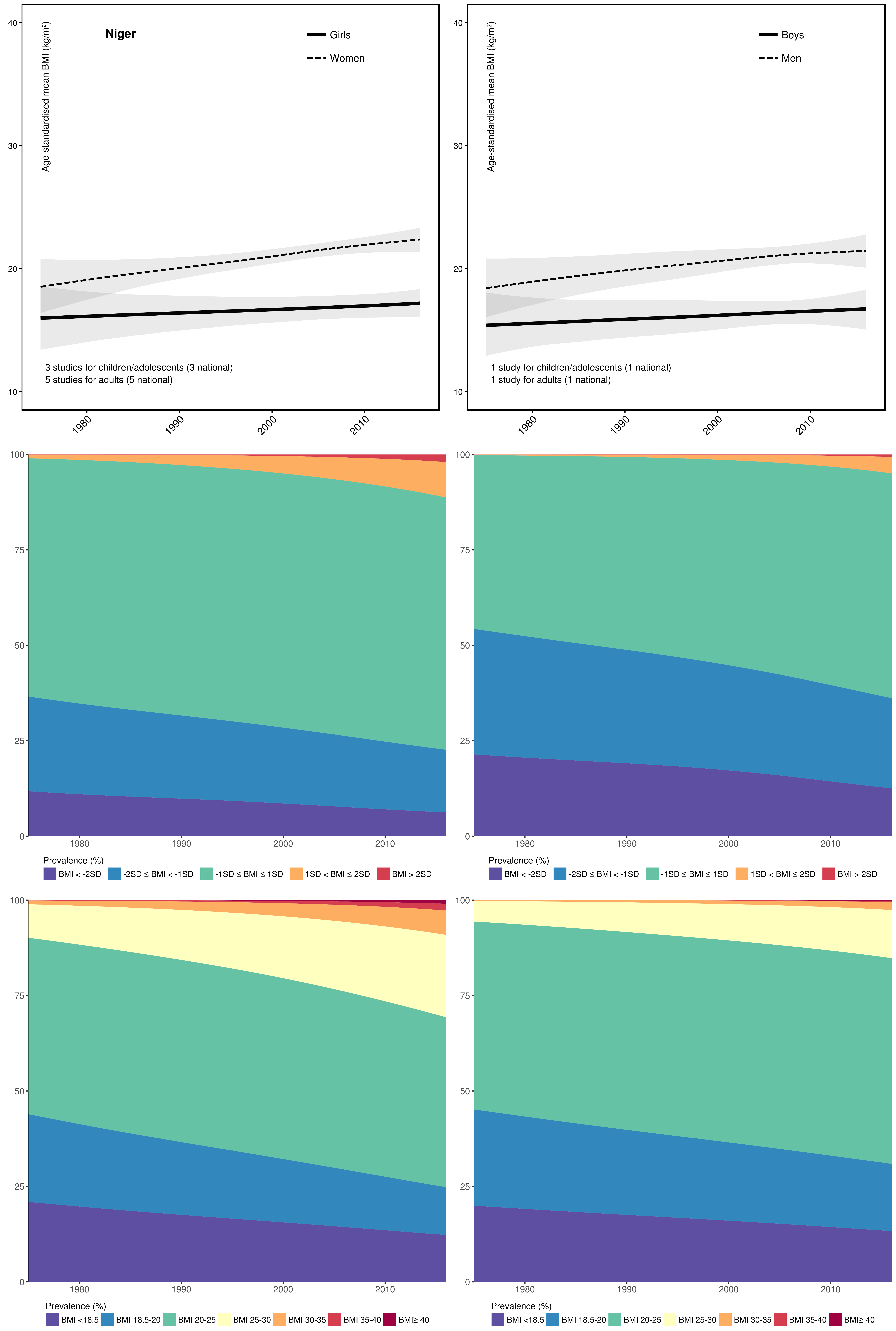
Niger
Girls
Women
Boys
Men
Age-standardised mean BMI (kg/m²)
3 studies for children/adolescents (3 national)
5 studies for adults (5 national)
1 study for children/adolescents (1 national)
1 study for adults (1 national)
Prevalence (%)
BMI < -2SD
-2SD ≤ BMI < -1SD
-1SD ≤ BMI ≤ 1SD
1SD < BMI ≤ 2SD
BMI > 2SD
BMI <18.5
BMI 18.5-20
BMI 20-25
BMI 25-30
BMI 30-35
BMI 35-40
BMI≥ 40

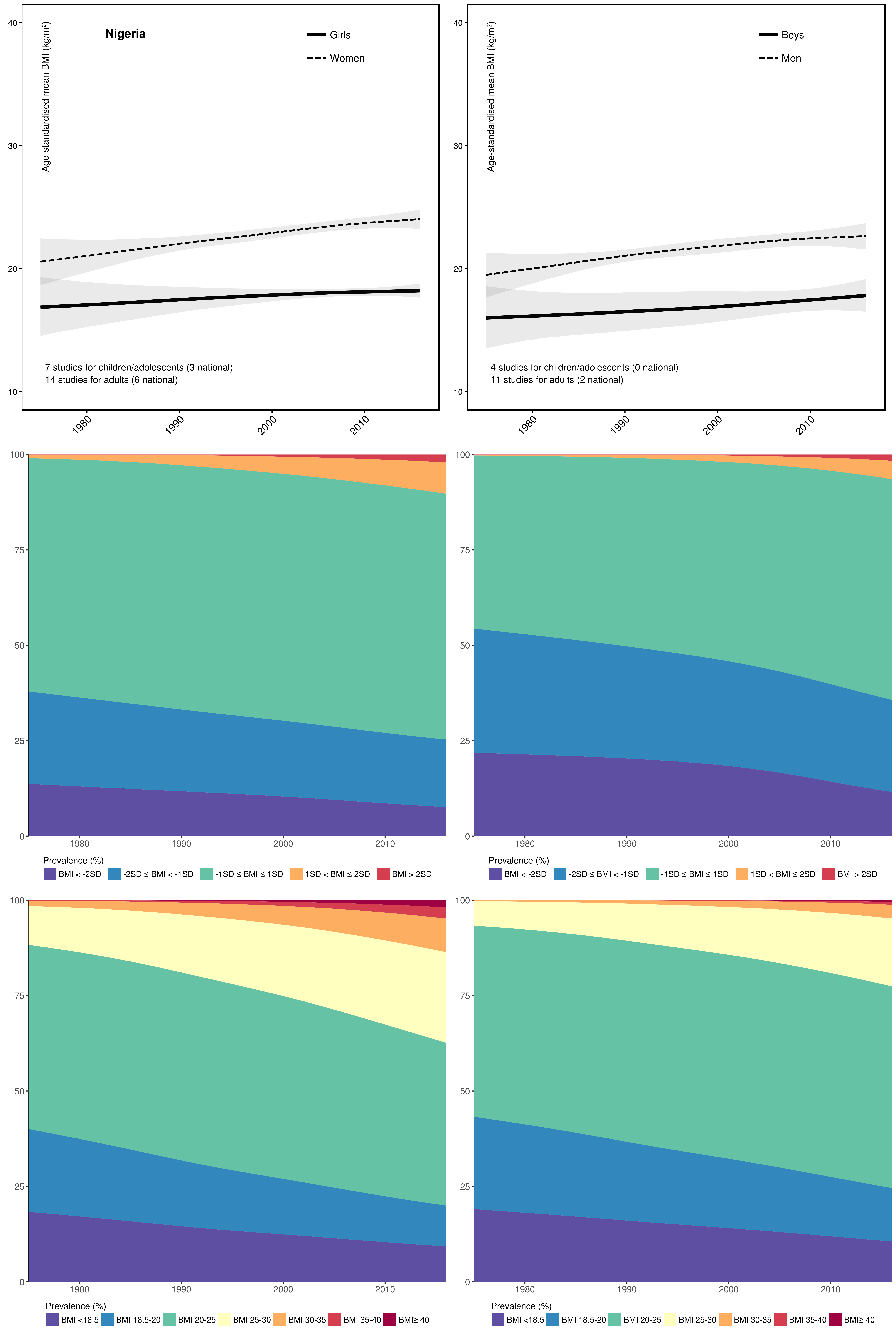
Nigeria
Girls
Women
Boys
Men
Age-standardised mean BMI (kg/m²)
7 studies for children/adolescents (3 national)
14 studies for adults (6 national)
4 studies for children/adolescents (0 national)
11 studies for adults (2 national)
Prevalence (%)
BMI < -2SD
-2SD ≤ BMI < -1SD
-1SD ≤ BMI ≤ 1SD
1SD < BMI ≤ 2SD
BMI > 2SD
BMI <18.5
BMI 18.5-20
BMI 20-25
BMI 25-30
BMI 30-35
BMI 35-40
BMI≥ 40

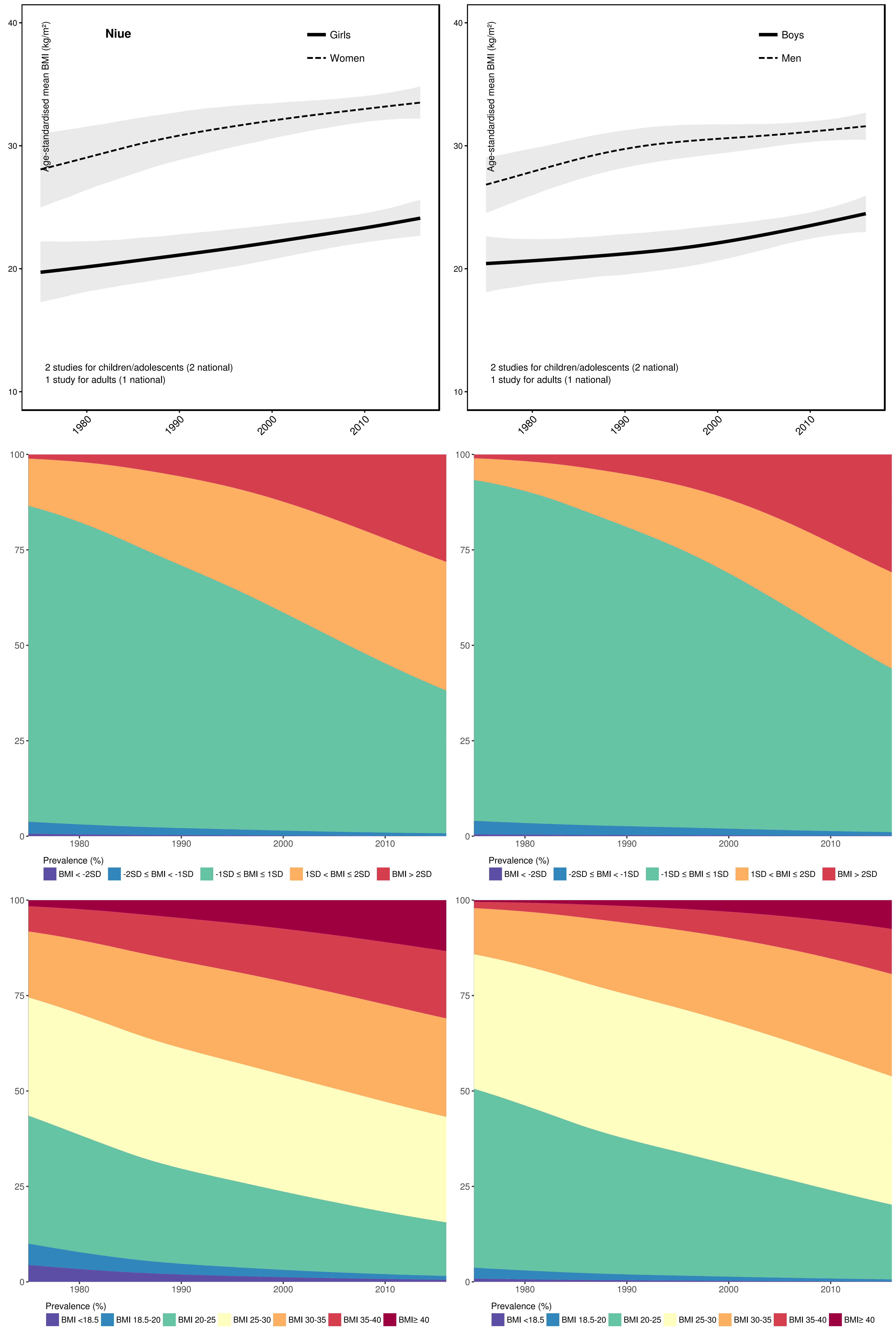
Niue
Girls
Women
Age-standardised mean BMI (kg/m²)
2 studies for children/adolescents (2 national)
1 study for adults (1 national)
Boys
Men
Age-standardised mean BMI (kg/m²)
2 studies for children/adolescents (2 national)
1 study for adults (1 national)
Prevalence (%)
BMI < -2SD
-2SD ≤ BMI < -1SD
-1SD ≤ BMI ≤ 1SD
1SD < BMI ≤ 2SD
BMI > 2SD
Prevalence (%)
BMI <18.5
BMI 18.5-20
BMI 20-25
BMI 25-30
BMI 30-35
BMI 35-40
BMI≥ 40

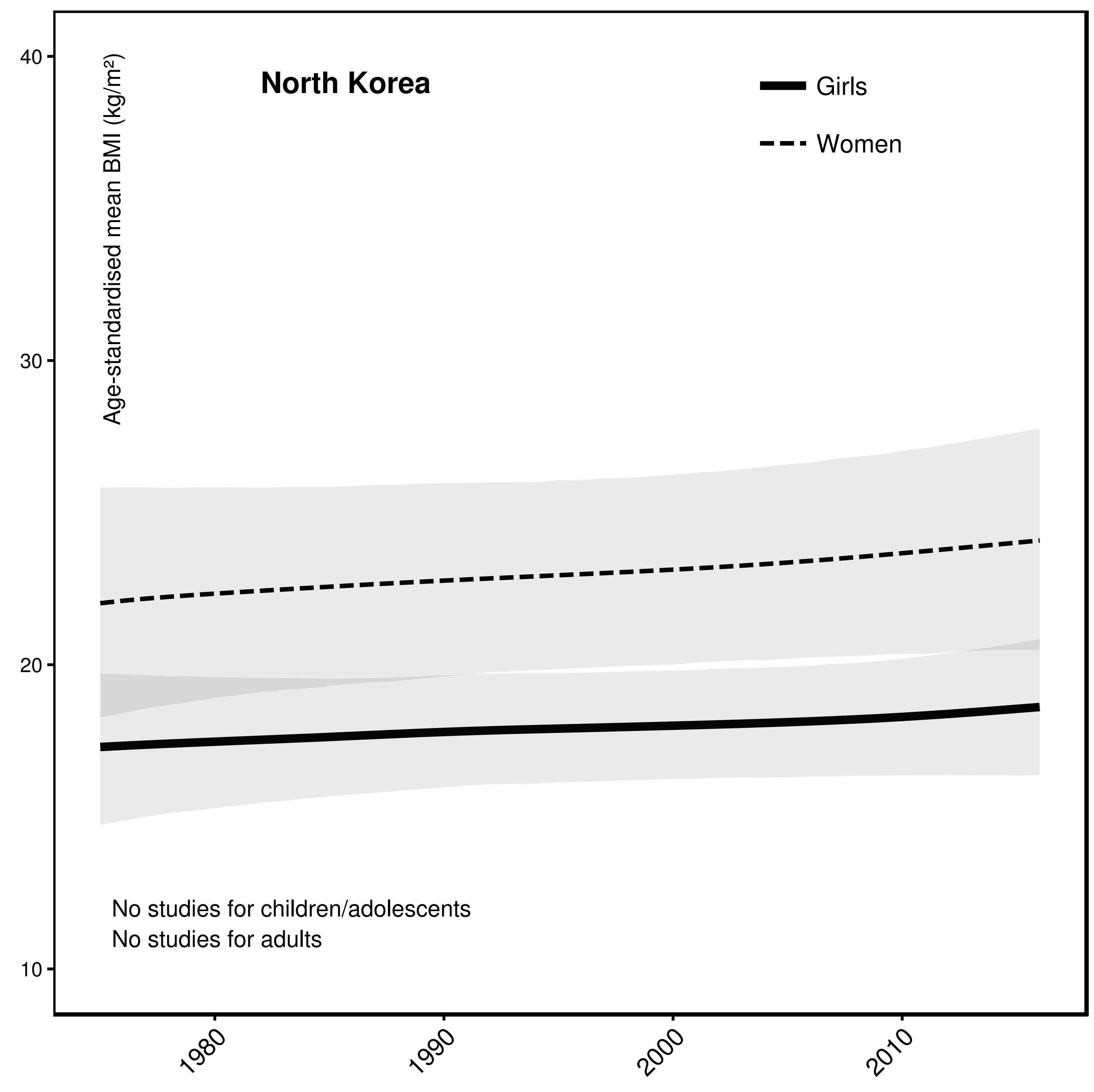
North Korea
Girls
Women
Age-standardised mean BMI (kg/m²)
40
30
20
10
No studies for children/adolescents
No studies for adults
1980
1990
2000
2010

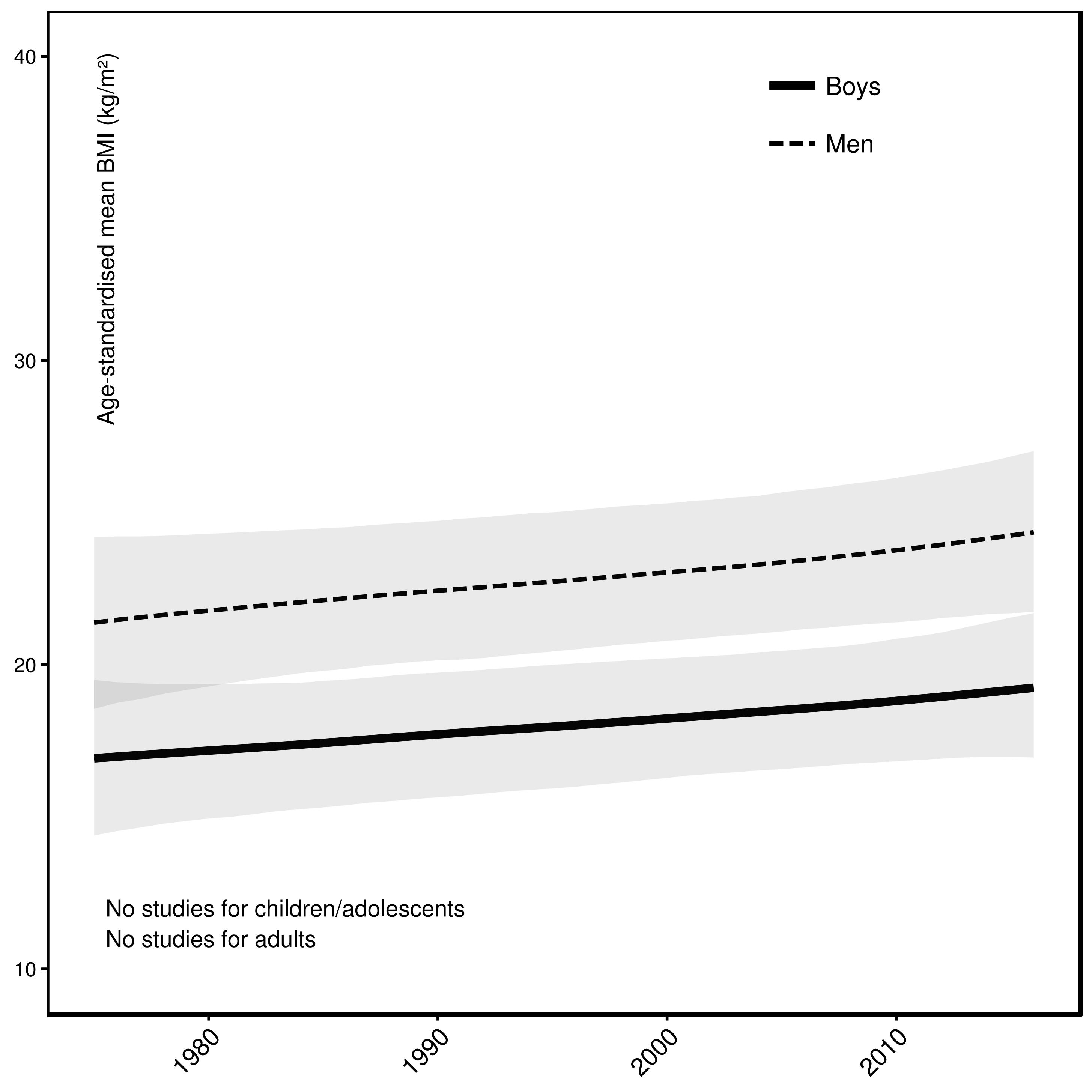
Boys
Men
Age-standardised mean BMI (kg/m²)
40
30
20
10
No studies for children/adolescents
No studies for adults
1980
1990
2000
2010

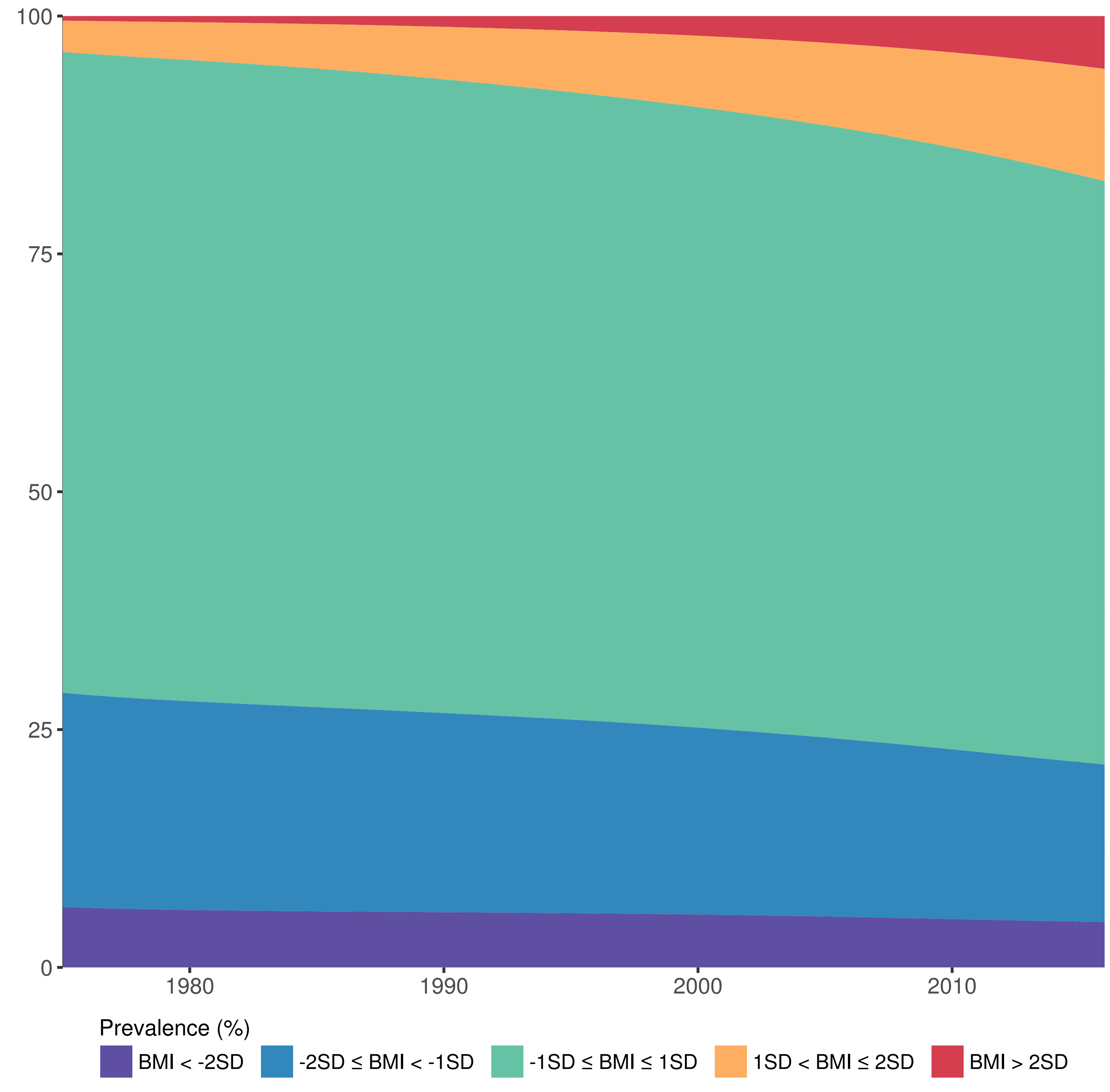
100
75
50
25
0
1980
1990
2000
2010
Prevalence (%)
BMI < -2SD
-2SD ≤ BMI < -1SD
-1SD ≤ BMI ≤ 1SD
1SD < BMI ≤ 2SD
BMI > 2SD

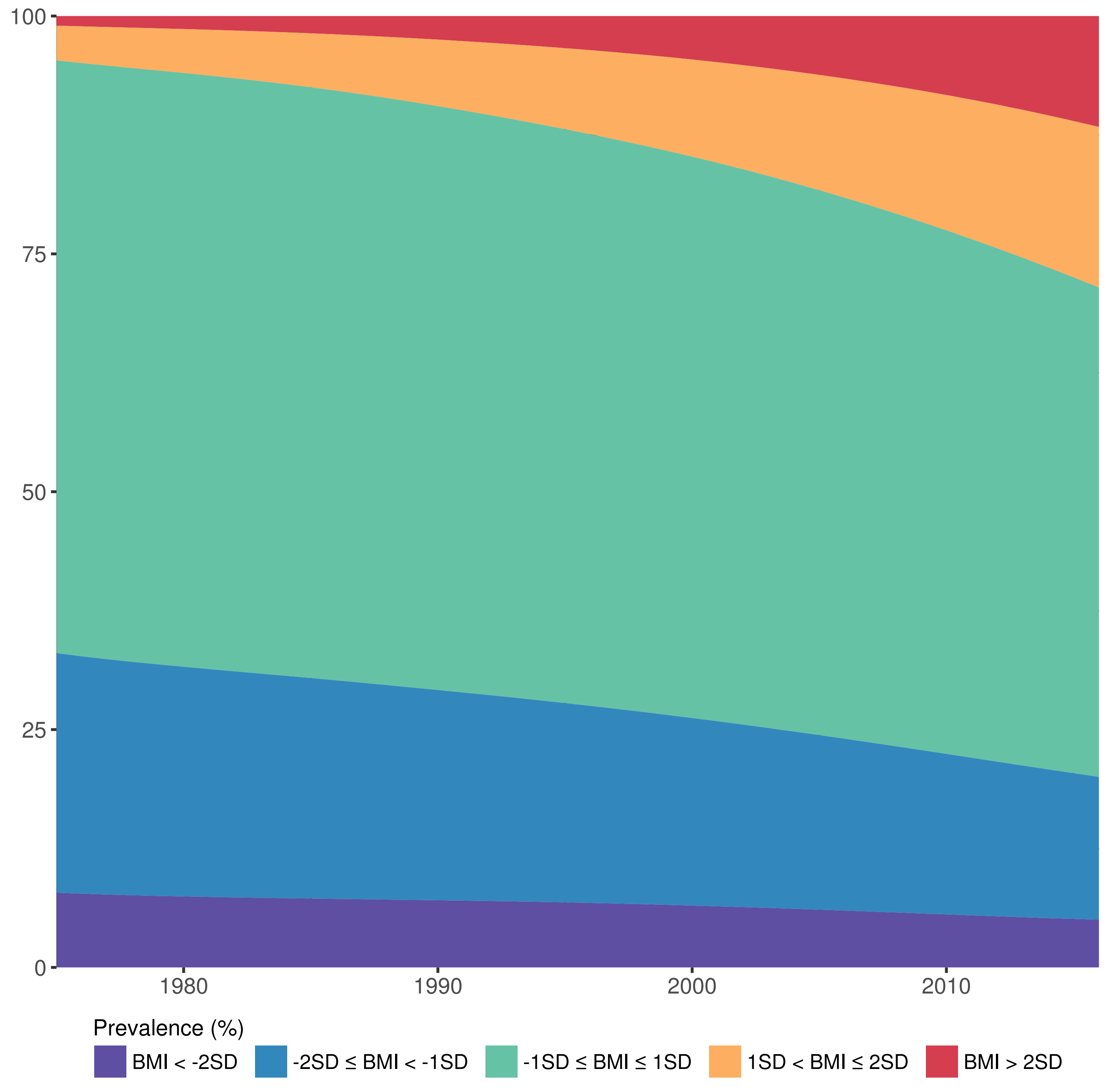
100
75
50
25
0
1980
1990
2000
2010
Prevalence (%)
BMI < -2SD
-2SD ≤ BMI < -1SD
-1SD ≤ BMI ≤ 1SD
1SD < BMI ≤ 2SD
BMI > 2SD

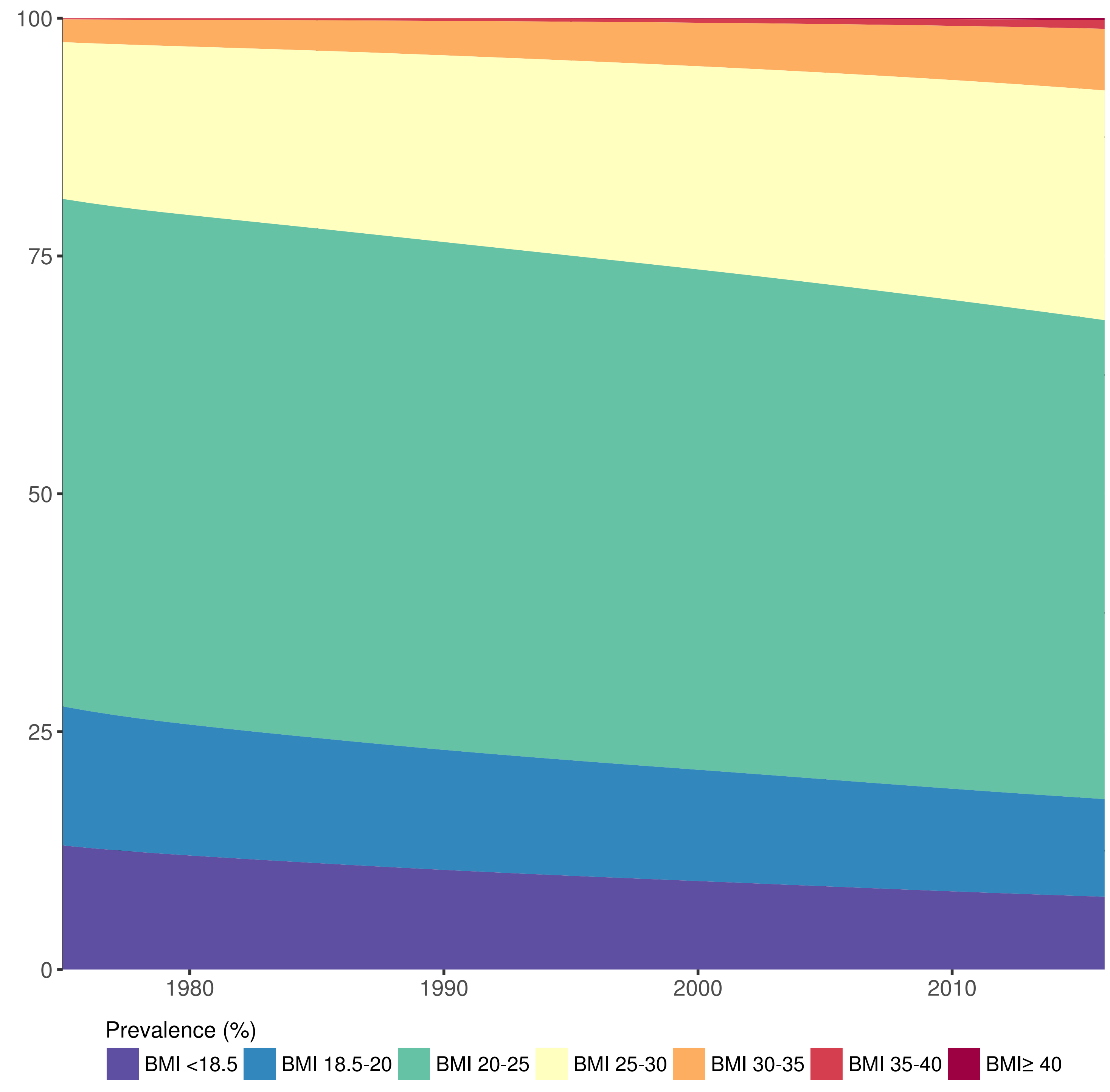
100
75
50
25
0
1980
1990
2000
2010
Prevalence (%)
BMI <18.5
BMI 18.5-20
BMI 20-25
BMI 25-30
BMI 30-35
BMI 35-40
BMI≥ 40

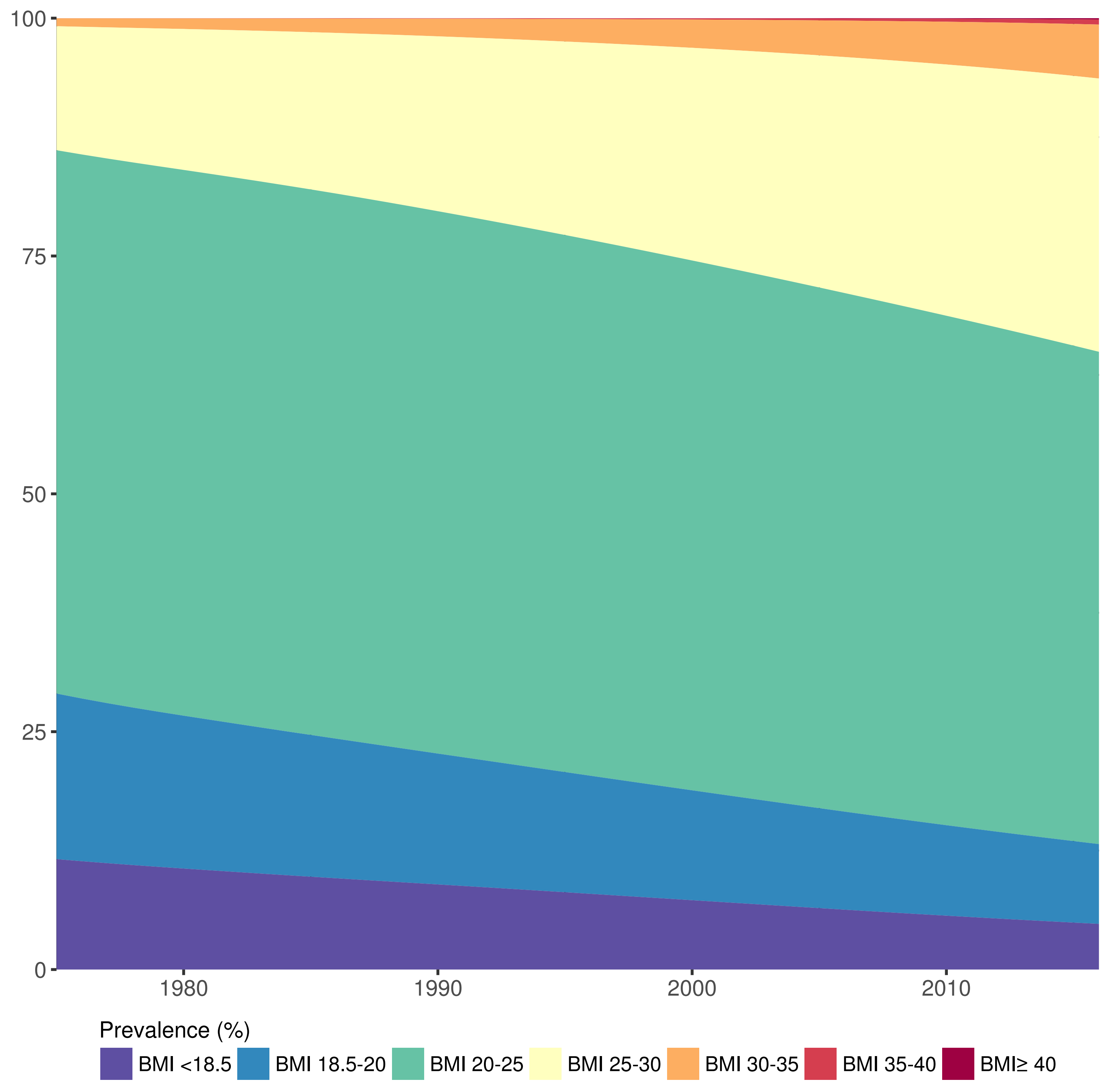
100
75
50
25
0
1980
1990
2000
2010
Prevalence (%)
BMI <18.5
BMI 18.5-20
BMI 20-25
BMI 25-30
BMI 30-35
BMI 35-40
BMI≥ 40

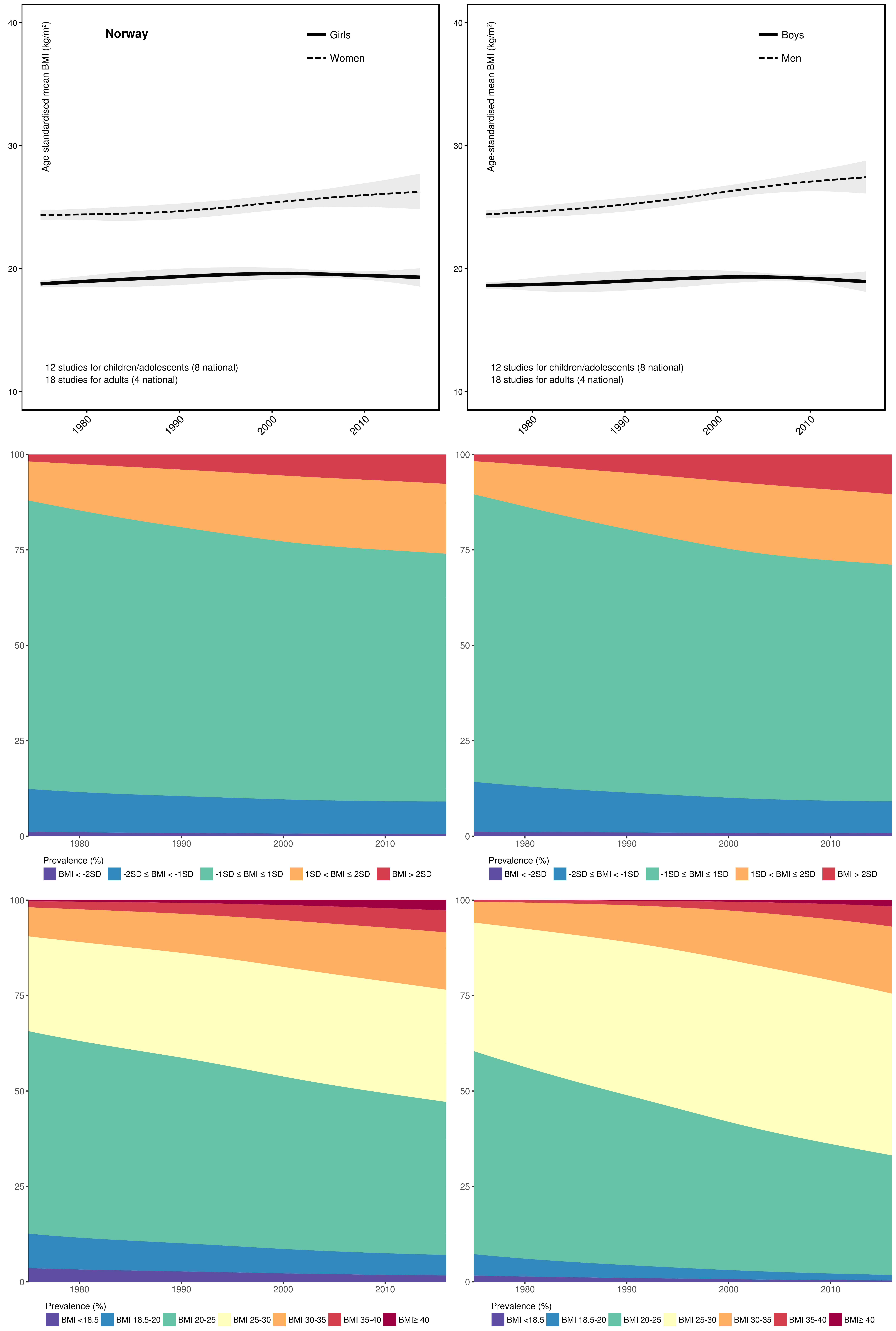
Norway
Girls
Women
Boys
Men
Age-standardised mean BMI (kg/m²)
12 studies for children/adolescents (8 national)
18 studies for adults (4 national)
Prevalence (%)
BMI < -2SD
-2SD ≤ BMI < -1SD
-1SD ≤ BMI ≤ 1SD
1SD < BMI ≤ 2SD
BMI > 2SD
BMI <18.5
BMI 18.5-20
BMI 20-25
BMI 25-30
BMI 30-35
BMI 35-40
BMI≥ 40

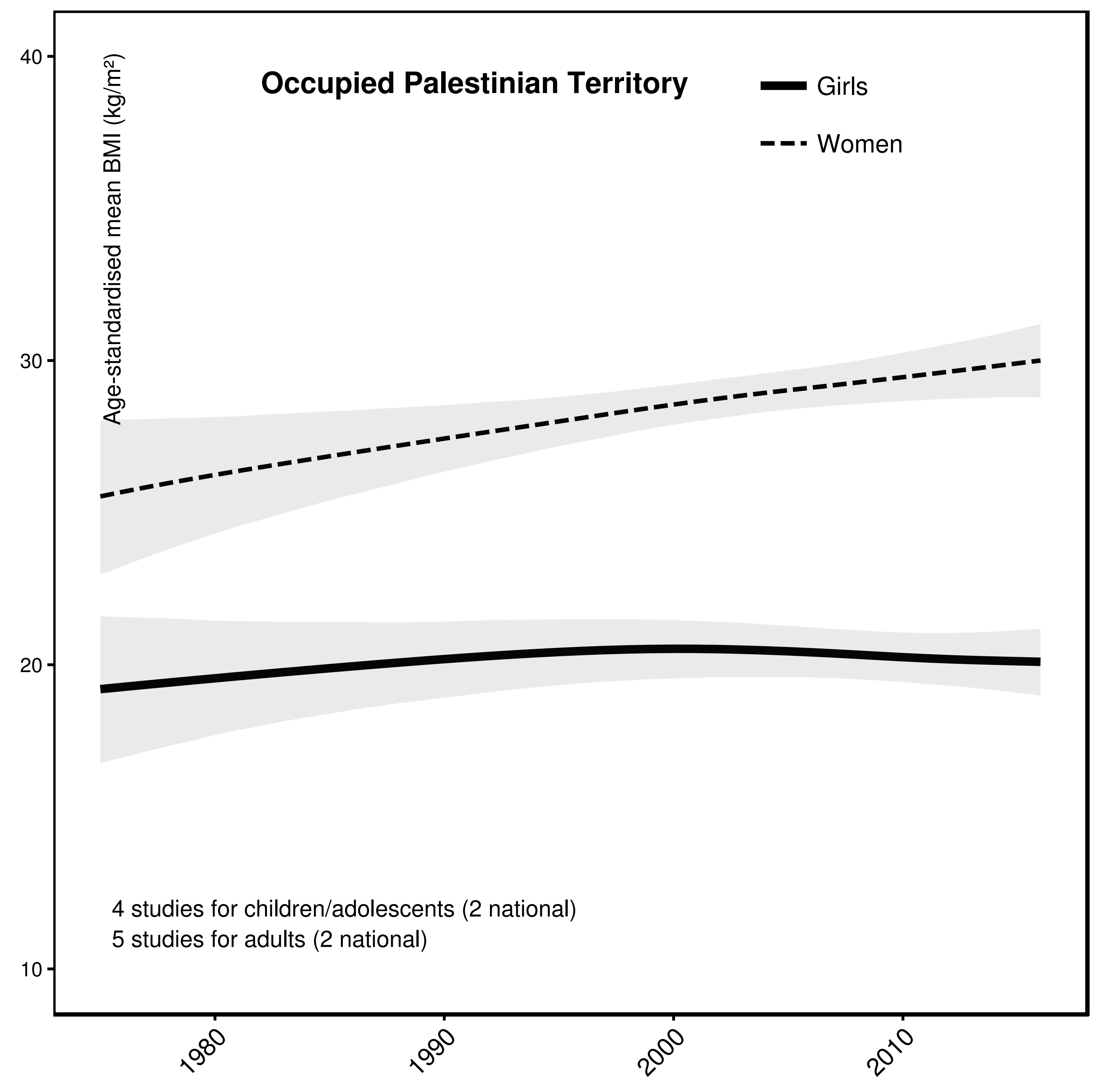

Occupied Palestinian Territory
Girls
Women
Age-standardised mean BMI (kg/m²)
40
30
20
10
4 studies for children/adolescents (2 national)
5 studies for adults (2 national)
1980
1990
2000
2010

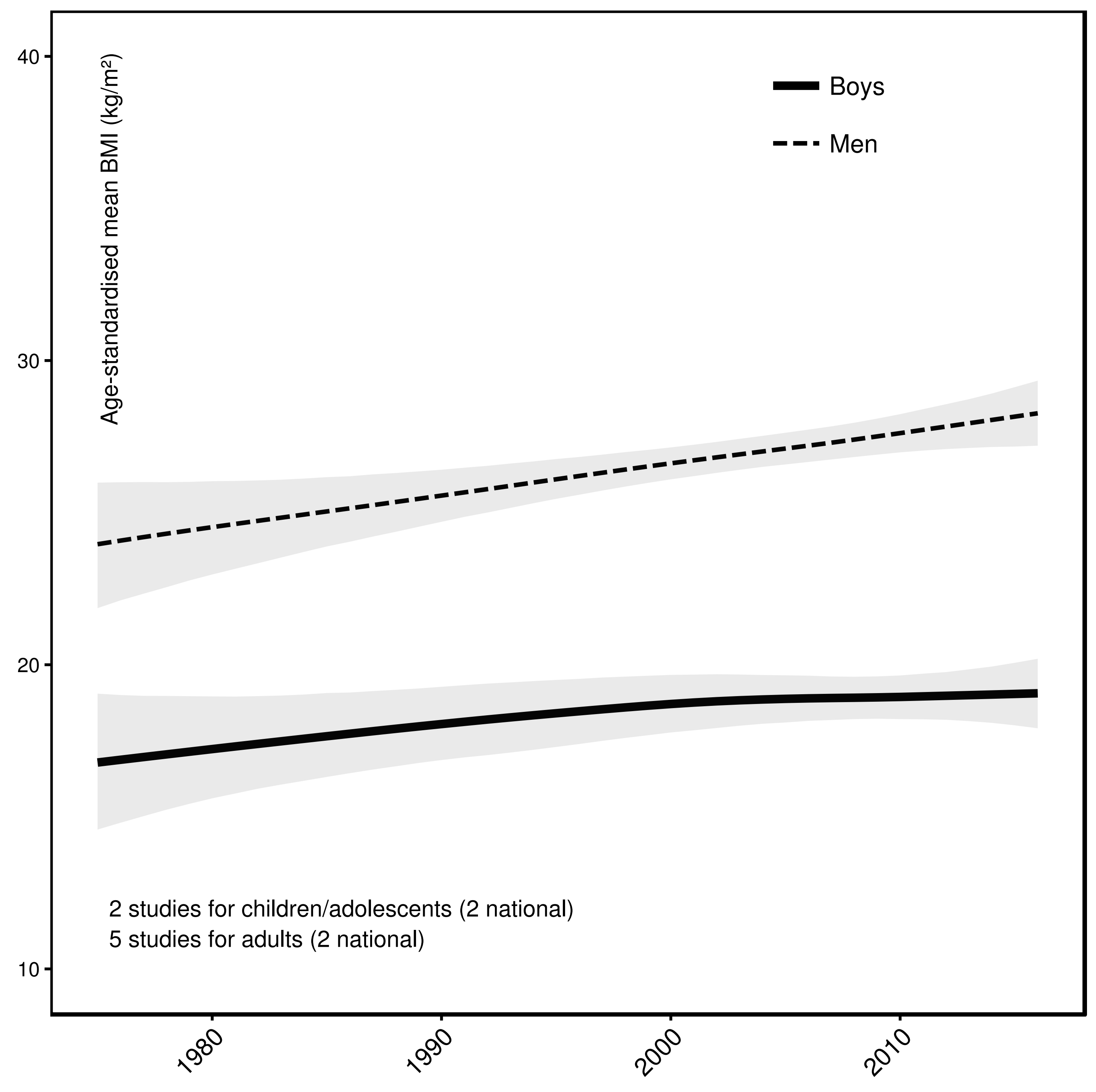

Boys
Men
Age-standardised mean BMI (kg/m²)
40
30
20
10
2 studies for children/adolescents (2 national)
5 studies for adults (2 national)
1980
1990
2000
2010

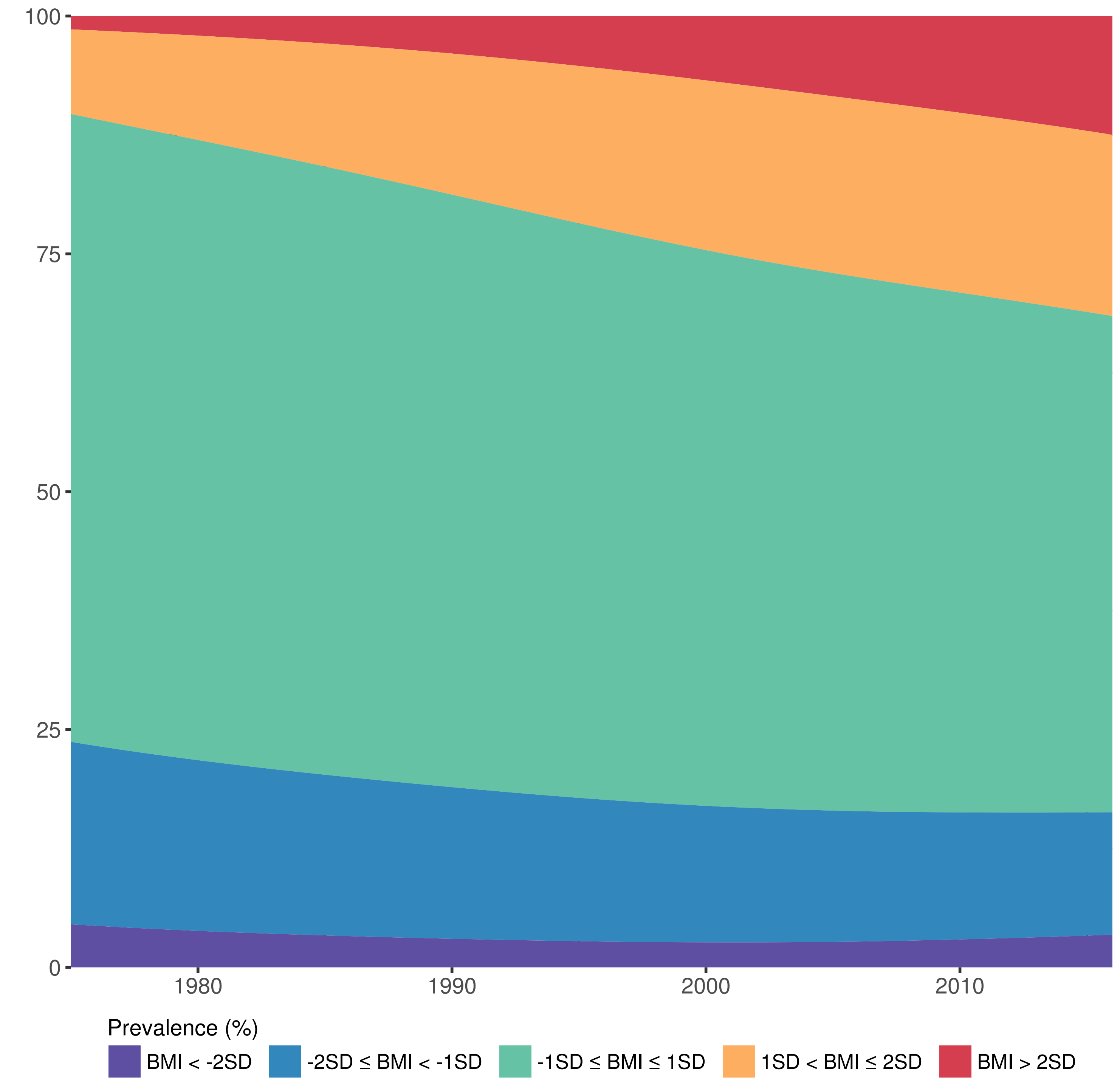

100
75
50
25
0
1980
1990
2000
2010
Prevalence (%)
BMI < -2SD
-2SD ≤ BMI < -1SD
-1SD ≤ BMI ≤ 1SD
1SD < BMI ≤ 2SD
BMI > 2SD

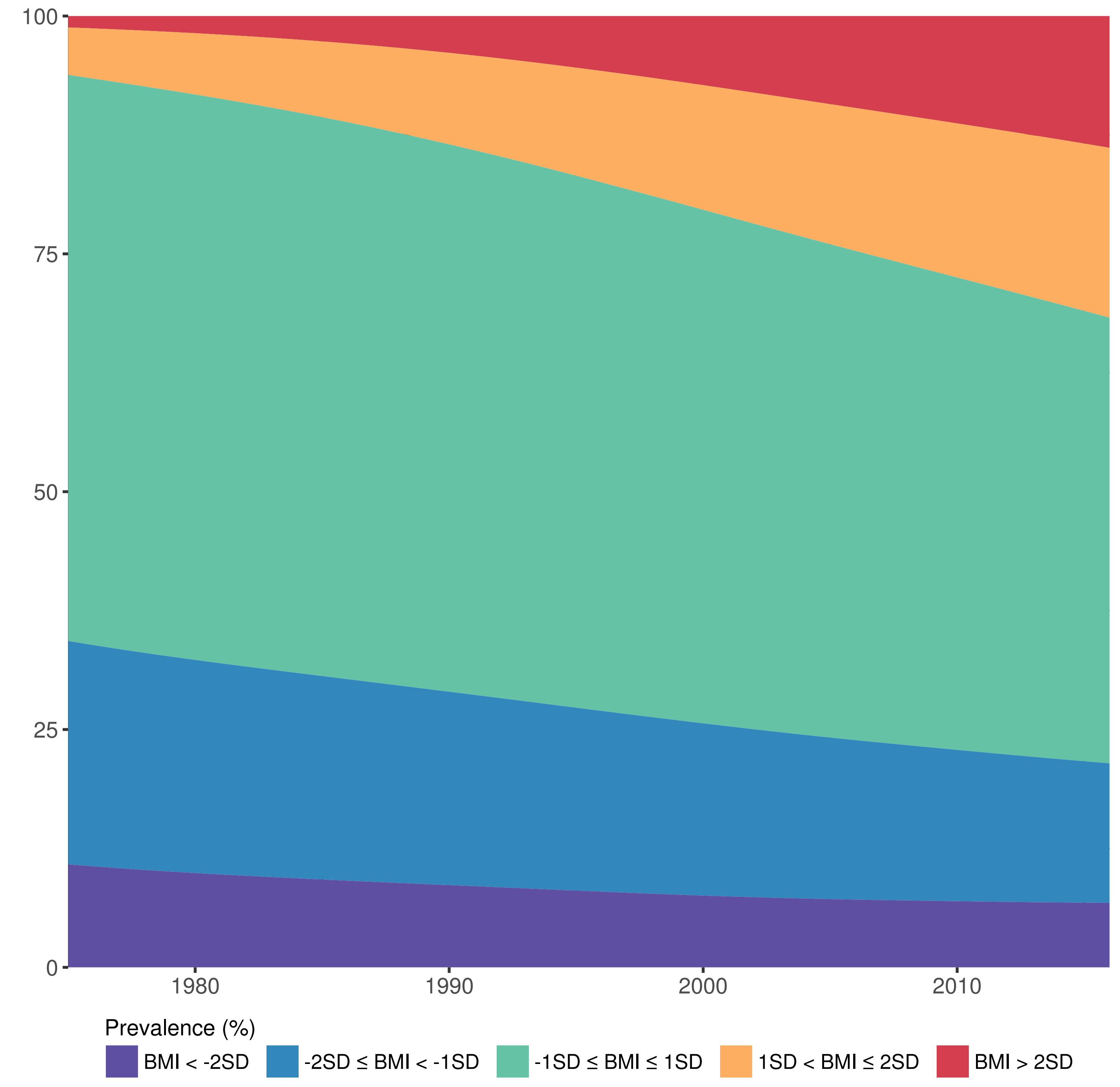

100
75
50
25
0
1980
1990
2000
2010
Prevalence (%)
BMI < -2SD
-2SD ≤ BMI < -1SD
-1SD ≤ BMI ≤ 1SD
1SD < BMI ≤ 2SD
BMI > 2SD

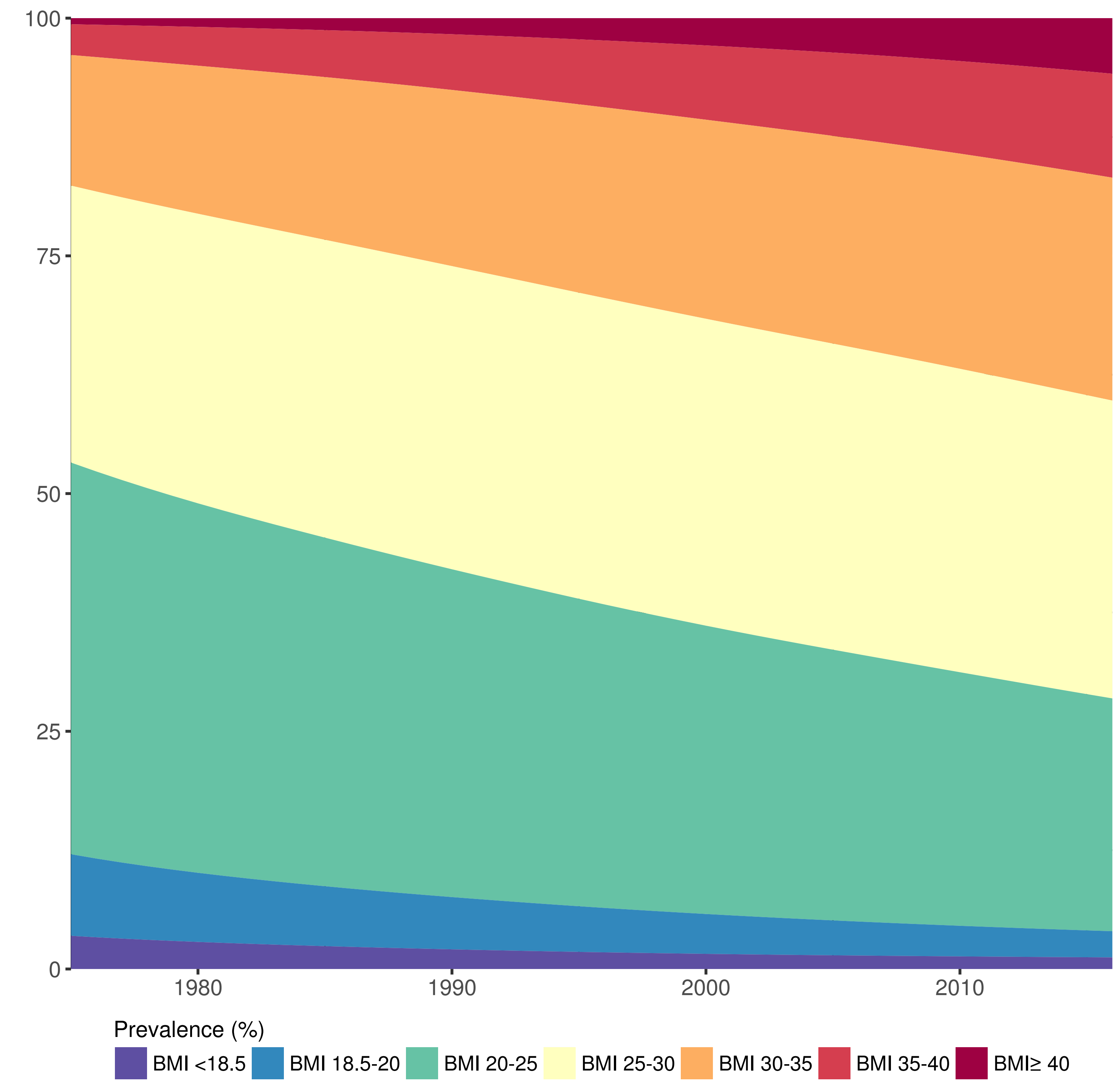

100
75
50
25
0
1980
1990
2000
2010
Prevalence (%)
BMI <18.5
BMI 18.5-20
BMI 20-25
BMI 25-30
BMI 30-35
BMI 35-40
BMI≥ 40

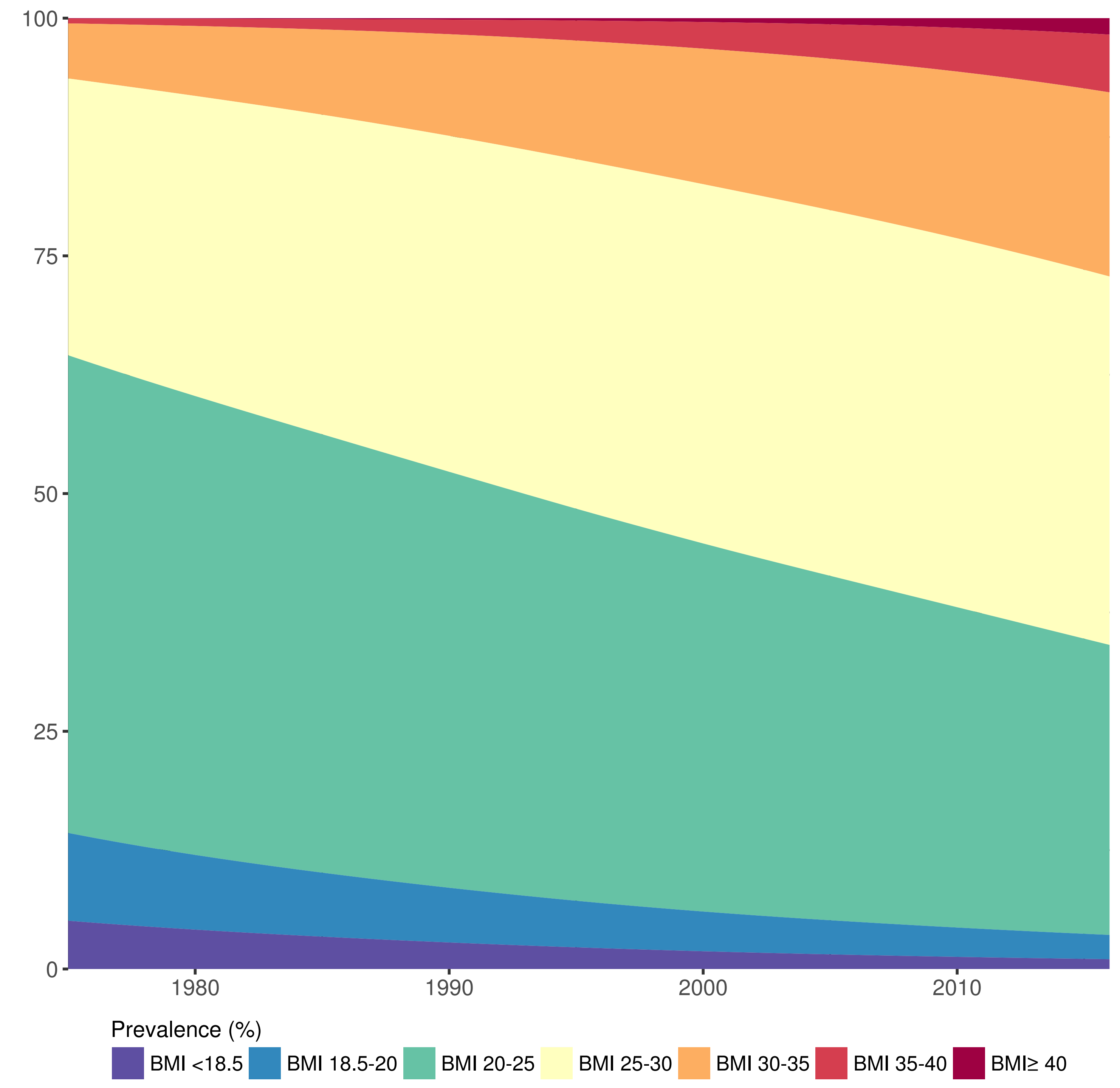

100
75
50
25
0
1980
1990
2000
2010
Prevalence (%)
BMI <18.5
BMI 18.5-20
BMI 20-25
BMI 25-30
BMI 30-35
BMI 35-40
BMI≥ 40

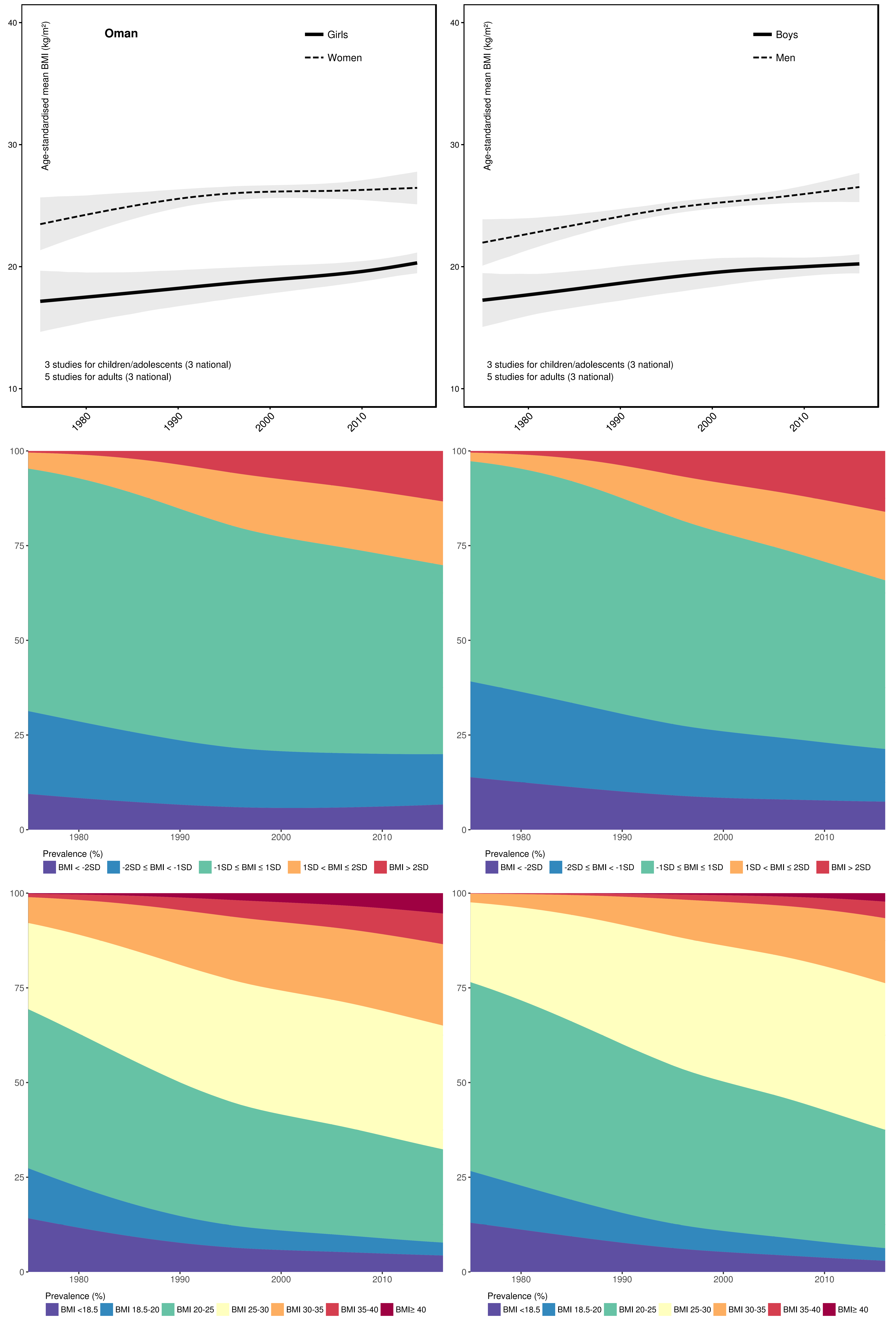
Oman
Girls
Women
Boys
Men
Age-standardised mean BMI (kg/m²)
3 studies for children/adolescents (3 national)
5 studies for adults (3 national)
Prevalence (%)
BMI < -2SD
-2SD ≤ BMI < -1SD
-1SD ≤ BMI ≤ 1SD
1SD < BMI ≤ 2SD
BMI > 2SD
BMI <18.5
BMI 18.5-20
BMI 20-25
BMI 25-30
BMI 30-35
BMI 35-40
BMI≥ 40

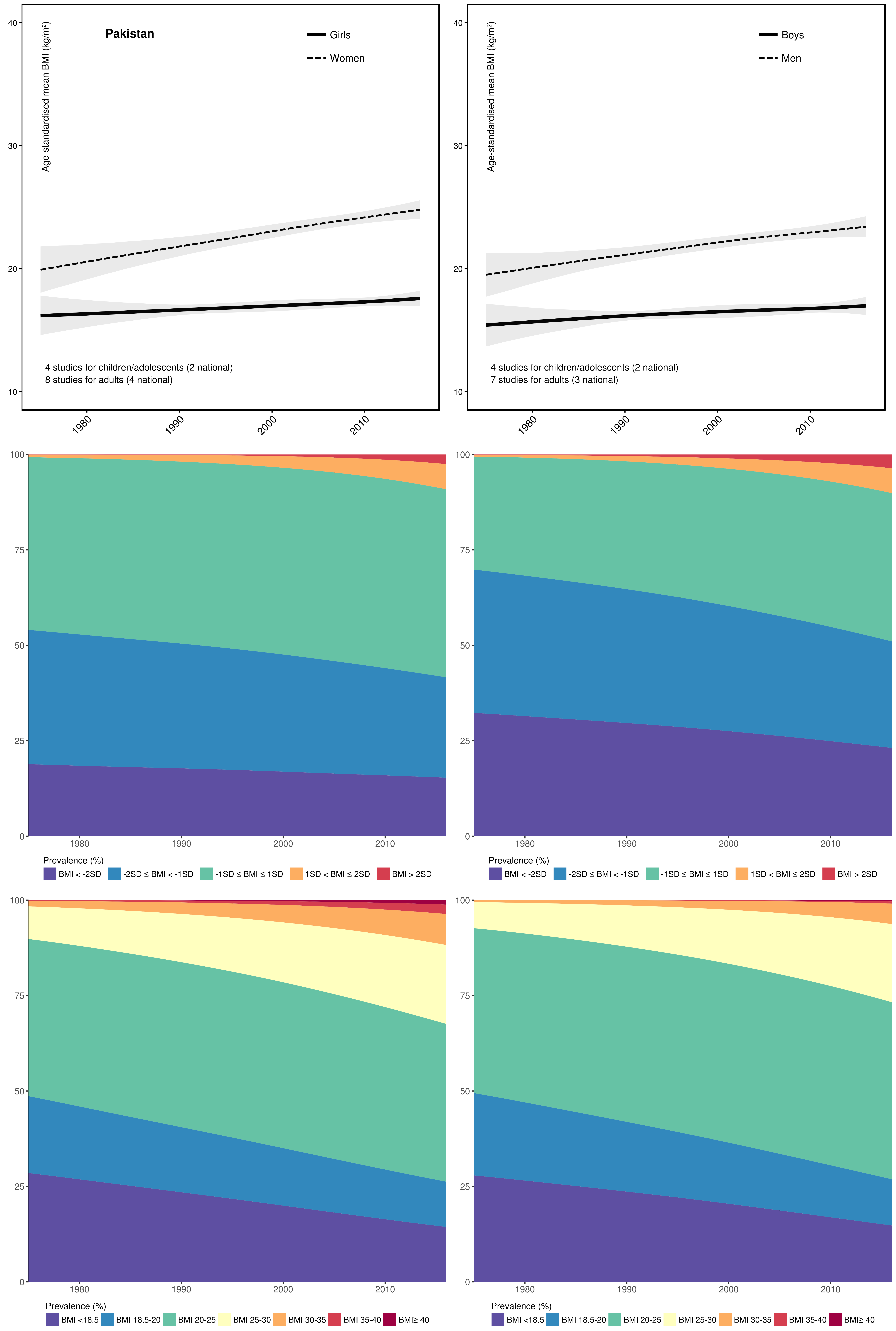

Pakistan
Girls
Women
Boys
Men
Age-standardised mean BMI (kg/m²)
40
30
20
10
1980
1990
2000
2010
4 studies for children/adolescents (2 national)
8 studies for adults (4 national)
4 studies for children/adolescents (2 national)
7 studies for adults (3 national)
100
75
50
25
0
Prevalence (%)
BMI < -2SD
-2SD ≤ BMI < -1SD
-1SD ≤ BMI ≤ 1SD
1SD < BMI ≤ 2SD
BMI > 2SD
Prevalence (%)
BMI <18.5
BMI 18.5-20
BMI 20-25
BMI 25-30
BMI 30-35
BMI 35-40
BMI≥ 40

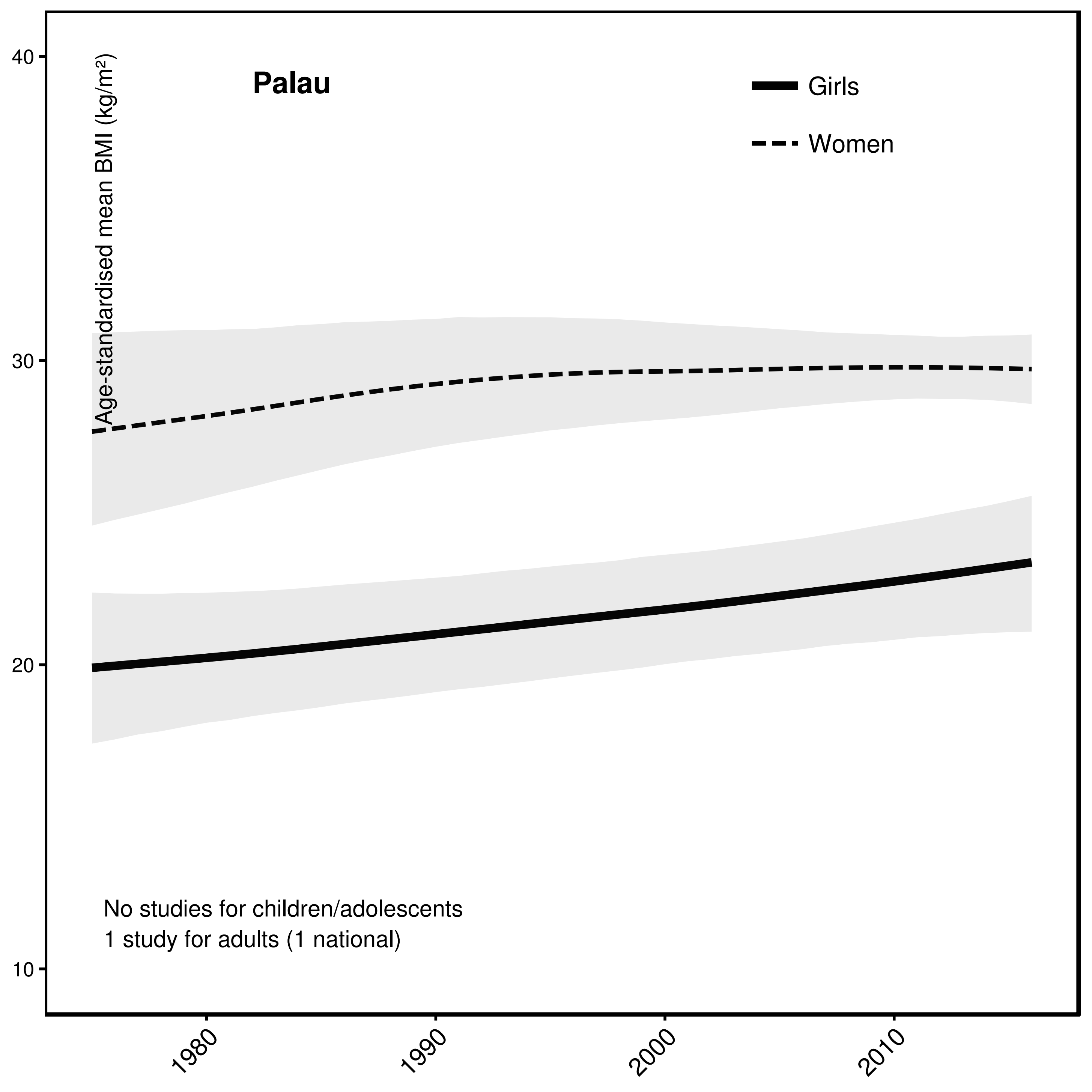
Palau
Girls
Women
Age-standardised mean BMI (kg/m²)
No studies for children/adolescents
1 study for adults (1 national)
40
30
20
10
1980
1990
2000
2010

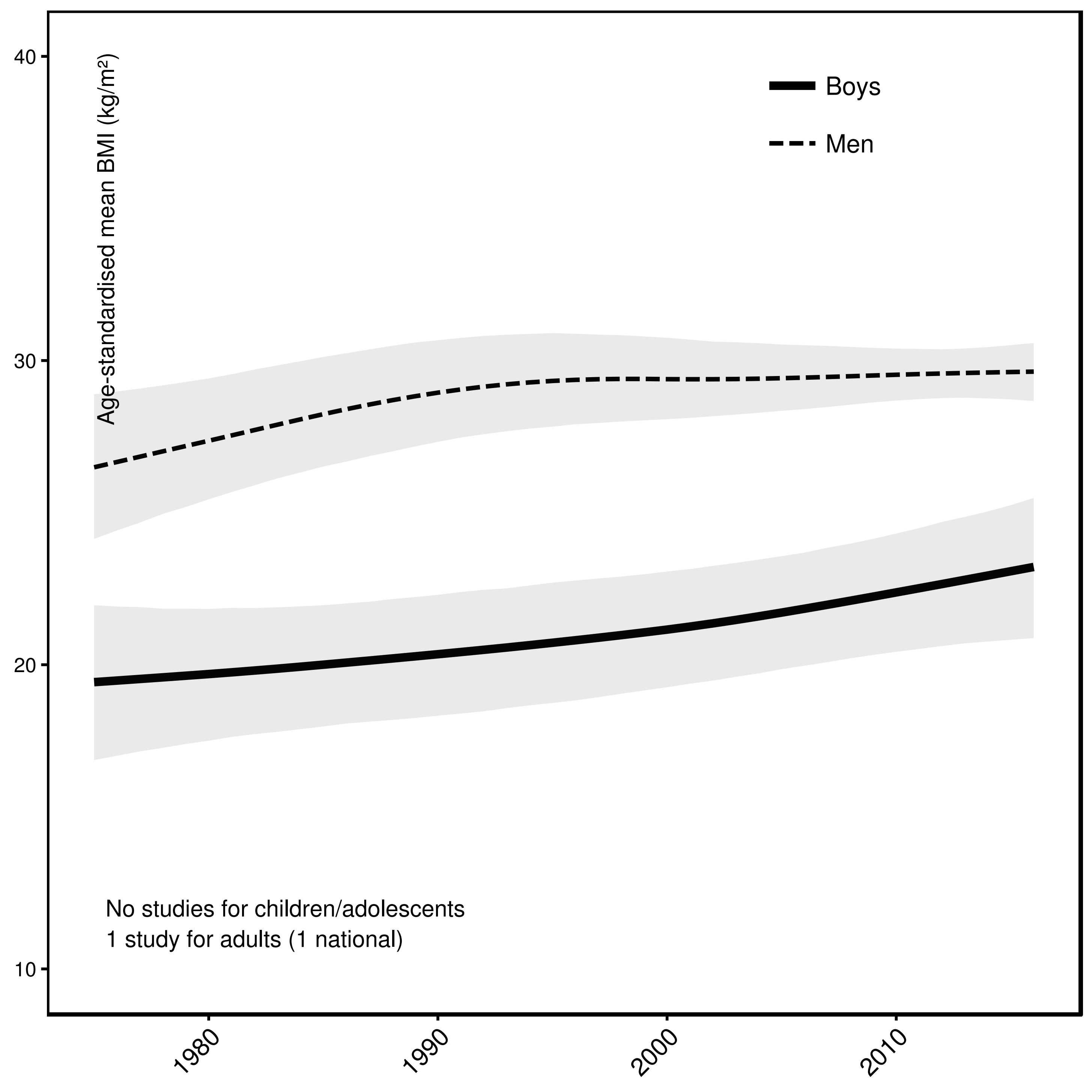
Boys
Men
Age-standardised mean BMI (kg/m²)
No studies for children/adolescents
1 study for adults (1 national)
40
30
20
10
1980
1990
2000
2010

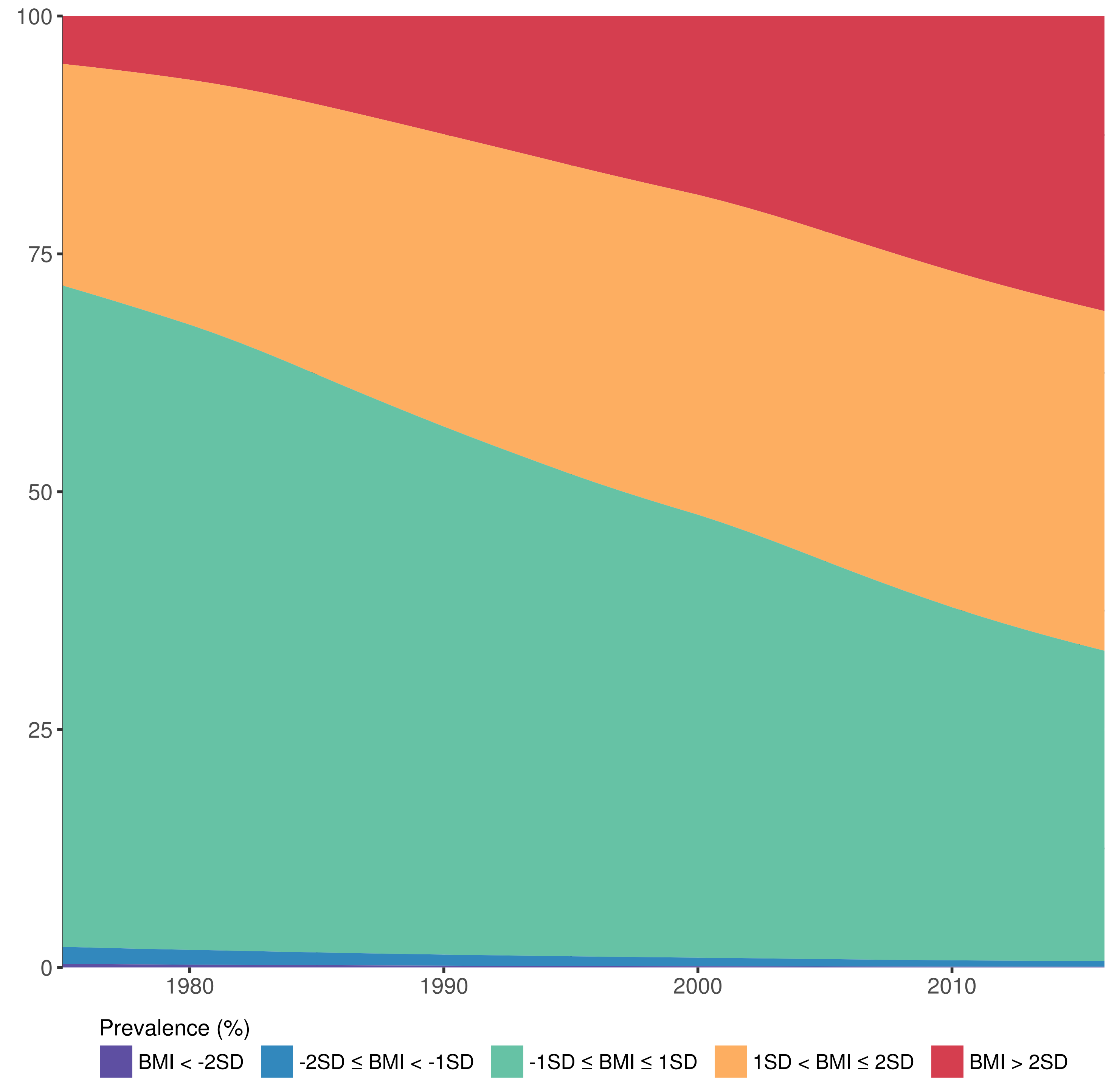
100
75
50
25
0
1980
1990
2000
2010
Prevalence (%)
BMI < -2SD
-2SD ≤ BMI < -1SD
-1SD ≤ BMI ≤ 1SD
1SD < BMI ≤ 2SD
BMI > 2SD

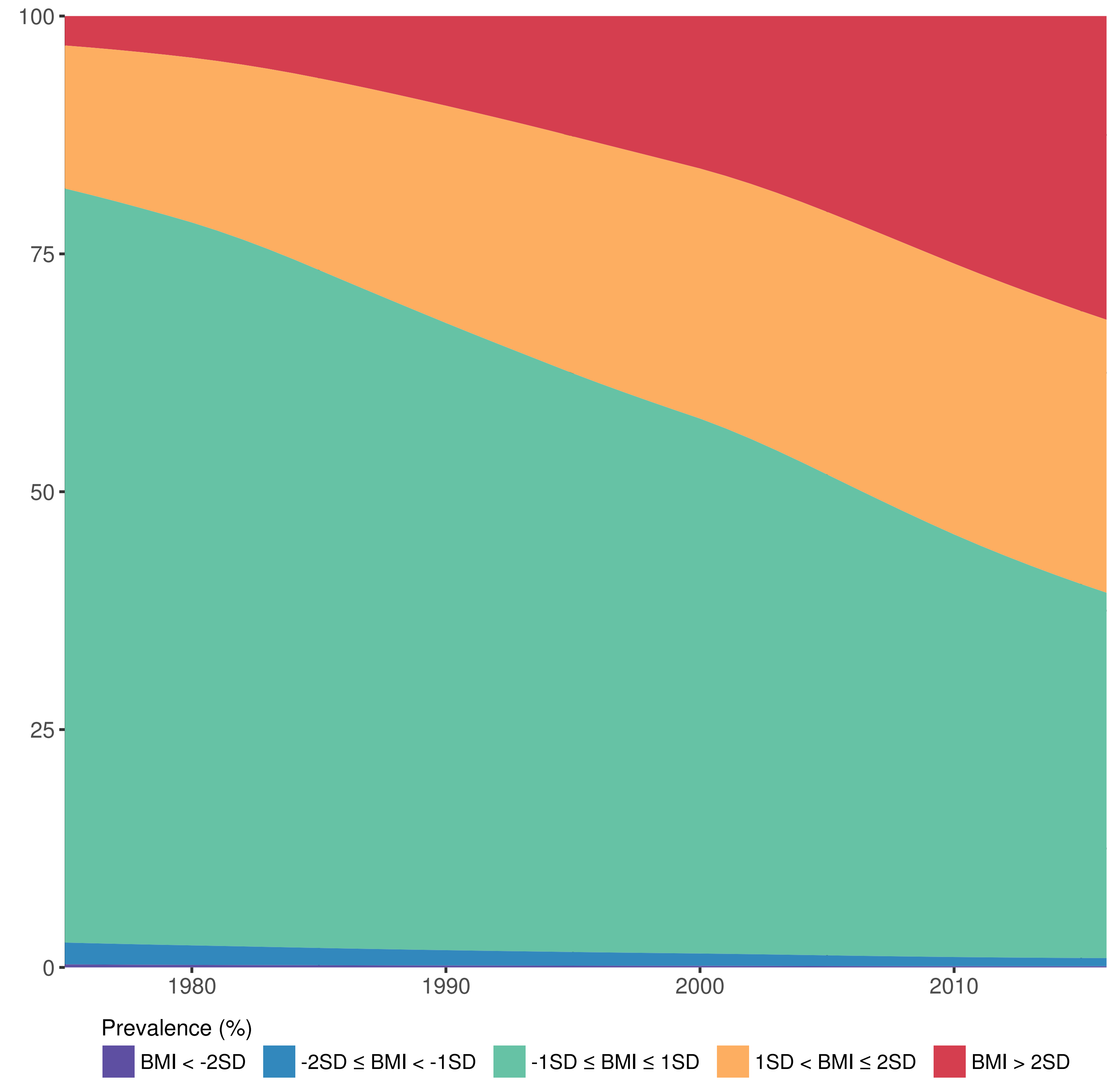
100
75
50
25
0
1980
1990
2000
2010
Prevalence (%)
BMI < -2SD
-2SD ≤ BMI < -1SD
-1SD ≤ BMI ≤ 1SD
1SD < BMI ≤ 2SD
BMI > 2SD

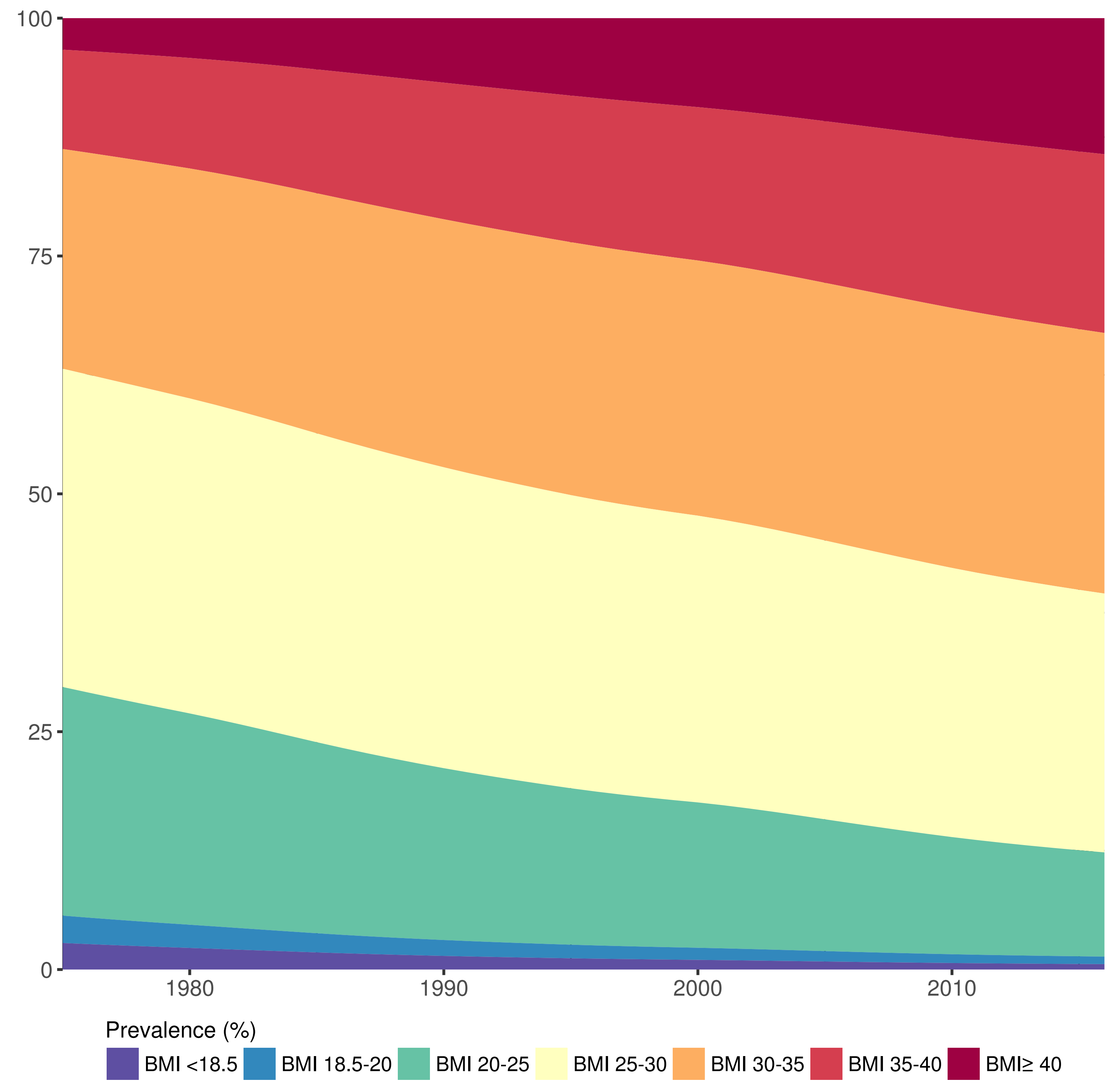
100
75
50
25
0
1980
1990
2000
2010
Prevalence (%)
BMI <18.5
BMI 18.5-20
BMI 20-25
BMI 25-30
BMI 30-35
BMI 35-40
BMI≥ 40

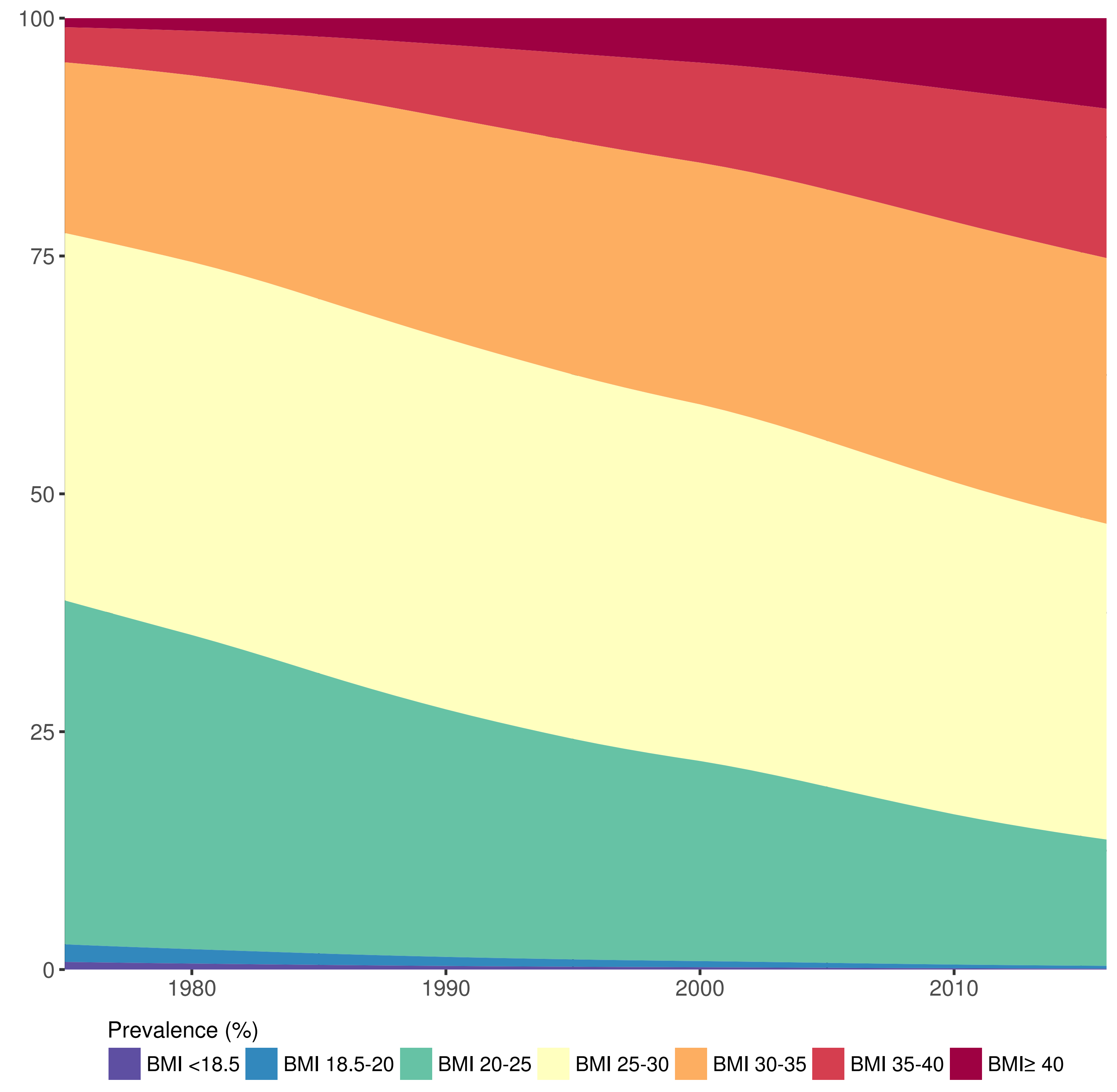
100
75
50
25
0
1980
1990
2000
2010
Prevalence (%)
BMI <18.5
BMI 18.5-20
BMI 20-25
BMI 25-30
BMI 30-35
BMI 35-40
BMI≥ 40

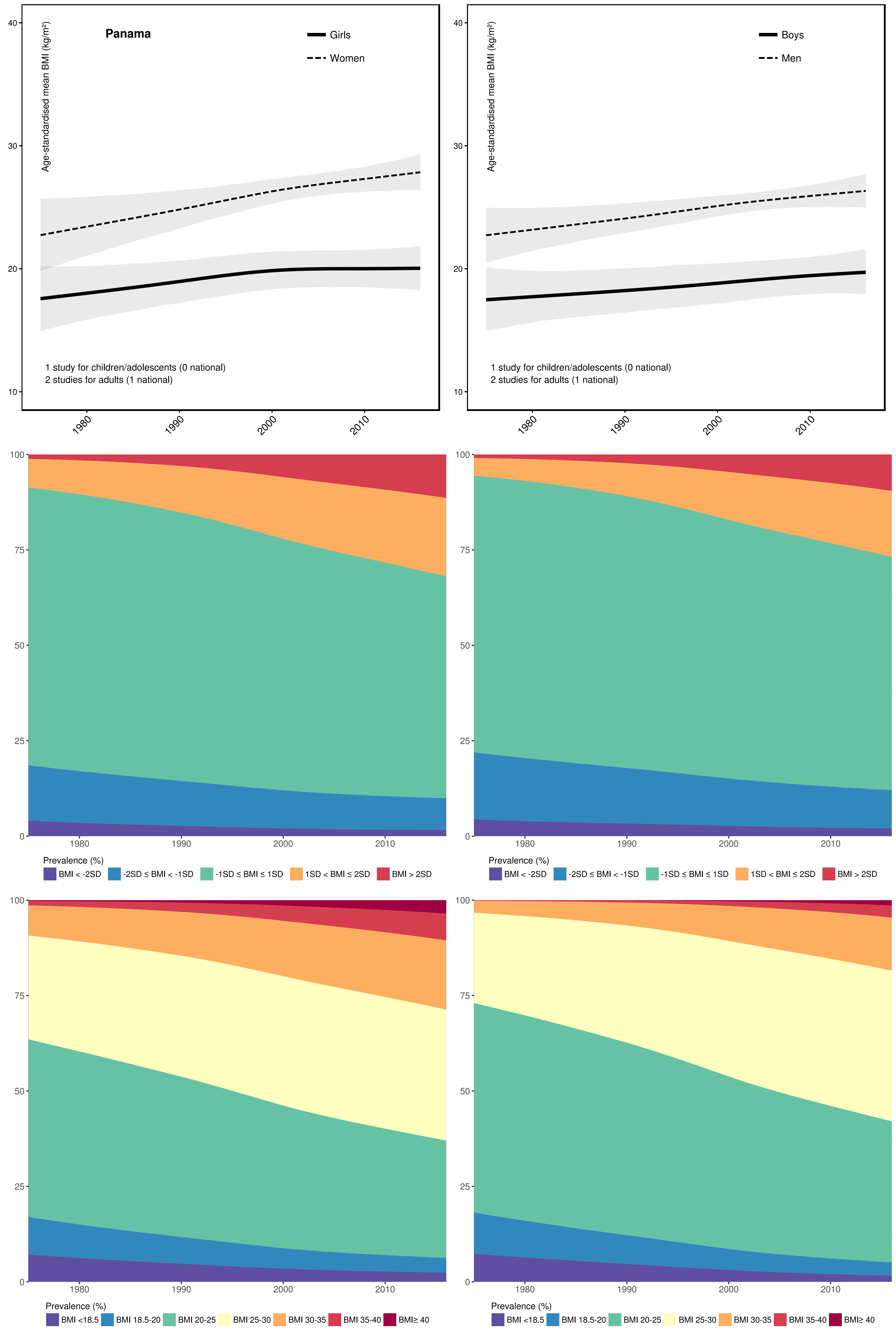
Panama
Girls
Women
Boys
Men
Age-standardised mean BMI (kg/m²)
1 study for children/adolescents (0 national)
2 studies for adults (1 national)
Prevalence (%)
BMI < -2SD
-2SD ≤ BMI < -1SD
-1SD ≤ BMI ≤ 1SD
1SD < BMI ≤ 2SD
BMI > 2SD
BMI <18.5
BMI 18.5-20
BMI 20-25
BMI 25-30
BMI 30-35
BMI 35-40
BMI≥ 40

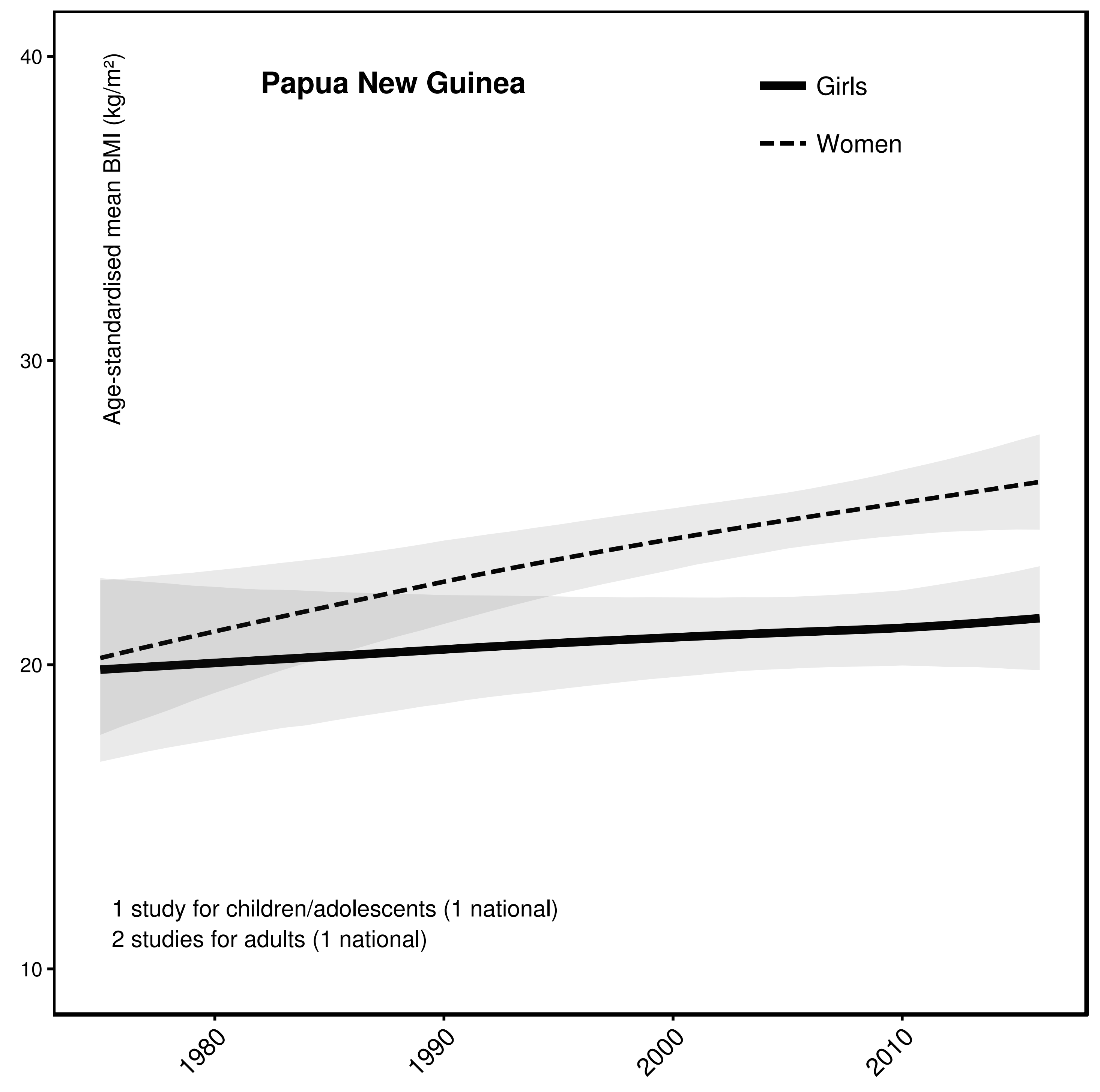
Papua New Guinea
Girls
Women
Age-standardised mean BMI (kg/m²)
1 study for children/adolescents (1 national)
2 studies for adults (1 national)

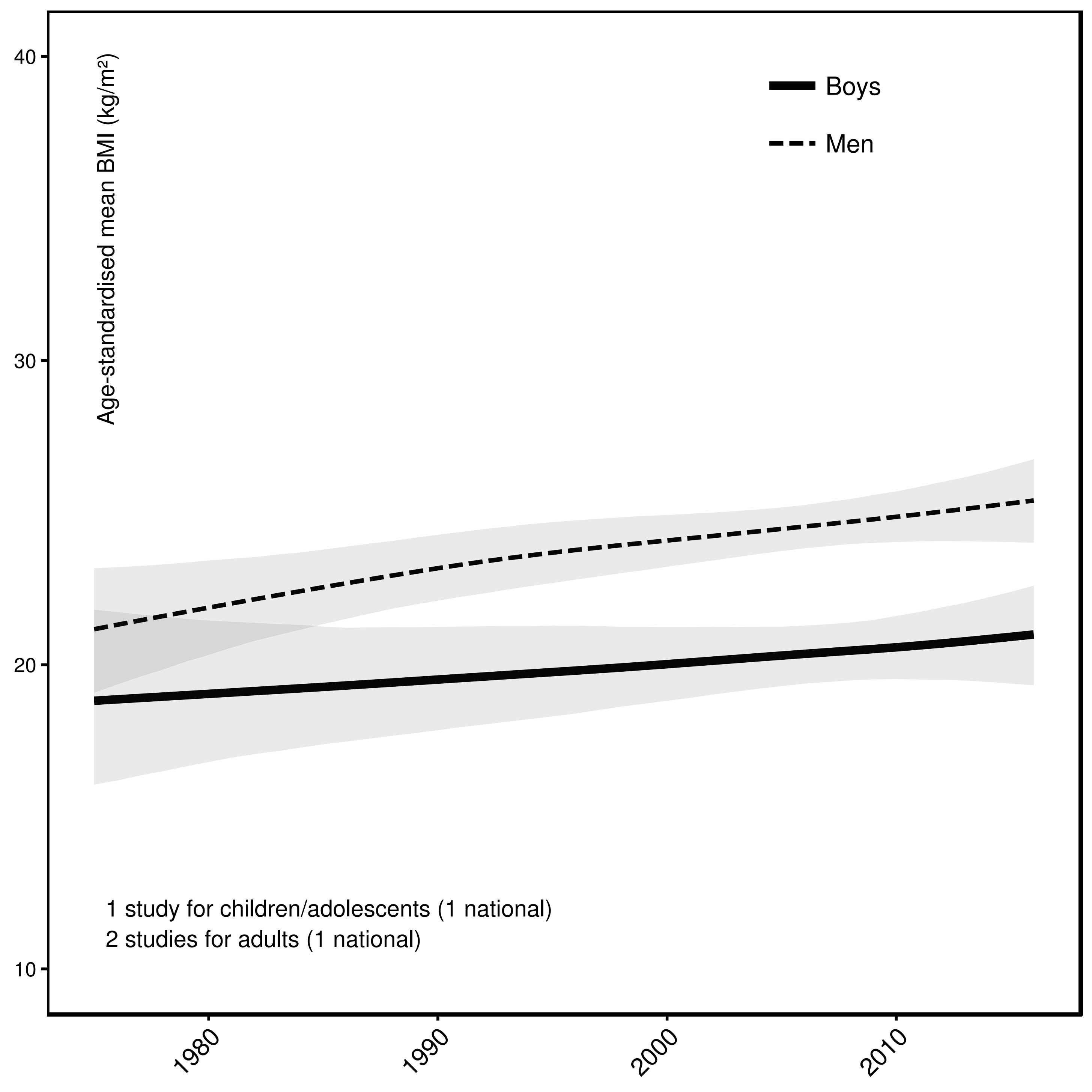
Boys
Men
Age-standardised mean BMI (kg/m²)
1 study for children/adolescents (1 national)
2 studies for adults (1 national)

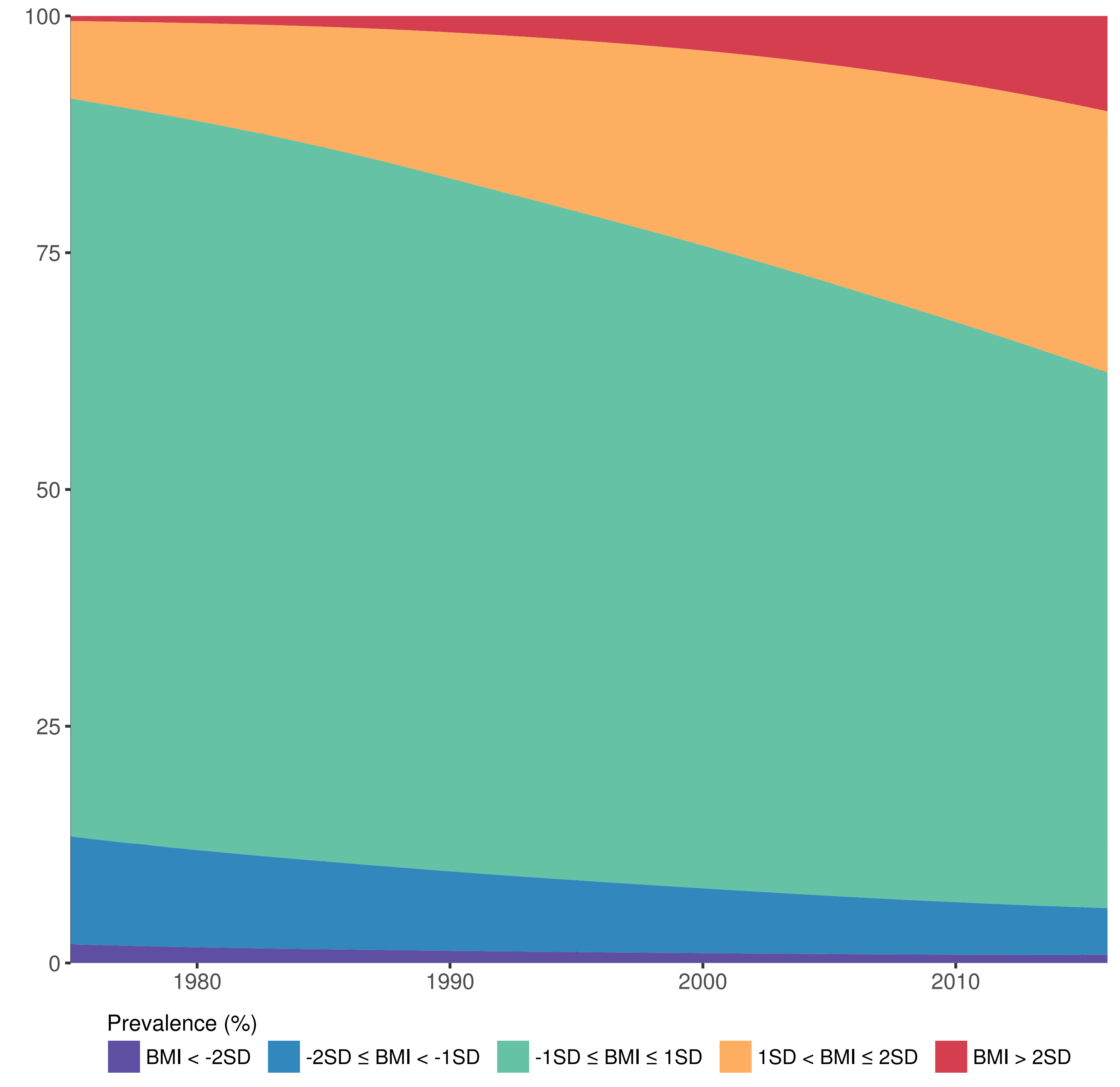
Prevalence (%)
BMI < -2SD
-2SD ≤ BMI < -1SD
-1SD ≤ BMI ≤ 1SD
1SD < BMI ≤ 2SD
BMI > 2SD

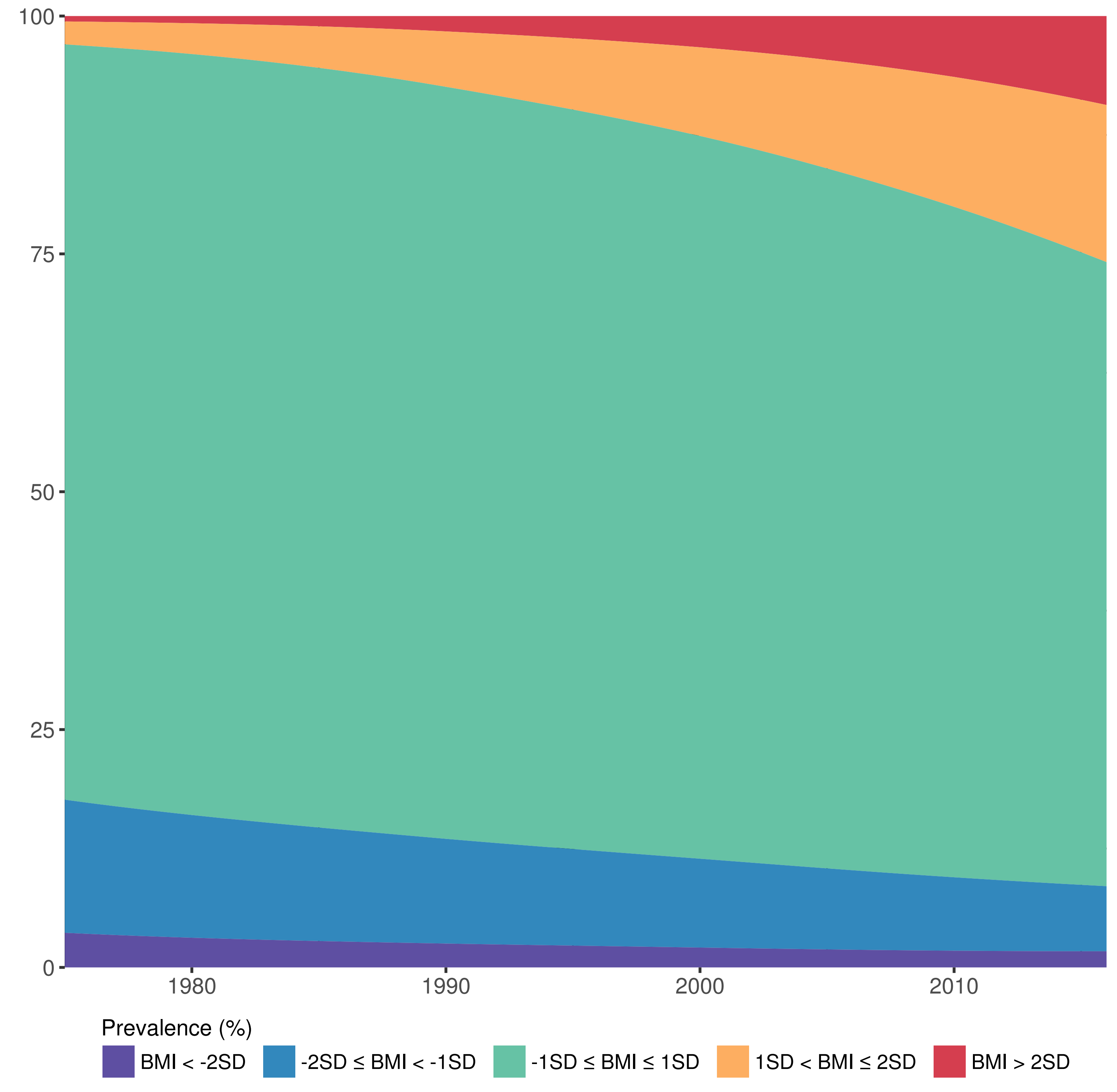
Prevalence (%)
BMI < -2SD
-2SD ≤ BMI < -1SD
-1SD ≤ BMI ≤ 1SD
1SD < BMI ≤ 2SD
BMI > 2SD

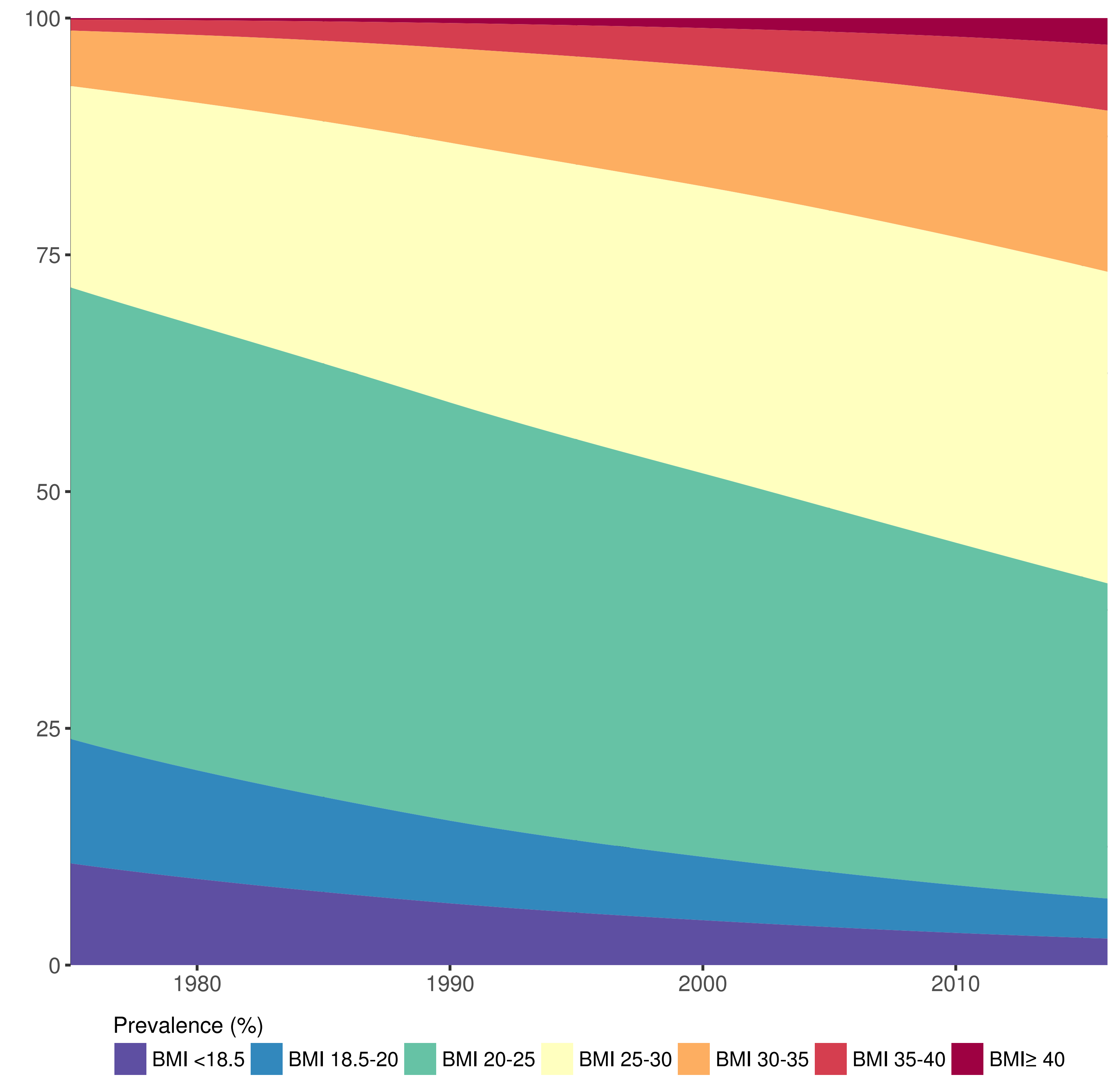
Prevalence (%)
BMI <18.5
BMI 18.5-20
BMI 20-25
BMI 25-30
BMI 30-35
BMI 35-40
BMI≥ 40

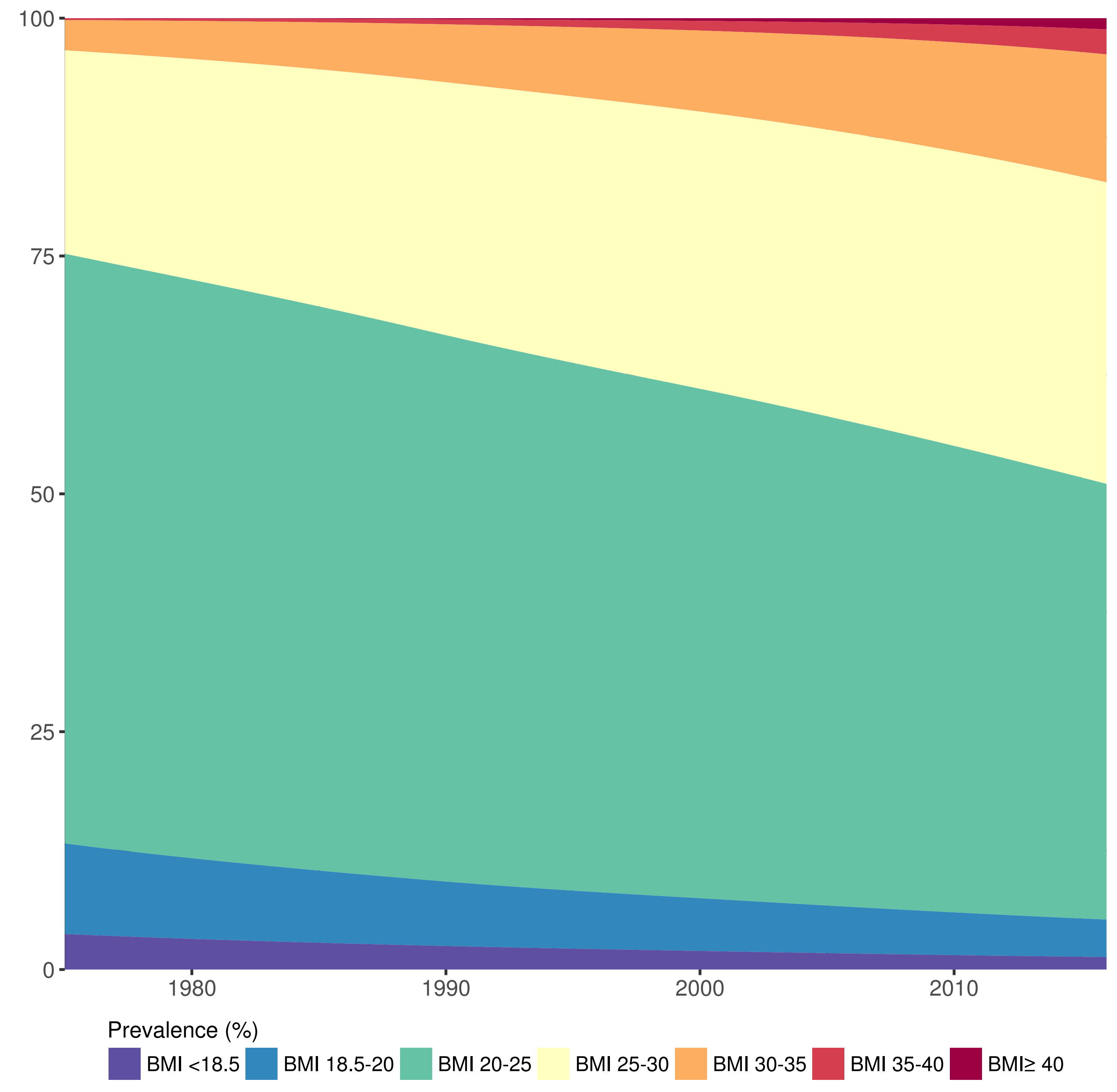
Prevalence (%)
BMI <18.5
BMI 18.5-20
BMI 20-25
BMI 25-30
BMI 30-35
BMI 35-40
BMI≥ 40

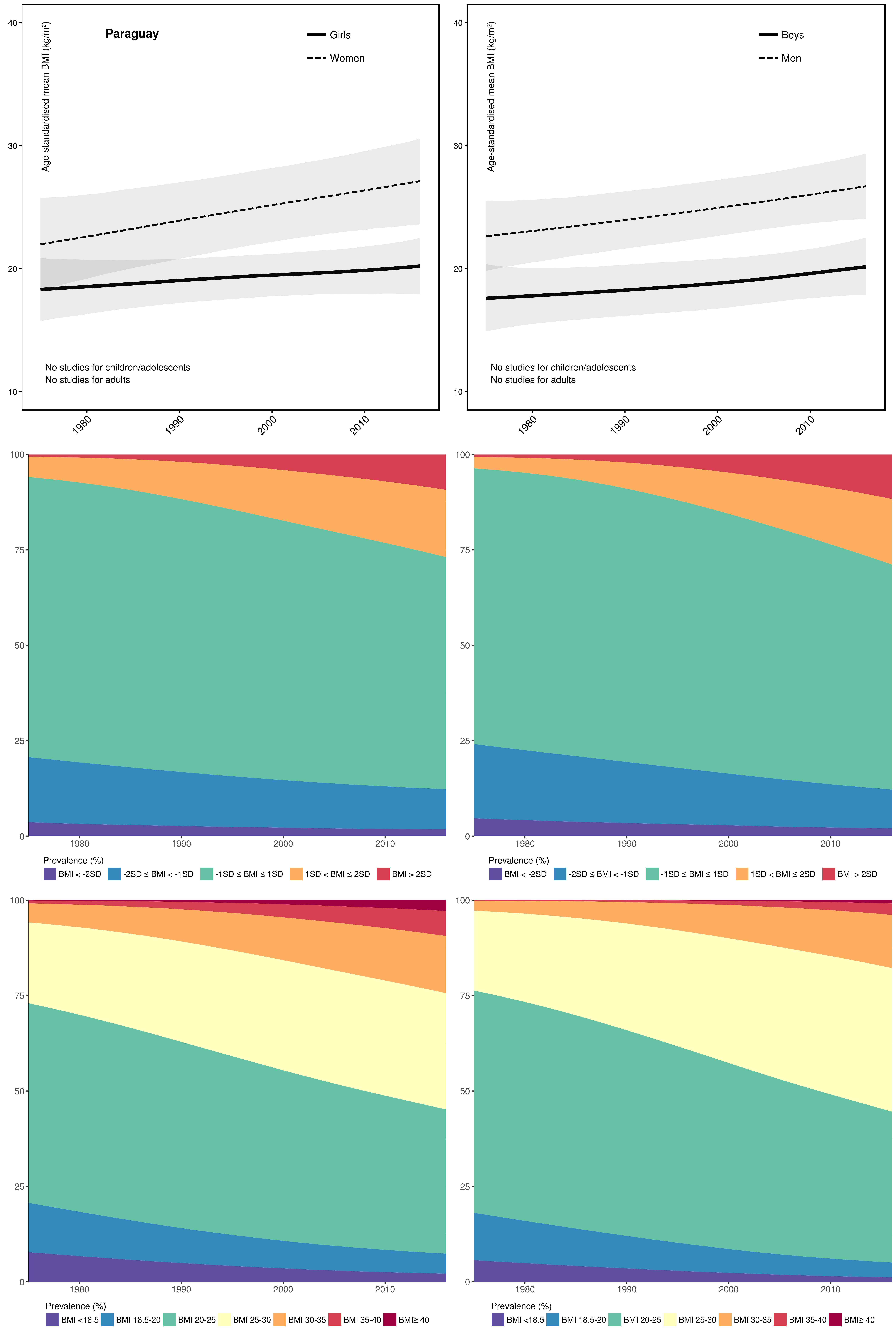
Paraguay
Girls
Women
Boys
Men
Age-standardised mean BMI (kg/m²)
No studies for children/adolescents
No studies for adults
Prevalence (%)
BMI < -2SD
-2SD ≤ BMI < -1SD
-1SD ≤ BMI ≤ 1SD
1SD < BMI ≤ 2SD
BMI > 2SD
BMI <18.5
BMI 18.5-20
BMI 20-25
BMI 25-30
BMI 30-35
BMI 35-40
BMI≥ 40

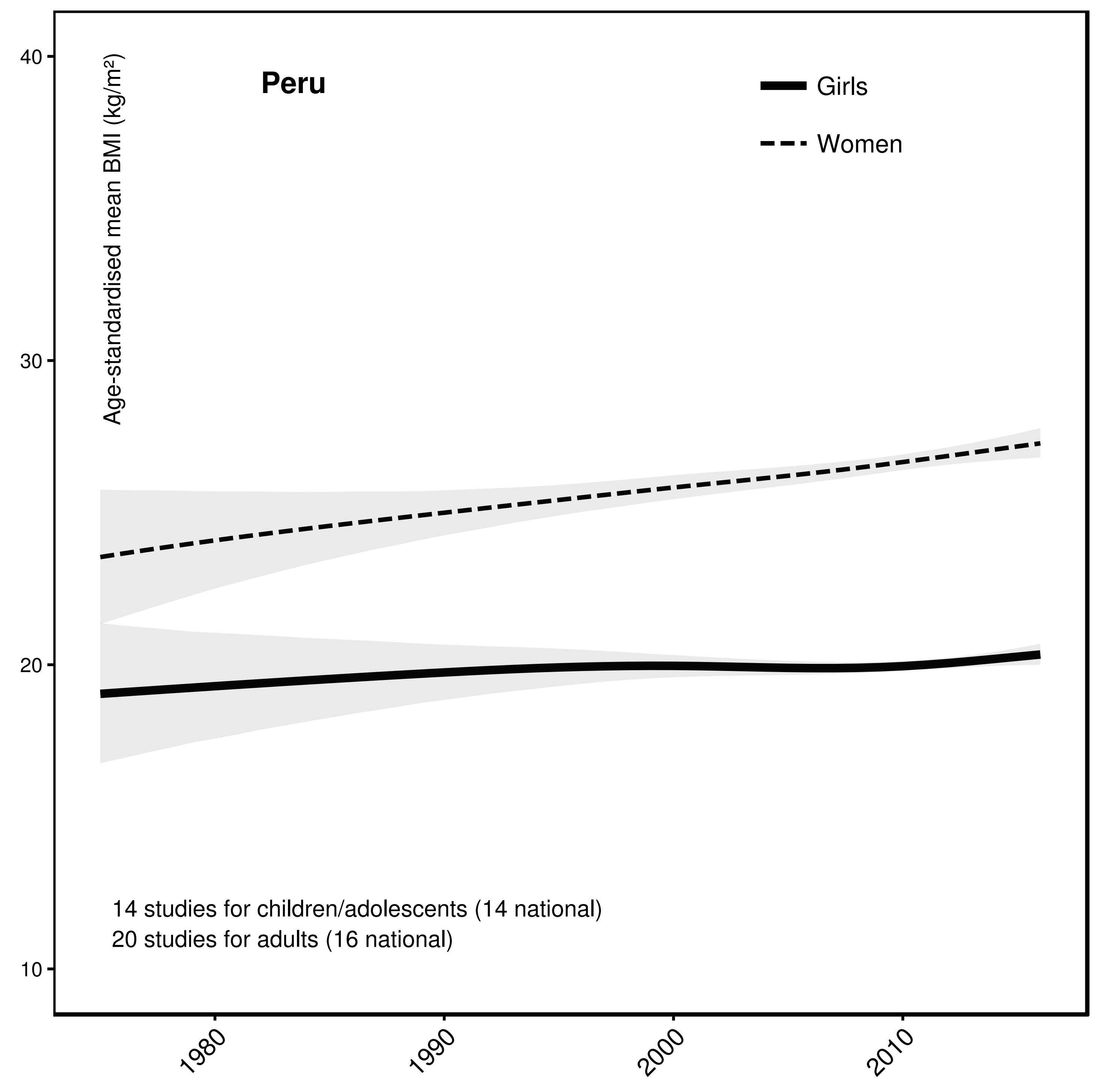
Peru
Girls
Women
Age-standardised mean BMI (kg/m²)
40
30
20
10
14 studies for children/adolescents (14 national)
20 studies for adults (16 national)
1980
1990
2000
2010

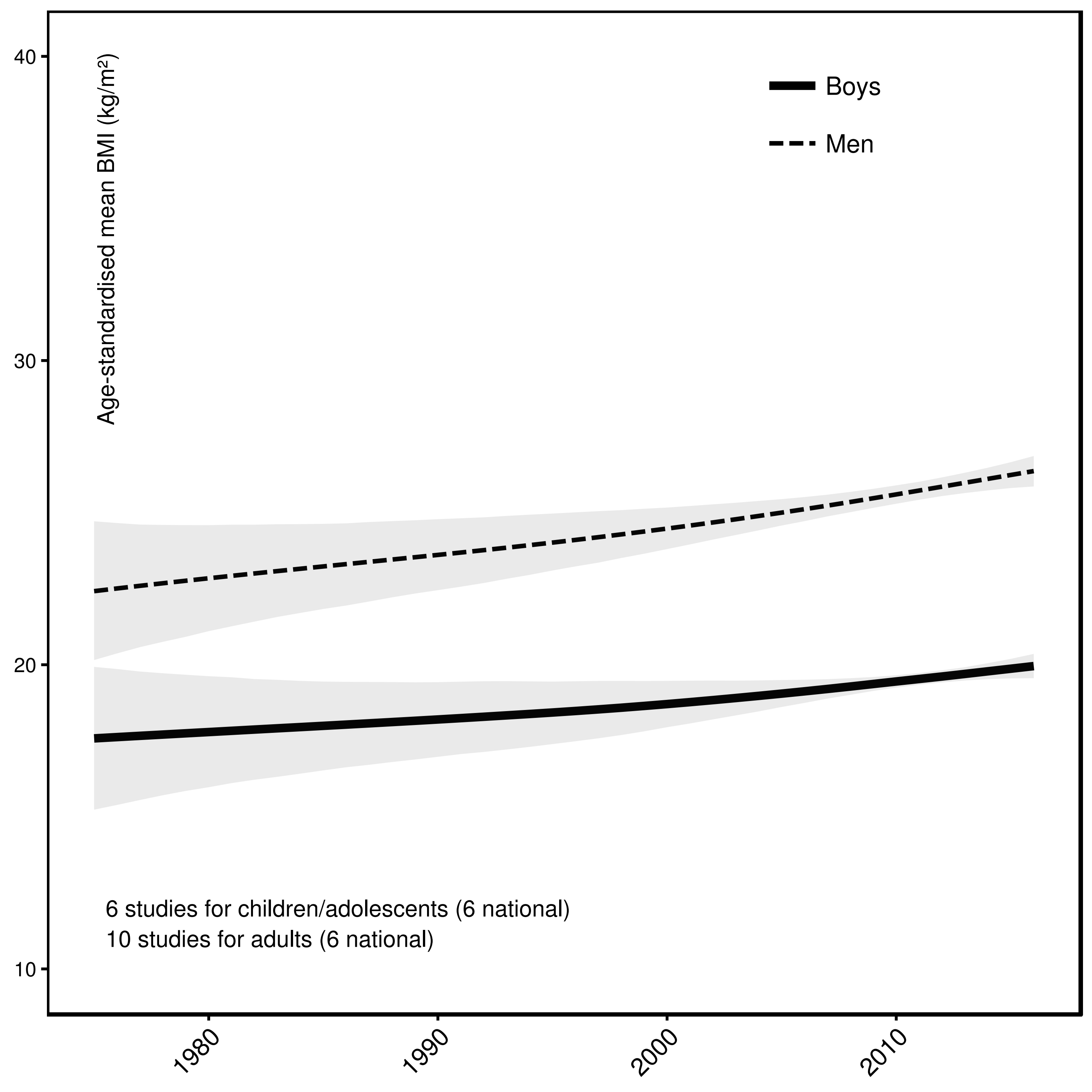
Boys
Men
Age-standardised mean BMI (kg/m²)
40
30
20
10
6 studies for children/adolescents (6 national)
10 studies for adults (6 national)
1980
1990
2000
2010

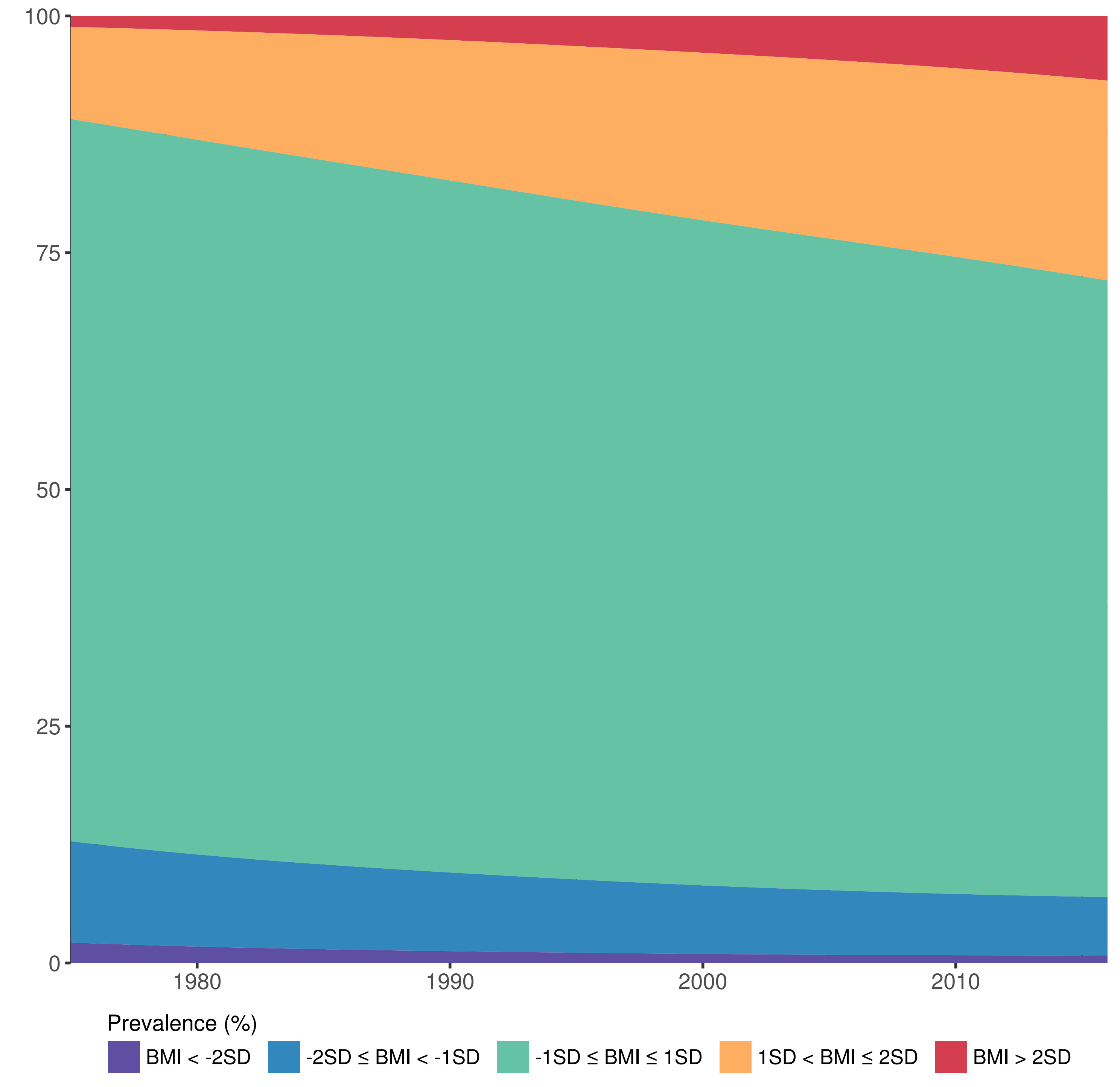
100
75
50
25
0
1980
1990
2000
2010
Prevalence (%)
BMI < -2SD
-2SD ≤ BMI < -1SD
-1SD ≤ BMI ≤ 1SD
1SD < BMI ≤ 2SD
BMI > 2SD

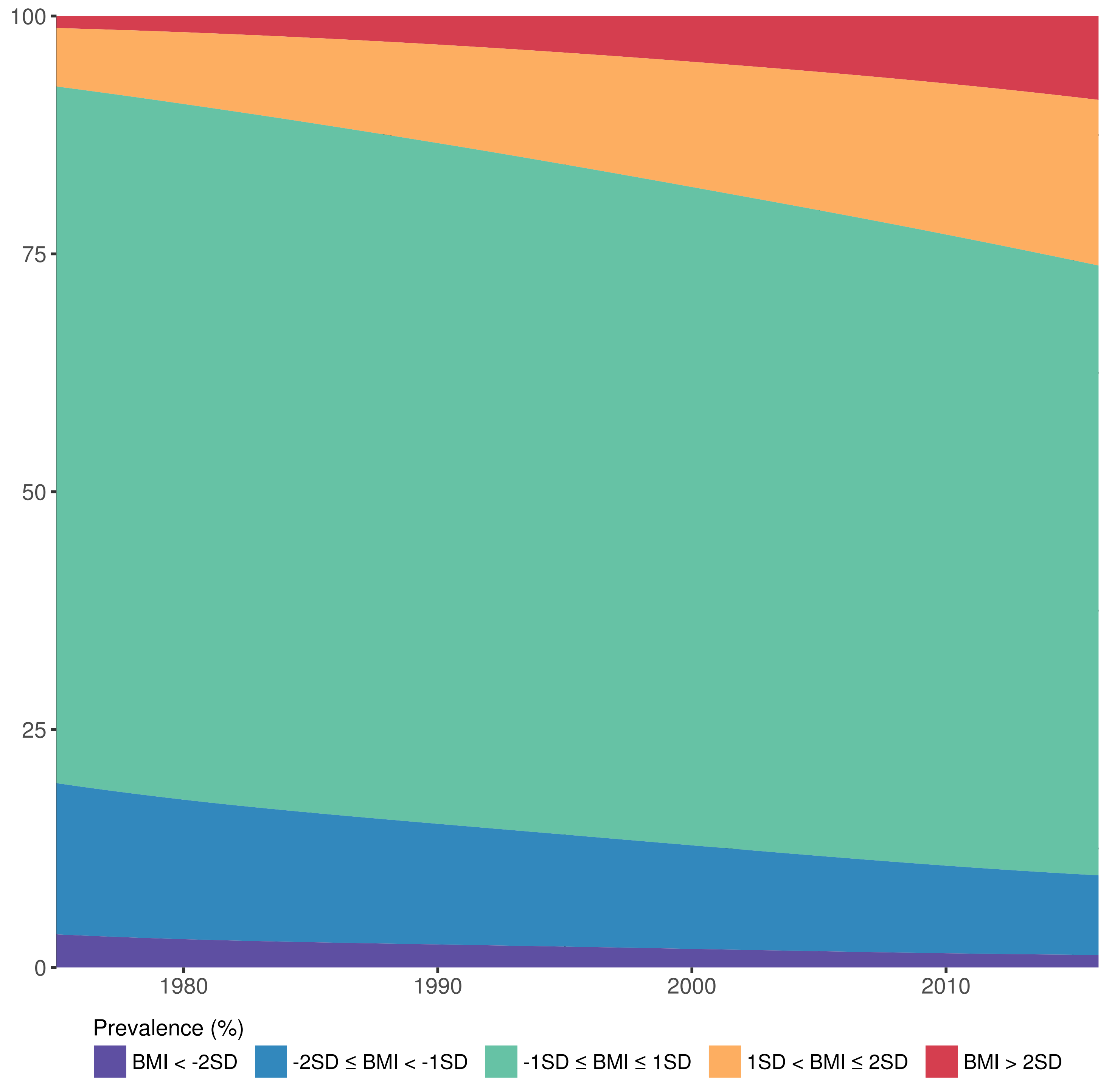
100
75
50
25
0
1980
1990
2000
2010
Prevalence (%)
BMI < -2SD
-2SD ≤ BMI < -1SD
-1SD ≤ BMI ≤ 1SD
1SD < BMI ≤ 2SD
BMI > 2SD

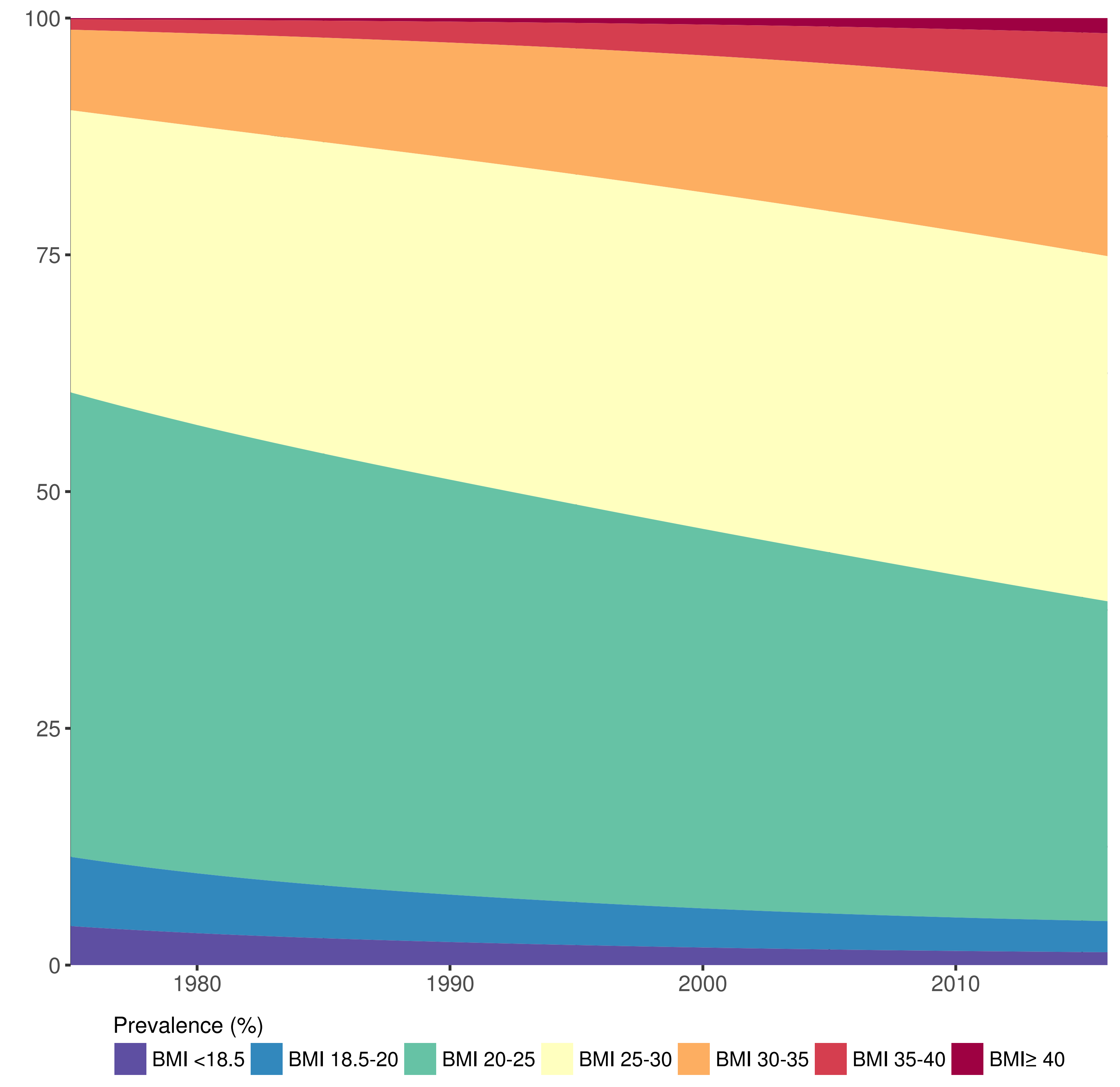
100
75
50
25
0
1980
1990
2000
2010
Prevalence (%)
BMI <18.5
BMI 18.5-20
BMI 20-25
BMI 25-30
BMI 30-35
BMI 35-40
BMI≥ 40

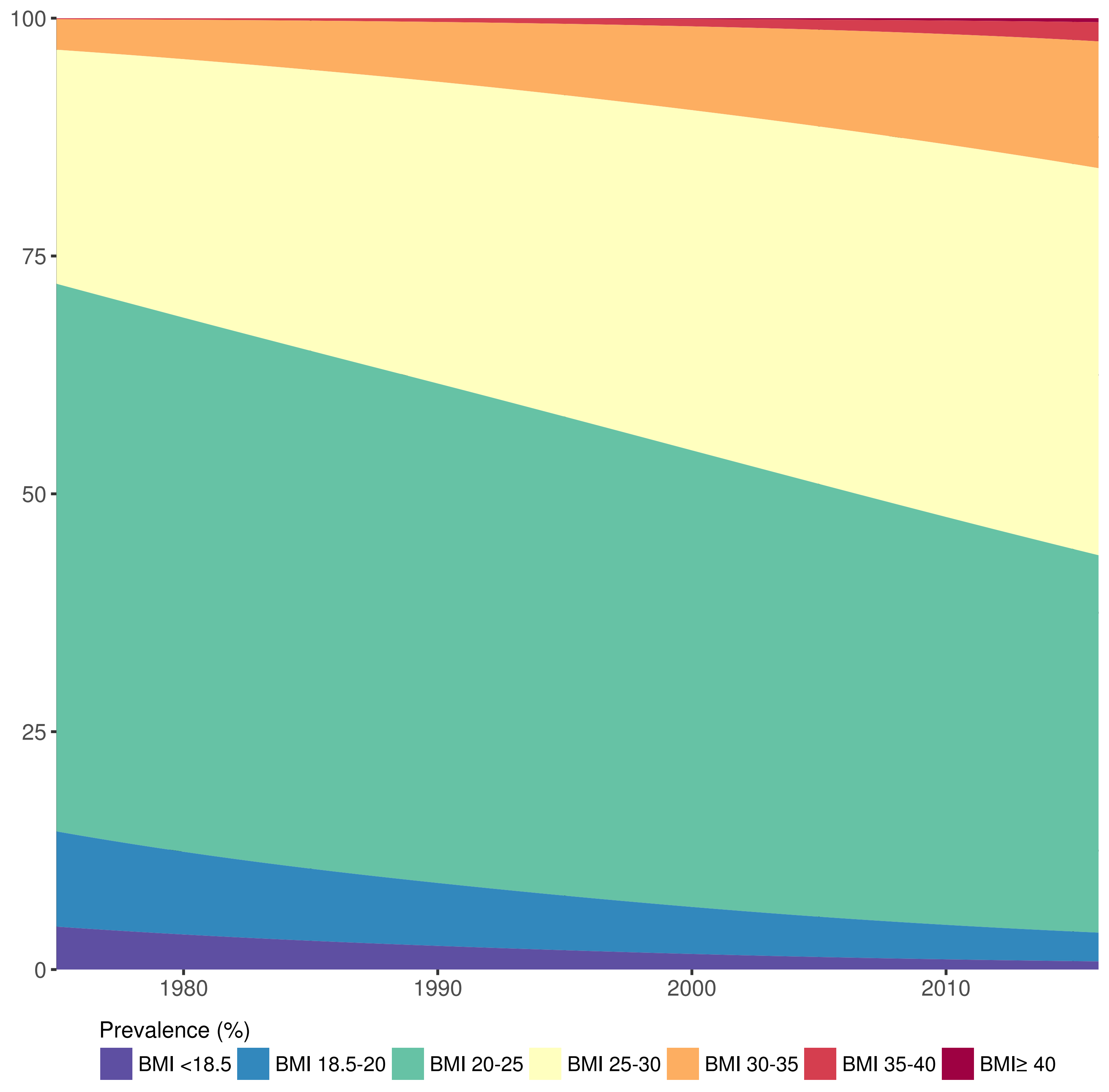
100
75
50
25
0
1980
1990
2000
2010
Prevalence (%)
BMI <18.5
BMI 18.5-20
BMI 20-25
BMI 25-30
BMI 30-35
BMI 35-40
BMI≥ 40

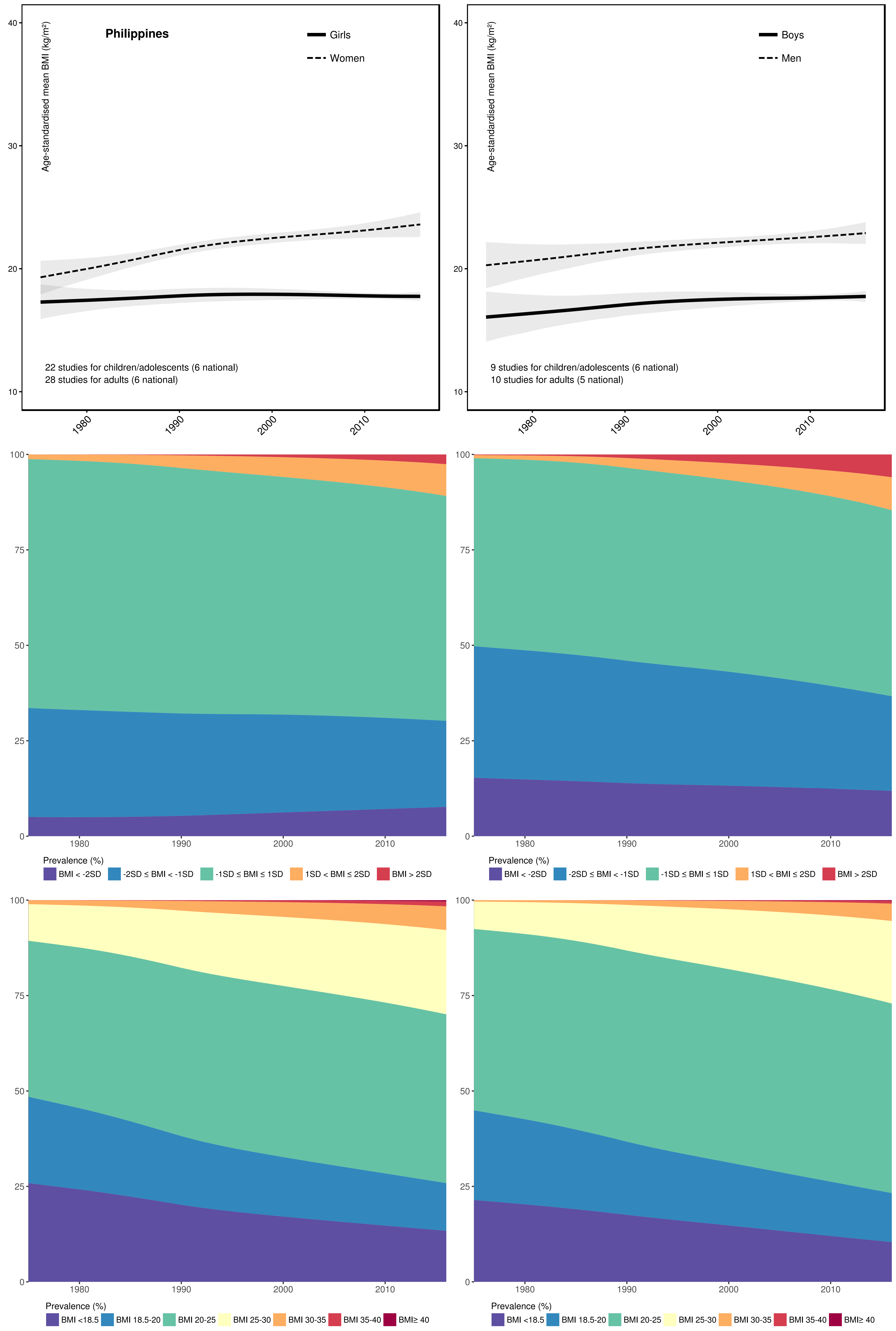
Philippines
Girls
Women
Boys
Men
Age-standardised mean BMI (kg/m²)
22 studies for children/adolescents (6 national)
28 studies for adults (6 national)
9 studies for children/adolescents (6 national)
10 studies for adults (5 national)
1980
1990
2000
2010
Prevalence (%)
BMI < -2SD
-2SD ≤ BMI < -1SD
-1SD ≤ BMI ≤ 1SD
1SD < BMI ≤ 2SD
BMI > 2SD
BMI <18.5
BMI 18.5-20
BMI 20-25
BMI 25-30
BMI 30-35
BMI 35-40
BMI≥ 40

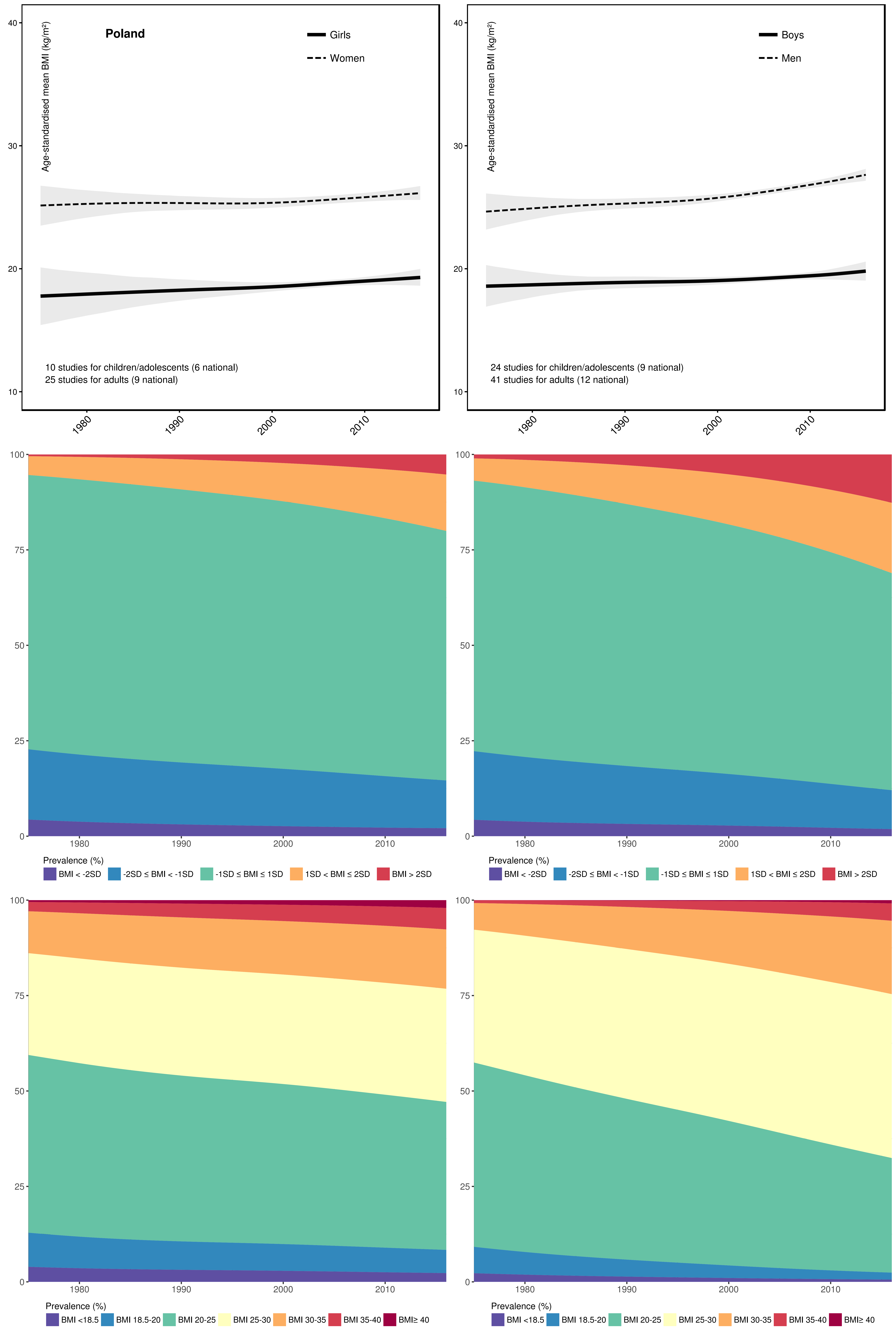
Poland
Girls
Women
Boys
Men
Age-standardised mean BMI (kg/m²)
10 studies for children/adolescents (6 national)
25 studies for adults (9 national)
24 studies for children/adolescents (9 national)
41 studies for adults (12 national)
Prevalence (%)
BMI < -2SD
-2SD ≤ BMI < -1SD
-1SD ≤ BMI ≤ 1SD
1SD < BMI ≤ 2SD
BMI > 2SD
BMI <18.5
BMI 18.5-20
BMI 20-25
BMI 25-30
BMI 30-35
BMI 35-40
BMI≥ 40

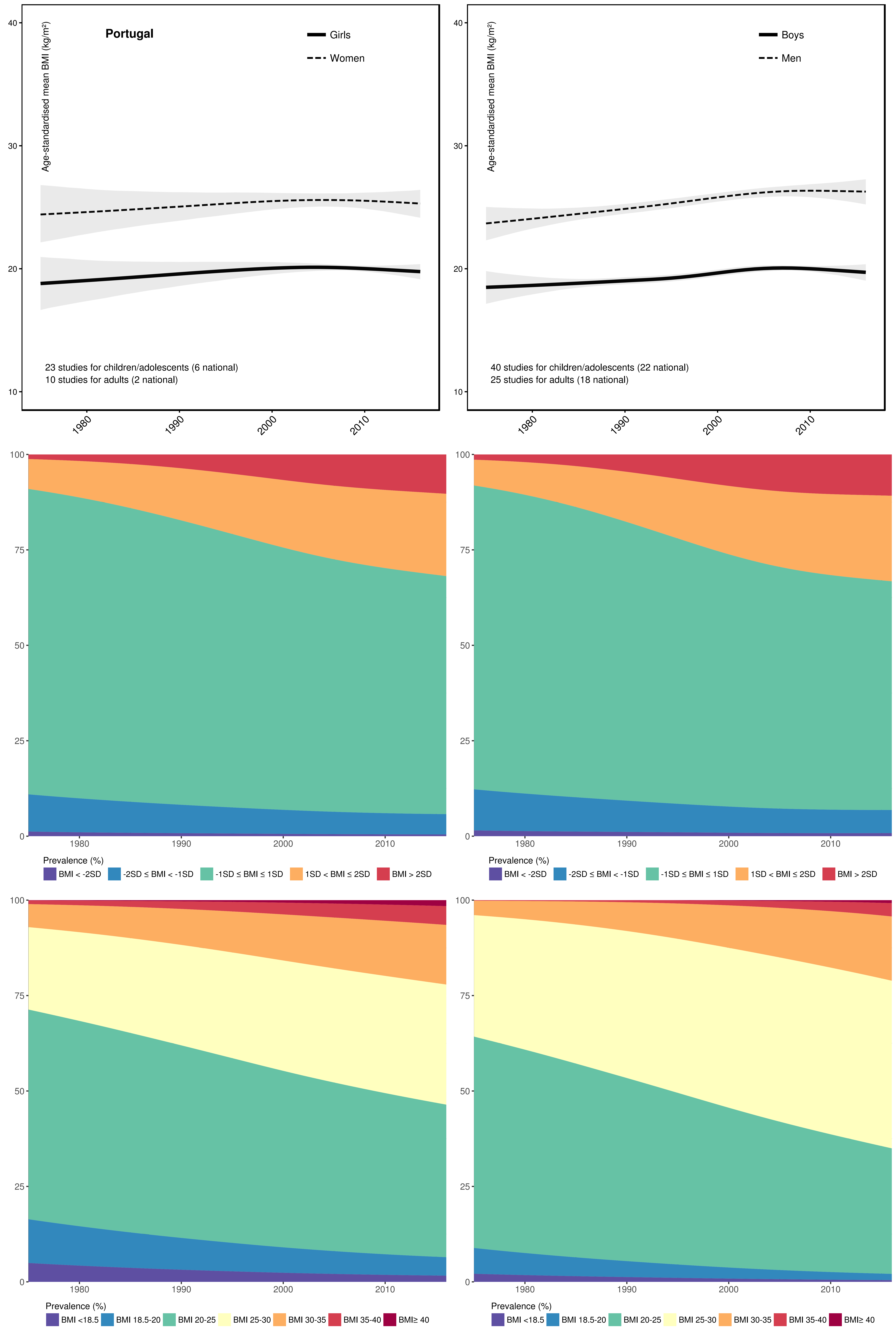
Portugal
Girls
Women
Boys
Men
Age-standardised mean BMI (kg/m²)
23 studies for children/adolescents (6 national)
10 studies for adults (2 national)
40 studies for children/adolescents (22 national)
25 studies for adults (18 national)
Prevalence (%)
BMI < -2SD
-2SD ≤ BMI < -1SD
-1SD ≤ BMI ≤ 1SD
1SD < BMI ≤ 2SD
BMI > 2SD
BMI <18.5
BMI 18.5-20
BMI 20-25
BMI 25-30
BMI 30-35
BMI 35-40
BMI≥ 40

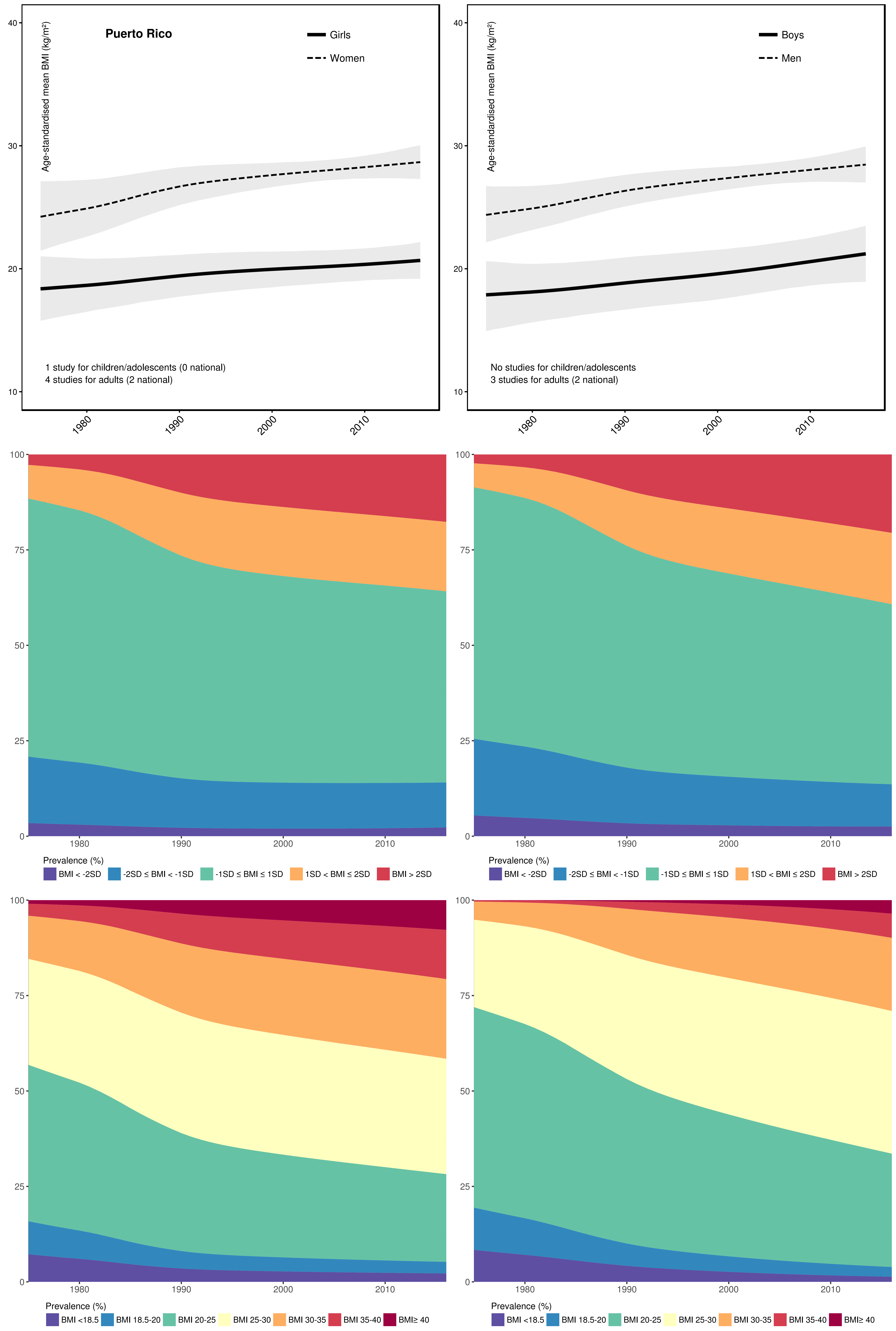
Puerto Rico
Girls
Women
Boys
Men
Age-standardised mean BMI (kg/m²)
40
30
20
10
1980
1990
2000
2010
1 study for children/adolescents (0 national)
4 studies for adults (2 national)
No studies for children/adolescents
3 studies for adults (2 national)
100
75
50
25
0
Prevalence (%)
BMI < -2SD
-2SD ≤ BMI < -1SD
-1SD ≤ BMI ≤ 1SD
1SD < BMI ≤ 2SD
BMI > 2SD
BMI <18.5
BMI 18.5-20
BMI 20-25
BMI 25-30
BMI 30-35
BMI 35-40
BMI≥ 40

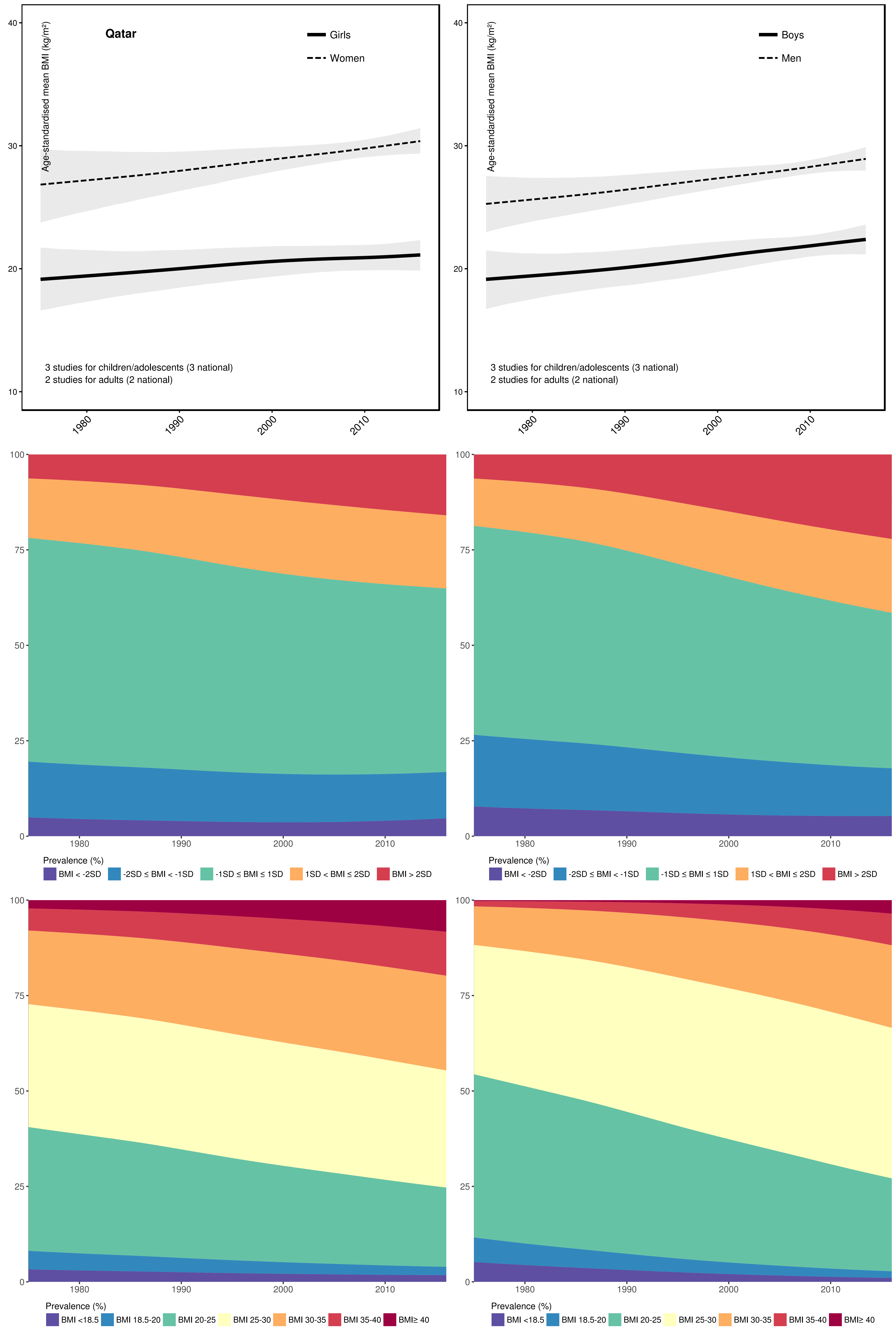
Qatar
Girls
Women
Boys
Men
Age-standardised mean BMI (kg/m²)
3 studies for children/adolescents (3 national)
2 studies for adults (2 national)
Prevalence (%)
BMI < -2SD
-2SD ≤ BMI < -1SD
-1SD ≤ BMI ≤ 1SD
1SD < BMI ≤ 2SD
BMI > 2SD
BMI <18.5
BMI 18.5-20
BMI 20-25
BMI 25-30
BMI 30-35
BMI 35-40
BMI≥ 40

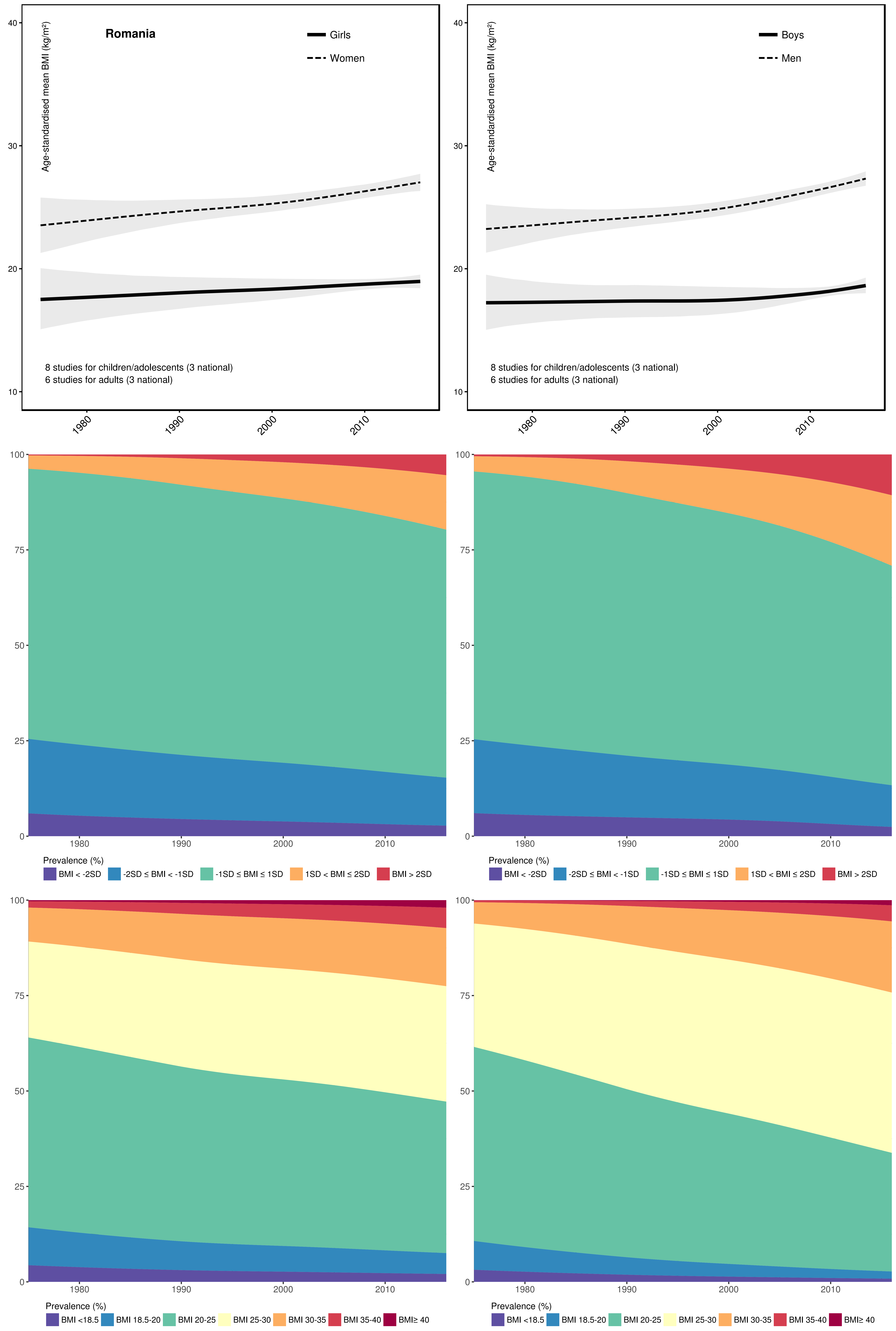
Romania
Girls
Women
Boys
Men
Age-standardised mean BMI (kg/m²)
8 studies for children/adolescents (3 national)
6 studies for adults (3 national)
Prevalence (%)
BMI < -2SD
-2SD ≤ BMI < -1SD
-1SD ≤ BMI ≤ 1SD
1SD < BMI ≤ 2SD
BMI > 2SD
BMI <18.5
BMI 18.5-20
BMI 20-25
BMI 25-30
BMI 30-35
BMI 35-40
BMI≥ 40

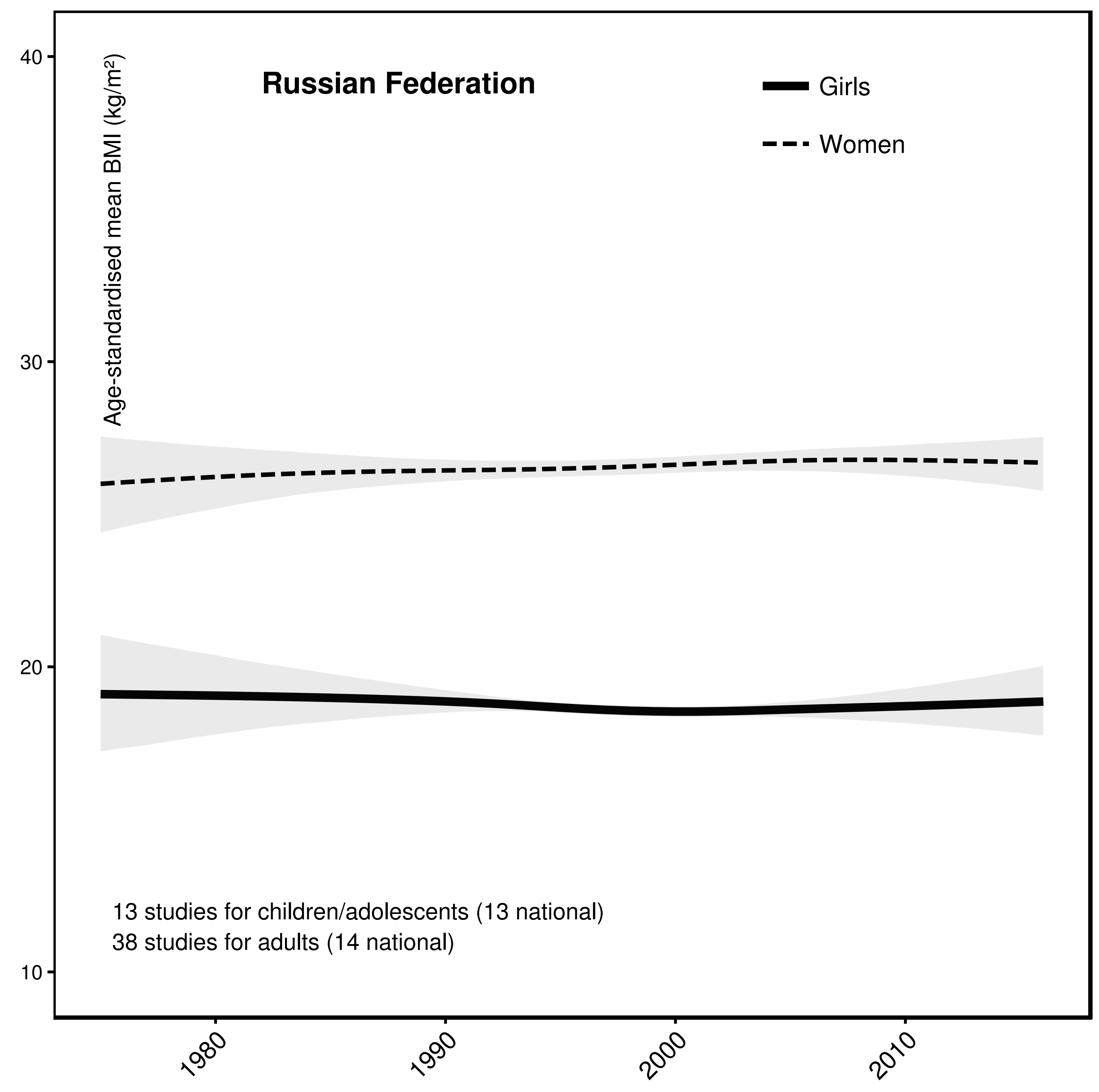
Russian Federation
Girls
Women
Age-standardised mean BMI (kg/m²)
13 studies for children/adolescents (13 national)
38 studies for adults (14 national)

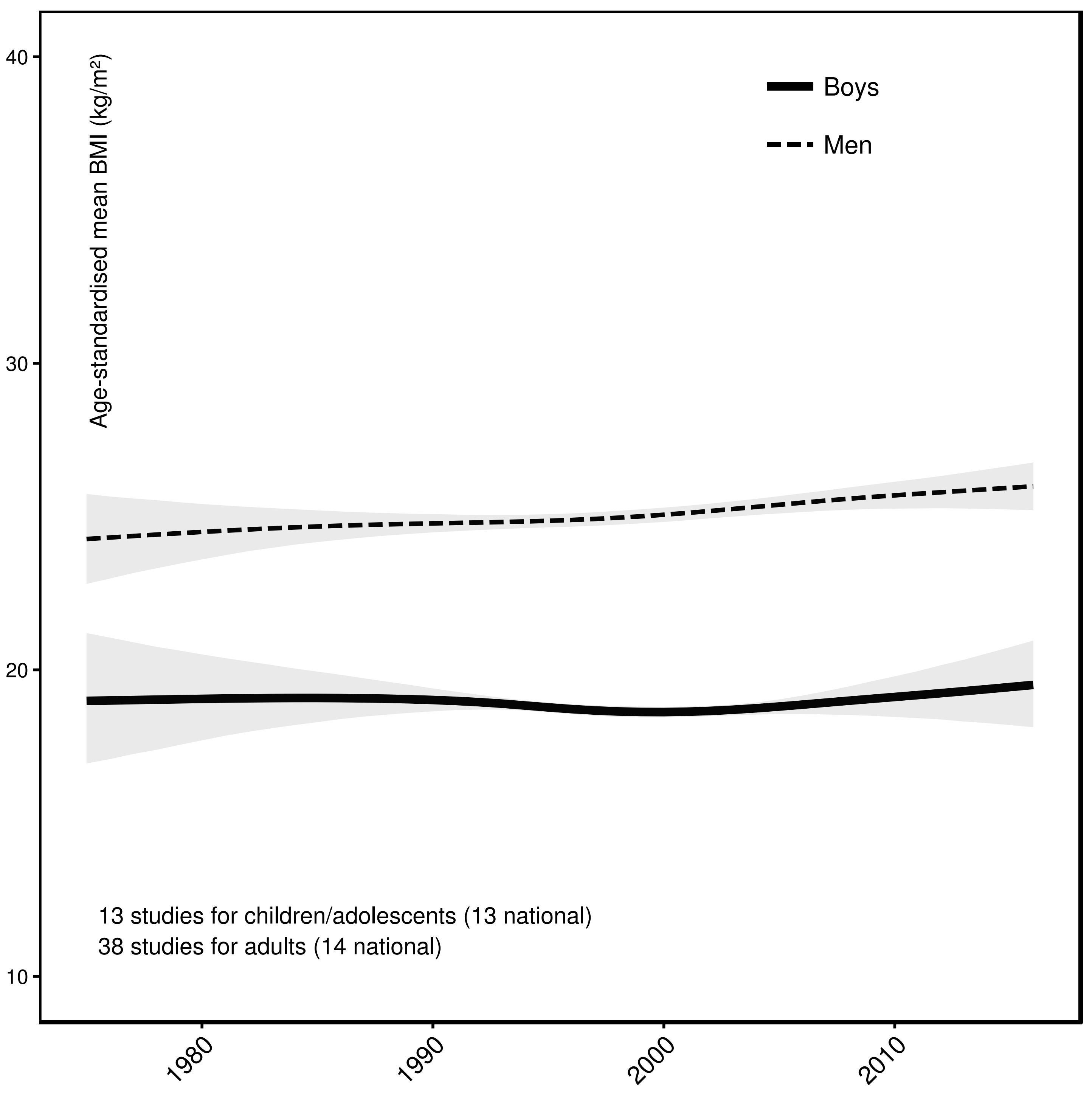
Boys
Men
Age-standardised mean BMI (kg/m²)
13 studies for children/adolescents (13 national)
38 studies for adults (14 national)

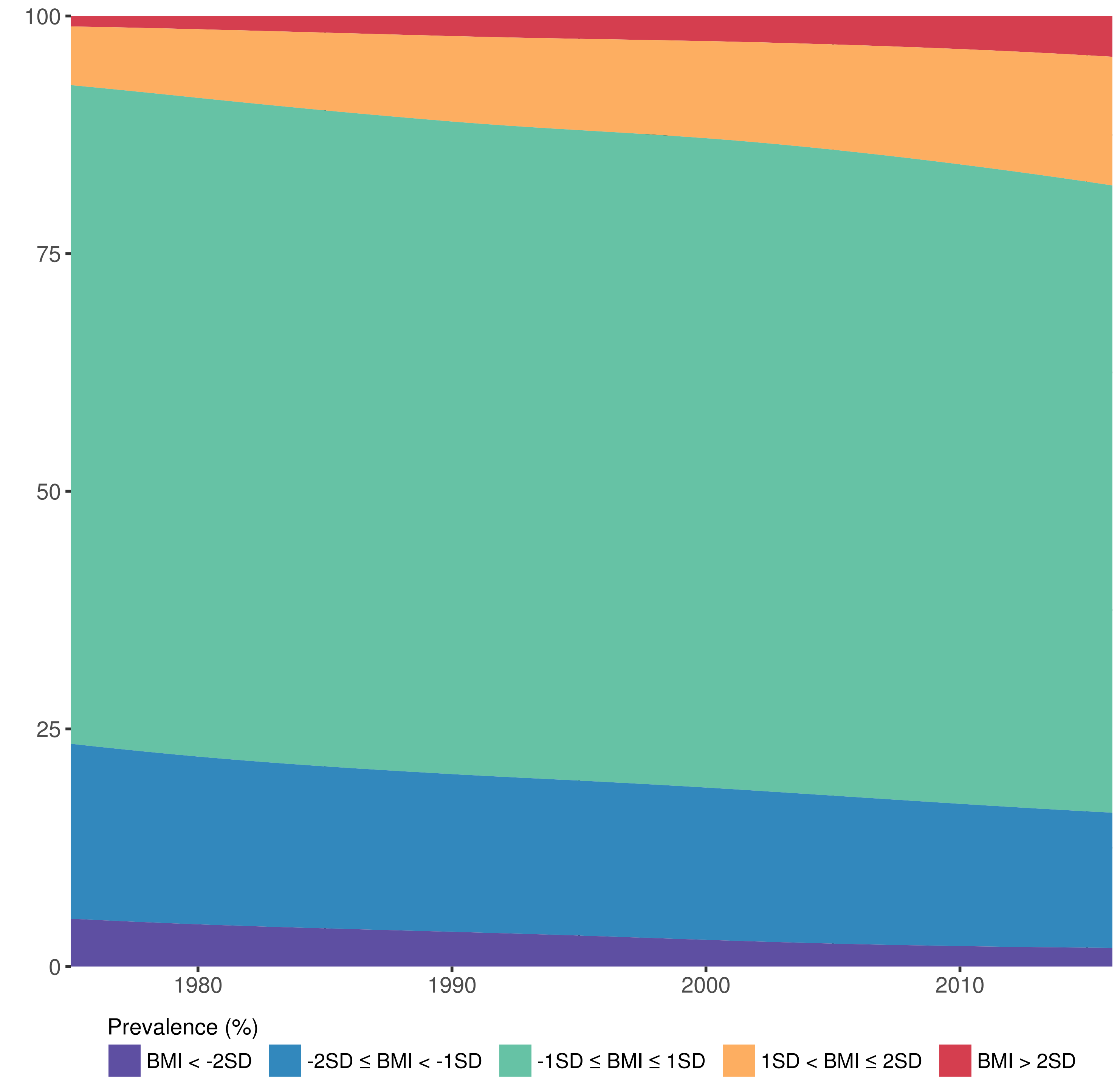
Prevalence (%)
BMI < -2SD
-2SD ≤ BMI < -1SD
-1SD ≤ BMI ≤ 1SD
1SD < BMI ≤ 2SD
BMI > 2SD

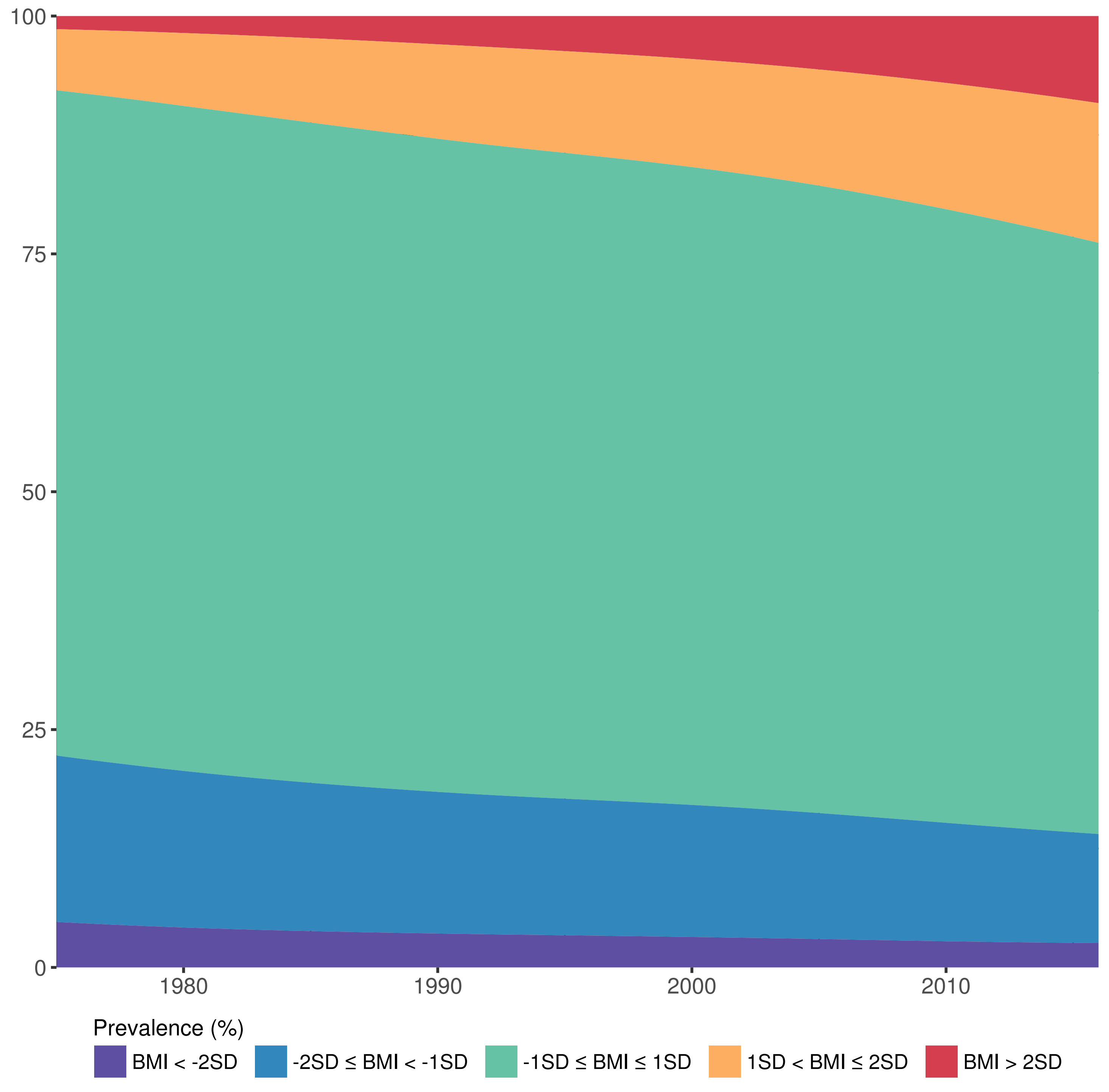
Prevalence (%)
BMI < -2SD
-2SD ≤ BMI < -1SD
-1SD ≤ BMI ≤ 1SD
1SD < BMI ≤ 2SD
BMI > 2SD

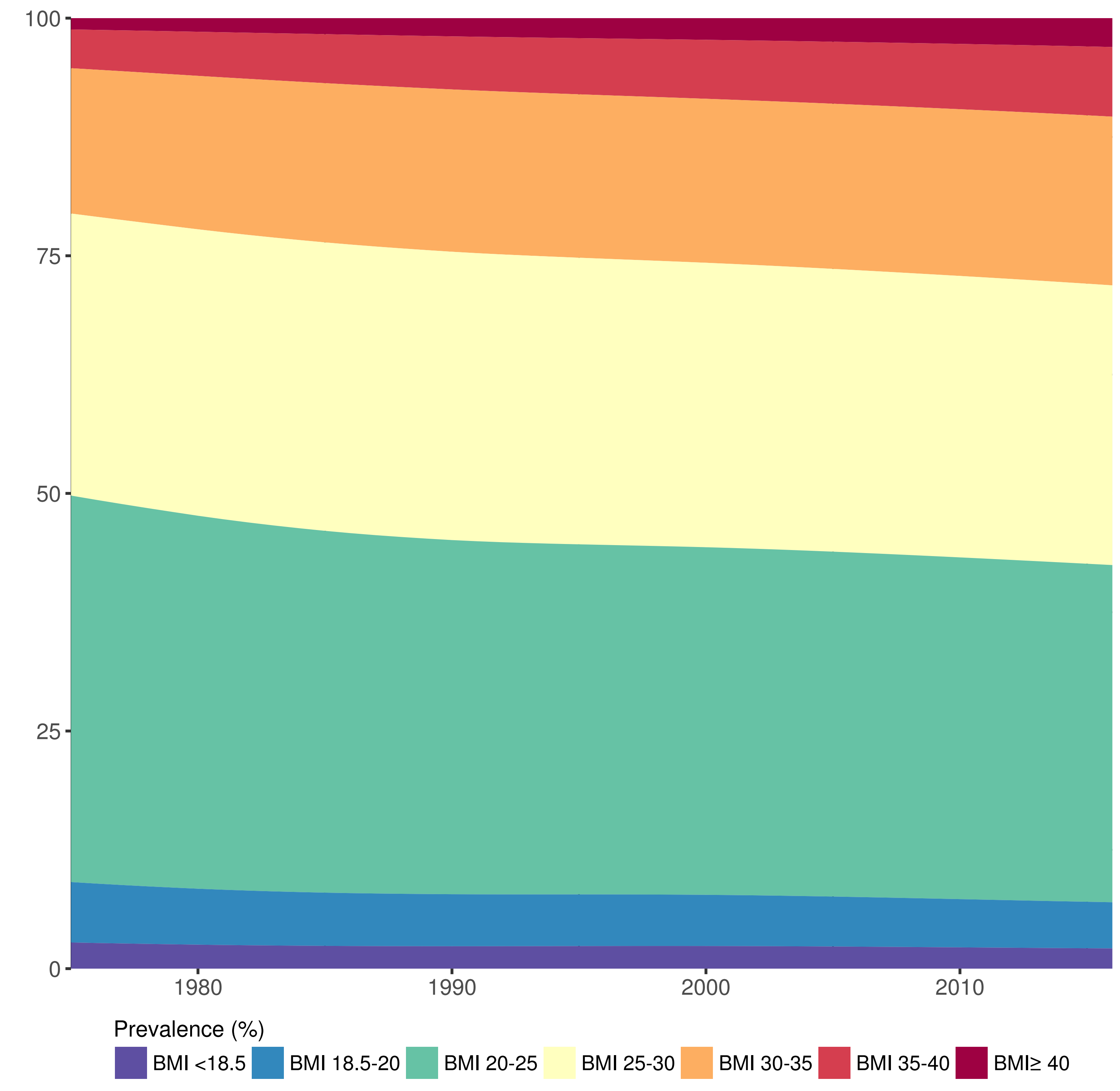
Prevalence (%)
BMI <18.5
BMI 18.5-20
BMI 20-25
BMI 25-30
BMI 30-35
BMI 35-40
BMI≥ 40

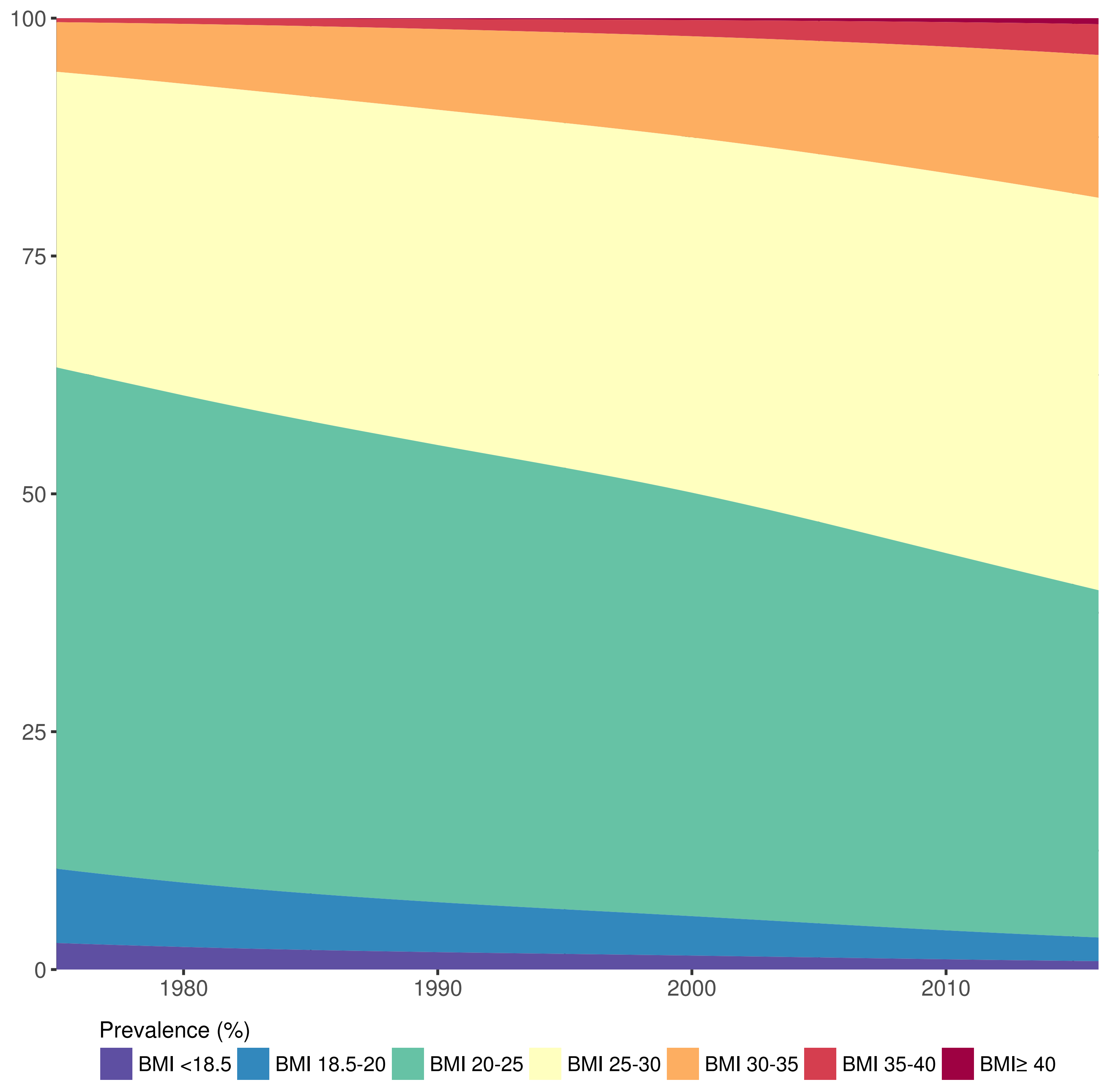
Prevalence (%)
BMI <18.5
BMI 18.5-20
BMI 20-25
BMI 25-30
BMI 30-35
BMI 35-40
BMI≥ 40

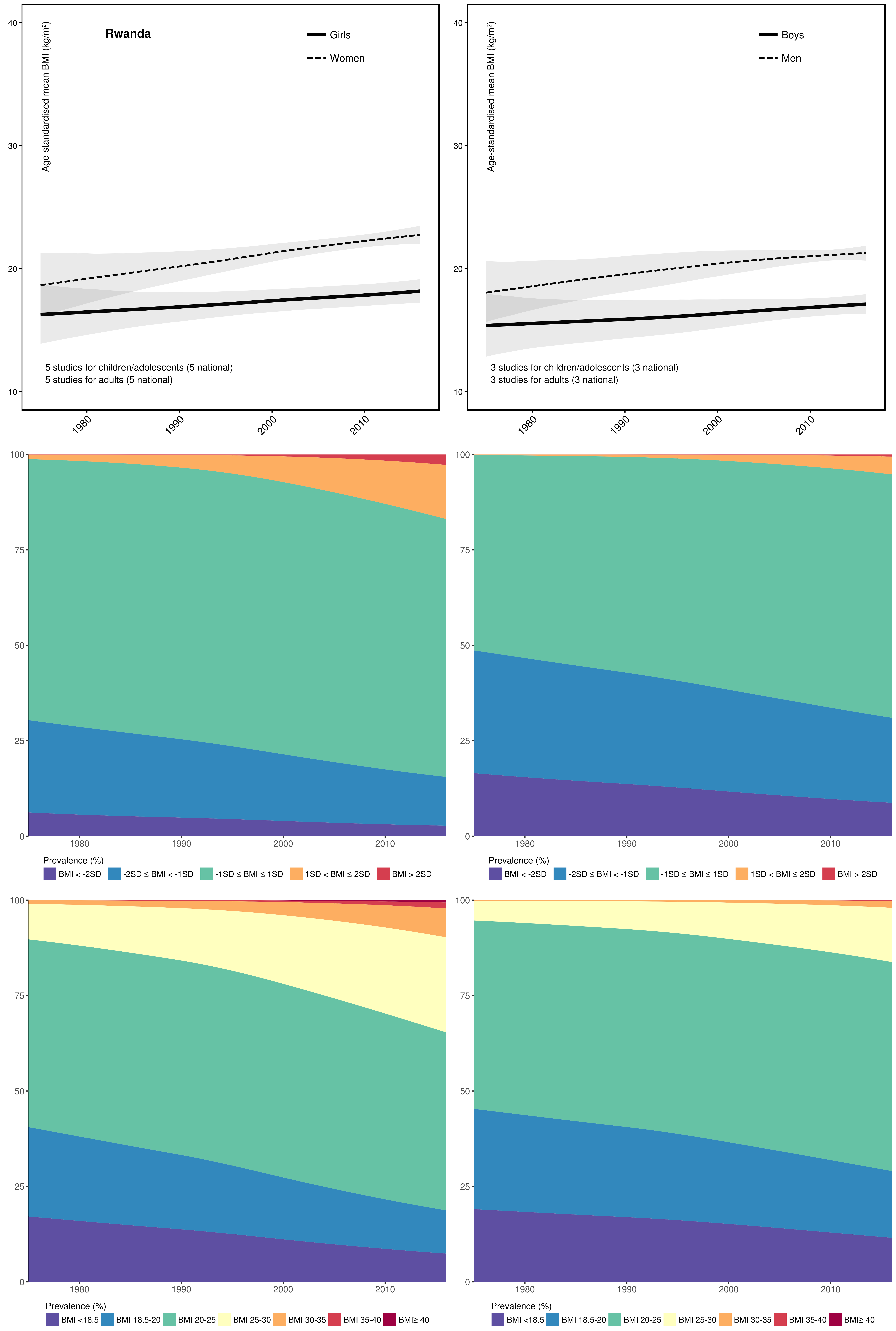
Rwanda
Girls
Women
Boys
Men
Age-standardised mean BMI (kg/m²)
5 studies for children/adolescents (5 national)
5 studies for adults (5 national)
3 studies for children/adolescents (3 national)
3 studies for adults (3 national)
Prevalence (%)
BMI < -2SD
-2SD ≤ BMI < -1SD
-1SD ≤ BMI ≤ 1SD
1SD < BMI ≤ 2SD
BMI > 2SD
BMI <18.5
BMI 18.5-20
BMI 20-25
BMI 25-30
BMI 30-35
BMI 35-40
BMI≥ 40

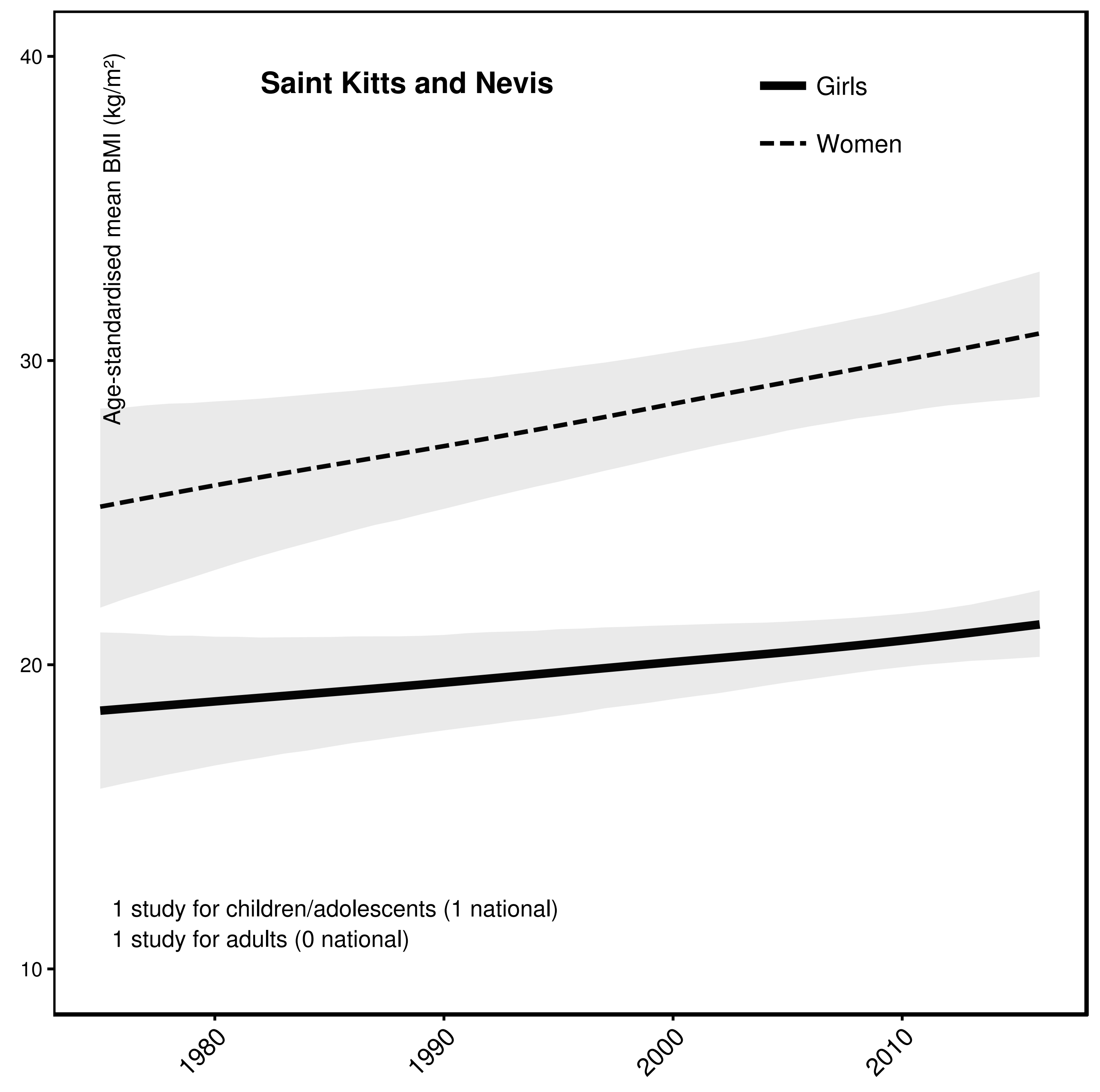
Saint Kitts and Nevis
Girls
Women
Age-standardised mean BMI (kg/m²)
40
30
20
10
1 study for children/adolescents (1 national)
1 study for adults (0 national)
1980
1990
2000
2010
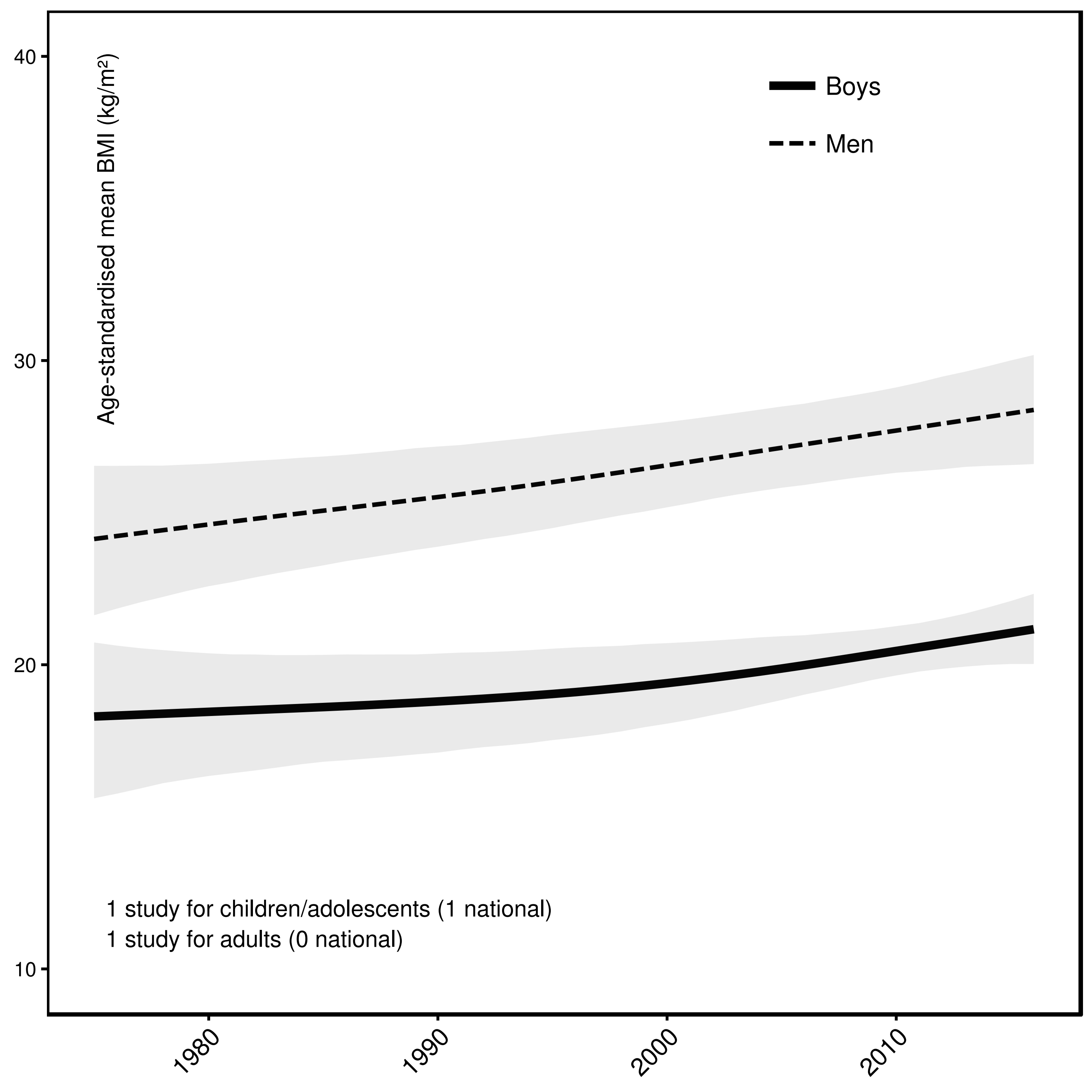
Boys
Men
Age-standardised mean BMI (kg/m²)
40
30
20
10
1 study for children/adolescents (1 national)
1 study for adults (0 national)
1980
1990
2000
2010
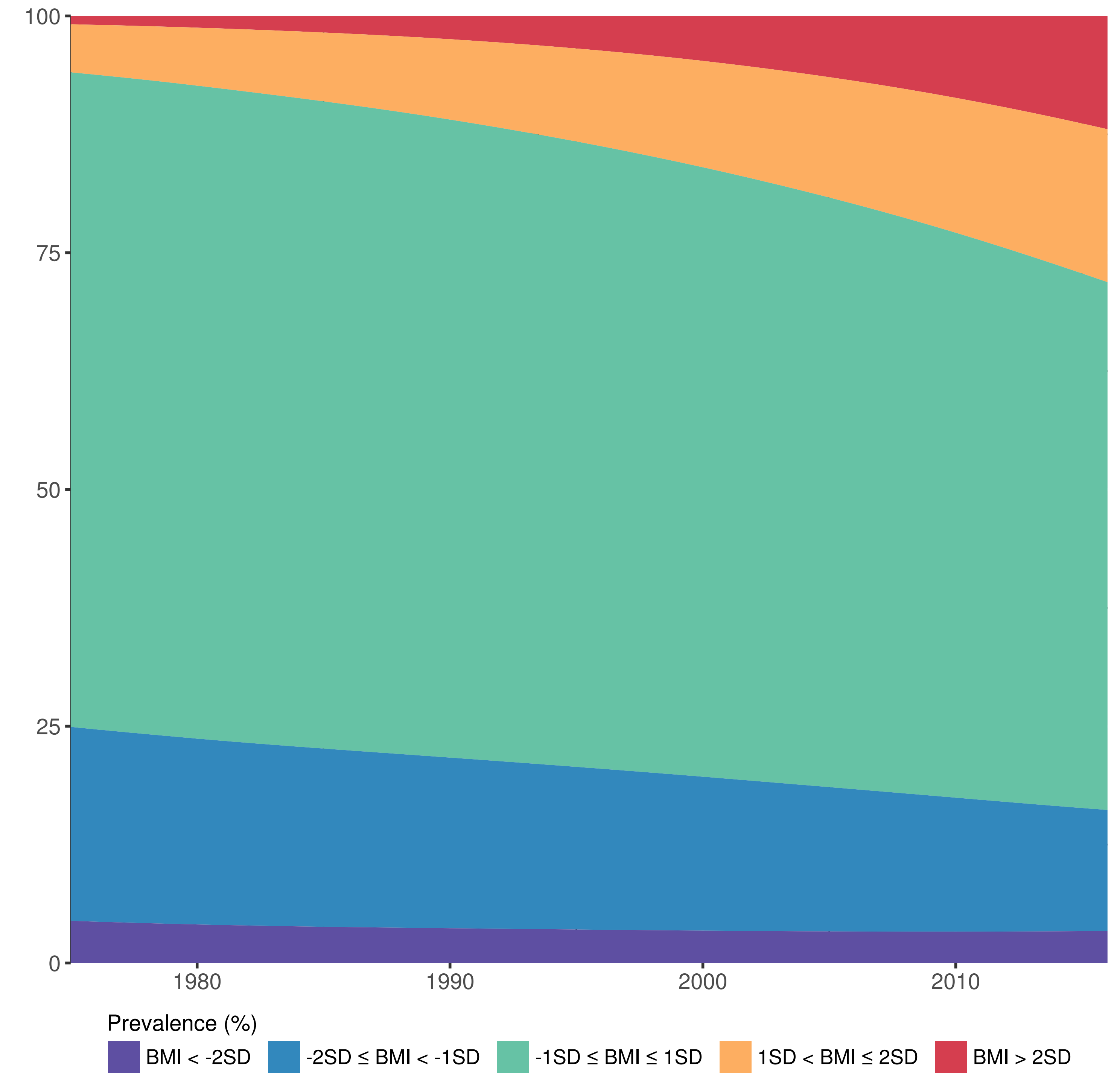
100
75
50
25
0
1980
1990
2000
2010
Prevalence (%)
BMI < -2SD
-2SD ≤ BMI < -1SD
-1SD ≤ BMI ≤ 1SD
1SD < BMI ≤ 2SD
BMI > 2SD
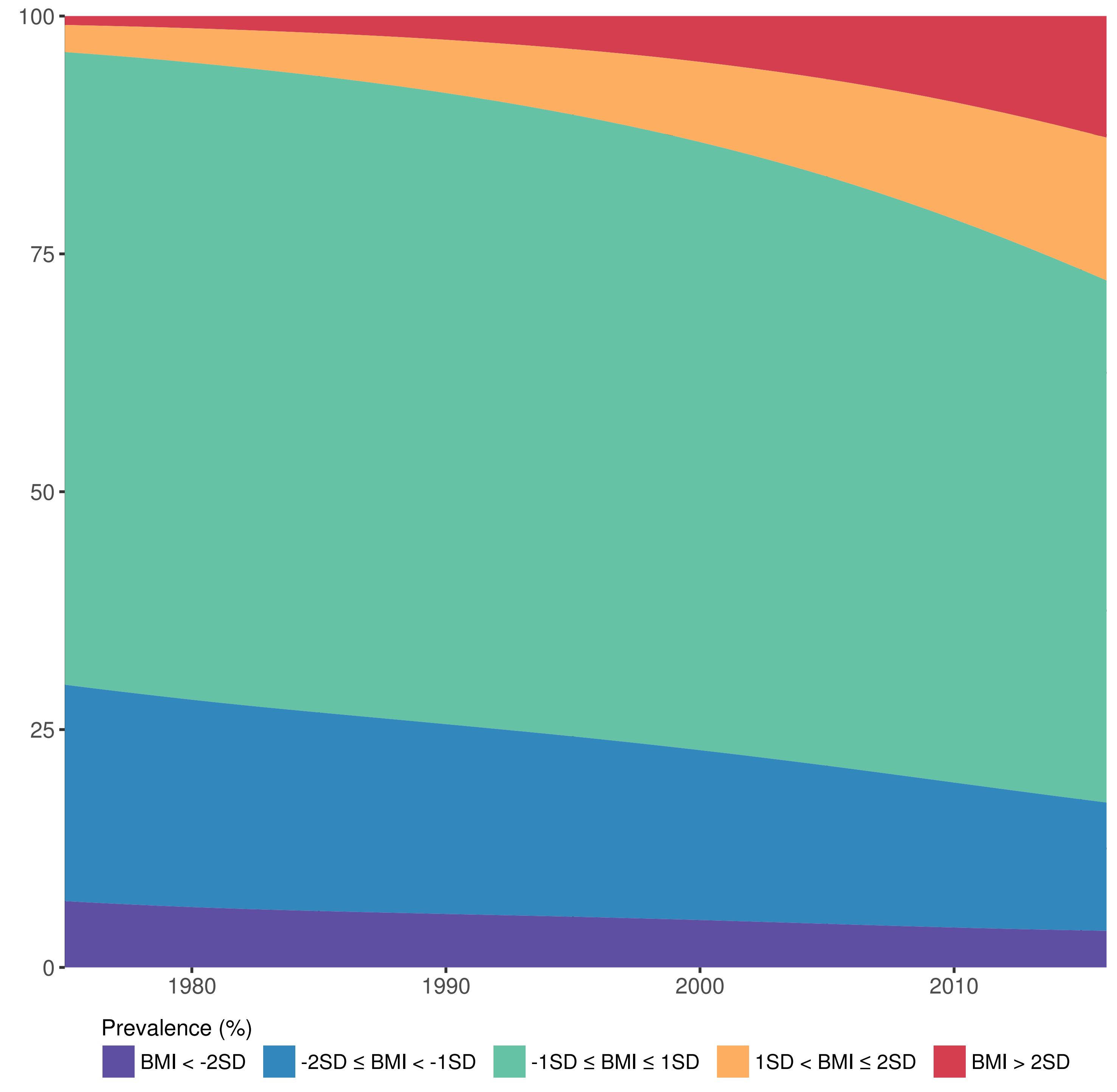
100
75
50
25
0
1980
1990
2000
2010
Prevalence (%)
BMI < -2SD
-2SD ≤ BMI < -1SD
-1SD ≤ BMI ≤ 1SD
1SD < BMI ≤ 2SD
BMI > 2SD
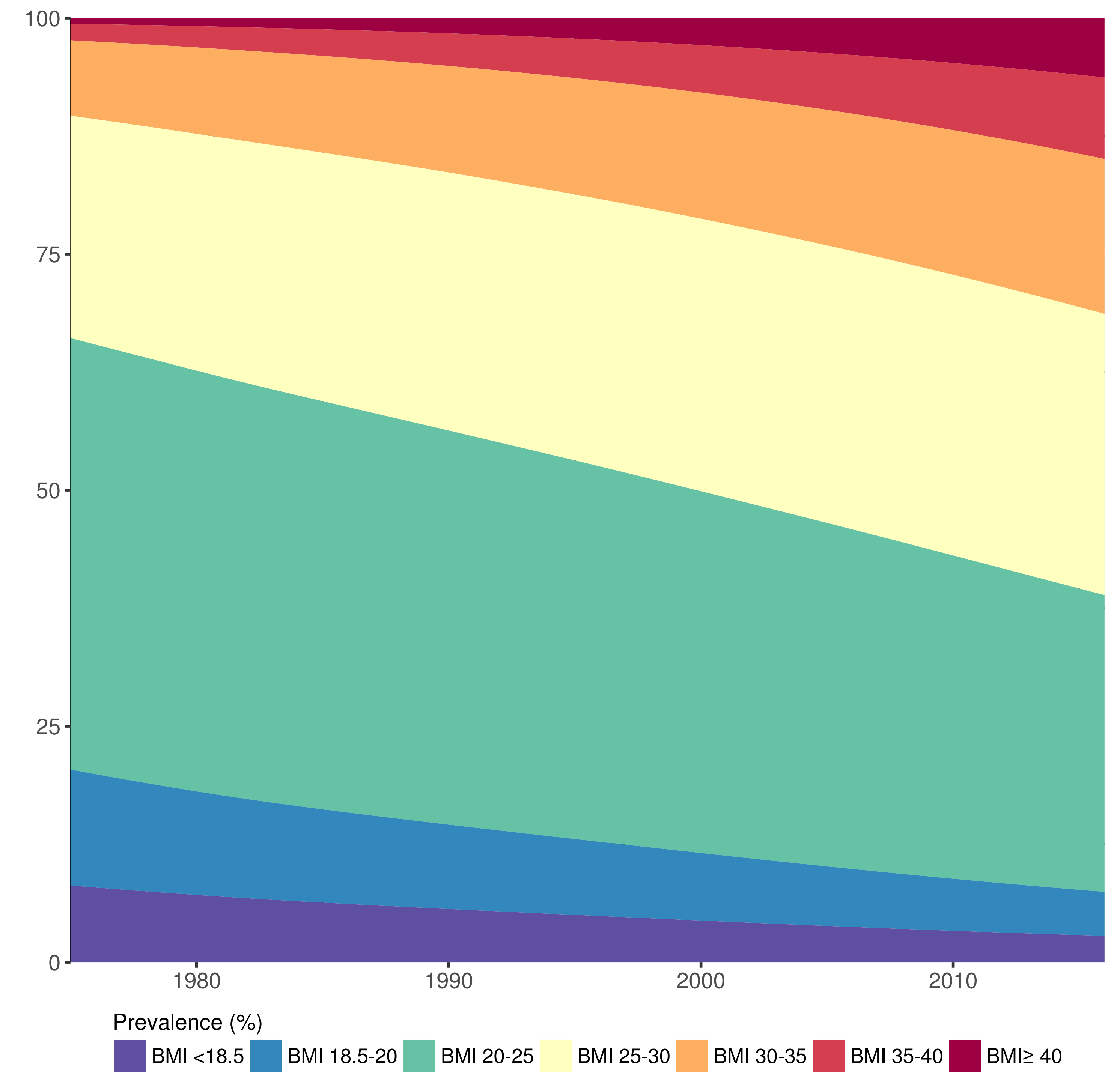
100
75
50
25
0
1980
1990
2000
2010
Prevalence (%)
BMI <18.5
BMI 18.5-20
BMI 20-25
BMI 25-30
BMI 30-35
BMI 35-40
BMI≥ 40
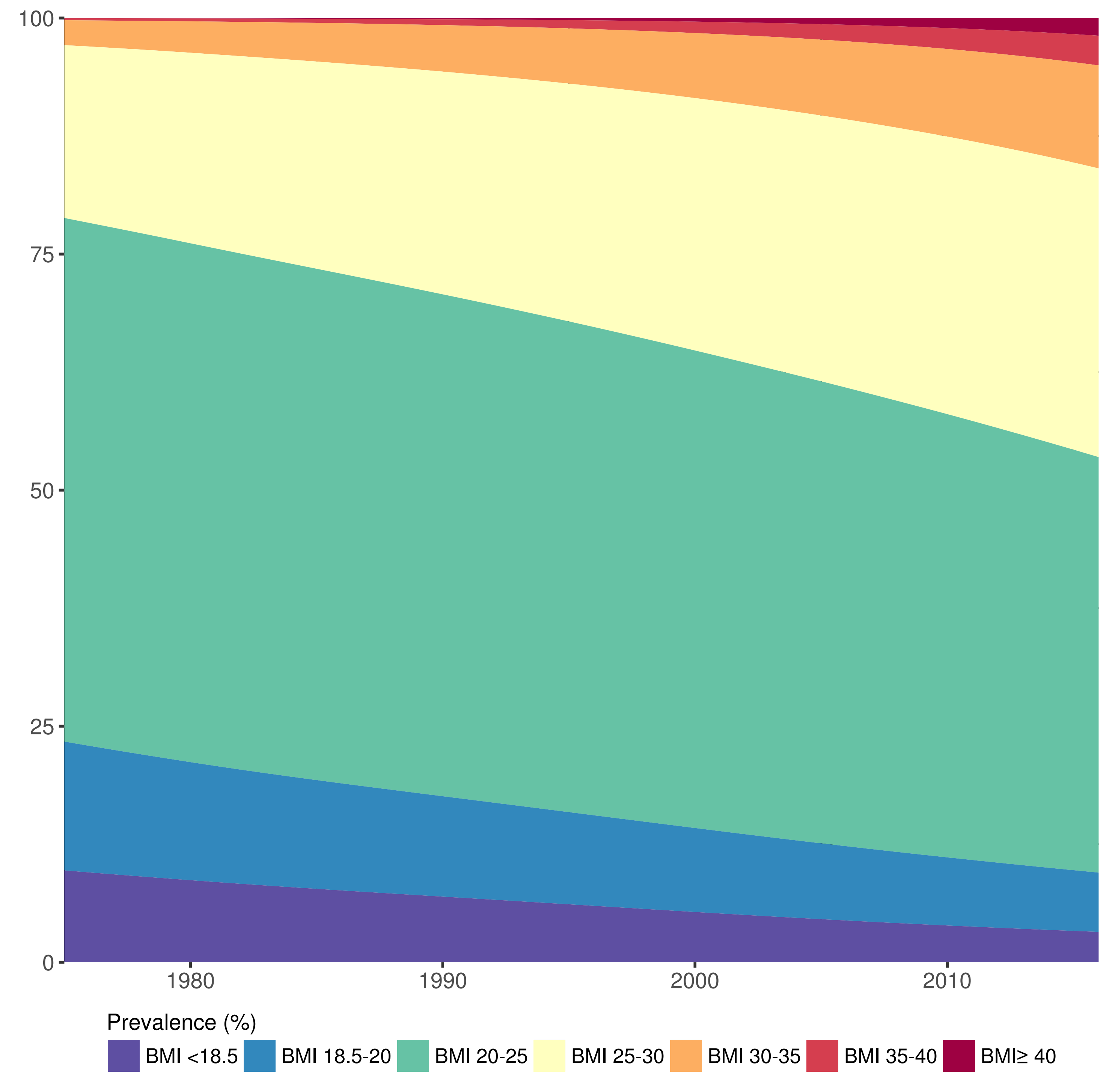
100
75
50
25
0
1980
1990
2000
2010
Prevalence (%)
BMI <18.5
BMI 18.5-20
BMI 20-25
BMI 25-30
BMI 30-35
BMI 35-40
BMI≥ 40

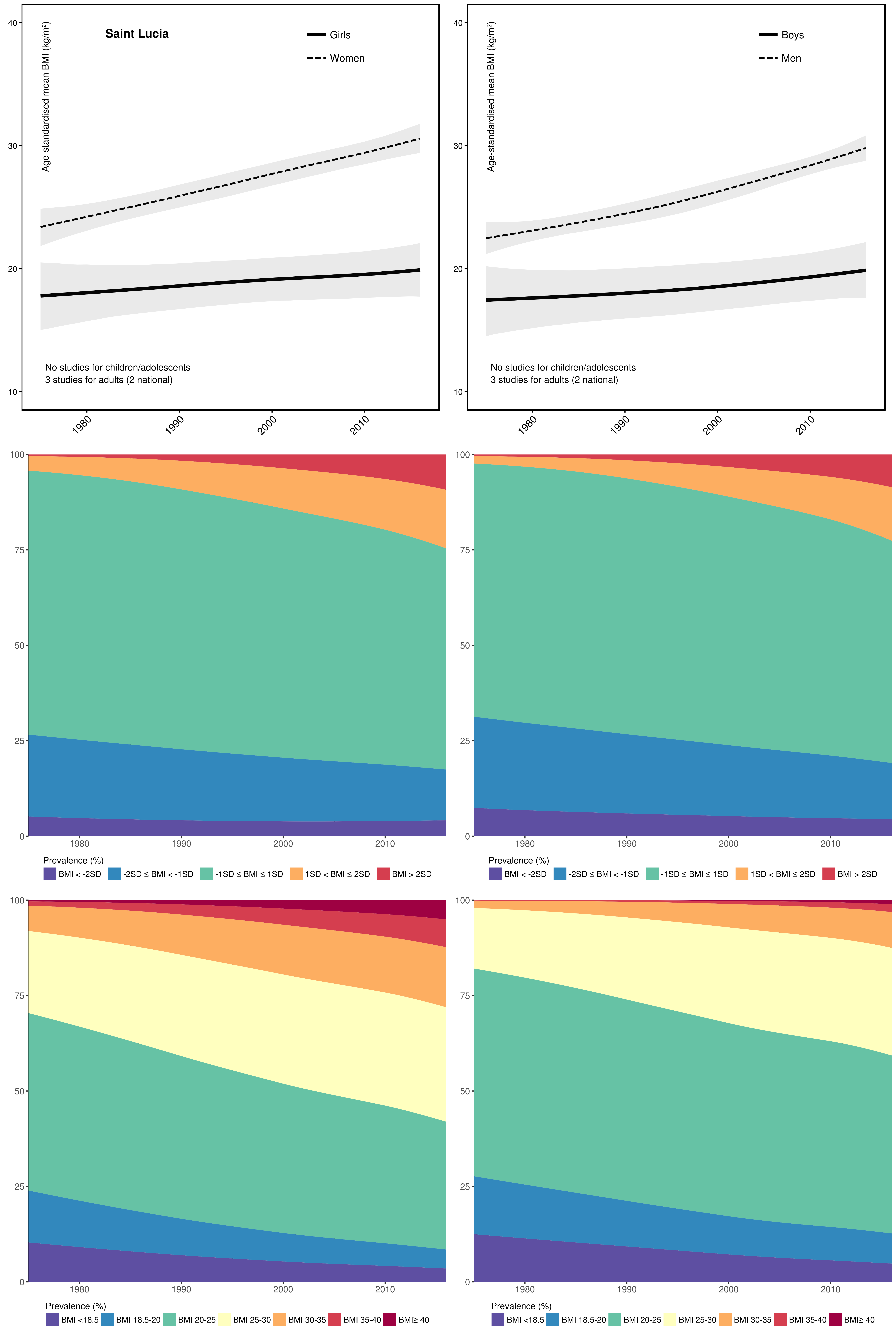
Saint Lucia
Girls
Women
Boys
Men
Age-standardised mean BMI (kg/m²)
No studies for children/adolescents
3 studies for adults (2 national)
10
20
30
40
1980
1990
2000
2010
0
25
50
75
100
Prevalence (%)
BMI < -2SD
-2SD ≤ BMI < -1SD
-1SD ≤ BMI ≤ 1SD
1SD < BMI ≤ 2SD
BMI > 2SD
BMI <18.5
BMI 18.5-20
BMI 20-25
BMI 25-30
BMI 30-35
BMI 35-40
BMI≥ 40

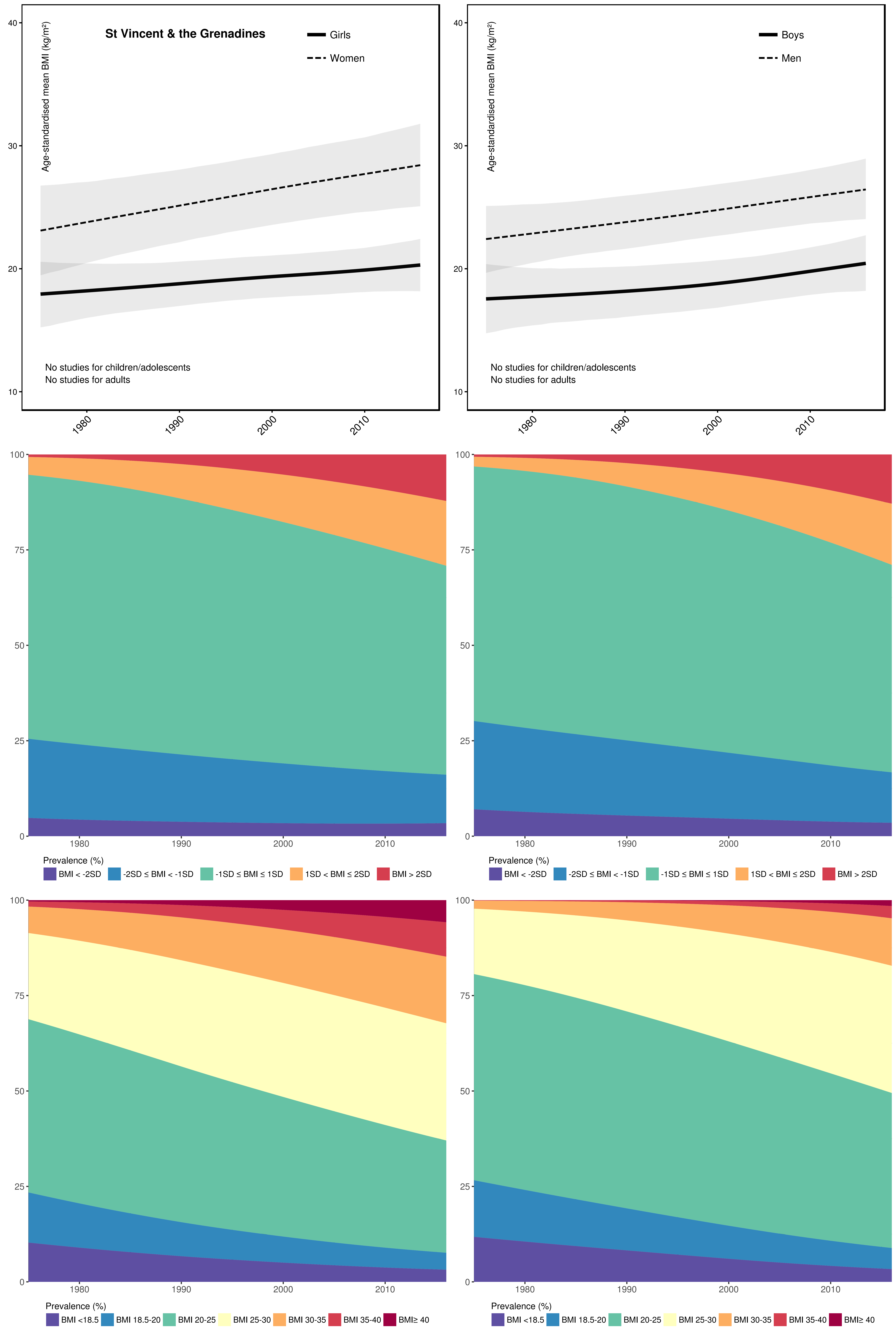

St Vincent & the Grenadines
Girls
Women
Boys
Men
Age-standardised mean BMI (kg/m²)
No studies for children/adolescents
No studies for adults
Prevalence (%)
BMI < -2SD
-2SD ≤ BMI < -1SD
-1SD ≤ BMI ≤ 1SD
1SD < BMI ≤ 2SD
BMI > 2SD
BMI <18.5
BMI 18.5-20
BMI 20-25
BMI 25-30
BMI 30-35
BMI 35-40
BMI≥ 40

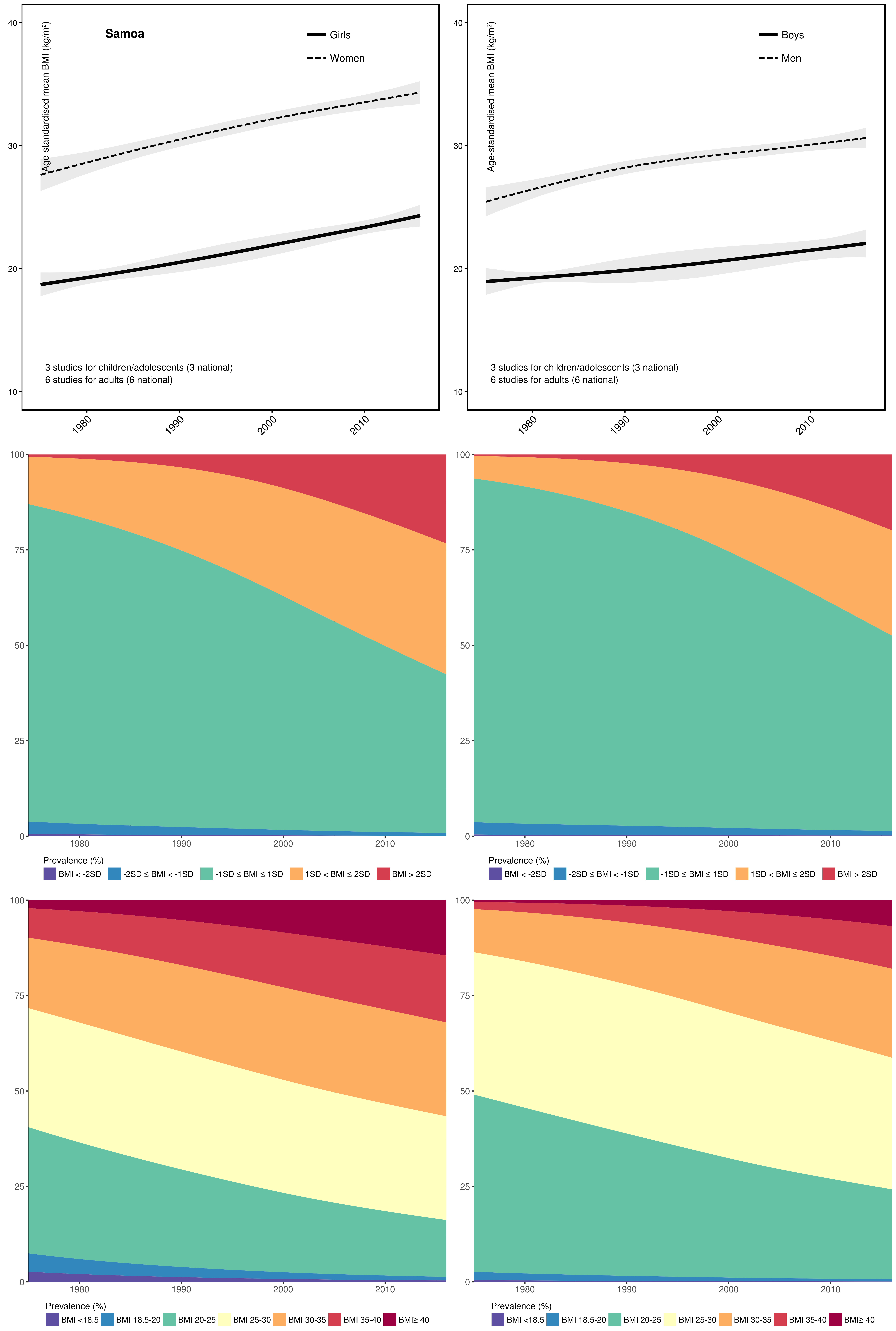
Samoa
Girls
Women
Boys
Men
Age-standardised mean BMI (kg/m²)
3 studies for children/adolescents (3 national)
6 studies for adults (6 national)
10
20
30
40
1980
1990
2000
2010
0
25
50
75
100
Prevalence (%)
BMI < -2SD
-2SD ≤ BMI < -1SD
-1SD ≤ BMI ≤ 1SD
1SD < BMI ≤ 2SD
BMI > 2SD
BMI <18.5
BMI 18.5-20
BMI 20-25
BMI 25-30
BMI 30-35
BMI 35-40
BMI≥ 40

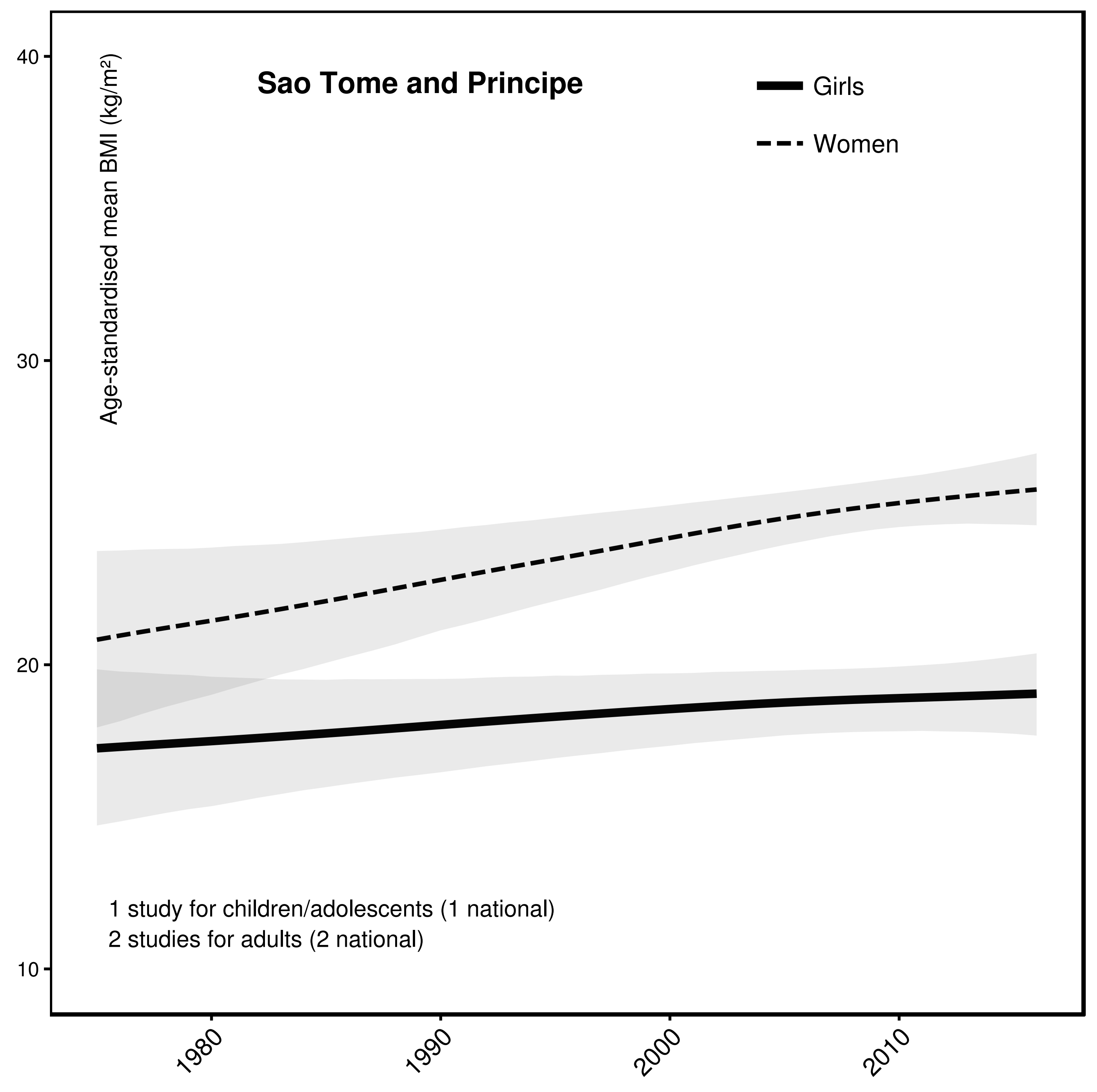
Sao Tome and Principe
Girls
Women
Age-standardised mean BMI (kg/m²)
1 study for children/adolescents (1 national)
2 studies for adults (2 national)
40
30
20
10
1980
1990
2000
2010

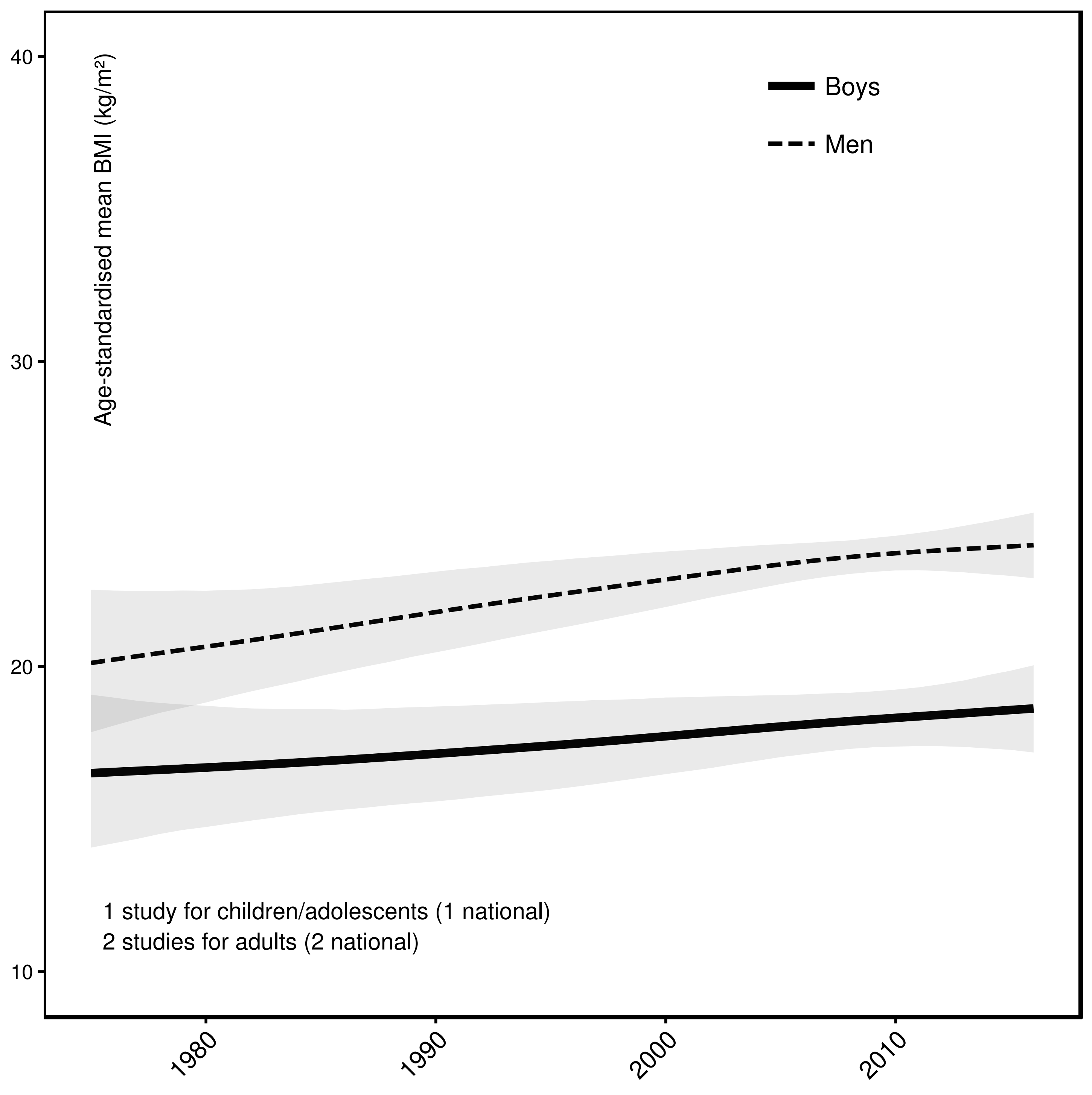
Boys
Men
Age-standardised mean BMI (kg/m²)
1 study for children/adolescents (1 national)
2 studies for adults (2 national)
40
30
20
10
1980
1990
2000
2010

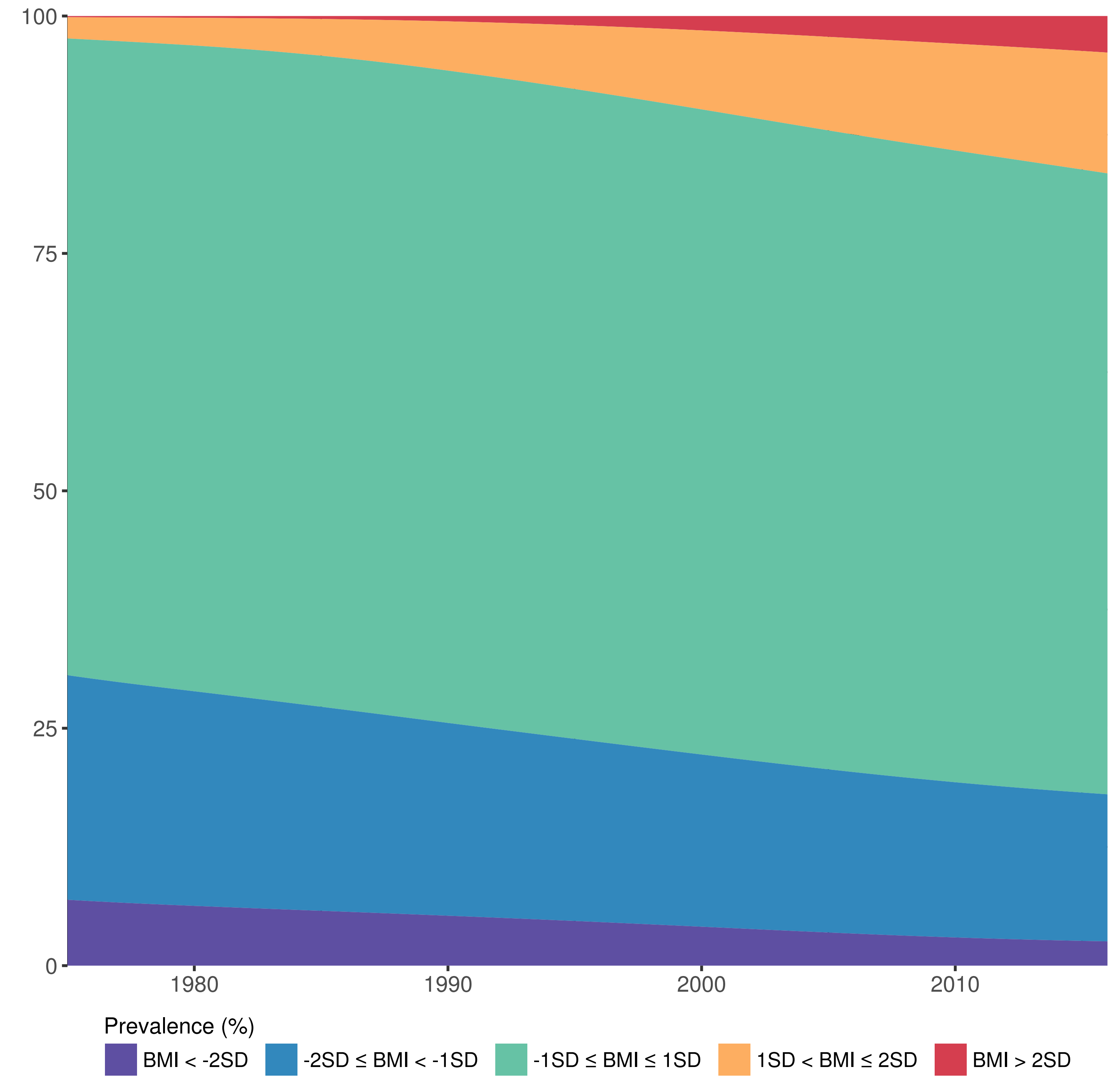
100
75
50
25
0
1980
1990
2000
2010
Prevalence (%)
BMI < -2SD
-2SD ≤ BMI < -1SD
-1SD ≤ BMI ≤ 1SD
1SD < BMI ≤ 2SD
BMI > 2SD

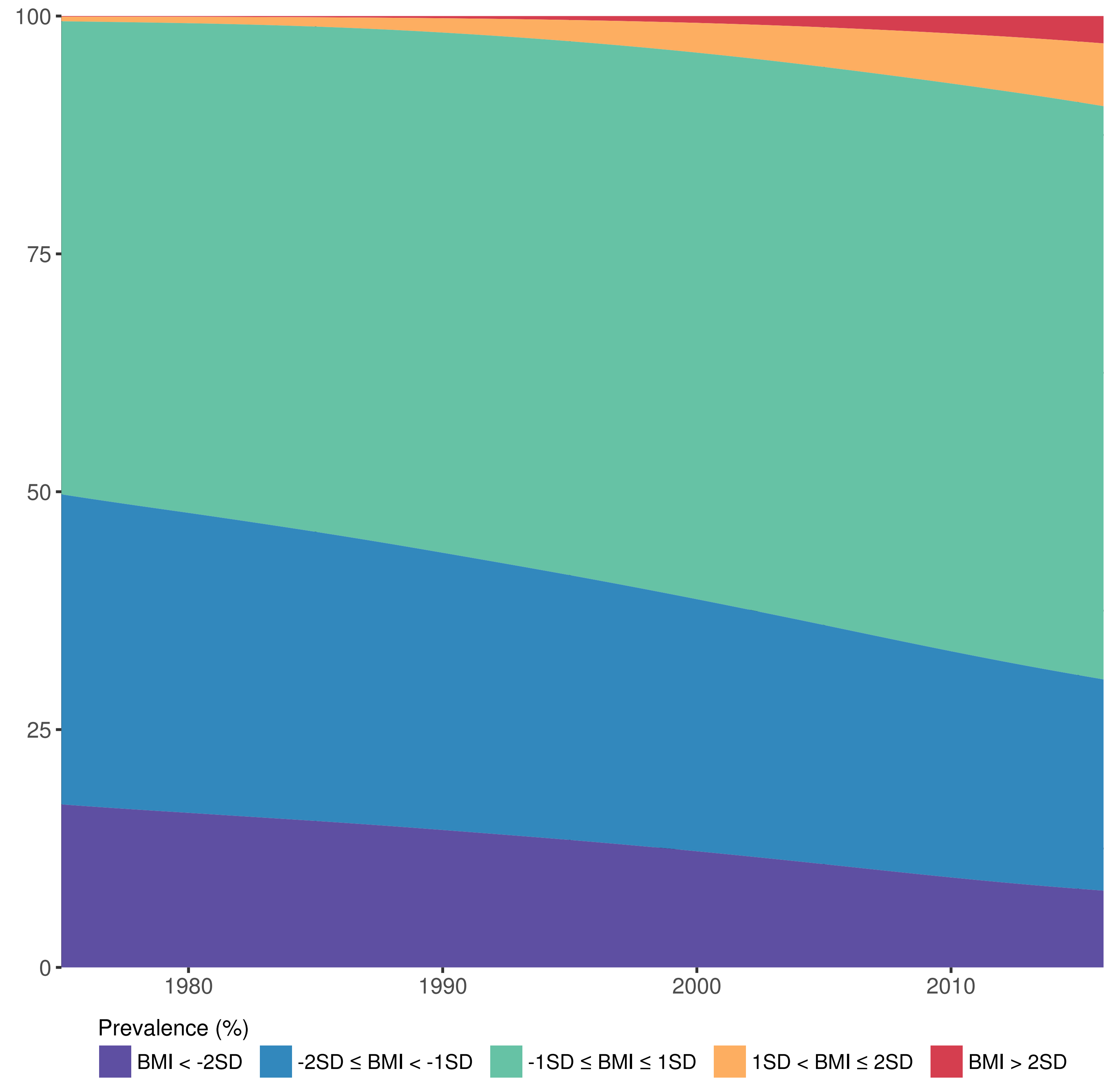
100
75
50
25
0
1980
1990
2000
2010
Prevalence (%)
BMI < -2SD
-2SD ≤ BMI < -1SD
-1SD ≤ BMI ≤ 1SD
1SD < BMI ≤ 2SD
BMI > 2SD

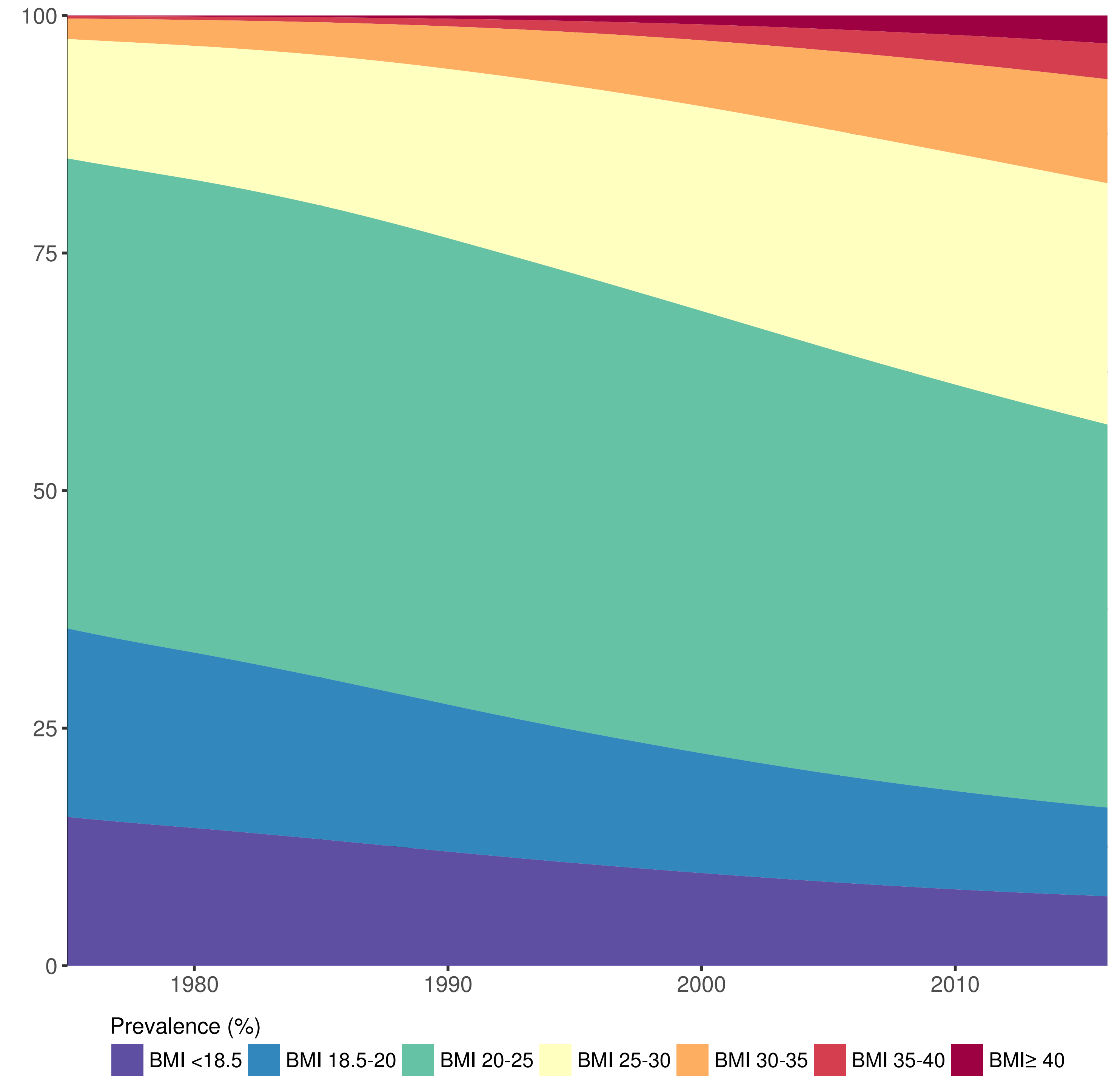
100
75
50
25
0
1980
1990
2000
2010
Prevalence (%)
BMI <18.5
BMI 18.5-20
BMI 20-25
BMI 25-30
BMI 30-35
BMI 35-40
BMI≥ 40

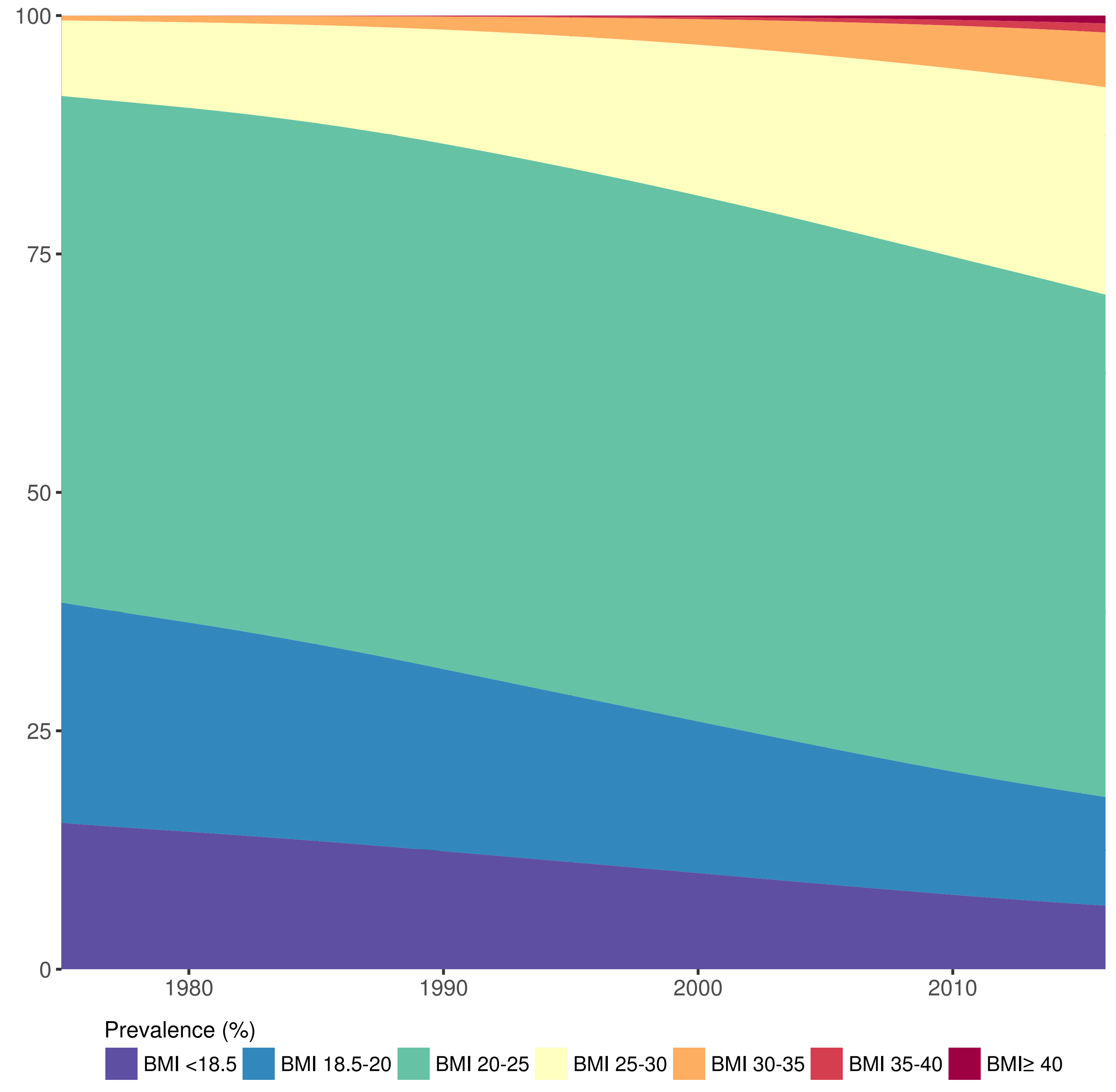
100
75
50
25
0
1980
1990
2000
2010
Prevalence (%)
BMI <18.5
BMI 18.5-20
BMI 20-25
BMI 25-30
BMI 30-35
BMI 35-40
BMI≥ 40

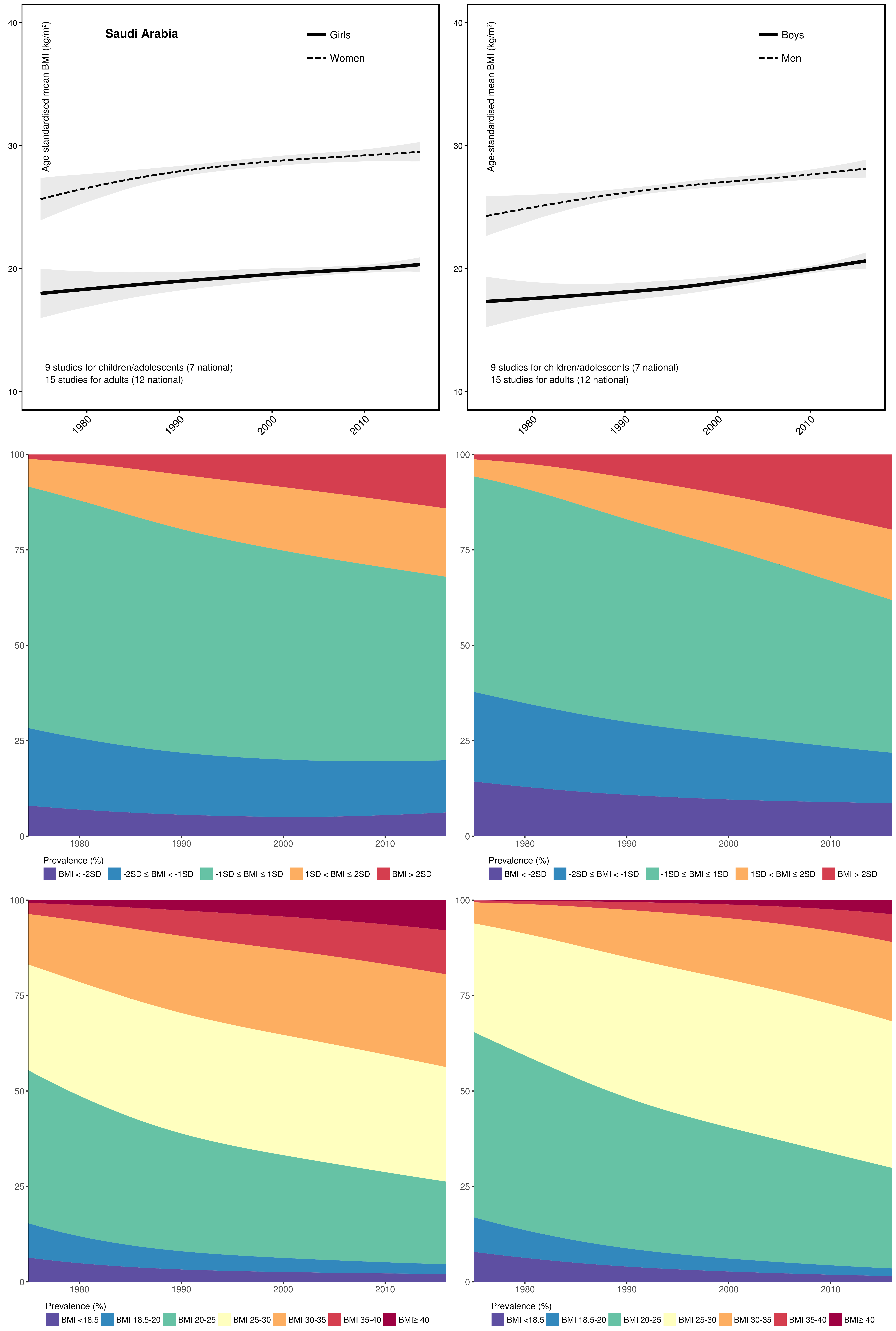

Saudi Arabia
Girls
Women
Boys
Men
Age-standardised mean BMI (kg/m²)
9 studies for children/adolescents (7 national)
15 studies for adults (12 national)
Prevalence (%)
BMI < -2SD
-2SD ≤ BMI < -1SD
-1SD ≤ BMI ≤ 1SD
1SD < BMI ≤ 2SD
BMI > 2SD
BMI <18.5
BMI 18.5-20
BMI 20-25
BMI 25-30
BMI 30-35
BMI 35-40
BMI≥ 40

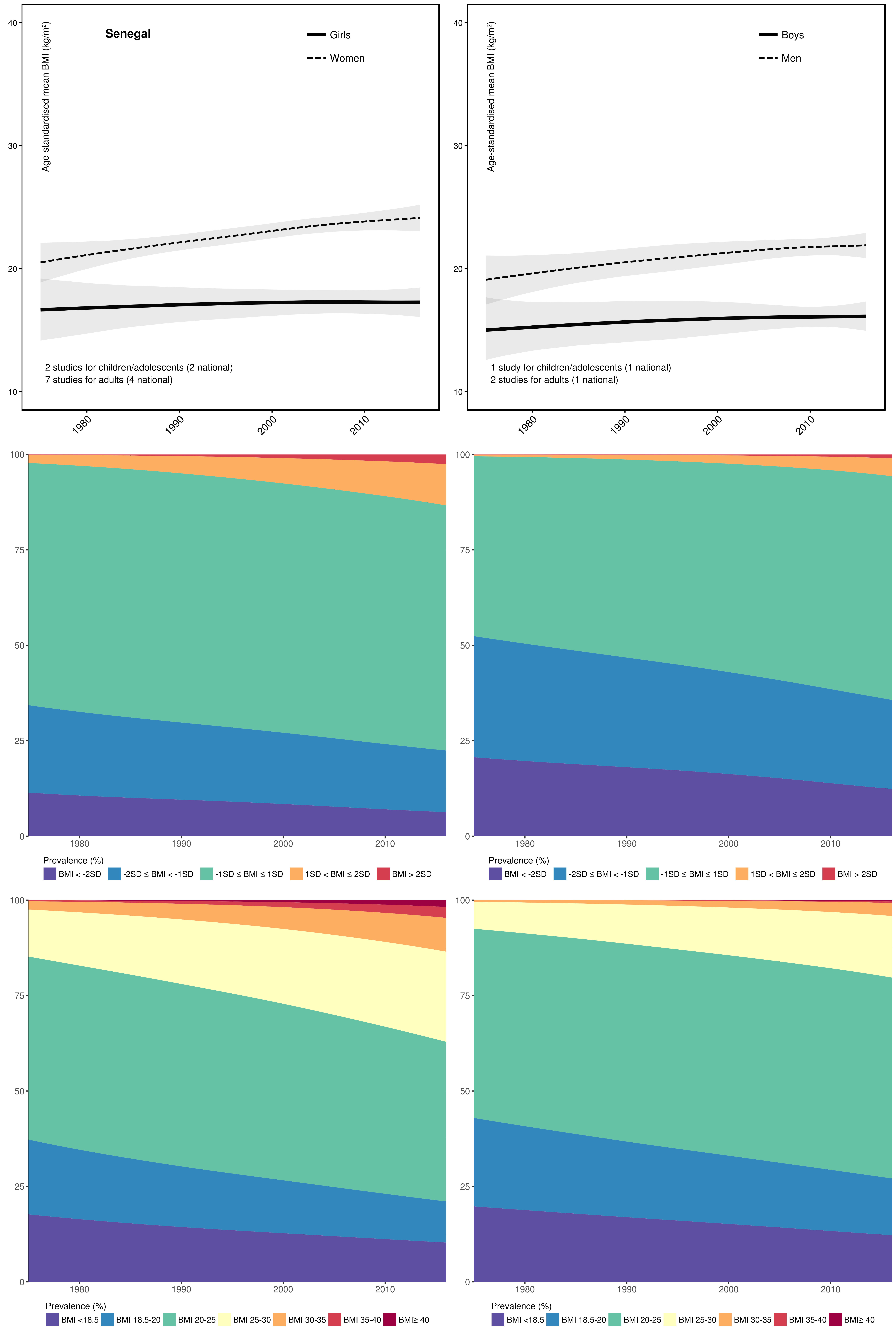
Senegal
Girls
Women
Age-standardised mean BMI (kg/m²)
2 studies for children/adolescents (2 national)
7 studies for adults (4 national)
Boys
Men
1 study for children/adolescents (1 national)
2 studies for adults (1 national)
1980
1990
2000
2010
Prevalence (%)
BMI < -2SD
-2SD ≤ BMI < -1SD
-1SD ≤ BMI ≤ 1SD
1SD < BMI ≤ 2SD
BMI > 2SD
BMI <18.5
BMI 18.5-20
BMI 20-25
BMI 25-30
BMI 30-35
BMI 35-40
BMI≥ 40

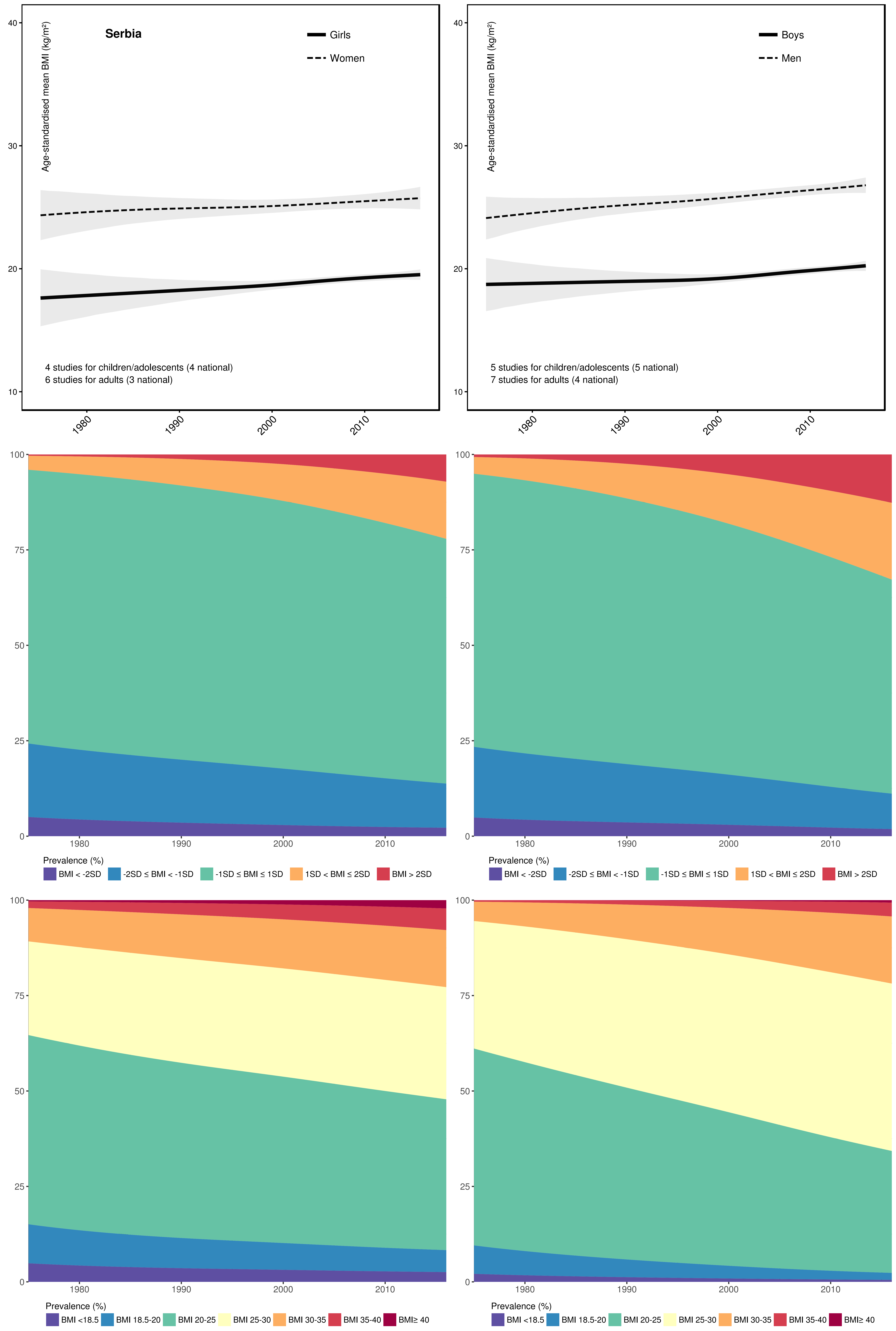
Serbia
Girls
Women
Boys
Men
Age-standardised mean BMI (kg/m²)
4 studies for children/adolescents (4 national)
6 studies for adults (3 national)
5 studies for children/adolescents (5 national)
7 studies for adults (4 national)
Prevalence (%)
BMI < -2SD
-2SD ≤ BMI < -1SD
-1SD ≤ BMI ≤ 1SD
1SD < BMI ≤ 2SD
BMI > 2SD
BMI <18.5
BMI 18.5-20
BMI 20-25
BMI 25-30
BMI 30-35
BMI 35-40
BMI≥ 40

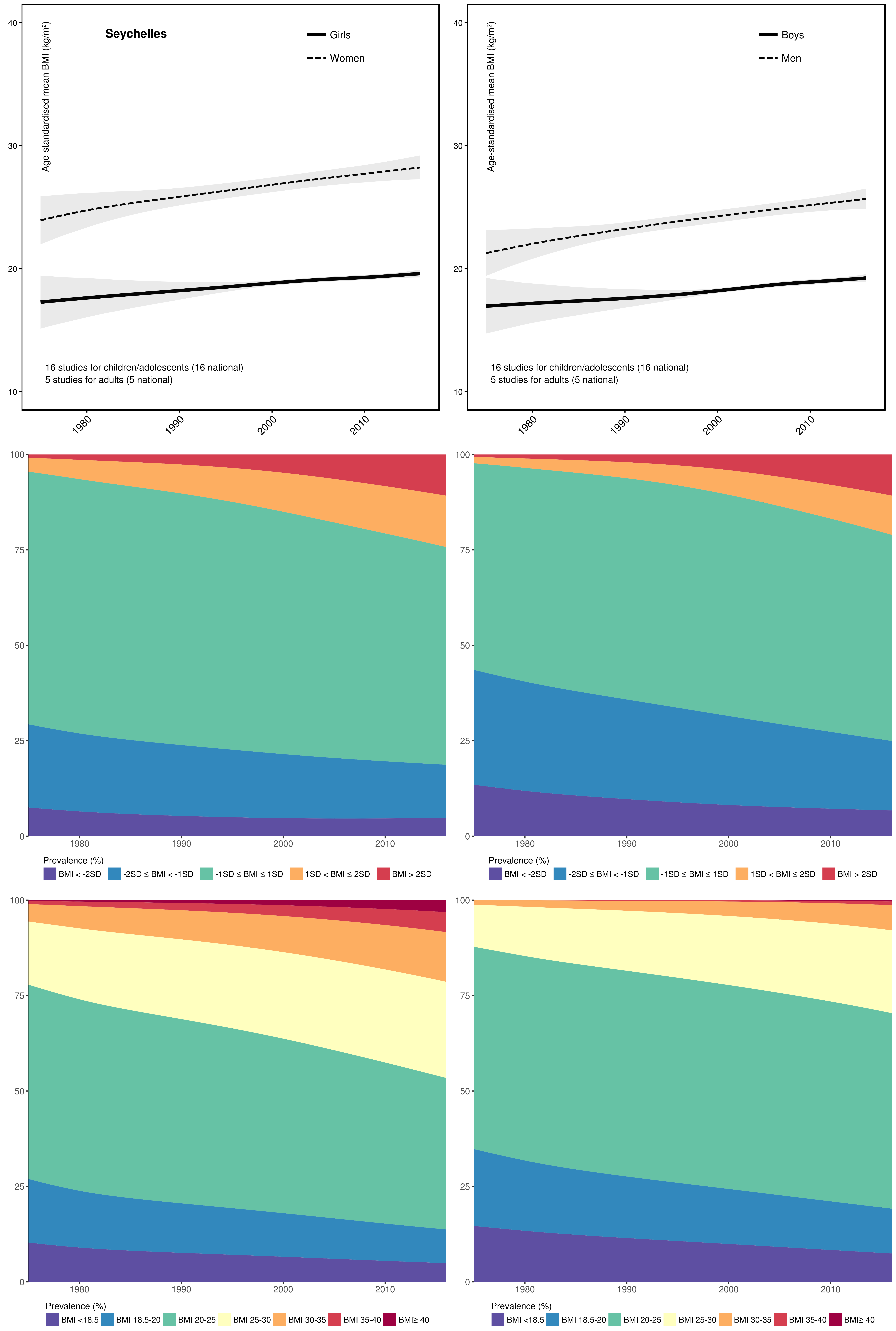
Seychelles
Girls
Women
Boys
Men
Age-standardised mean BMI (kg/m²)
16 studies for children/adolescents (16 national)
5 studies for adults (5 national)
Prevalence (%)
BMI < -2SD
-2SD ≤ BMI < -1SD
-1SD ≤ BMI ≤ 1SD
1SD < BMI ≤ 2SD
BMI > 2SD
BMI <18.5
BMI 18.5-20
BMI 20-25
BMI 25-30
BMI 30-35
BMI 35-40
BMI≥ 40
1980
1990
2000
2010

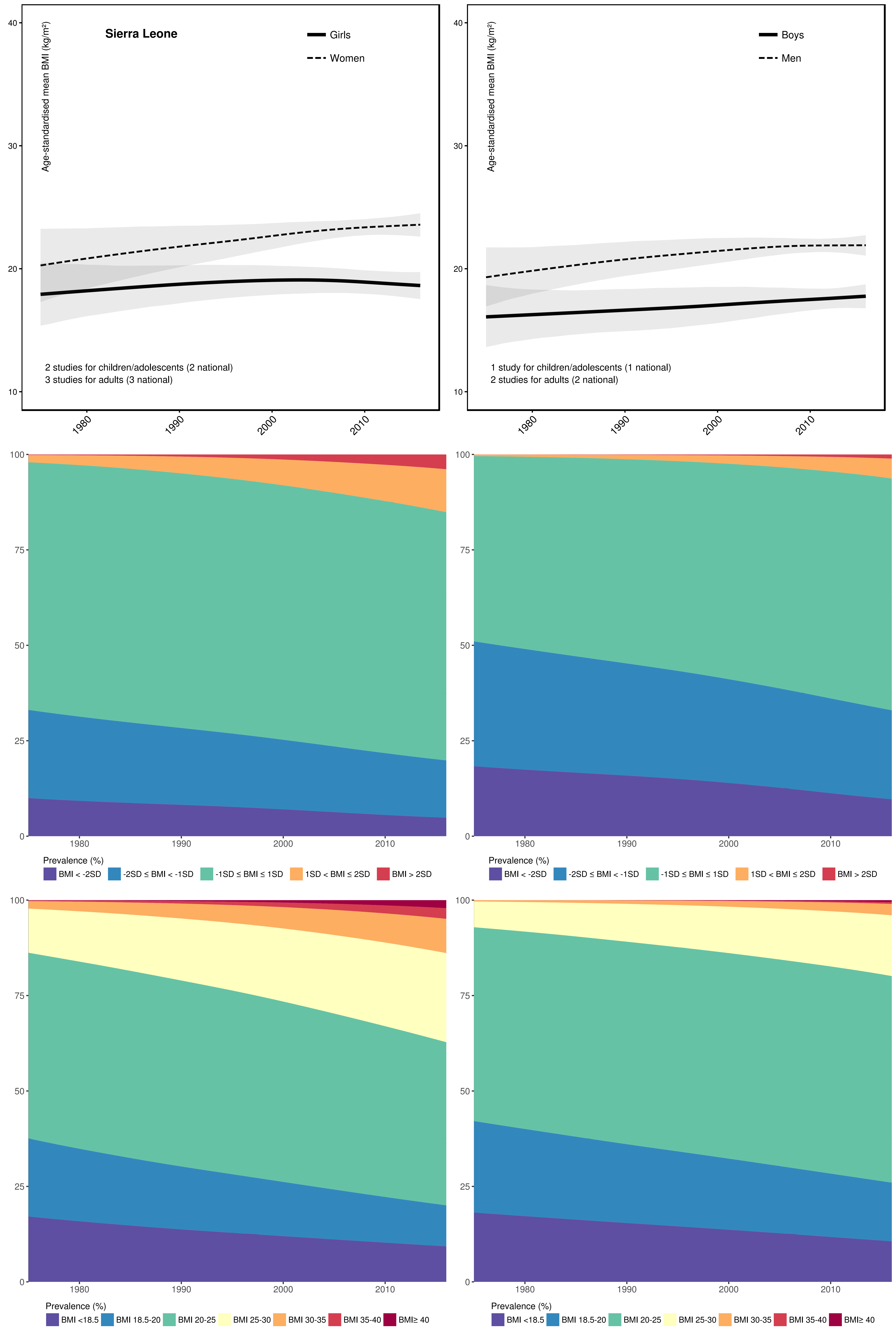
Sierra Leone
Girls
Women
Boys
Men
Age-standardised mean BMI (kg/m²)
2 studies for children/adolescents (2 national)
3 studies for adults (3 national)
1 study for children/adolescents (1 national)
2 studies for adults (2 national)
10
20
30
40
1980
1990
2000
2010
0
25
50
75
100
Prevalence (%)
BMI < -2SD
-2SD ≤ BMI < -1SD
-1SD ≤ BMI ≤ 1SD
1SD < BMI ≤ 2SD
BMI > 2SD
BMI <18.5
BMI 18.5-20
BMI 20-25
BMI 25-30
BMI 30-35
BMI 35-40
BMI≥ 40

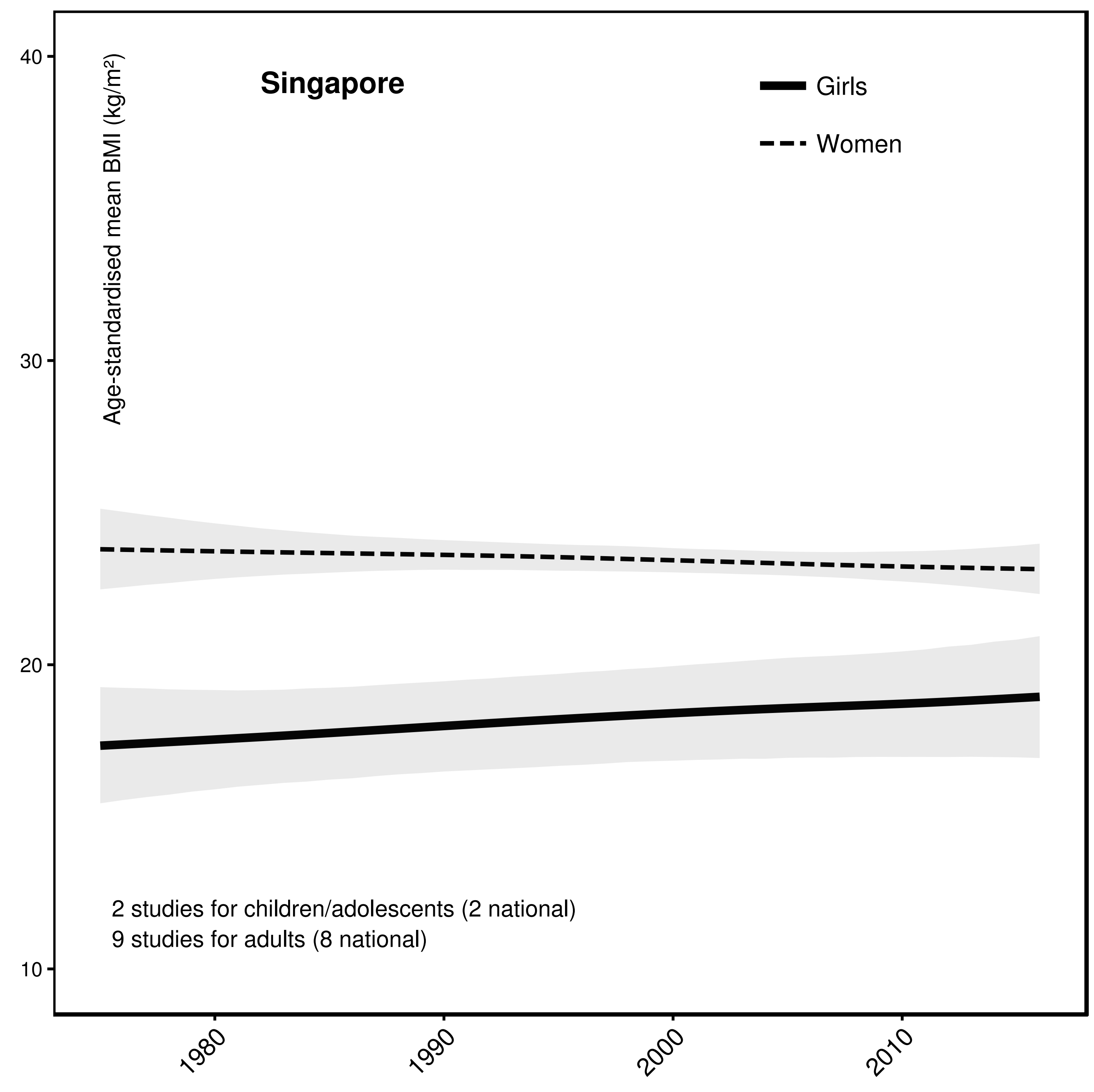
Singapore
Girls
Women
Age-standardised mean BMI (kg/m²)
2 studies for children/adolescents (2 national)
9 studies for adults (8 national)

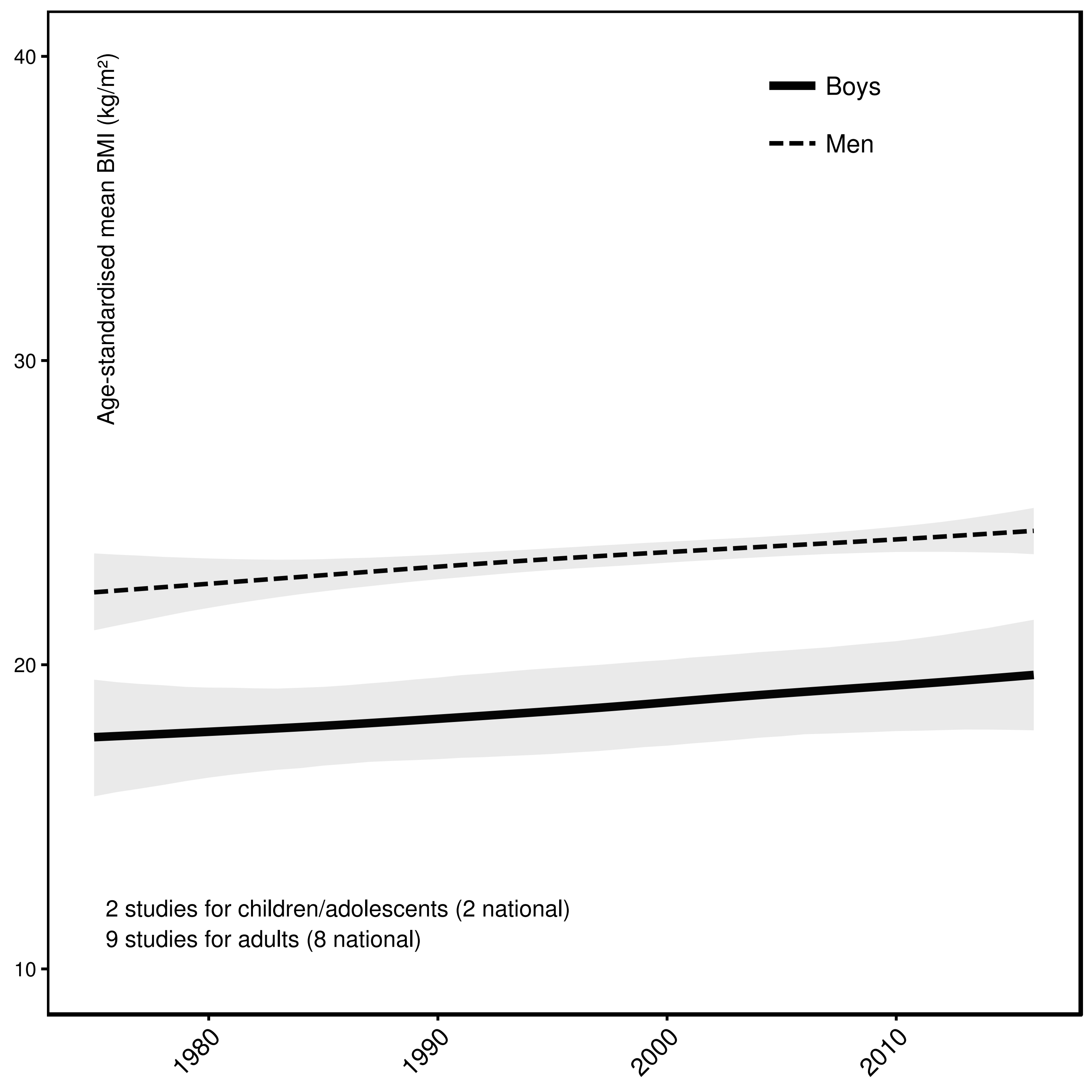
Boys
Men
Age-standardised mean BMI (kg/m²)
2 studies for children/adolescents (2 national)
9 studies for adults (8 national)

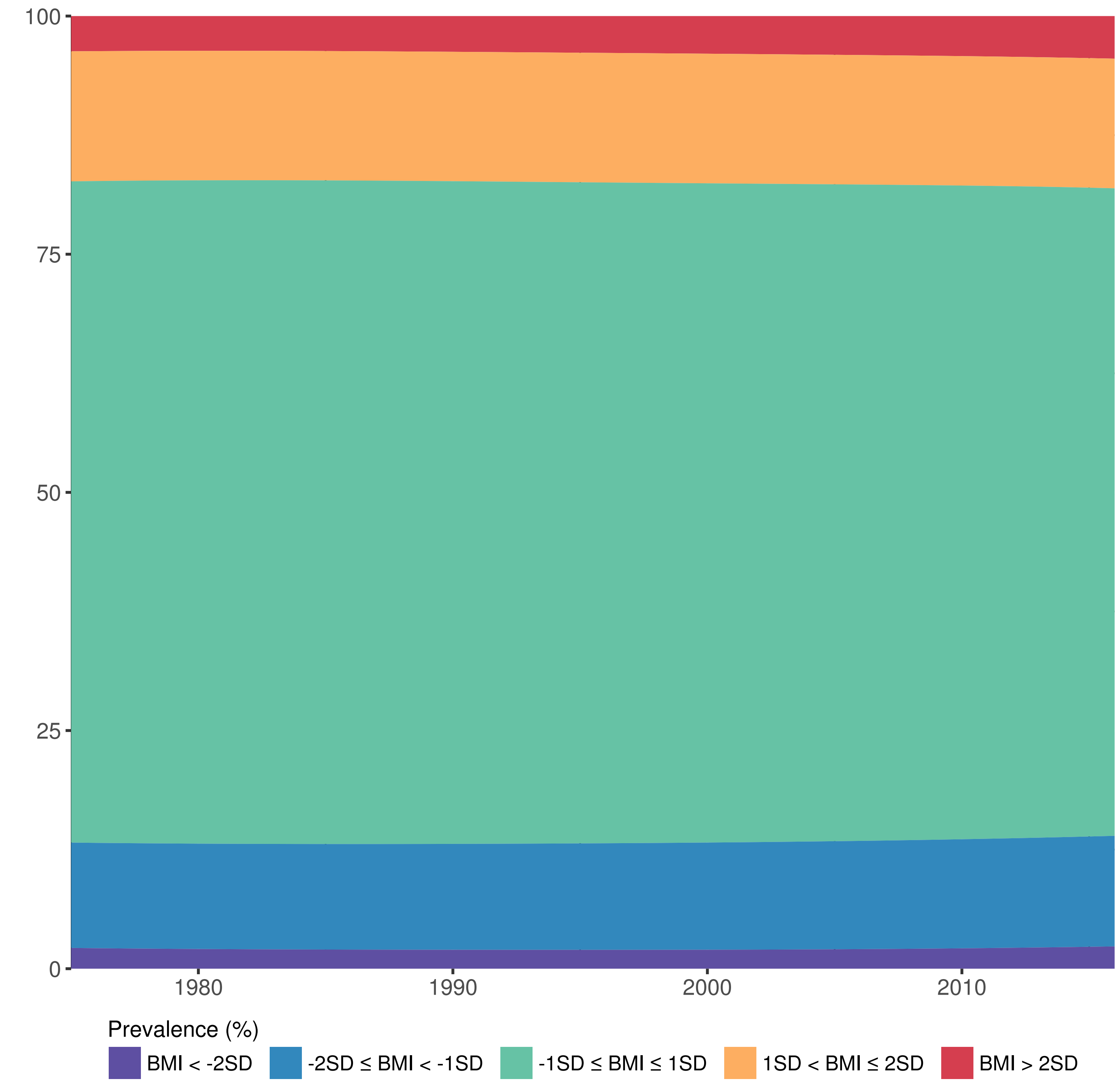
Prevalence (%)
BMI < -2SD
-2SD ≤ BMI < -1SD
-1SD ≤ BMI ≤ 1SD
1SD < BMI ≤ 2SD
BMI > 2SD

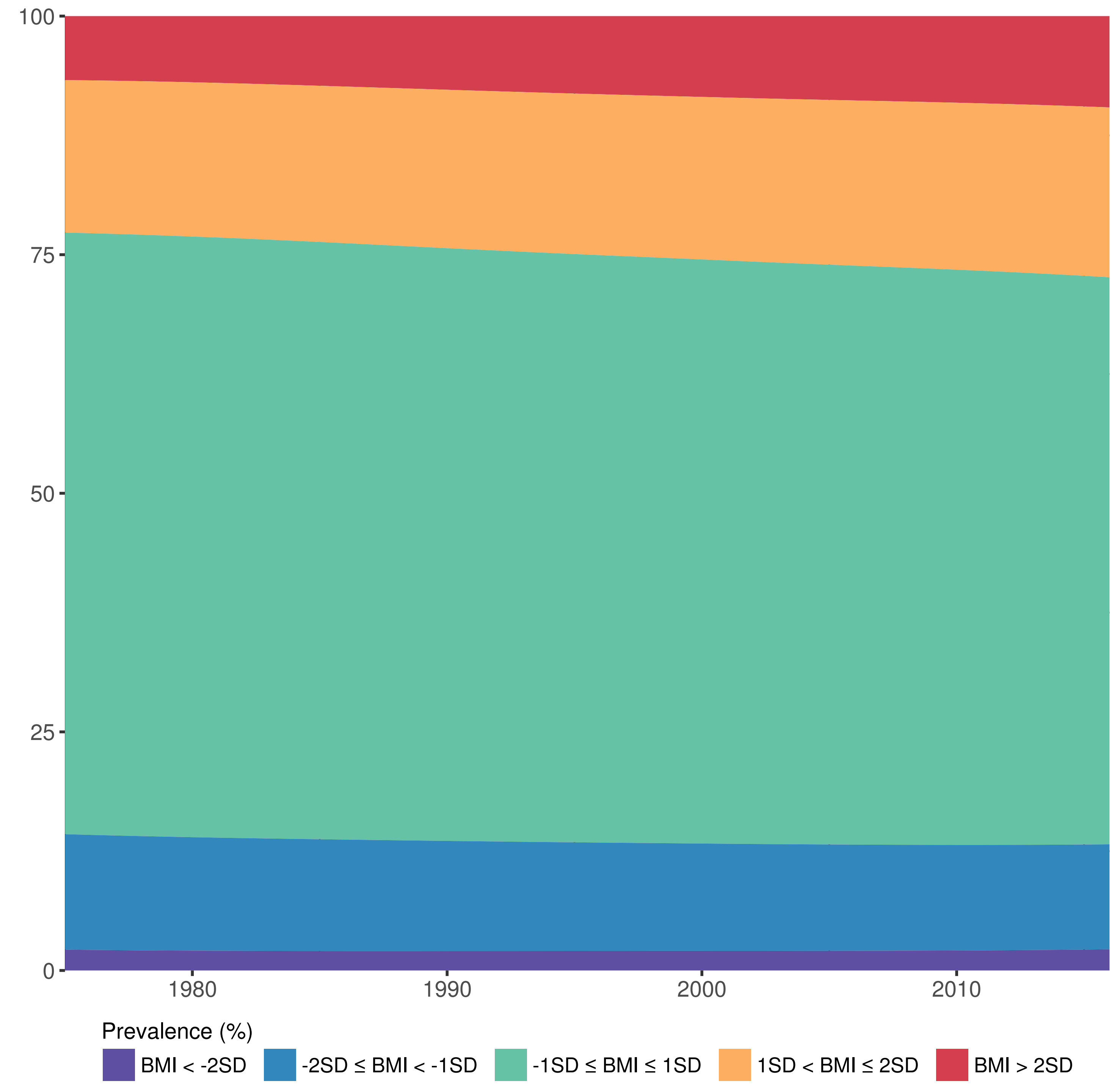
Prevalence (%)
BMI < -2SD
-2SD ≤ BMI < -1SD
-1SD ≤ BMI ≤ 1SD
1SD < BMI ≤ 2SD
BMI > 2SD

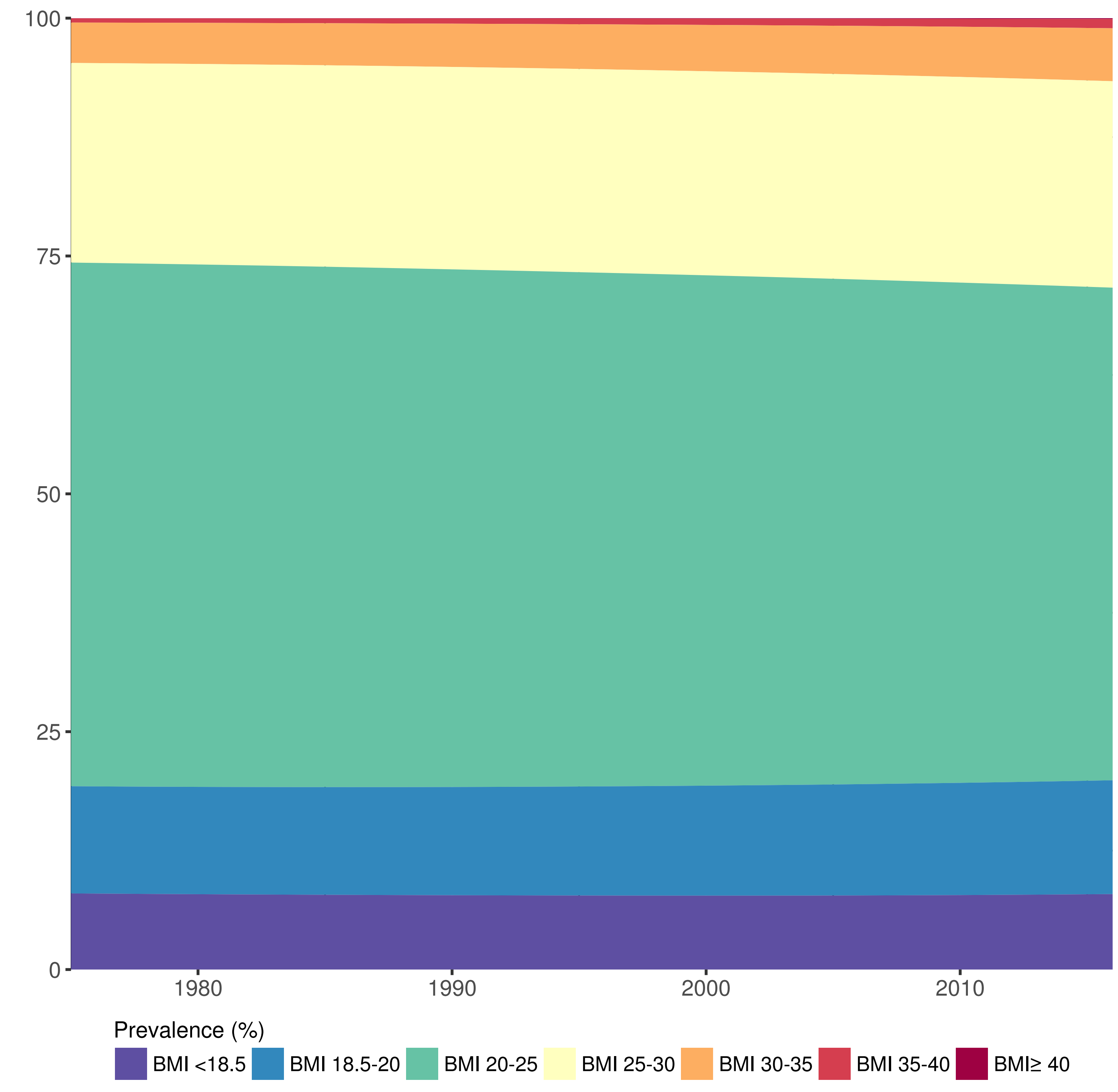
Prevalence (%)
BMI <18.5
BMI 18.5-20
BMI 20-25
BMI 25-30
BMI 30-35
BMI 35-40
BMI≥ 40

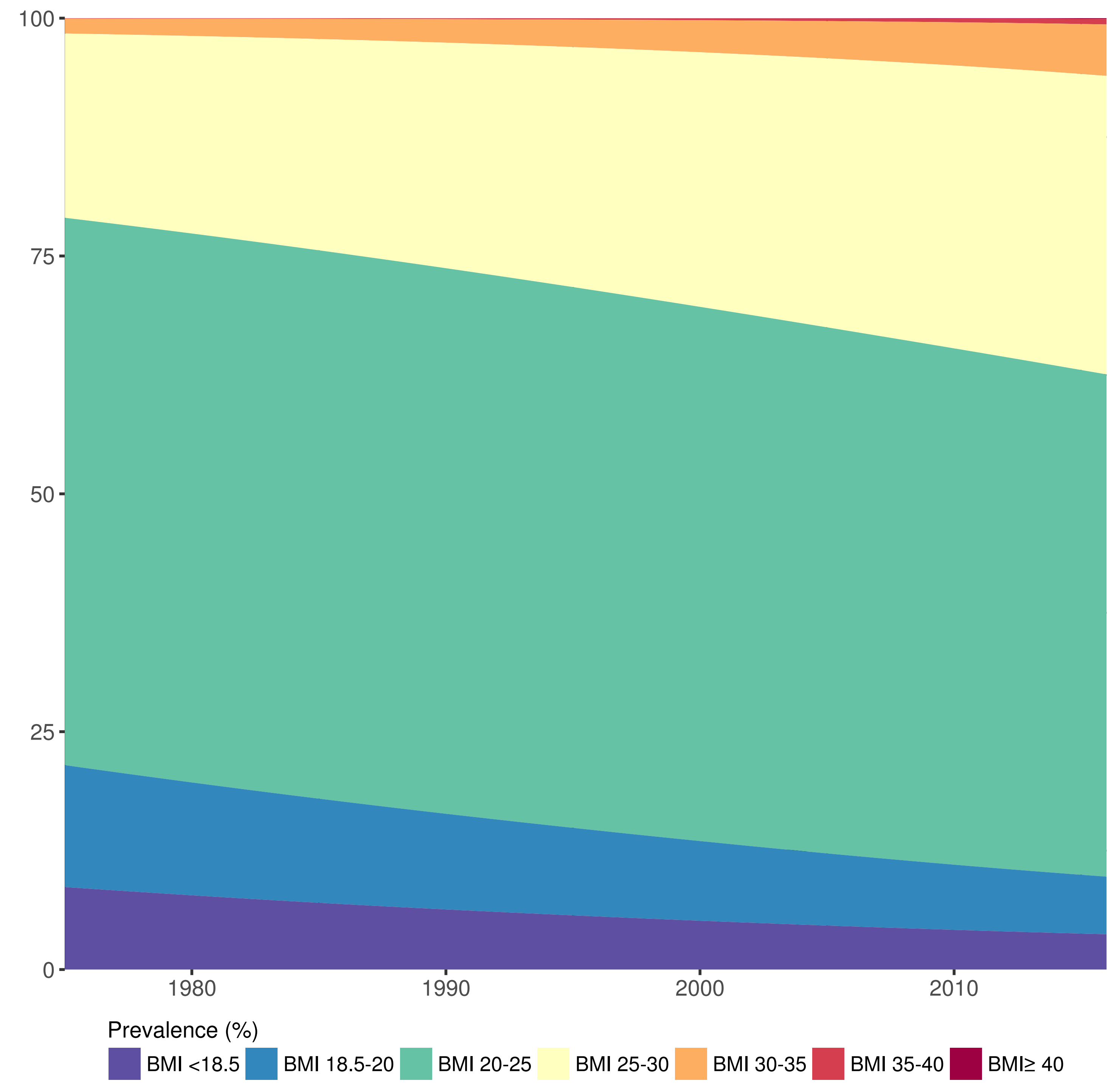
Prevalence (%)
BMI <18.5
BMI 18.5-20
BMI 20-25
BMI 25-30
BMI 30-35
BMI 35-40
BMI≥ 40

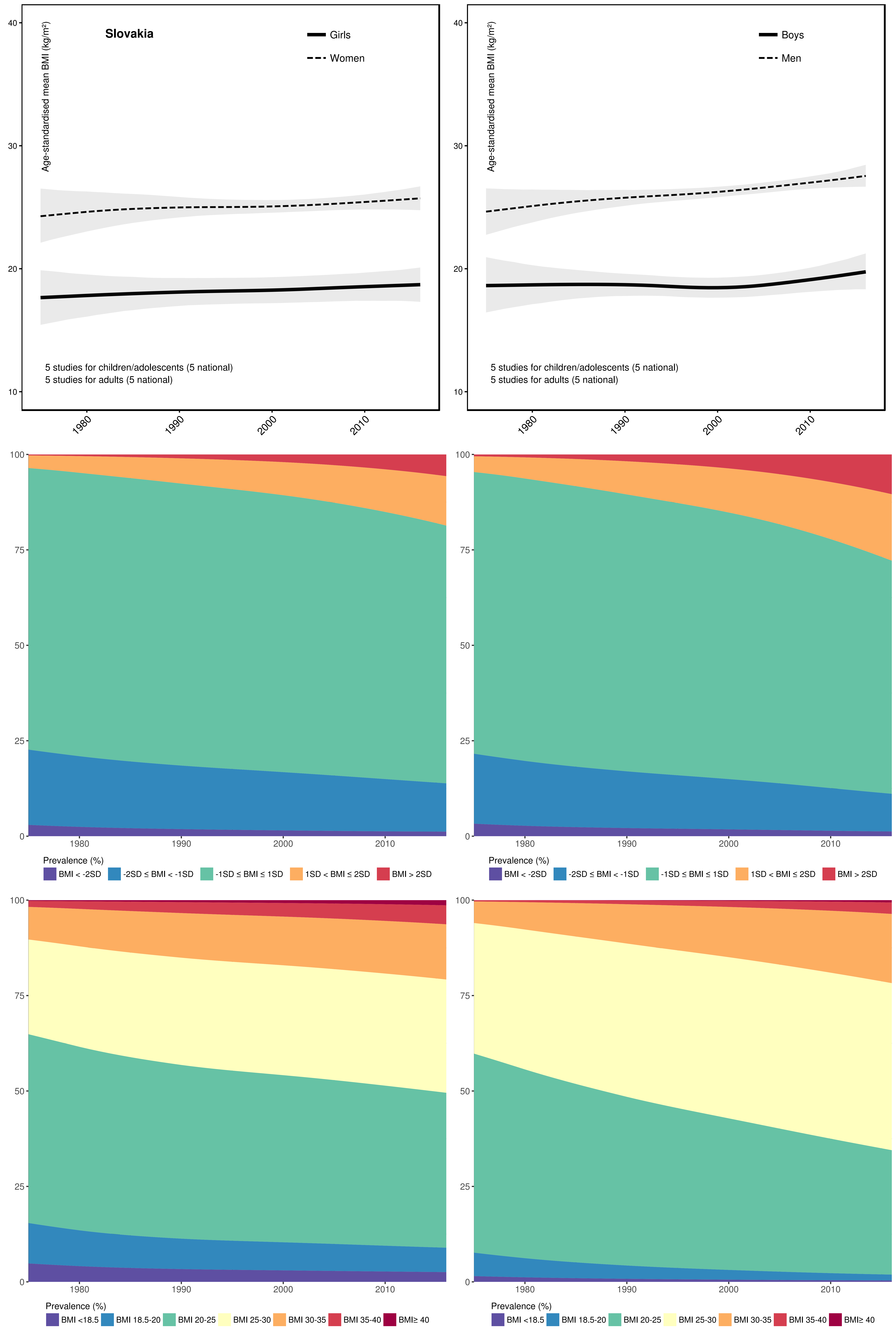
Slovakia
Girls
Women
Boys
Men
Age-standardised mean BMI (kg/m²)
5 studies for children/adolescents (5 national)
5 studies for adults (5 national)
Prevalence (%)
BMI < -2SD
-2SD ≤ BMI < -1SD
-1SD ≤ BMI ≤ 1SD
1SD < BMI ≤ 2SD
BMI > 2SD
BMI <18.5
BMI 18.5-20
BMI 20-25
BMI 25-30
BMI 30-35
BMI 35-40
BMI≥ 40

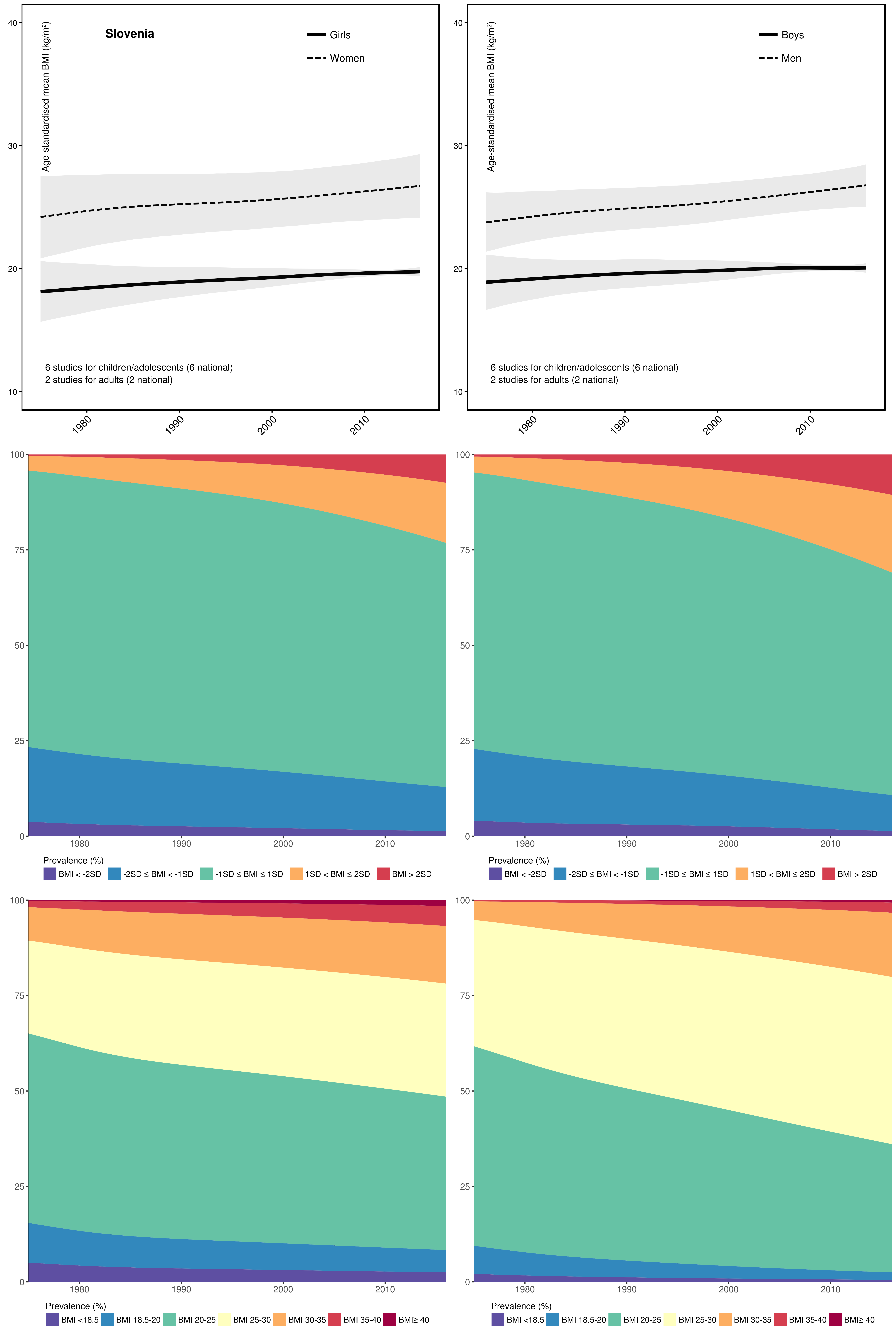
Slovenia
Age-standardised mean BMI (kg/m²)
Girls
Women
6 studies for children/adolescents (6 national)
2 studies for adults (2 national)
Boys
Men
6 studies for children/adolescents (6 national)
2 studies for adults (2 national)
Prevalence (%)
BMI < -2SD
-2SD ≤ BMI < -1SD
-1SD ≤ BMI ≤ 1SD
1SD < BMI ≤ 2SD
BMI > 2SD
Prevalence (%)
BMI <18.5
BMI 18.5-20
BMI 20-25
BMI 25-30
BMI 30-35
BMI 35-40
BMI≥ 40

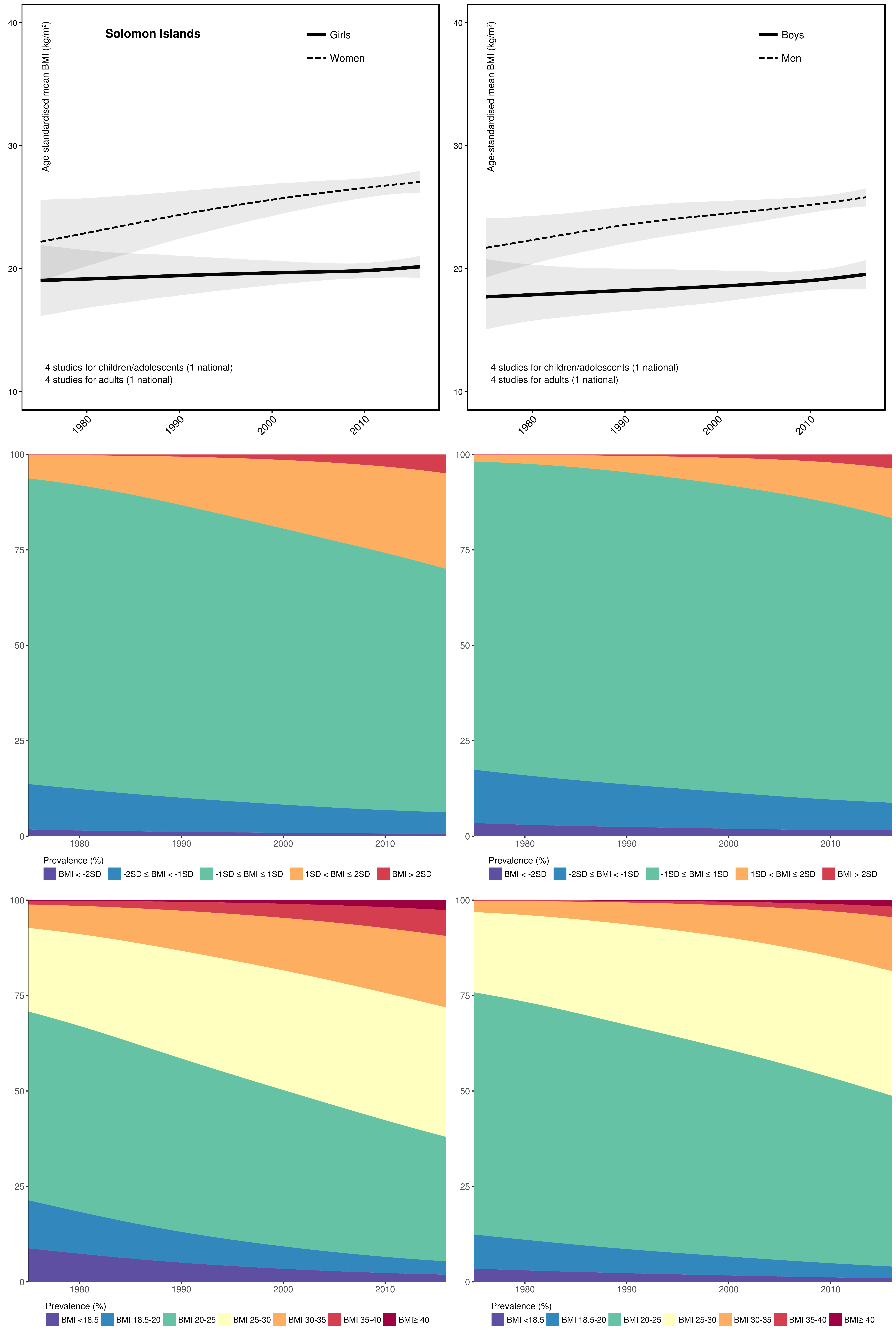
Solomon Islands
Girls
Women
Boys
Men
Age-standardised mean BMI (kg/m²)
40
30
20
10
1980
1990
2000
2010
4 studies for children/adolescents (1 national)
4 studies for adults (1 national)
100
75
50
25
0
Prevalence (%)
BMI < -2SD
-2SD ≤ BMI < -1SD
-1SD ≤ BMI ≤ 1SD
1SD < BMI ≤ 2SD
BMI > 2SD
BMI <18.5
BMI 18.5-20
BMI 20-25
BMI 25-30
BMI 30-35
BMI 35-40
BMI≥ 40

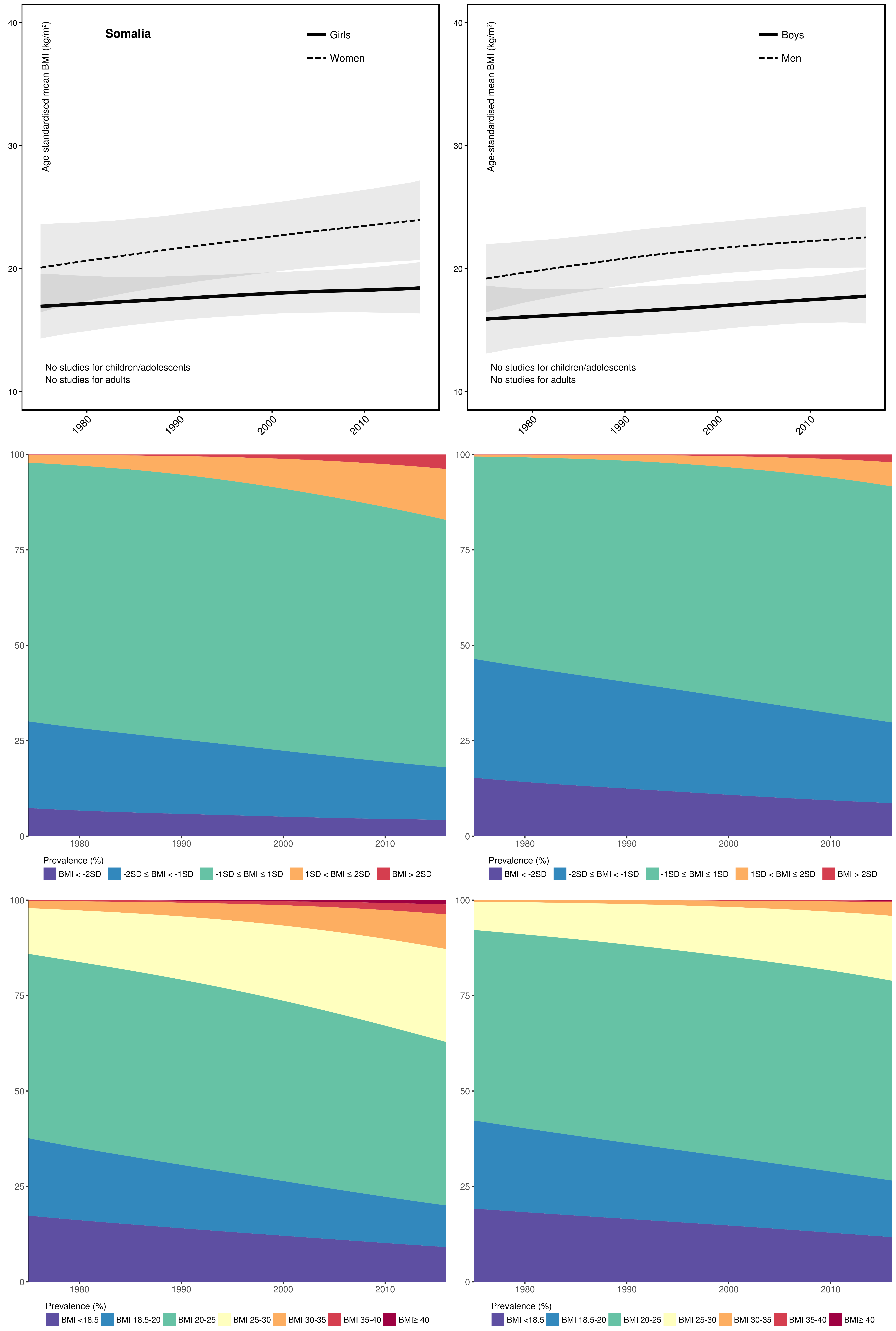
Somalia
Girls
Women
Boys
Men
Age-standardised mean BMI (kg/m²)
No studies for children/adolescents
No studies for adults
40
30
20
10
1980
1990
2000
2010
100
75
50
25
0
Prevalence (%)
BMI < -2SD
-2SD ≤ BMI < -1SD
-1SD ≤ BMI ≤ 1SD
1SD < BMI ≤ 2SD
BMI > 2SD
BMI <18.5
BMI 18.5-20
BMI 20-25
BMI 25-30
BMI 30-35
BMI 35-40
BMI≥ 40

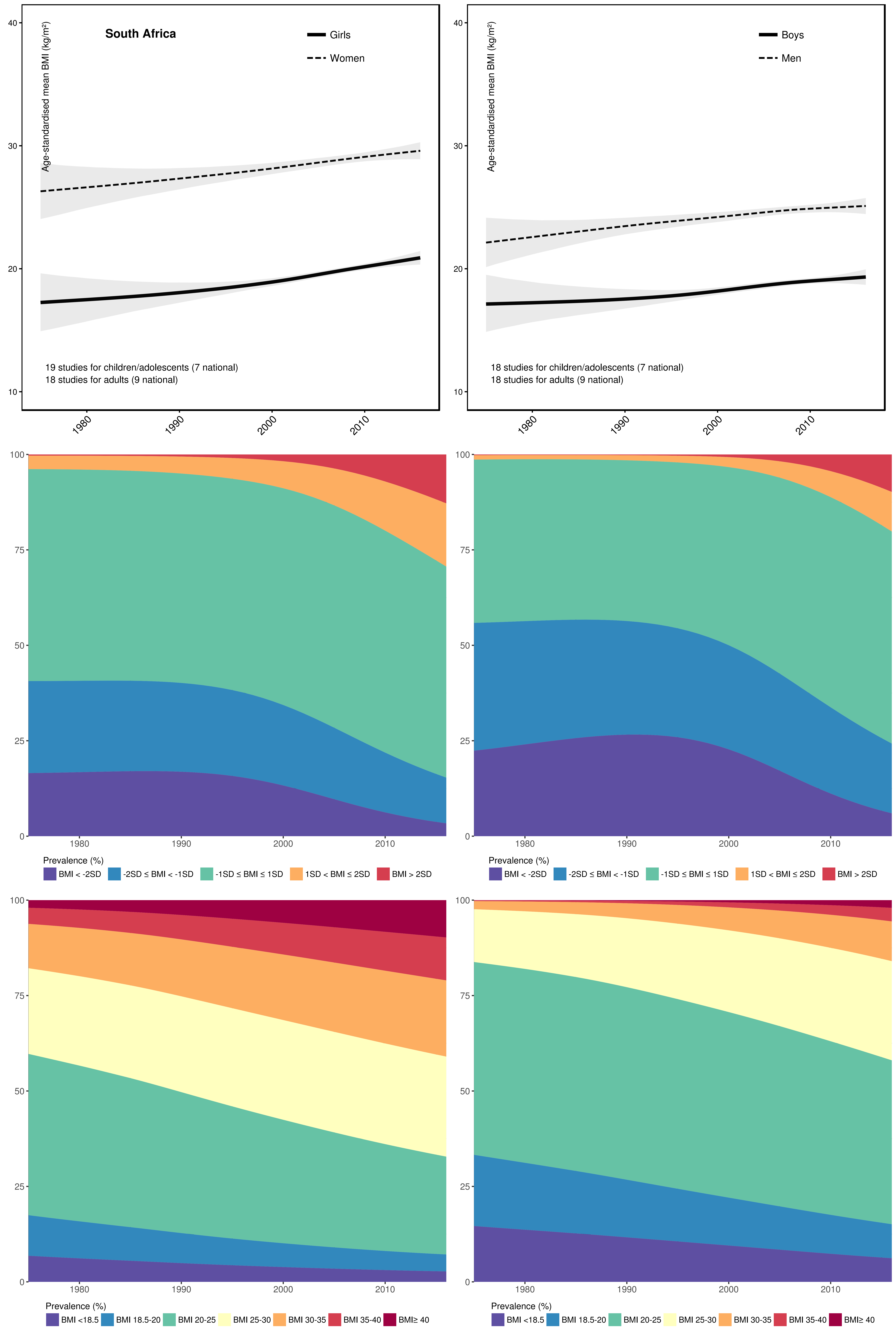
South Africa
Girls
Women
Boys
Men
Age-standardised mean BMI (kg/m²)
19 studies for children/adolescents (7 national)
18 studies for adults (9 national)
18 studies for children/adolescents (7 national)
18 studies for adults (9 national)
1980
1990
2000
2010
Prevalence (%)
BMI < -2SD
-2SD ≤ BMI < -1SD
-1SD ≤ BMI ≤ 1SD
1SD < BMI ≤ 2SD
BMI > 2SD
BMI <18.5
BMI 18.5-20
BMI 20-25
BMI 25-30
BMI 30-35
BMI 35-40
BMI≥ 40

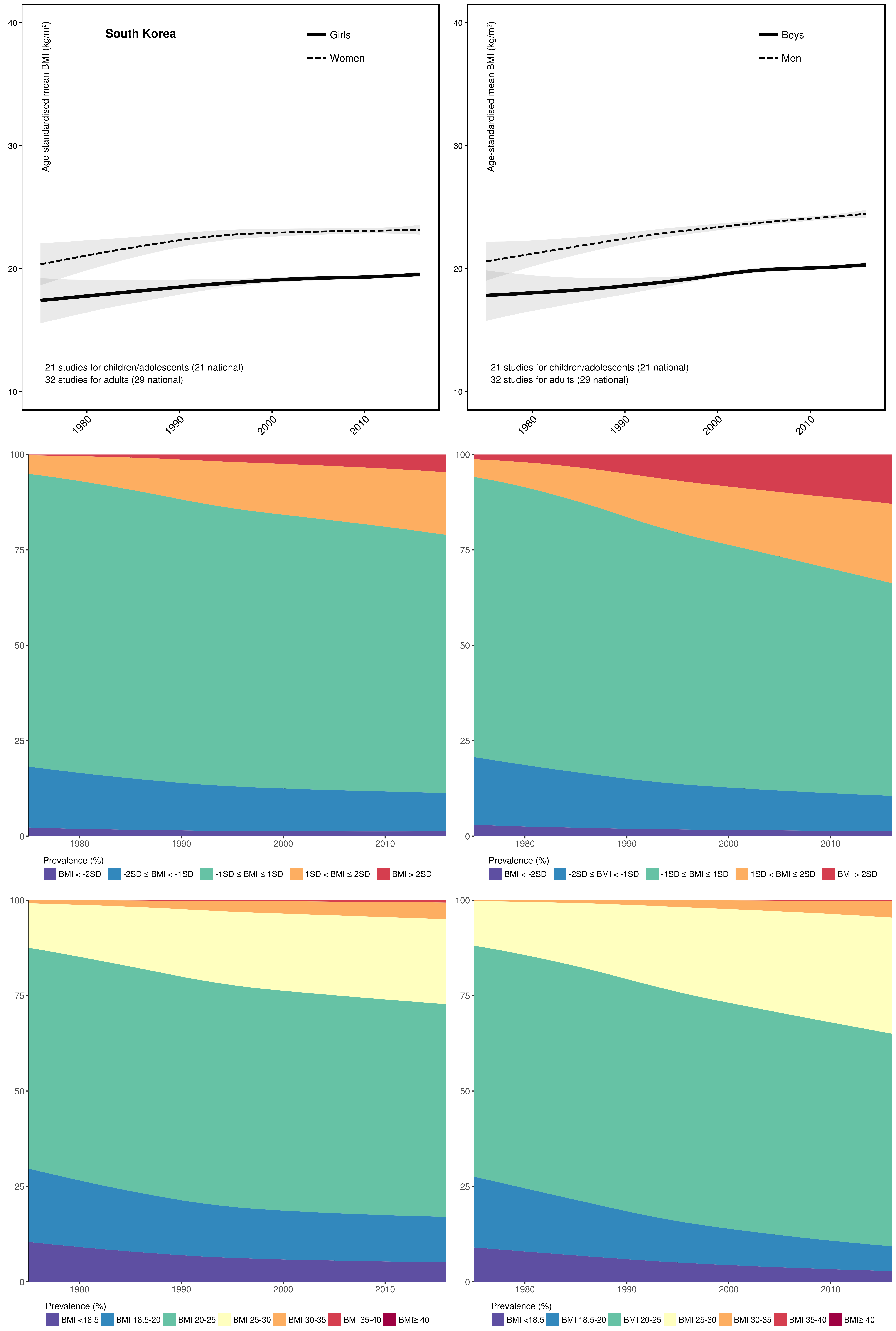
South Korea
Girls
Women
Boys
Men
Age-standardised mean BMI (kg/m²)
21 studies for children/adolescents (21 national)
32 studies for adults (29 national)
1980
1990
2000
2010
Prevalence (%)
BMI < -2SD
-2SD ≤ BMI < -1SD
-1SD ≤ BMI ≤ 1SD
1SD < BMI ≤ 2SD
BMI > 2SD
BMI <18.5
BMI 18.5-20
BMI 20-25
BMI 25-30
BMI 30-35
BMI 35-40
BMI≥ 40

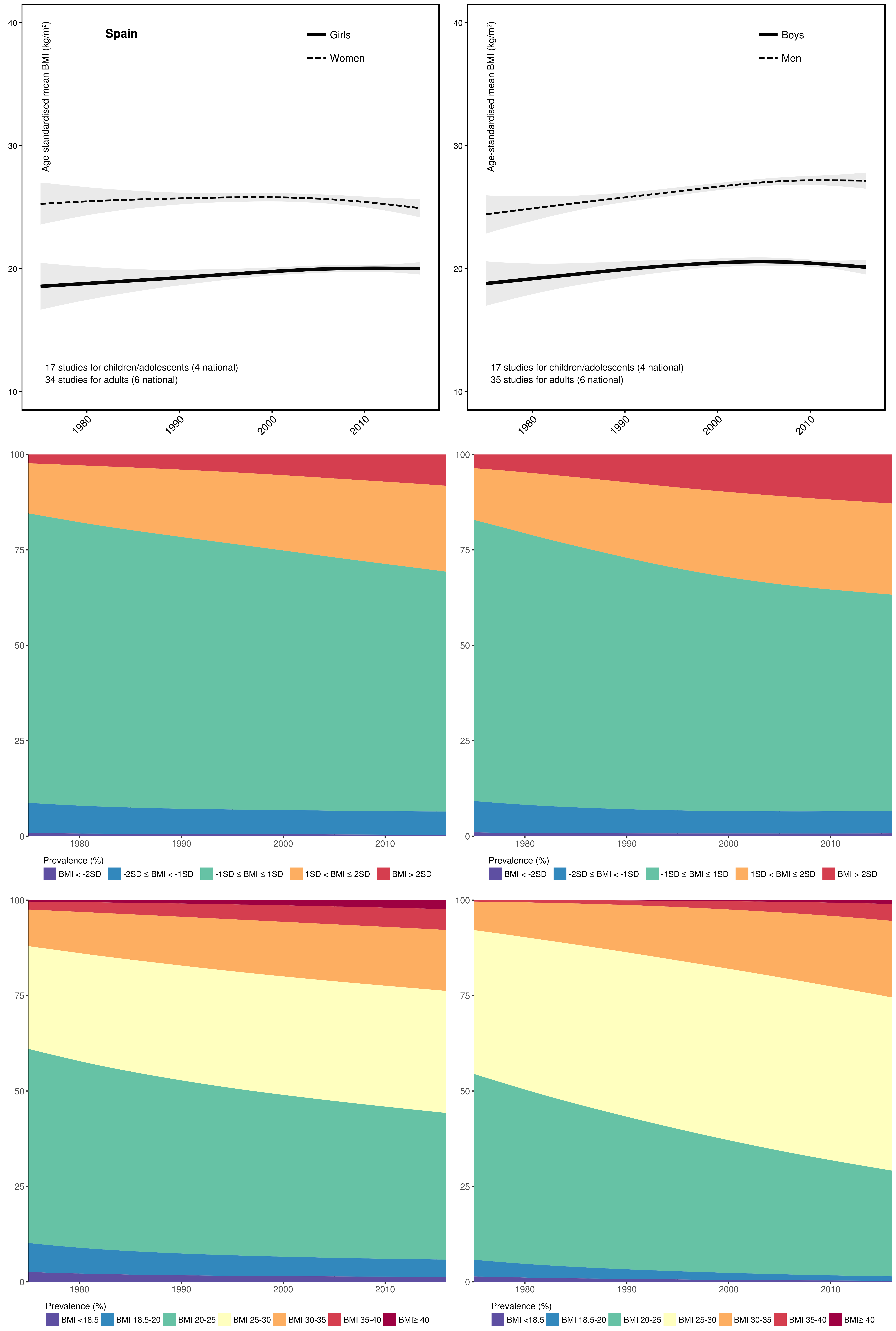
Spain
Girls
Women
Boys
Men
Age-standardised mean BMI (kg/m²)
17 studies for children/adolescents (4 national)
34 studies for adults (6 national)
17 studies for children/adolescents (4 national)
35 studies for adults (6 national)
Prevalence (%)
BMI < -2SD
-2SD ≤ BMI < -1SD
-1SD ≤ BMI ≤ 1SD
1SD < BMI ≤ 2SD
BMI > 2SD
BMI <18.5
BMI 18.5-20
BMI 20-25
BMI 25-30
BMI 30-35
BMI 35-40
BMI≥ 40

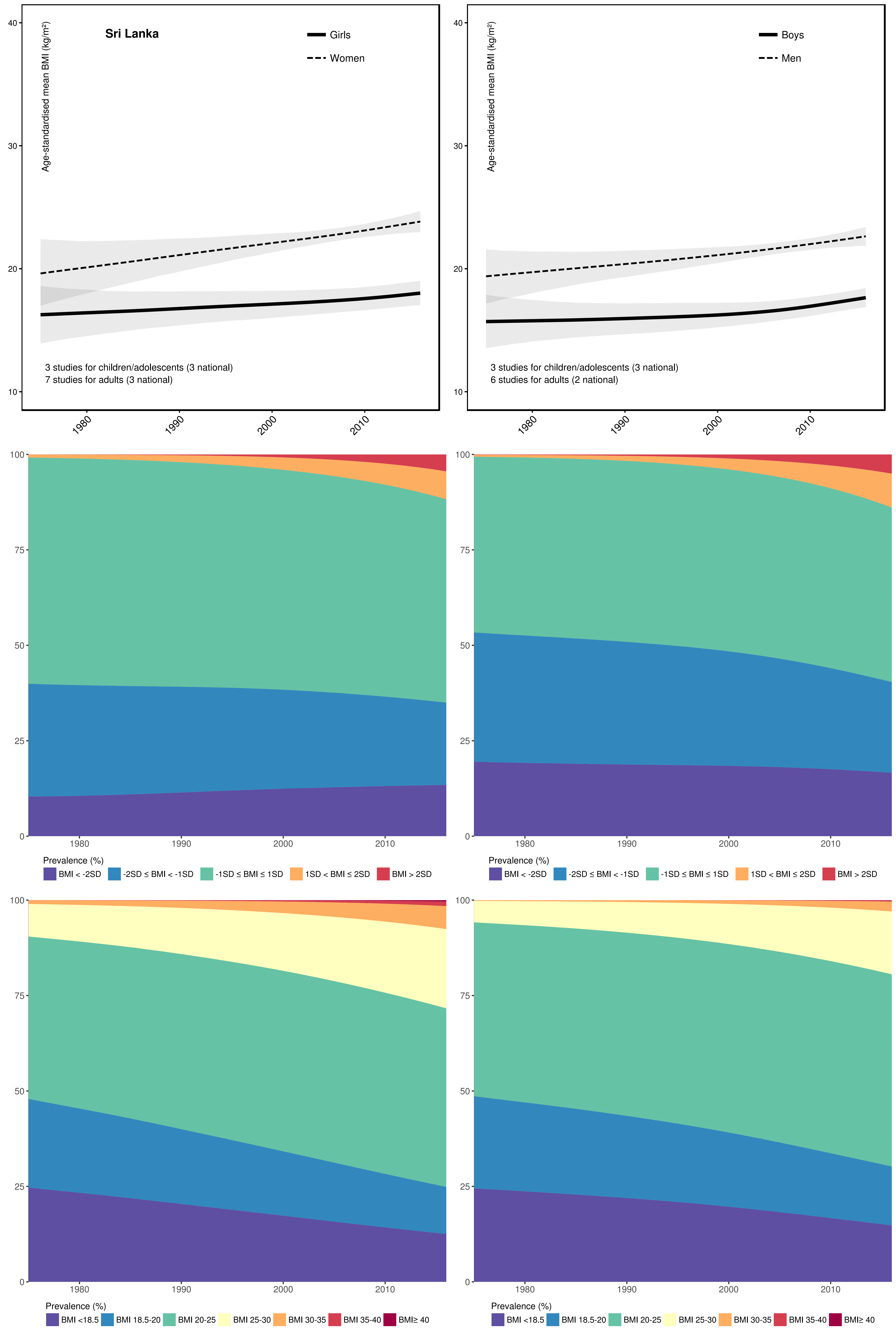
Sri Lanka
Girls
Women
Boys
Men
Age-standardised mean BMI (kg/m²)
3 studies for children/adolescents (3 national)
7 studies for adults (3 national)
3 studies for children/adolescents (3 national)
6 studies for adults (2 national)
Prevalence (%)
BMI < -2SD
-2SD ≤ BMI < -1SD
-1SD ≤ BMI ≤ 1SD
1SD < BMI ≤ 2SD
BMI > 2SD
BMI <18.5
BMI 18.5-20
BMI 20-25
BMI 25-30
BMI 30-35
BMI 35-40
BMI≥ 40

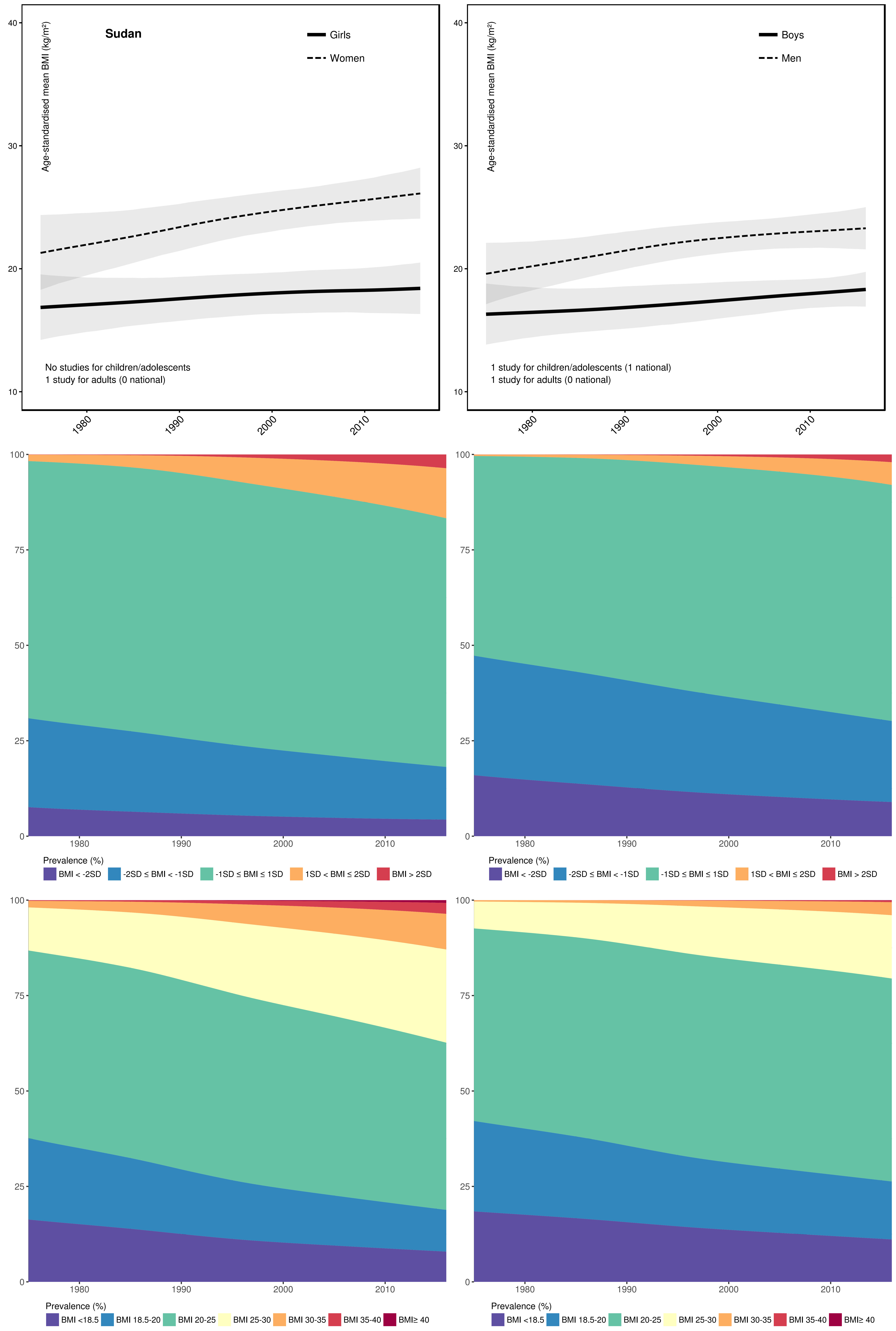
Sudan
Girls
Women
Boys
Men
Age-standardised mean BMI (kg/m²)
No studies for children/adolescents
1 study for adults (0 national)
1 study for children/adolescents (1 national)
1 study for adults (0 national)
Prevalence (%)
BMI < -2SD
-2SD ≤ BMI < -1SD
-1SD ≤ BMI ≤ 1SD
1SD < BMI ≤ 2SD
BMI > 2SD
BMI <18.5
BMI 18.5-20
BMI 20-25
BMI 25-30
BMI 30-35
BMI 35-40
BMI≥ 40

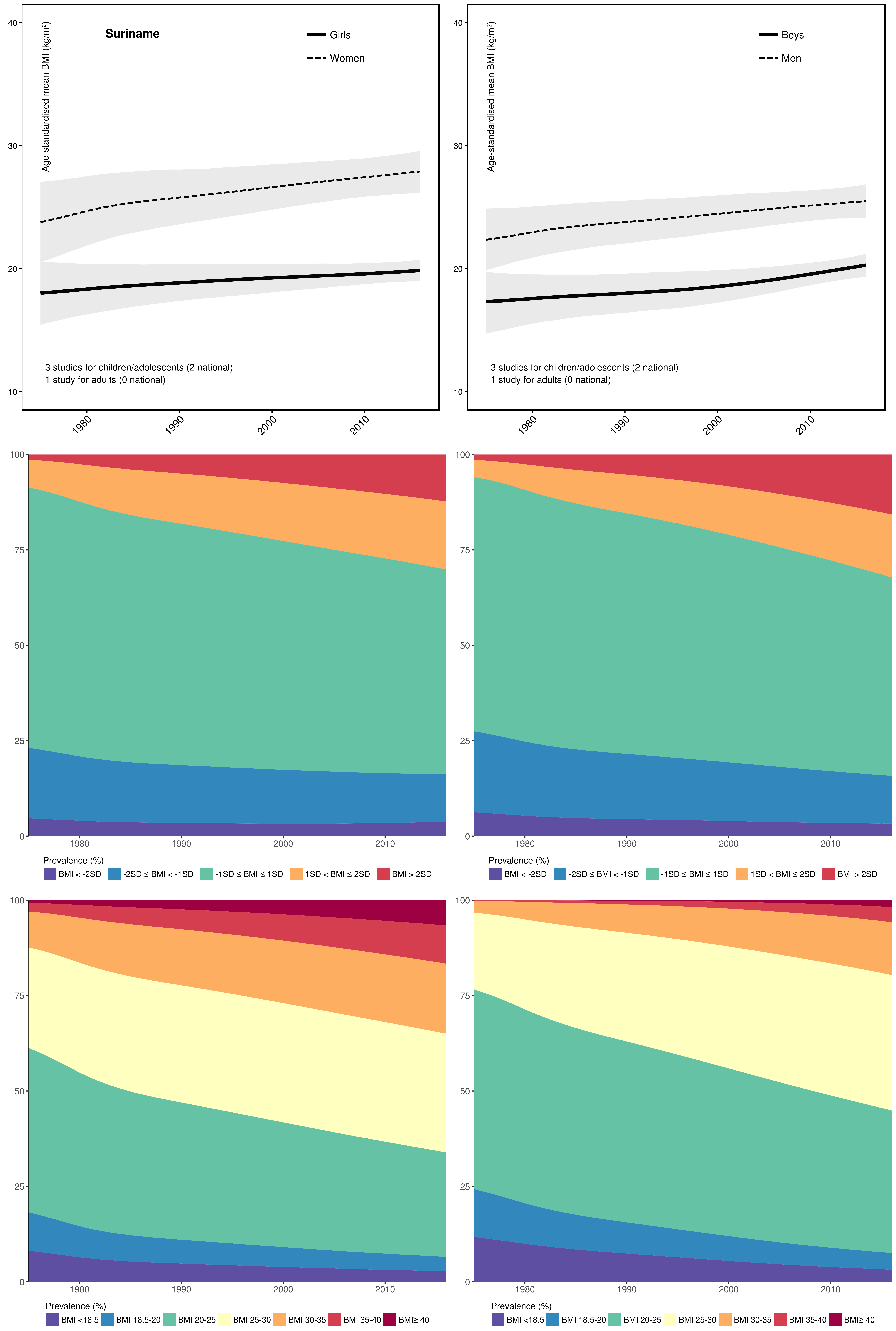

Suriname
Girls
Women
Boys
Men
Age-standardised mean BMI (kg/m²)
3 studies for children/adolescents (2 national)
1 study for adults (0 national)
3 studies for children/adolescents (2 national)
1 study for adults (0 national)
Prevalence (%)
BMI < -2SD
-2SD ≤ BMI < -1SD
-1SD ≤ BMI ≤ 1SD
1SD < BMI ≤ 2SD
BMI > 2SD
Prevalence (%)
BMI <18.5
BMI 18.5-20
BMI 20-25
BMI 25-30
BMI 30-35
BMI 35-40
BMI≥ 40

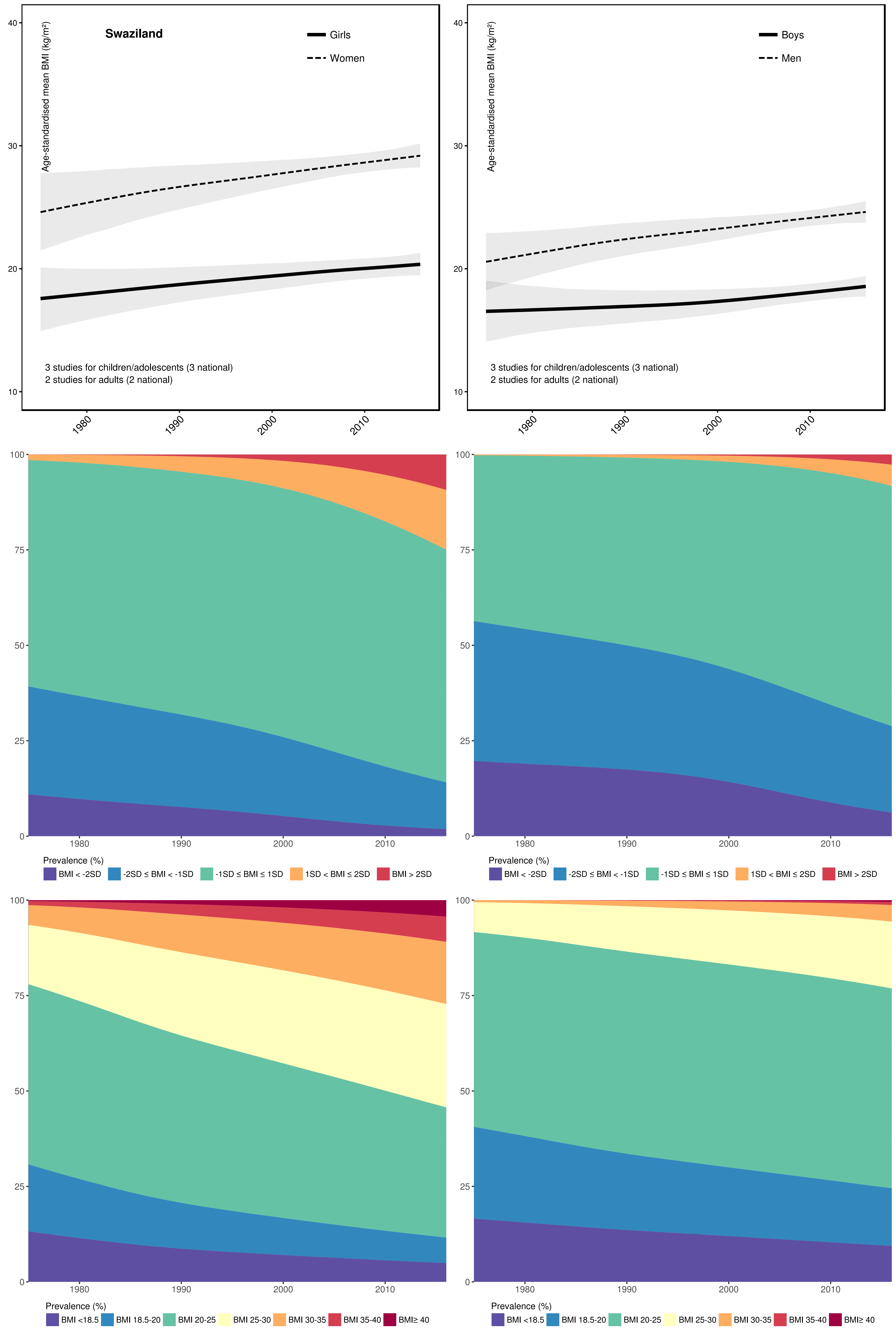
Swaziland
Girls
Women
Boys
Men
Age-standardised mean BMI (kg/m²)
3 studies for children/adolescents (3 national)
2 studies for adults (2 national)
Prevalence (%)
BMI < -2SD
-2SD ≤ BMI < -1SD
-1SD ≤ BMI ≤ 1SD
1SD < BMI ≤ 2SD
BMI > 2SD
BMI <18.5
BMI 18.5-20
BMI 20-25
BMI 25-30
BMI 30-35
BMI 35-40
BMI≥ 40

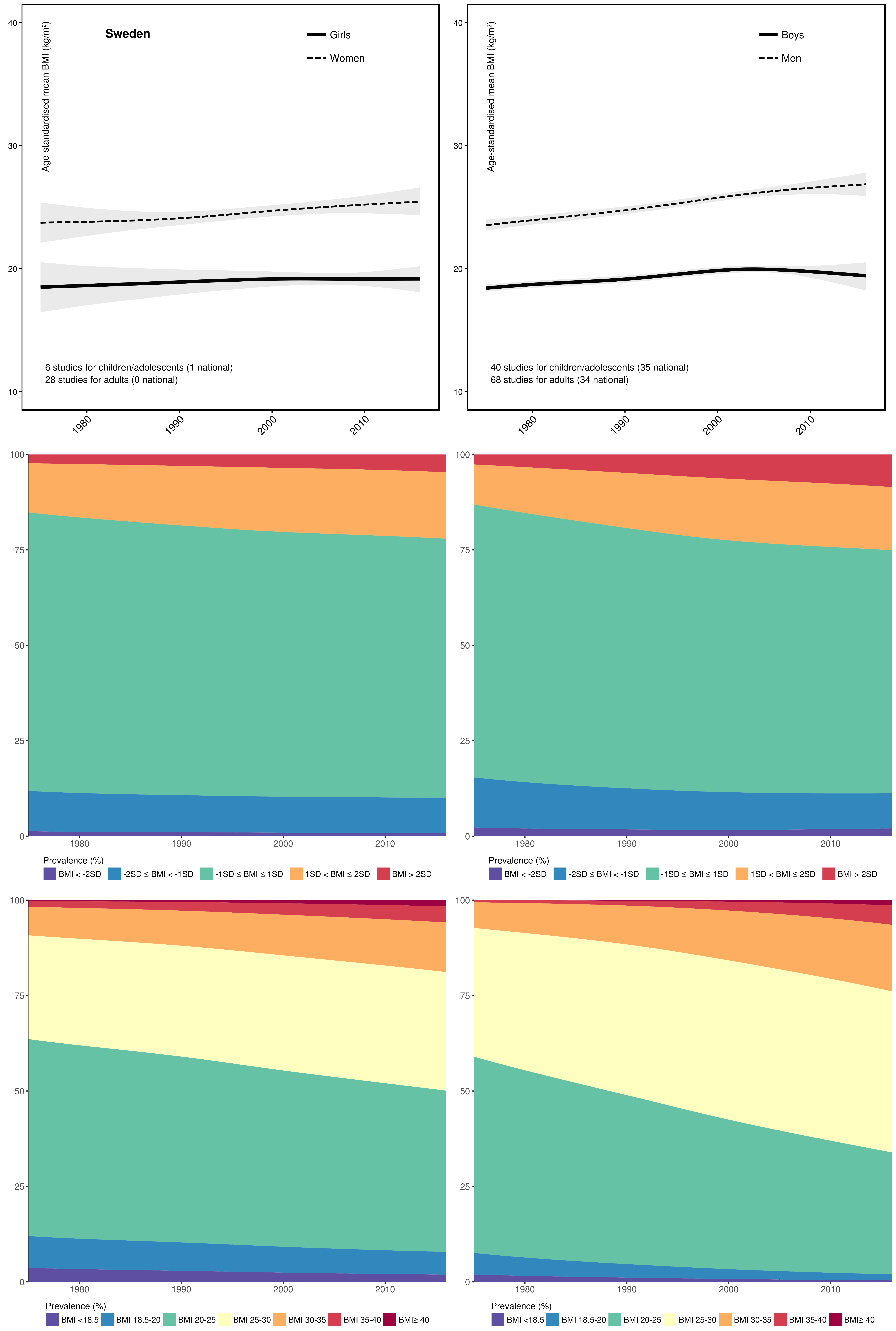
Sweden
Girls
Women
Age-standardised mean BMI (kg/m²)
6 studies for children/adolescents (1 national)
28 studies for adults (0 national)
Boys
Men
Age-standardised mean BMI (kg/m²)
40 studies for children/adolescents (35 national)
68 studies for adults (34 national)
10
20
30
40
1980
1990
2000
2010
0
25
50
75
100
Prevalence (%)
BMI < -2SD
-2SD ≤ BMI < -1SD
-1SD ≤ BMI ≤ 1SD
1SD < BMI ≤ 2SD
BMI > 2SD
Prevalence (%)
BMI <18.5
BMI 18.5-20
BMI 20-25
BMI 25-30
BMI 30-35
BMI 35-40
BMI≥ 40

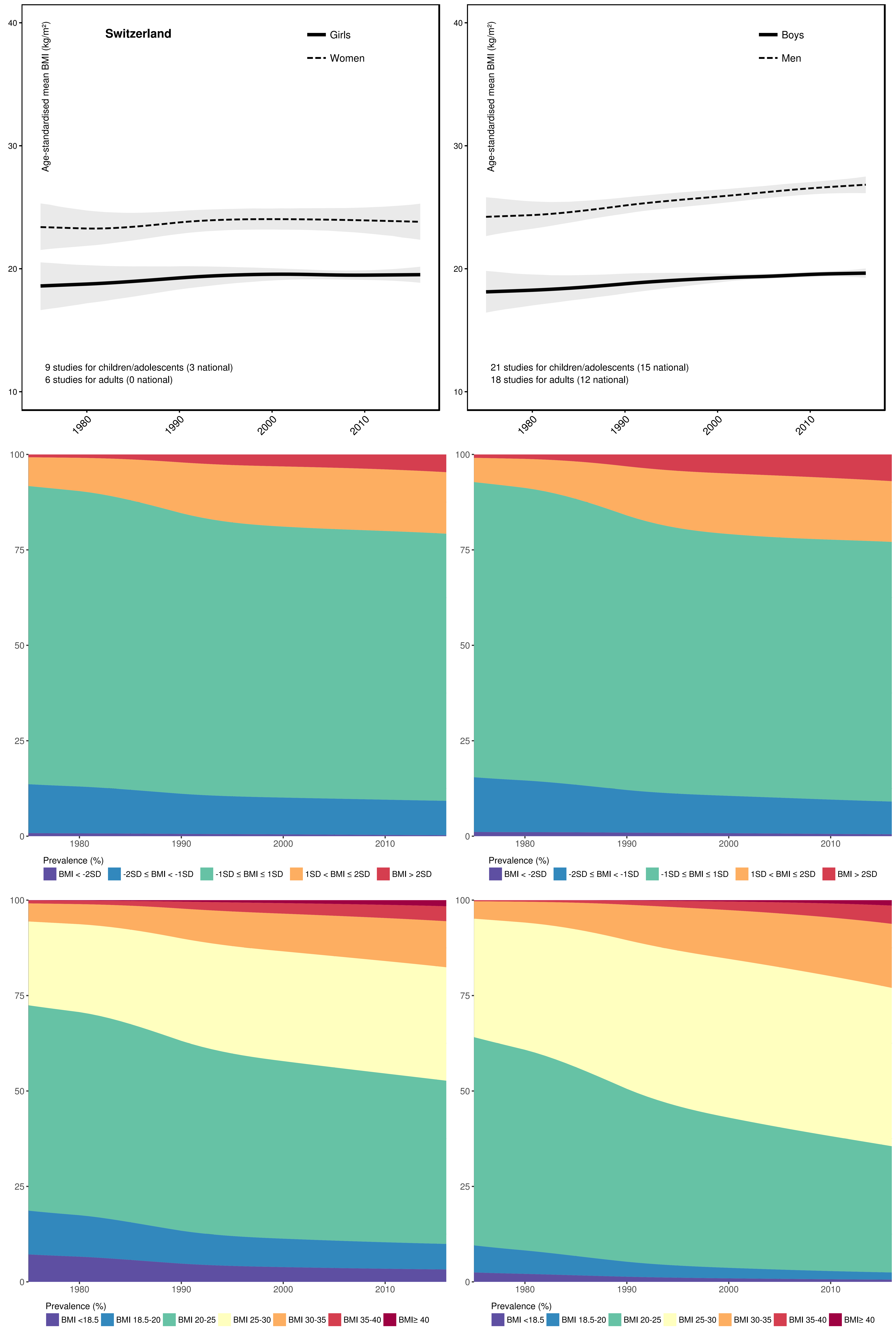
Switzerland
Girls
Women
Boys
Men
Age-standardised mean BMI (kg/m²)
9 studies for children/adolescents (3 national)
6 studies for adults (0 national)
21 studies for children/adolescents (15 national)
18 studies for adults (12 national)
Prevalence (%)
BMI < -2SD
-2SD ≤ BMI < -1SD
-1SD ≤ BMI ≤ 1SD
1SD < BMI ≤ 2SD
BMI > 2SD
BMI <18.5
BMI 18.5-20
BMI 20-25
BMI 25-30
BMI 30-35
BMI 35-40
BMI≥ 40

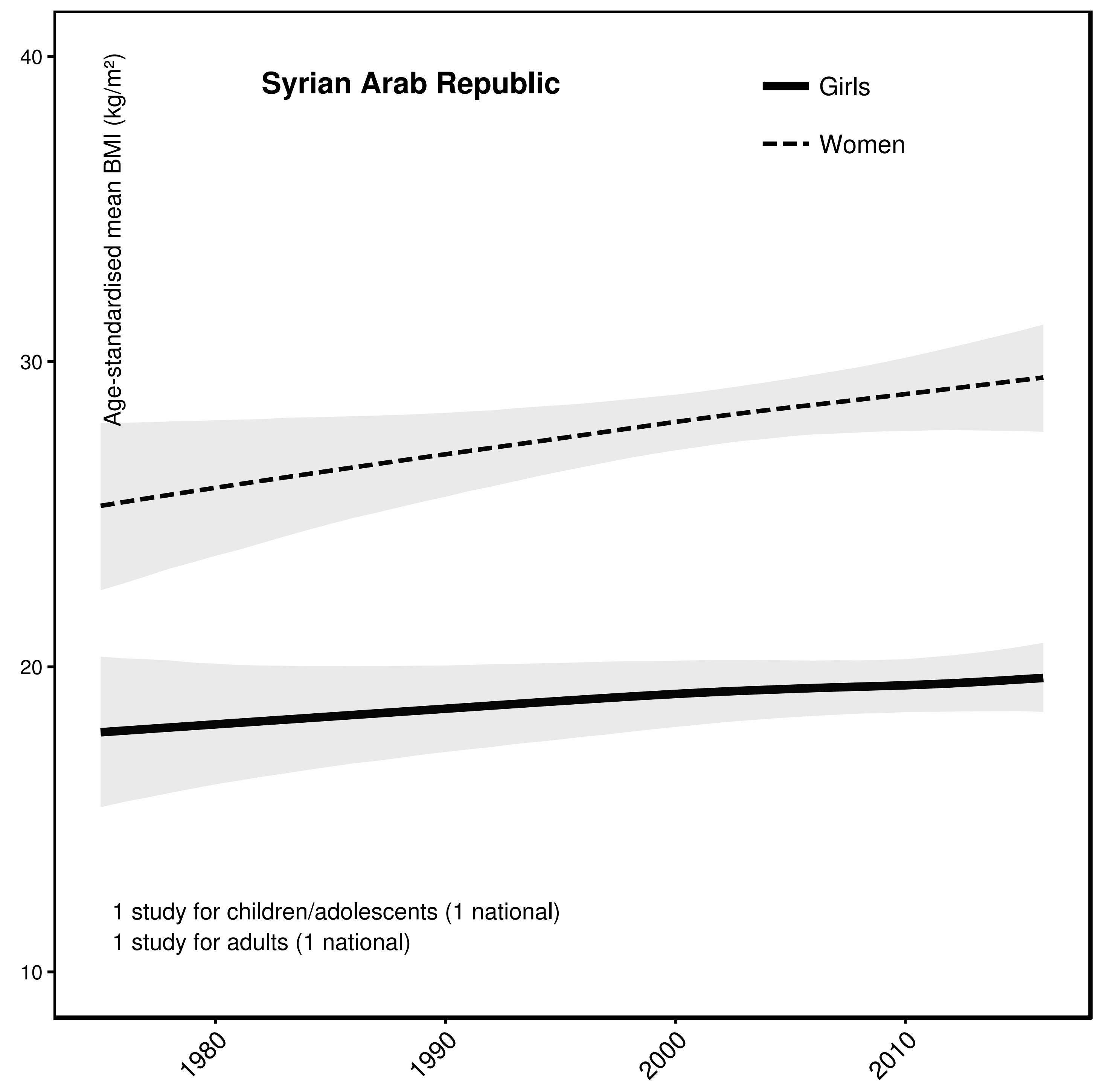
Syrian Arab Republic
Girls
Women
Age-standardised mean BMI (kg/m²)
1 study for children/adolescents (1 national)
1 study for adults (1 national)
40
30
20
10
1980
1990
2000
2010

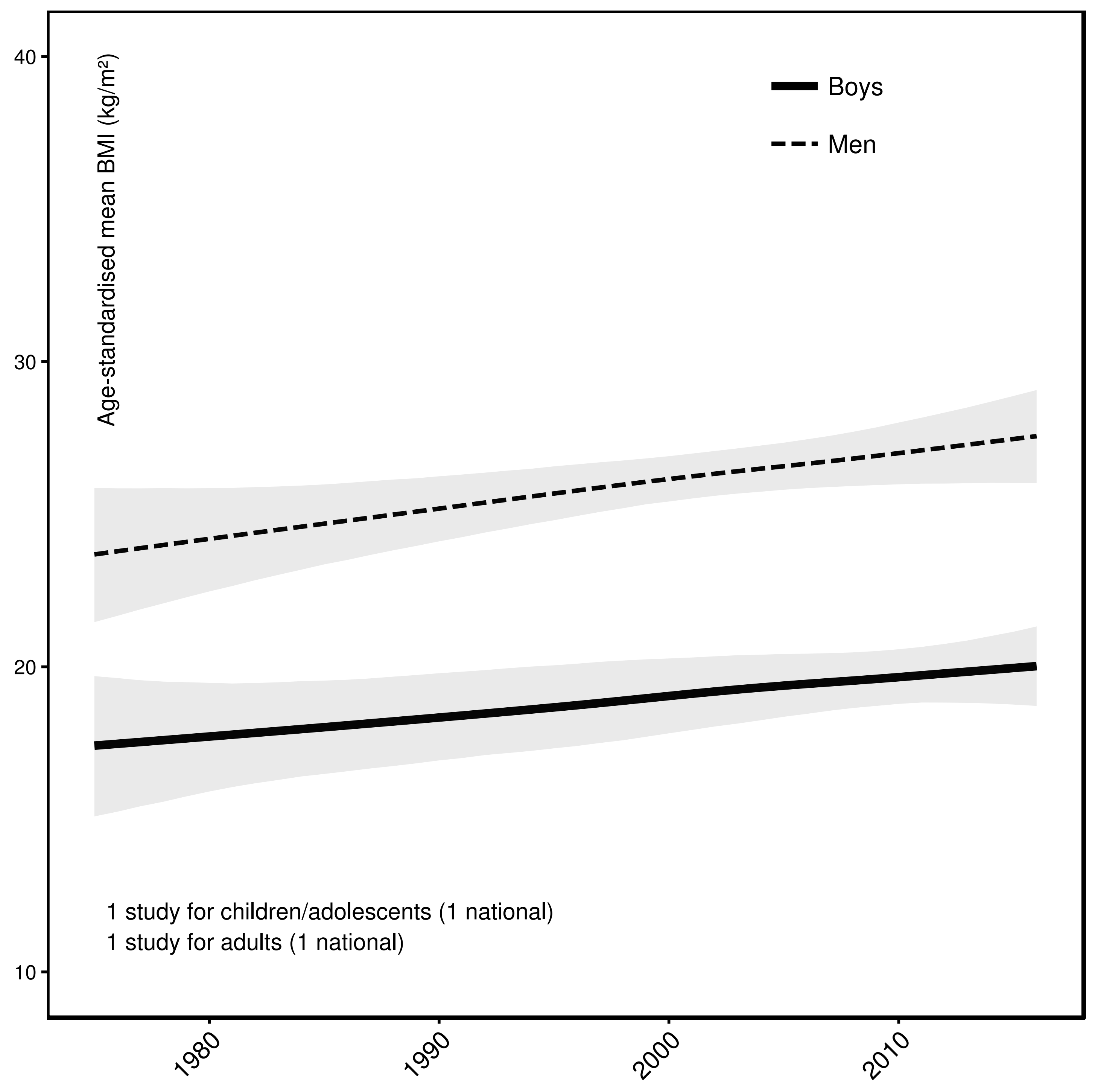
Boys
Men
Age-standardised mean BMI (kg/m²)
1 study for children/adolescents (1 national)
1 study for adults (1 national)
40
30
20
10
1980
1990
2000
2010

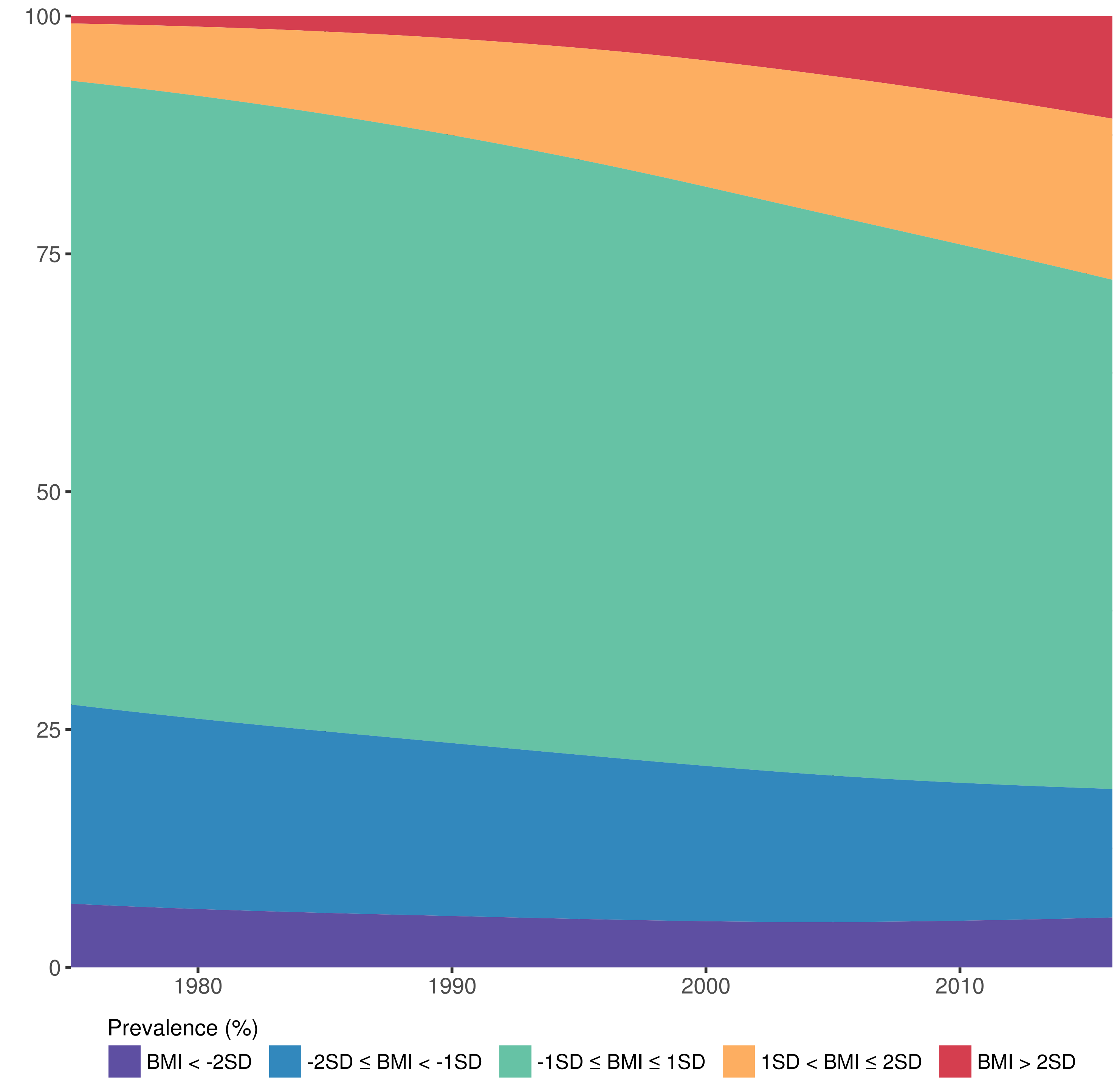
100
75
50
25
0
1980
1990
2000
2010
Prevalence (%)
BMI < -2SD
-2SD ≤ BMI < -1SD
-1SD ≤ BMI ≤ 1SD
1SD < BMI ≤ 2SD
BMI > 2SD

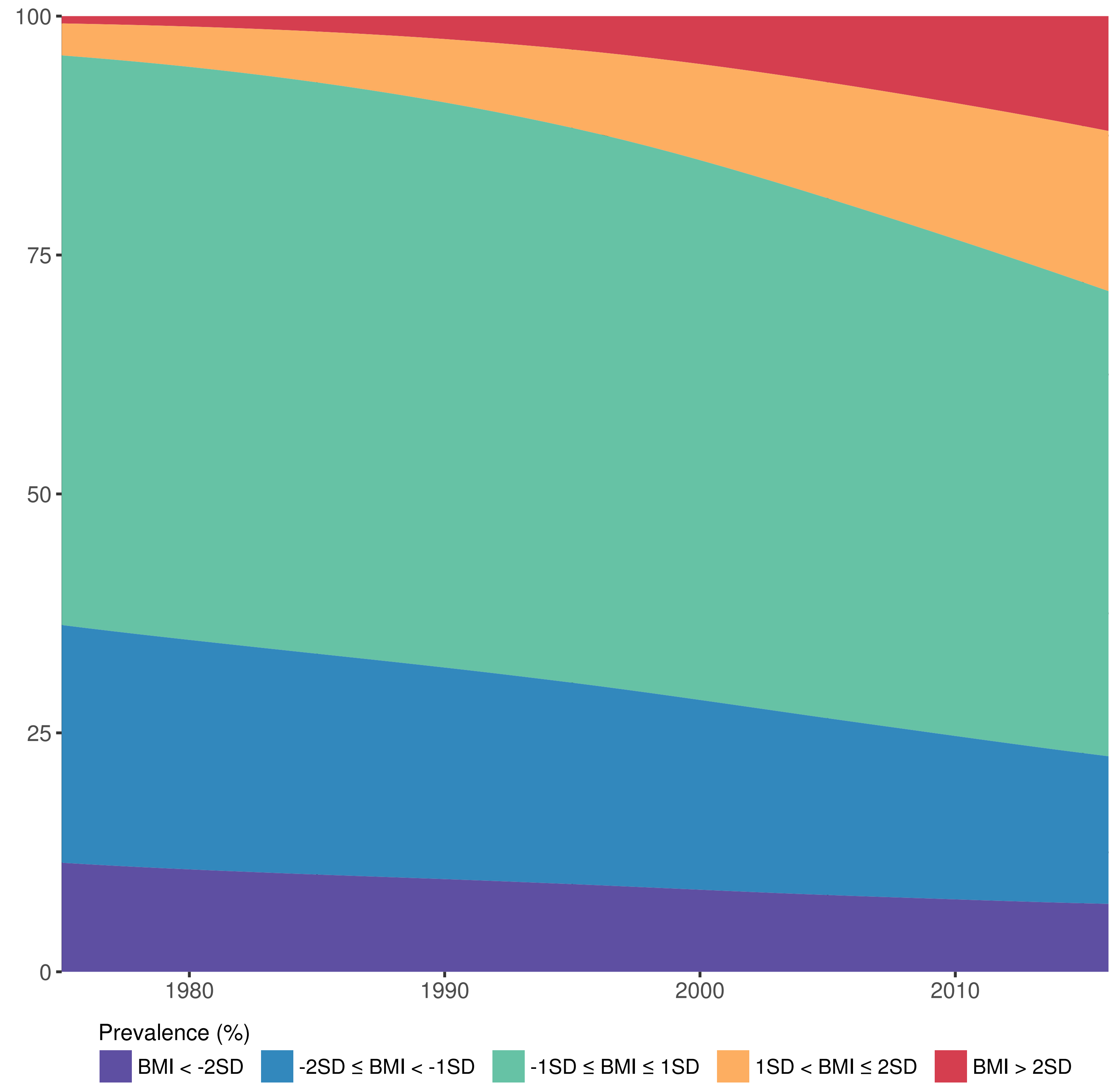
100
75
50
25
0
1980
1990
2000
2010
Prevalence (%)
BMI < -2SD
-2SD ≤ BMI < -1SD
-1SD ≤ BMI ≤ 1SD
1SD < BMI ≤ 2SD
BMI > 2SD

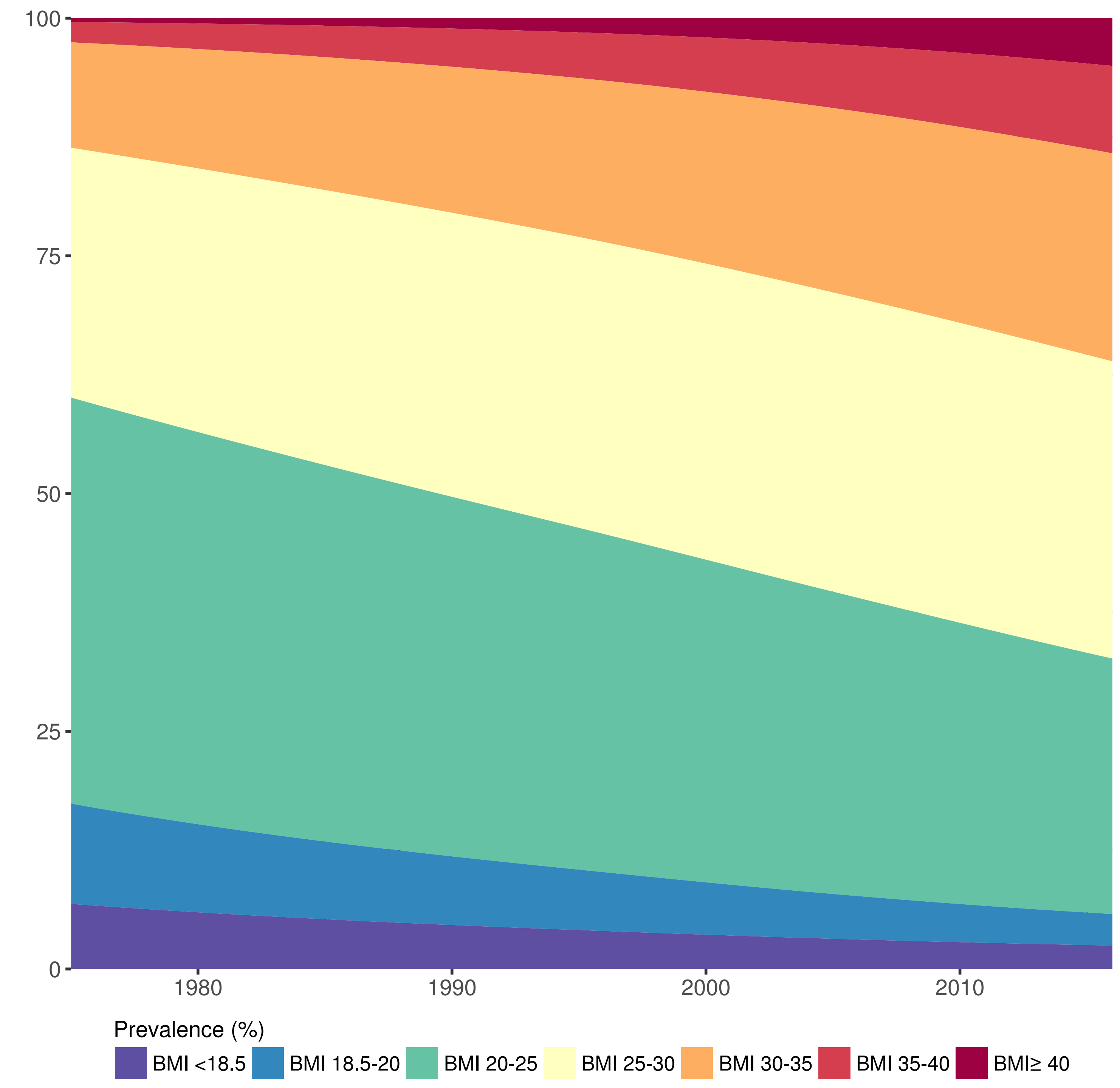
100
75
50
25
0
1980
1990
2000
2010
Prevalence (%)
BMI <18.5
BMI 18.5-20
BMI 20-25
BMI 25-30
BMI 30-35
BMI 35-40
BMI≥ 40

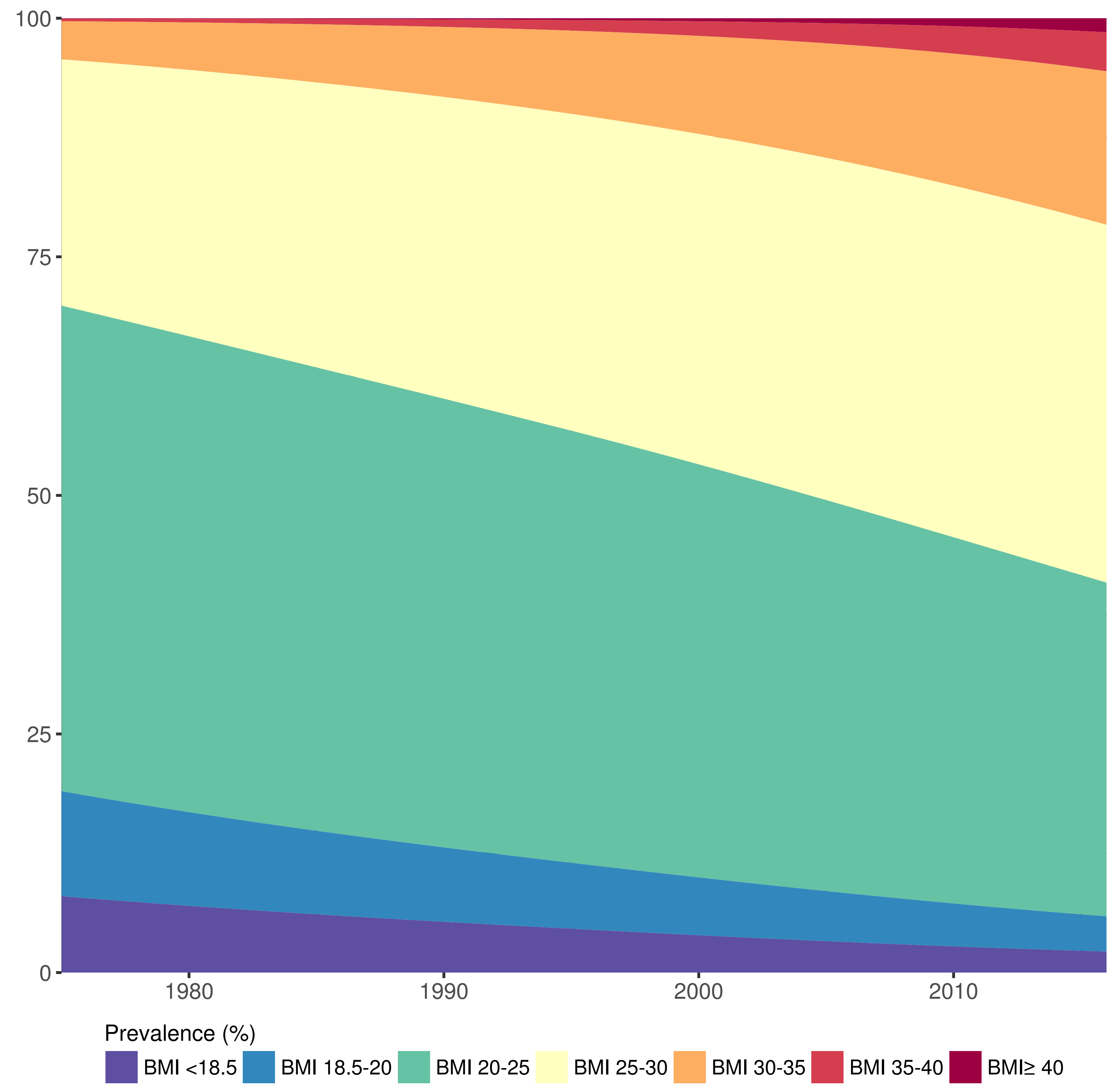
100
75
50
25
0
1980
1990
2000
2010
Prevalence (%)
BMI <18.5
BMI 18.5-20
BMI 20-25
BMI 25-30
BMI 30-35
BMI 35-40
BMI≥ 40

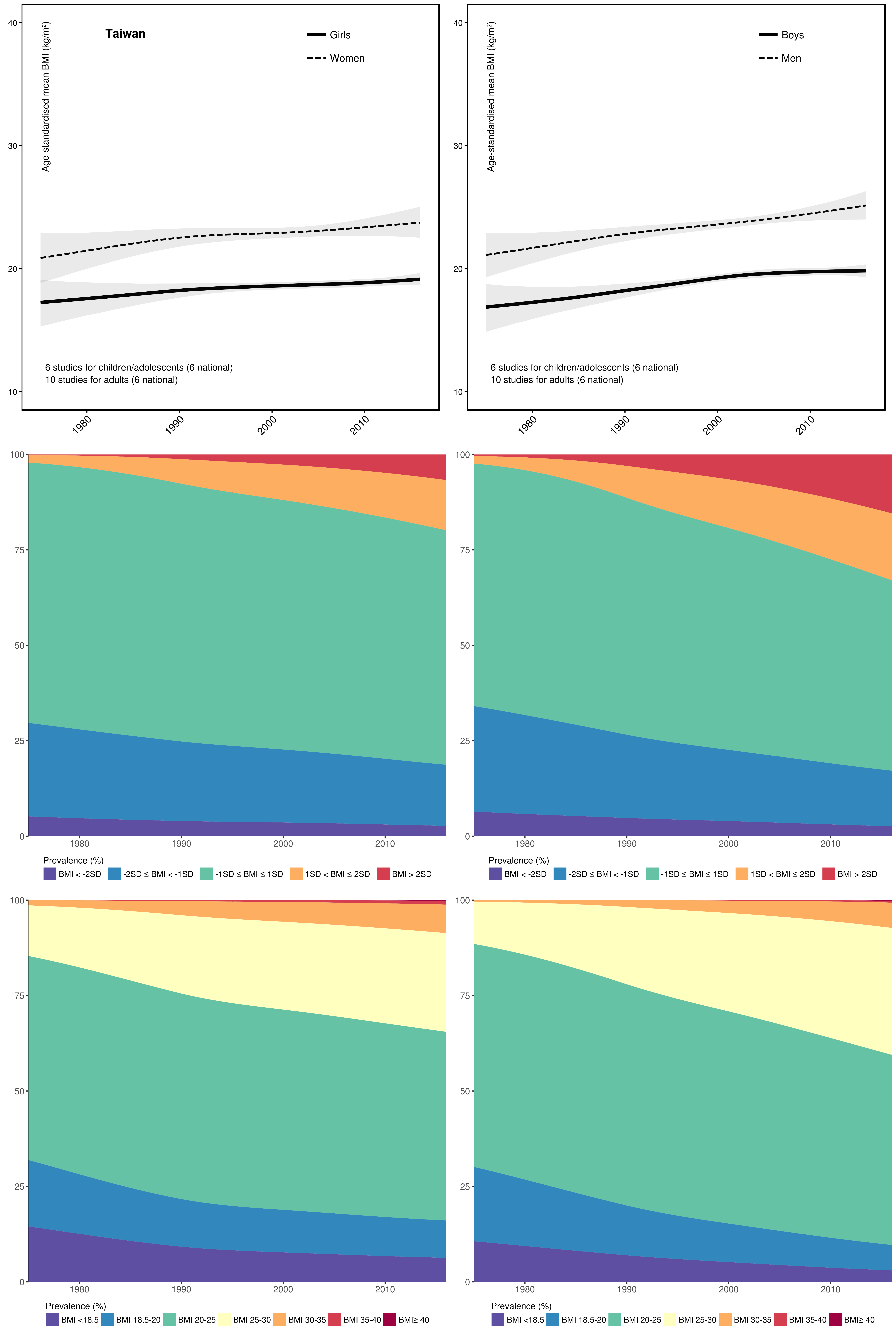
Taiwan
Girls
Women
Boys
Men
Age-standardised mean BMI (kg/m²)
6 studies for children/adolescents (6 national)
10 studies for adults (6 national)
Prevalence (%)
BMI < -2SD
-2SD ≤ BMI < -1SD
-1SD ≤ BMI ≤ 1SD
1SD < BMI ≤ 2SD
BMI > 2SD
BMI <18.5
BMI 18.5-20
BMI 20-25
BMI 25-30
BMI 30-35
BMI 35-40
BMI≥ 40

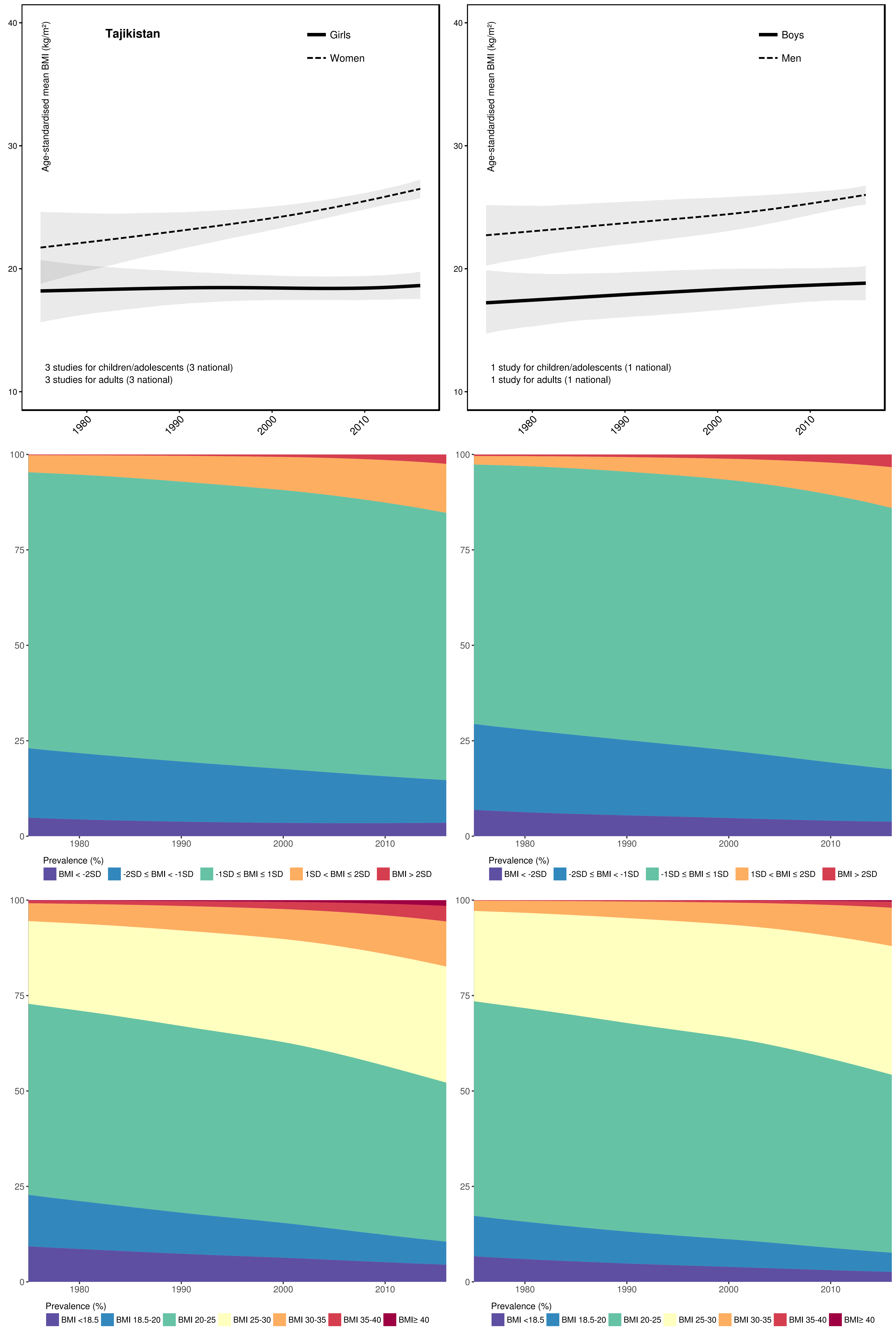
Tajikistan
Girls
Women
Boys
Men
Age-standardised mean BMI (kg/m²)
3 studies for children/adolescents (3 national)
3 studies for adults (3 national)
1 study for children/adolescents (1 national)
1 study for adults (1 national)
Prevalence (%)
BMI < -2SD
-2SD ≤ BMI < -1SD
-1SD ≤ BMI ≤ 1SD
1SD < BMI ≤ 2SD
BMI > 2SD
BMI <18.5
BMI 18.5-20
BMI 20-25
BMI 25-30
BMI 30-35
BMI 35-40
BMI≥ 40

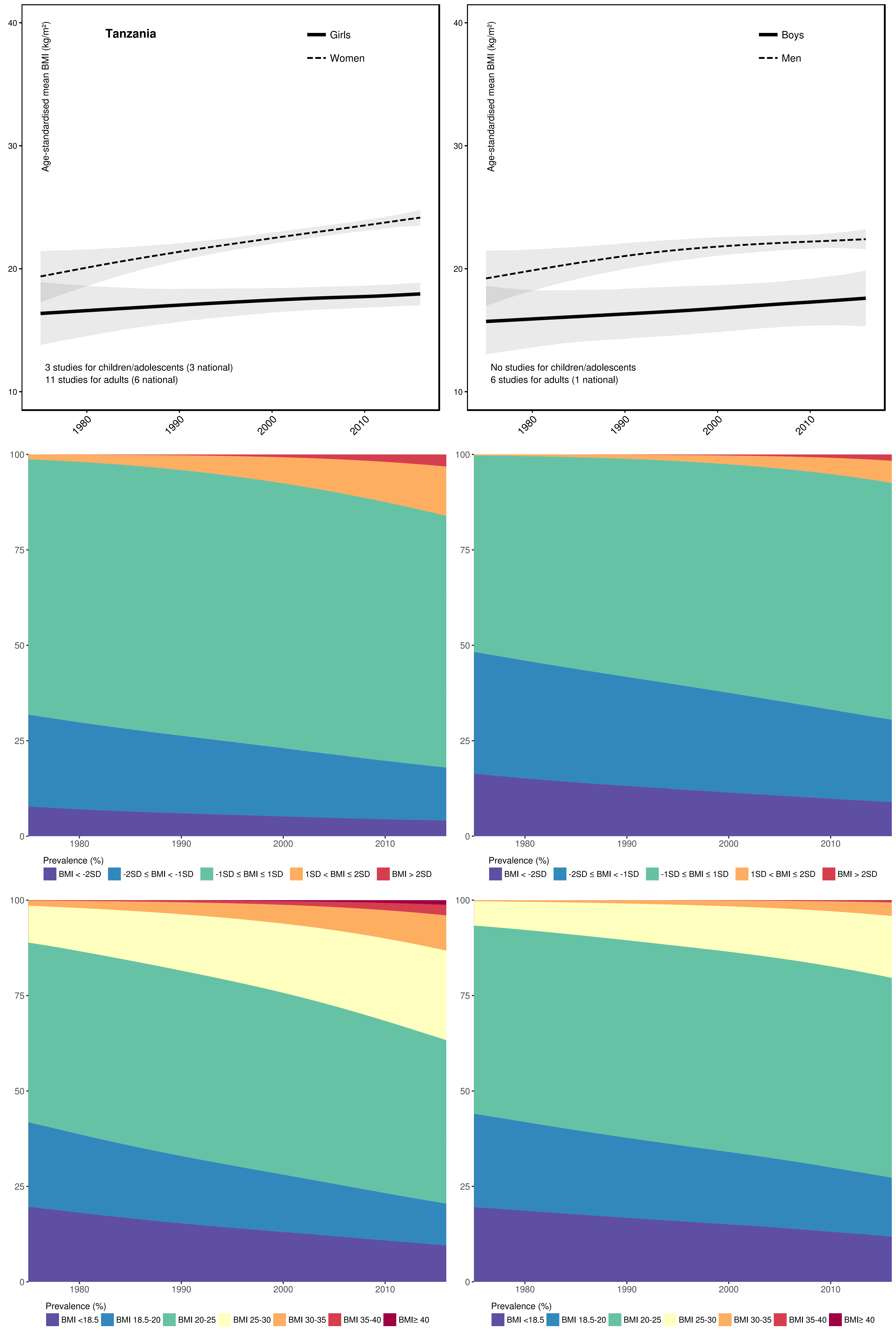
Tanzania
Girls
Women
Boys
Men
Age-standardised mean BMI (kg/m²)
3 studies for children/adolescents (3 national)
11 studies for adults (6 national)
No studies for children/adolescents
6 studies for adults (1 national)
Prevalence (%)
BMI < -2SD
-2SD ≤ BMI < -1SD
-1SD ≤ BMI ≤ 1SD
1SD < BMI ≤ 2SD
BMI > 2SD
BMI <18.5
BMI 18.5-20
BMI 20-25
BMI 25-30
BMI 30-35
BMI 35-40
BMI≥ 40

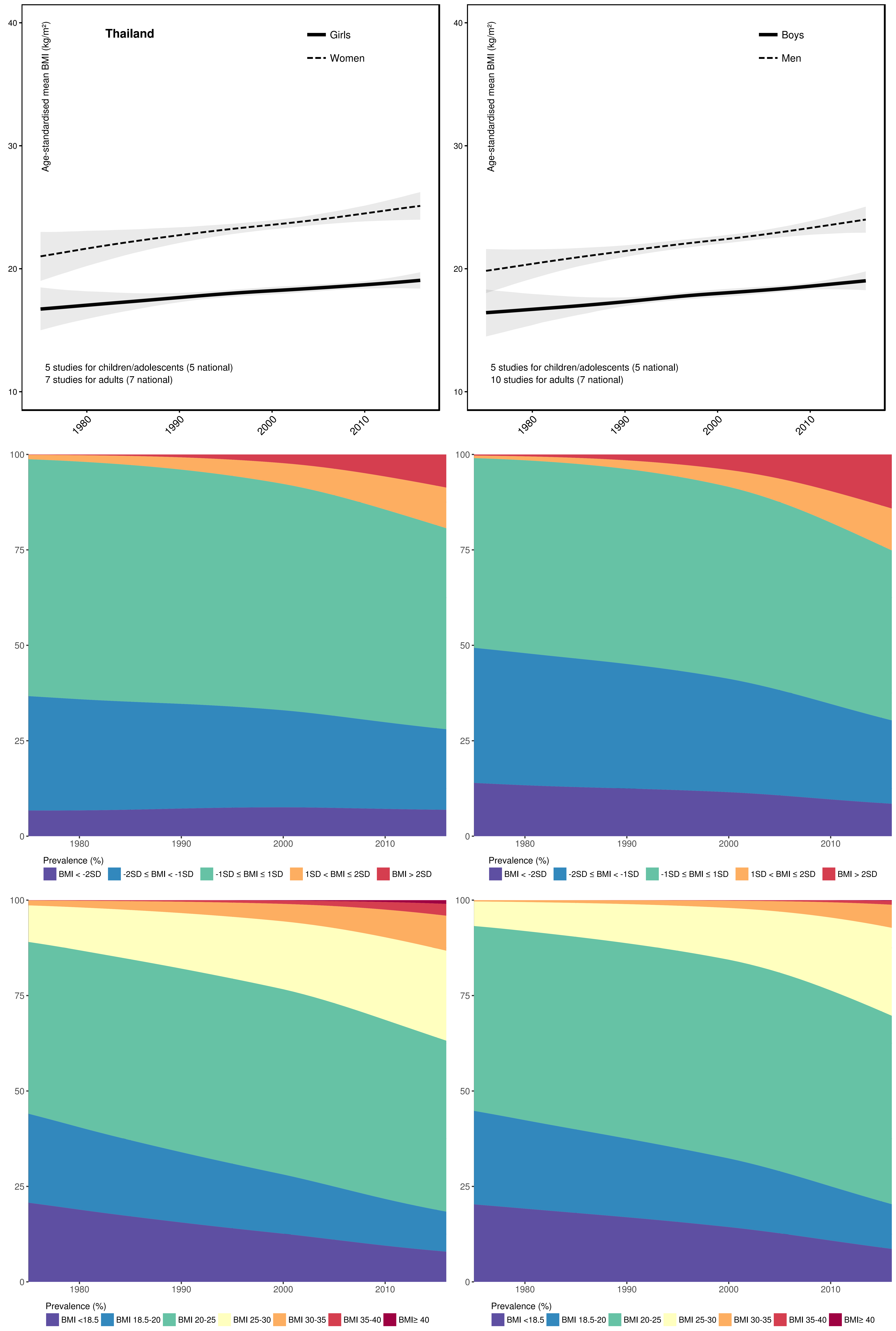
Thailand
Girls
Women
Boys
Men
Age-standardised mean BMI (kg/m²)
5 studies for children/adolescents (5 national)
7 studies for adults (7 national)
5 studies for children/adolescents (5 national)
10 studies for adults (7 national)
1980
1990
2000
2010
Prevalence (%)
BMI < -2SD
-2SD ≤ BMI < -1SD
-1SD ≤ BMI ≤ 1SD
1SD < BMI ≤ 2SD
BMI > 2SD
BMI <18.5
BMI 18.5-20
BMI 20-25
BMI 25-30
BMI 30-35
BMI 35-40
BMI≥ 40

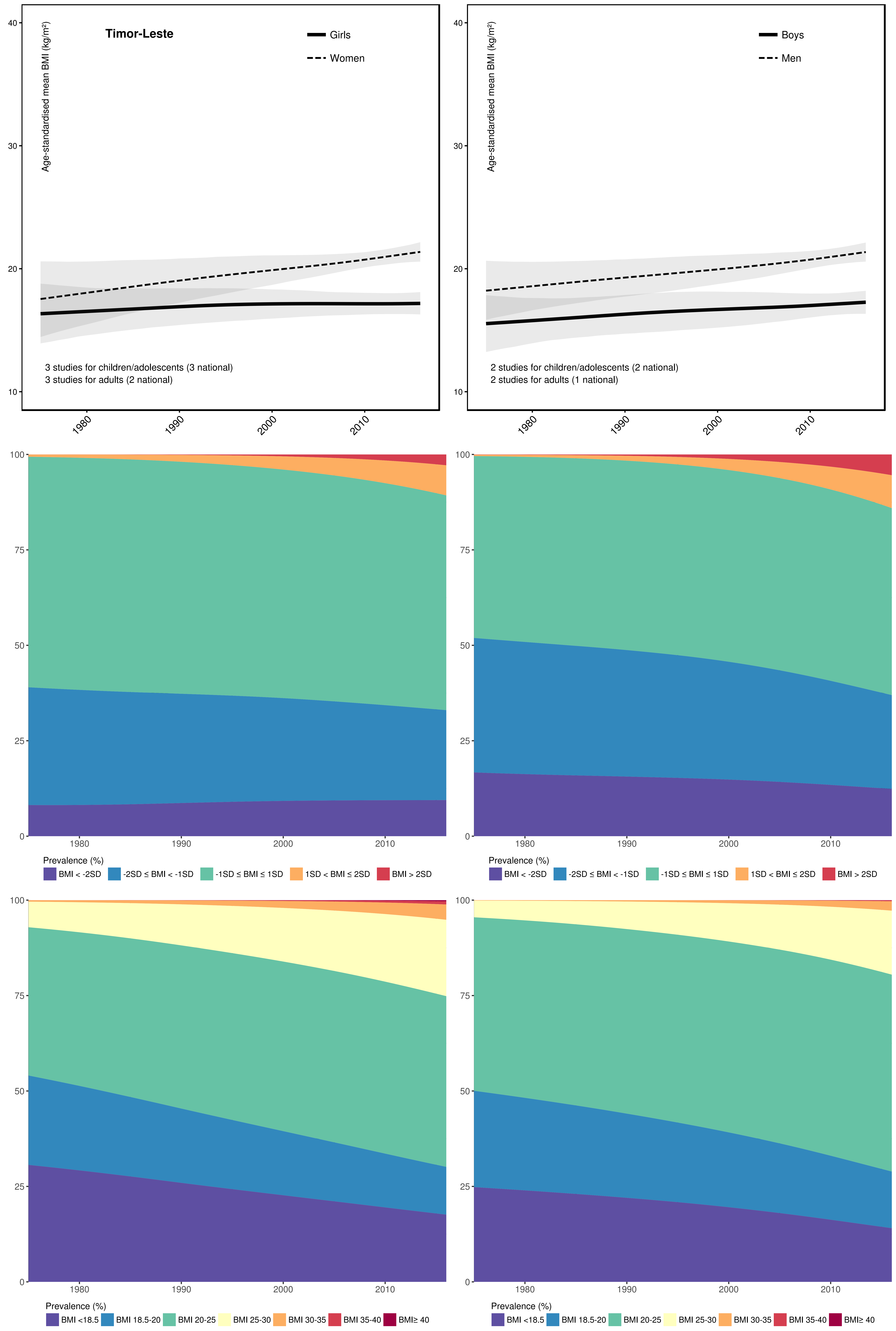

Timor-Leste
Girls
Women
Boys
Men
Age-standardised mean BMI (kg/m²)
40
30
20
10
1980
1990
2000
2010
3 studies for children/adolescents (3 national)
3 studies for adults (2 national)
2 studies for children/adolescents (2 national)
2 studies for adults (1 national)
100
75
50
25
0
Prevalence (%)
BMI < -2SD
-2SD ≤ BMI < -1SD
-1SD ≤ BMI ≤ 1SD
1SD < BMI ≤ 2SD
BMI > 2SD
BMI <18.5
BMI 18.5-20
BMI 20-25
BMI 25-30
BMI 30-35
BMI 35-40
BMI≥ 40

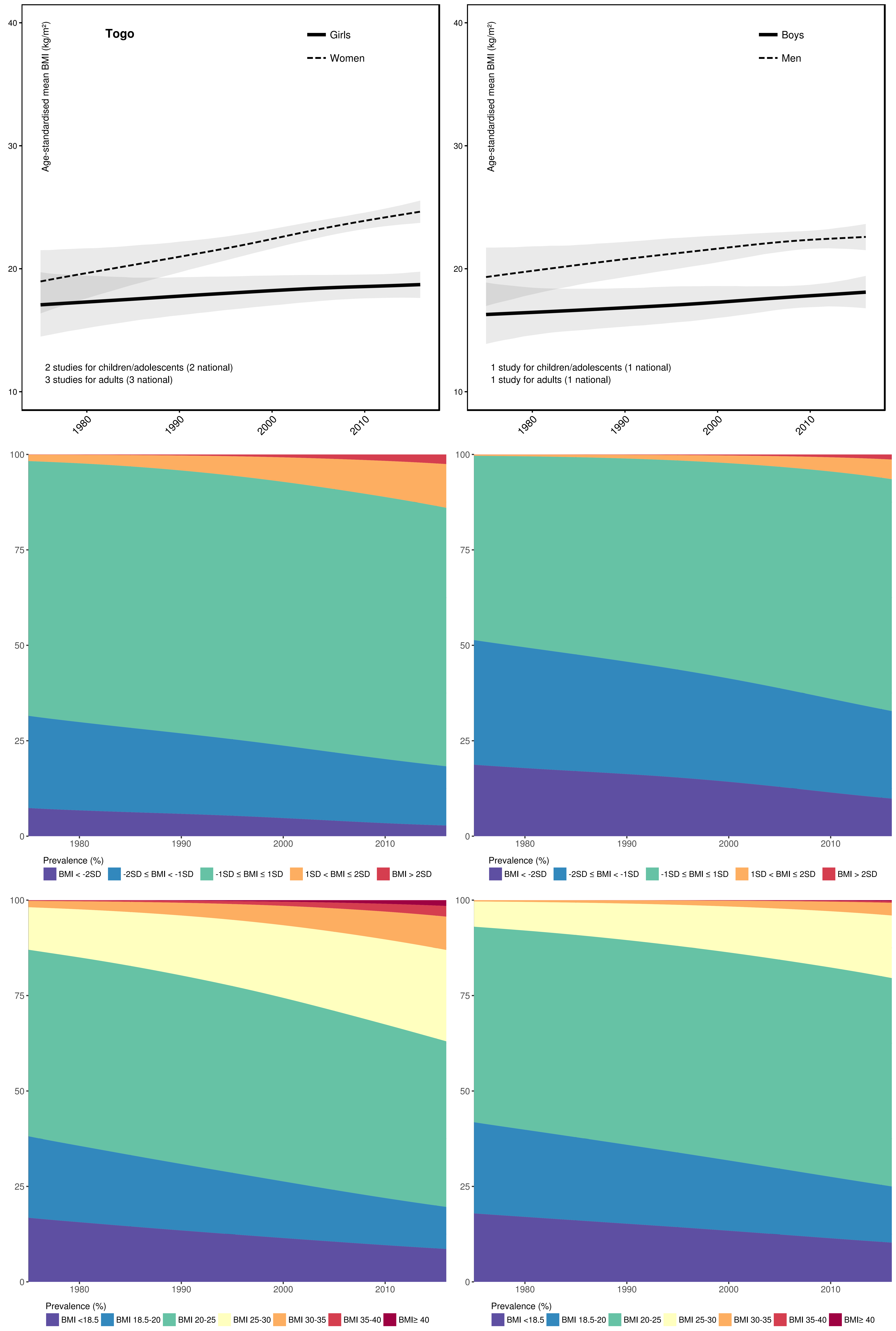
Togo
Girls
Women
Boys
Men
Age-standardised mean BMI (kg/m²)
2 studies for children/adolescents (2 national)
3 studies for adults (3 national)
1 study for children/adolescents (1 national)
1 study for adults (1 national)
Prevalence (%)
BMI < -2SD
-2SD ≤ BMI < -1SD
-1SD ≤ BMI ≤ 1SD
1SD < BMI ≤ 2SD
BMI > 2SD
BMI <18.5
BMI 18.5-20
BMI 20-25
BMI 25-30
BMI 30-35
BMI 35-40
BMI≥ 40

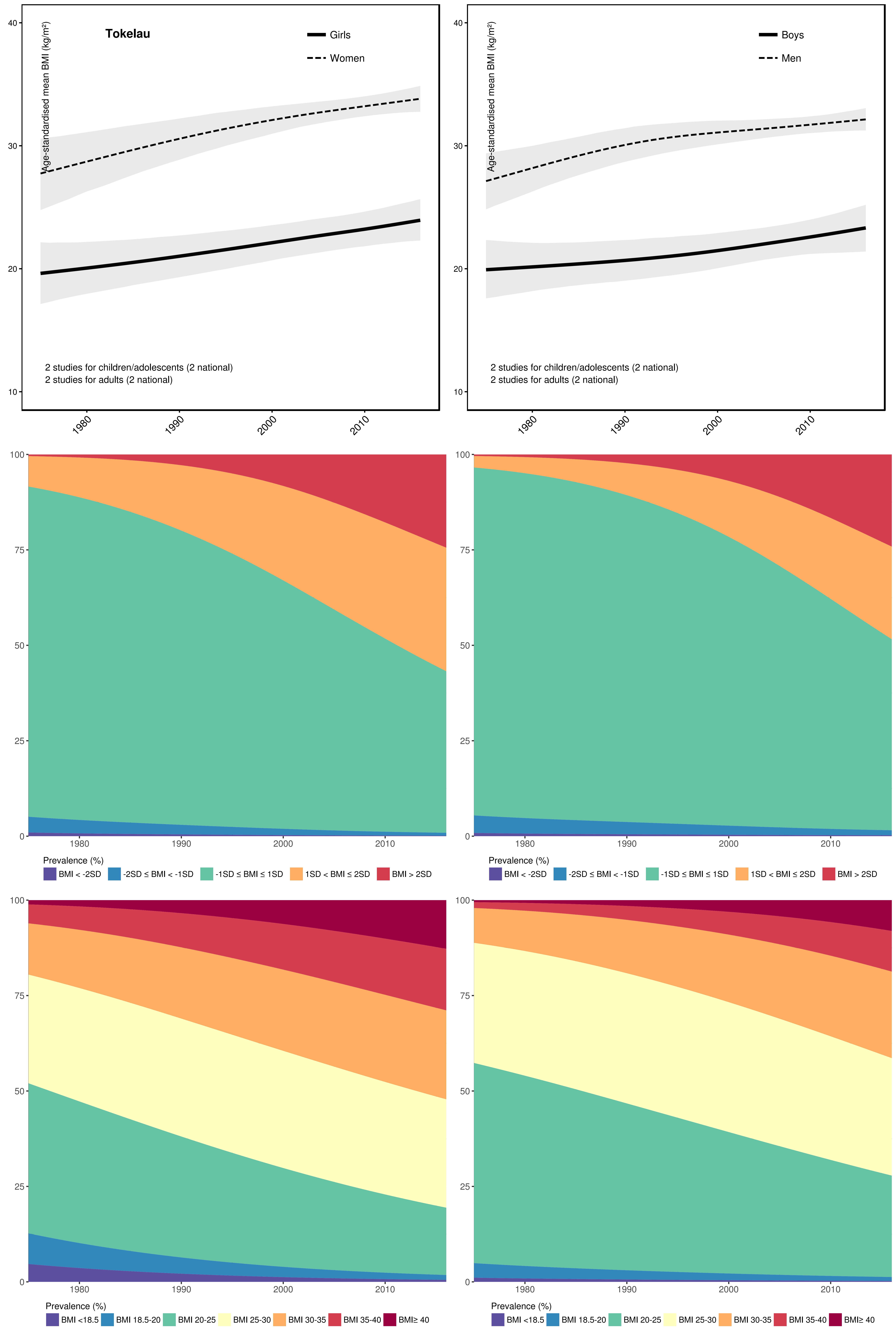
Tokelau
Girls
Women
Boys
Men
Age-standardised mean BMI (kg/m²)
2 studies for children/adolescents (2 national)
2 studies for adults (2 national)
Prevalence (%)
BMI < -2SD
-2SD ≤ BMI < -1SD
-1SD ≤ BMI ≤ 1SD
1SD < BMI ≤ 2SD
BMI > 2SD
BMI <18.5
BMI 18.5-20
BMI 20-25
BMI 25-30
BMI 30-35
BMI 35-40
BMI≥ 40

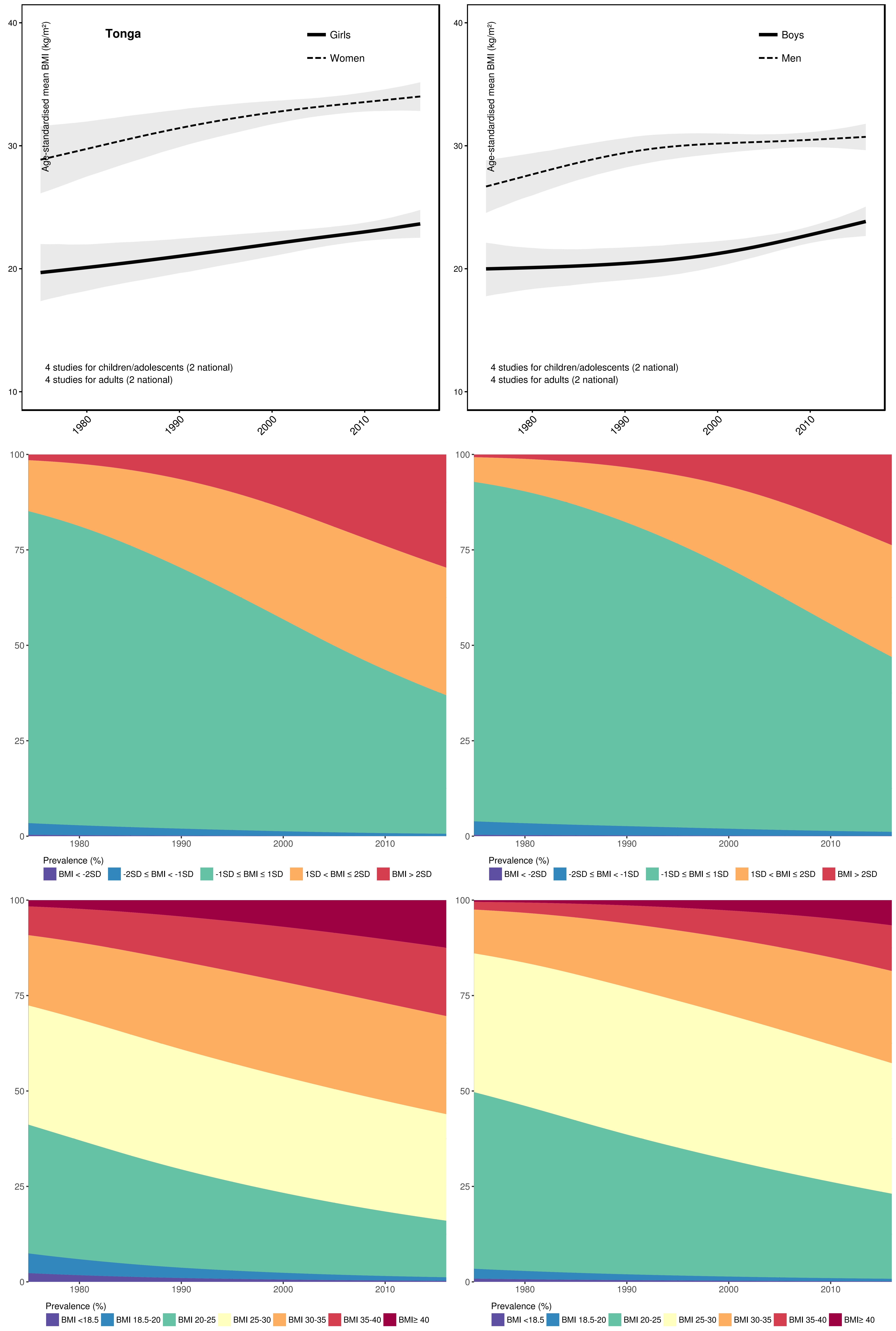
Tonga
Girls
Women
Age-standardised mean BMI (kg/m²)
4 studies for children/adolescents (2 national)
4 studies for adults (2 national)
Boys
Men
Age-standardised mean BMI (kg/m²)
4 studies for children/adolescents (2 national)
4 studies for adults (2 national)
Prevalence (%)
BMI < -2SD
-2SD ≤ BMI < -1SD
-1SD ≤ BMI ≤ 1SD
1SD < BMI ≤ 2SD
BMI > 2SD
Prevalence (%)
BMI <18.5
BMI 18.5-20
BMI 20-25
BMI 25-30
BMI 30-35
BMI 35-40
BMI≥ 40

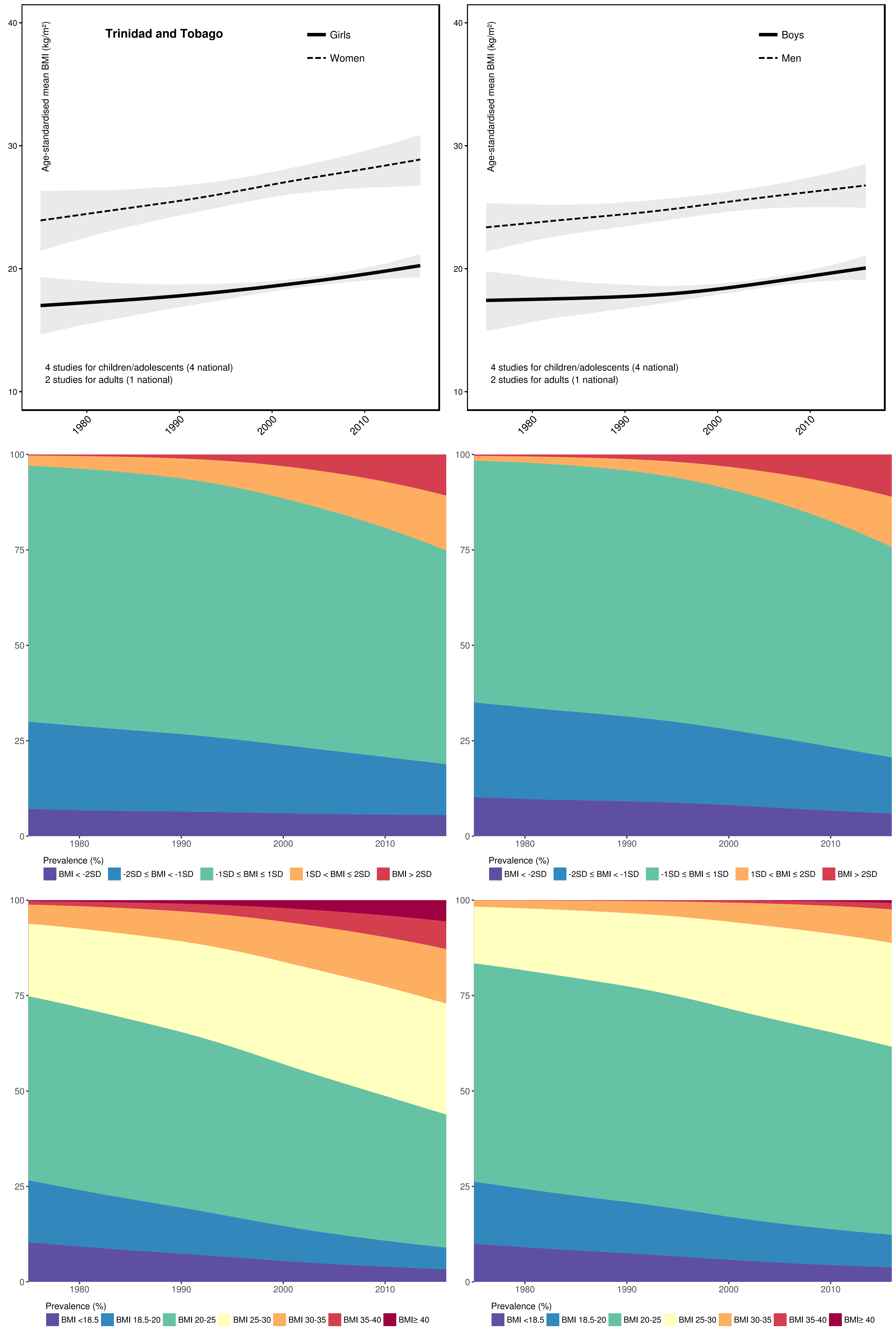
Trinidad and Tobago
Girls
Women
Boys
Men
Age-standardised mean BMI (kg/m²)
4 studies for children/adolescents (4 national)
2 studies for adults (1 national)
Prevalence (%)
BMI < -2SD
-2SD ≤ BMI < -1SD
-1SD ≤ BMI ≤ 1SD
1SD < BMI ≤ 2SD
BMI > 2SD
BMI <18.5
BMI 18.5-20
BMI 20-25
BMI 25-30
BMI 30-35
BMI 35-40
BMI≥ 40

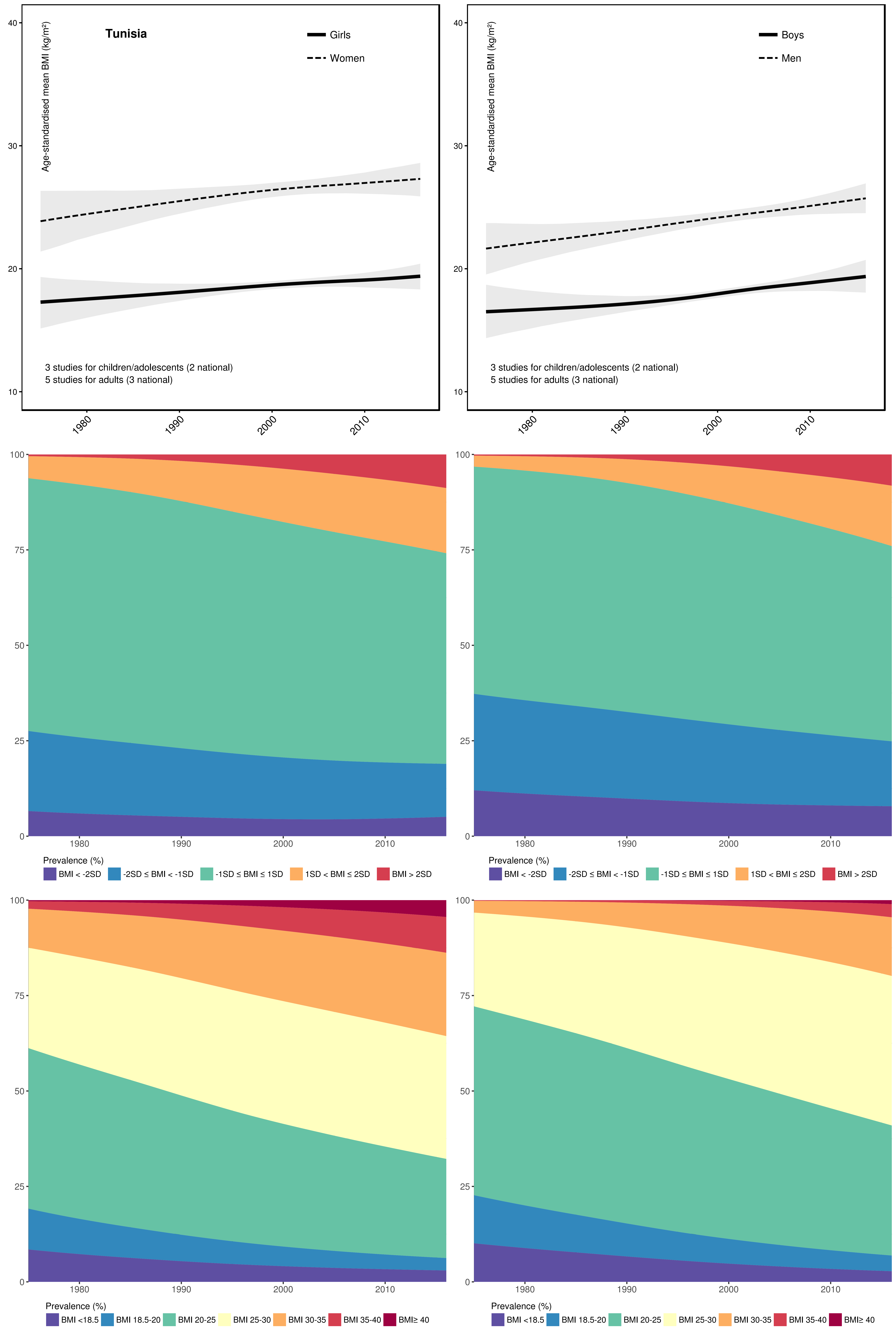
Tunisia
Girls
Women
Boys
Men
Age-standardised mean BMI (kg/m²)
3 studies for children/adolescents (2 national)
5 studies for adults (3 national)
1980
1990
2000
2010
Prevalence (%)
BMI < -2SD
-2SD ≤ BMI < -1SD
-1SD ≤ BMI ≤ 1SD
1SD < BMI ≤ 2SD
BMI > 2SD
BMI <18.5
BMI 18.5-20
BMI 20-25
BMI 25-30
BMI 30-35
BMI 35-40
BMI≥ 40

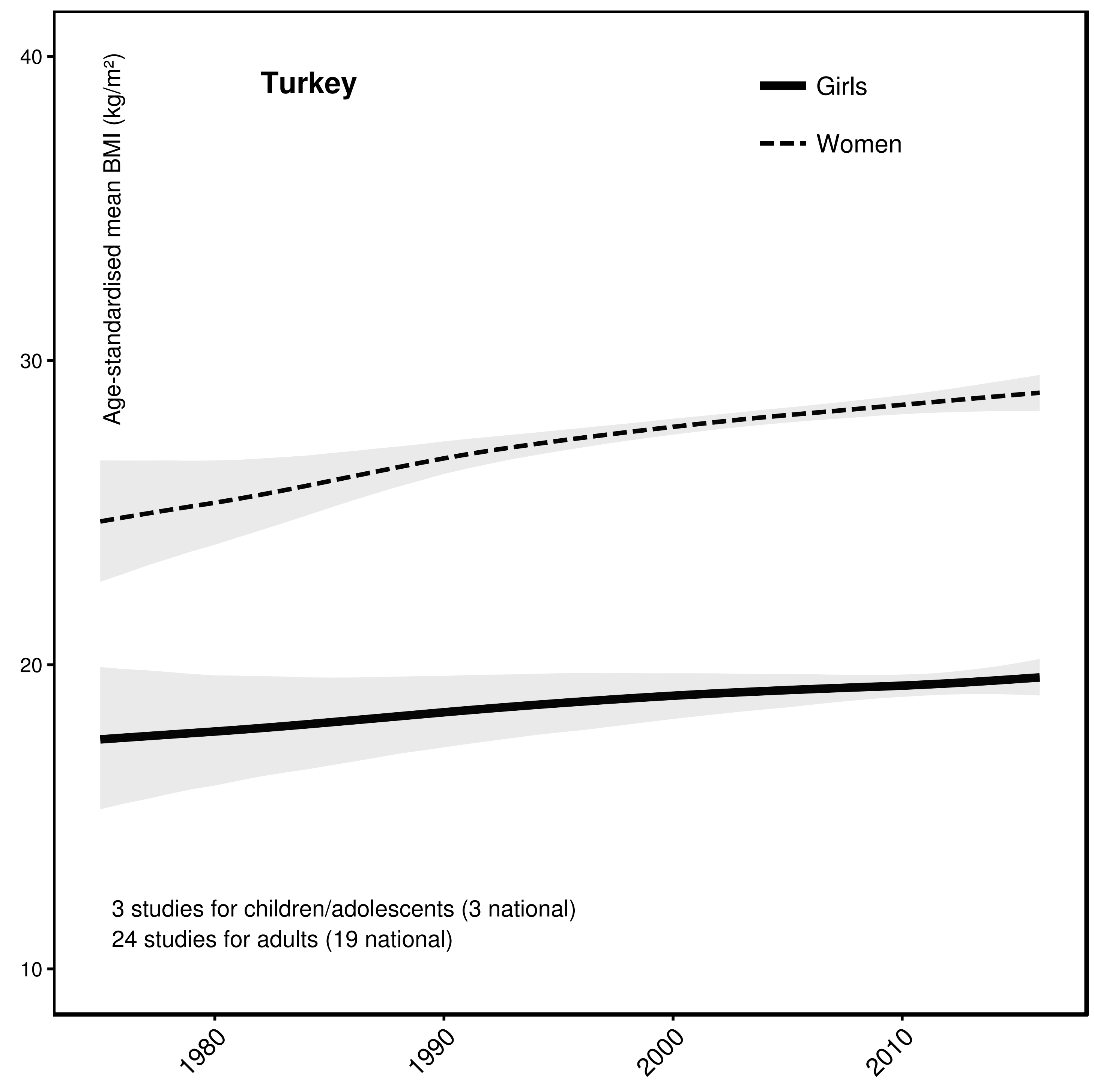
Turkey
Girls
Women
Age-standardised mean BMI (kg/m²)
40
30
20
10
3 studies for children/adolescents (3 national)
24 studies for adults (19 national)
1980
1990
2000
2010

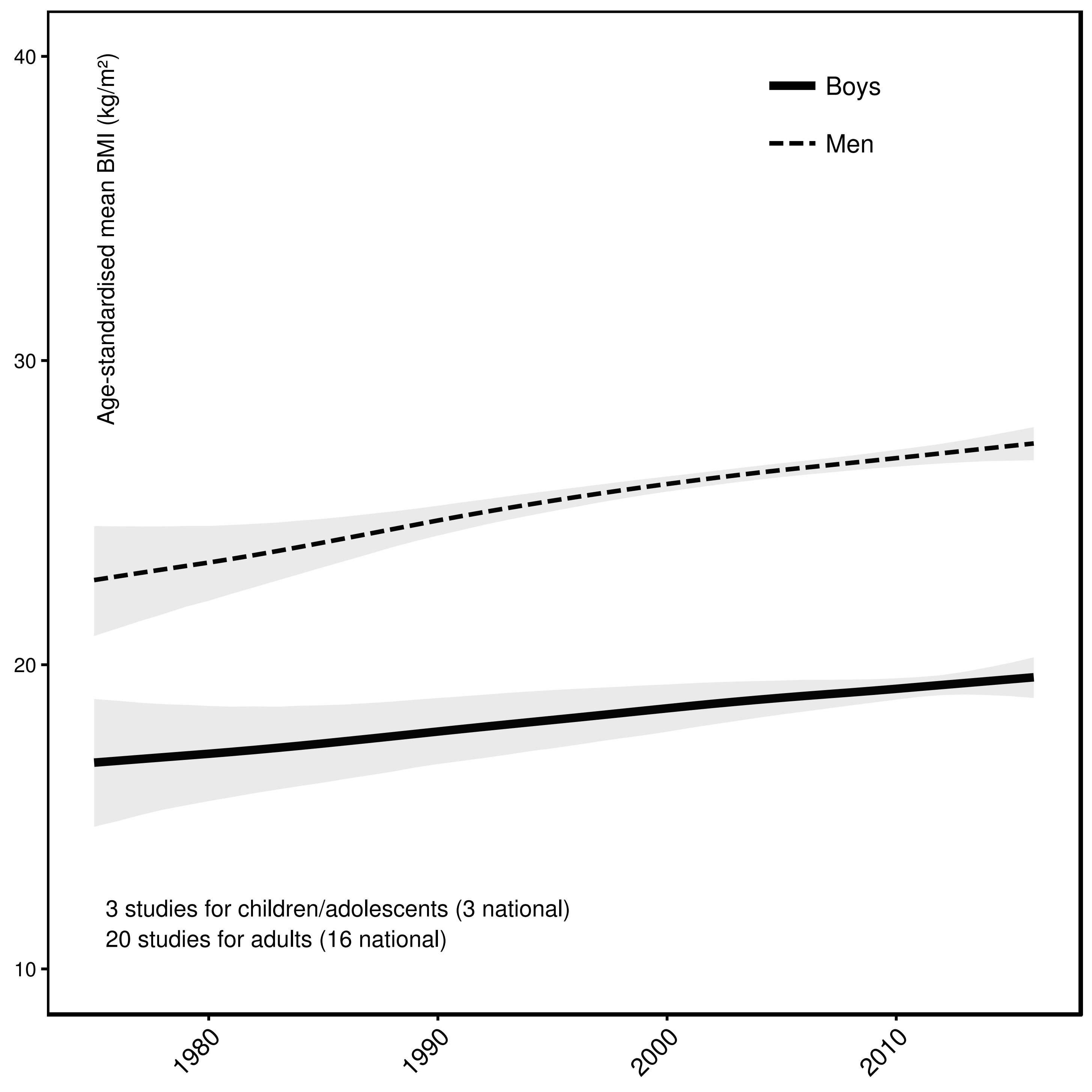
Boys
Men
Age-standardised mean BMI (kg/m²)
40
30
20
10
3 studies for children/adolescents (3 national)
20 studies for adults (16 national)
1980
1990
2000
2010

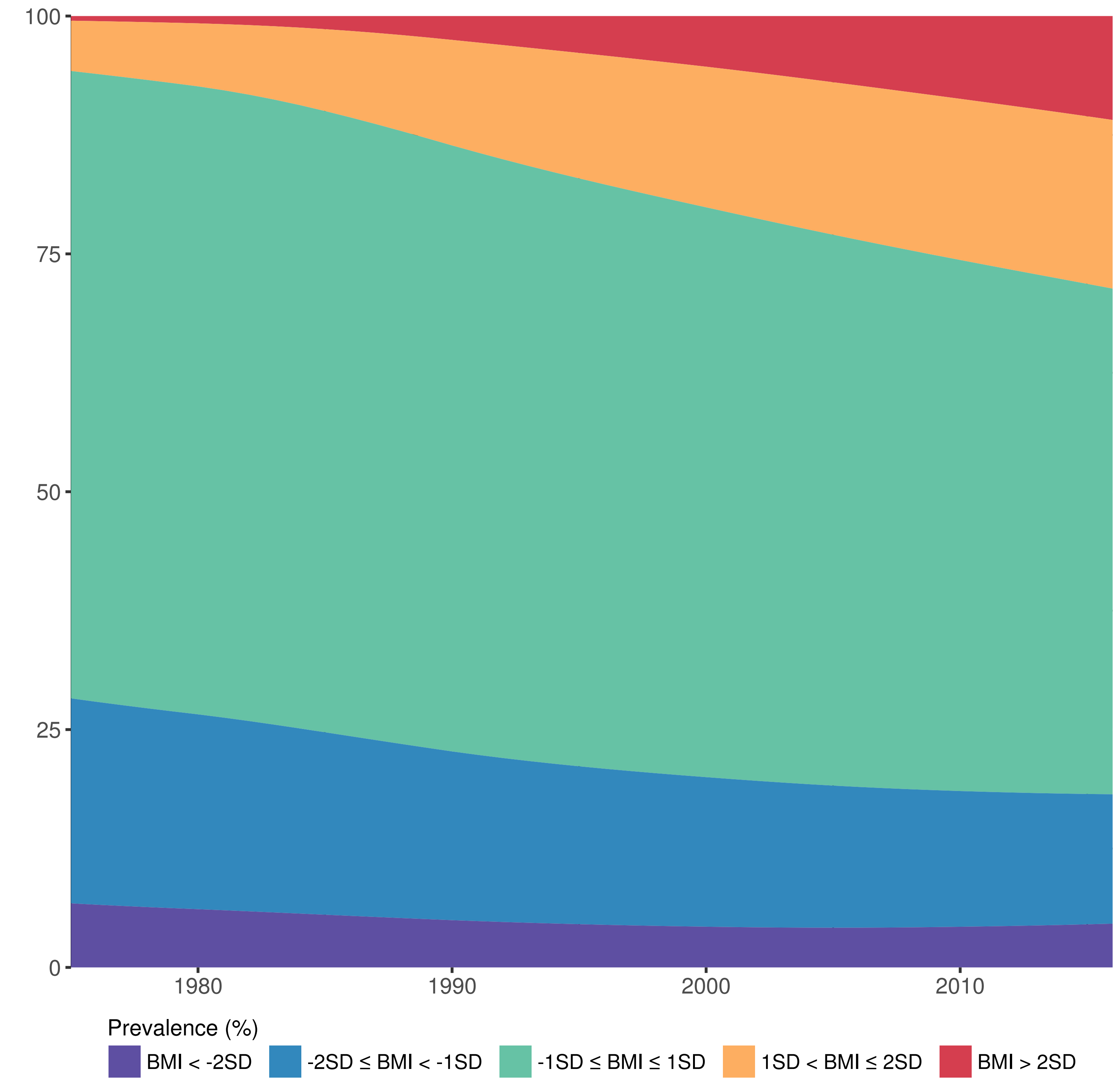
100
75
50
25
0
1980
1990
2000
2010
Prevalence (%)
BMI < -2SD
-2SD ≤ BMI < -1SD
-1SD ≤ BMI ≤ 1SD
1SD < BMI ≤ 2SD
BMI > 2SD

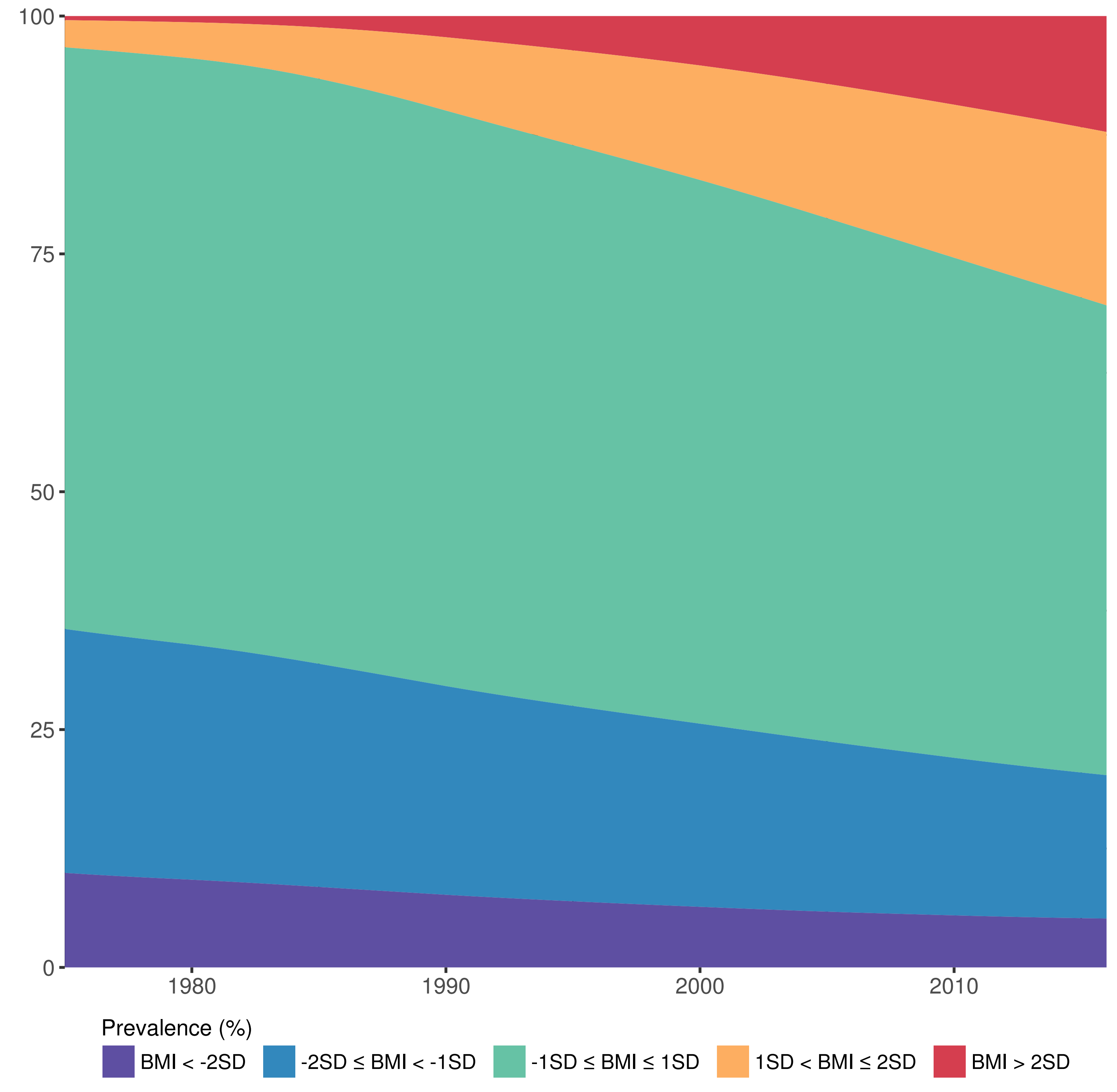
100
75
50
25
0
1980
1990
2000
2010
Prevalence (%)
BMI < -2SD
-2SD ≤ BMI < -1SD
-1SD ≤ BMI ≤ 1SD
1SD < BMI ≤ 2SD
BMI > 2SD

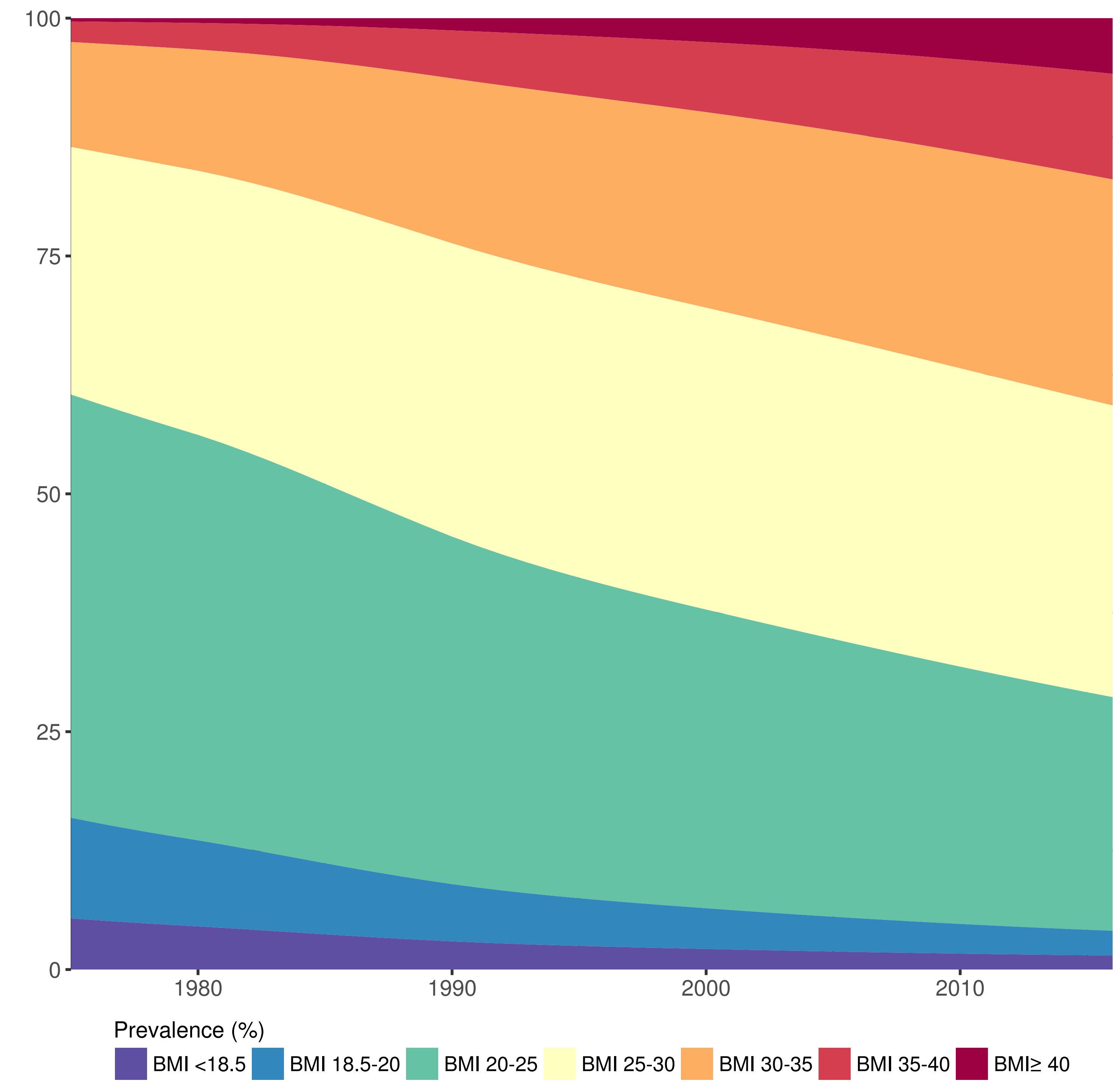
100
75
50
25
0
1980
1990
2000
2010
Prevalence (%)
BMI <18.5
BMI 18.5-20
BMI 20-25
BMI 25-30
BMI 30-35
BMI 35-40
BMI≥ 40

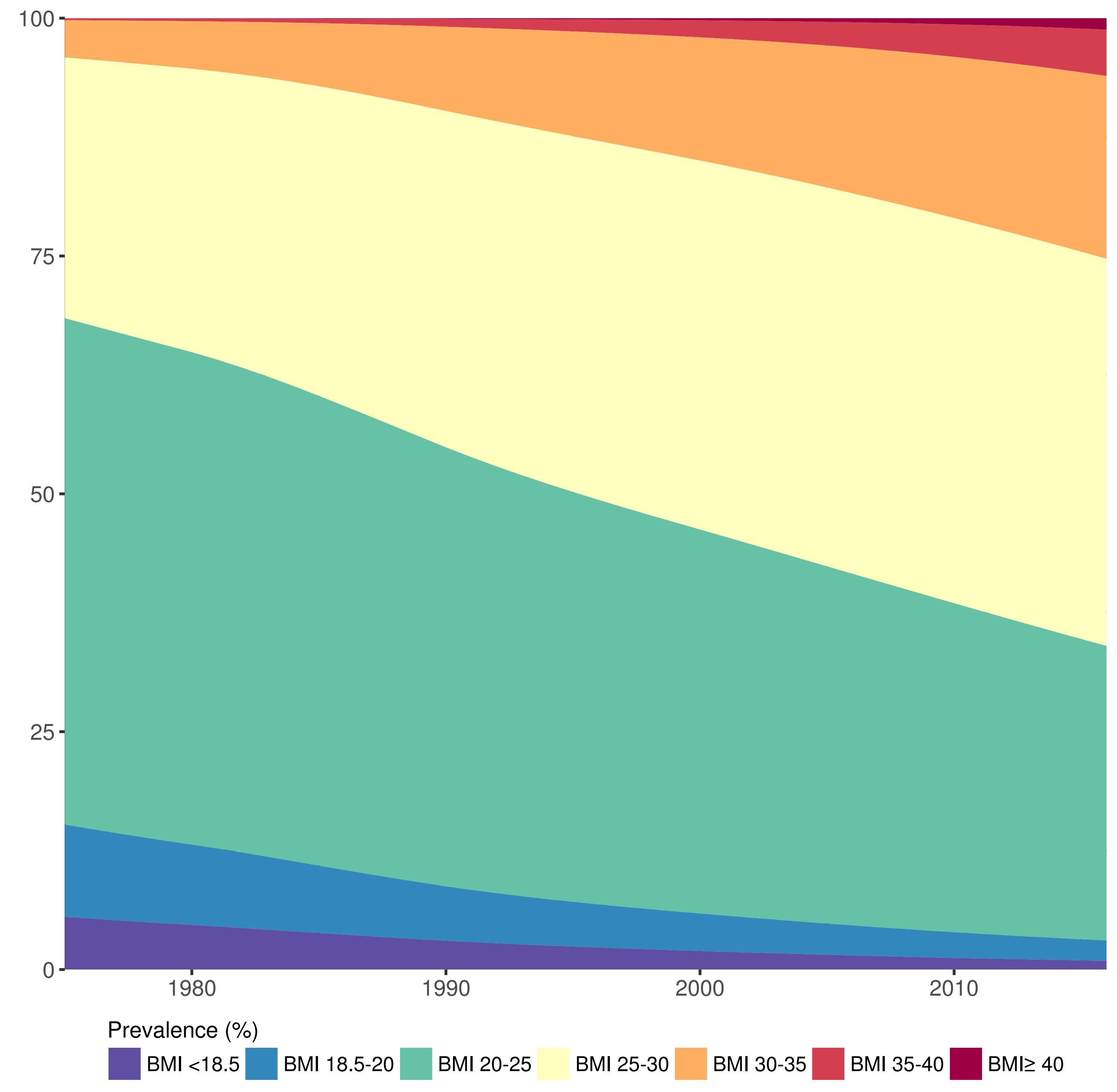
100
75
50
25
0
1980
1990
2000
2010
Prevalence (%)
BMI <18.5
BMI 18.5-20
BMI 20-25
BMI 25-30
BMI 30-35
BMI 35-40
BMI≥ 40

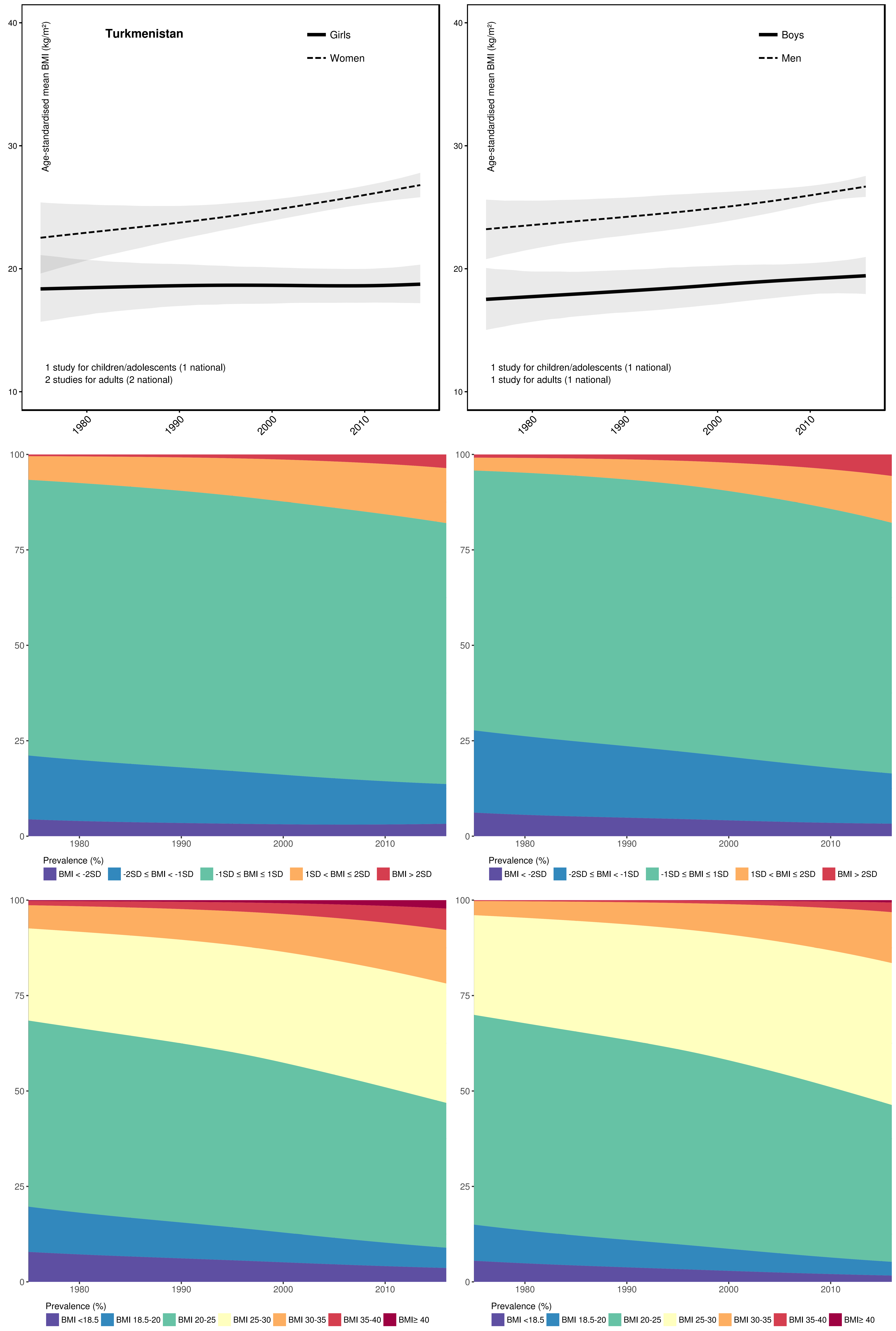
Turkmenistan
Girls
Women
Boys
Men
Age-standardised mean BMI (kg/m²)
1 study for children/adolescents (1 national)
2 studies for adults (2 national)
1 study for children/adolescents (1 national)
1 study for adults (1 national)
Prevalence (%)
BMI < -2SD
-2SD ≤ BMI < -1SD
-1SD ≤ BMI ≤ 1SD
1SD < BMI ≤ 2SD
BMI > 2SD
BMI <18.5
BMI 18.5-20
BMI 20-25
BMI 25-30
BMI 30-35
BMI 35-40
BMI≥ 40

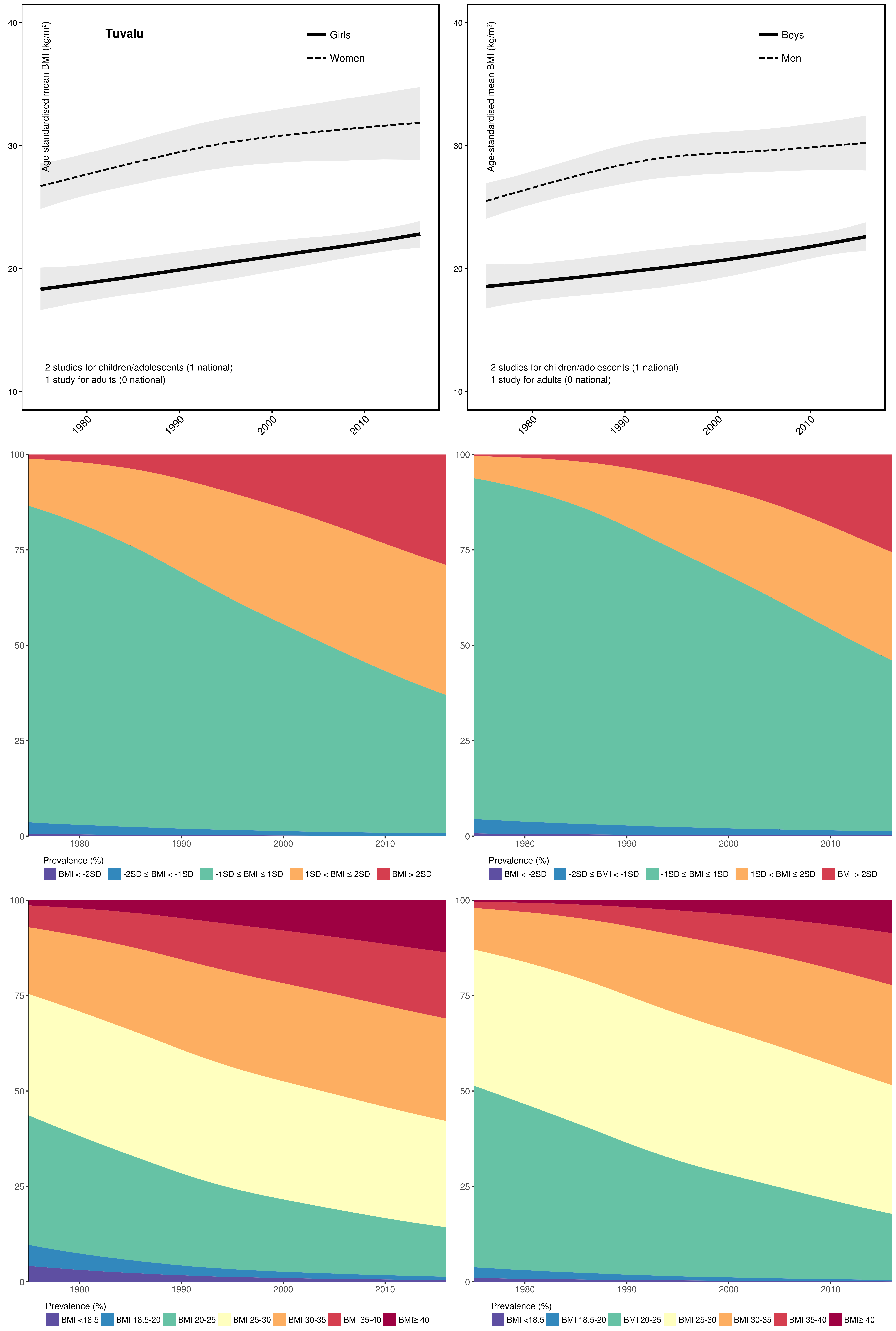
Tuvalu
Girls
Women
Boys
Men
Age-standardised mean BMI (kg/m²)
2 studies for children/adolescents (1 national)
1 study for adults (0 national)
Prevalence (%)
BMI < -2SD
-2SD ≤ BMI < -1SD
-1SD ≤ BMI ≤ 1SD
1SD < BMI ≤ 2SD
BMI > 2SD
BMI <18.5
BMI 18.5-20
BMI 20-25
BMI 25-30
BMI 30-35
BMI 35-40
BMI≥ 40

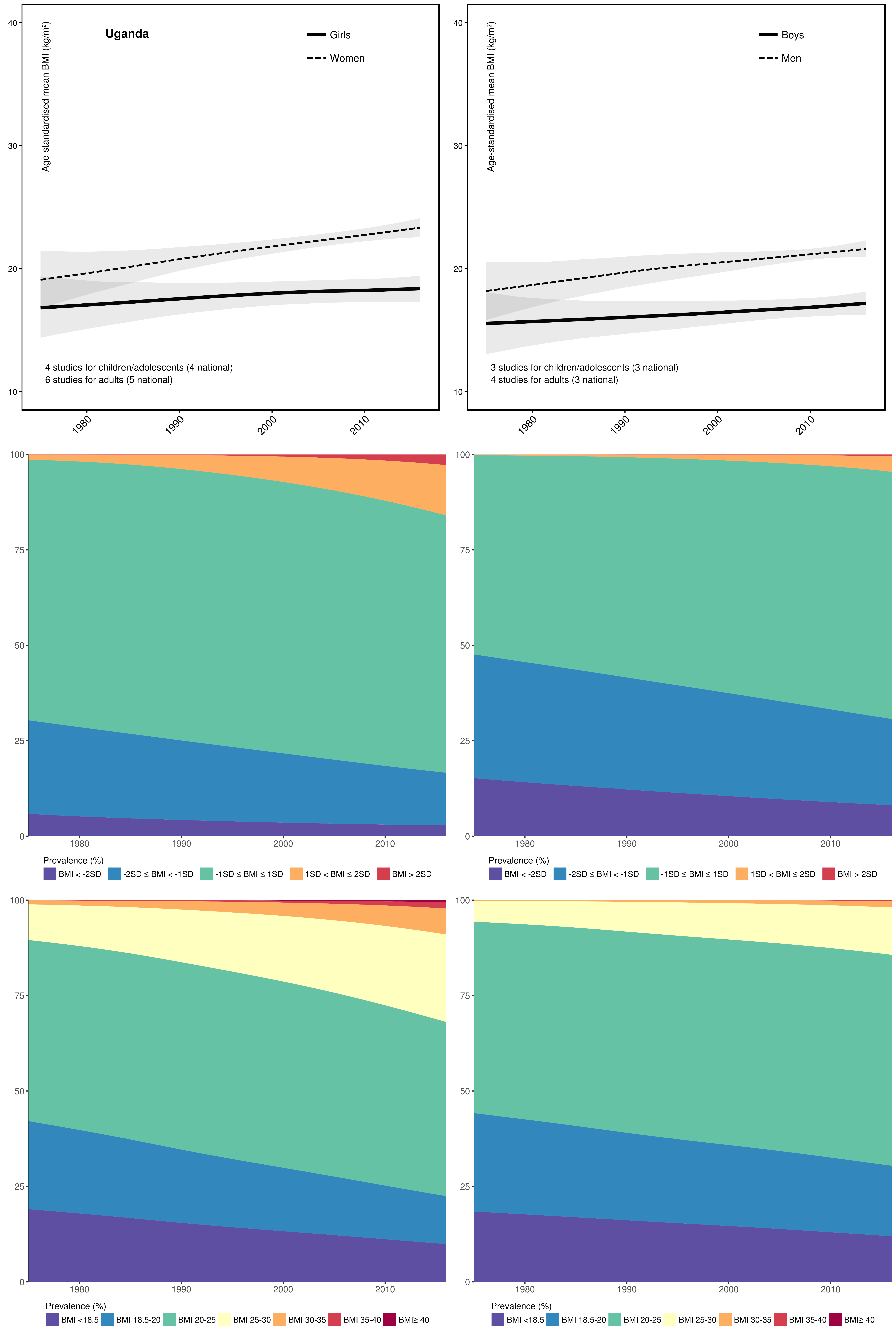
Uganda
Girls
Women
Boys
Men
Age-standardised mean BMI (kg/m²)
4 studies for children/adolescents (4 national)
6 studies for adults (5 national)
3 studies for children/adolescents (3 national)
4 studies for adults (3 national)
Prevalence (%)
BMI < -2SD
-2SD ≤ BMI < -1SD
-1SD ≤ BMI ≤ 1SD
1SD < BMI ≤ 2SD
BMI > 2SD
BMI <18.5
BMI 18.5-20
BMI 20-25
BMI 25-30
BMI 30-35
BMI 35-40
BMI≥ 40

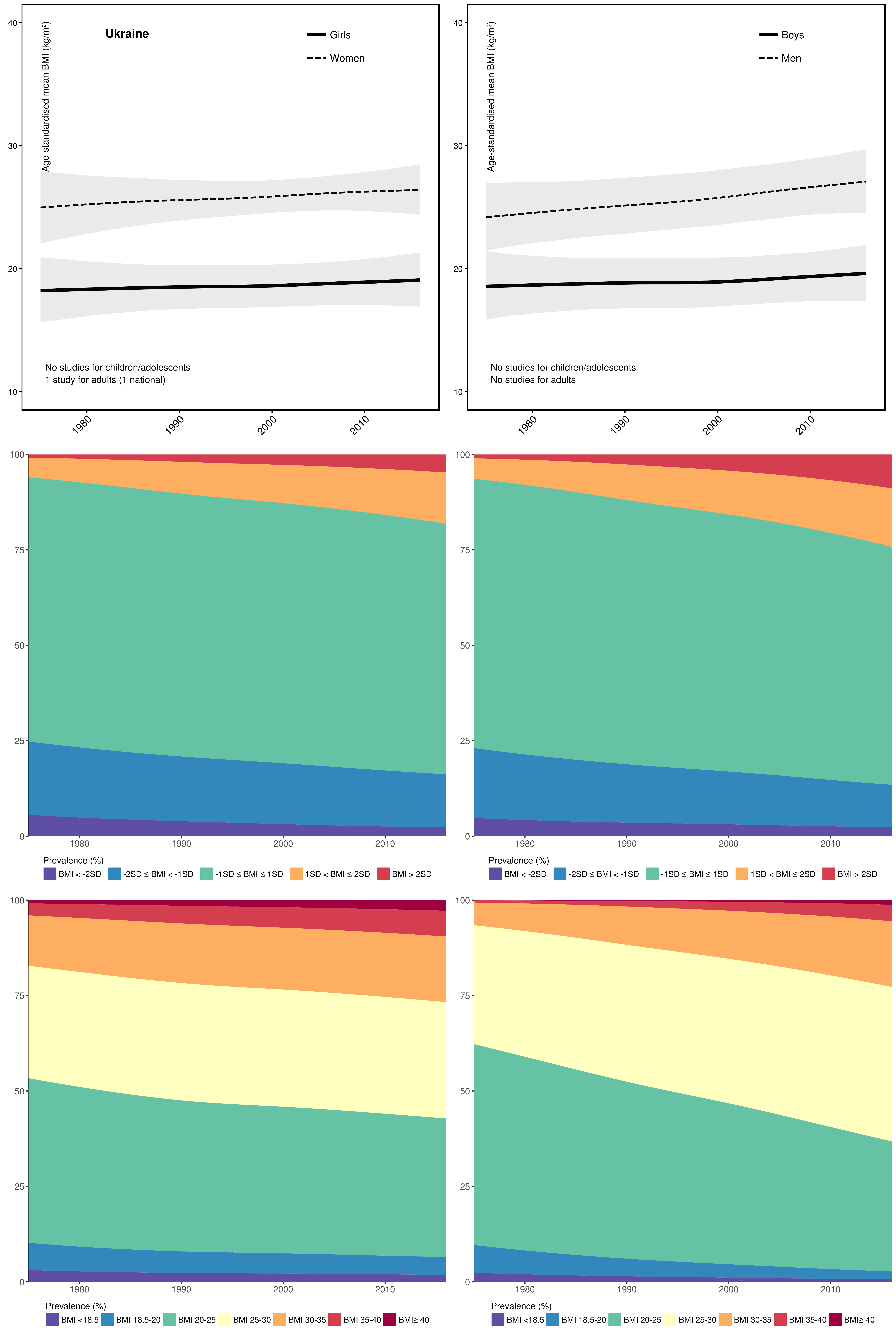
Ukraine
Girls
Women
Boys
Men
Age-standardised mean BMI (kg/m²)
No studies for children/adolescents
1 study for adults (1 national)
No studies for children/adolescents
No studies for adults
Prevalence (%)
BMI < -2SD
-2SD ≤ BMI < -1SD
-1SD ≤ BMI ≤ 1SD
1SD < BMI ≤ 2SD
BMI > 2SD
BMI <18.5
BMI 18.5-20
BMI 20-25
BMI 25-30
BMI 30-35
BMI 35-40
BMI≥ 40

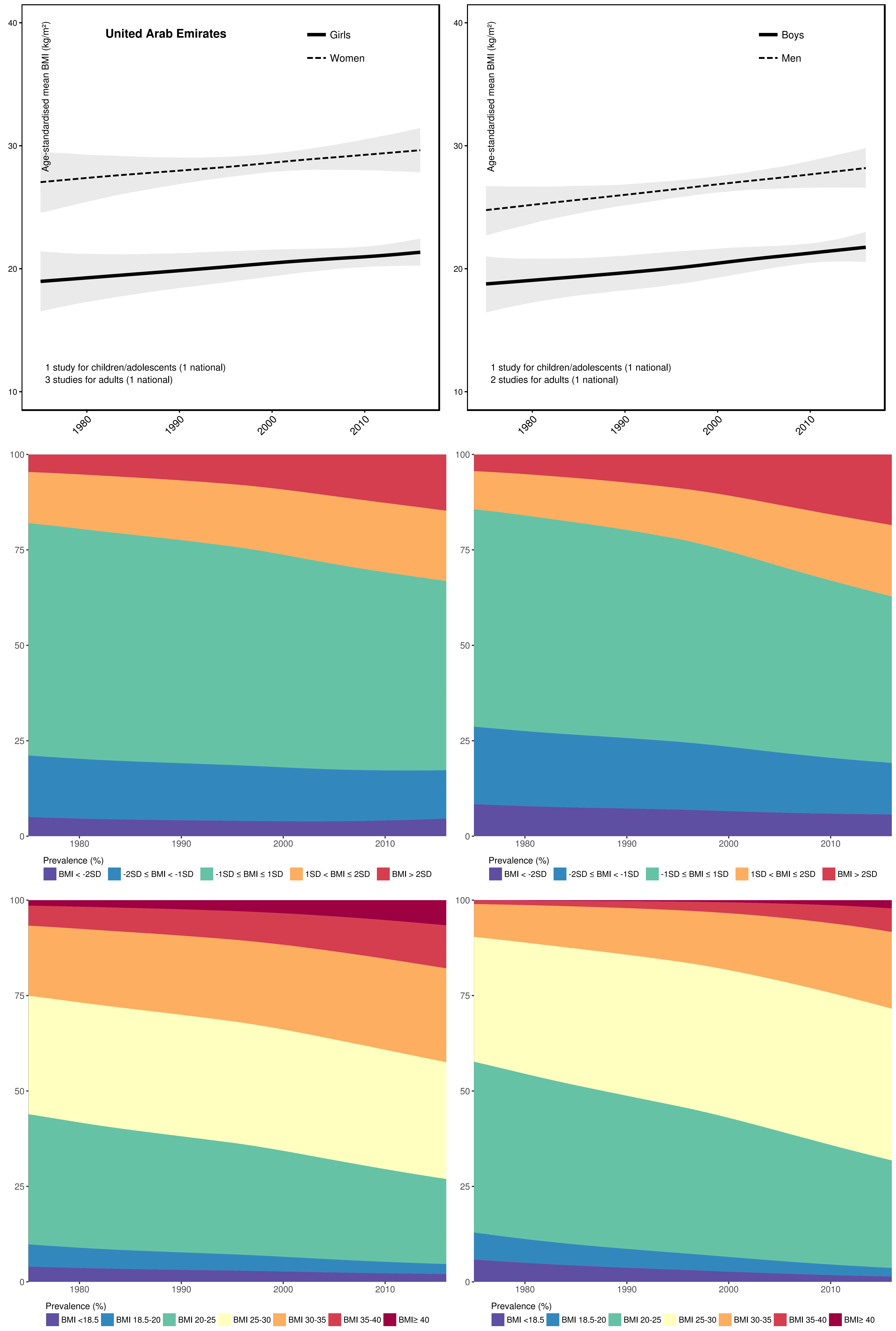
United Arab Emirates
Girls
Women
Age-standardised mean BMI (kg/m²)
1 study for children/adolescents (1 national)
3 studies for adults (1 national)
Boys
Men
Age-standardised mean BMI (kg/m²)
1 study for children/adolescents (1 national)
2 studies for adults (1 national)
1980
1990
2000
2010
Prevalence (%)
BMI < -2SD
-2SD ≤ BMI < -1SD
-1SD ≤ BMI ≤ 1SD
1SD < BMI ≤ 2SD
BMI > 2SD
Prevalence (%)
BMI <18.5
BMI 18.5-20
BMI 20-25
BMI 25-30
BMI 30-35
BMI 35-40
BMI≥ 40

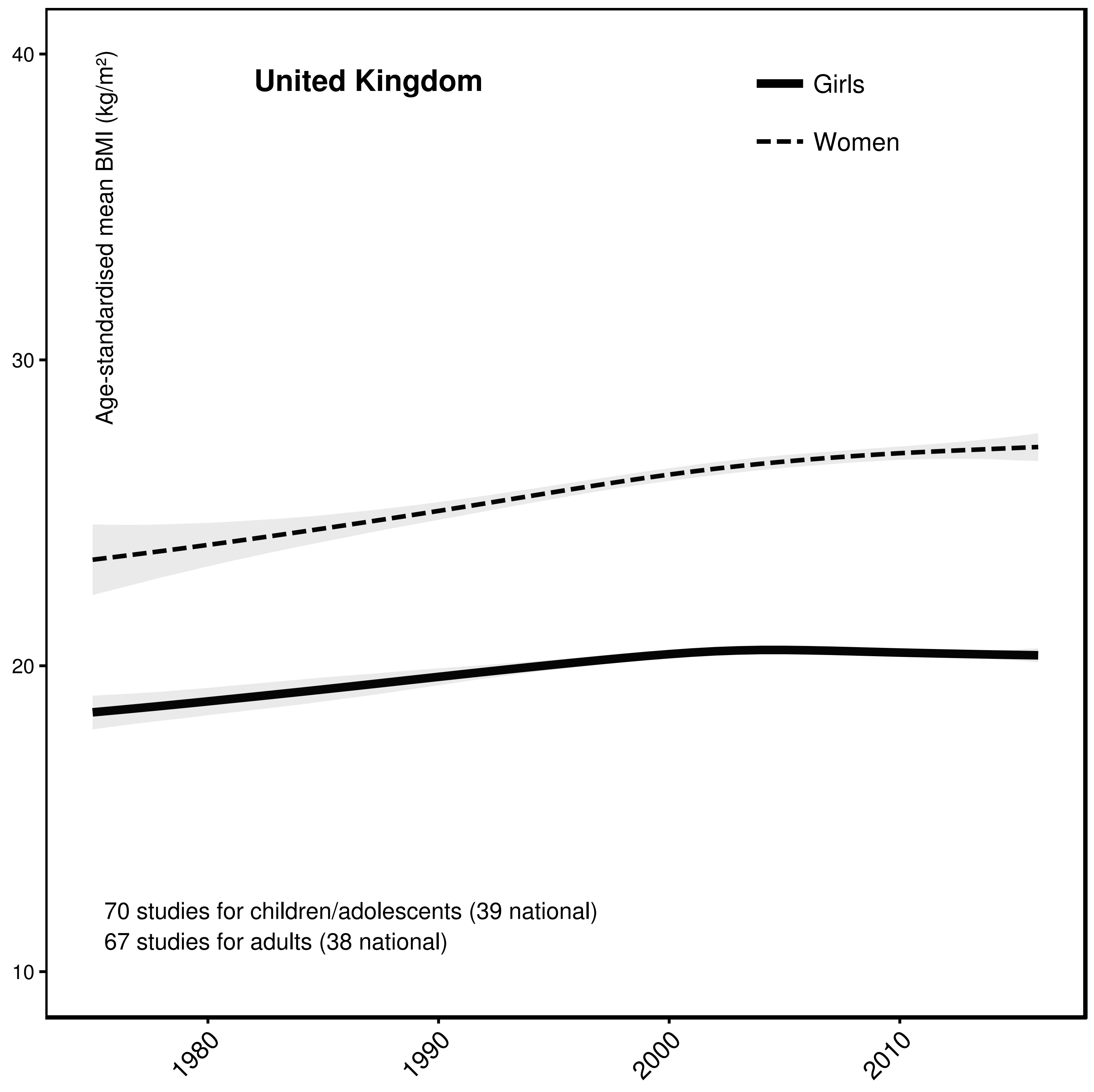
United Kingdom
Girls
Women
Age-standardised mean BMI (kg/m²)
40
30
20
10
1980
1990
2000
2010
70 studies for children/adolescents (39 national)
67 studies for adults (38 national)

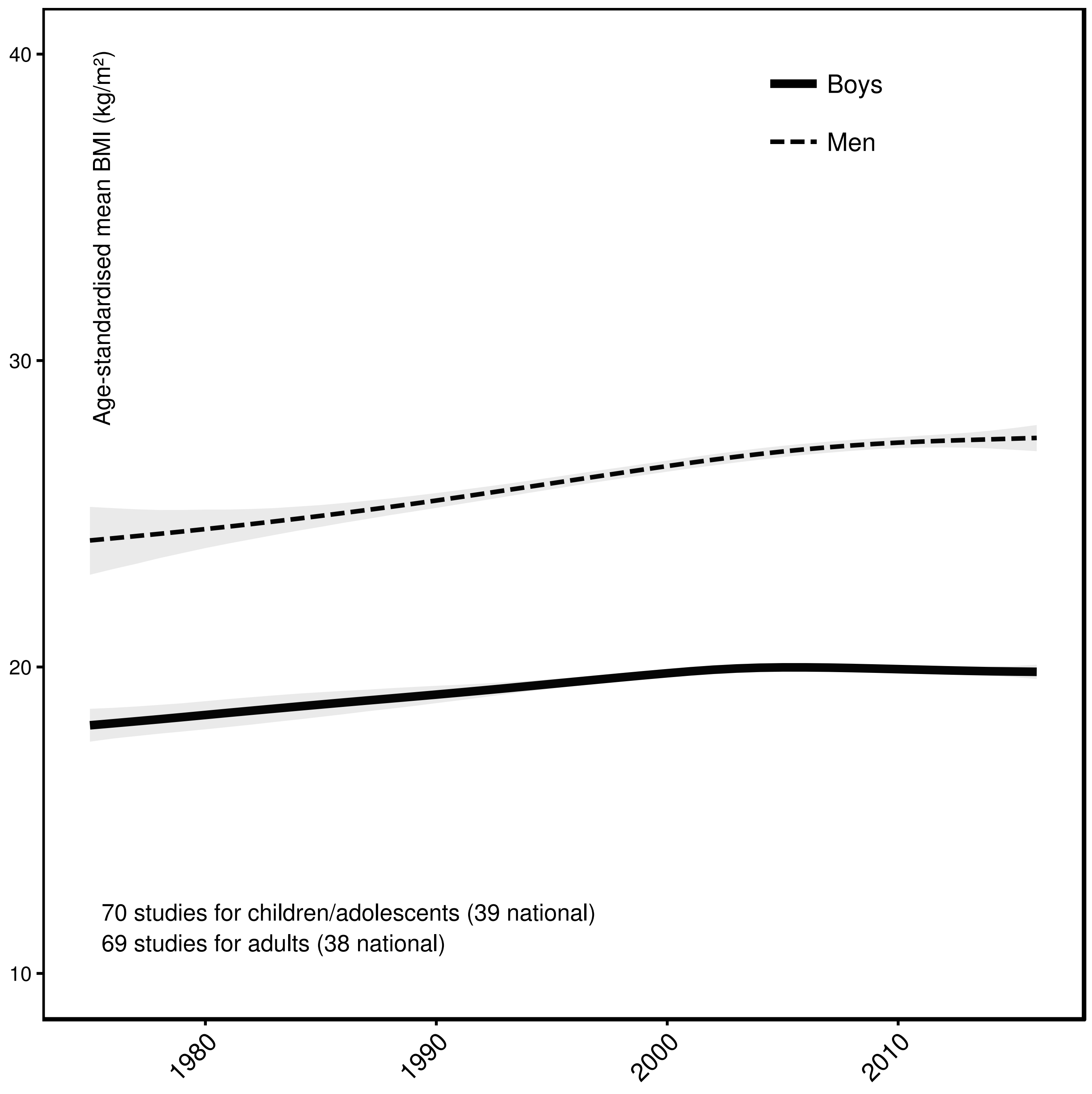
Boys
Men
Age-standardised mean BMI (kg/m²)
40
30
20
10
1980
1990
2000
2010
70 studies for children/adolescents (39 national)
69 studies for adults (38 national)

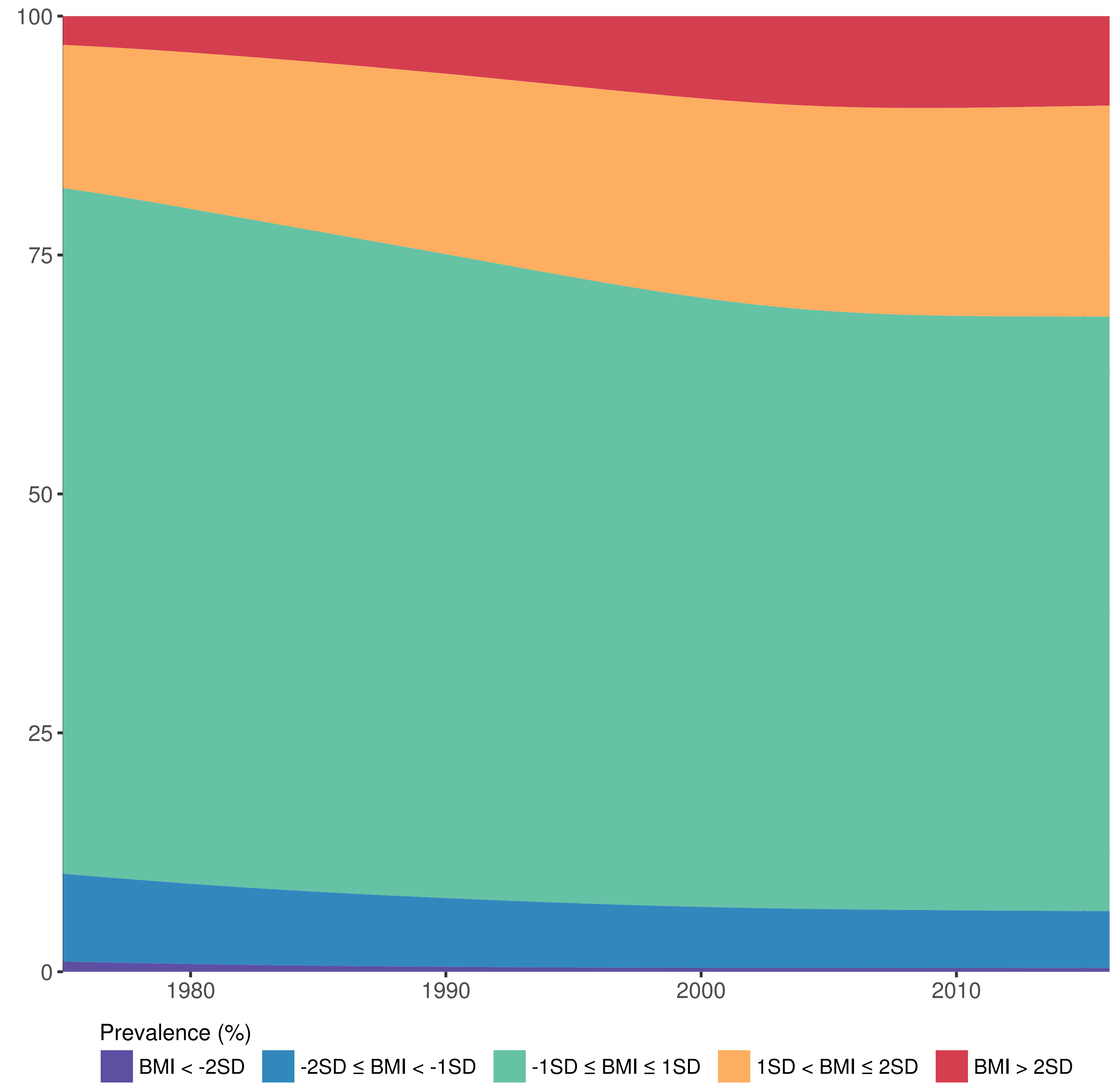
100
75
50
25
0
1980
1990
2000
2010
Prevalence (%)
BMI < -2SD
-2SD ≤ BMI < -1SD
-1SD ≤ BMI ≤ 1SD
1SD < BMI ≤ 2SD
BMI > 2SD

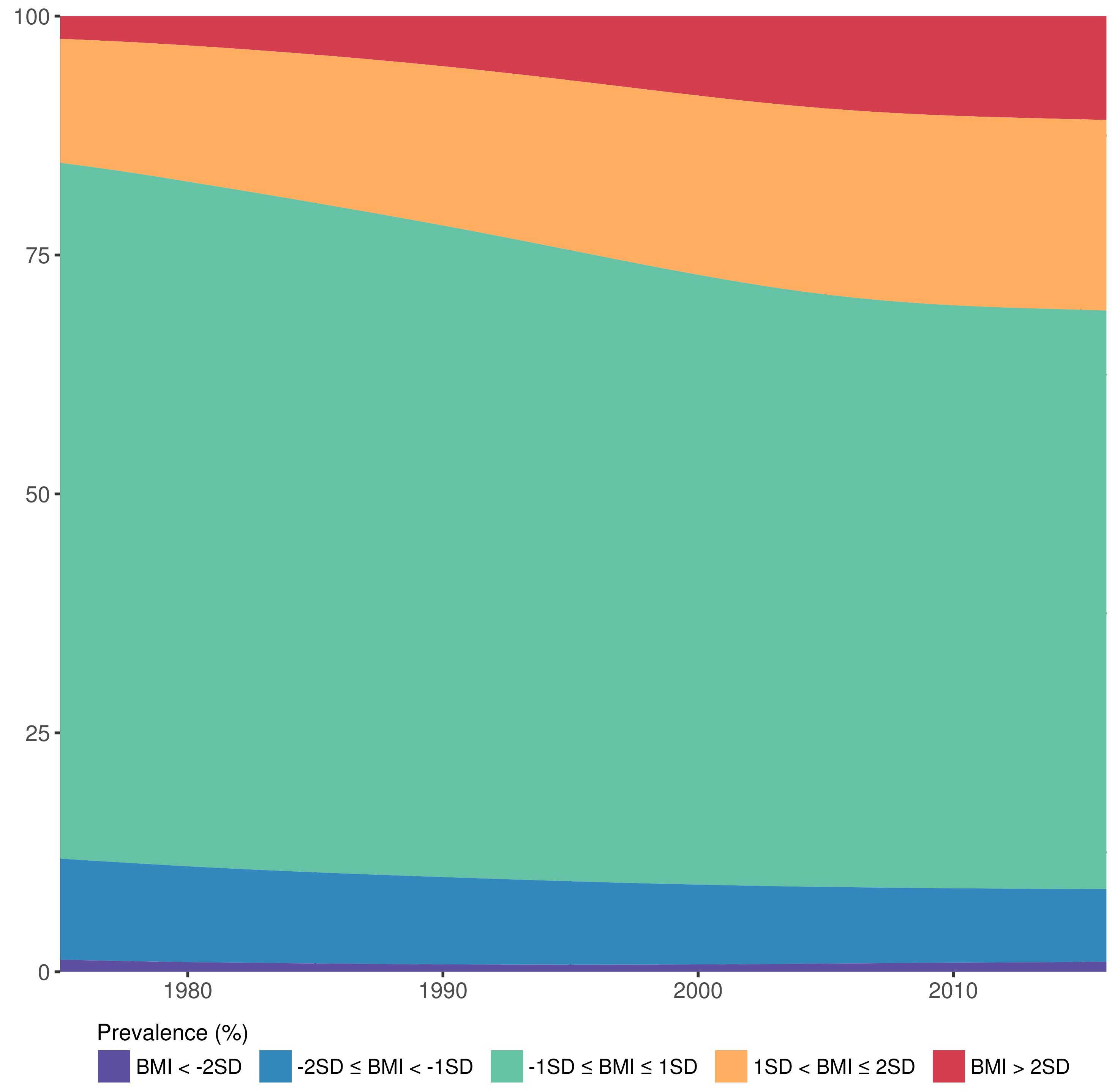
100
75
50
25
0
1980
1990
2000
2010
Prevalence (%)
BMI < -2SD
-2SD ≤ BMI < -1SD
-1SD ≤ BMI ≤ 1SD
1SD < BMI ≤ 2SD
BMI > 2SD

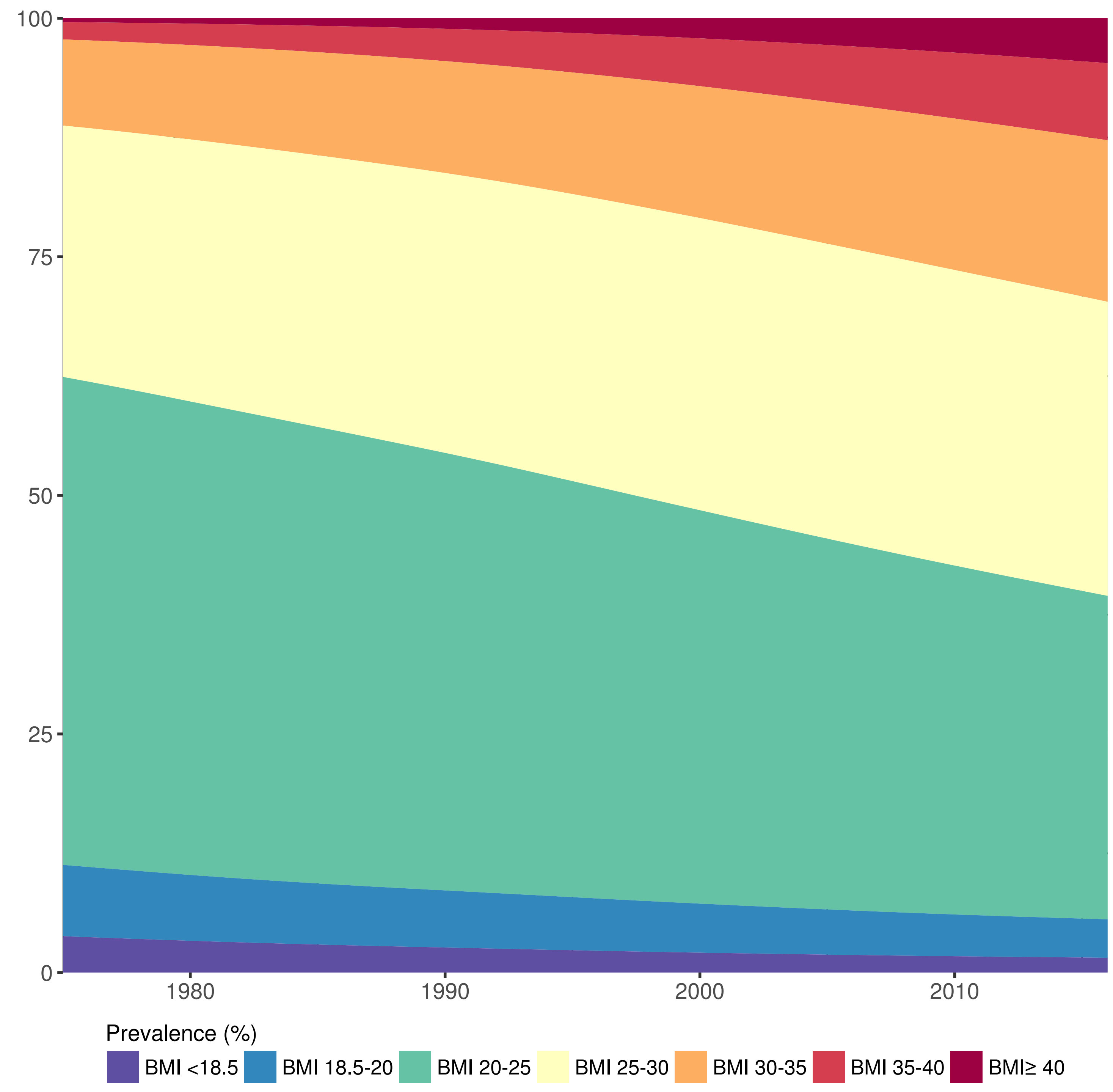
100
75
50
25
0
1980
1990
2000
2010
Prevalence (%)
BMI <18.5
BMI 18.5-20
BMI 20-25
BMI 25-30
BMI 30-35
BMI 35-40
BMI≥ 40

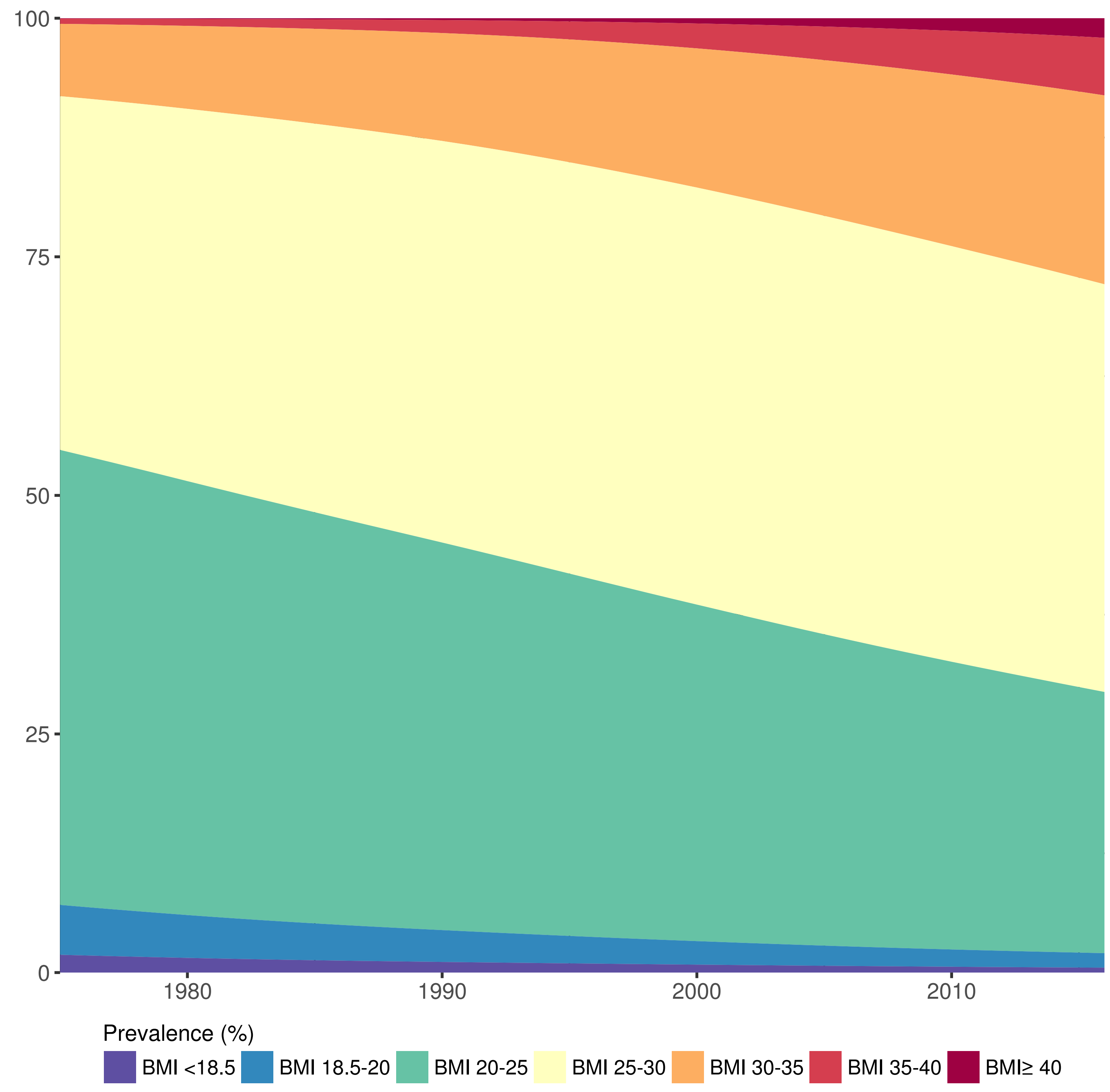
100
75
50
25
0
1980
1990
2000
2010
Prevalence (%)
BMI <18.5
BMI 18.5-20
BMI 20-25
BMI 25-30
BMI 30-35
BMI 35-40
BMI≥ 40

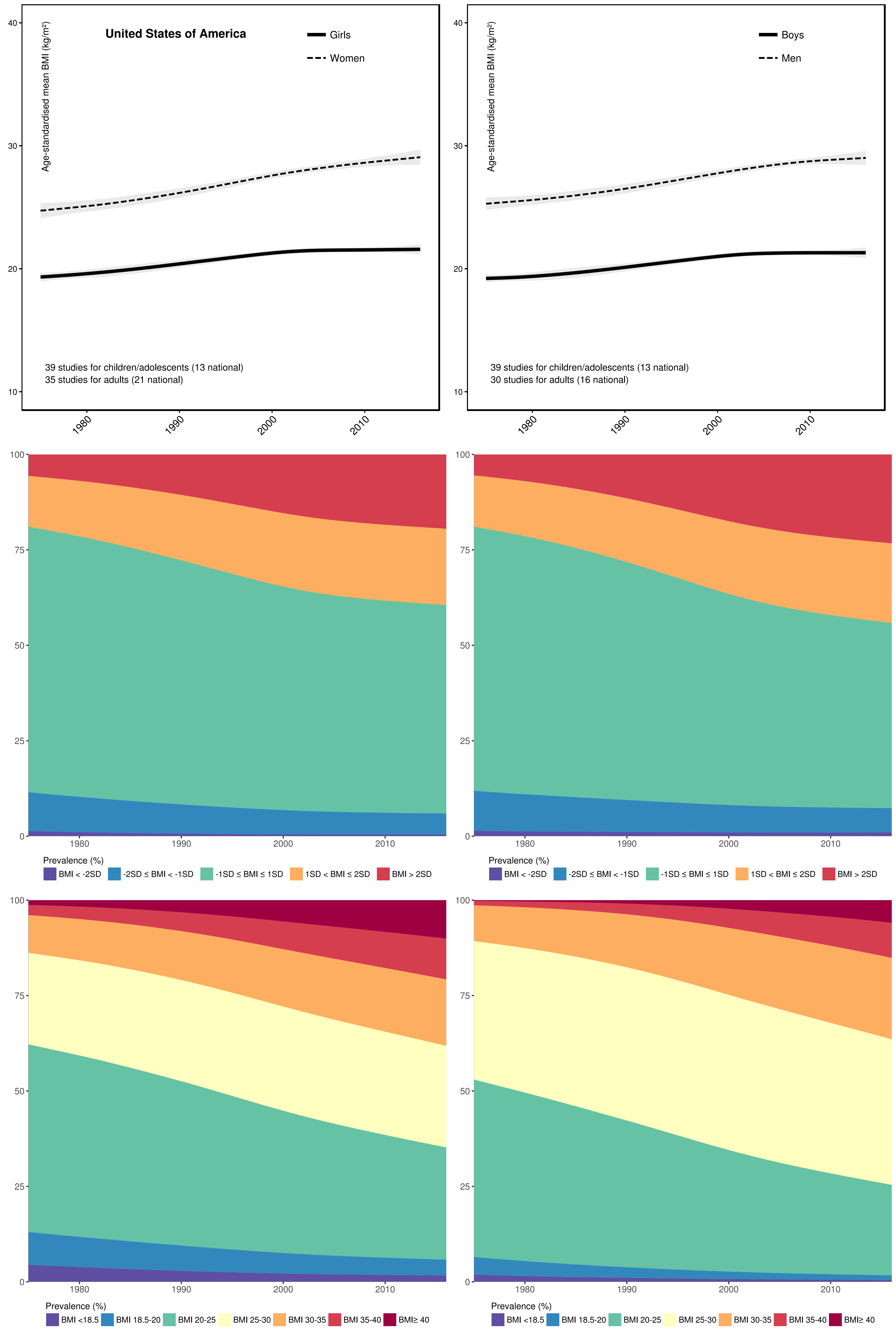
United States of America
Girls
Women
Boys
Men
Age-standardised mean BMI (kg/m²)
39 studies for children/adolescents (13 national)
35 studies for adults (21 national)
39 studies for children/adolescents (13 national)
30 studies for adults (16 national)
1980
1990
2000
2010
Prevalence (%)
BMI < -2SD
-2SD ≤ BMI < -1SD
-1SD ≤ BMI ≤ 1SD
1SD < BMI ≤ 2SD
BMI > 2SD
BMI <18.5
BMI 18.5-20
BMI 20-25
BMI 25-30
BMI 30-35
BMI 35-40
BMI≥ 40

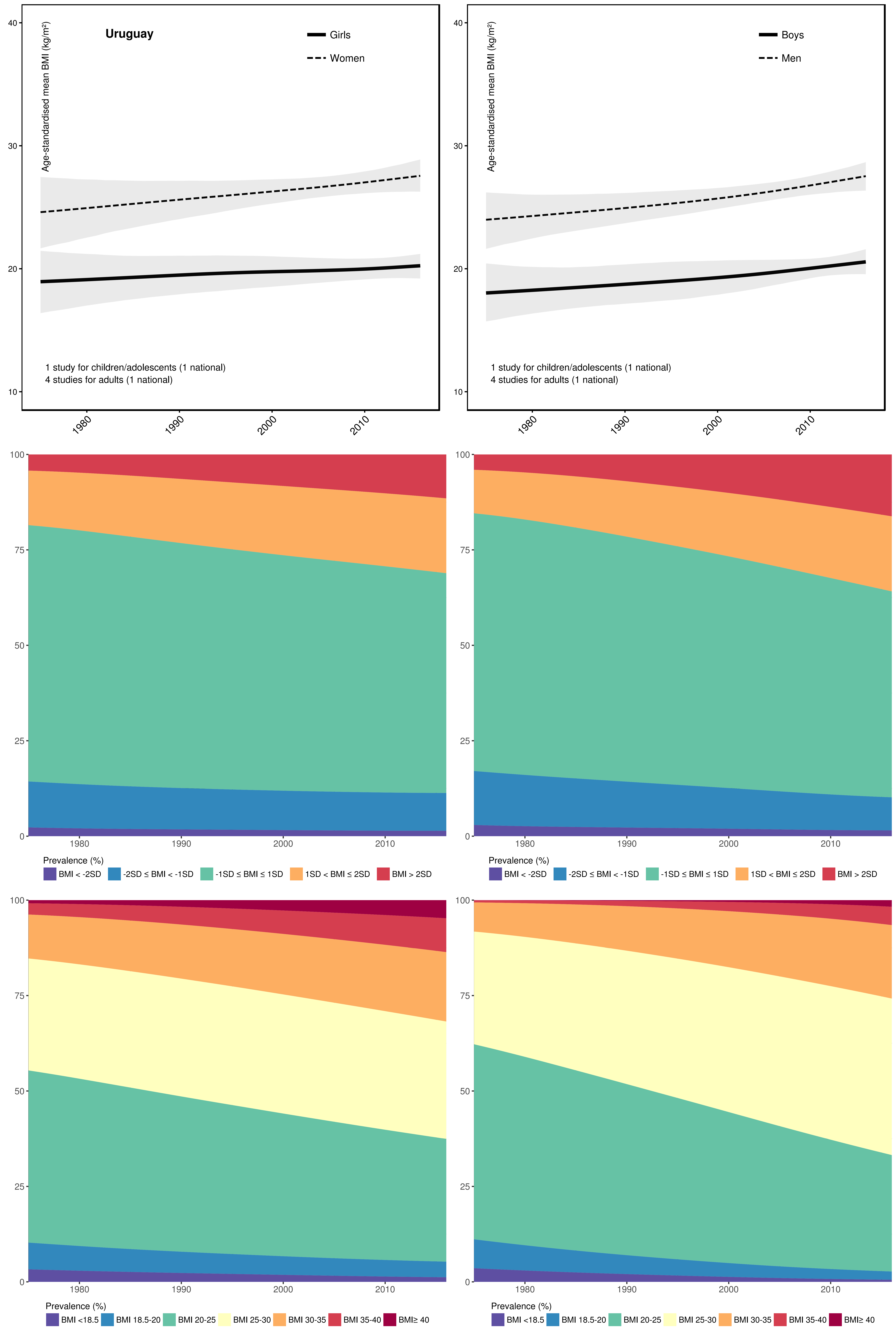
Uruguay
Girls
Women
Boys
Men
Age-standardised mean BMI (kg/m²)
1 study for children/adolescents (1 national)
4 studies for adults (1 national)
Prevalence (%)
BMI < -2SD
-2SD ≤ BMI < -1SD
-1SD ≤ BMI ≤ 1SD
1SD < BMI ≤ 2SD
BMI > 2SD
BMI <18.5
BMI 18.5-20
BMI 20-25
BMI 25-30
BMI 30-35
BMI 35-40
BMI≥ 40

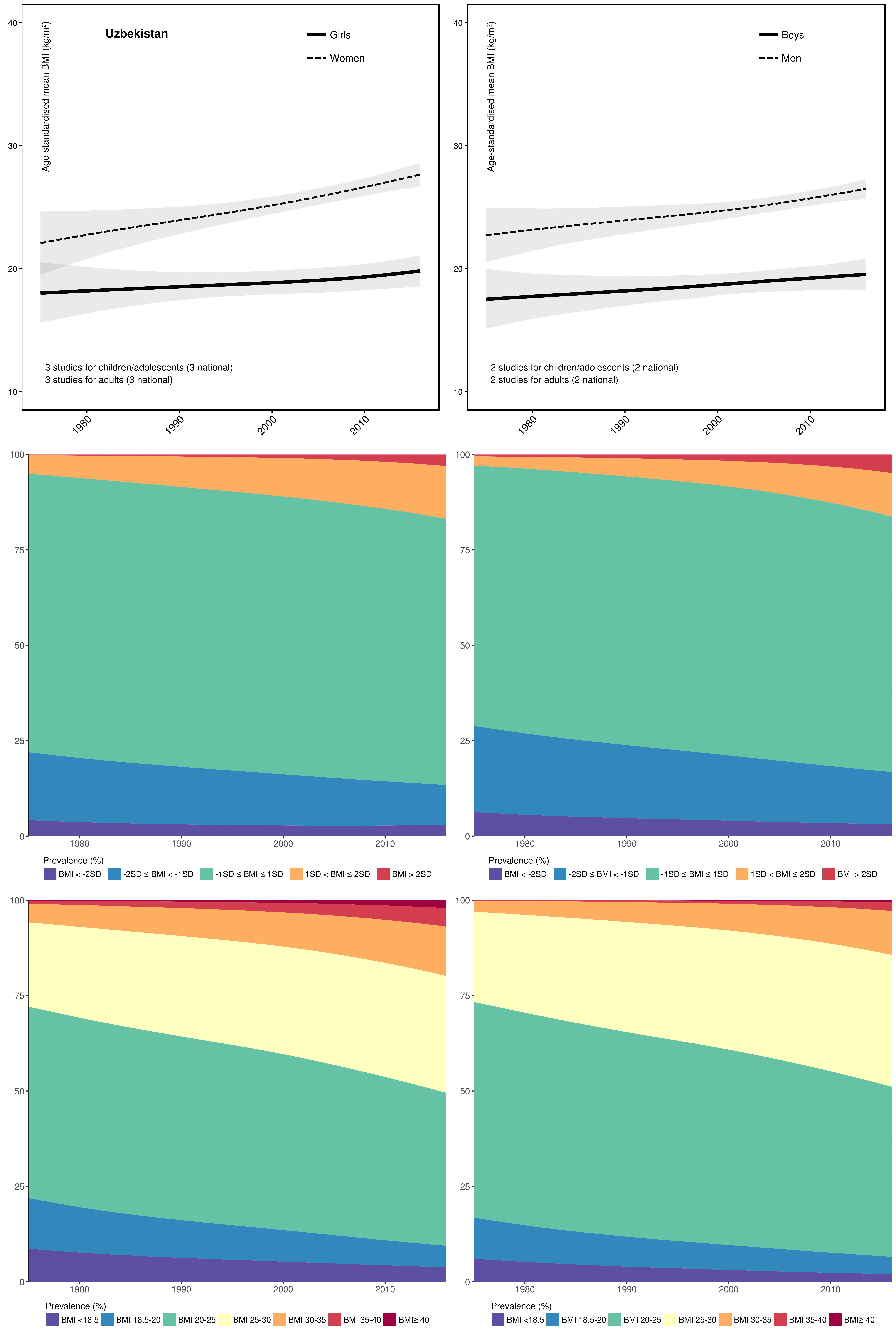

Uzbekistan
Girls
Women
Boys
Men
Age-standardised mean BMI (kg/m²)
3 studies for children/adolescents (3 national)
3 studies for adults (3 national)
2 studies for children/adolescents (2 national)
2 studies for adults (2 national)
Prevalence (%)
BMI < -2SD
-2SD ≤ BMI < -1SD
-1SD ≤ BMI ≤ 1SD
1SD < BMI ≤ 2SD
BMI > 2SD
BMI <18.5
BMI 18.5-20
BMI 20-25
BMI 25-30
BMI 30-35
BMI 35-40
BMI≥ 40

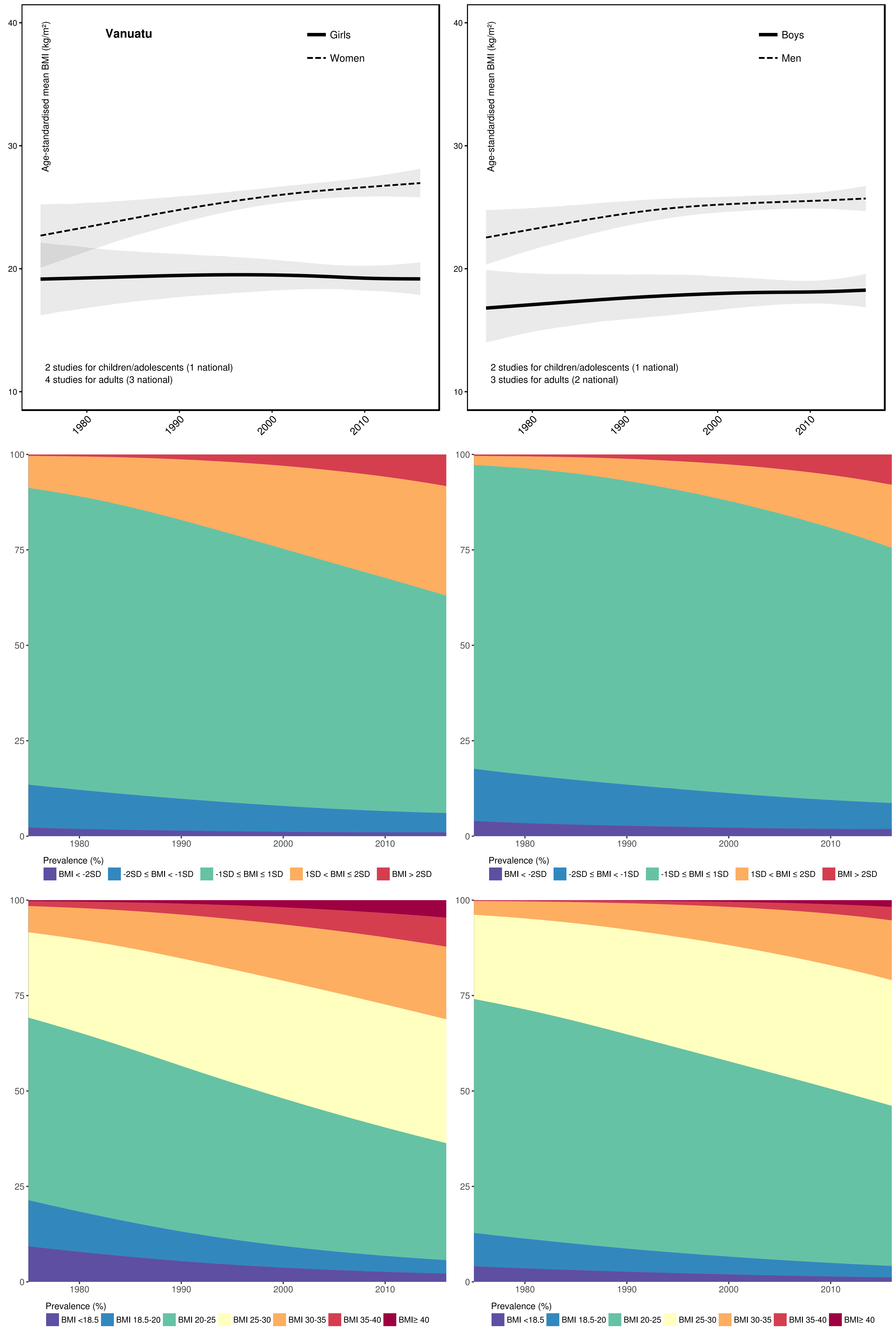
Vanuatu
Girls
Women
Boys
Men
Age-standardised mean BMI (kg/m²)
2 studies for children/adolescents (1 national)
4 studies for adults (3 national)
2 studies for children/adolescents (1 national)
3 studies for adults (2 national)
Prevalence (%)
BMI < -2SD
-2SD ≤ BMI < -1SD
-1SD ≤ BMI ≤ 1SD
1SD < BMI ≤ 2SD
BMI > 2SD
BMI <18.5
BMI 18.5-20
BMI 20-25
BMI 25-30
BMI 30-35
BMI 35-40
BMI≥ 40

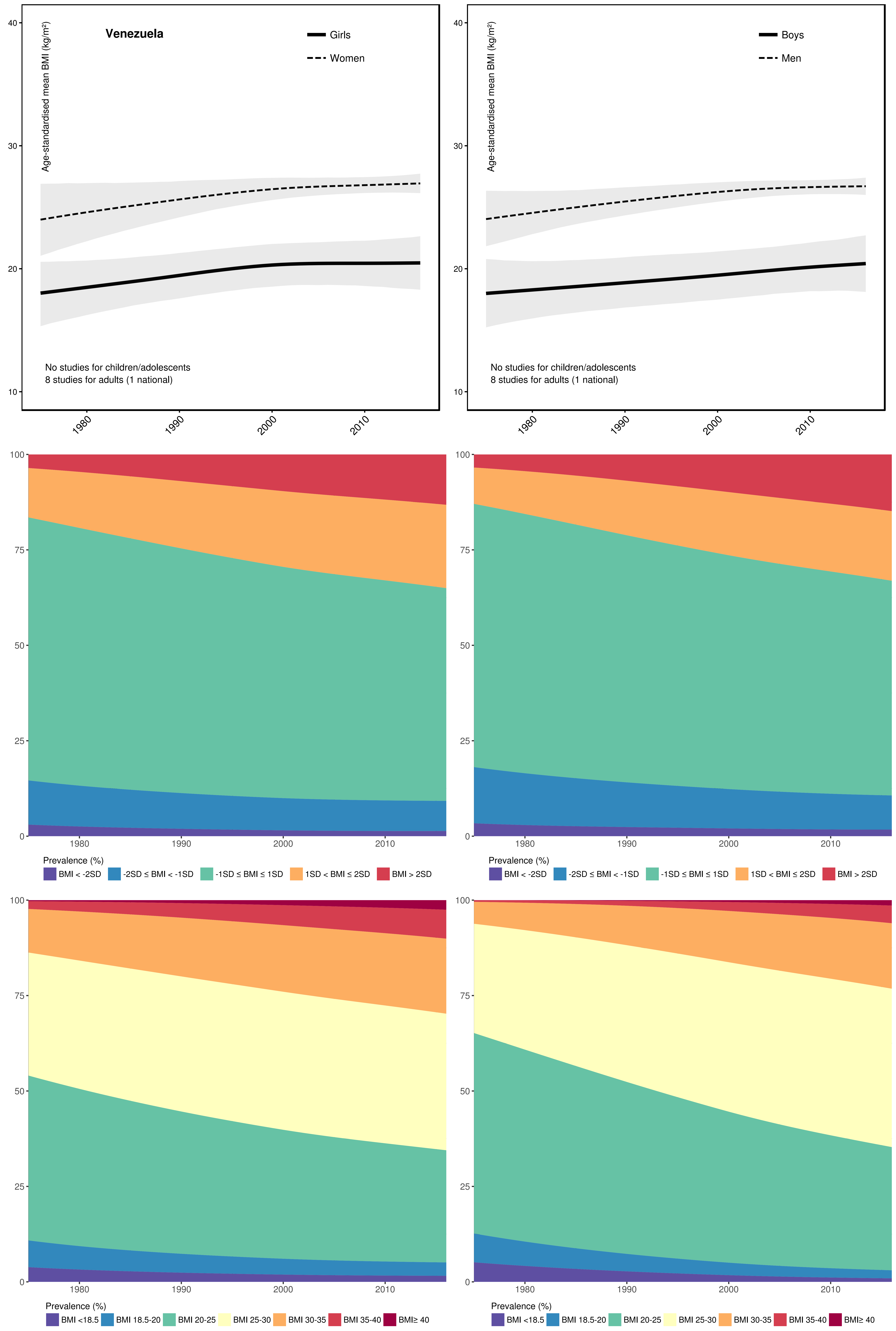
Venezuela
Girls
Women
Boys
Men
Age-standardised mean BMI (kg/m²)
No studies for children/adolescents
8 studies for adults (1 national)
1980
1990
2000
2010
Prevalence (%)
BMI < -2SD
-2SD ≤ BMI < -1SD
-1SD ≤ BMI ≤ 1SD
1SD < BMI ≤ 2SD
BMI > 2SD
BMI <18.5
BMI 18.5-20
BMI 20-25
BMI 25-30
BMI 30-35
BMI 35-40
BMI≥ 40

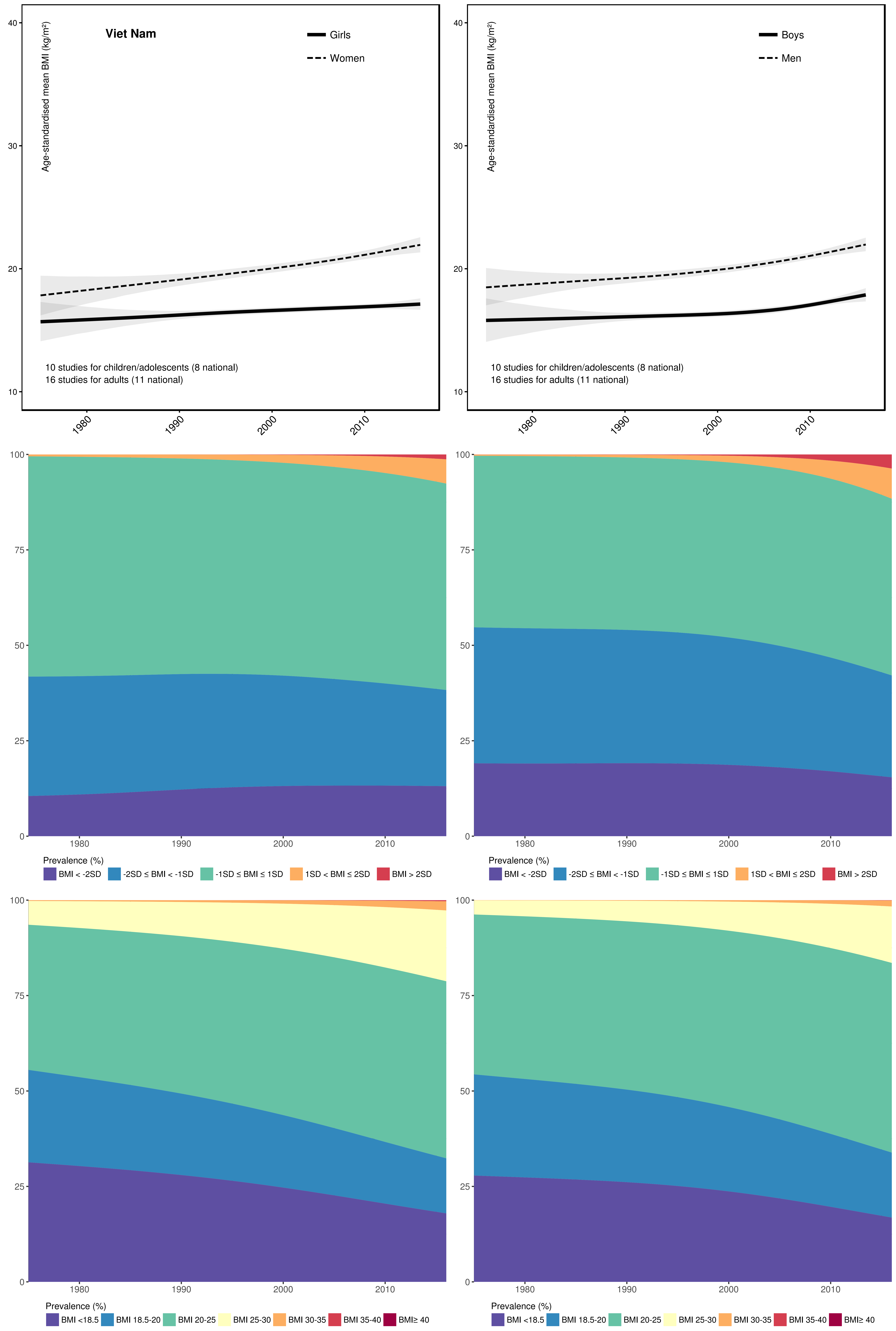

Viet Nam
Girls
Women
Boys
Men
Age-standardised mean BMI (kg/m²)
10 studies for children/adolescents (8 national)
16 studies for adults (11 national)
Prevalence (%)
BMI < -2SD
-2SD ≤ BMI < -1SD
-1SD ≤ BMI ≤ 1SD
1SD < BMI ≤ 2SD
BMI > 2SD
BMI <18.5
BMI 18.5-20
BMI 20-25
BMI 25-30
BMI 30-35
BMI 35-40
BMI≥ 40

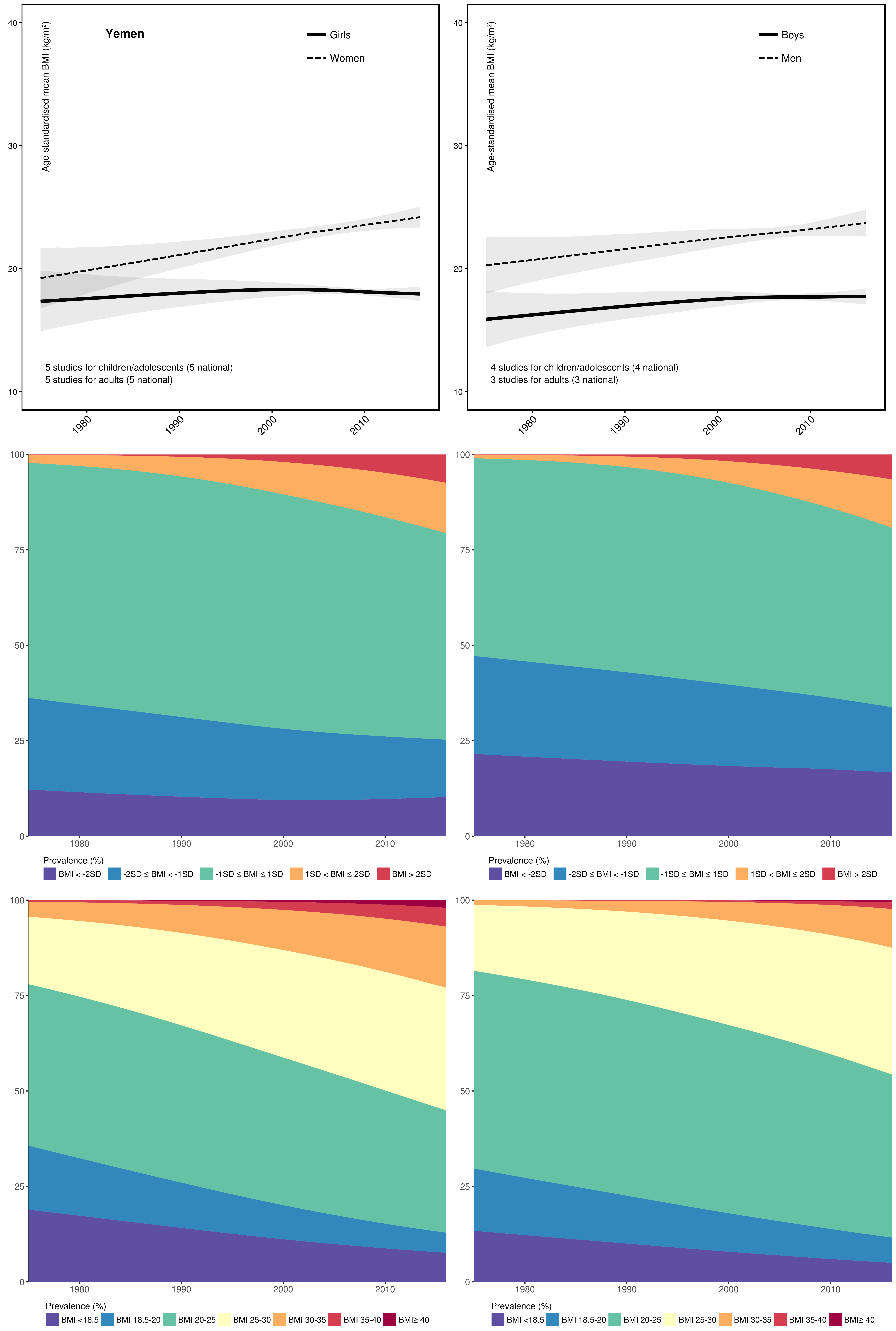
Yemen
Girls
Women
Boys
Men
Age-standardised mean BMI (kg/m²)
5 studies for children/adolescents (5 national)
5 studies for adults (5 national)
4 studies for children/adolescents (4 national)
3 studies for adults (3 national)
Prevalence (%)
BMI < -2SD
-2SD ≤ BMI < -1SD
-1SD ≤ BMI ≤ 1SD
1SD < BMI ≤ 2SD
BMI > 2SD
BMI <18.5
BMI 18.5-20
BMI 20-25
BMI 25-30
BMI 30-35
BMI 35-40
BMI≥ 40

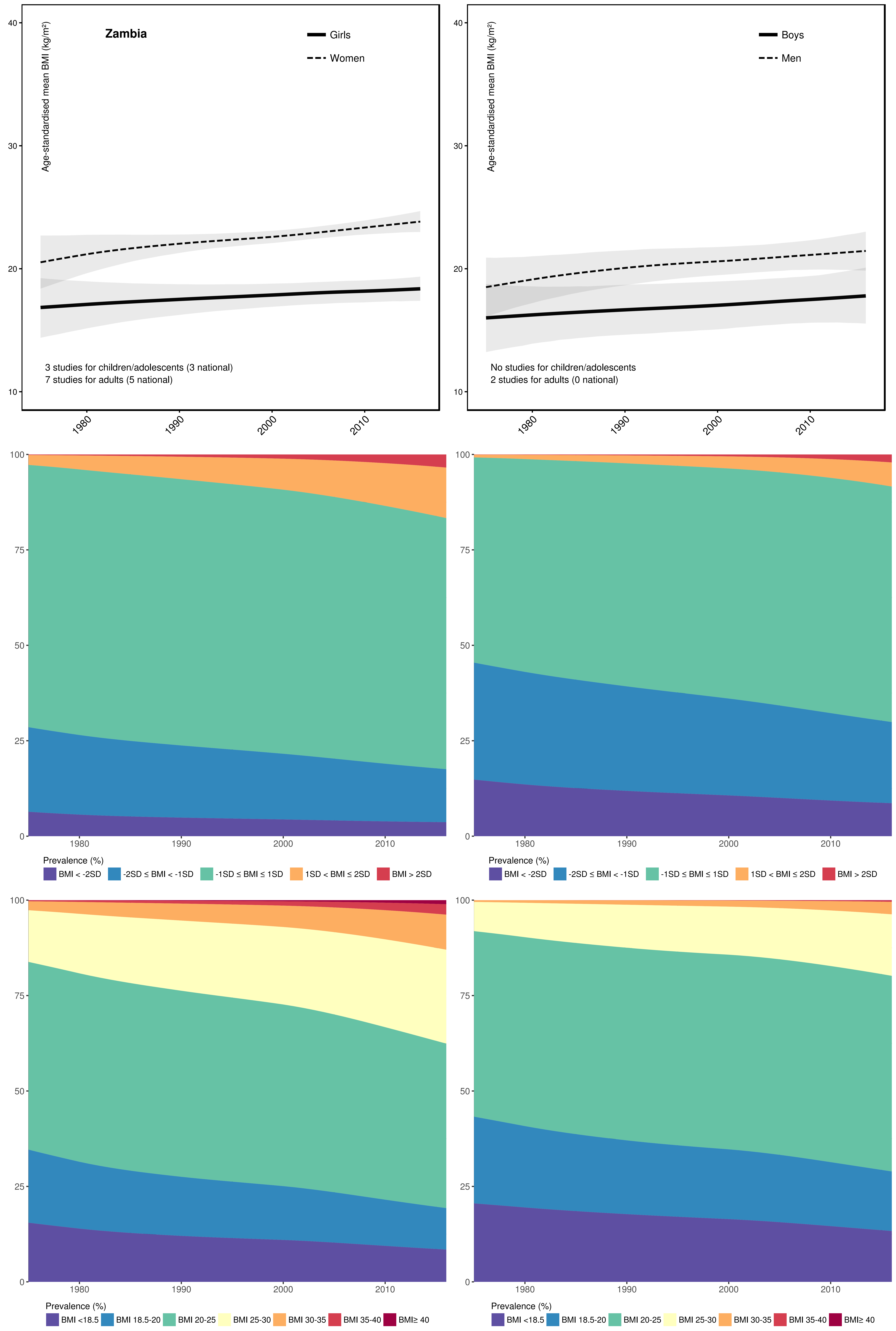

Zambia
Girls
Women
Boys
Men
Age-standardised mean BMI (kg/m²)
3 studies for children/adolescents (3 national)
7 studies for adults (5 national)
No studies for children/adolescents
2 studies for adults (0 national)
Prevalence (%)
BMI < -2SD
-2SD ≤ BMI < -1SD
-1SD ≤ BMI ≤ 1SD
1SD < BMI ≤ 2SD
BMI > 2SD
BMI <18.5
BMI 18.5-20
BMI 20-25
BMI 25-30
BMI 30-35
BMI 35-40
BMI≥ 40

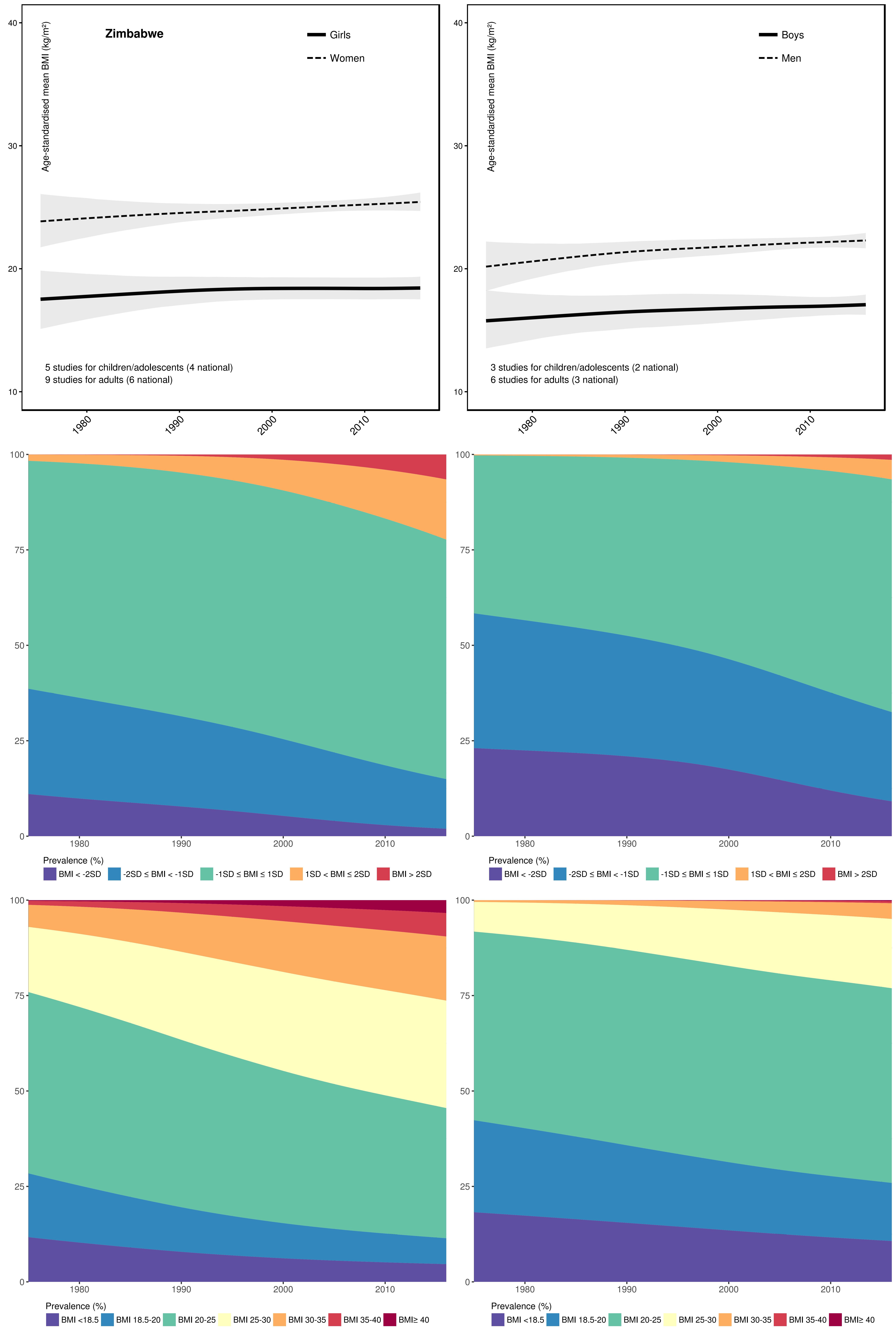
Zimbabwe
Girls
Women
Boys
Men
Age-standardised mean BMI (kg/m²)
5 studies for children/adolescents (4 national)
9 studies for adults (6 national)
3 studies for children/adolescents (2 national)
6 studies for adults (3 national)
1980
1990
2000
2010
Prevalence (%)
BMI < -2SD
-2SD ≤ BMI < -1SD
-1SD ≤ BMI ≤ 1SD
1SD < BMI ≤ 2SD
BMI > 2SD
BMI <18.5
BMI 18.5-20
BMI 20-25
BMI 25-30
BMI 30-35
BMI 35-40
BMI≥ 40

Appendix Figure 7: Age-standardised mean body mass index (BMI), prevalence of obesity (BMI ≥30 kg/m^2) and prevalence of underweight (BMI <18.5 kg/m^2) by sex and country in 2016 in adults (aged 20 years and older).

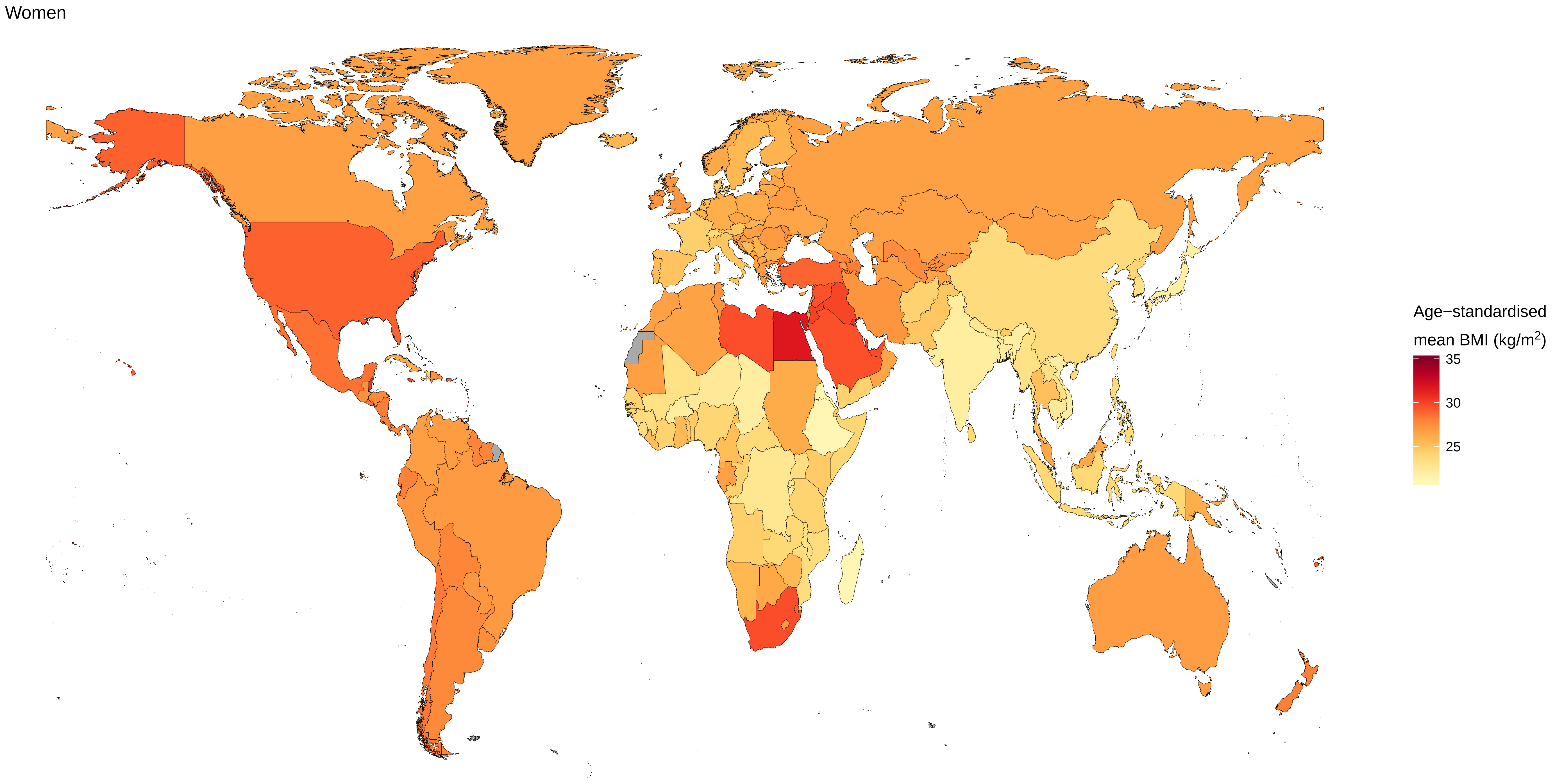
Women
Age−standardised
mean BMI (kg/m2)
35
30
25

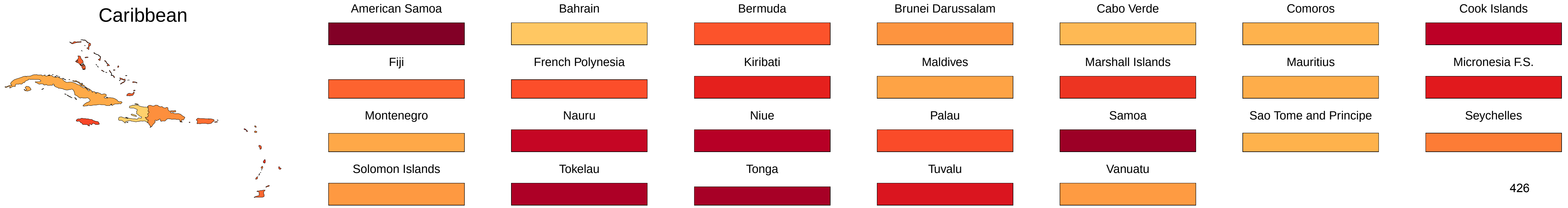
Caribbean
American Samoa
Bahrain
Bermuda
Brunei Darussalam
Cabo Verde
Comoros
Cook Islands
Fiji
French Polynesia
Kiribati
Maldives
Marshall Islands
Mauritius
Micronesia F.S.
Montenegro
Nauru
Niue
Palau
Samoa
Sao Tome and Principe
Seychelles
Solomon Islands
Tokelau
Tonga
Tuvalu
Vanuatu

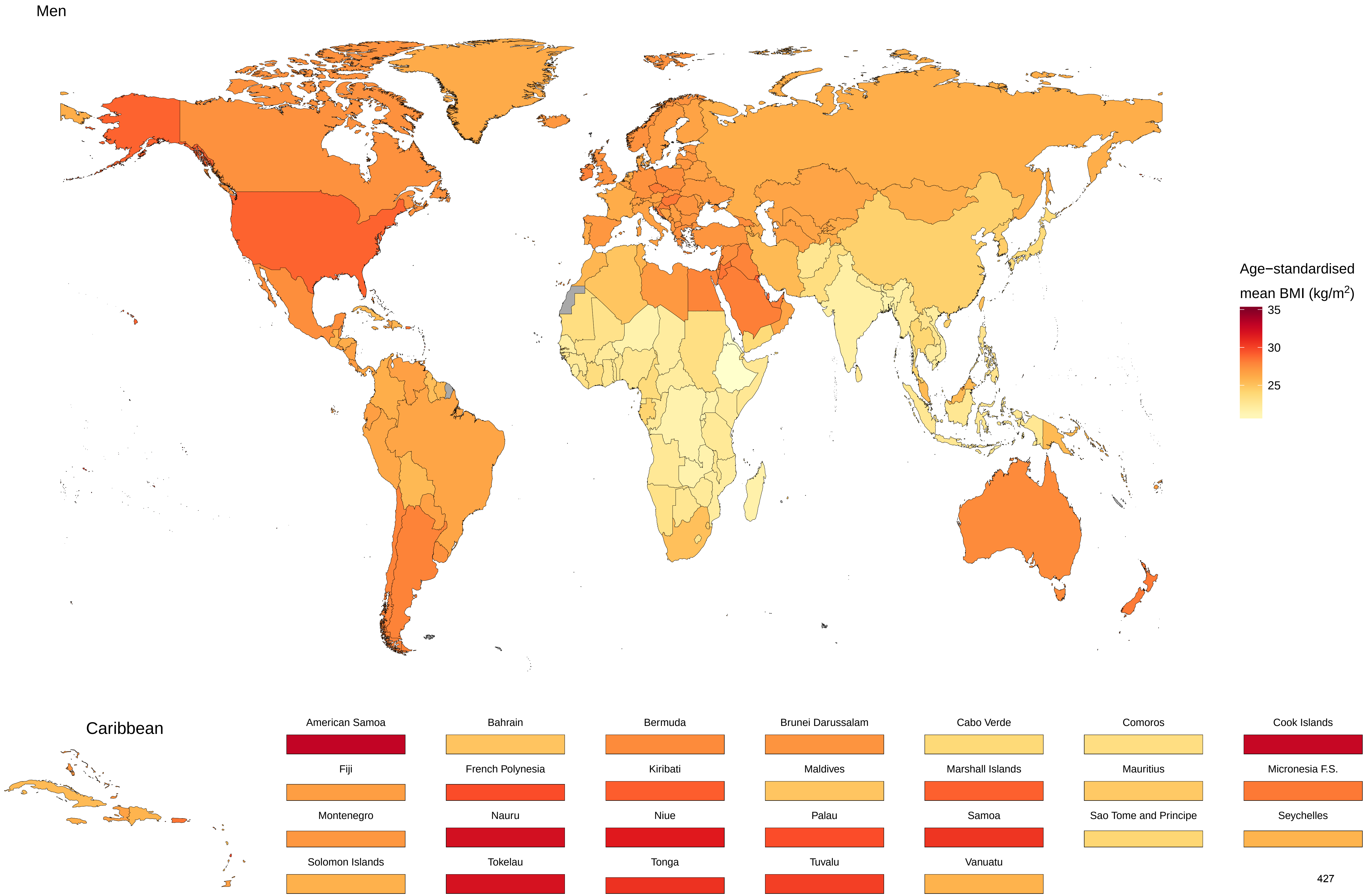
Men
Age−standardised
mean BMI (kg/m2)
35
30
25
Caribbean
American Samoa
Bahrain
Bermuda
Brunei Darussalam
Cabo Verde
Comoros
Cook Islands
Fiji
French Polynesia
Kiribati
Maldives
Marshall Islands
Mauritius
Micronesia F.S.
Montenegro
Nauru
Niue
Palau
Samoa
Sao Tome and Principe
Seychelles
Solomon Islands
Tokelau
Tonga
Tuvalu
Vanuatu

Women

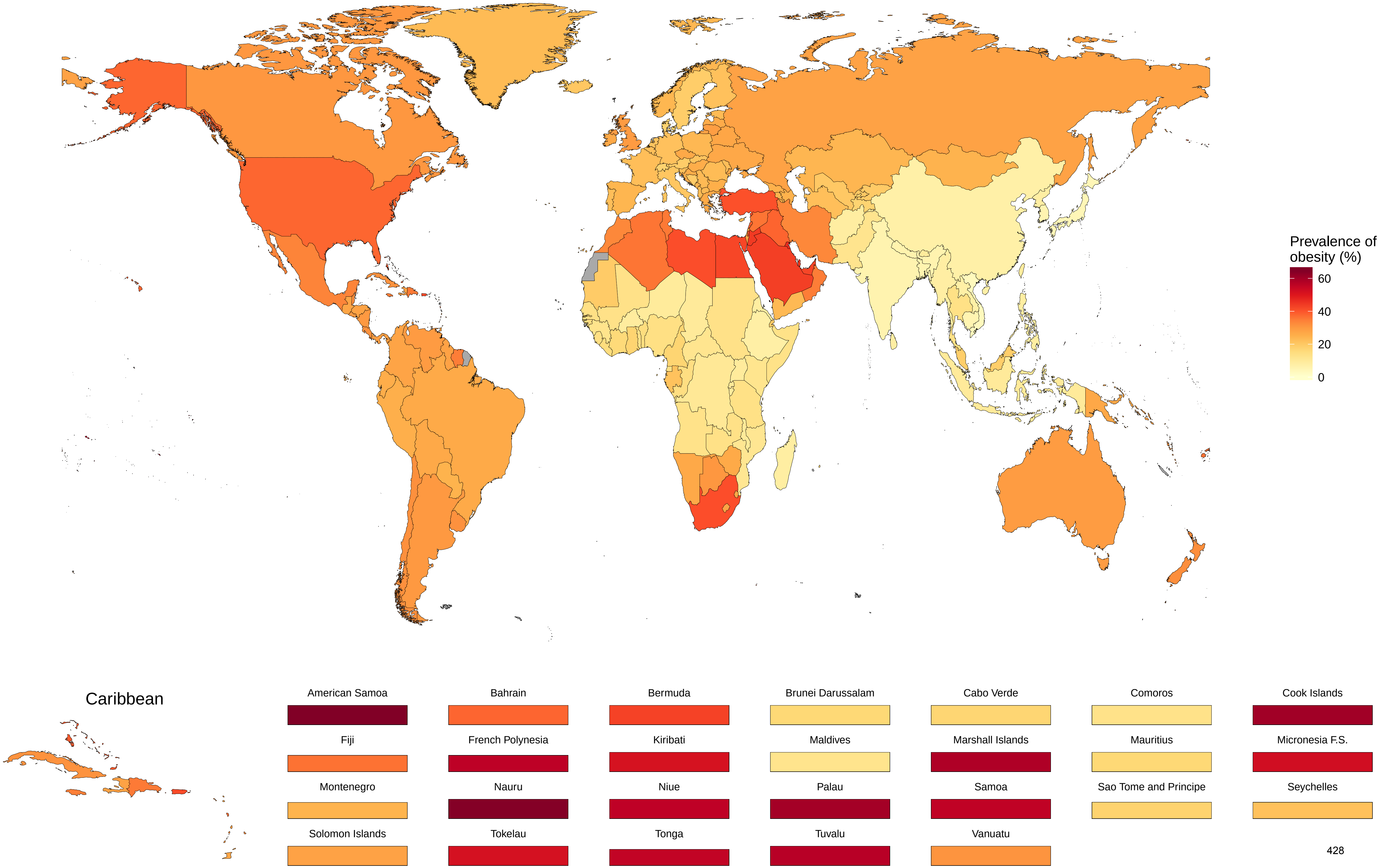
Prevalence of obesity (%)
60
40
20
0
Caribbean
American Samoa
Bahrain
Bermuda
Brunei Darussalam
Cabo Verde
Comoros
Cook Islands
Fiji
French Polynesia
Kiribati
Maldives
Marshall Islands
Mauritius
Micronesia F.S.
Montenegro
Nauru
Niue
Palau
Samoa
Sao Tome and Principe
Seychelles
Solomon Islands
Tokelau
Tonga
Tuvalu
Vanuatu

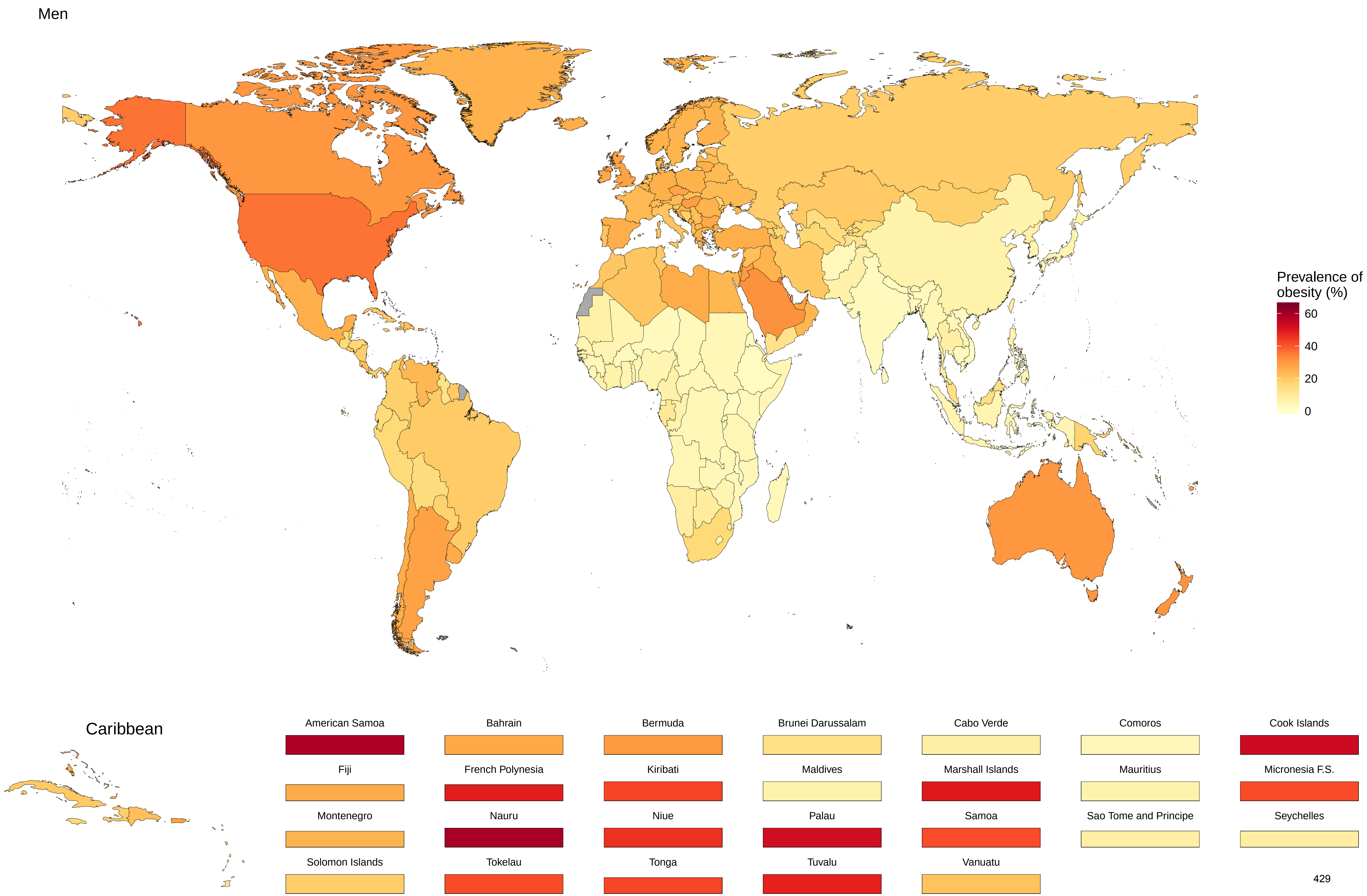
Men
Prevalence of obesity (%)
60
40
20
0
Caribbean
American Samoa
Bahrain
Bermuda
Brunei Darussalam
Cabo Verde
Comoros
Cook Islands
Fiji
French Polynesia
Kiribati
Maldives
Marshall Islands
Mauritius
Micronesia F.S.
Montenegro
Nauru
Niue
Palau
Samoa
Sao Tome and Principe
Seychelles
Solomon Islands
Tokelau
Tonga
Tuvalu
Vanuatu

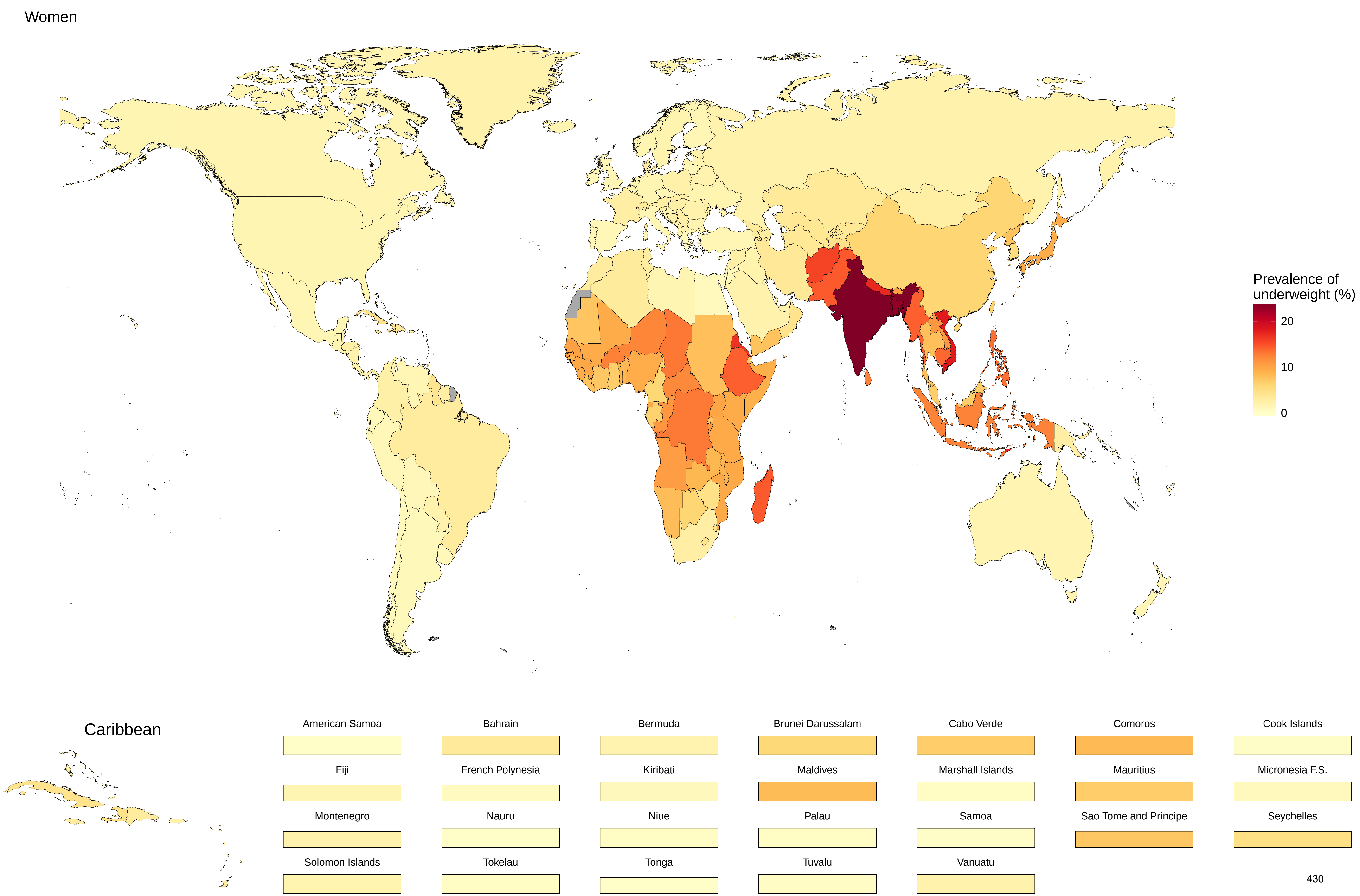
Women
Prevalence of underweight (%)
20
10
0
Caribbean
American Samoa
Bahrain
Bermuda
Brunei Darussalam
Cabo Verde
Comoros
Cook Islands
Fiji
French Polynesia
Kiribati
Maldives
Marshall Islands
Mauritius
Micronesia F.S.
Montenegro
Nauru
Niue
Palau
Samoa
Sao Tome and Principe
Seychelles
Solomon Islands
Tokelau
Tonga
Tuvalu
Vanuatu

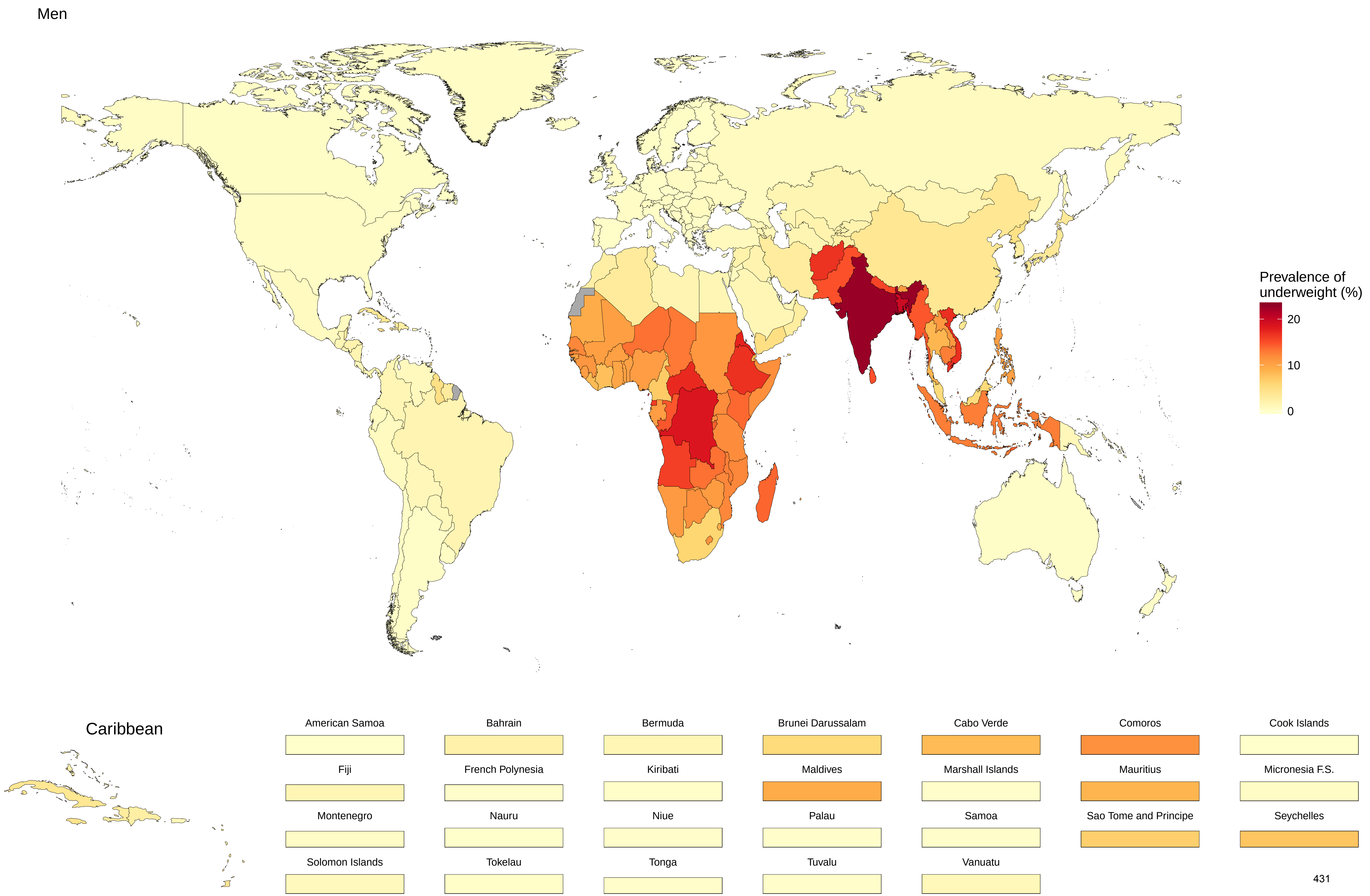
Men
Prevalence of underweight (%)
20
10
0
Caribbean
American Samoa
Bahrain
Bermuda
Brunei Darussalam
Cabo Verde
Comoros
Cook Islands
Fiji
French Polynesia
Kiribati
Maldives
Marshall Islands
Mauritius
Micronesia F.S.
Montenegro
Nauru
Niue
Palau
Samoa
Sao Tome and Principe
Seychelles
Solomon Islands
Tokelau
Tonga
Tuvalu
Vanuatu

Appendix Figure 8: Trends in age-standardised prevalence of body mass index (BMI) categories for adults (aged 20 years and older) by region.

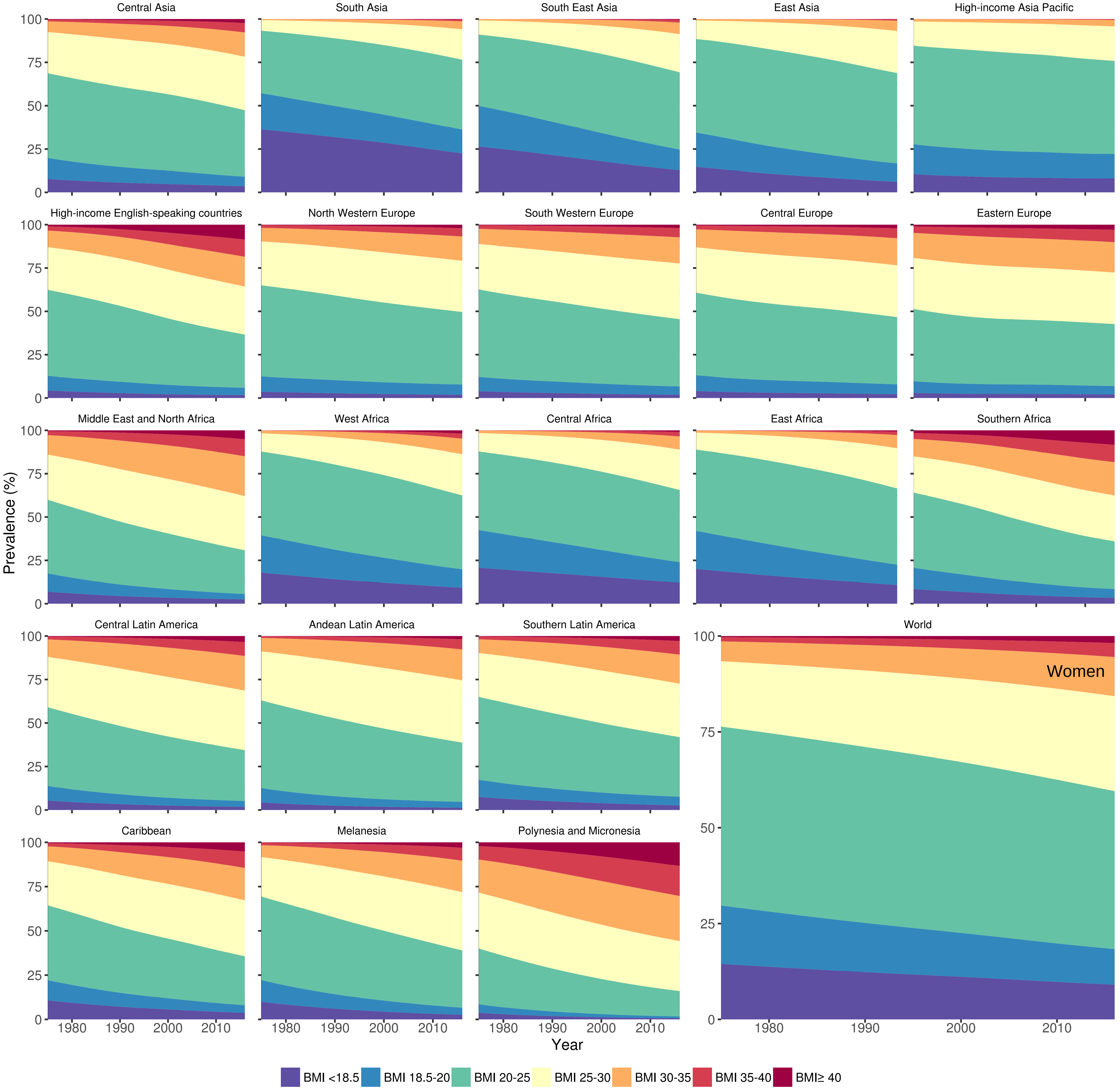
Central Asia
South Asia
South East Asia
East Asia
High-income Asia Pacific
High-income English-speaking countries
North Western Europe
South Western Europe
Central Europe
Eastern Europe
Middle East and North Africa
West Africa
Central Africa
East Africa
Southern Africa
Central Latin America
Andean Latin America
Southern Latin America
World
Caribbean
Melanesia
Polynesia and Micronesia
Women
Prevalence (%)
Year
BMI <18.5
BMI 18.5-20
BMI 20-25
BMI 25-30
BMI 30-35
BMI 35-40
BMI≥ 40

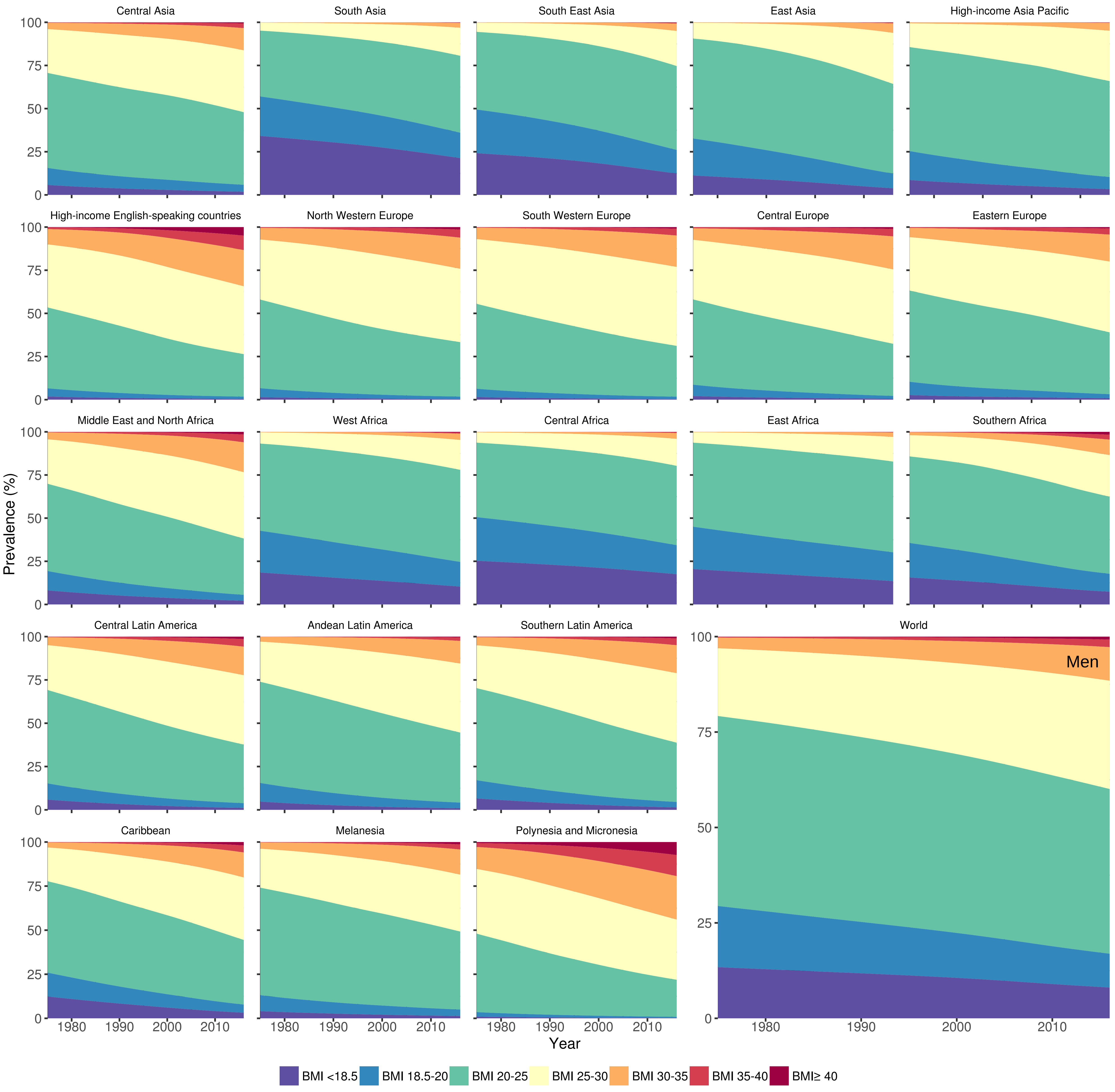
Central Asia
South Asia
South East Asia
East Asia
High-income Asia Pacific
High-income English-speaking countries
North Western Europe
South Western Europe
Central Europe
Eastern Europe
Middle East and North Africa
West Africa
Central Africa
East Africa
Southern Africa
Central Latin America
Andean Latin America
Southern Latin America
World
Men
Caribbean
Melanesia
Polynesia and Micronesia
Prevalence (%)
Year
BMI <18.5
BMI 18.5-20
BMI 20-25
BMI 25-30
BMI 30-35
BMI 35-40
BMI≥ 40

Appendix Figure 9: Trends in the number of obese and underweight adults (aged 20 years and older) by region.

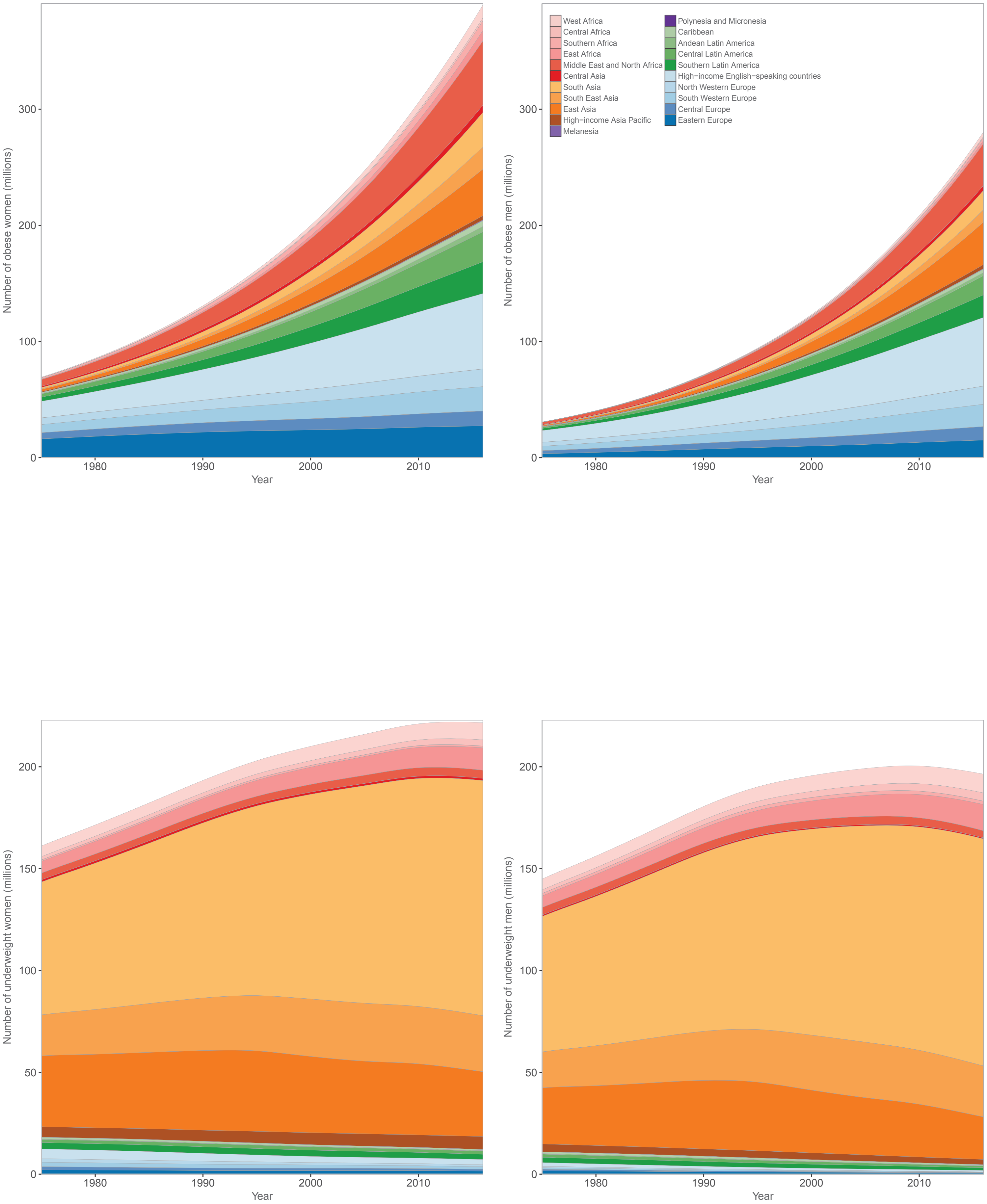

Number of obese women (millions)
Number of obese men (millions)
Number of underweight women (millions)
Number of underweight men (millions)
Year
0
50
100
150
200
300
1980
1990
2000
2010
West Africa
Central Africa
Southern Africa
East Africa
Middle East and North Africa
Central Asia
South Asia
South East Asia
East Asia
High−income Asia Pacific
Melanesia
Polynesia and Micronesia
Caribbean
Andean Latin America
Central Latin America
Southern Latin America
High−income English−speaking countries
North Western Europe
South Western Europe
Central Europe
Eastern Europe

References

1. Finucane MM, Stevens GA, Cowan MJ, et al. National, regional, and global trends in body-mass index since 1980: systematic analysis of health examination surveys and epidemiological studies with 960 country-years and 9.1 million participants. *Lancet* 2011; **377**(9765): 557-67.
2. Danaei G, Finucane MM, Lin JK, et al. National, regional, and global trends in systolic blood pressure since 1980: systematic analysis of health examination surveys and epidemiological studies with 786 country-years and 5.4 million participants. *Lancet* 2011; **377**(9765): 568-77.
3. Danaei G, Finucane MM, Lu Y, et al. National, regional, and global trends in fasting plasma glucose and diabetes prevalence since 1980: systematic analysis of health examination surveys and epidemiological studies with 370 country-years and 2· 7 million participants. *Lancet* 2011; **378**(9785): 31-40.
4. Farzadfar F, Finucane MM, Danaei G, et al. National, regional, and global trends in serum total cholesterol since 1980: systematic analysis of health examination surveys and epidemiological studies with 321 country-years and 3· 0 million participants. *Lancet* 2011; **377**(9765): 578-86.
5. NCD Risk Factor Collaboration. Trends in adult body-mass index in 200 countries from 1975 to 2014: a pooled analysis of 1698 population-based measurement studies with 19.2 million participants. *Lancet* 2016; **387**(10026): 1377-96.
6. de Onis M, Onyango AW, Borghi E, Siyam A, Nishida C, Siekmann J. Development of a WHO growth reference for school-aged children and adolescents. *Bull World Health Organ* 2007; **85**(9): 660-7.
7. Schwarz G. Estimating the dimension of a model. *Ann Stat* 1978; **6**(2): 461-4.
8. NCD Risk Factor Collaboration. A century of trends in adult human height. *eLife* 2016; **5**: e13410.
9. NCD Risk Factor Collaboration. Worldwide trends in blood pressure from 1975 to 2015: a pooled analysis of 1479 population-based measurement studies with 19.1 million participants. *Lancet* 2017; **389**(10064): 37-55.
10. NCD Risk Factor Collaboration. Worldwide trends in diabetes since 1980: a pooled analysis of 751 population-based studies with 4.4 million participants. *Lancet* 2016; **387**(10027): 1513-30.
11. Nakagawa S, Schielzeth H. A general and simple method for obtaining R2 from generalized linear mixed-effects models. *Methods Ecol Evol* 2013; **4**(2): 133-42.
12. McFadden D. Conditional Logit Analysis of Qualitative Choice Behavior. In: Zarembka P, ed. Frontiers in econometrics: Academic Press; 1974: 105-42.
